# Supplementary material for: Nature portrayed in images in Dutch Brazil: Tracing the sources of the plant woodcuts in the Historia Naturalis Brasiliae (1648)
Source: PLoS One. 2024 Jul 16;19(7):e0276242. doi: 10.1371/journal.pone.0276242 (PMC11251624; doi:10.1371/journal.pone.0276242)
Supplement: S1 Appendix — This PDF file includes the repertoire of woodcut images of the plants depicted in the HNB and their corresponding images retrieved from older or contemporary sources by cross-referencing their scientific names. This appendix allows us to visualize–among botanical illustrations, herbarium vouchers, plant sketches, and other plant materials that crossed the Atlantic–the potential sources that were used to elaborate the woodcuts in the HNB. (PDF) [file pone.0276242.s001.pdf]

Nature portrayed in images in Dutch Brazil

Appendix (S2)

Sources of the plant woodcuts in the *Historia Naturalis Brasiliae* (1648) Database

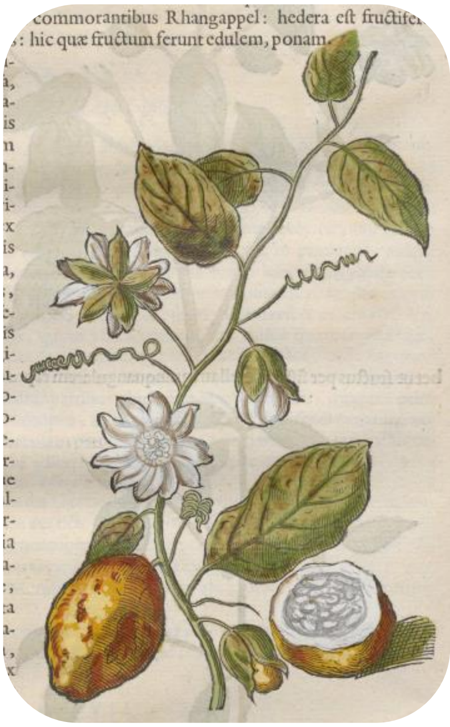

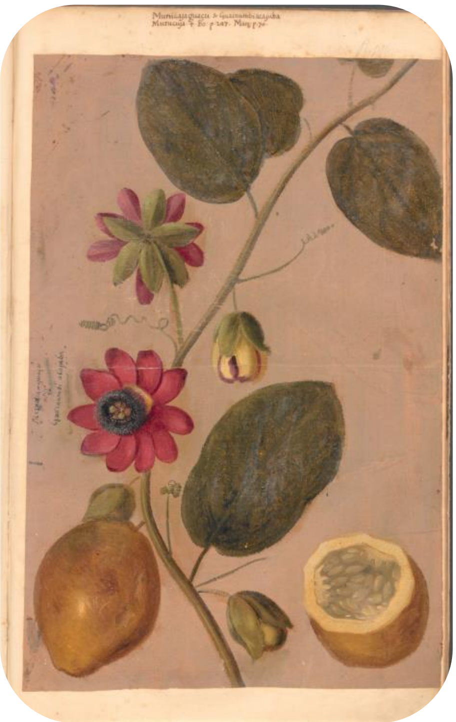

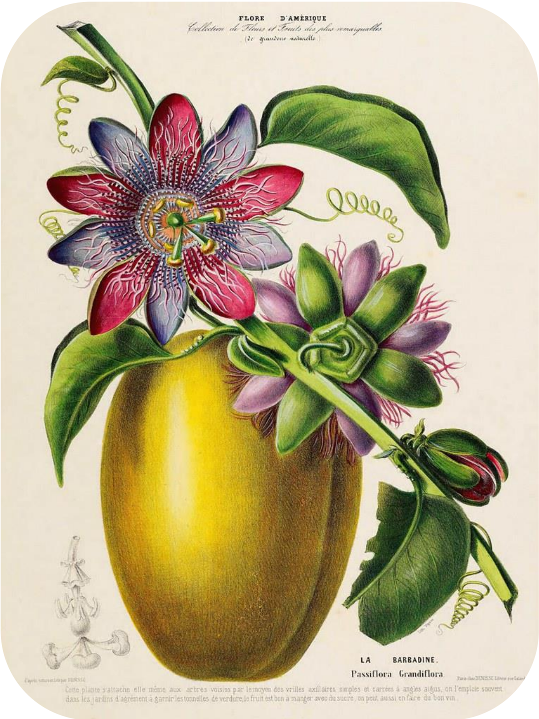

Mireia Alcantara Rodriguez

Nature portrayed in images in Dutch Brazil

Appendix (S2)

Sources of the plant woodcuts in the *Historia Naturalis Brasiliae* (1648) Database

Mireia Alcantara Rodriguez

The main research paper to which this appendix belongs is:

Nature portrayed in images in Dutch Brazil: Tracing the sources of the plant woodcuts in the Historia Naturalis Brasiliae (1648) by Mireia Alcantara Rodriguez, Tinde van Andel, and Mariana Françozo.

This appendix is available via the repository of Leiden University, 2022 (https://doi.org/10.17026/dans-zk4-ercv).

Cover image (from left to right): woodcut of *Passiflora quadrangularis* L. in the *Historia Naturalis Brasiliae* (Marcgrave 1648: 70), oil-based illustration of the same species in the *Theatrum Rerum Naturalium* (p. 437), engraving of the same species in the “Flore d’Amérique” by Denisse, E. t. (1843-1846: t. 166).

The research leading to this database received funding from the European Research Council (ERC) Horizon 2020 Research and Innovation Program (Agreement No. 715423), ERC Project BRASILIAE: Indigenous Knowledge in the Making of Science, directed by Dr. M. Françozo at Leiden University.

Preface

I designed this publication to accompany the research article Nature portrayed in images in Dutch Brazil: Tracing the sources of the plant woodcuts in the Historia Naturalis Brasiliae (1648) by Mireia Alcantara Rodriguez, Tinde van Andel, and Mariana Françozo. This visual database is directed to everyone attracted to the fascinating world of tropical plants and Renaissance and early modern botanical art, with the main focus on the flora of Dutch Brazil (the northeastern territories of Brazil colonized by the Dutch from 1630 to 1654). This PDF file was built with Filemaker Pro software in a database format, and both files are available (open access) in the repository of Leiden University via: https://doi.org/10.17026/dans-zk4-ercv.

The main purpose of this file is to present in a visual form the potential sources used to create the plant woodcuts embedded in the *Historia Naturalis Brasiliae* (HNB). A brief text accompanies every plant entry in which I discuss the various levels of correlation among sources. The HNB is a western treatise that documents the flora, fauna, medicine, climate, and the inhabitants coexisting in Dutch Brazil as perceived by German naturalist George Marcgrave (1610-1643) and Dutch physician Willem Piso (1611-1678) – among others. This work was funded by count Johan Maurits of Nassau-Siegen (1604-1679), whose governorship in Dutch Brazil lasted from 1636 to 1644, and edited by cartographer Johannes de Laet (1581-1649), one of the directors of the Dutch West India Company. This file can be read independently but its main function is to complement and depict the data analyzed in the main paper, which is also displayed in an Excel spreadsheet (S1). The main paper aimed to answer what were the sources used to create the plant woodcuts in the HNB, first, by arranging together the woodcut images and their corresponding species in other visual sources from Dutch Brazil and analyzing their similarities; and second, by

searching for the remaining sources in contemporary or older treatises that included engravings similar to the HNB woodcuts. By doing so, the various methods of visual-knowledge-making are discussed, including the methods of plant collection and flora representation. This publication presents a catalog of the data on plant woodcuts in the HNB, accounting for 243 database entries with 1458 images that correspond to 237 taxa. Related to this research, I created a second dataset (S3) and its corresponding PDF file (Appendix S4) to analyze the sources of the plant woodcuts in the *India Utriusque re Naturali et Medica* (IURNM, Piso 1658). The images and text of this treatise were edited by Piso ten years after the publication of the HNB. It is recommended to check both visual files (S2 vs. S4) to observe the diversity of visual knowledge production in the HNB and the IURNM.

The woodcuts are organized in the same order as in the HNB and all the visual sources are arranged by their corresponding species. The section *Medicina Brasiliense* by Piso (1648) includes 37 plant woodcuts, which corresponds to pages 1 to 74 in this file, and the *Historia Rerum Naturalium* by Marcgrave (1648) portrays 206 plant woodcuts (pages 75 to 486). Each entry can be read independently from the others. The database and its entry forms provide information on the vernacular plant names, species, botanical families, and notes on the correlation between the woodcuts and contemporary or older plant images that represent the same species. Following, the plant woodcut's images and the associated images are displayed. These images were retrieved by the main author from the HNB (Marcgrave 1648; Piso 1648), the IURNM (Piso 1658), Marcgrave's herbarium (https://samlinger.snm.ku.dk/en/dry-and-wet-collections/botany/general-herbarium/the-marcgrave-herbarium/), De Laet manuscript (BL Sloane MS 1554), the *Theatrum Rerum Naturalium* (provided as digital images by the Jagiellonian

library), the *Miscellanea Cleyeri* (https://jbc.bj.uj.edu.pl/dlibra/doccontent?id=197455) and the *Libri Principis* (https://jbc.bj.uj.edu.pl/dlibra/publication/193892/edition/183824/content); as well as from treatises digitized and available open access via digital libraries (archive.org, biodiversitylibrary.org). Often, this file includes modern photos of the species portrayed in the woodcuts, mostly retrieved from Creative Commons and Plantillustrations.org.

Medicina Brasiliensi Piso, 1648 Page number 50

Vernacular name(s) Saccharo

Species Saccharum officinarum L.

Family Poaceae

Notes

This woodcut portrays a sugar mill, ox-driven and operated by four enslaved Africans. Two Dutch foremen supervise the job. The Dutch painter Frans Post presumably drew this image (Joost 1983), which bears resemblance to the images depicted in Caspar Barleus's (1647) treatise on Johan Maurits' accounts in Brazil. It is common to find scenes of African slaves in Post's landscape paintings, sometimes interacting with Europeans. These interactions captured in images the unequal power relationships they entailed, which reflect the slave society of Dutch Brazil (Monteiro 2019).

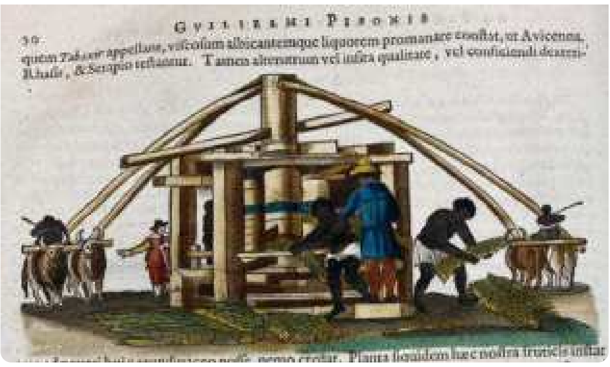

De Facultatibus Simplicium: 50

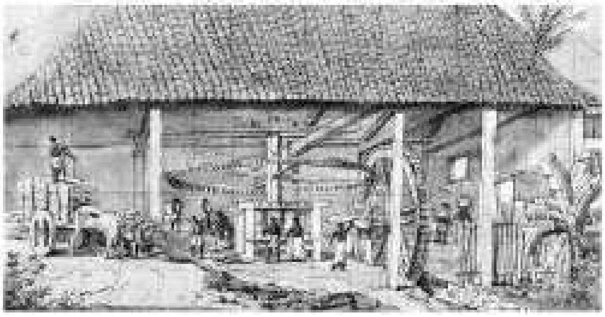

Frans Post,
c. 1637–44, ink on paper (KIK-IRPA,Brussels) (Liza 2013)

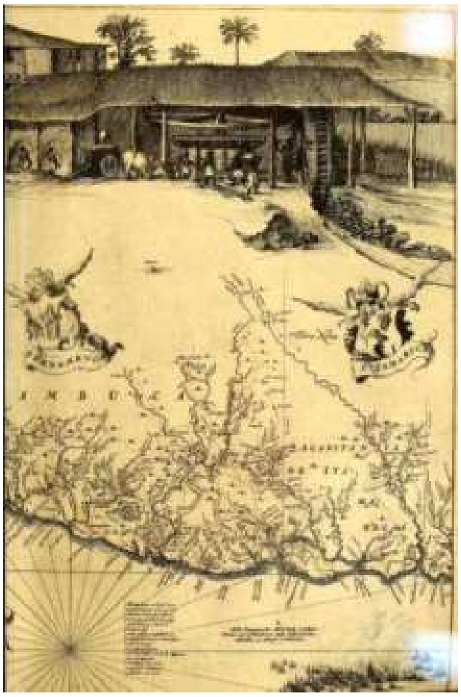

Engraving in Barlaeus' "Rerum per octennium in Brasilia" (1647) as part of a map made by Marcgrave with sugar mills by Post

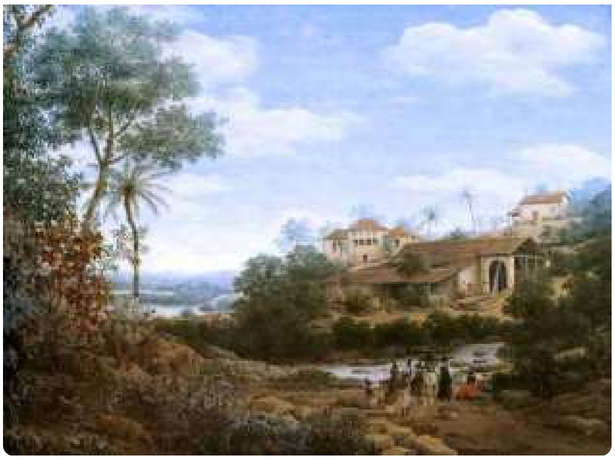

F. Post, Brazilian Landscape with Manor House and Sugar Mill, c. 1660. Staatliches Museum by Jean Louis Mazieres

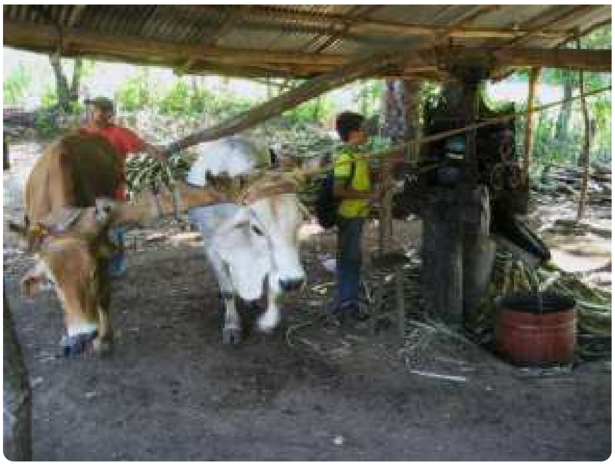

Sugar mill; by LShave (CC BY-NC-SA 2.0)

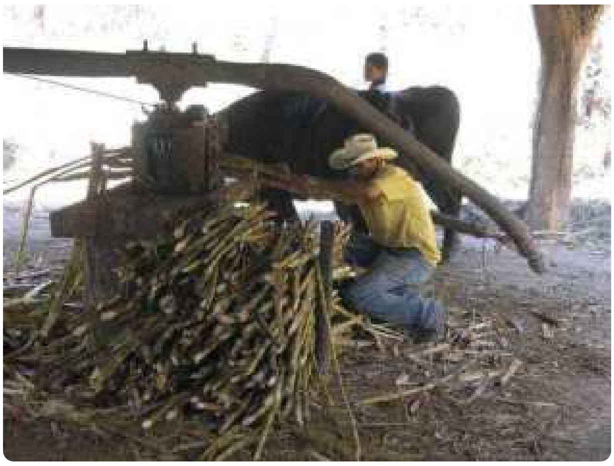

Sugar cane mill; by Lon&Queta (CC BY-NC-SA 2.0)

Medicina Brasiliensi Piso, 1648 Page number 51

Vernacular name(s) Saccharo

Species Saccharum officinarum L.

Family Poaceae

Notes

Likely, this woodcut was made after a drawing of Frans Post. This engraving portrays the sugar ovens where the sugar was processed by the forced labor of four African slaves. A Dutch foreman supervises the scene while holding a rod in his right hand, with which he probably flogged the slaves. See https://www.mauritshuis.nl/en/our-collection/stories/johan-maurits/ for more about Dutch Brazil and the role of Johan Maurits in slavery. Albert Eckhout shows a sugar cane plantation in his portrait "Biracial man". A similar sugarcane branch was depicted in Marcgrave (1648: 83).

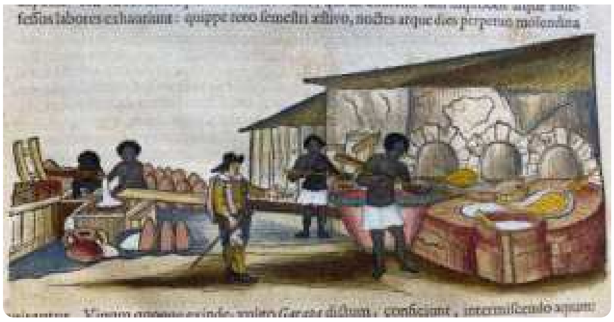

De Facultatibus Simplicium: 51

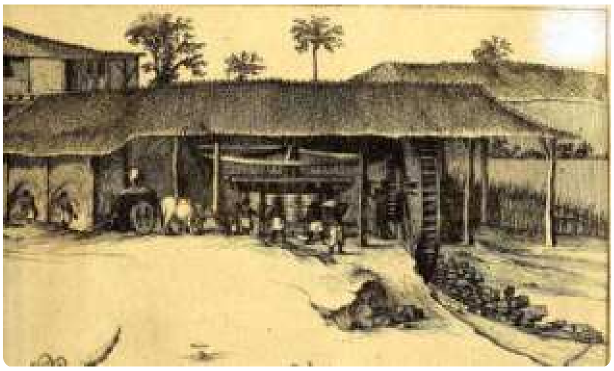

Engraving in Barlaeus' "Rerum per octennium in Brasilia" (1647), Sugar ovens depicted on the left

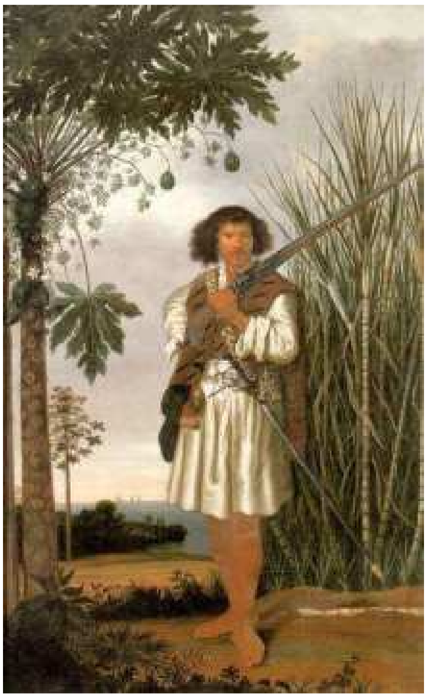

Ekchout portray (Biracial man) 1641

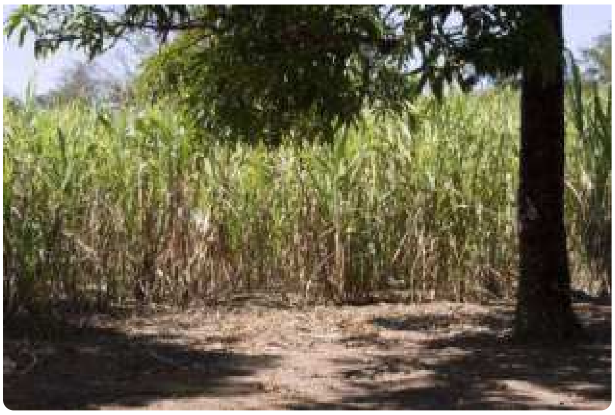

Sugar cane plantation, Minas Gerais, Brasil 2009; by Royal Olive (CC BY-NC-ND 2.0)

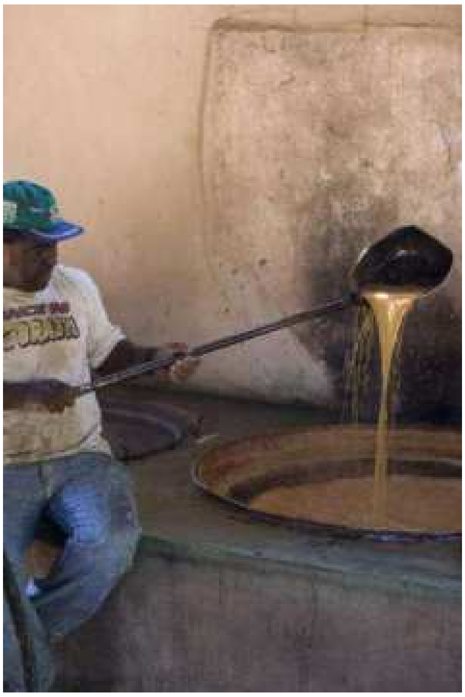

Garapa, Paracatu 2009; by Royal Olive (CC BY-NC-ND 2.0)

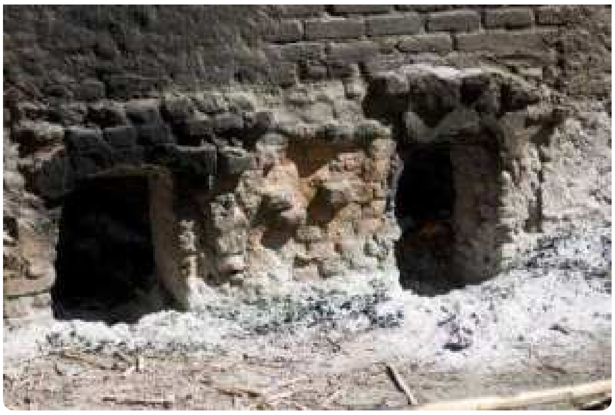

Sugar cane ovens, Paracatu 2009; by Royal Olive (CC BY-NC-ND 2.0)

Medicina Brasiliensi Piso, 1648 Page number 53

Vernacular name(s) Mandihoca

Species Manihot esculenta Crantz

Family Euphorbiaceae

Notes

This woodcut shows a cassava processing machine and five enslaved Africans operating it. The engraver Joan Blaeu (1647) portrayed a similar image in "Brasilia qua parte Belgis", based on maps designed by Marcgrave and illustrations by Post. De Laet added an image of the processing of the cassava, rather than an image of the plant. There are no images of the cultivated cassava in Marcgrave either. This plant is found in the Misc. Cleyeri, in a colored chalk drawing, presumably made by Eckhout. The still-life painting of Eckhout "Manioc" and his portrait of the "Tupinamba man" depict it in a very similar way

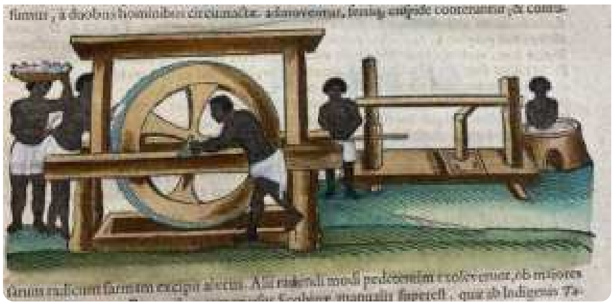

De Facultatibus Simplicium: 53

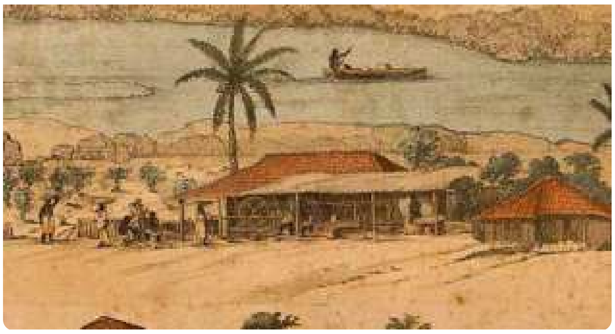

Detail of the map engraved by Blaeu (1647) showing a manioc processed machine. In: *Brasilia qua parte paret Belgis, da Coleção Brasiliana Iconográfica*

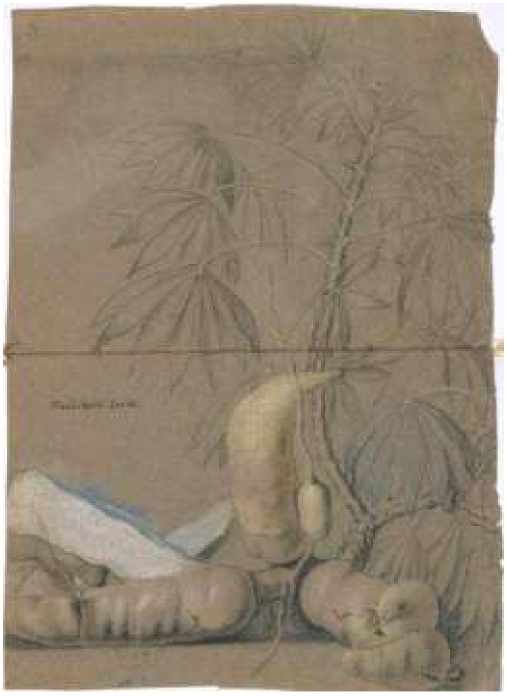

Miscellanea Cleyeri c.1637-44: 69v and 70r

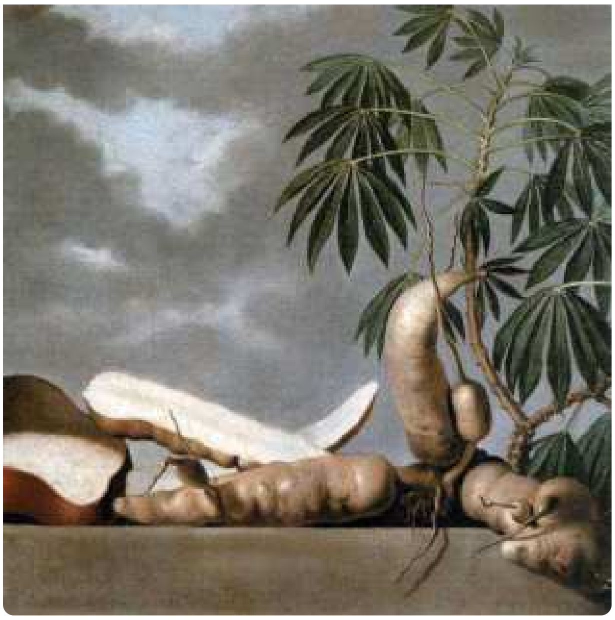

Eckhout still-life (Manioc)

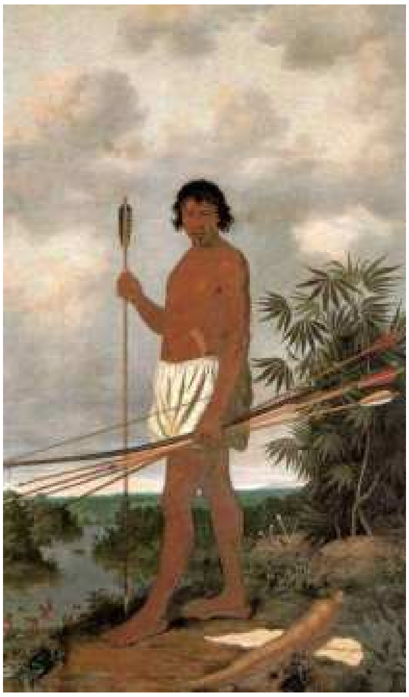

Eckhout portray (Tupinamba man)

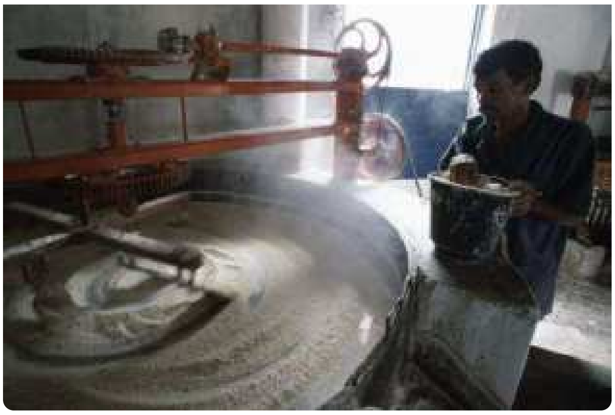

Processing manioc at a rural cooperative, northeast Brazil 2009; by World Bank Photo Collection (CC BY-NC-ND 2.0)

Medicina Brasiliensi Piso, 1648 Page number 70

Vernacular name(s) Araticu-pana

Species Annona glabra L.

Family Annonaceae

Notes

The woodcut is very similar to the *Theatrum* image (non-reversed), although the round and big fruit do not appear in the *Theatrum*. The fruit of *A. glabra* can be found in the *Misc. Cleyeri* (cut in half) and in the still-life painting "Brazilian fruits" by Eckhout.

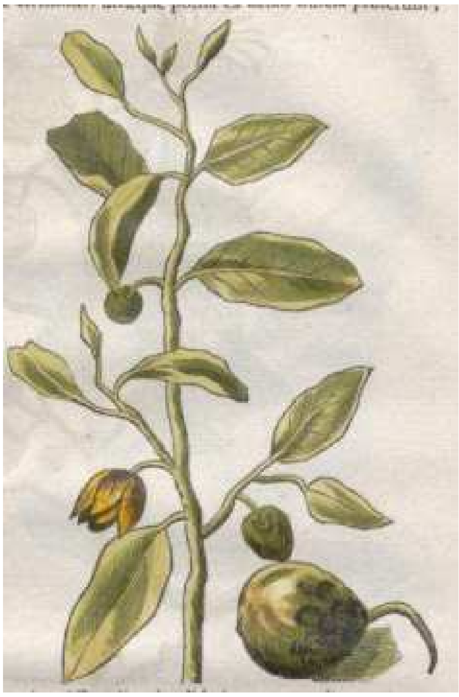

De Facultatibus Simplicium: 70

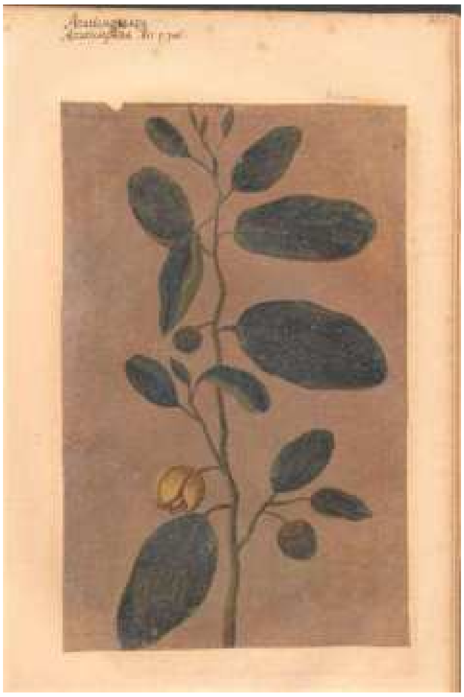

Theatrum Rerum Naturalium bound c. 1660-1664: 333

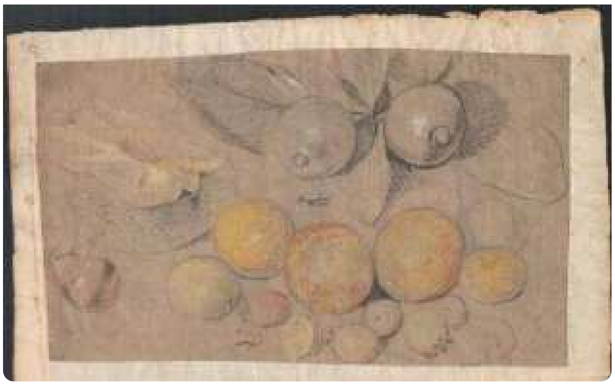

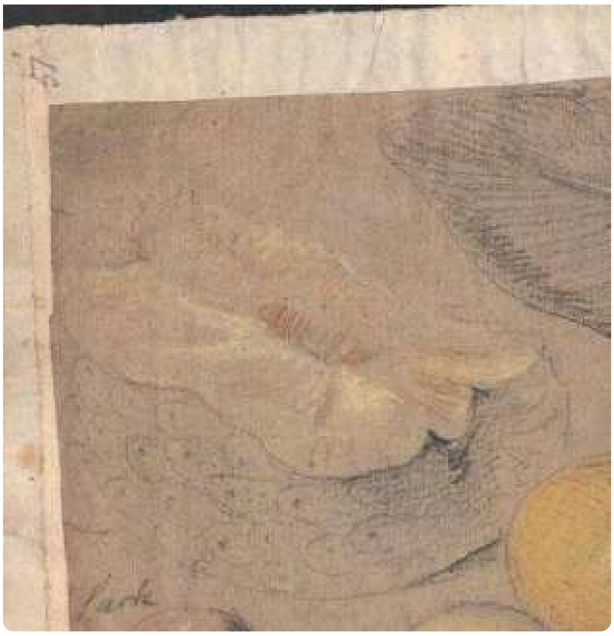

Miscellanea Cleyeri c.1637-44: 51r

Miscellanea Cleyeri c.1637-44: 51r

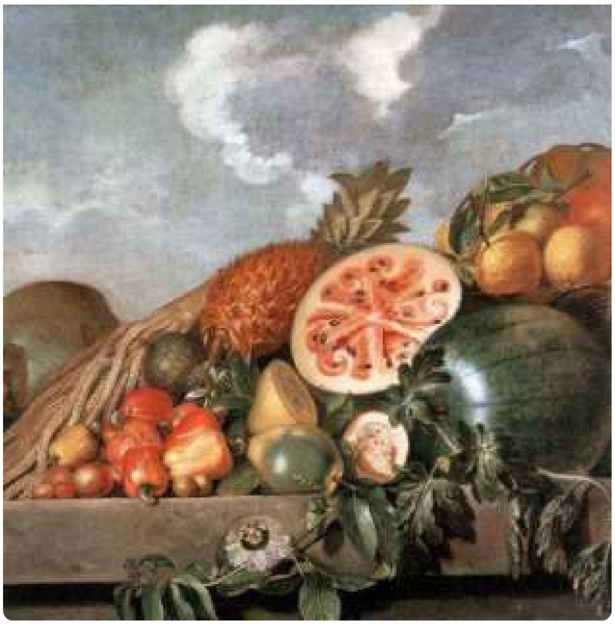

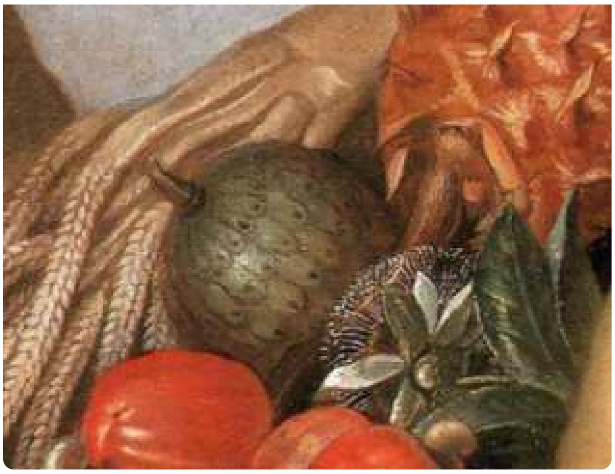

Eckhout still-life (Brazilian fruits), c. 1637-44

Eckhout still-life (Brazilian fruits) c. 1637-44

Medicina Brasiliensi Piso, 1648 Page number 71

Vernacular name(s) Embira. Pindaiba

Species Xylopia frutescens Aubl.

Family Annonaceae

Notes

The woodcut looks different than its corresponding image in the *Theatrum*. The same species is described and depicted in Marcgrave (1648: 99) and the woodcut in that section is very similar to *X. frutescens* in the *Theatrum*.

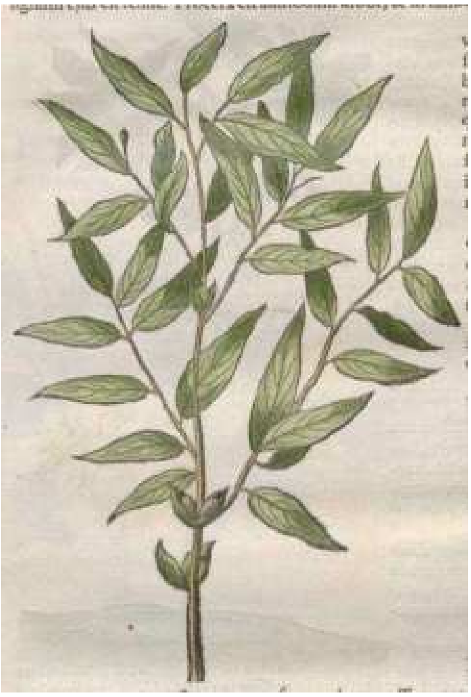

De Facultatibus Simplicium: 71

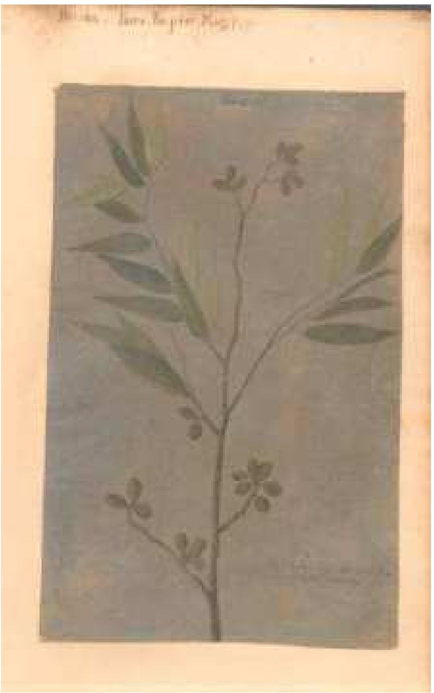

Theatrum Rerum Naturalium bound c. 1660-1664: 131

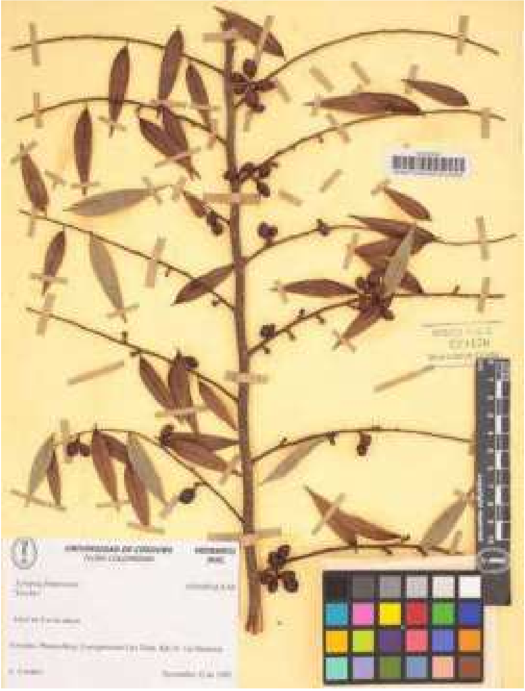

Specimen in herbarium HUC (CC BY-NC-ND 2.0)

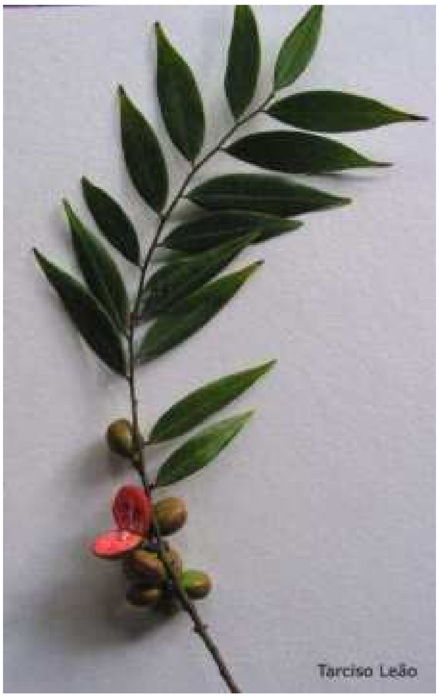

Fruiting branch, Pernambuco, Brazil 2004; "Embira-vermelha" by Tarciso Leão (CC BY-NC 2.0)

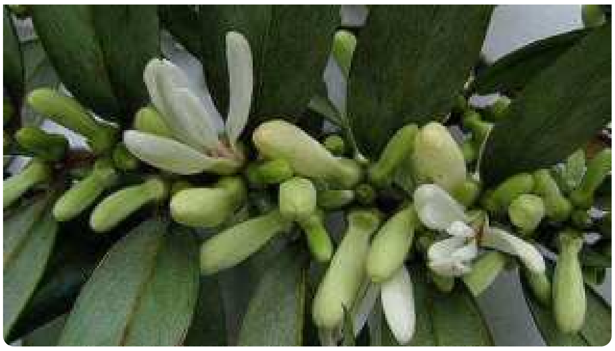

Flowering branch, Bahia, Brazil 2008; by Alex Popovkin (CC BY-NC-SA 2.0)

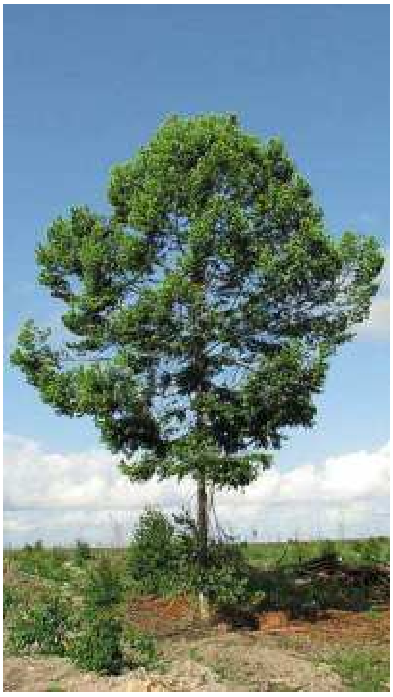

Tree, Bahia, Brazil 2008; by Alex Popovkin (CC BY-NC-SA 2.0)

Medicina Brasiliensi Piso, 1648 Page number 73a

Vernacular name(s) Guabiraba

Species Campomanesia dichotoma (O.Berg) Mattos

Family Myrtaceae

Notes

The woodcut looks different from its corresponding image in the *Theatrum.* The same species is described and depicted in Marcgrave (1648: 117) and the woodcut in that section is also different from the *C. dichotoma* in the *Theatrum*. The woodcut shows three flower buds and one open flower, while the oil painting depicts a fruiting branch. The specimen in Marcgrave's herbarium is a flowering one although there is no strong resemblance between woodcut and exsicata.

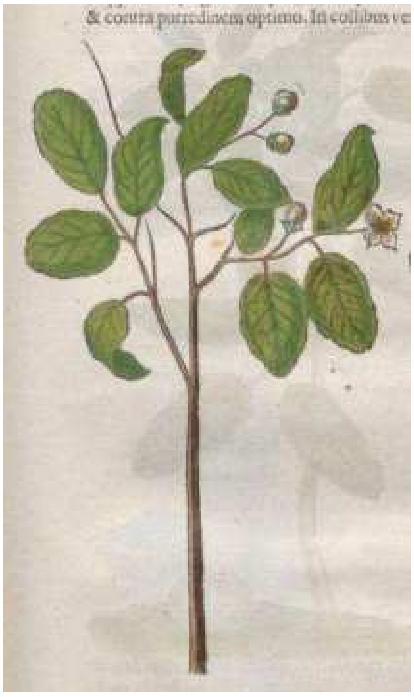

De Facultatibus Simplicium: 73

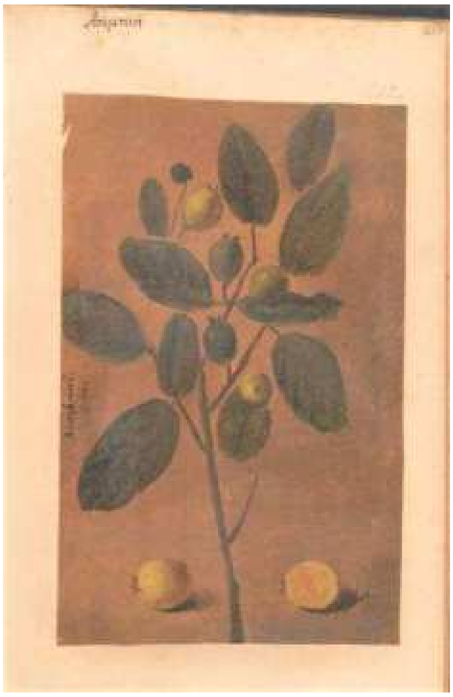

Theatrum Rerum Naturalium bound c. 1660-1664: 217

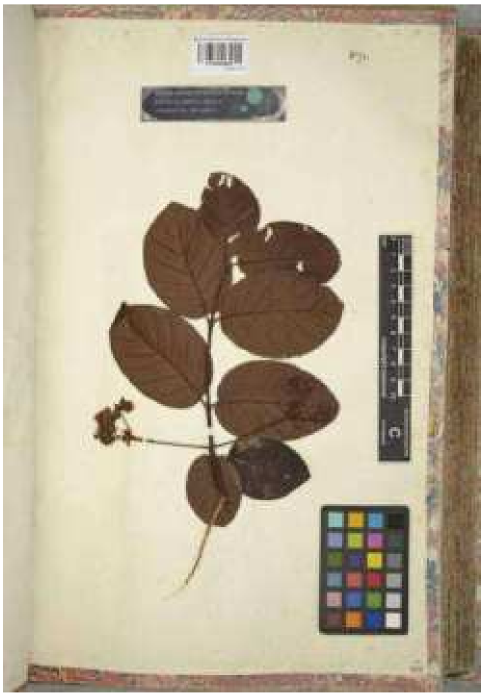

Marcgrave's herbarium: 67

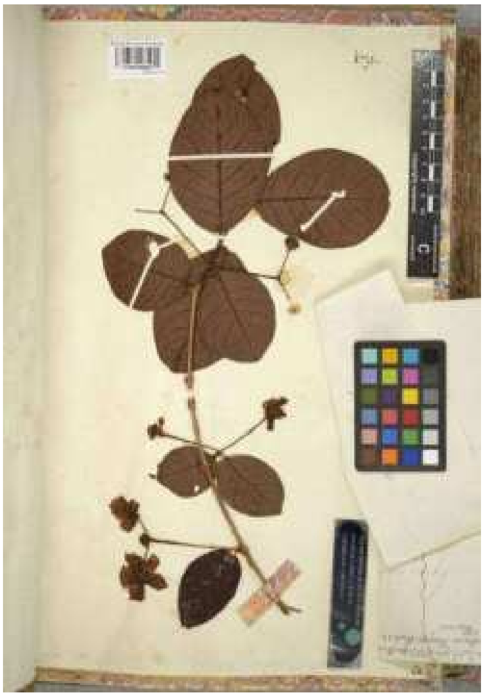

Marcgrave's herbarium 68

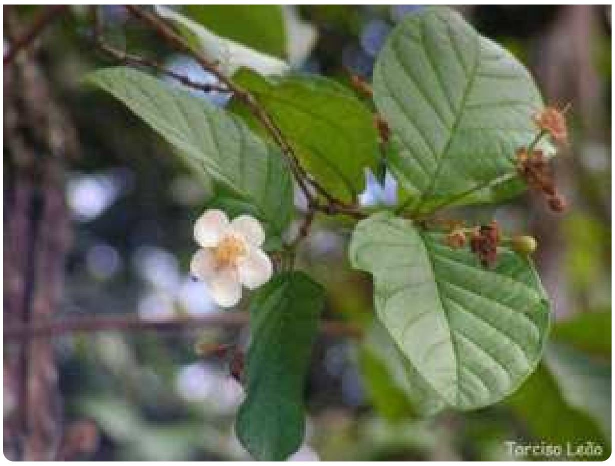

Flowering branch, Pernambuco, Brazil 2004;
"Guabiraba" by Tarciso Leão (CC BY 2.0)

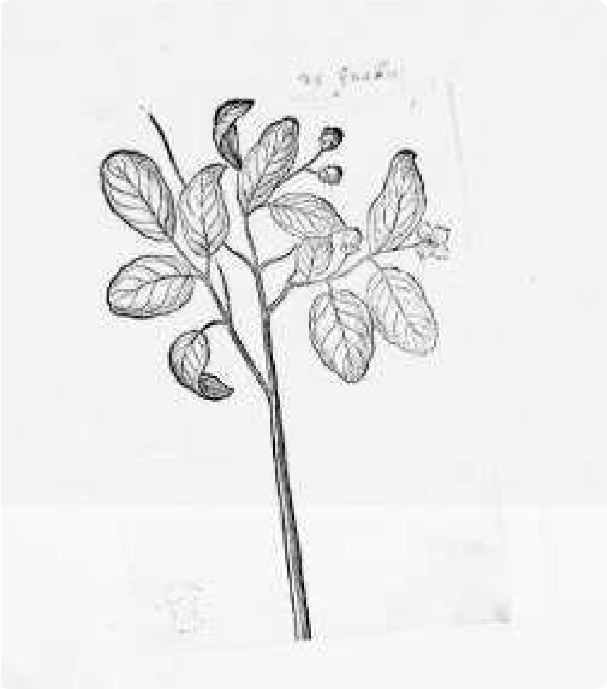

Proof-woodcut in Sloane Ms 1554 f. 65v

Medicina Brasiliensi Piso, 1648 Page number 73b

Vernacular name(s) Guabiraba

Species Campomanesia dichotoma (O.Berg) Mattos

Family Myrtaceae

Notes

The woodcut looks different from its corresponding image in the *Theatrum*. The same species is described and depicted in Marcgrave (1648: 117) and the woodcut in that section is also different to the *C. dichotoma* in the *Theatrum*. The woodcut shows a sterile twig with four leaves, while the oil-painting illustration depicts a fruiting branch. The specimen in Marcgrave's herbarium is a flowering one.

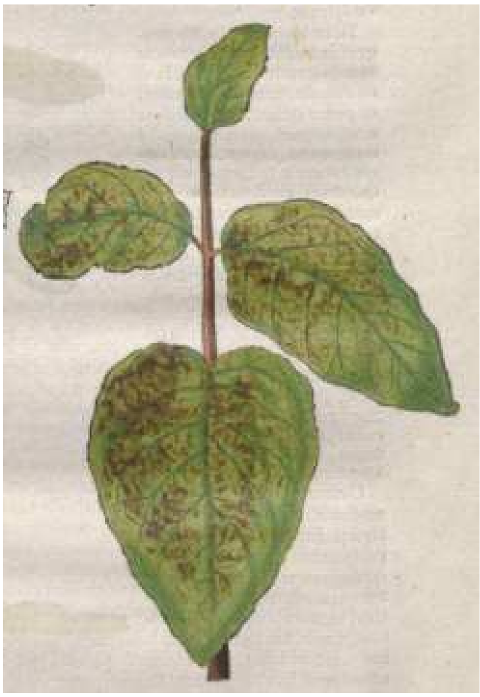

De Facultatibus Simplicium: 73

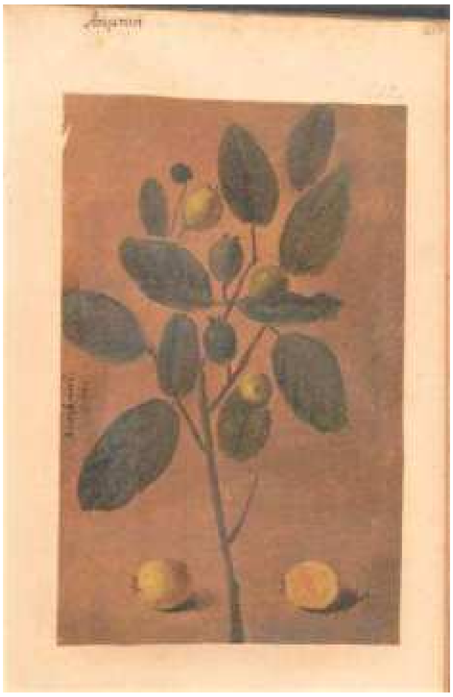

Theatrum Rerum Naturalium bound c. 1660-1664: 217

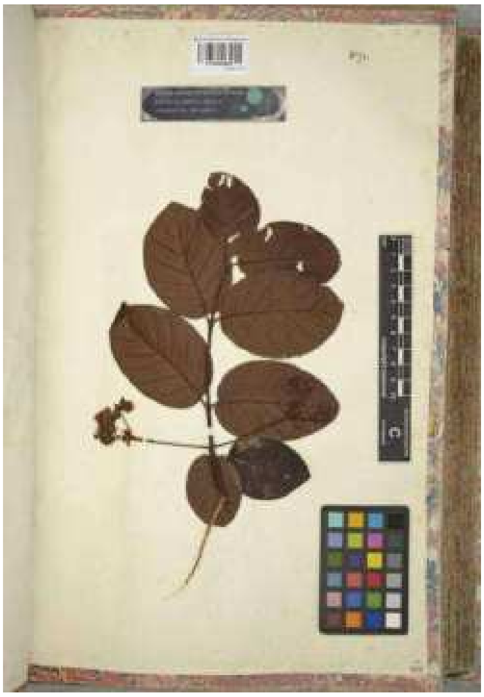

Marcgrave's herbarium: 67

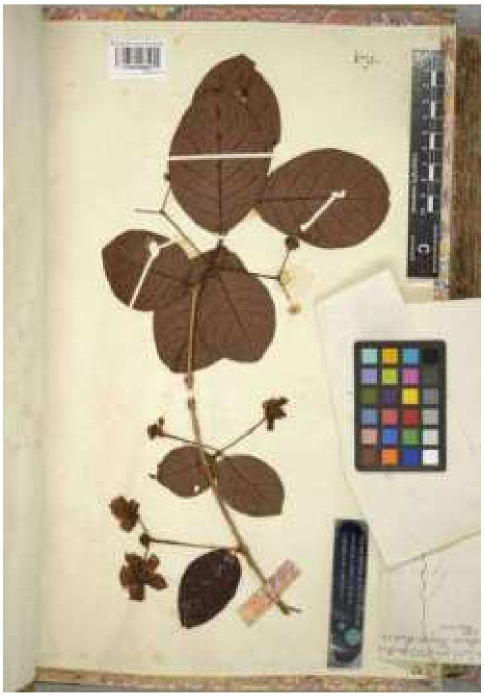

Marcgrave's herbarium: 68

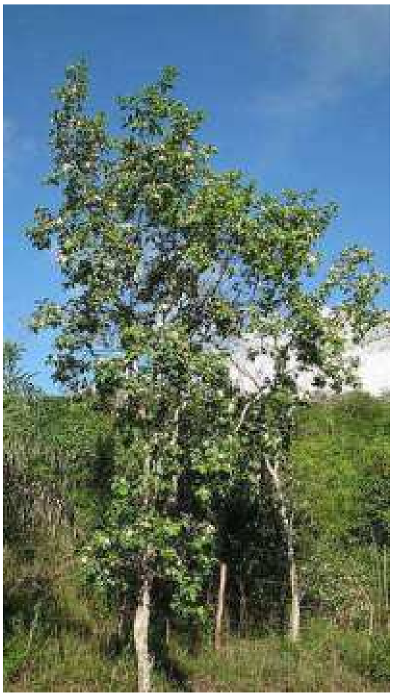

Tree, Bahia 2008; by Alex Popovkin (CC BY 2.0)

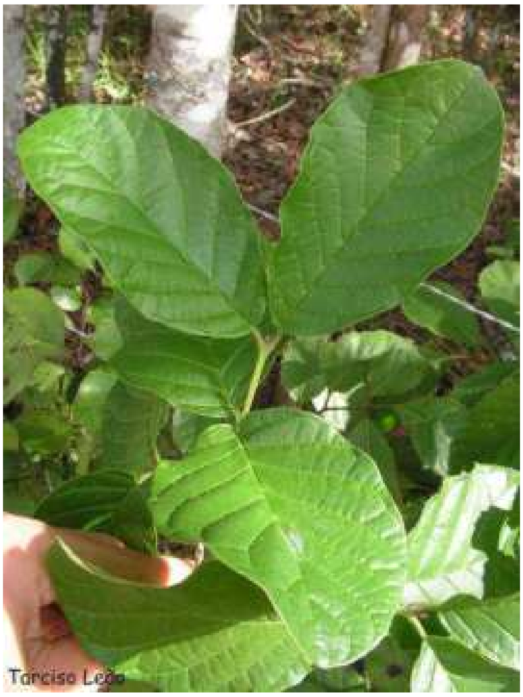

Sterile branch, Pernambuco, Brazil 2004; "Guabiraba" by Tarciso Leão (CC BY 2.0)

Medicina Brasiliensi Piso, 1648 Page number 74

Vernacular name(s) Araça iba. Araça miri

Species <u>Psidium guineense Sw.</u>

Family Myrtaceae

Notes

The woodcut is very similar to the *Theatrum* image (non-reversed), although one small fruit does not appear in the woodcut (left and upper corner). The fruit of *P. guineense* can be found in the *Misc. Cleyeri* and in the still-life painting by Eckhout (Brazilian fruits) that was based on the chalk drawing.

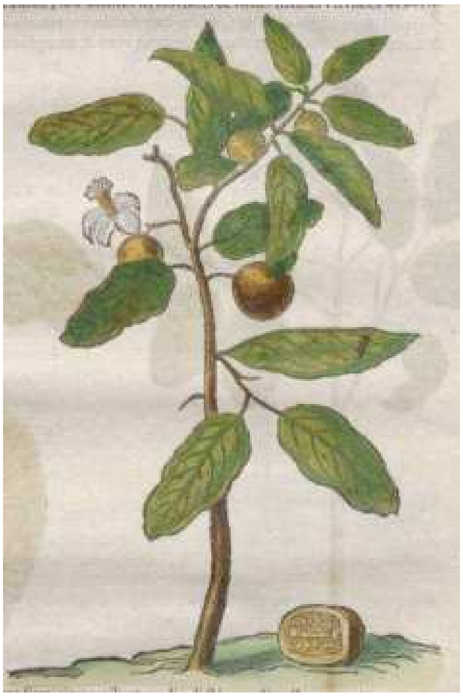

De Facultatibus Simplicium: 74

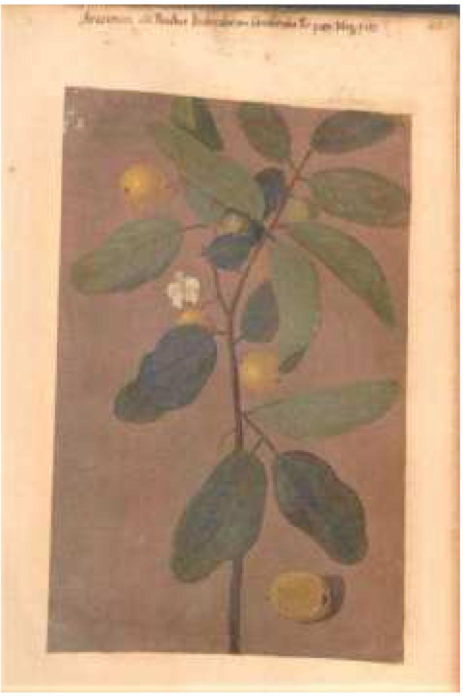

Theatrum Rerum Naturalium bound c. 1660-1664: 215

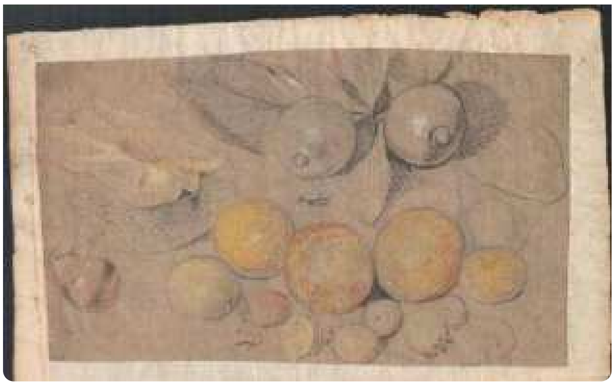

Miscellanea Cleyeri c.1637-44: 51r

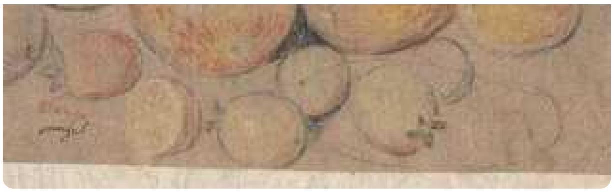

Miscellanea Cleyeri c.1637-44: 51r

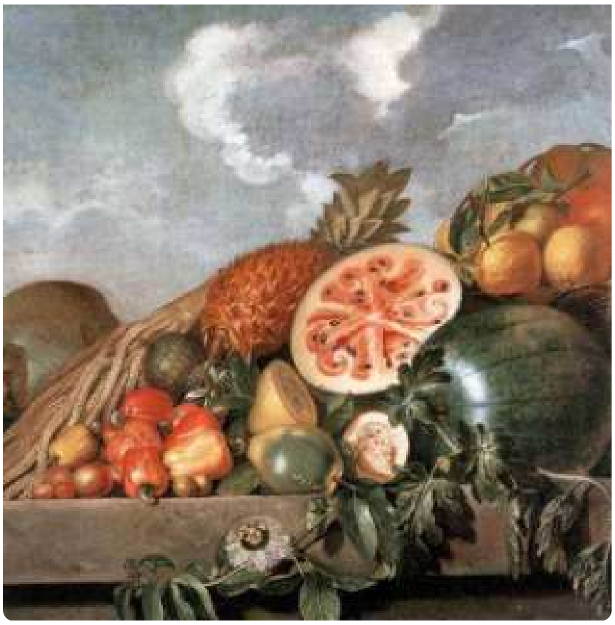

Eckhout still-life (Brazilian fruits)

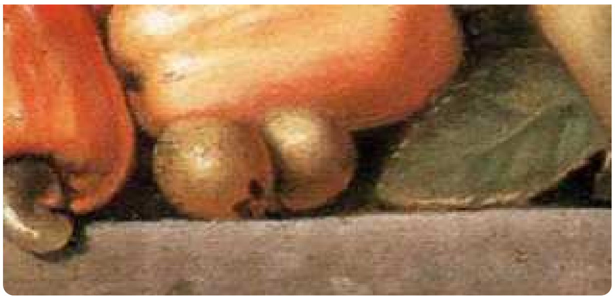

Eckhout still-life (Brazilian fruits)

Medicina Brasiliensi Piso, 1648 Page number 77

Vernacular name(s) Abaremo temo

Species Abarema cochliocarpos (Gomes) Barneby & J.W.Grimes

Family Fabaceae

Notes

The woodcut is very similar to the *Theatrum* image (non-reversed).

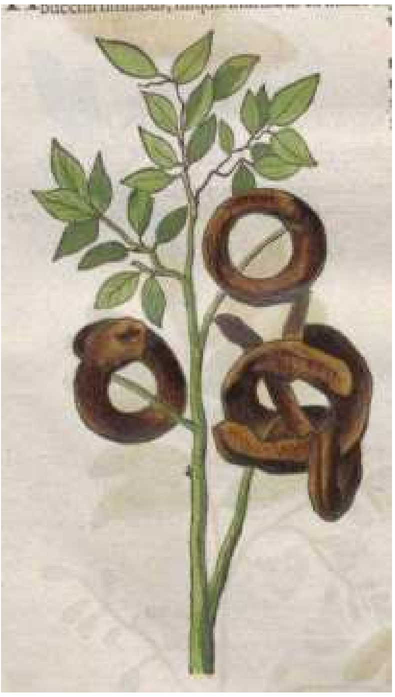

De Facultatibus Simplicium: 77

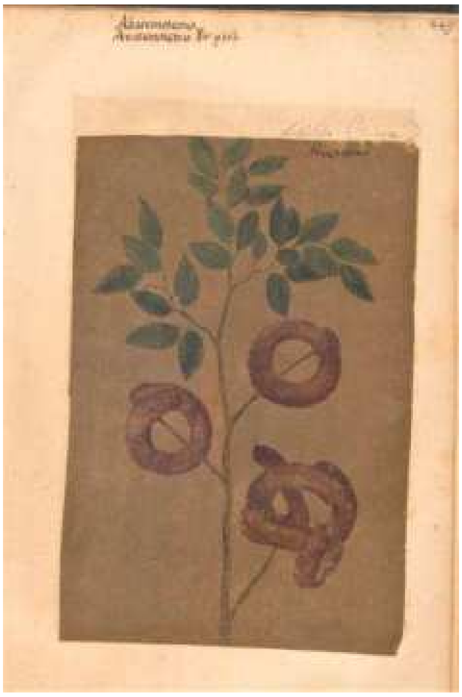

Theatrum Rerum Naturalium bound c. 1660-1664: 249

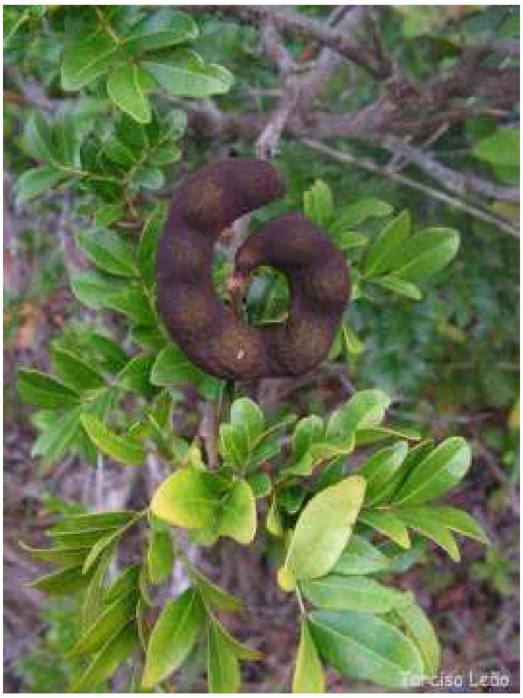

Photo by Tarciso Leão (CC BY 2.0)

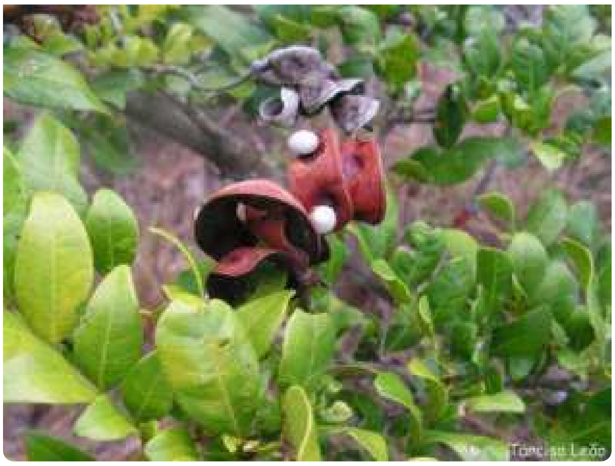

Photo by Tarciso Leão (CC BY 2.0)

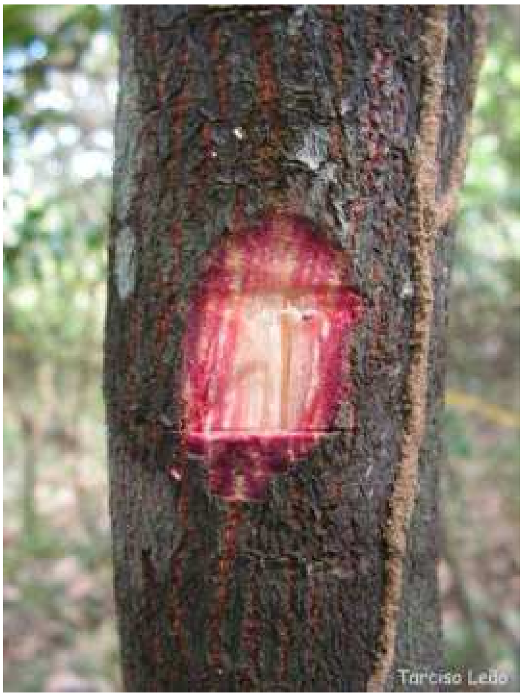

Trunk, Paraíba, Brazil 2004; by Tarciso Leão (CC BY 2.0)

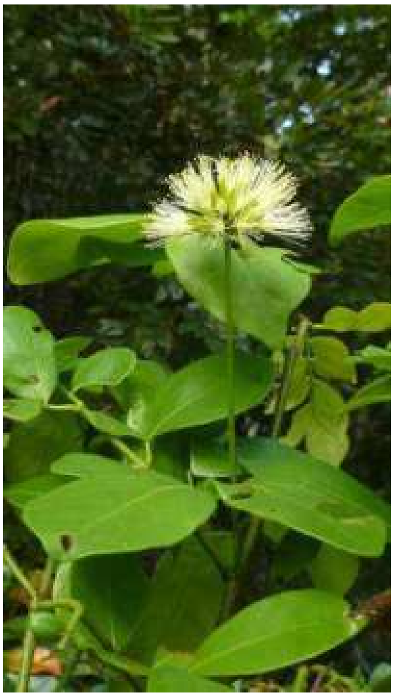

Flowering branch, Bahia, Brazil; by Alex Popovkin (CC BY 2.0)

Medicina Brasiliensi Piso, 1648 Page number 80a

Vernacular name(s) Jito. Urape guaçu

Species Trichilia hirta L.

Family Meliaceae

Notes

The woodcut resembles the pencil drawing found in De Laet's manuscript, although is not exactly the same. We did not find a voucher of this species in Marcgrave's herbarium but based on the flat and two-dimensional print of the leaves, the woodcut could have been made after an exsicata (see a fruiting specimen in S2).

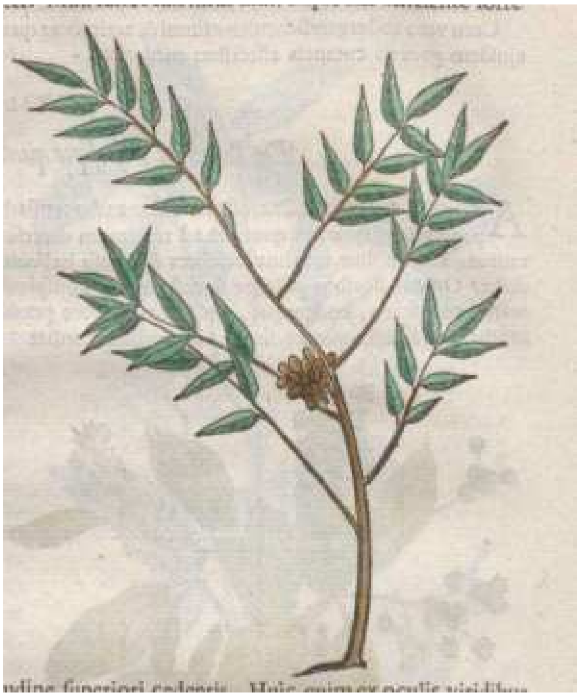

De Facultatibus Simplicium: 80

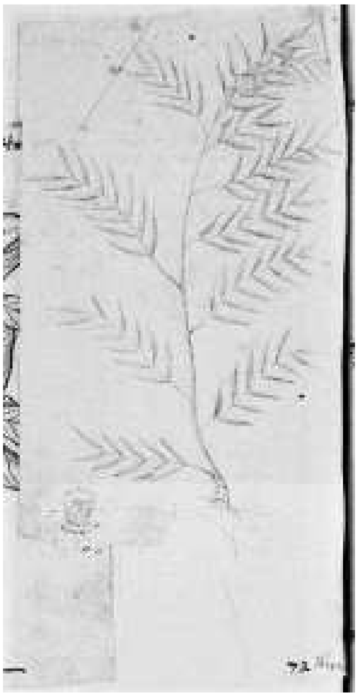

Pencil drawing in Sloane Ms 1554 f. 77v

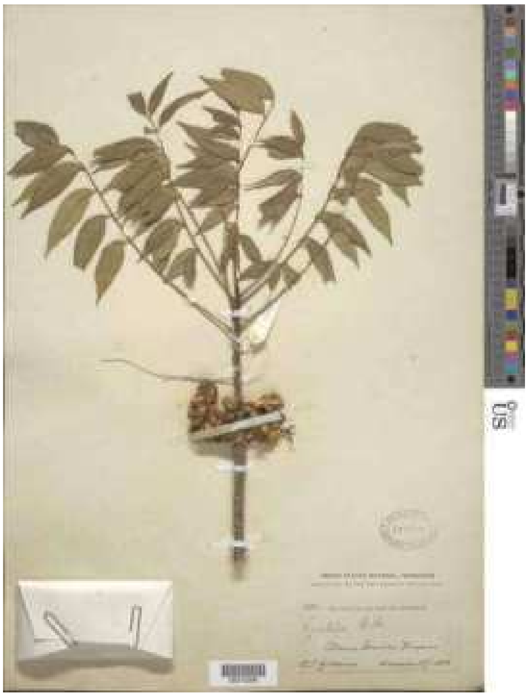

Photo by Edward A. Goldman (CC0 1.0)

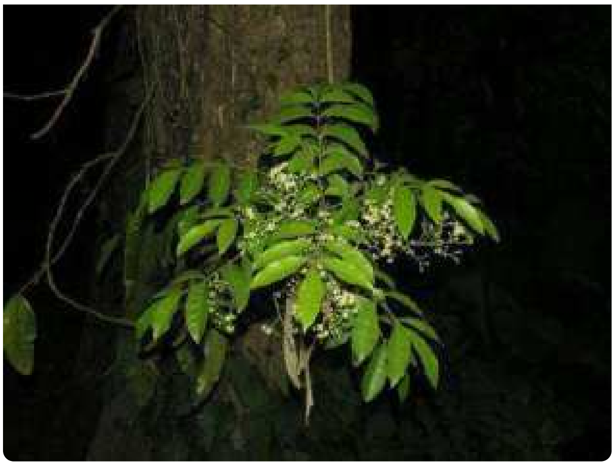

Photo by Reinaldo Aguilar (CC BY-NC-SA 2.0)

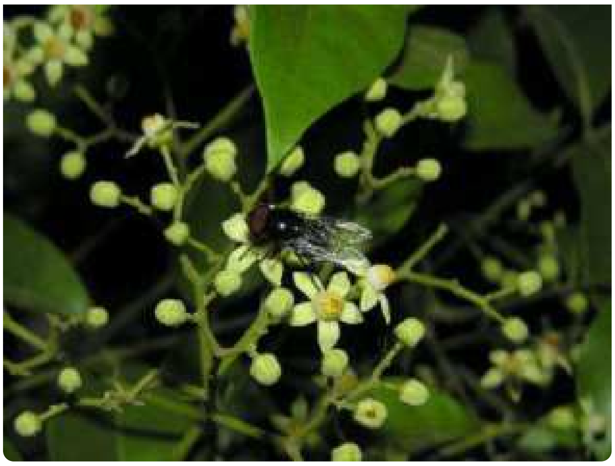

Photo by Reinaldo Aguilar (CC BY-NC-SA 2.0)

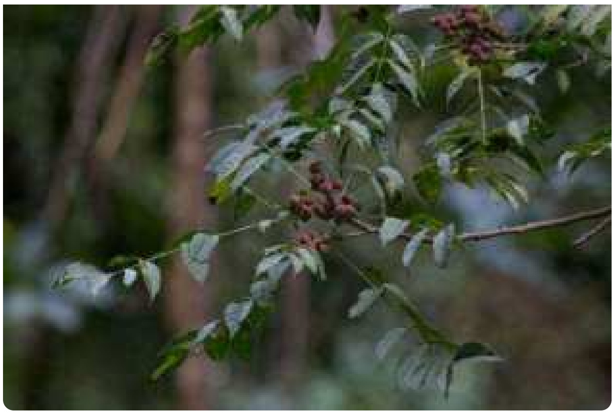

Fruiting branch, botanical garden Cupaynicú in Cuba; by Christian Pirkl (CC BY-SA 4.0)

Medicina Brasiliensi Piso, 1648 Page number 81a

Vernacular name(s) Zamouna. Cabireiba

Species Ceiba erianthos (Cav.) K.Schum.

Family Malvaceae

Notes

We did not find any correspondence between this woodcut and the contemporary or older sources. This is the image of three palmatisect leaves, characteristic of *C. erianthos*, although the drawing does not show the prickles at the stem. Piso adopted the woodcut in his book (Piso 1658: 175), showing the spiny trunk that thickens at the base, the reason why is called "barriguda" [pot-belly] in Brazil). Piso probably did not see the impressive flowers and fruits, which appear in the dry season after the leaves have fallen (Pickel 2008: 146).

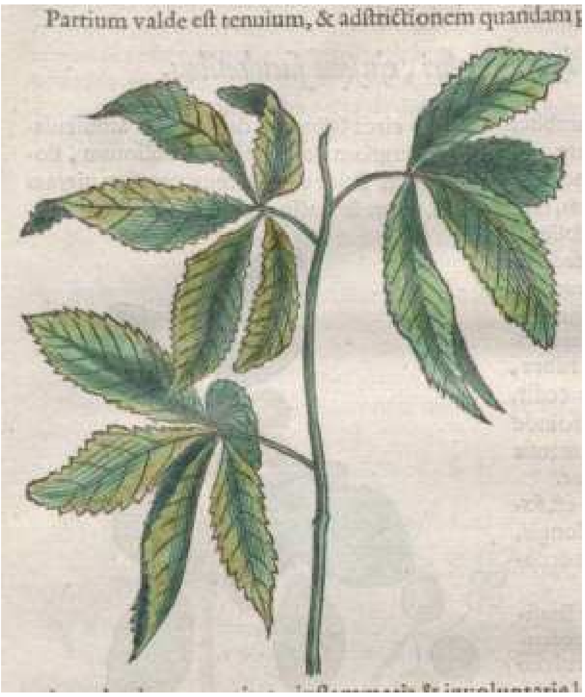

De Facultatibus Simplicium: 81

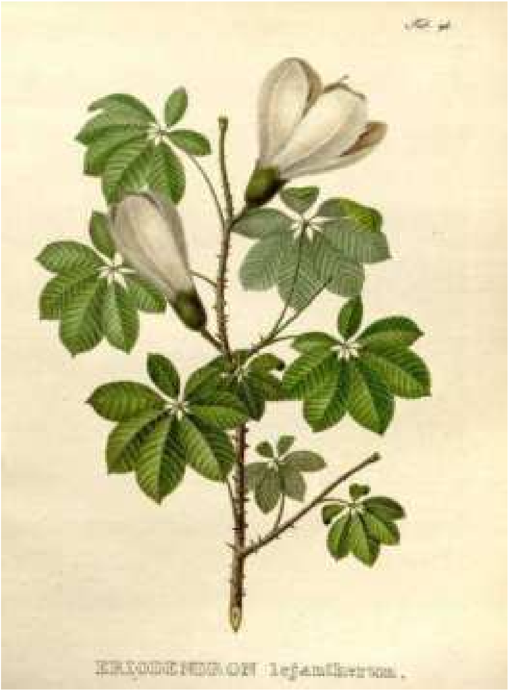

Nova genera et species plantarum. Monachi(1824 -1829) Biodiversity Heritage Library (CC-BY-SA 2.0)

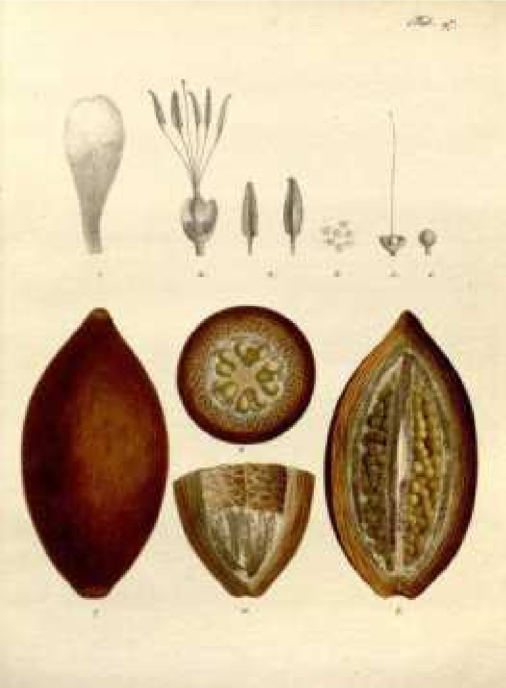

Nova genera et species plantarum. Monachi(1824 -1829) Biodiversity Heritage Library (CC-BY-SA 2.0)

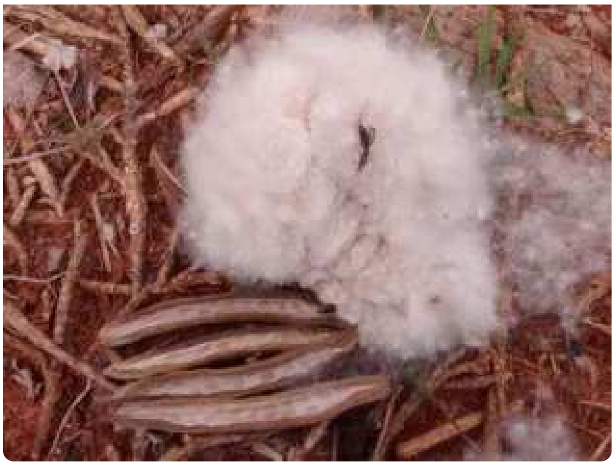

Fruit, São Paulo, Brazil 2017; by Jorge EFO Silva (CC BY-SA 4.0)

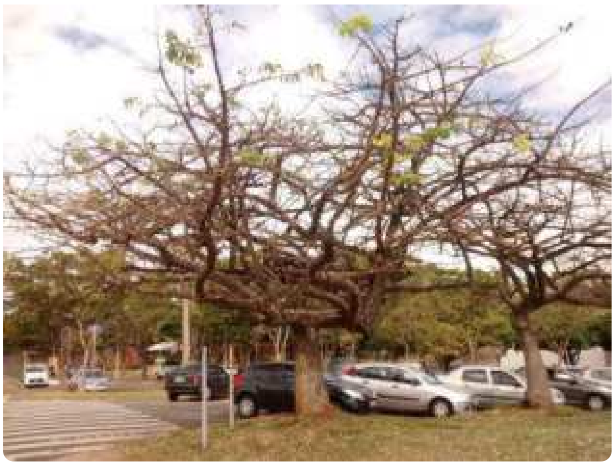

Tree, São Paulo, Brazil 2017; by Jorge EFO Silva (CC BY-SA 4.0)

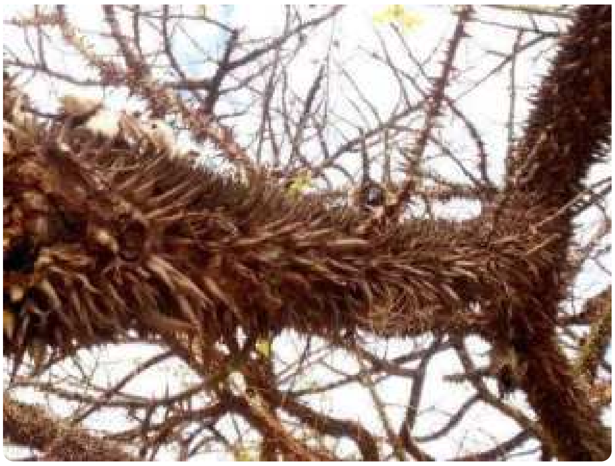

Trunk, São Paulo, Brazil 2017; by Jorge EFO Silva (CC BY-SA 4.0)

Medicina Brasiliensi Piso, 1648 Page number 81b

Vernacular name(s): Andira. Ibaiariba. Angelin

Species: Andira fraxinifolia Benth.

Family: Fabaceae

Notes

The woodcut is very similar to the *Theatrum* image (non-reversed). The *Theatrum* depicts one more compound leaf and a smaller and less dense inflorescence. The "imperfection" of the broken or bitten leaf in the upper left, resembles the lifelike style of botanical illustrations of Renaissance botanist Otto Brunfels (1488-1534) and artist Hans Weiditz (1495-1537). However, combining flowering and fruiting stages in the same drawing recalls the engravings of his contemporary Leonhart Fuchs (1505-1566). Fuchs aimed for a realistic look but also showed a complete botanical portrait, which allowed identification of the plant in any season.

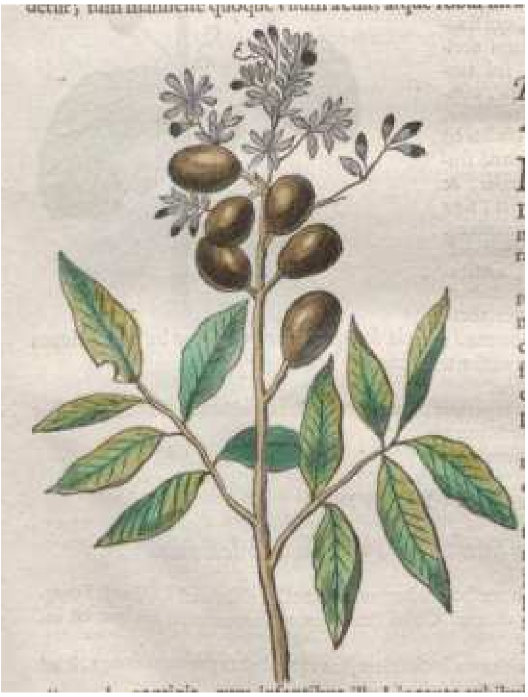

De Facultatibus Simplicium: 81

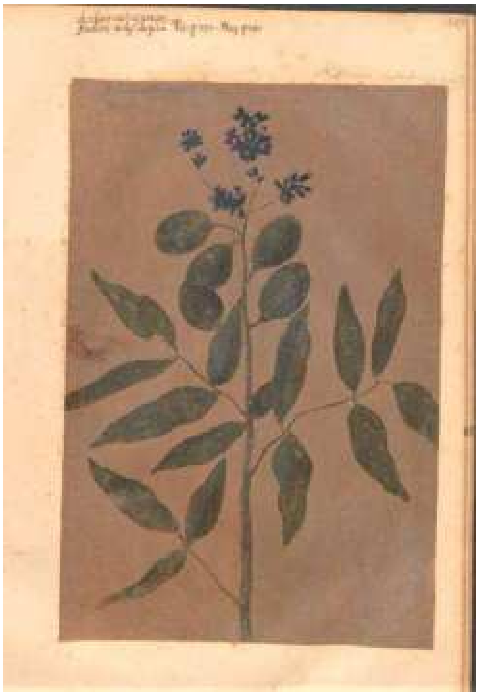

Theatrum Rerum Naturalium bound c. 1660-1664: 489

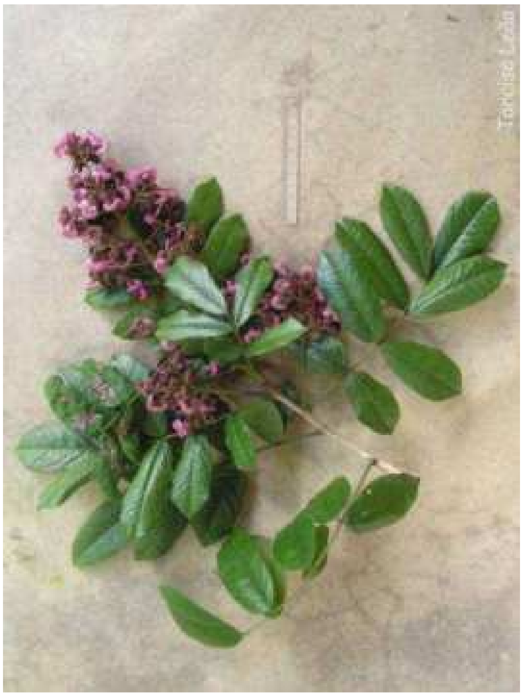

Flowering branch. Photo by Tarciso Leão (CC BY 2.0)

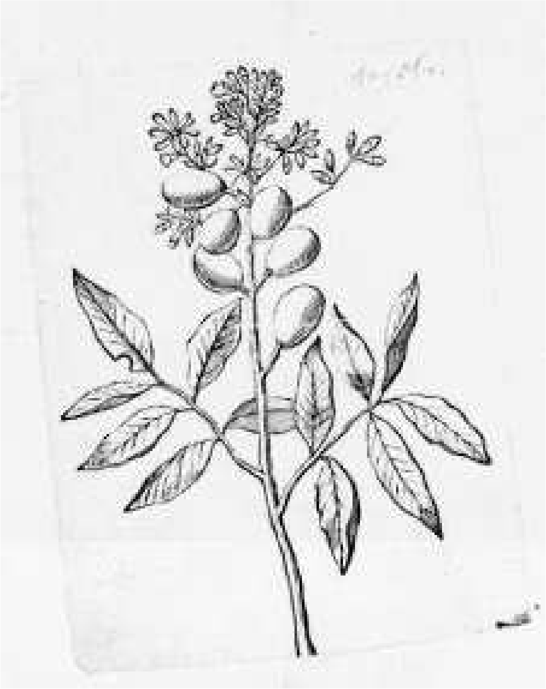

Proof-woodcut in Sloane Ms 1554 f. 73v

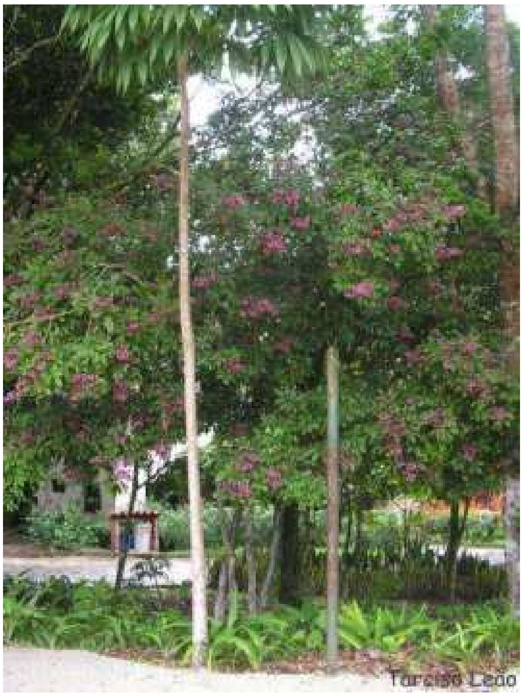

Flowering tree, Pernambuco 2004; "Angelim" by Tarciso Leão (CC BY-NC-SA 2.0)

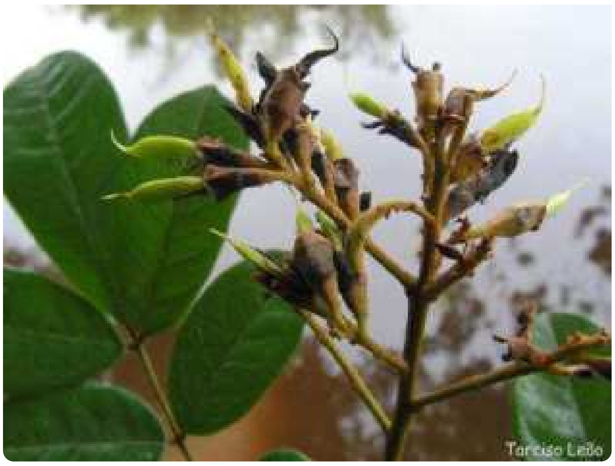

Inmature fruits, "Angelim" by Tarciso Leão (CC BY 2.0)

Medicina Brasiliensi Piso, 1648 Page number 82

Vernacular name(s) Cambui. Myrto silvestris

Species <u>Eugenia involucrata DC.</u>

Family Myrtaceae

Notes

Details of two broken leaves are depicted, as well as flowering and fruiting stages together in one image (see *A. fraxinifolia*). There is a pencil drawing in De Laet's manuscript, in reversed format, which was most likely used to create the woodcut (Whitehead and Boeseman 1989). If we look closer, we can read "blom wit" (white flower in Dutch) and something about a "rot" fruit (red fruit in German). The color of the stem and leaves is indicated as "grún" (green in German).

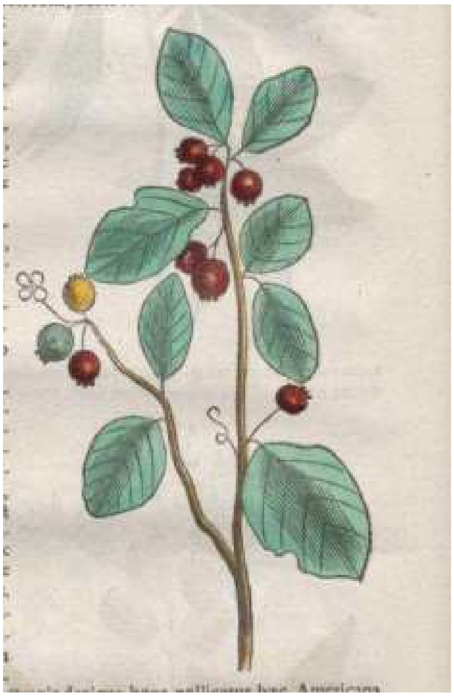

De Facultatibus Simplicium: 82

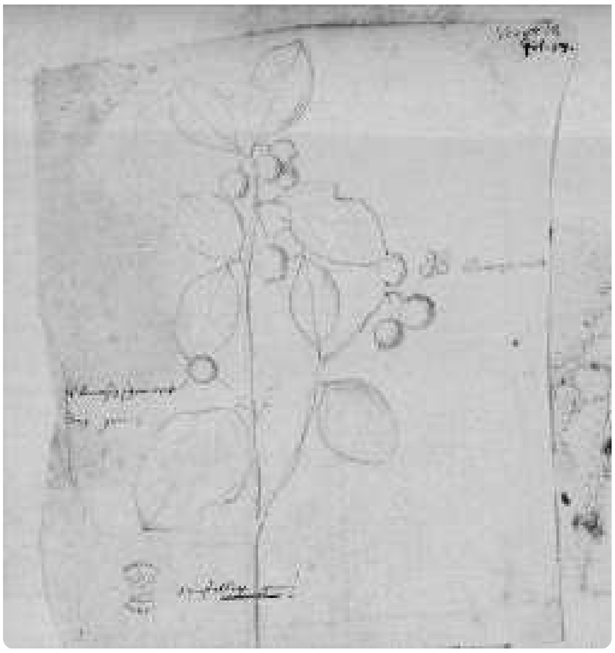

Pencil lead drawing in Sloane Ms 1554 f. 60v

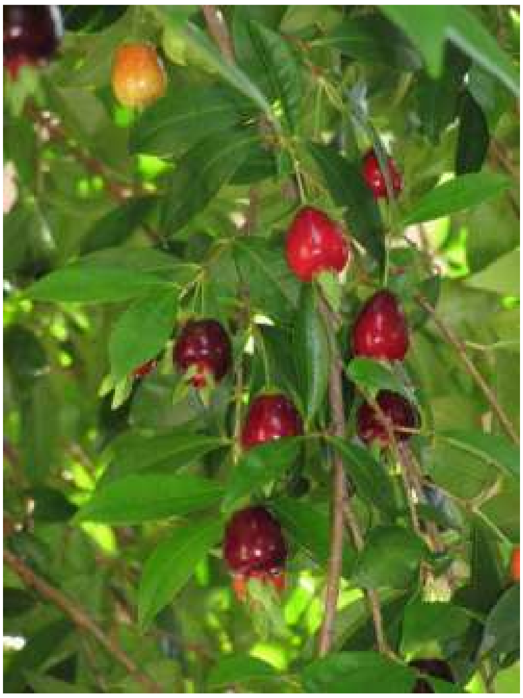

Fruiting branch

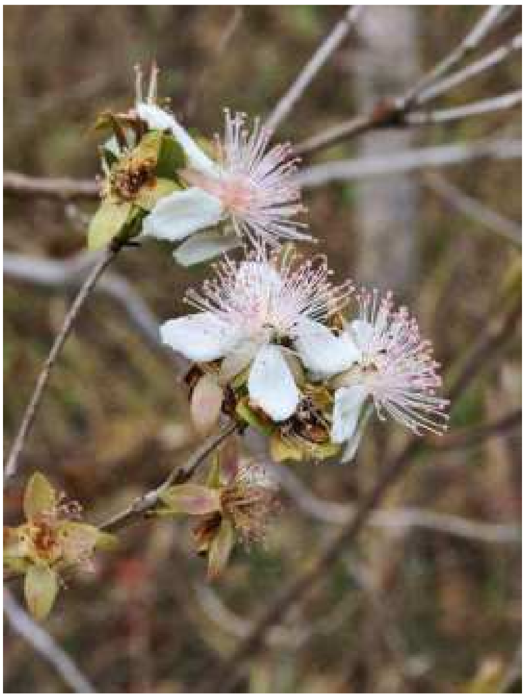

Flowering branch, Brazília, Brazil 2010; by Mauricio Mercadante (CC BY-NC-SA 2.0)

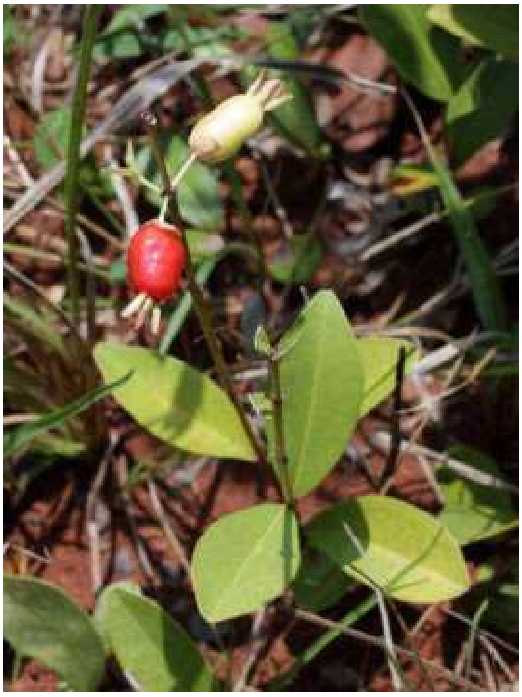

Fruiting branch, Brasília, Brazil 2010; by Mauricio Mercadante (CC BY 2.0)

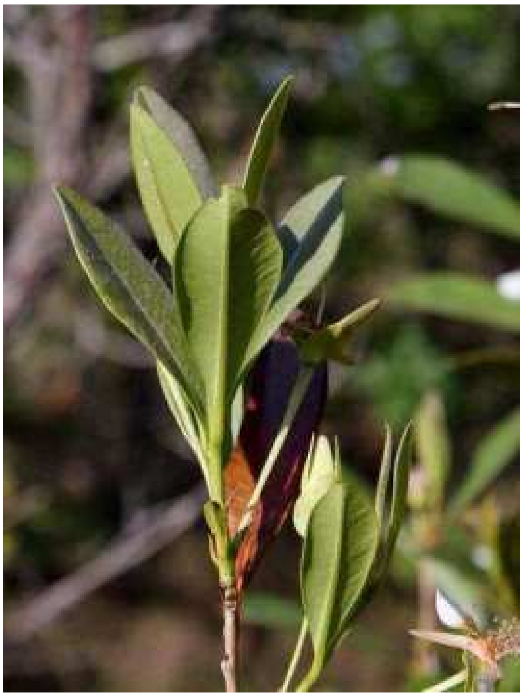

Branch and detail of venation, Brasília, Brazil 2010; by Mauricio Mercadante (CC BY 2.0)

Medicina Brasiliensi Piso, 1648 Page number 84b

Vernacular name(s) Iuripeba minor

Species Solanum paludosum Moric

Family Solanaceae

Notes

We did not find any correspondence between this woodcut and the contemporary or older sources. The figure represents a sterile branch.

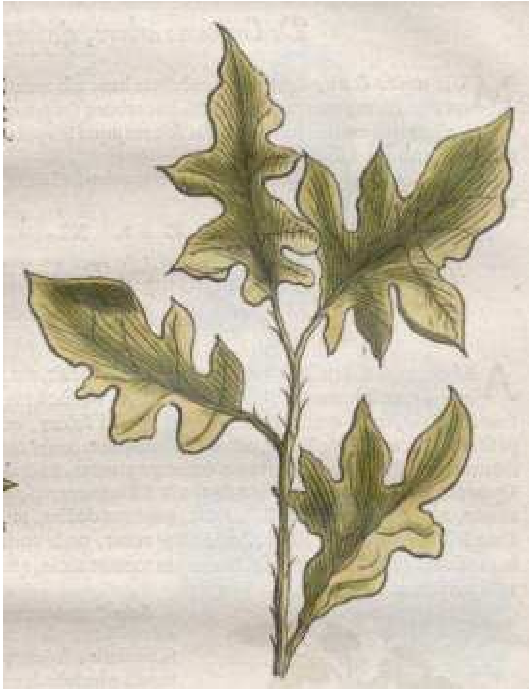

De Facultatibus Simplicium: 84

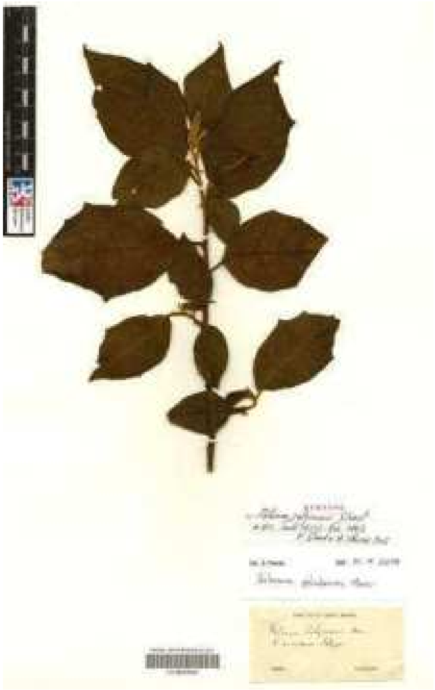

Collected by: Salzmann / ID : LYJB005581" by Jardin Botanique Lyon (CC BY-NC-SA 2.0)

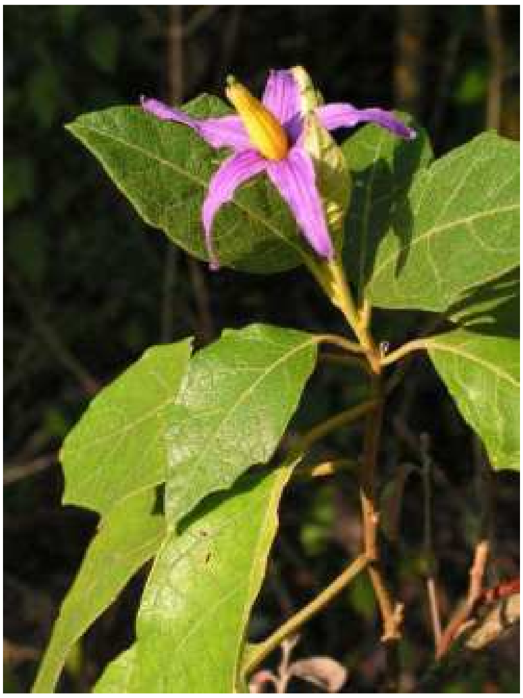

Flowering branch, Paraíba, Brazil; by Tarciso Leão (CC BY 2.0)

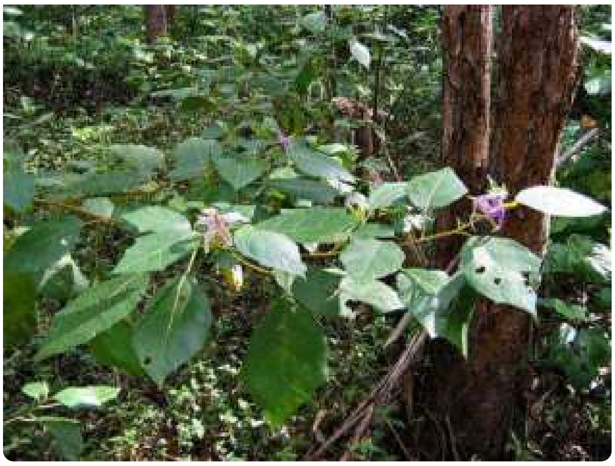

Flowering shrub, Bahia, Brazil 2007; by Alex Popovkin (CC BY-NC-SA 2.0)

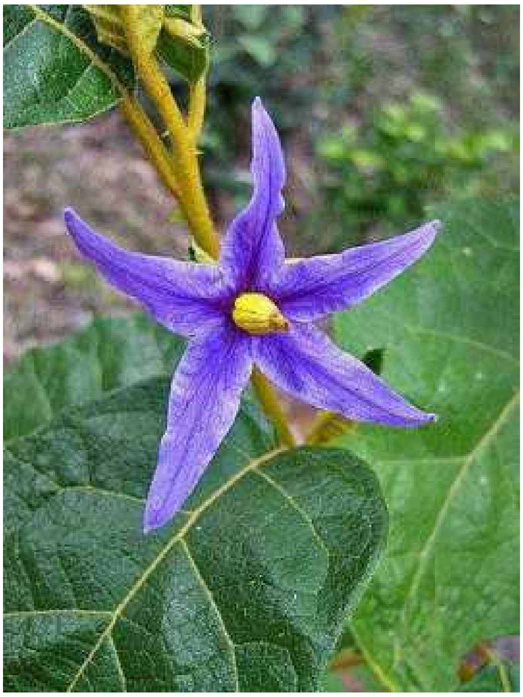

Detail of flower, Bahia, Brazil 2007; by Alex Popovkin (CC BY-NC-SA 2.0)

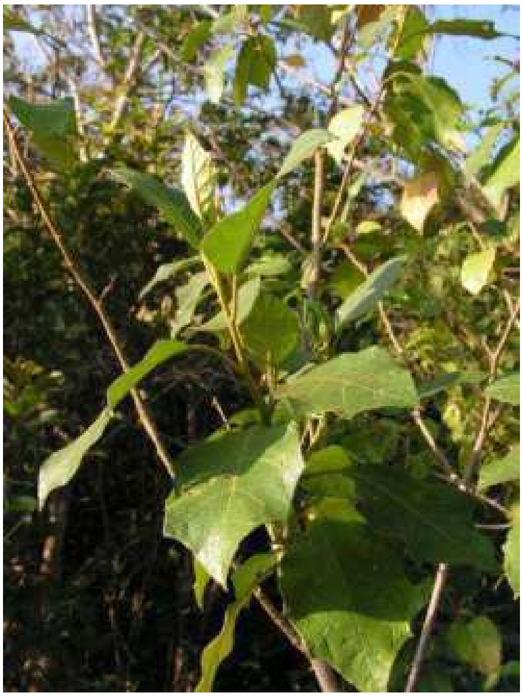

Sterile branch, Paraíba, Brazil; by Tarciso Leão (CC BY 2.0)

Medicina Brasiliensi Piso, 1648 Page number 85a

Vernacular name(s) Mamanga. Lava pratos

Species Senna latifolia (G.Mey.) H.S. Irwin & Barneby

Family Fabaceae

Notes

The woodcut could have been elaborated after a living specimen that ultimately ended up in Marcgrave's herbarium. However, this is hard to prove because the exsicata has a pod and one loose flower, while the woodcut shows a flowering branch, full of leaves and with a certain three-dimensionality.

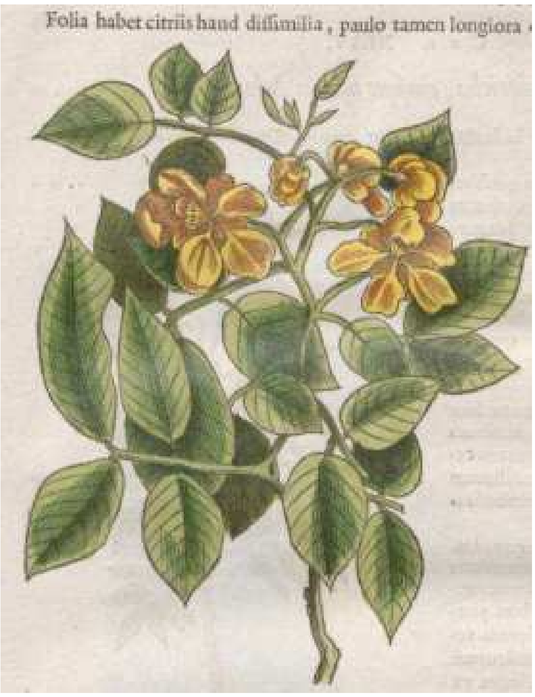

De Facultatibus Simplicium: 85

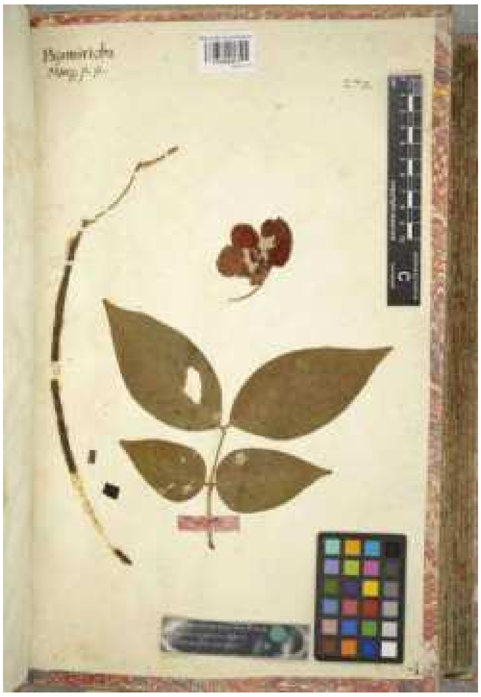

Marcgrave's herbarium: 71

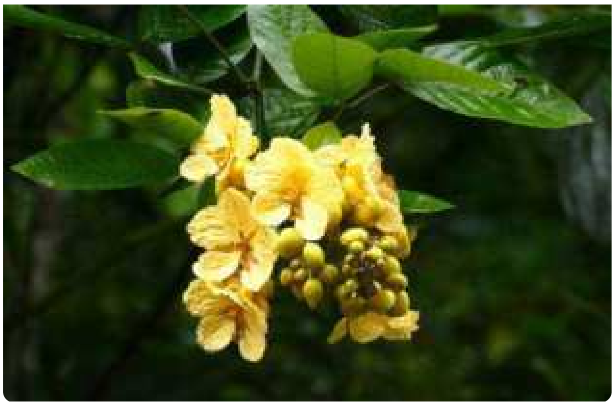

Flowering branch, French Guyana 2018; by Bernard Dupont (CC BY-NC-SA 2.0)

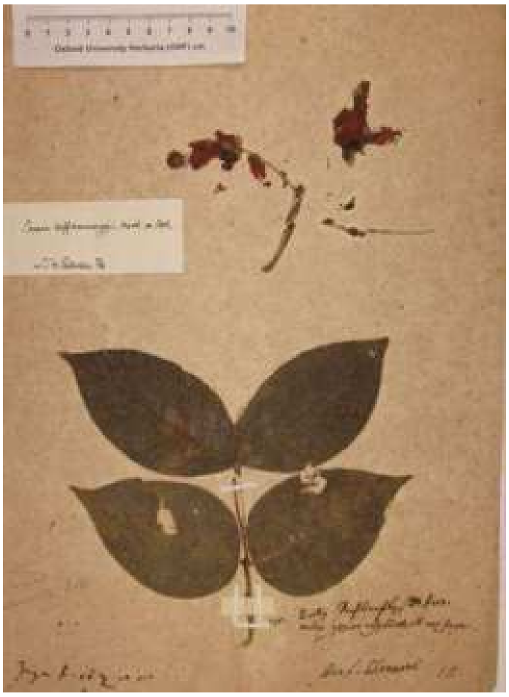

Specimen collected by Marcgrave, kept in Sherard herbaria (f. 15) in Oxford. Retrieved from https://herbaria.plants.ox.ac.uk/bol/sherard

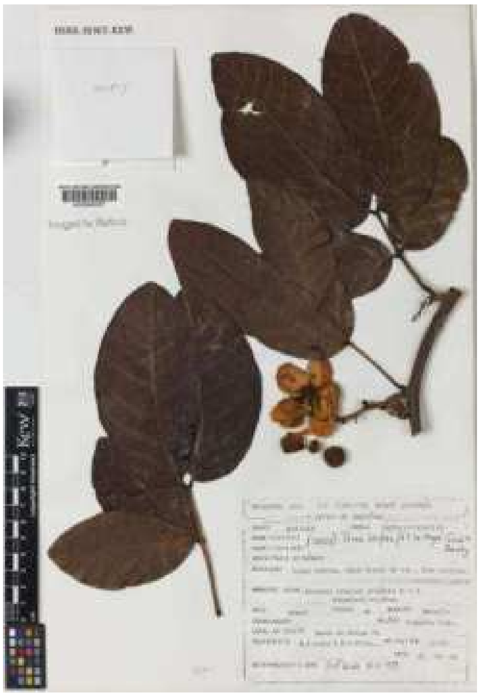

A specimen from Kew's Herbarium - K000840287 retrieved from Plants of the world online

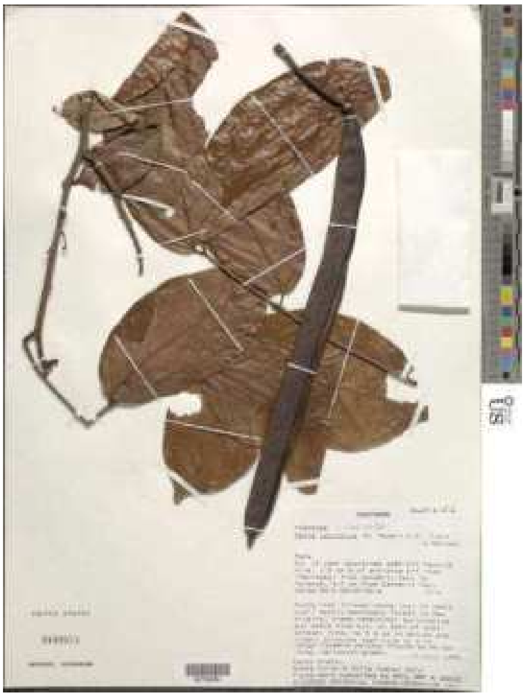

Specimen from Suriname 1995: 2316; by Randy Evans; Steven Corso and Sylva Koemar; Public Domain

Medicina Brasiliensi Piso, 1648 Page number 92b

Vernacular name(s) Tangaraca. Erva do rato

Species Palicourea bracteocardia (DC.) Delprete & J.H.Kirkbr.

Family Rubiaceae

Notes

The woodcut is moderately similar to the *Theatrum* illustration. Interesting to notice is that the artist who colored the HNB copy made the sepals green. The *Theatrum* shows a more accurate drawing, as in nature the sepals are reddish or purplish, just like the petals of the plant.

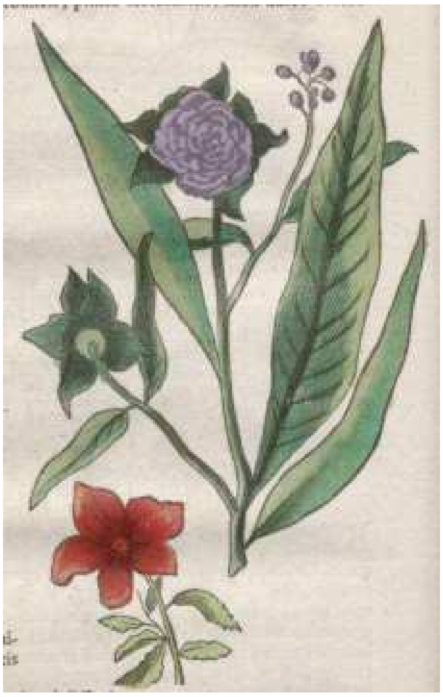

De Facultatibus Simplicium: 92

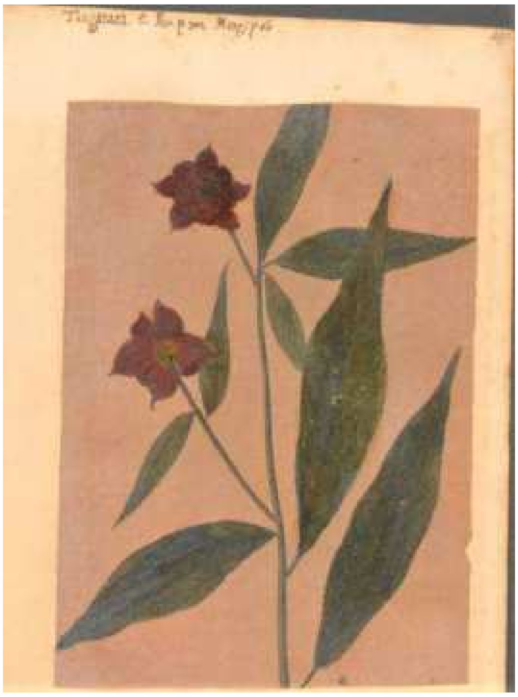

Theatrum Rerum Naturalium bound c. 1660-1664: 197

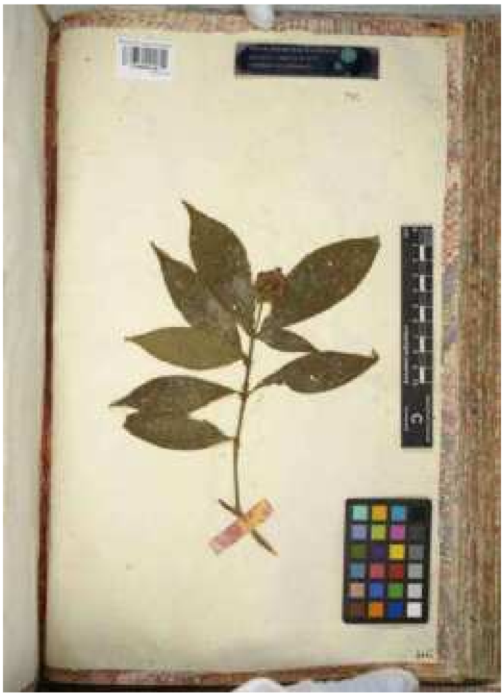

Marcgrave's herbarium: 146

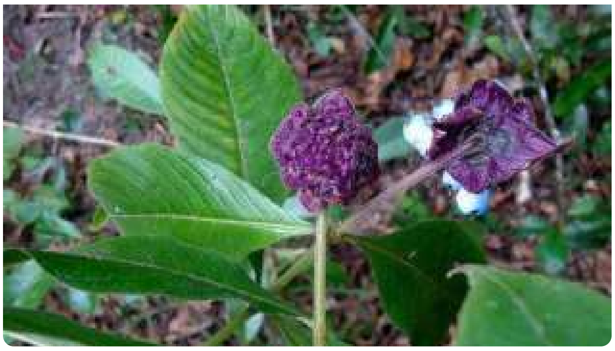

Flowering branch, Bahia, Brazil 2012; by Alex Popovkin (CC BY-NC-SA 2.0)

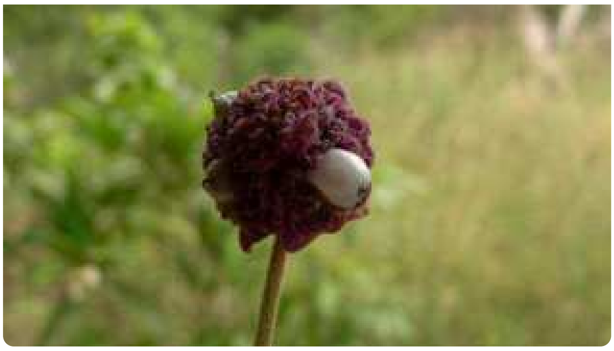

Emerging fruit, Bahia, Brazil 2012; by Alex Popovkin (CC BY-NC-SA 2.0)

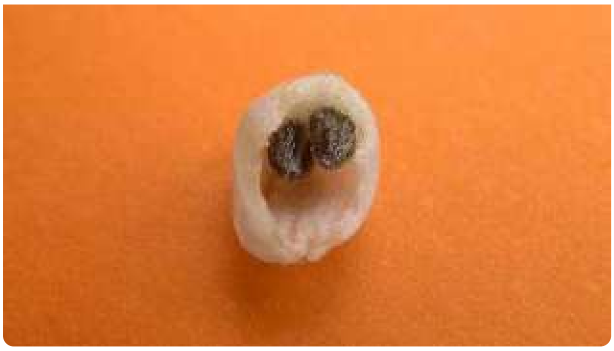

Fruit open with seeds, Bahia, Brazil 2012; by Alex Popovkin (CC BY-NC-SA 2.0)

Medicina Brasiliensi Piso, 1648 Page number 92c

Vernacular name(s) Tangaraca. Erva do rato

Species Palicourea bracteocardia (DC.) Delprete & J.H.Kirkbr.

Family Rubiaceae

Notes

This woodcut does not match the illustration, but it could have been made after an exsicata, based on the two-dimensional plane that is depicted. The specimen in Marcgrave's herbarium does not bear much resemblance to the woodcut.

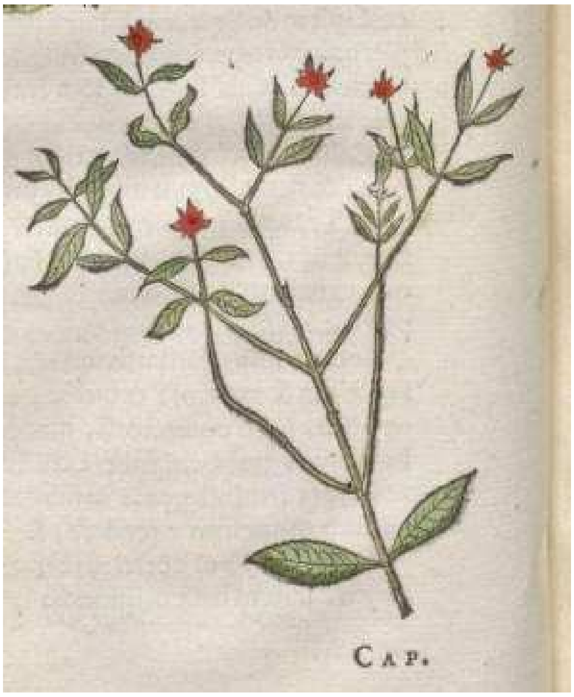

De Facultatibus Simplicium: 92

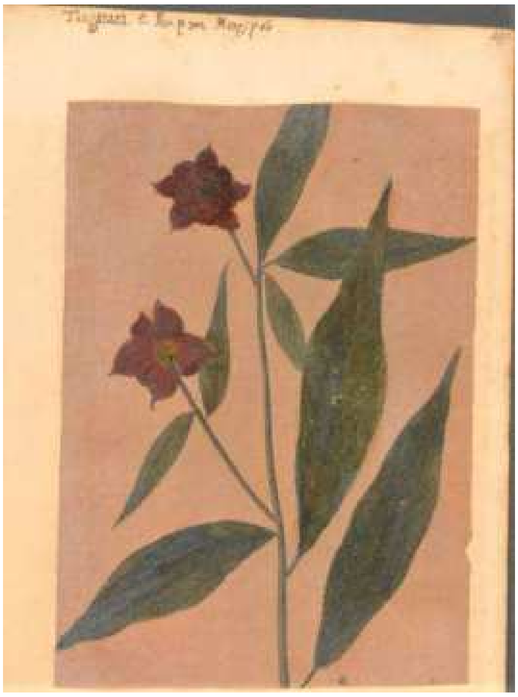

Theatrum Rerum Naturalium bound c. 1660-1664: 197

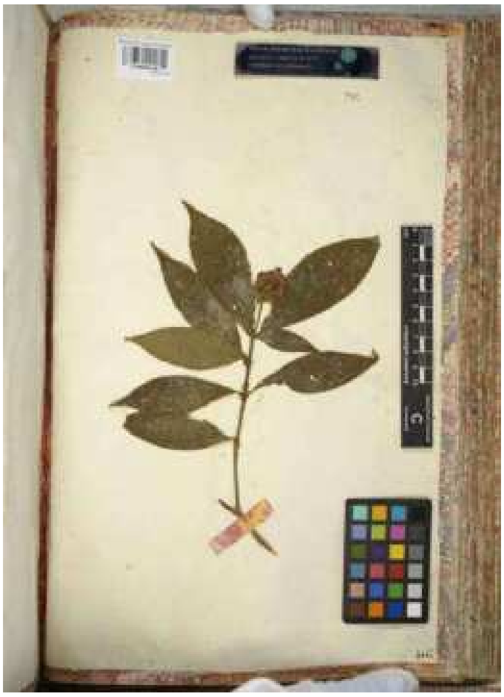

Marcgrave's herbarium: 146

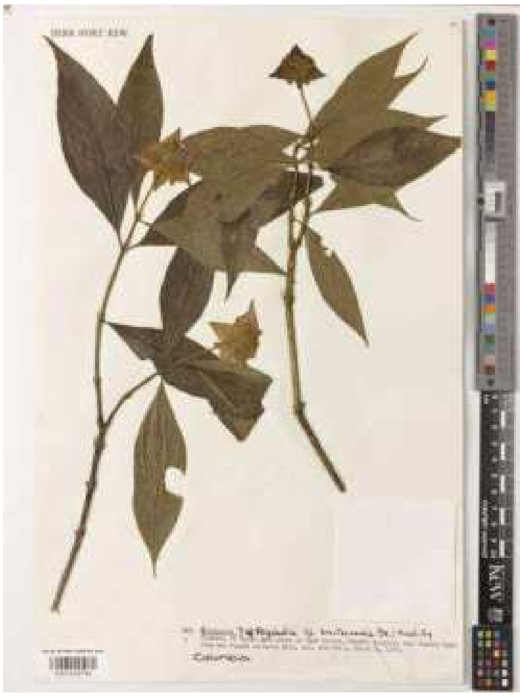

A specimen from Kew's Herbarium - K001429799

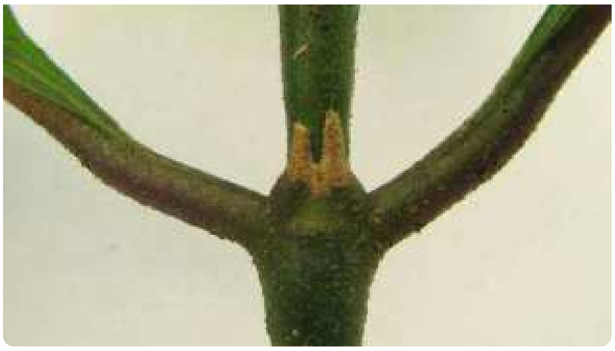

Stipules, Bahia, Brazil 2009; by Alex Popovkin (CC0 1.0)

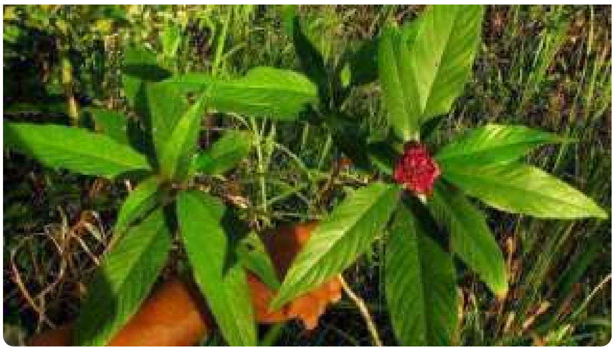

Flowering branch, Bahia, Brazil 2009; by Alex Popovkin 2012 (CC BY-NC-SA 2.0)

Medicina Brasiliensi Piso, 1648 Page number 97a

Vernacular name(s) Iaborandi

Species Piper anisum (Spreng.) Angely

Family Piperaceae

Notes

The woodcut is very similar to the *Theatrum* image (non-reversed).

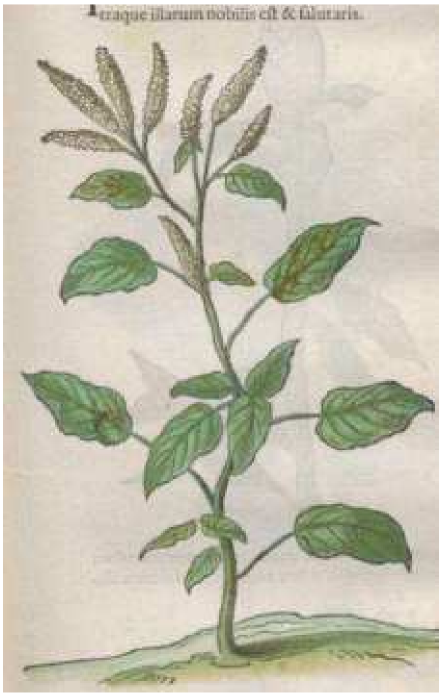

De Facultatibus Simplicium: 97

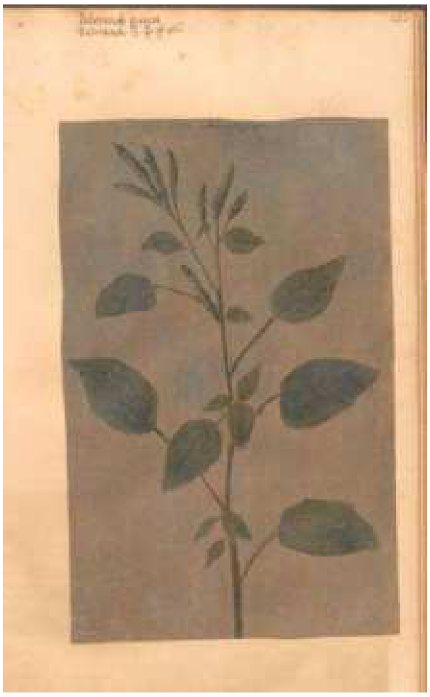

Theatrum Rerum Naturalium bound c. 1660-1664: 313

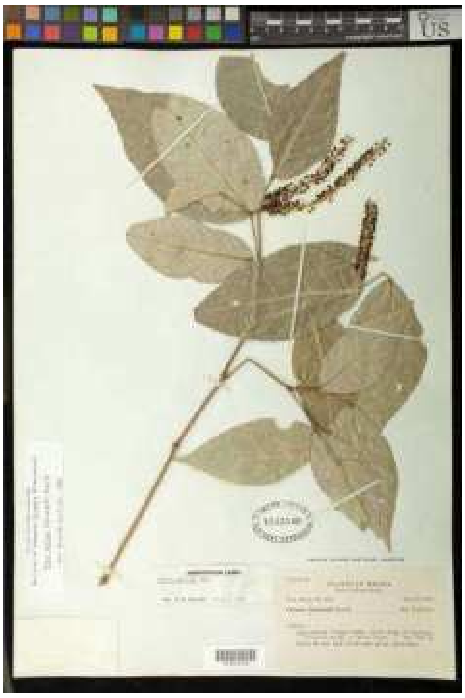

Specimen from Brazil 1930:1543540; collected by Ynes E. J. Mexia
retrieved online in Smithsonian 175

Medicina Brasiliensi Piso, 1648 Page number 97b

Vernacular name(s)	Iaborandi
Species	Piper tuberculatum Jacq.
Family	Piperaceae
Notes	

The woodcut looks different than the *Theatrum* image.

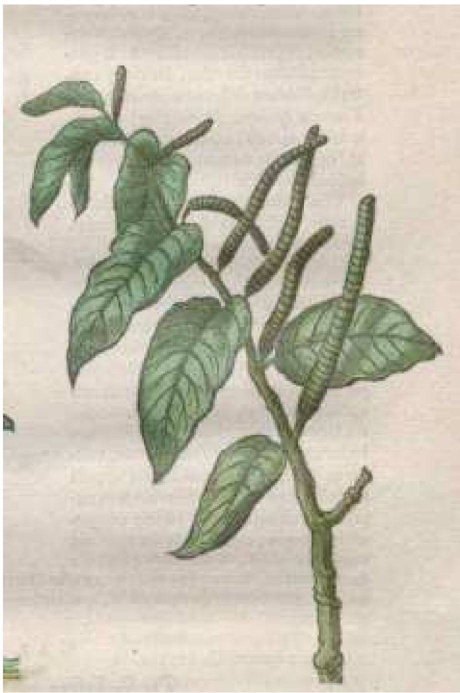

De Facultatibus Simplicium: 97

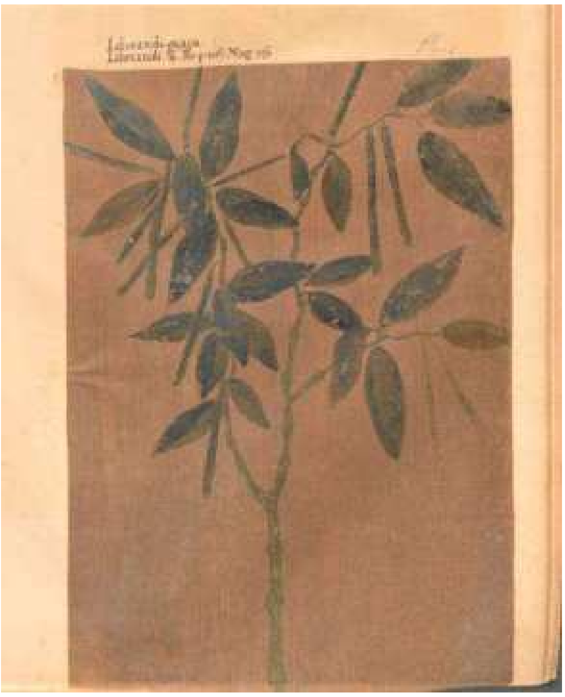

Theatrum Rerum Naturalium bound c. 1660-1664: 315

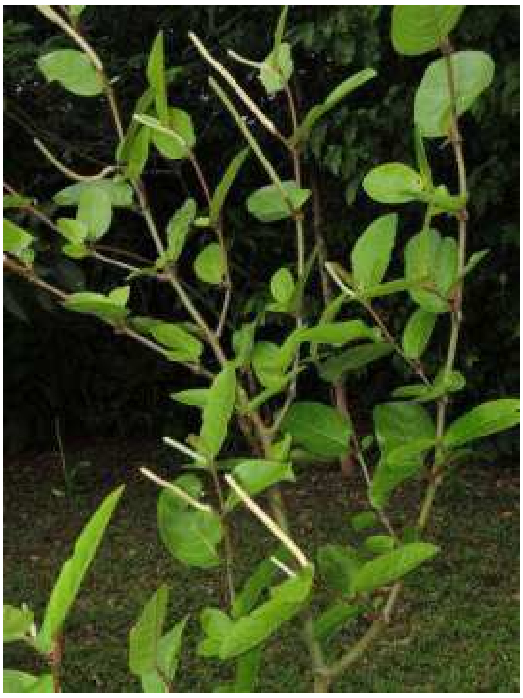

Photo by Barry Hammel (CC BY-NC-SA 2.0)

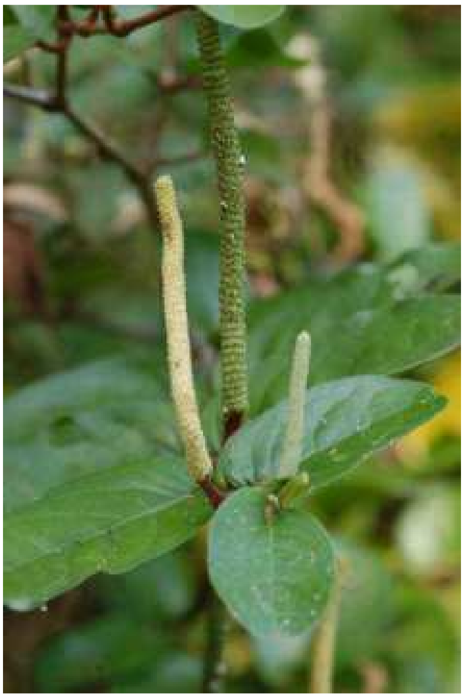

Photo by Barry Hammel (CC BY-NC-SA 2.0)

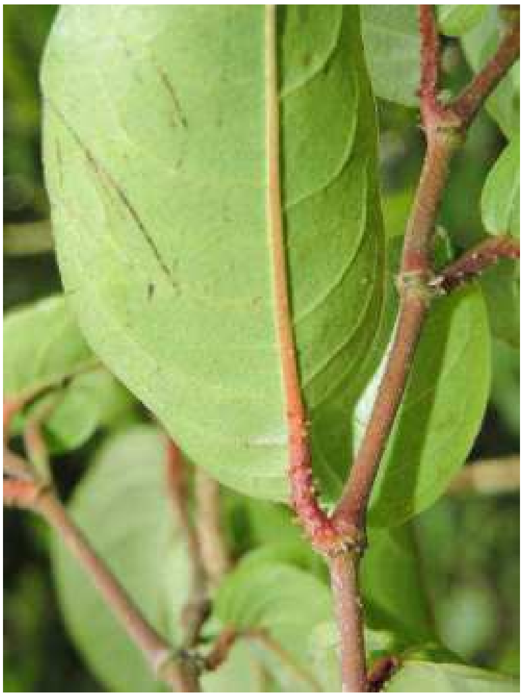

Stipules, Costa Rica 2020; by Barry Hammel (CC BY-NC-SA 2.0)

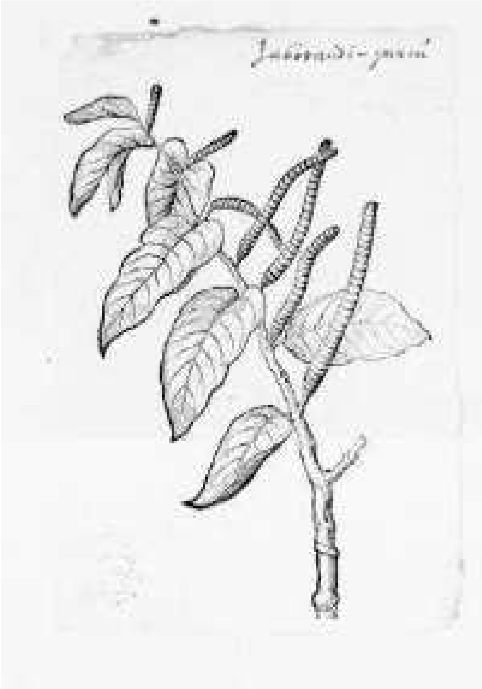

Sloane Ms 1554 f. 25v

Medicina Brasiliensi Piso, 1648 Page number 99

Vernacular name(s) Iupicanga

Species Smilax rufescens Griseb.

Family Smilacaceae

Notes

We did not find any correspondence between this woodcut and the contemporary or older sources. The figure represents a fruiting branch.

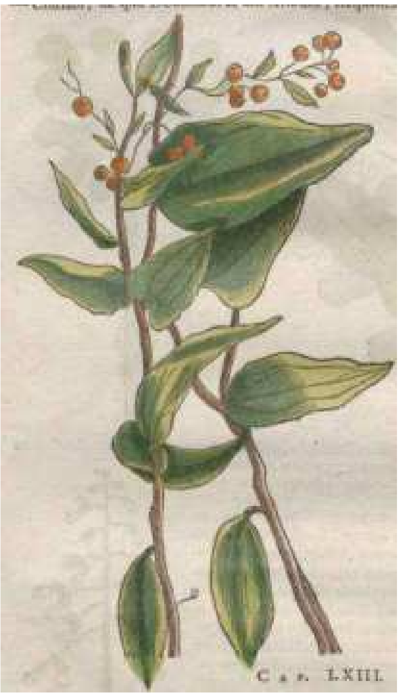

De Facultatibus Simplicium: 99

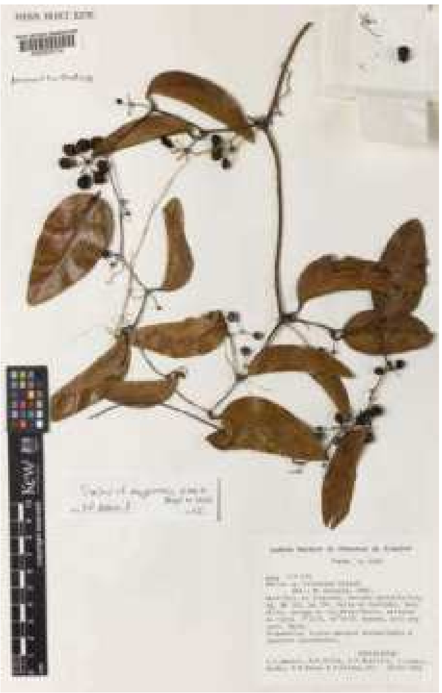

A specimen from Kew's Herbarium - K000828104
Retrieved from Plants of the World Online

Medicina Brasiliensi Piso, 1648 Page number 100b

Vernacular name(s) Urumbeba

Species Opuntia ficus-indica (L.) Mill.

Family Cactaceae

Notes

We did not find any correspondence between this woodcut and the contemporary or older sources. The figure represents an sterile plant with its trunk and leaves.

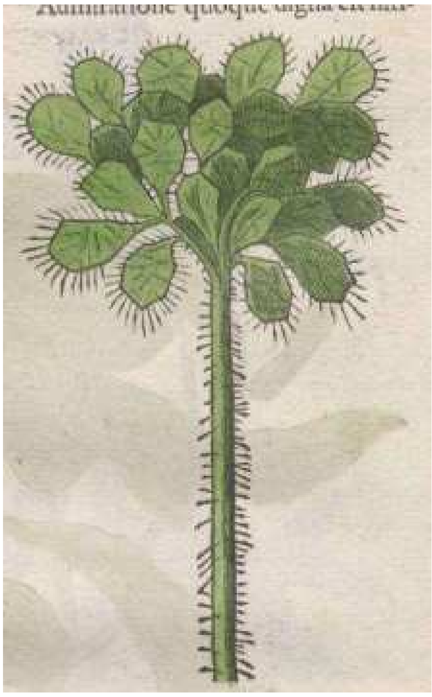

De Facultatibus Simplicium: 100

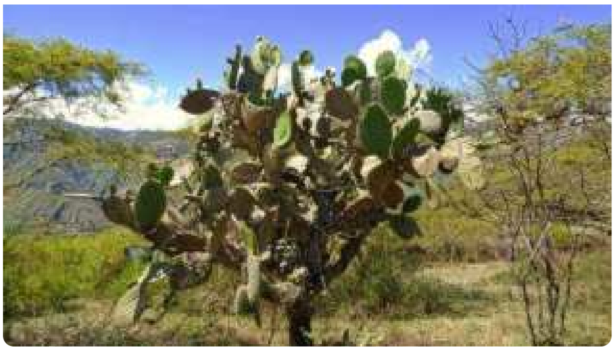

Plant, Ecuador 2011; by Andreas Kay, In Memoriam: Ecuador Megadiverso (CC BY-SA 2.0)

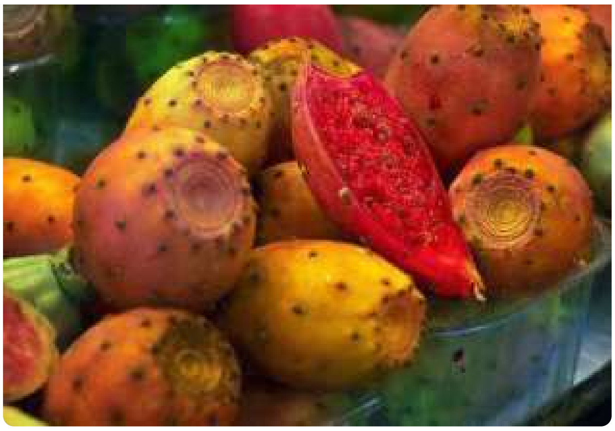

Fruits, 2009; by Carlos Lorenzo (CC BY 2.0)

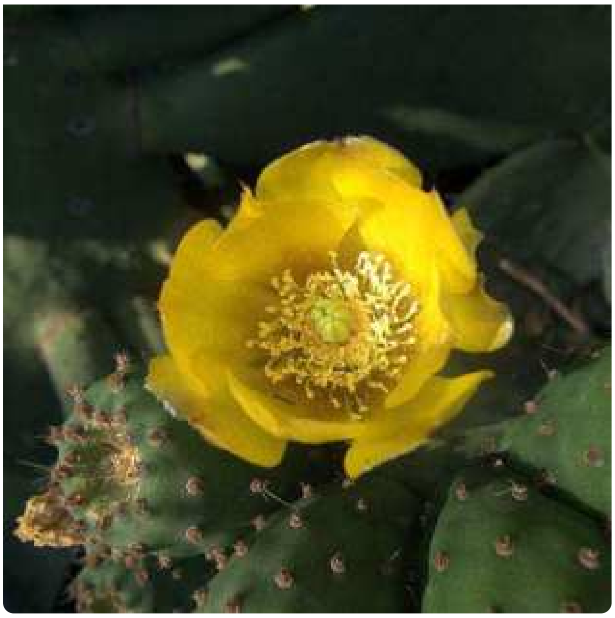

Flower, California 2014; by John Rusk (CC BY 2.0)

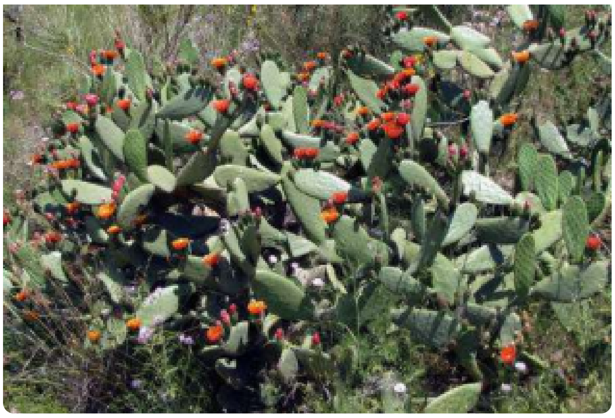

Flowering plant, España 2009; by José María Escolano (CC BY-NC-SA 2.0)

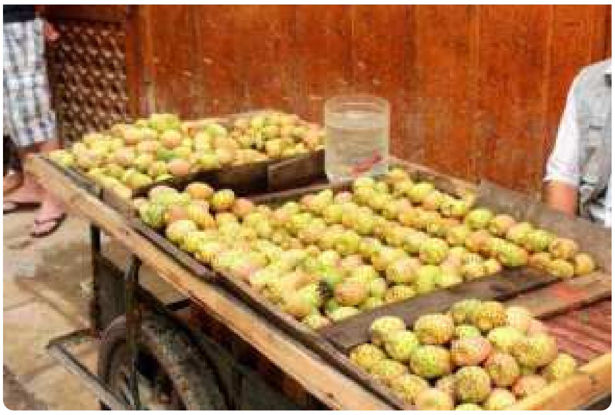

Market stall with fruits, Morocco 2014; by Arthur Chapman (CC BY-NC 2.0)

Medicina Brasiliensi Piso, 1648 Page number 101a

Vernacular name(s) Potincoba. Pulguera

Species Persicaria acuminata (Kunth) M.Gómez

Family Polygonaceae

Notes

We did not find any correspondence between this woodcut and the contemporary or older sources. The figure represents a sterile branch with leaves.

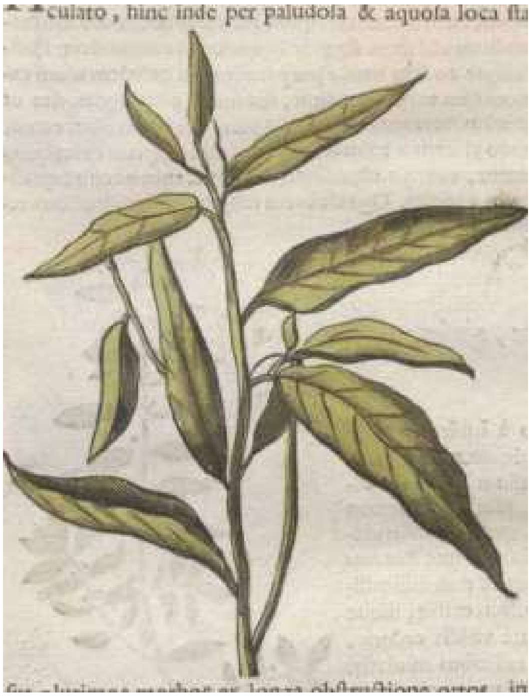

De Facultatibus Simplicium: 101

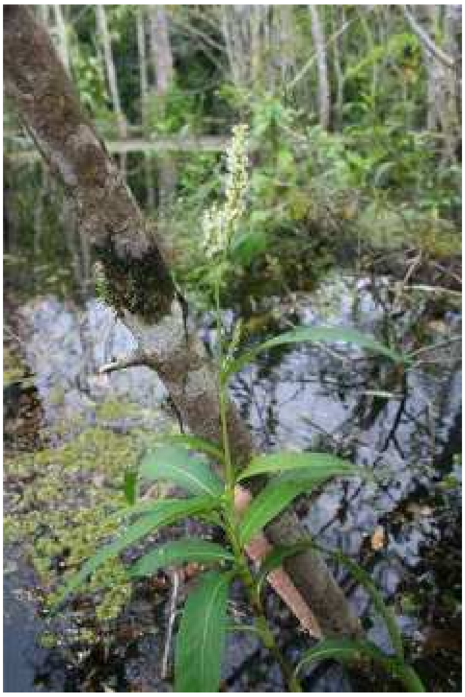

Flowering branch. Retrieved from Plants of the World Online

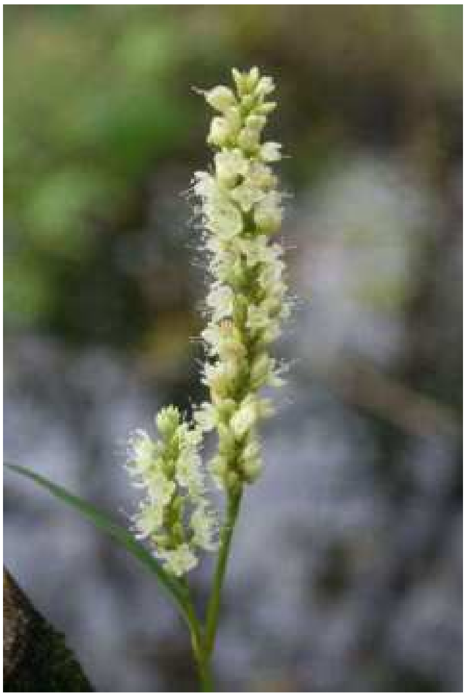

Inflorescence. Retrieved from Plants of the World Online

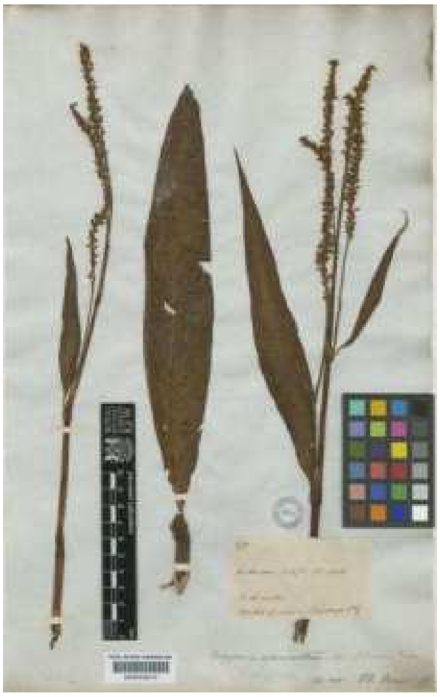

A specimen from Kew's Herbarium - K000532015
Retrieved from Plants of the World Online

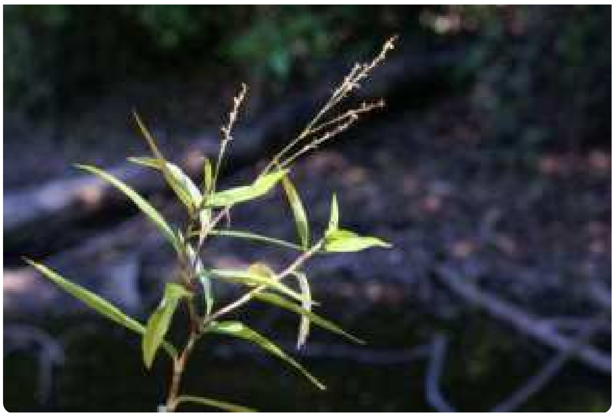

Retrieved from Plants of the World Online

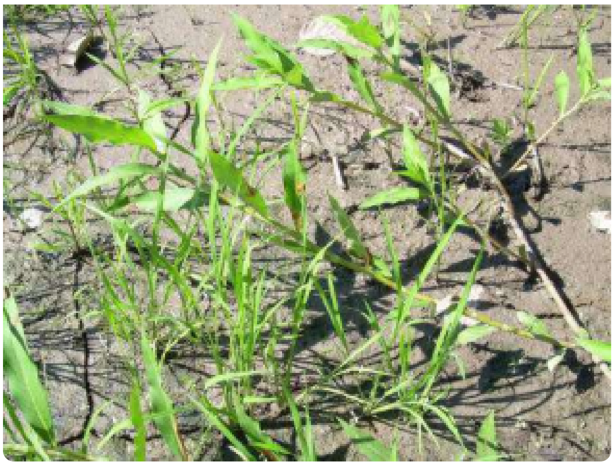

Peru 2007; by Rob Westerduijn (CC BY-NC-SA 2.0)

Medicina Brasiliensi Piso, 1648 Page number 103a

Vernacular name(s) Salsa do Praya. Carca do litoral

Species Ipomoea pes-caprae (L.) R. Br.

Family Convolvulaceae

Notes

The woodcut image resembles the creeping vine on Eckhout's portrait known as the "African man". It could have been made after the same drawing that was used as a model for the portrait. The drawing could also be based on the crayon drawing of the *Misc. Cleyeri*, although the resemblance is not that accurate.

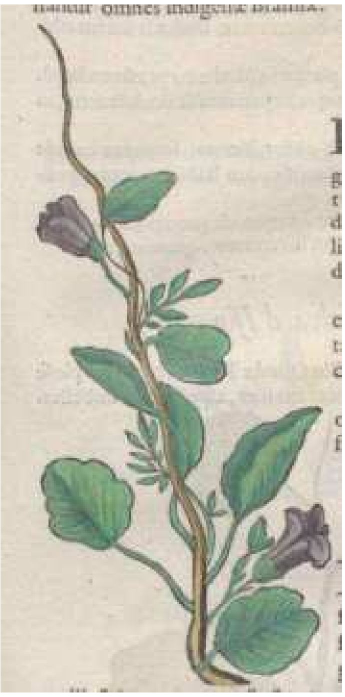

De Facultatibus Simplicium: 103

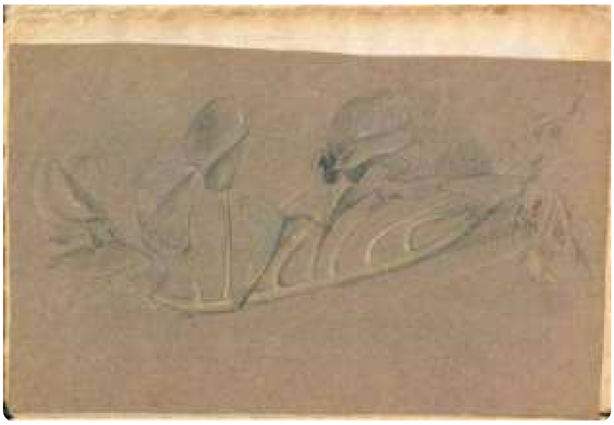

Miscellanea Cleyeri c.1637-44: 12v

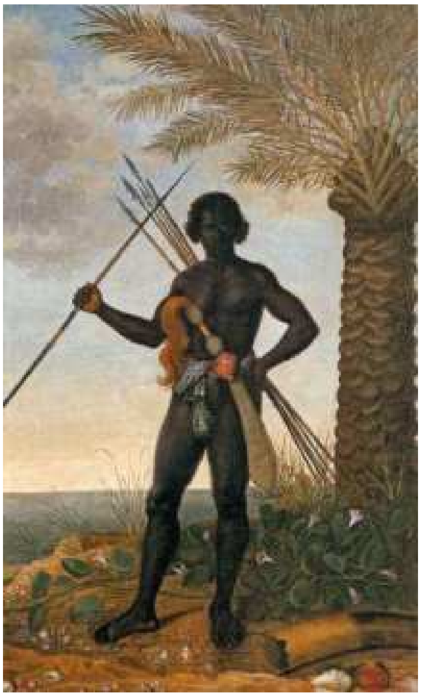

Eckhout portrait (African man, Brazil 1641: National Museum Denmark - Public domain)

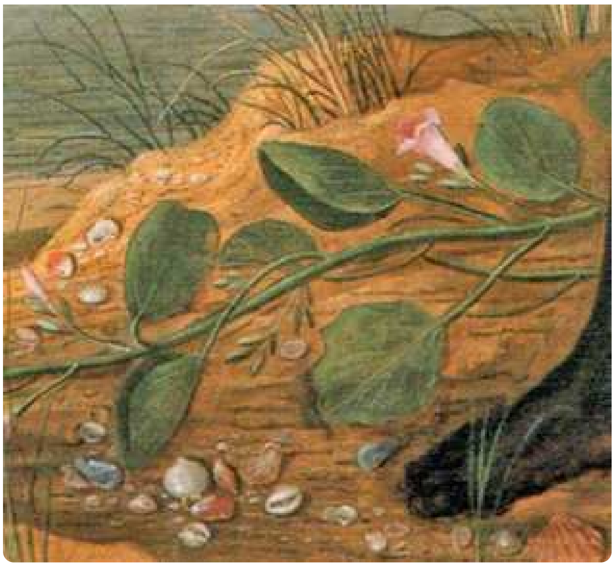

Eckhout portrait (African man)

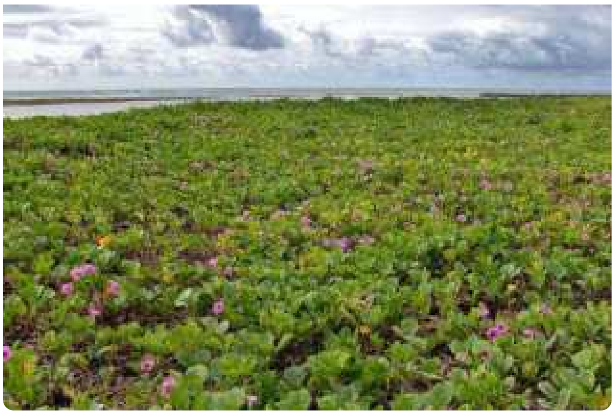

Pernambuco, Brazil 2012; by Mauricio Mercadante, (CC BY-NC-SA 2.0)

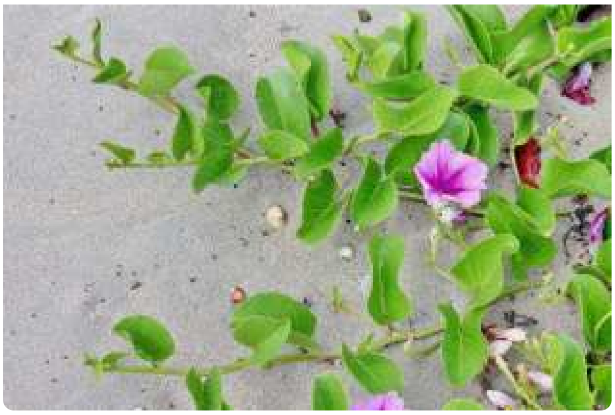

Pernambuco, Brazil 2012; by Mauricio Mercadante, (CC BY-NC-SA 2.0)

Medicina Brasiliensi Piso, 1648 Page number 103b

Vernacular name(s) Aninga iba

Species Montrichardia linifera (Arruda) Schott

Family Araceae

Notes

The woodcut image resembles the plant that is depicted in Eckhout's portrait of the "Tarairiu woman". It shows the inflorescence and fruit, while the portrait only represents the fruit. Someone added both fertile structures in the woodcut to display a more complete botanical portrait (e.g., for others to identify it in the field in all seasons). The *Theatrum* included an image that resembles the fruit of this species. Moreover, De Laet's manuscript included a proof-woodcut of this species.

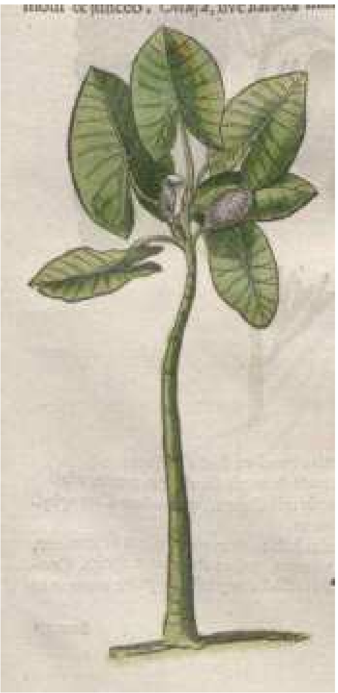

De Facultatibus Simplicium: 103

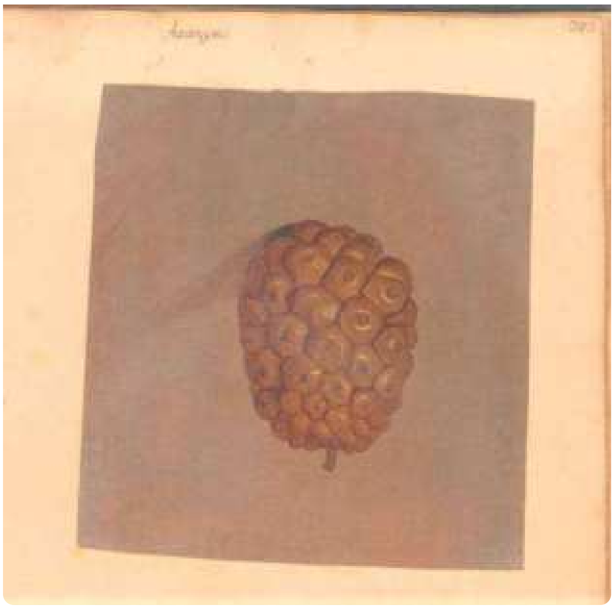

Theatrum Rerum Naturalium bound c.1660-1664: 383

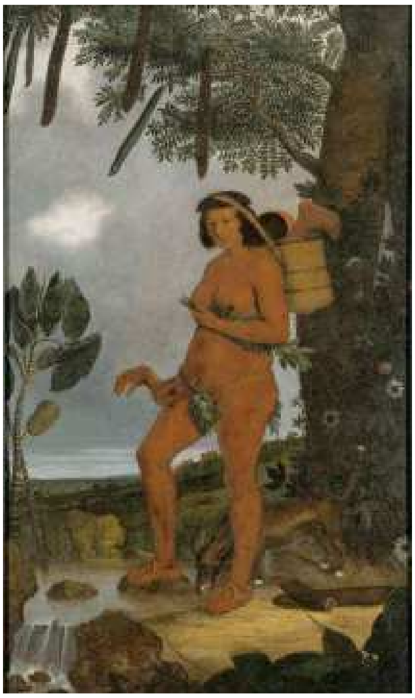

Eckhout portrait (Tarairiu woman, Brazil 1641: National Museum Denmark - CC BY-NC-SA 2.0)

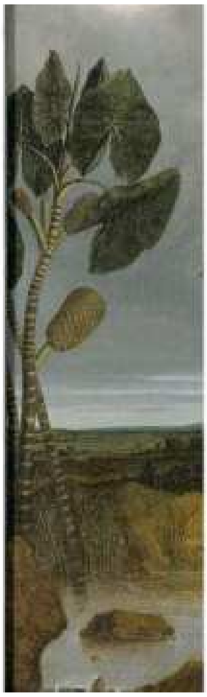

Eckhout portrait (Tarairiu woman)

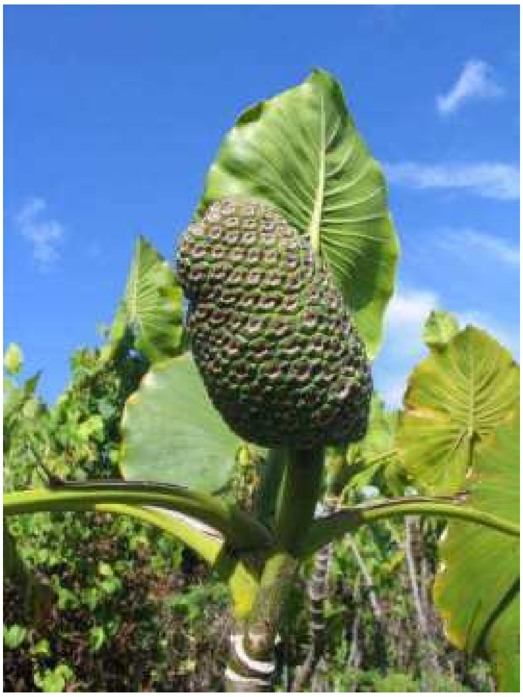

Fruit. Ceará 2006; "Aninga" by Antonio S. F. Castro (CC BY-NC-SA 2.0)

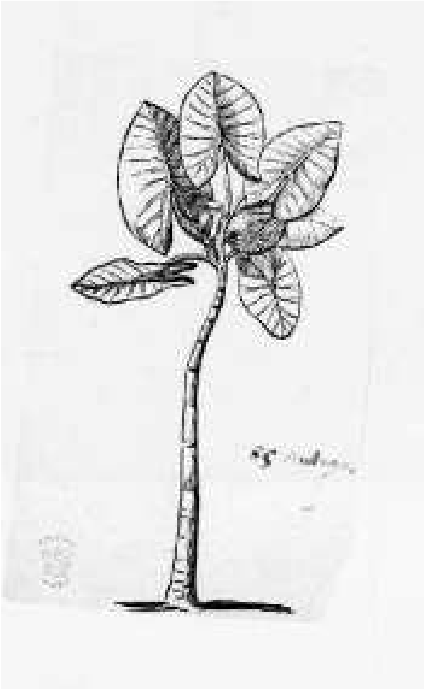

Proof-woodcut in Sloane Ms 1554 f. 66v

Medicina Brasiliensi Piso, 1648 Page number 104

Vernacular name(s) Aguaxima. Malva d' Isco

Species Piper umbellatum L.

Family Piperaceae

Notes

The woodcut is very similar to the *Theatrum* image (reversed)

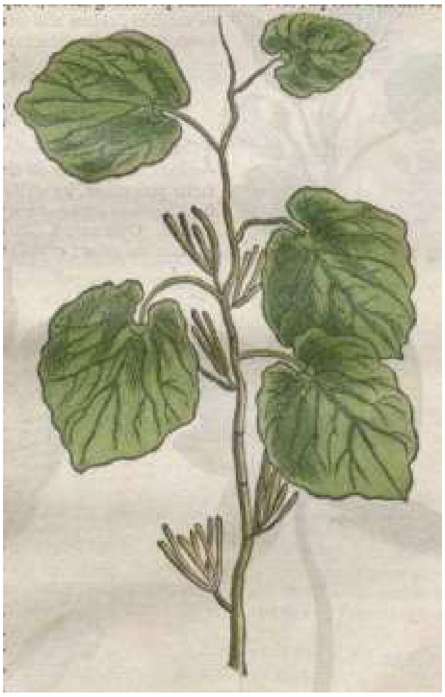

De Facultatibus Simplicium: 104

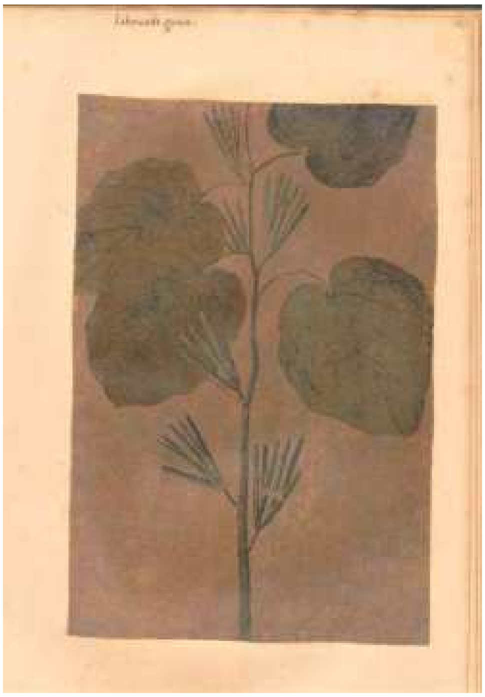

Theatrum Rerum Naturalium bound c.1660-1664: 319

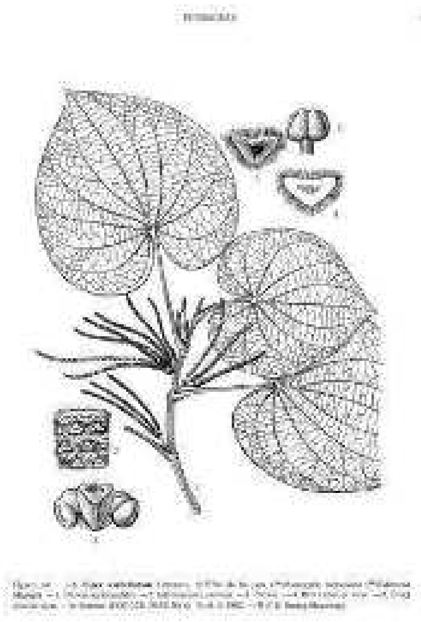

Line drawing; by filibot.web (CC BY-NC-SA 2.0)

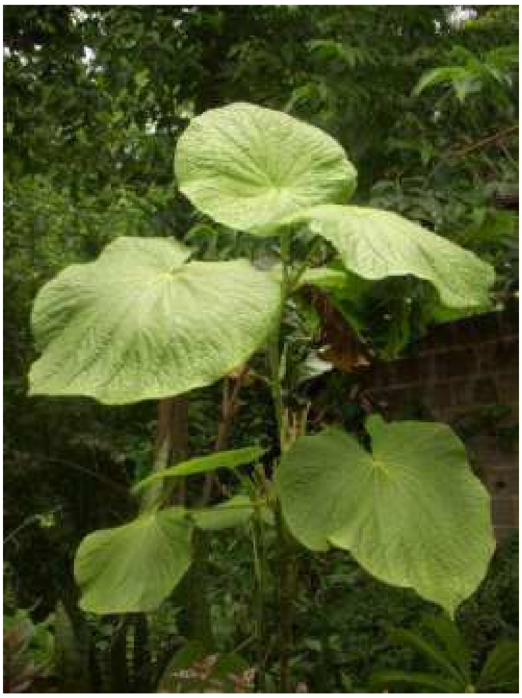

Plant; by Diana K. Cury (CC-BY-SA-4.0)

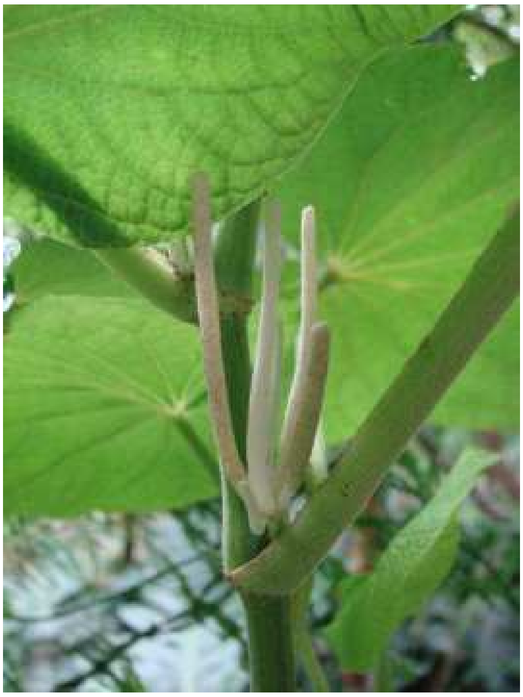

Inflorescence spikes. Brasília 2010; by João de Deus Medeiros (CC BY 2.0)

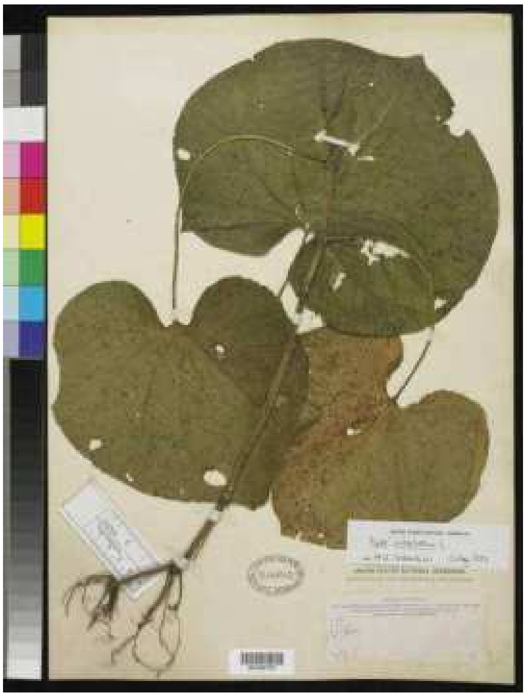

Herbarium specimen; US National Herbarium: collected by Cook O.F. in Liberia, 946083 (CC BY 2.0)

Medicina Brasiliensi Piso, 1648 Page number 110a

Vernacular name(s) Basourinha. Basoura. Tupeicava

Species Scoparia dulcis L.

Family Plantaginaceae

Notes

We did not find any correspondence between this woodcut and the contemporary or older sources. However, it could have been made after the specimen in Marcgrave's herbarium, which also depicts a flowering branch.

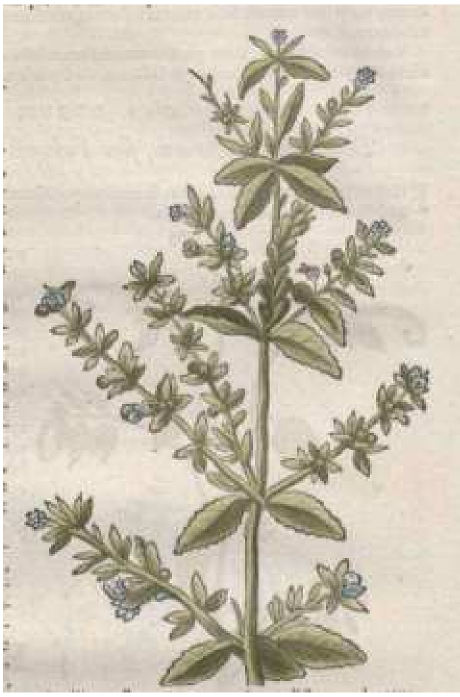

De Facultatibus Simplicium: 110

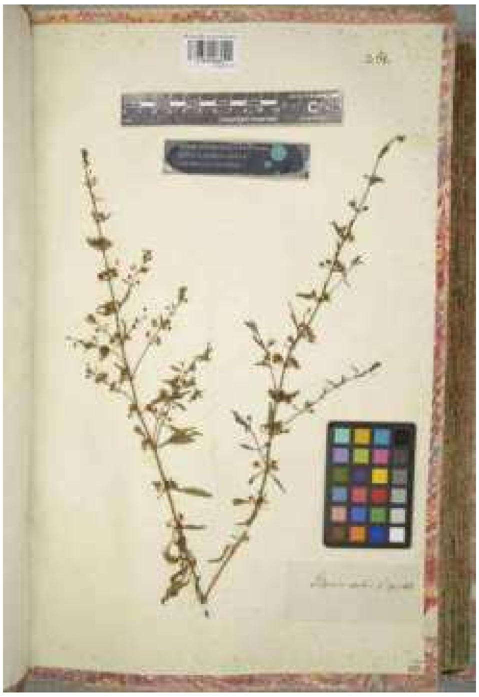

Marcgrave's herbarium: 66

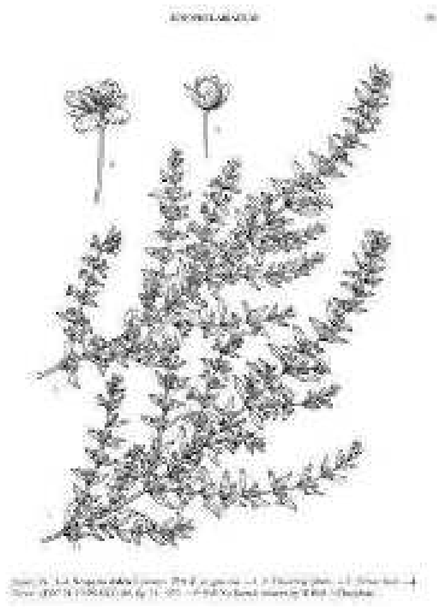

Line drawing; by filibot.web (CC BY 2.0)

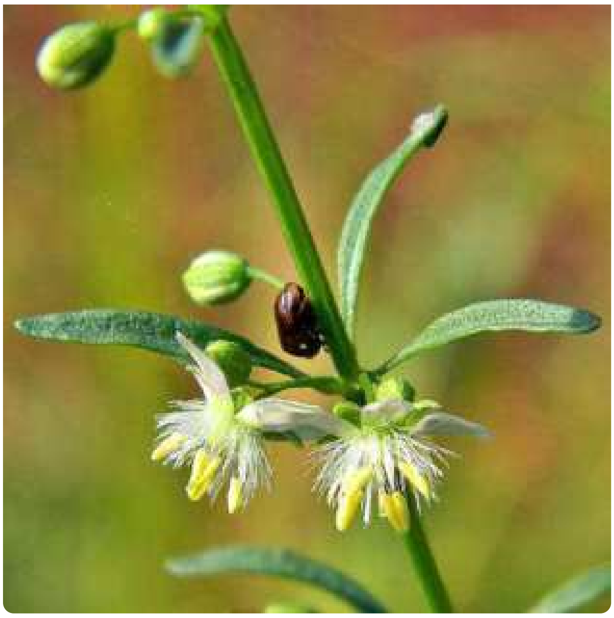

Flowers, Florida 2011; by Bob Peterson (CC0 1.0)

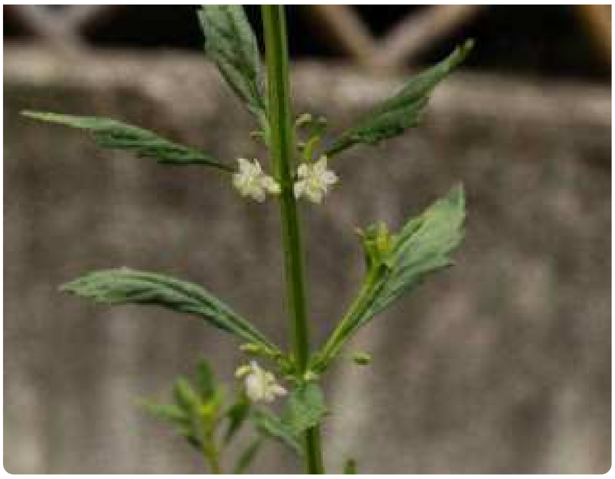

Thailand 2011; by Tony Rodd (CC BY-SA 4.0)

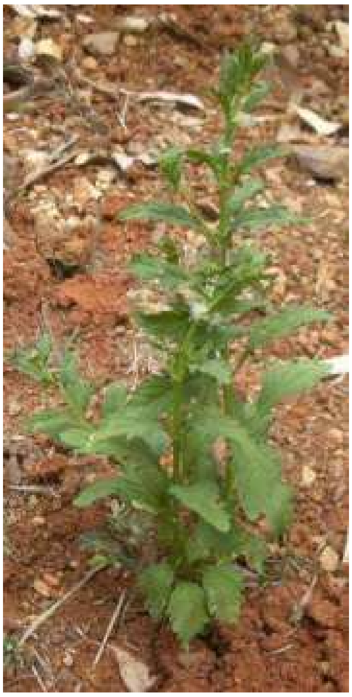

Plant; by Mark Marathon (CC-BY-SA-3.0)

Medicina Brasiliensi Piso, 1648 Page number 111b

Vernacular name(s) Caraguata acanga

Species Bromelia karatas L.

Family Bromeliaceae

Notes

De Laet took the woodcut from his previous work based on his ethnographical accounts of the Americas. The image shows an open dry fruit of *B. karatas* with several seeds. This fruit was sent to De Laet from Brazil and it is represented at its natural size.

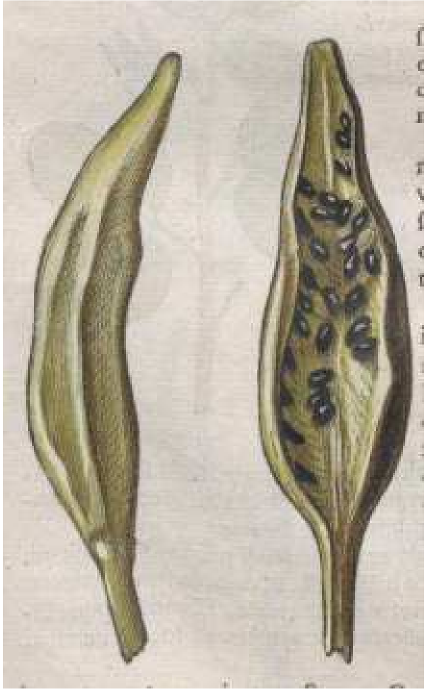

De Facultatibus Simplicium: 111

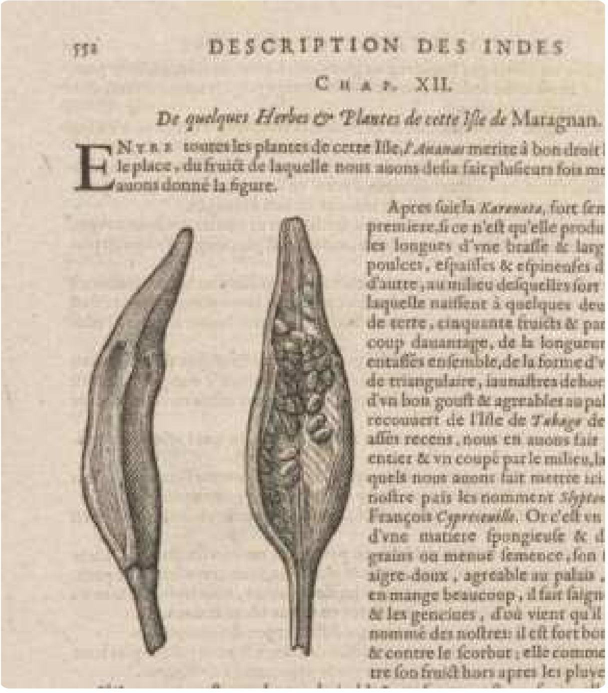

552 DESCRIPTION DES INDES

CHAP. XII.

De quelques Herbes & Plantes de cette Isle de Maragnan.

ENTRE toutes les plantes de cette Ile, *l'Ananas* merite à bon droit l[illegible] le place, du fruict de laquelle nous auons desia fait plusieurs fois me[illegible] auons donné la figure.

Apres suit la *Karenata*, fort sem[illegible] premiere, si ce n'est qu'elle produ[illegible] les longues d'vne brasse & larg[illegible] poulces, espaisses & espineuses d[illegible] d'autre, au milieu desquelles sort [illegible] laquelle naissent à quelques deu[illegible] de terre, cinquante fruicts & par[illegible] coup dauantage, de la longueur [illegible] entassés ensemble, de la forme d'v[illegible] de triangulaire, iaunastres dehor[illegible] d'vn bon goust & agreables au pal[illegible] recouuert de l'Isle de *Tabago* de[illegible] assés recens, nous en auons fait [illegible] entier & vn coupé par le milieu, la[illegible] quels nous auons fait mettre ici[illegible] nostre païs les nomment *Slypto*[illegible] François *Cypresseville*. Or c'est vn [illegible] d'vne matiere spongieuse & d[illegible] grains ou menue semence, son [illegible] aigre-doux, agreable au palais, [illegible] en mange beaucoup, il fait saign[illegible] & les gencives, d'où vient qu'il [illegible] nommé des nostres: il est fort bo[illegible] & contre le scorbut; elle comme[illegible] tre son fruict hors apres les pluye[illegible]

De Laet, J. *L'histoire du Nouveau Monde, ou, Description des Indes Occidentales* (Leiden: Elzevir, 1640: 552)

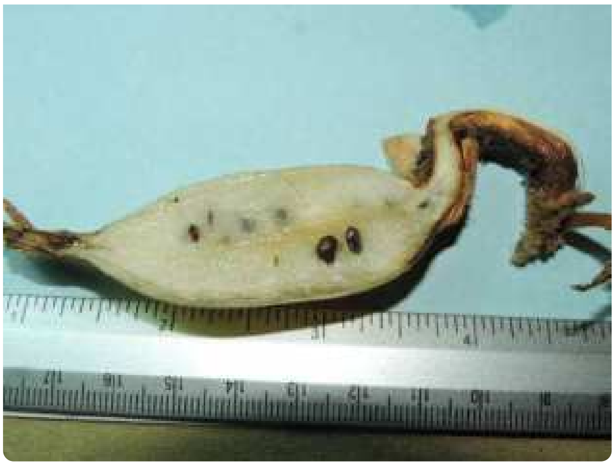

Fruit. Costa Rica 2016. "Piñuela" by Reinaldo Aguilar (CC BY-NC-SA 2.0)

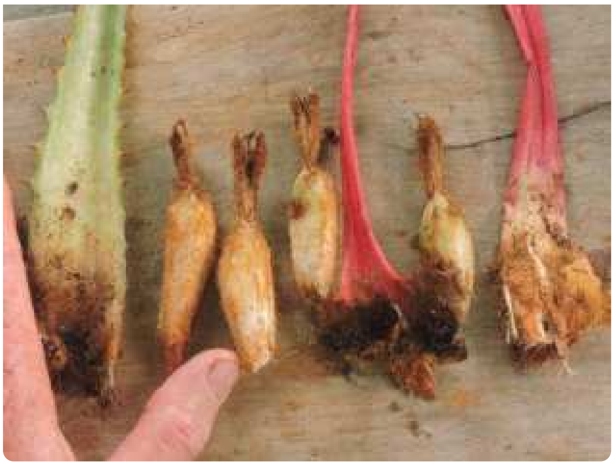

Leaf and fruits. Costa Rica 2017; by Barry Hammel (CC BY-NC-SA 2.0)

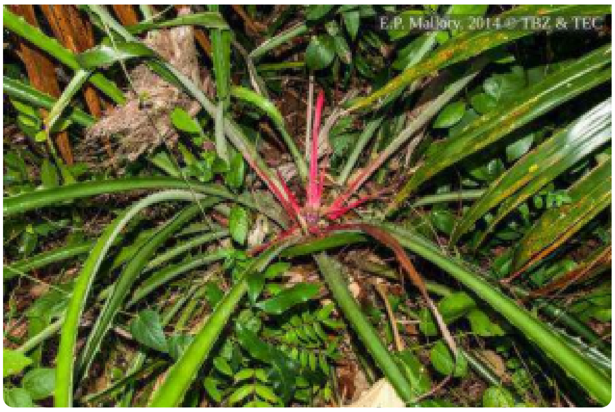

Plant. Belize 2014; by E.P. Mallory (CC BY-NC-SA 2.0)

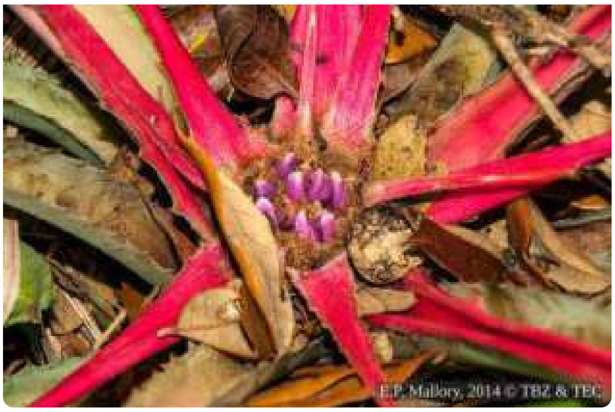

Inflorescence. Belize 2014; by E.P. Mallory (CC BY-NC-SA 2.0)

Medicina Brasiliensi Piso, 1648 Page number 112

Vernacular name(s) Trevo. Erva d' Amor

Species Desmodium incanum (Sw.) DC.

Family Fabaceae

Notes

We did not find a very strong correspondence between this woodcut and the contemporary or older sources. However, the woodcut could have been based on the specimen collected by Marcgrave. There is a male flower of *Carica papaya* misplaced and mixed with the specimen of *D. incanum* (Andrade-Lima et al. 1977).

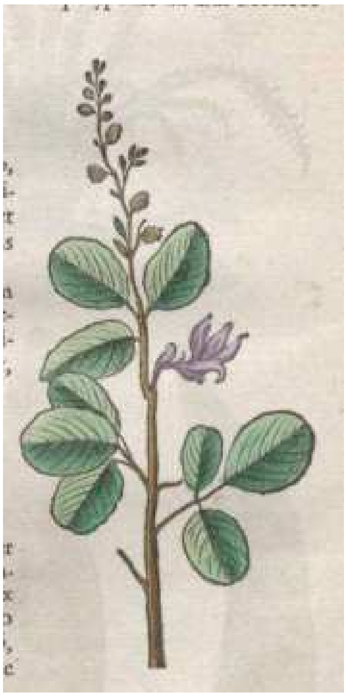

De Facultatibus Simplicium: 112

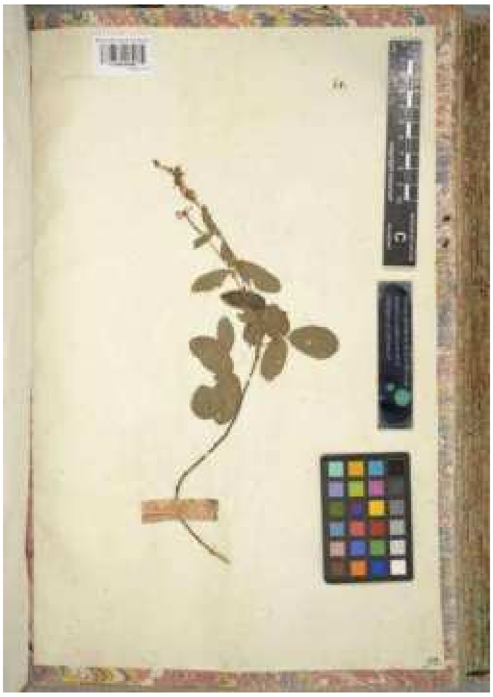

Marcgrave's herbarium: 93

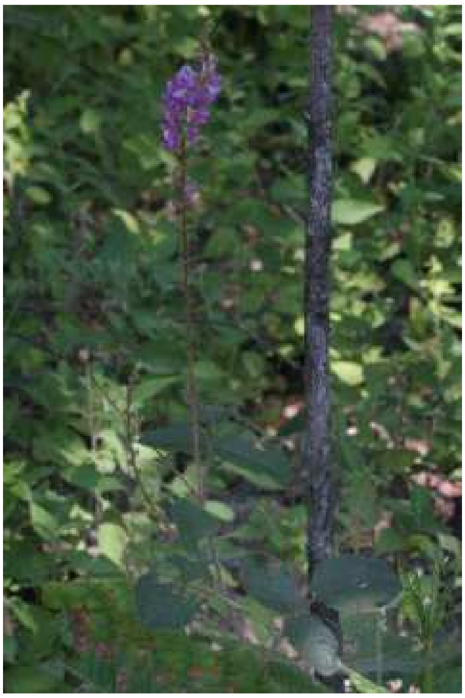

Plant; by Jonathan Amith, Retrieved from Plants of the World Online

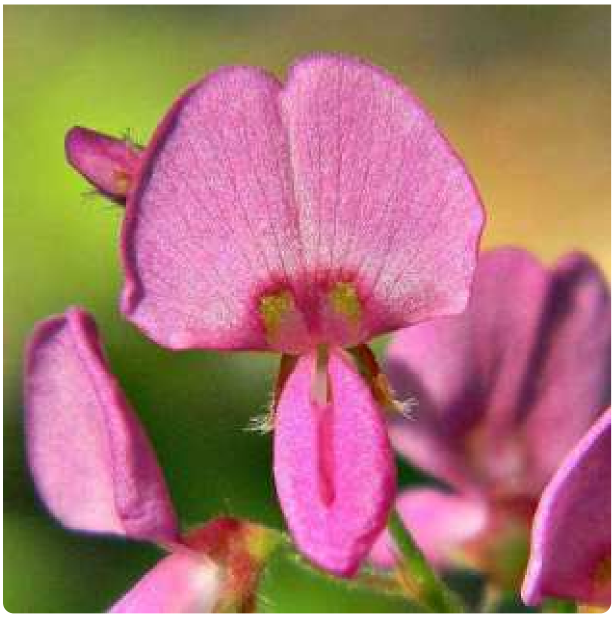

Flowers; by Bob Peterson (CC BY-NC 2.0)

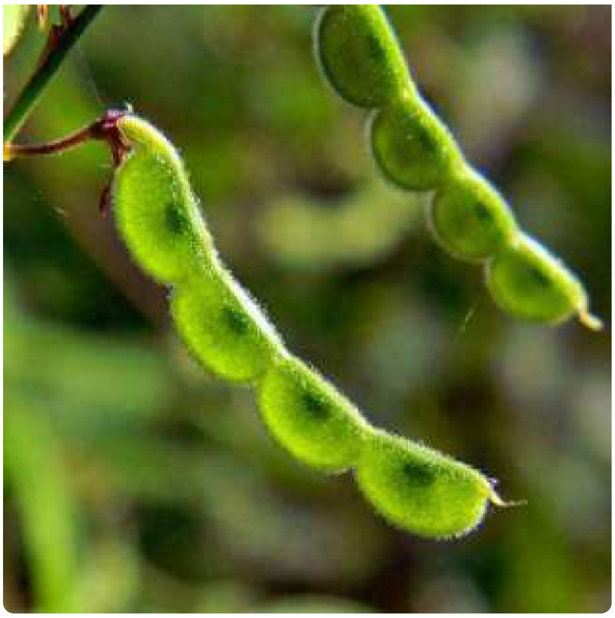

Pods; by Bob Peterson (CC BY-NC 2.0)

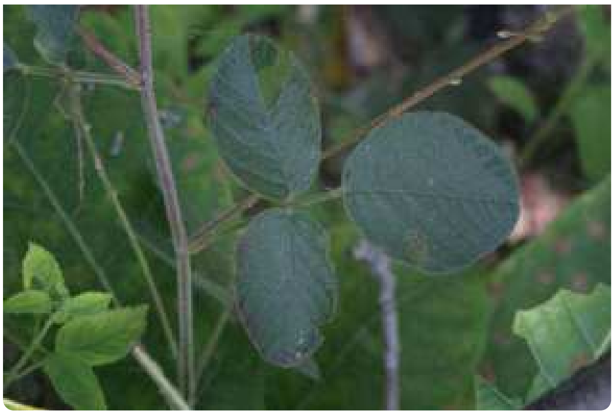

Detail of leaves; by Jonathan Amith, Retrieved from Plants of the World Online

Medicina Brasiliensi Piso, 1648 Page number 113a

Vernacular name(s) Herba lanuginosa

Species Unknown

Family Unknown

Notes

We did not find any correspondence between this woodcut and the contemporary or older sources. We could not identify the plant represented by the woodcut, hence we could not cross-reference it with the visual sources. Accurate identification of such plant will facilitate this analysis and provide us with more insights into its origin.

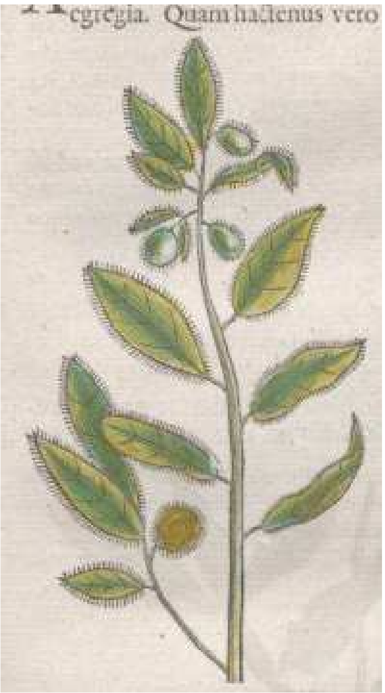

De Facultatibus Simplicium:: 113

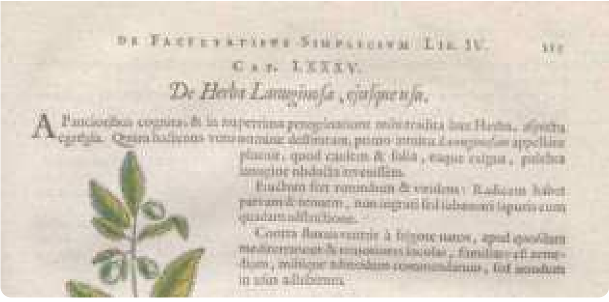
DE FACULTATIBUS SIMPLICIUM LIB. IV.

CAP. LXXXV.

De Herba Lanuginosa, ejusque usu.

[illegible]

Latin edition 1648
Leiden University Library (The Netherlands)

do, danoso [illegible] De modo que [illegible]
de apóstema. De modo que [illegible]
dicos como por cirurgiões (390).

CAPÍTULO LXXXV

DA ERVA LANUGINOSA E DO SEU USO

Conhecida de poucos, foi-me apresentada, em recentíssima excursão, esta erva de lindo aspecto. Não tendo até agora verdadeiro nome, aprouve-me chamar-lhe pela primeira impressão, *lanuginosa*, por ter o caule e as pequenas fôlhas cobertas de lanugem.

Dá um fruto redondo e verde; raiz pequena e fina, de sabor não desagradável, mas amargoso, algo adstringente.

E' remédio familiar, entre certos habitantes mediterrâneos e afastados, contra os fluxos do ventre. Foi-me muito gabada, mas ainda não tive ocasião de aplicá-la (391).

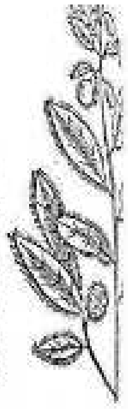

Portuguese edition 1942

"Known to few, this beautiful-looking herb was presented to me. Not having a real name so far, I was pleased to call it by the first impression, *lanuginosa*, because it has the stem and small leaves covered with *lanugo* [wool]. It gives a round and green fruit; small and thin root, not pleasant, but bitter, something astringent. Family remedy, among certain Mediterranean and remote inhabitants, against the flows of the womb. It was very boastful to me, but I haven't had a chance to apply it yet" (Piso 1648: 113).

English translation, by M. Alcantara-Rodriguez

Medicina Brasiliensi Piso, 1648 Page number 113b

Vernacular name(s) Tertia. Mangue Guaparaiba

Species Rhizophora racemosa G.Mey.

Family Rhizophoraceae

Notes

We did not find any correspondence between this woodcut and the contemporary or older sources.

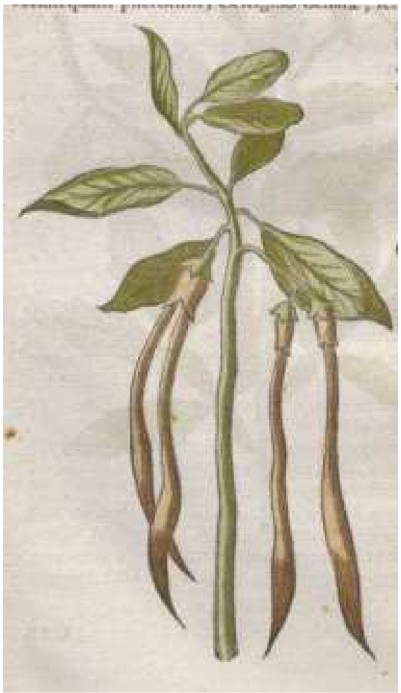

De Facultatibus Simplicium: 113

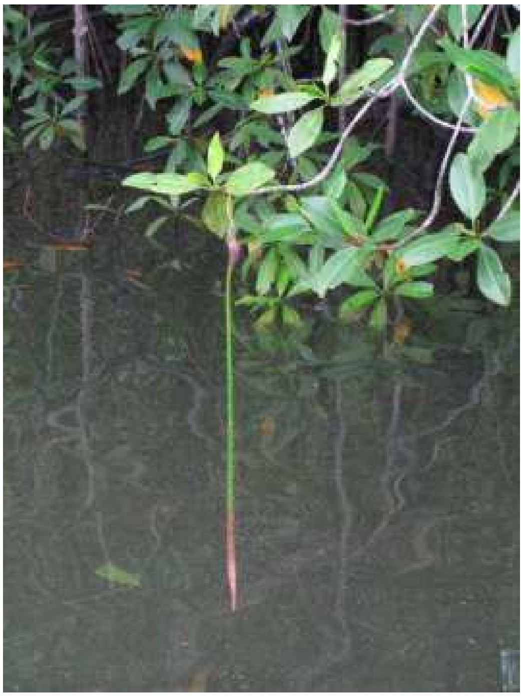

Rhizophora sp. showing the hypocotyle emerging from the fruit; by wan_hong (CC BY-NC-SA 2.0)

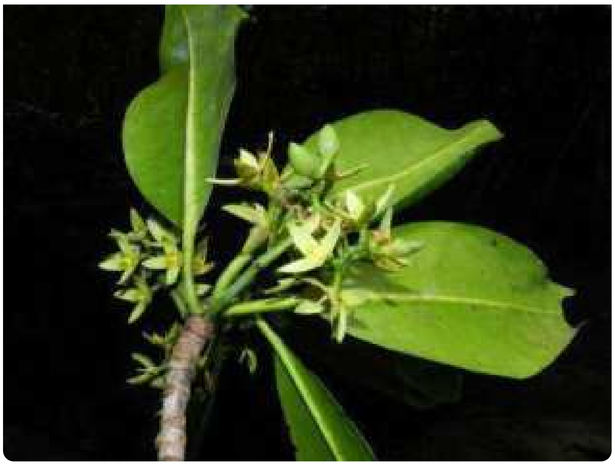

Flowers; Costa Rica 2014; by Reinaldo Aguilar (CC BY-NC-SA 2.0)

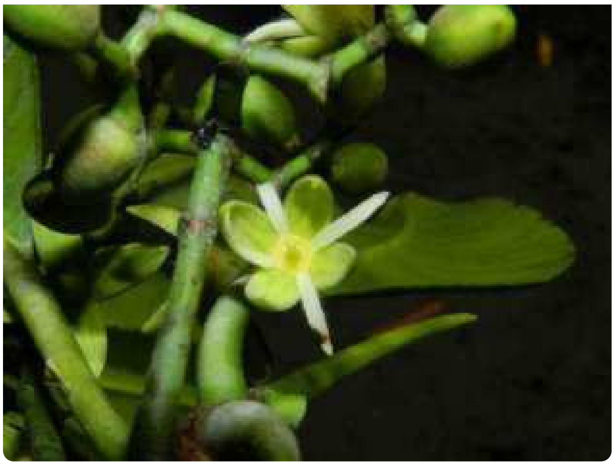

Detail of a flower, Costa Rica 2014; by Reinaldo Aguilar (CC BY-NC-SA 2.0)

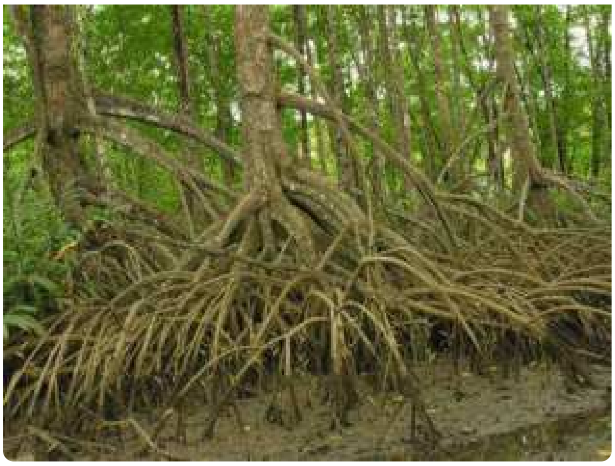

Roots system; by Reinaldo Aguilar (CC BY-NC-SA 2.0)

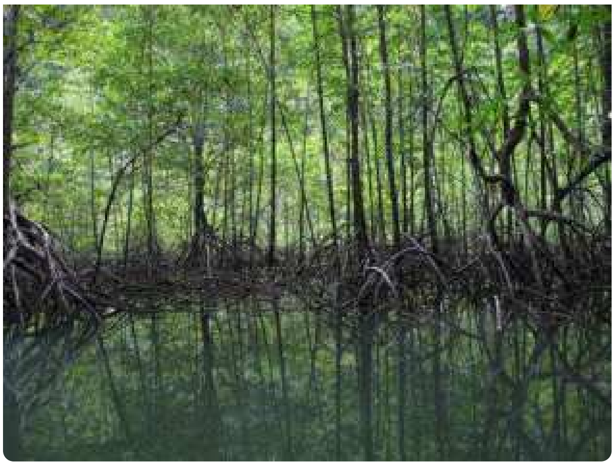

"The main mangrove forest (mainly *R. racemosa*), Costa Rica" by wan_hong (CC BY-NC-SA 2.0)

Medicina Brasiliensi Piso, 1648 Page number 114

Vernacular name(s) Cururu ape

Species Paullinia pinnata L.

Family Sapindaceae

Notes

The woodcut is moderately similar to the *Theatrum* oil painting. The leaves do not match completely although the fruits show a strong resemblance. A specimen of this species is placed in Marcgrave's herbarium, but we cannot compare it properly with the woodcut as the specimen seems incomplete. De Laet's manuscript included a proof-woodcut of this species, but no description, likely because *P. pinnata* was never described or depicted in Marcgrave's chapters on plants.

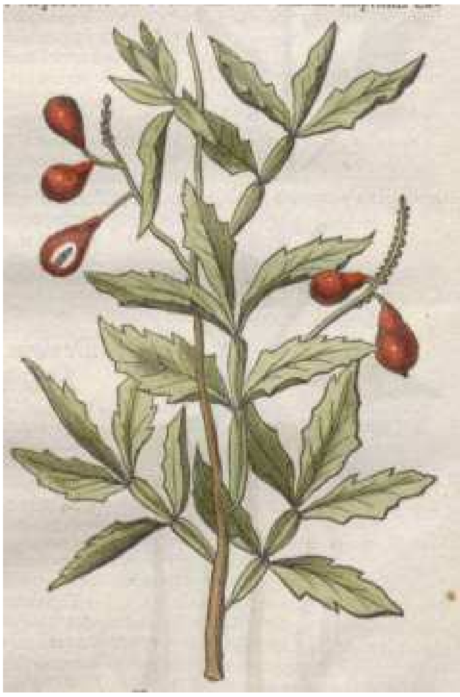

De Facultatibus Simplicium: 114

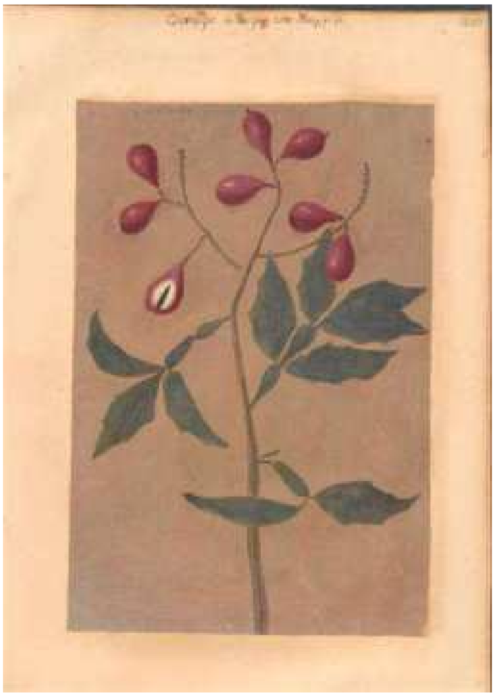

Theatrum Rerum Naturalium bound c.1660-1664: 283

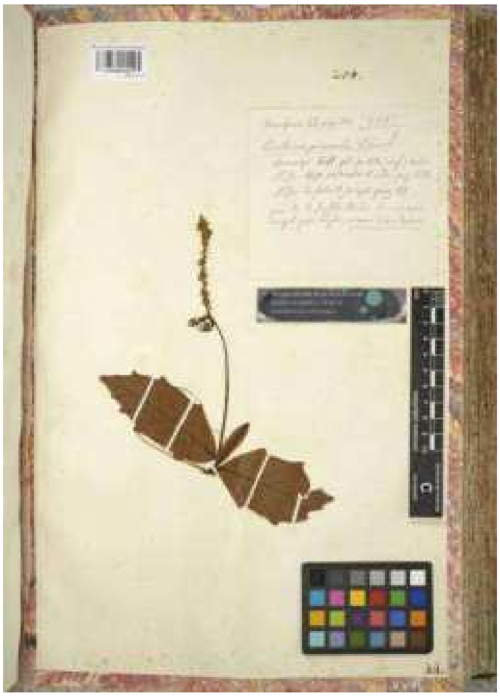

Marcgrave's herbarium: 101

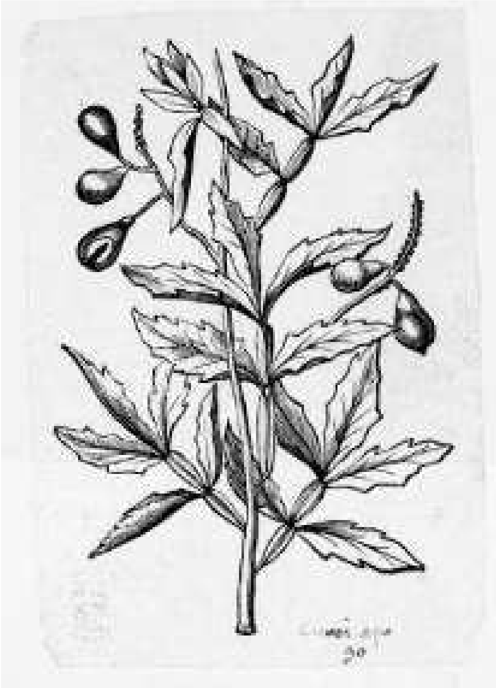

Proof-woodcut in Sloane Ms 1554 f. 69v

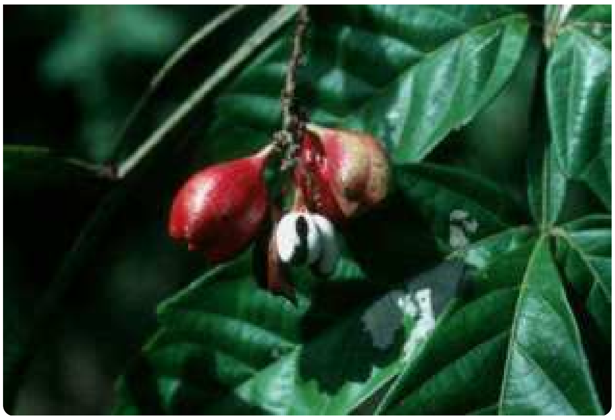

Fruits; Collection of the Smithsonian Institution (CC BY 2.0)

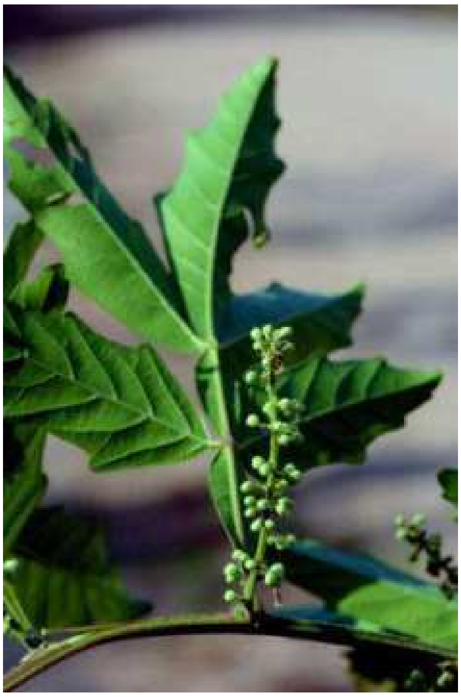

Inflorescence; Collection of the Smithsonian Institution (CC BY 2.0)

Medicina Brasiliensi Piso, 1648 Page number 116a

Vernacular name(s)	Aninga peri
Species	Clidemia octona (Bonpl.) L.O. Williams
Family	Melastomataceae

Notes

We did not find any correspondence between this woodcut and the contemporary or older sources.

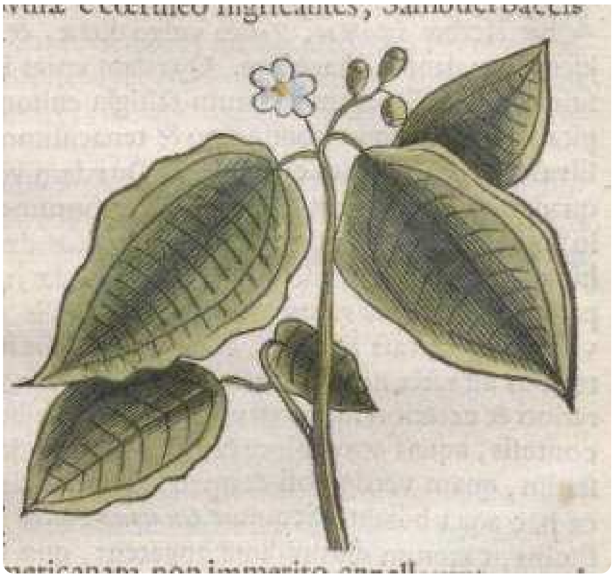

De Facultatibus Simplicium: 116

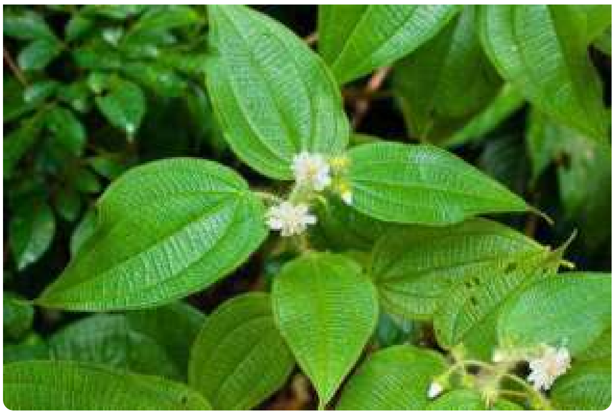

Plant; by Andres Hernandez S. (CC0 1.0)

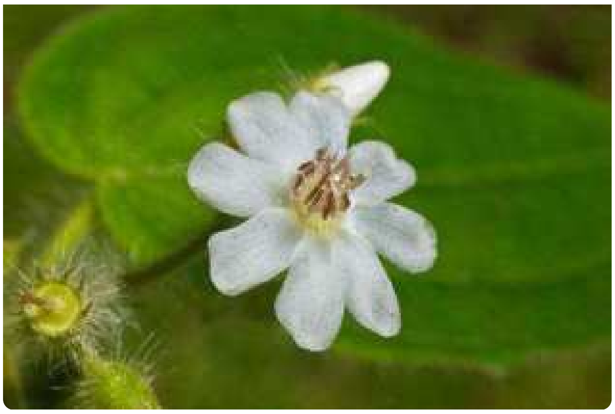

Flower; by Andres Hernandez S. (CC0 1.0)

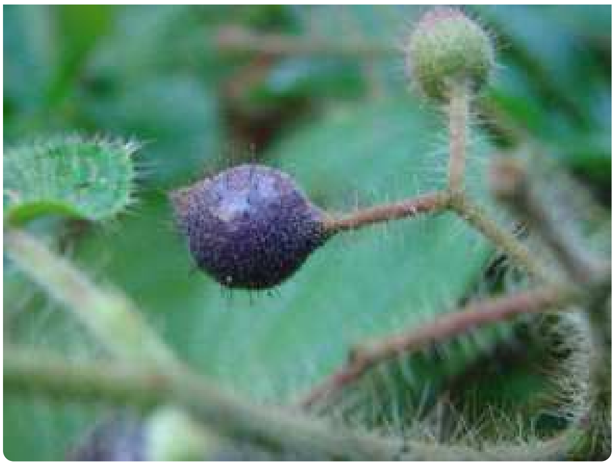

Fruit; by João Medeiros (CC BY-NC-SA 2.0)

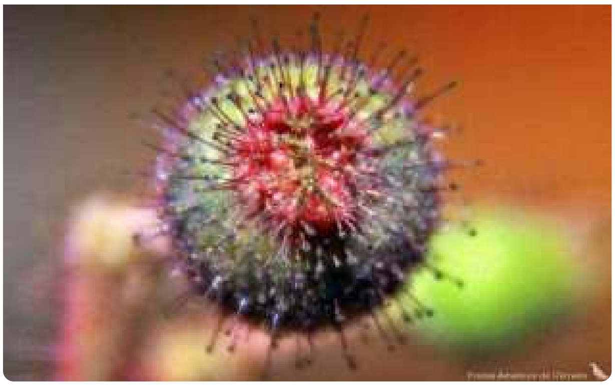

Fruit; by Marcelo_Kuhlmann (CC BY 2.0)

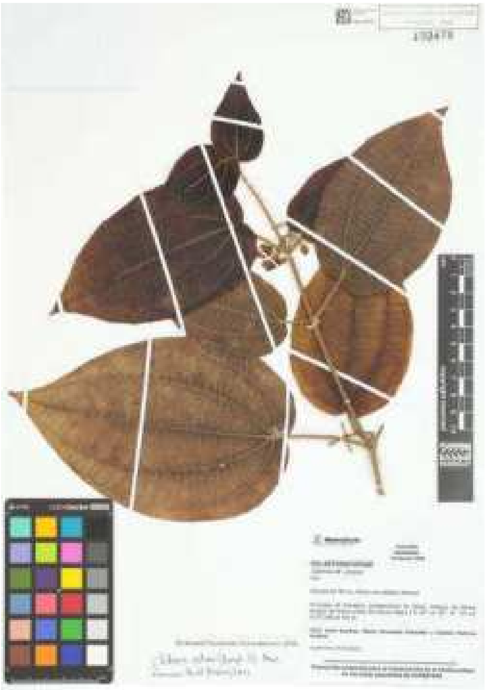

Specimen; by Herbario virtual FMB (CC BY-NC-SA 2.0)

Medicina Brasiliensi Piso, 1648 Page number 119a

Vernacular name(s) Jupicai. Erva d' Empige

Species Xyris jupicai Rich.

Family Xyridaceae

Notes

We did not find any correspondence between this woodcut and the contemporary or older sources.

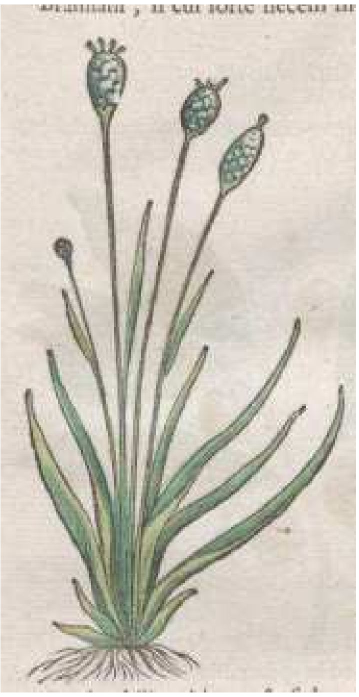

De Facultatibus Simplicium: 119

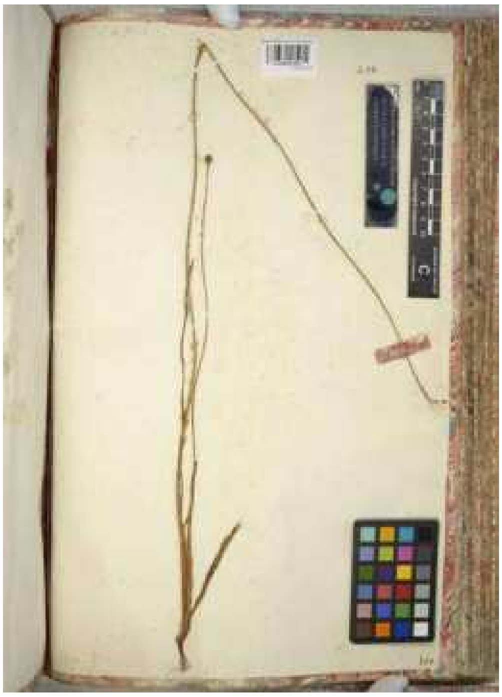

Marcgrave's herbarium: 150

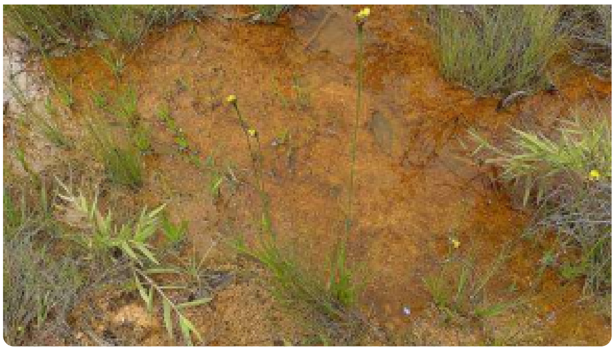

Plant, Bahia, Brazil; by Alex Popovkin (CC BY-NC 2.0)

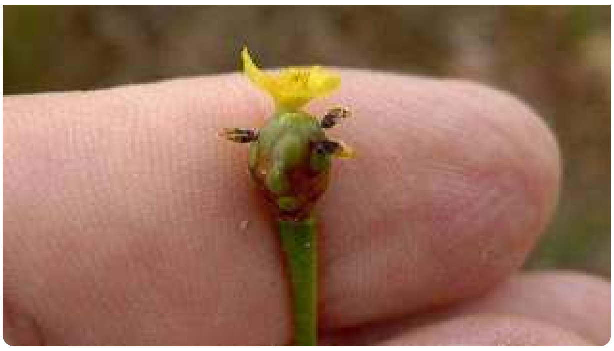

Inflorescence, Bahia, Brazil; by Alex Popovkin (CC BY-NC-SA 2.0)

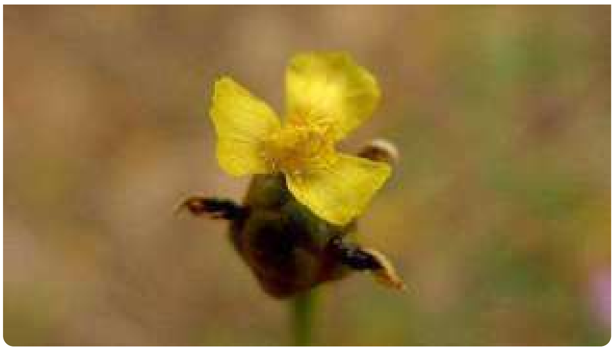

Detail of flower, Inflorescence, Bahia, Brazil; by Alex Popovkin (CC BY-NC-SA 2.0)

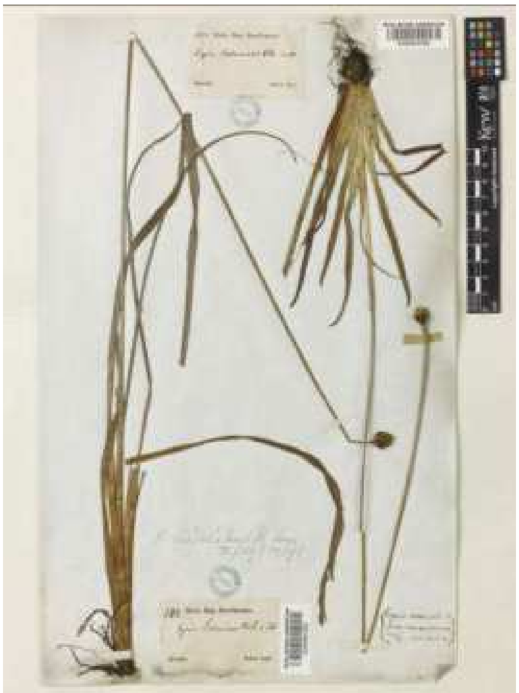

A specimen from Kew's Herbarium - K000894360. Retrieved from Plants of the World Online

Medicina Brasiliensi Piso, 1648 Page number 119b

Vernacular name(s): Guaianá-Timbó (name in p. 115)

Species: Tanaecium pyramidatum (Rich.) L.G.Lohmann

Family: Bignoniaceae

Notes

We did not find any correspondence between this woodcut and the contemporary or older sources.

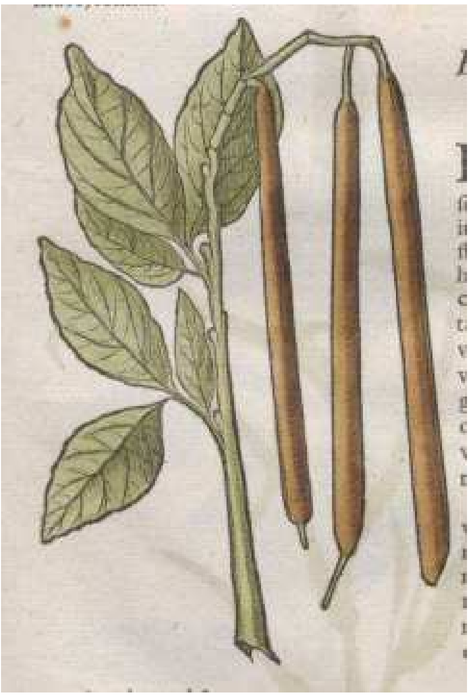

De Facultatibus Simplicium: 119

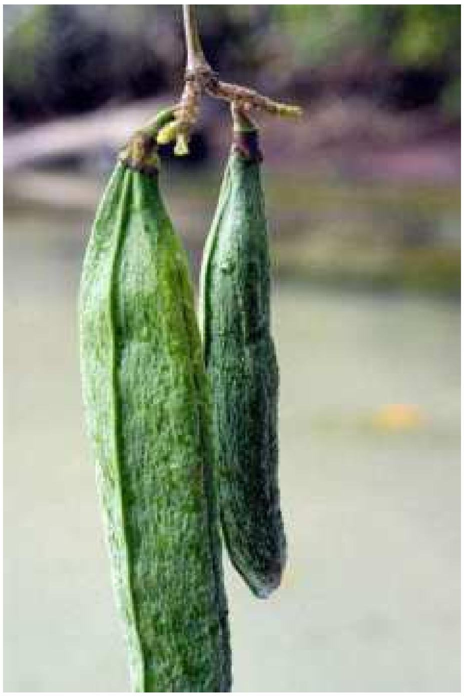

Fruits; by Pedro Acevedo-Rodríguez and O. Hernandez (CC0 1.0)

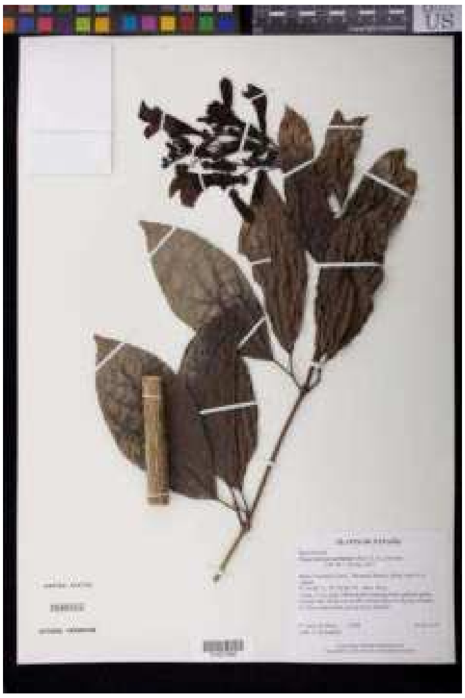

Specimen; by Pedro Acevedo-Rodríguez and O. Hernandez, Smithsonian Collections: 15208 (CC0 1.0)

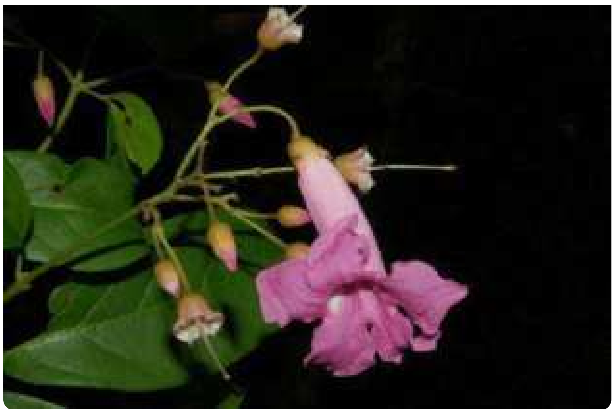

Specimen: Aona L.Y.S. 1941 (K000992383). Retrieved from Plants of the World Online

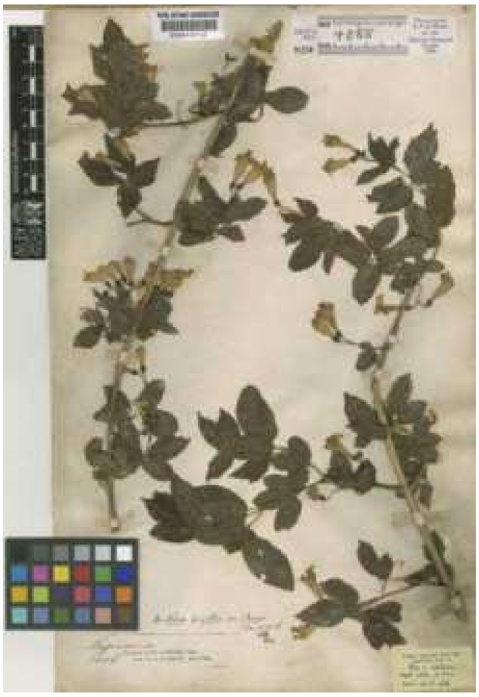

A specimen from Kew's Herbarium - K000449146. Retrieved from Plants of the World Online

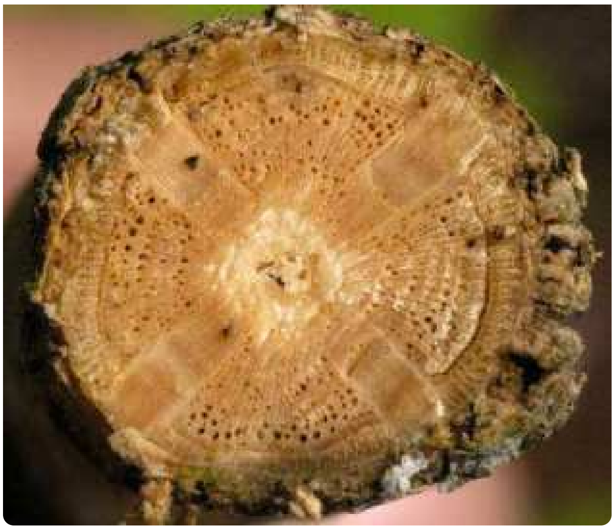

Wood; by Pedro Acevedo-Rodríguez and O. Hernandez (CC BY-NC 2.0)

Medicina Brasiliensi Piso, 1648 Page number 120a

Vernacular name(s) Masarandiba

Species Manilkara salzmannii (A.DC.) H.J.Lam

Family Sapotaceae

Notes

We did not find any correspondence between this woodcut and the contemporary or older sources.

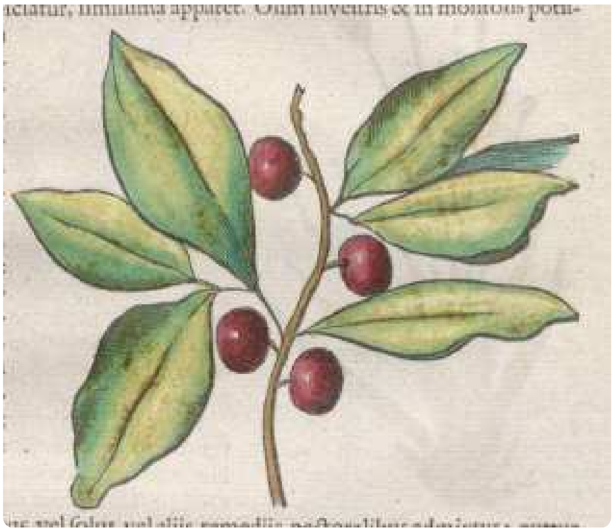

De Facultatibus Simplicium: 120

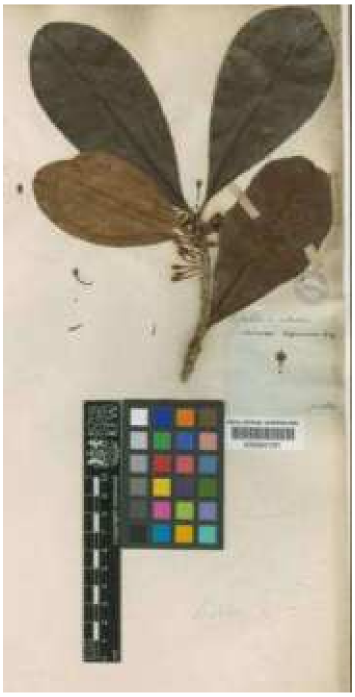

A specimen from Kew's Herbarium - K000641047.
Retrieved from Plants of the World Online

Medicina Brasiliensi Piso, 1648 Page number 120b

Vernacular name(s) Amongeaba

Species Setaria parviflora (Poir.) Kerguélen

Family Poaceae

Notes

The woodcut is very similar to the *Theatrum i*mage (non-reversed).

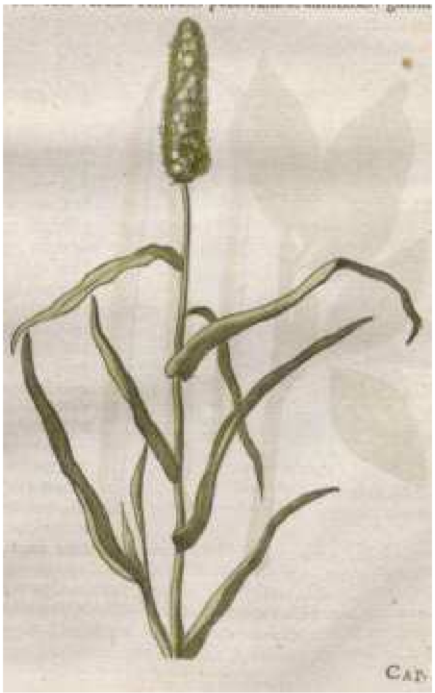

De Facultatibus Simplicium: 120

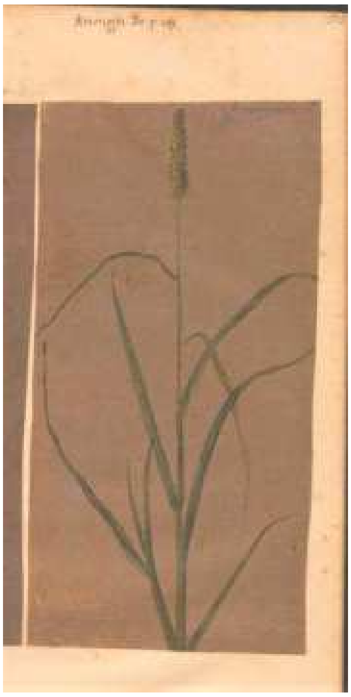

Theatrum Rerum Naturalium bound c.1660-1664: 373

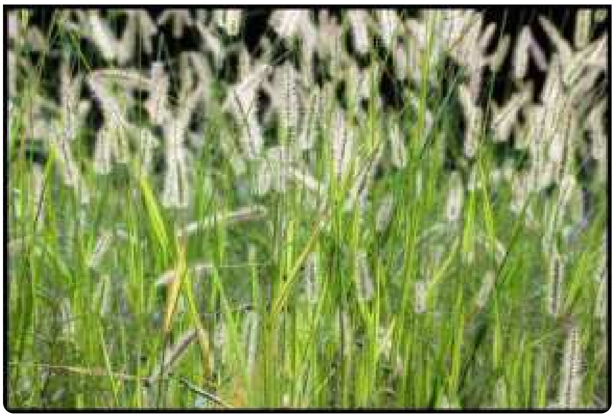

Plant; by Mauricio Mercadante (CC0 1.0)

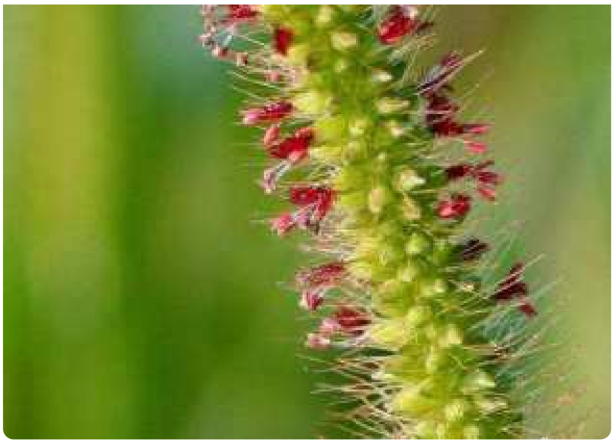

Flowers; by Mary Keim (CC BY-NC-SA 2.0)

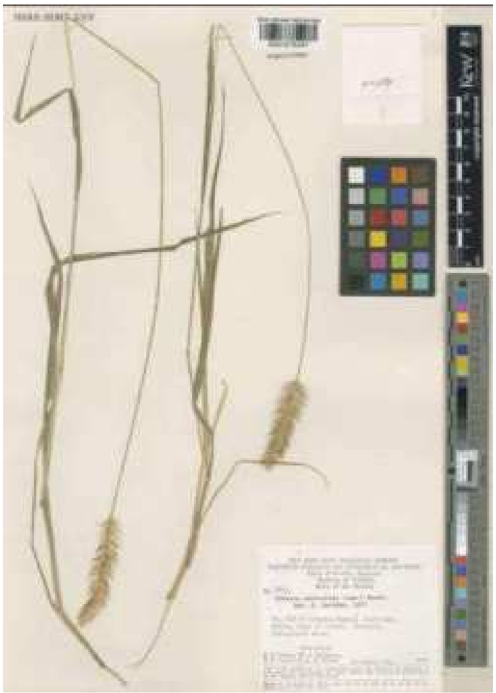

A specimen from Kew's Herbarium - K001078347.
Retrieved from Plants of the World Online

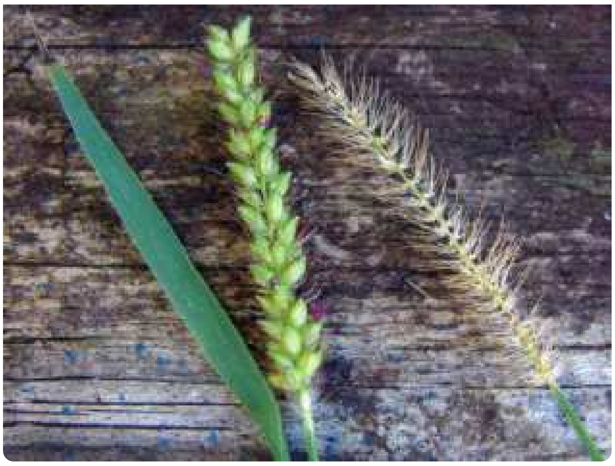

Plant; by Forest & Kim Starr (CC BY-NC 2.0)

Medicina Brasiliensi Piso, 1648 Page number 111c

Vernacular name(s) Caraguata guaçu

Species Furcraea tuberosa (Mill.) W.T.Aiton

Family Asparagaceae

Notes

We did not find any correspondence between this woodcut and the contemporary or older sources. The stem of this plant was sent to the *Hortus Botanicus* in Leiden (Piso 1648: 111). Piso (1658: 192) specified that he gave this stem to D. Vórstio. Adolpho Vorstius, also known as Adolph van Voorst (1597-1663), was the director of the Hortus in 1633 and compiled a catalog (*Horti academici Lugduno-Batavi catalogus*, 1633) of its living collection. This included many plants shipped from the Americas (Schoneveld 1996:22). He was Marcgrave´s former professor of botany at Leiden University. Identification by botanist Dr. Abisai Josue García Mendoza.

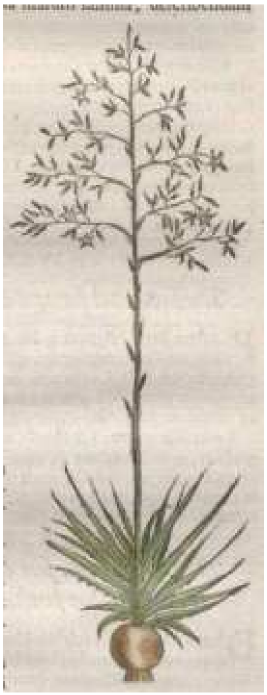

De Facultatibus Simplicium: 111

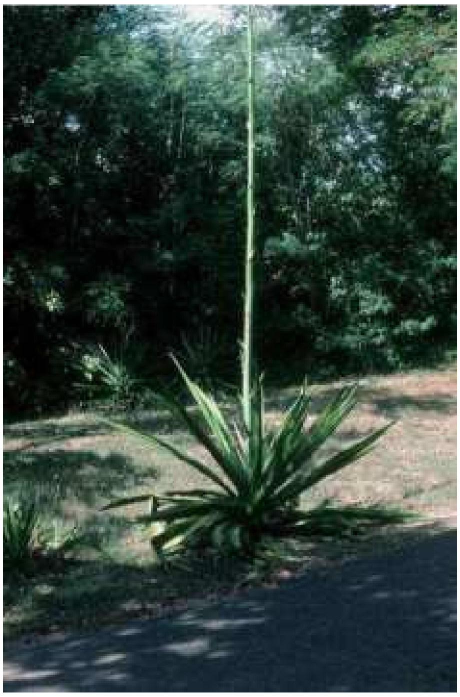

Collection of the Smithsonian Institution (CC BY-SA 4.0)

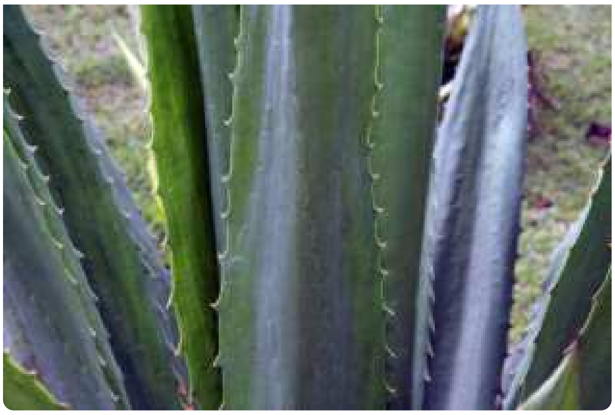

Spiny leaves; by David J. Stang (CC0 1.0)

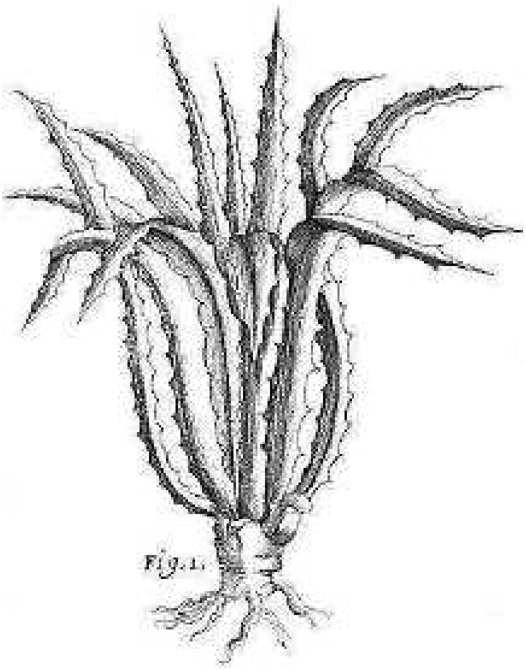

Type. Plukenet, Leonard (1696), "Tab. 258, Fig. 1", Phytographia, London. Retrieved from Wikipedia

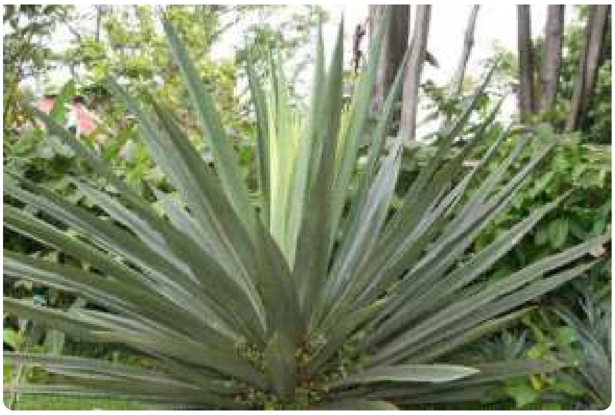

Leaves; by David J. Stang. Retrieved from Wikipedia

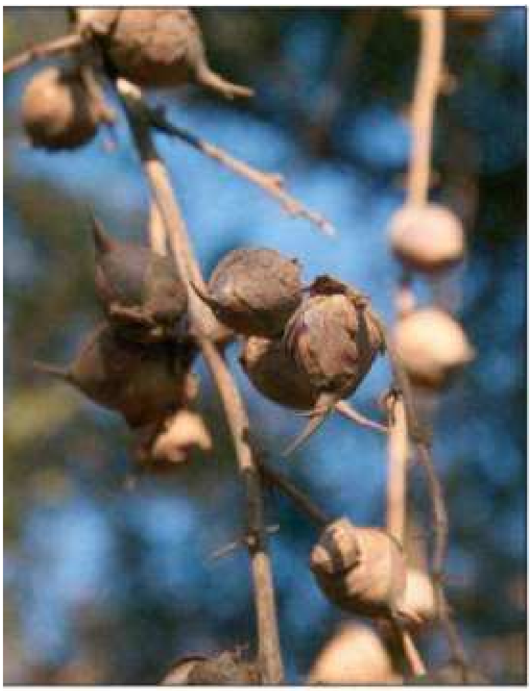

Bulbils; by Gideon F. Smith. Taken from Smith, G. F. & Figueiredo, E. (2012), Bradleya, 30: 107–110

Historiae Rerum Naturalium Brasiliae Marcgrave, 1648 Page number 3

Vernacular name(s) Iataboca

Species Guadua angustifolia Kunth

Family Poaceae

Notes

We did not find any correspondence between this woodcut and the contemporary or older sources

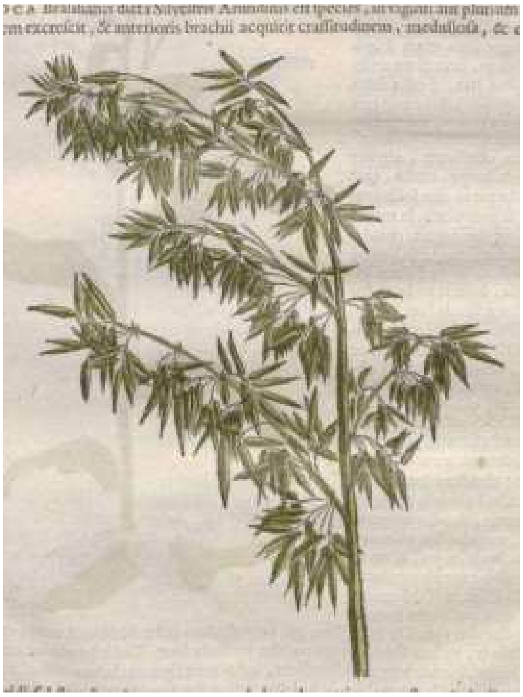

Historiae Plantarum – Herbis: 3

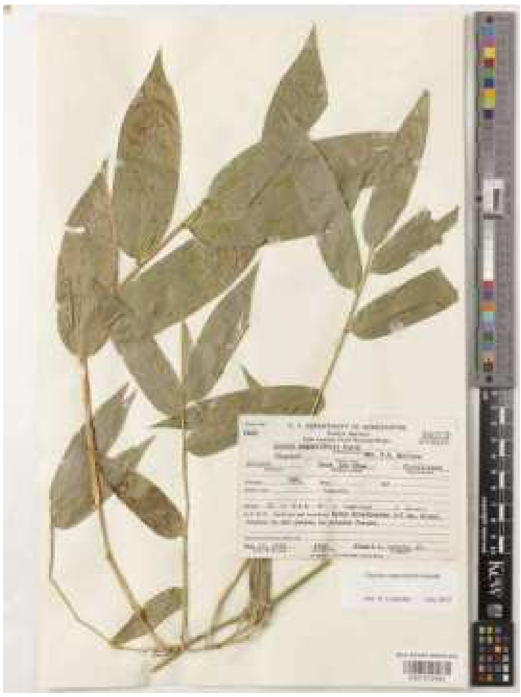

A specimen from Kew's Herbarium - K001472492. Retrieved from Plants of the Wold Online

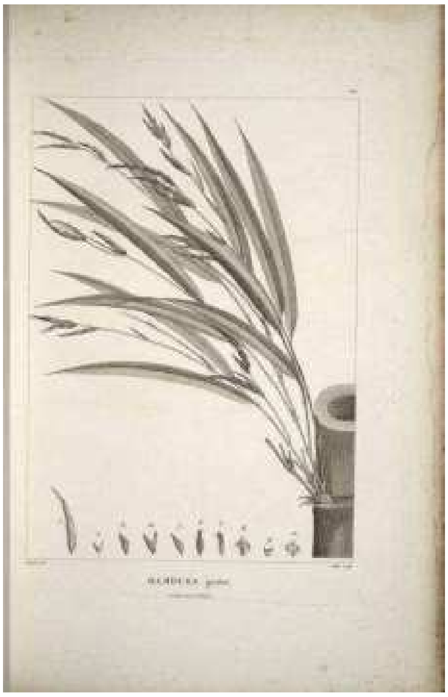

Engraving of *G. angustifolia* in *Plantes equinoxiales* (1808-1809) by Humboldt, F.H.A. von, Bonpland, A. (1808: Vol I, t. 20)

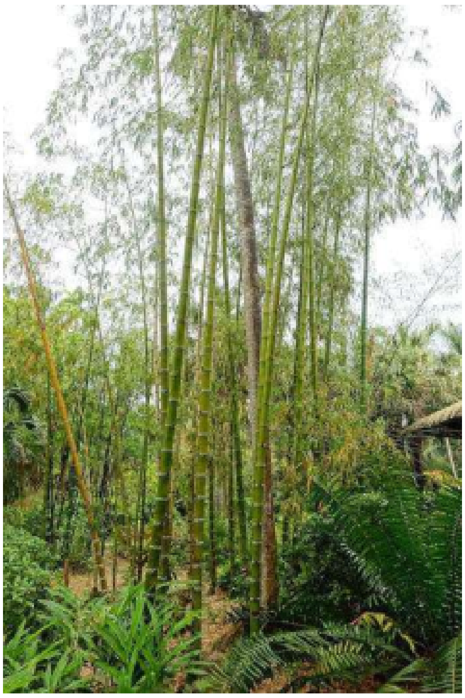

G. angustifolia in McKee Botanical Garden - USA, by Daderot (CC0 1.0)

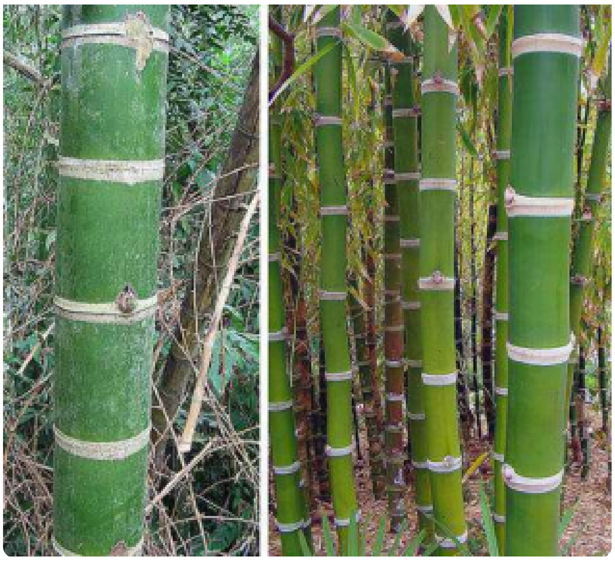

"*G. angustifolia*, the Giant Neotropical Bamboo" by Dick Culbert (CC BY 2.0)

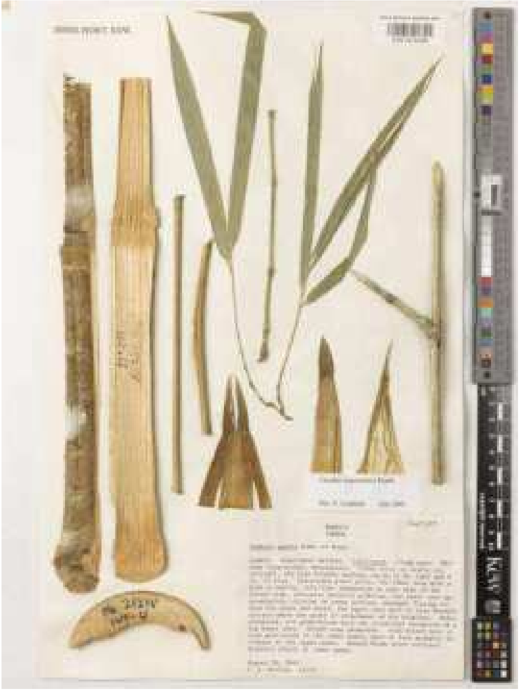

A specimen from Kew's Herbarium - K001472436. Retrieved from Plants of the Wold Online

Historiae Rerum Naturalium Brasiliae Marcgrave, 1648 Page number 4

Vernacular name(s) Meeru

Species Canna indica L.

Family Cannaceae

Notes

Slightly similar to the *Theatrum* image but it bears more resemblance to the *C. indica* in the *Misc. Cleyeri* (in reversed format) than in the *Theatrum*. The folio from the *Misc. Cleyeri* came together with the illustrations from the *Theatrum* before Mentzel leave them out of the *Theatrum* collection when assembling it.

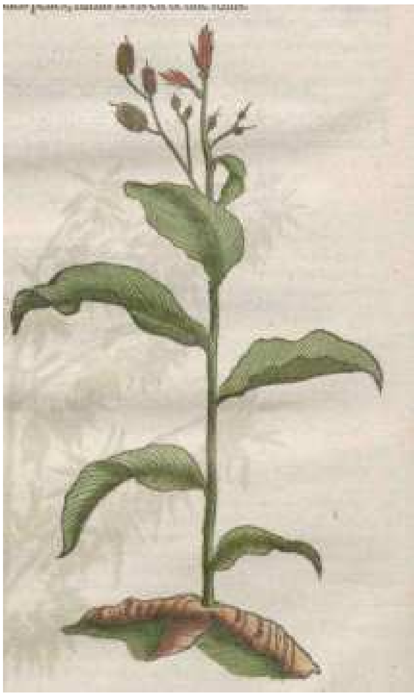

Historiae Plantarum – Herbis: 4

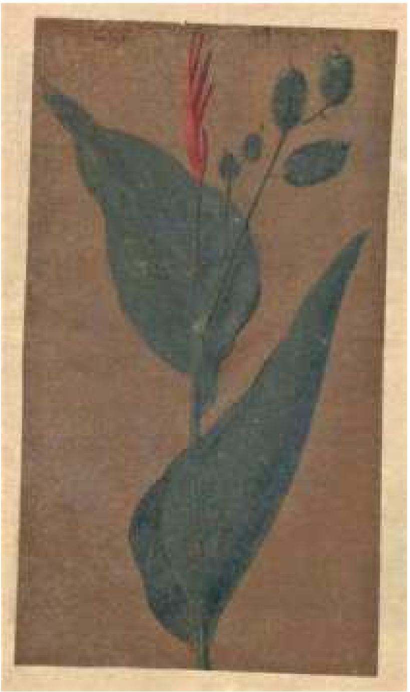

Miscellanea Cleyeri c.1637-44: 54r

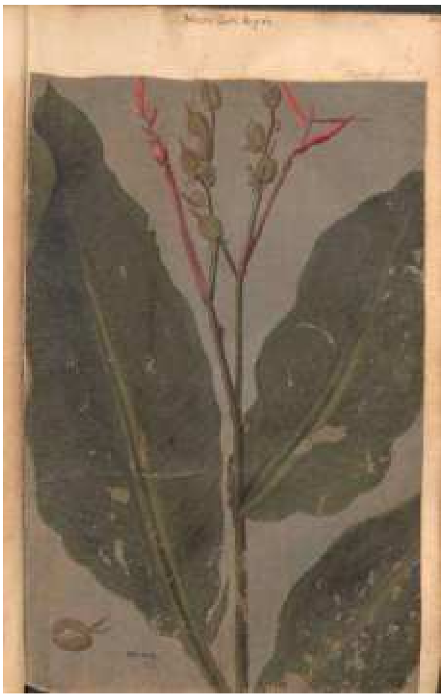

Theatrum Rerum Naturalium: 173

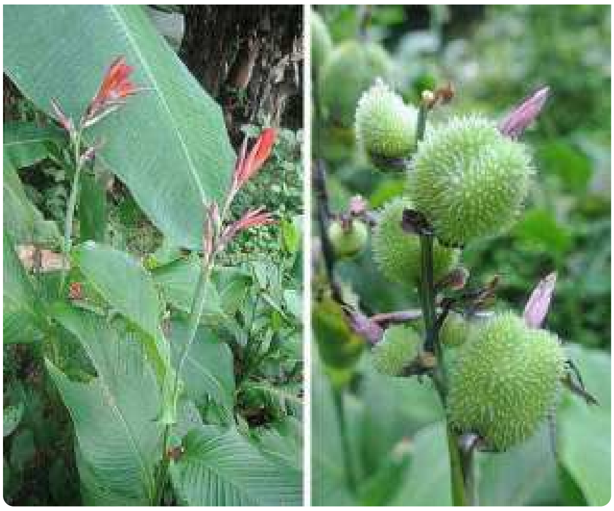

Flowers and fruits of *Canna indica* by Dick Culbert. retrieved from https://www.flickr.com/ (CC BY 2.0)

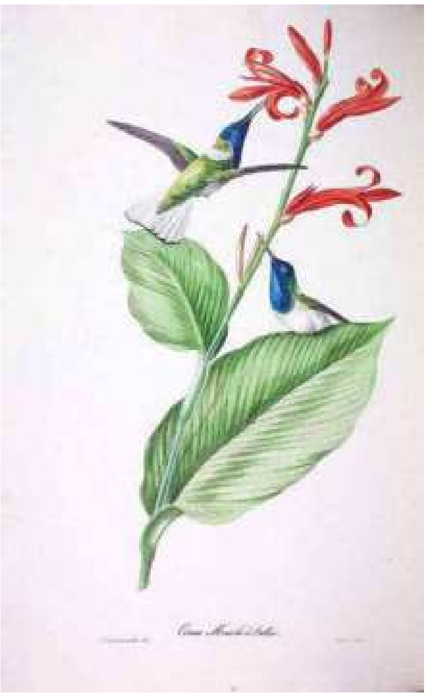

C. indica with hummingbirds, in *Oiseaux brillans du Brésil* by Descourtilz, J.T. (1834: t. 50)

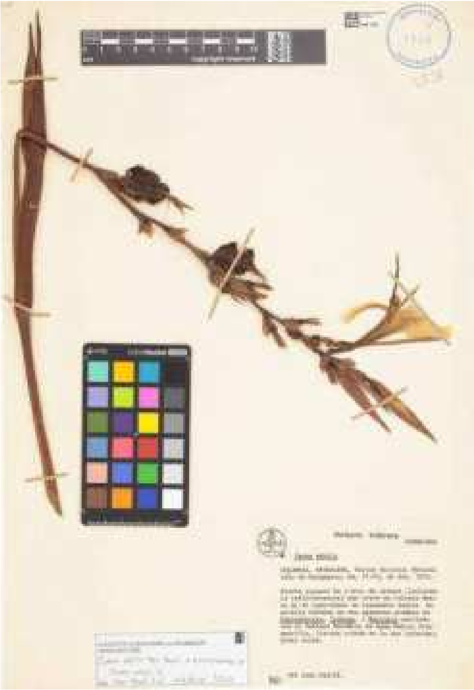

Specimen of *C. indica*, by Herbario virtual FMB - FMB1592 (CC BY-NC 2.0)

Historiae Rerum Naturalium Brasiliae Marcgrave, 1648 Page number 5

Vernacular name(s) Alia species

Species Canna glauca L.

Family Cannaceae

Notes

We did not find any correspondence between this woodcut and the contemporary or older sources

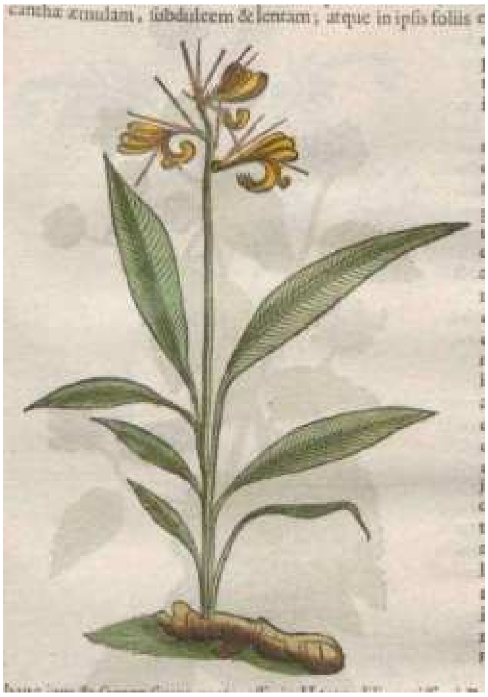

Historiae Plantarum – Herbis: 5

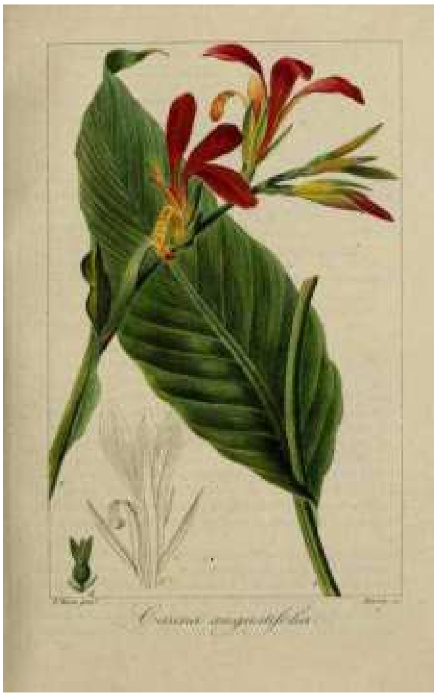

C. glauca illustration, in
Herb. Gén. Amat. by Delaunay, M., Loiseleur-Deslongchamps, J.L.A. (1819: Vol. III, t. 189)

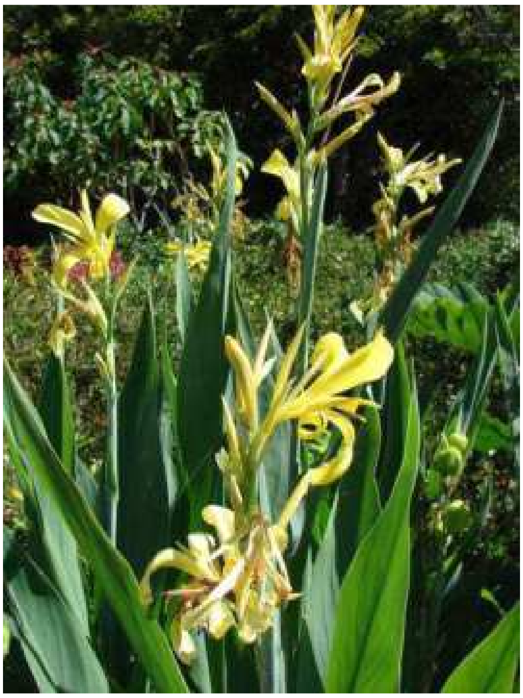

"*C. glauca* flowers and leaves *Enchanting Floral Gardens of Kula-Maui*" by Forest and Kim Starr (CC BY 3.0)

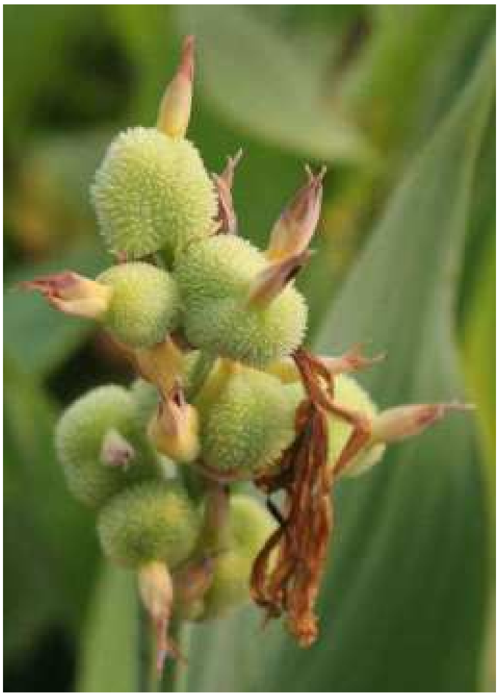

Fruits. "*C. glauca*" by Cerlin Ng (CC BY-NC-SA 2.0)

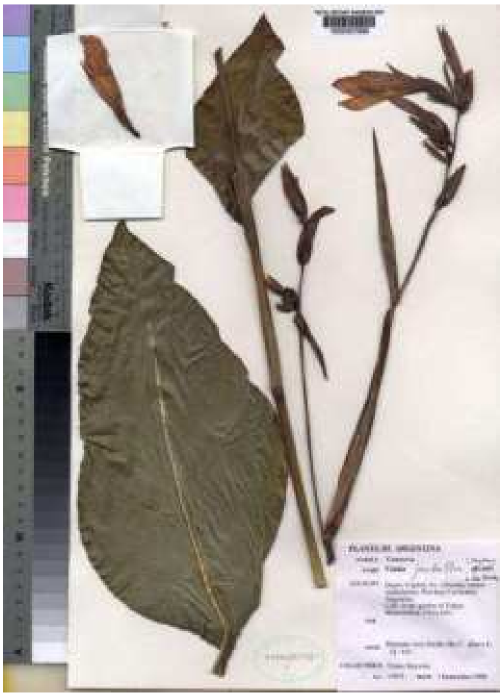

C. glauca specimen from Kew's Herbarium - K000307936. Retrieved from Plants of the World Online

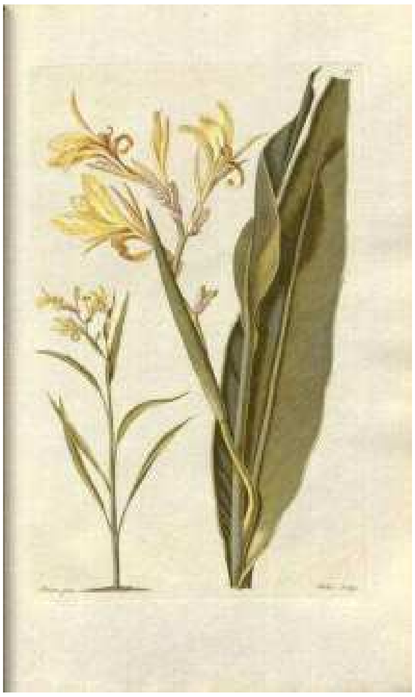

Engraving of *C. glauca* in *Thesaurus Botanicus* by Trattinnick, L. (1805-1819: t. 25). Retrieved from Plantillustrations.org

Historiae Rerum Naturalium Brasiliae Marcgrave, 1648 Page number 6

Vernacular name(s) Camara. Camara iuba

Species Lantana camara L.

Family Verbenaceae

Notes

The woodcut is very similar to the *Theatrum* image (reversed).

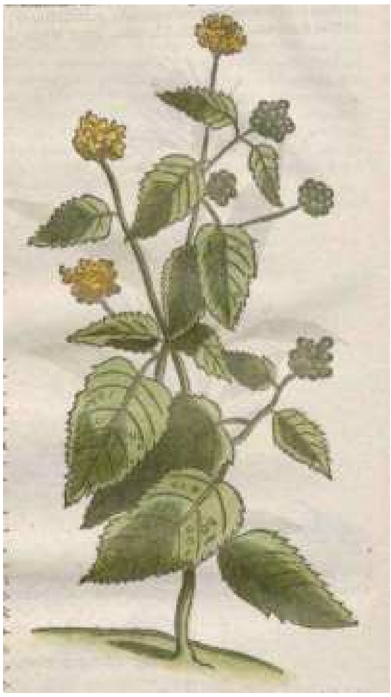

Historiae Plantarum – Herbis: 6

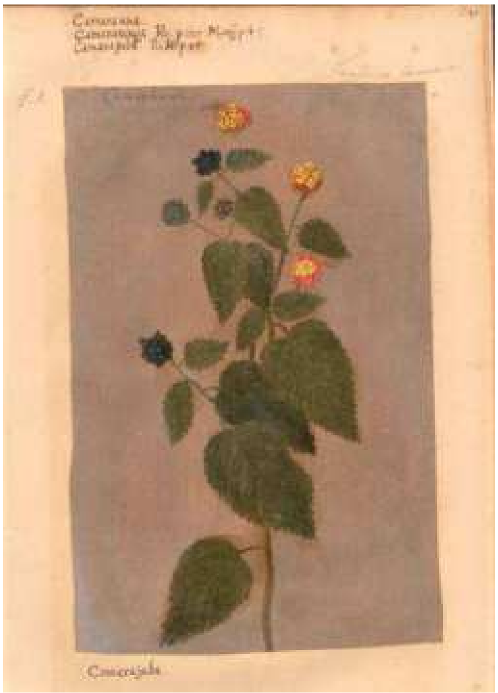

Theatrum Rerum Naturalium: 341

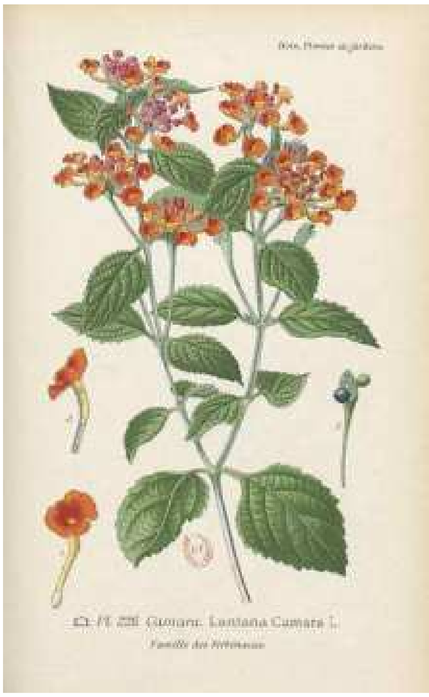

Illustration of *L. camara* in *Atlas des plantes de jardins et d'appartements* by Bois, D. (1891-1896: Vol. II, t. 226). Univ. of Illinois Urbana-Champaign, U.S.A.

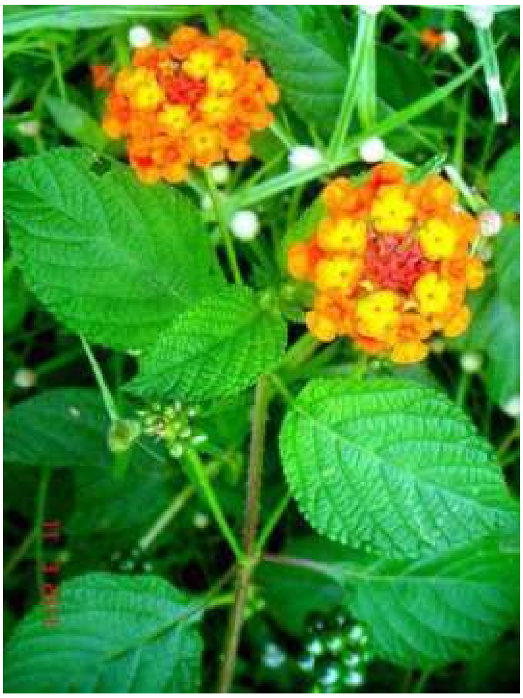

Flowers and fruits. "*L. camara*" by Ahmad Fuad Morad (CC BY-NC-SA 2.0)

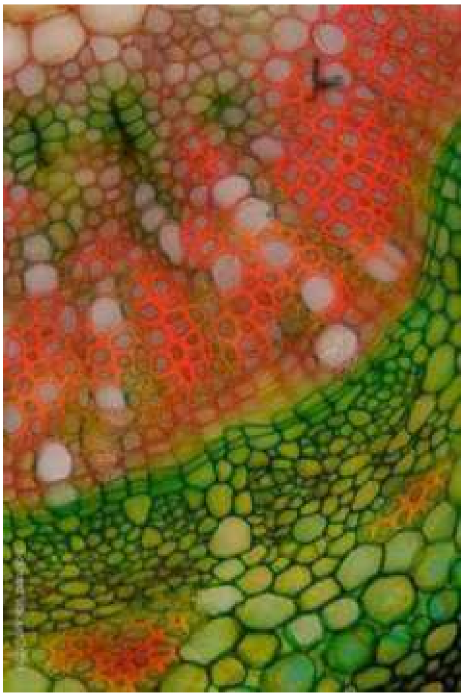

Plant tissue of the stem of *L. camara* under a microscope (20x), by Tatcher a Hainu (CC BY-NC 2.0)

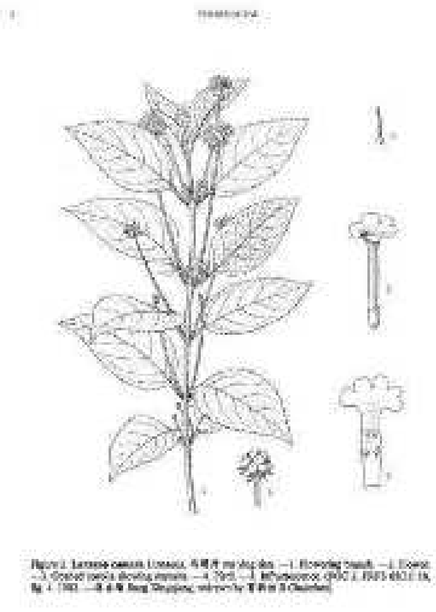

"*L. camara*, line drawing" by filibot.web (CC BY-NC-SA 2.0)

Historiae Rerum Naturalium Brasiliae Marcgrave, 1648 Page number 7a

Vernacular name(s) Iacua Acanga. Fedagozo

Species Heliotropium indicum L.

Family Boraginaceae

Notes

The woodcut is slightly similar to the *Theatrum* image.

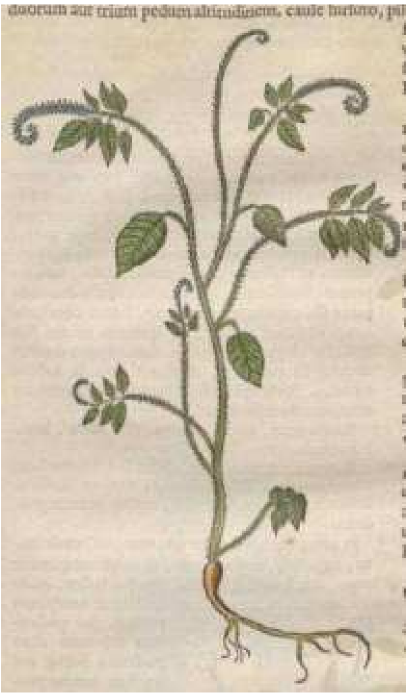

Historiae Plantarum – Herbis: 7a

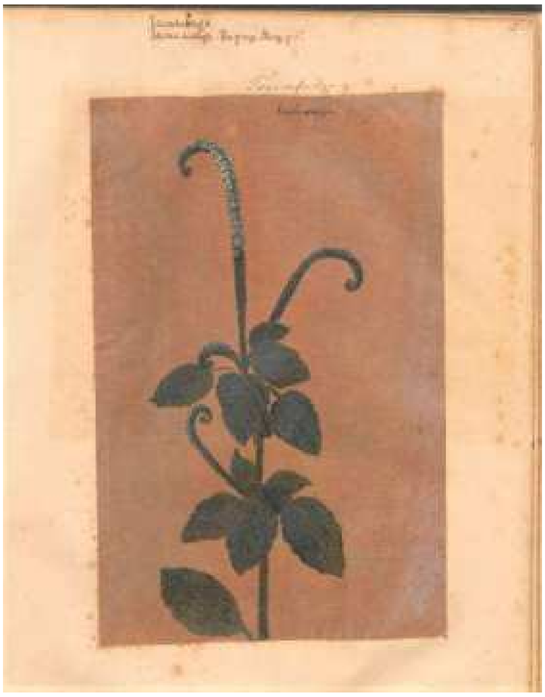

Theatrum Rerum Naturalium: 517

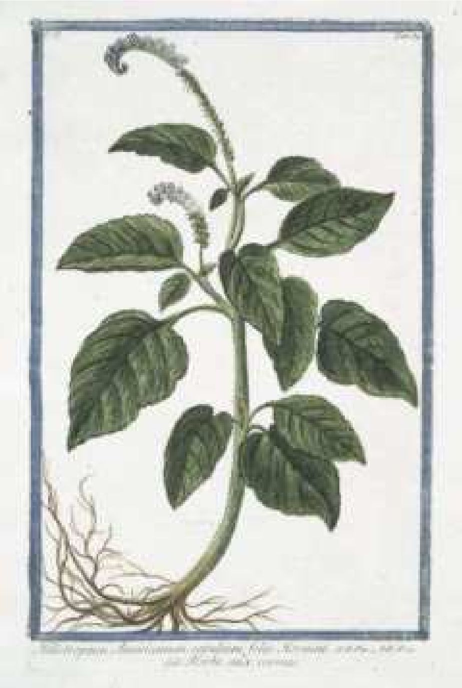

H. indicum in *Hortus Romanus juxta Systema Tournefortianum* by Bonelli, G. (1772-1793, Vol. II, t. 34)

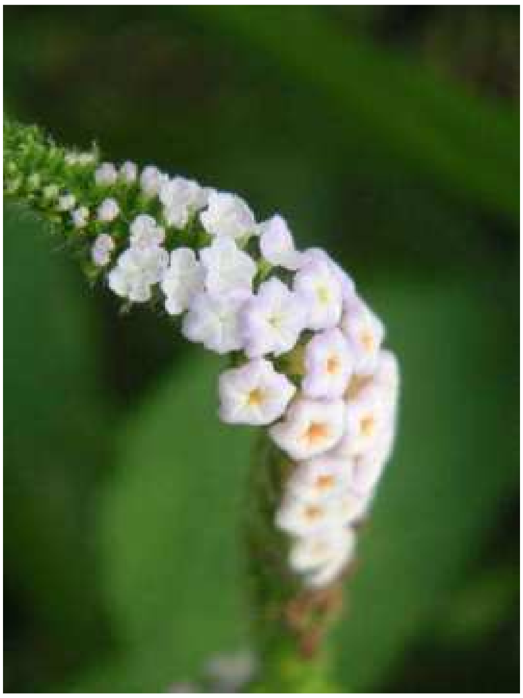

Flowers "*H. indicum*" by acerhwng (CC BY 2.0)

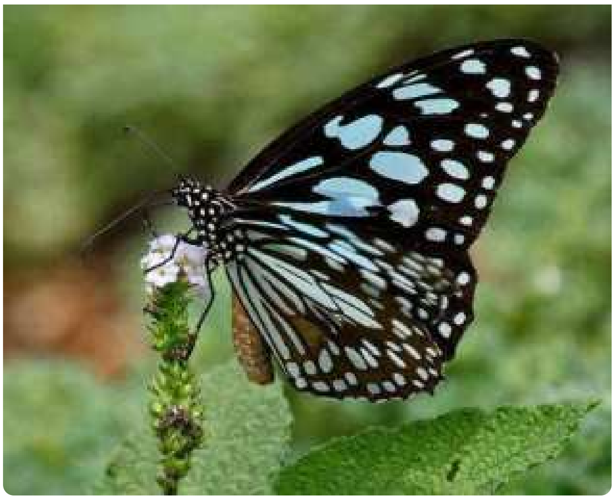

"Blue Tiger (*Tirumala limniace*) on *Heliotropium indicum* W IMG 9939.jpg" by J.M.Garg (CC BY 3.0)

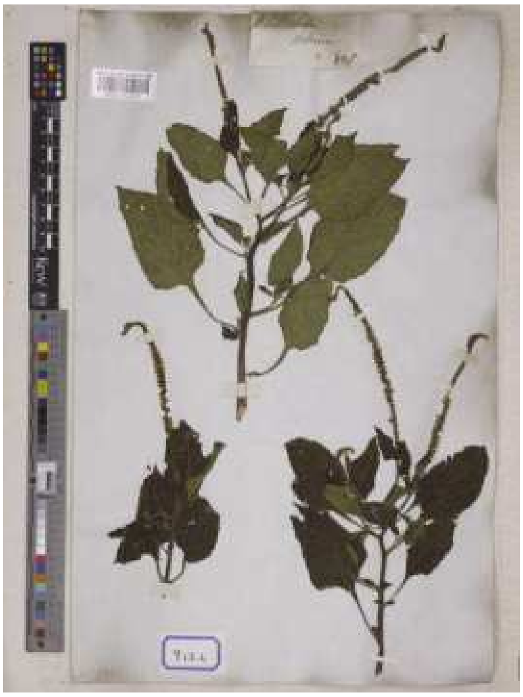

H. indicum specimen from Kew's Herbarium - K001110275. Retrieved from Plants of the World Online

Historiae Rerum Naturalium Brasiliae | Marcgrave, 1648 | Page number 7b

Vernacular name(s) Caacica

Species Euphorbia hirta L.

Family Euphorbiaceae

Notes

We did not find any correspondence between this woodcut and the contemporary or older sources.

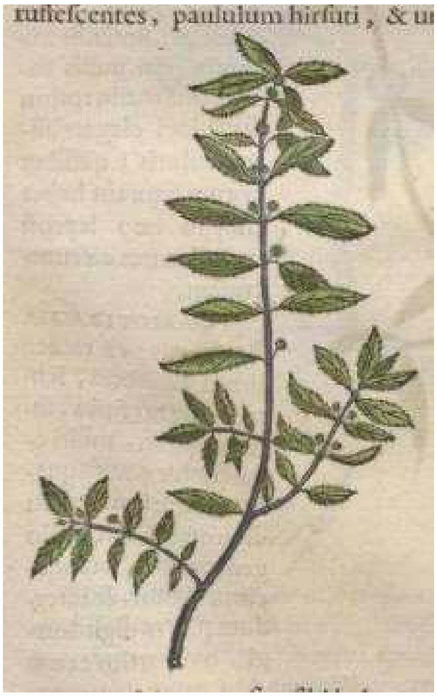

Historiae Plantarum – Herbis: 7b

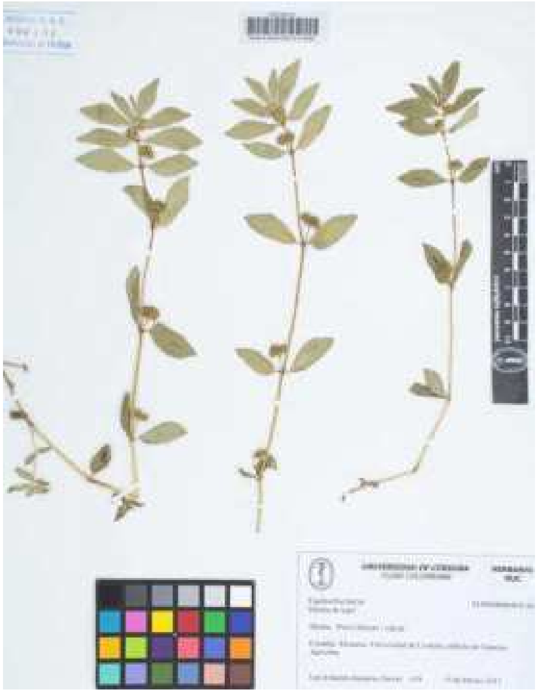

Specimen of "*E. hirta*" by herbariohuc, Colombia (CC BY-NC 2.0)

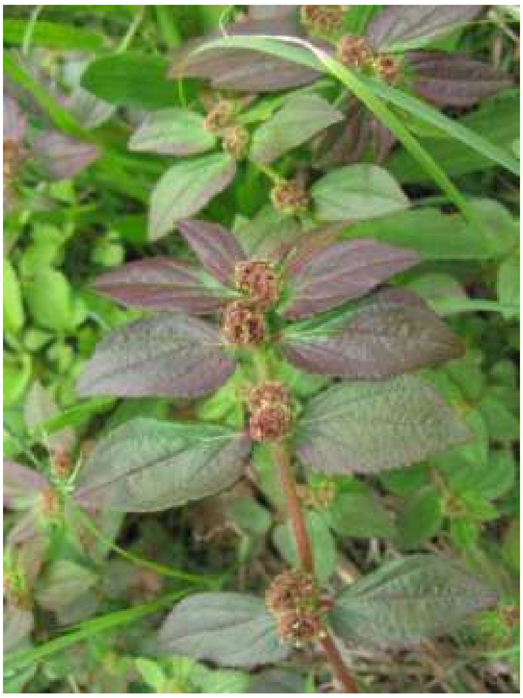

"*E. hirta* branch5" by Macleay Grass Man (CC BY 2.0)

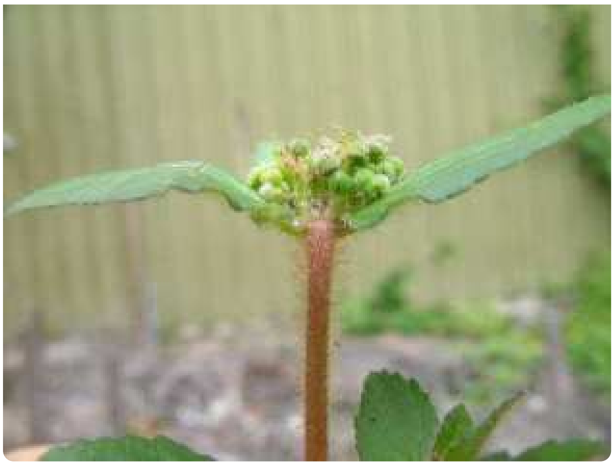

"*E. hirta* flower2" by Macleay Grass Man (CC BY 2.0)

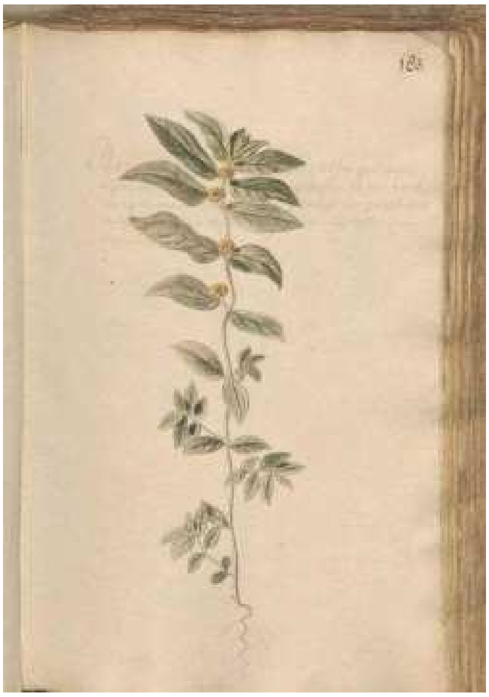

Illustration of *E. hirta* in *Plantarum Malabaricum icones*, Anonymous(1694-1710, Vol. II, t. 183). Leiden University Library, Netherlands

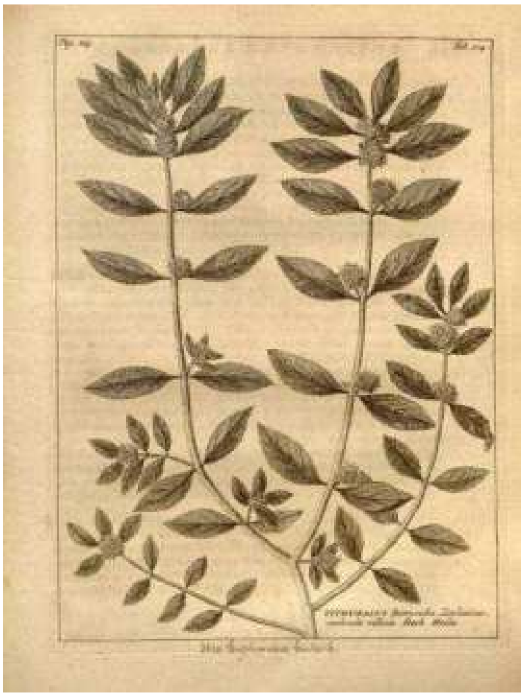

Engraving of *E. hirta* in *Thesaurus Zeylanicus* by Burman, J. (1737: t. 104). Missouri Botanical Garden, St. Louis, U.S.A.

Historiae Rerum Naturalium Brasiliae Marcgrave, 1648 Page number 8a

Vernacular name(s) Planta haec

Species Commelina erecta L.

Family Commelinaceae

Notes

No resemblance between the woodcut image and the watercolor. The flower is painted with more detail in the *Libri Principis* (petals and stamens are visible).

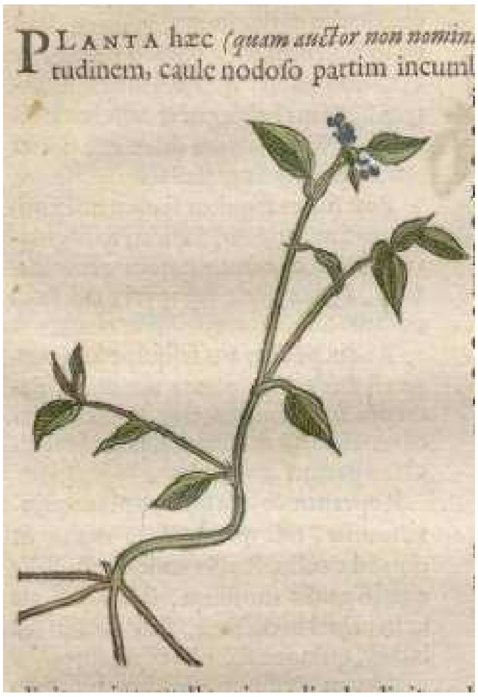

Historiae Plantarum – Herbis: 8a

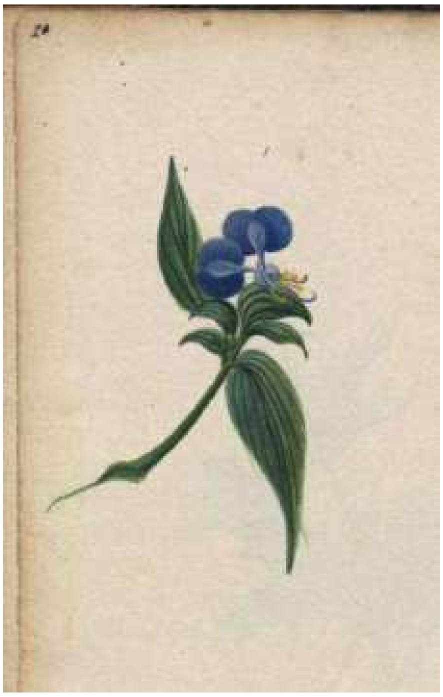

Libri Principis f. 20

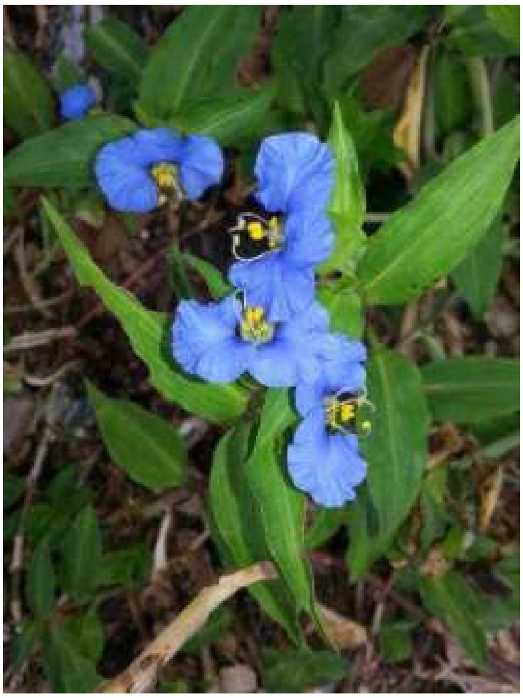

"*C. erecta* 2" by Zachi Evenor (CC BY 2.0)

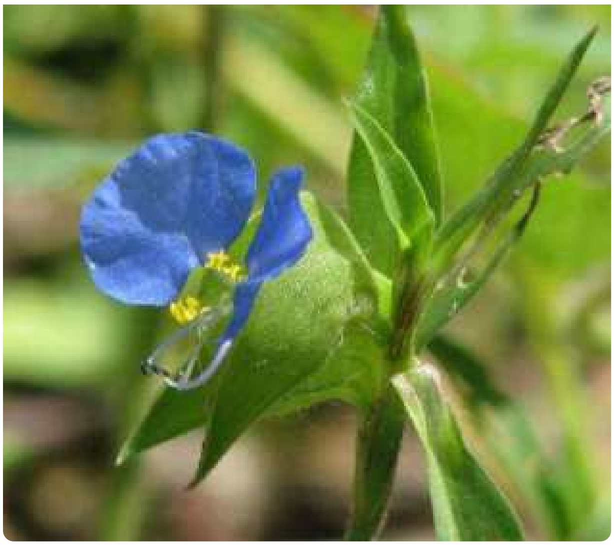

"Common Dayflower (*C. erecta*) at Presidio San Saba" by mlhradio (CC BY-NC 2.0)

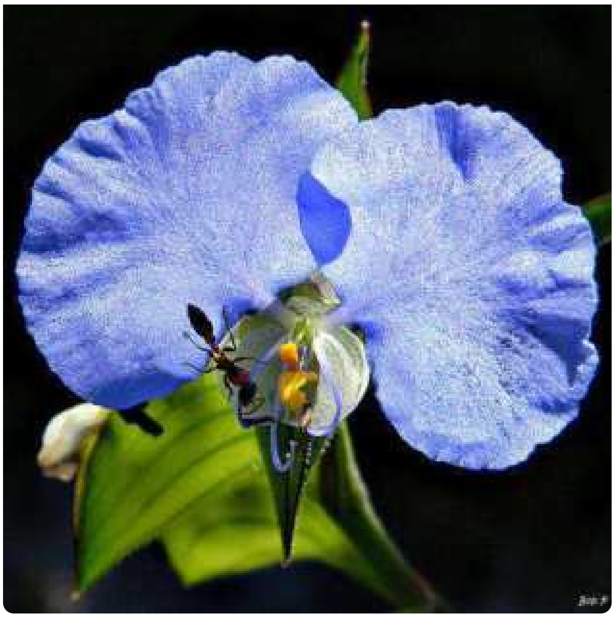

"Whitemouth dayflower (*C. erecta*)" by Bob Peterson (CC BY 2.0)

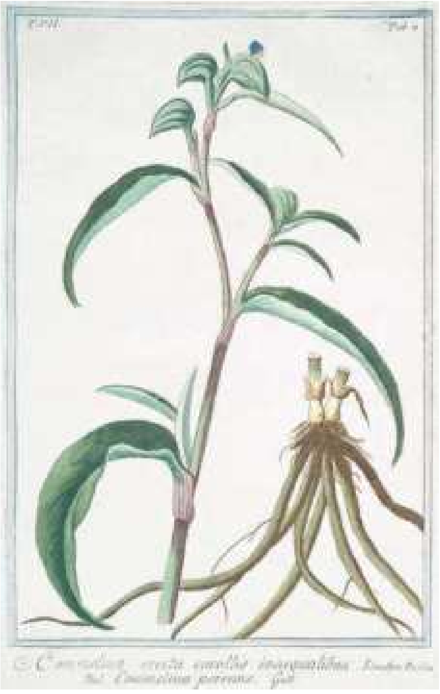

Illustrated Botanical Books: Flowers, Plants, and Trees from NYPL collections. (PICRYL-(http://digitalcollections.nypl.org/)

Historiae Rerum Naturalium Brasiliae Marcgrave, 1648 Page number 8b

Vernacular name(s) Caapotiragóa

Species Mitracarpus hirtus (L.) DC.

Family Rubiaceae

Notes

We did not find any correspondence between this woodcut and the contemporary or older sources.

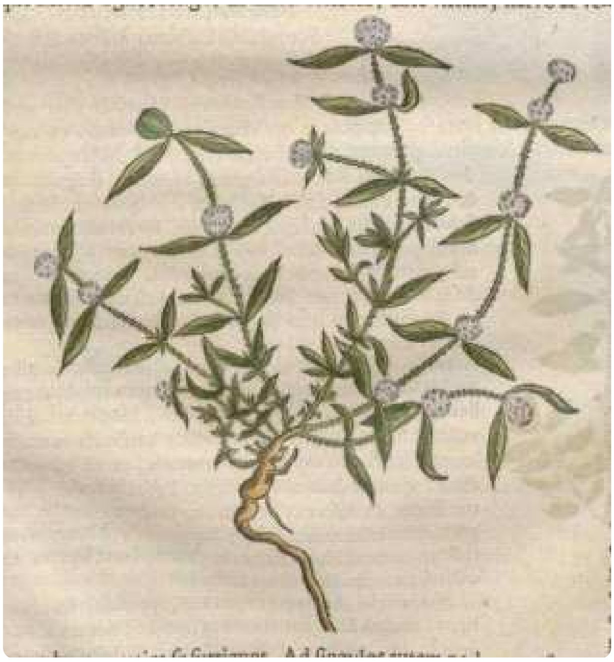

Historiae Plantarum – Herbis: 8b

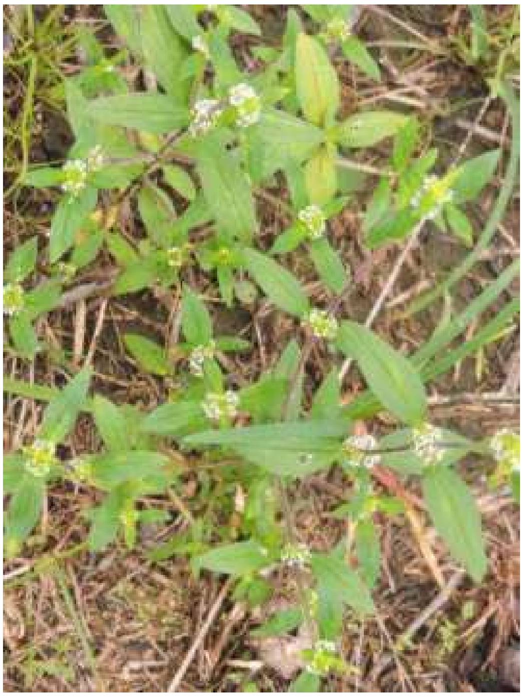

"*M. hirtus*" by Barry Hammel (CC BY-NC-SA 2.0)

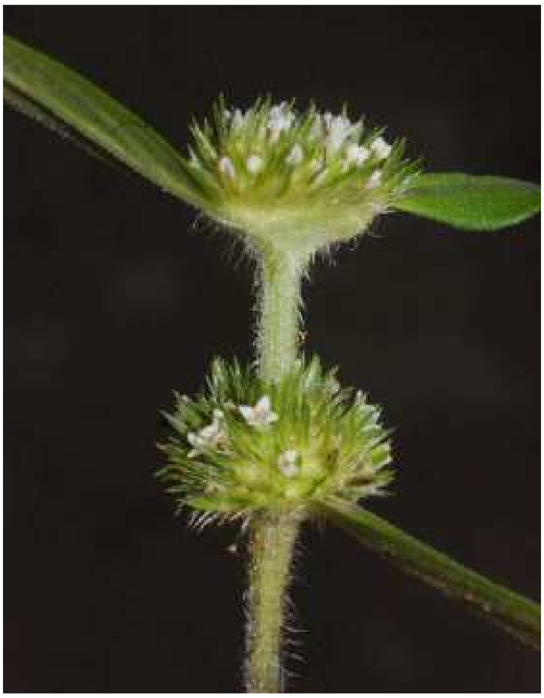

"*M. hirtus*" by Barry Hammel (CC BY-NC-SA 2.0)

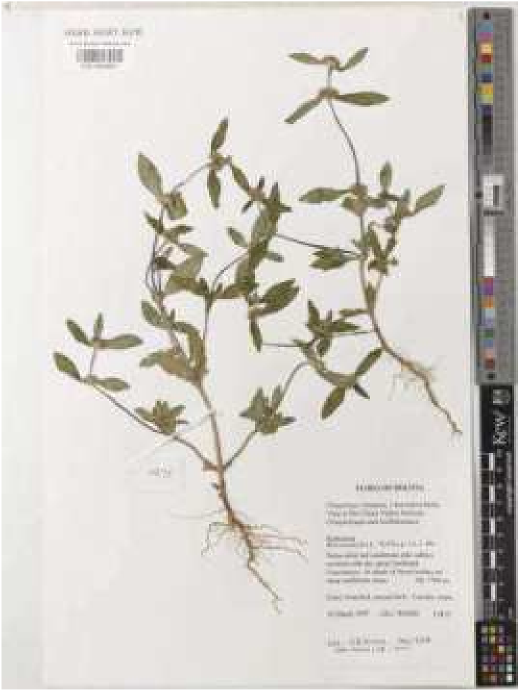

M. hirtus specimen from Kew's Herbarium - K001493651. Retrieved from Plants of the World Online

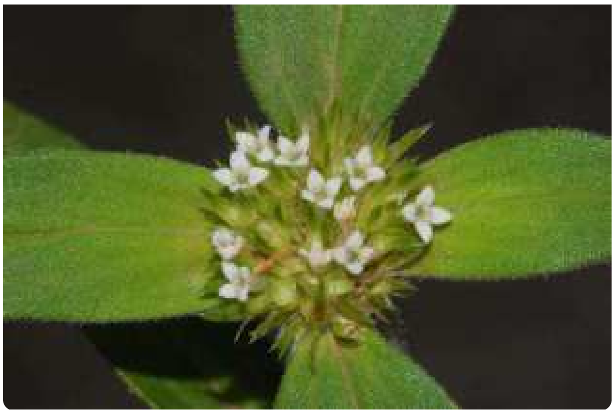

Flowers. "*M. hirtus*" by Barry Hammel (CC BY-NC-SA 2.0)

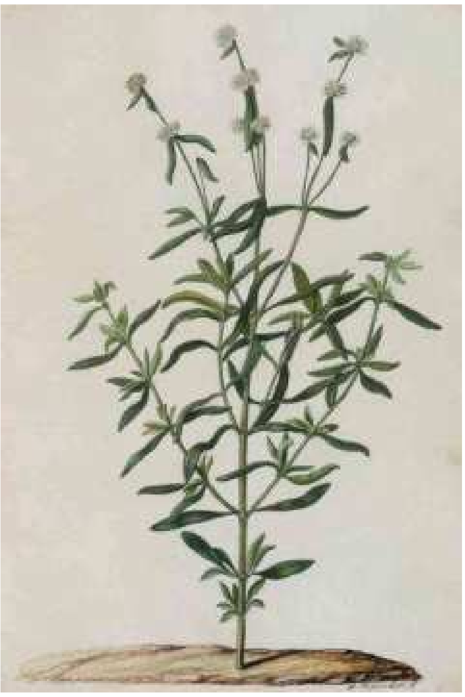

Illustration of *M. hirtus* in *Moninckx Atlas* by Moninckx, J. (1682: Vol. 8, t. 13). University of Amsterdam, Netherlands

Historiae Rerum Naturalium Brasiliae | Marcgrave, 1648 | Page number 9

Vernacular name(s) Paiomirioba

Species Senna occidentalis (L.) Link

Family Fabaceae

Notes

We did not find any correspondence between this woodcut and the contemporary or older sources.

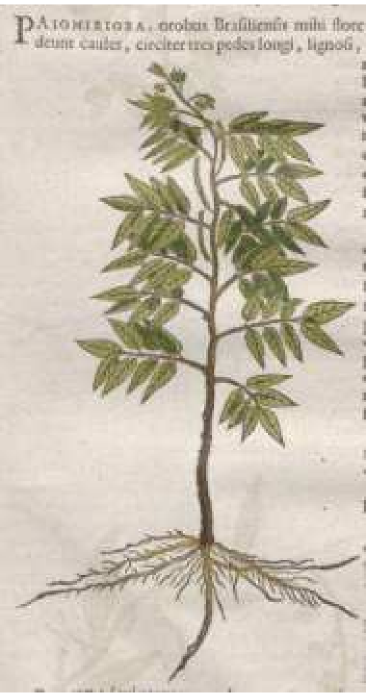

Historiae Plantarum – Herbis: 9

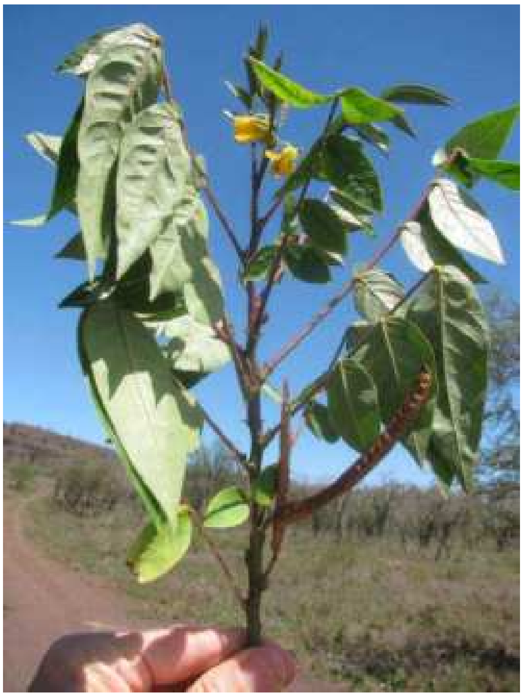

"*S. occidentalis* leaves and seedpods Ulupalakua Ranch Maui (25219812195).jpg" by Forest and Kim Starr (CC BY 3.0)

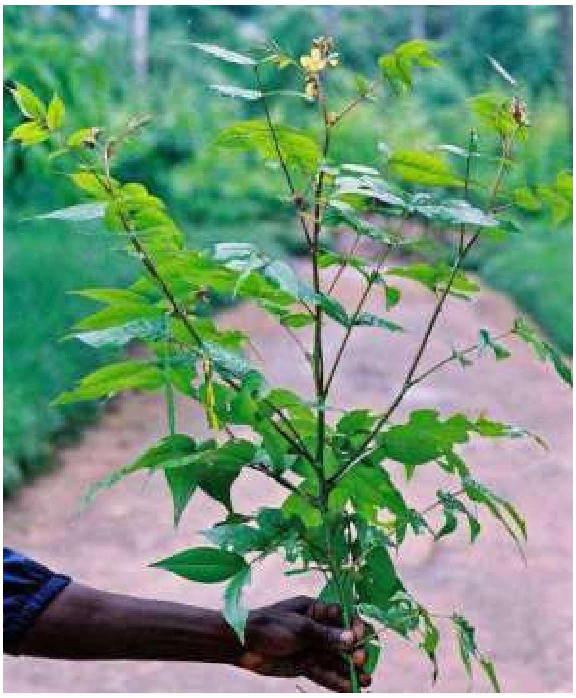

"*S. occidentalis* (FABACEAE)" by Scamperdale (CC BY-NC 2.0)

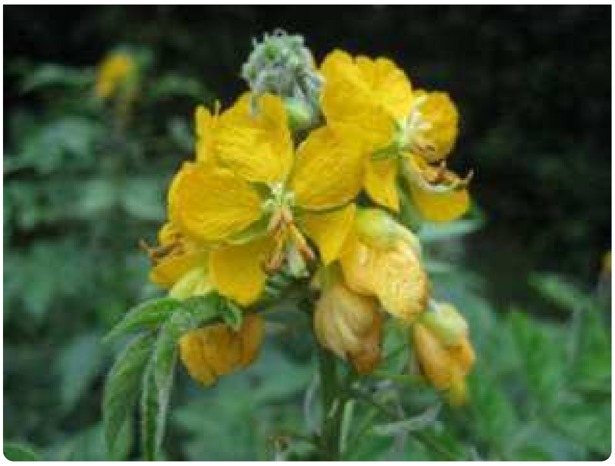

"*S. occidentalis* (FABACEAE)" by Scamperdale (CC BY-NC 2.0)

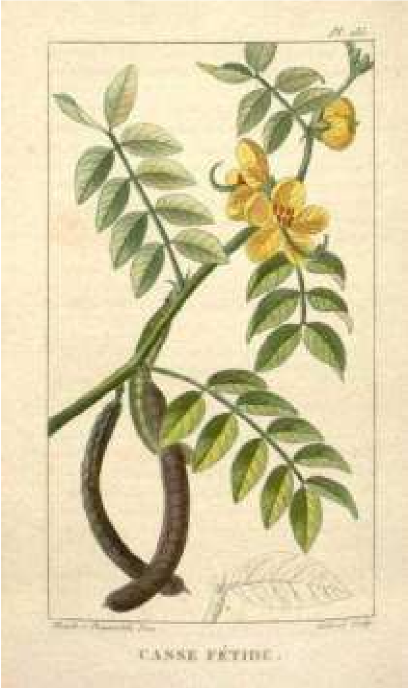

Flore [pittoresque et] médicale des Antilles by Descourtilz, M.E. (1822: Vol. II, t. 135). Missouri Botanical Garden, St. Louis, U.S.A.

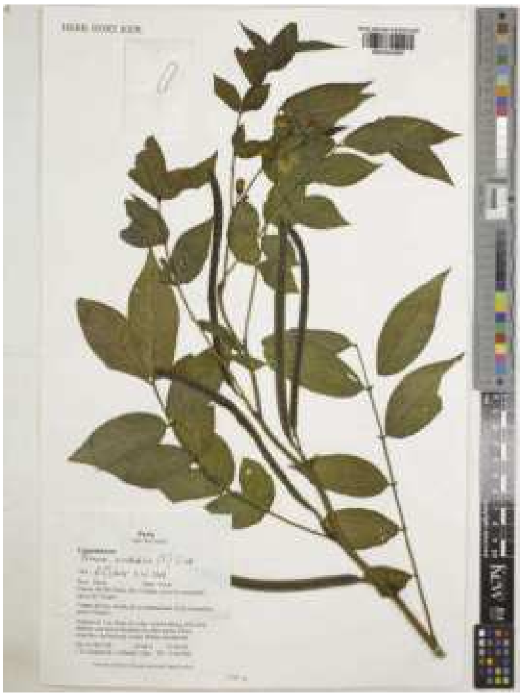

S. occidentalis specimen from Kew's Herbarium - K001041857. Retrieved from Plants of the World Online

Historiae Rerum Naturalium Brasiliae	Marcgrave, 1648	Page number	10a

Vernacular name(s) Tareroqui. Matapasto

Species Senna obtusifolia (L.) H.S.Irwin & Barneby

Family Fabaceae

Notes

No resemblance between the woodcut image and the watercolor. The woodcut shows an sterile branch while the watercolor depicts a branch with yellow flowers.

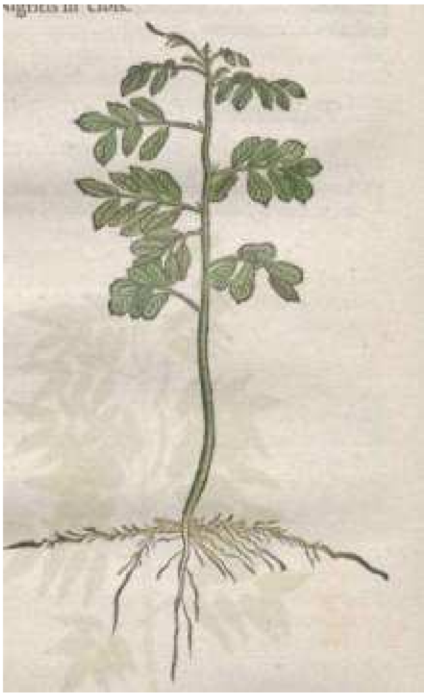

Historiae Plantarum – Herbis: 10a

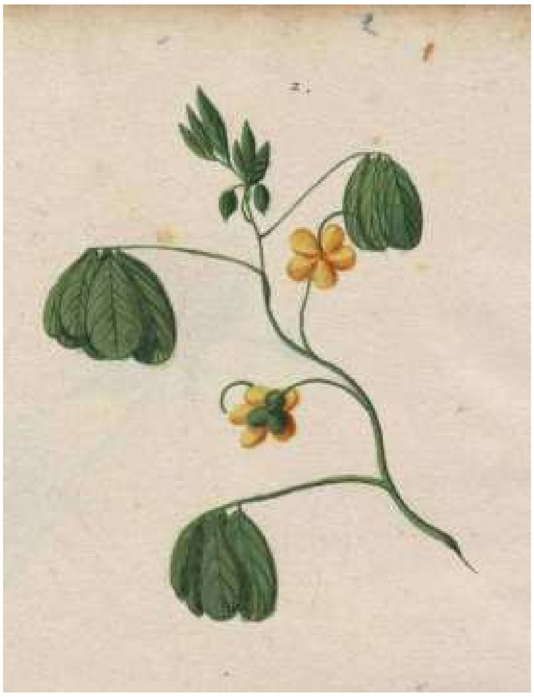

Libri Principis f. 20

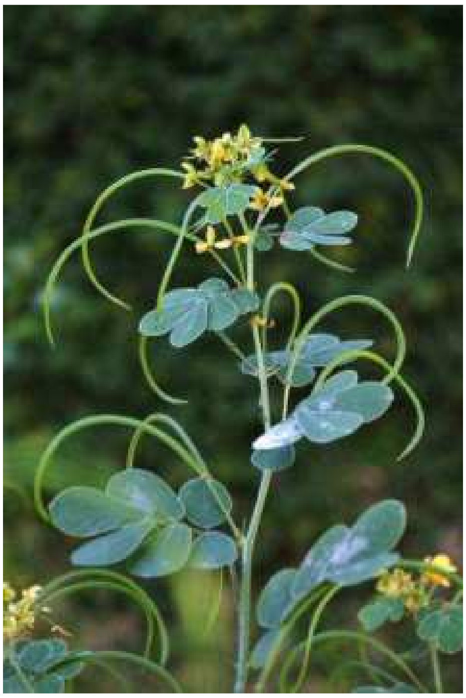

"*S. obtusifolia*" by Mauricio Mercadante (CC BY-NC-SA 2.0)

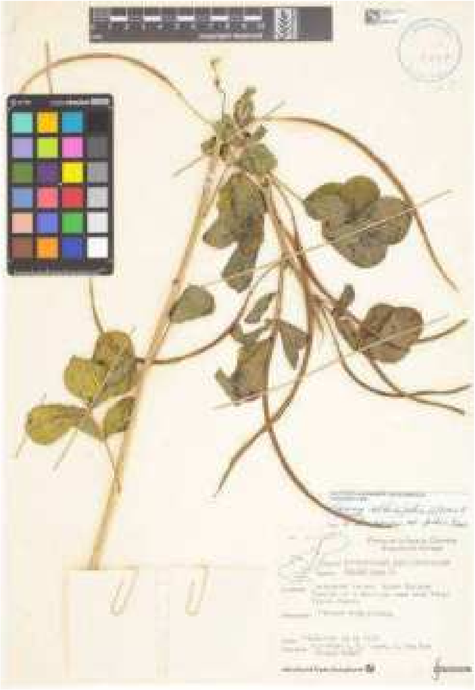

Specimen. "*S. obtusifolia*" by Herbario virtual FMB (CC BY-NC 2.0)

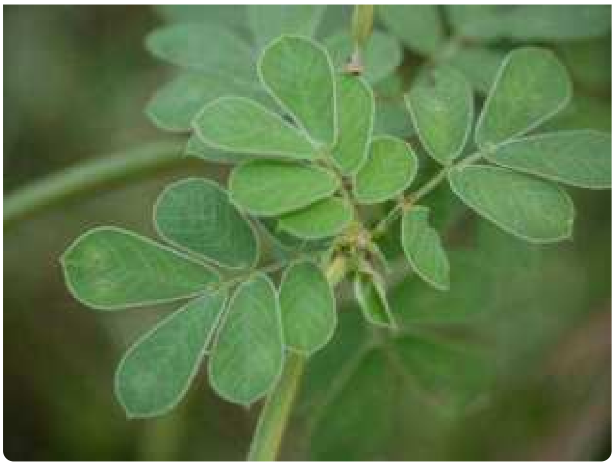

Leaves. "*S. obtusifolia*" by Dinesh Valke (CC BY-SA 2.0)

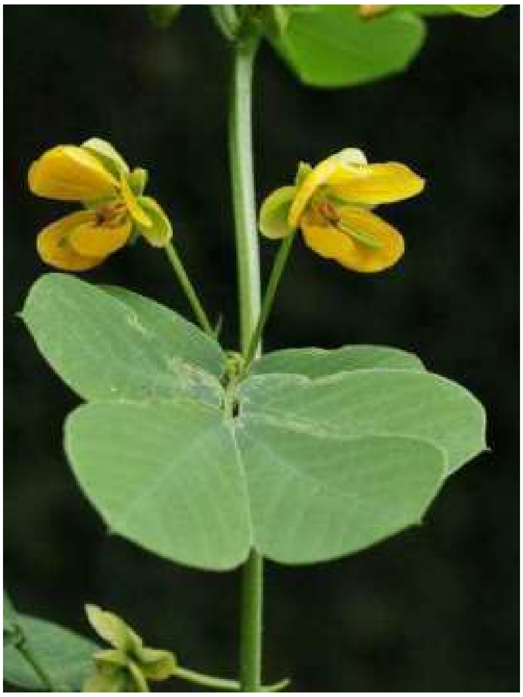

Flowers. "*S. obtusifolia*" by Mauricio Mercadante (CC BY-NC-SA 2.0)

Historiae Rerum Naturalium Brasiliae	Marcgrave, 1648	Page number	10b

Vernacular name(s) Iuapecanga. Sarcaparrilla

Species Smilax cf. schomburgkiana Kunth

Family Smilacaceae

Notes

There is a certain resemblance between the specimen and the woodcut (both are fruiting branches). The specimen could have been a model to design the woodcut, but it is hard to tell because the specimen is not that well preserved.

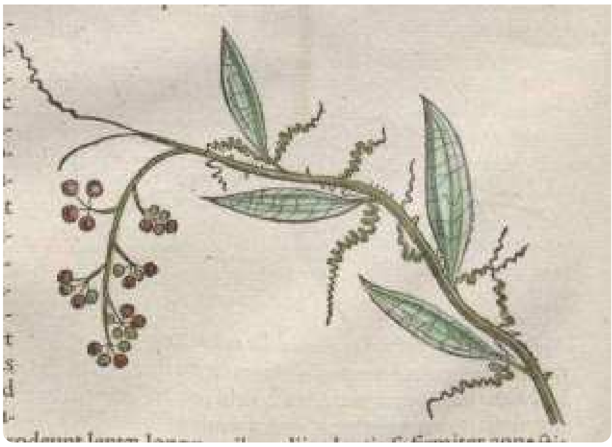

Historiae Plantarum – Herbis: 10b

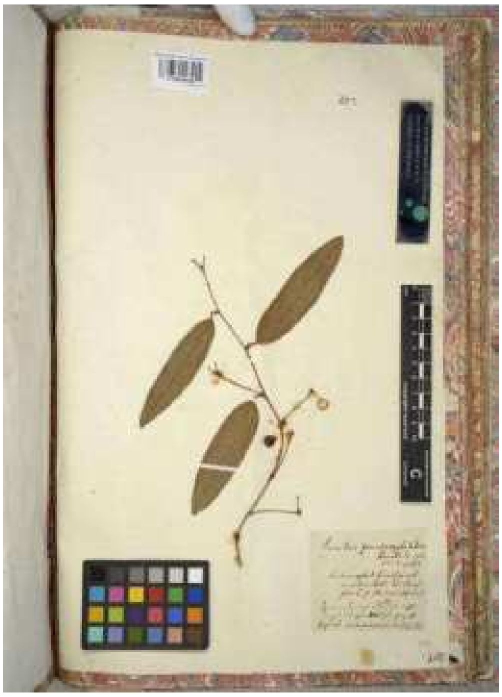

Marcgrave's herbarium: 166

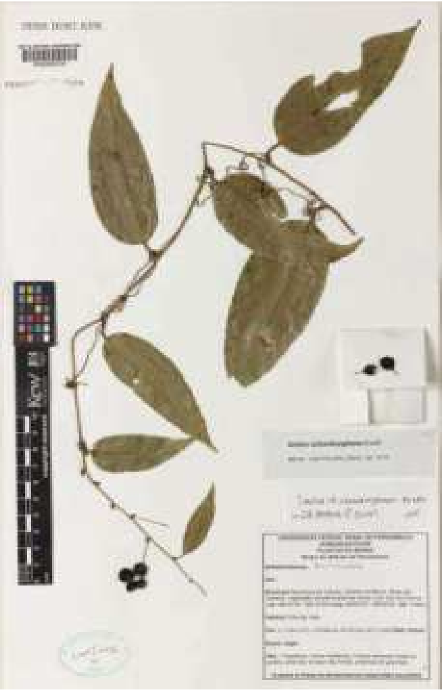

S. schomburgkiana specimen from Kew's Herbarium - K000828335. Retrieved from Plants of the World Online

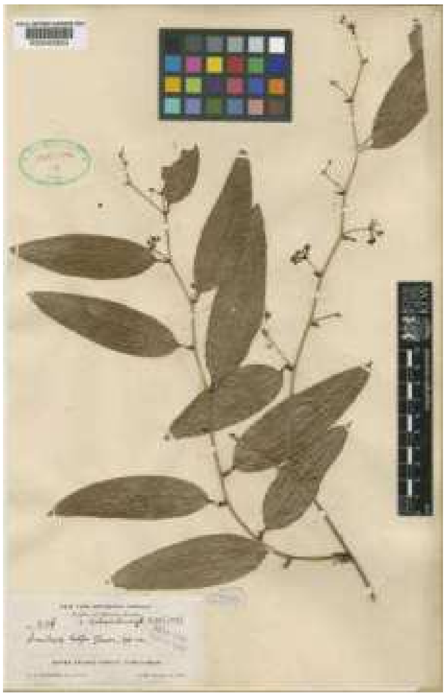

S. schomburgkiana specimen from Kew's Herbarium - K000400603. Retrieved from Plants of the World Online

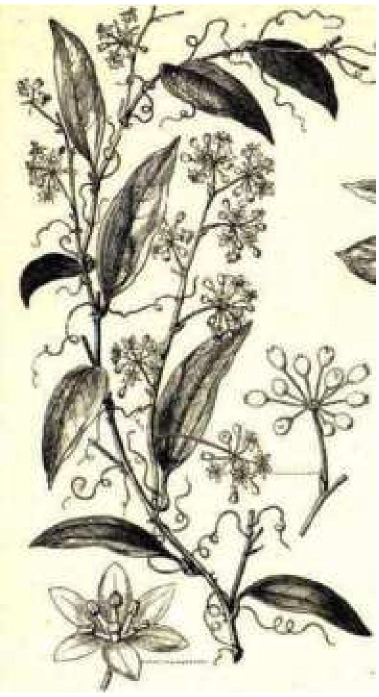

Drawing of *S. schomburgkiana* in *Meyers Konversations-Lexikon* by Meyer J.(1885: Vol. I, p. 894)

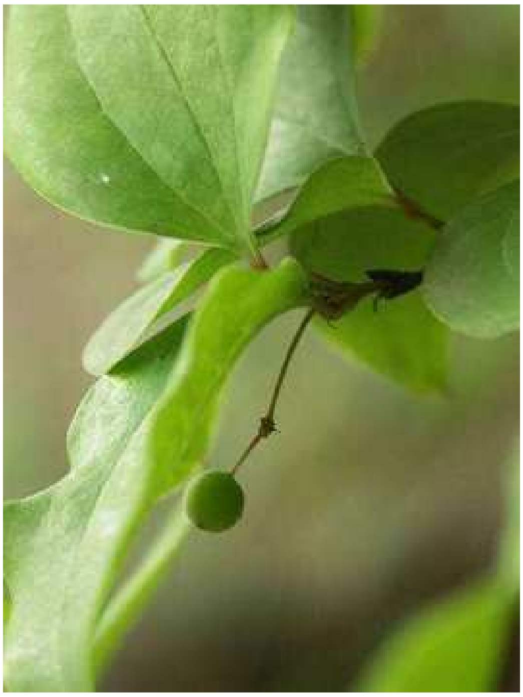

Hanging fruit in a related species, *S. china* by Cory (CC BY-SA 3.0)

Historiae Rerum Naturalium Brasiliae Marcgrave, 1648 Page number 12

Vernacular name(s) Camaru

Species Physalis cf. pubescens L.

Family Solanaceae

Notes

The woodcut looks different than the *Theatrum* image. There is no resemblance to the specimen in the herbarium. Only the fruit from *P. pubescens* is mounted in the sheet, which is attached to a branch of *Rivina humilis* (Andrade-Lima et al. 1977). The woodcut does not resemble the watercolor from the *Libri Principis*, which depicts a flowering branch with the fruit isolated in the bottom right. The woodcut shows a sterile branch with the flower and encapsulated (immature) fruit placed at different angles below it.

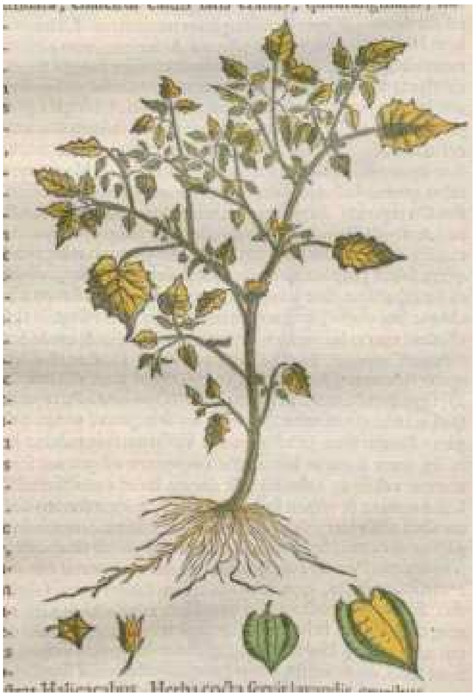

Historiae Plantarum – Herbis: 12

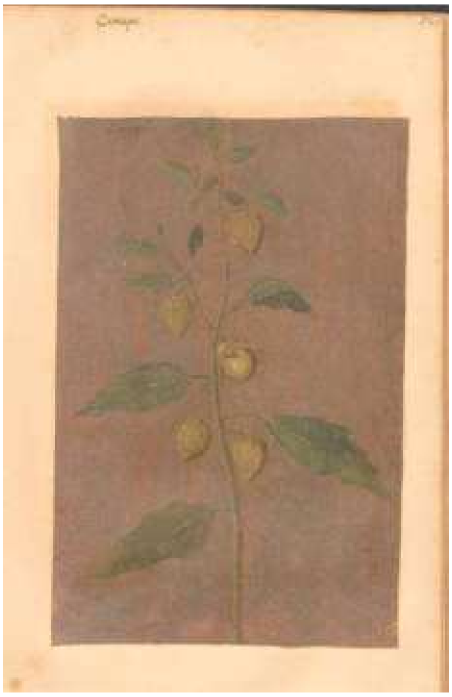

Theatrum Rerum Naturalium: 329

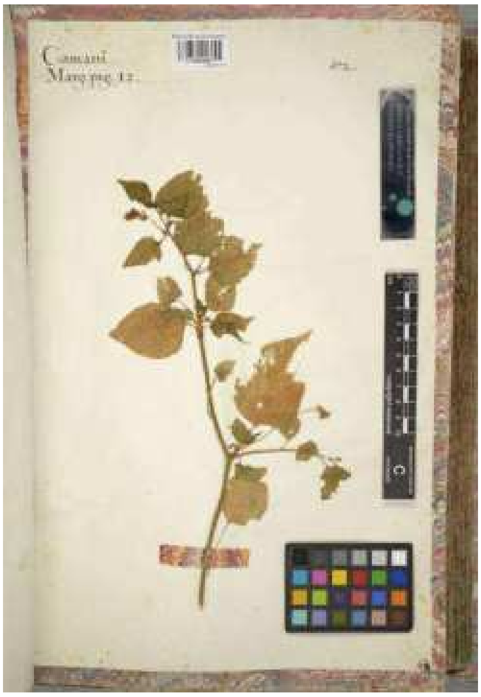

Marcgrave's herbarium: 61

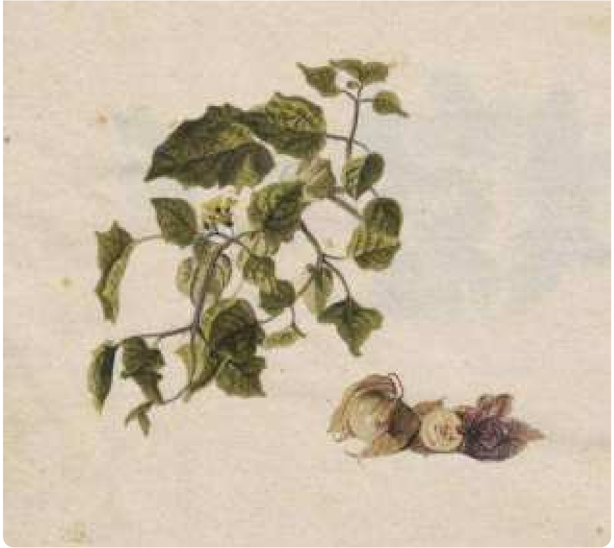

Libri Principis f. 8

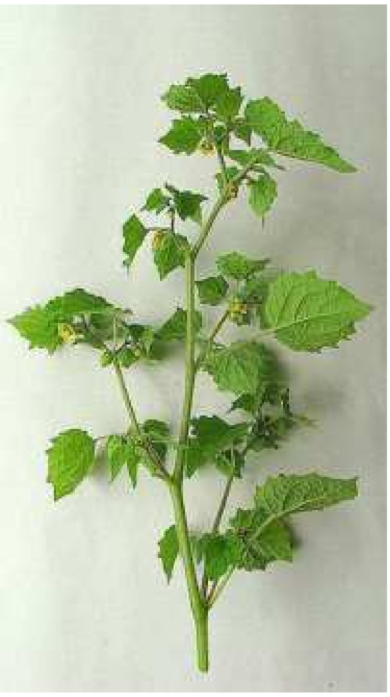

Flowering branch. "*P. pubescens* L." by Alex Popovkin, Bahia, Brazil (CC BY-NC-SA 2.0)

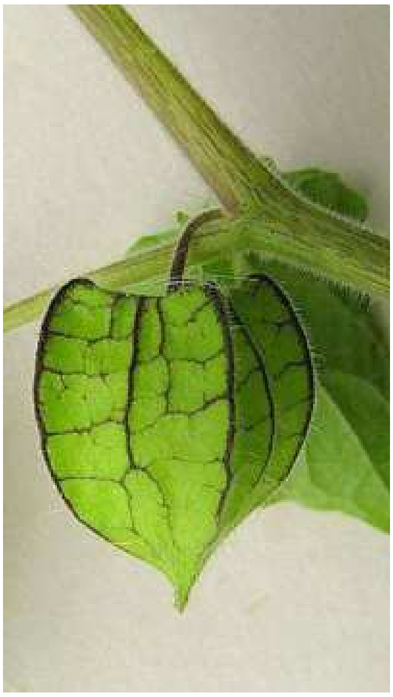

Fruit. "*P. pubescens* L." by Alex Popovkin, Bahia, Brazil (CC BY-NC-SA 2.0)

Historiae Rerum Naturalium Brasiliae	Marcgrave, 1648	Page number	13a

Vernacular name(s) Cararu. Bredos

Species Amaranthus viridis L.

Family Amaranthaceae

Notes

There is a certain resemblance between the specimen and the woodcut (both are flowering herbs with their roots). The plant could have been a model to design the woodcut before ending up as an exsicata.

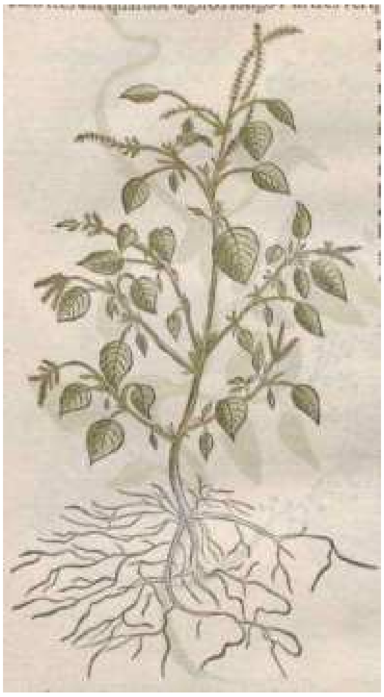

Historiae Plantarum – Herbis: 13a

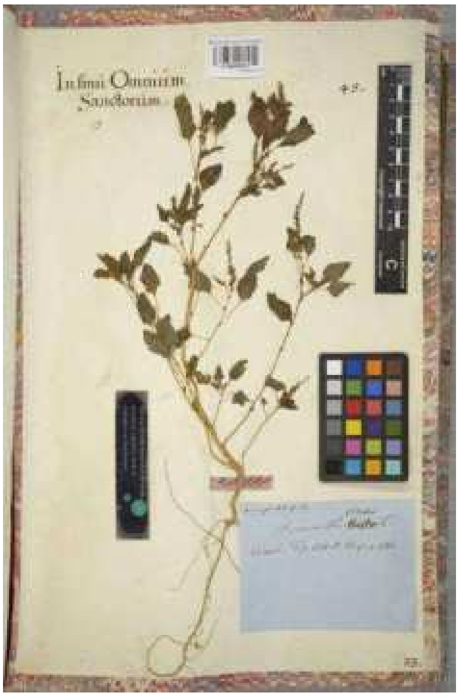

Marcgrave's herbarium: 33

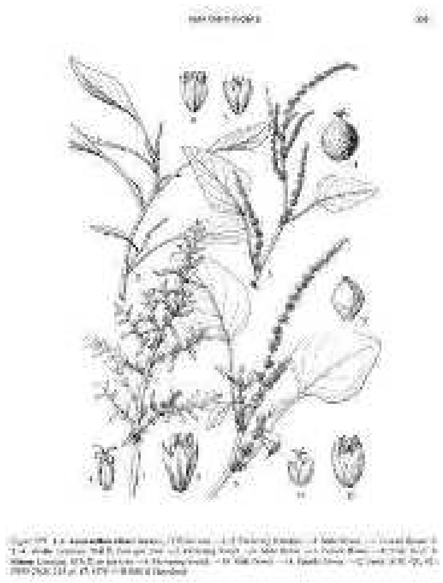

"*A. viridis*, line drawing, MBG-FOC" by filibot.web (CC BY-SA 2.0)

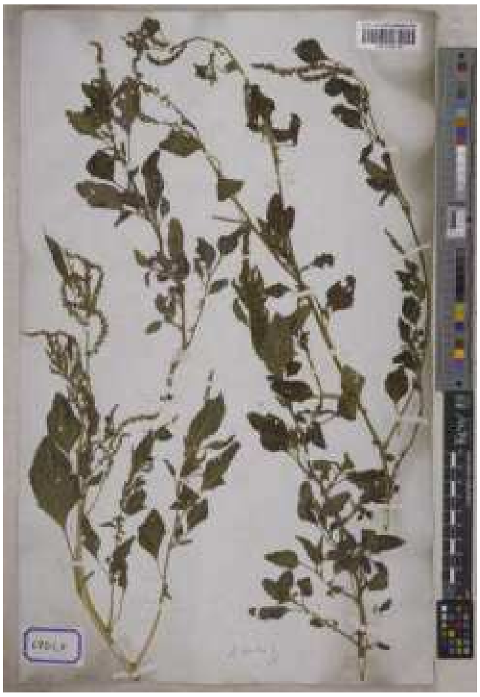

A. viridis specimen from Kew's Herbarium - K001126118. Retrieved from Plants of the World Online

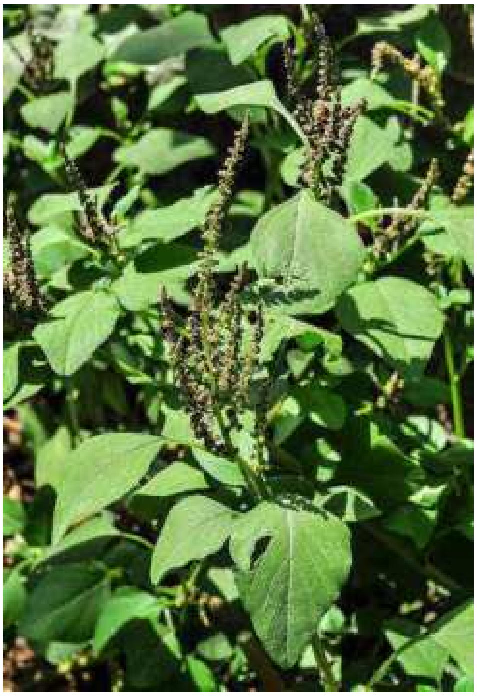

A. viridis flower. Burdwan, West Bengal, India, by Joydeep (CC BY-SA 3.0)

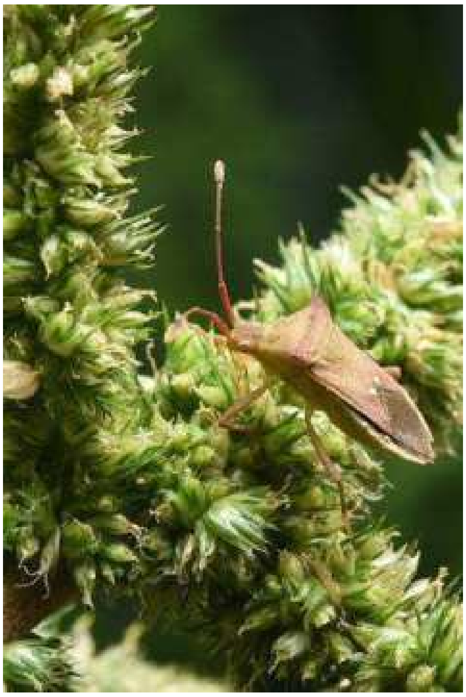

Cletus trigonus in inflorescence of *Amaranthus viridis*, by plj.johnny (CC BY-NC-SA 2.0)

Historiae Rerum Naturalium Brasiliae	Marcgrave, 1648	Page number	13b

Vernacular name(s)	Sempervivum marinum bacciferum
Species	Scaevola plumieri (L.) Vahl
Family	Goodeniaceae
Notes	

We did not find any correspondence between this woodcut and the contemporary or older sources.

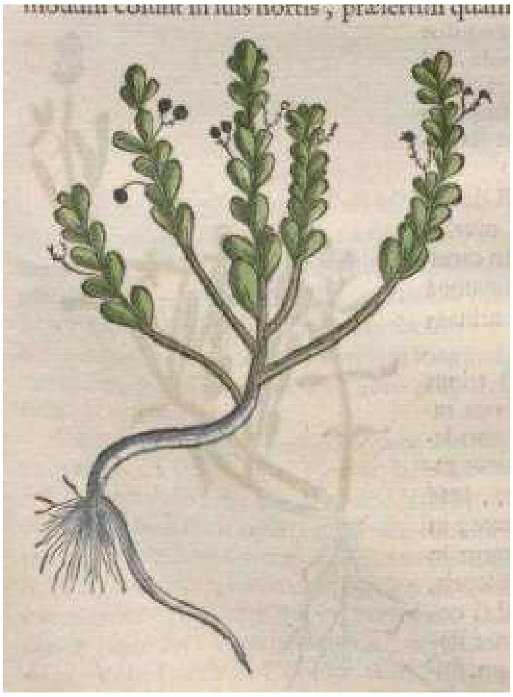

Historiae Plantarum – Herbis: 13b

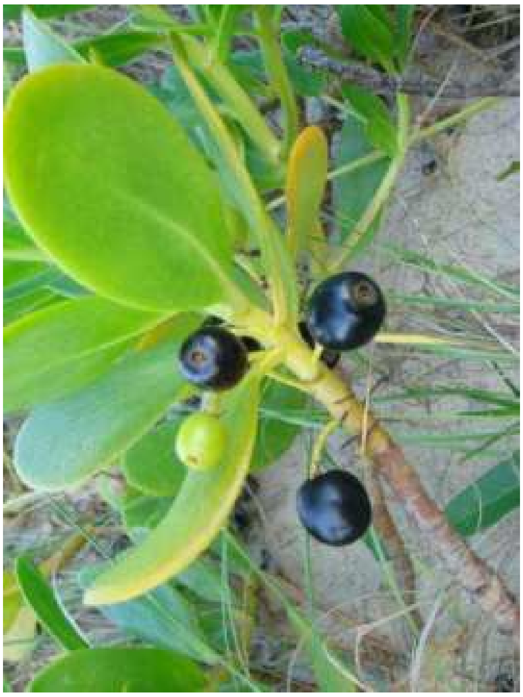

Fruiting branch. "*Scaevola plumieri*" by Marcia Stefani (CC BY 2.0)

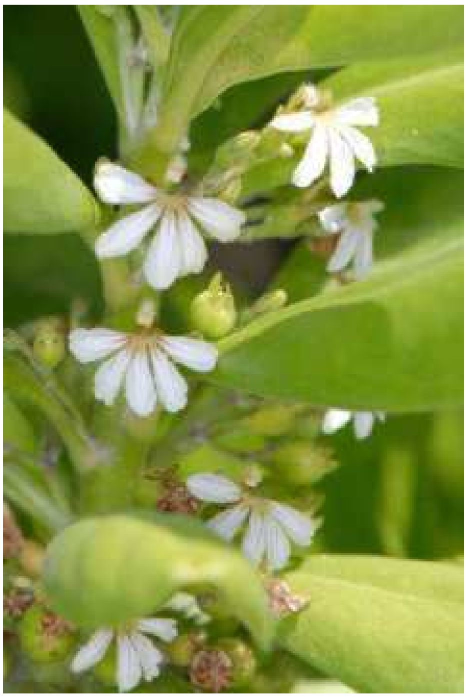

Flowering branch. "*S. plumieri* - gullfeed" by Sam Fraser-Smith (CC BY 2.0)

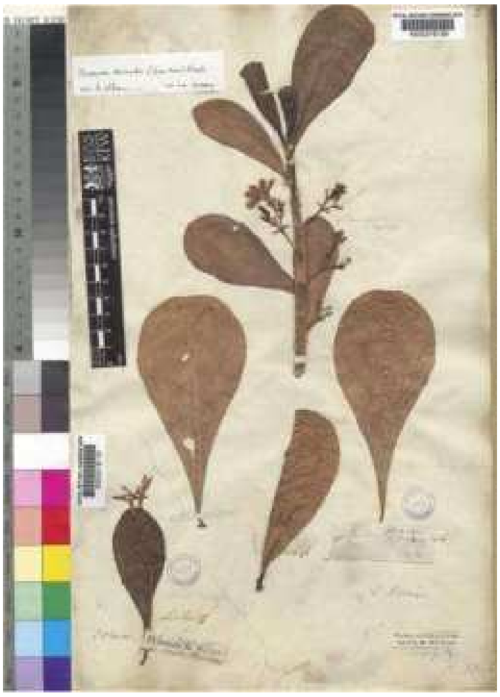

Specimen of S. plumieri (Goodeniaceae) - Board of Trustees of the Royal Botanic Gardens, Kew. Retrieved from Plants of the World Online

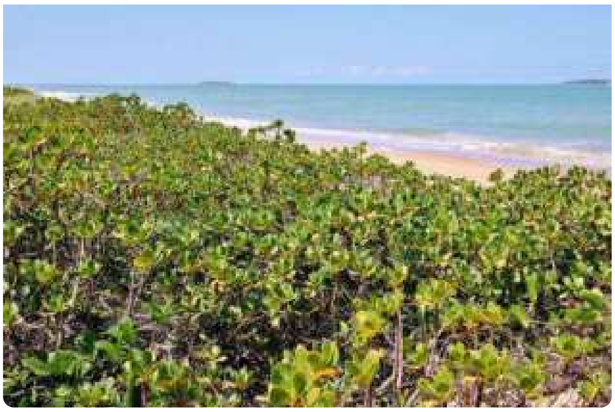

Restinga, Mata Atlântica, ES, Brazil. "*S. plumieri*" by Mauricio Mercadante (CC BY-NC-SA 2.0)

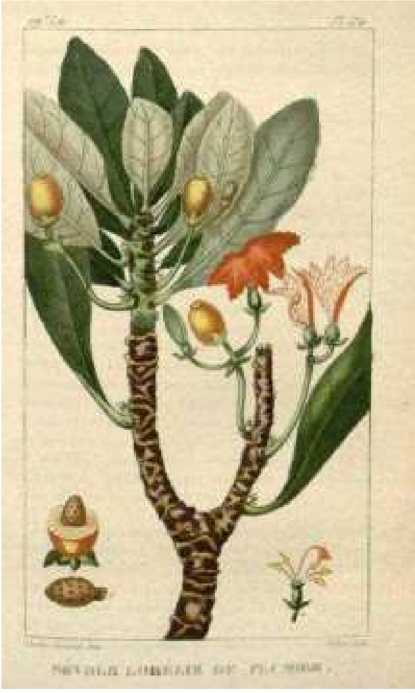

Flore [pittoresque et] médicale des Antilles by Descourtilz, M.E. (1829: Vol. VII, t. 474) Missouri Botanical Garden, U.S.A.

Historiae Rerum Naturalium Brasiliae Marcgrave, 1648 Page number 14a

Vernacular name(s) Icipo

Species Tetracera cf. breyniana Schltdl.

Family Dilleniaceae

Notes

We did not find any correspondence between this woodcut and the contemporary or older sources. Interestingly, there is a copper plate engraving in J. P. Breyne (1739: 20) depicting this species, and which specimen was found by his dad (Jakob Breyne) in Marcgrave's herbarium and hence, collected in Brazil (Andrade-Lima et al. 1977: 139). This, and other, specimens collected by Marcgrave that were used by Jakob's dad and son are today kept in the Sherard's herbarium in Oxford. The image in Breyne's (1739) bears a strong resemblance to the specimen in Oxford, but the woodcut in the HNB does not look alike, and it could also correspond to a relative species.

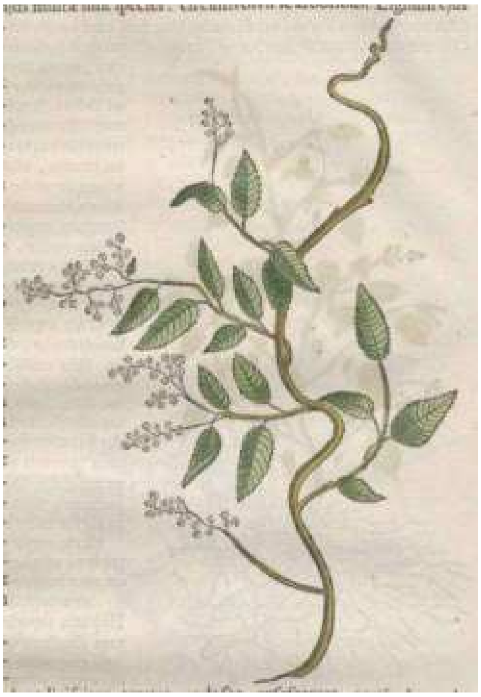

Historiae Plantarum – Herbis: 14a

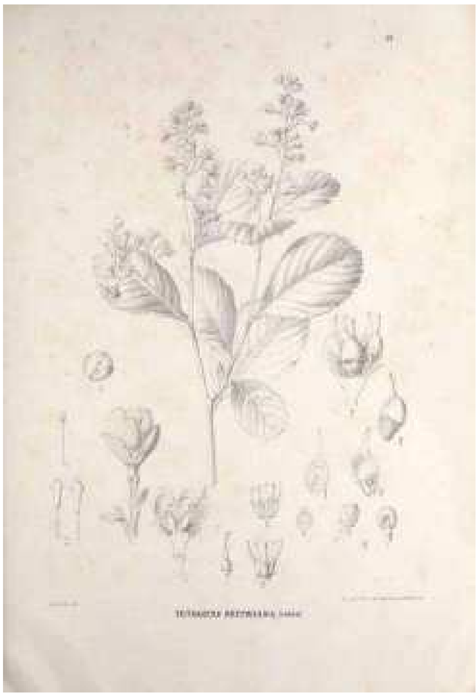

Botanischen Ergebnisse [Brasilien] by Wawra von Fernsee, H. (1866: t. 56)

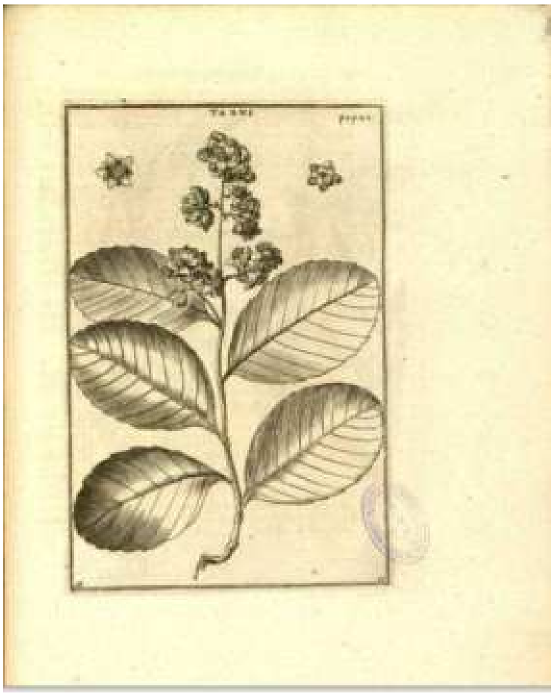

Engraving *T. breyniana* in *Prodromi fasciculi rariorum plantarum primus et secundus* by Breyne J.P. (1739: 20)

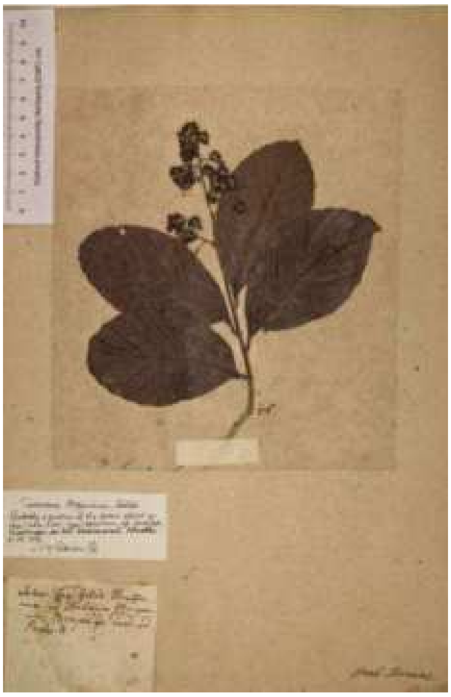

T. breyniana in Sherard's herbarium (https://herbaria.plants.ox.ac.uk/bol/sherard/record/details/e027eca3-3dcc-42e8-9975-d8679d2f7c75)

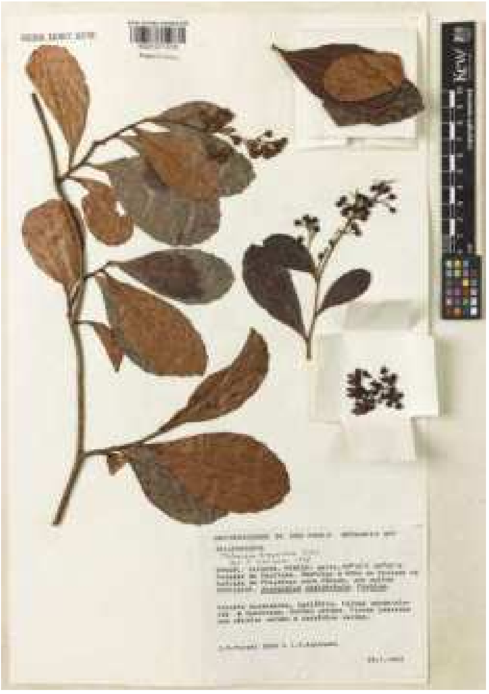

T. breyniana specimen from Kew's Herbarium - K001071318. Retrieved from Plants of the World Online

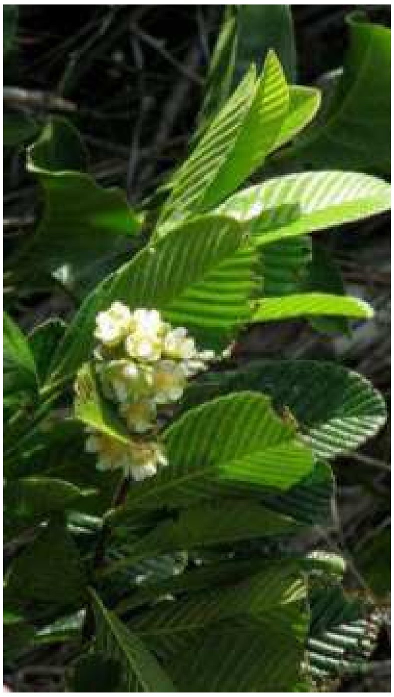

A related species of *T. breyniana* "*Tetracera boomii* G. Aymard C." by Alex Popovkin, Bahia, Brazil (CC BY-NC-SA 2.0)

Historiae Rerum Naturalium Brasiliae	Marcgrave, 1648	Page number	14b

Vernacular name(s) Perexxil. Caaponga

Species Blutaparon vermiculare (L.) Mears

Family Amaranthaceae

Notes

There is a certain resemblance between the specimen and the woodcut (both are flowering, albeit only the woodcut depicts the roots). The plant could have been a model to design the woodcut before ending up as an exsicata or the specimen could have been a model to design the woodcut.

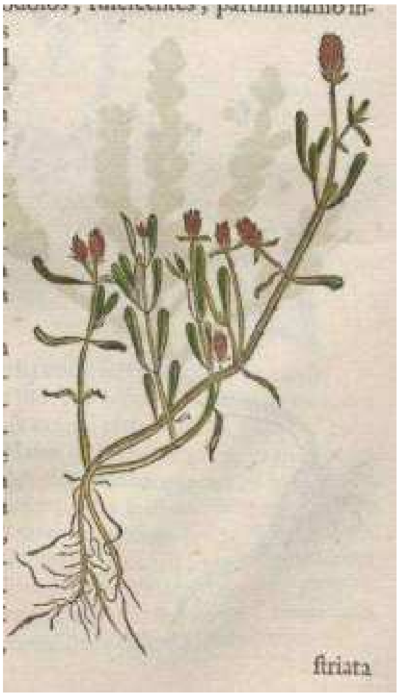

Historiae Plantarum – Herbis: 14b

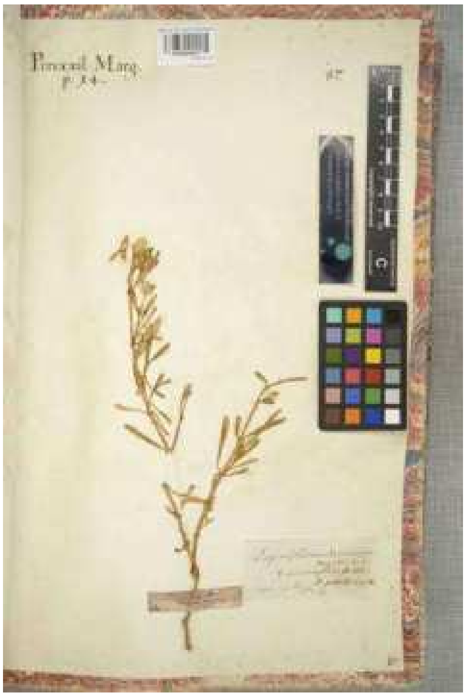

Marcgrave's herbarium: 17

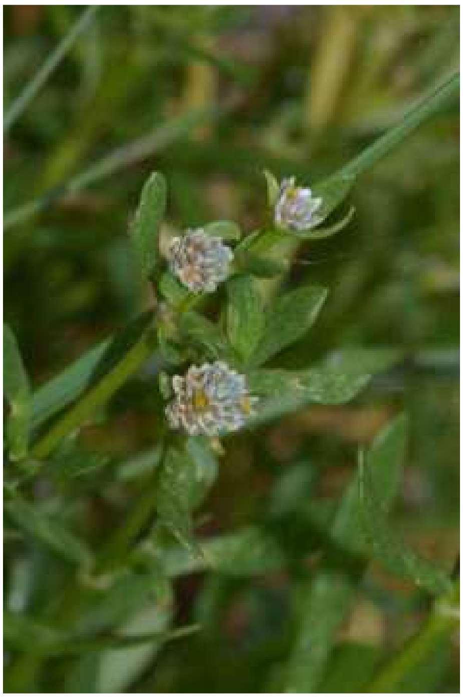

"*B. vermiculare* 6aIR" by jimduggan24 (CC BY 2.0)

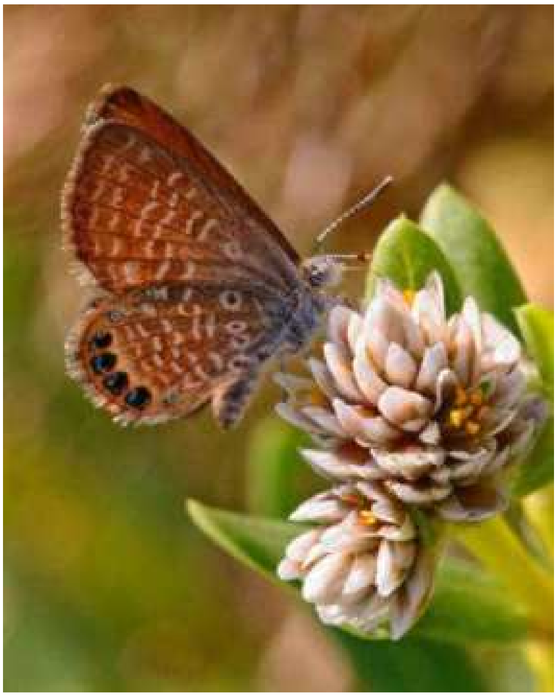

"Amaranthaceae, Eastern Pygmy-Blue (*Brephidium isophthalma*) on Silverhead (*B. vermiculare*)" by Mary Keim (CC BY-NC-SA 2.0)

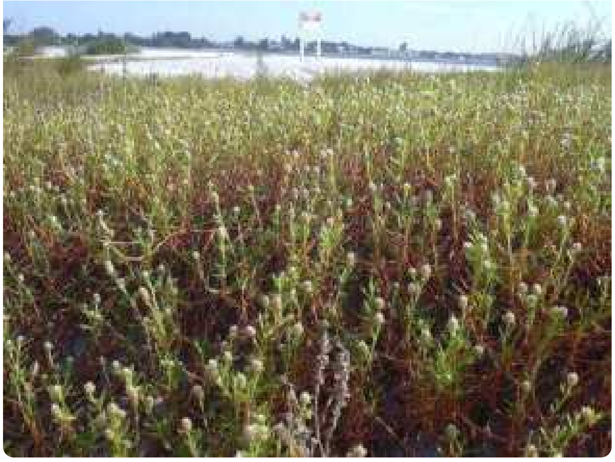

Blutaparon vermiculare (syn. Philoxerus vermicularis). Location: Florida, South Lido Beach by BotMultichillT (CC-BY-3.0)

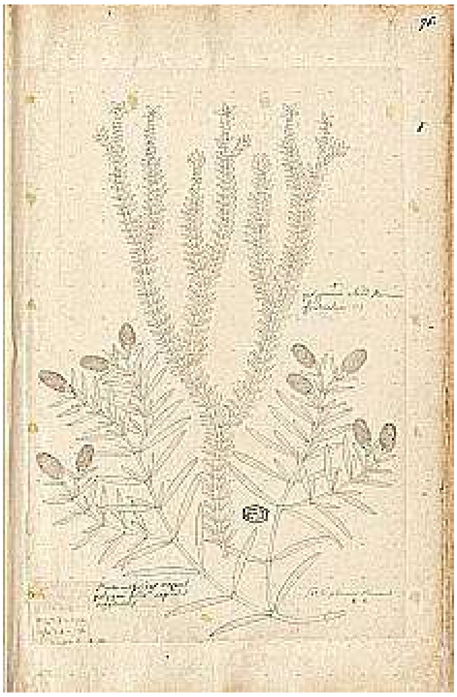

Botanicon Americanum seu historia plantarum Americanis insulis nascentium by Plumier, C. (1689 -1697) Vol. IV, t. 96 f. 2

Historiae Rerum Naturalium Brasiliae | Marcgrave, 1648 | Page number 15

Vernacular name(s): Ambuyaembo

Species: Aristolochia labiata Willd.

Family: Aristolochiaceae

Notes

The woodcut image is very similar to the *Theatrum* image (reversed and upside-down).

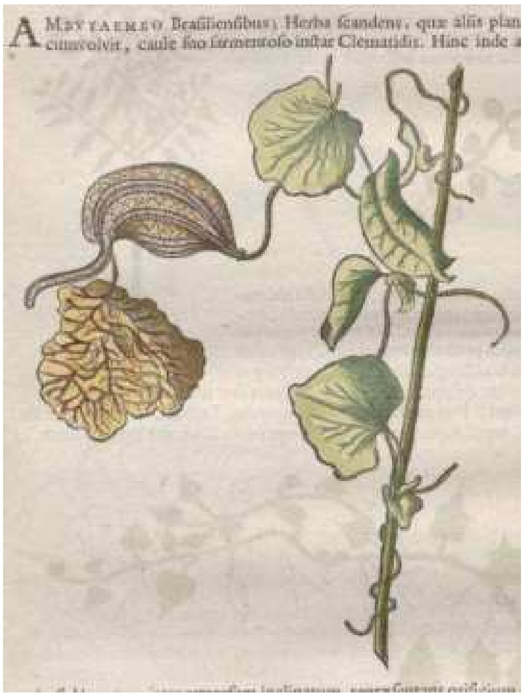

Historiae Plantarum – Herbis: 15

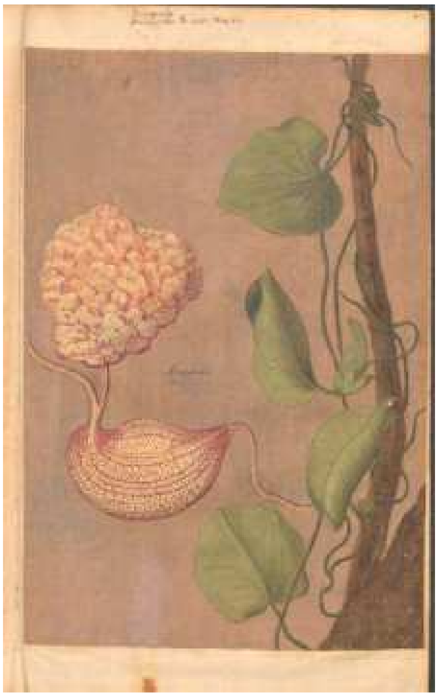

Theatrum Rerum Naturalium: 455

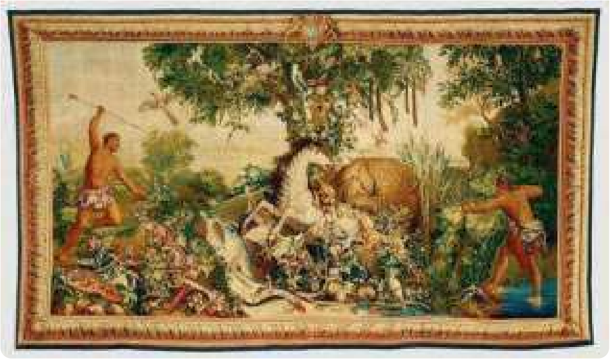

The Striped Horse, From the tapestry series *Les Anciennes Indes* (Old Indies), French, Paris. Ca. 1692 - 1730

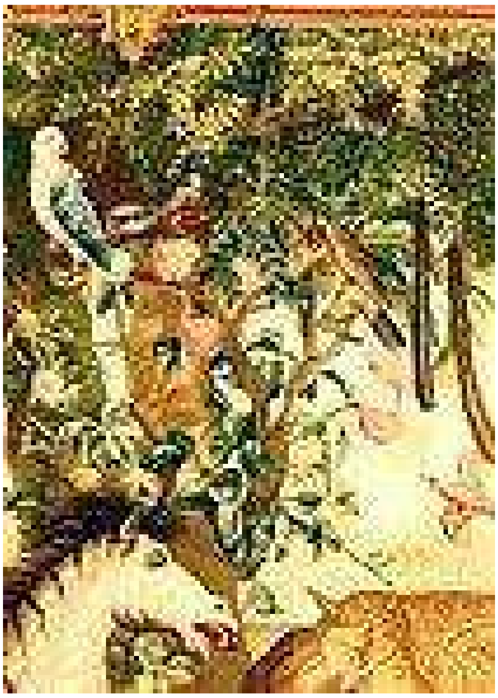

Close -up of *A. labiata* hanging from the *Cassia grandis* tree, from the tapestry series *Les Anciennes Indes*

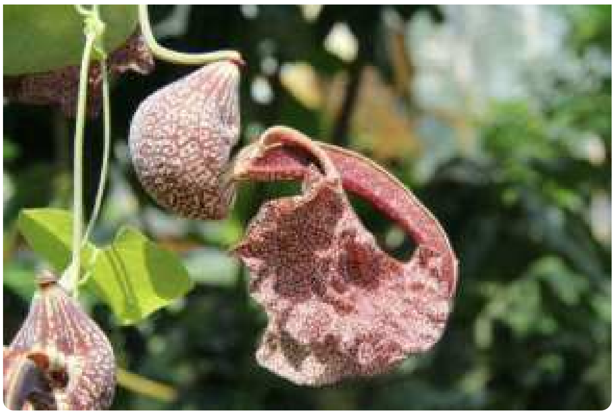

"Blüte der *A. labiata* - Palmengarten Frankfurt.jpg" by Maulaff (CC BY-SA 3.0)

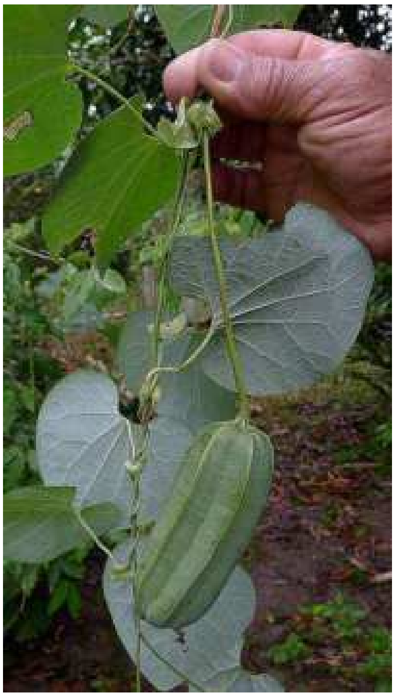

Fruit. "*A. labiata*" by Alex Popovkin, Bahia, Brazil (CC BY-NC-SA 2.0)

Historiae Rerum Naturalium Brasiliae	Marcgrave, 1648	Page number	16a

Vernacular name(s)	Planta
Species	Lippia sp.?
Family	Verbenaceae
Notes	

We did not find any correspondence between this woodcut and the contemporary or older sources.

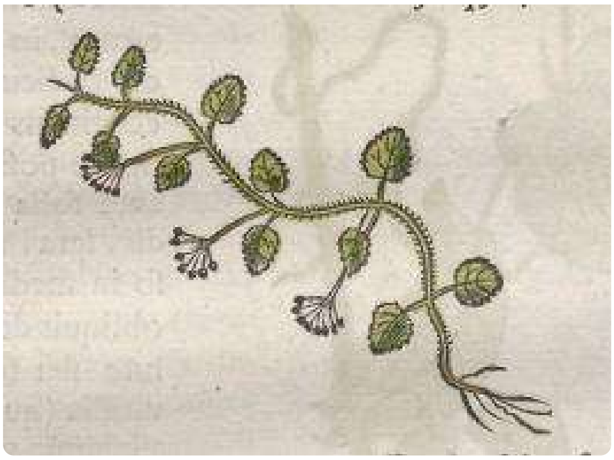

Historiae Plantarum – Herbis: 16a

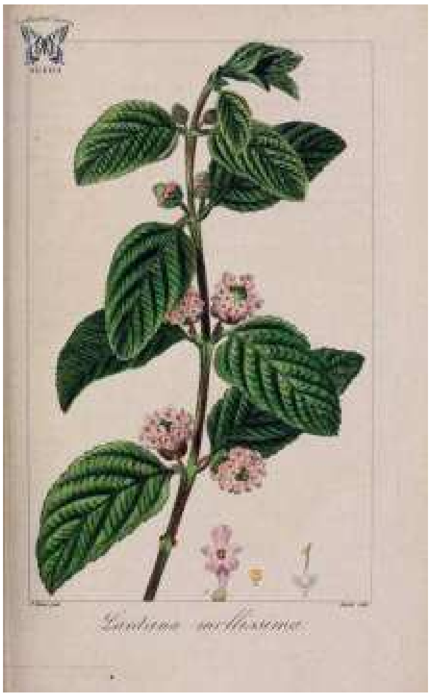

"*Lippia alba* [as Lantana mollissima] Herbier général de l'amateur, vol. 8 (1817-1827) [P. Bessa]" by Swallowtail Garden Seeds (CC BY 2.0)

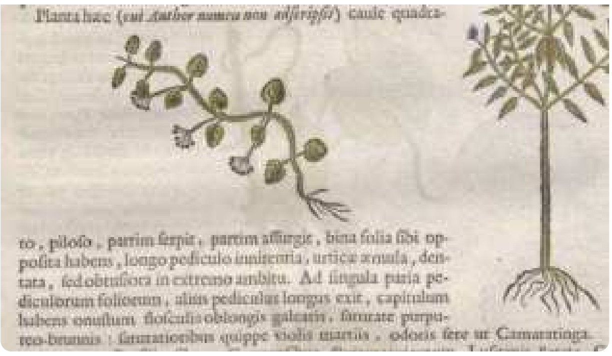

Planta hæc (*cui Author nomen non adſcripſit*) caule quadra-to, piloſo, partim ſerpit, partim aſſurgit, bina folia ſibi op-poſita habens, longo pediculo innitentia, urticæ æmula, den-tata, ſed obtuſiora in extremo ambitu. Ad ſingula paria pe-diculorum foliorum, alius pediculus longus exit, capitulum habens onuſtum floſculis oblongis galeatis, ſaturate purpu-reo-brunnis: ſaturationibus quippe violis martiis, odoris fere ut Camaratinga.

HNB (Marcgrave 1648: 16) Latin edition

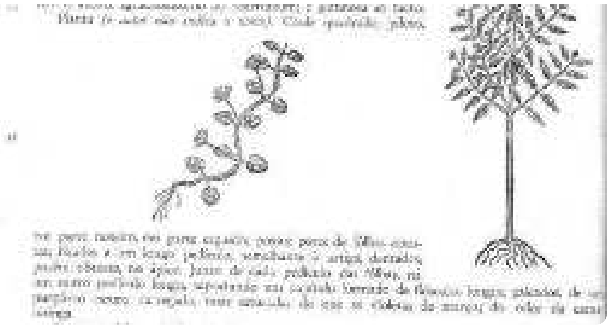

[illegible]

HNB (1942 [1648]) Portuguese edition

"Square, hairy stem, partly flat, partly erected; it has pairs of opposite leaves, attached to a long pedicle, similar to the nettle, notched but obtuse at the apex. Next to each pedicle of the leaves, another long pedicle emerges, supporting a chapter formed of long, curved florets, of a charged dark purple, more saturated than the violets of March; with the smell of Camaratinga [referring to *Lantana* sp.]"

English translation, by M. Alcantara-Rodriguez

Historiae Rerum Naturalium Brasiliae | Marcgrave, 1648 | Page number 16b

Vernacular name(s) Marrubium

Species Stemodia foliosa Benth.

Family Plantaginaceae

Notes

There is a certain resemblance between the specimen and the woodcut (both are flowering and show the main stem with lateral branches). However, it is uncertain whether they are related to each other because they do not have the branches at the same angle and the specimen lacks roots, although this could be due to damage over time. The pencil drawing in De Laet's manuscript is exact to the woodcut but reversed, and this was likely the basis to create the woodblock.

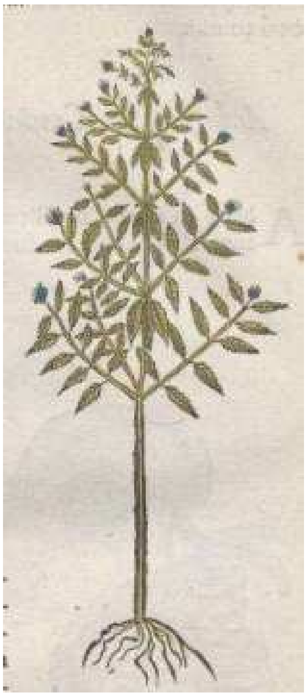

Historiae Plantarum – Herbis: 16b

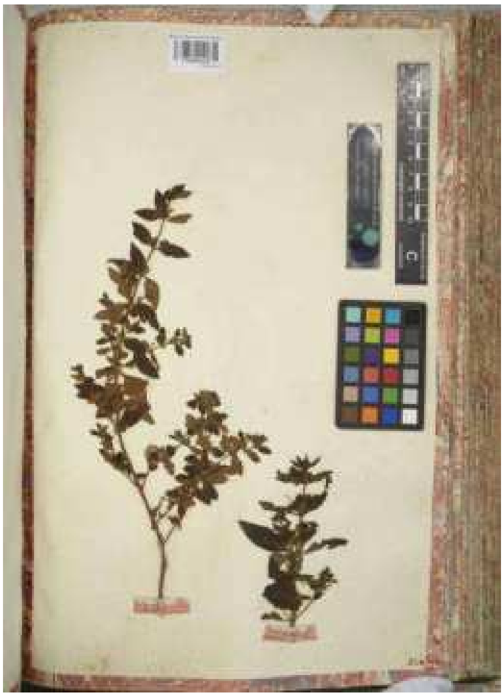

Marcgrave's herbarium: 124

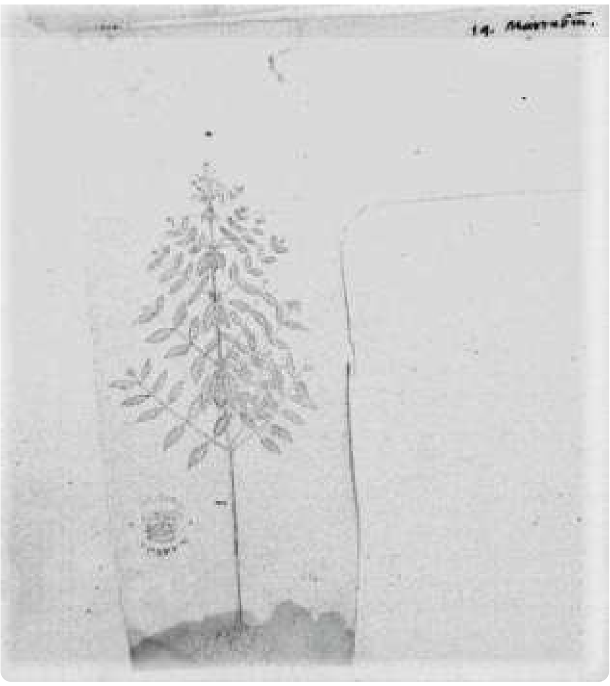

Pencil drawing in Sloane MS 1554, f. 14v

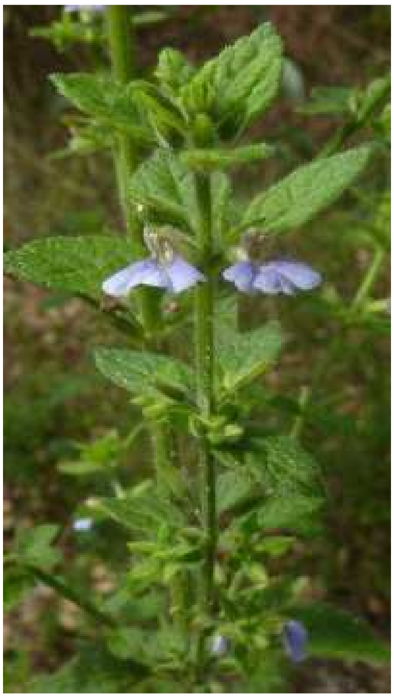

"*S. foliosa*" by Alex Popovkin, Bahia, Brazil (CC BY-NC-SA 2.0)

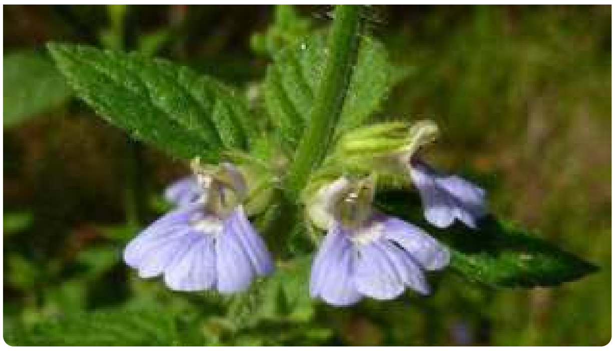

Flowers. "*S. foliosa*" by Alex Popovkin, Bahia, Brazil (CC BY-NC-SA 2.0)

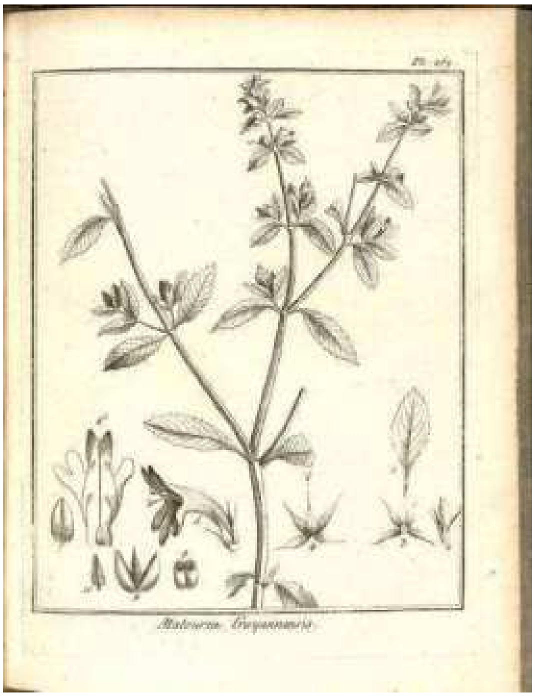

Histoire des plantes de la Guiane Françoise by Aublet, J.B.C.F. (1775: Vol. IV, t. 259) Missouri Botanical Garden, St. Louis, U.S.A.

Historiae Rerum Naturalium Brasiliae	Marcgrave, 1648	Page number	16c

Vernacular name(s)	Ietica. Quiquoaquianputu. Batata
Species	Ipomoea batatas (L.) Lam.
Family	Convolvulaceae
Notes	

The woodcut is different from the oil-painting, which shows only the tubers, and from the specimen, which consists of a flowering twig.

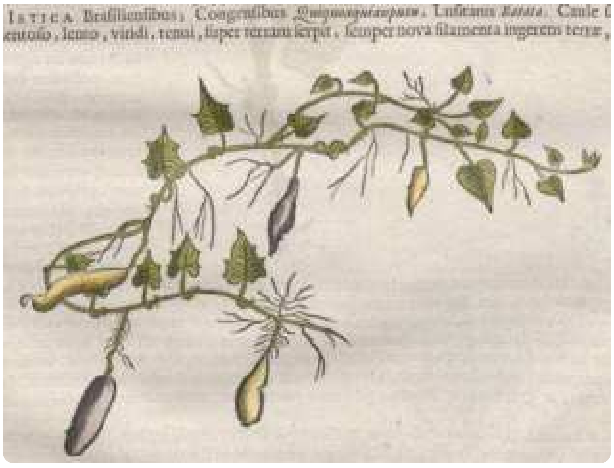

Historiae Plantarum – Herbis: 16c

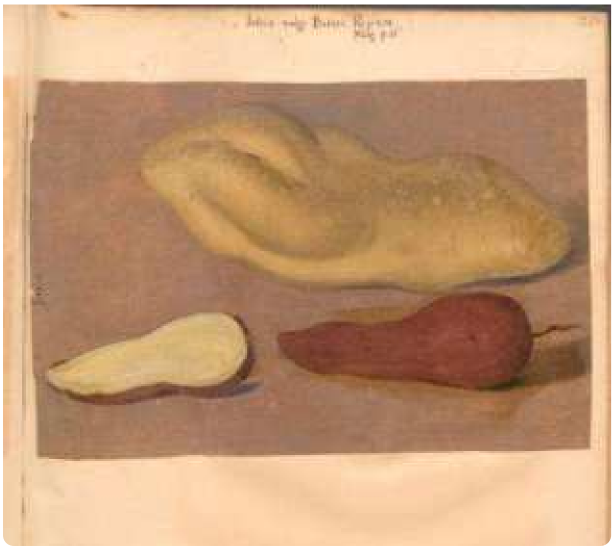

Theatrum Rerum Naturalium: 263

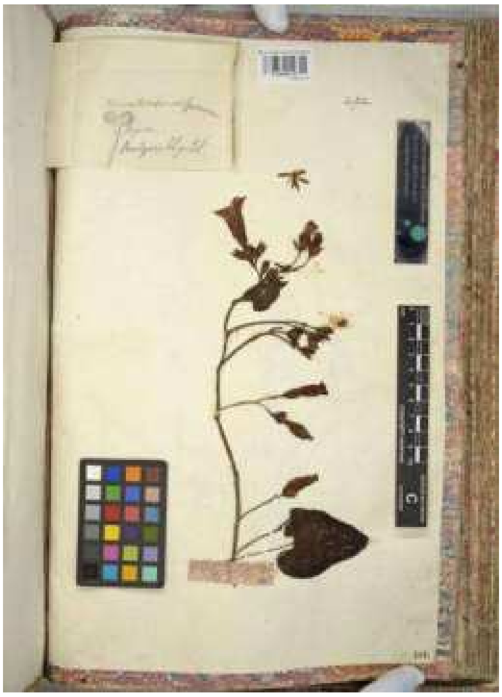

Marcgrave's herbarium: 151

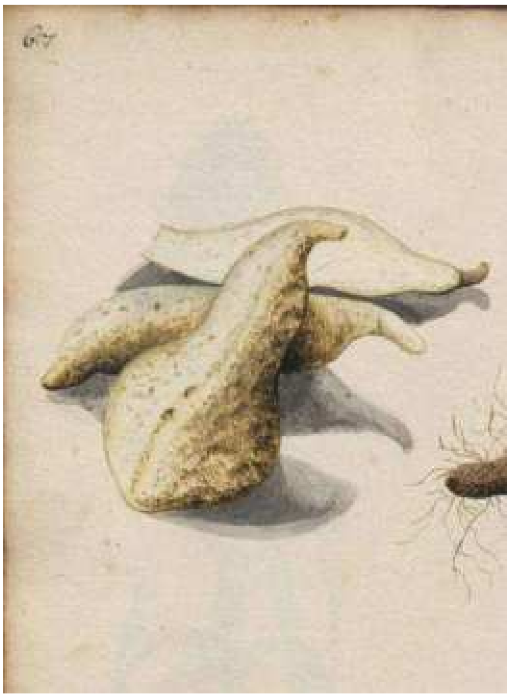

Libri Principis f. 87 [67]

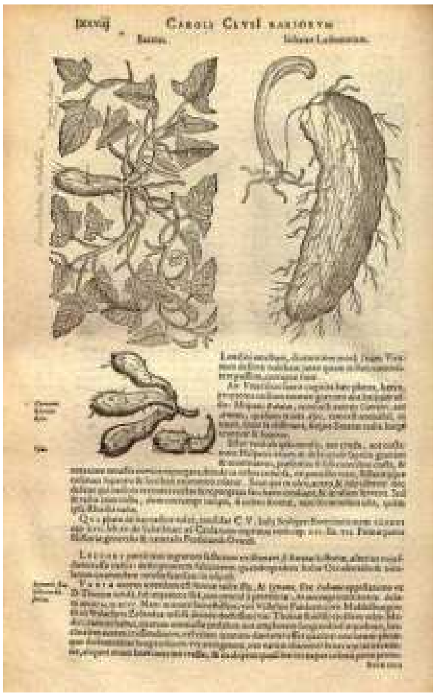

Batatas in *Rariorum plantarum historia* by Clusius (1601: 78). Missouri Botanical Garden, St. Louis, USA

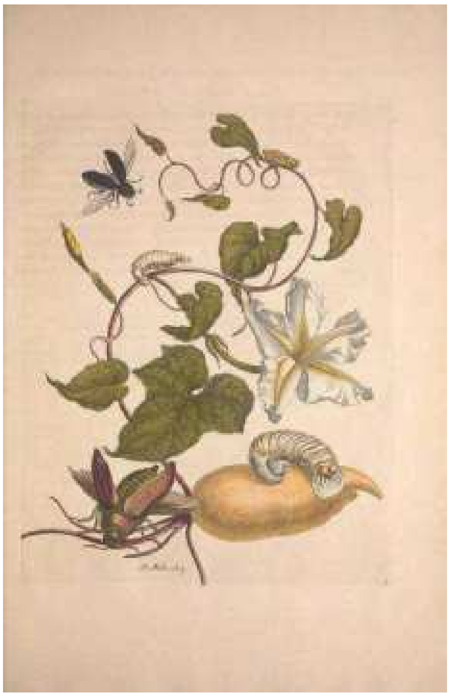

I. batatas in *Metamorphosibus insectorum Surinamensium, of te verandering der Surinaamsche insecten* by Merian, M.S. (1714: t. 50)

Historiae Rerum Naturalium Brasiliae | Marcgrave, 1648 | Page number 17

Vernacular name(s) Ipecacoanha

Species Carapichea ipecacuanha (Brot.) L.Andersson

Family Rubiaceae

Notes

The specimens bears certain resemblance with the apical branch of the woodcut: both have one flower at the apex and eight leaves similarly arranged.

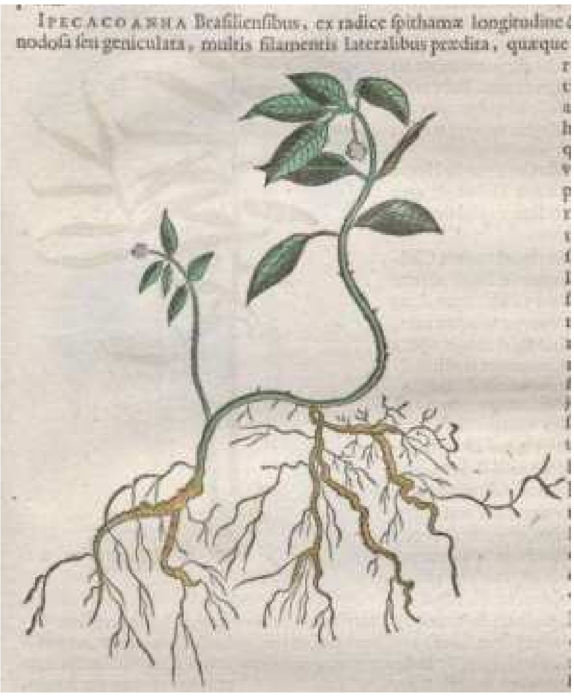

Historiae Plantarum – Herbis: 17

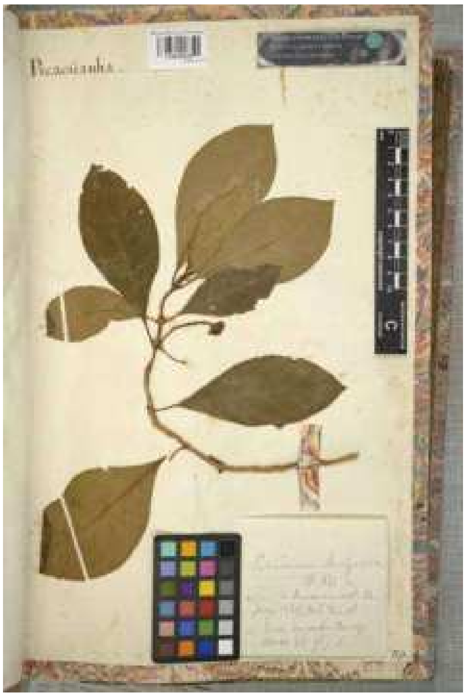

Marcgrave's herbarium: 39

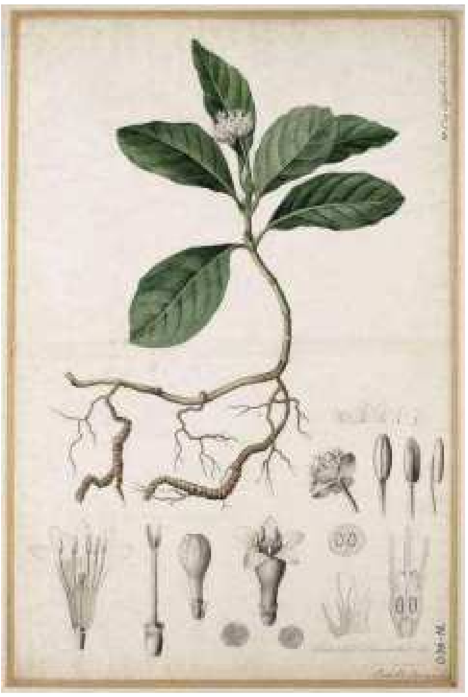

Botanische wandplaten
Stichting Academisch Erfgoed/geheugenvannederland.nl, the Netherlands

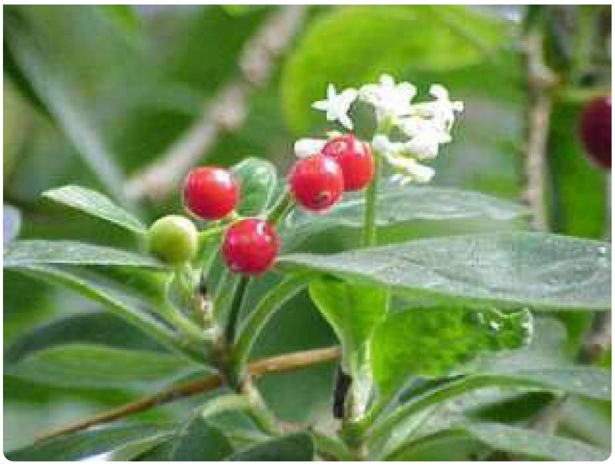

Cephaelis acuminata [syn. of *C. ipecacuanha*] ;Family: Rubiaceae by Kurt Stüber (CC-BY-SA-3.0)

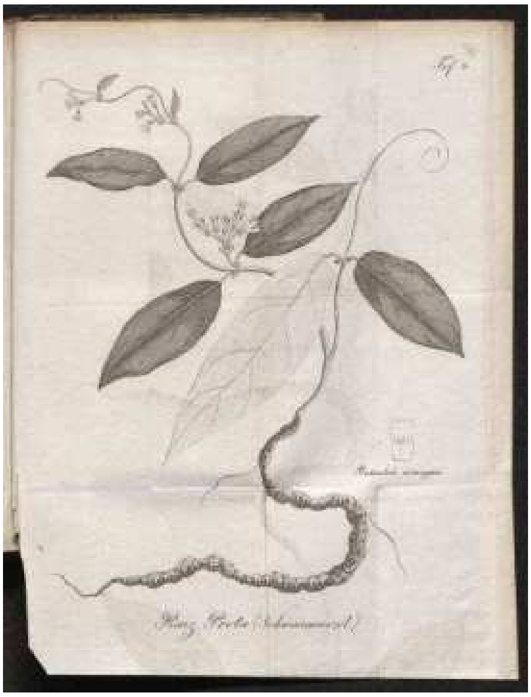

Journal von Brasilien, oder vermischte Nachrichten aus Brasilien by Eschwege, W. C. von (1818: Vol. I)

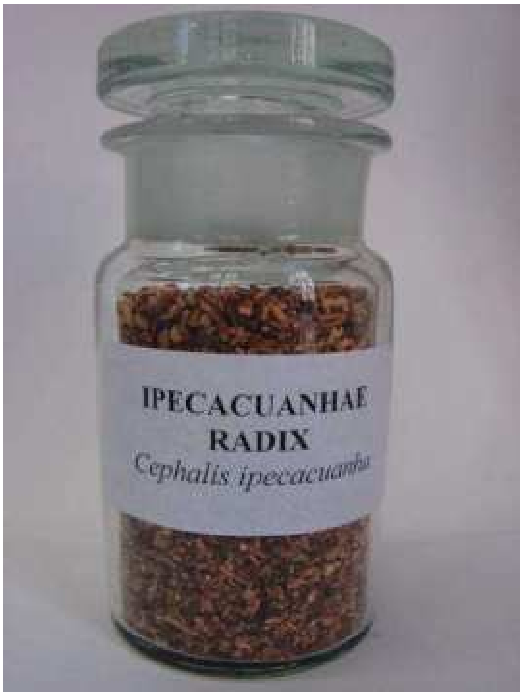

Ipecacuanhae radix, *Cephalis ipecacuanha* [syn. of C. ipecacuanha] by Maša Sinreih in Valentina Vivod (CC-BY-SA-3.0)

Historiae Rerum Naturalium Brasiliae | Marcgrave, 1648 | Page number 18

Vernacular name(s): Zinziber

Species: Zingiber officinale Roscoe

Family: Zingiberaceae

Notes

We did not find any correspondence between this woodcut and the contemporary or older sources.

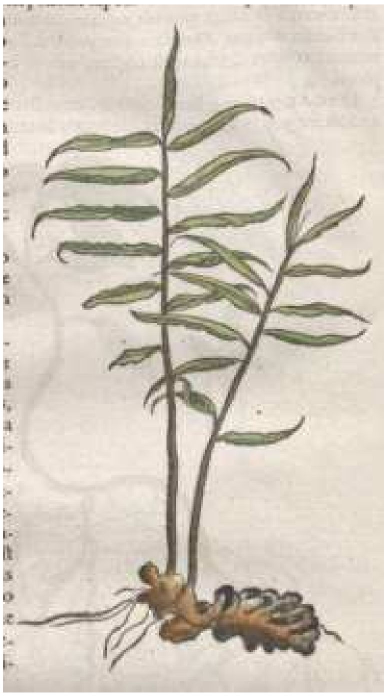

Historiae Plantarum – Herbis: 18

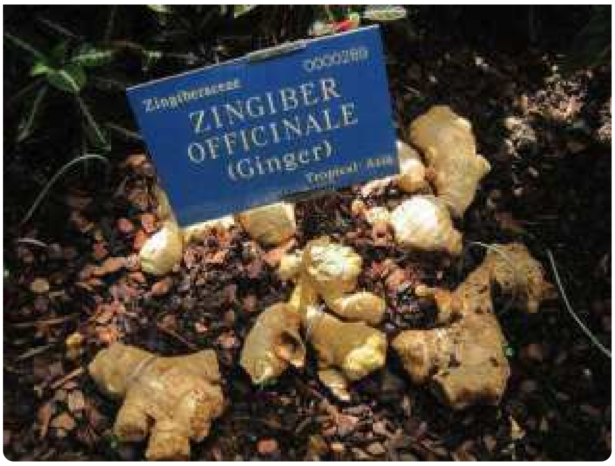

"*Zingiber Officinale* (Ginger)" by Snapshooter46 (CC BY-NC-SA 2.0)

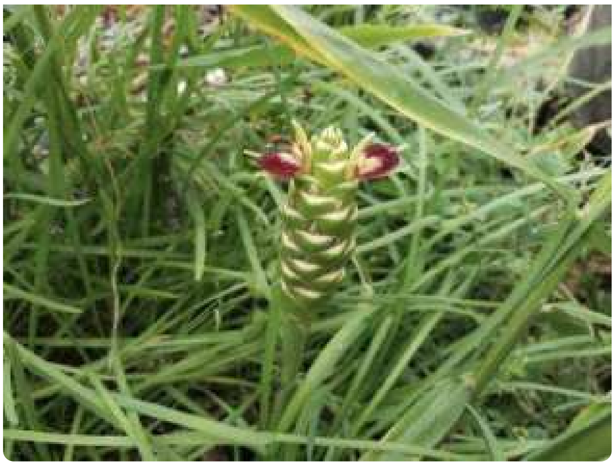

"*Zingiber officinale*" by D.Eickhoff (CC BY-NC-SA 2.0)

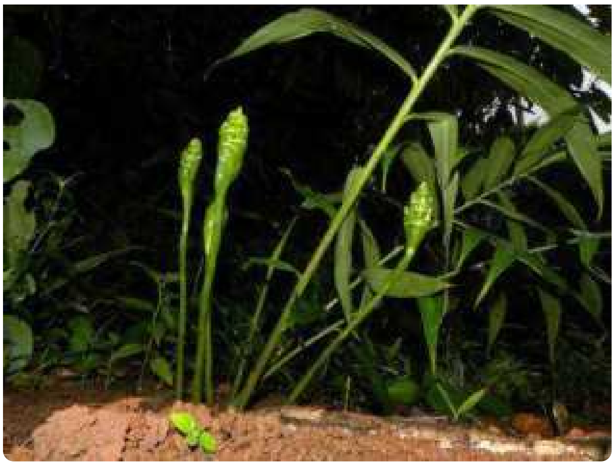

"*Zingiber officinale* Roscoe (cultivado)" by Reinaldo Aguilar (CC BY-NC-SA 2.0)

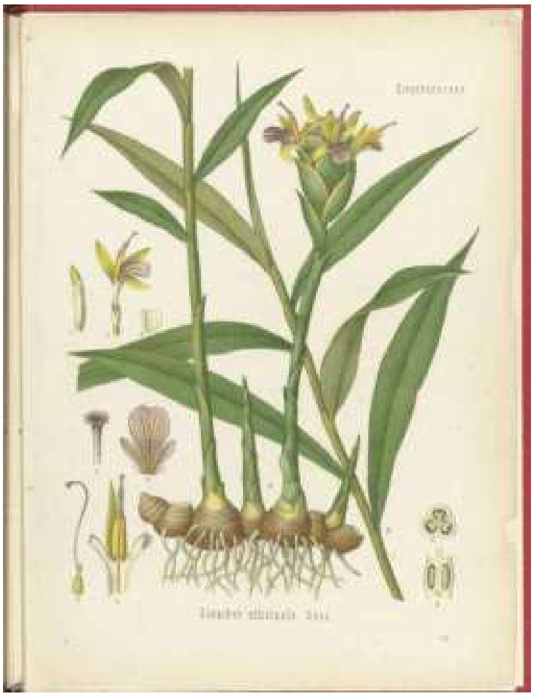

Z. officinale in *Köhler's Medizinal Pflanzen* (1883 -1914) by Köhler, F.E. (1890: Vol. II, t. 172)

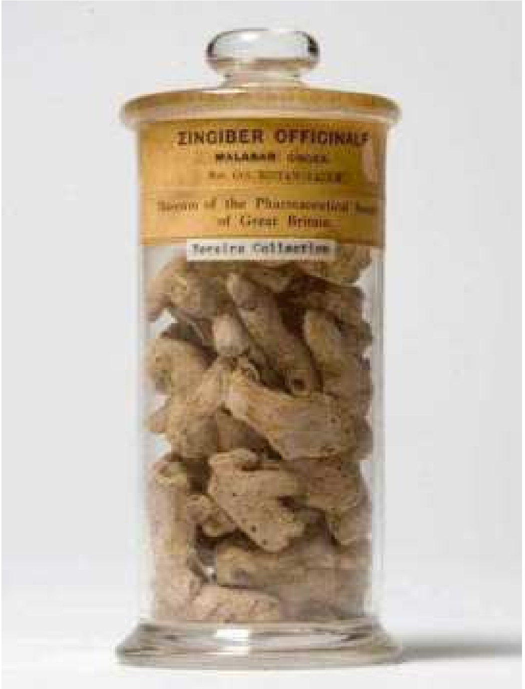

Z. officinale (ginger) jar of rhizomes - Dried specimen from Kew's Economic Botany Collection. Retrieved from Plants of the World Online

Historiae Rerum Naturalium Brasiliae | Marcgrave, 1648 | Page number 19a

Vernacular name(s) Mucuna

Species Mucuna urens (L.) Medik.

Family Fabaceae

Notes

The woodcut is very similar to the *Theatrum* image (non-reversed).

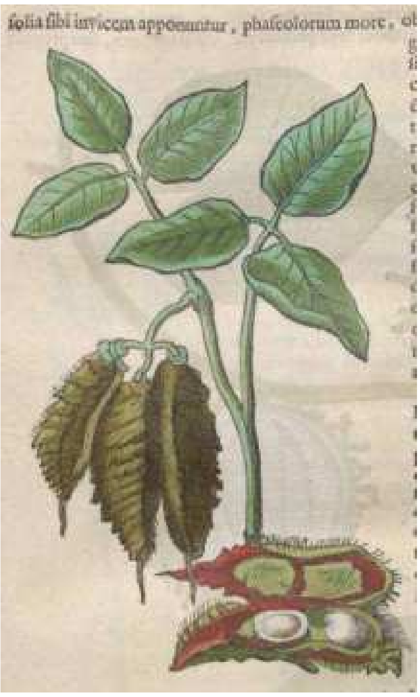

Historiae Plantarum – Herbis: 19a

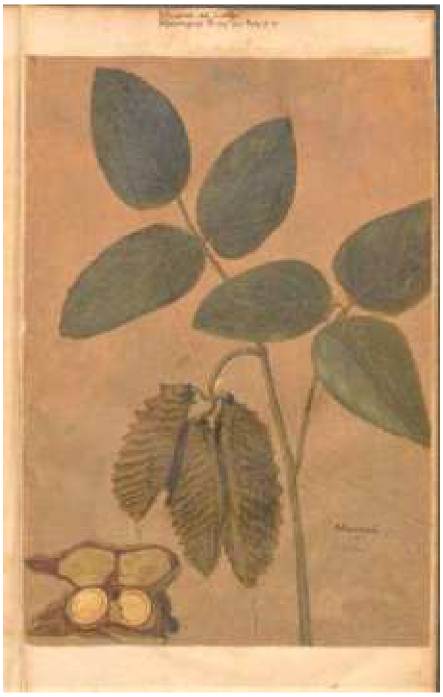

Theatrum Rerum Naturalium: 393

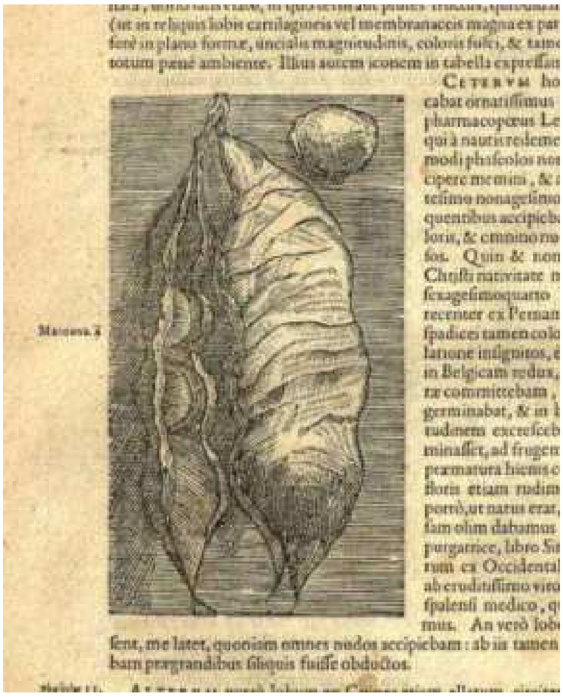

(ut in reliquis lobis cartilagineis vel membranaceis magna ex par
ferè in plano formæ, uncialis magnitudinis, coloris fusci, & tame
totum penè ambiente. Illius autem iconem in tabella expressan
CETERVM ho
cabat ornatissimus
pharmacopœus Le
qui à nautis redeme
modi phaseolos non
cipere memini, & a
tesimo nonagesimo
quentibus accipieba
loris, & omnino ma
sos. Quin & non
Christi nativitate n
sexagesimoquarto
recenter ex Peruan
spadices tamen colo
latione insignitos, e
in Belgicam redux,
ræ committebam,
germinabat, & in l
tudinem excrescebe
minasset, ad frugem
præmatura hiemis c
floris etiam rudim
porrò, ut natus erat,
sam olim dabamus
purgatrice, libro Si
rum ex Occidental
ab eruditissimo viro
spalensi medico, q
mus. An verò lobo
sent, me latet, quoniam omnes nudos accipiebam: ab iis tamen
bam prægrandibus siliquis fuisse obductos.

Macouna 2

Pod of *M. urens* (*Macouna*) in *Exoticorum libri decem* by Clusius (1605: 68)

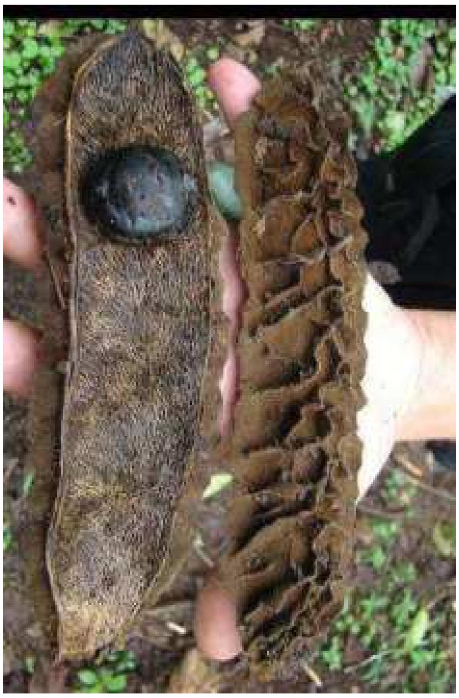

"*Mucuna urens*, the Ox-eye Bean pod" by Dick Culbert (CC BY 2.0)

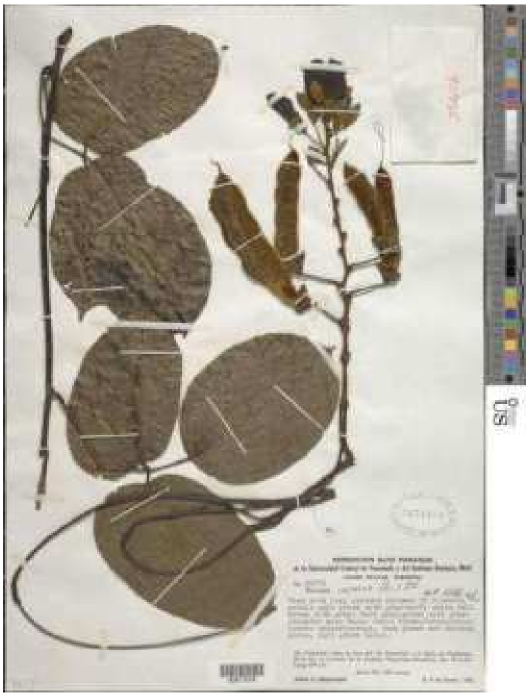

"Mucuna urens (L.) DC." by Julian A. Steyermark is marked with CC0 1.0.

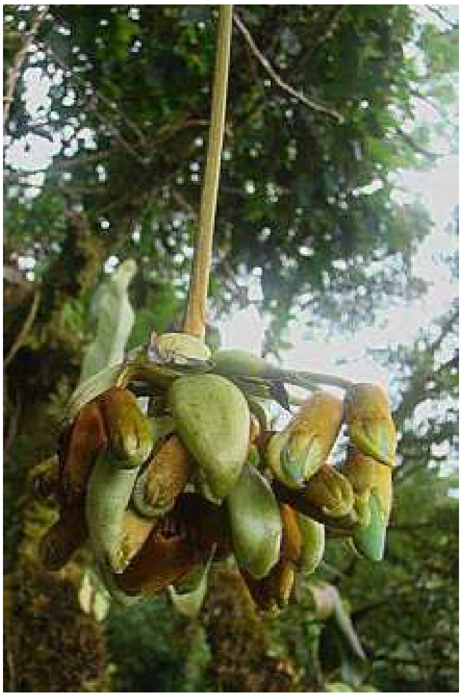

Flowers. "*Mucuna urens*" by Dick Culbert (CC BY 2.0)

Historiae Rerum Naturalium Brasiliae Marcgrave, 1648 Page number 19b

Vernacular name(s) Herba

Species Ipomoea alba L.

Family Convolvulaceae

Notes

We did not find any correspondence between this woodcut and the contemporary or older sources.

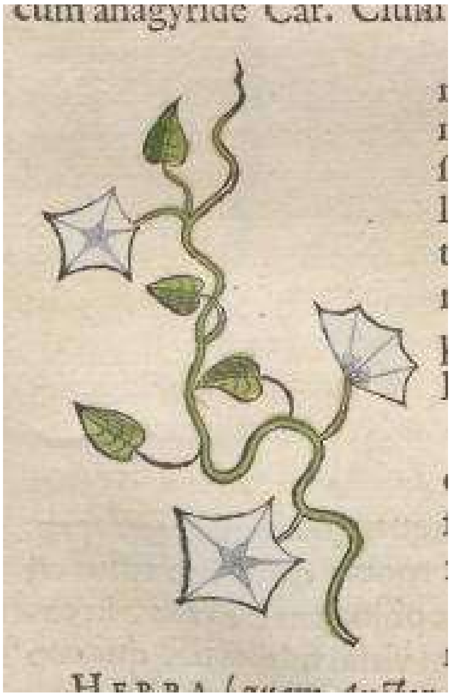

Historiae Plantarum – Herbis: 19b

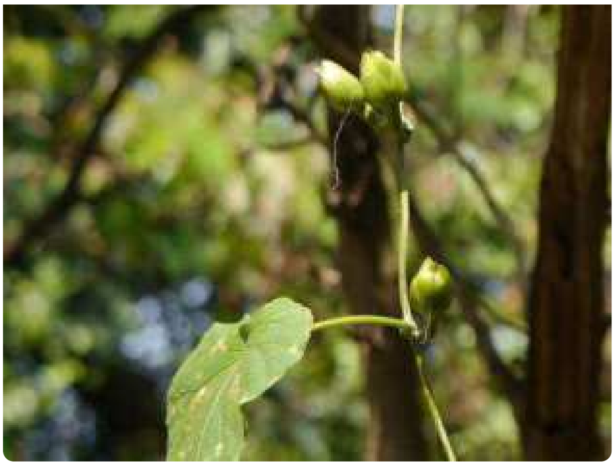

"*I. alba.*" by Dinesh Valke (CC BY-SA 2.0)

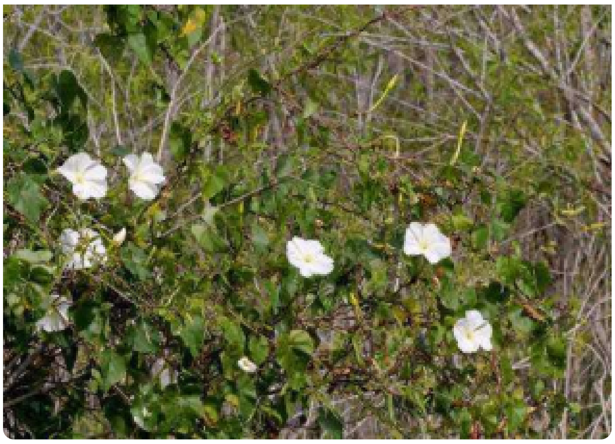

"Moonflowers (*Ipomoea alba*)" by Mary Keim (CC BY-NC-SA 2.0)

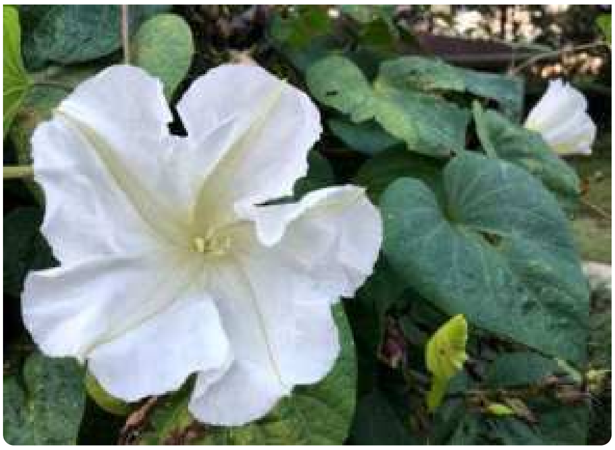

"*I. alba*" by readerwalker (CC BY-NC-SA 2.0)

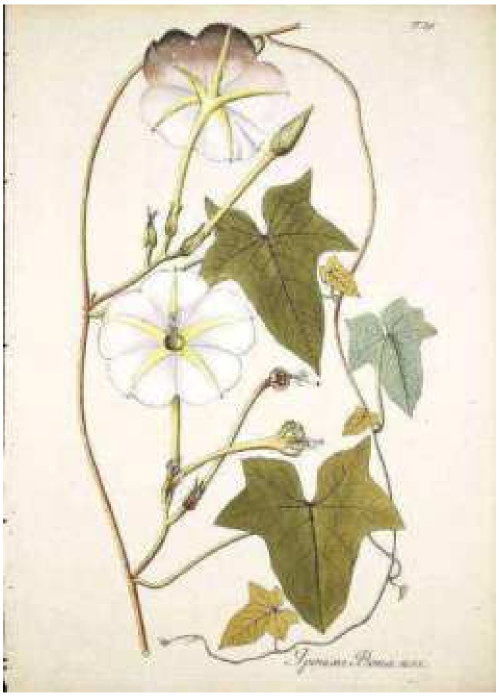

Plantarum rariorum horti caesarei Schoenbrunnensis descriptiones et icones by Jacquin, N.J. von (1797: Vol. I). Missouri Botanical Garden, St. Louis, U.S.A.

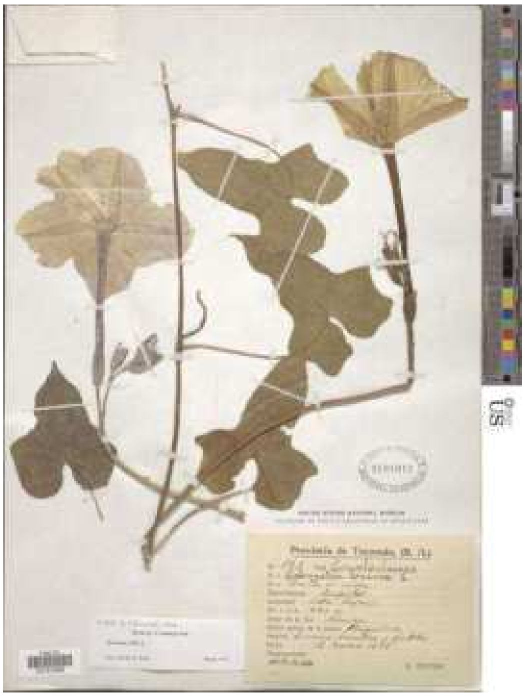

"*Ipomoea alba* L." 03151926 in Smithsonian National Museum of Natural History, by S. Venturi (CC0 1.0)

Historiae Rerum Naturalium Brasiliae | Marcgrave, 1648 | Page number 20

Vernacular name(s) Ibati

Species Ibatia ganglinosa (Vell.) Morillo

Family Apocynaceae

Notes

The woodcut is different to the *Theatrum* image. The image in De Laet's manuscript is a pencil lead drawing, likely the basis for the woodcut in the HNB, exact but reversed, and turned upright (Whitehead and Boeseman 1989).

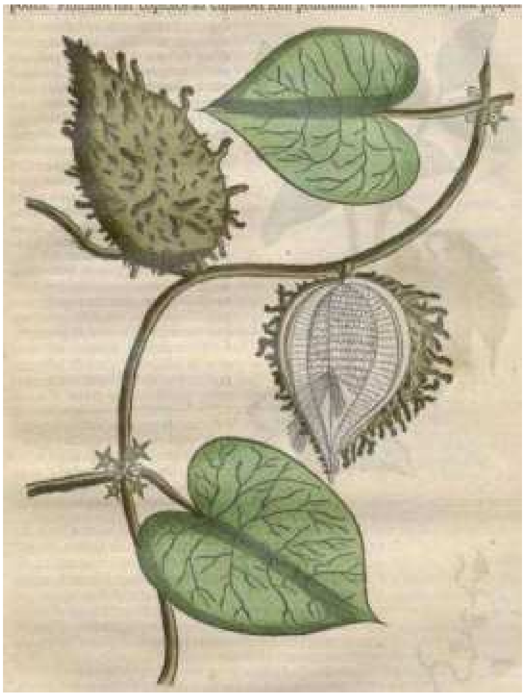

Historiae Plantarum – Herbis: 20

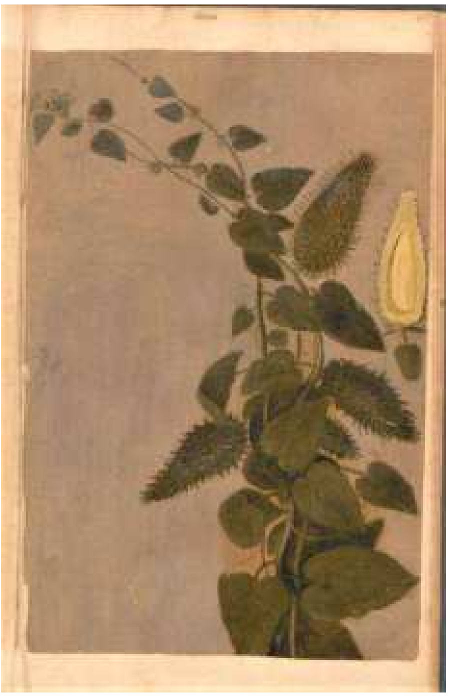

Theatrum Rerum Naturalium: 457

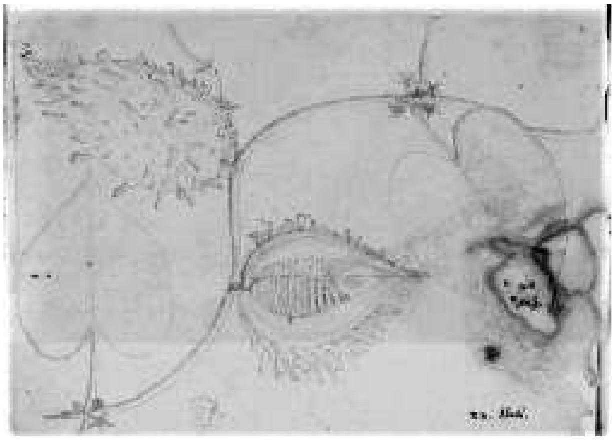

Pencil lead drawing of *I. ganglinosa* in De Laet's manuscript: Sloane MS 1554, f. 22v

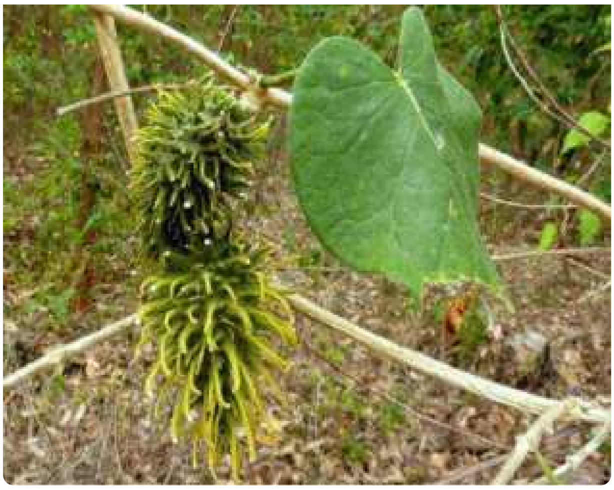

Leaf and fruits. "*M. ganglinosa*" by Alex Popovkin, Bahia, Brazil (CC BY-NC-SA 2.0)

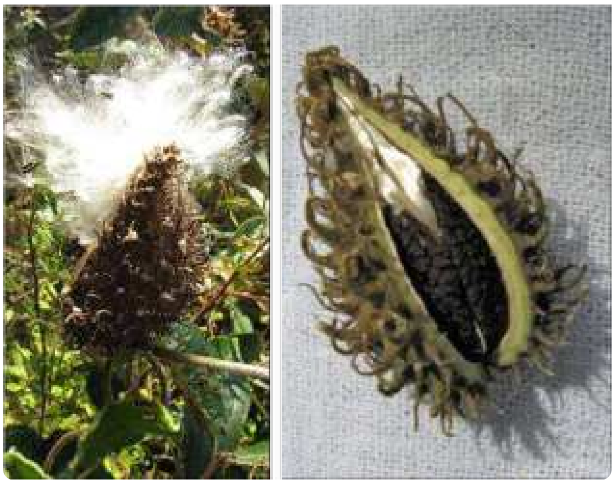

Fruit. "*M. ganglinosa*" by Alex Popovkin, Bahia, Brazil (CC BY-NC-SA 2.0)

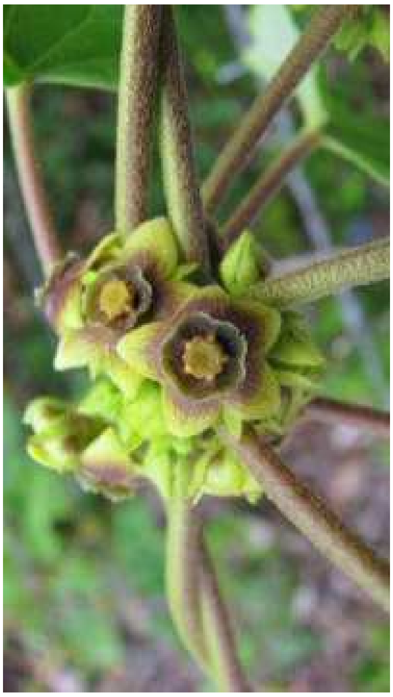

Flowers. "*M. ganglinosa*" by Alex Popovkin, Bahia, Brazil (CC BY-NC-SA 2.0)

Historiae Rerum Naturalium Brasiliae Marcgrave, 1648 Page number 21a

Vernacular name(s) Sesamum. Gangila. Girgilim

Species Sesamum indicum L.

Family Pedaliaceae

Notes

We did not find any correspondence between this woodcut and the contemporary or older sources.

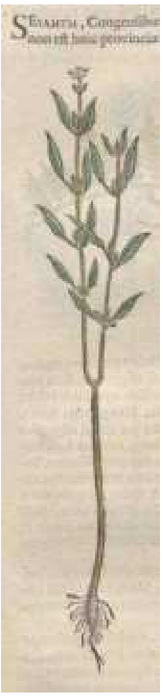

Historiae Plantarum – Herbis: 21a

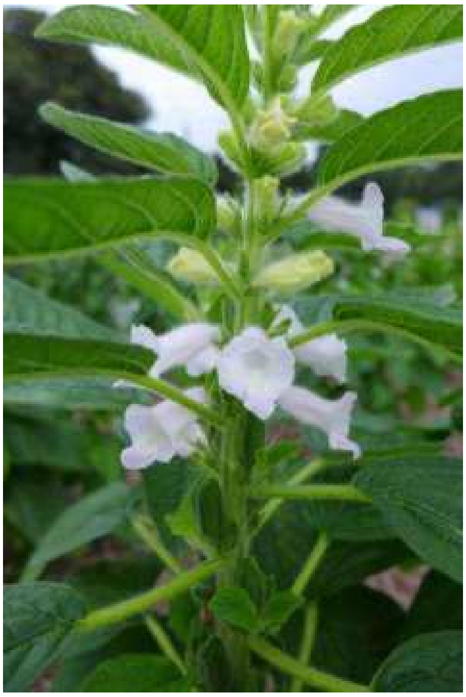

Flowering stem."*S. indicum*" by Scott Zona (CC BY-NC 2.0)

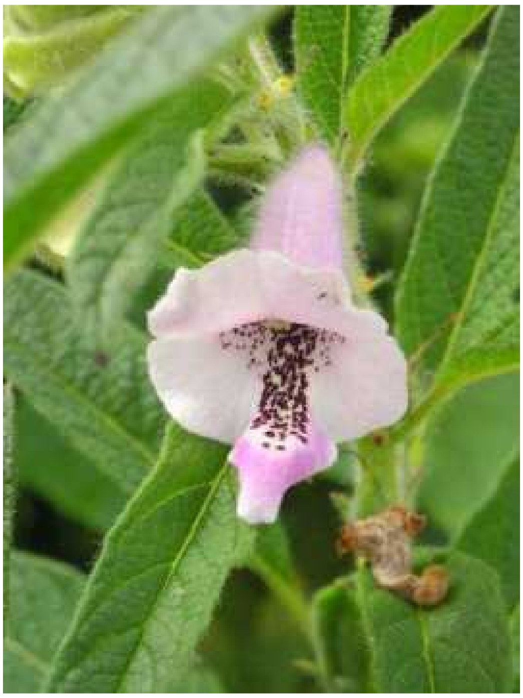

Flower. "*S. indicum*" by .Annna (CC BY 2.0)

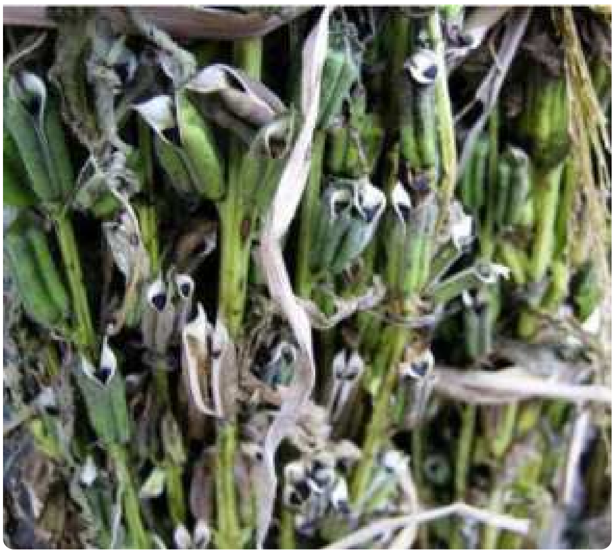

Pods and seeds. "*S. indicum*" by liloh (CC BY-NC-SA 2.0)

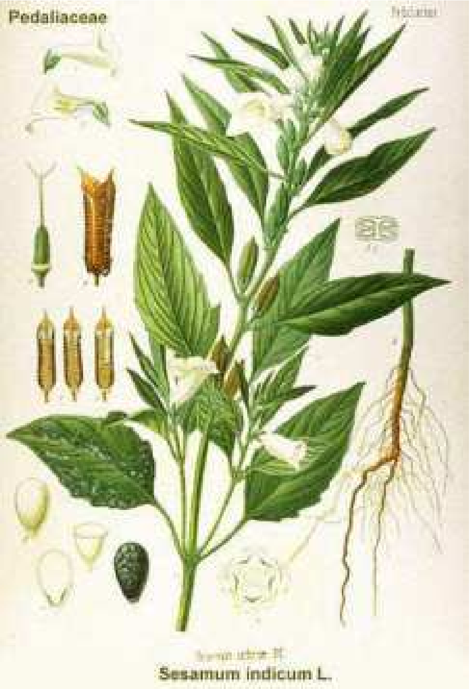

"*S. indicum*, colored lithograph" by filibot.web (CC BY-SA 2.0)

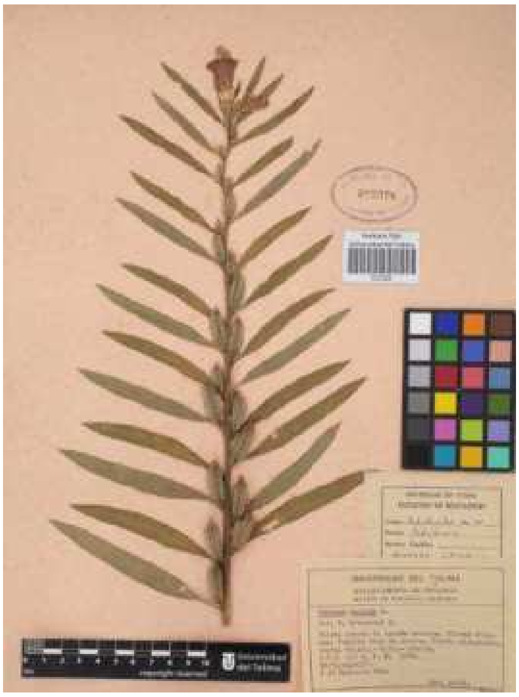

"*S. indicum* L." by Herbario Toli -TOLI-003328-Colombia (CC BY-NC 2.0)

Historiae Rerum Naturalium Brasiliae Marcgrave, 1648 Page number 21b

Vernacular name(s) Herba

Species <u>Turnera ulmifolia L.</u>

Family Passifloraceae

Notes

There is a certain resemblance with the specimen in Marcgrave's herbarium (p. 126) as they both depict the flowering stem and four lateral short branches. However, the specimen is poorly preserved, so it is hard to tell. The isolated flower is a *Passiflora* sp. (mixed collection).

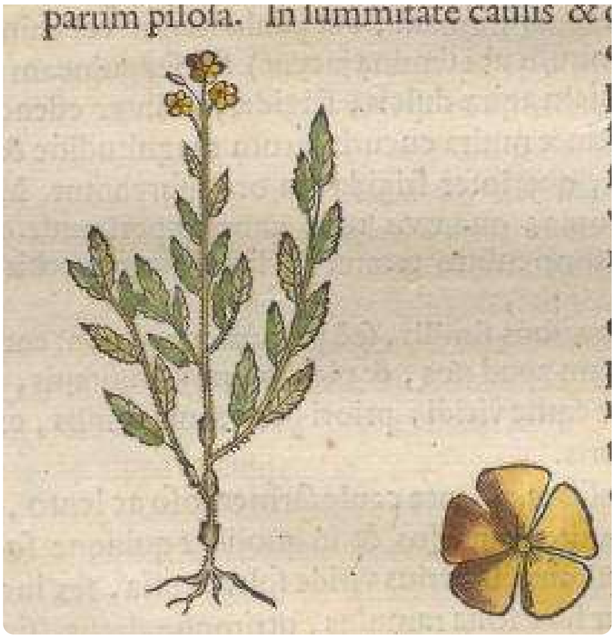

Historiae Plantarum – Herbis: 21b

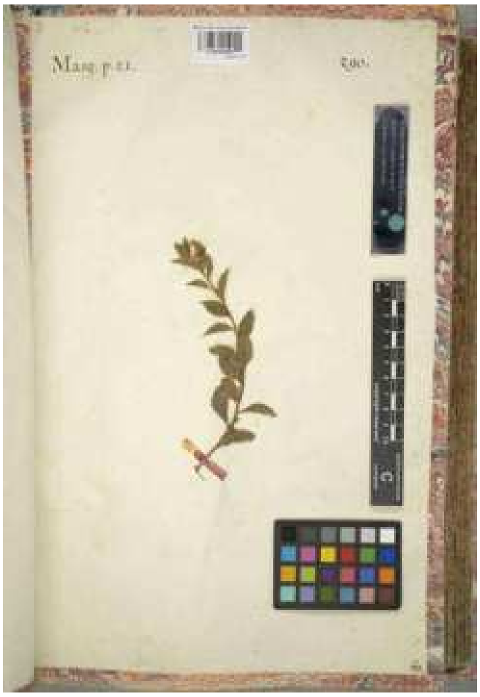

Marcgrave's herbarium: 60

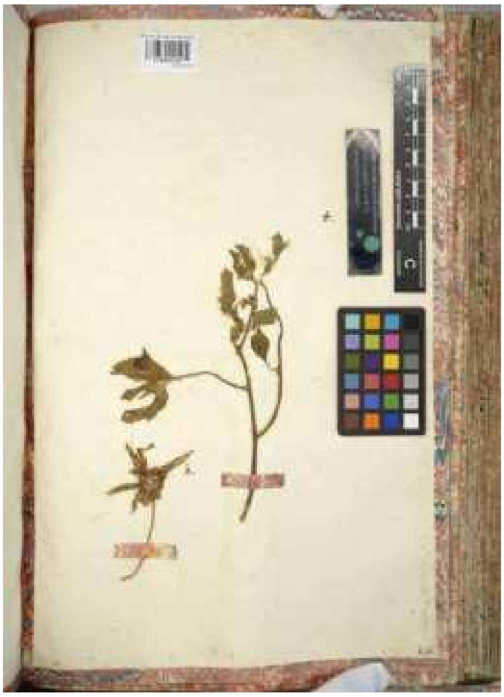

Marcgrave's herbarium: 126

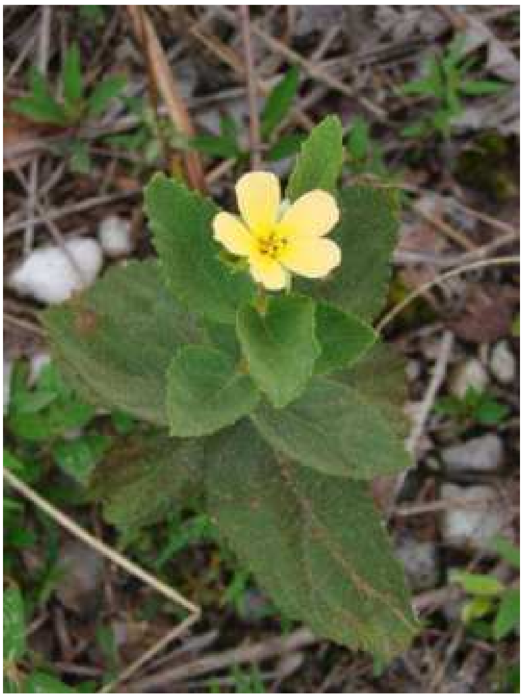

"T. ulmifolia" by João de Deus Medeiros (CC BY 2.0)

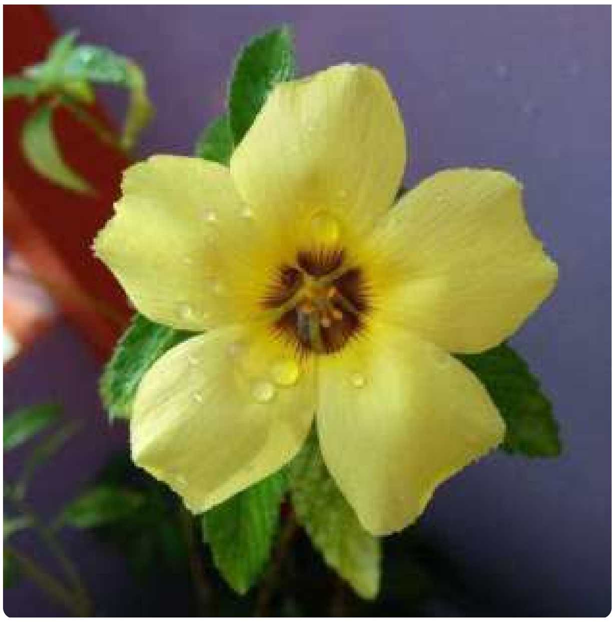

Flower. "Bunga Pukul Delapan (*T. ulmifolia*).jpg" by InTheLA (CC BY-SA 4.0)

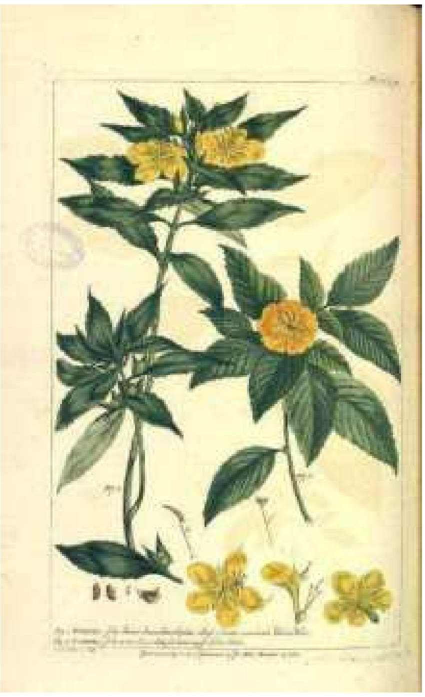

Figures of the most beautiful, useful and uncommon plants, described in the gardeners' dictionary by Miller P. (1755-1760). Real Jardín Botánico, Madrid, [illegible]

Historiae Rerum Naturalium Brasiliae	Marcgrave, 1648	Page number	22

Vernacular name(s)	Iaee. Balancia. Watermeloen. Melo aquaticus
Species	Citrullus lanatus (Thunb.) Matsum. & Nakai
Family	Cucurbitaceae

Notes

The leaves and fruits in the woodcuts look different than those in the *Misc. Cleyeri* and Eckhout's still-life painting (which looks alike the *Misc. Cleyeri*). The specimen in the herbarium shows some resemblance to the vine in the woodcut, as both portray the leaves and the tendrils.

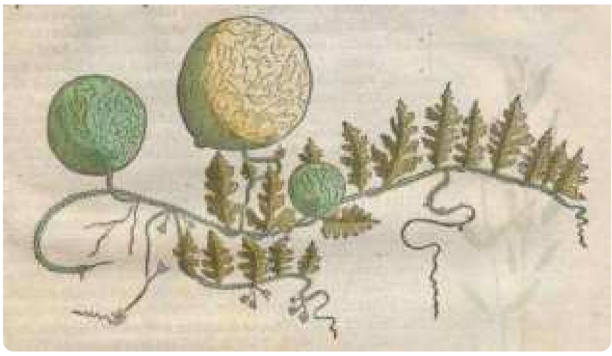

Historiae Plantarum – Herbis: 22

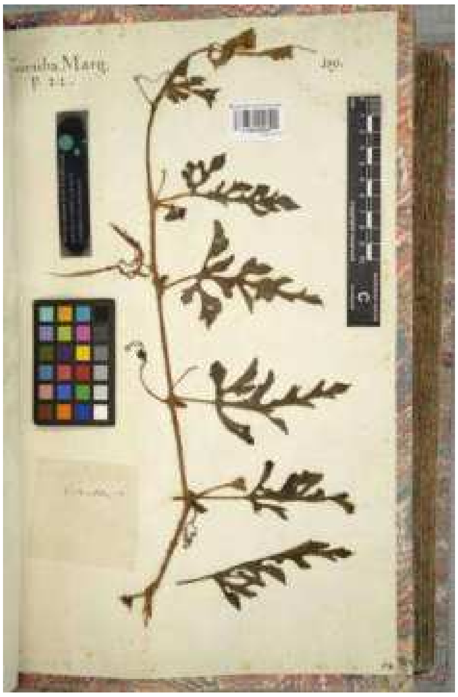

Marcgrave's herbarium: 54

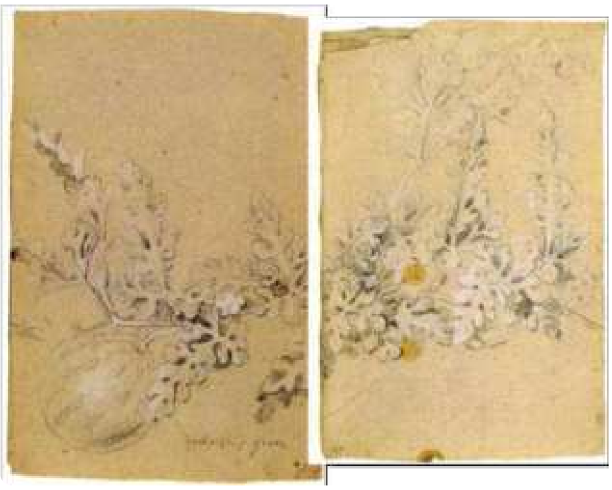

Misc. Cleyeri: 63v and 64r

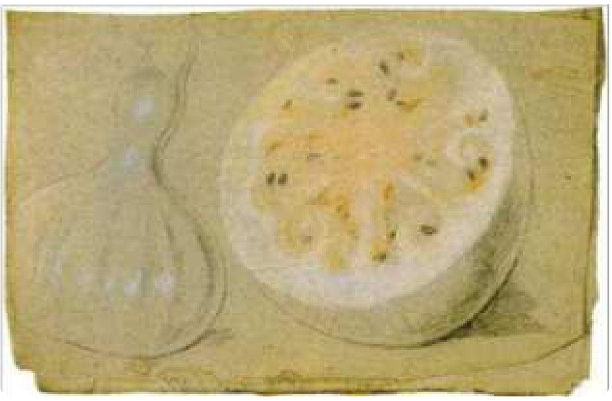

Misc. Cleyeri: 66v

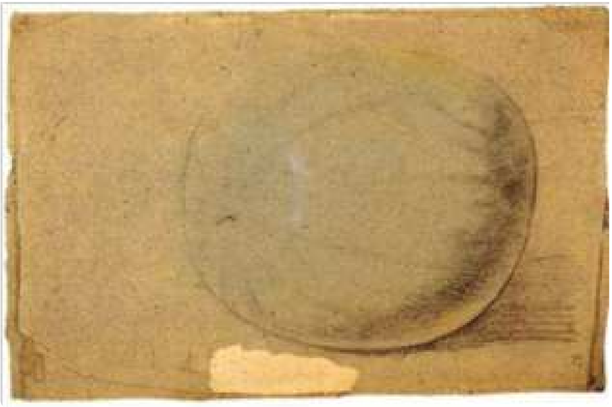

Misc. Cleyeri: 65r

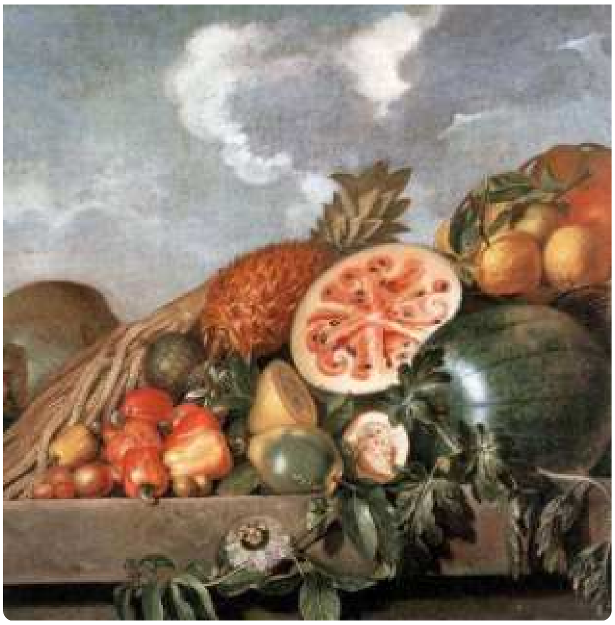

Still-life painting by Eckhout *Watermelon, pineapple and other fruits,* ca. 1640. National Museum of Copenhagen

Historiae Rerum Naturalium Brasiliae Marcgrave, 1648 Page number 23a

Vernacular name(s) Avenqua. Avenca. Aviantum. Avenca major

Species Adiantum latifolium Lam.

Family Pteridaceae

Notes

We did not find any correspondence between this woodcut and the contemporary or older sources.

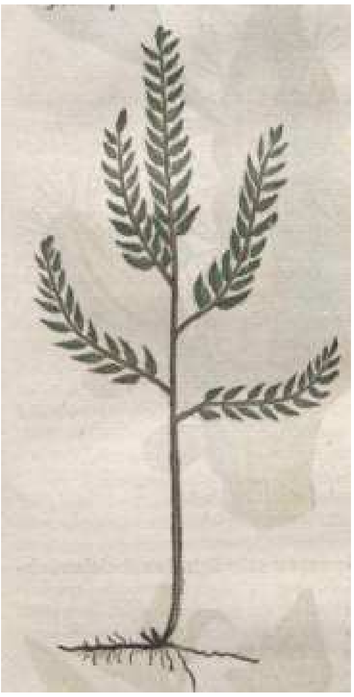

Historiae Plantarum – Herbis: 23a

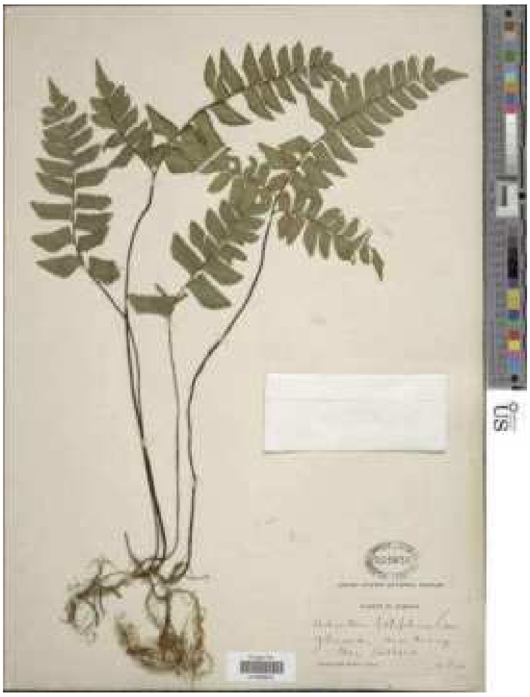

"*A. latifolium* Lam." by A. Moore -01468875- Smithsonian National Museum of Natural History (CC01.0)

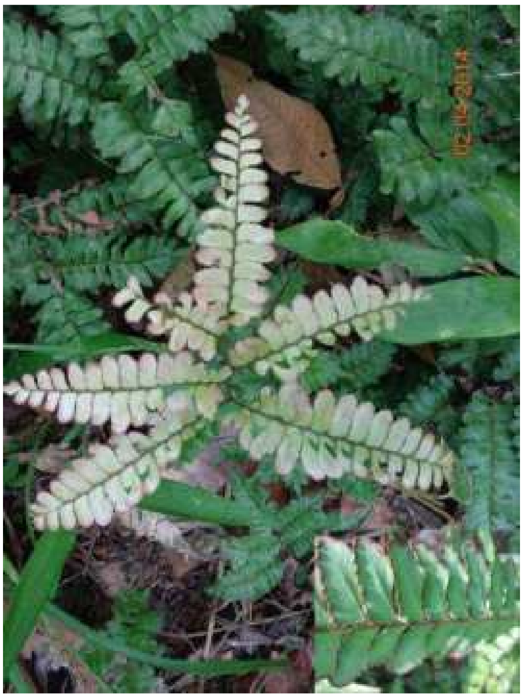

"*A. latifolium*" by Gelo--2014 (CC BY-NC 2.0)

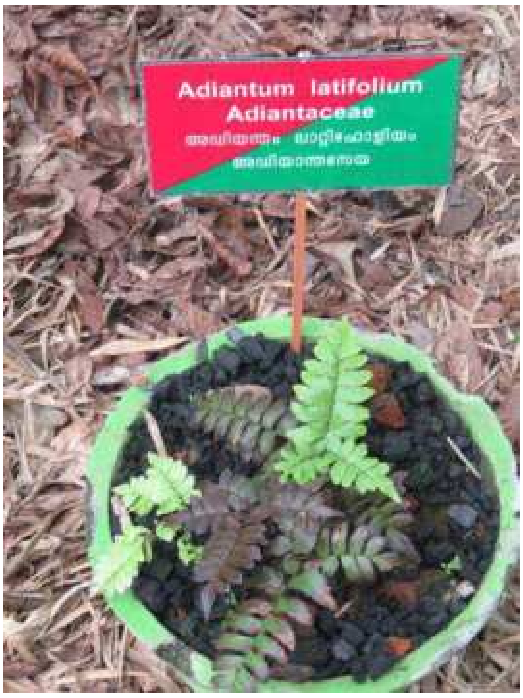

Some ferns from Periya, Wayanad by Vinayaraj (CC-BY-SA-4.0)

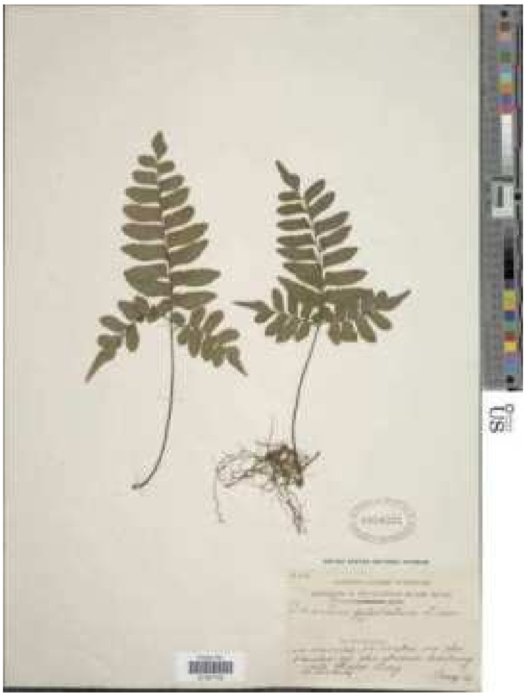

"*A. latifolium* Lam." by A. N. Stewart -01467169- Smithsonian National Museum of Natural History (CC0 1.0)

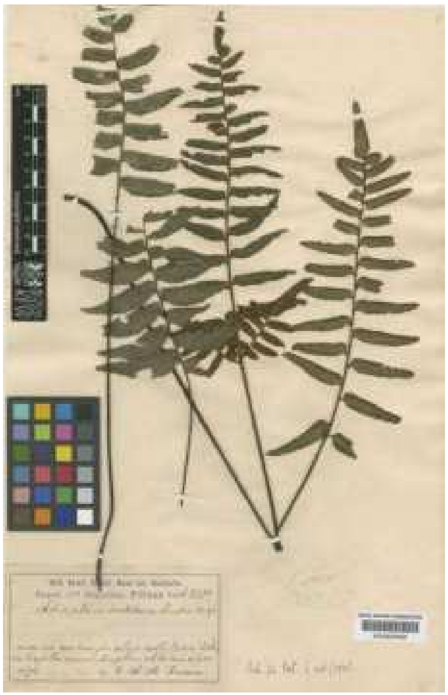

A. latifolium specimen from Kew's Herbarium - K000633058. Retrieved from Plants of the World Online

Historiae Rerum Naturalium Brasiliae — Marcgrave, 1648 — Page number 23b

Vernacular name(s): Avenqua. Avenca. Aviantum. Avenca minor

Species: Pityrogramma calomelanos (L.) Link

Family: Pteridaceae

Notes

We did not find any correspondence between this woodcut and the contemporary or older sources.

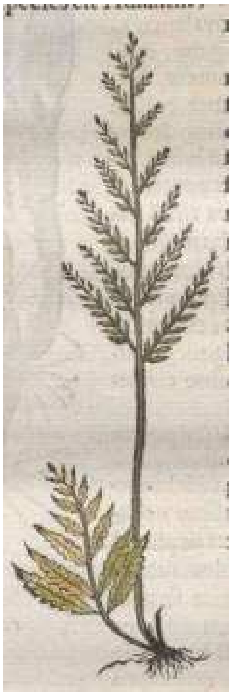

Historiae Plantarum – Herbis: 23b

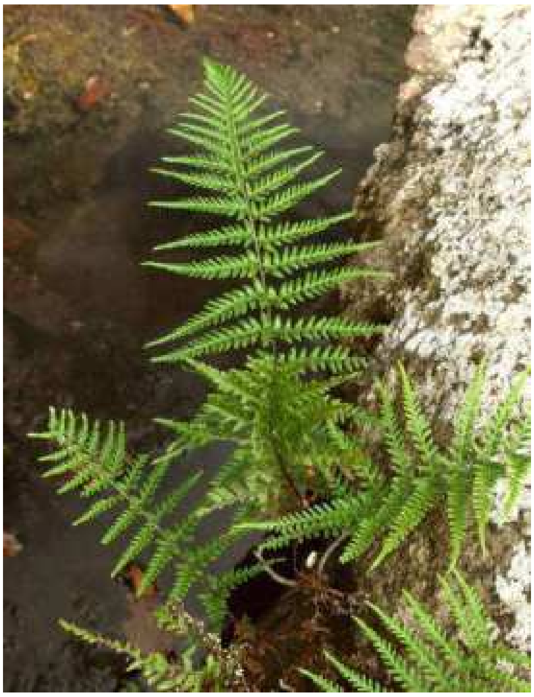

"File:14847-*P. calomelanos*-Tabaro.jpg" by Vojtěch Zavadil (CC BY-SA 4.0)

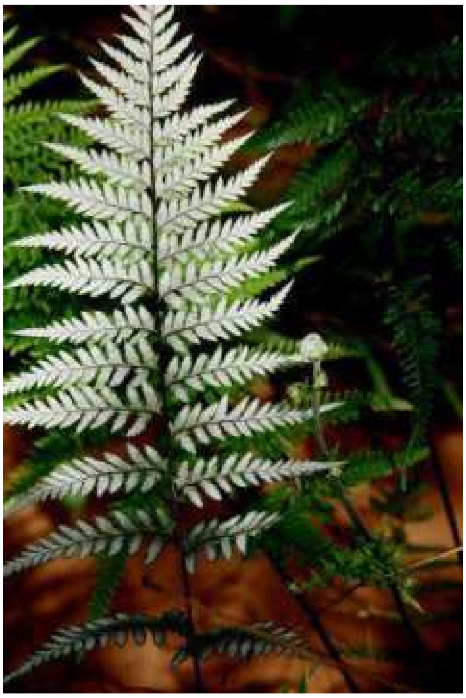

"*P. calomelanos*" by Ahmad Fuad Morad (CC BY-NC-SA 2.0)

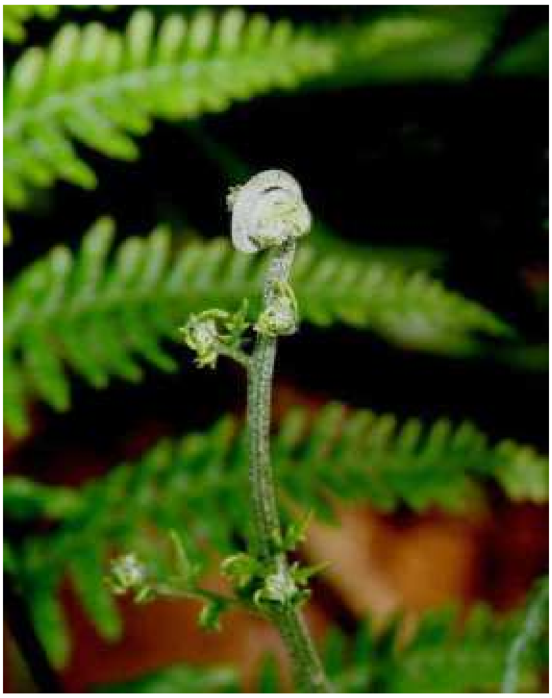

"*P. calomelanos*" by Ahmad Fuad Morad (CC BY-NC-SA 2.0)

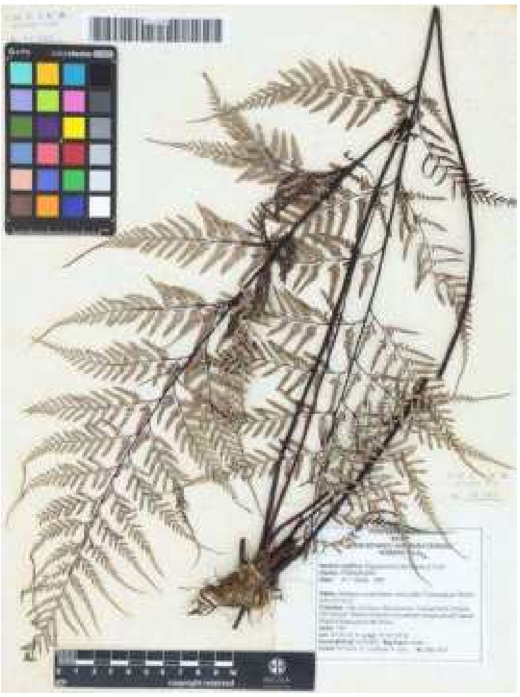

"*P. calomelanos*" by Herbario virtual TULV10340 (CC BY-NC 2.0)

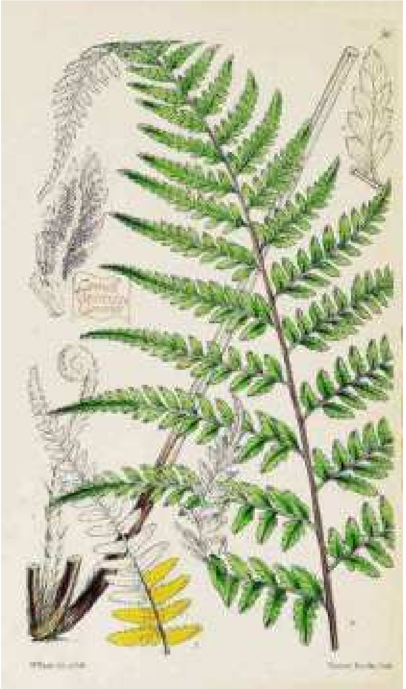

Garden ferns by Hooker, W.J. (1862: t.50)

Historiae Rerum Naturalium Brasiliae | Marcgrave, 1648 | Page number 24a

Vernacular name(s) Iamacaru

Species Cereus fernambucensis Lem.

Family Cactaceae

Notes

The woodcut looks different than the *Theatrum* image. The illustration looks like the painting of the Brazilian cactus and birds (*Guirataieima* and *Ipecu*) at the Hoflößnitz Palace near Dresden, presumably made by Eckhout after sketches made in Brazil (Beschorner 1904; Thomsen 1938). It could have been the result of a collaborative effort with Christian Schiebling, an artist working at the Dresden court (Whitehead and Boeseman 1989). The image in De Laet's manuscript is a proof-woodcut. The same species is depicted differently in Marcgrave's chapter on trees.

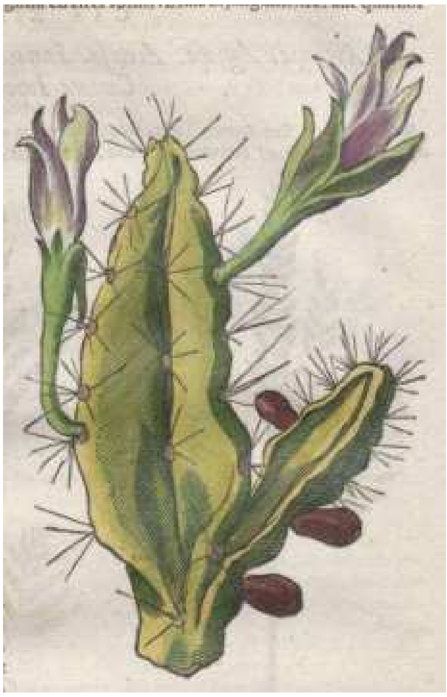

Historiae Plantarum – Herbis: 24a

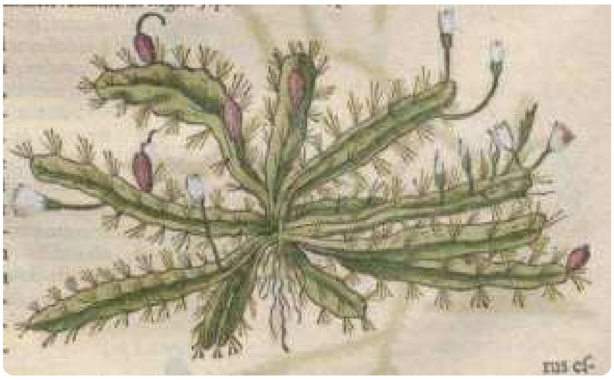

C. fernambucenses depicted in a different woodcut in the HNB *(Marcgrave 1648:* 126c)

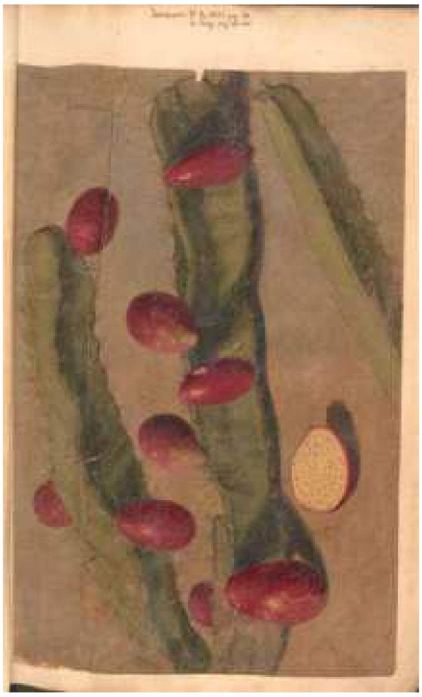

Theatrum Rerum Naturalium: 23

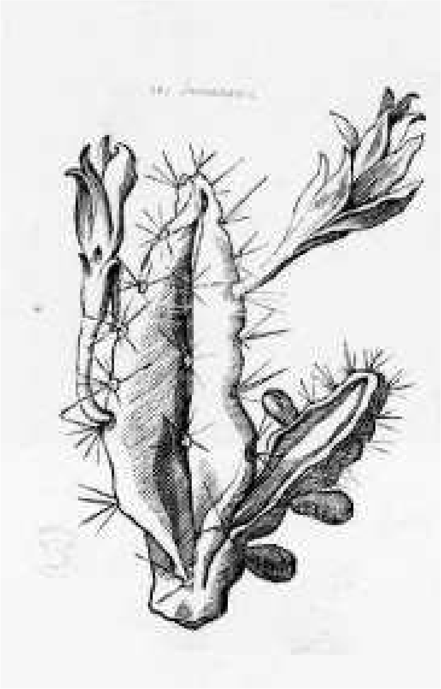

Proof woodcut of *C. fernambucensis* in De Laet's manuscript: Sloane MS 1554, f. 26v

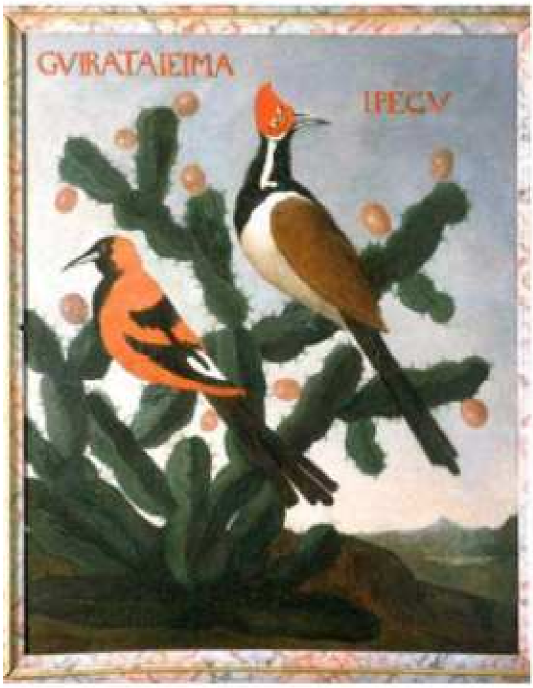

Hoflößnitz Palace (Radebeul, Germany) oil painting with birds and cactus (*C. fernambucensis*)

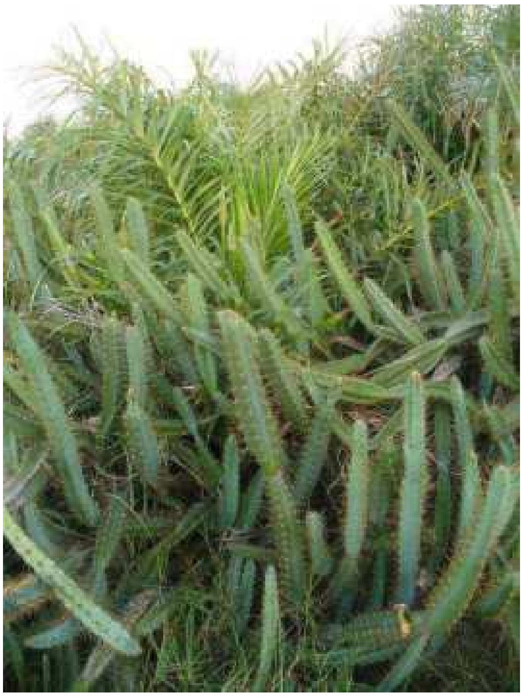

"*C. fernambucensis* subsp. *sericifer*" [syn. *C. fernambucensis*] by Scott Zona (CC BY-NC 2.0)

Historiae Rerum Naturalium Brasiliae | Marcgrave, 1648 | Page number 24b

Vernacular name(s) Belingela. Melongela. Tongu. Macumba

Species <u>Solanum cf. aethiopicum L.</u>

Family Solanaceae

Notes

The woodcut, which shows a flowering and fruiting branch with its roots, looks different than the *Theatrum* image, which only depicts the fruit cut in half and its seeds. A related African species, *S. macrocorpon* could also be the plant described and depicted in the HNB.

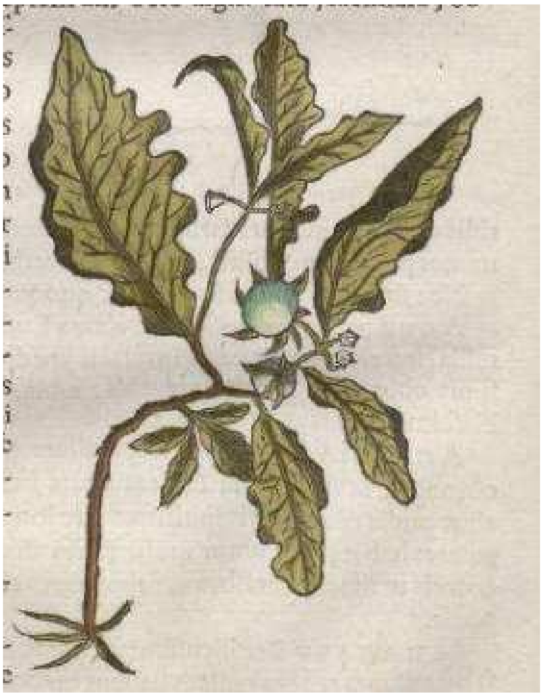

Historiae Plantarum – Herbis: 24b

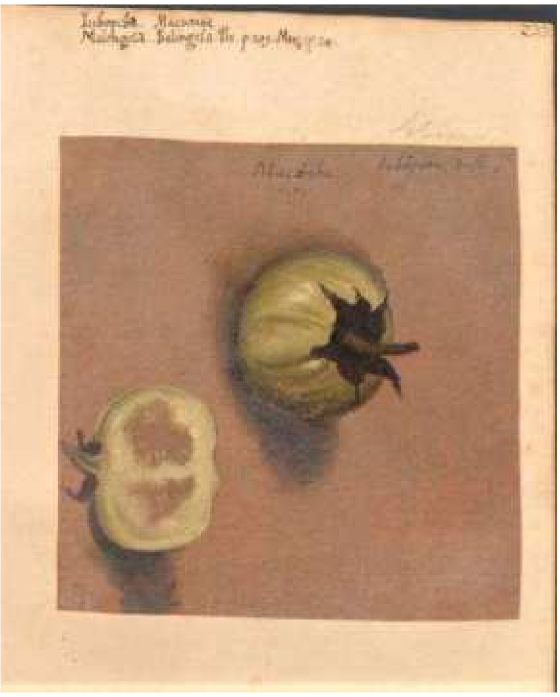

Theatrum Rerum Naturalium: 237

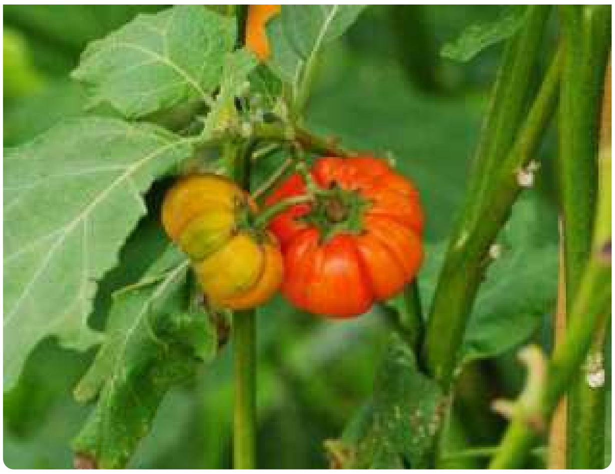

S. aethiopicum. "African Eggplant" by HorsePunchKid (CC BY-NC-SA 2.0)

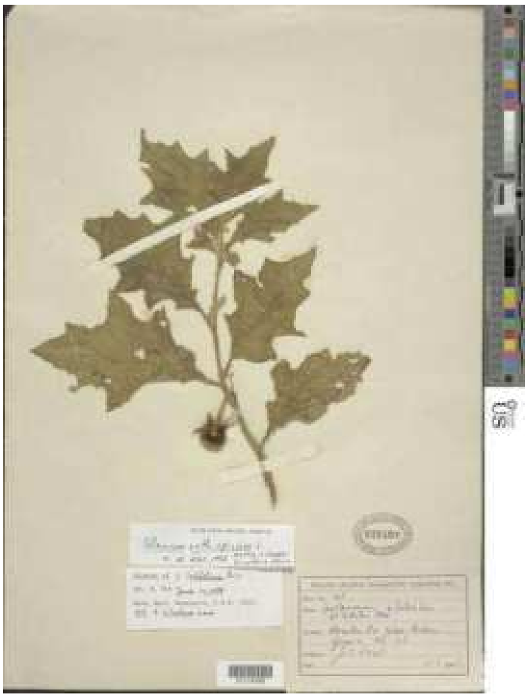

Specimen. "*S. aethiopicum* L." by J. S. Dash (CC0 1.0)

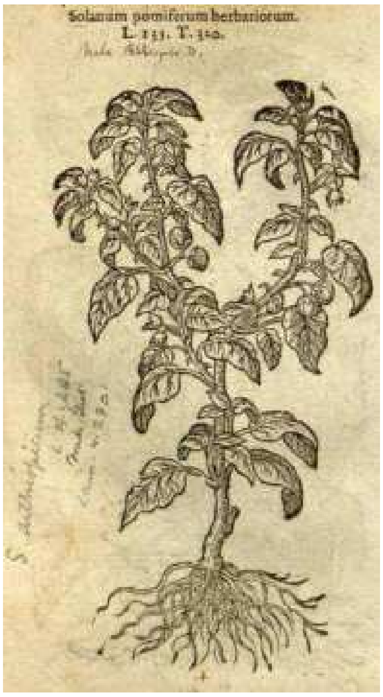

Woodcut of *S. aethiopicum* in *Plantarum seu stirpium icones* by Obel (1591: Vol. I, p. 264)

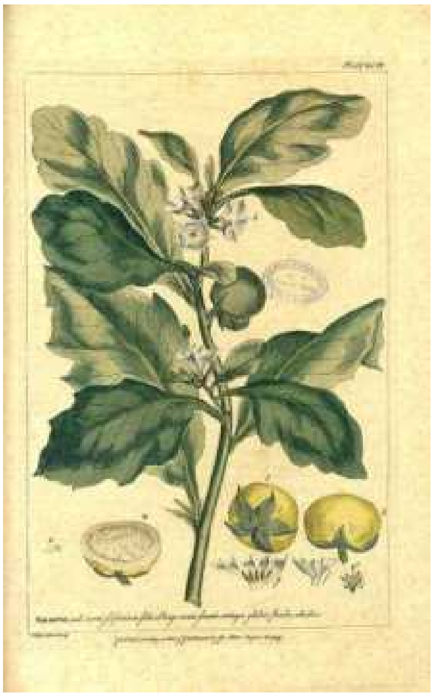

S. macrocorpon engraving, in *Figures of the most beautiful, useful and uncommon plants, described in the gardeners' dictionary* by Miller, P. (1755-1760)

Historiae Rerum Naturalium Brasiliae Marcgrave, 1648 Page number 25a

Vernacular name(s) Iaroba. Casaca (casca?) amargosa

Species Tanaecium cyrtanthum Mart. ex DC.) Bureau & K.Schum.

Family Bignoniaceae

Notes

We did not find any correspondence between this woodcut and the contemporary or older sources. The long pod of *T. cyrtanthum* (as identified by Pickel 2008: 213) does not resemble the rounded one depicted and described in the HNB. This species could be *Tanaecium jaroba* Sw., with oblong fruits (Frazão and Lohmann 2019), which Pickel ruled out because it is not distributed in the northeast of Brazil. However, its current distribution does not exclude the possibility of being present there in the past.

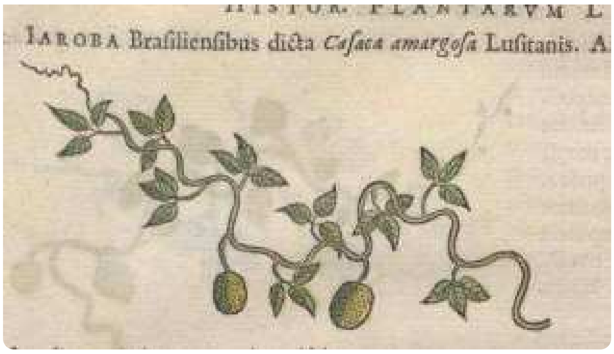

Historiae Plantarum – Herbis: 25a

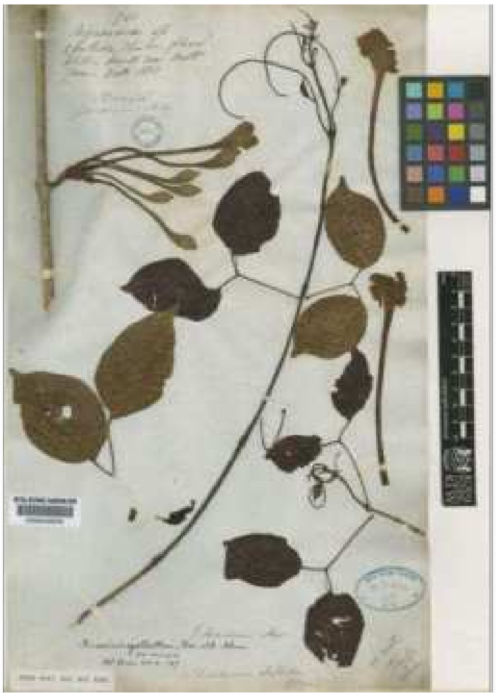

T. cyrtanthum specimen -G. Gardner, 1765, NY 948798, Kew Botanic Gardens- Retrieved from Flora e Funga do Brasil

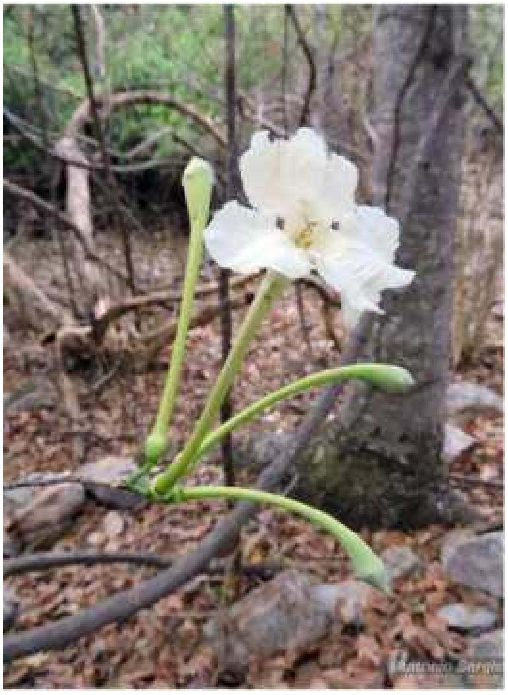

Flowers of *T. cyrtanthum*. Published online by: Annelise Frazão Nunes in Flora e Funga do Brasil. Author: Antonio Sergio

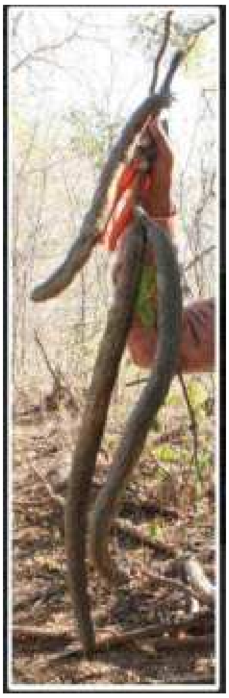

Fruit of *T. cyrtanthum*. Published online by Annelise Frazão Nunes in Flora e Funga do Brasil. Author: Carolina Siniscalchi

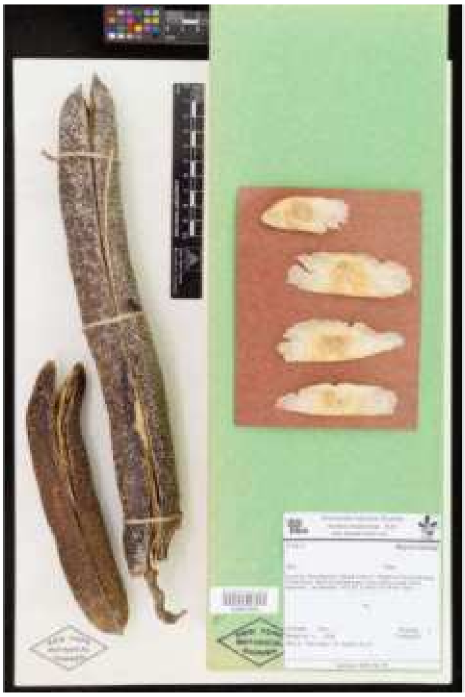

T. cyrtanthum specimen -H. Medeiros, 1239, NY Univ. Federal de Rondônia- Retrieved from Flora e Funga do Brasil

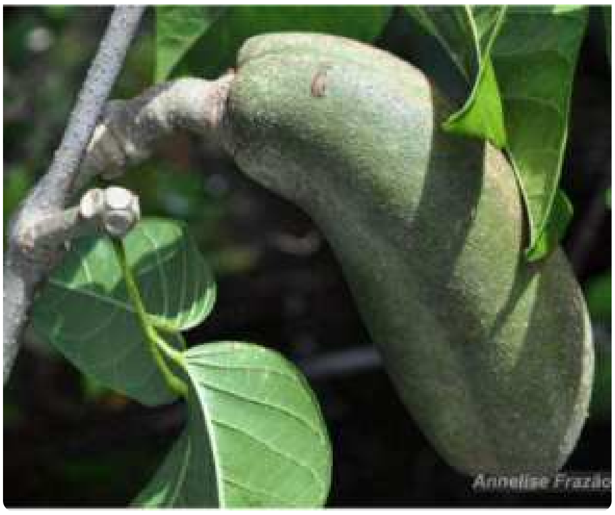

Fruit of *T. jaroba*. Published online by: Annelise Frazão Nunes in Flora e Funga do Brasil. Author: Annelise Frazão

Historiae Rerum Naturalium Brasiliae	Marcgrave, 1648	Page number	25b

Vernacular name(s) Camara Iapo. Mentastro

Species Ageratum conyzoides (L.) L.

Family Asteraceae

Notes

Although both images show flowering plants, the woodcut is different than the *Theatrum* illustration, which in addition depicts a day-flying Brazilian moth (the "White striped black moth" *Trichodezia albovittata*).

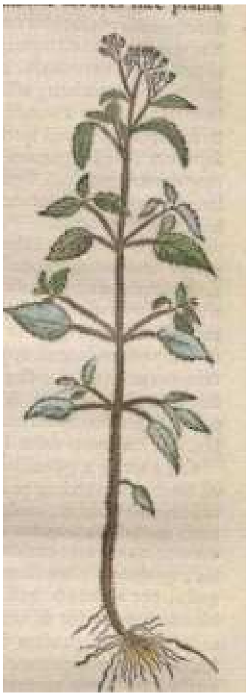

Historiae Plantarum – Herbis: 25b

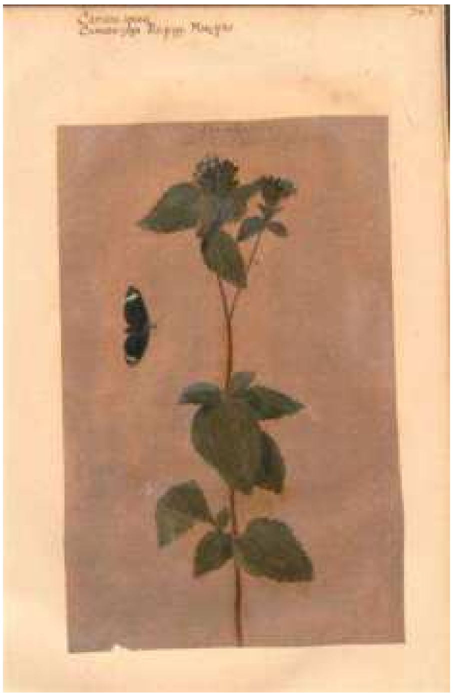

Theatrum Rerum Naturalium: 343

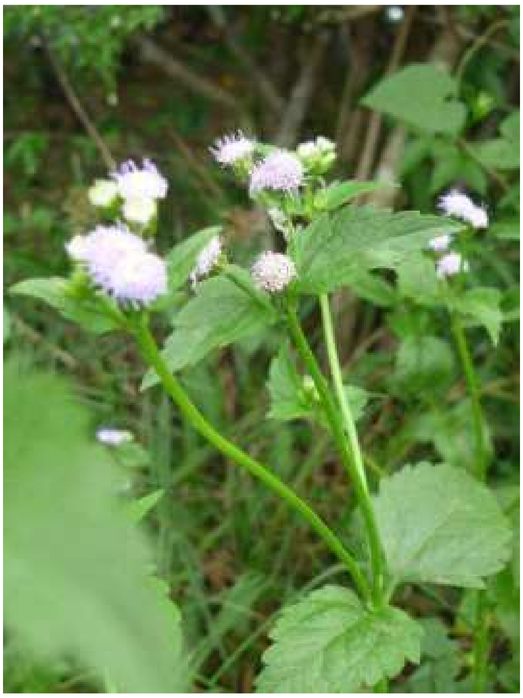

"Cỏ hôi *Ageratum conyzoides* L., thuộc họ Cúc – Asteraceae" by Hoa Trai Viet Nam (CC BY-ND 2.0)

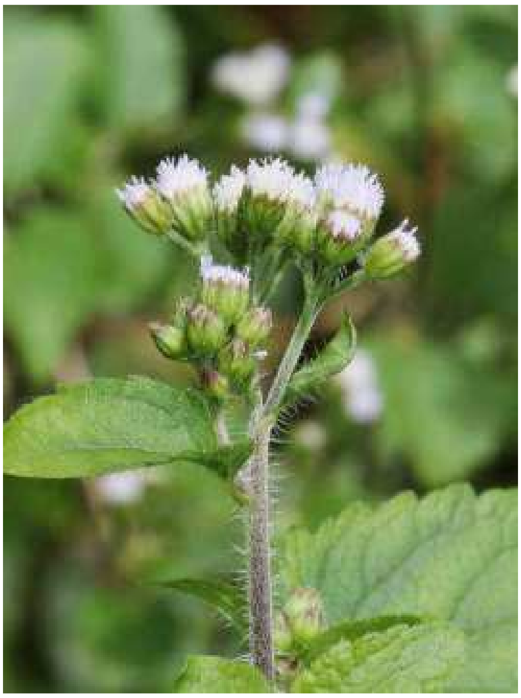

"*A. conyzoides*" by Mauricio Mercadante (CC BY-NC-SA 2.0)

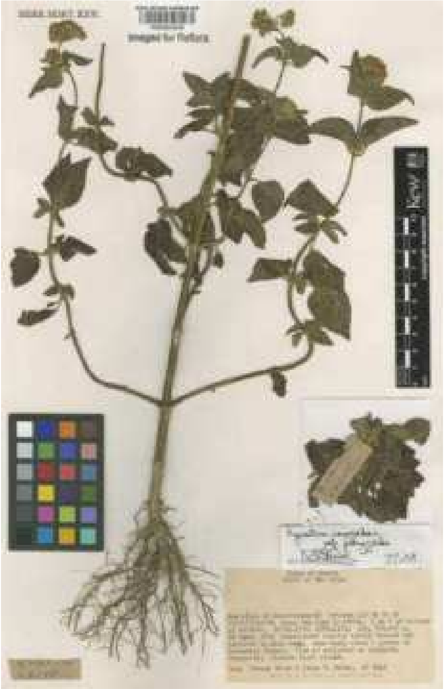

A. conyzoides specimen from Kew's Herbarium - K000924540. Retrieved from Plants of the World Online

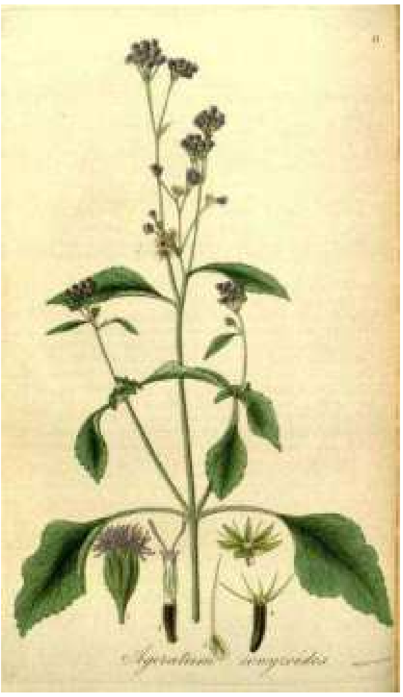

A. conyzoides engraving in *Exotic Flora* by Hooker, W. J. (1823: Vol. I, t. 15)

Historiae Rerum Naturalium Brasiliae Marcgrave, 1648 Page number 26a

Vernacular name(s) Caapeba. Erva de Nossa Senhora. Cipó de cobras

Species Cissampelos glaberrima A.St.-Hil.

Family Menispermaceae

Notes

The herbarium shows a sterile twig while the woodcut shows a flowering shoot with its roots. Seven years after the publication of the HNB, Danish physician and Historian, Ole Worm published an image of the root of *C. glaberrima* in *Museum Wormiamum* (1655: 157), called *Raiz de Nossa Seinora*, which came from Brazil. De Laet and Worm maintained a fruitful correspondence in which natural objects, such as plant material collected by Marcgrave, were shared (Andrade-Lima et al. 1977: 124). Hence, this dry root could have been part of the material shipped to De Laet from Brazil.

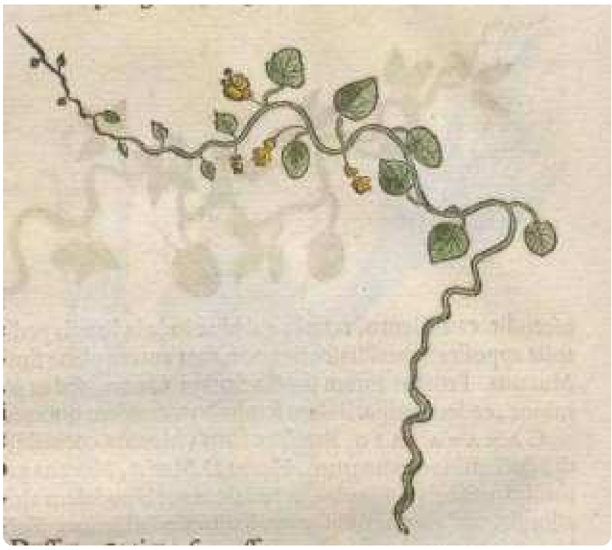

Historiae Plantarum – Herbis: 26a

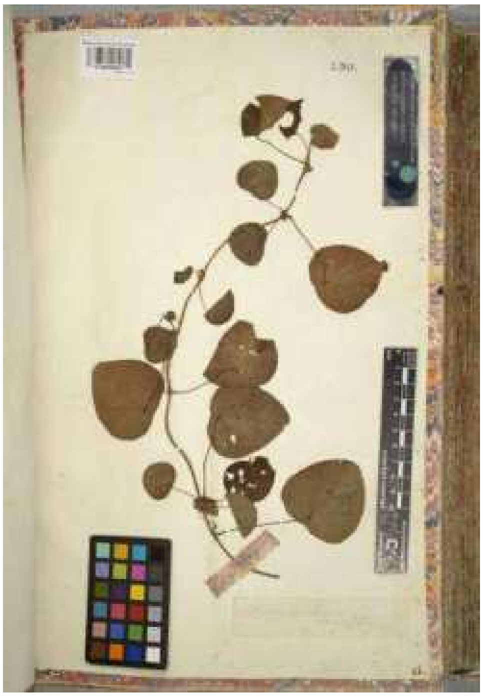

Marcgrave's herbarium: 81

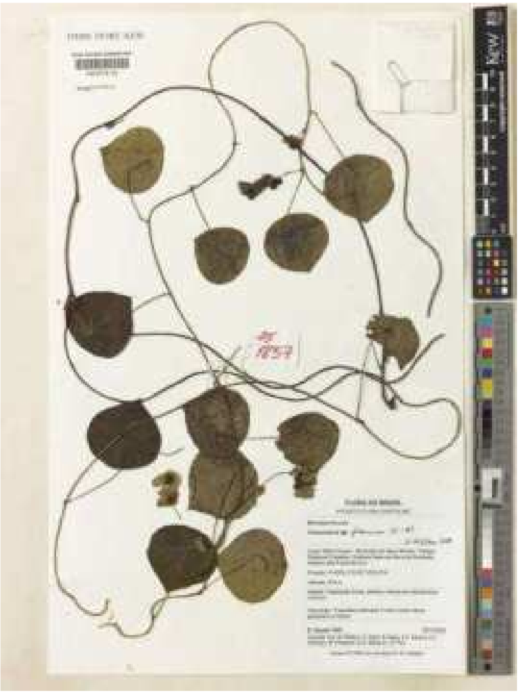

C. glaberrima specimen from Kew's Herbarium - K000578140. Retrieved from Plants of the World Online

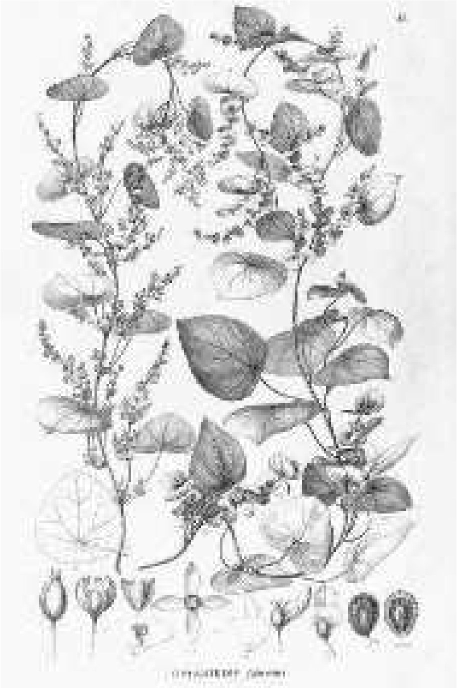

Engraving of *C. glaberrima* in Martius, C.F.P. von, Eichler, A.G., Urban, I., *Flora Brasiliensis* (1841-1872) Vol. 13 (1): 45

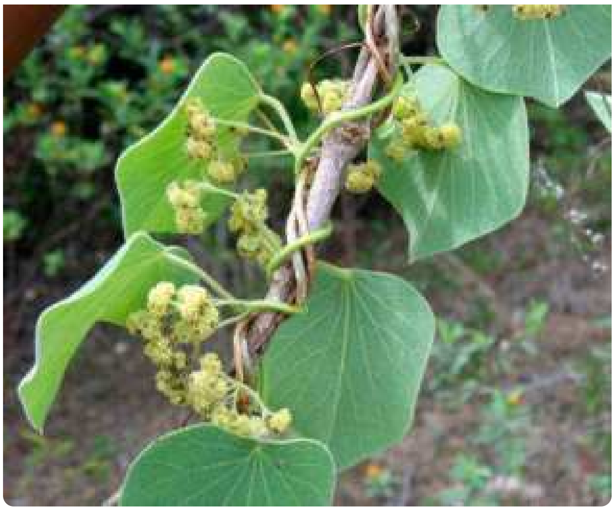

Cissampelos glaberrima A.St.-Hil. "Randrianasolo - 554 - collected in Madagascar by Richard Randrianaivo (CC BY-NC-ND 3.0)

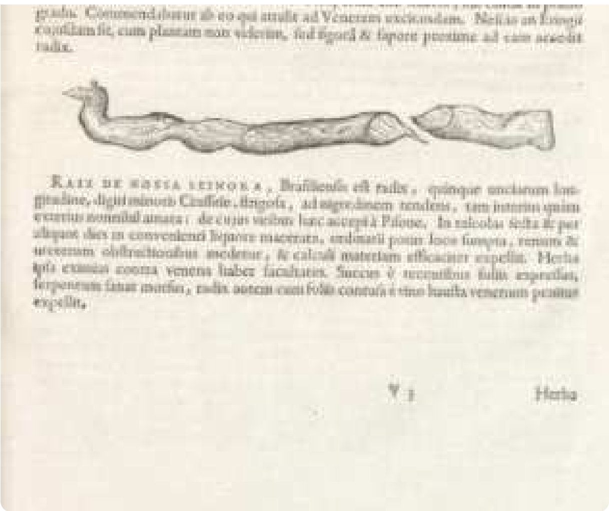

[illegible] gradu. Commendabatur ab eo qui attulit ad Venerem excitandam. Nescio an [illegible] cujusdam sit, cum plantam non viderim, sed figurâ & sapore proxime ad eam accedit radix.

RAIZ DE NOSSA SEINORA, Brasiliensis est radix, quinque unciarum longitudine, digiti minoris Crassitie, fibrosa, ad nigredinem tendens, tam interius quam exterius nonnihil amara: de cujus viribus haec accepi à Pisone. In taleolas secta & per aliquot dies in convenienti liquore macerata, ordinarii potus loco sumpta, renum & uterorum obstructionibus medetur, & calculi materiam efficaciter expellit. Herba ipsa eximias contra venena habet facultates. Succus è tenerioribus foliis expressus, serpentum sanat morsus, radix autem cum foliis contusa è vino hausta venenum potenter expellit.

Y 3 Herba

Root of *C. glaberrima* in *Museum Wormianum* by . Worm (1655: 157)

Historiae Rerum Naturalium Brasiliae Marcgrave, 1648 Page number 26b

Vernacular name(s) Embuayembo. Occoembo

Species Elaphoglossum latifolium (Sw.) J.Sm.

Family Polypodiaceae

Notes

We did not find any correspondence between this woodcut and the contemporary or older sources. The description could correspond to this species, but certainly not the woodcut, as this is not a fern. It could have been misplaced by De Laet.

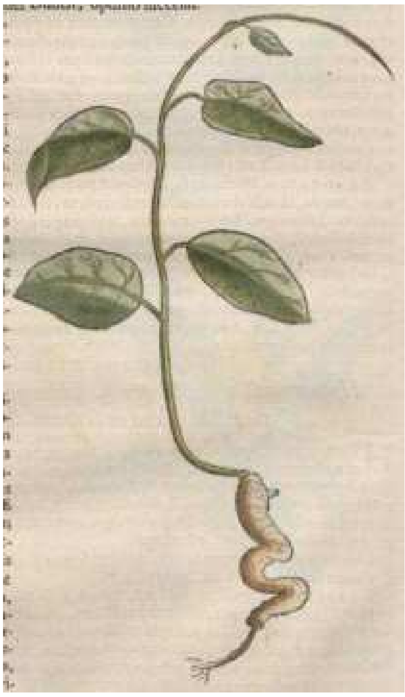

Historiae Plantarum – Herbis: 26b

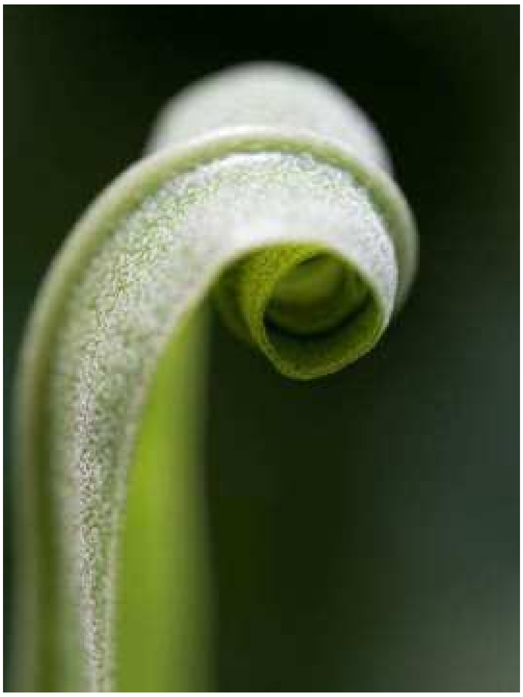

"*E. latifolium*" by wundoroo (CC BY-NC-SA 2.0)

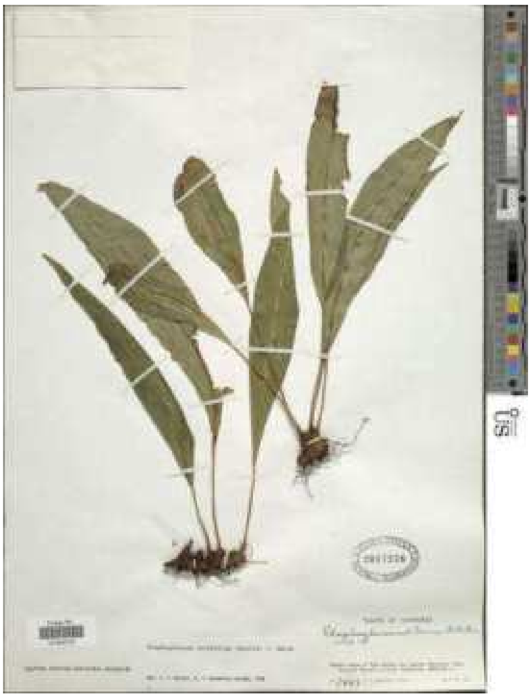

Specimen. "*E. latifolium*" by Conrad V. -01564079- Smithsonian National Museum of Natural History (CC0 1.0)

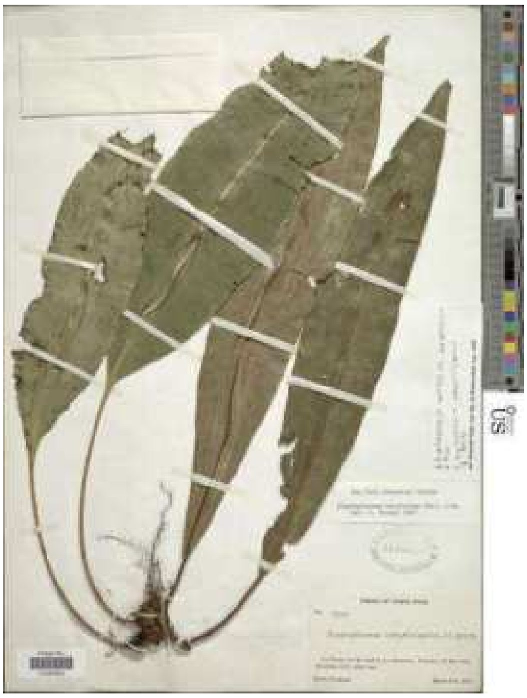

Specimen. "*E. latifolium*" by E. Scamman -01564224- Smithsonian National Museum of Natural History (CC0 1.0)

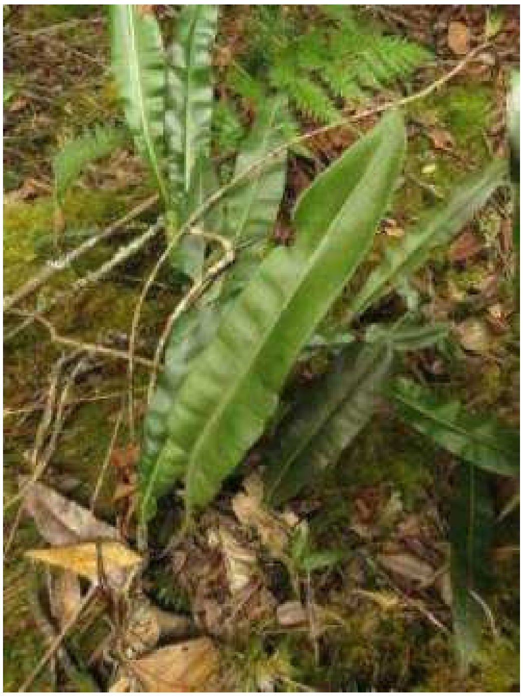

E. latifolium - Subachoque, Cundinamarca, Colombia mateohernandezschmidt (CC). Retrieved from Plants of the World Online

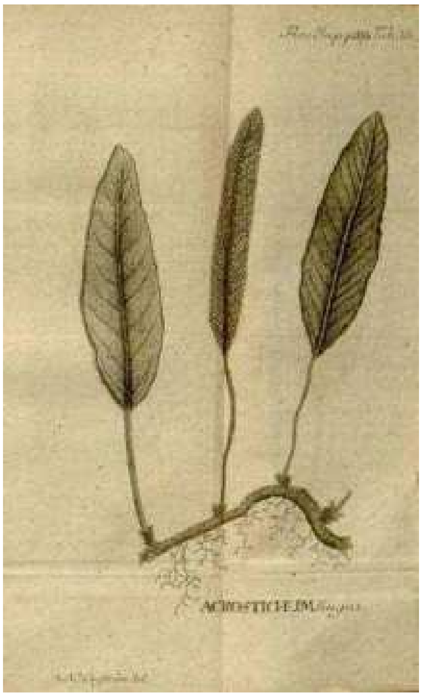

Engraving of a related species of E. latifolium (*E. lingua*) in Flora Japonica by Thunberg, C.P. (1784: t. 33)

Historiae Rerum Naturalium Brasiliae	Marcgrave, 1648	Page number	27a

Vernacular name(s) Taioia

Species Cayaponia tayuya (Vell.) Cogn.

Family Cucurbitaceae

Notes

The image in De Laet's manuscript is a pencil lead drawing, likely the basis for the woodcut in the HNB, reversed and turned upright (Whitehead and Boeseman 1989).

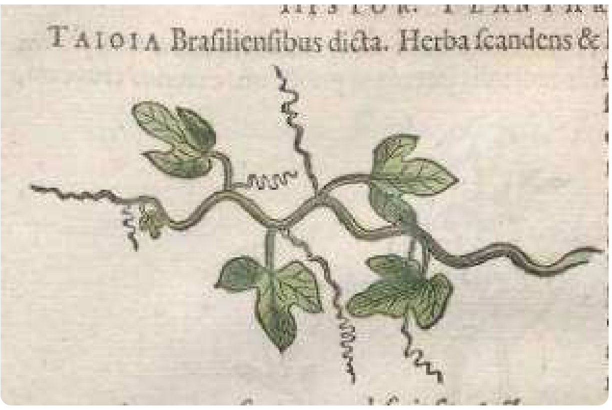

Historiae Plantarum – Herbis: 27a

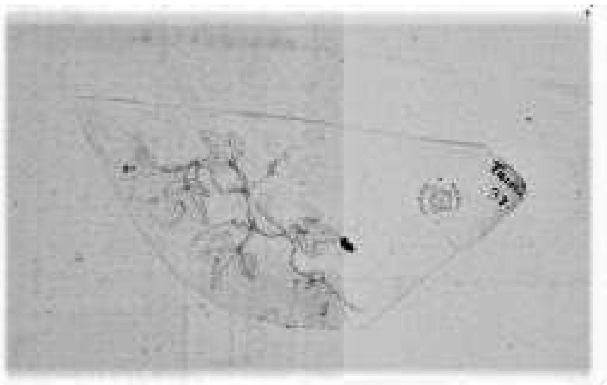

Pencil drawing of *C. tatuya* in De Laet's manuscript: Sloane MS 1554, f. 40v

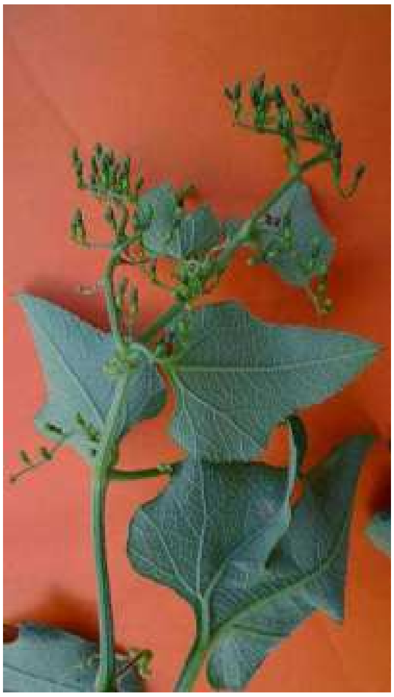

Shoot with leaves and buds. "*C. tayuya*" by Alex Popovkin, Bahia, Brazil (CC BY-NC-SA 2.0)

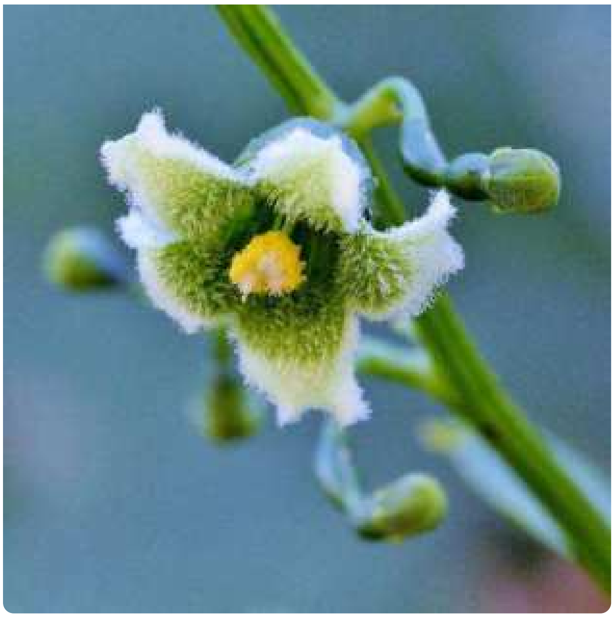

Flower. "*C. tayuya*" by Mauricio Mercadante (CC BY-NC-SA 2.0)

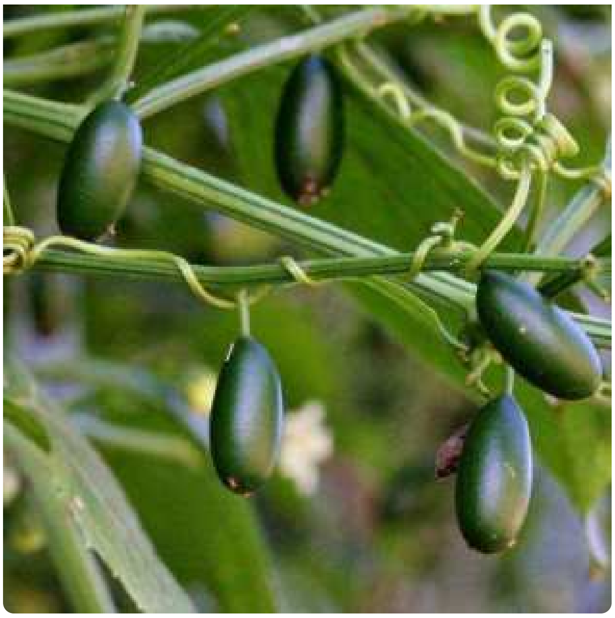

Fruits. "*C. tayuya*" by Mauricio Mercadante (CC BY-NC-SA 2.0)

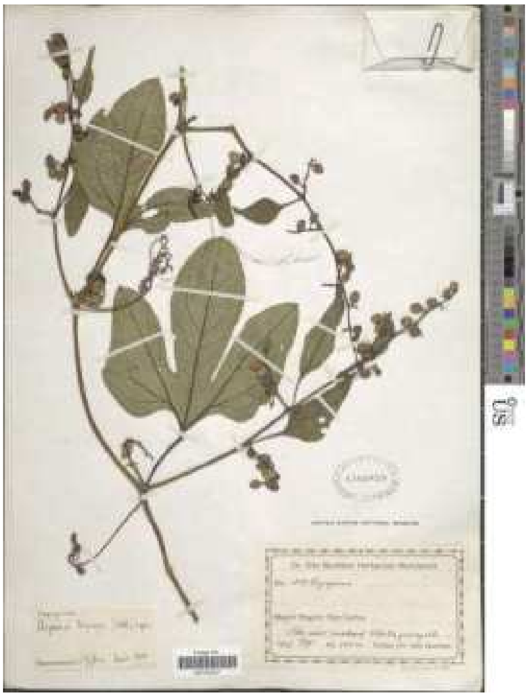

Specimen. "*C. tayuya*" by Otto Buchtien -02722547- Smithsonian National Museum of Natural History (CC0 1.0)

Historiae Rerum Naturalium Brasiliae	Marcgrave, 1648	Page number 27b

Vernacular name(s) Erva do Capitaon

Species Hydrocotyle bonariensis Comm. ex Lam.

Family Araliaceae

Notes

The woodcut looks moderately similar to the *Theatrum* image.

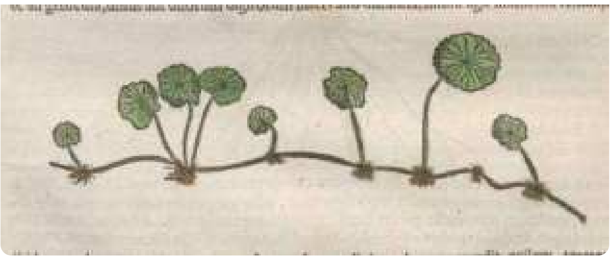

Historiae Plantarum – Herbis: 27b

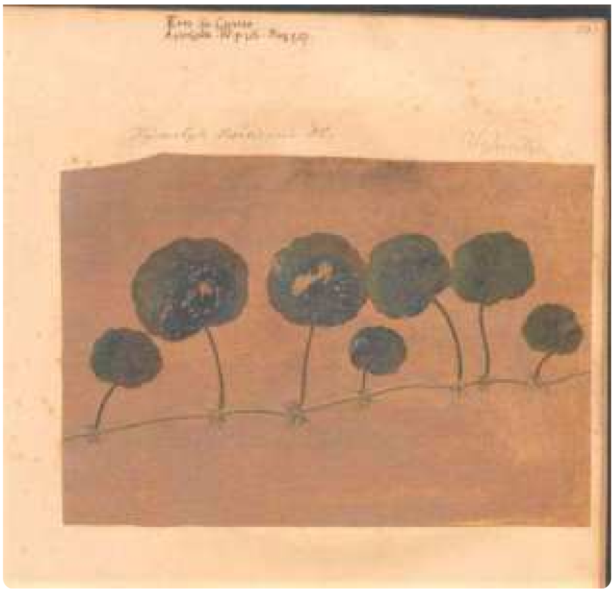

Theatrum Rerum Naturalium: 363

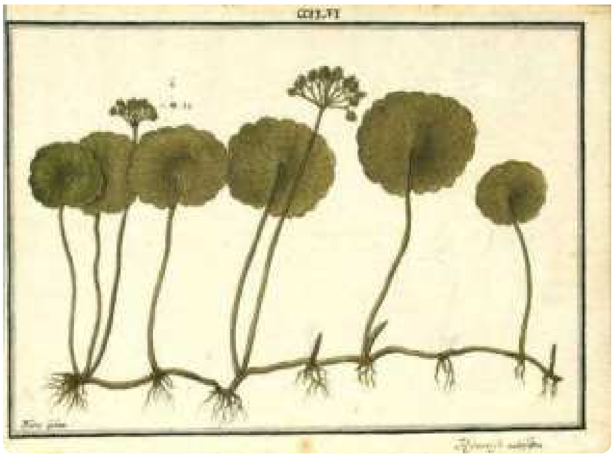

Drawings of the Royal Botanical Expedition to the Viceroyalty of Peru by Ruiz, H., Pavón, J. (1777: t. 246). Real Jardín Botánico, Madrid, Spain

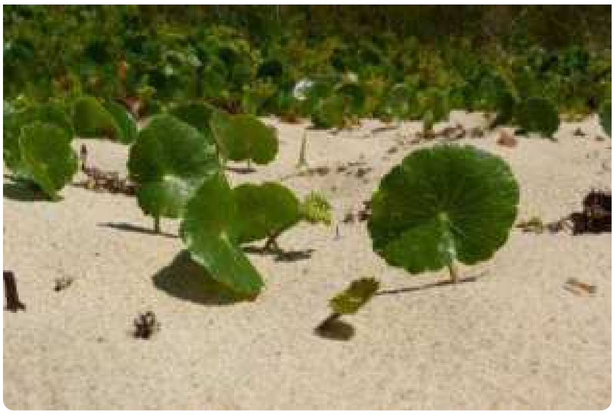

Habitat. "*H. bonariensis*" by dhobern (CC BY 2.0)

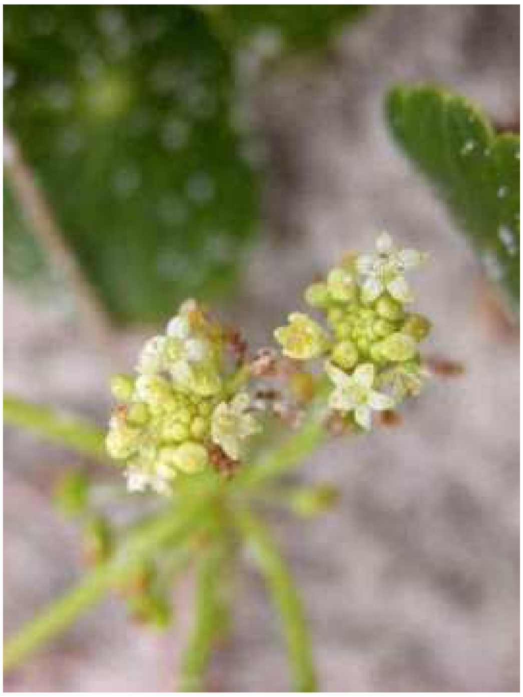

Flowers. "*H. bonariensis* 2809" by Bruce Kirchoff (CC BY 2.0)

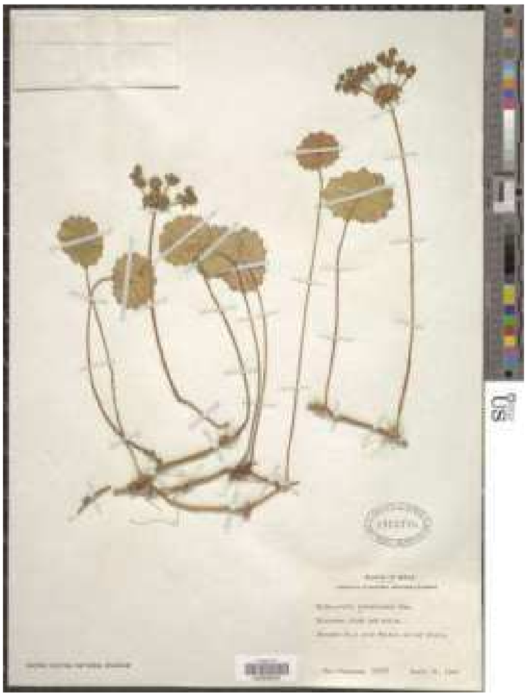

Specimen. "*H. bonariensis*" by E. Whitehouse -03069946- Smithsonian National Museum of Natural History (CC0 1.0)

Historiae Rerum Naturalium Brasiliae Marcgrave, 1648 Page number 28

Vernacular name(s) Caapomonga. Erva do vina

Species Plumbago zeylanica L.

Family Plumbaginaceae

Notes

The woodcut looks slightly similar to the *Theatrum* image, especially the leaves, in contrast to the *Libri principis* image. The watercolor in the *Libri Principis* bears some resemblance to the same species depicted in Eckhout's painting of the "Mameluca woman".

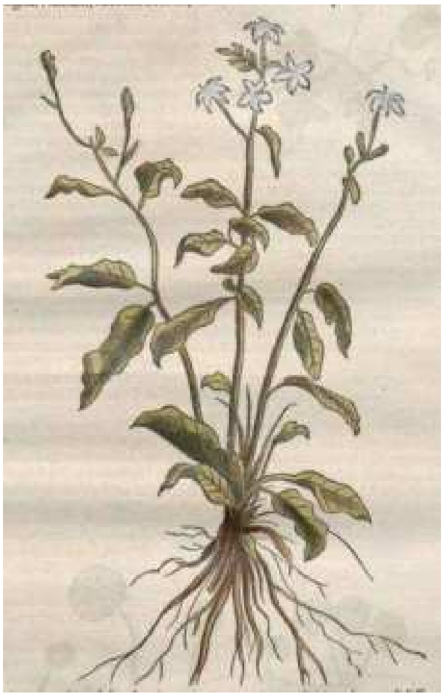

Historiae Plantarum – Herbis: 28

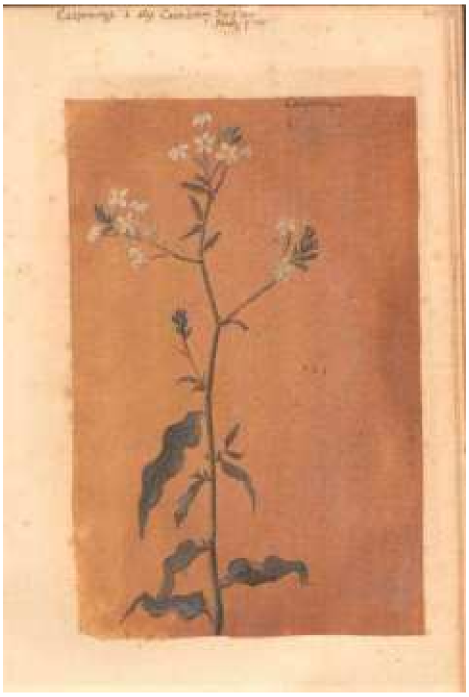

Theatrum Rerum Naturalium: 481

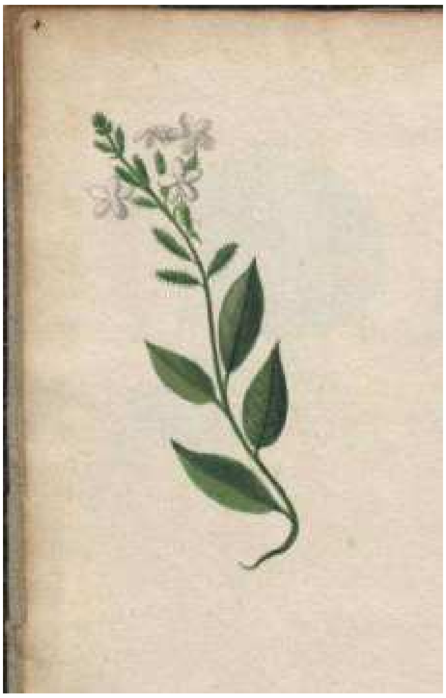

Libri Principis f. 4

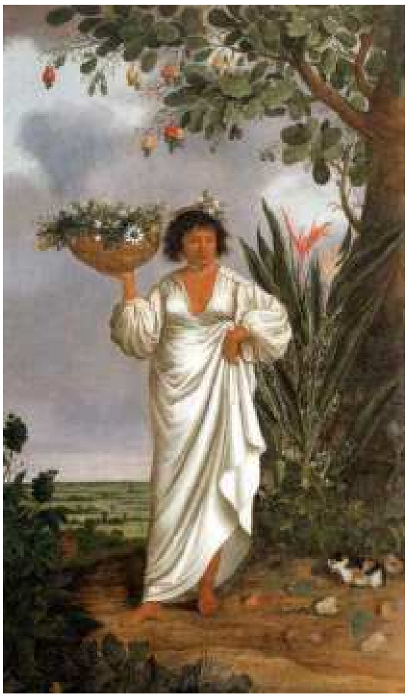

Portrait by Eckhout "Makeluka with basket of flowers", ca. 1641. National Museum of Copenhagen

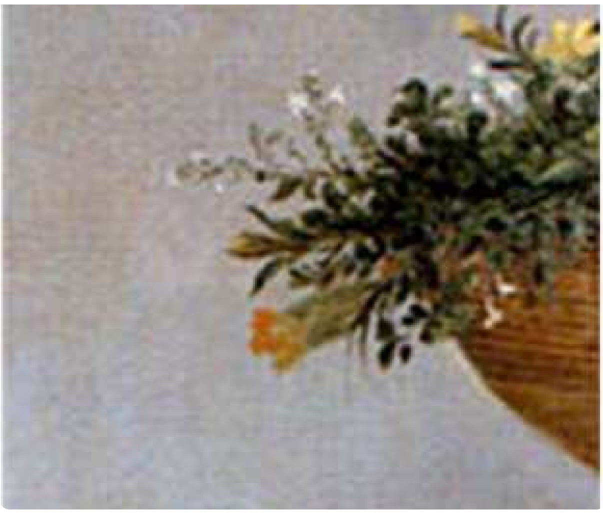

Close - up of Eckhout's painting, in which *P. zeylanica* is depicted as part of the floral arrangement in the basket

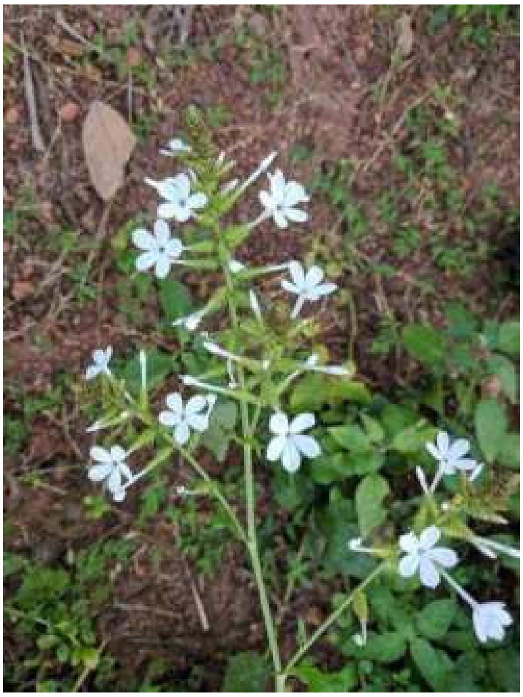

P. zeylanica in plantlife by Vengolis (CC-BY-4.0)

Historiae Rerum Naturalium Brasiliae — Marcgrave, 1648 — Page number 29

Vernacular name(s): Cara. Inhame de S. Thome. Quiquoaquicongo

Species: Dioscorea alata L.

Family: Dioscoreaceae

Notes

No similarities between the woodcut and the watercolor in the *Libri Principis*. The watercolor only portrays the tuber or yam while the woodcut shows the flowering plants and their tuber.

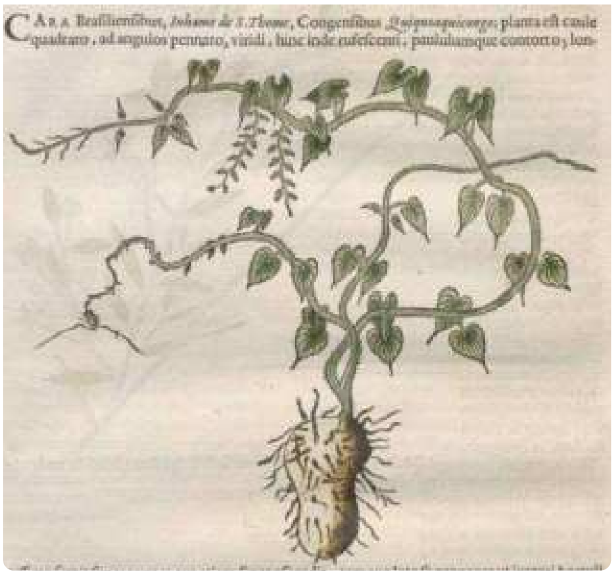

Historiae Plantarum – Herbis: 29

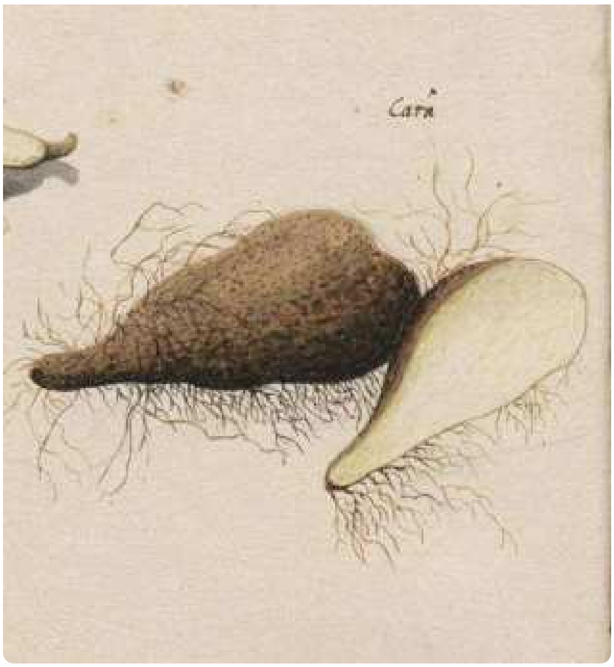

Libri Principis f. 87 [67]

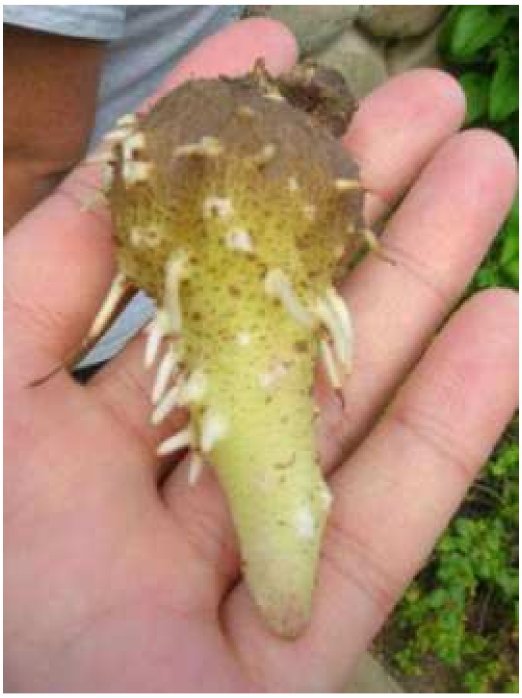

Yam or tuber of *D. alata*. Maui Nui Botanical Garden by Forest & Kim Starr (CC-BY-3.0)

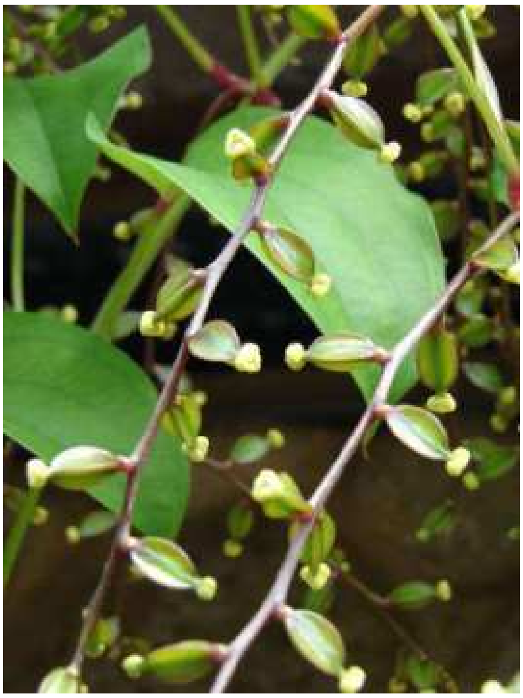

Flowers of *D. alata*. Maui Nui Botanical Garden by Forest and Kim Starr (CC BY 2.0)

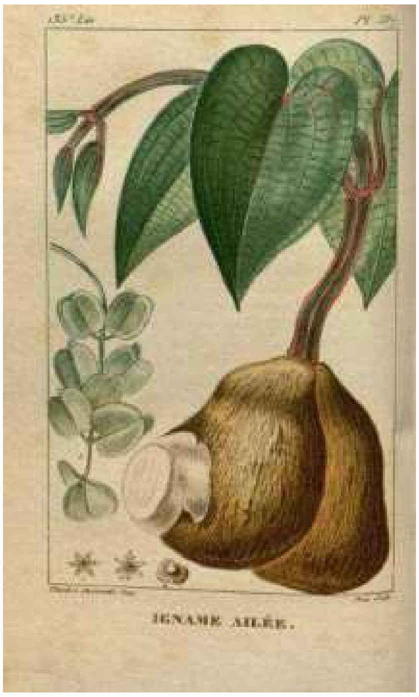

Flore [pittoresque et] médicale des Antilles by Descourtilz, M.E. (1829: Vol. VIII, t. 537). Missouri Botanical Garden, St. Louis, U.S.A.

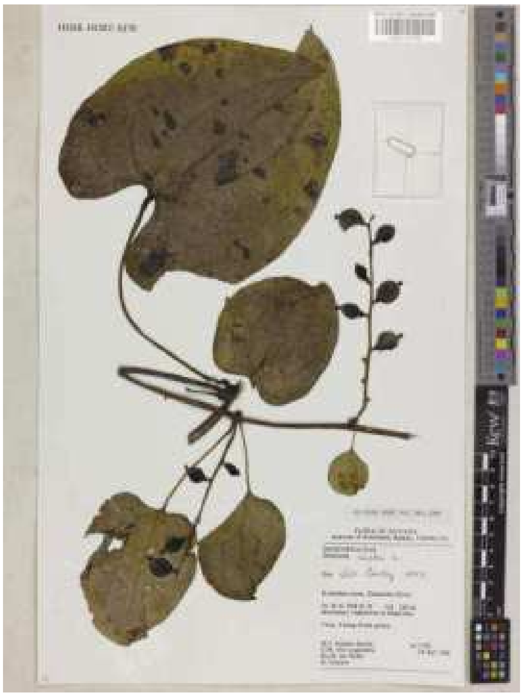

Specimen of *D. alata* from Kew's Herbarium - K001171729. Retrieved from Plants of the World Online

Historiae Rerum Naturalium Brasiliae Marcgrave, 1648 Page number 30a

Vernacular name(s) Camaranbaya. Reperitur hic & alia ejus species

Species Ludwigia octovalvis (Jacq.) P.H.Raven

Family Onagraceae

Notes

There are no strong similarities between the woodcut and the specimen, although both represent flowering branches.

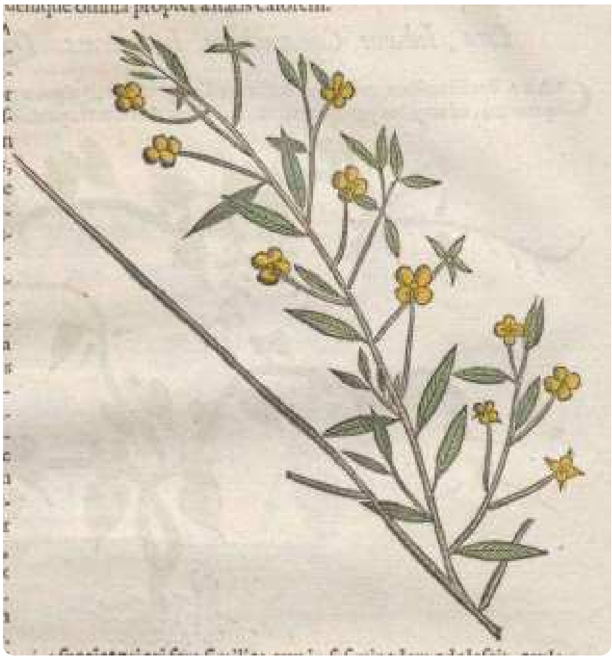

Historiae Plantarum – Herbis: 30a

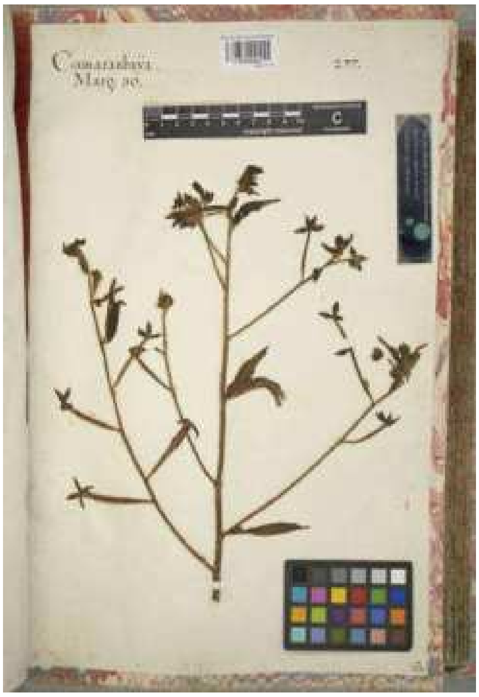

Marcgrave's herbarium: 62

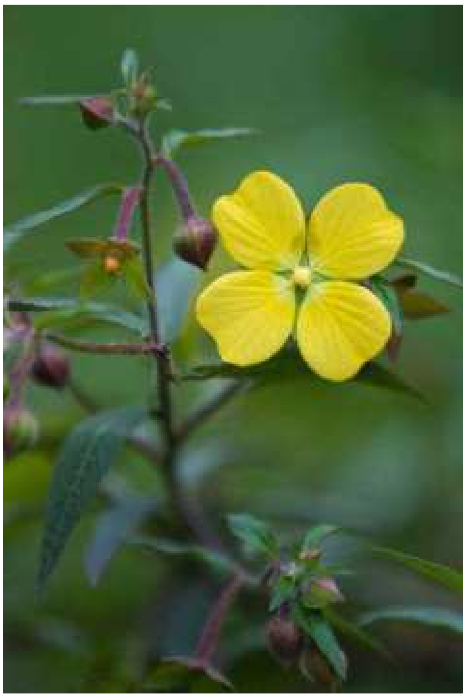

Flower. "*L. octovalvis* (girofle d'eau)" by Création CARAVEO (CC BY-ND 2.0)

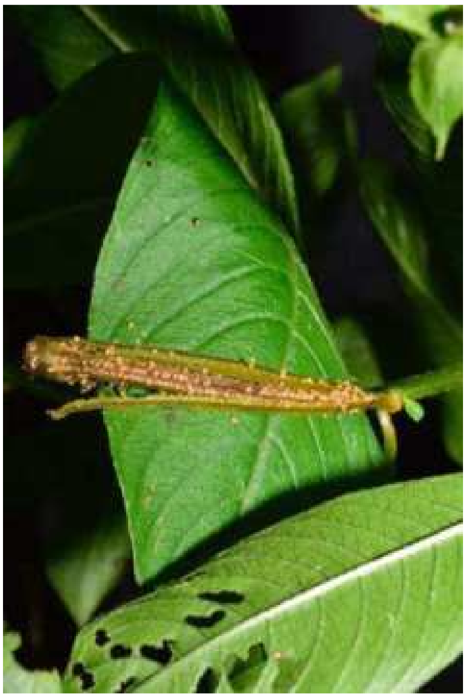

Fruit and seeds. "*L. octovalvis*" by Reinaldo Aguilar (CC BY-NC-SA 2.0)

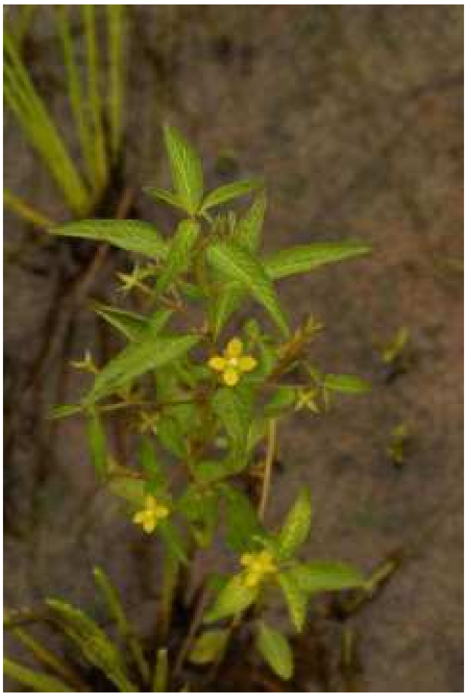

"*L. octovalvis* 110809-8814" by Tony Rodd (CC BY-NC-SA 2.0)

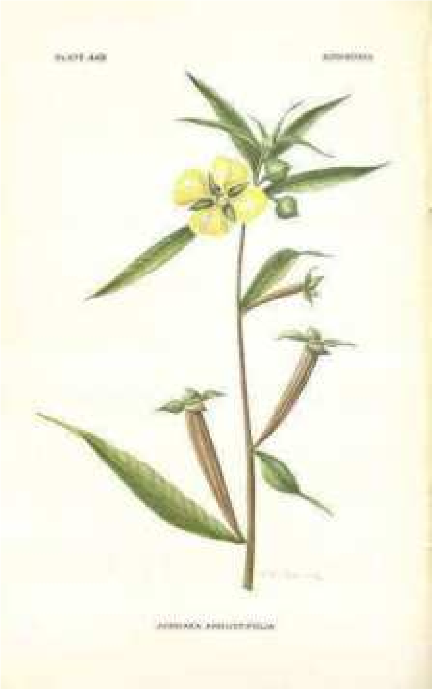

Engraving of *L. octovalvis* in *Addisonia* (1928: vol. 13, t. 442). Missouri Botanical Garden, St. Louis, U.S.A.

Historiae Rerum Naturalium Brasiliae Marcgrave, 1648 Page number 30b

Vernacular name(s) Planta

Species Romanoa cf. tamnoides (A.Juss.) Radcl.-Sm.

Family Euphorbiaceae

Notes

We did not find any correspondence between this woodcut and the contemporary or older sources.

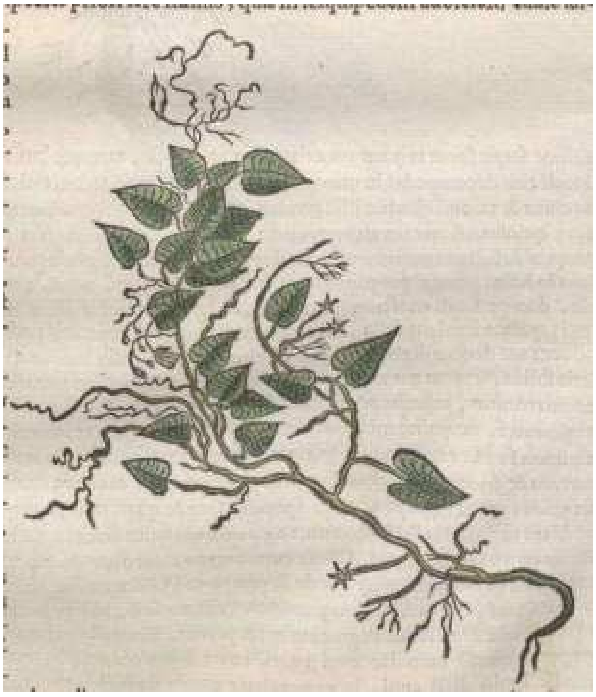

Historiae Plantarum – Herbis: 30b

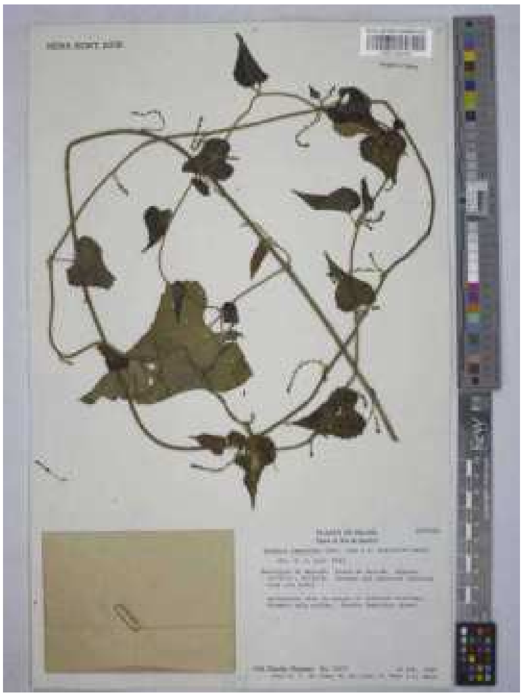

R. tamnoides specimen from Kew's Herbarium - K001205036. Retrieved from Plants of the World Online

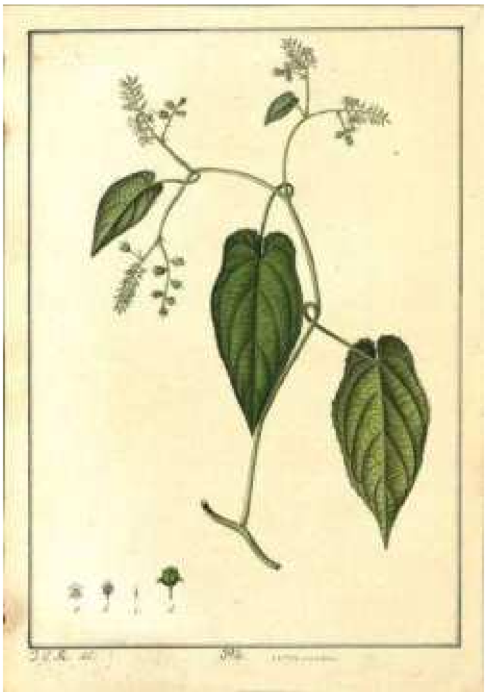

Drawings of the Royal Botanical Expedition to the Viceroyalty of Peru by Ruiz, H., Pavón, J. (1777). Real Jardín Botánico, Madrid, Spain

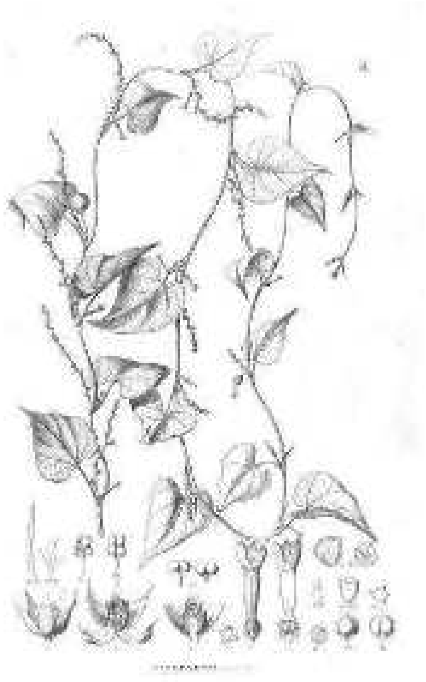

Engraving of *R. tamnoides* in Martius, C.F.P. von, Eichler, A.G., Urban, I., *Flora Brasiliensis* (1873-1874) Vol. 11 (2): 51

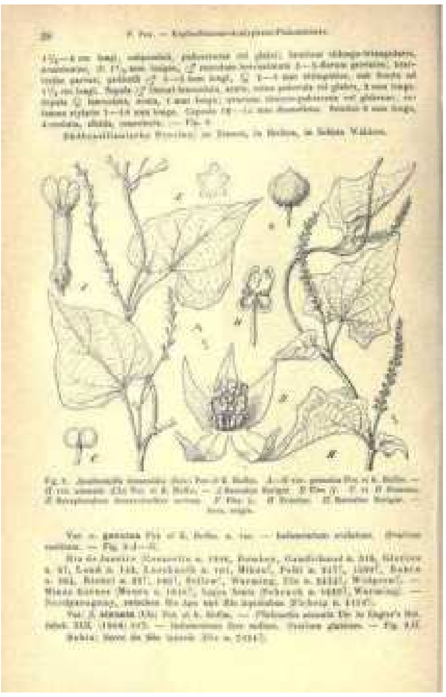

Drawing of *R. tamnoides* in *Pflanzenreich* by Engler, H.G.A. (1919: Vol. 147). Missouri Botanical Garden, St. Louis, U.S.A.

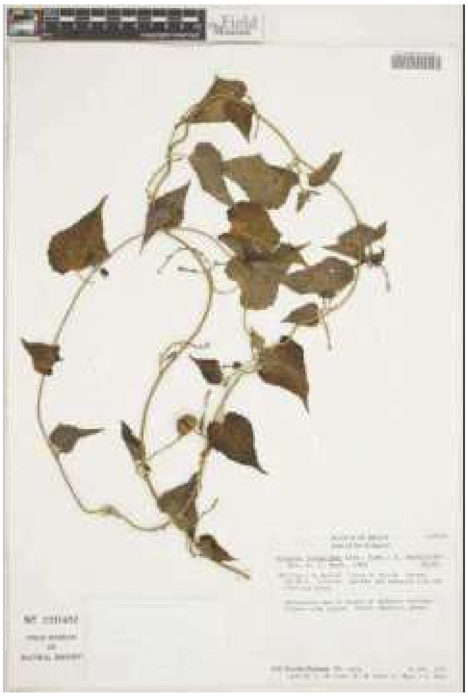

Specimen of *R. tamnoides*. The Field Museum of Natural History, Brazil -V0191200F- Retrieved from GBIF.org

Historiae Rerum Naturalium Brasiliae | Marcgrave, 1648 | Page number 31a

Vernacular name(s) Quingombo. Quillobo

Species Abelmoschus esculentus (L.) Moench

Family Malvaceae

Notes

We did not find any correspondence between this woodcut and the contemporary or older sources

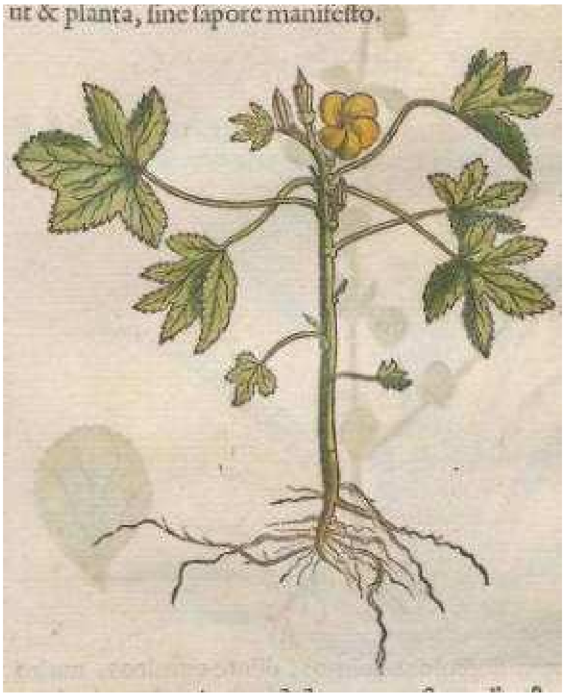

Historiae Plantarum – Herbis: 31a

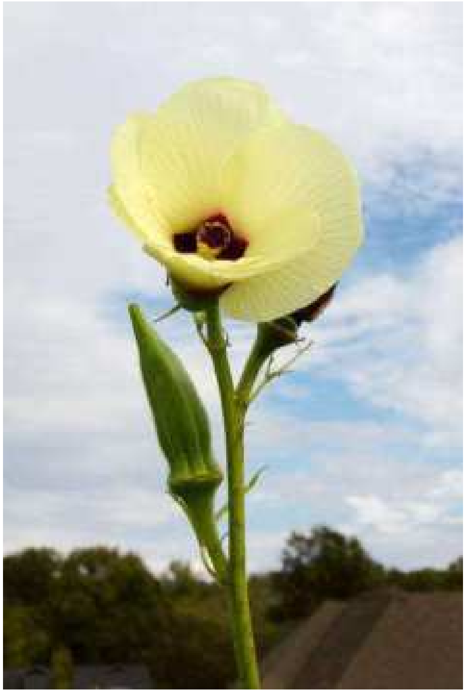

"Okra (*A. esculentus*)" by Ted LaBar (CC BY-NC 2.0)

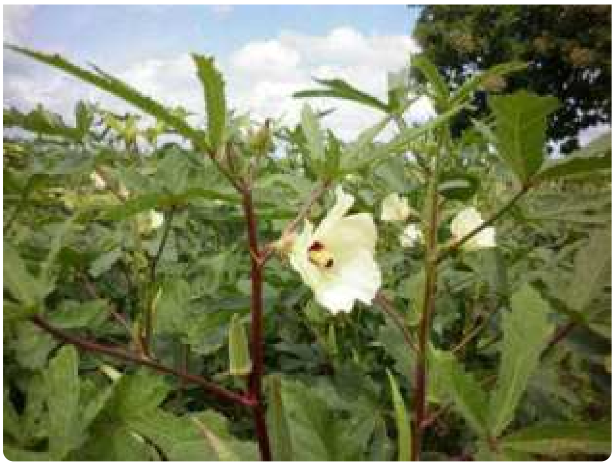

"Okra (*A. esculentus*).JPG" by Delince.J (CC BY-SA 3.0)

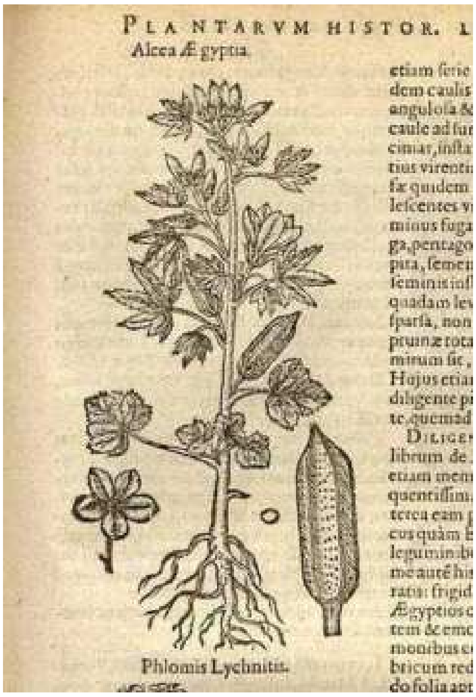

Woodcut of *A. esculentus* in *Rariorum plantarum historia* by Clusius (1601: Vol. II, p. 27)

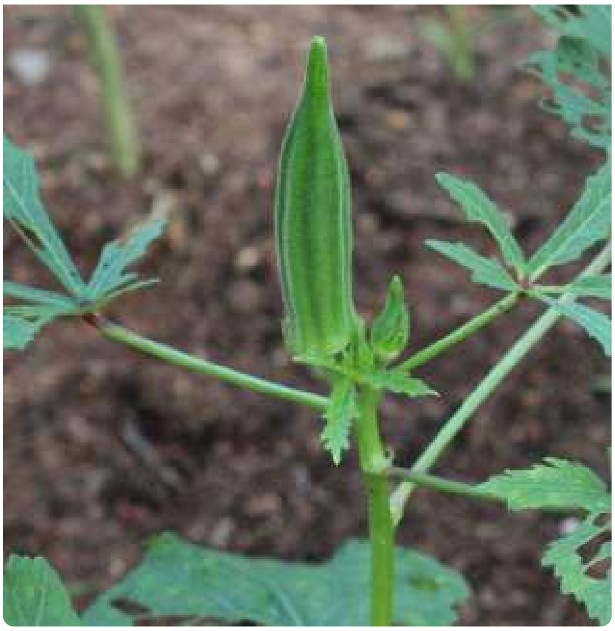

Fruits- "*A. esculentus*" by Cerlin Ng (CC BY-NC-SA 2.0)

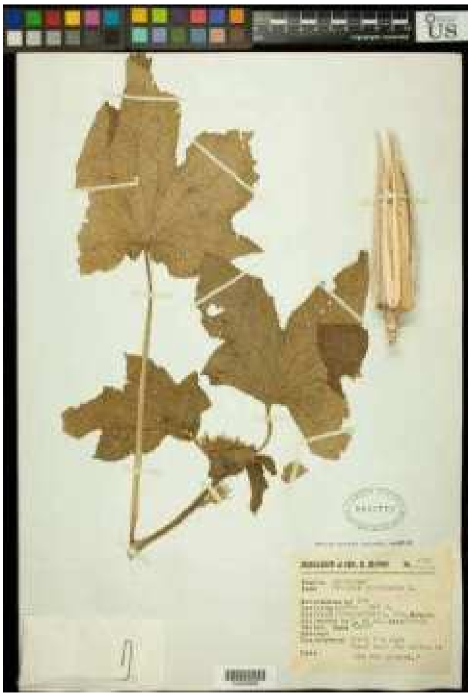

Specimen. "*A. esculentus*" by George B. Hinton - 01222652- Smithsonian National Museum of Natural History (CC0 1.0)

Historiae Rerum Naturalium Brasiliae | Marcgrave, 1648 | Page number 31b

Vernacular name(s) Planta

Species Gnaphalium cf. polycaulon Pers.

Family Asteraceae

Notes

We did not find any correspondence between this woodcut and the contemporary or older sources.

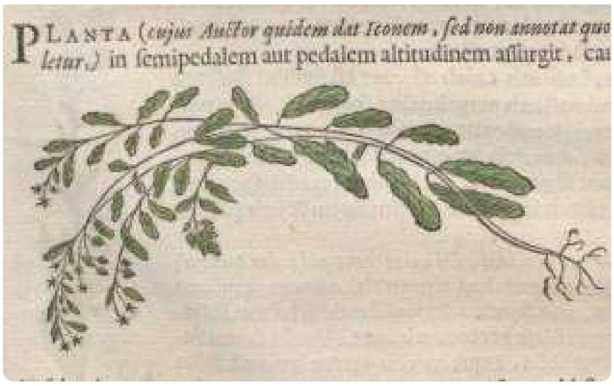

Historiae Plantarum – Herbis: 31b

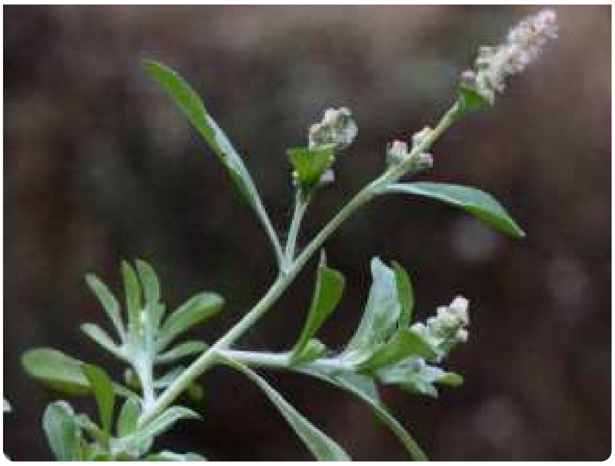

"*Gnaphalium* cf. *polycaulon* Pers." by Dinesh Valke (CC BY-SA 2.0)

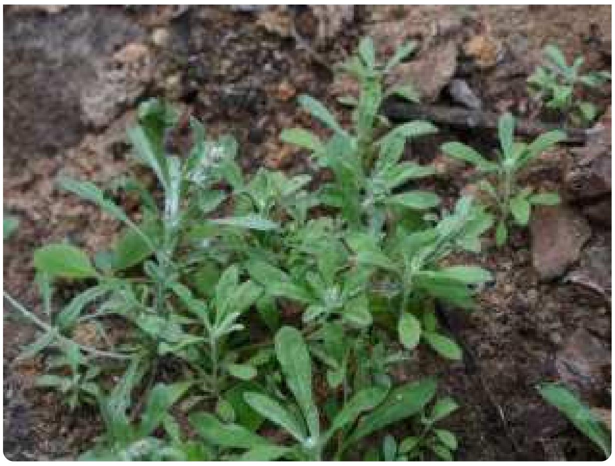

"*Gnaphalium* cf. *polycaulon* Pers." by Dinesh Valke (CC BY-SA 2.0)

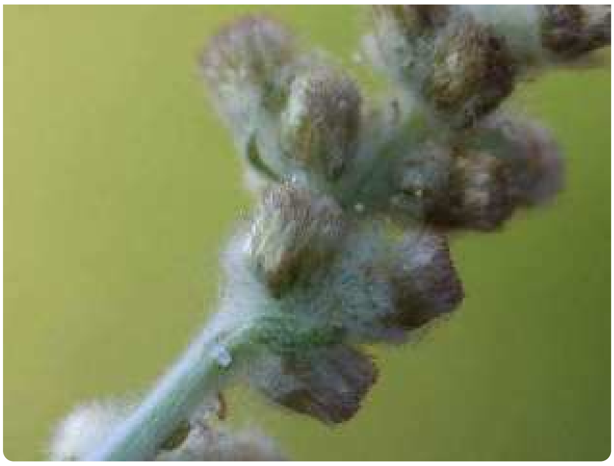

"*G. polycaulon* Pers." by Dinesh Valke (CC BY-SA 2.0)

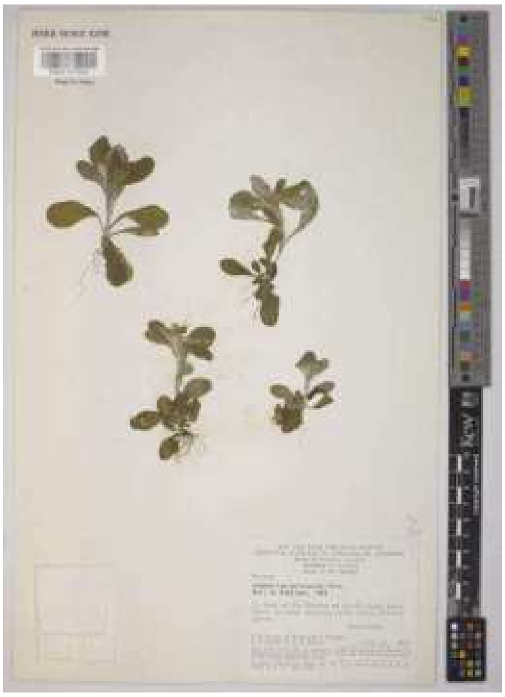

Specimen of *G. polycaulon* from Kew's Herbarium - K001107686. Retrieved from Plants of the World Online

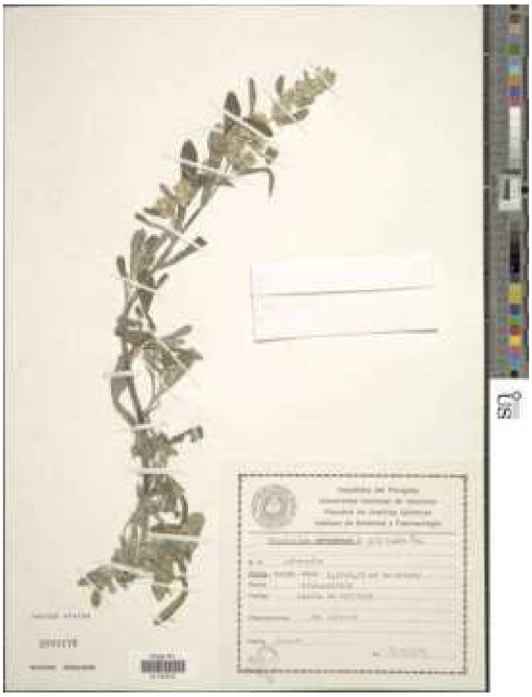

Specimen. "*G. polycaulon*." -01737070- Smithsonian National Museum of Natural History (CC0 1.0)

Historiae Rerum Naturalium Brasiliae | Marcgrave, 1648 | Page number 32a

Vernacular name(s) Caa ataya. Erva de purga de San Paez

Species Lindernia diffusa (L.) Wettst.

Family Linderniaceae

Notes

The woodcut looks moderately similar to the *Theatrum* illustration.

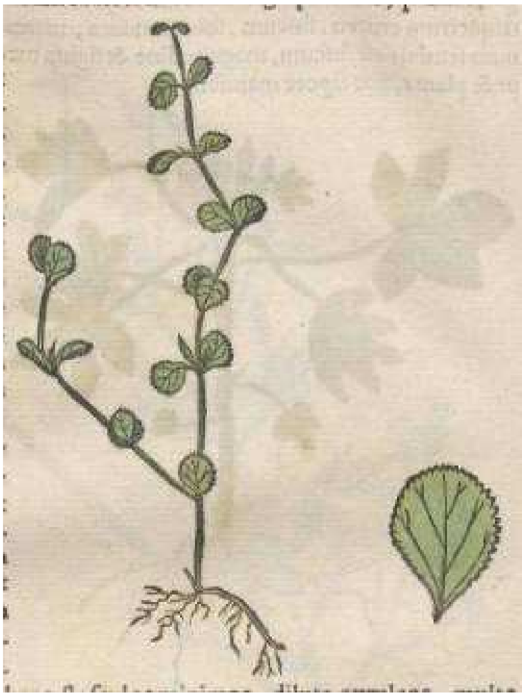

Historiae Plantarum – Herbis: 32a

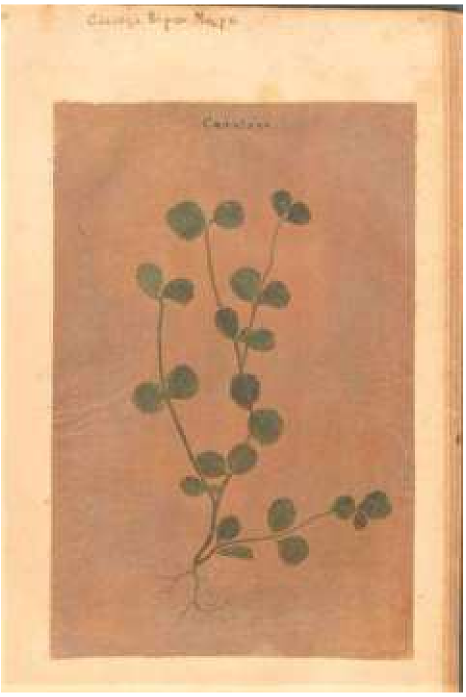

Theatrum Rerum Naturalium: 459

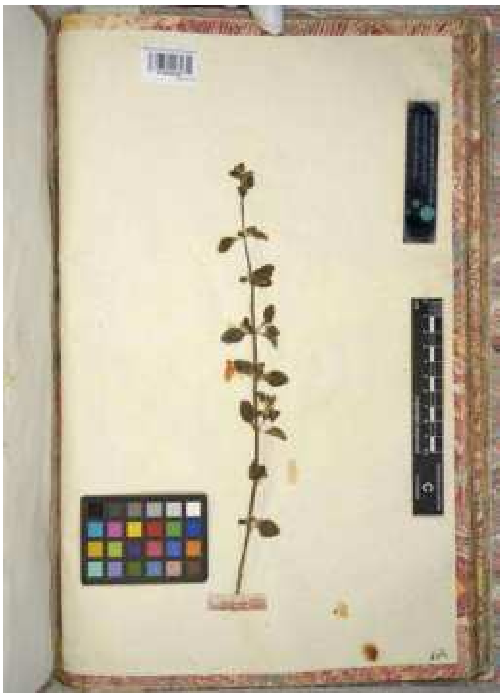

Marcgrave's herbarium: 167

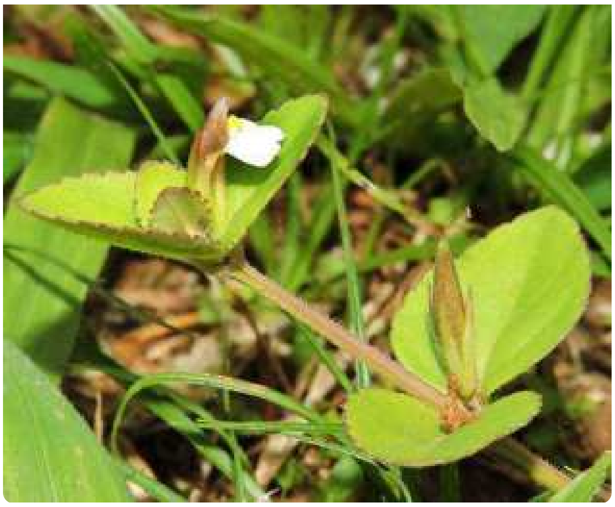

Flowering shoot. "*L. diffusa*" by Barry Hammel (CC BY-NC-SA 2.0)

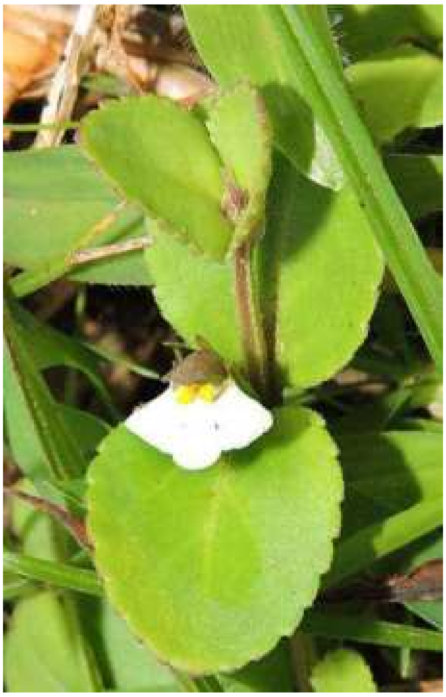

Flower and leaves. "*L. diffusa*" by Barry Hammel (CC BY-NC-SA 2.0)

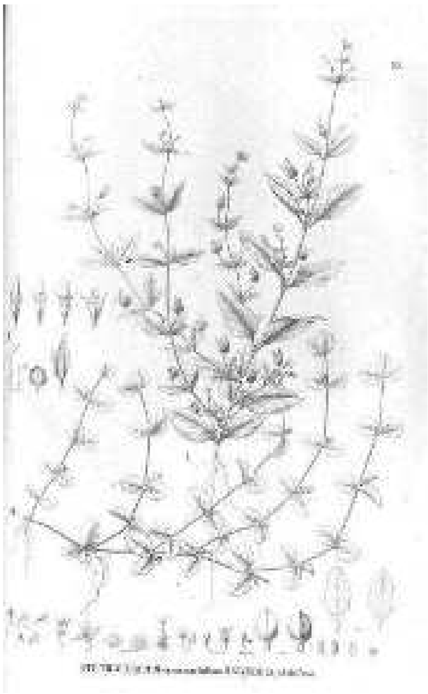

Engraving of *L. diffusa* in Martius, C.F.P. von, Eichler, A.G., Urban, I., *Flora Brasiliensis* (1857-1864) Vol. 8 (1): 55

Historiae Rerum Naturalium Brasiliae | Marcgrave, 1648 | Page number 32b

Vernacular name(s) Herba

Species Unknown

Family Unknown

Notes

We did not find any correspondence between this woodcut and the contemporary or older sources. We could not identify the plant represented by the woodcut, hence we could not cross-reference it with the visual sources. Accurate identification of such plant will facilitate this analysis and provide us with more insights about its origin.

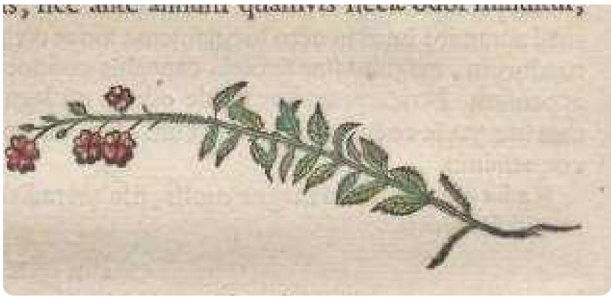

Historiae Plantarum – Herbis: 32b

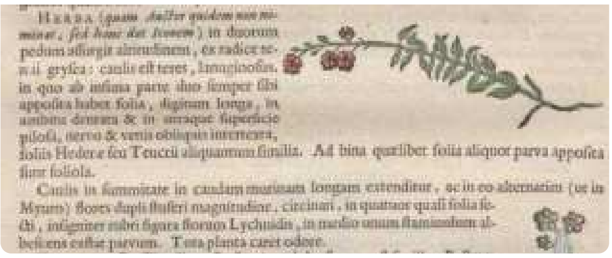

HERBA (*quam Auctor quidem non nominat, sed hanc dat Iconem*) in duorum pedum assurgit altitudinem, ex radice [illegible] gryfea: caulis est teres, lanuginosus, in quo ab infima parte duo semper sibi apposita habet folia, digitum longa, in ambitu dentata & in utraque superficie pilosa, nervo & venis obliquis intertexta, foliis Hederæ seu Teucrii aliquatenus similia. Ad bina quælibet folia aliquot parva apposita sunt foliola.

Caulis in summitate in caudam murinam longam extenditur, ac in eo alternatim (ut in Myuro) flores [illegible] magnitudine, circinati, in quatuor quasi folia secti, insigniter rubri figura florum Lychnidis, in medio unum stamineum albescens [illegible] parvum. Tota planta caret odore.

HNB (Marcgrave 1648: 32) Latin edition

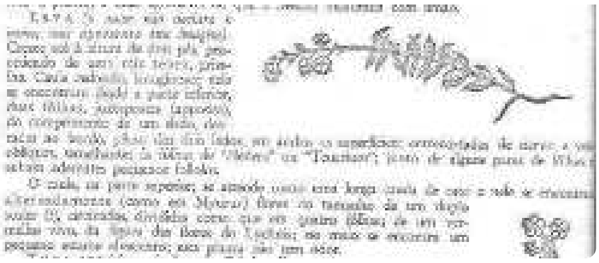

HNB (1942 [1648]) Portuguese edition

"It grows to the height of two feet, proceeding from a tender, greying root. Round, lanuginose stem; in it are found from the bottom, two leaves, juxtaposed (opposite), the length of a finger, notched on the edge, hairy on both sides, on both surfaces; interspersed with oblique nerves and veins, similar to the leaves of *Hedera* or *Teucrium*; small leaflets are attached to some pairs of leaves. The stem, at the top, extends like a long rat's tail and alternately (as in *Myurus*) flowers are the size of a double stuffer (?), curved, divided as if in four leaves; bright red, the figure of the *Lychnis* flowers; in the middle is a small white stamen; this plant has no odour"

English translation, by M. Alcantara-Rodriguez

Historiae Rerum Naturalium Brasiliae Marcgrave, 1648 Page number 32c

Vernacular name(s) Herba

Species Unknown

Family Unknown

Notes

We did not find any correspondence between this woodcut and the contemporary or older sources. We could not identify the plant represented by the woodcut, hence we could not cross-reference it with the visual sources. Accurate identification of such plant will facilitate this analysis and provide us with more insights about its origin.

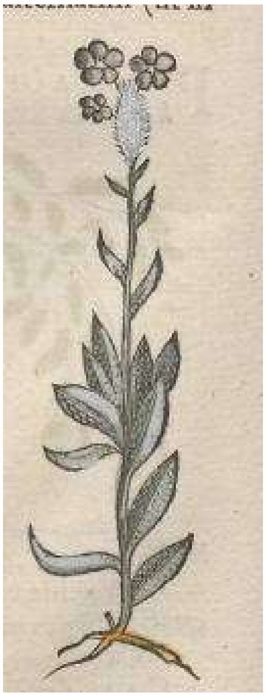

Historiae Plantarum – Herbis: 32c

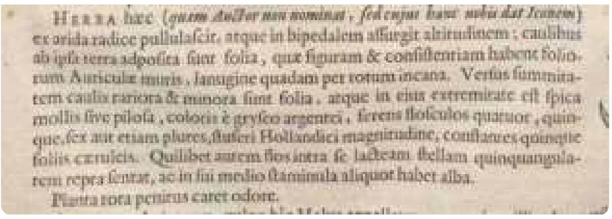

HERBA hæc (*quam Auctor non nominat, ſed cujus hanc nobis dat Iconem*) ex arida radice pullulaſcit, atque in bipedalem aſſurgit altitudinem; caulibus ab ipſa terra adpoſita ſunt folia, quæ figuram & conſiſtentiam habent foliorum Auriculæ muris, lanugine quadam per totum incana. Verſus ſummitatem caulis rariora & minora ſunt folia, atque in ejus extremitate eſt ſpica mollis ſive piloſa, coloris è gryſeo argentei, ferens floſculos quatuor, quinque, ſex aut etiam plures, ſtuferi Hollandici magnitudine, conſtantes quinque foliis cæruleis. Quilibet autem flos intra ſe lacteam ſtellam quinquangularem repræſentat, ac in ſui medio ſtaminula aliquot habet alba.

Planta tota penitus caret odore.

HNB (Marcgrave 1648: 32) Latin edition

mais verdes.

E r v a (o autor não menciona o nome, mas dá sua imagem). Pululando de uma raiz árida, cresce até à altura de dois pés; nos caules, desde a flor da terra, se acham fixadas flores, que têm a figura e consistência das fôlhas da orelha-de-rato com uma certa lanugem esbranquiçada em tôda sua superfície. Para o ápice do caule, escasseiam as fôlhas e se tornam menores; no ponta se acha uma espiga mole ou pilosa, de côr argêntea [illegible], que produz quatro, cinco, seis ou mais flósculos, do tamanho do [illegible] (?) holandês, formados por cinco fôlhas cerúleas. Cada flor representa uma estrêla láctea de cinco ângulos, tendo no meio uns pequenos estames (staminula) brancos; esta planta não tem odor.

HNB (1942 [1648]) Portuguese edition

"Swarming from an arid root, it grows to the height of two feet; to the stems, from the flower of the earth, flowers are attached, which have the figure and consistency of the leaves of *oreiha-do-rato* with a certain whitish fluff on its entire surface. For the apex of the stem, the leaves are rare and become smaller; at the tip is a soft or hairy ear, of a grey silver color, which produces four, five, six or more florets, the size of Dutch stuffer (?), formed by five cerulean leaves. Each flower represents a milky star with five angles, with small white stamens (stamineal) in the middle; this plant has no odor"

English translation, by M. Alcantara-Rodriguez

Historiae Rerum Naturalium Brasiliae | Marcgrave, 1648 | Page number 33

Vernacular name(s) Nana. Ananas. Iajama.

Species Ananas comosus (L.) Merr.

Family Bromeliaceae

Notes

The woodcut looks slightly similar to the *Theatrum* illustration. There is no resemblance to the herbarium specimen as this one shows only one leaf. There is a pencil lead in In De Laet's manuscript, which was likely the basis for the woodcut (exact but reversed).

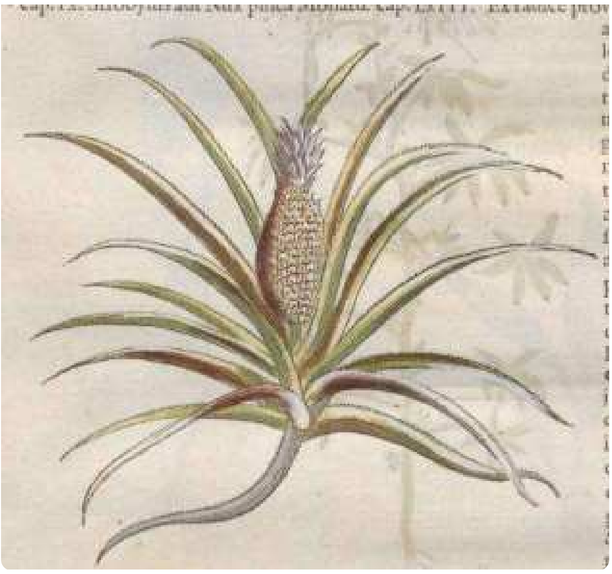

Historiae Plantarum – Herbis: 33

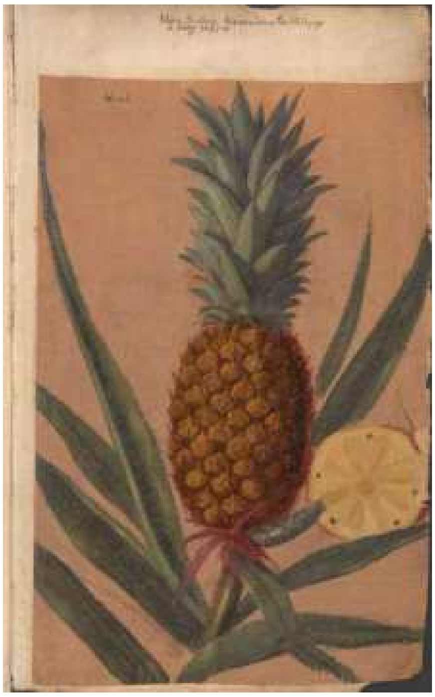

Theatrum Rerum Naturalium: 1

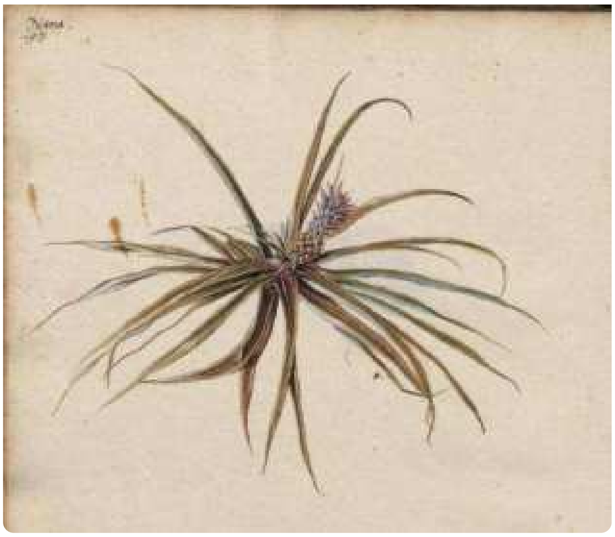

Libri Principis f. 73 [53]

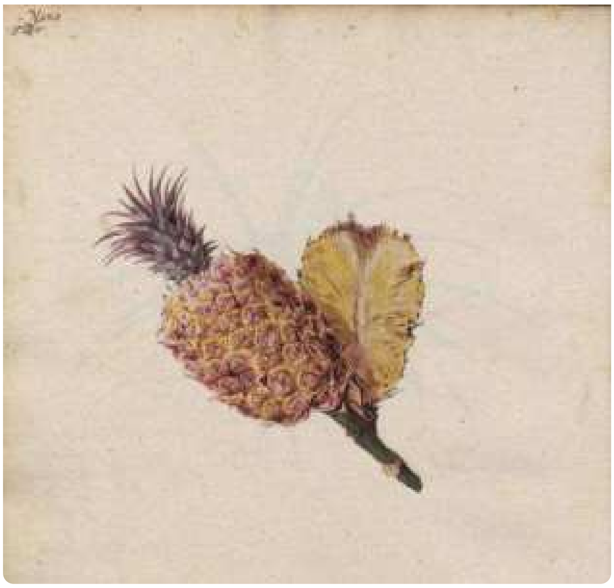

Libri Principis f. 75 [55]

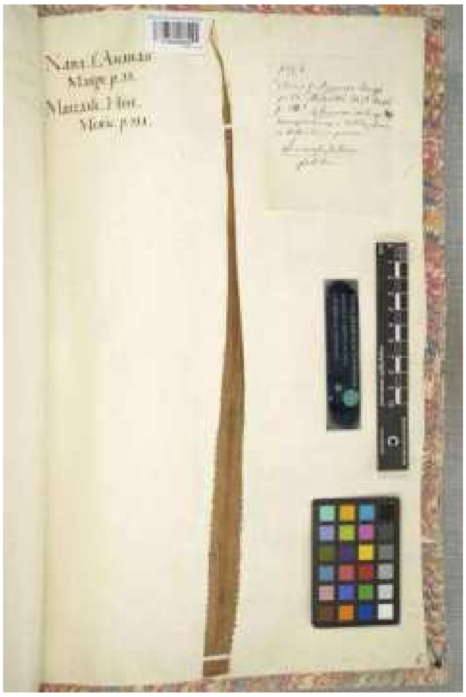

Marcgrave's herbarium: 6

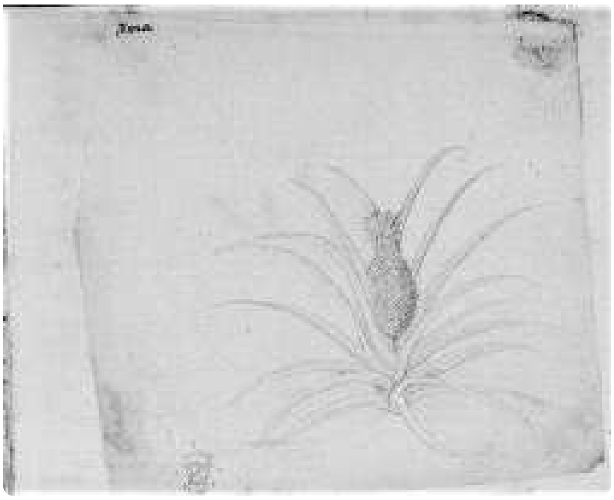

Pencil drawing of *C. tatuya* in De Laet's manuscript:
Sloane MS 1554, f. 15v

Historiae Rerum Naturalium Brasiliae | Marcgrave, 1648 | Page number 34

Vernacular name(s) Tareriaya

Species Cleome spinosa Jacq.

Family Fabaceae

Notes

We did not find any correspondence between this woodcut and the contemporary or older sources.

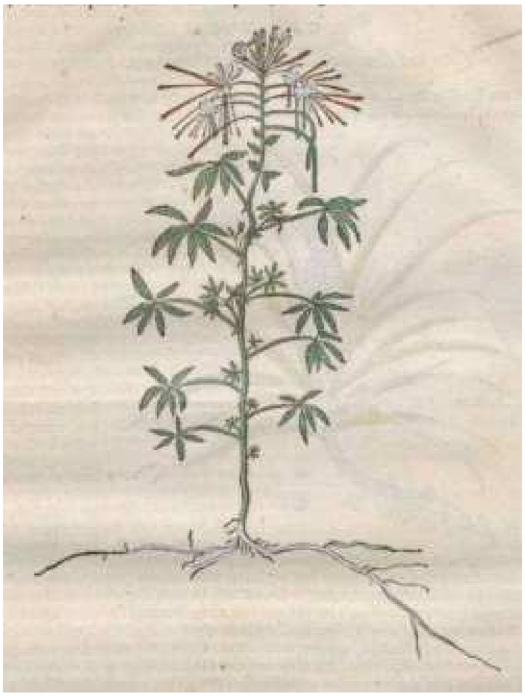

Historiae Plantarum – Herbis: 34

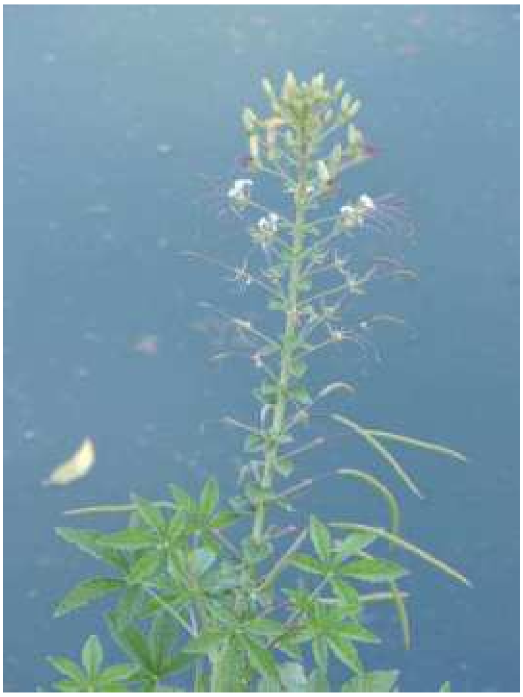

"*C. spinosa* Jacq." by Barry Hammel (CC BY-NC-SA 2.0)

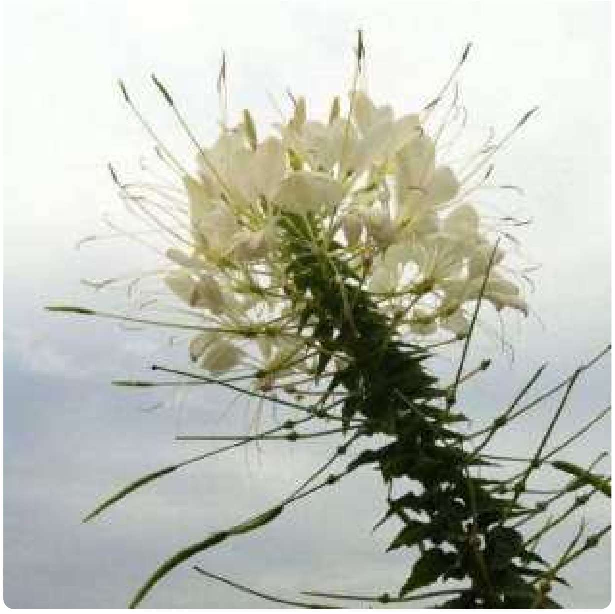

"*C. spinosa* 'Spinnenpflanze' #buga2011" by ThoMo1969 (BCC BY 2.0.)

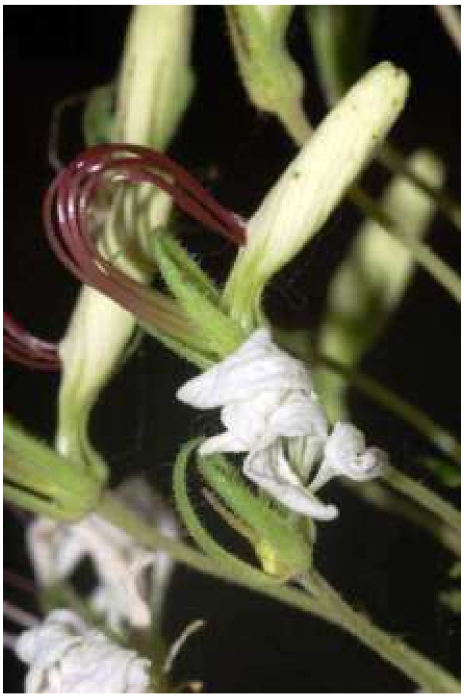

Flower. "*C. spinosa*" by Barry Hammel (CC BY-NC-SA 2.0)

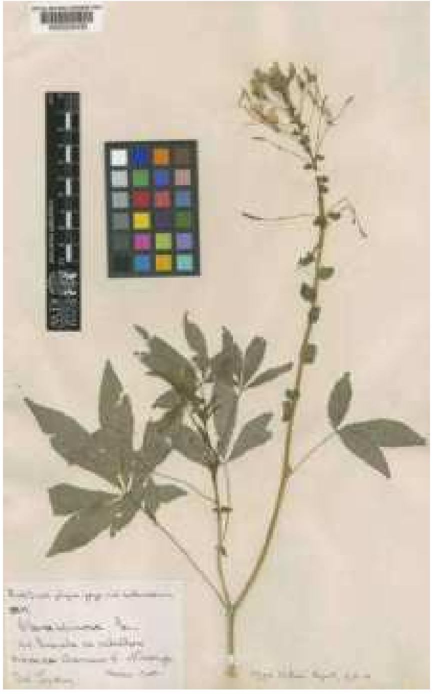

Specimen of *C. spinosa* from Kew's Herbarium - K000220438. Retrieved from Plants of the World Online

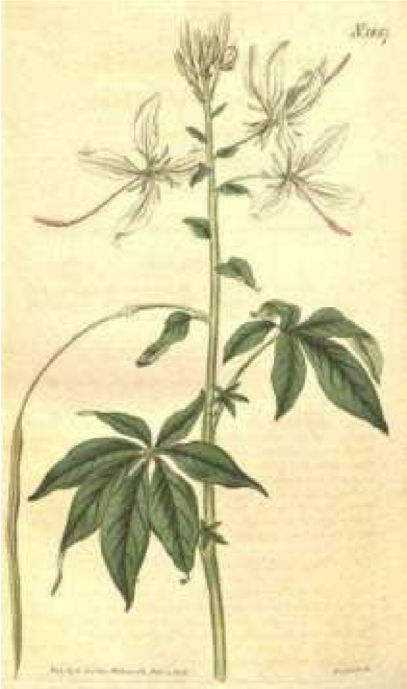

Botanical Magazine by Curtis, W. (1816: Vol. 43, t. 1857). Missouri Botanical Garden, St. Louis, U.S.A.

Historiae Rerum Naturalium Brasiliae | Marcgrave, 1648 | Page number 35a

Vernacular name(s) Arapabaca

Species Spigelia anthelmia L.

Family Loganiaceae

Notes

The woodcut looks slightly similar to the *Theatrum* illustration and strongly similar to the herbarium specimen. Hence, it was likely made after the latter, or both the oil painting and the woodcut were made after the specimen.

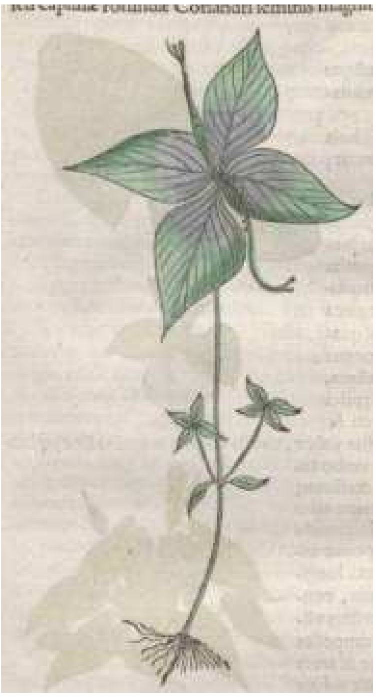

Historiae Plantarum – Herbis: 35a

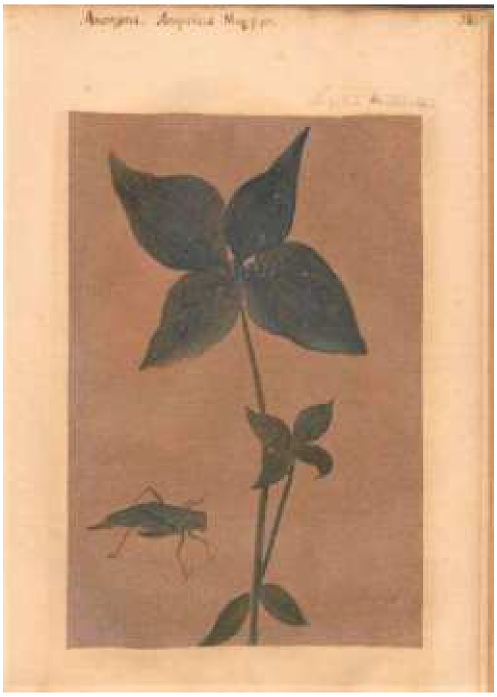

Theatrum Rerum Naturalium: 323

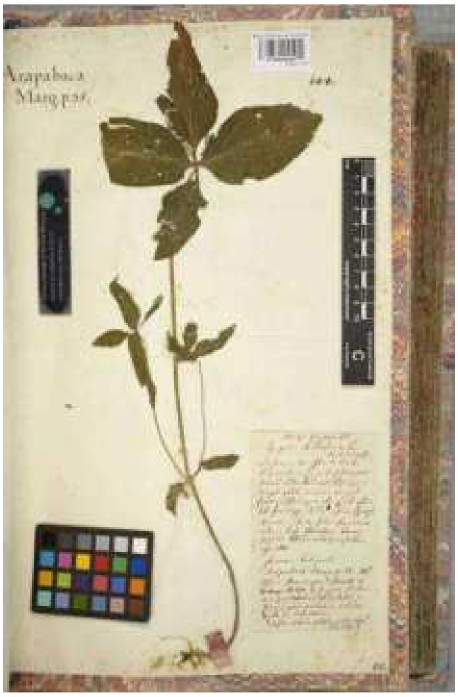

Marcgrave's herbarium: 55

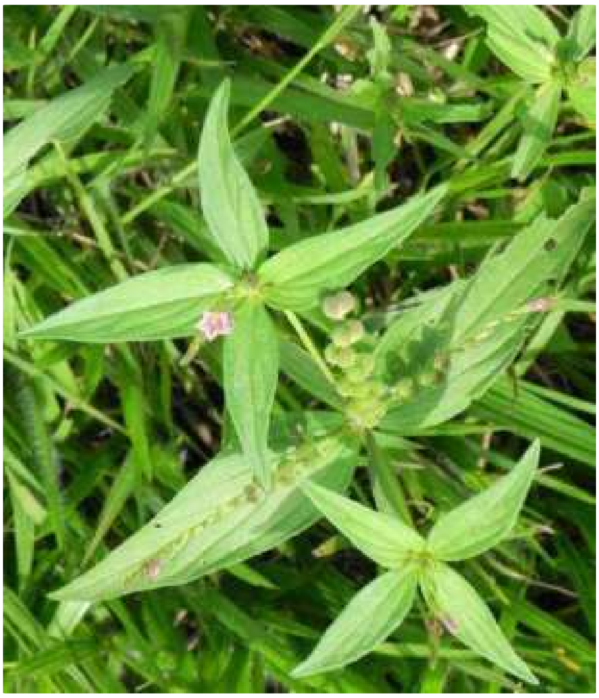

"Flickr - João de Deus Medeiros - *Spigelia anthelmia* (1).jpg" by João Medeiros (CC BY 2.0)

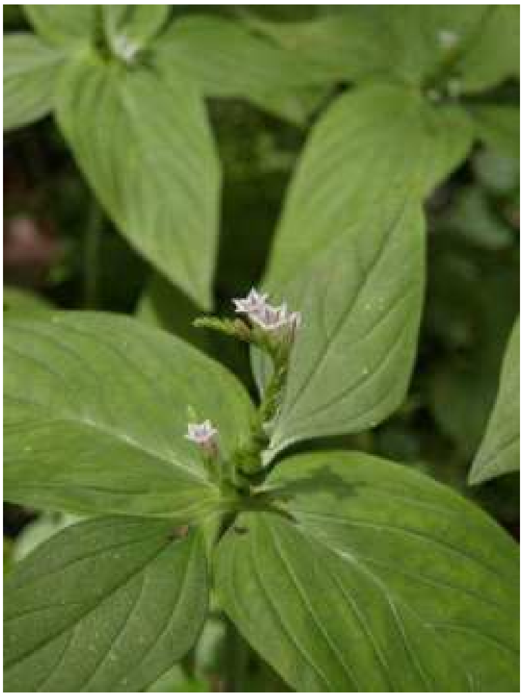

"*Spigelia anthelmia* 5" by Scott Zona (CC BY-NC 2.0)

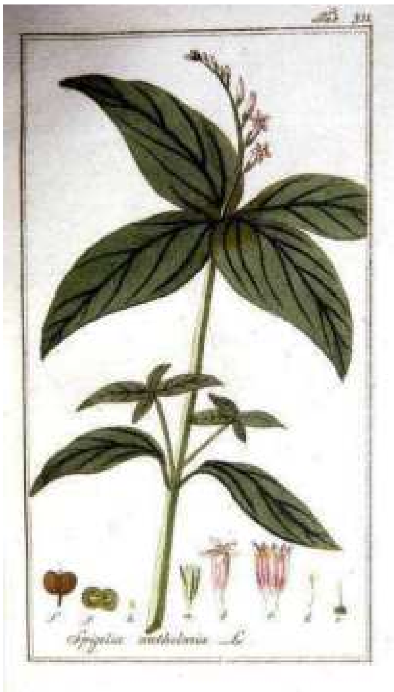

Engraving of *S. anthelmia* in Afbeeldingen der artseny-gewassen by Zorn, J., Oskamp, D.L (1800: Vol. VI, t. 511). www.BioLib.de

Historiae Rerum Naturalium Brasiliae Marcgrave, 1648 Page number 35b

Vernacular name(s) Urucatu

Species Catasetum macrocarpum Rich. ex Kunth

Family Orchidaceae

Notes

We did not find any correspondence between this woodcut and the contemporary or older sources.

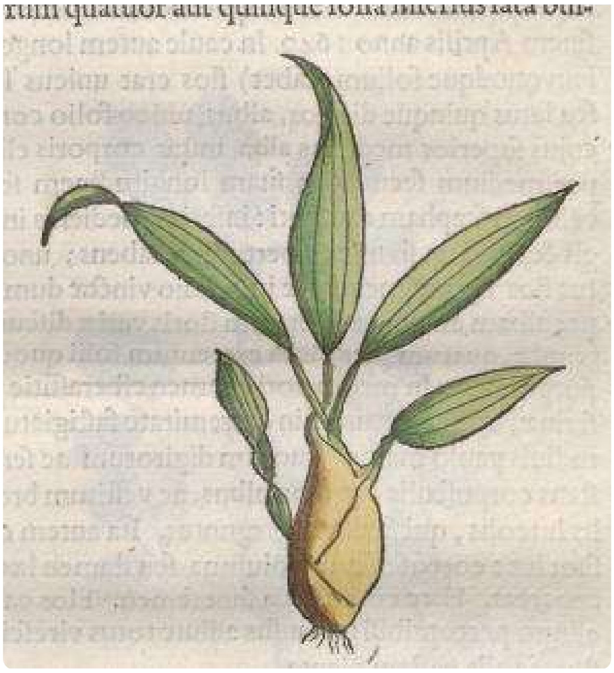

Historiae Plantarum – Herbis: 35b

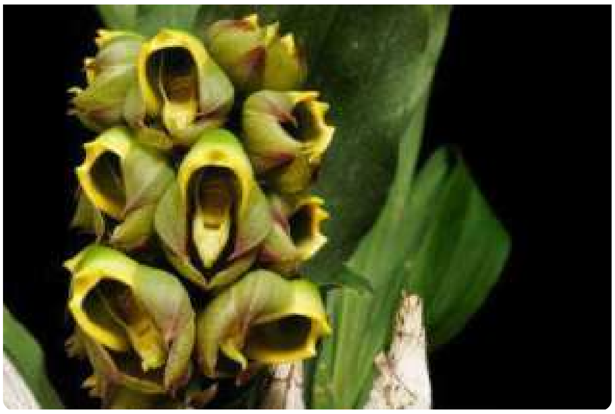

"*C. macrocarpum* (Flor Masculina)" by José Pestana (CC BY-NC-SA 2.0)

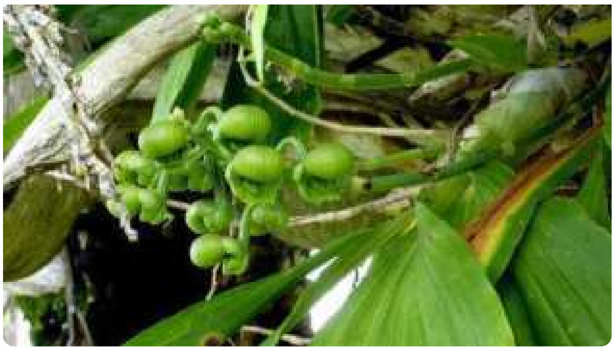

"Habit of *C. macrocarpum*" by Alex Popovkin, Bahia, Brazil (CC BY-NC-SA 2.0)

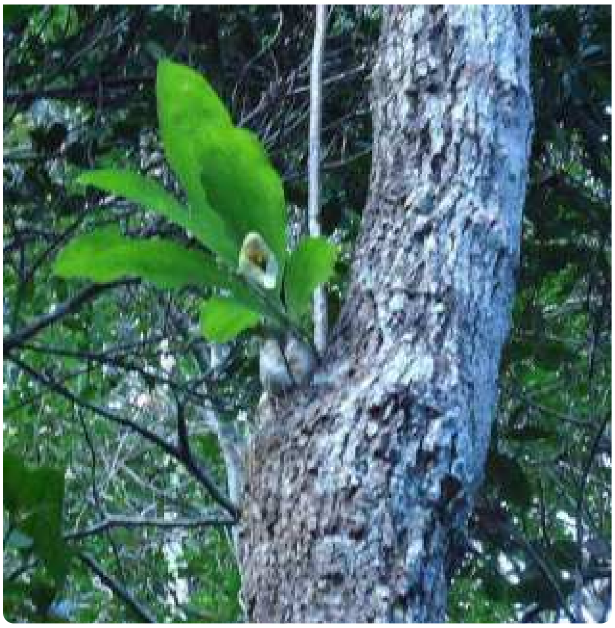

"*C. macrocarpum*" by Alex Popovkin, Bahia, Brazil (CC BY-NC-SA 2.0)

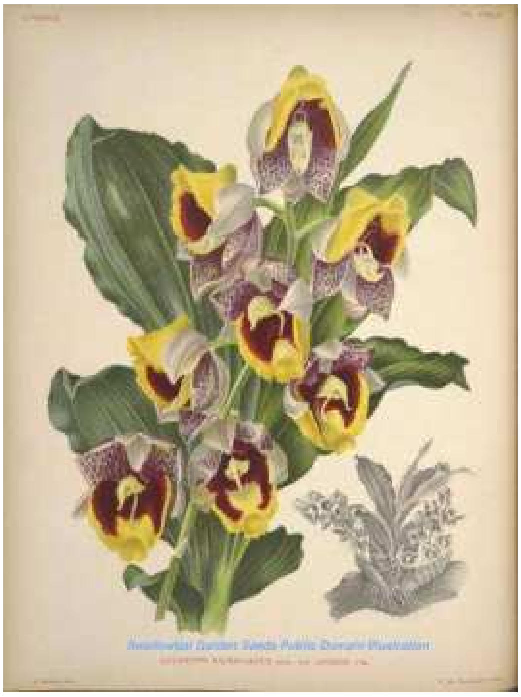

Illustration of *Catasetum macrocarpum* in *Iconography of orchids* vol. 8 (1894)" by Swallowtail Garden Seeds (CC BY 2.0)

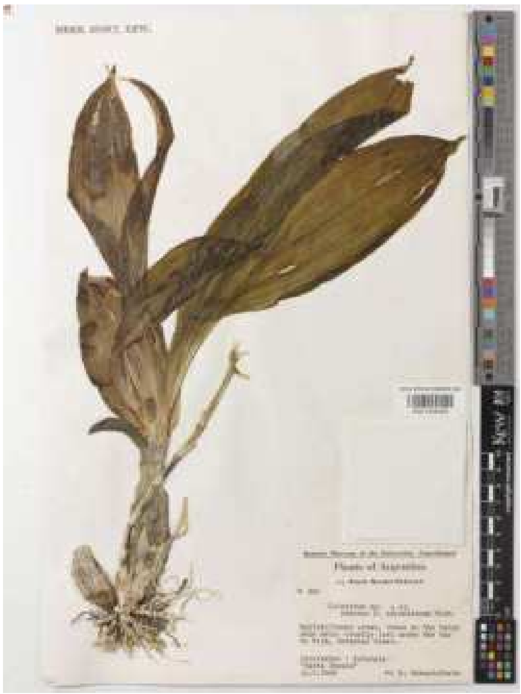

Specimen of *C. macrocarpum* from Kew's Herbarium - K001458485. Retrieved from Plants of the World Online

Historiae Rerum Naturalium Brasiliae	Marcgrave, 1648	Page number	35c

Vernacular name(s) Taiaoba. Taja (root)

Species Colocasia esculenta (L.) Schott

Family Araceae

Notes

The leaf in the herbarium could have been used as a model to draw the leaves of the woodcut.

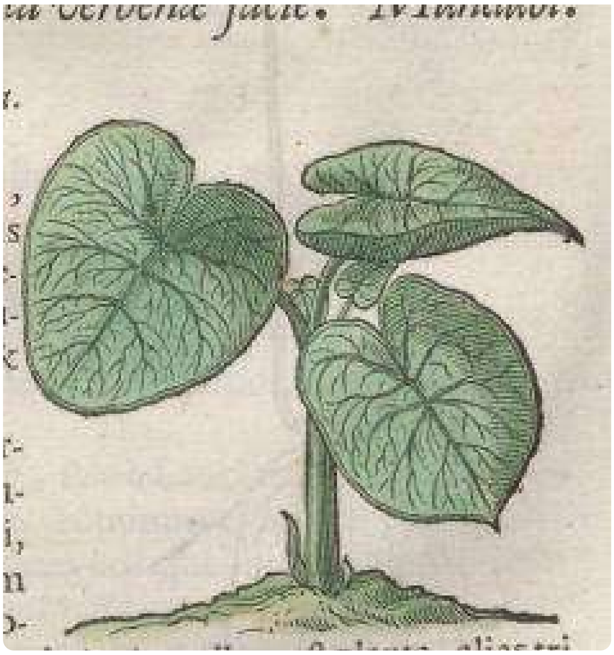

Historiae Plantarum – Herbis: 35c

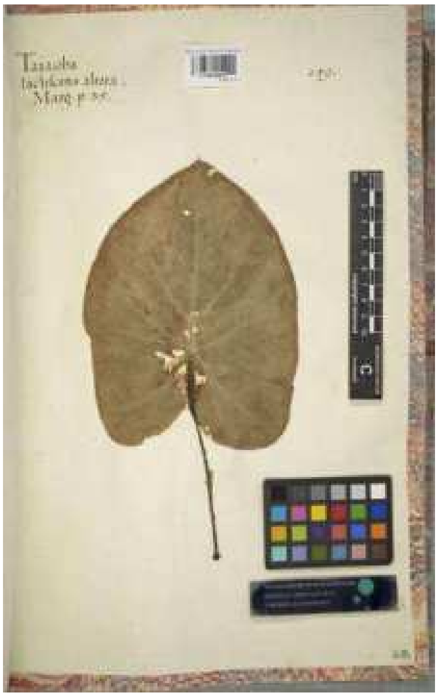

Marcgrave's herbarium: 23

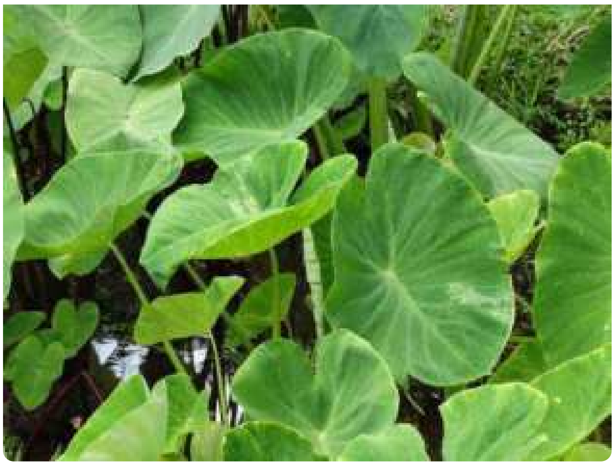

"Taro (*C. esculenta*): Dasheen mosaic" by Plant pests and diseases (CC0 1.0)

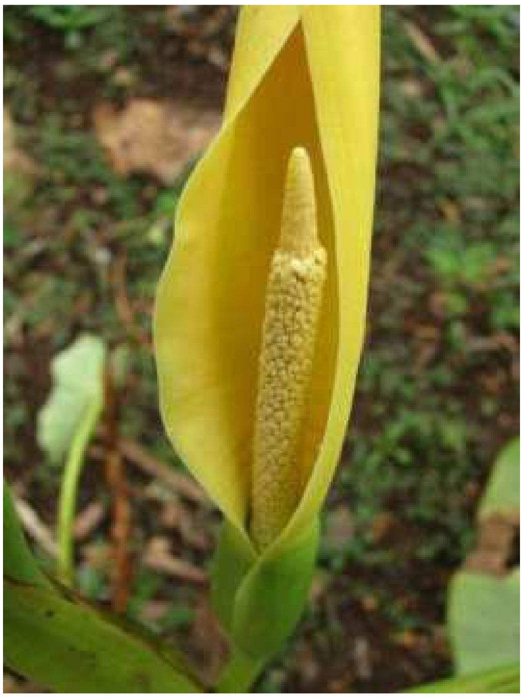

Flower. "*C. esculenta* 'Piko Kea'" by D.Eickhoff (CC BY-NC-SA 2.0)

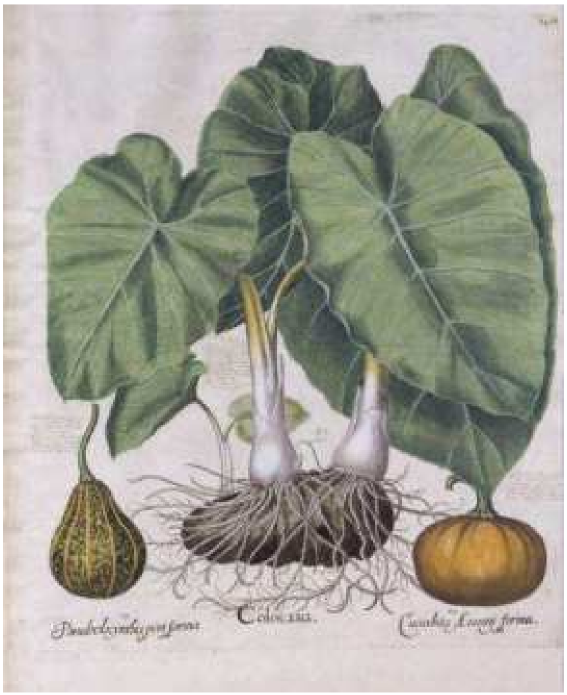

"Elephant's Ear, Taro Root. (*C. esculenta*), and Summer Squash (*Curcurbita pepo*) in *Hortus Eystettensis* by Bessler, Basilius (1620: Vol. III: t. 34)

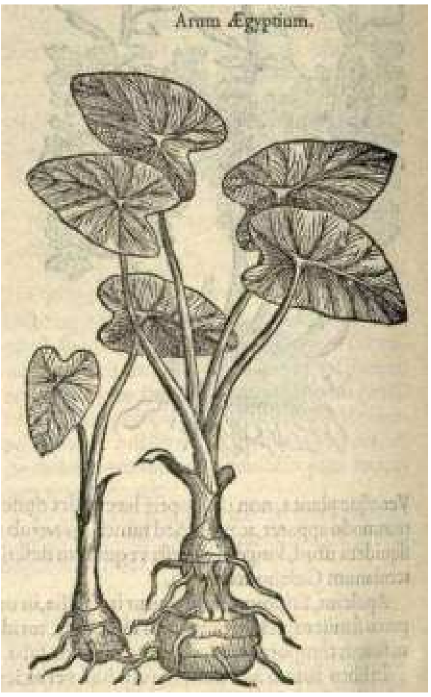

Engraving of *C. esculenta* in *Stirpium historiae pemptades sex, sive libri XXX* by Dodoens (1583: 326)

Historiae Rerum Naturalium Brasiliae Marcgrave, 1648 Page number 36a

Vernacular name(s) Taiaoba (secunda folio)

Species Xanthosoma sagittifolium (L.) Schott

Family Araceae

Notes

The leaf in the herbarium could have been used as a model to draw the leaves of the woodcut before ending up as an exsicata.

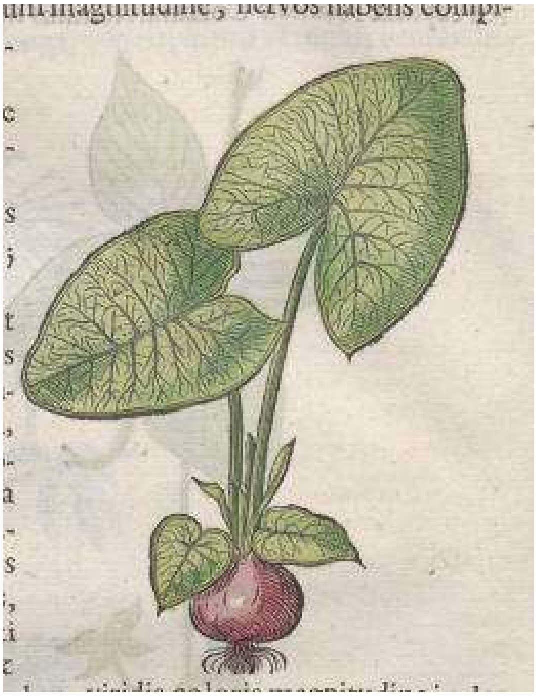

Historiae Plantarum – Herbis: 36a

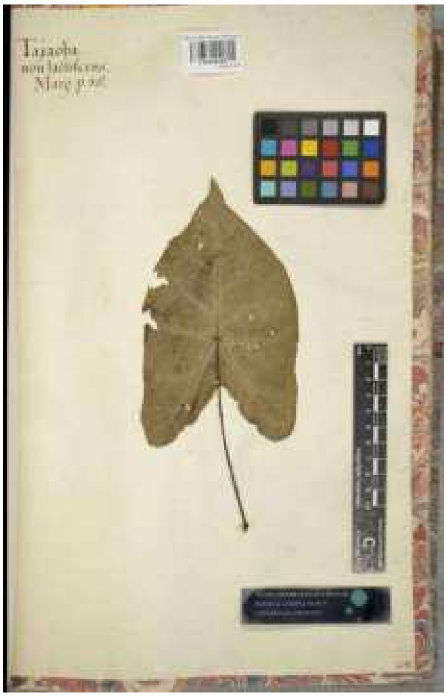

Marcgrave's herbarium: 24

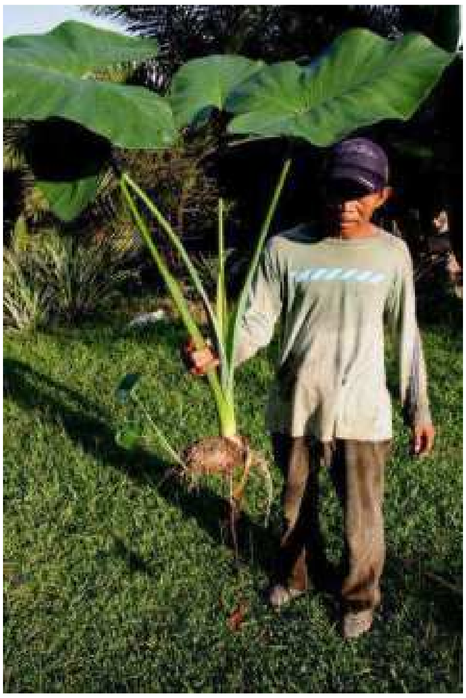

"*X. sagittifolium*" by Ahmad Fuad Morad (CC BY-NC-SA 2.0)

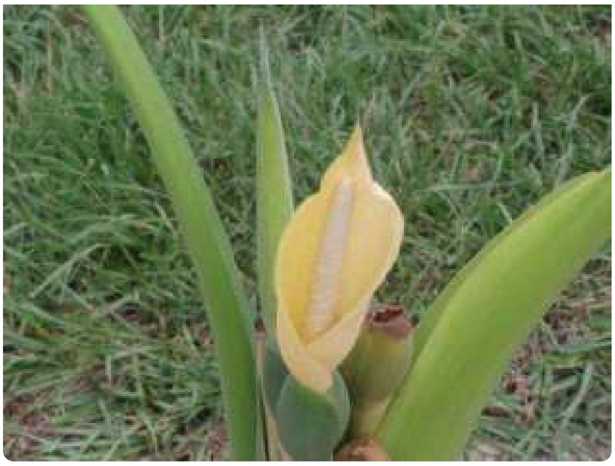

"*Xanthosoma sagittifolium* flower" by HedyCs (CC BY 2.0)

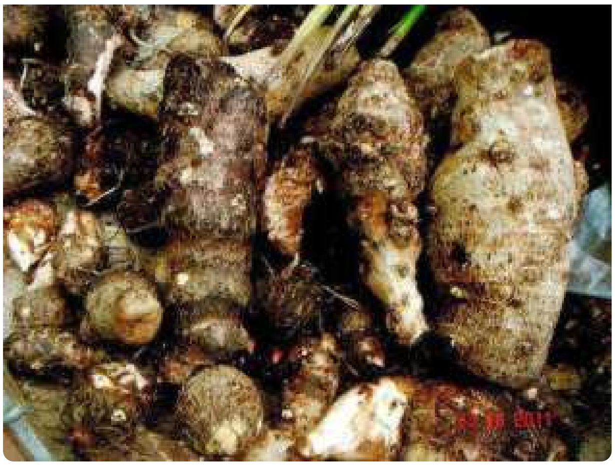

Edible tubers. "*X. sagittifolium*" by Ahmad Fuad Morad (CC BY-NC-SA 2.0)

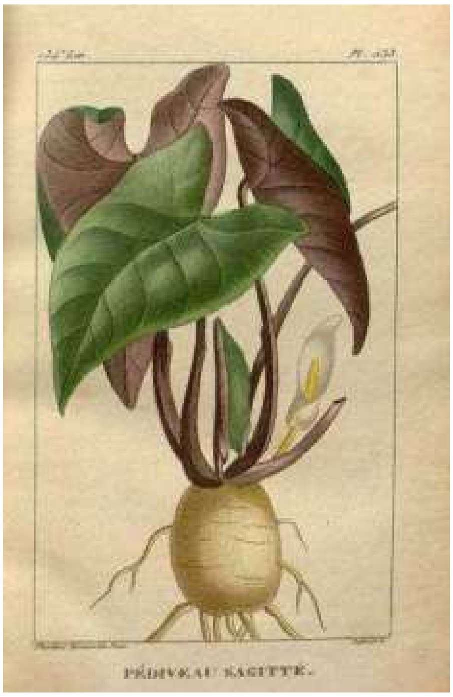

Flore [pittoresque et] médicale des Antilles by *Descourtilz, M. E.* (1829: Vol. VII, t. 533). Missouri Botanical Garden, St. Louis, U.S.A.

Historiae Rerum Naturalium Brasiliae Marcgrave, 1648 Page number 36b

Vernacular name(s) Taiaoba (tertia folio)

Species Caladium bicolor (Ait) Vent.

Family Araceae

Notes

We did not find any correspondence between this woodcut and the contemporary or older sources.

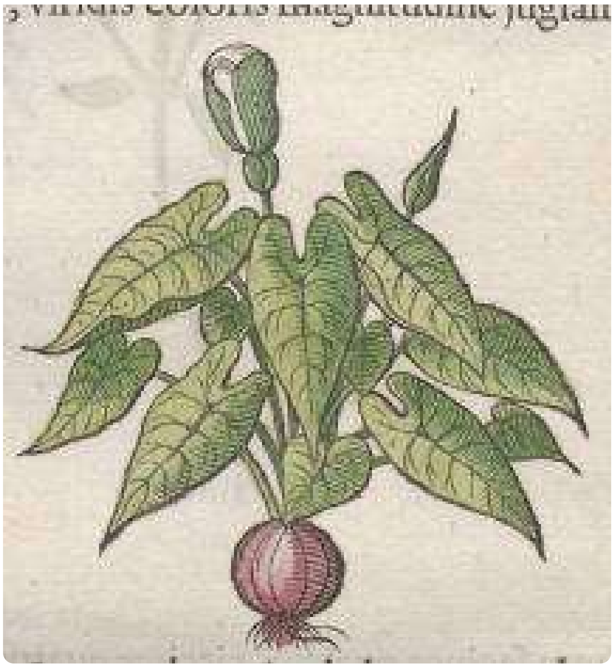

Historiae Plantarum – Herbis: 36b

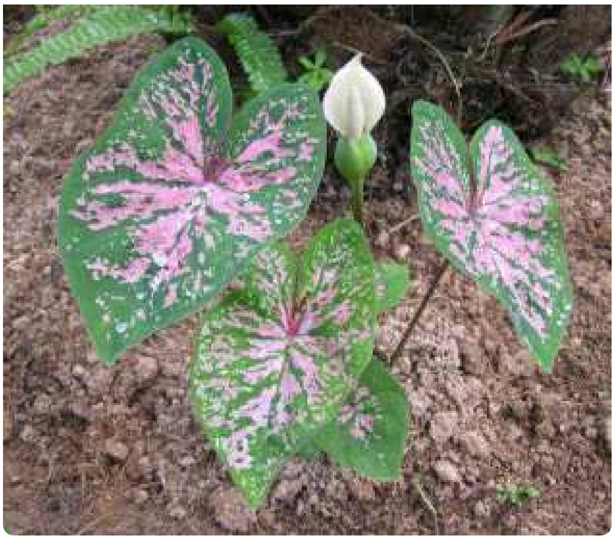

"*Caladium bicolor*, Tsing Yi Park, Hong Kong" by KHQ Flower Guide (CC BY-SA 2.0)

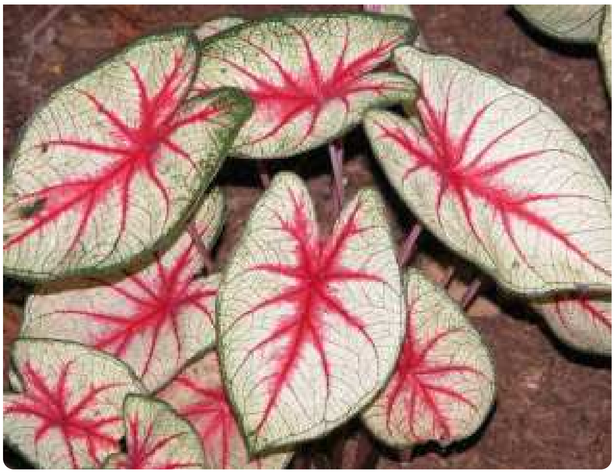

"*C. bicolor*" by Carl E Lewis (CC BY 2.0)

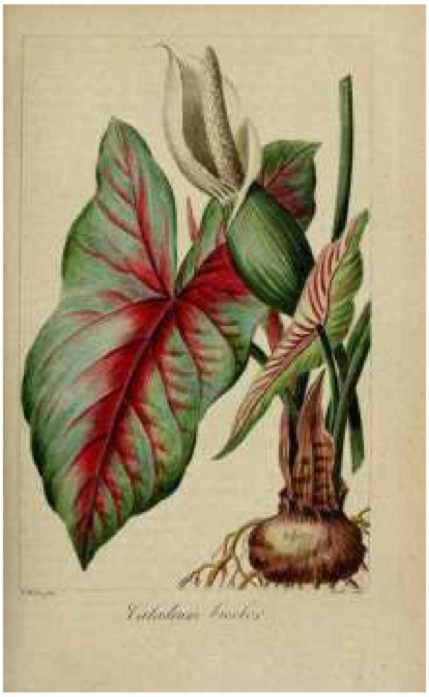

Herbier général de l'amateur by Delaunay, M., Loiseleur-Deslongchamps, J.L.A. (1824: Vol. VII, t. 492). Natural History Museum, London

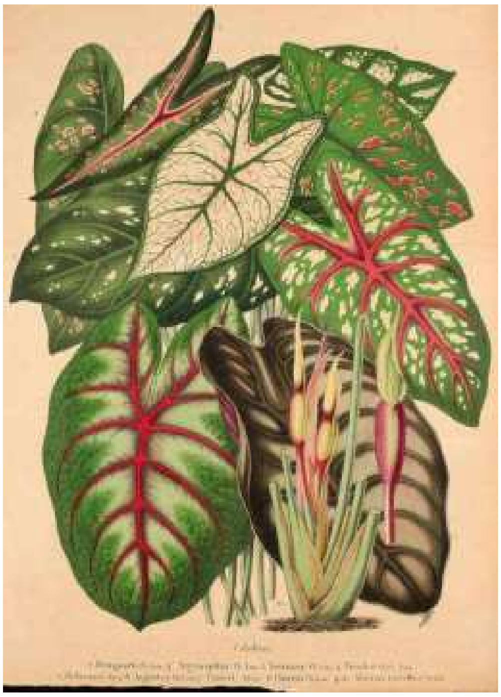

Belgique horticole, journal des jardins et des vergers (1861: Vol. XI, t. 1). New York Botanical Garden, USA

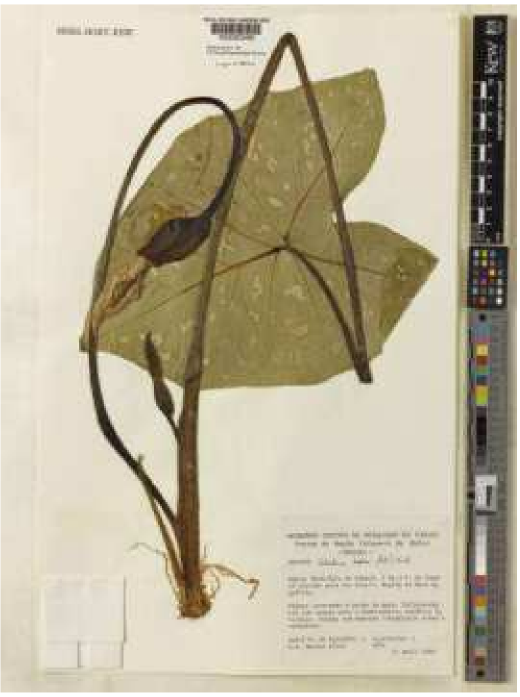

Specimen of *C. bicolor* from Kew's Herbarium - K000303460. Retrieved from Plants of the World Online

Historiae Rerum Naturalium Brasiliae Marcgrave, 1648 Page number 36c

Vernacular name(s) Iaborandi

Species Ertela trifolia (L.) Kuntze

Family Rutaceae

Notes

We did not find any correspondence between this woodcut and the contemporary or older sources.

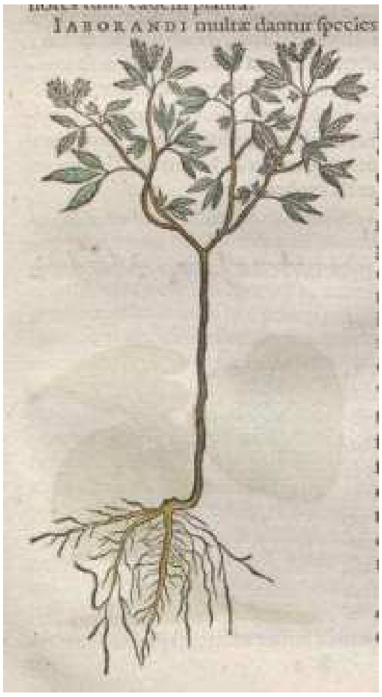

Historiae Plantarum – Herbis: 36c

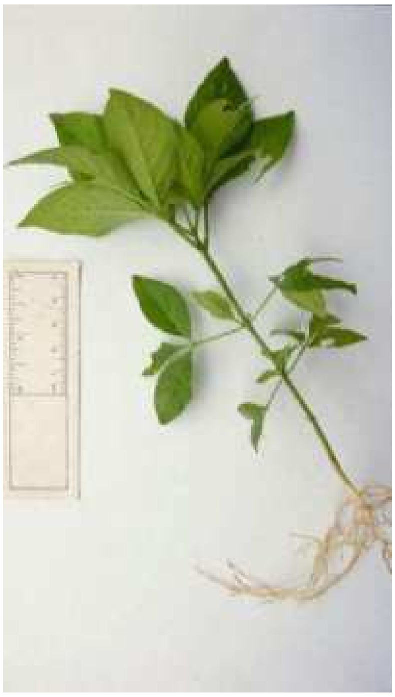

"*Ertela trifolia* (L.) Kuntze" by Alex Popovkin, Bahia, Brazil (CC BY-NC-SA 2.0)

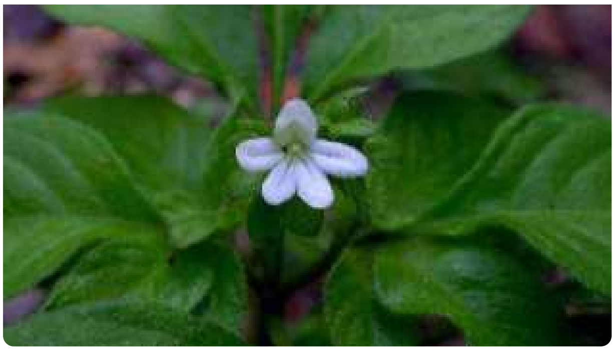

Flower. "*Ertela trifolia* (L.) Kuntze" by Alex Popovkin, Bahia, Brazil (CC BY-NC-SA 2.0)

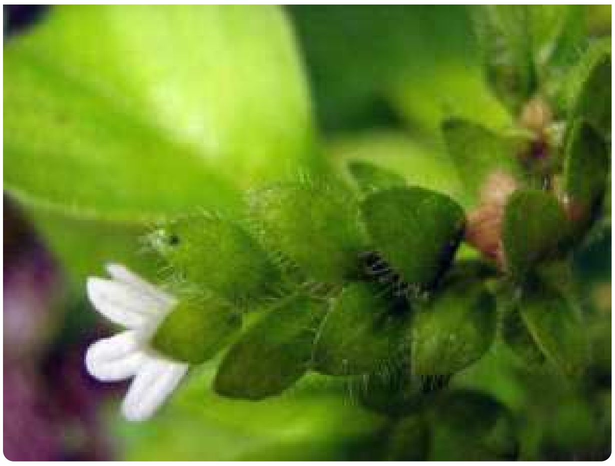

Flower bracts. "*Ertela trifolia* (L.) Kuntze" by Alex Popovkin, Bahia, Brazil (CC BY-NC-SA 2.0)

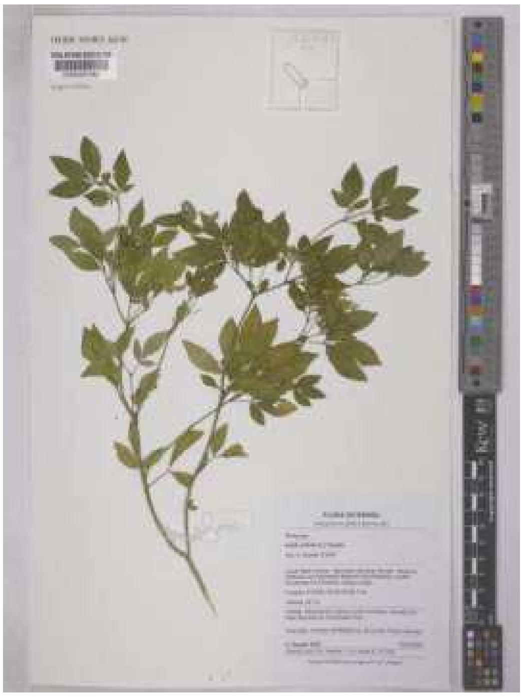

Specimen of *E. trifolium* from Kew's Herbarium - K000447255. Retrieved from Plants of the World Online

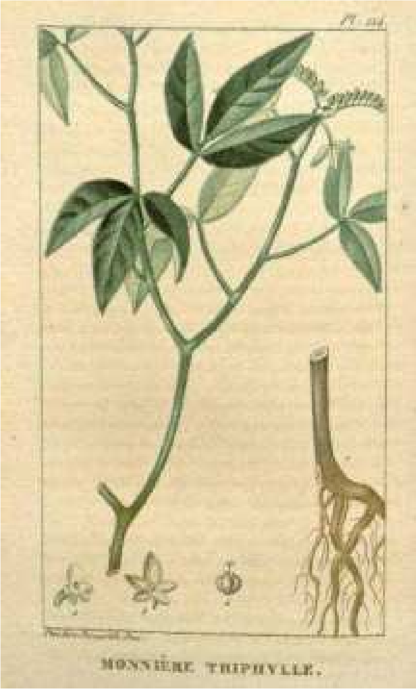

Engraving of *E. trifolium* in *Flore [pittoresque et] médicale des Antilles* by Descourtilz, M.E. (1827: Vol. III, t. 224). Missouri Botanical Garden, St. Louis, USA

Historiae Rerum Naturalium Brasiliae — Marcgrave, 1648 — Page number 37

Vernacular name(s)	Mundubi
Species	Arachis hypogaea L.
Family	Fabaceae
Notes	

The *Libri Principis* only shows an image of three fruits, one of them longitudinally open and showing its seeds (peanuts). This image is more similar to the still-life painting by Eckhout than to the woodcut, which depicts the whole plant with the root nodules and the underground fruits.

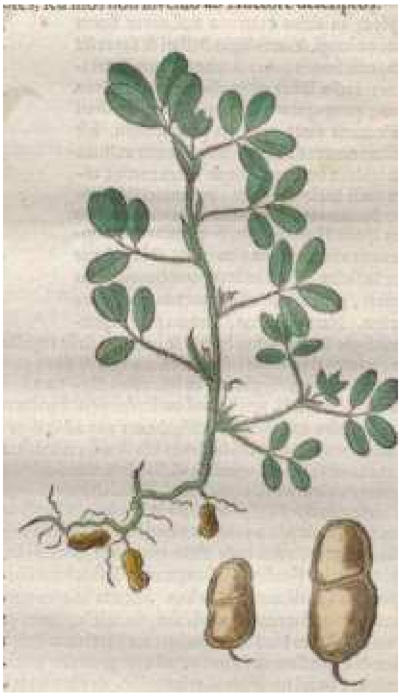

Historiae Plantarum – Herbis: 37

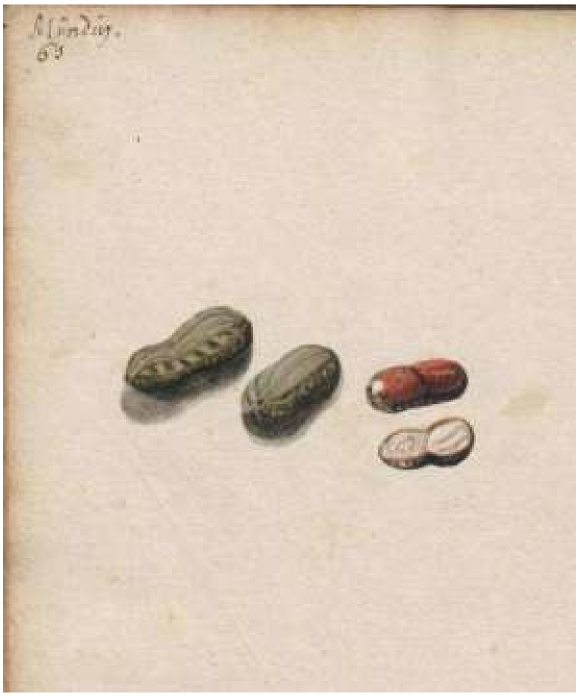

Libri Principis f. 81 [61]

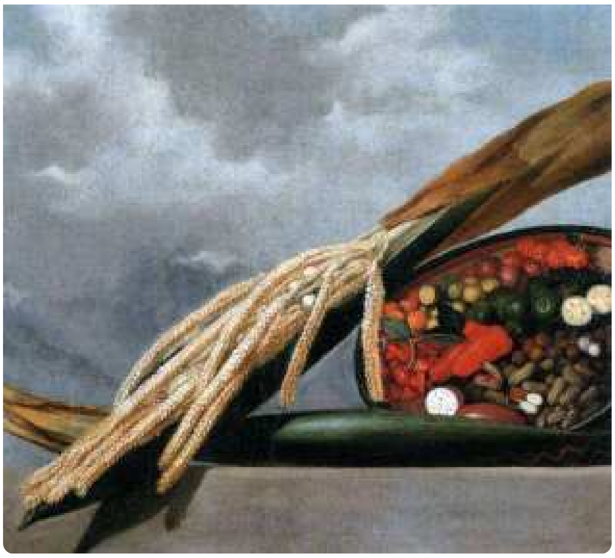

Still-life "with the inflorescence of the coconut palm and a brimming basket" by Eckhout, ca. 1640. National Museum of Copenhagen

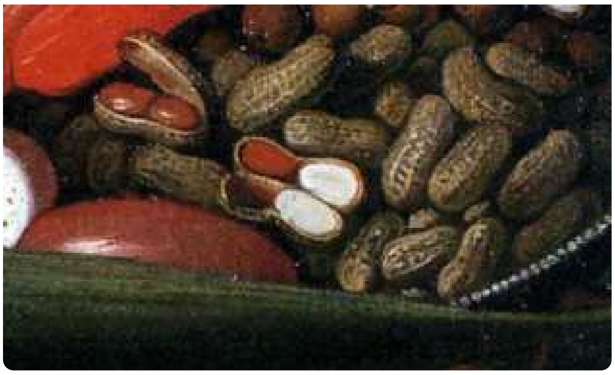

Close - up of the painting by Ekchout showing the peanuts

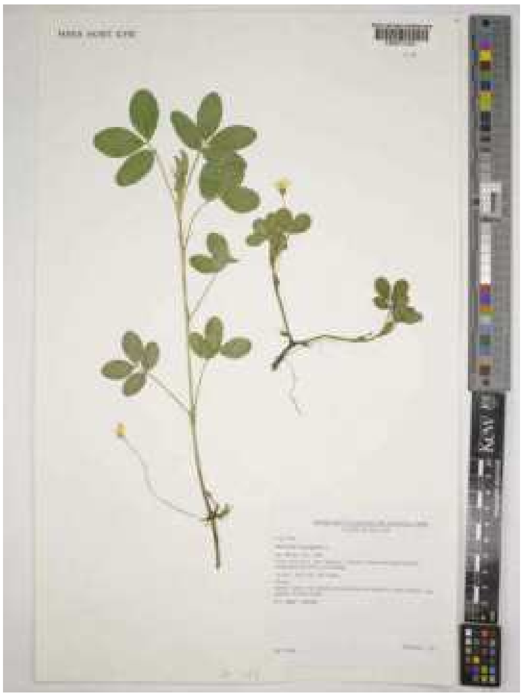

Specimen of *A. hypogaea* from Kew's Herbarium - K000731401. Retrieved from Plants of the World Online

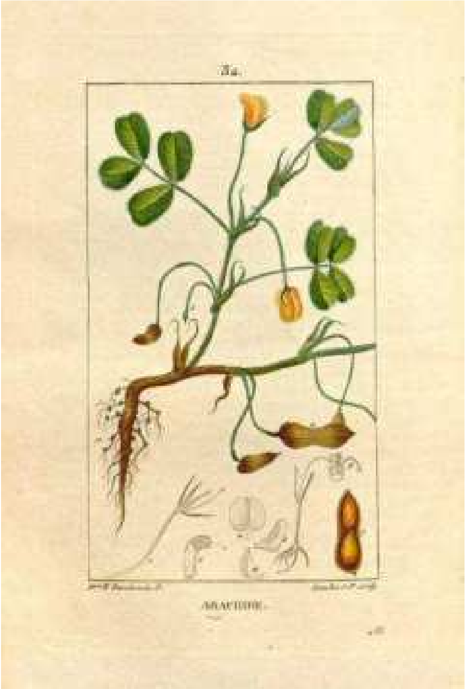

Engraving of *A. hypogaea* in *Flore médicale* by Chaumeton, F.P.(1833-1835: Vol. I, t. 32)

Historiae Rerum Naturalium Brasiliae Marcgrave, 1648 Page number 38

Vernacular name(s) Caraguata. Erva babosa. Alöe. Alöe americana Dodonei

Species Aloe vera (L.) Burm.f.

Family Asphodelaceae

Notes

The woodcut looks moderately similar to the *Theatrum* image, although the latter shows in addition a cut of the branch. The rhizome is not shown in the oil painting but it could be due to the cutting of the illustration to fit it into the sheet.

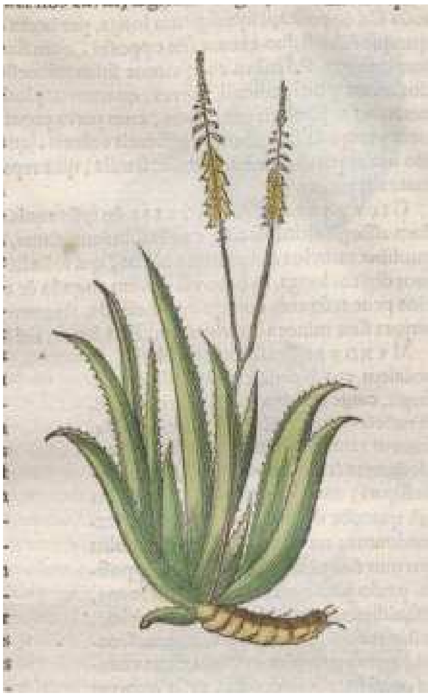

Historiae Plantarum – Herbis: 38

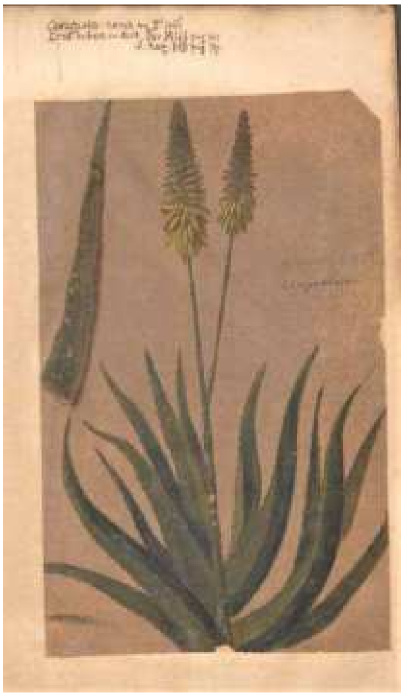

Theatrum Rerum Naturalium: 11

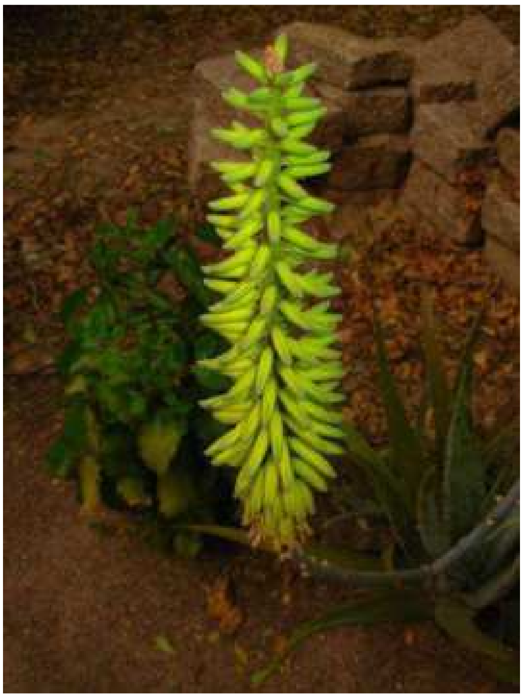

Inflorescence. "Babosa - *A. vera*" by Marcelo A. H. Penna / Beliche (CC BY-NC-SA 2.0)

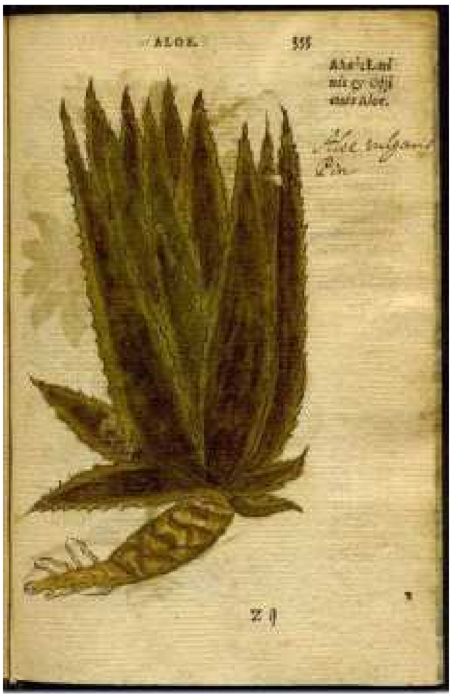

Woodcut of *A. vera* in *Stirpium historia commentariorum* by Dodoens (1553: Vol. I, p. 355)

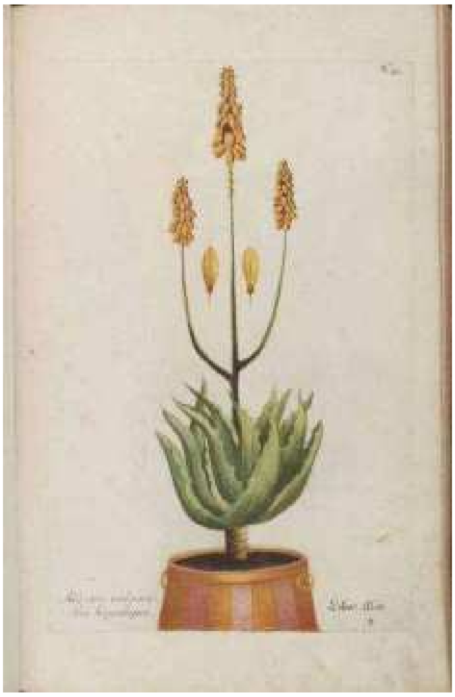

Engraving of *A. vera* in *Phytanthoza iconographia* by Weinmann, J.W. (1737: Vol. I, t. 43). Missouri Botanical Garden, St. Louis, U.S.A.

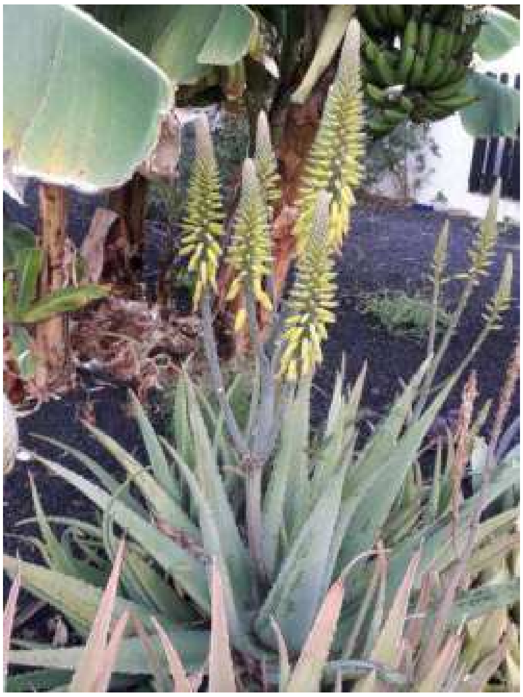

Aloe vera - Castillo Caleta de Fuste, Fuerteventura, Canary Is., 2020, by Rafaël Govaerts. Retrieved from Plants of the World Online

Historiae Rerum Naturalium Brasiliae Marcgrave, 1648 Page number 39

Vernacular name(s) Herba

Species Sesuvium portulacastrum (L.) L.

Family Aizoaceae

Notes

The woodcut is different to the *Theatrum*. Both are flowering shoots, so it could be that the specimen was used to make the woodcut. However, the herbarium voucher is a small shoot and it does not show the roots, as in the woodcut image.

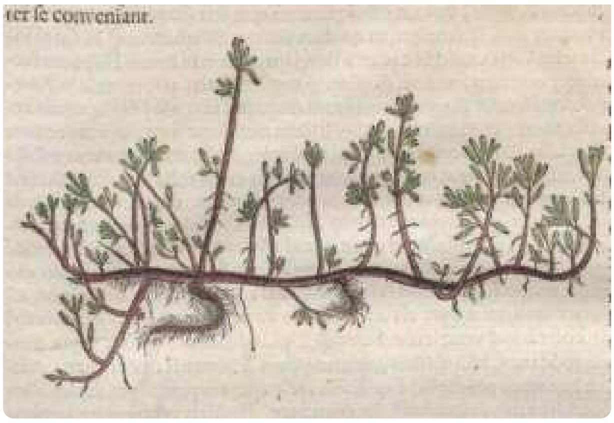

Historiae Plantarum – Herbis: 39

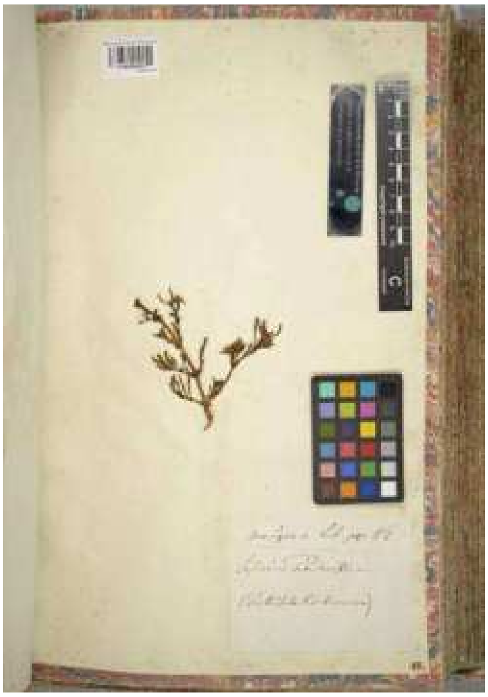

Marcgrave's herbarium: 83

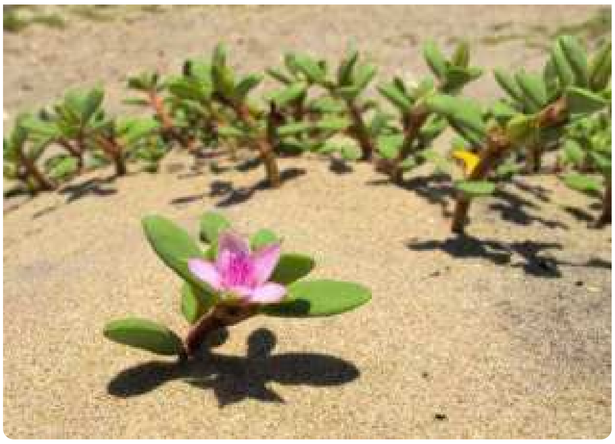

Habitat. "*Sesuvium portulacastrum*" by Josh*m (CC BY-NC-SA 2.0)

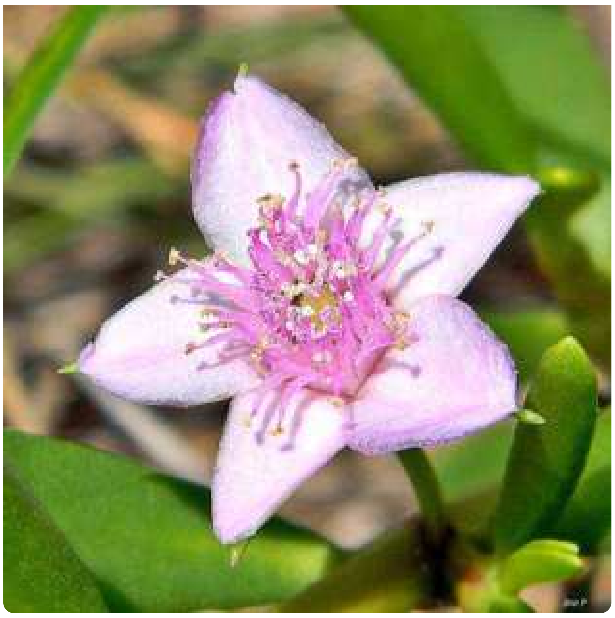

"Shoreline Sea Purslane (*S. portulacastrum*)" by Bob Peterson (CC BY 2.0)

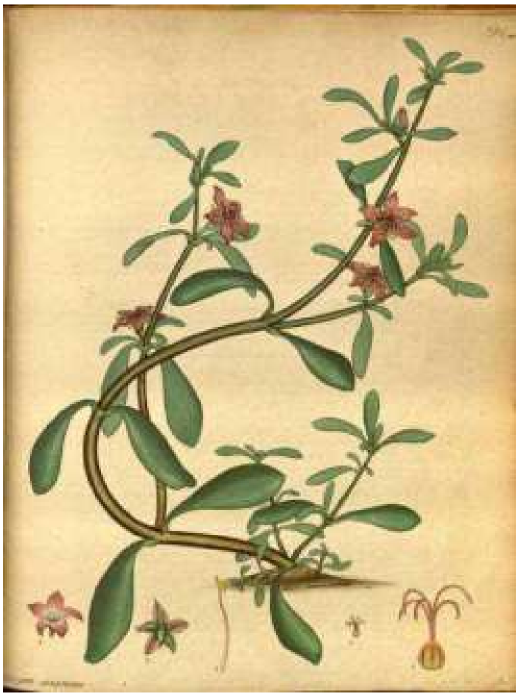

Botanist's repository by Andrews, H.C. (1801: Vol. III, t. 201). Missouri Botanical Garden, St. Louis, U.S.A

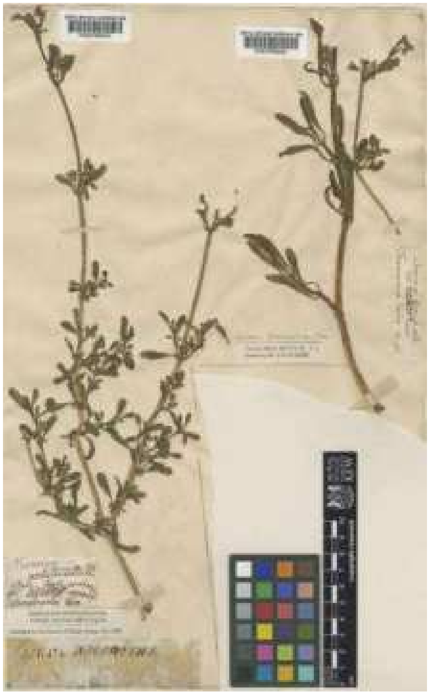

Specimen of *S. portulacastrum* from Kew's Herbarium - K000768444. Retrieved from Plants of the World Online

Historiae Rerum Naturalium Brasiliae — Marcgrave, 1648 — Page number 41

Vernacular name(s) Ieticucu. Radix Mechoacan. Batata de purga

Species Operculina hamiltonii (G. Don) D.F. Austin & Staples

Family Convolvulaceae

Notes

We did not find any correspondence between this woodcut and the contemporary or older sources.

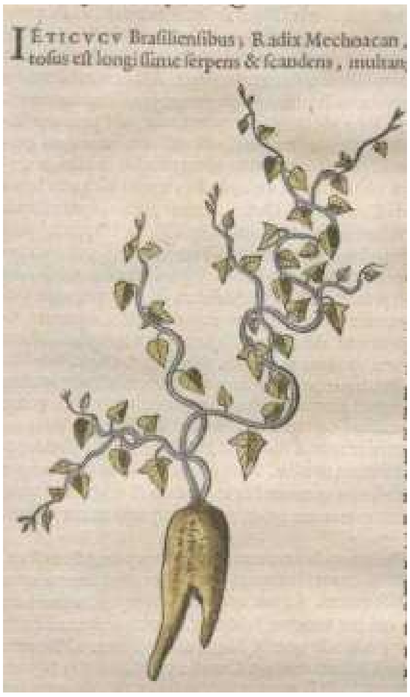

Historiae Plantarum – Herbis: 41

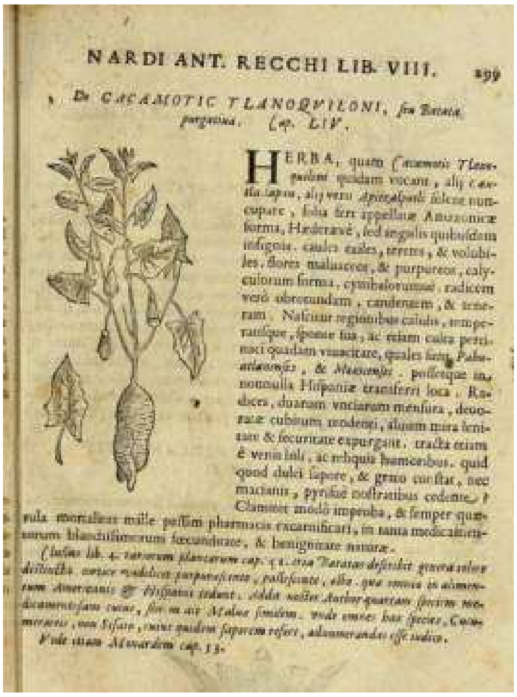

Woodcut of *O. hamiltonii in Rerum medicarum Novae Hispaniae Thesaurus* by Hernandez, F. (1628: 299). Getty Research Institute, Los Angeles, U.S.A.

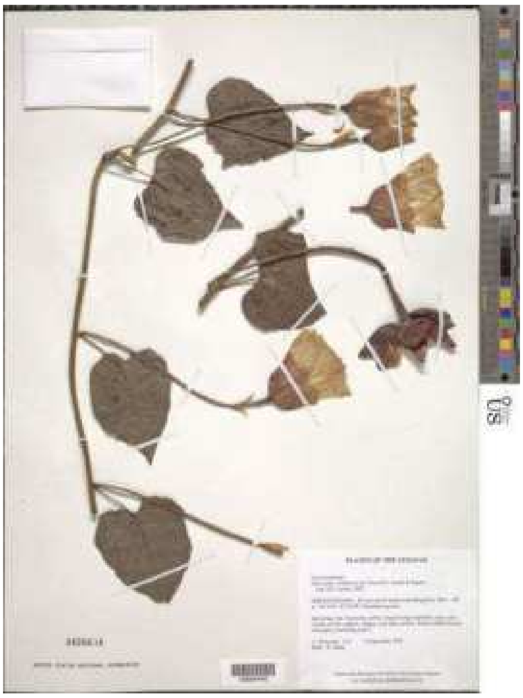

Specimen. "*O. hamiltonii*" by Anna L. Weitzman and W. Hahn -00684445- Smithsonian National Museum of Natural History (CC0 1.0)

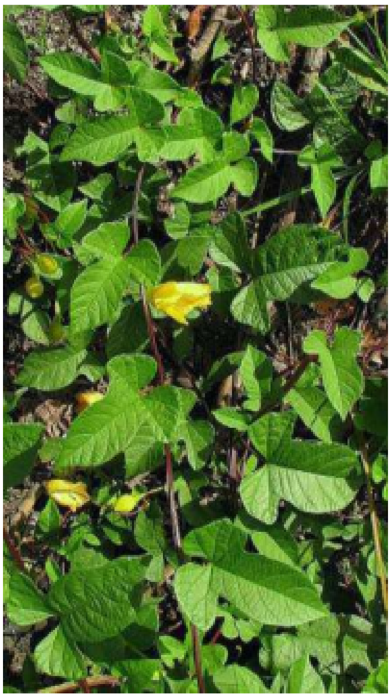

O. alata [syn. *O. hamiltonii*] by Alex Popovkin, Bahia, Brazil (CC-BY-2.0)

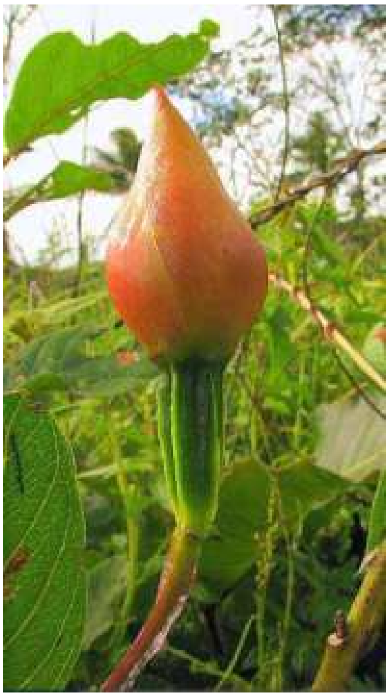

Developing fruit of *O. alata* [syn. *O. hamiltonii*] by Alex Popovkin, Bahia, Brazil (CC-BY -2.0)

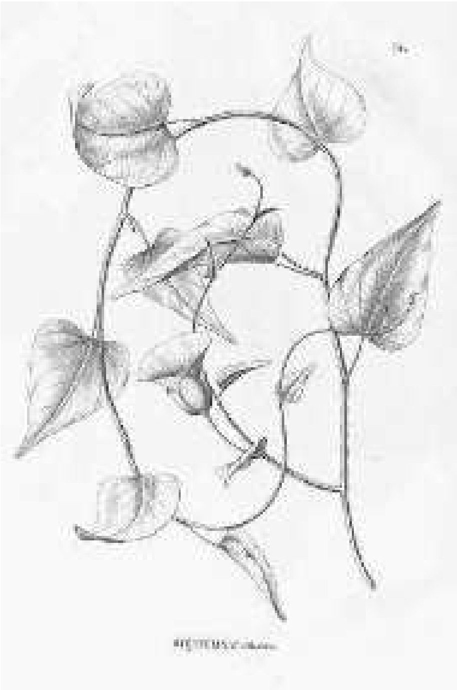

Engraving of *O. hamiltonii* in Martius, C.F.P. von, Eichler, A.G., Urban, I., *Flora Brasiliensis* (1856-1871) Vol. 7: 75

Historiae Rerum Naturalium Brasiliae | Marcgrave, 1648 | Page number 43

Vernacular name(s): Planta

Species: Sida linifolia Juss. ex Cav.

Family: Malvaceae

Notes

We did not find any correspondence between this woodcut and the contemporary or older sources.

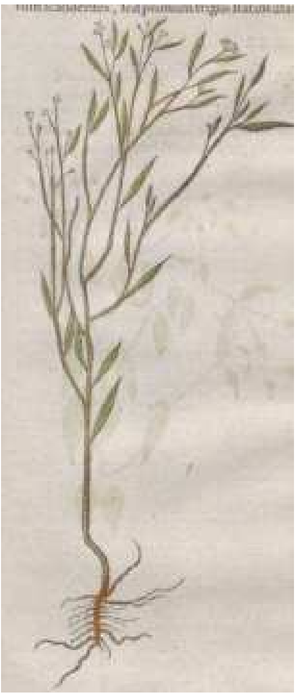

Historiae Plantarum – Herbis: 43

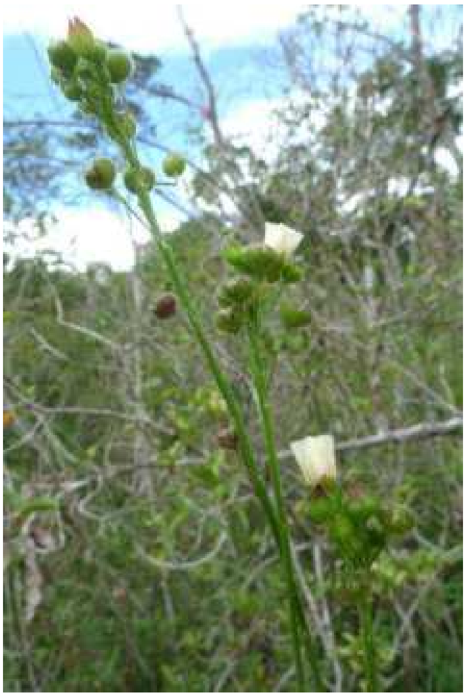

Sida linifolia - W.Milliken/RBG, Kew. Retrieved from Plants of the World Online

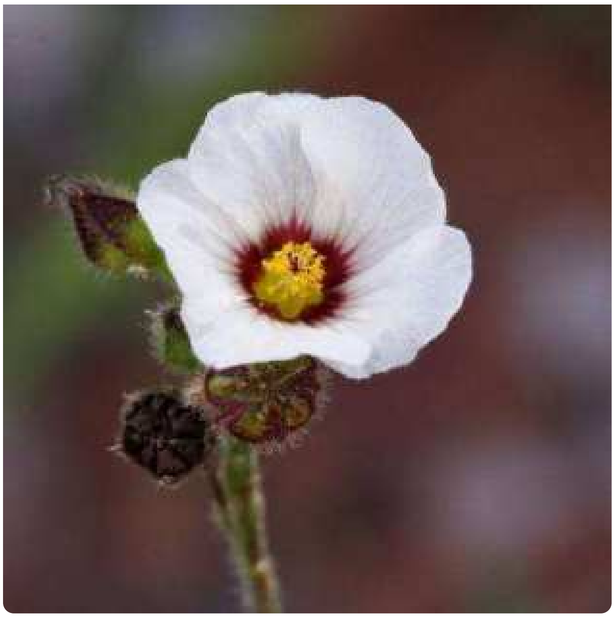

Flower. "*S. linifolia*" by Mauricio Mercadante, Brasília, Brasil (CC BY-NC-SA 2.0)

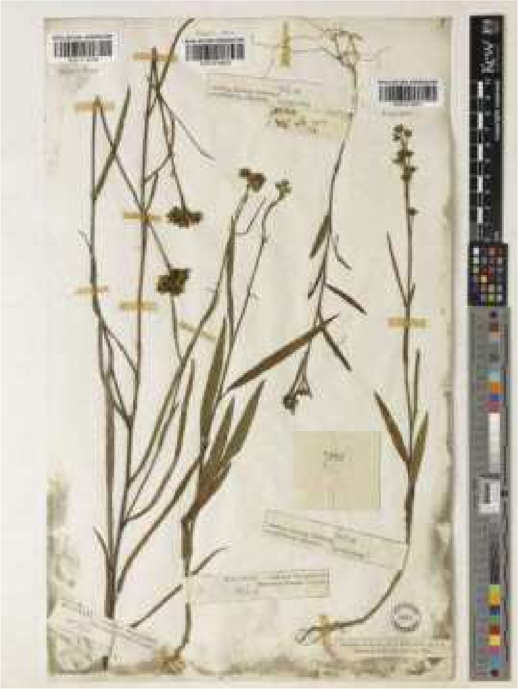

Specimen of *S. linifolia* from Kew's Herbarium - K001214639. Retrieved from Plants of the World Online

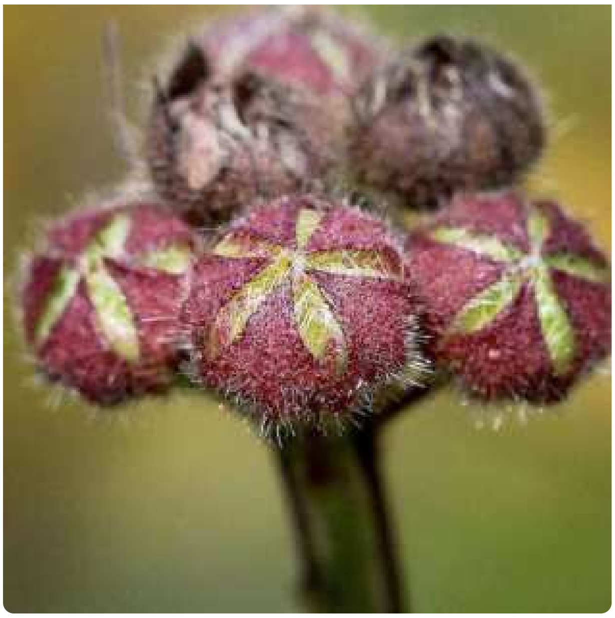

Flower buds. "*S. linifolia*" by Mauricio Mercadante, Brasília, Brasil (CC BY-NC-SA 2.0)

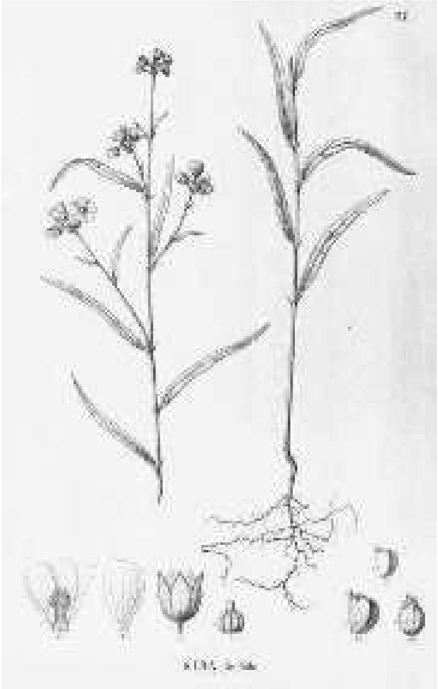

Engraving of *S. linifolia* in Martius, C.F.P. von, Eichler, A.G., Urban, I., *Flora Brasiliensis* (1886-1892) Vol. 12 (3): 57

Historiae Rerum Naturalium Brasiliae	Marcgrave, 1648	Page number	44a

Vernacular name(s) Iurumu. Bobora. Pompoen

Species Cucurbita pepo L.

Family Cucurbitaceae

Notes

The woodcut is different than the *Theatrum* and the *Libri Principis* images. The *Theatrum* only shows the pumpkin fruit and the watercolors in the *Libri Principis* bear more resemblance to Eckhout's still-life paintings. The calabash in one of the watercolors of the Libri Principis (f. 93 [63]) as well as the still-life paintings could also be the related species *Cucurbita maxima* Duchesne.

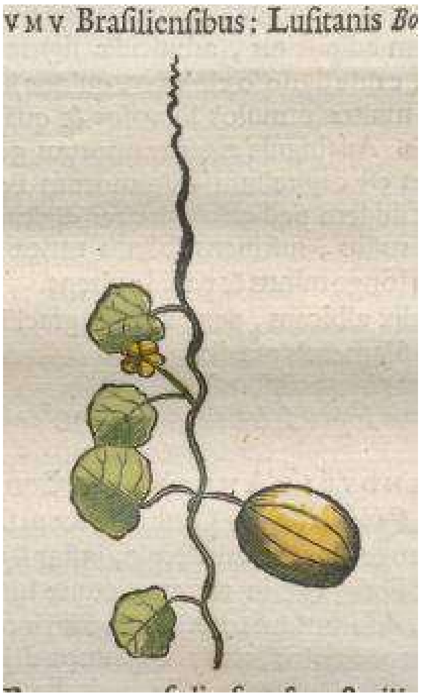

Historiae Plantarum – Herbis: 44a

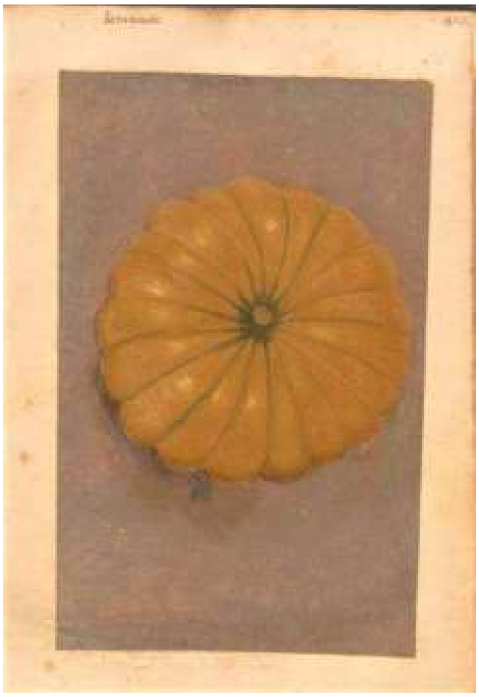

Theatrum Rerum Naturalium: 453

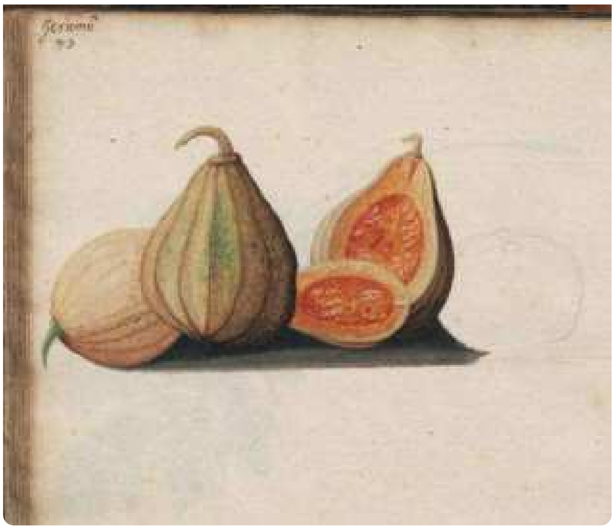

Libri Principis f. 6 [30]

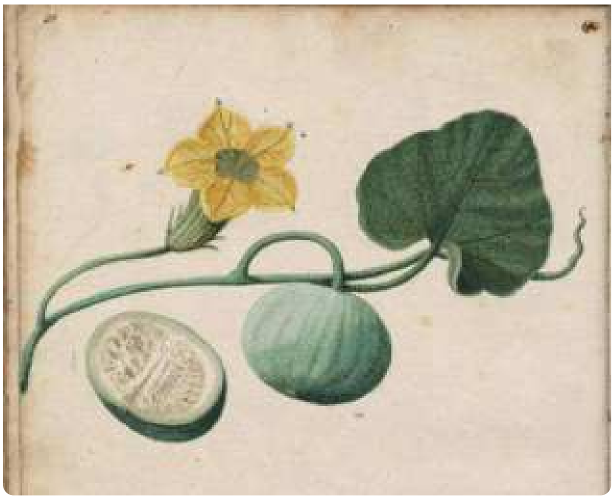

Libri Principis f. 93 [63]

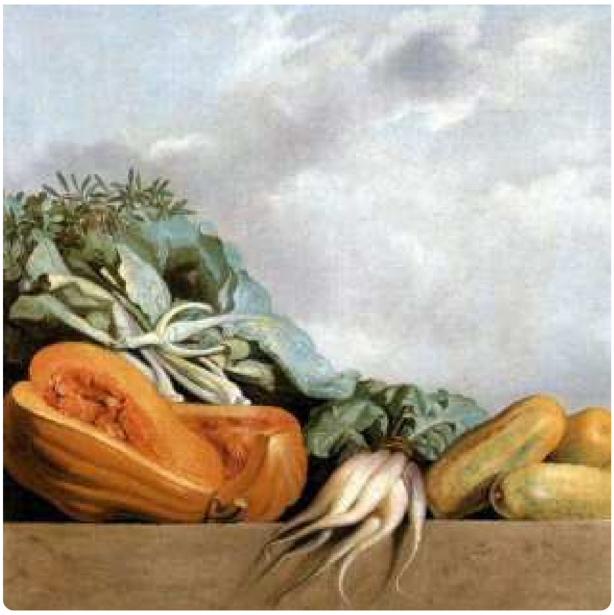

Still-life "with vegetables" by Eckhout, ca. 1640. National Museum of Copenhagen

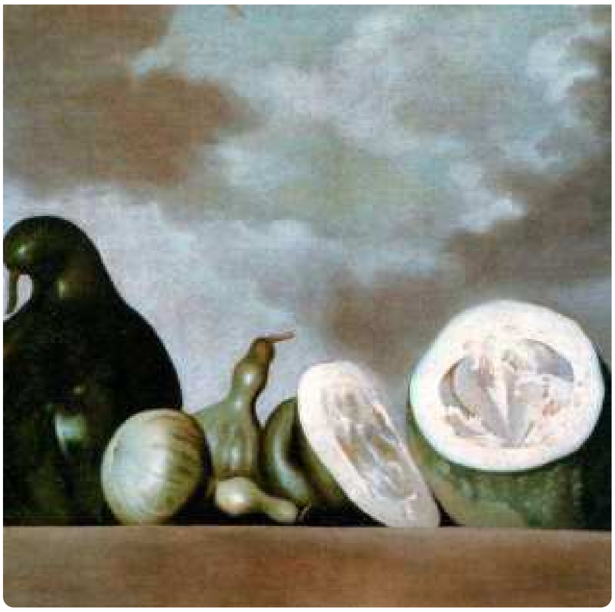

Still-life "with calabash" by Eckhout, ca. 1640. National Museum of Copenhagen

Historiae Rerum Naturalium Brasiliae	Marcgrave, 1648	Page number	44b

Vernacular name(s) Herba

Species Blainvillea acmella (L.) Philipson

Family Asteraceae

Notes

We did not find any correspondence between this woodcut and the contemporary or older sources.

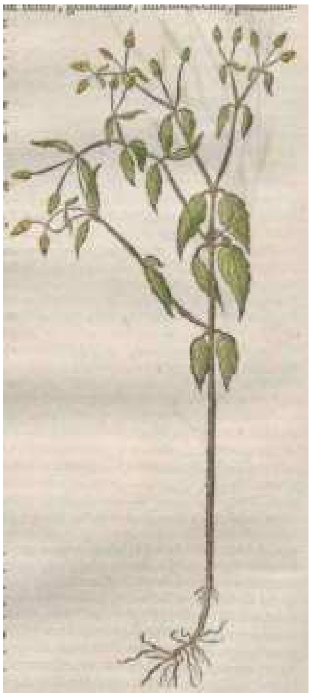

Historiae Plantarum – Herbis: 44b

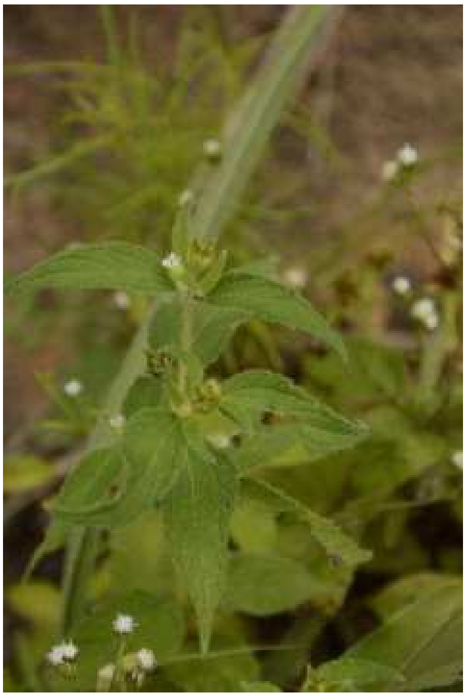

"*Blainvillea acmella* (1)" by siddarth.machado (CC BY-NC-ND 2.0)

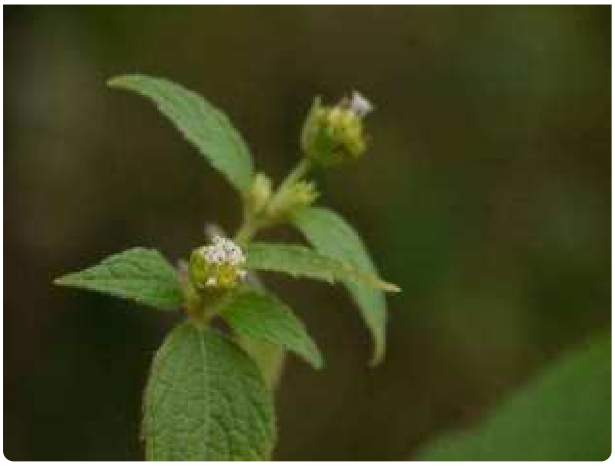

"*B. acmella*" by Dinesh Valke (CC BY-SA 2.0)

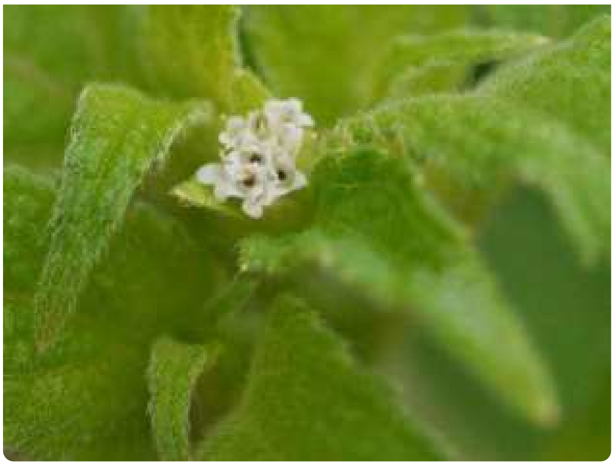

"*B. acmella*" by Dinesh Valke (CC BY-SA 2.0)

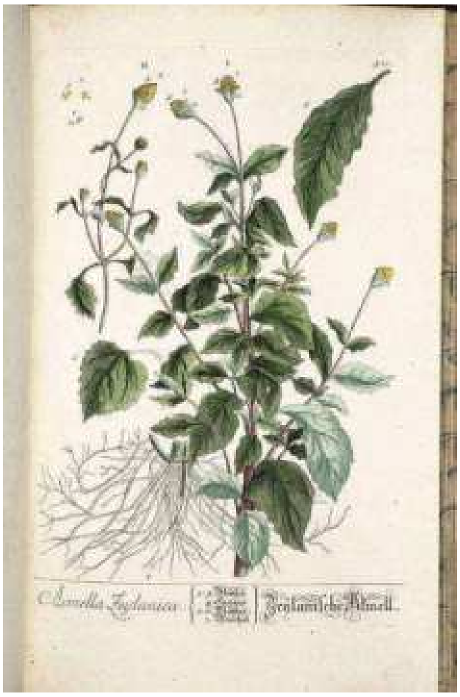

B. acmella in *Herbarium Blackwellianum* by Blackwell, E. (1773: Vol. VI, t. 518). Missouri Botanical Garden, St. Louis, U.S.A.

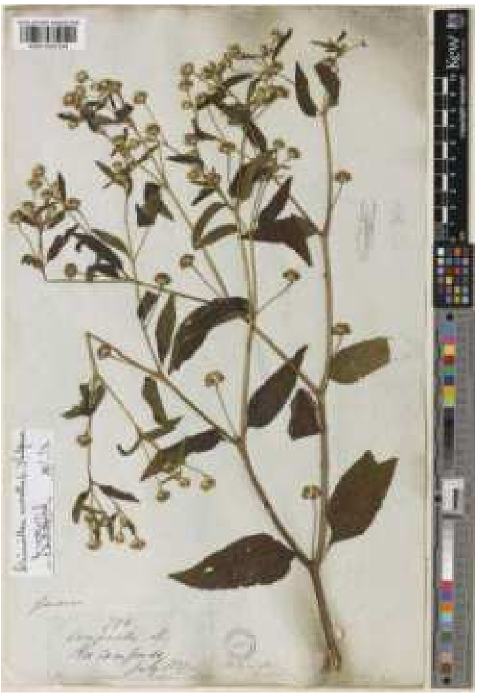

A specimen of *B. acmella* from Kew's Herbarium - K001092334. Retrieved from Plants of the World Online

Historiae Rerum Naturalium Brasiliae | Marcgrave, 1648 | Page number 45

Vernacular name(s) Alcaea hirsuta

Species <u>Abelmoschus moschatus Medik.</u>

Family Malvaceae

Notes

There is no resemblance between the woodcut and the drawing in the *Misc. Cleyeri* or the specimen in the herbarium.

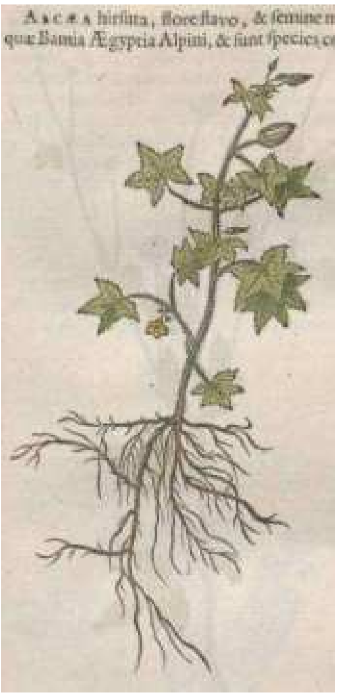

Historiae Plantarum – Herbis: 45

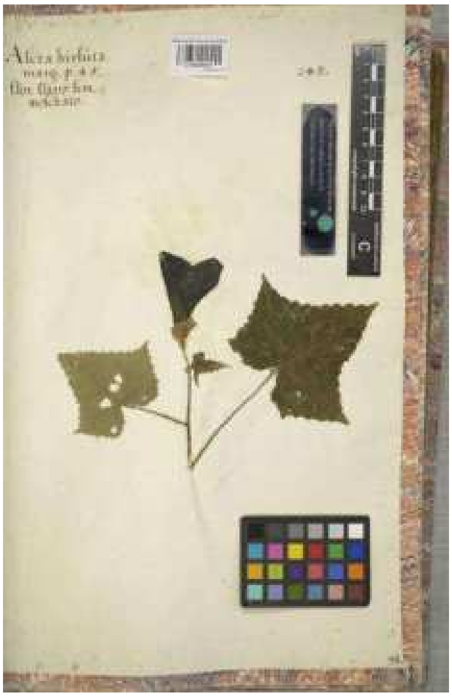

Marcgrave's herbarium: 31

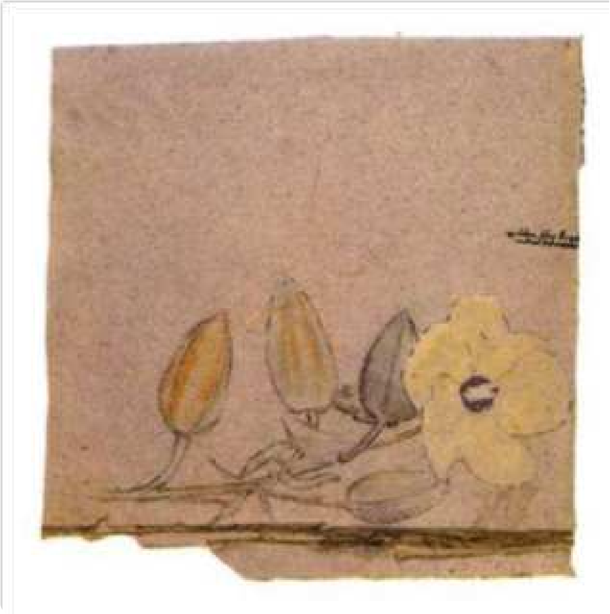

Misc. Cleyeri: 68v

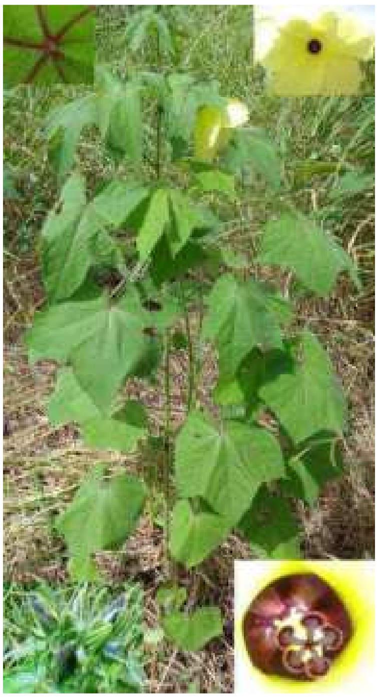

"*A. moschatus*" by Gelo--2014 (CC BY-NC 2.0)

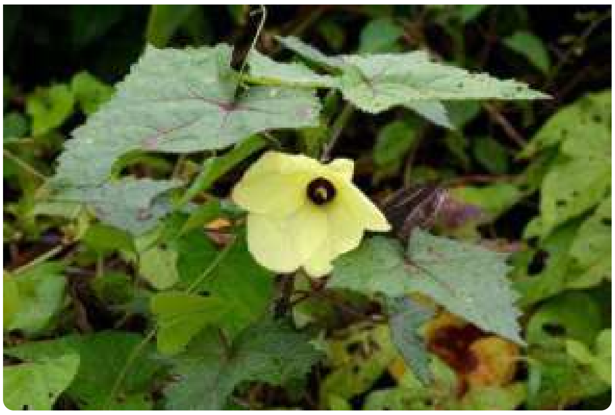

Flower. "*A. moschatus* (Malvaceae)" by Dr. Alexey Yakovlev (CC BY-SA 2.0)

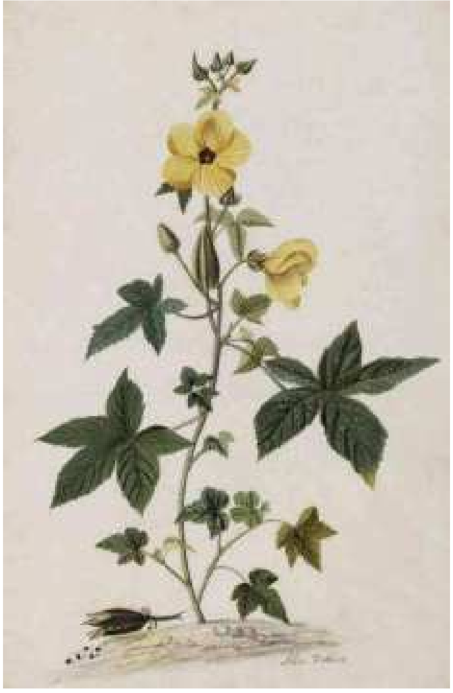

Engraving of *A. moschatus* in *Moninckx atlas* by Moninckx, J. (1682: Vol. IV, t. 25). University of Amsterdam, Netherlands

Historiae Rerum Naturalium Brasiliae	Marcgrave, 1648	Page number	46a

Vernacular name(s) Garyophillata herba

Species Bidens riparia Kunth

Family Asteraceae

Notes

We did not find any correspondence between this woodcut and the contemporary or older sources.

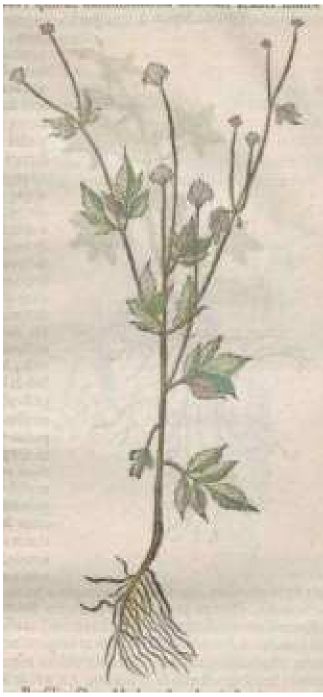

Historiae Plantarum – Herbis: 46a

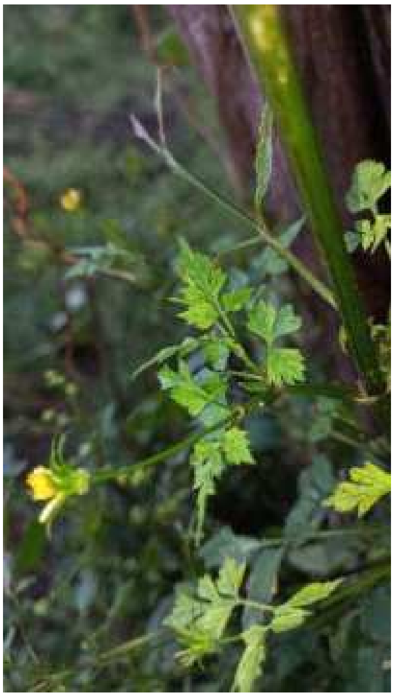

B. riparia observed in Mexico for iNaturalist by Miguel Angel Tornez Alvarez (CC BY-NC 4.0)

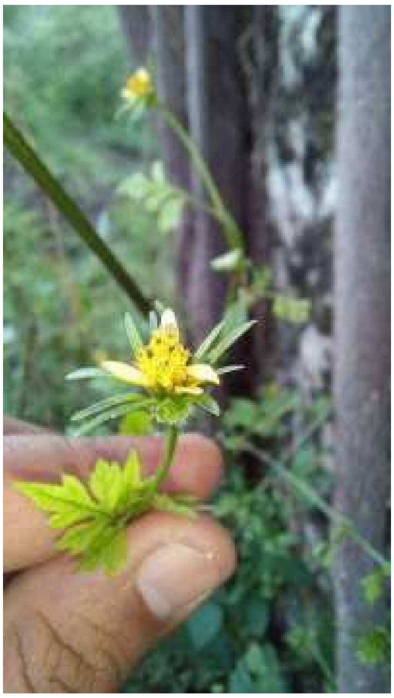

B. riparia observed in Mexico for iNaturalist by Miguel Angel Tornez Alvarez (CC BY-NC 4.0)

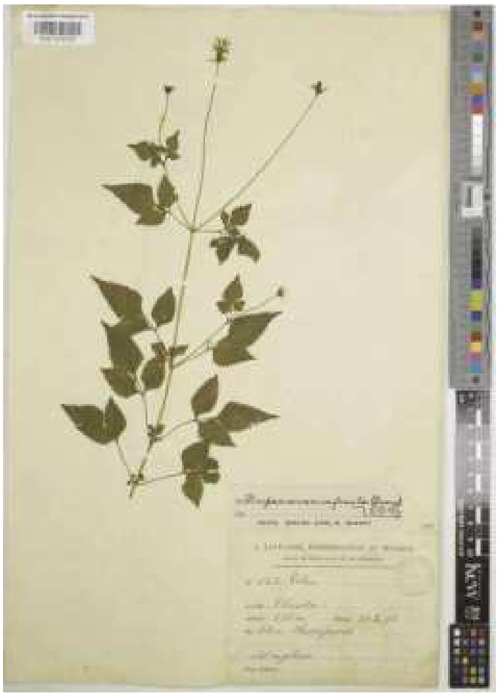

Specimen of *B. riparia* from Kew's Herbarium - K001479725. Retrieved from Plants of the World Online

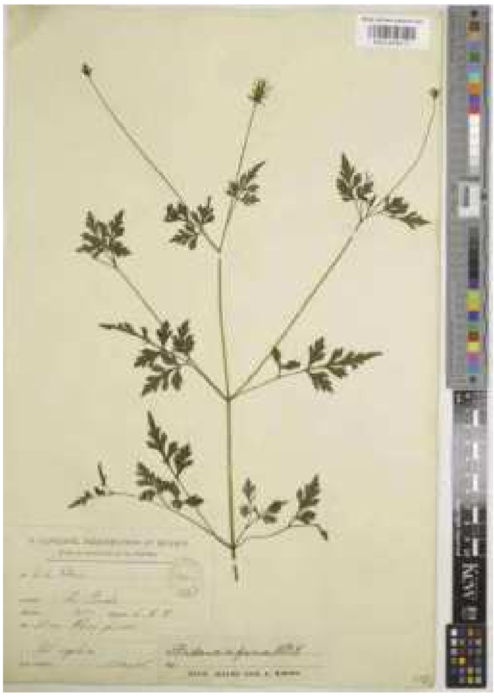

A specimen from Kew's Herbarium - K001479717. Retrieved from Plants of the World Online

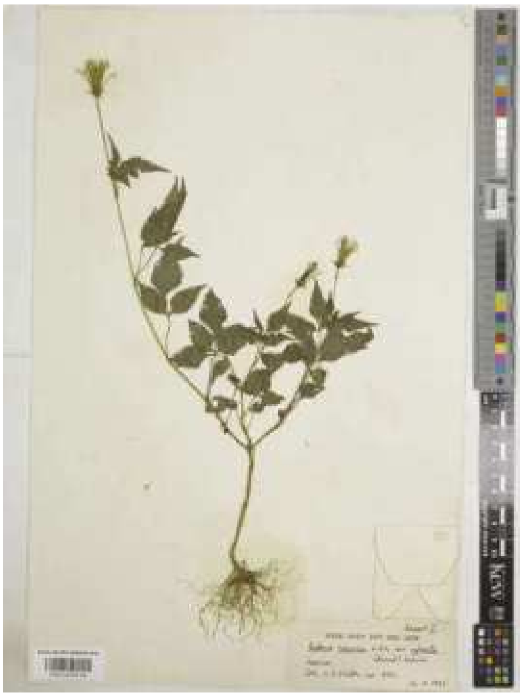

A specimen from Kew's Herbarium - K001479718. Retrieved from Plants of the World Online

Historiae Rerum Naturalium Brasiliae Marcgrave, 1648 Page number 46b

Vernacular name(s) Ghandiroba. Nhandiroba. Hederae scandentis species

Species Fevillea trilobata L.

Family Cucurbitaceae

Notes

The woodcut is very similar to the *Theatrum* image (non-reversed). The crayon drawing of the *Misc. Cleyeri* shows a shoot with dropping leaves, as if the painter placed it on a surface after cutting it from the main plant (that explains the droopiest leaves), while the oil painting depicts the leaves in an upward position (flattened like an exsicata). The fruit of these images bears some resemblance, although it is attached to the shoot in the *Theatrum* and woodcut, but loose in the *Misc. Cleyeri*. The drawing was made long after gathering the shoot from the main plant; the fruit was no longer attached and this was likely added to the *Theatrum* as a "composite" rather than copying it directly after nature.

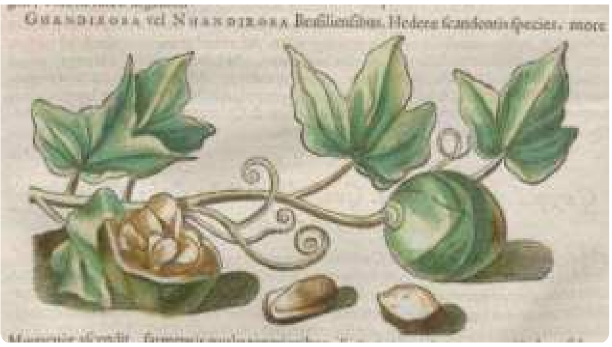

Historiae Plantarum – Herbis: 46b

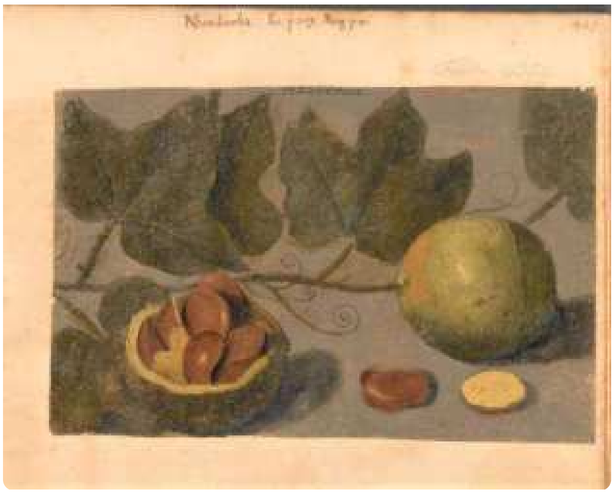

Theatrum Rerum Naturalium: 449

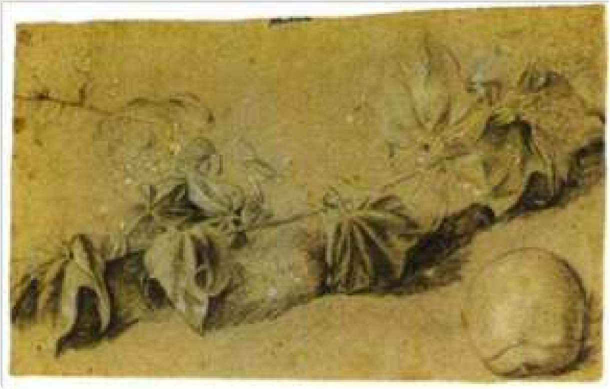

Misc. Cleyeri: 58r

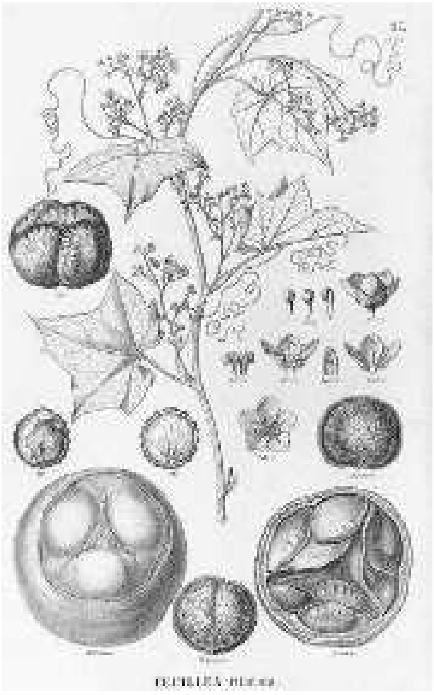

Engraving of *S. linifolia* in Martius, C.F.P. von, Eichler, A.G., Urban, I., *Flora Brasiliensis* (1878-1885) Vol. 6 (4): 117

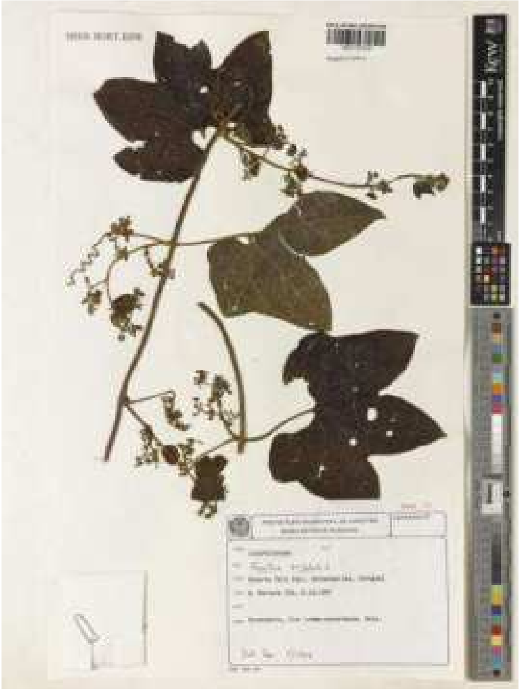

Specimen of *F. trilobata* from Kew's Herbarium - K001137318. Retrieved from Plants of the World Online

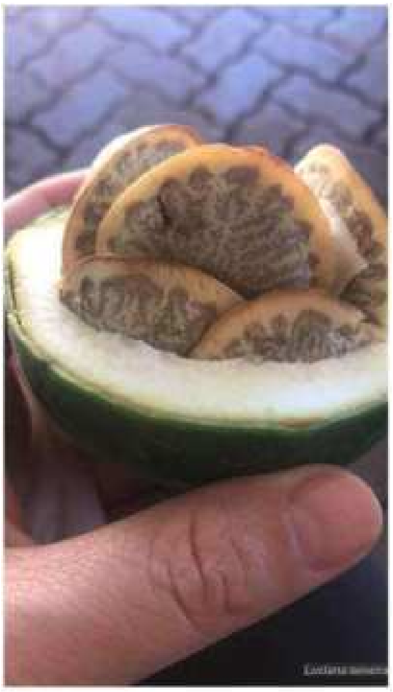

Published by: Fruit of *F. trilobata*. Published online by: Luis Fernando Paiva Lima in Flora e Funga do Brasil. Author: Luciana teixeira

Historiae Rerum Naturalium Brasiliae	Marcgrave, 1648	Page number	47

Vernacular name(s)	Planta
Species	Hibiscus furcellatus Desr.
Family	Malvaceae

Notes

The woodcut is different to the oil painting. The leaves of both images are completely different, although it could still be the same species as *H. furcellatus* can include intraspecific variability on their leaves.

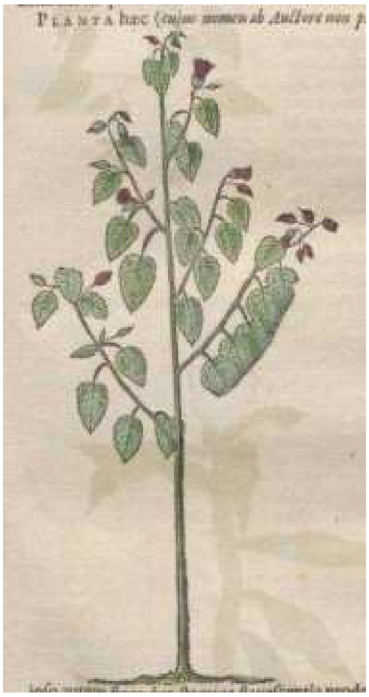

Historiae Plantarum – Herbis: 47

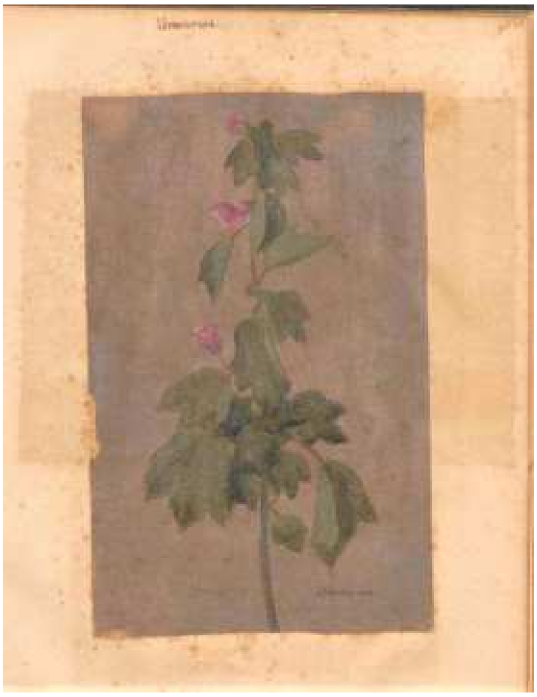

Theatrum Rerum Naturalium: 513

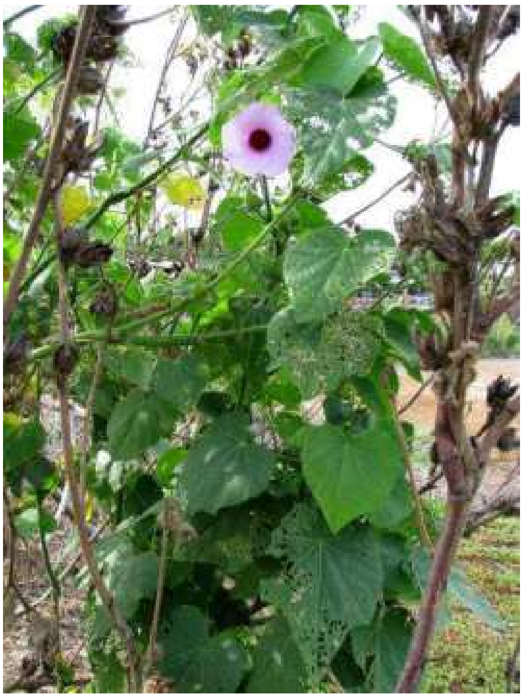

"*H. furcellatus*" by D.Eickhoff (CC BY 2.09

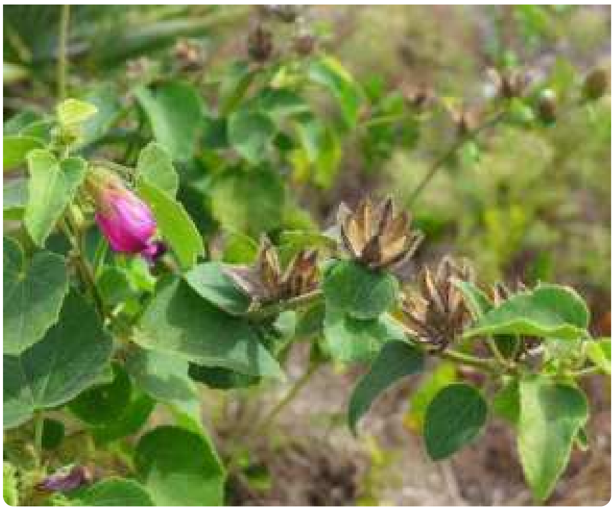

Flower and open fruits. "*Hibiscus furcellatus* 4" by Scott Zona (CC BY-NC 2.0)

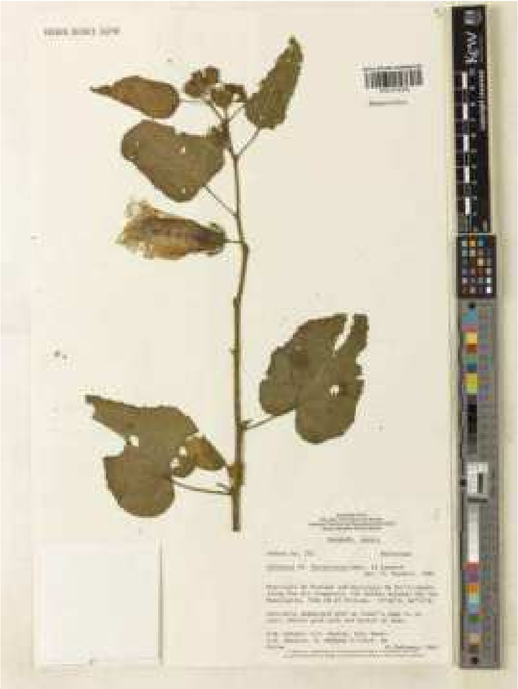

Specimen of *H. furcellatus* from Kew's Herbarium - K001218365. Retrieved from Plants of the World Online

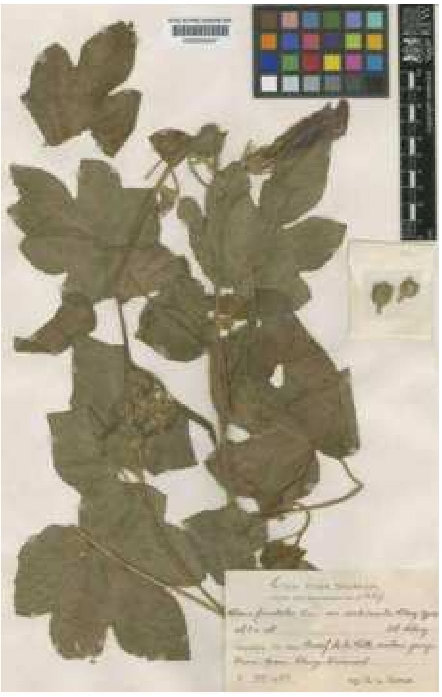

Specimen of *H. furcellatus* from Kew's Herbarium - K000535447. Retrieved from Plants of the World Online

Historiae Rerum Naturalium Brasiliae | Marcgrave, 1648 | Page number 48a

Vernacular name(s) Pino. Urticae Urentis species

Species Laportea aestuans (L.) Chew

Family Urticaceae

Notes

We did not find any correspondence between this woodcut and the contemporary or older sources.

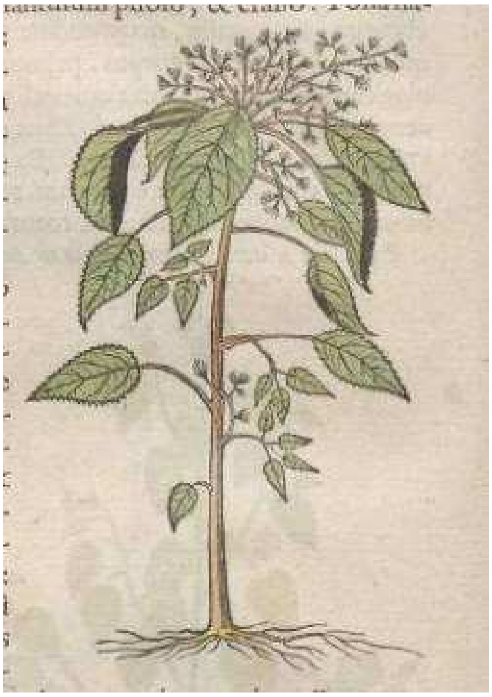

Historiae Plantarum – Herbis: 48a

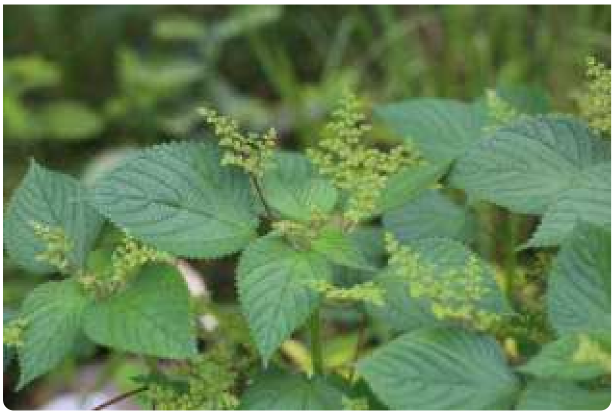

"*Laportea aestuans* (Urticaceae)" by Dr. Alexey Yakovlev (CC BY-SA 2.0)

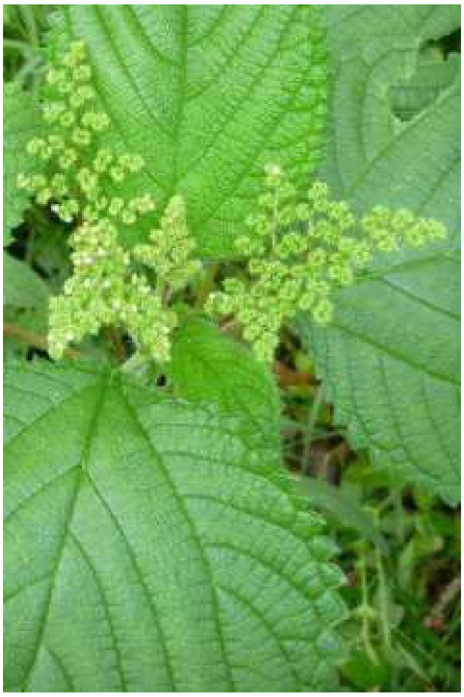

"*L. aestuans*" by IITA Image Library (CC BY-NC 2.0)

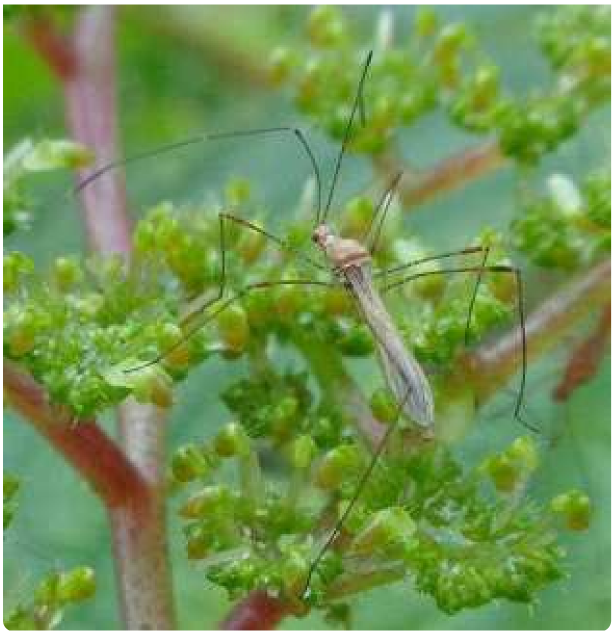

"*Heniptera* y *Laportea aestuans* (1)" by Gelo--2014 (CC BY-NC 2.0)

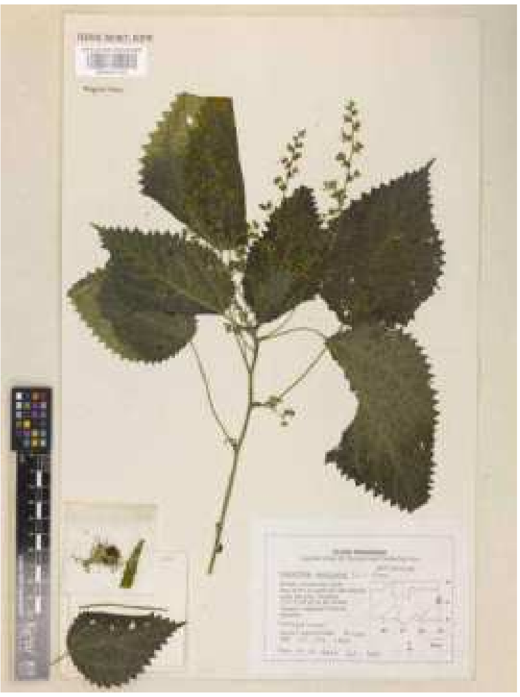

Specimen of *L. aestuans* from Kew's Herbarium - K000973183. Retrieved from Plants of the World Online

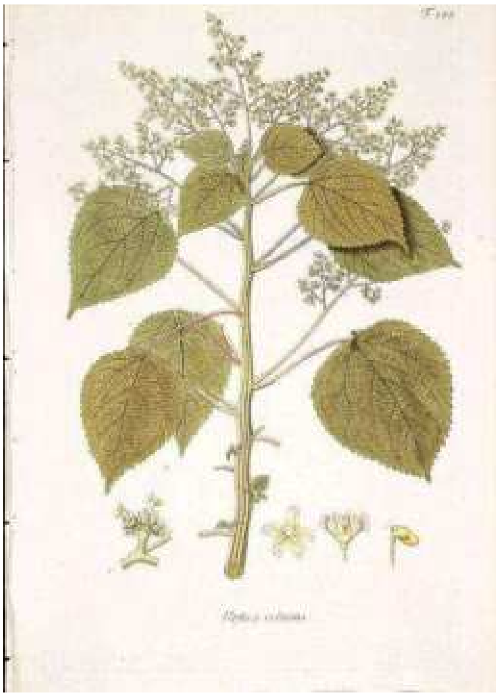

Plantarum rariorum horti caesarei Schoenbrunnensis descriptiones et icones by Jacquin, N.J. von (1798: Vol. III, t. 388). Missouri Botanical Garden, U.S.A.

Historiae Rerum Naturalium Brasiliae | Marcgrave, 1648 | Page number 48b

Vernacular name(s) Paco Caatinga. Cana do mato

Species Costus arabicus L.

Family Costaceae

Notes

The woodcut is slightly similar to the *Theatrum* image.

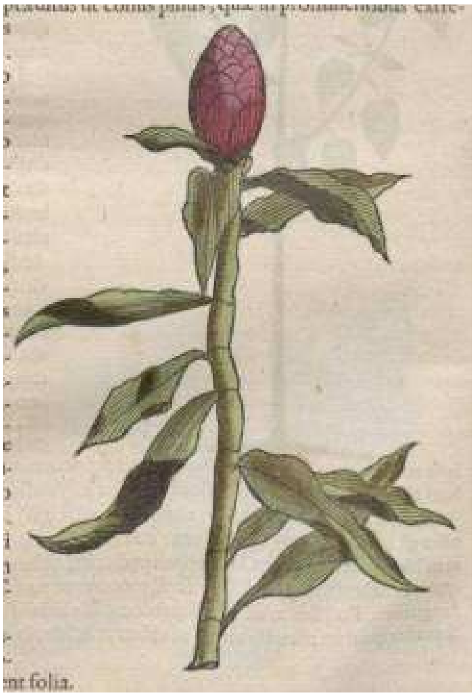

Historiae Plantarum – Herbis: 48b

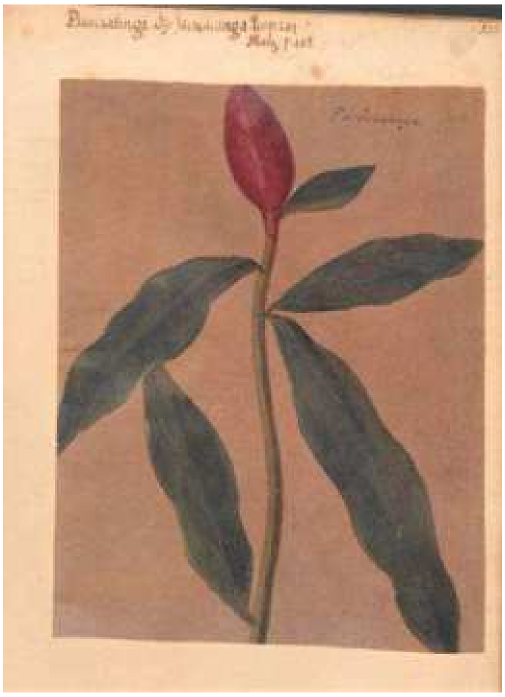

Theatrum Rerum Naturalium: 181

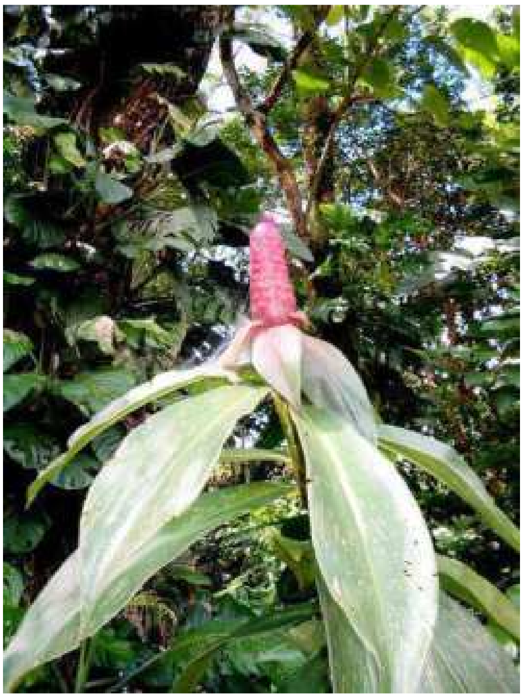

"*Costus arabicus* (Costaceae)" by Diego Rodriguez. Naturalist Photographer (CC BY 2.0)

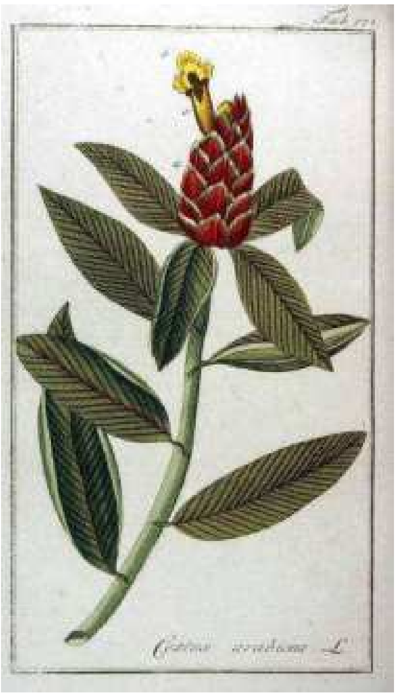

Engraving of *C. arabicus* in *Afbeeldingen der artseny-gewassen* by Zorn, J., Oskamp, D.L. (1800: Vol. V, t. 471). www.BioLib.de

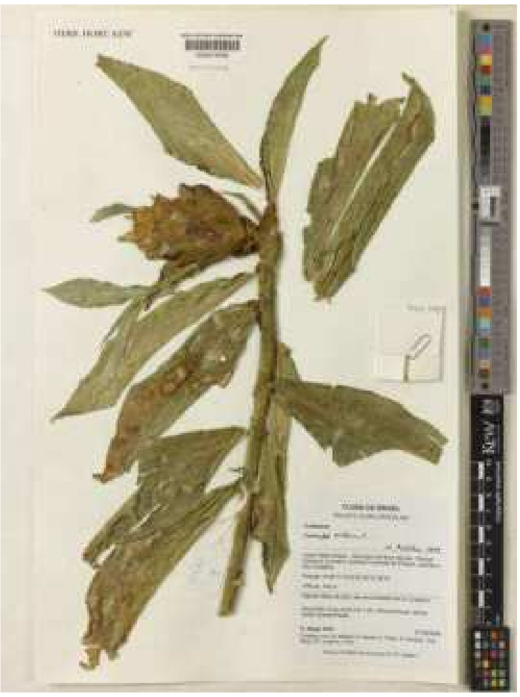

Specimen of *C. arabicus* collected in Brazil from Kew's Herbarium - K000579088. Retrieved from Plants of the World Online

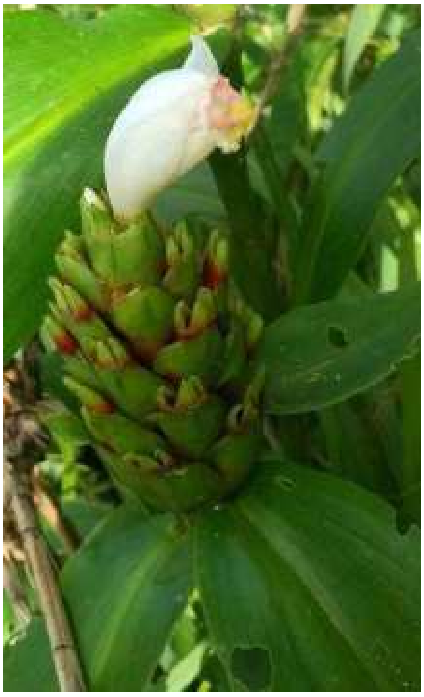

C. arabicus observed in Brazil for iNaturalist by Martin Acosta (CC BY-NC 4.0)

Historiae Rerum Naturalium Brasiliae Marcgrave, 1648 Page number 49a

Vernacular name(s) Paco Seroca

Species Renealmia alpinia (Rottb.) Maas

Family Zingiberaceae

Notes

The woodcut is very similar to the *Theatrum* image (non reversed).

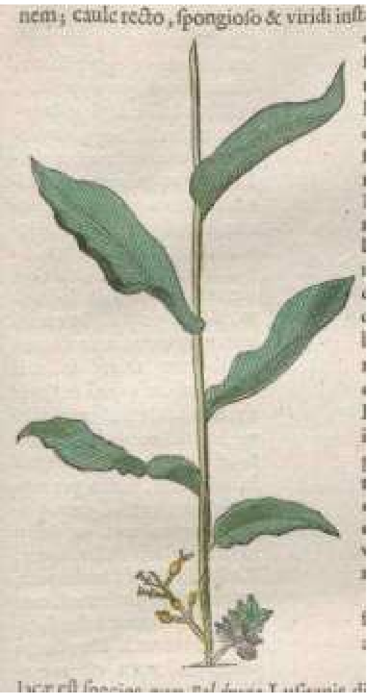

Historiae Plantarum – Herbis: 49a

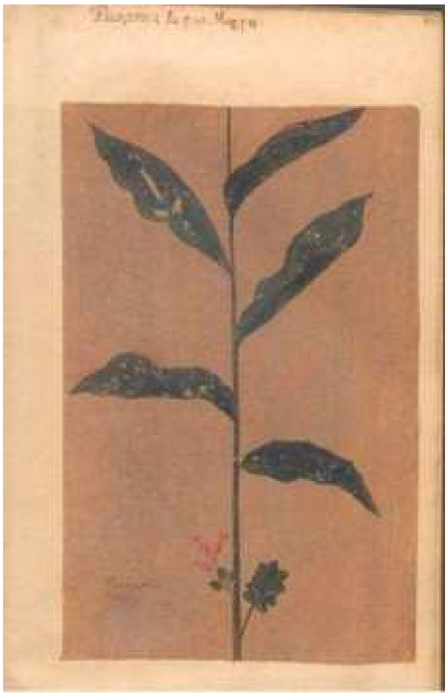

Theatrum Rerum Naturalium: 177

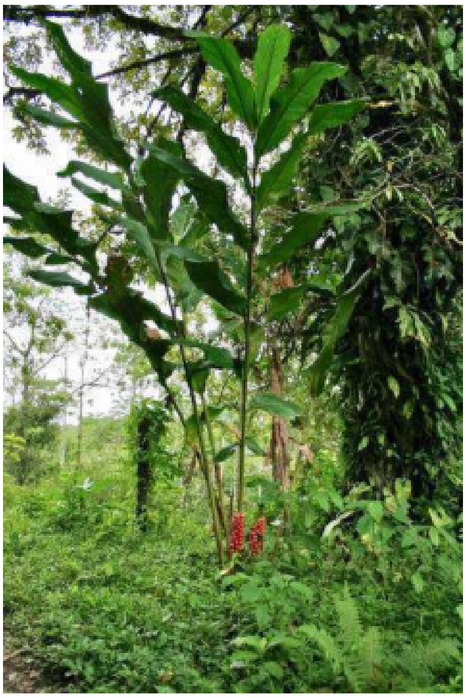

"*R. alpinia* (Zingiberaceae)" by Dr. Alexey Yakovlev (CC BY-SA 2.0)

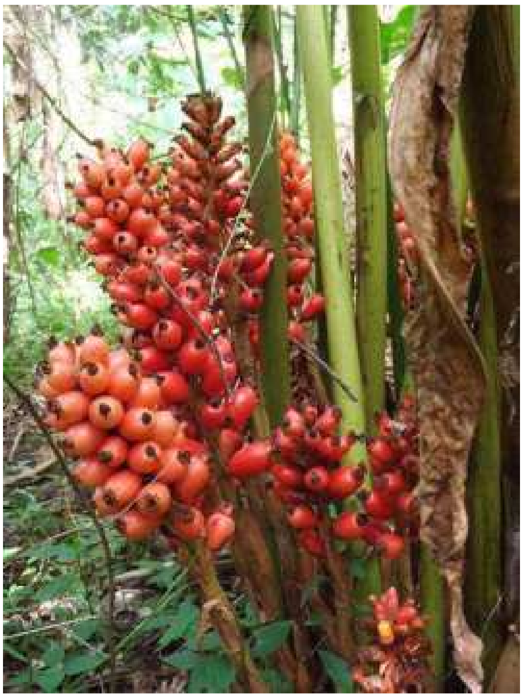

"*R. alpinia* (Family Zingiberaceae)" by Dis da fi we (CC BY-NC-SA 2.0)

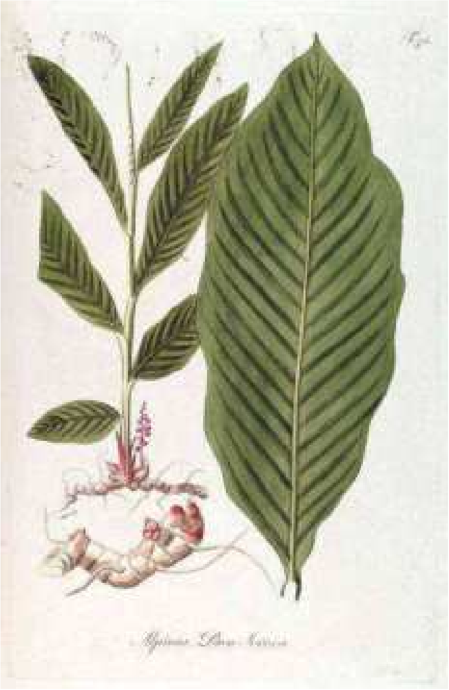

Fragmenta botanica, figuris coloratis illustrata by Jacquin, N.J. von (1809: 76). Missouri Botanical Garden, St. Louis, U.S.A.

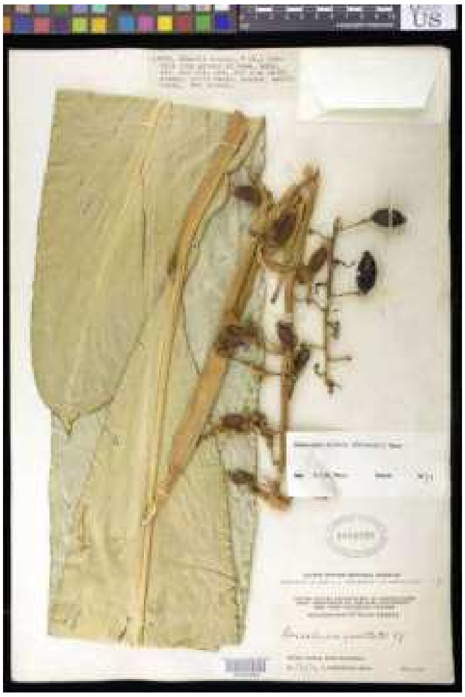

Specimen. "*R. alpinia*" by Albert S. Hitchcock -00336450- Smithsonian National Museum of Natural History (CC0 1.0)

Historiae Rerum Naturalium Brasiliae Marcgrave, 1648 Page number 49b

Vernacular name(s) Caaponga. Bel droga

Species Portulaca halimoides L.

Family Portulacaceae

Notes

The resemblance between woodcut and specimen is hard to tell because the specimen is not that well preserved. They are both flowering but the specimen does not have its leaves, as these could have been lost over time.

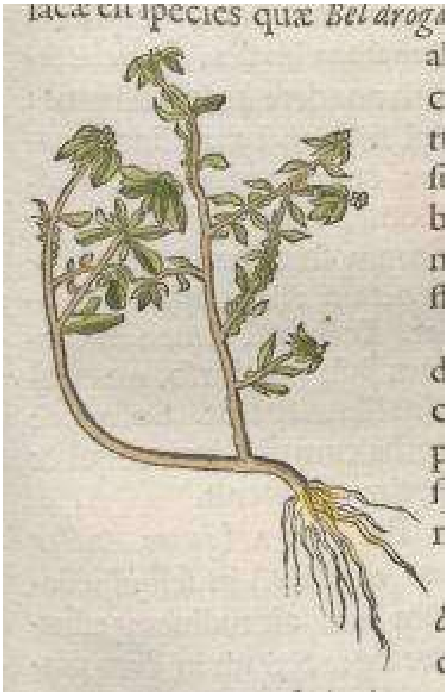

Historiae Plantarum – Herbis: 49b

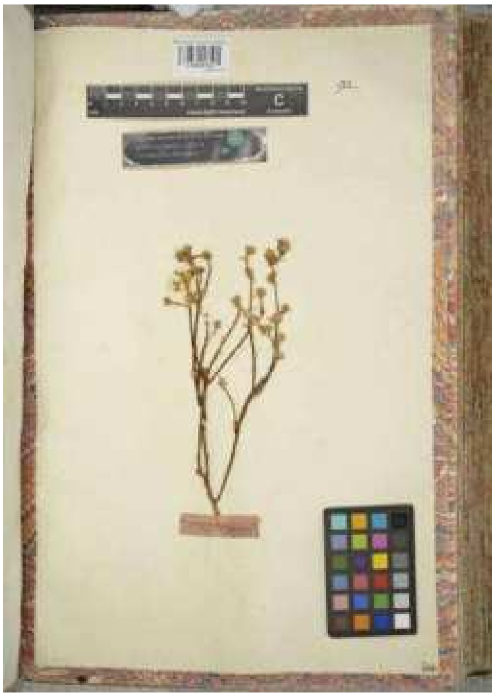

Marcgrave's herbarium: 104

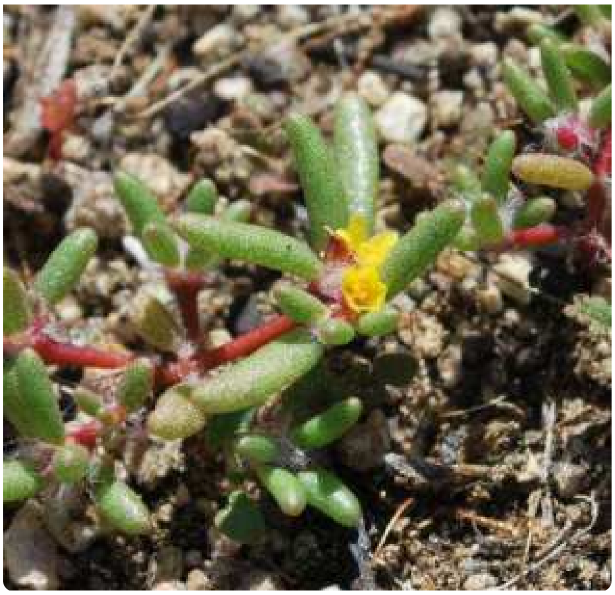

"*P. halimoides*; Silkcotton purslane, desert portulaca, Purslane" by Don M. Davis (CC BY-NC-ND 2.0)

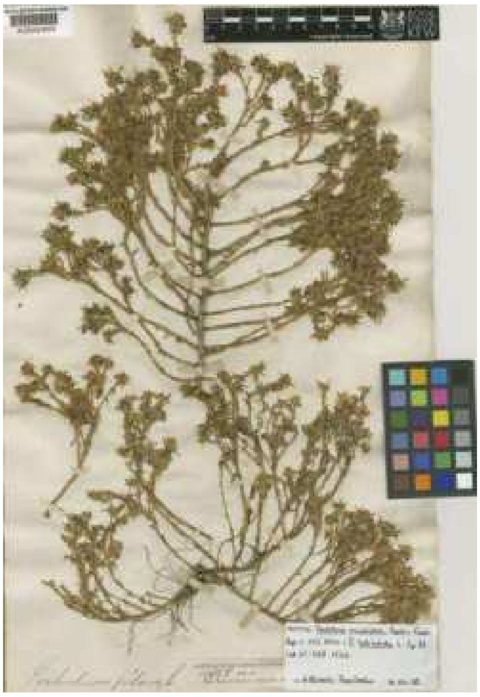

Specimen of *P. halimoides* from Kew's Herbarium - K000424620. Retrieved from Plants of the World Online

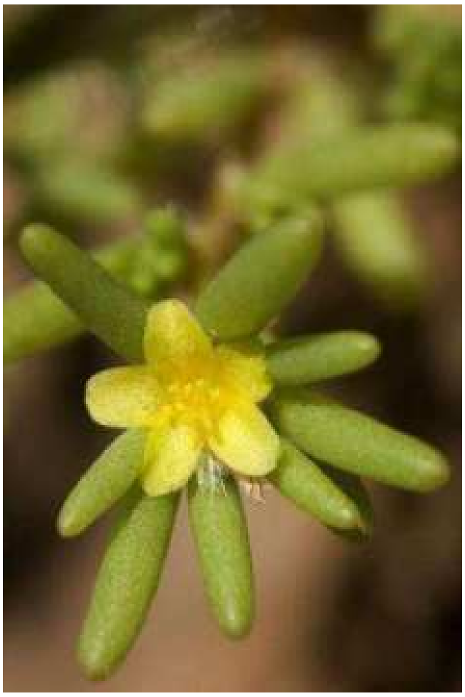

Flower. "*P. halimoides*" by aspidoscelis (CC0 1.0)

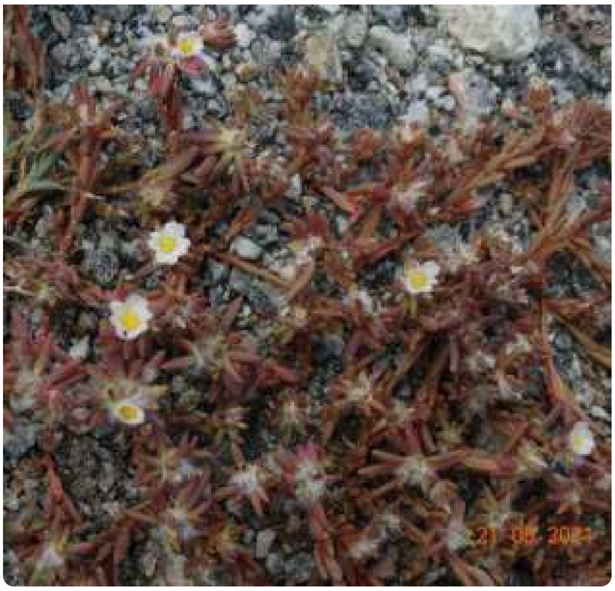

P. halimoides observed in Brazil for iNaturalist by M. A. Lagares (CC BY-NC 4.0)

Historiae Rerum Naturalium Brasiliae	Marcgrave, 1648	Page number	49c

Vernacular name(s) Planta

Species Centratherum punctatum Cass.

Family Asteraceae

Notes

We did not find any correspondence between this woodcut and the contemporary or older sources.

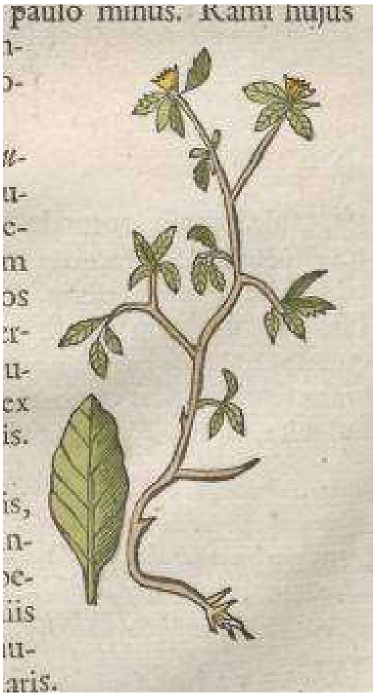

Historiae Plantarum – Herbis: 49c

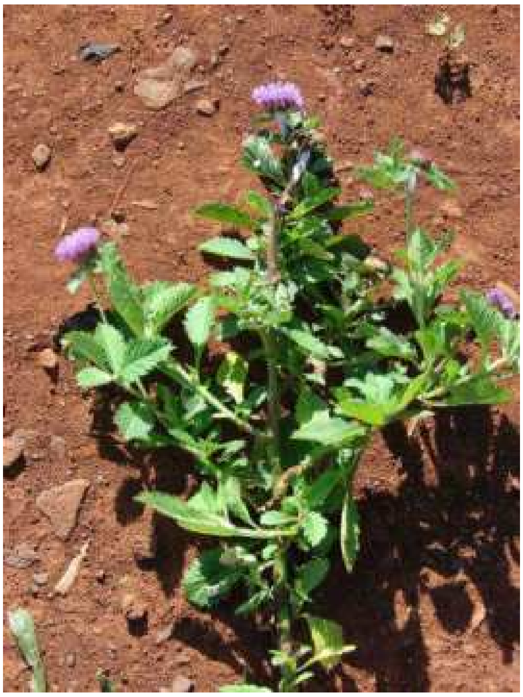

"File:Starr 070402-6336 *C. punctatum*.jpg" by Forest & Kim Starr (CC BY 3.0)

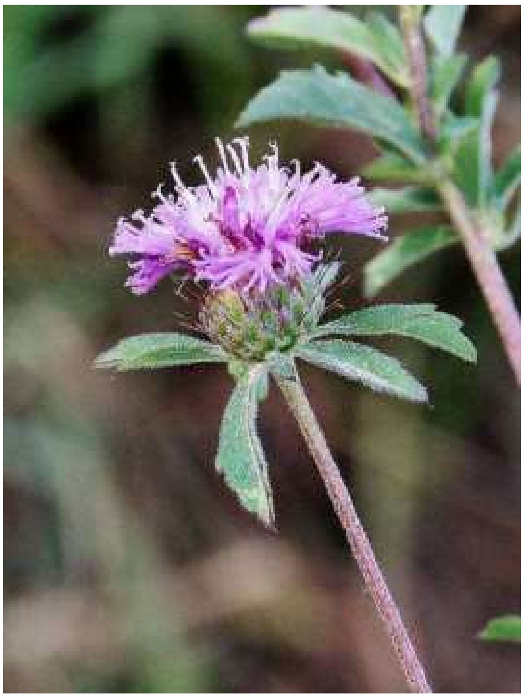

Flower calyx. "*C. punctatum*" by Mauricio Mercadante (CC BY-NC-SA 2.0)

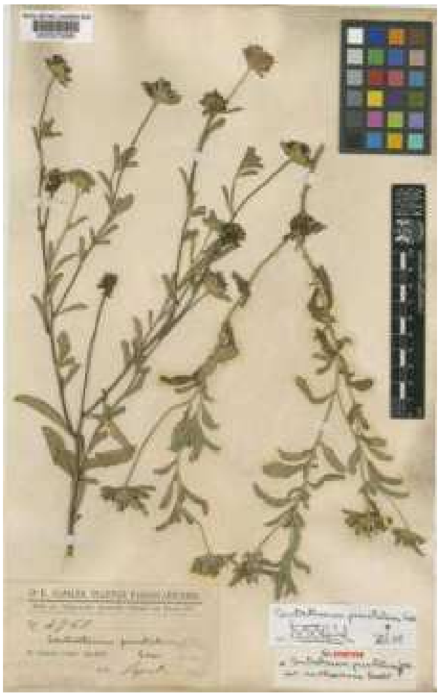

Specimen from Kew's Herbarium - K000373089. Retrieved from Plants of the World Online

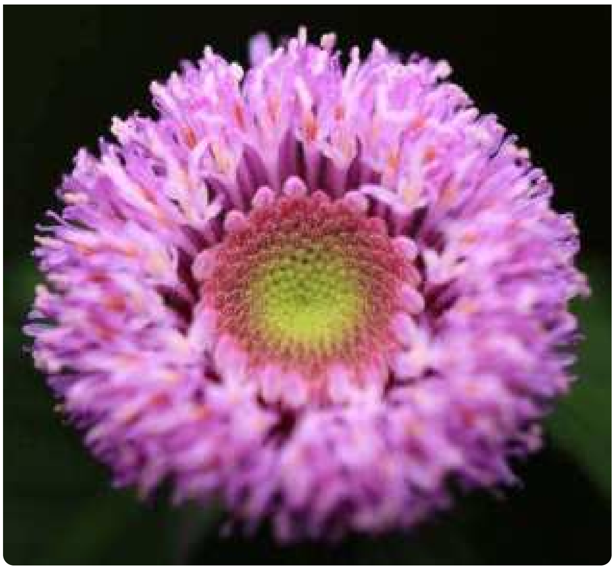

"Brazilian Buttonflower / *Centratherum punctatum* by TANAKA Juuyoh (田中十洋) (CC BY 2.0)

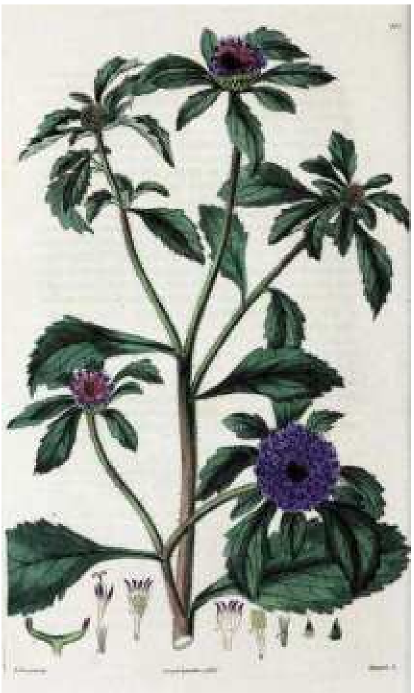

Engraving of *C. punctatum* in *British flower garden* by Sweet, R. (1827-1829: Vol. III, t. 225). Getty Research Institute, Los Angeles, U.S.A.

Historiae Rerum Naturalium Brasiliae | Marcgrave, 1648 | Page number 50

Vernacular name(s): Nhambi

Species: Eryngium foetidum L.

Family: Apiaceae

Notes

We did not find any correspondence between this woodcut and the contemporary or older sources. It could be that, even though the description and vernacular name correspond to *E. foetidum*, the woodcut image represents another species. Likely a member of the Asteraceae family.

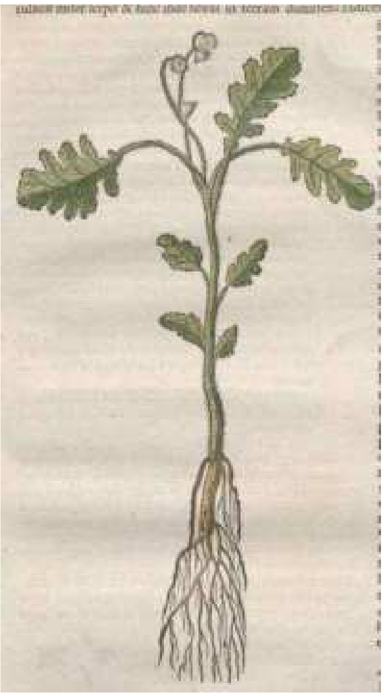

Historiae Plantarum – Herbis: 50

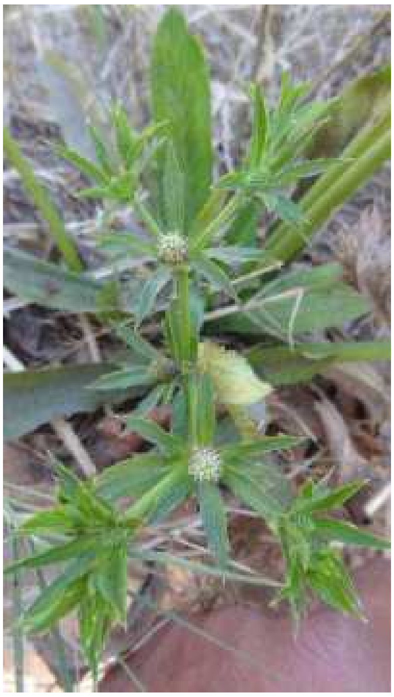

"*Eryngium foetidum* (2)" by siddarth.machado (CC BY-NC 2.0)

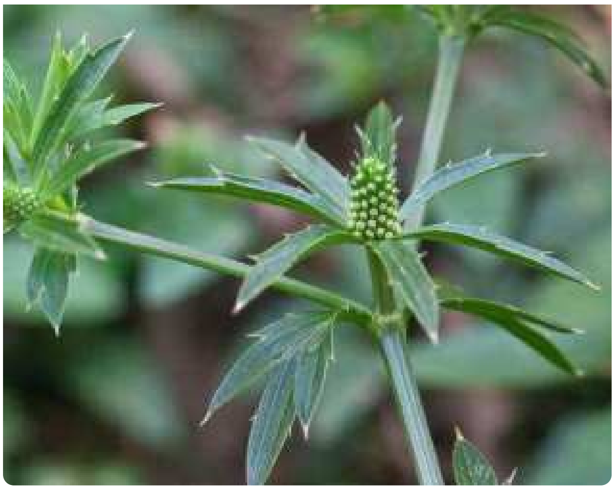

"*E. foetidum*" by Andres Hernandez S. (CC BY-NC-SA 2.0)

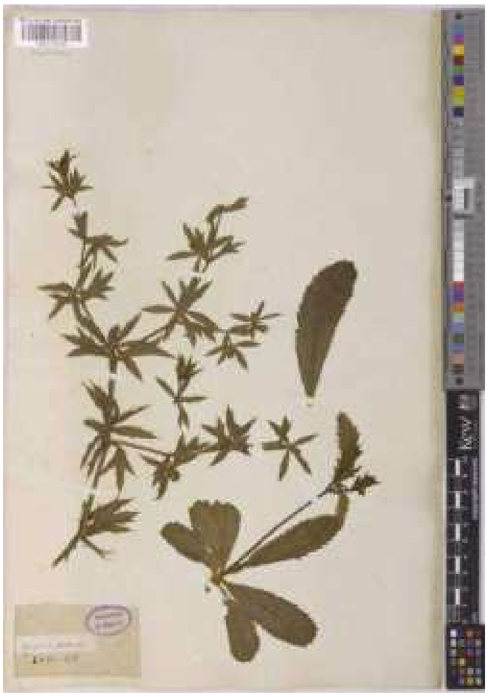

Specimen of *E. foetidum* from Kew's Herbarium - K001130392. Retrieved from Plants of the World Online

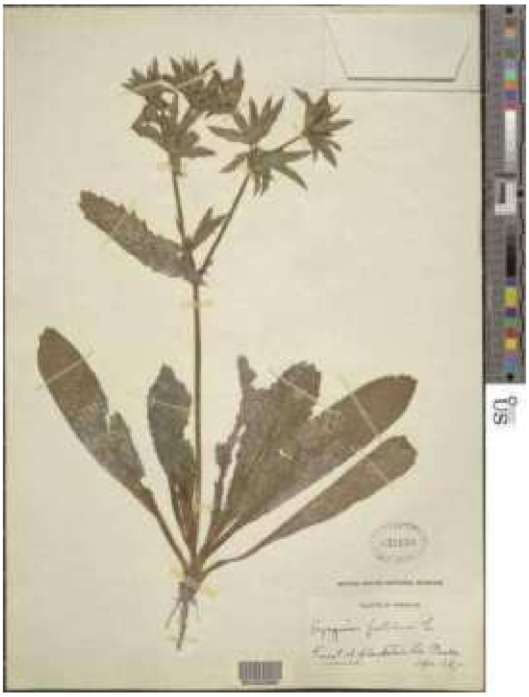

Specimen. "*E. foetidum*" by Jacob A. Samuels -00468968- Smithsonian National Museum of Natural History (CC0 1.0)

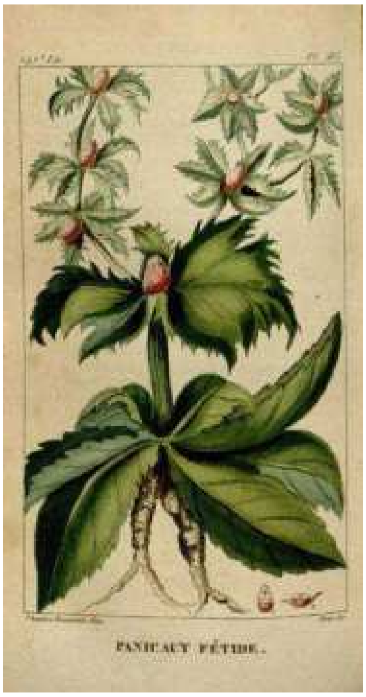

Flore [pittoresque et] médicale des Antilles by Descourtilz, M.E. (1829: Vol. 8, t. 585). Missouri Botanical Garden, St. Louis, U.S.A.

Historiae Rerum Naturalium Brasiliae Marcgrave, 1648 Page number 51a

Vernacular name(s) Planta

Species Goeppertia effusa Saka & Lombardi

Family Marantaceae

Notes

We did not find any correspondence between this woodcut and the contemporary or older sources.

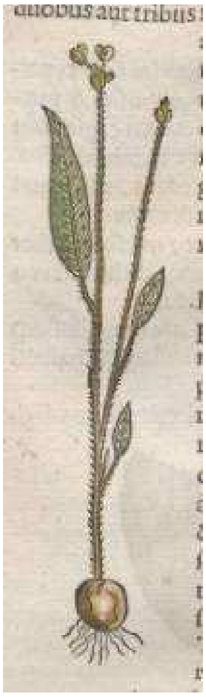

Historiae Plantarum – Herbis: 51a

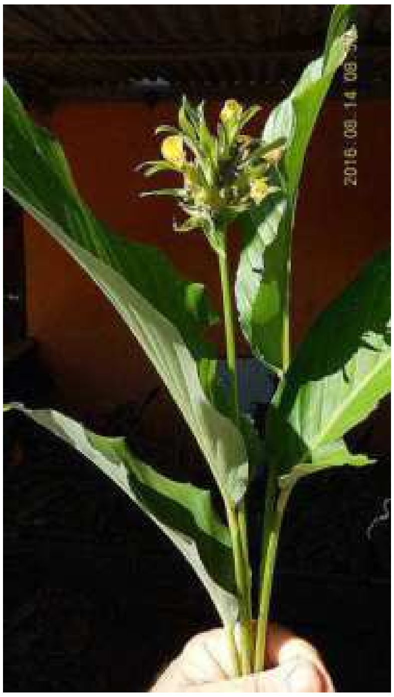

"*Goeppertia effusa*" by Alex Popovkin, Bahia, Brazil (CC BY-NC-SA 2.0)

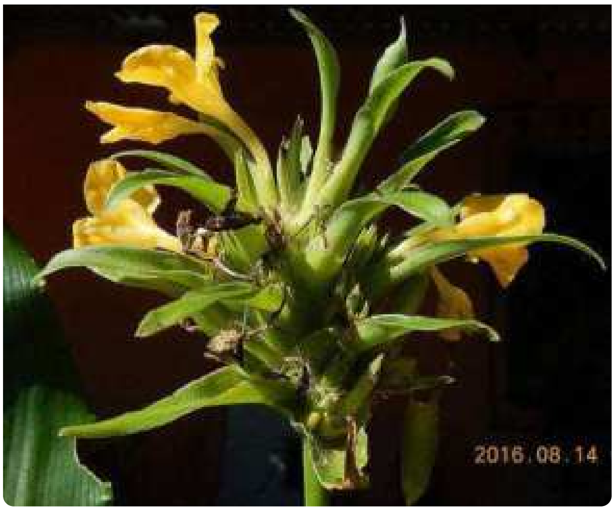

"*Goeppertia effusa*" by Alex Popovkin, Bahia, Brazil
(CC BY-NC-SA 2.0)

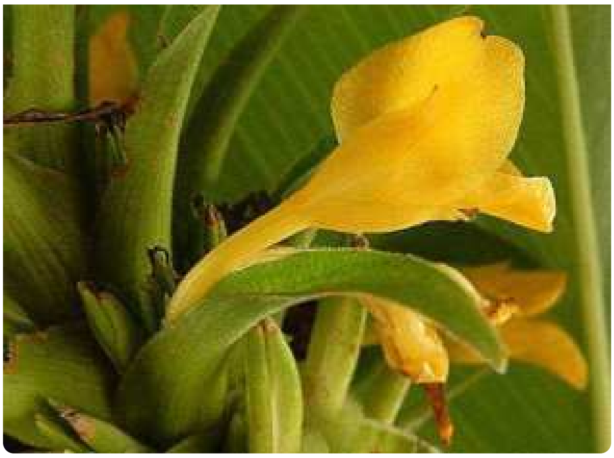

"*Goeppertia effusa*" by Alex Popovkin, Bahia, Brazil
(CC BY-NC-SA 2.0)

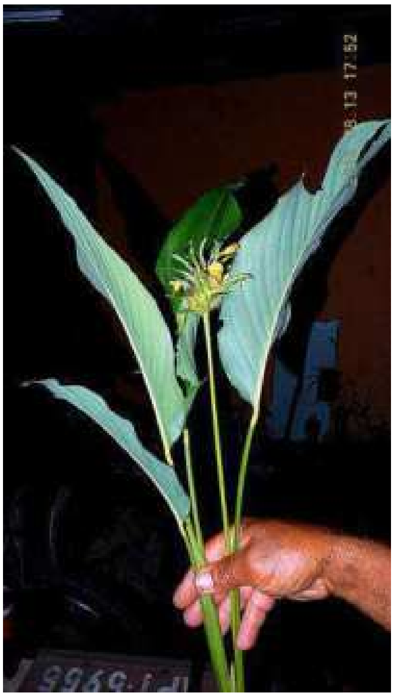

"*Goeppertia effusa*" by Alex Popovkin, Bahia, Brazil
(CC BY-NC-SA 2.0)

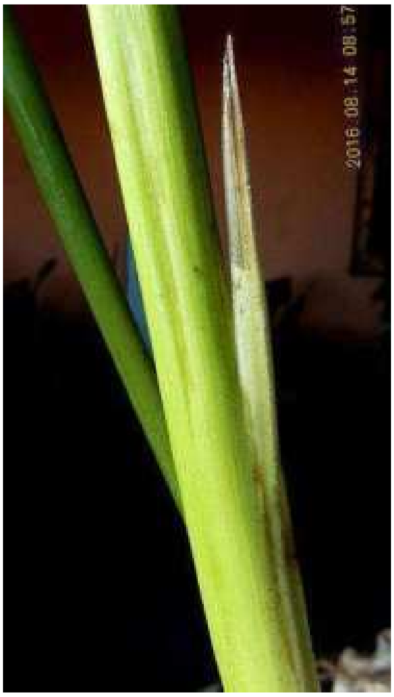

"*Goeppertia effusa*" by Alex Popovkin, Bahia, Brazil
(CC BY-NC-SA 2.0)

Historiae Rerum Naturalium Brasiliae	Marcgrave, 1648	Page number	51b

Vernacular name(s) Planta

Species Unknown

Family Unknown

Notes

We did not find any correspondence between this woodcut and the contemporary or older sources. We could not identify the plant represented by the woodcut, hence we could not cross-reference it with the visual sources. Accurate identification of such plant will facilitate this analysis and provide us with more insights about its origin.

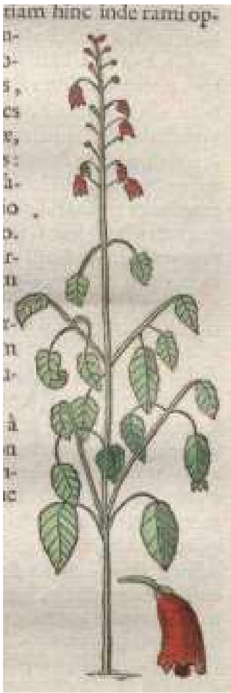

Historiae Plantarum – Herbis: 51b

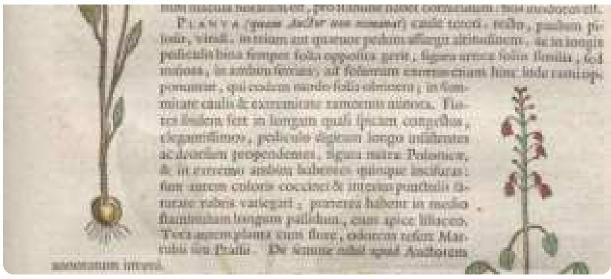

HNB (Marcgrave 1648: 51) Latin edition

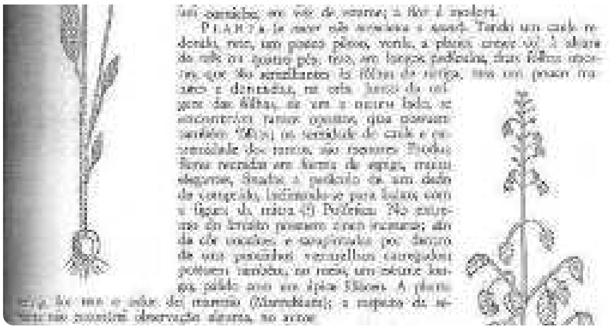

[illegible]

HNB (1942 [1648]) Portuguese edition

"Having a round, straight, slightly hairy, green stem, the plant grows to the height of three or four feet: it has, on long pedicles, two opposite leaves, which are similar to the nettle leaves, but a little larger and toothed, on the edge. Near the origin of the leaves, on either side, are opposite branches, which also have leaves; in the thickness of the stem and end of the branches, they are smaller. It produces very elegant flowers gathered in a spike; fixed to the pedicle of a long finger, sloping downwards, with the figure of the Polish miter. At the end of the scope they also have in the middle, a long pale stamen, with a lily-like apex. The plant, with the flower, has the odor of the heather (Marrubium); regarding the seed I found no observation, in the author"

English translation, by M. Alcantara-Rodriguez

Historiae Rerum Naturalium Brasiliae | Marcgrave, 1648 | Page number 51c

Vernacular name(s) Convolvulus marinus. Soldanella

Species Ipomoea pes-caprae (L.) R. Br.

Family Convolvulaceae

Notes

The woodcut does not show a resemblance to any of those sources and it is also different from the woodcut placed in Piso (1648) for the same species. Interestingly, an image of this species was previously published in de l' Obel (1591: vol. I, p. 602), and named *Convolvulus Soldanella*. The woodcuts differ, thus the HNB is presenting new woodcut images of this plant, likely made after nature in Brazil.

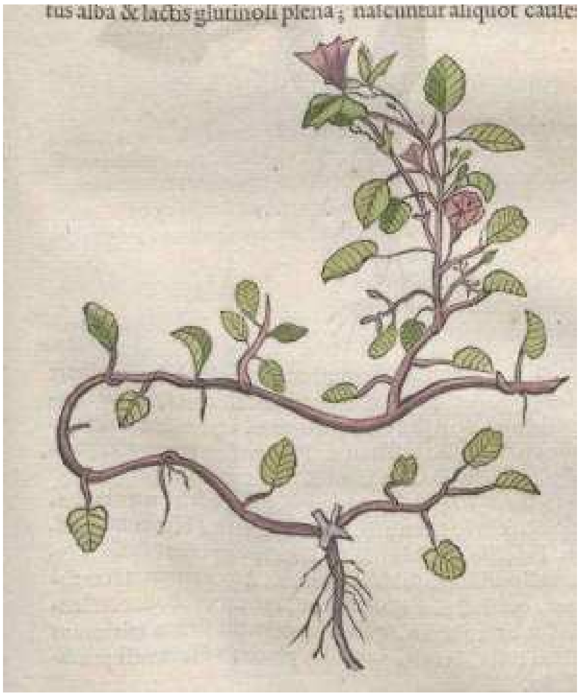

Historiae Plantarum – Herbis: 51c

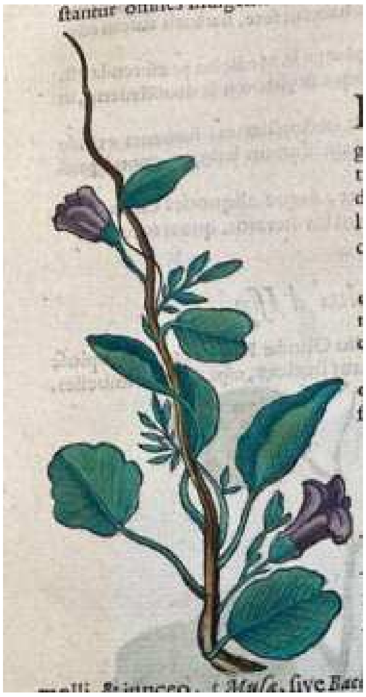

The same species represented by a different woodcut in the HNB (Piso 1648: 103)

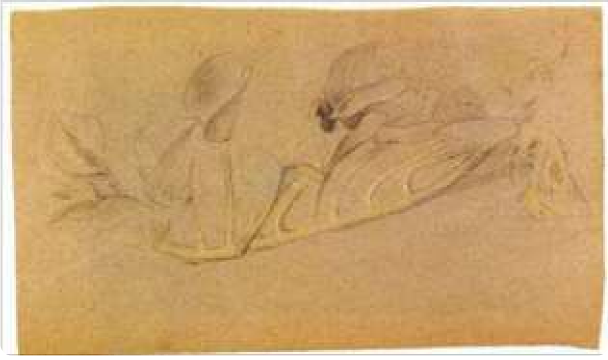

Misc. Cleyeri: 12v

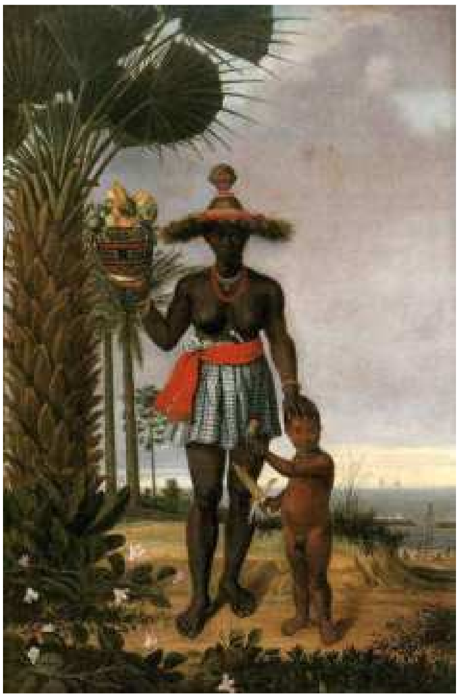

Creeping vine *I. pes-caprae* in the foreground of the Portrait "African woman" by Eckhout, ca. 1641. National Museum of Copenhagen, Denmark

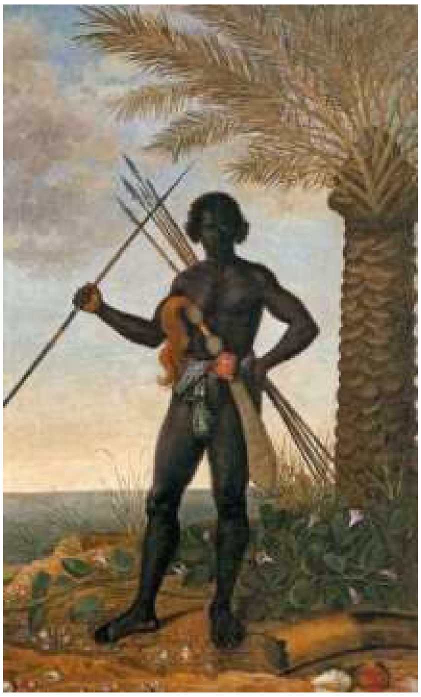

Portrait "African man" by Eckhout, ca. 1641. National Museum of Copenhagen, Denmark

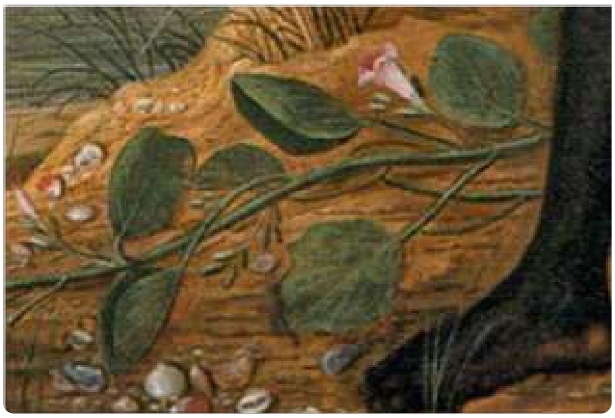

Close - up of *I. pes-caprae* in the portrait by Eckhout

Historiae Rerum Naturalium Brasiliae Marcgrave, 1648 Page number 52

Vernacular name(s) Caa apia

Species Dorstenia brasiliensis Lam.

Family Moraceae

Notes

The woodcut is very similar to the *Theatrum* image (non-reversed). The woodcut depicts its roots, as in the specimen in the herbarium, and it could have been made after it, although the flower is absent in the latter.

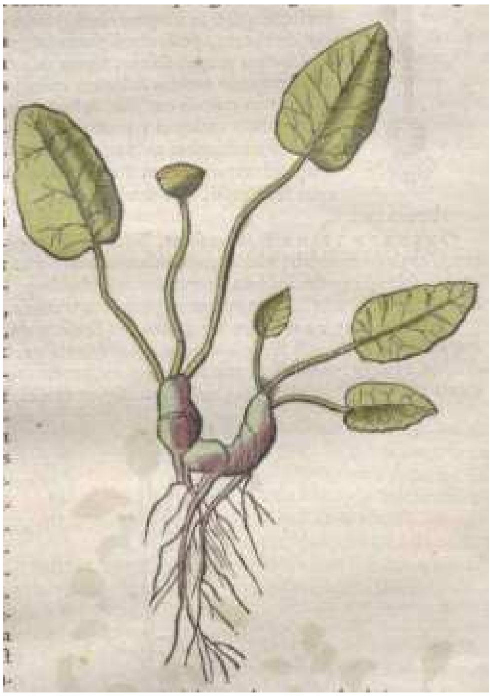

Historiae Plantarum – Herbis: 52

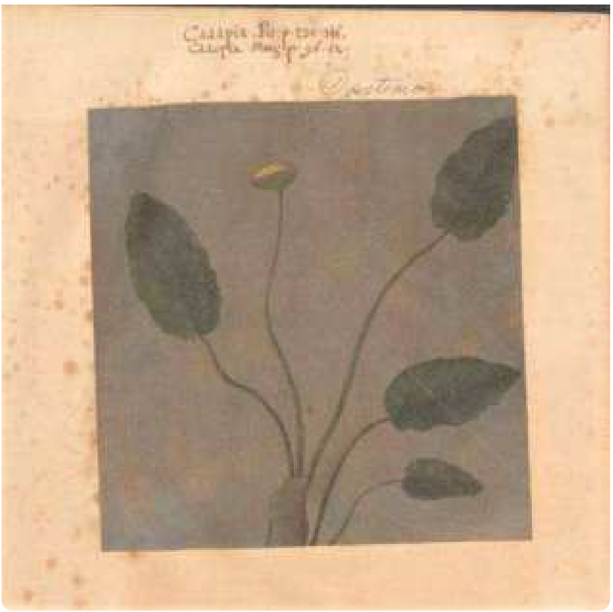

Theatrum Rerum Naturalium: 511

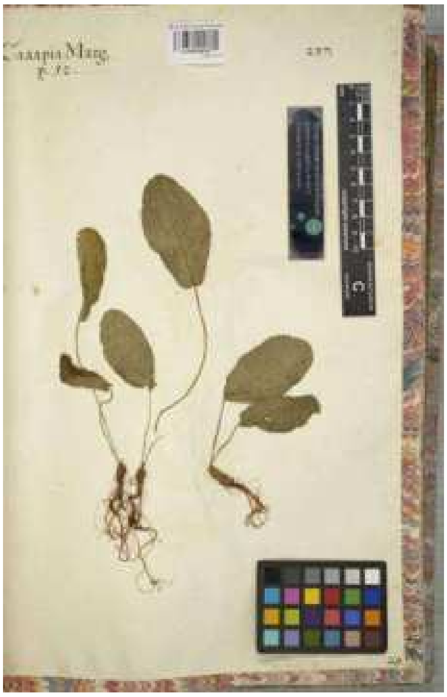

Marcgrave's herbarium: 29

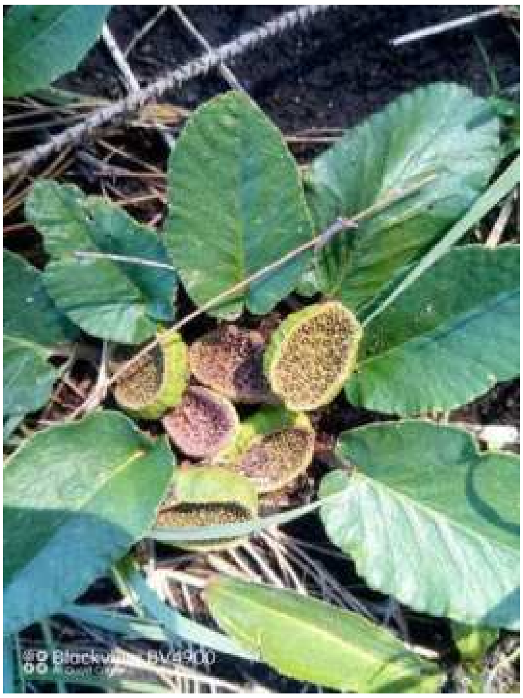

D. brasiliensis observed in Uruguay
for iNaturalist by Cruz Raven (CC BY-NC 4.0)

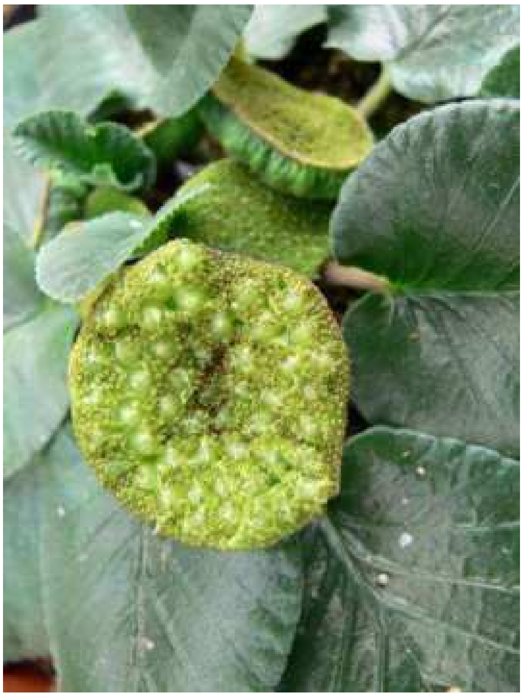

D. brasiliensis. Trinidad, Tobago. Aufnahme aus dem botanischen Garten der Ruhruniversität Bochum. Winfried Bruenken (Amrum). (CC-BY-SA-2.5)

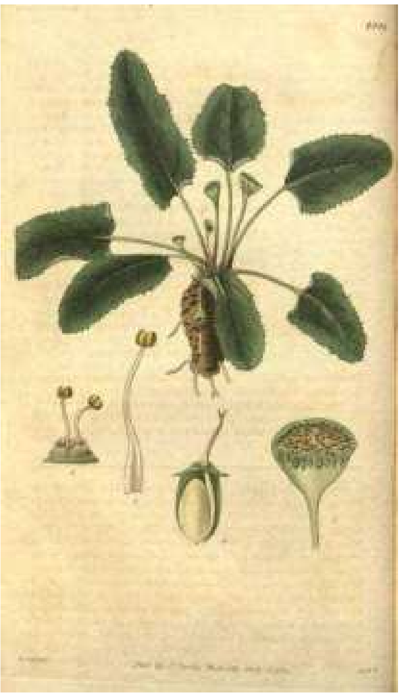

Engraving of *D. brasiliensis* in *Botanical Magazine* by Curtis, W. (1828: Vol. 55, t. 2804). Missouri Botanical Garden, St. Louis, U.S.A.

Historiae Rerum Naturalium Brasiliae | Marcgrave, 1648 | Page number 53a

Vernacular name(s) Agutiguepo-obi

Species Heliconia cf. acuminata A.Rich.

Family Heliconaceae

Notes

We did not find any correspondence between this woodcut and the contemporary or older sources. There is a plant identified by Buvelot et al. (2004) as *Heliconia psittacorum* L.f. in Eckhout's portrait "Mameluke with a basket of flowers", ca. 1641. The figure in the HNB does not show its impressive flowers, so it is hard to make a firm identification, but we follow the identification by Brazilian botanist Pickel (2008: 60) and we show here its relative, *H. psittacorum*, depicted by Eckhout.

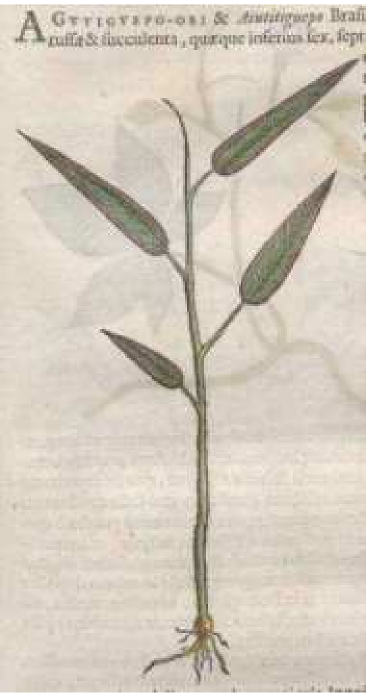

Historiae Plantarum – Herbis: 53a

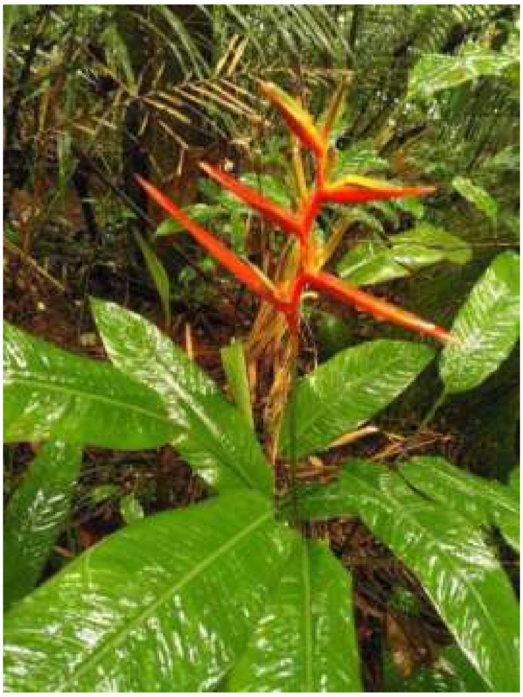

H. acumintata observed in French Guiana
for iNaturalist by alexennis269 (CC BY-NC 4.0)

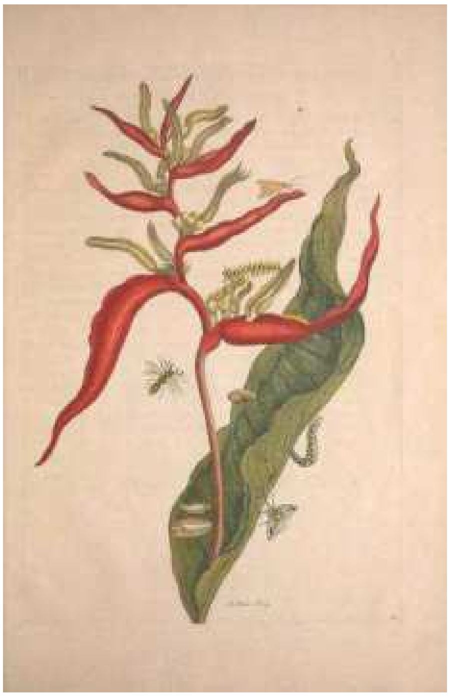

H. acuminata in *Metamorphosibus insectorum Surinamensium* by Merian, M.S. (1714: t. 54). Smithsonian Institute, Washington

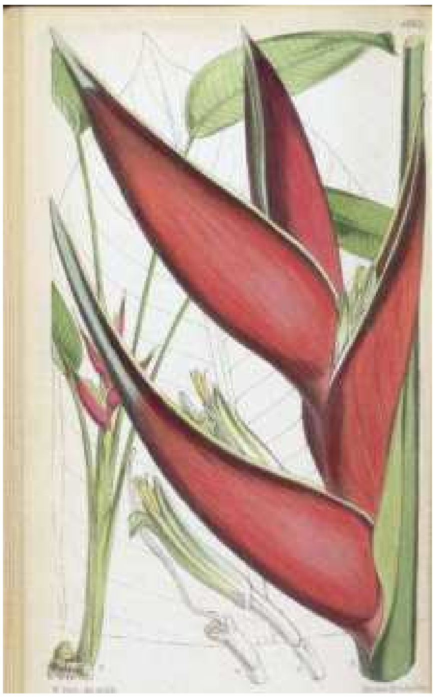

H. psittacorum in Botanical Magazine by Curtis, W. (1866: Vol. 92, t. 5613). Retrieved from Plants of the World Online

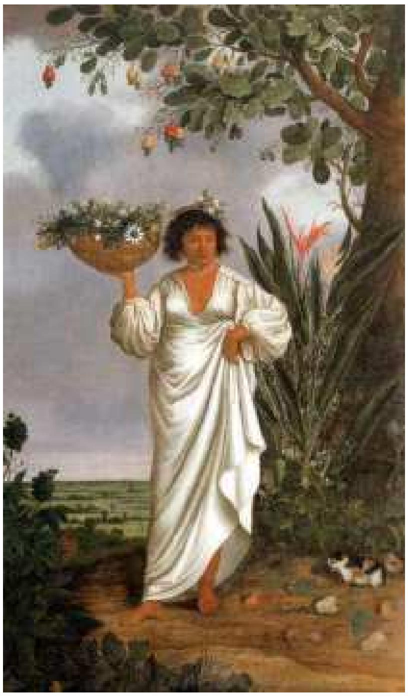

Portrait "Mameluke with a basket of flowers" by Eckhout, ca. 1641. National Museum of Copenhagen, Denmark

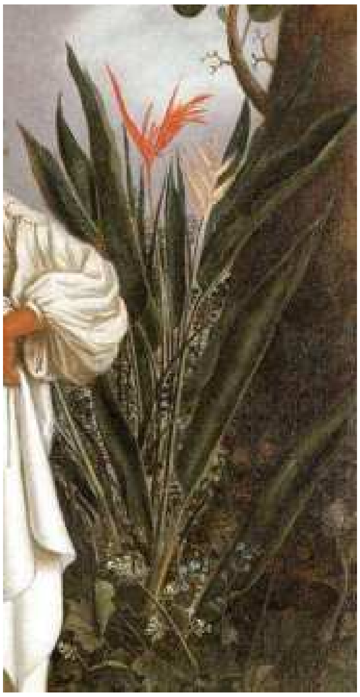

Close - up of *H. psittacorum* in the portrait by Eckhout

Historiae Rerum Naturalium Brasiliae Marcgrave, 1648 Page number 53b

Vernacular name(s) Planta

Species Eichhornia paniculata (Spreng.) Solms

Family Ponteridaceae

Notes

The woodcut differs from the *Theatrum* image. There is some resemblance between the woodcut and the flowering specimens in the herbarium. These could have been used to design the image the woodcut was based on.

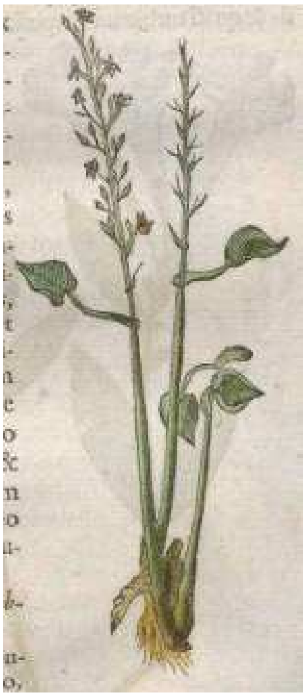

Historiae Plantarum – Herbis: 53b

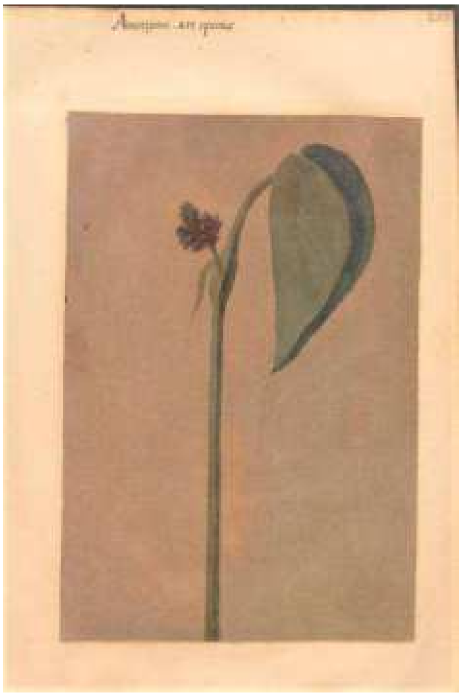

Theatrum Rerum Naturalium: 253

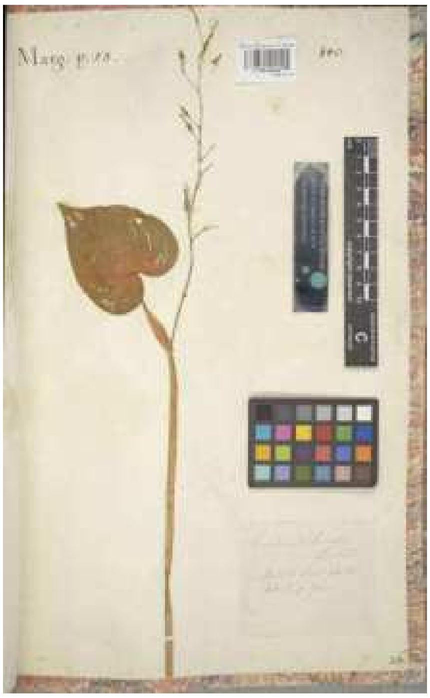

Marcgrave's herbarium: 26

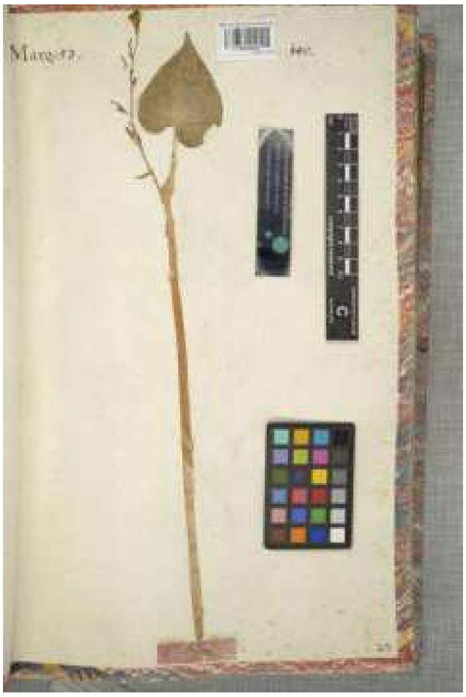

Marcgrave's herbarium: 27

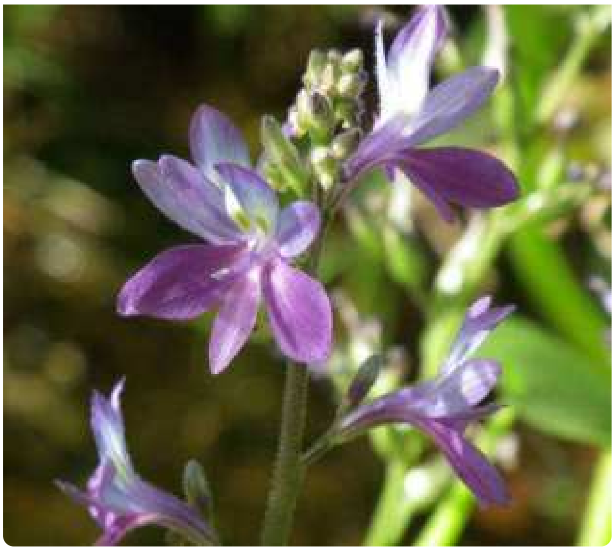

Flowers. *E. paniculata* observed in Brazil for iNaturalist by Frederico Acaz Sonntag (CC BY-NC 4.0)

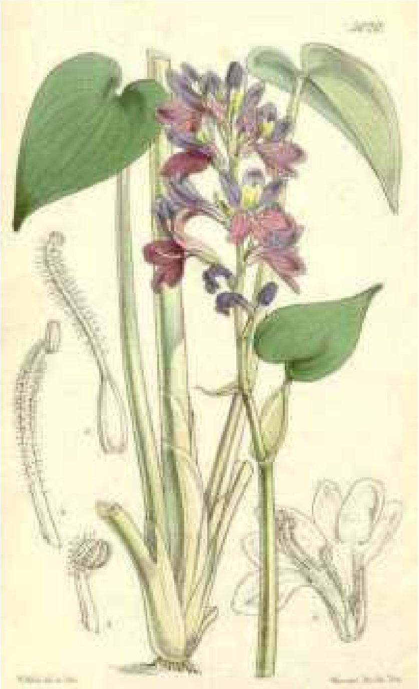

Botanical Magazine by Curtis, W. Vol. 83, t. 5020) Missouri Botanical Garden, St. Louis, U.S.A.

Historiae Rerum Naturalium Brasiliae	Marcgrave, 1648	Page number	53c

Vernacular name(s) Tamoatarana

Species Myrosma cannifolia L.f.

Family Marantaceae

Notes

We did not find any correspondence between this woodcut and the contemporary or older sources.

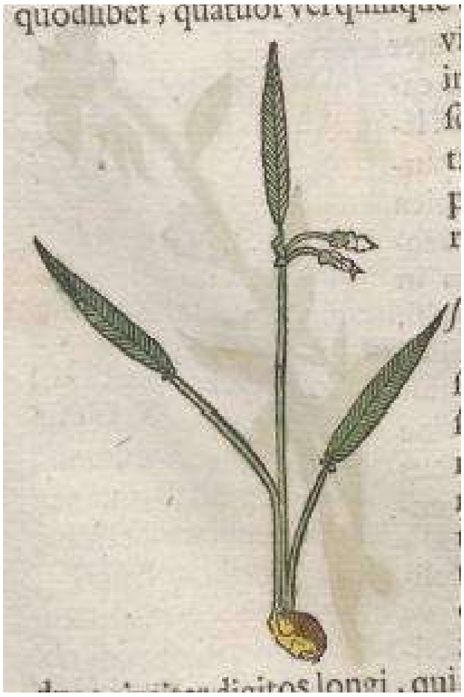

Historiae Plantarum – Herbis: 53c

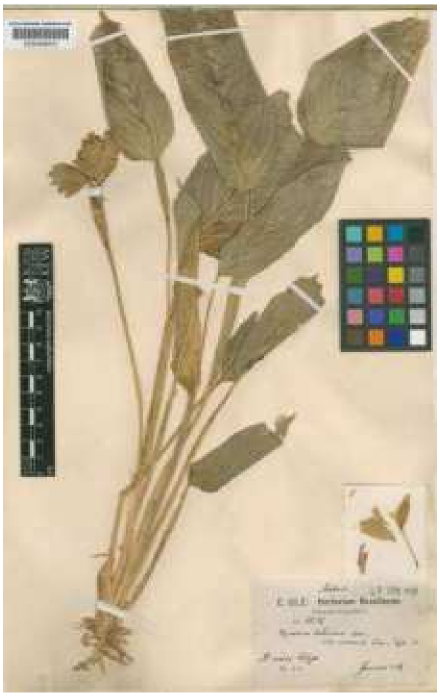

Specimen of *M. cannifolia* from Kew's Herbarium - K000586912. Retrieved from Plants of the World Online

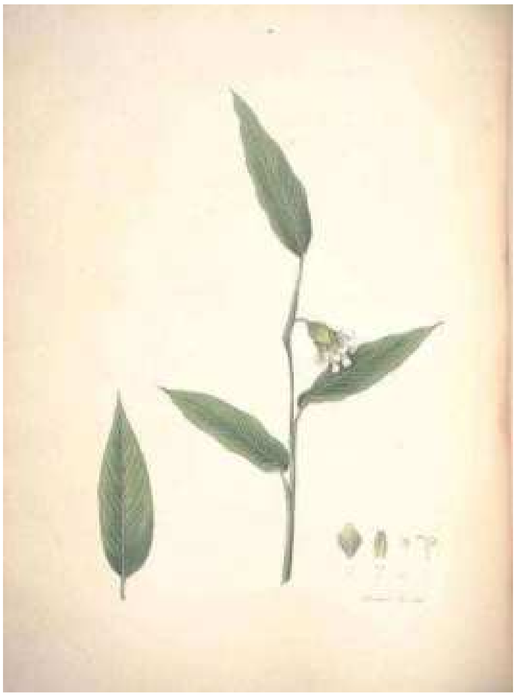

M. cannifolia in *Monandrian plants of the order Scitamineae* by Roscoe, W. (1828). Missouri Botanical Garden, St. Louis, U.S.A.

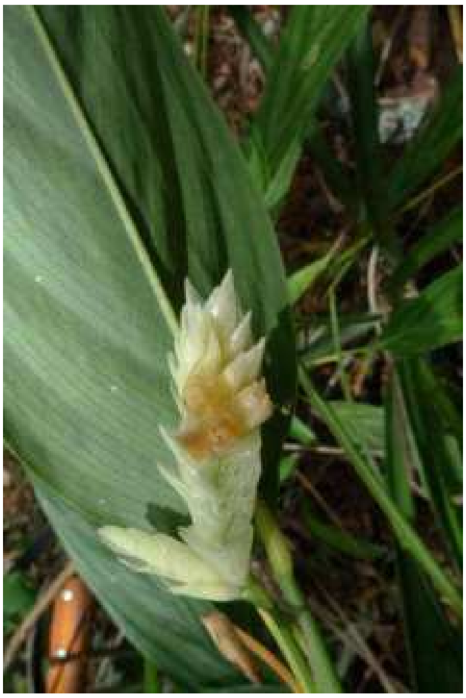

Myrosma cannifolia L.f. - Specimen W. Milliken 4236 ID:1108149 © RBG Kew (CC BY 3.0)

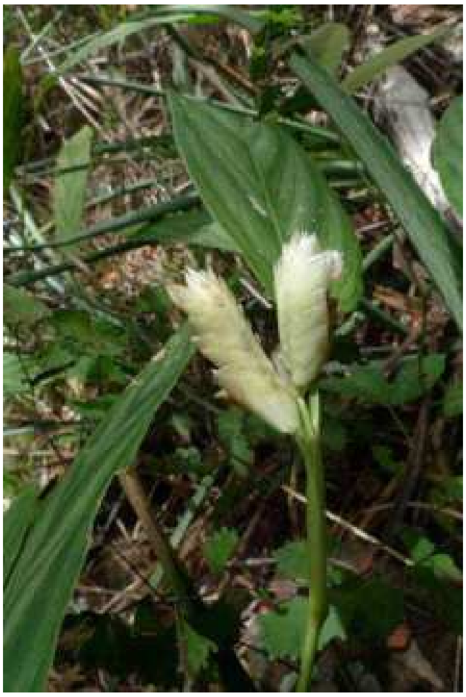

Myrosma cannifolia L.f. - Specimen W. Milliken 4236 ID:1108148 © RBG Kew (CC BY 3.0)

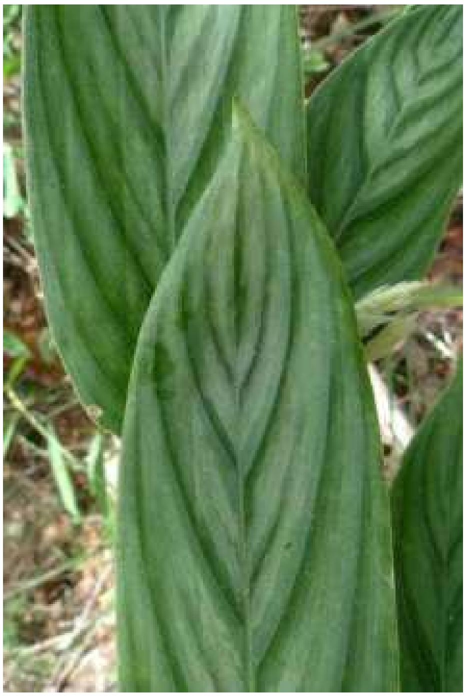

Myrosma cannifolia L.f. - Specimen W. Milliken 4236 ID:1108150 © RBG Kew (CC BY 3.0)

Historiae Rerum Naturalium Brasiliae Marcgrave, 1648 Page number 54a

Vernacular name(s) Herba

Species Distimake aegyptius (L.) A.R.Simões & Staples

Family Convolvulaceae

Notes

We did not find any correspondence between this woodcut and the contemporary or older sources.

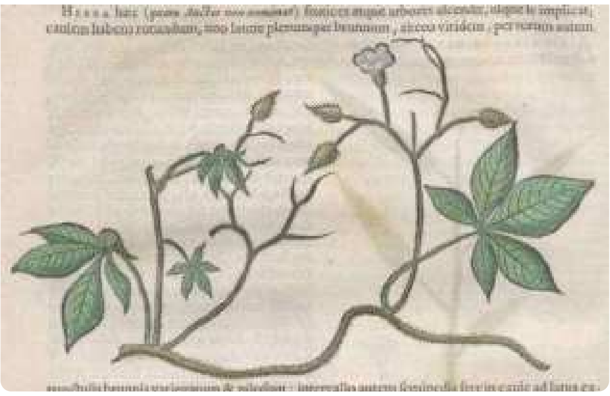

Historiae Plantarum – Herbis: 54a

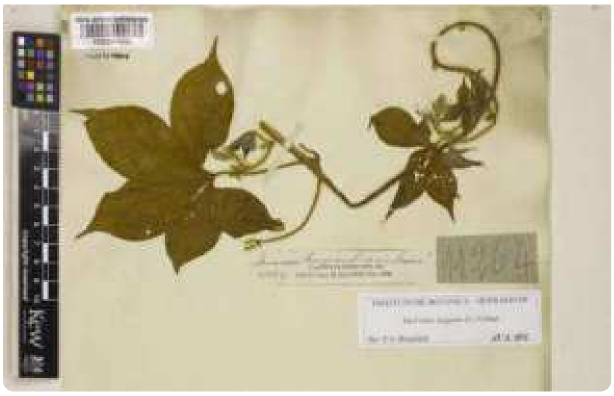

Specimen of *D. aegyptius* from Brazil in Kew's Herbarium - K000944988. Retrieved from Plants of the World Online

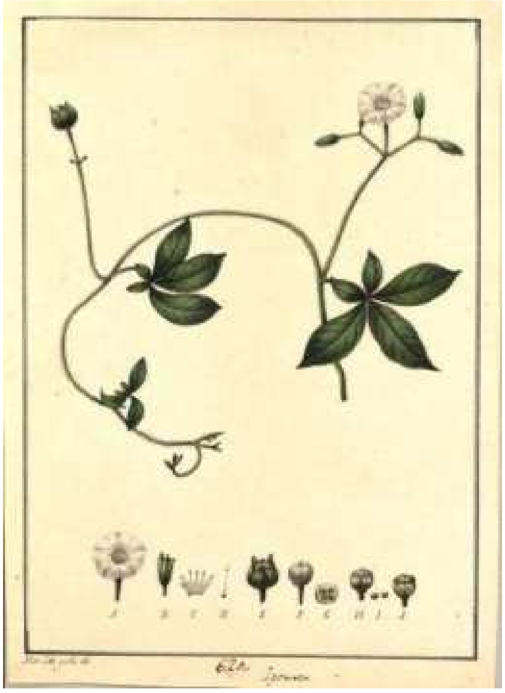

Drawings of the Royal Botanical Expedition to the Viceroyalty of Peru by Ruiz, H., Pavón, J. (1777). Real Jardín Botánico, Madrid, Spain

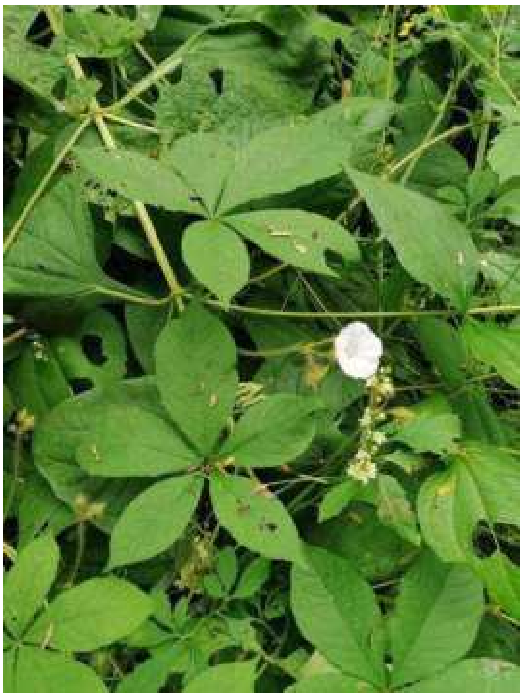

D. aegyptius observed in México for iNaturalist by Carlos Domínguez-Rodríguez (CC BY-NC 4.0)

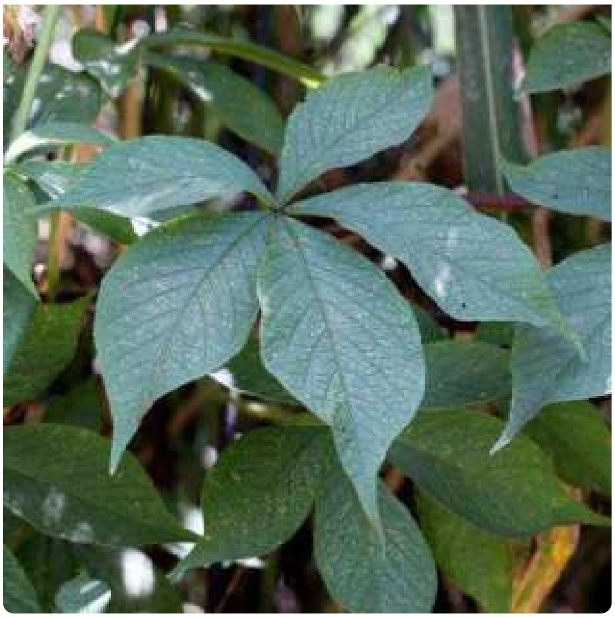

Leaves. "*D. aegyptius*" by Mauricio Mercadante (CC BY-NC-SA 2.0)

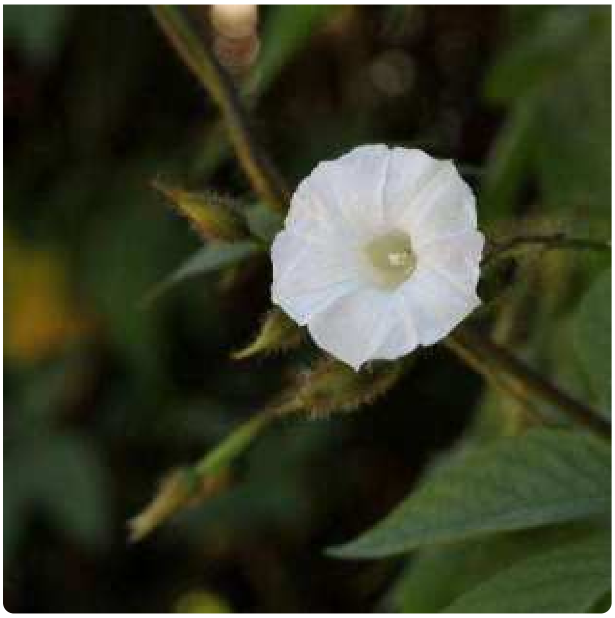

Flower. "*D. aegyptius*" by Mauricio Mercadante (CC BY-NC-SA 2.0)

Historiae Rerum Naturalium Brasiliae Marcgrave, 1648 Page number 54b

Vernacular name(s) Planta

Species Ruellia bahiensis (Nees) Morong

Family Acanthaceae

Notes

The woodcut was made after the specimen in Marcgrave's herbarium, as indicated by De Laet in the text (p. 54). The folded leaves of the exsicata can be appreciated in the image. One of the leaves is missing, as the specimen must have lost it after the drawing was made.

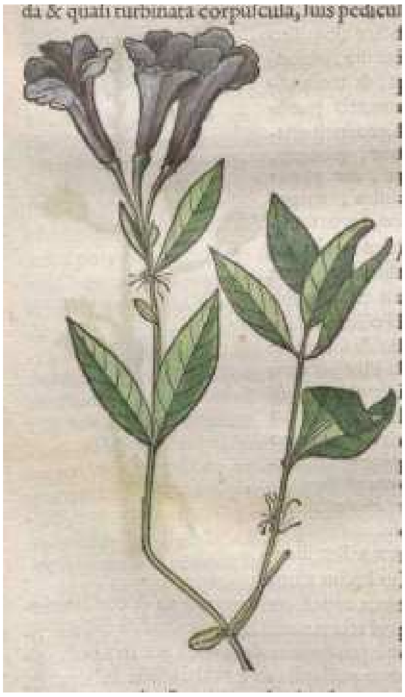

Historiae Plantarum – Herbis: 54b

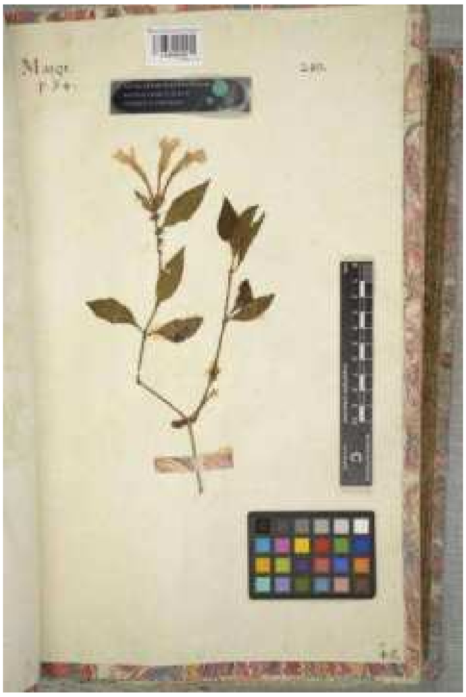

Marcgrave's herbarium: 45

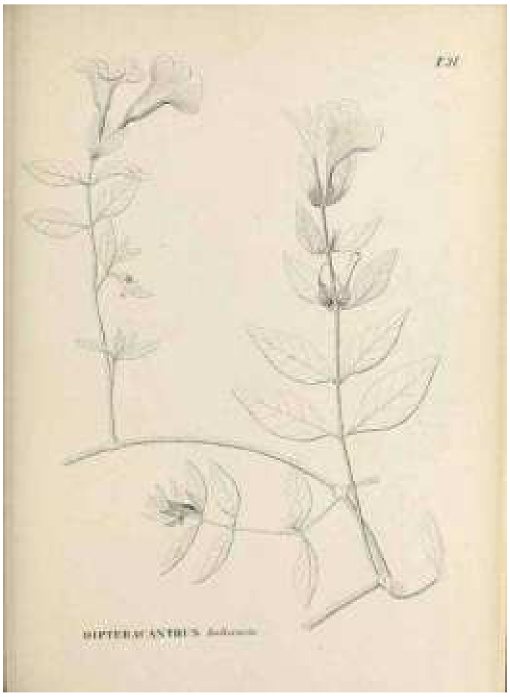

R. bahiensis in *Plantes nouvelles d'Amérique* by
Moricand, M.E. (1833-1846: Vol. 9, t. 91)

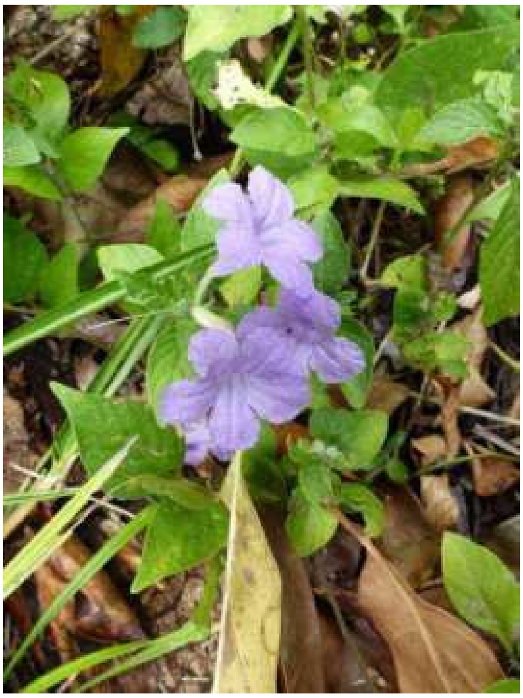

R. bahiensis observed in Brazil
for iNaturalist by Helio Lourencini (CC BY-NC 4.0)

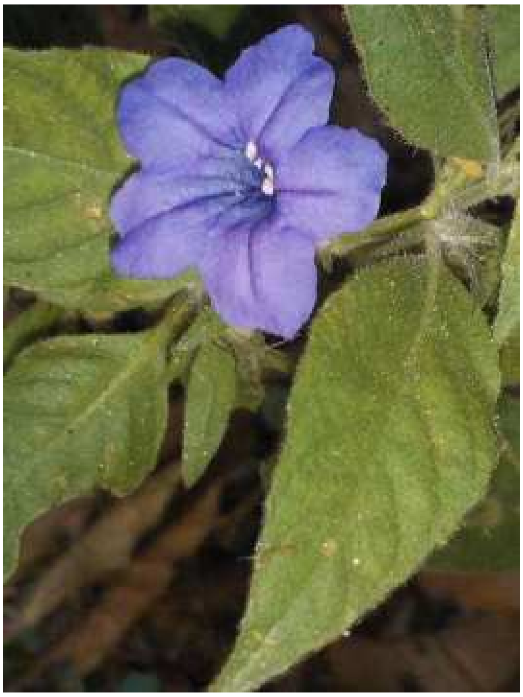

R. bahiensis observed in Brazil
for iNaturalist by Gabriel M. Rolim (CC BY-NC 4.0)

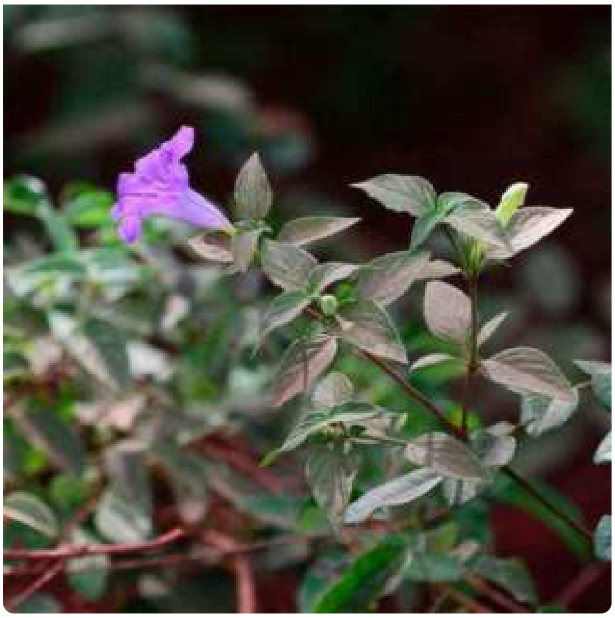

R. bahiensis observed in Brazil
for iNaturalist by Arne Holgersson (CC BY-NC 4.0)

Historiae Rerum Naturalium Brasiliae Marcgrave, 1648 Page number 54c

Vernacular name(s) Planta

Species Pombalia calceolaria (L.) Paula-Souza

Family Violaceae

Notes

The similarity is not striking, but the woodcut was made after the specimen in Marcgrave's herbarium, according to De Laet (p. 55). He also indicates that the painter did not represent the tiny teeth in the leaves and that he could not see the flowers (although the specimen is a flowering one).

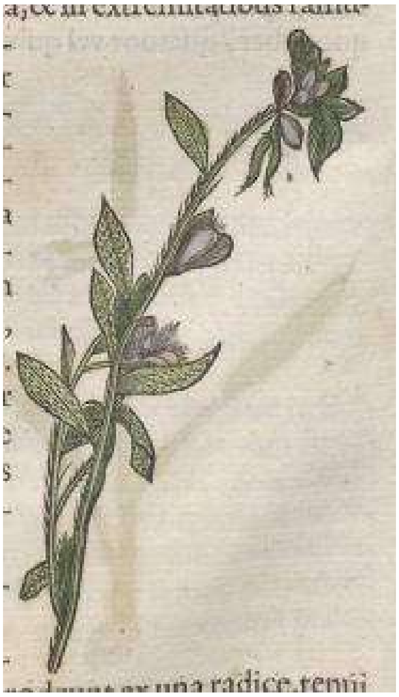

Historiae Plantarum – Herbis: 54c

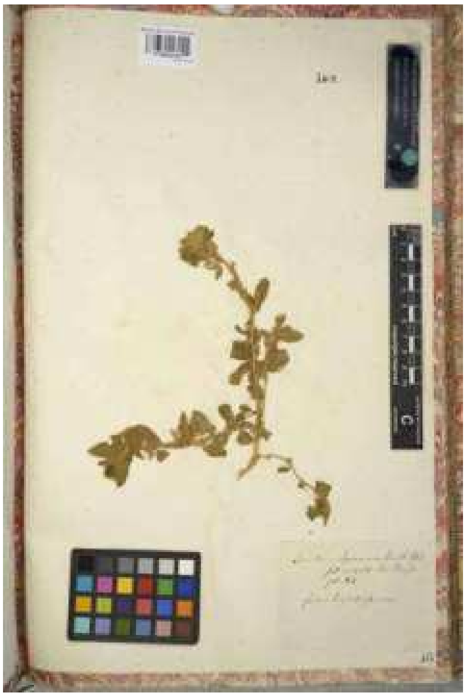

Marcgrave's herbarium: 164

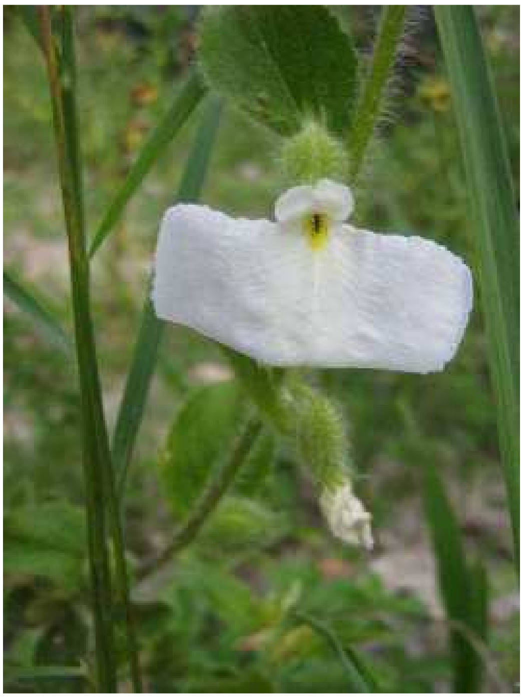

"*Pombalia calceolaria*" by Alex Popovkin, Bahia, Brazil
(CC BY-NC-SA 2.0)

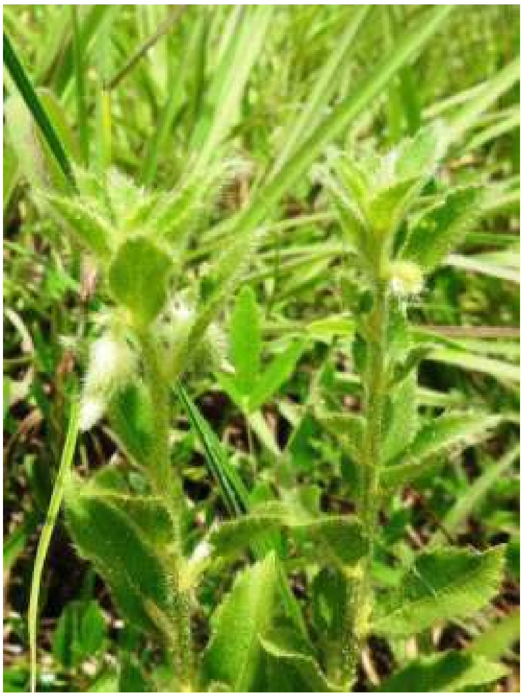

P. calceolaria observed in Brazil
for iNaturalist by Jan Meerman (CC BY-NC 4.0)

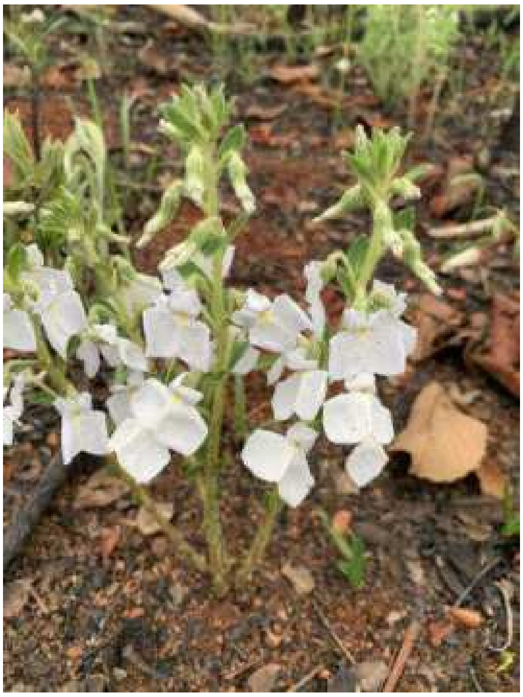

P. calceolaria observed in Brazil
for iNaturalist by Maria Clara Gil (CC BY-NC 4.0)

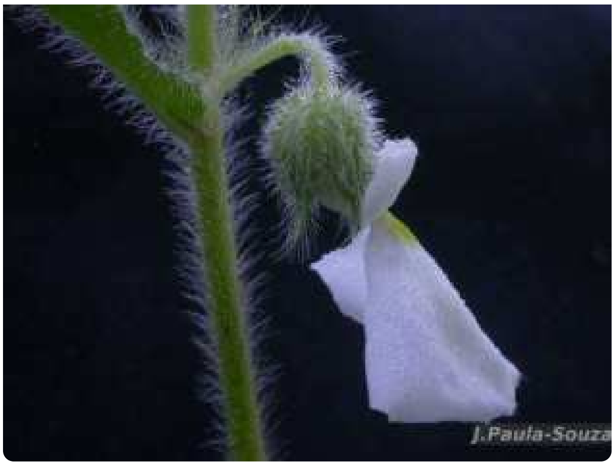

Flower of *P. calceolaria*. Published online by:Juliana de Paula-Souza in Flora e Funga do Brasil. Author: J. Paula-Souza

Historiae Rerum Naturalium Brasiliae Marcgrave, 1648 Page number 55a

Vernacular name(s) Aguara quiya

Species Solanum chenopodioides Lam.

Family Solanaceae

Notes

The woodcut looks slightly similar to the *Theatrum* image. They both depict flowers and fruits, although the woodcut shows the roots, (but these could have been cut off in the oil painting to fit the bound manuscript).

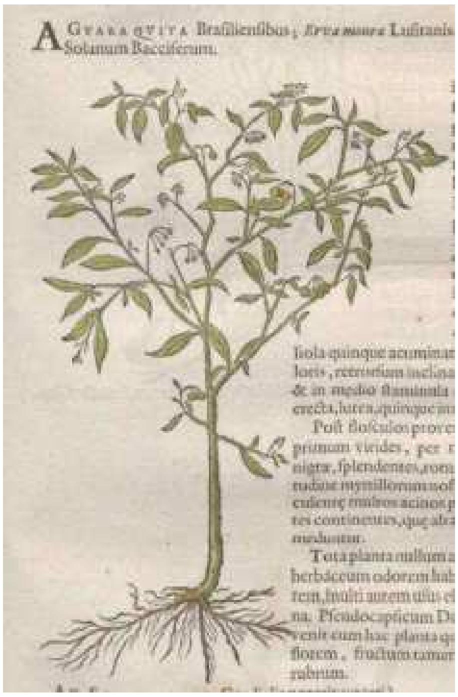

Historiae Plantarum – Herbis: 55a

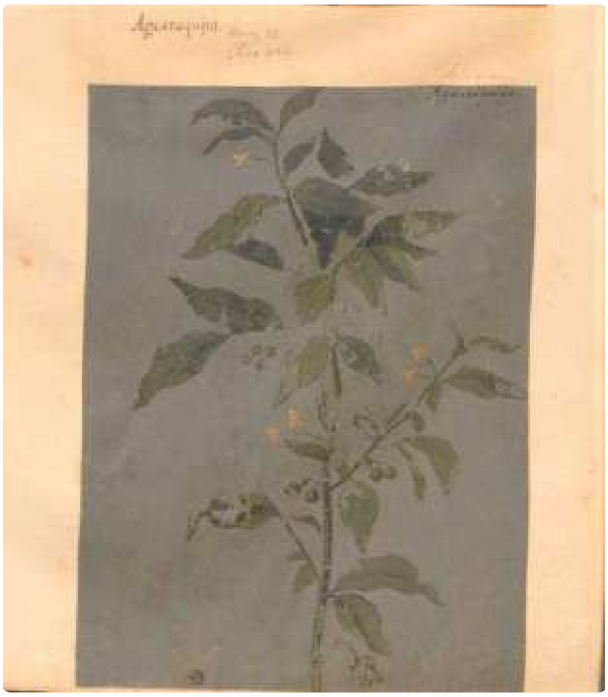

Theatrum Rerum Naturalium: 303

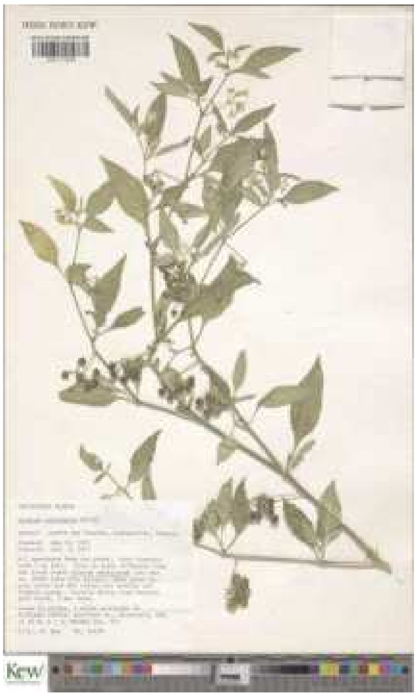

Specimen of *S. chenopodioides* from Kew's Herbarium - K001170295. Retrieved from Plants of the World Online

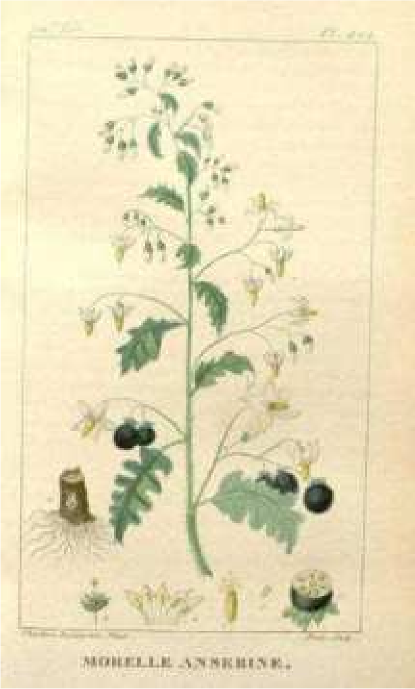

S. chenopodioides in *Flore [pittoresque et] médicale des Antilles* by Descourtilz, M.E. (1828: Vol. VI, t. 404). Missouri Botanical Garden, St. Louis, U.S.A.

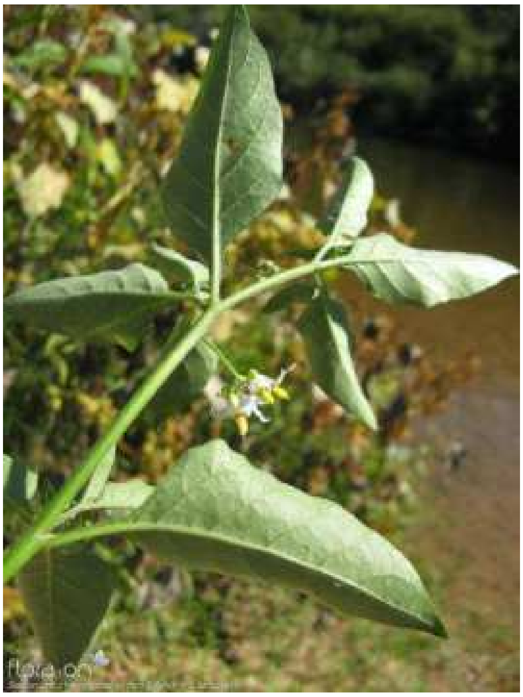

Flowering branch. "*S. chenopodioides*" by André Carapeto (CC BY-NC 4.0)

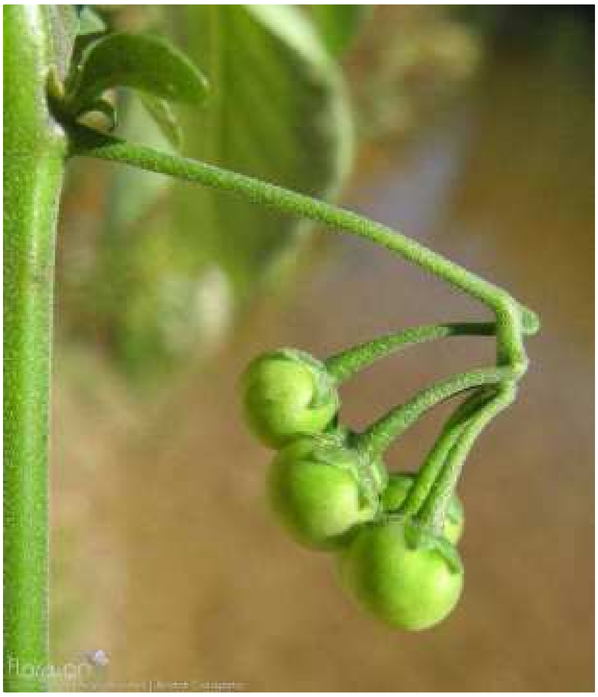

Fruits. "*S. chenopodioides*" by André Carapeto (CC BY-NC 4.0)

Historiae Rerum Naturalium Brasiliae Marcgrave, 1648 Page number 55b

Vernacular name(s) An Sagittaria

Species Crotalaria stipularia Desv.

Family Fabaceae

Notes

The woodcut was made after the specimen collected by Marcgrave, according to De Laet (p. 56). The fruits are lacking in the specimen. They either fell after mounting it, or De Laet ordered them to be drawn after the description. Interestingly, Marcgrave stated that the seeds have a "half-moon or heart-like shape, as it is normally painted" (p. 55). This suggests the practice of painting or sketching flora with different hands, hence obtaining multiple outcomes. He also cited that this plant is "often full of holes, as eaten by worms, hence making it difficult to find one without holes" (p. 55). This implies a method of observation and selective gathering before portraying the plants.

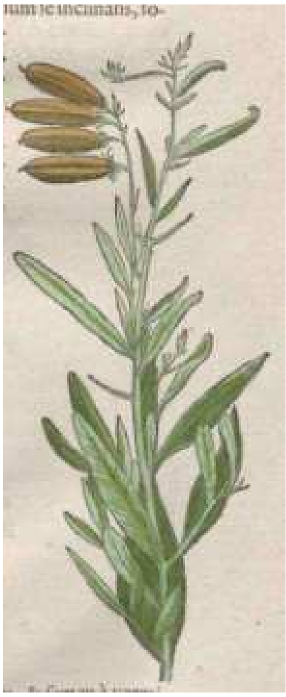

Historiae Plantarum – Herbis: 55b

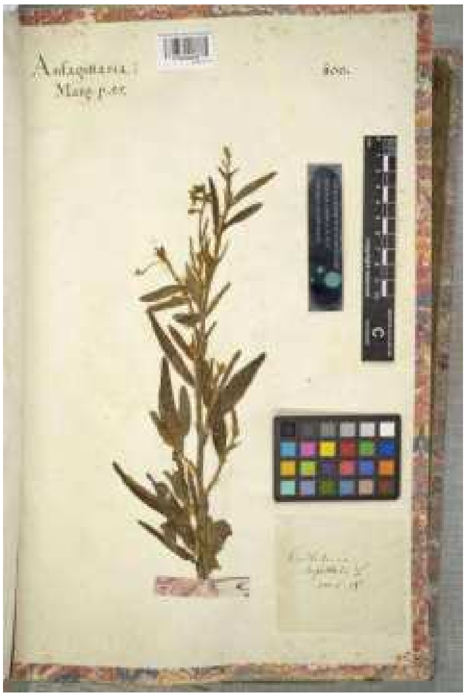

Marcgrave's herbarium: 36

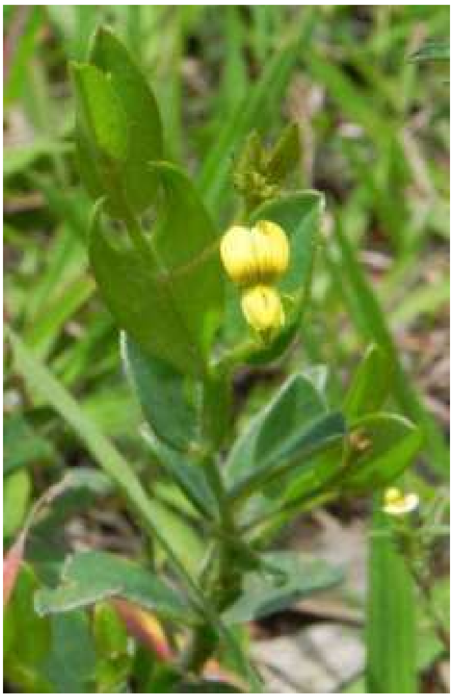

C. stipularia" by João de Deus Medeiros (CC BY 2.0)

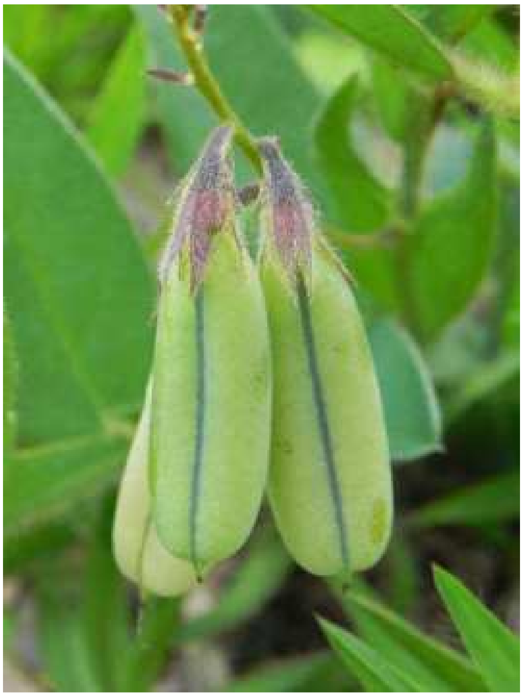

" *C. stipularia*" by João de Deus Medeiros (CC BY 2.0)

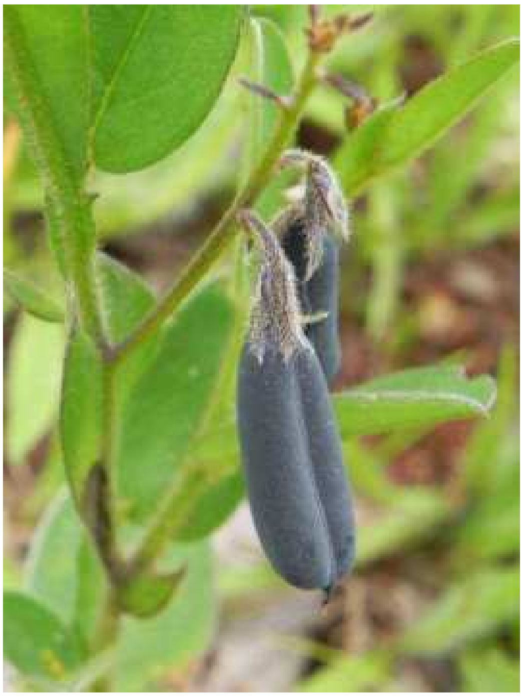

C. stipularia" by João de Deus Medeiros (CC BY 2.0)

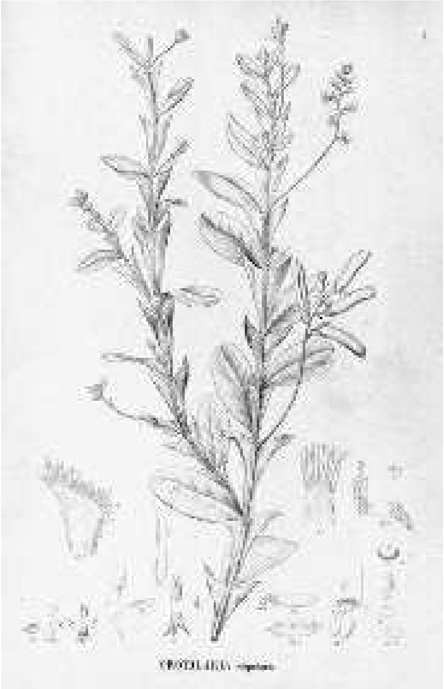

Engraving of *C. stipularia* in Martius, C.F.P. von, Eichler, A.G., Urban, I., *Flora Brasiliensis* (1859-1862) Vol. 15(1): 3

Historiae Rerum Naturalium Brasiliae	Marcgrave, 1648	Page number	56a

Vernacular name(s) Herba

Species Microtea celosioides (Spreng.) Moq. ex Sennikov & Sukhor.

Family Microteaceae

Notes

The woodcut was made after one of the two specimens kept in Marcgrave's herbarium (p. 12), as indicated by De Laet (p. 56). They are both flowering and, despite a few leaves missing in the specimen, there is a strong resemblance.

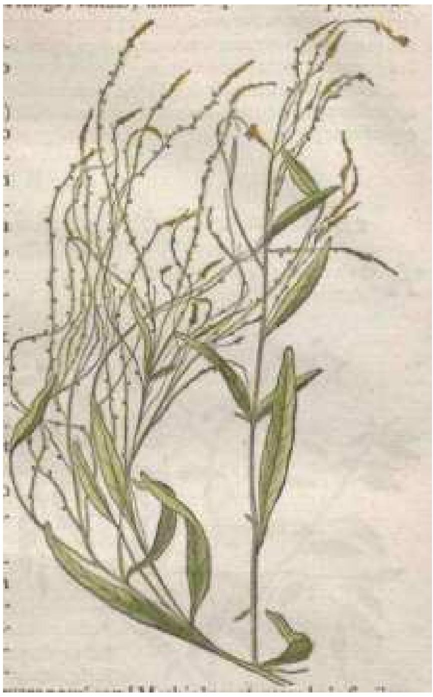

Historiae Plantarum – Herbis: 56a

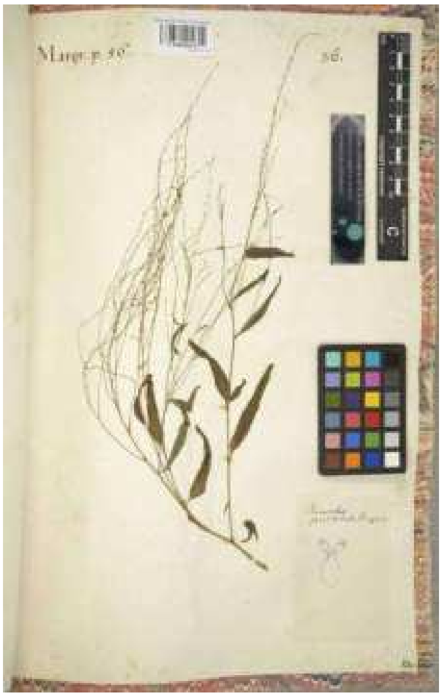

Marcgrave's herbarium: 12

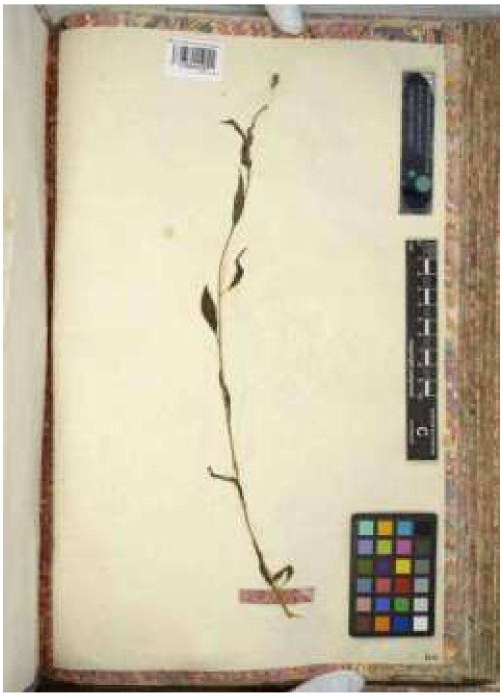

Marcgrave's herbarium: 148

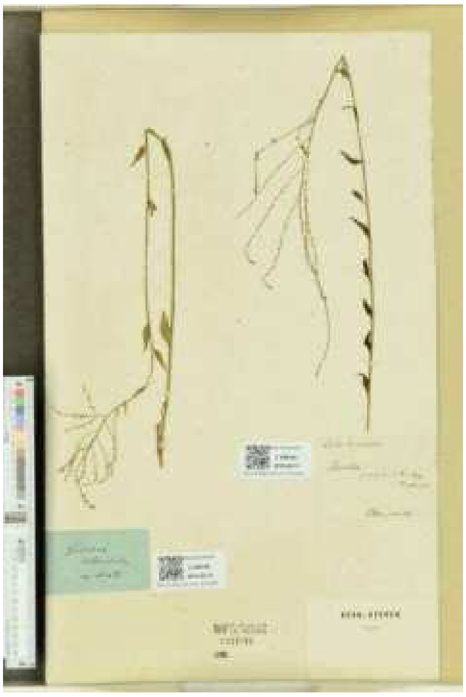

Specimen of *M. celosioides*. Retrieved from GBIF-EPSG:4326- https://www.gbif.org/occurrence/3439852065

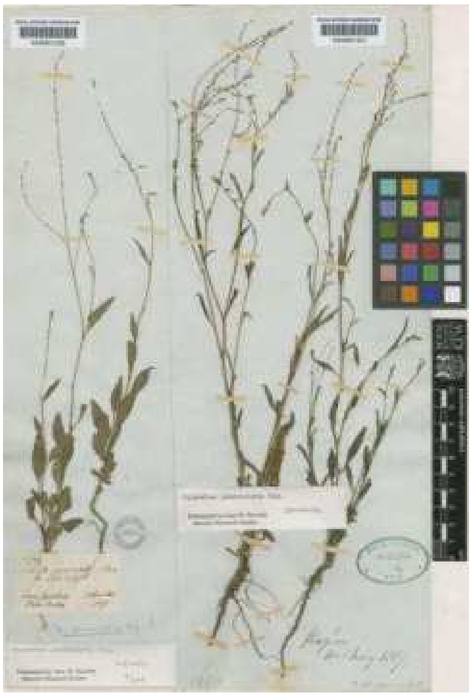

Specimen of *M. celosioides* from Kew's Herbarium - K000601207. Retrieved from Plants of the World Online

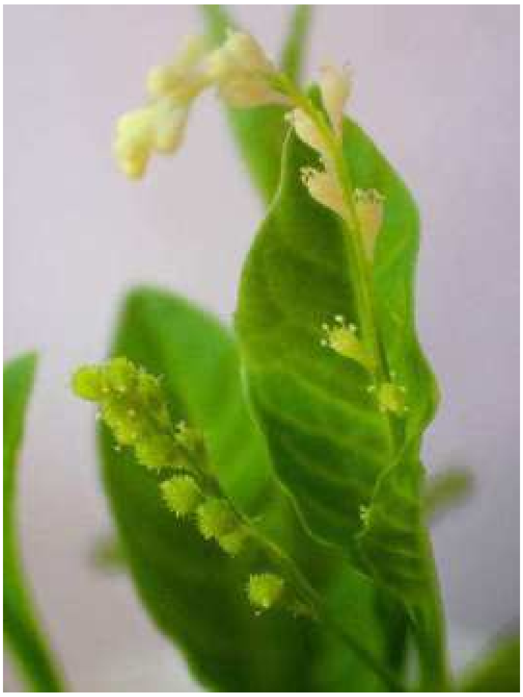

Related species to *M. celosioides*: "*Microtea debilis* (Phytolaccaceae)2.jpg" by Anonyme973 (CC BY-SA 4.0)

Historiae Rerum Naturalium Brasiliae Marcgrave, 1648 Page number 56b

Vernacular name(s) Inimboy

Species Guilandina bonduc L.

Family Fabaceae

Notes

The woodcut is very similar to the *Theatrum* image (reversed). The *Misc. Cleyeri* shows a flowering branch; while flowers, unripe and ripe dehiscent fruits, and seeds are displayed in the *Theatrum* and the HNB. J. Breyne (1739: 33) published an engraving of this plant based on a specimen collected by Marcgrave. This image is made after a voucher (characterized by the flat arrangement of the leaves) and it is similar to the specimen in Copenhagen (p. 7).

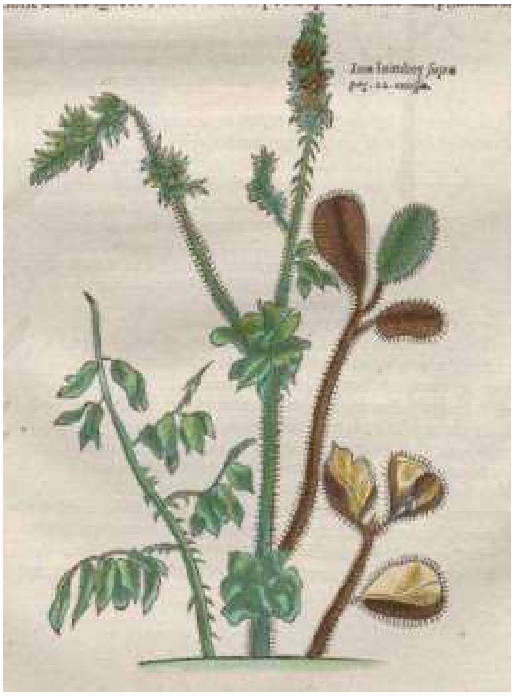

Historiae Plantarum – Herbis: 56b

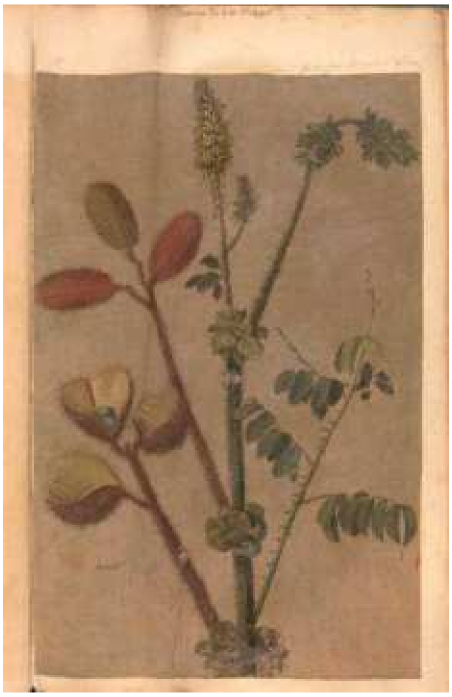

Theatrum Rerum Naturalium: 149

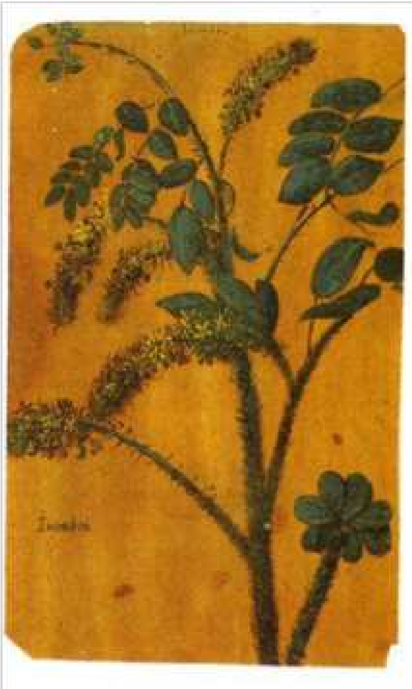

Miscellanea Cleyeri: 52r

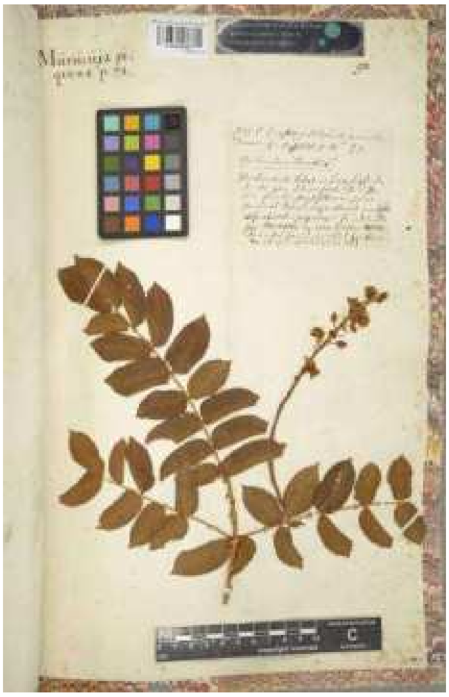

Marcgrave's herbarium: 7

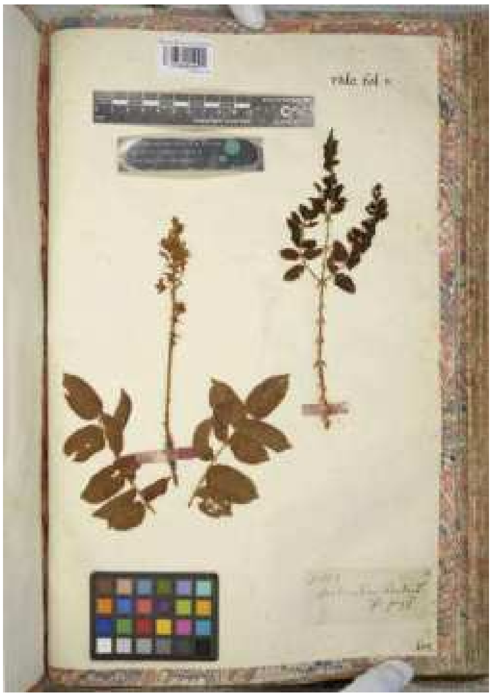

Marcgrave's herbarium: 153

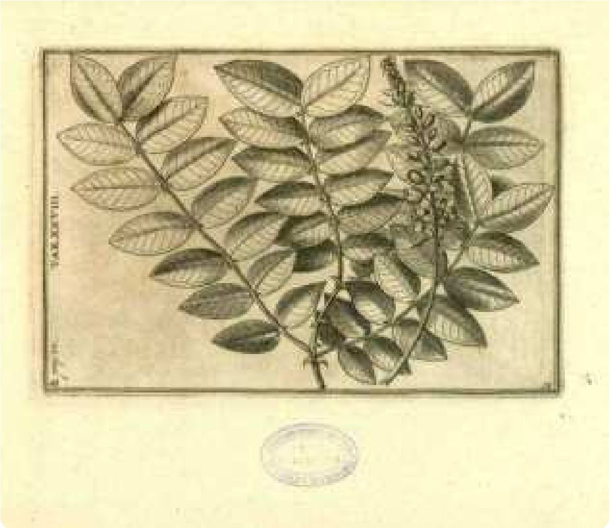

Engraving of *G. bonduc* in *Prodromi fasciculi rariorum plantarum* by Breyne, J. P. (1739: p. 33, t. 28). https://www.biodiversitylibrary.org/bibliography/72155

Historiae Rerum Naturalium Brasiliae | Marcgrave, 1648 | Page number 57

Vernacular name(s) Erva d'Anir

Species Indigofera microcarpa Desv.

Family Fabaceae

Notes

There is no strong resemblance between the specimen and the woodcut image, although the branch in the herbarium could have been part of the plant depicted in the HNB.

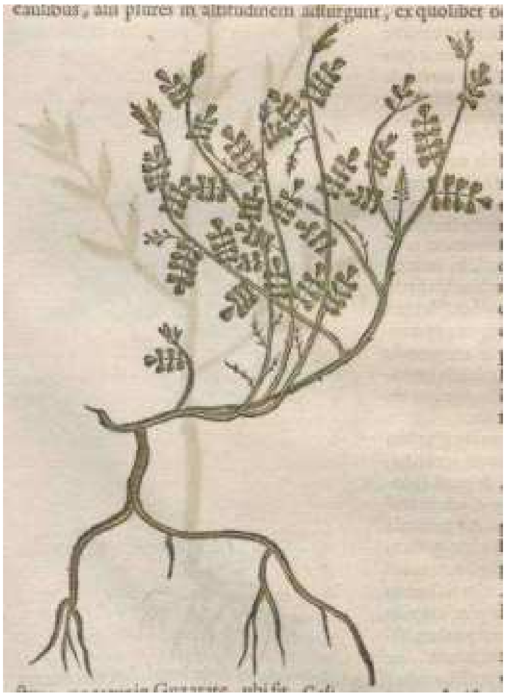

Historiae Plantarum – Plantis Frutescentibus & Fruticibus: 57

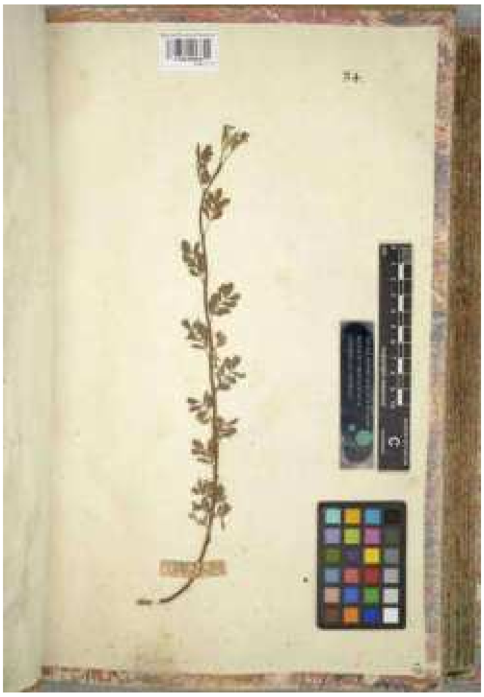

Marcgrave's herbarium: 69

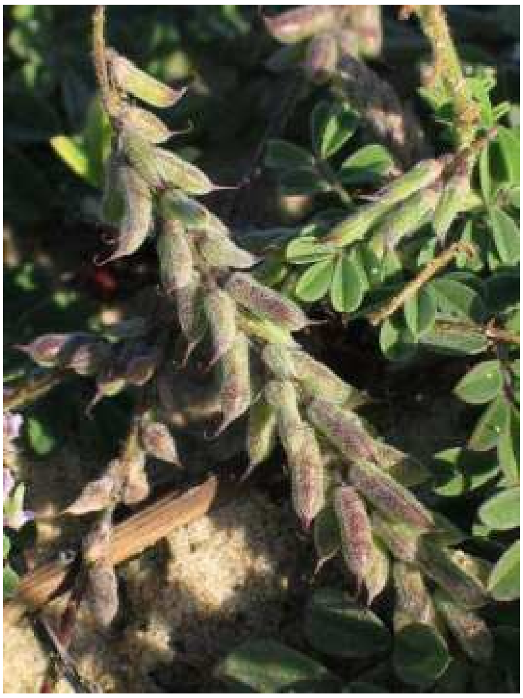

"Indigofera microcarpa LEGUMINOSAE" by antoniosergio25 is licensed under CC BY-NC-SA 2.0.

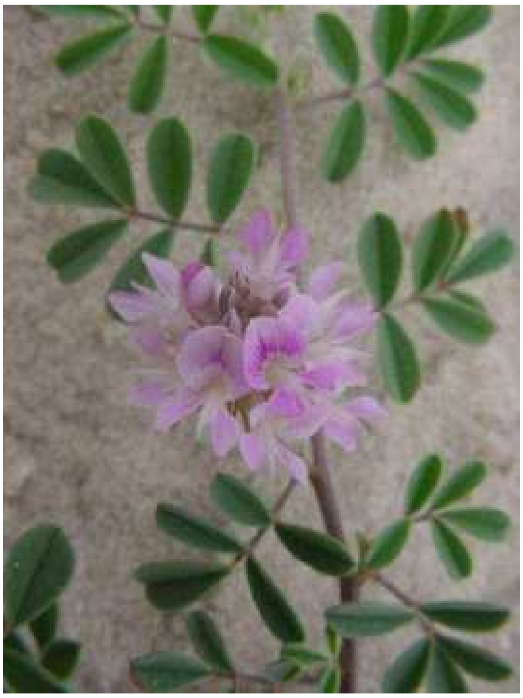

Indigofera sabulicola [syn. of *I. microcarpa*] - FABACEAE - Lagoa dos Patos - RS - Brasil by berichard (CC-BY-2.0)

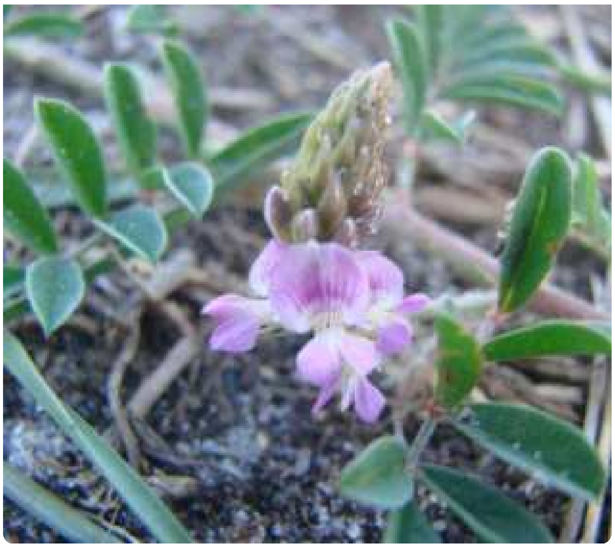

Indigofera sabulicola [syn. of *I. microcarpa*] - FABACEAE - Lagoa dos Patos - RS - Brasil by berichard (CC-BY-2.0)

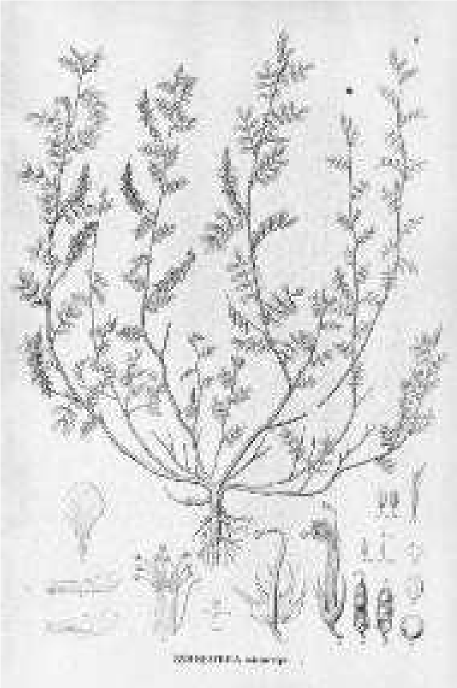

Engraving of *I. microcarpa* in Martius, C.F.P. von, Eichler, A.G., Urban, I., *Flora Brasiliensis* (1859-1862) Vol. 15(1): 6

Historiae Rerum Naturalium Brasiliae Marcgrave, 1648 Page number 58

Vernacular name(s) Alia species

Species Eclipta prostrata (L.) L.

Family Asteraceae

Notes

The woodcut is different than the *Theatrum.* The roots are depicted in the woodcut but lacking in the illustration, while the flowers are depicted in more detail in the oil painting.

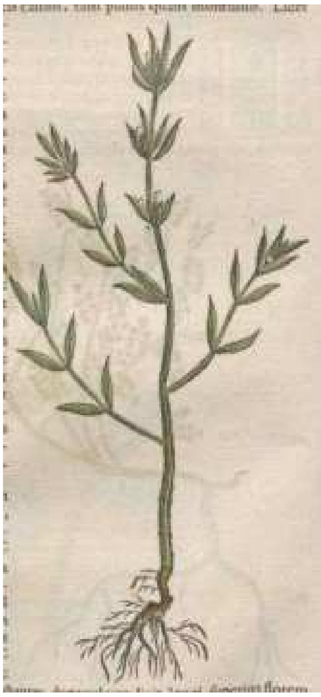

Historiae Plantarum – Plantis Frutescentibus & Fruticibus: 58

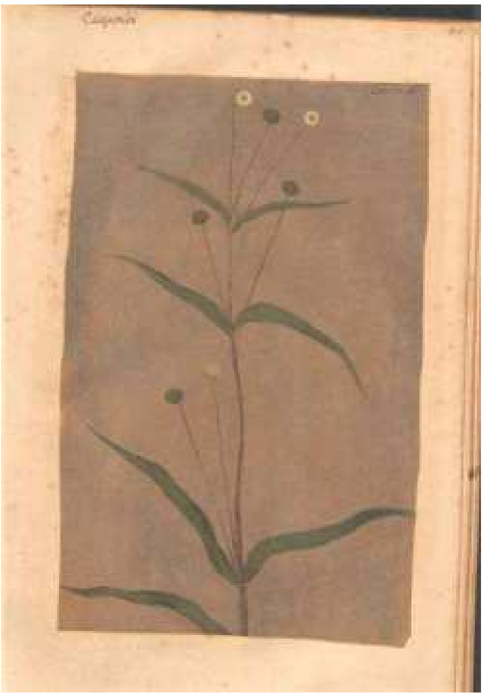

Theatrum Rerum Naturalium: 485

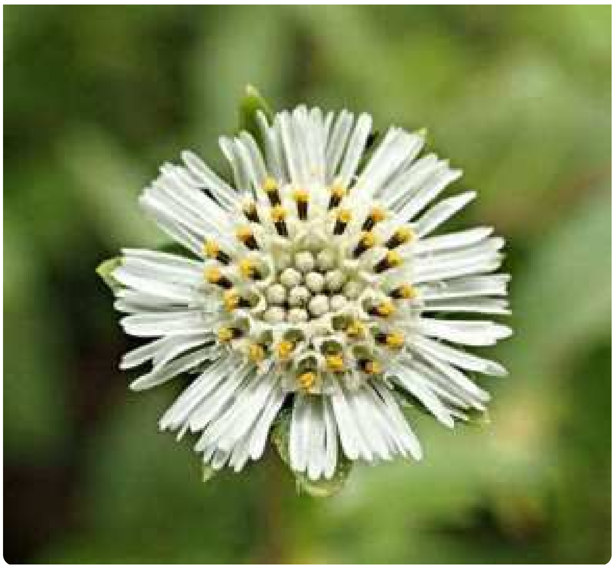

Flower. "*E. prostrata*" by Ahmad Fuad Morad (CC BY-NC-SA 2.0)

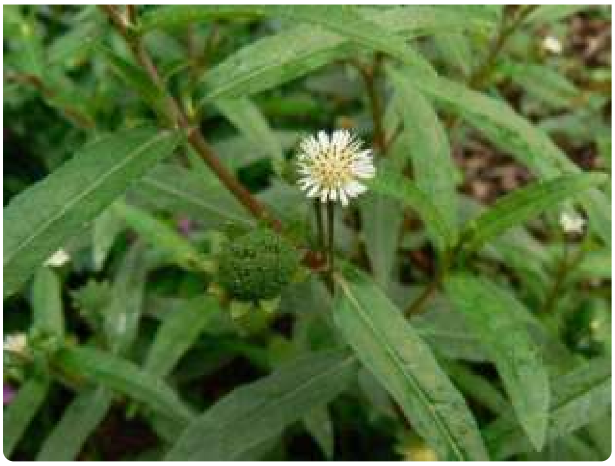

"*Eclipta prostrata*" by Dinesh Valke (CC BY-SA 2.0)

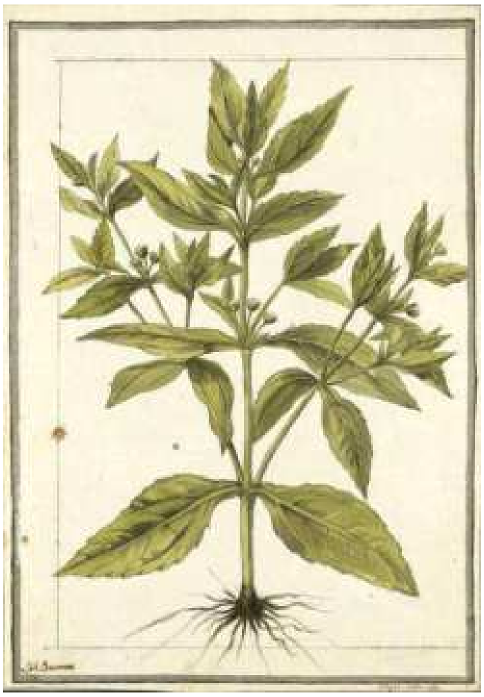

Drawings of the Royal Botanical Expedition to the Viceroyalty of Peru by Ruiz, H., Pavón, J. (1777 -1816). Real Jardín Botánico, Madrid, Spain

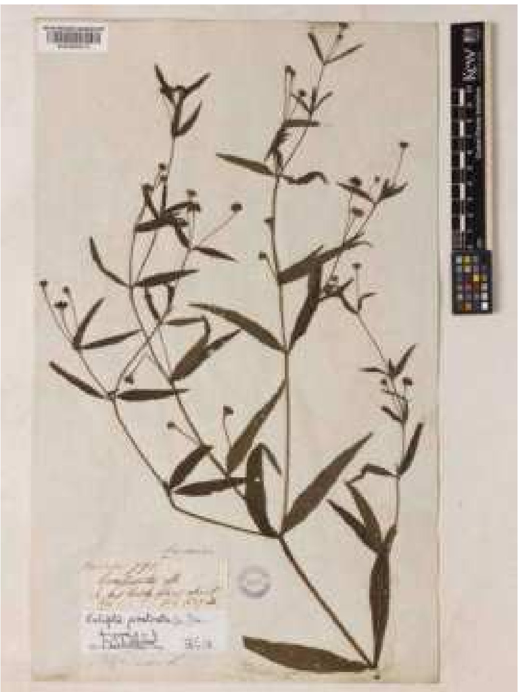

Specimen of *E. prostrata* from Kew's Herbarium - K000895513. Retrieved from Plants of the World Online

Historiae Rerum Naturalium Brasiliae	Marcgrave, 1648	Page number	59

Vernacular name(s)	Caaghiyuyo
Species	Clidemia biserrata DC.
Family	Melastomataceae
Notes	

The duplicates in Marcgrave's herbarium (both numbered 39 in the upper right corner), are flowering branches that could have been used as models to elaborate the image depicted in the woodcut. The woodcut, however, also shows the fruits.

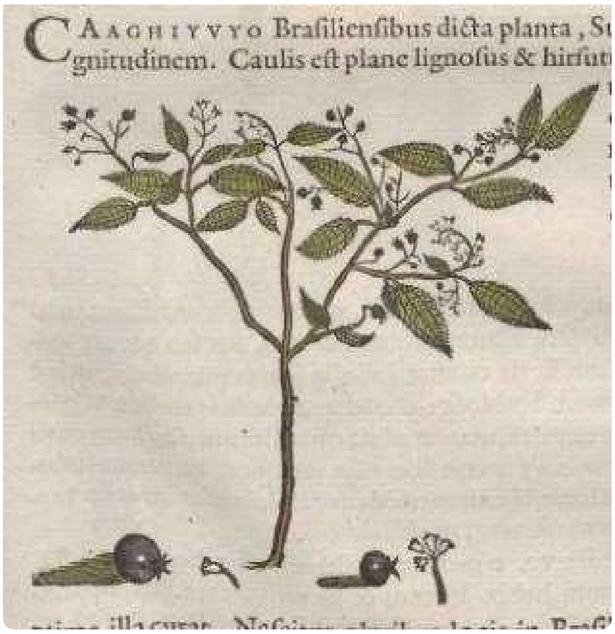

Historiae Plantarum – Plantis Frutescentibus & Fruticibus: 59

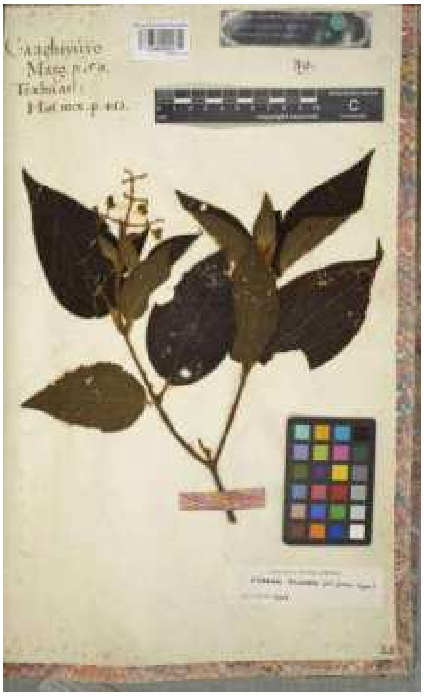

Marcgrave's herbarium: 25

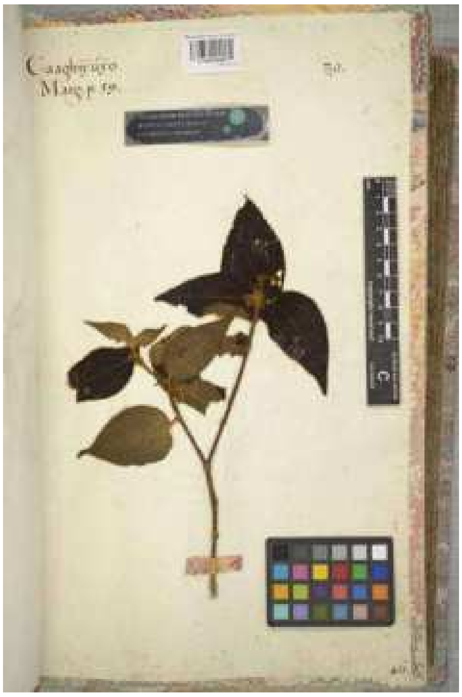

Marcgrave's herbarium: 46

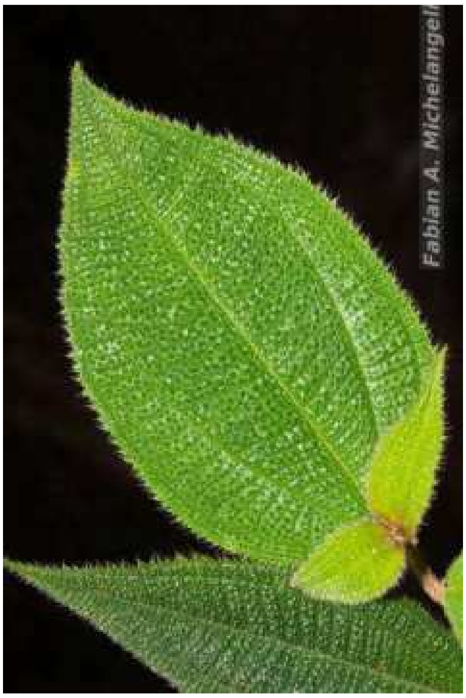

C. biserrata. Published online by:JFabián Armando Michelangeli in Flora e Funga do Brasil. Author: Fabian A. Michelangeli

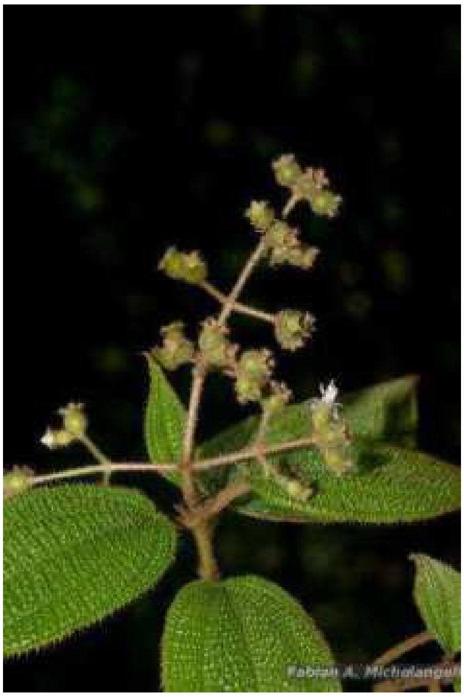

C. biserrata. Published online by:JFabián Armando Michelangeli in Flora e Funga do Brasil. Author: Fabian A. Michelangeli

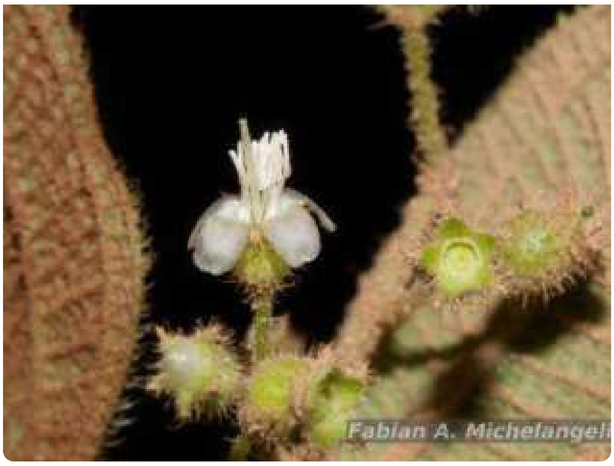

C. biserrata. Published online by:JFabián Armando Michelangeli in Flora e Funga do Brasil. Author: Fabian A. Michelangeli

Historiae Rerum Naturalium Brasiliae Marcgrave, 1648 Page number 60a

Vernacular name(s) Tangaraca. Erva do rato

Species Boerhavia coccinea Mill.

Family Nyctaginaceae

Notes

The woodcut differs from the *Theatrum*. The leaves and the inflorescences are densely crowded in the *Theatrum*, while the woodcut shows a shoot with the root.

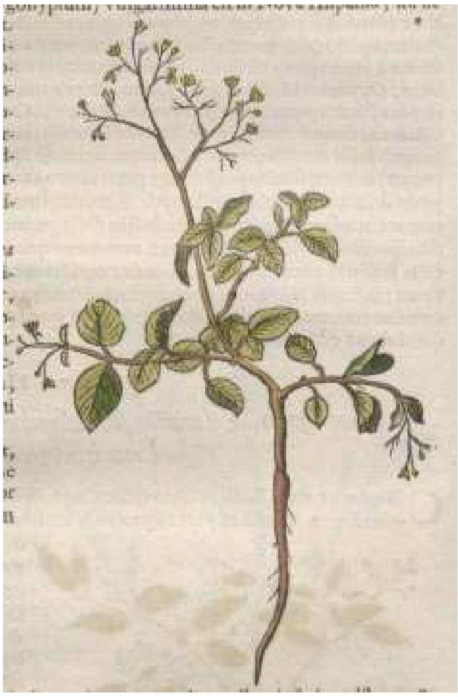

Historiae Plantarum – Plantis Frutescentibus & Fruticibus: 60a

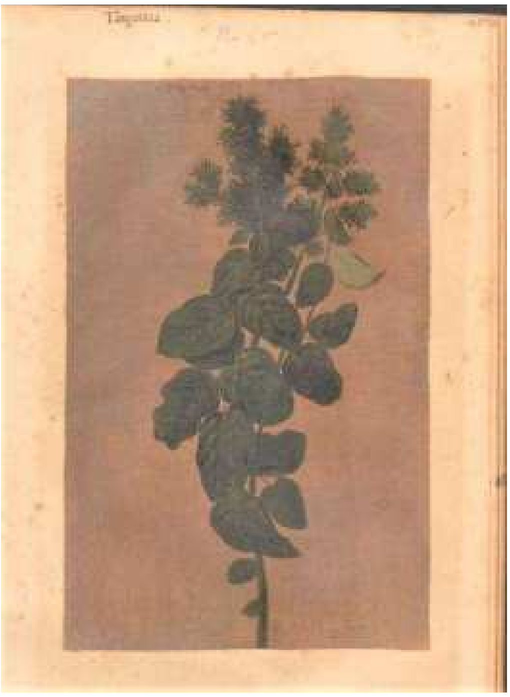

Theatrum Rerum Naturalium: 487

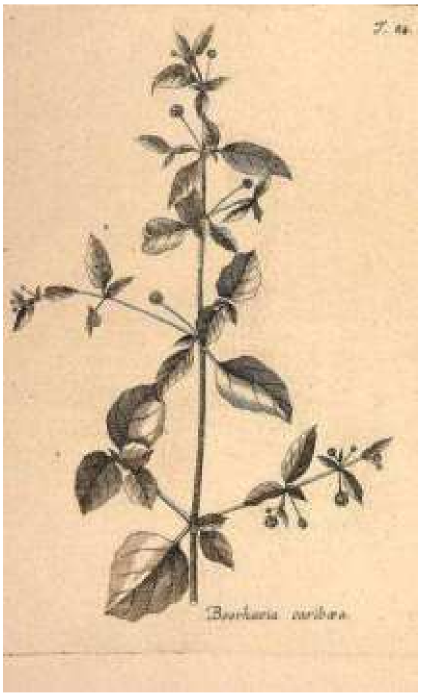

B. coccinea in *Observationum botanicarum* by Jacquin, N.J. von, (1767: Vol. IV, t. 84). www.BioLib.de

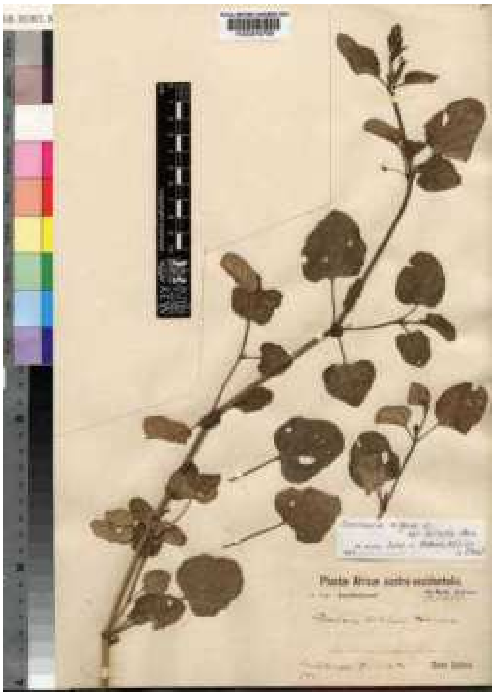

Specimen of *B. coccinea* from Kew's Herbarium - K000243788. Retrieved from Plants of the World Online

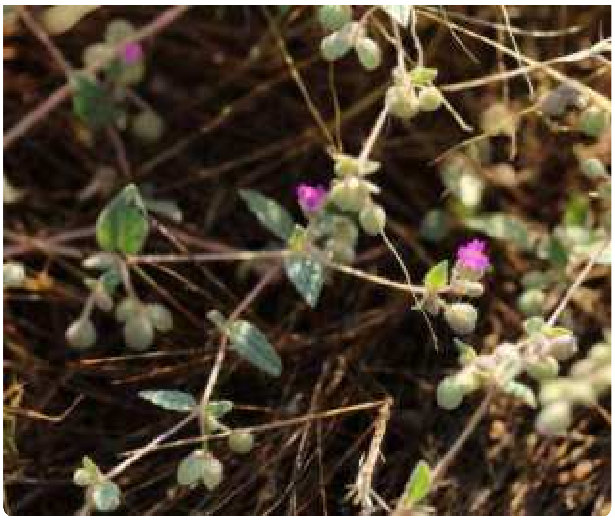

"*B. coccinea*" by anthony_mendoza (CC BY-NC-SA 2.0)

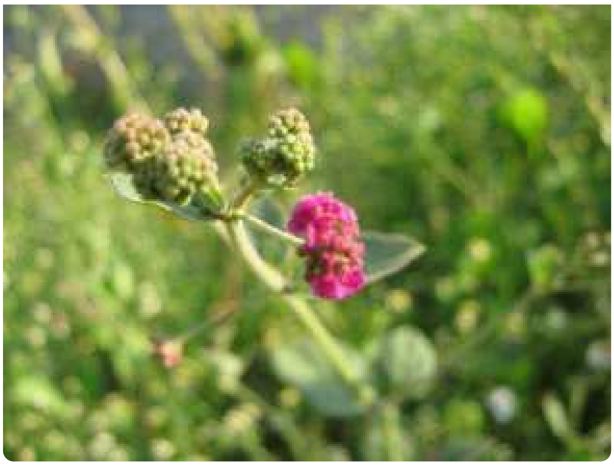

"*B. coccinea*" by Tyrrhium(CC BY 2.0)

Historiae Rerum Naturalium Brasiliae | Marcgrave, 1648 | Page number 60b

Vernacular name(s) Tangaraca. Erva do rato

Species Palicourea crocea (Sw.) Schult.

Family Rubiaceae

Notes

The woodcut differs from the *Theatrum*. The leaves and flowers are more crowded in the *Theatrum* than in the woodcut and the leaves are more rounded in the oil painting. The intraspecific differences observed in the leaves could be the result of climatic and geographic variability (i.e., collected at different times or from different areas).

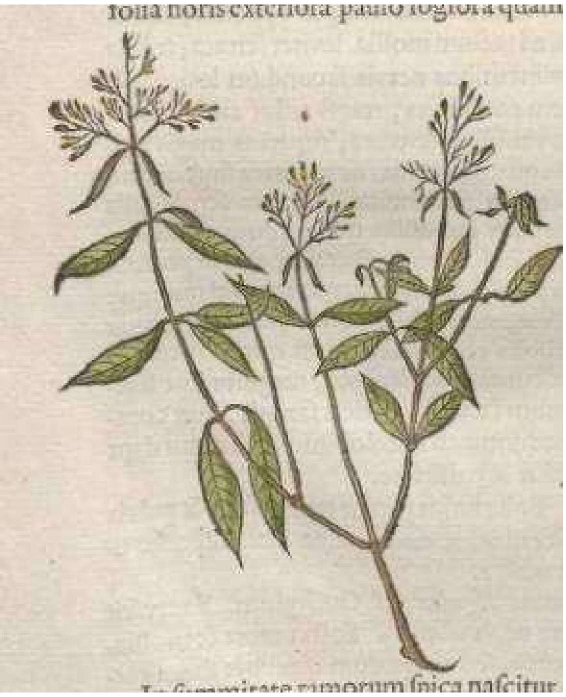

Historiae Plantarum – Plantis Frutescentibus & Fruticibus: 60b

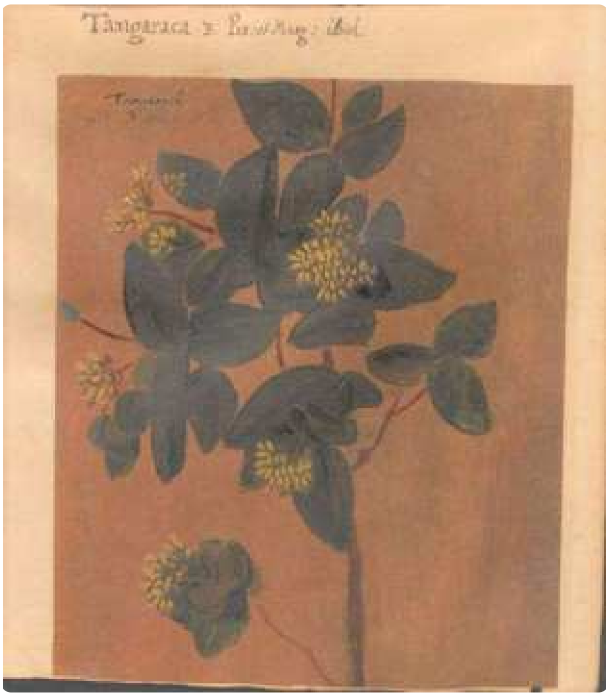

Theatrum Rerum Naturalium: 197

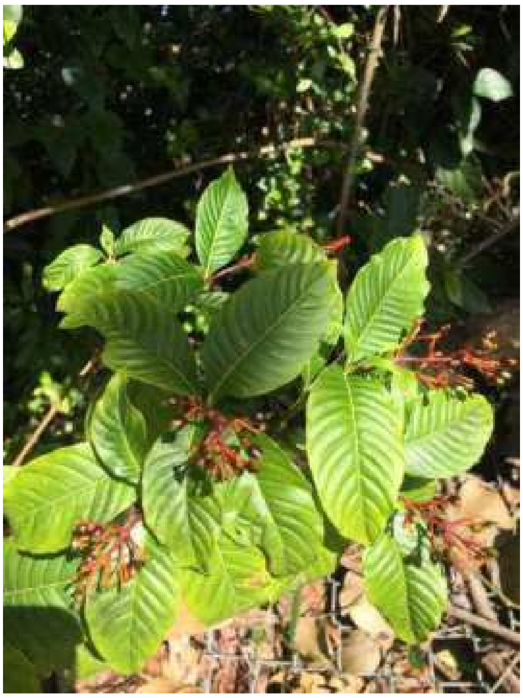

P. crocea observed in Puerto Rico
for iNaturalist by Steve Maldonado Silvestrini (CC BY-NC 4.0)

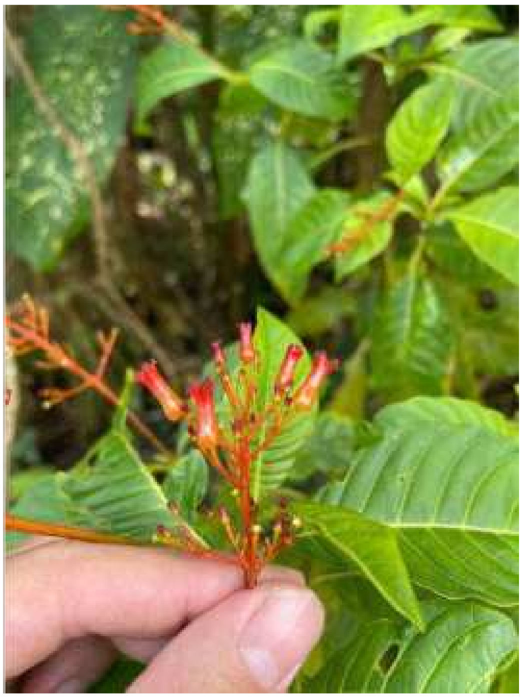

P. crocea observed in Puerto Rico
for iNaturalist by Steve Maldonado Silvestrini (CC BY-NC 4.0)

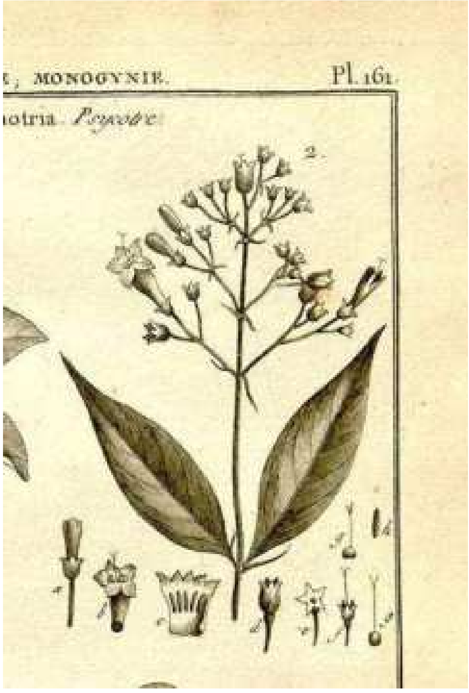

Recueil de planches de botanique de l'encyclopédie by Lamarck, J.B.P.A. de Monet de, Poiret, J.L.M. (1791: Vol. I, t. 161 f. 2). Missouri Botanical Garden

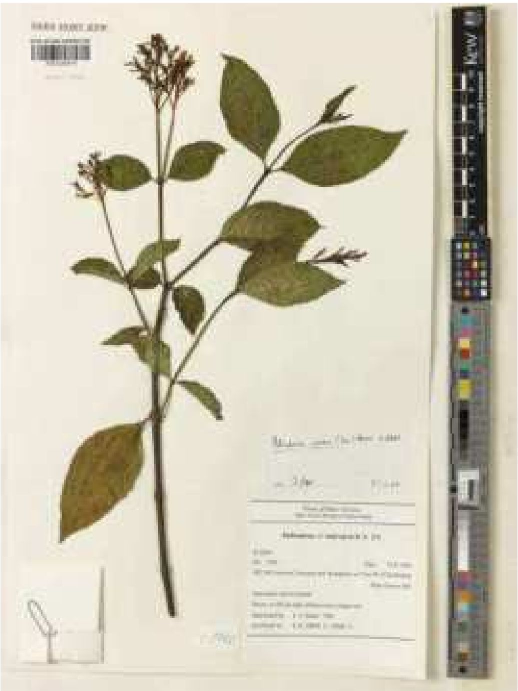

Specimen of *P. crocea* from Kew's Herbarium - K001200814. Retrieved from Plants of the World Online

Historiae Rerum Naturalium Brasiliae	Marcgrave, 1648	Page number	61a

Vernacular name(s) Frutex arborescens

Species Ouratea fieldingiana (Gardner) Engl.

Family Ochnaceae

Notes

We did not find any correspondence between this woodcut and the contemporary or older sources.

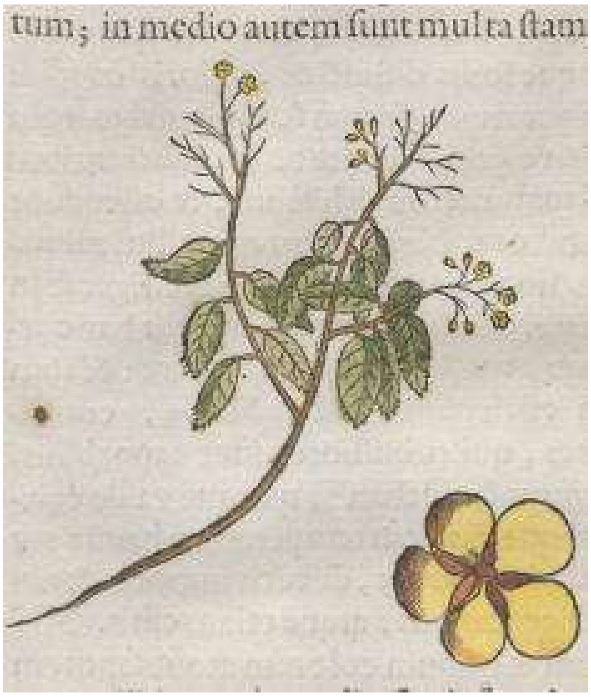

Historiae Plantarum – Plantis Frutescentibus & Fruticibus: 61a

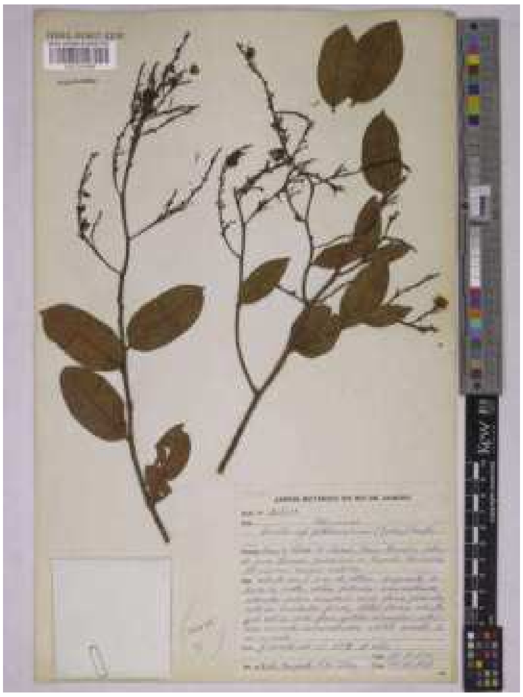

Specimen of *O. fieldingiana* from Kew's Herbarium - K001201668. Retrieved from Plants of the World Online

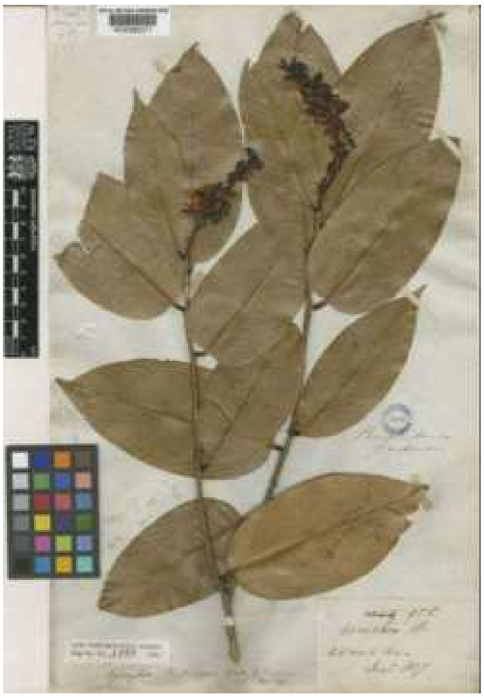

Specimen of *O. fieldingiana*. Collected in Pernambuco, Brazil, G. Gardner, 958 -NS2883- In Flora e Funga do Brasil.

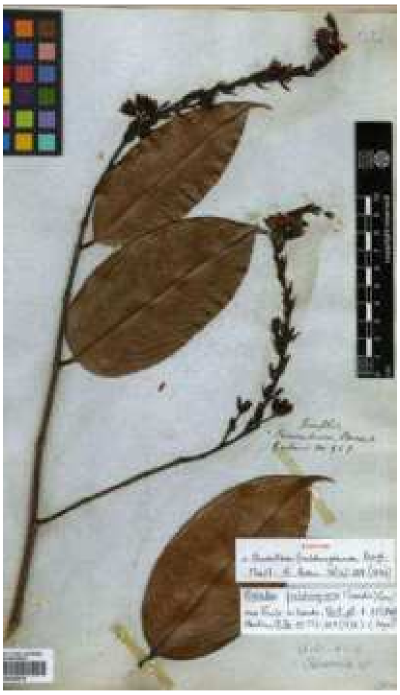

Specimen of *O. fieldingiana*. Collected in Pernambuco, Brazil, G. Gardner, 958 -E00326376- In Flora e Funga do Brasil.

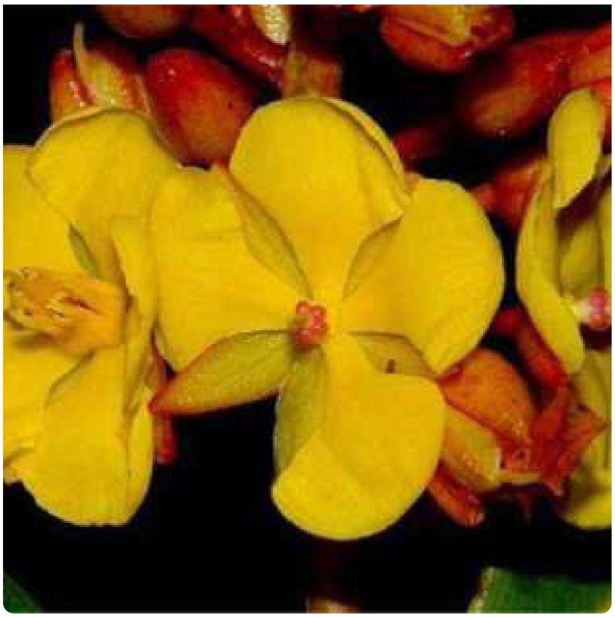

Flower of a relative of O. *fieldingiana*, *O. caudata*, also represented in the HNB (Marcgrave 1648: 101). Cláudio Nicoletti de Fraga, Plants of the World Online

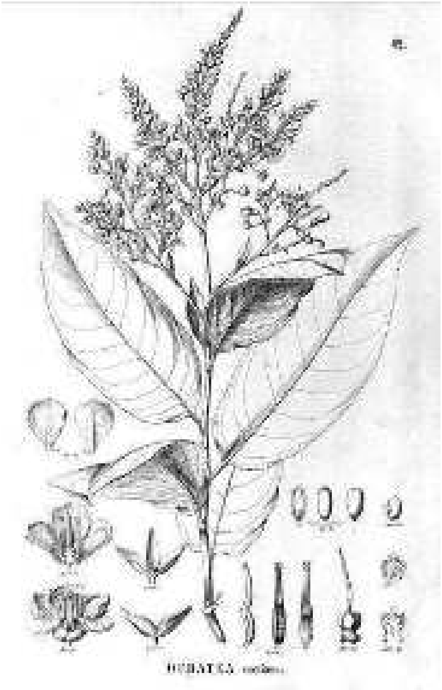

A relative of O. *fieldingiana*, *O. coccinea*, distributed in North Brazil and illustrated in Martius, C.F.P. von, Eichler, A.G., Urban, I., *Flora Brasiliensis* (1872-1877)

Historiae Rerum Naturalium Brasiliae Marcgrave, 1648 Page number 61b

Vernacular name(s) Urucu

Species Bixa orellana L.

Family Bixaceae

Notes

The woodcut differs from the *Theatrum*. The woodcut shows the flowers and a few fruits about to open, while the oil painting shows a very accurate image of the mature fruits, two of them open and showing bright red seeds. These seeds were used as a dye by the Indigenous population and are still used this way today. They were also traded among Indigenous groups and European settlers, who in turn, shipped them to Europe to be used as a food colorant and cosmetic (Norton 2006; Donkin 1977).

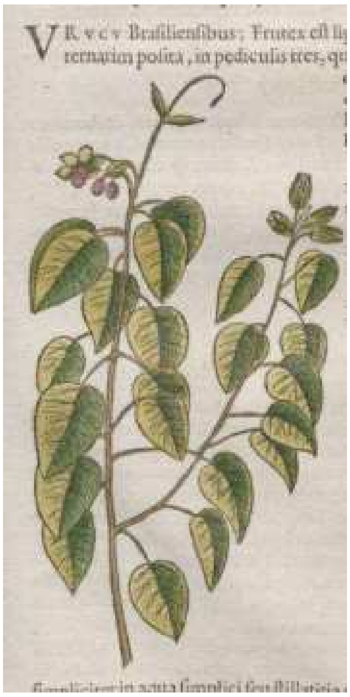

Historiae Plantarum – Plantis Frutescentibus & Fruticibus: 61b

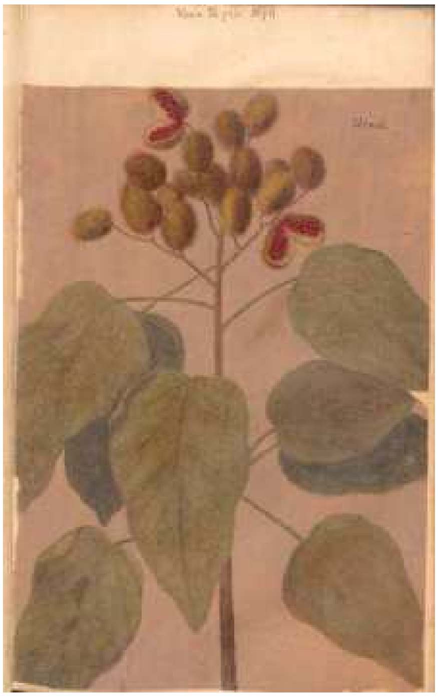

Theatrum Rerum Naturalium: 95

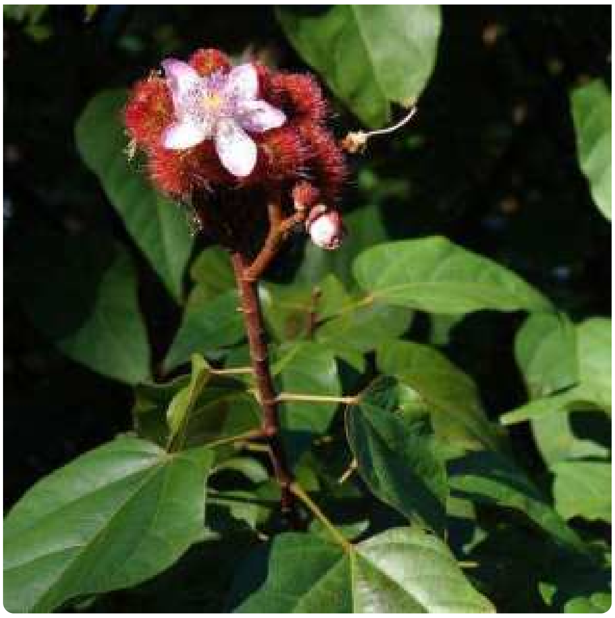

"*B. orellana* -'Lipstick tree'" by Tatters (CC BY 2.0)

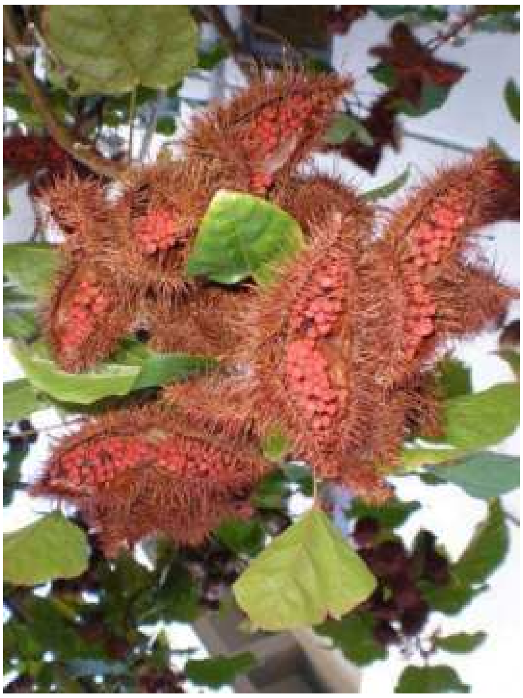

"Orange seeds of ripe annato/achiote (*B. orellana*)" by Joel Abroad (CC BY-NC-SA 2.0)

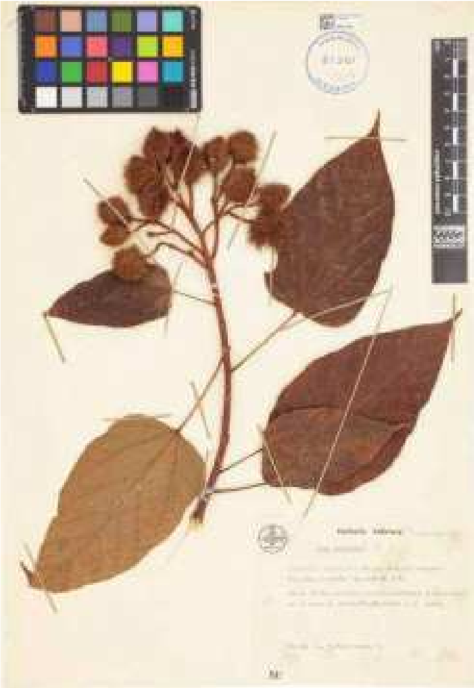

Specimen. "*B. orellana*" -FMB2136- by Herbario virtual FMB, Colombia (CC BY-NC 2.0)

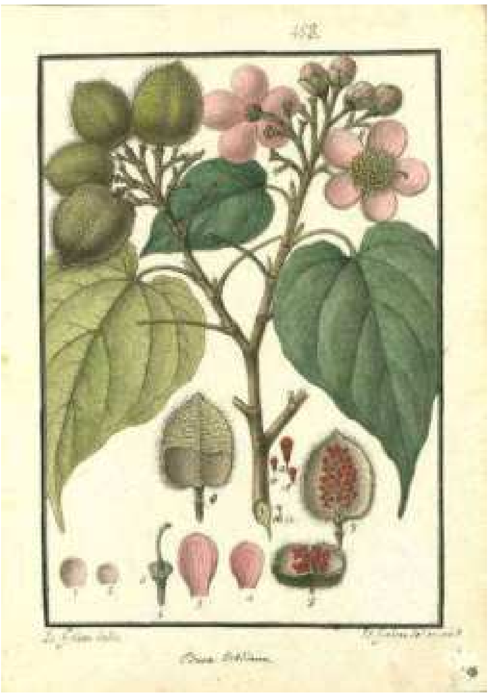

Drawings of the Royal Botanical Expedition to the Viceroyalty of Peru by Ruiz, H., Pavón, J. (1777). Real Jardín Botánico, Madrid, Spain

Historiae Rerum Naturalium Brasiliae | Marcgrave, 1648 | Page number 63a

Vernacular name(s): Frutex seu potius viscum quoddam

Species: Psittacanthus dichrous (Mart. ex Schult. & Schult.f.) Mart.

Family: Loranthaceae

Notes

We did not find any correspondence between this woodcut and the contemporary or older sources.

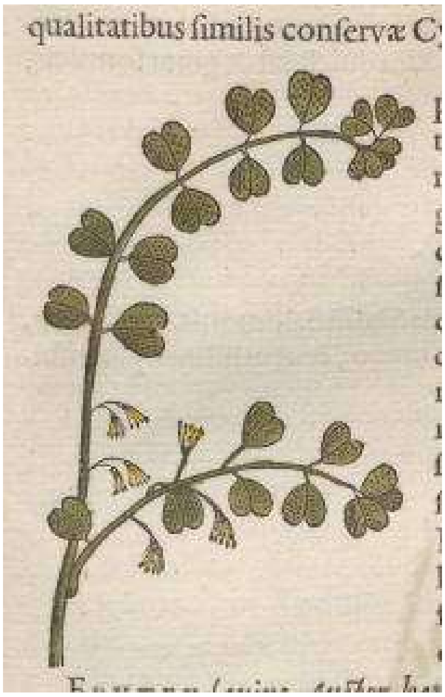

Historiae Plantarum – Plantis Frutescentibus & Fruticibus: 63a

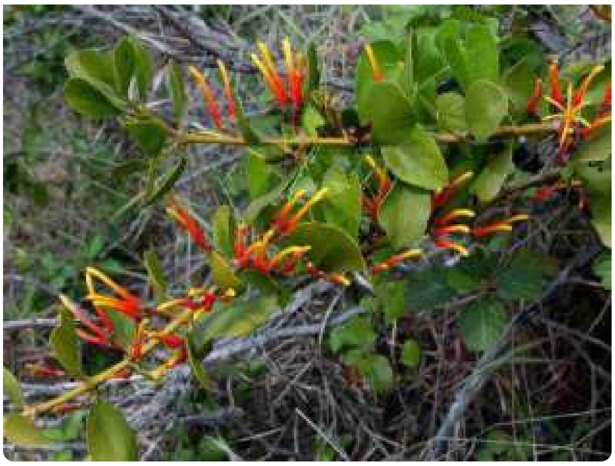

"*P. dichrous*" by Mauricio Mercadante (CC BY-NC-SA 2.0)

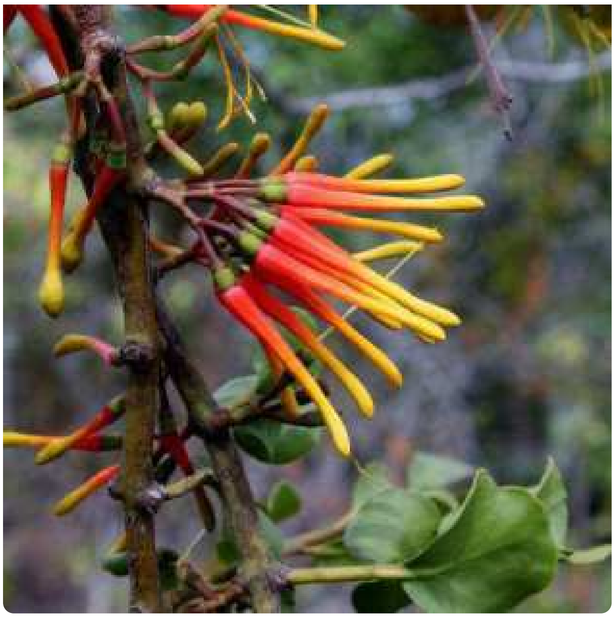

"*P. dichrous*" by Mauricio Mercadante (CC BY-NC-SA 2.0)

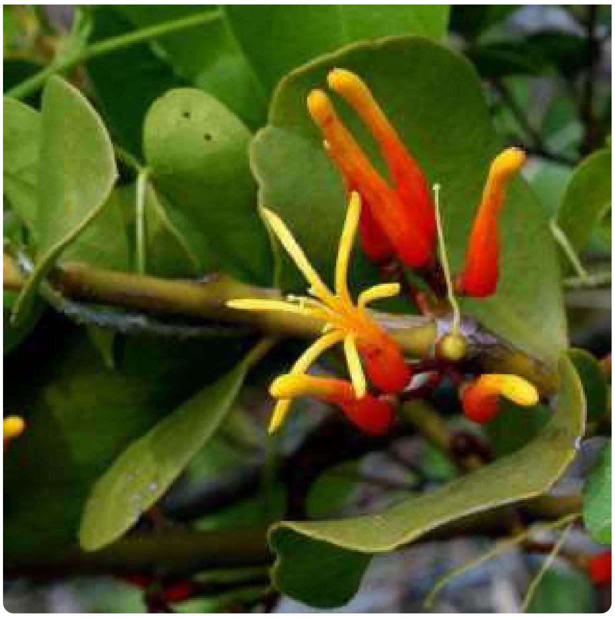

"*P. dichrous*" by Mauricio Mercadante (CC BY-NC-SA 2.0)

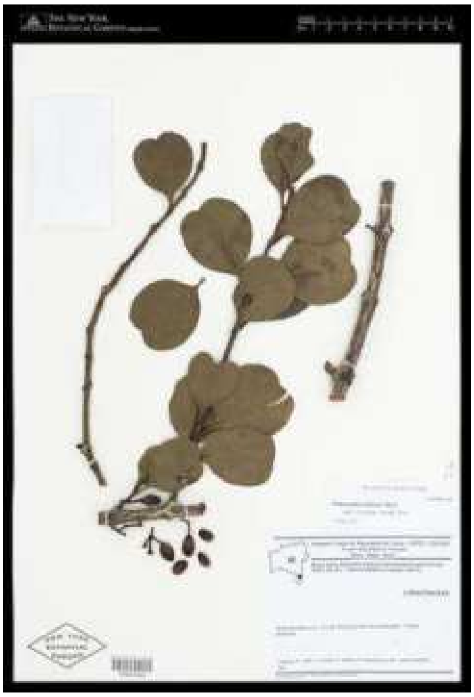

Specimen of *P. dichrous*. Collected in Brazil by The New York Botanical Garden - 03687059- (CC BY 4.0)

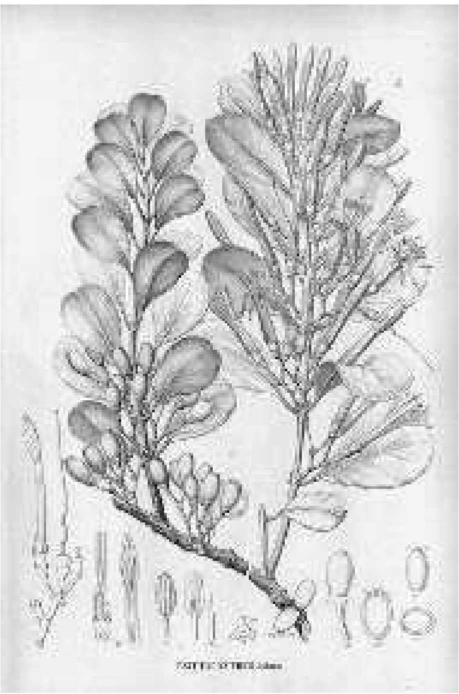

Engraving of *P. dichrous* in Martius, C.F.P. von, Eichler, A.G., Urban, I., *Flora Brasiliensis* (1866-1868) Vol. 5(2): 5

Historiae Rerum Naturalium Brasiliae | Marcgrave, 1648 | Page number 63b

Vernacular name(s) Futex

Species Cynophalla hastata (Jacq.) J.Presl

Family Capparaceae

Notes

The woodcut differs from the *Theatrum* image. The woodcut shows a flowering branch, while the oil painting depicts two long pods, one of them open and showing numerous seeds.

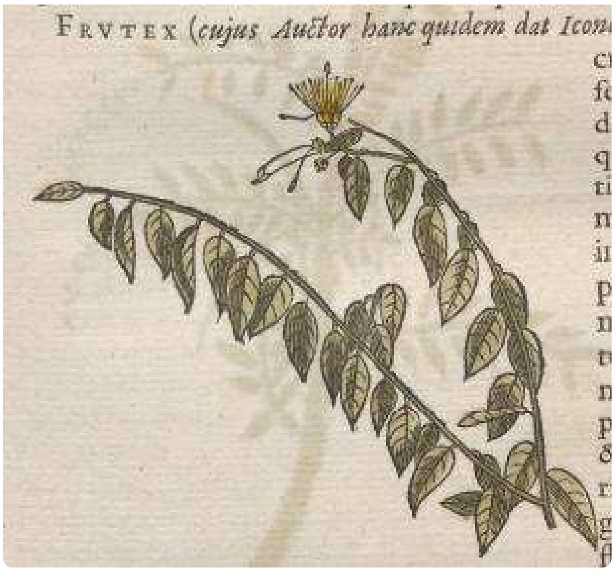

Historiae Plantarum – Plantis Frutescentibus & Fruticibus: 63b

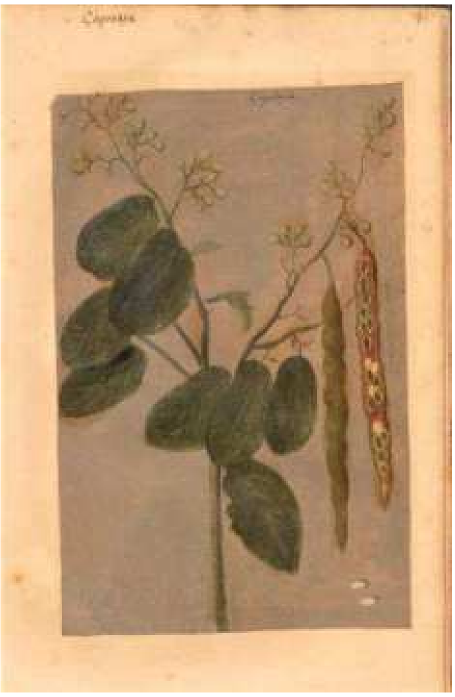

Theatrum Rerum Naturalium: 417

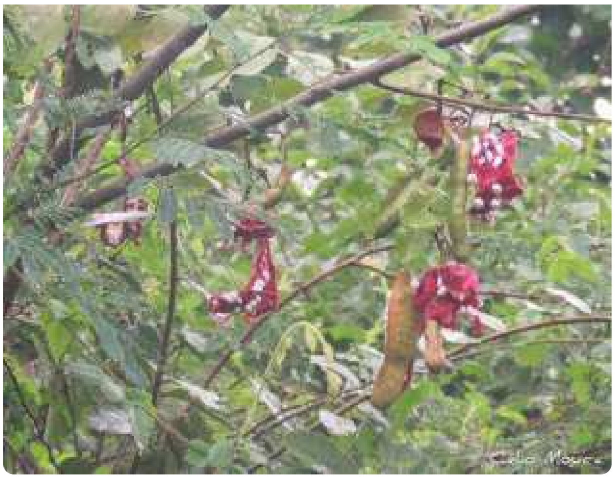

C. hastata observed in Brazil
for iNaturalist by Célio Moura Neto (CC BY-NC 4.0)

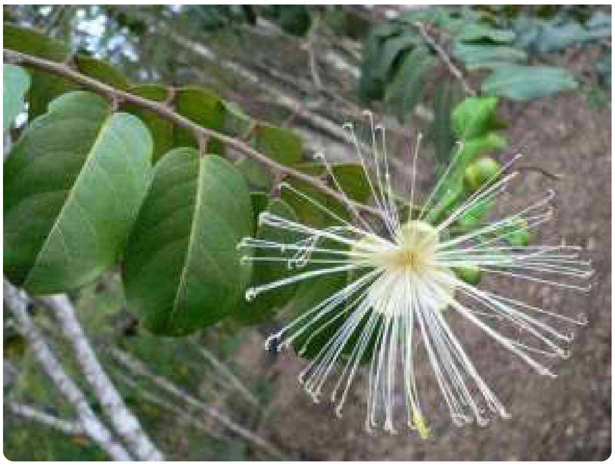

Capparis hastata [syn. of *Cynophalla hastata*], feuilles et fleurs, Guadeloupeby Pancrat (CC BY-SA 3.0)

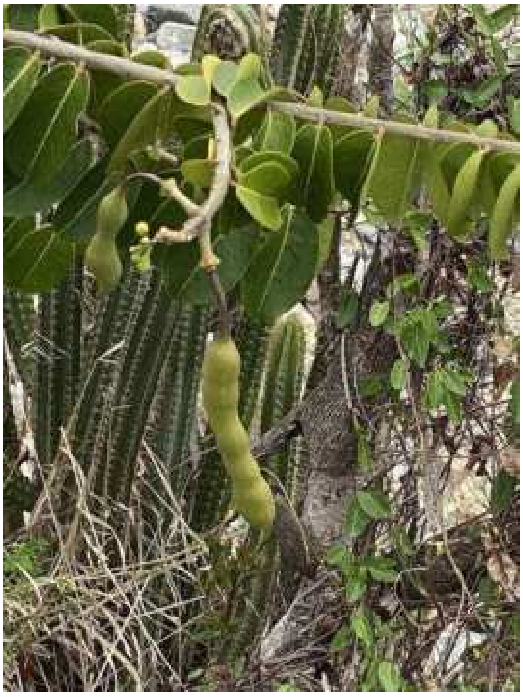

C. hastata observed in Saint Martin
for iNaturalist by endretoth (CC BY-NC 4.0)

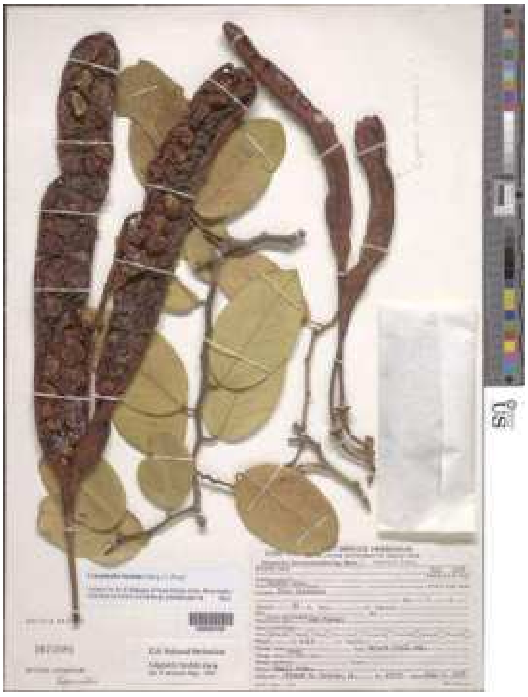

"*C. hastata*" by Elbert Luther Little Jr.- 00658108- Smithsonian National Museum of Natural History (CC0 1.0)

Historiae Rerum Naturalium Brasiliae	Marcgrave, 1648	Page number	63c

Vernacular name(s) Iva, Iurepeba

Species Solanum lycocarpum A. St.-Hil.

Family Solanaceae

Notes

We did not find any correspondence between this woodcut and the contemporary or older sources.

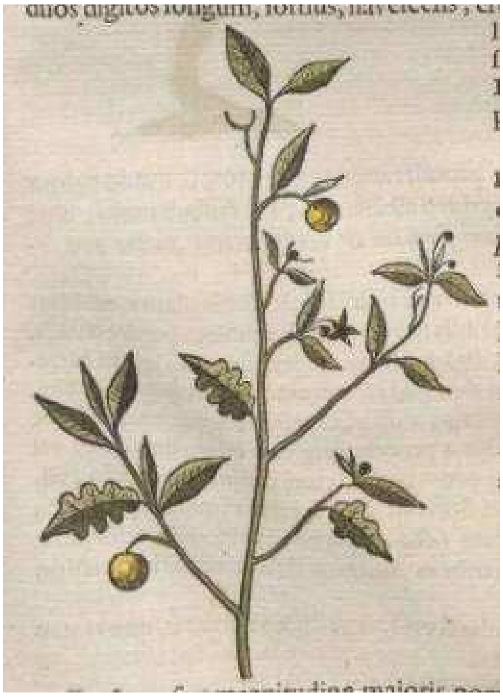

Historiae Plantarum – Plantis Frutescentibus & Fruticibus: 63c

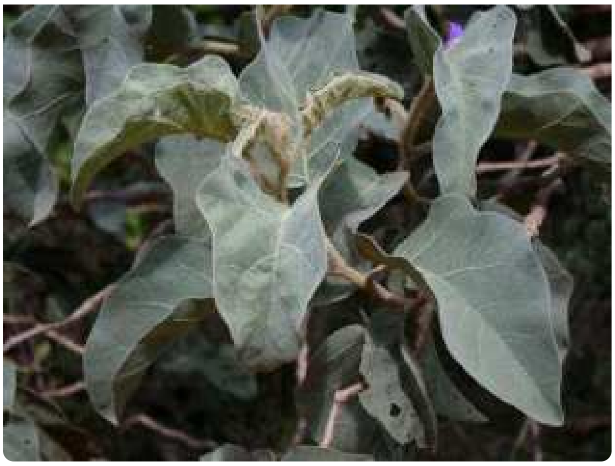

"*Solanum lycocarpum*" by Mauricio Mercadante (CC BY-NC-SA 2.0)

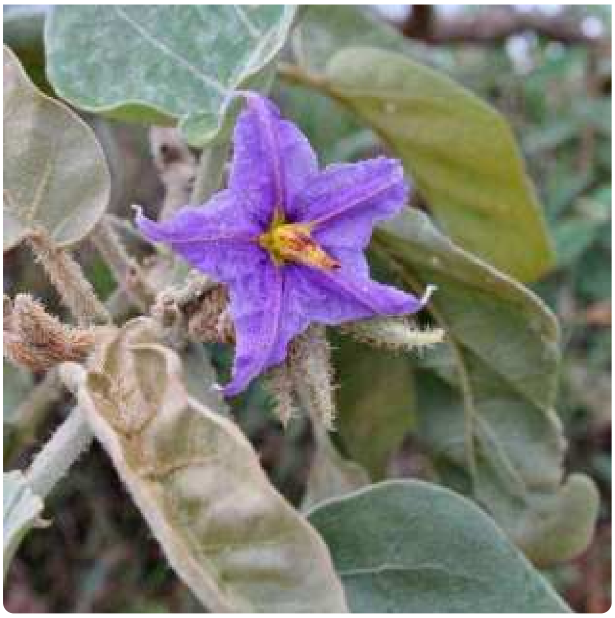

Flower. "*Solanum lycocarpum*" by Mauricio Mercadante (CC BY-NC-SA 2.0)

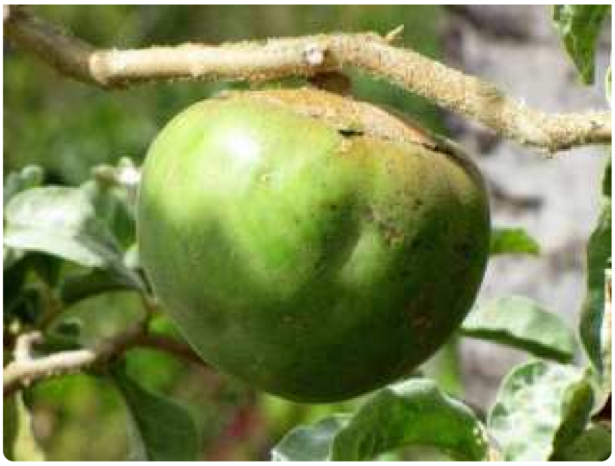

Fruit. "*Solanum lycocarpum*" by Mauricio Mercadante (CC BY-NC-SA 2.0)

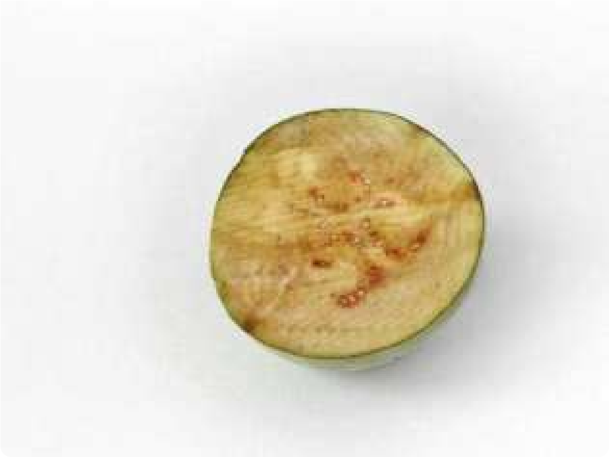

Open fruit and seeds, "*Solanum lycocarpum*" by Mauricio Mercadante (CC BY-NC-SA 2.0)

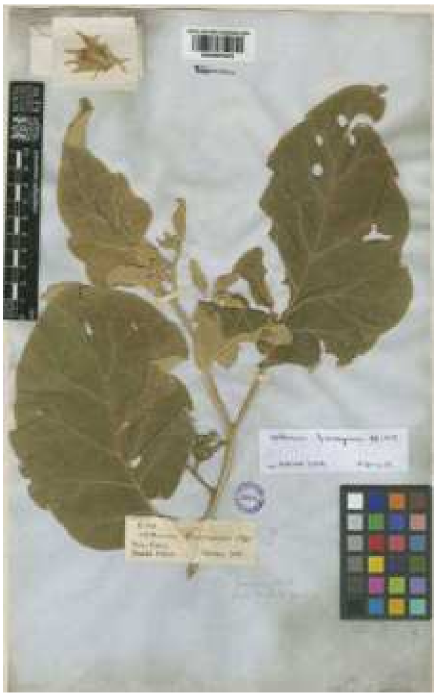

Specimen of *S. lycocarpum* from Kew's Herbarium - K000983083. Retrieved from Plants of the World Online

Historiae Rerum Naturalium Brasiliae Marcgrave, 1648 Page number 64

Vernacular name(s) Atitara. Iatitara

Species Desmoncus polyacanthos Mart.

Family Arecaceae

Notes

Although the woodcut and the specimens do not bear a strong resemblance, the compound leaves of the specimens could have been used as models to elaborate the image depicted in the woodcut, which attempts to portray the palm tree.

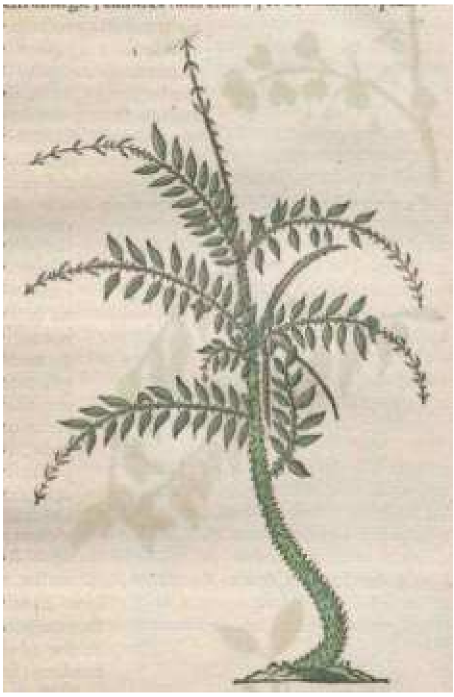

Historiae Plantarum – Plantis Frutescentibus & Fruticibus: 64

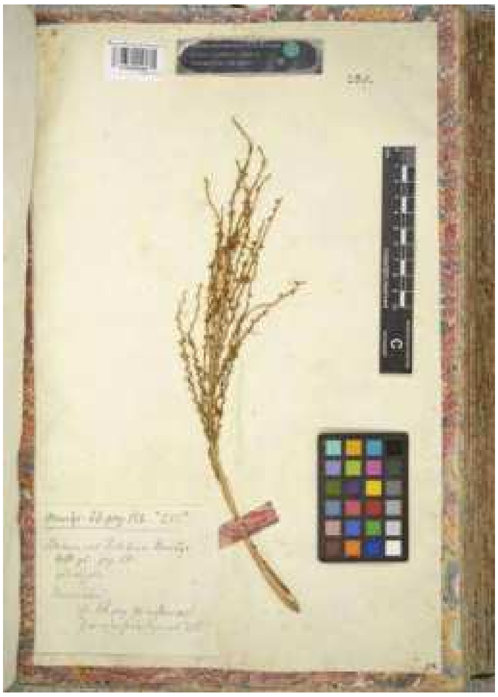

Marcgrave's herbarium: 98

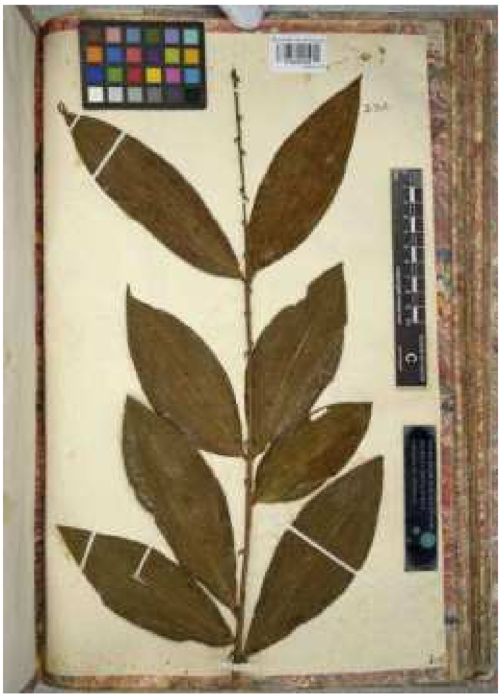

Marcgrave's herbarium: 156

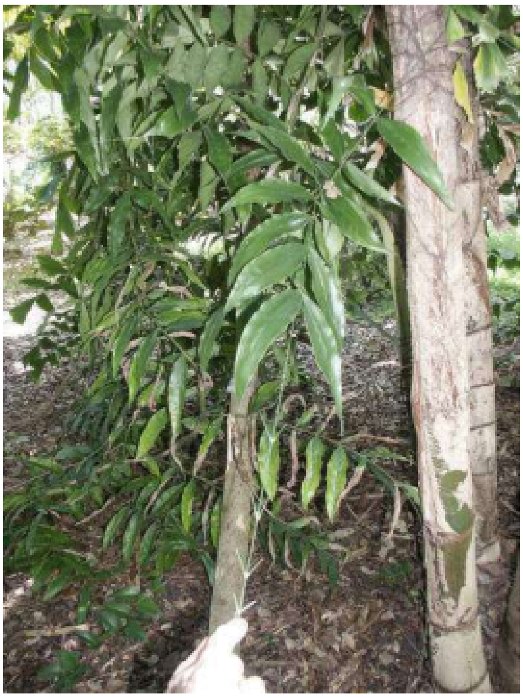

"*D. polyacanthos*" by Scott Zona (CC BY-NC 2.0)

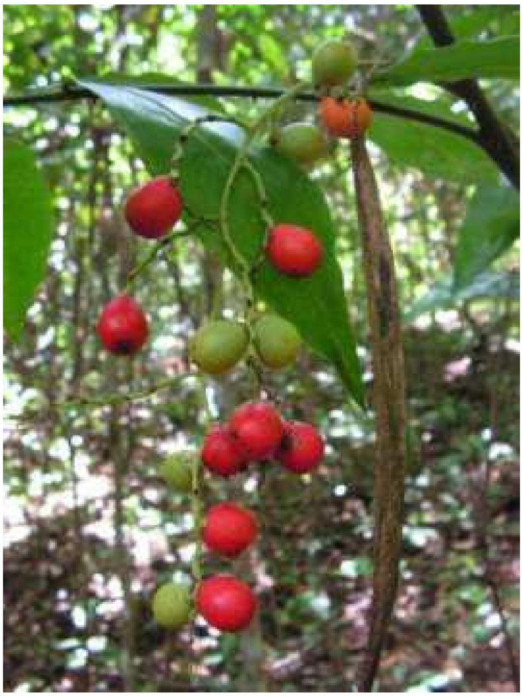

"*D. polyacanthos*, titara" by Tarciso Leão (CC BY 2.0)

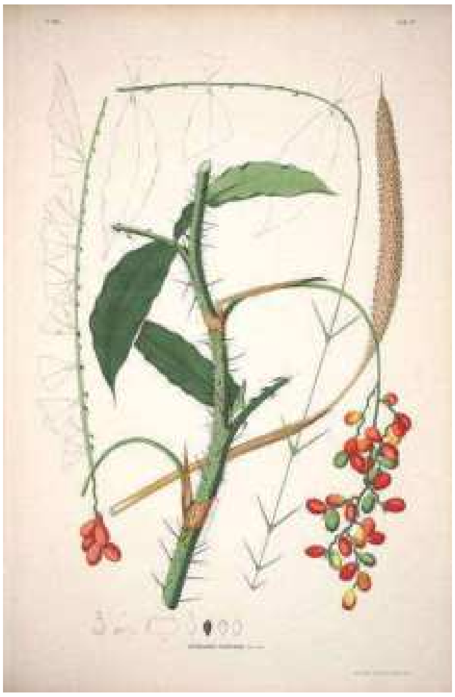

Sertum palmarum brasiliensium by Rodrigues, J. Barbosa (1903: Vol. II, t. 57). Missouri Botanical Garden, St. Louis, U.S.A.

Historiae Rerum Naturalium Brasiliae Marcgrave, 1648 Page number 69a

Vernacular name(s) Manaca

Species Brunfelsia uniflora (Pohl) D.Don

Family Solanaceae

Notes

The woodcut is moderately similar to the *Theatrum*. The woodcut seems a composite image created after the two illustrations. These were likely made by two different artists, who wrote the vernacular names differently in the illustration. One of the illustrations depicts flowers with different colors (as in Marcgrave's description) and some fruits, while the other shows green fruits enclosed by the calyx. The long flower peduncles are well depicted in the woodcut and the red fruits from the illustration in the *Theatrum* (above), with the star-like five crevices in the upper part (as also described by Marcgrave) are lacking in the HNB, but are illustrated wrongly in the *Theatrum.*

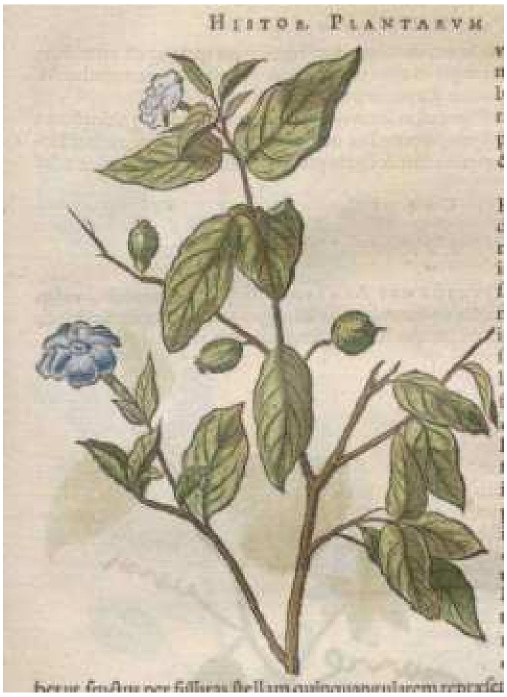

Historiae Plantarum – Plantis Frutescentibus & Fruticibus: 69a

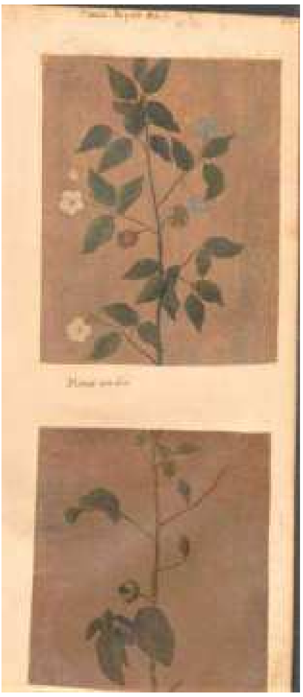

Theatrum Rerum Naturalium: 233

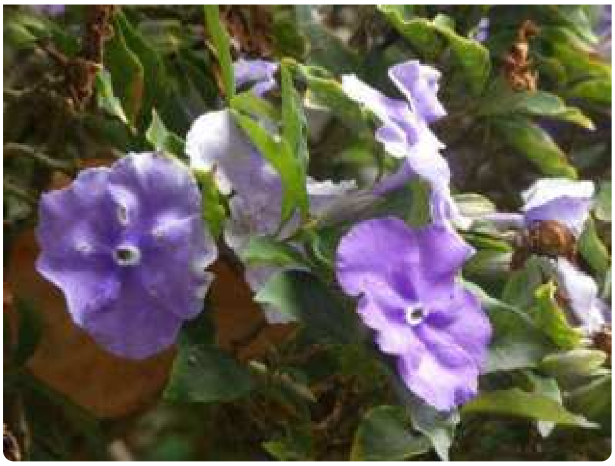

Flowers. "*B. uniflora*" by Linda DV (CC BY-NC-ND 2.0)

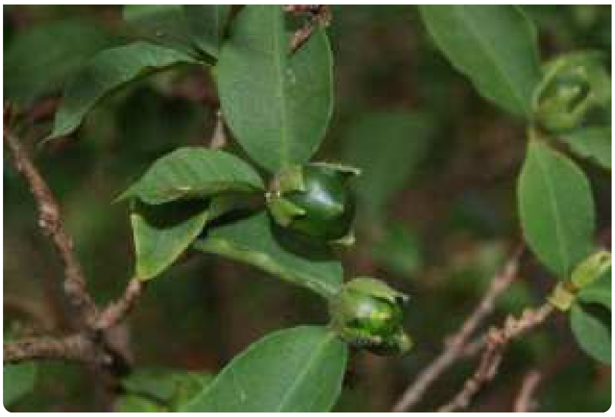

Fruits. *"Brunfelsia uniflor*a, Frucht" by Danny S. (CC-BY-SA-4.0)

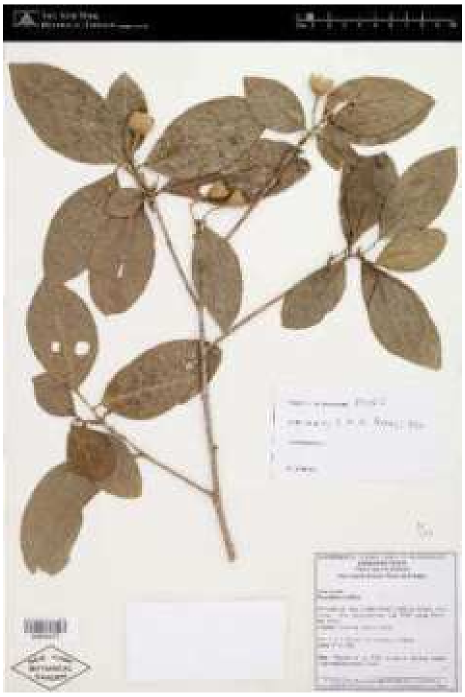

Specimen of *B. uniflora*, collected in Brazil by The New York Botanical Garden -655973- (CC BY 4.0)

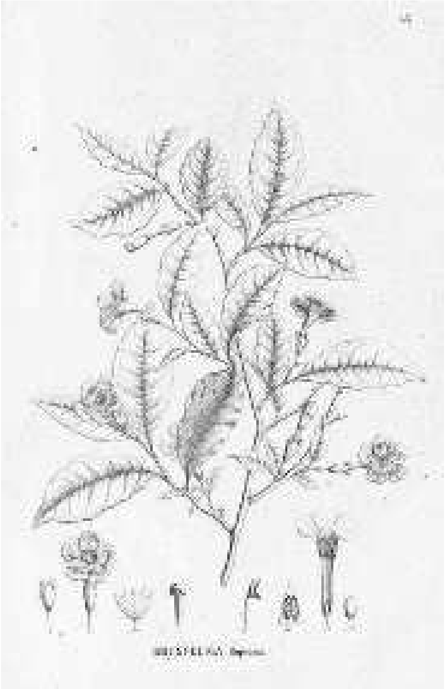

Engraving of *B. uniflora* in Martius, C.F.P. von, Eichler, A.G., Urban, I., *Flora Brasiliensis* (1857-1864) Vol. 8 (1): 43

Historiae Rerum Naturalium Brasiliae Marcgrave, 1648 Page number 69b

Vernacular name(s) Iaborandi

Species Piper anisum (Spreng.) Angely

Family Piperaceae

Notes

The woodcut differs from the *Theatrum* image. While the former depicts a sterile plant, the latter shows the inflorescence in the form of spikes characteristic of the Piperaceae botanical family. This species was depicted twice in the HNB, but differently. The other woodcut is present in Piso's chapter on medicinal plants and it is very similar to the *Theatrum* illustration for the same species.

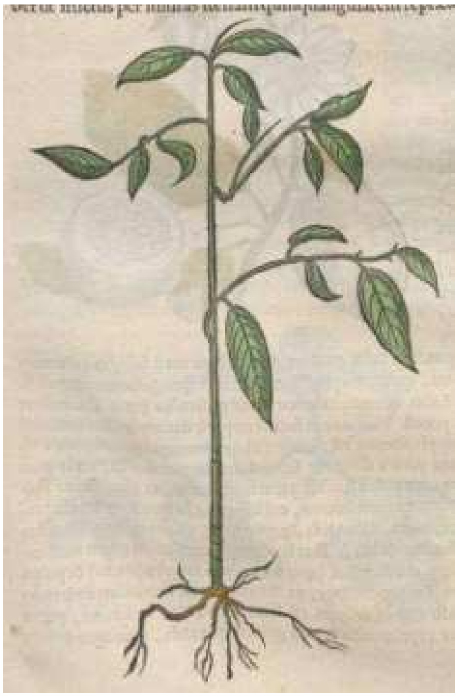

Historiae Plantarum – Plantis Frutescentibus & Fruticibus: 69b

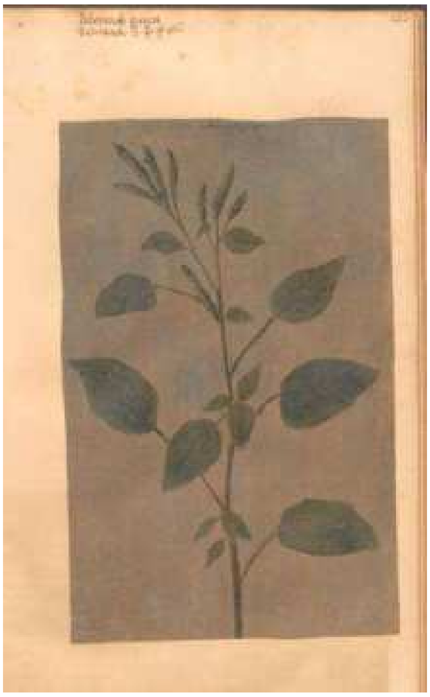

Theatrum Rerum Naturalium: 313

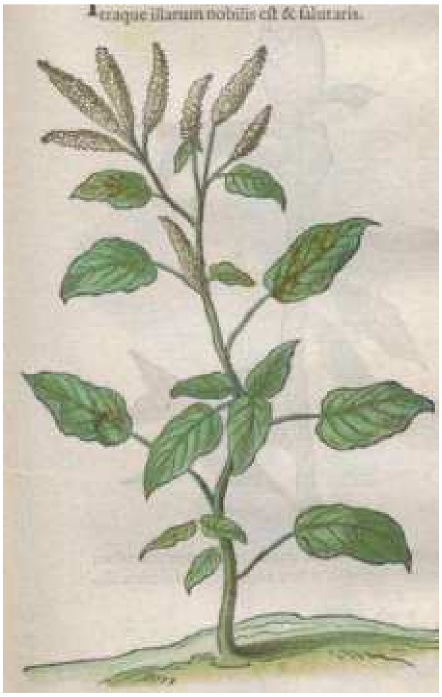

P. anisum depicted in Piso's chapter on medicinal plants (HNB, 1648: 97)

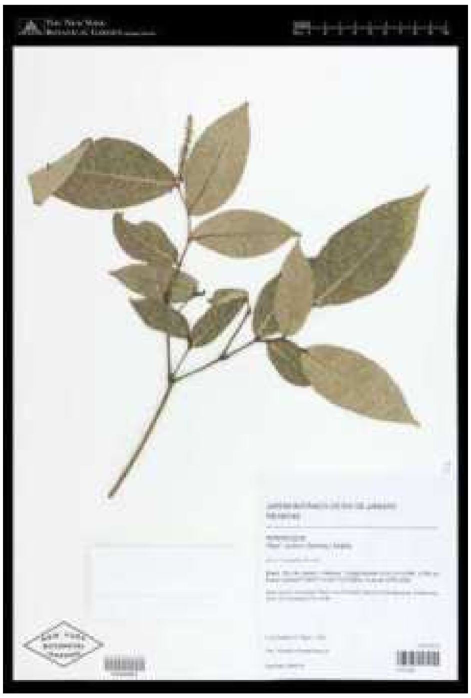

Specimen of *P. anisum* collected in Brazil -04088992- for The New York Botanical Garden (CC BY 4.0)

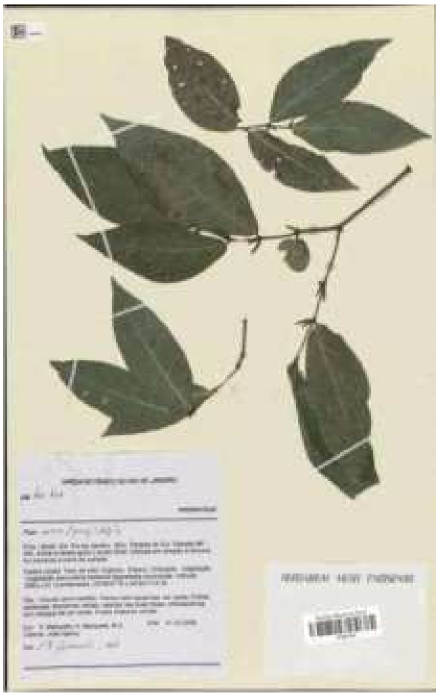

Specimen of *P. anisum* collected in Brazil - P01981384- For the Muséum national d'Histoire Naturelle, Paris (CC BY 4.0)

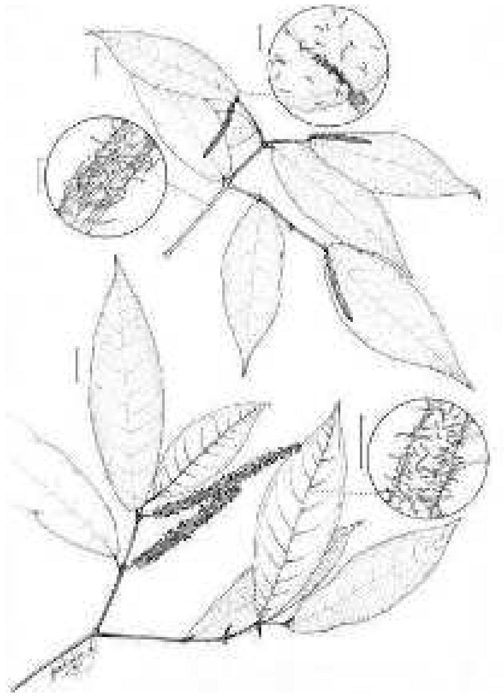

P. anisum. Monteiro 2013. *Piperaceae in a fragment of atlantic florest of the Serra da Mantiqueira, Minas Gerais, Brazil.* Rodriguésia, 64: 379-398

Historiae Rerum Naturalium Brasiliae	Marcgrave, 1648	Page number	70

Vernacular name(s) Murucuia guaçu

Species Passiflora quadrangularis L.

Family Passifloraceae

Notes

The woodcut is very similar to the *Theatrum* image (non-reversed). The color of the flower differs, although we found that the copy of the HNB in the Berlin State Library (Staatsbibliothek zu Berlin) bears a flower that was colored similarly to the *Theatrum* (https://digital.staatsbibliothek-berlin.de/werkansicht/?PPN=PPN1735987395)

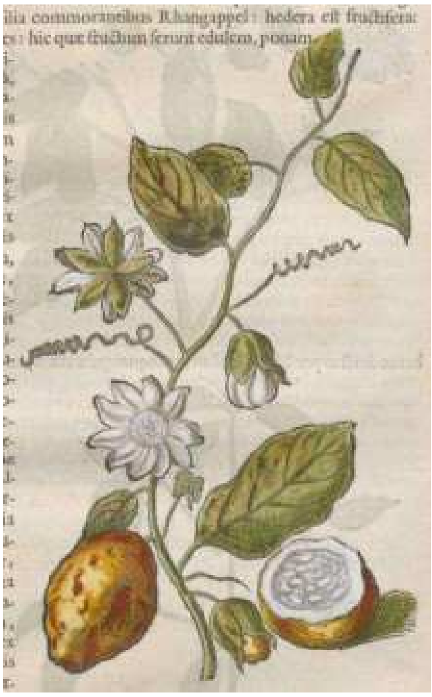

Historiae Plantarum – Plantis Frutescentibus & Fruticibus: 70

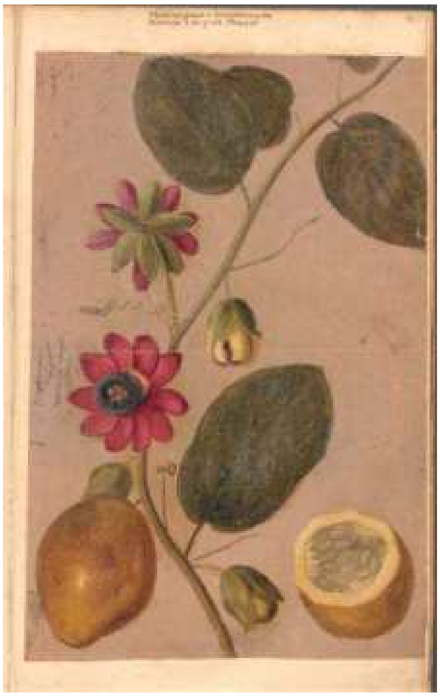

Theatrum Rerum Naturalium: 437

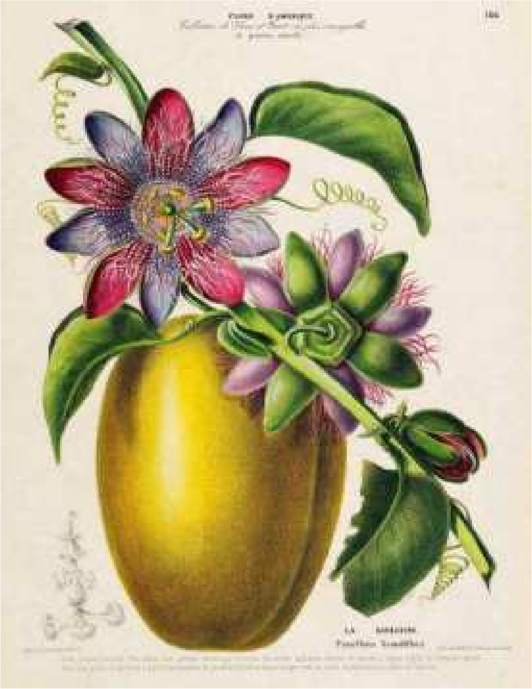

Flore d'Amérique by Denisse, E.,t. (1843-1846: t. 166) "La Barbadine, giant granadilla. *P. quadrangularis* by Swallowtail Garden Seeds

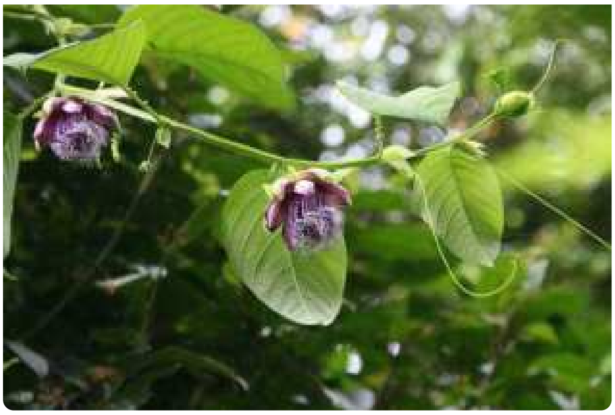

"*Passiflora quadrangularis* (Passifloraceae)" by Dr. Alexey Yakovlev (CC BY-SA 2.0)

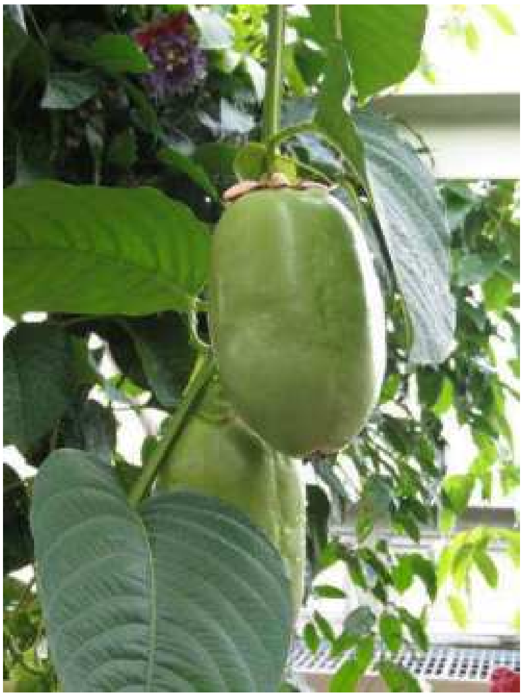

Fruit. "*P. quadrangularis*" by mmmavocado (CC BY 2.0.)

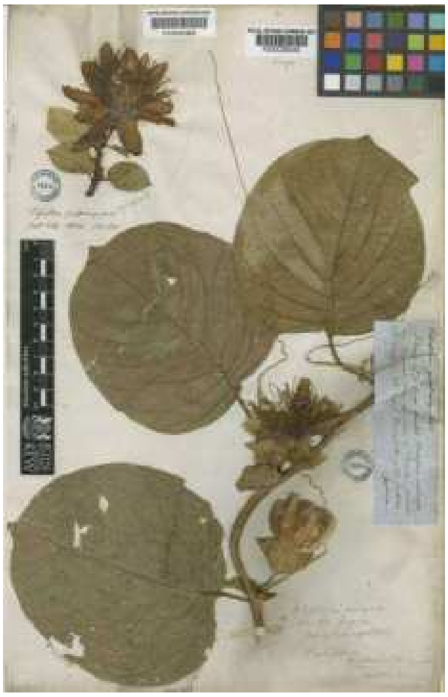

Specimen of *P. quadrangualaris* from Kew's Herbarium - K000036546. Retrieved from Plants of the World Online

Historiae Rerum Naturalium Brasiliae Marcgrave, 1648 Page number 71a

Vernacular name(s) Murucuia maliformis

Species Passiflora cincinnata Mast

Family Passifloraceae

Notes

The woodcut does not resemble the watercolor in the *Libri Principis*. Notice that the woodcut depicts also the fruit. Both images accurately depict the five-lobed leaves, which can also be middle-lobed, as seen in the woodcut. This species was represented with a different woodcut in Piso's (1648: 106) section on medicinal plants in the HNB.

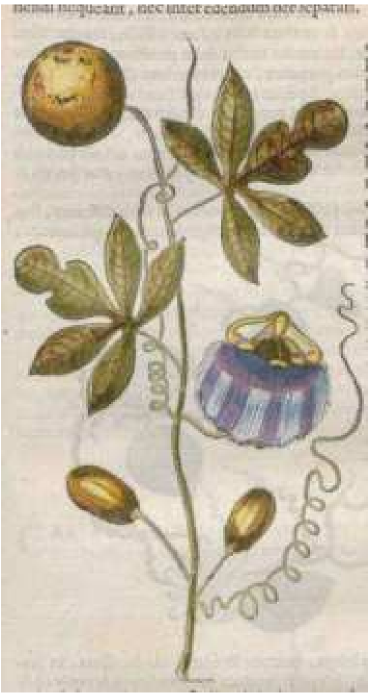

Historiae Plantarum – Plantis Frutescentibus & Fruticibus: 71a

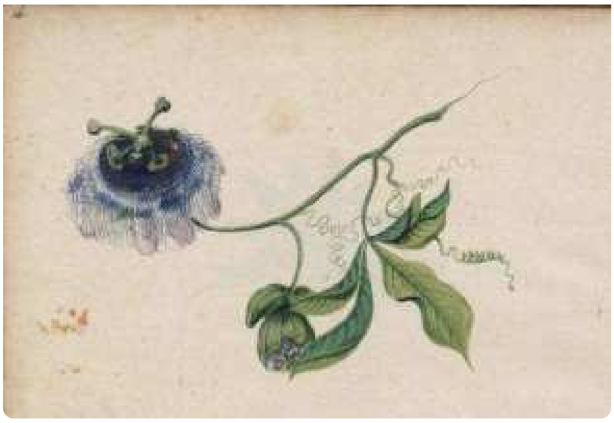

Libri Principis: f. 16

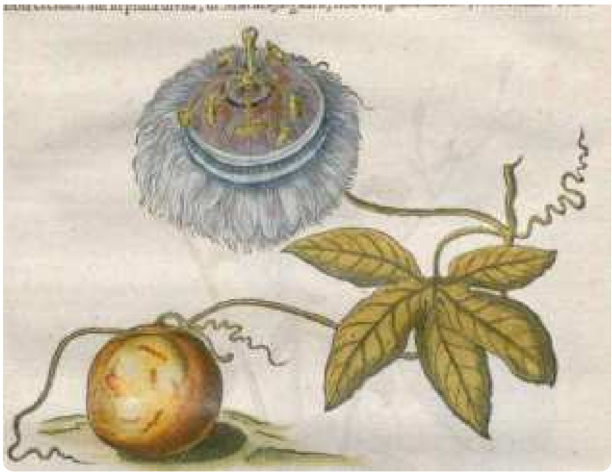

The other woodcut of *P. cincinnata* in Piso (HNB, 1648: 106)

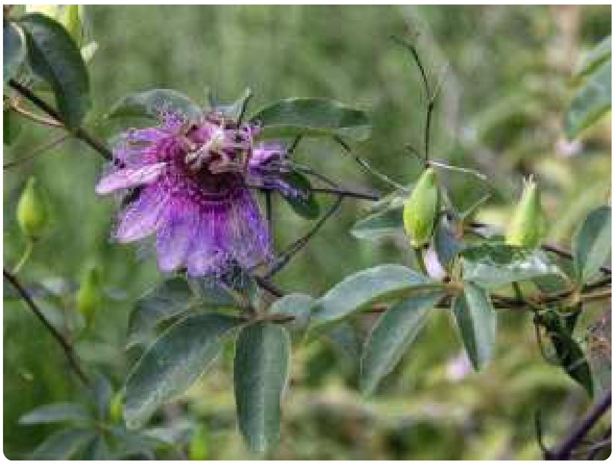

"*P. cincinnata*" by Mauricio Mercadante (CC BY-NC-SA 2.0)

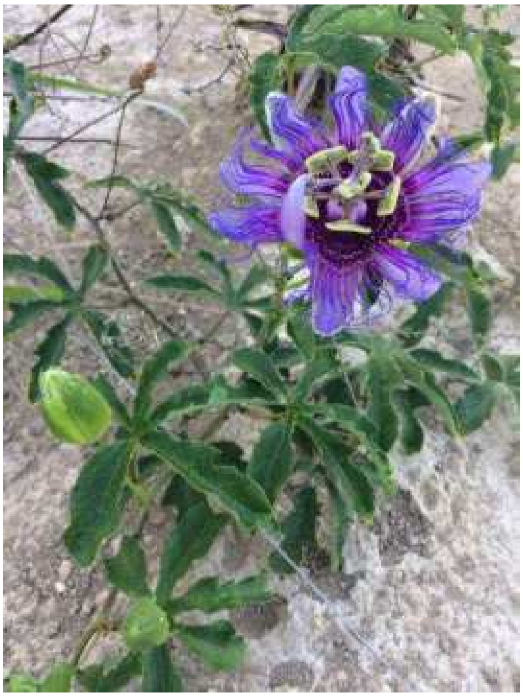

P. cincinnata observed in Brazil for iNaturalist by Ben P. (CC BY-NC 4.0)

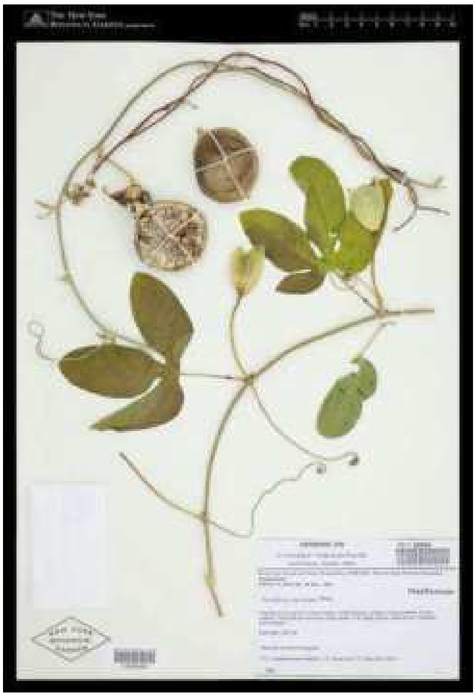

Specimen of *P. cincinnata* collected in Brazil -01806460- for The New York Botanical Garden (CC BY 4.0)

Historiae Rerum Naturalium Brasiliae Marcgrave, 1648 Page number 71b

Vernacular name(s) Murucuia maliformis alia

Species Passiflora edulis Sims

Family Passifloraceae

Notes

The woodcut differs from the other sources. The flowering vine in the basket of flowers depicted by Ekchout represents the same species as the woodcut. The still-life " watermelon, pineapple and other fruits," by Ekchout also shows *P. edulis*. However, the woodcut shows one fruit and no flowers, while the painting depicts flowers and fruits, with the fruit being more similar to *P. alata* (Buvelot et al. 2004) than *P. edulis* and the shoot with flowers to *P. edulis*.

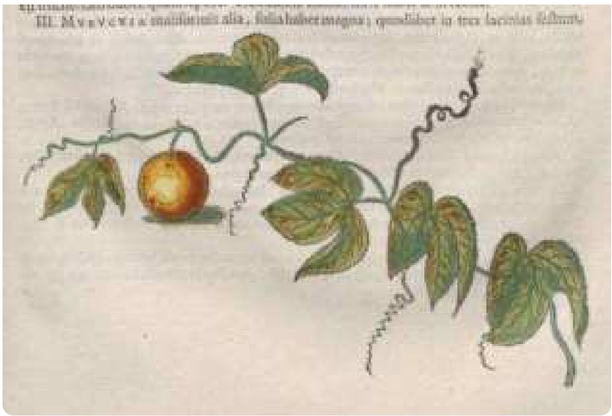

Historiae Plantarum – Plantis Frutescentibus & Fruticibus: 71b

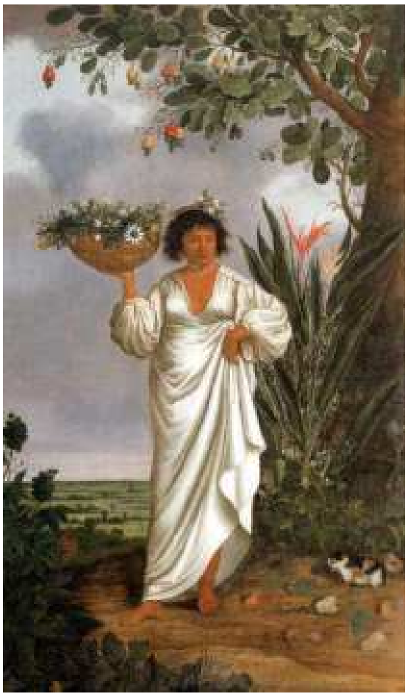

Ekchout portrait "Mameluka with a basket of flowers", ca. 1640. Copenhagen, National Museum of Denmark

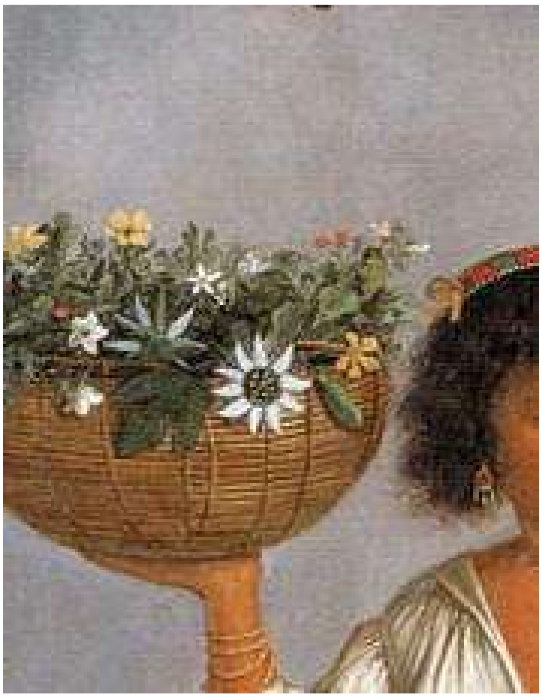

Close - up of the *P. edulis* shoot in Ekchout's portrait "Mameluka with a basket of flowers", ca. 1640. Copenhagen, National Museum of Denmark

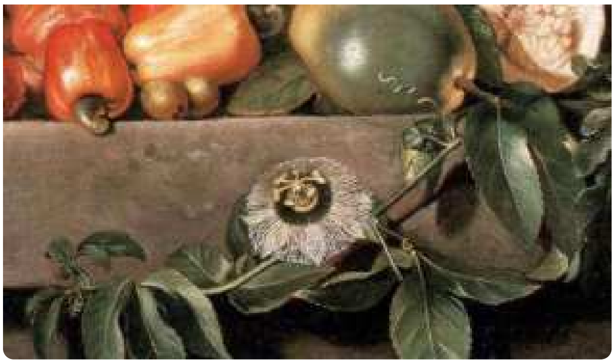

Close - up of the *P. edulis* shoot in Ekchout's still-life with watermelon, pineapple and other fruits, ca. 1640. Copenhagen, National Museum of Denmark

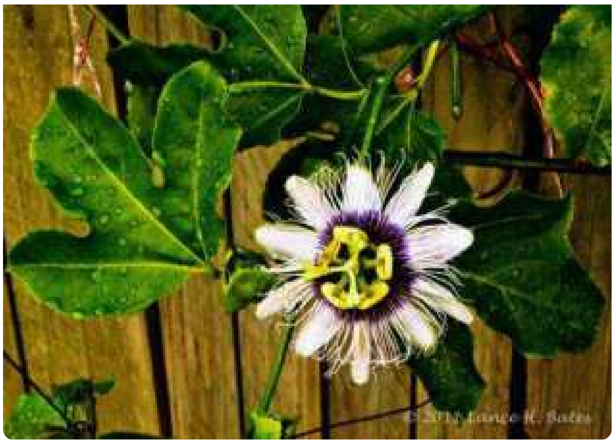

"20110510 Passion Fruit (*P. edulis*) Flower with Rain-drops" by Degilbo on flickr (CC BY-NC-ND 2.0)

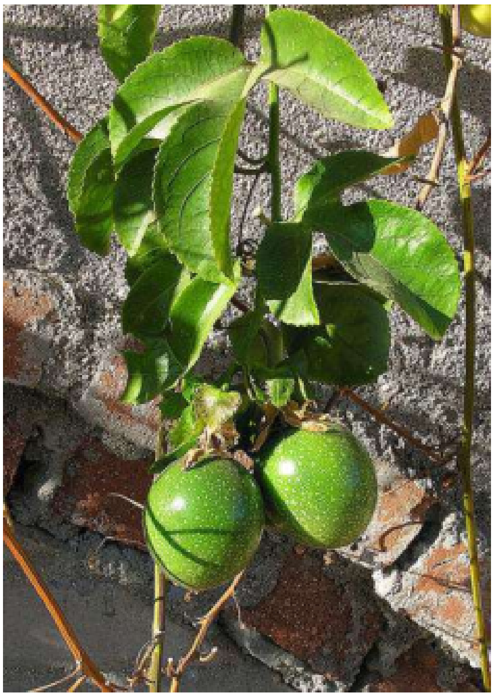

"*P. edulis*, a species of the southern Neotropics, but widely planted for its flowers and edible fruit". Chihuahua, Mexico, by Dick Culbert (CC BY 2.0)

Historiae Rerum Naturalium Brasiliae Marcgrave, 1648 Page number 72

Vernacular name(s) Murucuia piriformis altera

Species Passiflora alata Curtis

Family Passifloraceae

Notes

The correspondence between woodcut and specimen is hard to tell because the exsicata seems incomplete. The watercolor in the *Libri Principis* could represent another species of *Passiflora*, as the flower does not match completely with *P. alata*. The color of the flower differs between the copy of the HNB in the Staatsbibliothek Zu Berlin and the copy of the HNB kept in Leiden University (see *P. quadrangularis*).

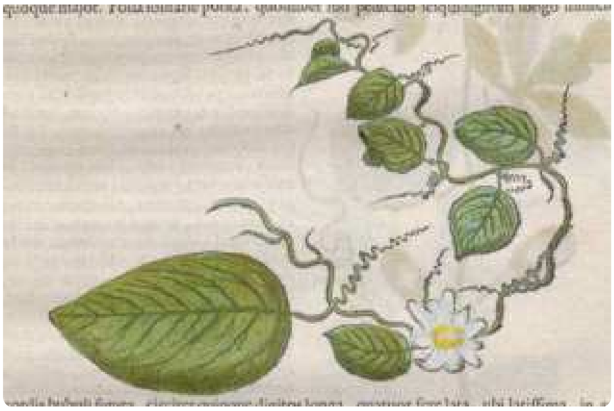

Historiae Plantarum – Plantis Frutescentibus & Fruticibus: 72

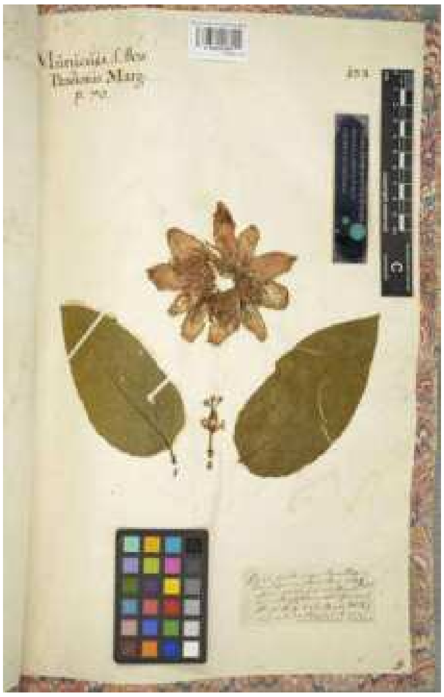

Marcgrave's herbarium: 9

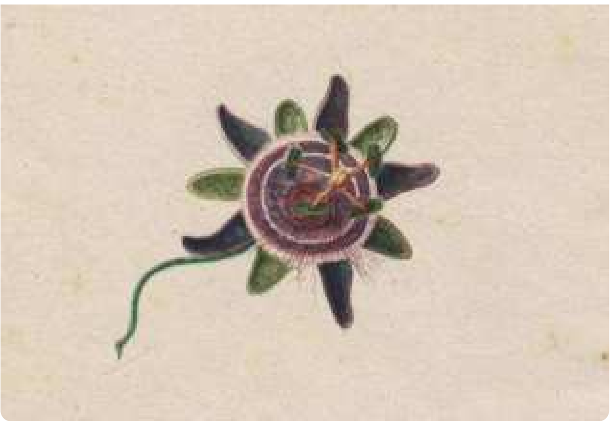

Libri Principis: f. 14

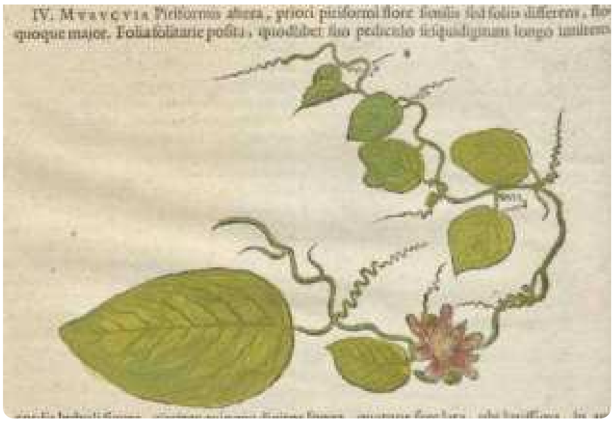

HNB colored copy in the Staatsbibliothek zu Berlin (Marcgrave 1648: 72)

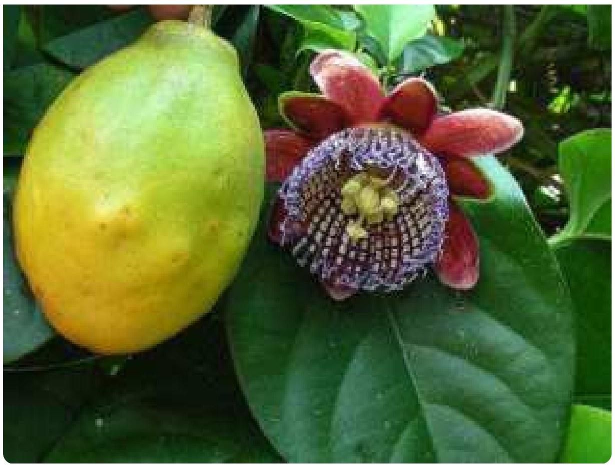

"*P. alata*" by Dick Culbert (CC BY 2.0)

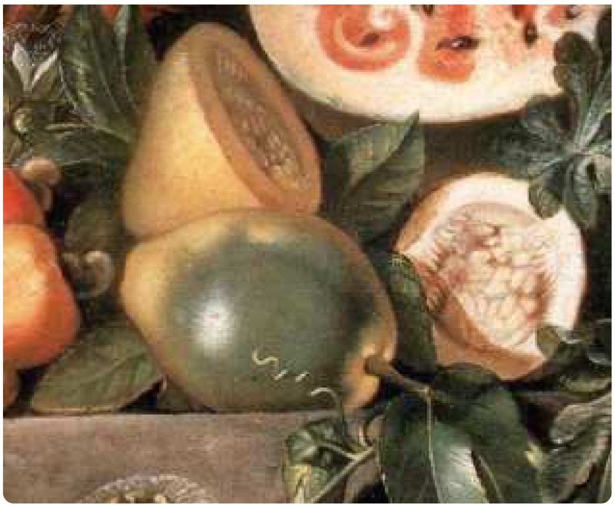

Close - up of the *P. alata* fruit in Ekchout's still-life with watermelon, pineapple and other fruits, ca. 1640. Copenhagen, National Museum of Denmark

Historiae Rerum Naturalium Brasiliae	Marcgrave, 1648	Page number	73

Vernacular name(s)	Caaeo. Herba viva
Species	Mimosa candollei R.Grether
Family	Fabaceae
Notes	

The correlation is not very strong, but both are flowering shoots that could be related. In Jakob Breyne (1678: 37) there is an engraving of this species, which he likely made after a duplicate collected by Marcgrave in Brazil.

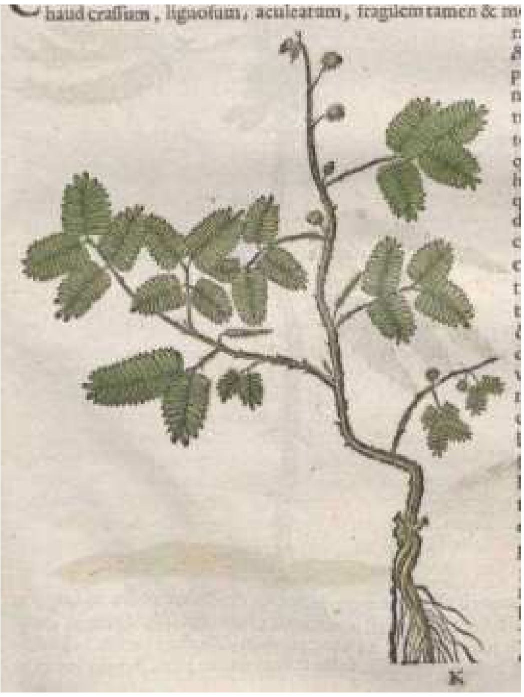

Historiae Plantarum – Plantis Frutescentibus & Fruticibus: 73

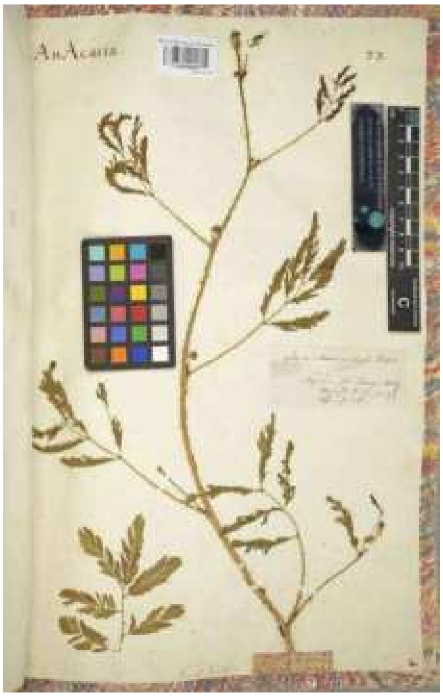

Marcgrave's herbarium: 2

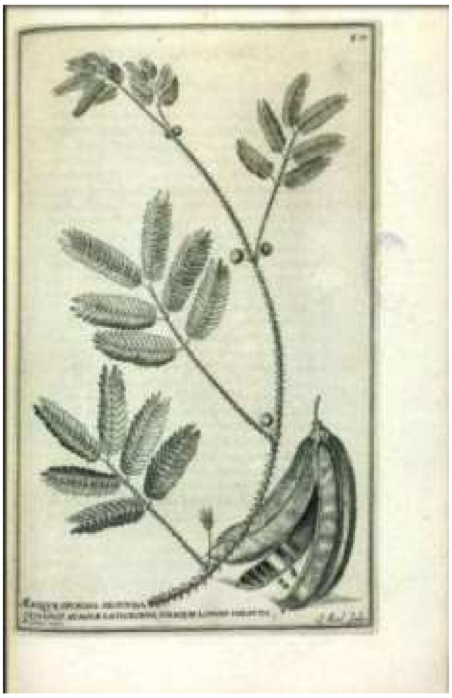

Engraving of *M.* cf. *candollei* in *Exoticarum aliarumque minus cognitarum plantarum centuria prima* by Jakob Breyne (1678: 37)

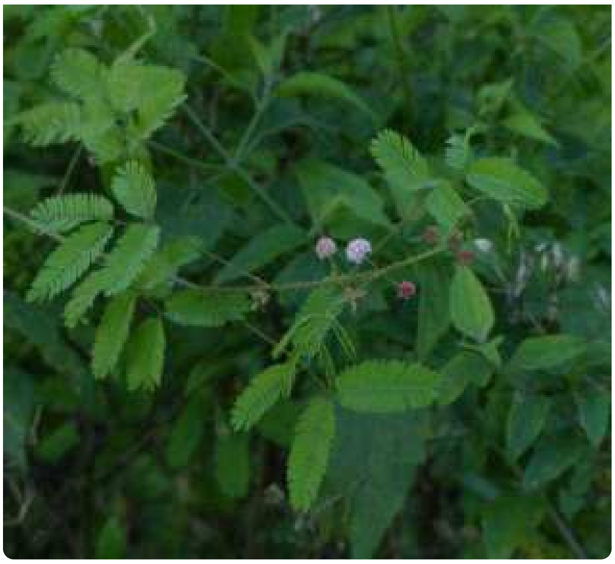

M. candollei observed in Brazil
for iNaturalist by Helio Lourencini (CC BY-NC 4.0)

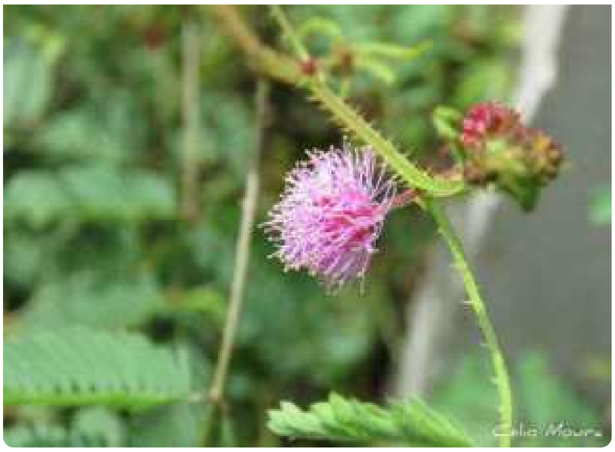

M. candollei observed in Brazil
for iNaturalist by Célio Moura Neto (CC BY-NC 4.0)

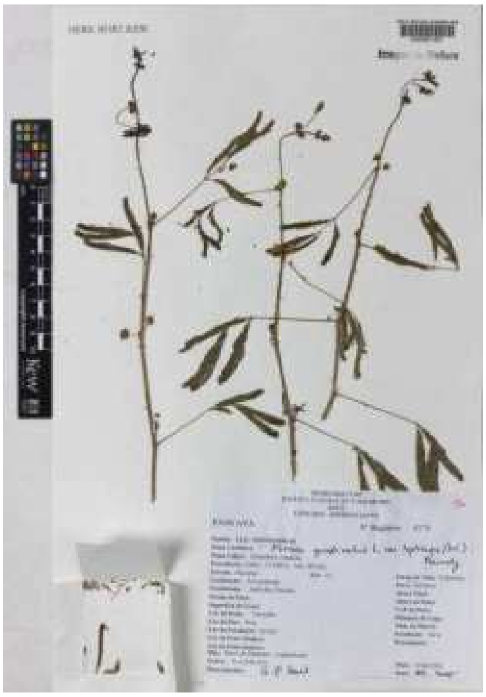

Specimen of *M. candollei* collected in Brazil
by Royal Botanic Gardens, Kew -K000851567-
Retrieved from GBIF

Historiae Rerum Naturalium Brasiliae Marcgrave, 1648 Page number 74

Vernacular name(s) Tertia spécies

Species Mimosa polydactyla Humb. et Bonpl.

Family Fabaceae

Notes

There is a strong correlation between the woodcut and the specimen, especially if we compare the woodcut with the upper half of the specimen.

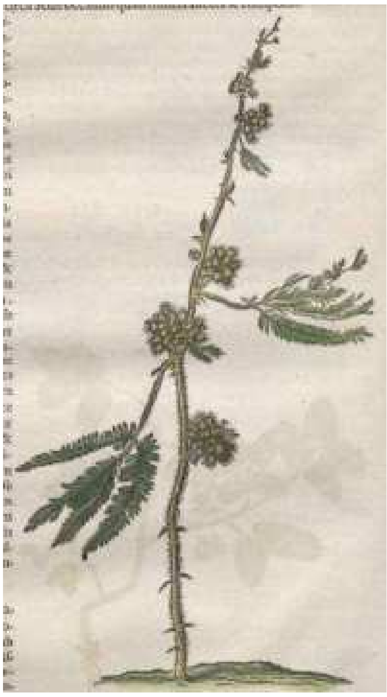

Historiae Plantarum – Plantis Frutescentibus & Fruticibus: 74

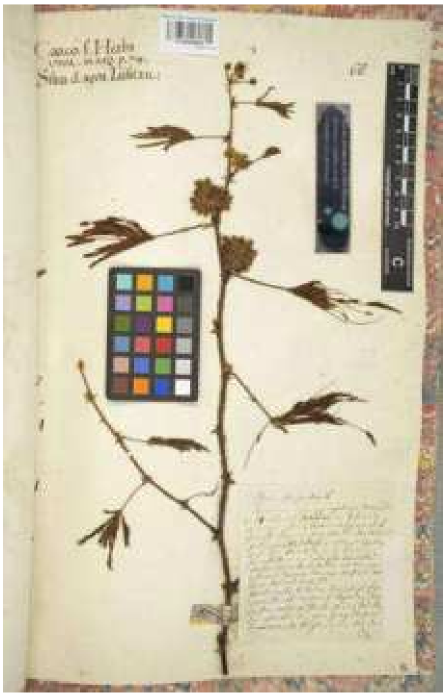

Marcgrave's herbarium: 8

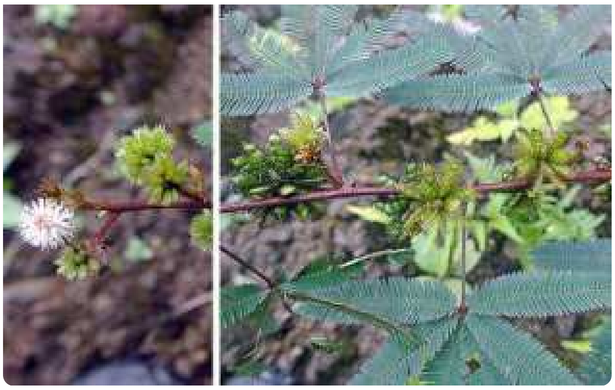

"*Mimosa polydactyla*" by Dick Culbert (CC BY 2.0)

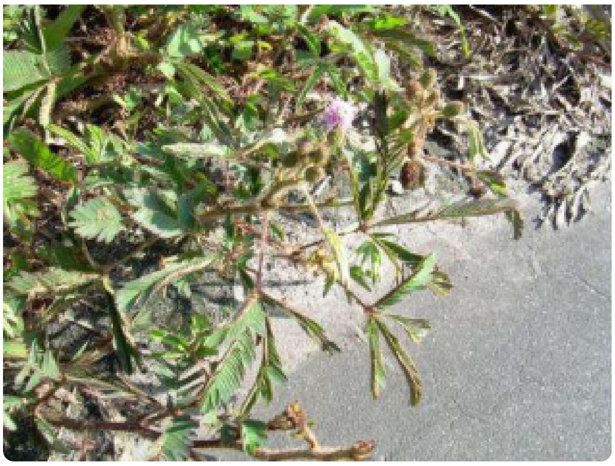

"Fabaceae - *Mimosa polydactyla*" by Colaspidae (CC BY-NC-SA 2.0)

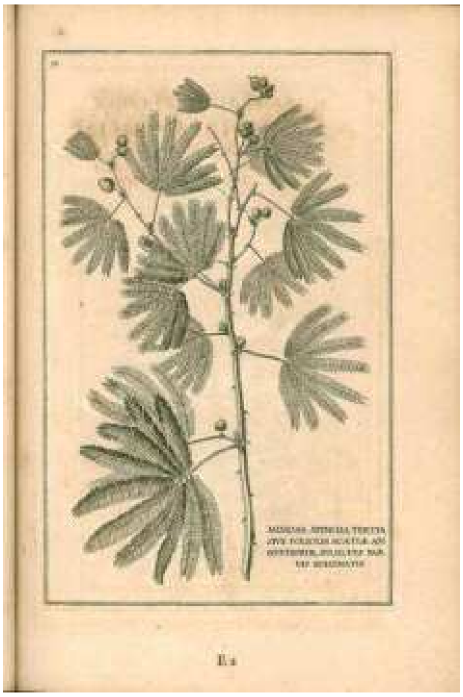

Engraving of *M. polydactila* in *Exoticarum aliarumque minus cognitarum plantarum centuria prima* by Jakob Breyne (1678: t. 18)

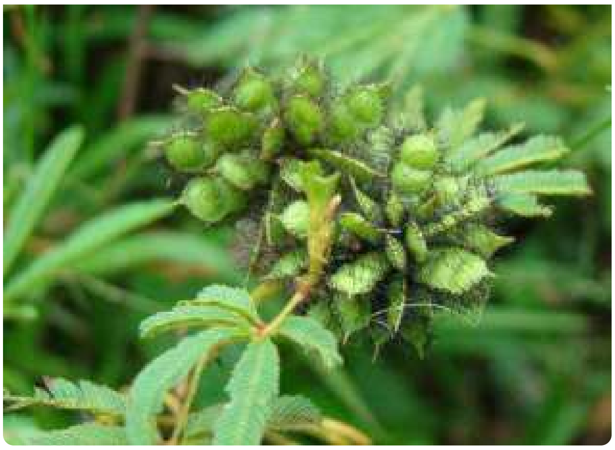

M. polydactyla collected in Perú for iNaturalist by Huamán - 441 - Peru (CC BY-NC-SA 3.0)

Historiae Rerum Naturalium Brasiliae Marcgrave, 1648 Page number 75a

Vernacular name(s) Reperitur hic et alia Herba viva. Silva d' Agoa

Species Mimosa pigra L.

Family Fabaceae

Notes

The woodcut represents a fruit of *M. pigra*, which does not bear a resemblance with the other sources. De Laet ordered to make the woodcut at "natural size" after a pod he received from Brazil via some friends, although he said that the painter could not represent that well its elegance (commentaries by De Laet, in Marcgrave 1648: 74). An ink drawing of this pod is included in De Laet's manuscript, which was likely used to make the woodblock.

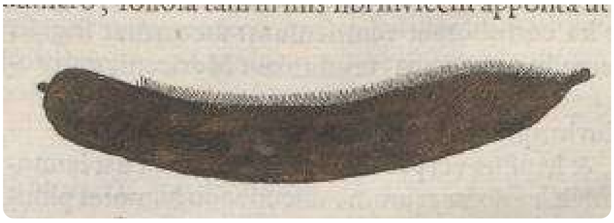

Historiae Plantarum – Plantis Frutescentibus & Fruticibus: 75a

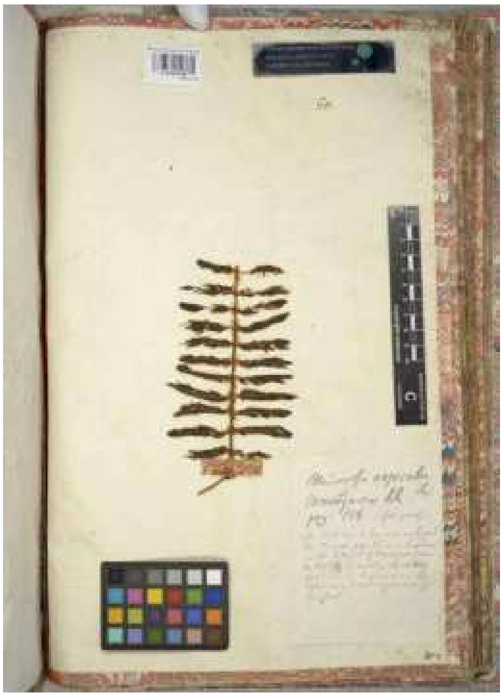

Marcgrave's herbarium: 158

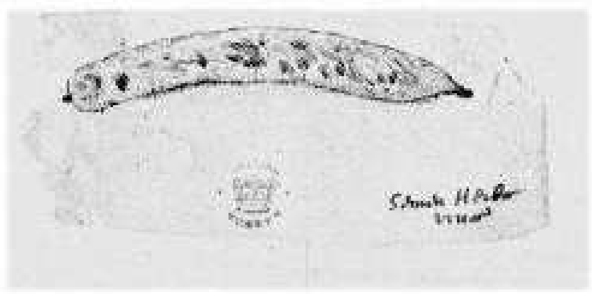

Ink drawing of *M. pigra* in De Laet's manuscript: Sloane MS 1554, f. 9v

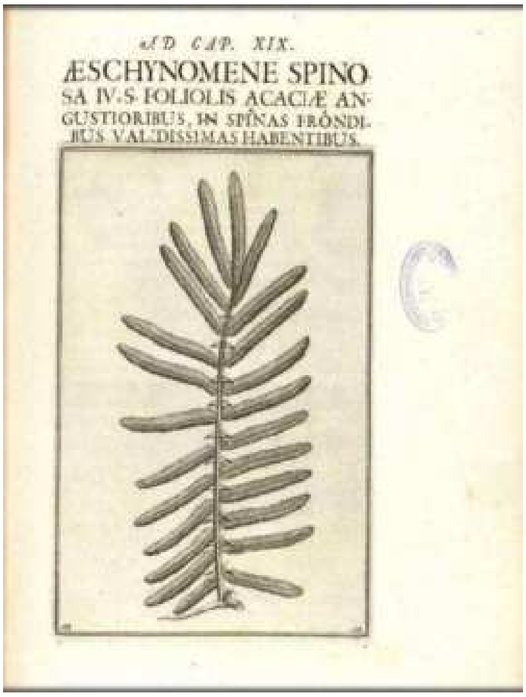

Engraving of *M. pigra* in *Exoticarum aliarumque minus cognitarum plantarum centuria prima* by Jakob Breyne (1678: 44)

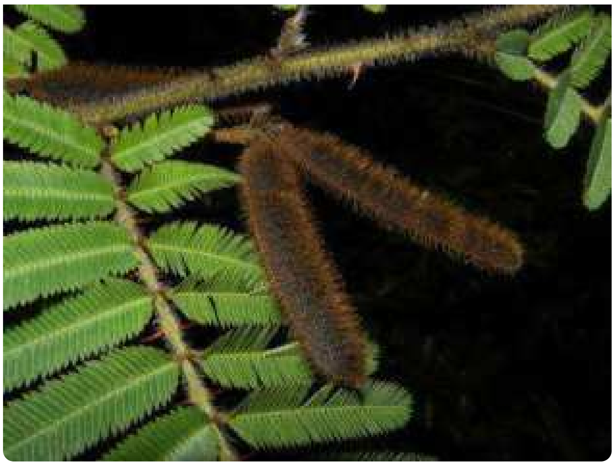

Pods. "*Mimosa pigra* L" by Reinaldo Aguilar (CC BY-NC-SA 2.0)

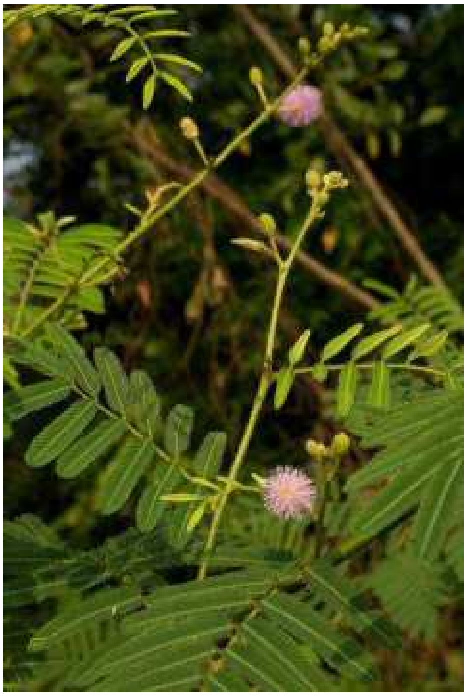

Flowers. "*Mimosa pigra* 091026-0860" by Tony Rodd (CC BY-NC-SA 2.0)

Historiae Rerum Naturalium Brasiliae	Marcgrave, 1648	Page number	75b

Vernacular name(s) Piper caudatum. Betre. Nhamdu

Species Piper marginatum Jacq.

Family Piperaceae

Notes

Woodcut and specimen represent a fertile plant, but there is no strong resemblance between them.

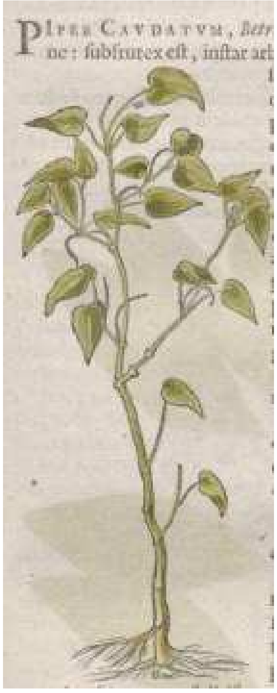

Historiae Plantarum – Plantis Frutescentibus & Fruticibus: 75b

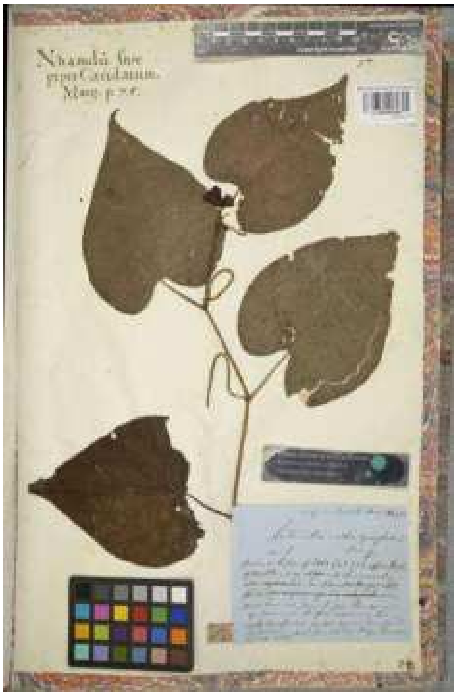

Marcgrave's herbarium: 34

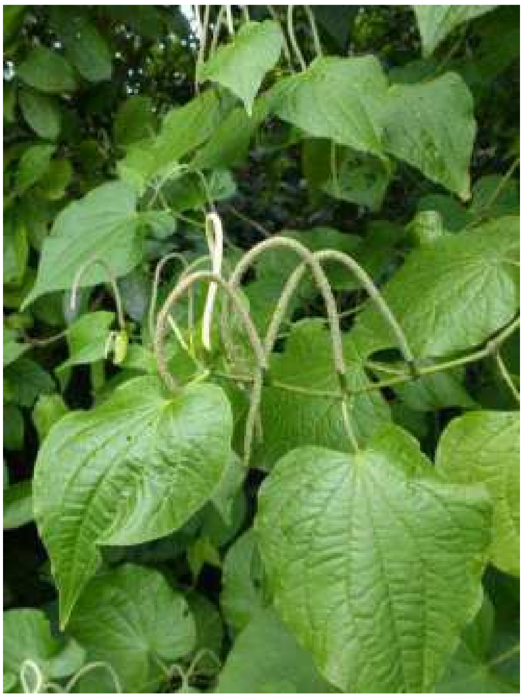

"*P. marginatum*" by Helio Lourencini (CC BY-NC-SA 2.0)

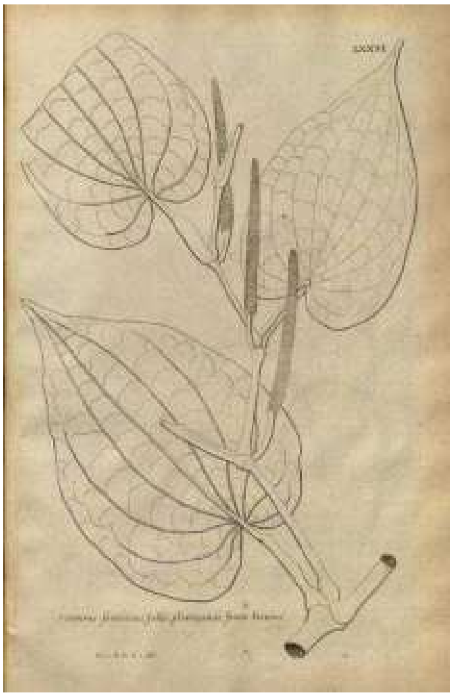

P. marginatum in Description des plantes de l'Amerique by Plumier,C. (1713: 76). Missouri Botanical Garden, St. Louis, U.S.A.

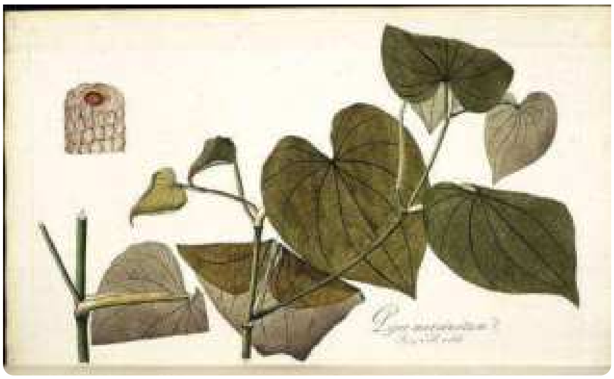

P. marginatum in I*cones plantarum rariorum* by Jacquin, N.J. von (1786: Vol. II, t. 215). Missouri Botanical Garden, St. Louis, U.S.A.

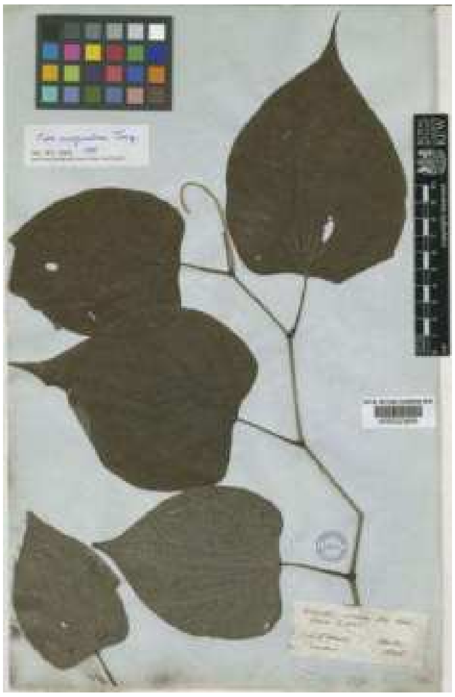

Specimen of *P. marginatum* from Kew's Herbarium - K000323655. Retrieved from Plants of the World Online

Historiae Rerum Naturalium Brasiliae Marcgrave, 1648 Page number 76a

Vernacular name(s) Frutex

Species Dodonaea viscosa Jacq.

Family Sapindaceae

Notes

According to De Laet, he ordered to make the woodcut after a specimen collected by Marcgrave. This voucher is not present in the herbarium at Copenhagen, although it is included in De Laet's manuscript with four dots (such as other plants he ordered to make after the herbarium). J. P. Breyne (1739: 39) mentioned he saw the dry specimen in his father's [Jakob Breyne] herbarium, which was likely collected by Marcgrave (Andrade-Lima et al. 1977). Breyne (1739: 39-footnote) pointed out the book about the natural history of Jamaica by Hans Sloane, who used an engraving of this species.

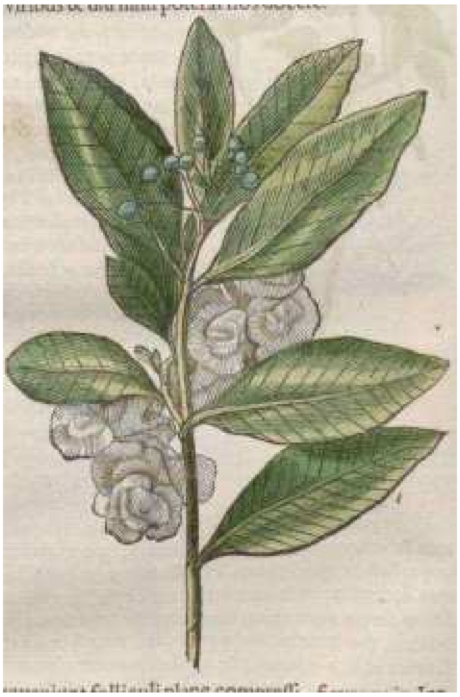

Historiae Plantarum – Plantis Frutescentibus & Fruticibus: 76a

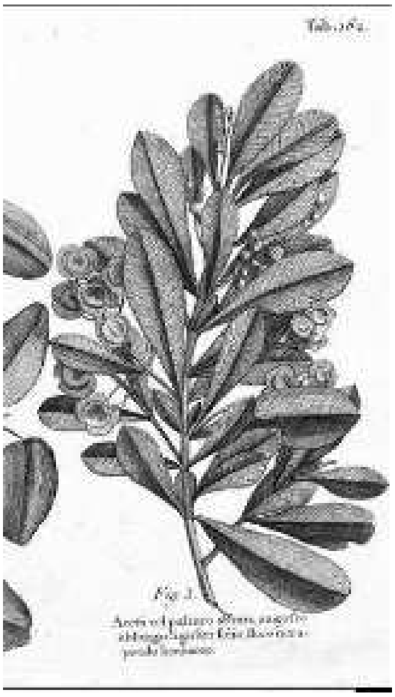

Engraving of *D. viscosa* in *Historia Natural de Jamaica* by Sloane, H. (1707: Vol. II, p. 27, t. 162)

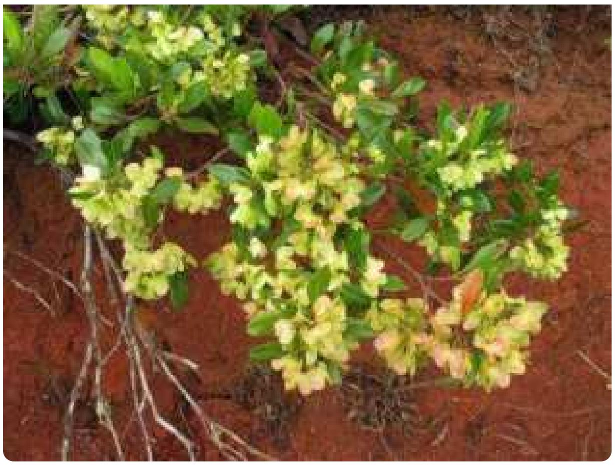

"*D. viscosa*" by D.Eickhoff (CC BY 2.0)

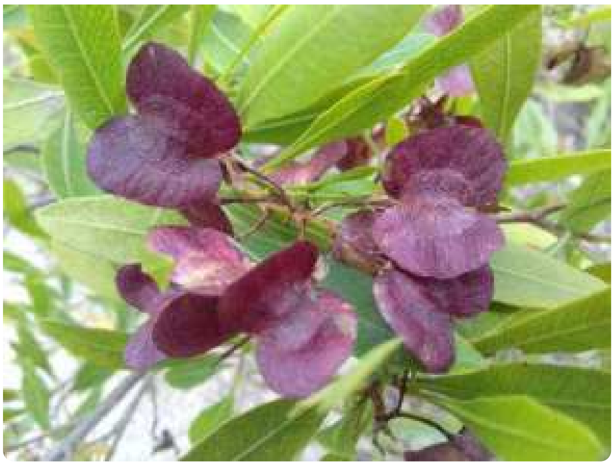

"00027 *D. viscosa*, chamana" by Unidad Educativa Técnica Mitad del Mundo (UETMM) (CC BY-NC-SA 2.0)

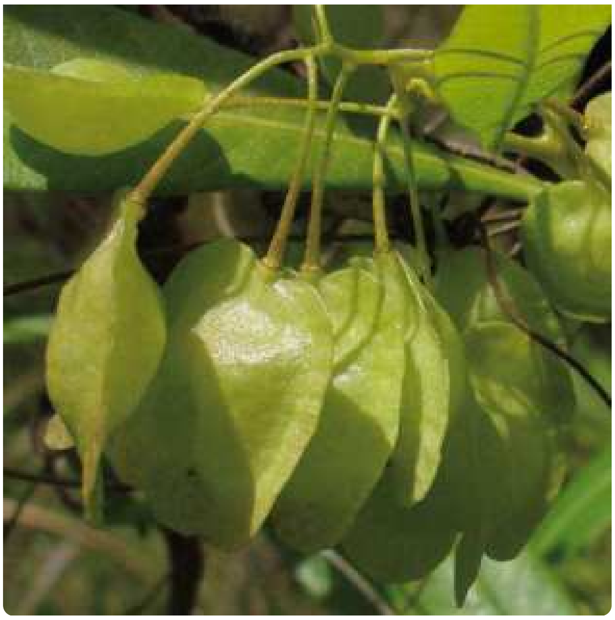

"*D. viscosa*" by Lauren Gutierrez (CC BY-ND 2.0)

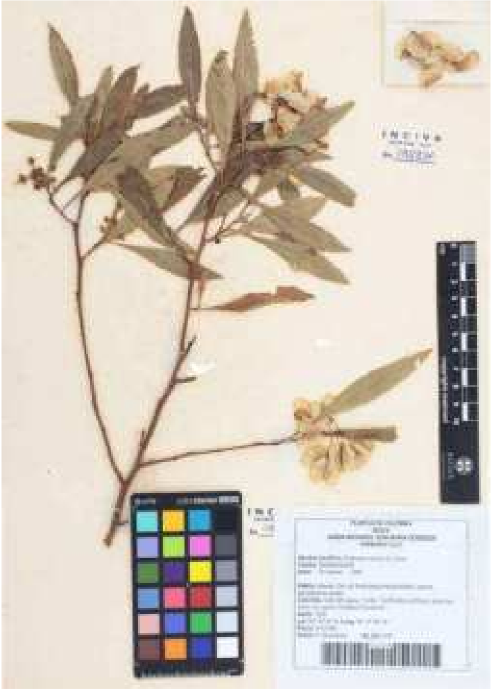

"TULV2236 *D. viscosa*" by Herbario virtual TULV (CC BY-NC 2.0)

Historiae Rerum Naturalium Brasiliae	Marcgrave, 1648	Page number	76b

Vernacular name(s) Frutex

Species Conocarpus erectus L.

Family Combretaceae

Notes

Strong correspondence between woodcut and specimen (p. 28). According to De Laet, he ordered to make the woodcut after a specimen collected by Marcgrave and he also finished the description based on the specimen (Marcgrave 1648: 77).

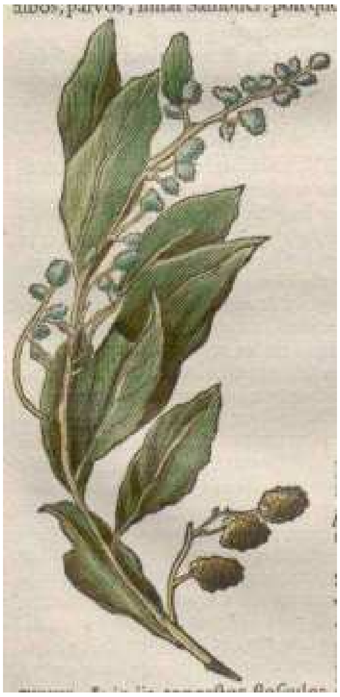

Historiae Plantarum – Plantis Frutescentibus & Fruticibus: 76b

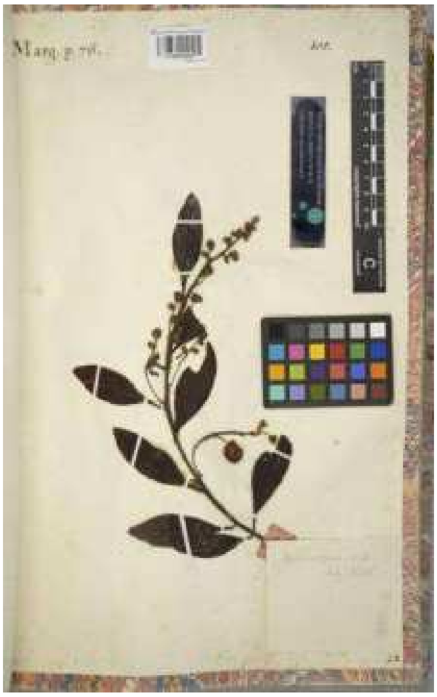

Marcgrave's herbarium: 28

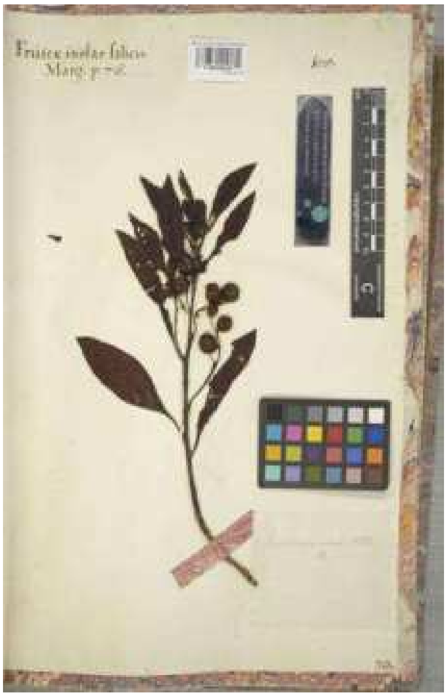

Marcgrave's herbarium: 30

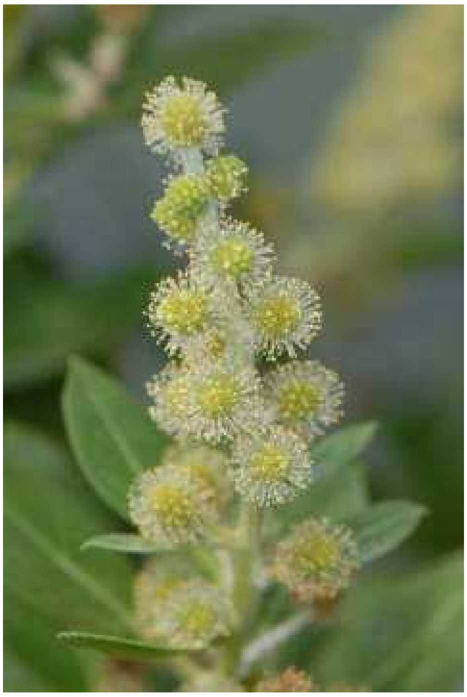

Flowers. "*C. erectus* - button mangrove" by Sam Fraser-Smith (CC BY 2.0)

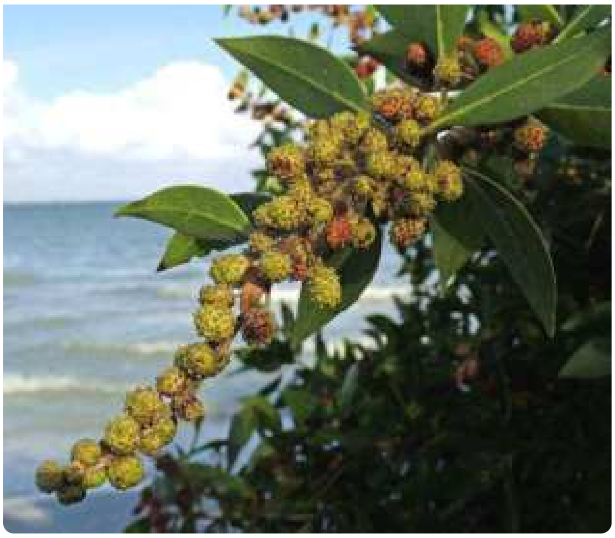

Fruits. "*C. erectus* (buttonwood) (Sanibel Island, Florida, USA) 4" by James St. John (CC BY 2.0)

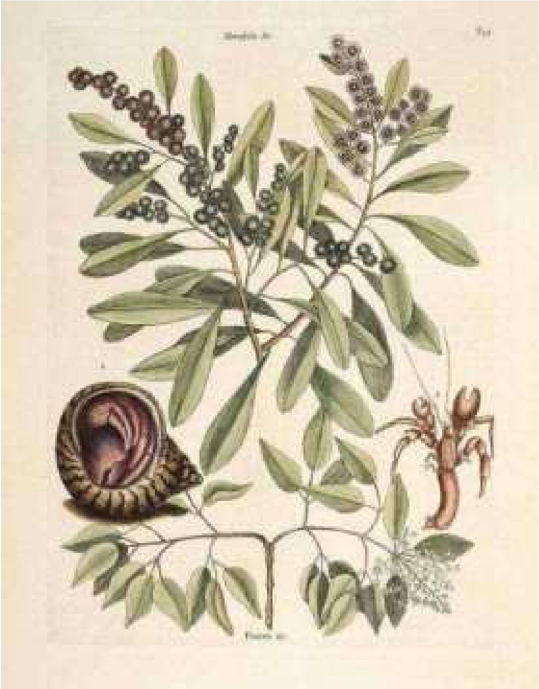

C. erectus in *Natural history of Carolina, Florida, and the Bahama Islands* by Catesby, M. (1754: Vol. II, t. 33). Missouri Botanical Garden, St. Louis, U.S.A.

Historiae Rerum Naturalium Brasiliae Marcgrave, 1648 Page number 77

Vernacular name(s) Guaieru

Species Chrysobalanus icaco L.

Family Chrysobalanaceae

Notes

The woodcut, which shows a flowering branch, differs from the illustration in the *Theatrum*, which shows a fruiting branch. There is a strong correspondence between the woodcut and the specimen (p. 10) collected by Marcgrave. According to De Laet, he ordered to make the woodcut after this specimen because the author had not made any drawing (Marcgrave 1648: 77). This duplicate shows traces of glue where a leaf was present, as observed in the woodcut before it fell off.

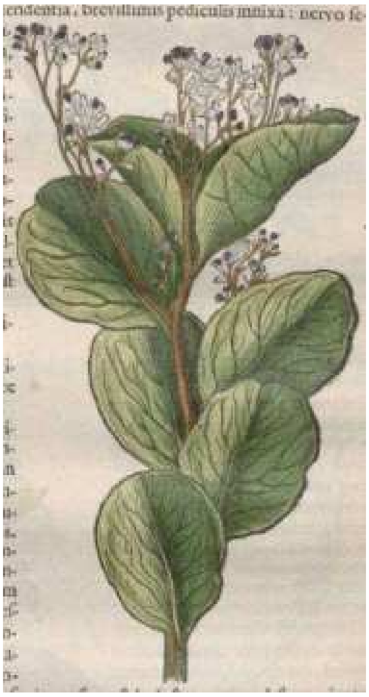

Historiae Plantarum – Plantis Frutescentibus & Fruticibus: 77

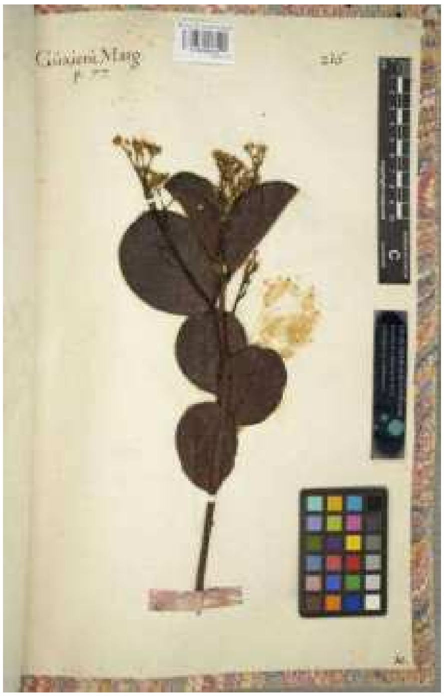

Marcgrave's herbarium: 10

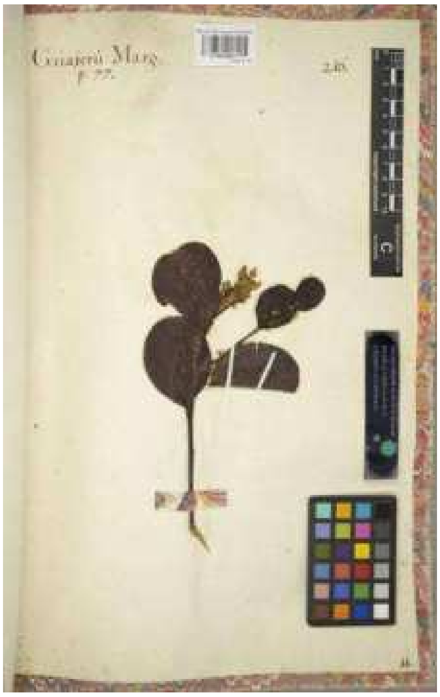

Marcgrave's herbarium: 11

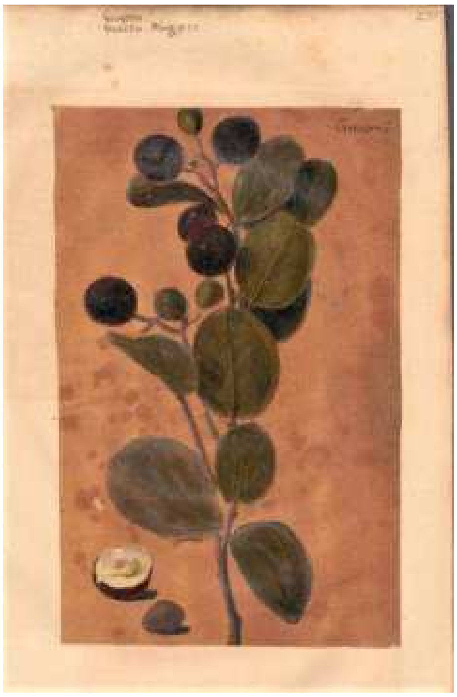

Theatrum Rerum Naturalium: 275

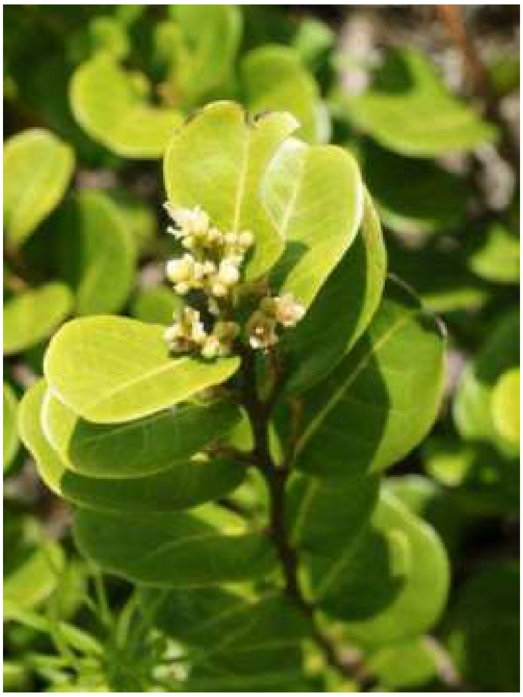

Flowers. "*C. icaco*" by Hans Hillewaert (CC BY-NC-ND 2.0)

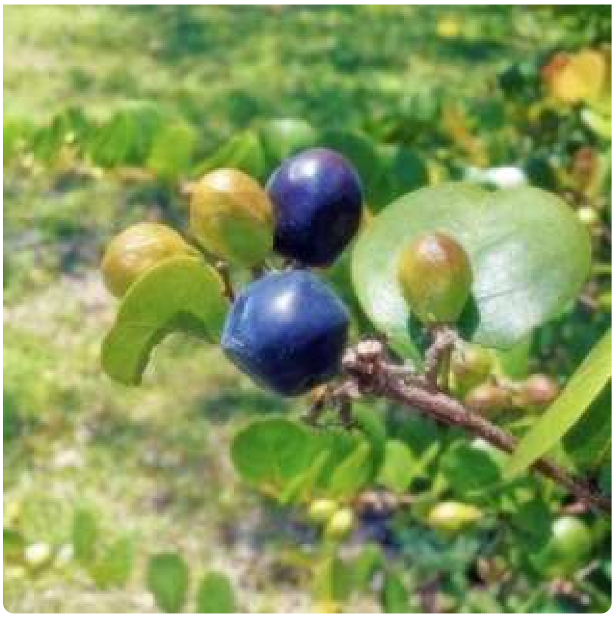

Fruits. "Oleta River State Park - *C. icaco* - Cocoplum fruit 01.jpg" by Daniel Di Palma (CC BY-SA 4.0)

Historiae Rerum Naturalium Brasiliae | Marcgrave, 1648 | Page number 78a

Vernacular name(s): Frutex

Species: Posoqueria latifolia (Rudge) Schult.

Family: Rubiaceae

Notes

We did not find any correspondence between this woodcut and the contemporary or older sources.

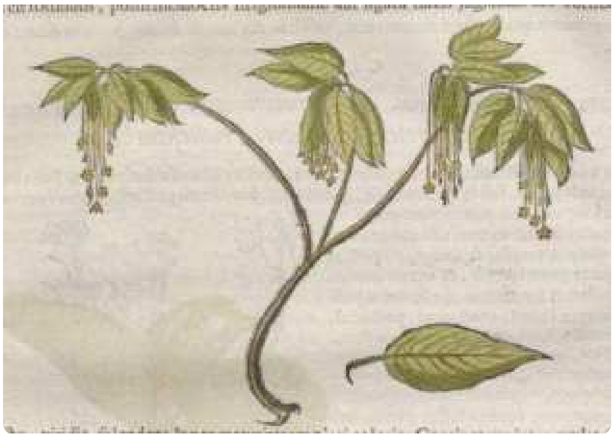

Historiae Plantarum – Plantis Frutescentibus & Fruticibus: 78a

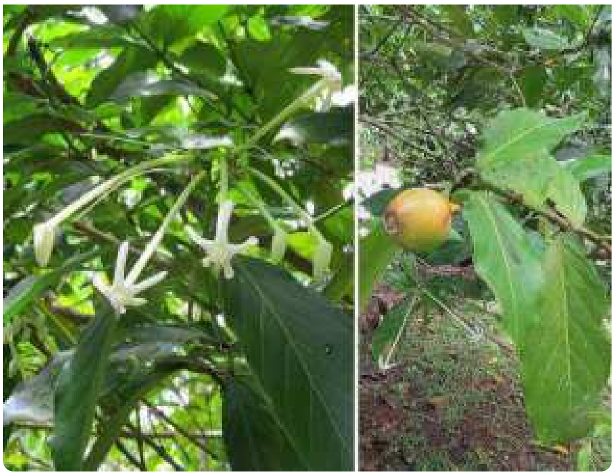

"Posoqueria latifolia" by Dick Culbert is licensed under CC BY 2.0)

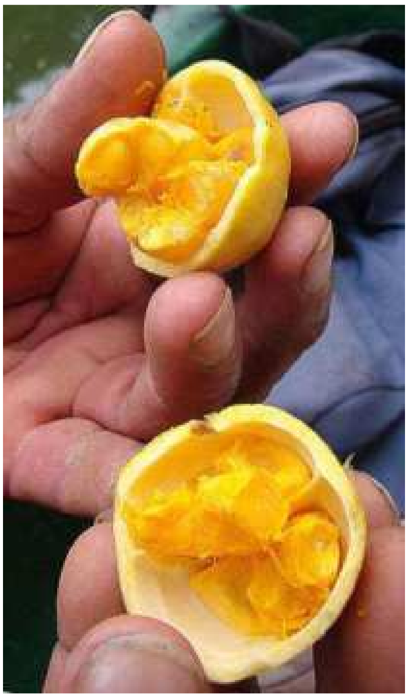

"*P. latifolia*, the Monkey Apple." by Dick Culbert (CC BY 2.0)

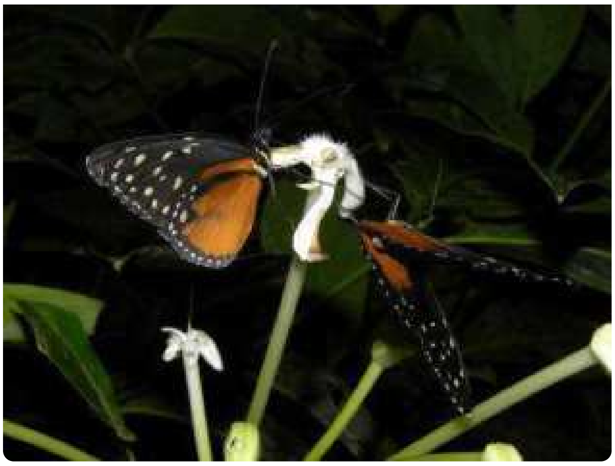

"*Tithorea tarricina* pinthias Godman & Salvin, 1878, visitando *P. latifolia*" by Reinaldo Aguilar (CC BY-NC-SA 2.0)

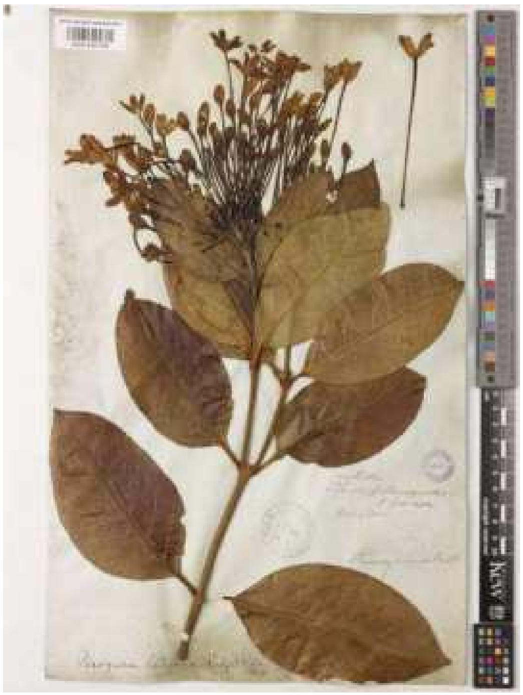

Specimen of *P. latifolia* from Kew's Herbarium - K001445159. Retrieved from Plants of the World Online

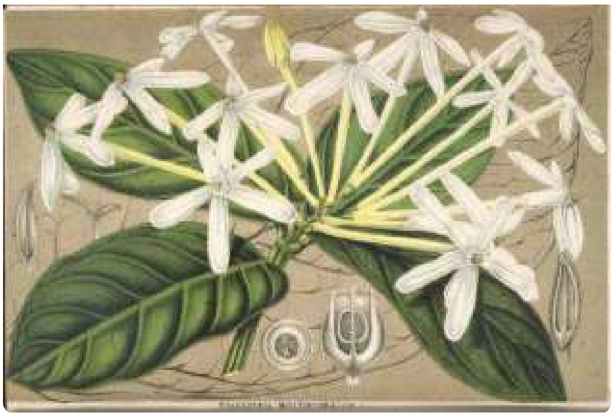

P. latifolia in *L' Illustration horticole* (1869: Vol. 16, t. 597). Missouri Botanical Garden, St. Louis, U.S.A.

Historiae Rerum Naturalium Brasiliae | Marcgrave, 1648 | Page number 78b

Vernacular name(s) Frutex

Species Myriopus candidulus (Miers) Feuillet

Family Boraginaceae

Notes

There is a certain resemblance between the woodcut and the specimen. According to De Laet, he ordered to make the woodcut after a dry branch by Marcgrave, and in that way, he represented the black color of the leaves (Marcgrave 1648: 78). According to Andrade-Lima et al. (1977: 130), the woodcut was likely made after a duplicate of this specimen.

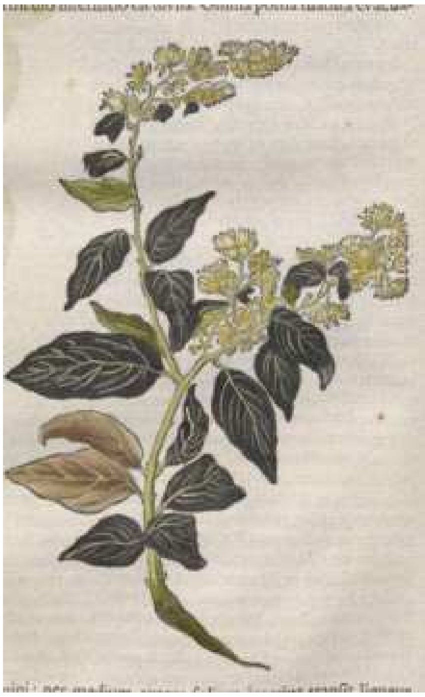

Historiae Plantarum – Plantis Frutescentibus & Fruticibus: 78b

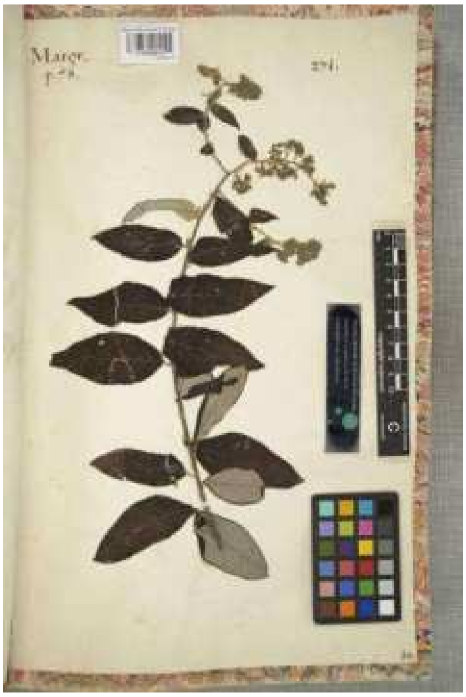

Marcgrave's herbarium: 14

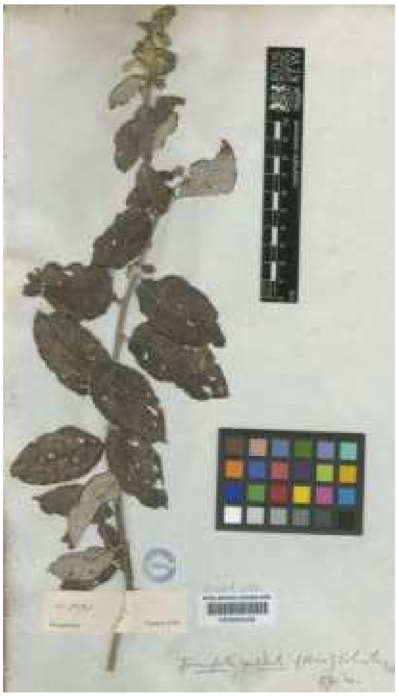

Specimen of *M. candidulus* from Kew's Herbarium - K000583448. Retrieved from Plants of the World Online

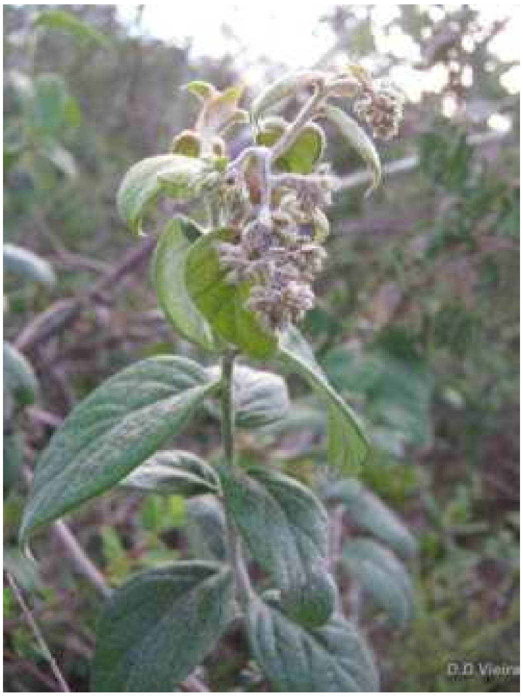

M. candidulus. Published online José Iranildo Miranda de Melo in Flora e Funga do Brasil. Author: D.D.Vieira

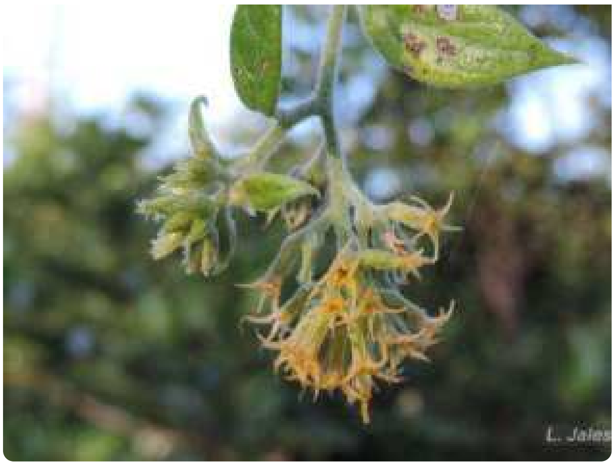

M. candidulus. Published online José Iranildo Miranda de Melo in Flora e Funga do Brasil. Author: L. Jales

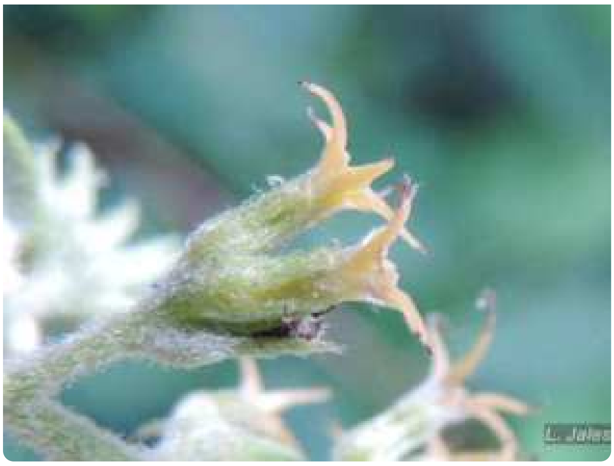

M. candidulus. Published online José Iranildo Miranda de Melo in Flora e Funga do Brasil. Author: L. Jales

Historiae Rerum Naturalium Brasiliae | Marcgrave, 1648 | Page number 79a

Vernacular name(s) Canambaya

Species Epiphyllum phyllanthus (L.) Haw.

Family Cactaceae

Notes

The woodcut is slightly similar to the *Theatrum* image. The pencil drawing in De Laet's manuscript is very similar to the woodcut, but not reversed. This drawing was likely the basis to make the woodblock.

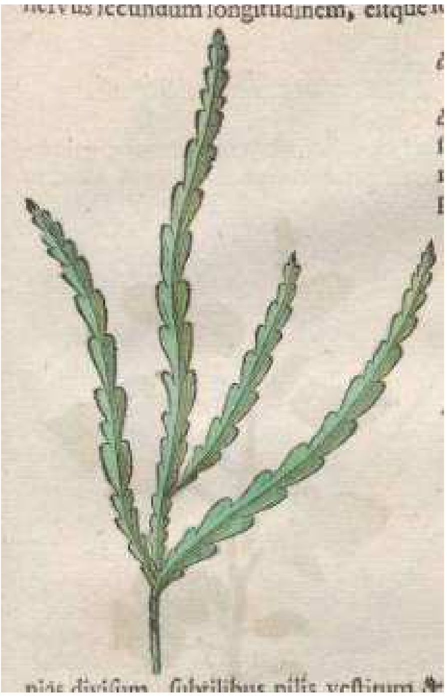

Historiae Plantarum – Plantis Frutescentibus & Fruticibus: 79a

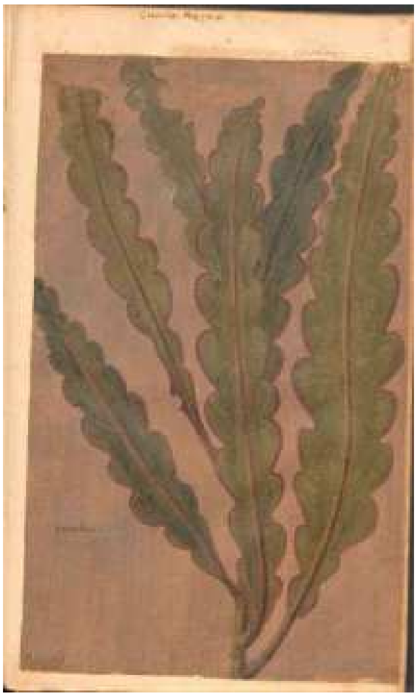

Theatrum Rerum Naturalium: 381

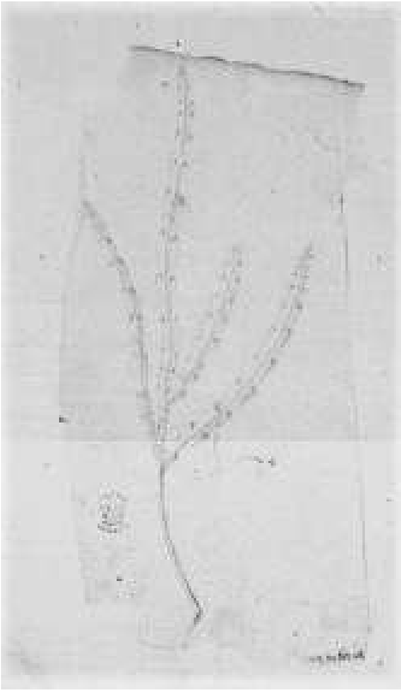

Pencil drawing of *E. phyllanthus* n De Laet's manuscript: Sloane MS 1554, f. 52v

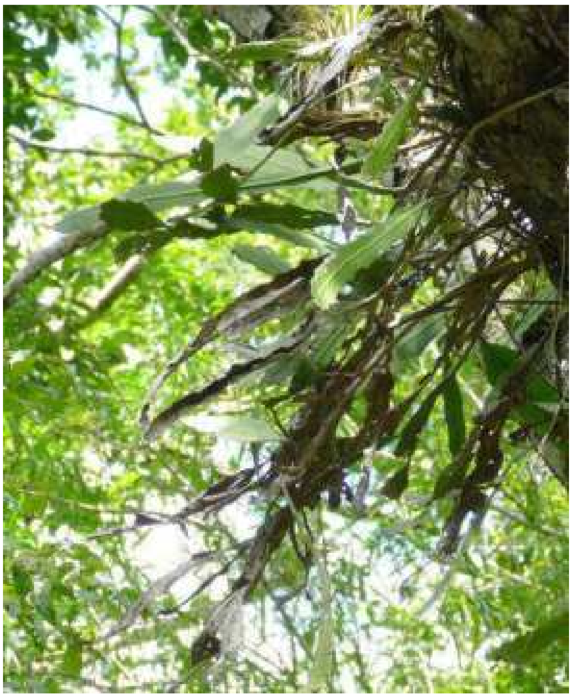

"Climbing Cactus (*Epiphyllum phyllanthus*)" by berniedup (CC BY-SA 2.0)

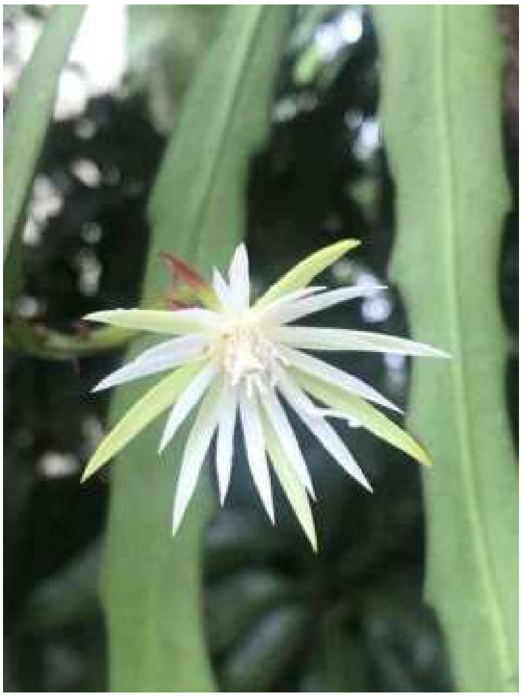

E. phyllanthus observed in Colombia for iNaturalist by Luis German Naranjo (CC BY-NC 4.0)

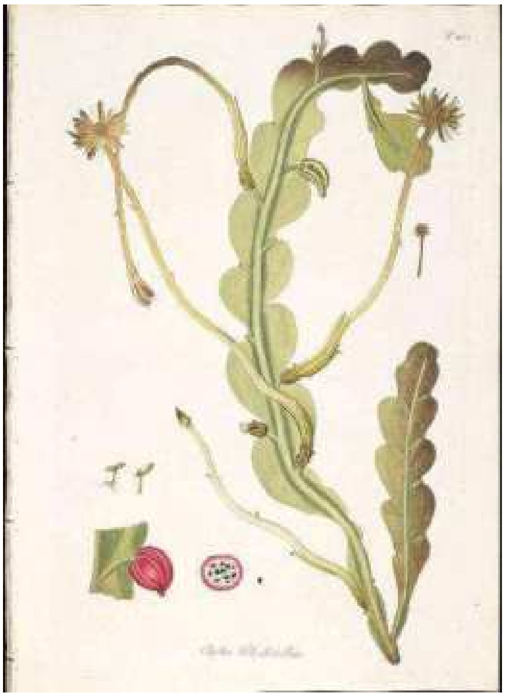

Plantarum rariorum horti caesarei Schoenbrunnensis descriptiones et icones by Jacquin, N.J. von (1804: Vol. IV, t. 403)

Historiae Rerum Naturalium Brasiliae | Marcgrave, 1648 | Page number 79b

Vernacular name(s) Frutex

Species Cnidoscolus urens (L.) Arthur

Family Euphorbiaceae

Notes

The woodcut differs from the *Theatrum* image. Both depict flowering plants, although the oil painting shows the flowers with their calyx more in detail, and the leaves seem wrinkled and droopy in the woodcut as if the plant was picked and not that fresh anymore.

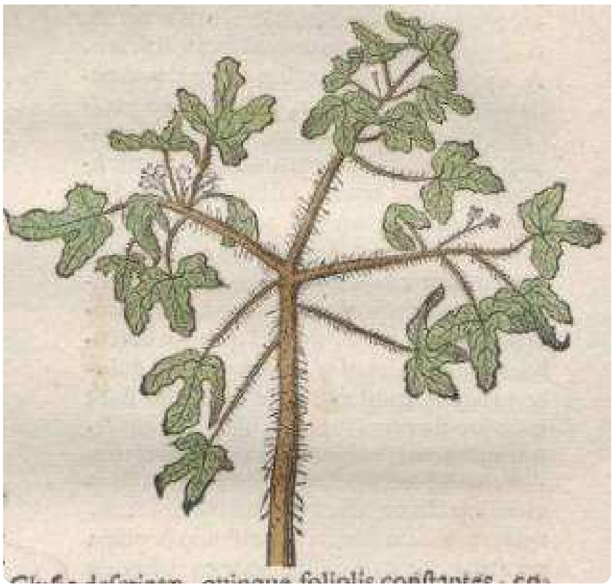

Historiae Plantarum – Plantis Frutescentibus & Fruticibus: 79b

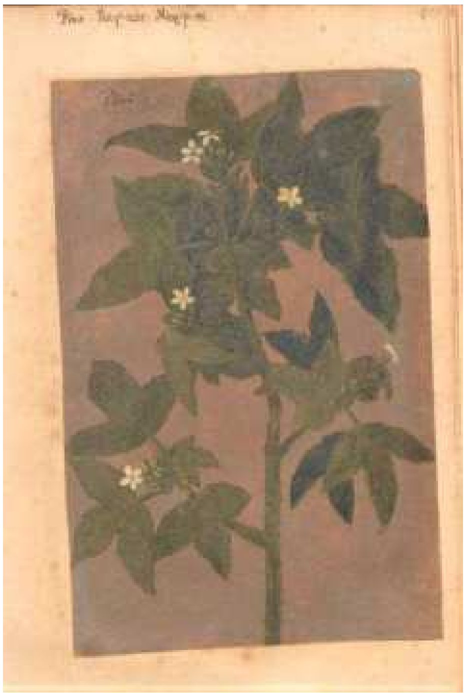

Theatrum Rerum Naturalium: 483

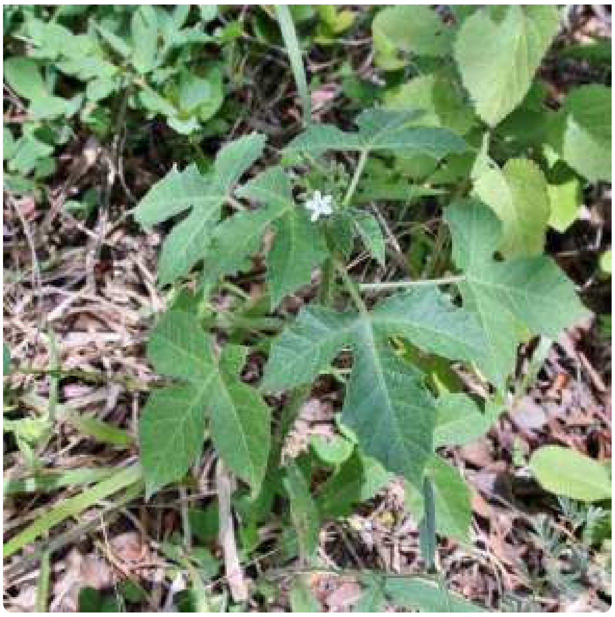

"*C. urens*" by Mauricio Mercadante (CC BY-NC-SA 2.0)

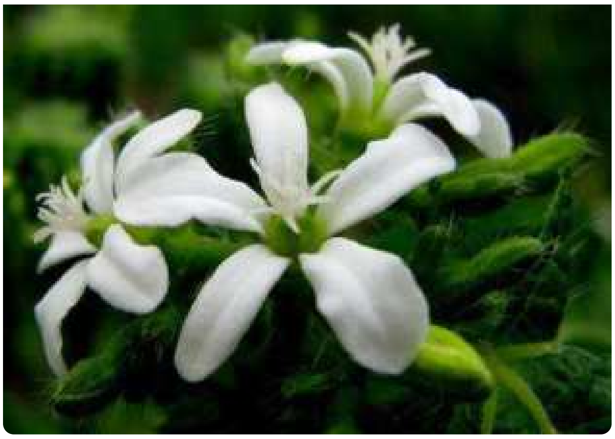

Flowers. "*C. urens*" by Alex Popovkin, Bahia, Brazil (CC BY-NC-SA 2.0)

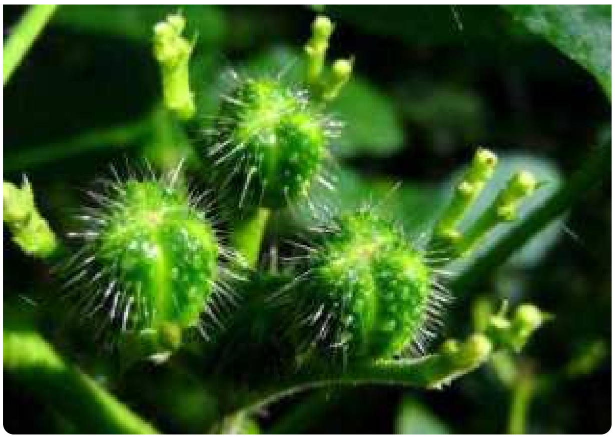

Fruits. "*C. urens*" by Alex Popovkin, Bahia, Brazil (CC BY-NC-SA 2.0)

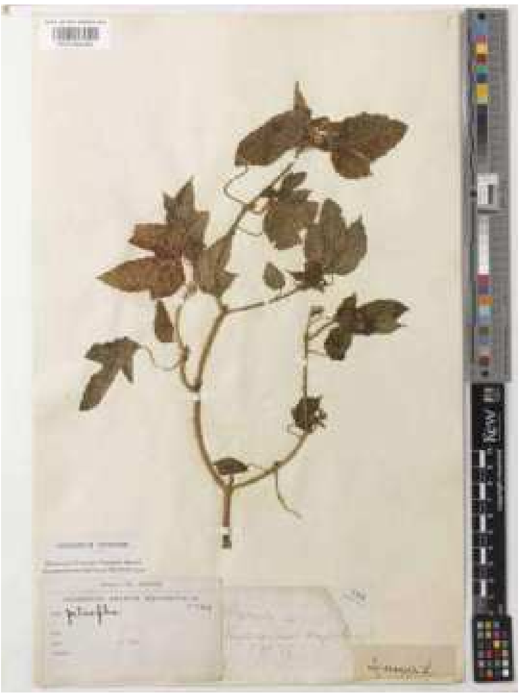

Specimen of *C. urens* from Kew's Herbarium - K001494062. Retrieved from Plants of the World Online

Historiae Rerum Naturalium Brasiliae	Marcgrave, 1648	Page number	79c

Vernacular name(s) Frutiscencens haec herba

Species Galphimia brasiliensis (L.) A.Juss.

Family Malpighiaceae

Notes

Strong correspondence between woodcut and specimen (p. 20). There are four points accompanying the description in De Laet's manuscript (Andrade-Lima et al. 1977). De Laet, however, does not indicate he ordered to make the woodcut after the specimen, although he could have forgotten.

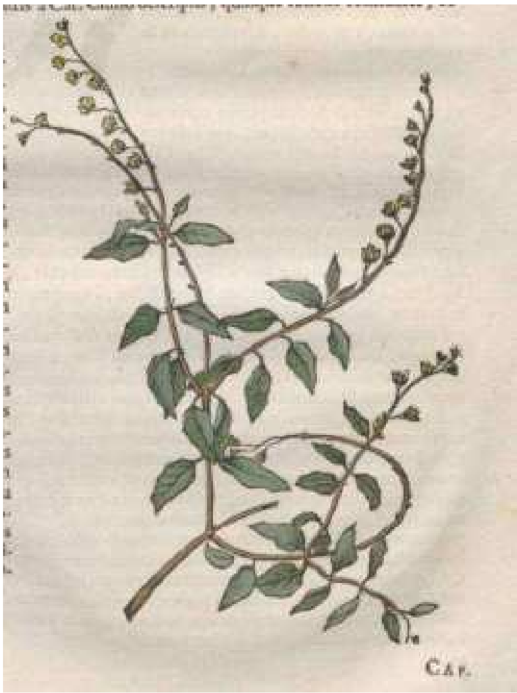

Historiae Plantarum – Plantis Frutescentibus & Fruticibus: 79c

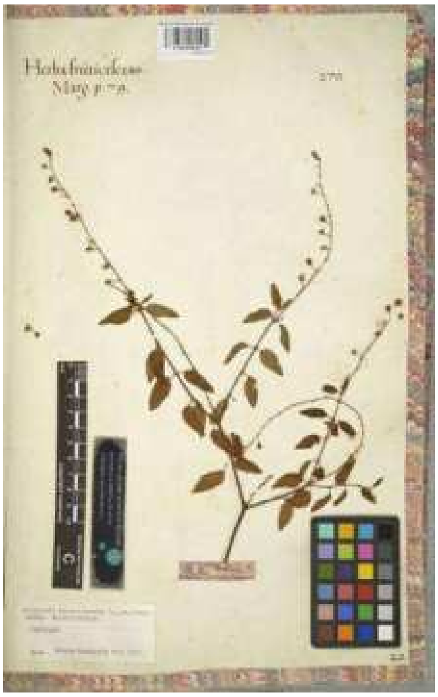

Marcgrave's herbarium: 20

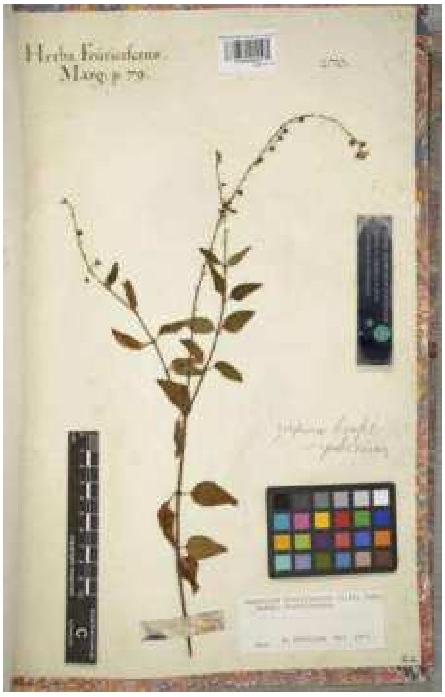

Marcgrave's herbarium: 22

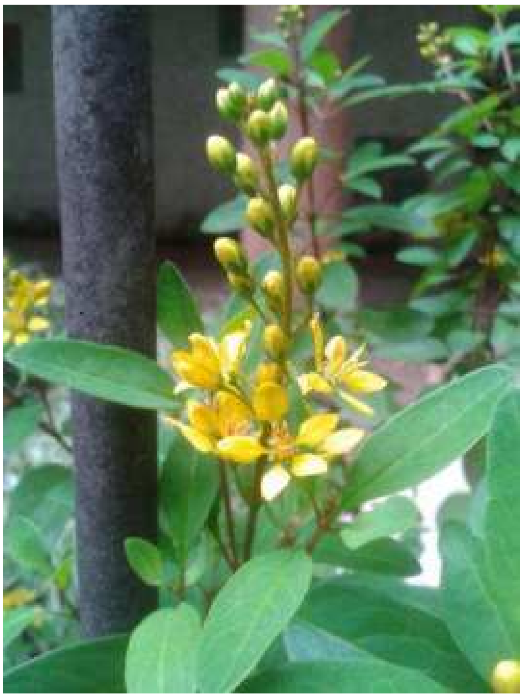

Flowering of *Galphimia gracilis* (syn. *G. brasiliensis*). São Paulo city. State of São Paulo, Brazil, by Mário NET (CC-BY-SA-4.0)

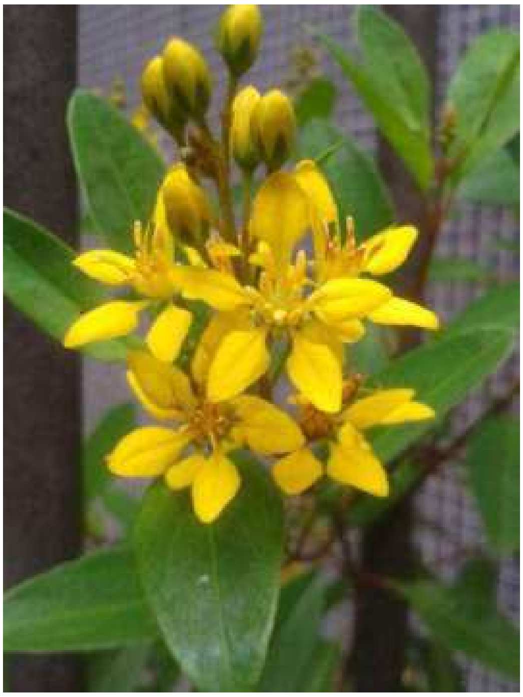

Flowering of *G. brasiliensis*. São Paulo, Brazil, by Mário NET (CC-BY-SA-4.0)

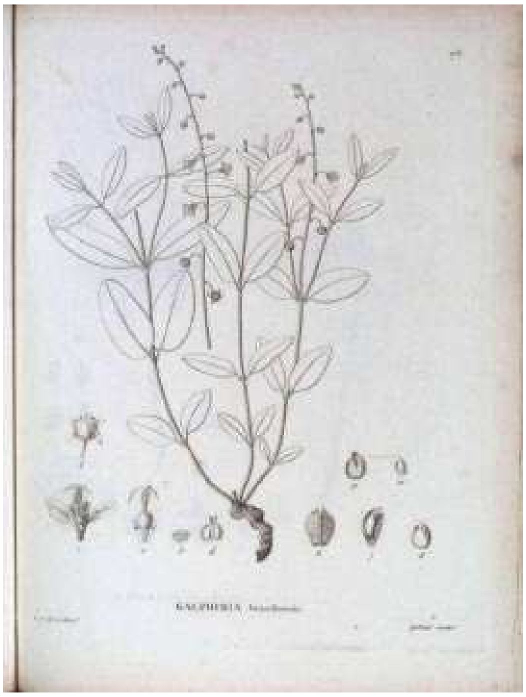

G. brasiliensis.in *Flora Brasiliae meridionalis* by Saint-Hilaire, A.F.C.P. de (1832: Vol. III, t. 178). Missouri Botanical Garden, St. Louis, U.S.A.

Historiae Rerum Naturalium Brasiliae Marcgrave, 1648 Page number 80

Vernacular name(s) Frutex hic

Species Solanum agrarium Sendtn.

Family Solanaceae

Notes

There is a certain resemblance between the woodcut and the specimen (p. 42). According to De Laet (Marcgrave 1648: 80), he ordered to make this image after some dry plants preserved by the author [Marcgrave]. Interestingly, De Laet also stated that he kept an image of this plant made after a plant growing in the botanical garden of Brabant by a painter he sent there for that purpose. The seed was sent from Brazil in 1638 and he could not draw it with the fruit because the cold killed the plant before it ripened (De Laet commentaries, in Marcgrave 1648: 80).

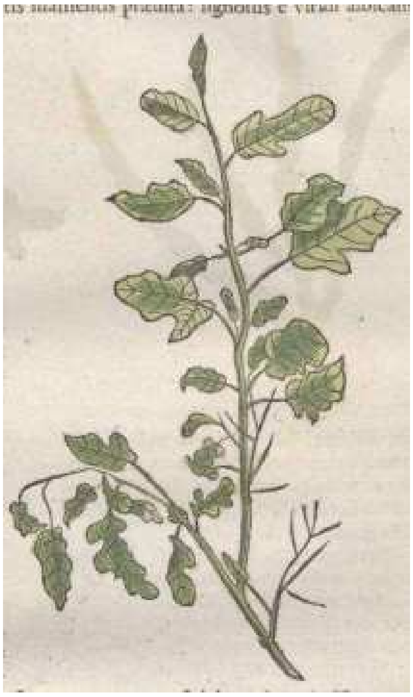

Historiae Plantarum – Plantis Frutescentibus & Fruticibus: 80

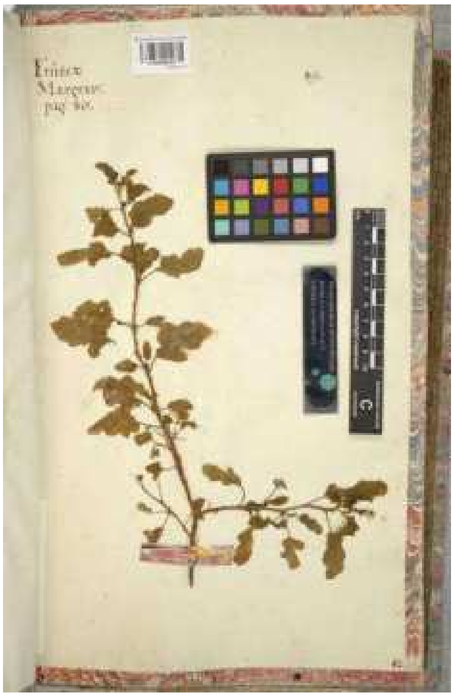

Marcgrave's herbarium: 42

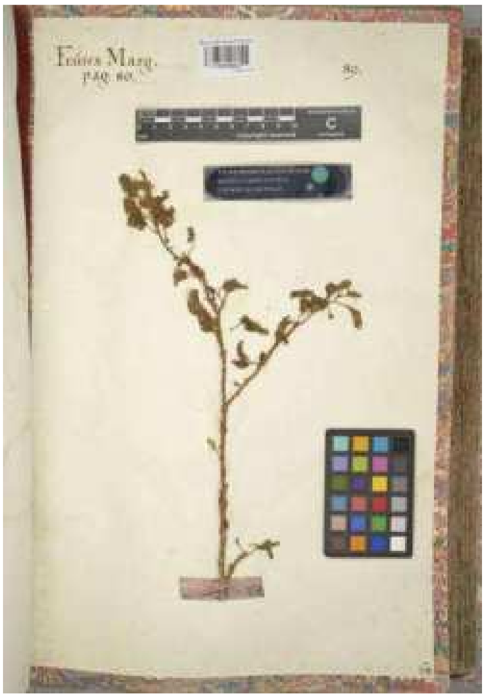

Marcgrave's herbarium: 63

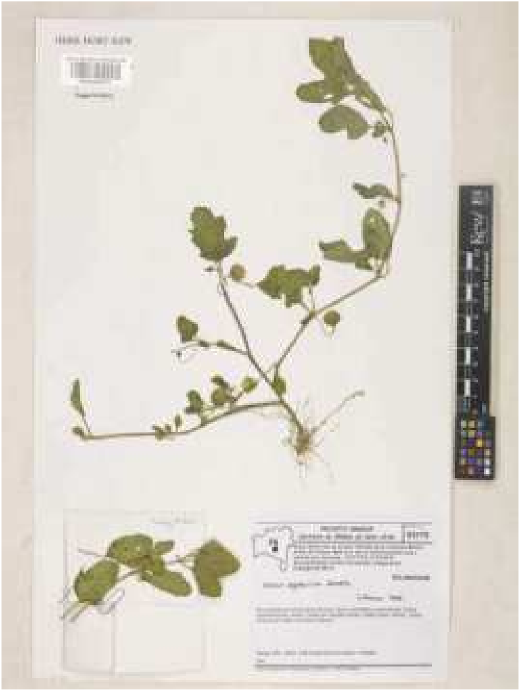

Specimen of *S. agrarium* with fruits from Kew's Herbarium - K000982210. Retrieved from Plants of the World Online

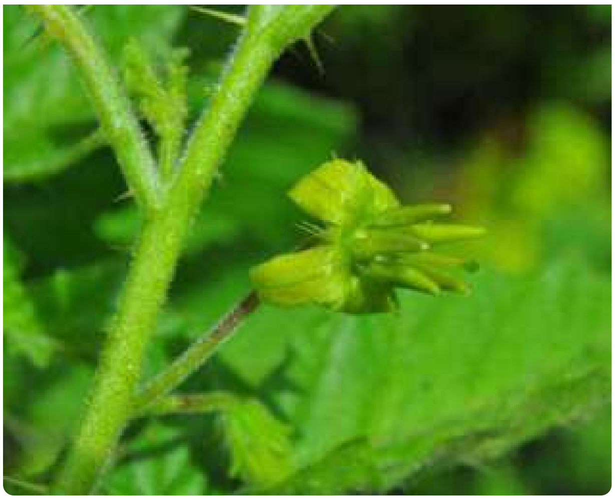

Flower. *S. agrarium* observed in Brazil for iNaturalist by Frederico Acaz Sonntag (CC BY-NC 4.0)

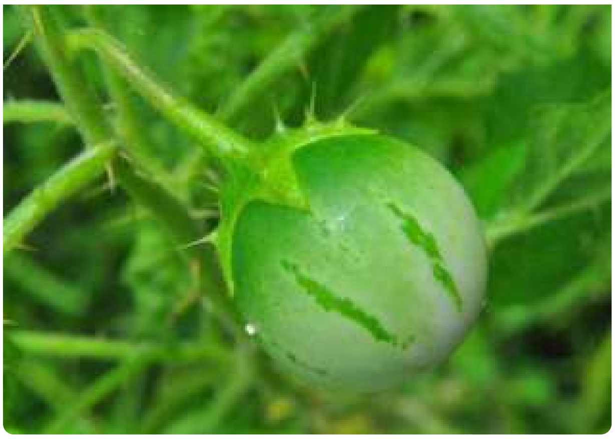

Fruit. *S. agrarium* observed in Brazil for iNaturalist by Frederico Acaz Sonntag (CC BY-NC 4.0)

Historiae Rerum Naturalium Brasiliae — Marcgrave, 1648 — Page number 81a

Vernacular name(s) Tremate

Species Vernonanthura brasiliana (L.) H.Rob.

Family Asteraceae

Notes

Strong correspondence between woodcut and specimen. De Laet indicated "with dry plants, and with a lot of care, we tried to delineate this plant" (Marcgrave 1648: 81).

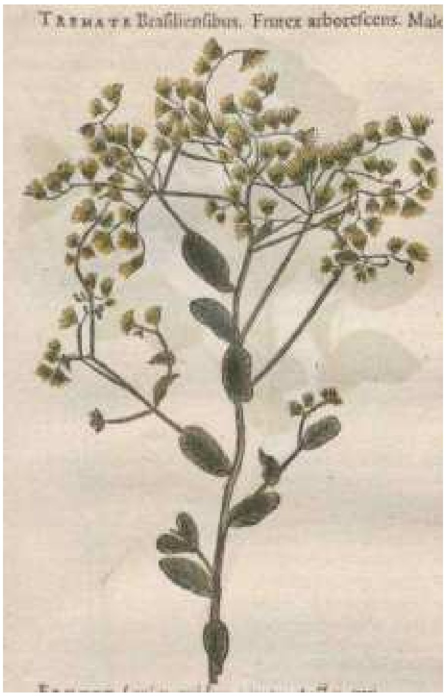

Historiae Plantarum – Plantis Frutescentibus & Fruticibus: 81a

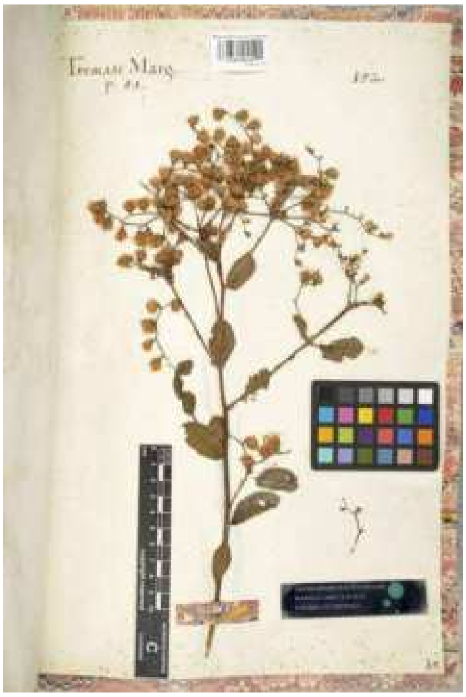

Marcgrave's herbarium: 15

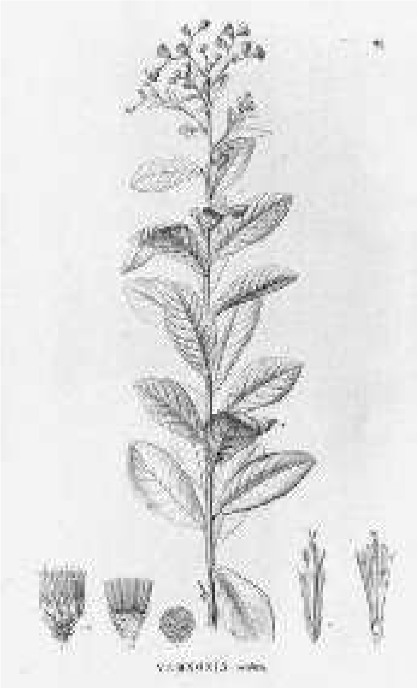

Engraving of *V. brasiliana* in Martius, C.F.P. von, Eichler, A.G., Urban, I., *Flora Brasiliensis* (1873-1874) Vol. 6(2): t. 21, p. 100

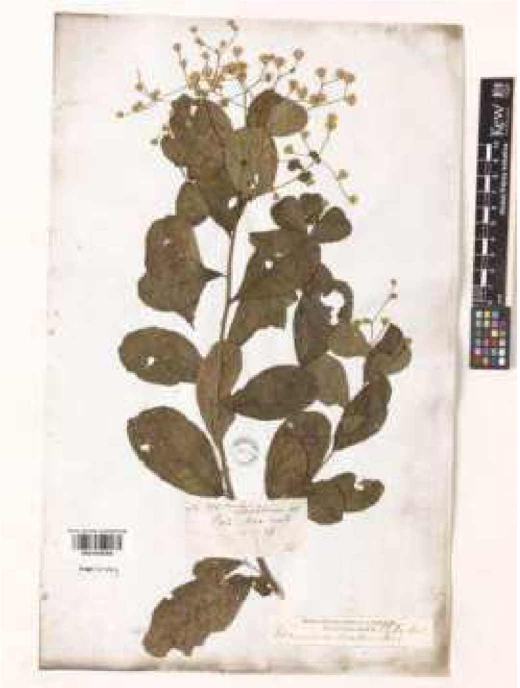

Type specimen of *V. brasiliana* collected by von Martius in Brazil. Kew's Herbarium - K001058990. Retrieved from Plants of the World Online

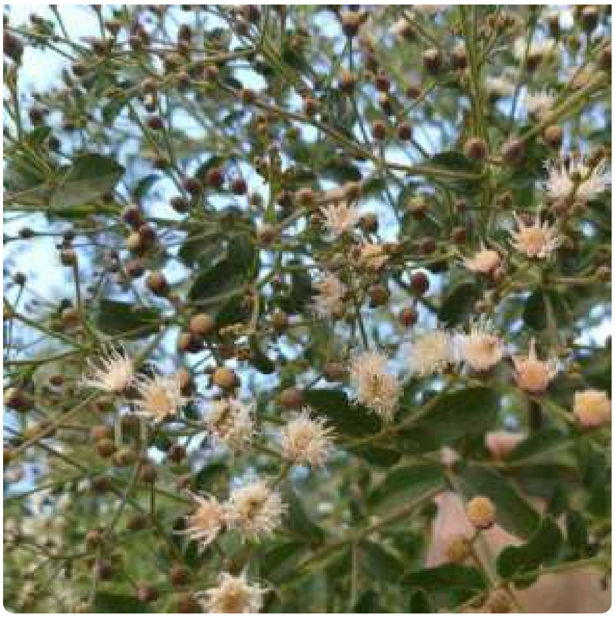

V. brasiliana observed in Brazil for iNaturalist by Fábio Luis dos Santos (CC BY-NC 4.0)

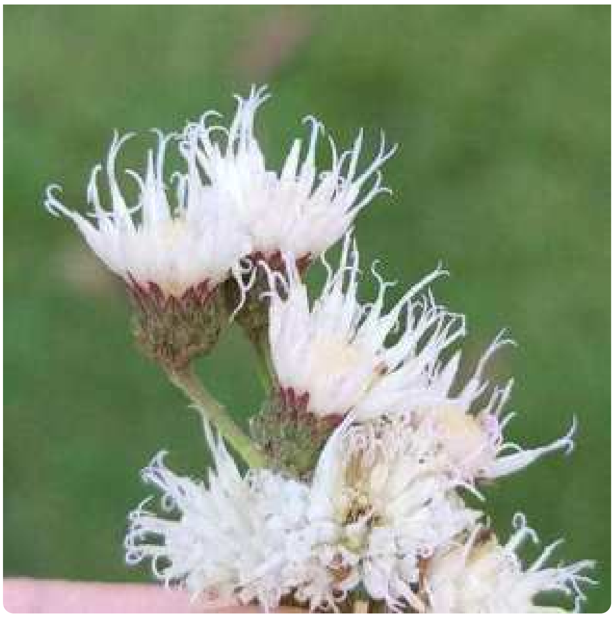

Flowers. *V. brasiliana* observed in Brazil for iNaturalist by Fábio Luis dos Santos (CC BY-NC 4.0)

Historiae Rerum Naturalium Brasiliae Marcgrave, 1648 Page number 81b

Vernacular name(s) Frutex

Species Unknown

Family Unknown

Notes

We did not find any correspondence between this woodcut and the contemporary or older sources. We could not identify the plant represented by the woodcut, hence we could not cross-reference it with the visual sources. Accurate identification of such plant will facilitate this analysis and provide us with more insights about its origin.

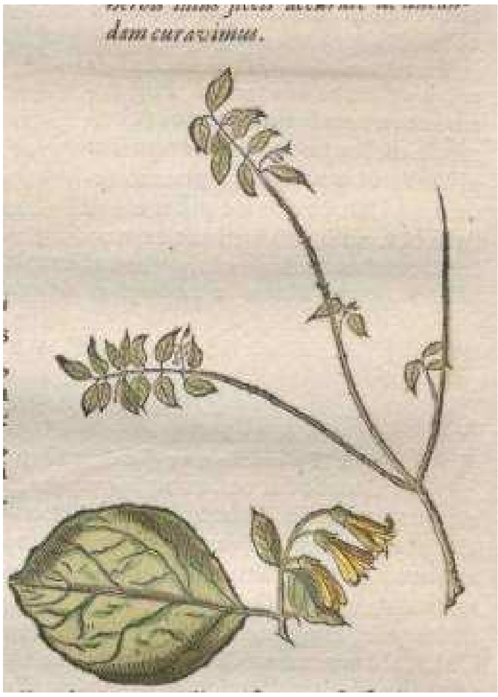

Historiae Plantarum – Plantis Frutescentibus & Fruticibus: 81b

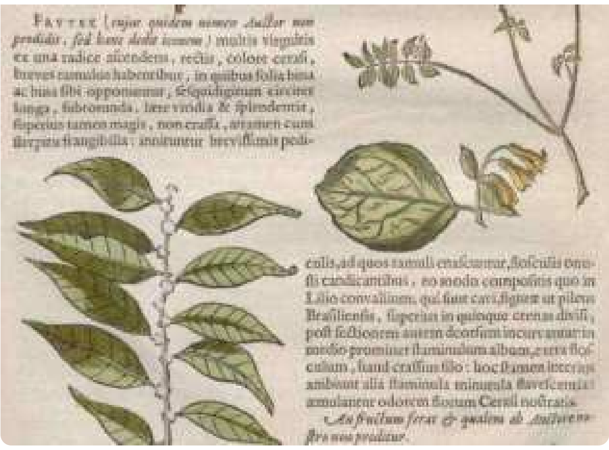

HNB (Marcgrave 1648: 81) Latin edition

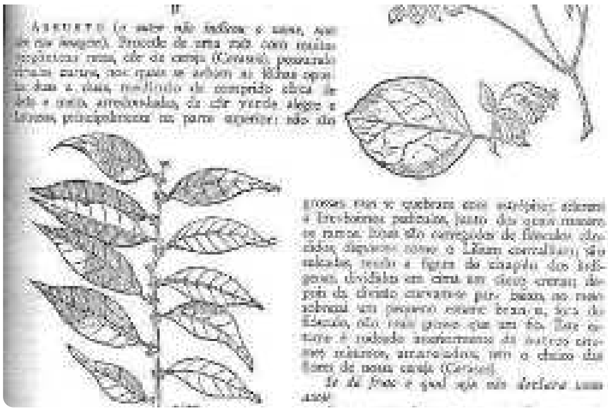

HNB (1942 [1648]) Portuguese edition

"It comes from a root with many straight, cherry-colored (*Cerasus*) spikes having short branches, in which the opposite leaves are two by two, measuring about a finger and a half in length, rounded, of a cheerful and glossy green color, mainly on top; they are not thick, but they break with a clatter; adhere to very short pedicles, from which the branches are born. These are loaded with candid florets, arranged like *Lilium convallium*; they are furrowed, having the figure of the hat of the indigenous people, divided above in five crenes; after dividing, they curve downwards, in the middle a small white stamen protrudes out of the flower, no thicker than a thread. This stamen is surrounded internally by other yellowish minimum stamens; it smells like the flowers of our cherry (*Cerasus*). If it bears fruit and what it is do not declare our author" (HNB 1648: 81)

English translation, by M. Alcantara-Rodriguez

Historiae Rerum Naturalium Brasiliae | Marcgrave, 1648 | Page number 81c

Vernacular name(s) Frutex

Species Casearia sylvestris Sw.

Family Salicaceae

Notes

Strong correspondence between woodcut and specimen. De Laet (Marcgrave 1648: 82) indicated that they made this figure after some dry plants because the author did not leave an image; however, he claimed that the serrated leaves were not well represented in the woodcut.

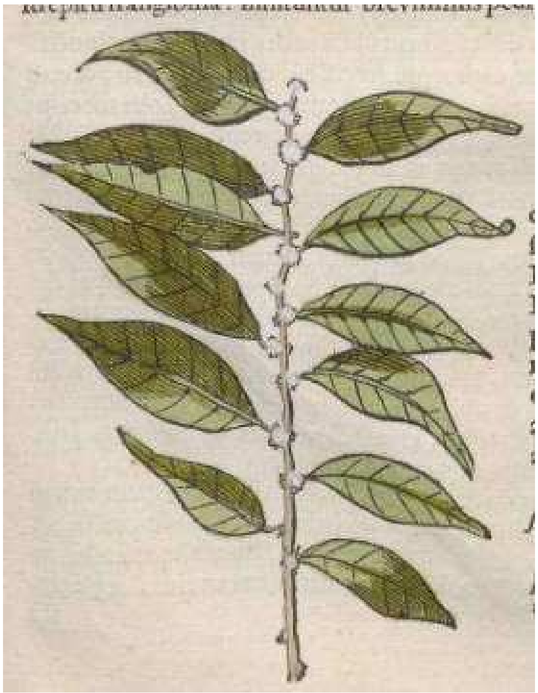

Historiae Plantarum – Plantis Frutescentibus & Fruticibus: 81c

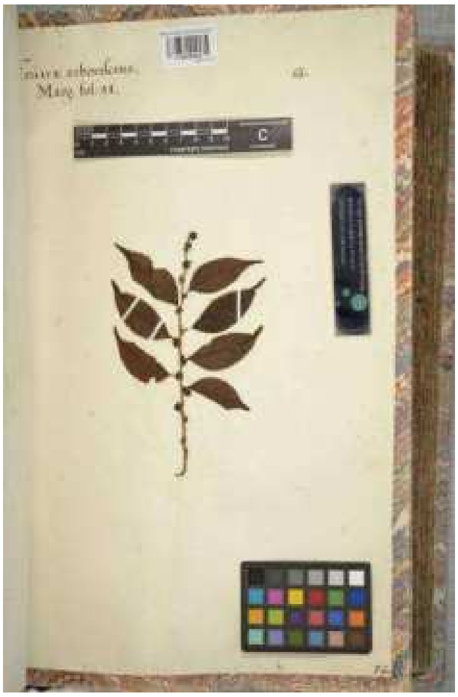

Marcgrave's herbarium: 52

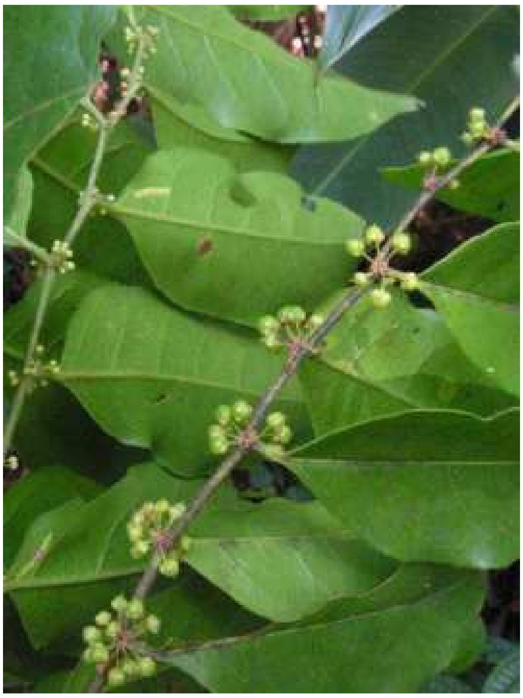

Fruiting branch. "*C. sylvestris*, caiubim" by Tarciso Leão (CC BY 2.0)

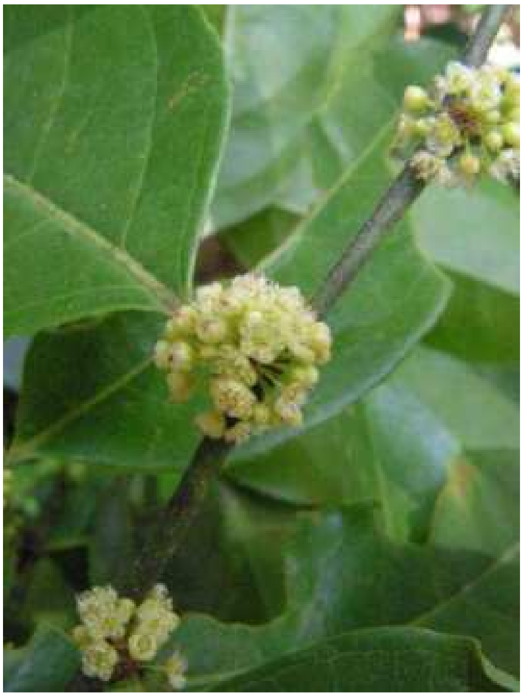

Flowers. "*C. sylvestris*, caiubim" by Tarciso Leão (CC BY 2.0)

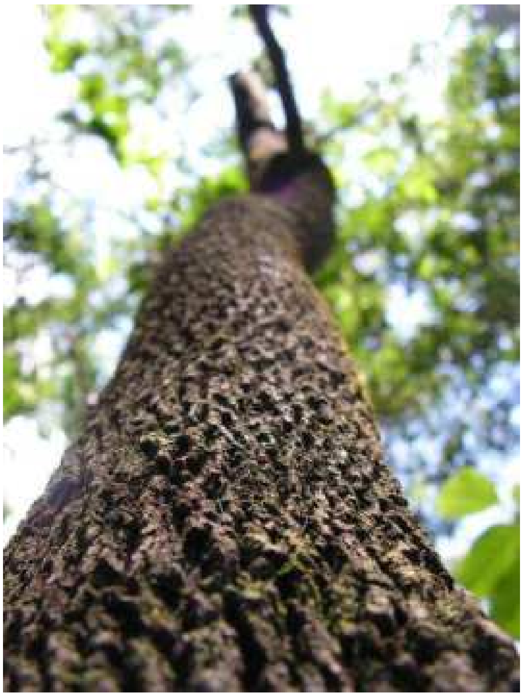

Habit. "*C. sylvestris*, caiubim" by Tarciso Leão (CC BY 2.0)

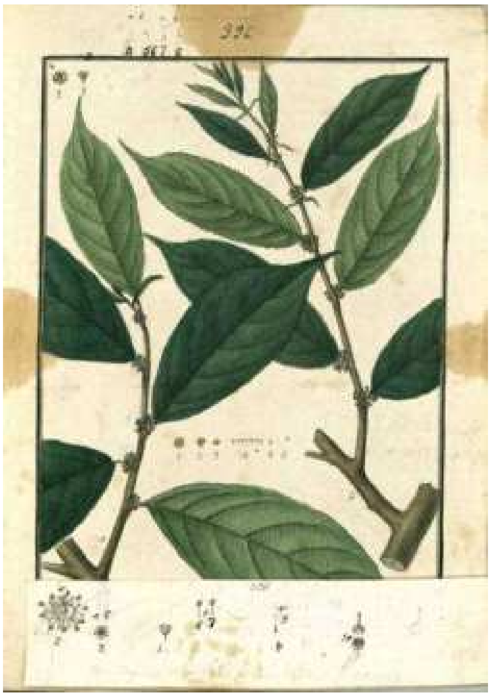

Drawings of the Royal Botanical Expedition to the Viceroyalty of Peru by Ruiz, H., Pavón, J. (1777: t. 396 f. b). Real Jardín Botánico, Madrid, Spain

Historiae Rerum Naturalium Brasiliae | Marcgrave, 1648 | Page number 82

Vernacular name(s) Frutex

Species Sophora tomentosa L.

Family Fabaceae

Notes

Strong correspondence between woodcut and specimen. De Laet (Marcgrave 1648: 82) indicated that they made this figure after some dry leaves .

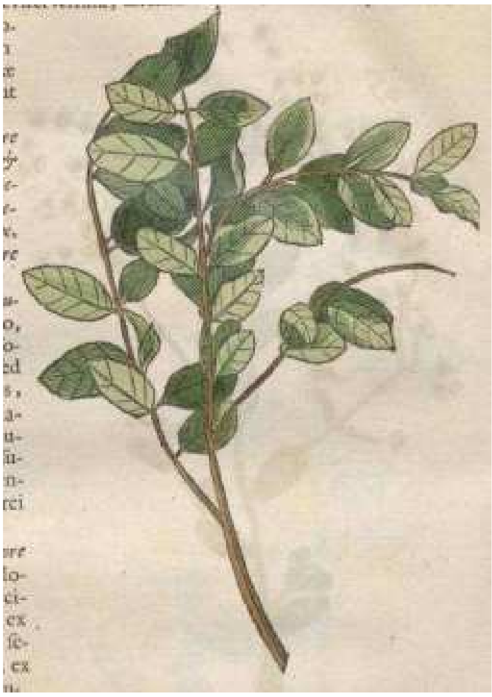

Historiae Plantarum – Plantis Frutescentibus & Fruticibus: 82

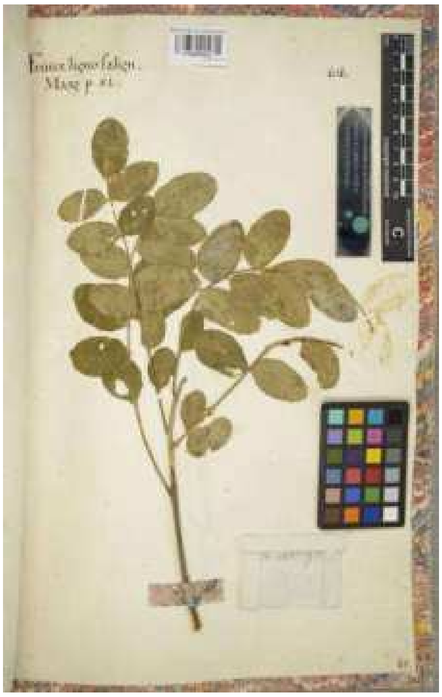

Marcgrave's herbarium: 13

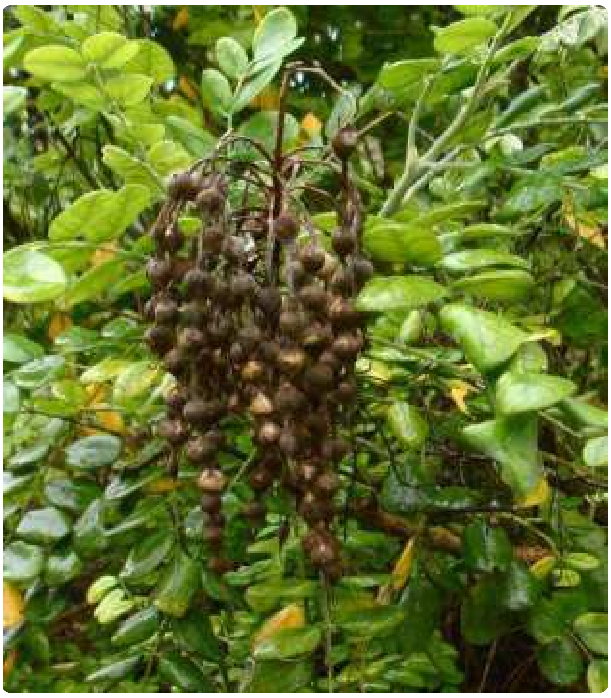

"*S. tomentosa*" by Lauren Gutierrez (CC BY-ND 2.0)

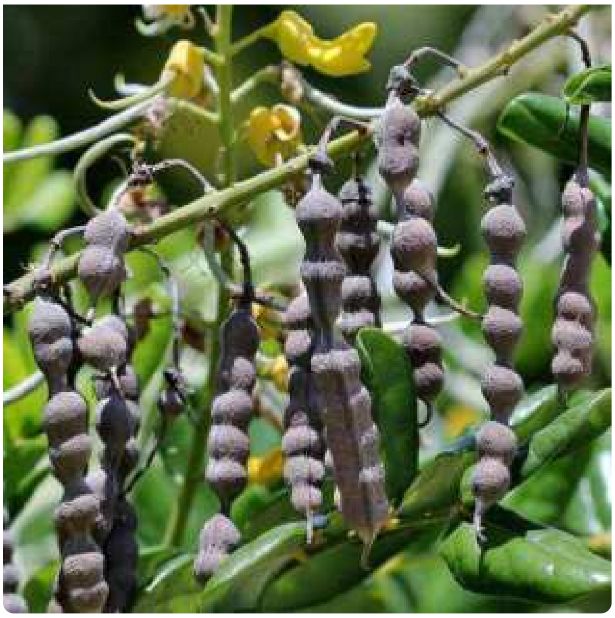

Flowers and pods. "*S. tomentosa*" by Mauricio Mercadante (CC BY-NC-SA 2.0)

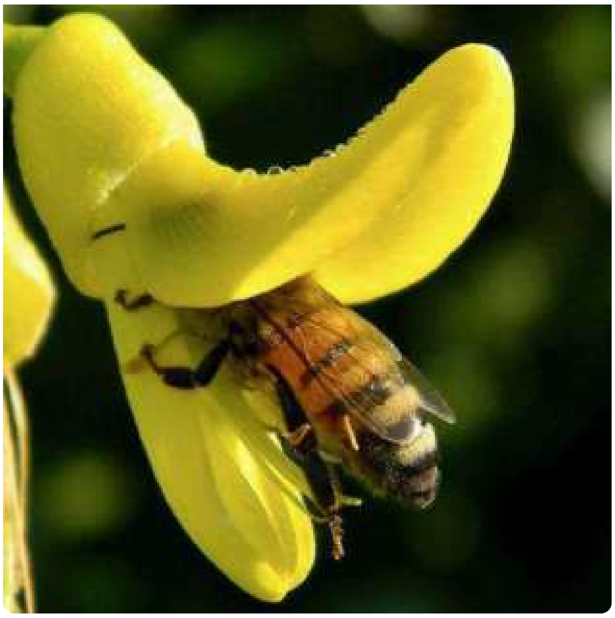

Flower and bee. "Necklacepod (*S. tomentosa*)" by Bob Peterson (CC BY 2.0)

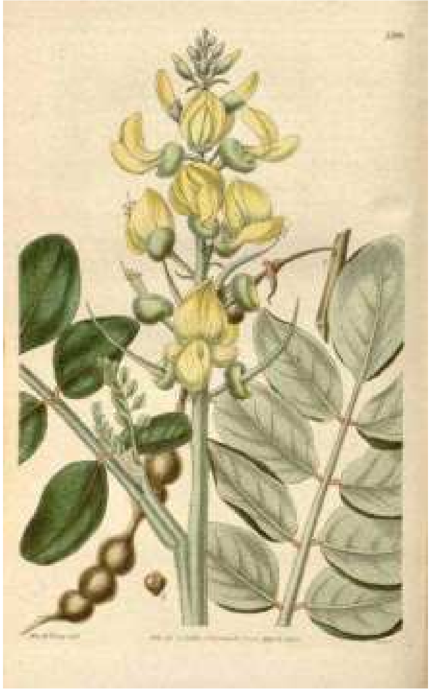

S. tomentosa in Botanical Magazine by Curtis, W. (1835: Vol. 62, t. 3390). Missouri Botanical Garden, St. Louis, U.S.A.

Historiae Rerum Naturalium Brasiliae Marcgrave, 1648 Page number 83

Vernacular name(s) Uubae. Tacomaree. Alfeloa da zuquere. Cana d' azuquere. [illegible]

Species Saccharum officinarum L.

Family Poaceae

Notes

The image of a sugar cane field appears in one of Eckhout's portraits. A similar image appears in the series of tapestries of the Old Indies by the Gobelins (1692 - 1730), likely made after a drawing or sketch by Ekchout that was copied into a cartoon by Alexandre-François Desportes. The woodcut here, however, could have been misplaced. In De Laet's manuscript there is a description for *Gynerium sagittatum* (a relative of *S. officinarum*) with the name *Uuba Brasiliana* and "Icon" written next to it, but without woodcut in the HNB. Hence, De Laet could have arranged the images wrongly.

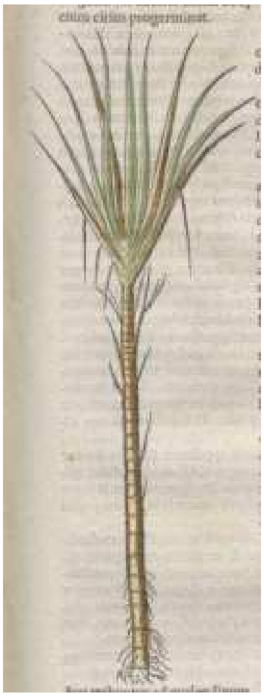

Historiae Plantarum – Plantis Frutescentibus & Fruticibus: 83

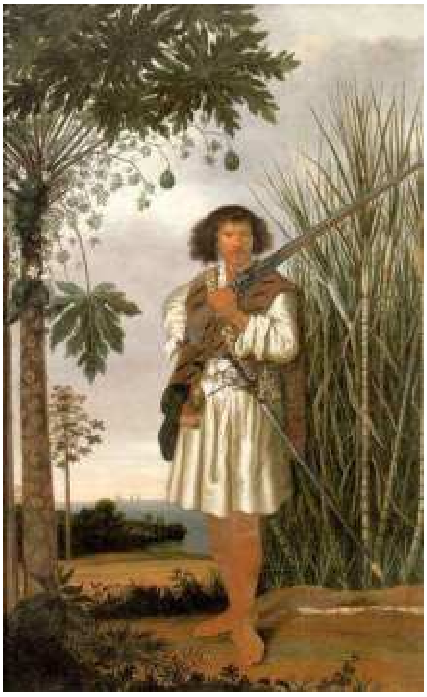

Ekchout's portrait "Biracial man with riffle and sword", ca. 1641. Copenhagen, National Museum of Denmark

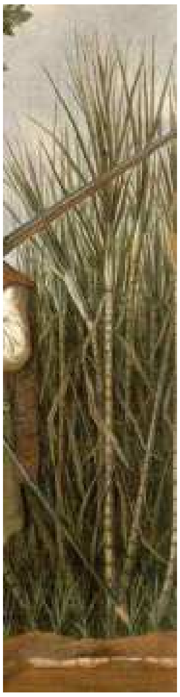

Close - up of the *S. officinarum* in Ekchout's portrait "Biracial man with riffle and sword", ca. 1641. Copenhagen, National Museum of Denmark

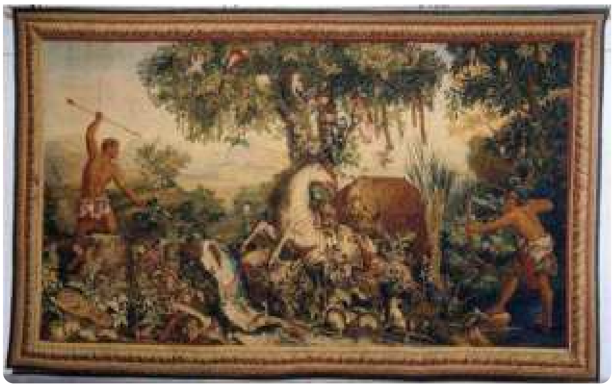

S. officinarum next to the rhinoceros in "The Striped Horse", From the tapestry series *Les Anciennes Indes* (Old Indies), French, Paris, ca. 1692 - 1730

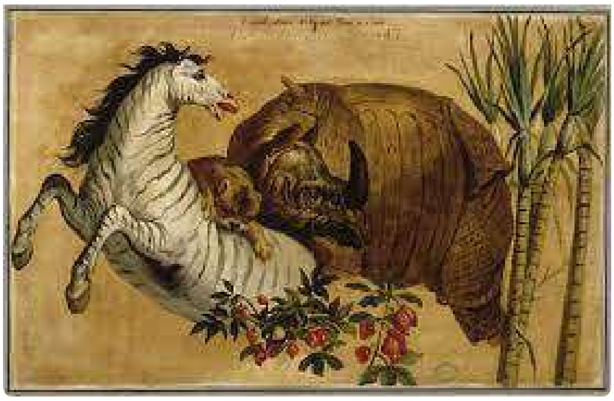

Sugar cane image on the right, In the cartoon "Horse attacked by a lion and rhinoceros". Sevres, I. 123 (177) by Desportes, ca. 1737 (Duclaux 1982)

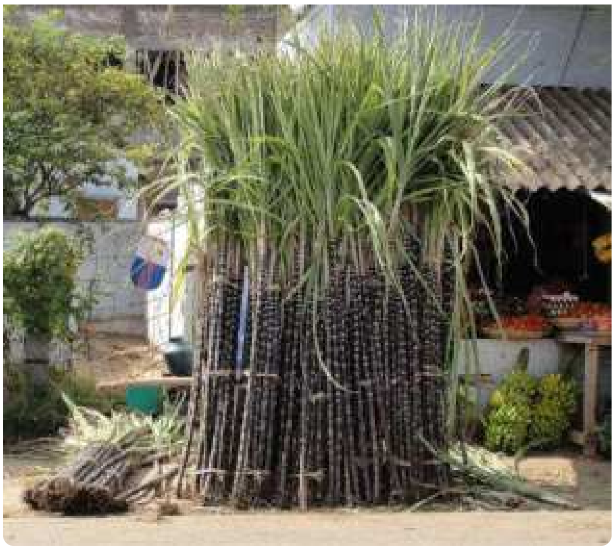

"Sugarcane - *Saccharum officinarum*" by Thamizhpparithi Maari (CC BY-SA 2.0)

Historiae Rerum Naturalium Brasiliae	Marcgrave, 1648	Page number	87a

Vernacular name(s)	Caraguata acanga (88)
Species	<u>Bromelia karatas L.</u>
Family	Bromeliaceae
Notes	

We did not find any correspondence between this woodcut and the contemporary or older sources, although the same plant is described in Piso (1648: 111) and illustrated with a woodcut of a fruit taken by De Laet from his previous work on the Americas (1640: 552) (See S4).

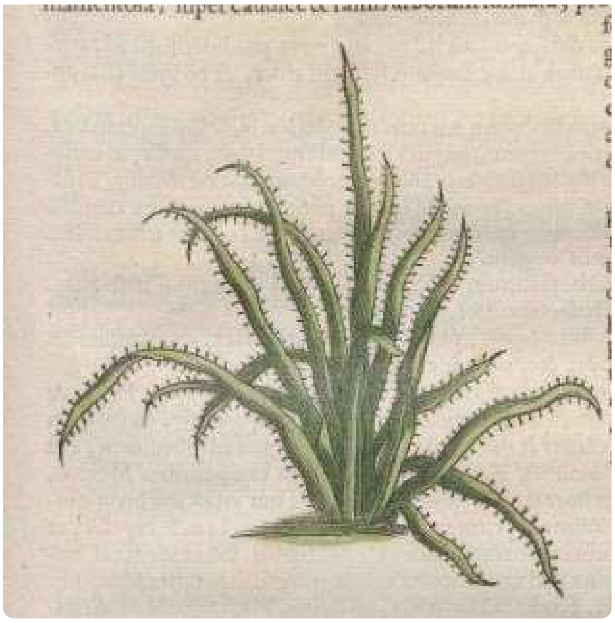

Historiae Plantarum – Plantis Frutescentibus & Fruticibus: 87a

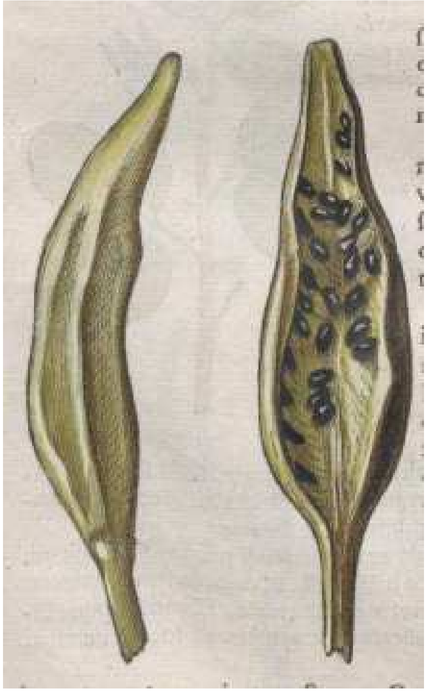

Another woodcut image of *B. karatas* in the HNB (Piso (1648: 111), which was taken from De Laet (1640: 552)

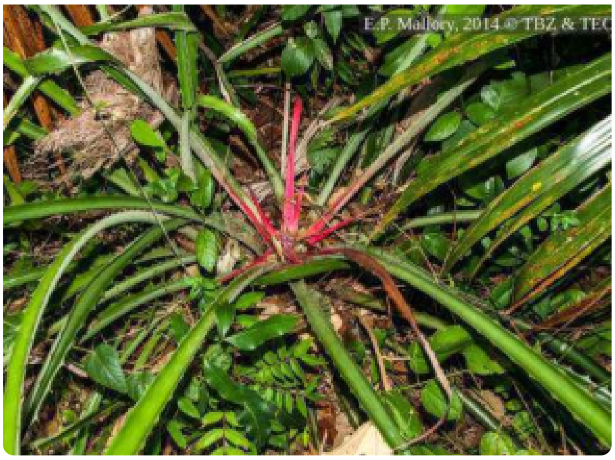

"2014-07-20 TEC-0656 *B. karatas* - E.P. Mallory" by E.P. Mallory (CC BY-NC-SA 2.0)

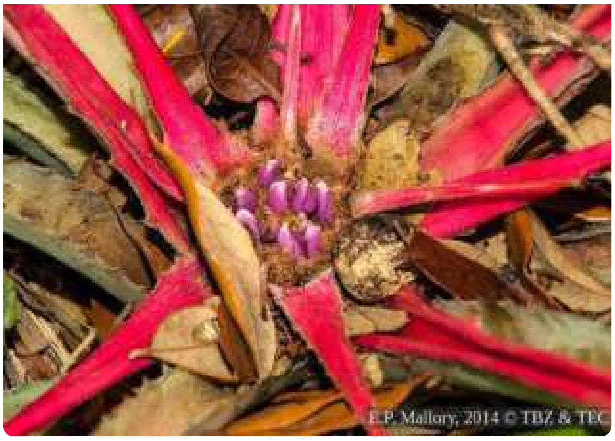

"2014-07-20 TEC-0658 *B. karatas* - E.P. Mallory" by E.P. Mallory (CC BY-NC-SA 2.0)

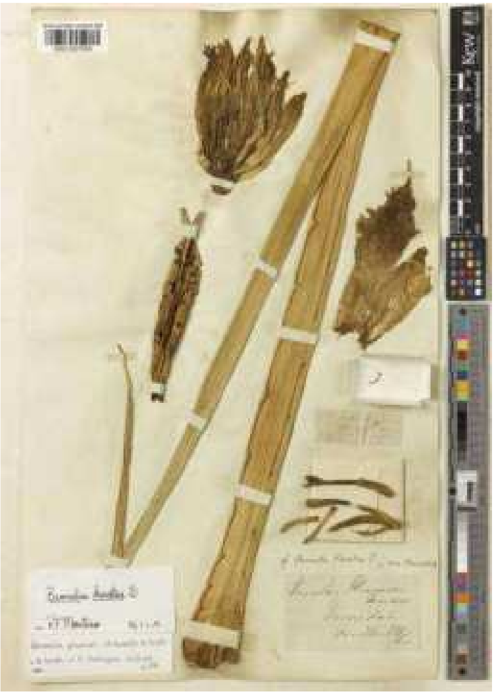

Specimen of *B. karatas* from Kew's Herbarium - K001097585. Retrieved from Plants of the World Online

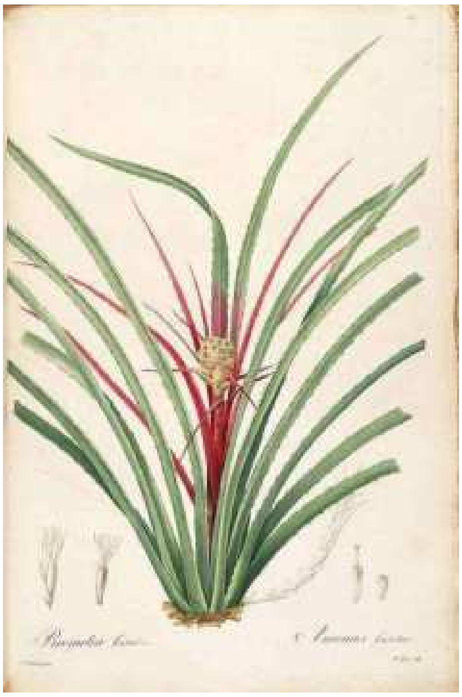

B. karatas in *Liliacées* by Redouté, P.J. (1805: Vol. 8, t. 457). Missouri Botanical Garden, St. Louis, U.S.A.

Historiae Rerum Naturalium Brasiliae — Marcgrave, 1648 — Page number 87b

Vernacular name(s)	Caraguata
Species	Aechmea tomentosa Mez
Family	Bromeliaceae
Notes	

The woodcut is very similar to the *Theatrum* image (reversed).

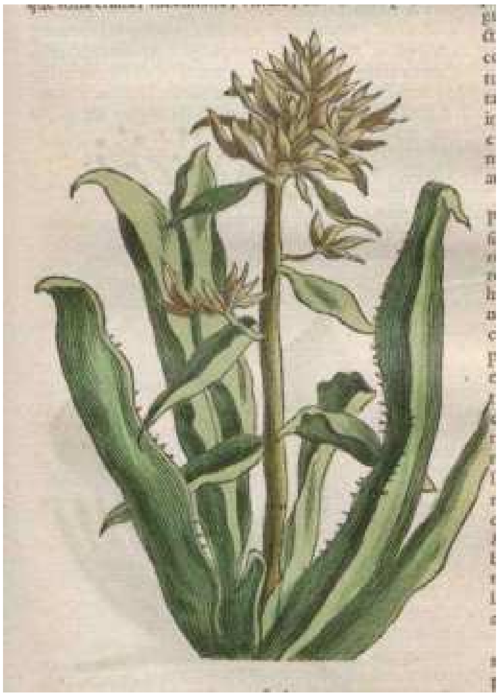

Historiae Plantarum – Plantis Frutescentibus & Fruticibus: 87b

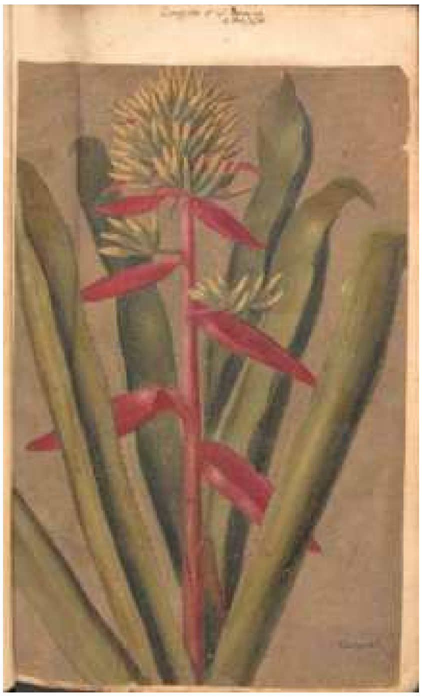

Theatrum Rerum Naturalium: 13

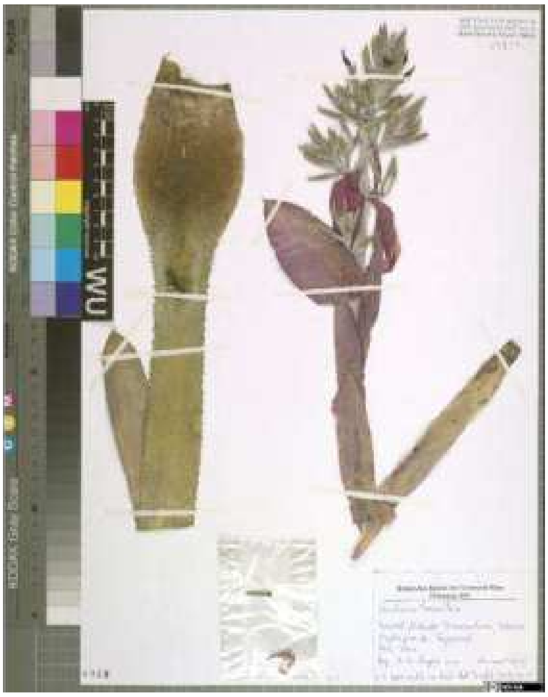

A. tomentosa specimen collected in Pernambuco, Brazil - 0013771- Retrieved from GBIF

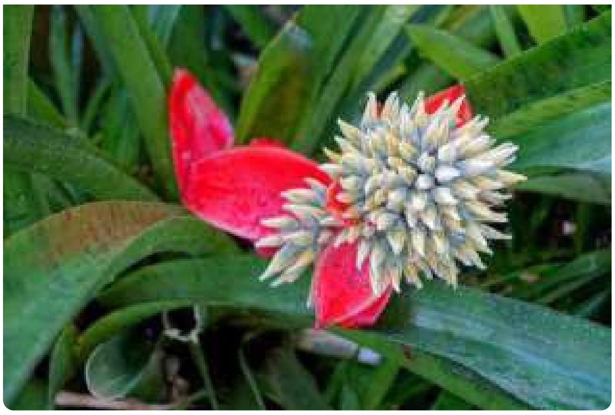

Botanical specimen in the Marie Selby Botanical Gardens - Sarasota, Florida, USA, by Daderot

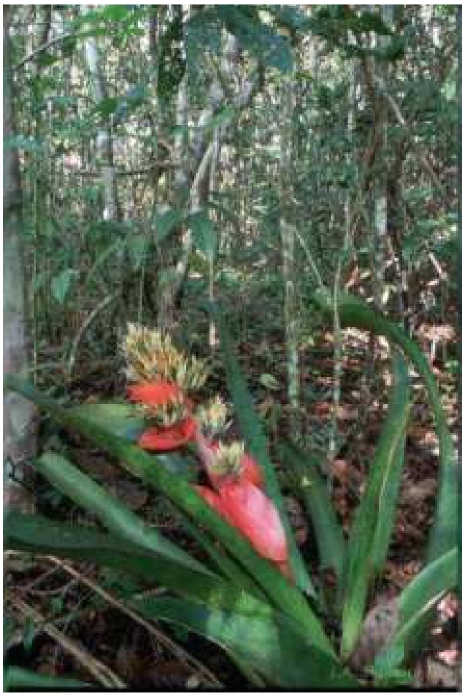

A. tomentosa. Published online JJosé Alves Siqueira Filho in Flora e Funga do Brasil. Author: J.A. Siqueira Filho

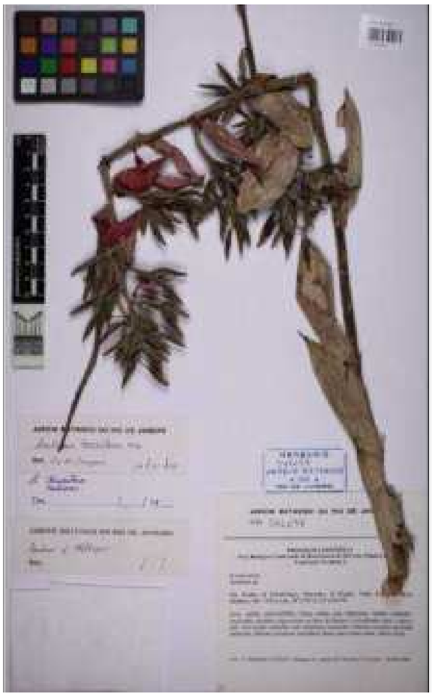

A. tomentosa collected in Pernambuco, Brazil. G. Martinelli, 15129, RB -341697- Retrieved from Flora e Funga do Brazil

Historiae Rerum Naturalium Brasiliae	Marcgrave, 1648	Page number	88

Vernacular name(s) Caraguata guacu (87)

Species Furcraea hexapetala (Jacq.) Urb.

Family Asparagaceae

Notes

De Laet reused the woodcut he previously published in his books on the Americas (De Laet 1633: 666; 1640: 608). He received this image from a friend that came back from the island of Tobago (Marcgrave 1648: 88).

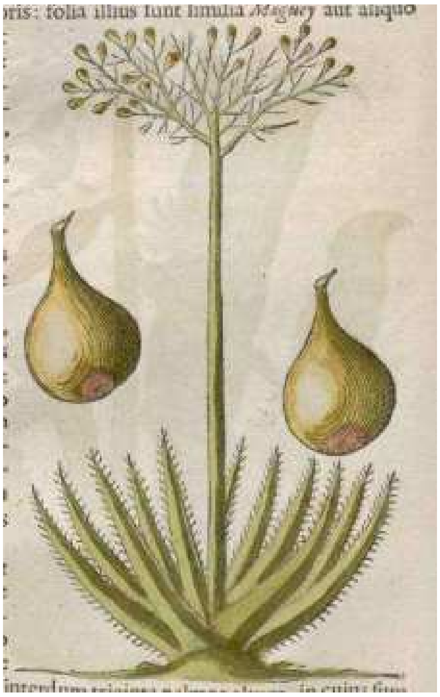

Historiae Plantarum – Plantis Frutescentibus & Fruticibus: 88

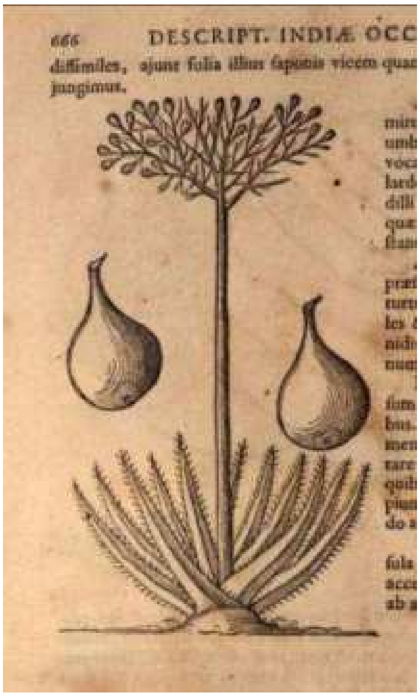

Woodcut of *F. hexapetala* in *Novus Orbis seu descriptionis Indiae Occidentalis* by Johannes De Laet (1633: 666)

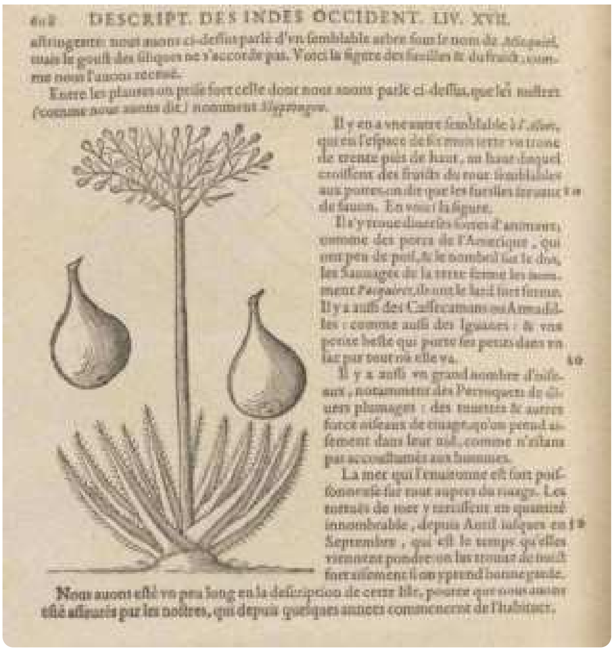

608 DESCRIPT. DES INDES OCCIDENT. LIV. XVII.

astringente: nous auons ci-dessus parlé d'vn semblable arbre sous le nom de *[illegible]*, mais le goust des siliques ne s'accorde pas. Voici la figure des fueilles & du fruict, comme nous l'auons receuë.

Entre les plantes on prise fort celle dont nous auons parlé ci-dessus, que les nostres (comme nous auons dit) nomment *[illegible]*.

Il y en a vne autre semblable à l'*Aloe*, qui en l'espace de six mois iette vn tronc de trente pieds de haut, au haut duquel croissent des fruicts du tout semblables aux poires, on dit que les fueilles seruent en lieu de sauon. En voici la figure.

Il s'y trouue diuerses sortes d'animaux, comme des porcs de l'Amerique, qui ont peu de poil, & le nombril sur le dos, les Sauuages de la terre ferme les nomment *Paquires*, ils ont le lard fort ferme. Il y a aussi des Cassicamans ou Armadilles: comme aussi des Iguanes: & vne petite beste qui porte ses petits dans vn sac par tout où elle va.

Il y a aussi vn grand nombre d'oiseaux, notamment des Perroquets de diuers plumages: des tourtres & autres sortes oiseaux de riuage, qu'on prend aisement dans leur nid, comme n'estans pas accoustumés aux hommes.

La mer qui l'enuironne est fort poissonneuse sur tout autour du riuage. Les tortuës de mer y naissent en quantité innombrable, depuis Auril iusques en Septembre, qui est le temps qu'elles viennent pondre: on les trouue de nuict fort aisement si on y prend bonne garde.

Nous auons esté vn peu long en la description de cette Isle, pource que nous auons esté asseurés par les nostres, qui depuis quelques années commencent de l'habiter.

Same woodcut of *F. hexapetala* in *L'histoire du Nouveau Monde, ou, Description des Indes Occidentales*.by Johannes De Laet (1640: 608)

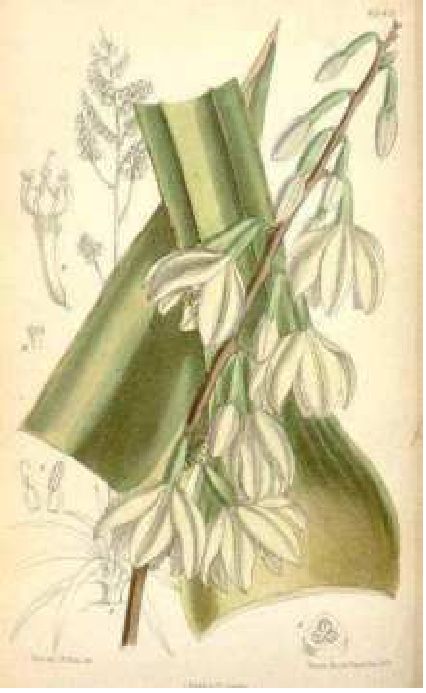

F. hexapetala in *Botanical Magazine* by Curtis, W. (1881: Vol. 107, t. 6543). Missouri Botanical Garden, St. Louis, U.S.A.

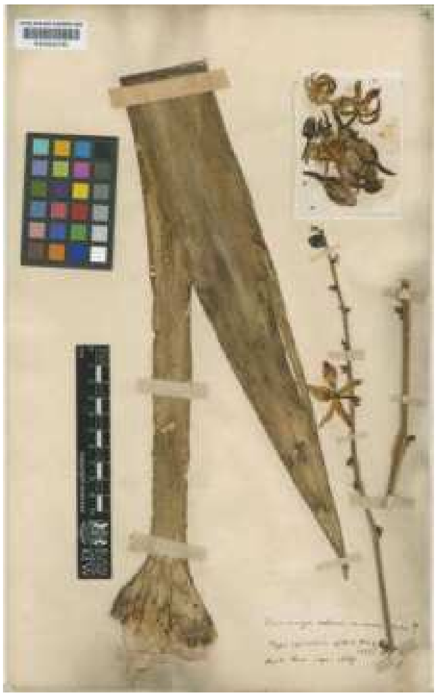

Specimen of *F. hexapetala* from Kew's Herbarium - K000524783. Retrieved from Plants of the World Online

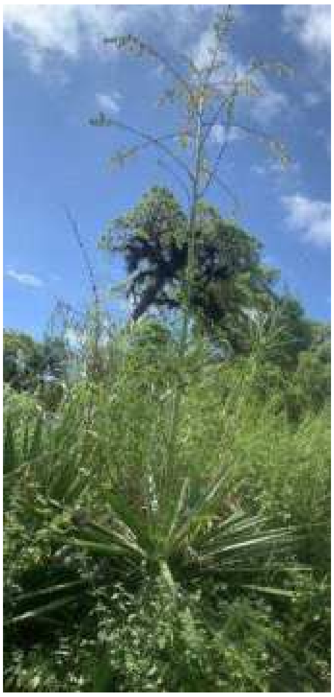

F. hexapetala observed in Ecuador by John G. Phillips for iNaturalist (CC BY-NC 4.0)

Historiae Rerum Naturalium Brasiliae Marcgrave, 1648 Page number 89

Vernacular name(s) Iurepeba

Species Solanum paniculatum L.

Family Solanaceae

Notes

The woodcut differs from the *Theatrum*. The woodcut also differs from the drawing in the *Misc. Cleyeri* and the specimen, although both the woodcut and the specimen consist of flowering branches.

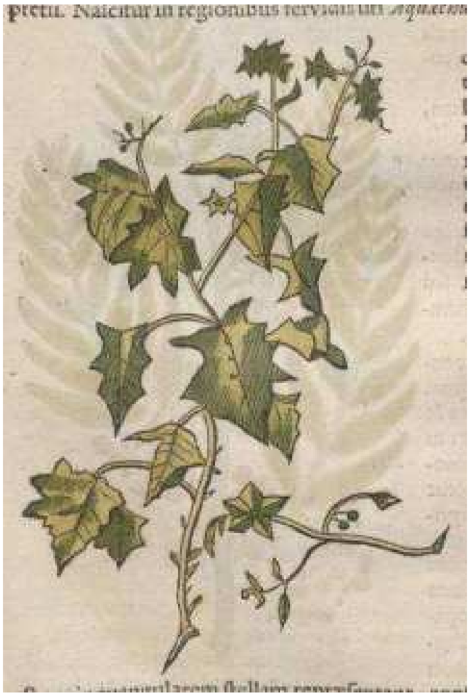

Historiae Plantarum – Plantis Frutescentibus & Fruticibus: 89

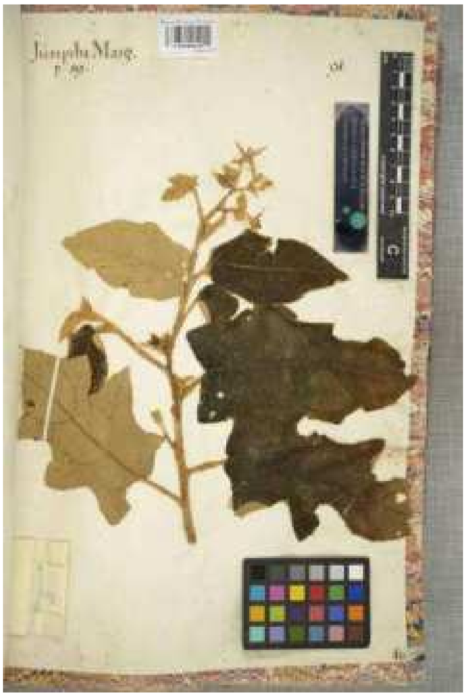

Marcgrave's herbarium: 16

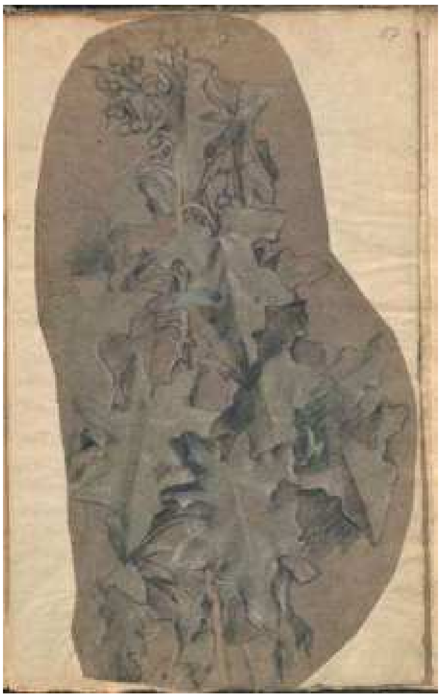

Miscellanea Cleyeri: 57r

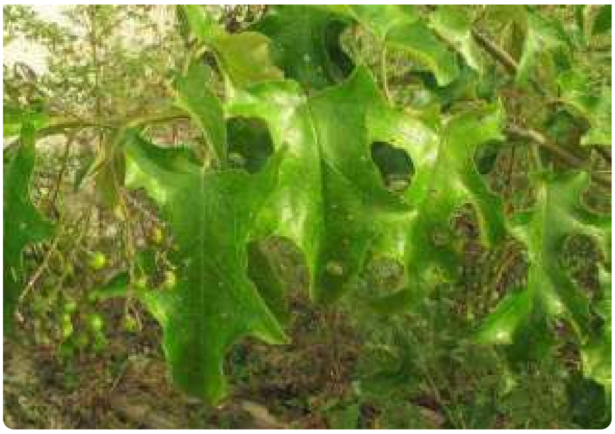

"*Solanum paniculatum* L." by Alex Popovkin, Bahia, Brazil (CC BY-NC-SA 2.0)

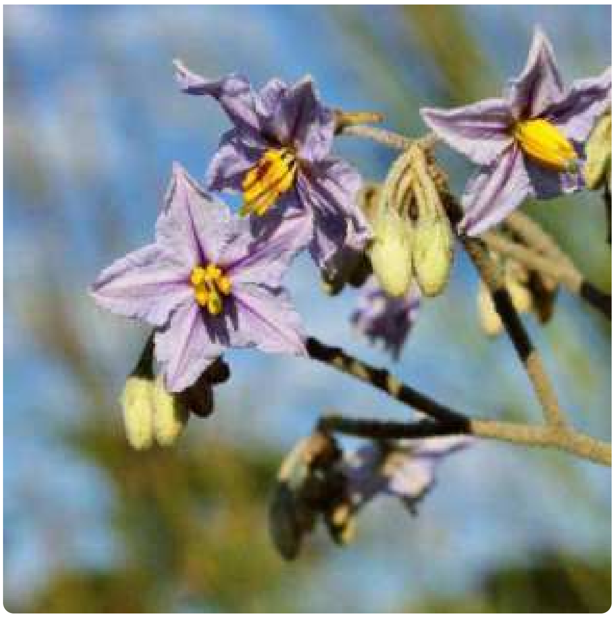

"*S. paniculatum*" by Mauricio Mercadante (CC BY-NC-SA 2.0)

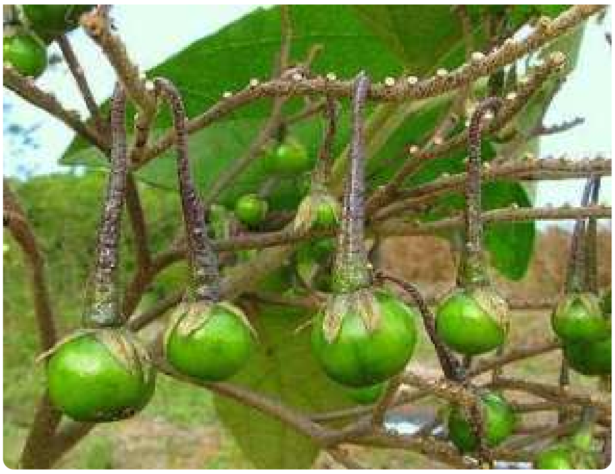

"*S. paniculatum*" by Alex Popovkin, Bahia, Brazil (CC BY-NC-SA 2.0)

Historiae Rerum Naturalium Brasiliae Marcgrave, 1648 Page number 90

Vernacular name(s) Aroeira. Lentiscus

Species Schinus terebinthifolia Raddi

Family Anacardiaceae

Notes

The woodcut is different from the *Theatrum* illustration. De Laet used this image in his book, so he could have had the woodblock which originally belonged to Christopher Plantin's printing business; likely owned by their successors Elzeviers. It was originally used in Clusius' work, in which he wrote commentaries on the work on Amerindian plants by Spanish physician Monardes (1580: 39). This image, as well as the woodcut, corresponds to a relative species (*Schinus molle* L.) and according to De Laet (1640: 327), he was describing a Peruvian tree.

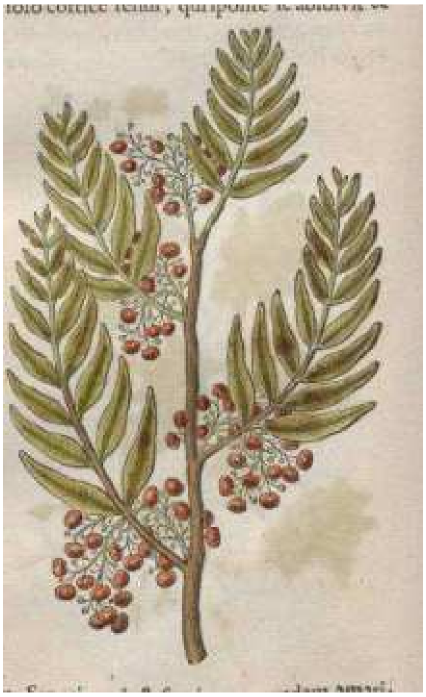

Historiae Plantarum – Arboribus: 90

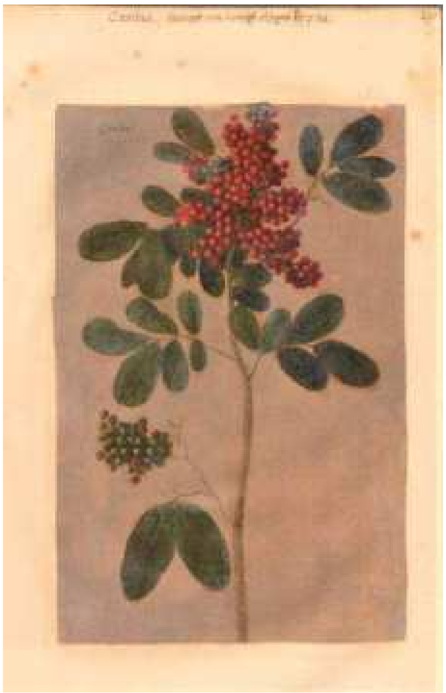

Theatrum Rerum Naturalium: 295

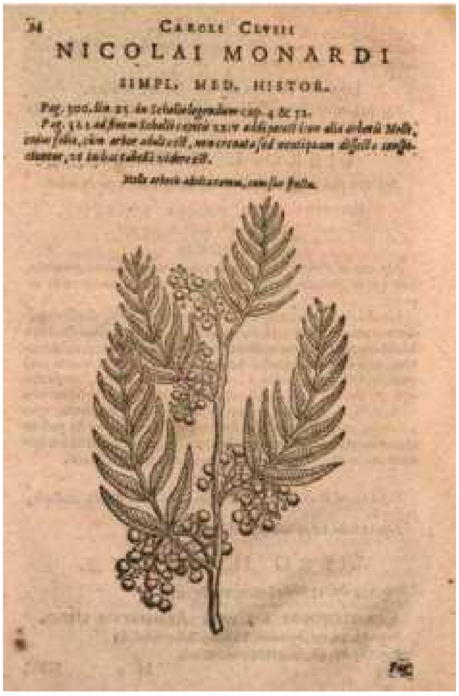

Schinus molle branch depicted in *Curae Posteriores* by Clusius (1611: 94)

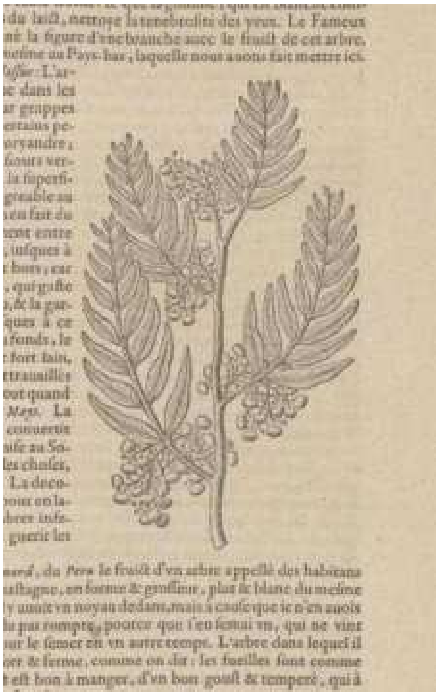

S. molle woodcut copied from Clusius' one in *History of the New World and the Description of the West Indies* by De Laet (1640: 327)

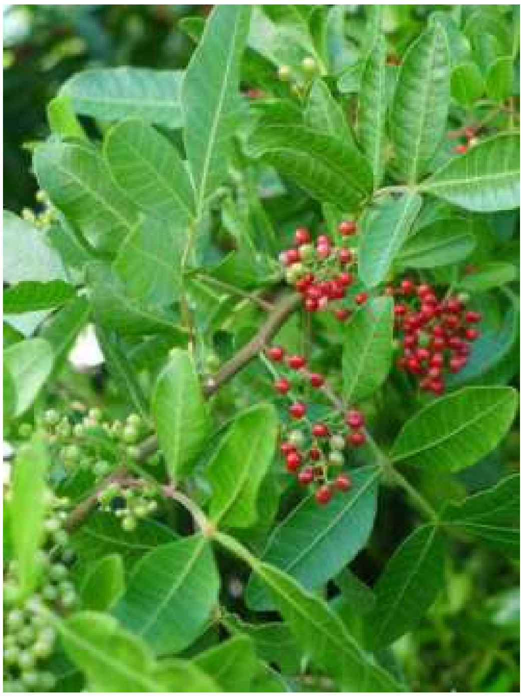

Fruiting branch. "*S. terebinthifolia* 2" by Scott Zona (CC BY-NC 2.0)

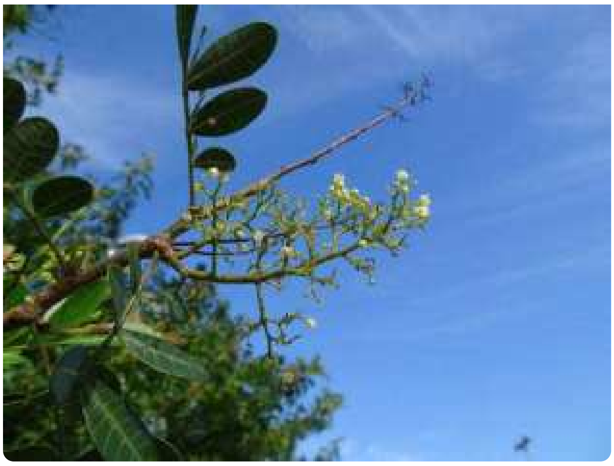

Flowering branch- "Aroeira-vermelha, aroeira-pimenteira, poivre-rose '*Schinus terebinthifolia*'" by mauro halpern

Historiae Rerum Naturalium Brasiliae Marcgrave, 1648 Page number 91

Vernacular name(s) Ambaiba

Species Cecropia pachystachya Trécul

Family Urticaceae

Notes

The woodcut image is very similar to the *Theatrum* image (non-reversed). The specimen does not resemble the woodcut, but several loose leaves could have been used to draw the leaves that emerge from the tree trunk in the woodcut and the illustration.

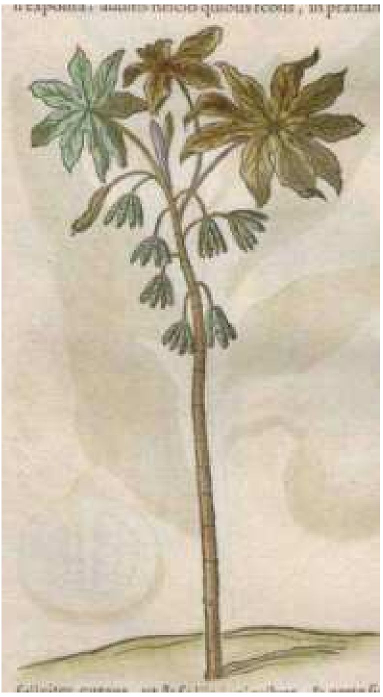

Historiae Plantarum – Arboribus: 91

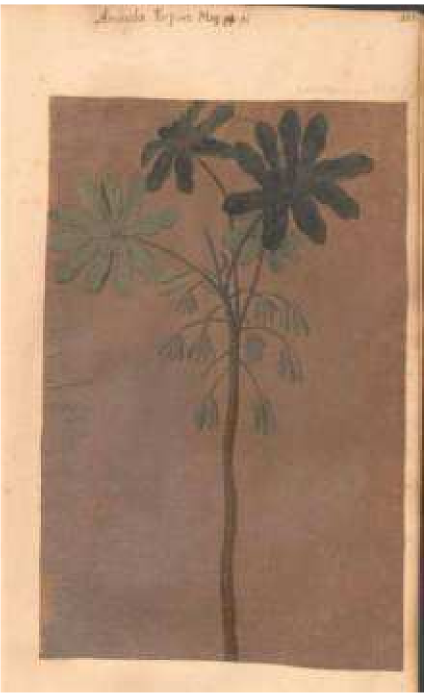

Theatrum Rerum Naturalium: 137

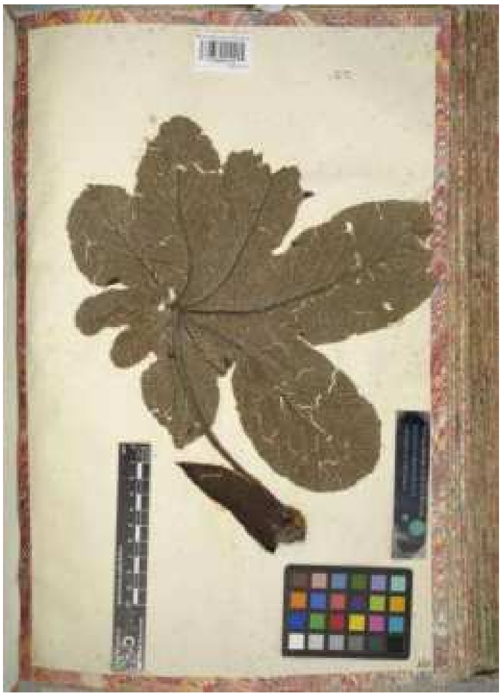

Marcgrave's herbarium: 115

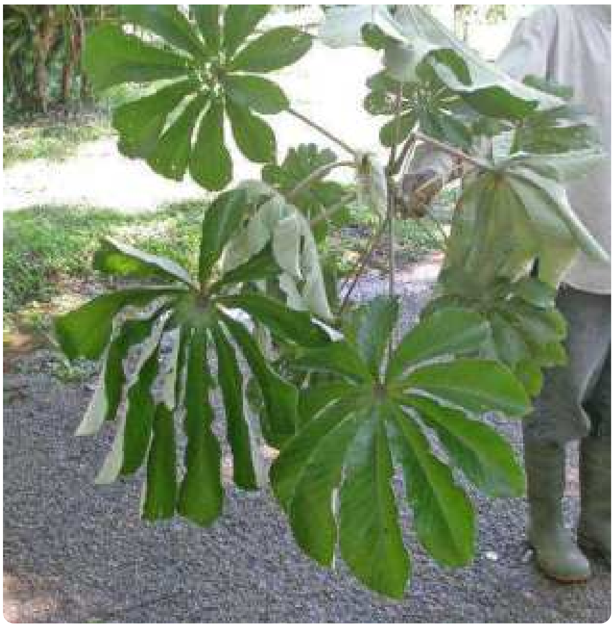

"*C. pachystachya* (URTICACEAE)" by Scamperdale (CC BY-NC 2.0)

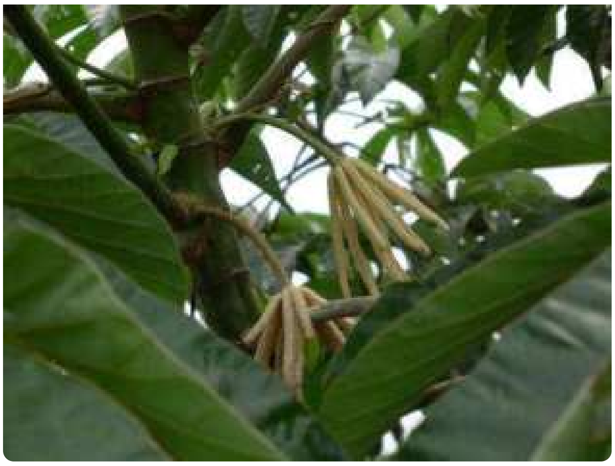

"*C. pachystachya* (URTICACEAE)" by Scamperdale (CC BY-NC 2.0)

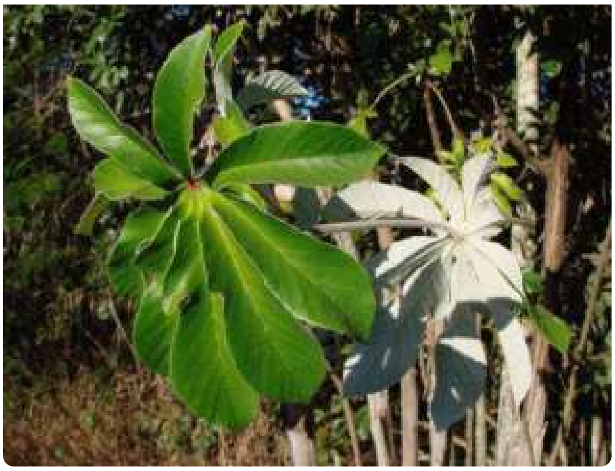

"*C. pachystachya*" by João de Deus Medeiros (CC BY 2.0)

Historiae Rerum Naturalium Brasiliae Marcgrave, 1648 Page number 92

Vernacular name(s) Ianipaba. Ienipapo

Species Genipa americana L.

Family Rubiaceae

Notes

The woodcut differs from the *Theatrum* illustration. The woodcut shows a more botanical "complete" image, combining flowers and fruits (one open and showing the seeds).

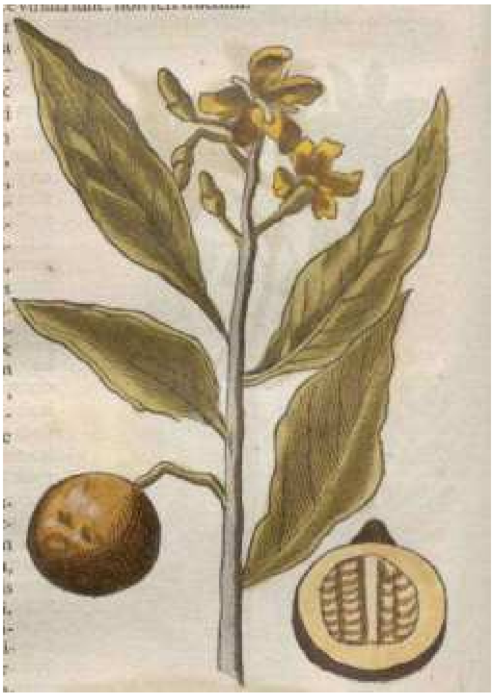

Historiae Plantarum – Arboribus: 92

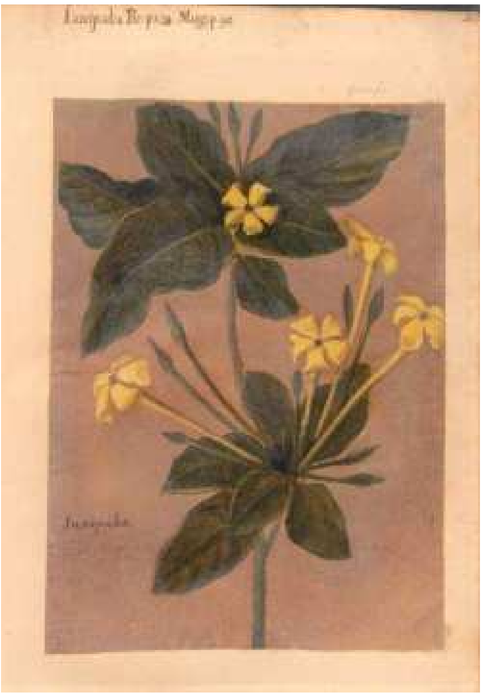

Theatrum Rerum Naturalium: 109

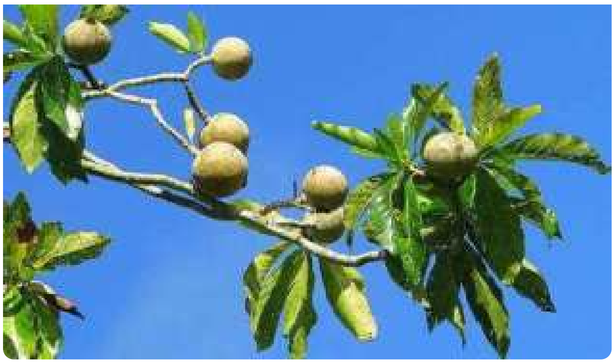

Fruiting branch. "*G. americana*." by Alex Popovkin, Bahia, Brazil (CC BY-NC-SA 2.0)

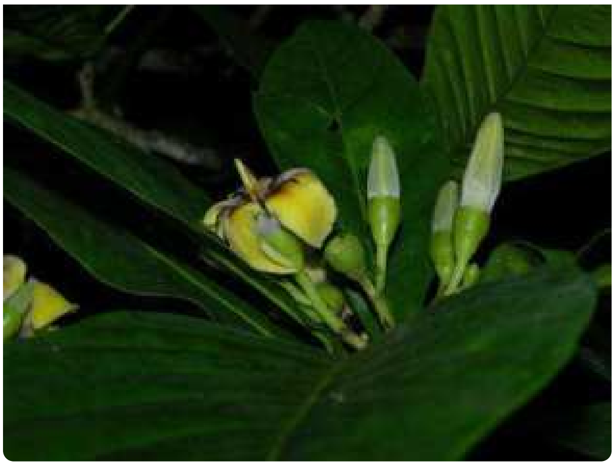

Flowers. "*G. americana*." by Reinaldo Aguilar (CC BY-NC-SA 2.0)

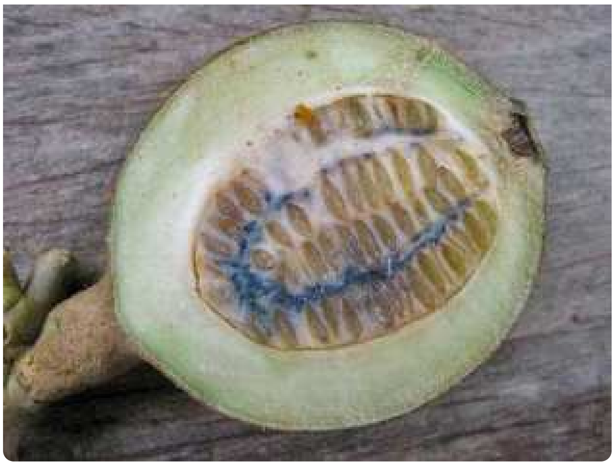

Open fruit and seeds. "*G. americana*." by Alex Popovkin, Bahia, Brazil (CC BY-NC-SA 2.0)

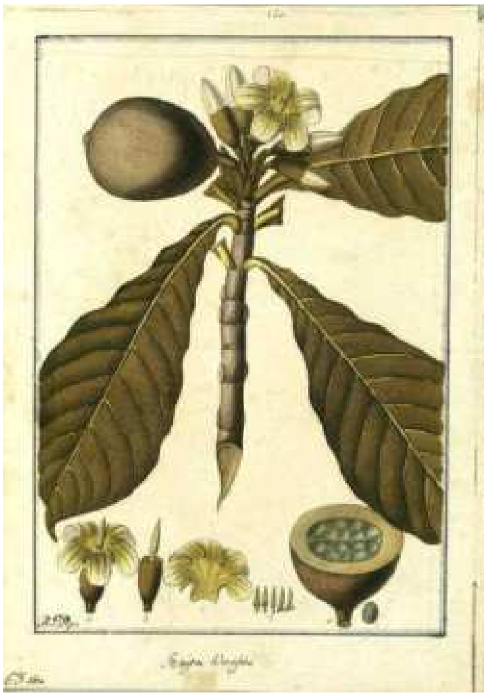

Drawings of the Royal Botanical Expedition to the Viceroyalty of Peru by Ruiz, H., Pavón, J. (1777: t. 220). Real Jardín Botánico, Madrid, Spain

Historiae Rerum Naturalium Brasiliae Marcgrave, 1648 Page number 93

Vernacular name(s) Araticu-pohne. Araticu ape

Species Annona montana Macfad.

Family Annonaceae

Notes

The woodcut is very similar to the *Theatrum* image (non-reversed). There is no resemblance between the woodcut and the specimen, which only consists of infertile material. The oil painting looks more loaded with leaves.

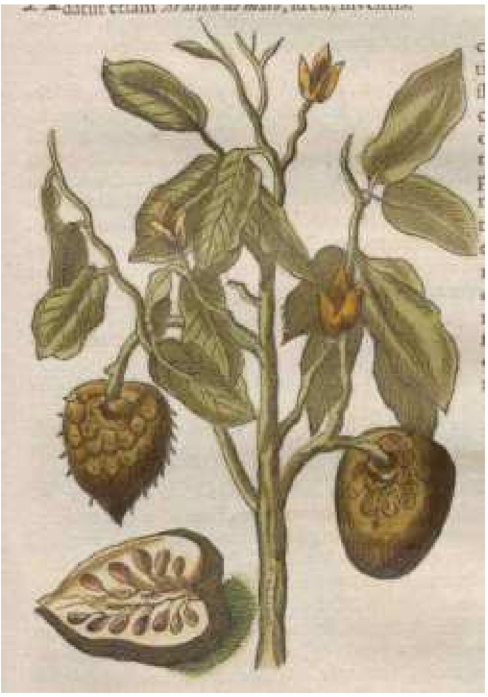

Historiae Plantarum – Arboribus: 93

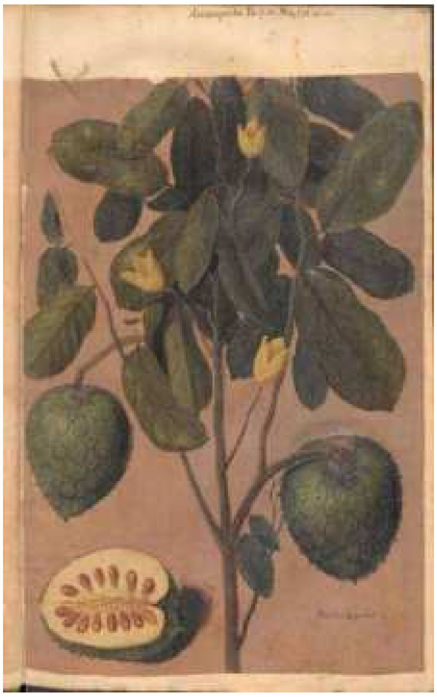

Theatrum Rerum Naturalium: 117

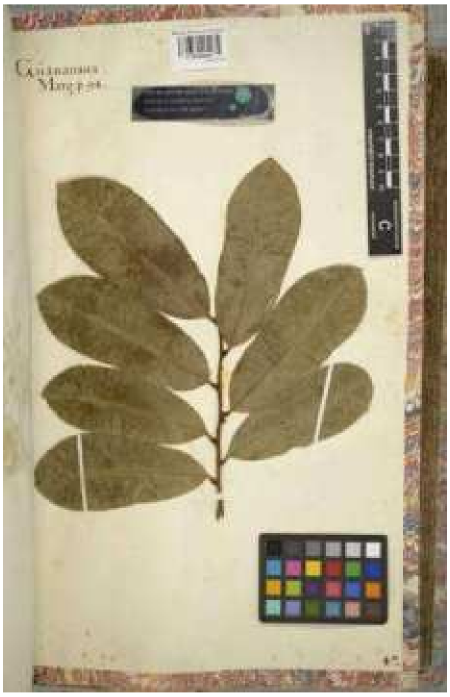

Marcgrave's herbarium: 47

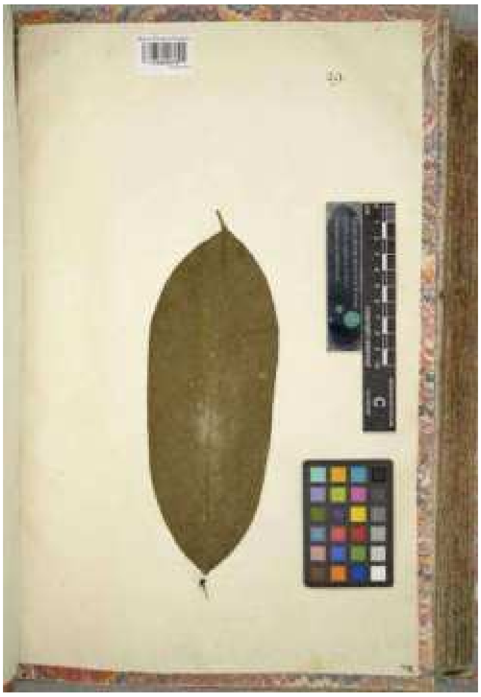

Marcgrave's herbarium: 78

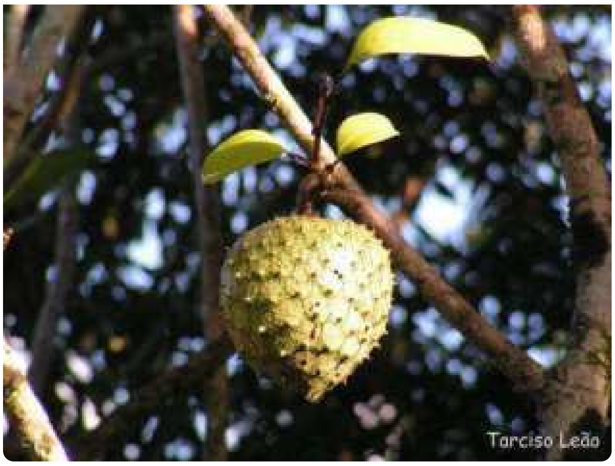

Fruit. "*A. montana*, aticum" by Tarciso Leão (CC BY 2.0)

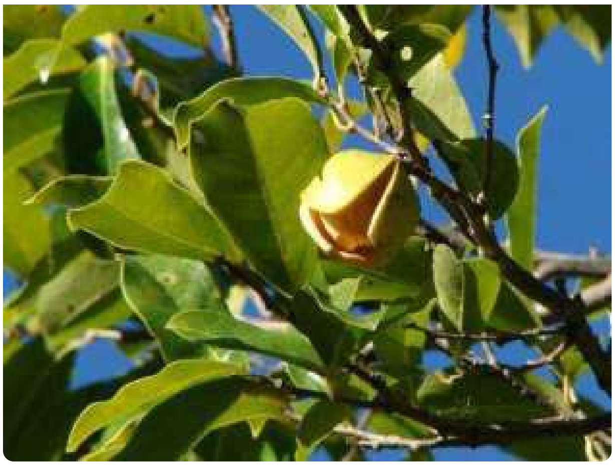

"Flower of Mountain Soursop (*Annona montana*)" by Tatters (CC BY 2.0)

Historiae Rerum Naturalium Brasiliae | Marcgrave, 1648 | Page number 95a

Vernacular name(s) Acaiaiba.Acaiuiba

Species Anacardium occidentale L.

Family Anacardiaceae

Notes

The woodcut differs from the *Theatrum* image and the herbarium specimen. The specimen's flowers differ greatly from the *Theatrum* illustration, which resembles the fruiting and flowering branches of the cashew tree depicted in one of Eckhout's portraits.

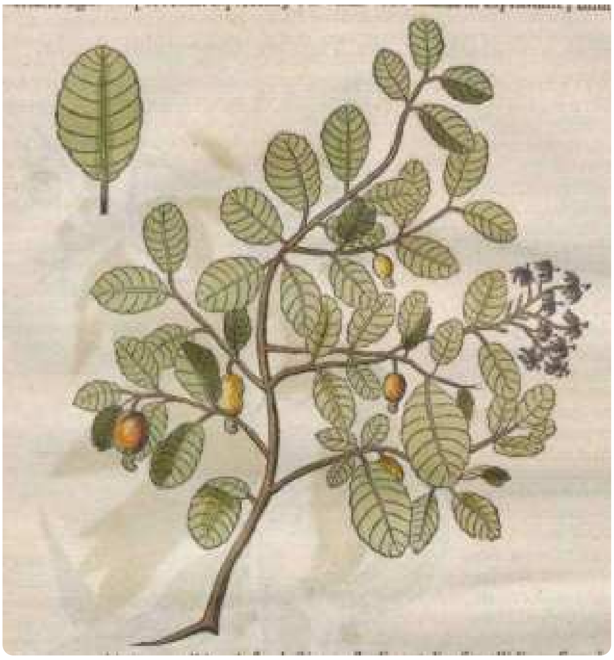

Historiae Plantarum – Arboribus: 95a

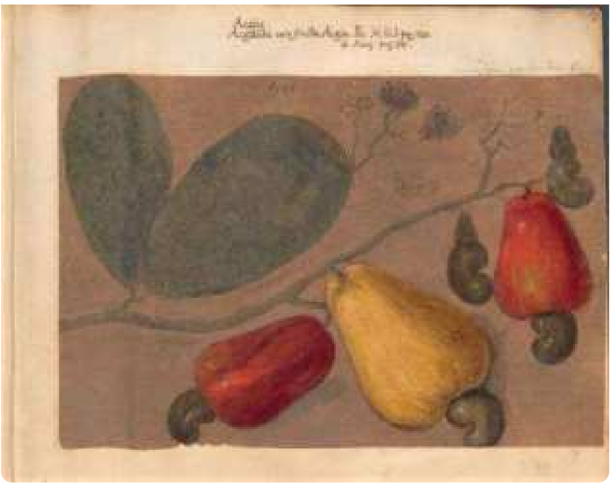

Theatrum Rerum Naturalium: 19

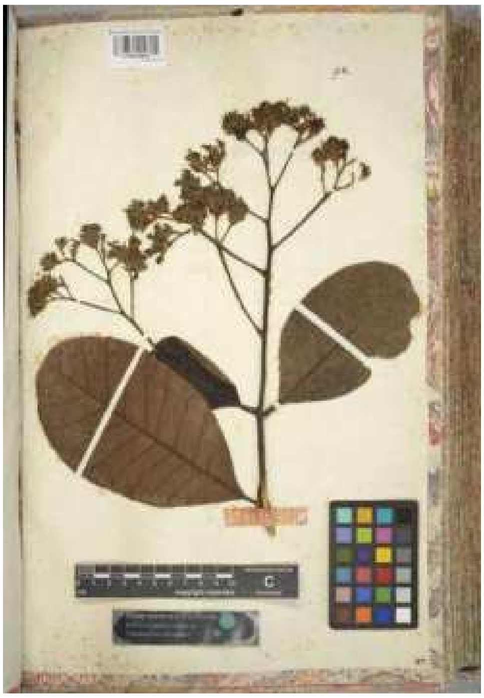

Marcgrave's herbarium: 87

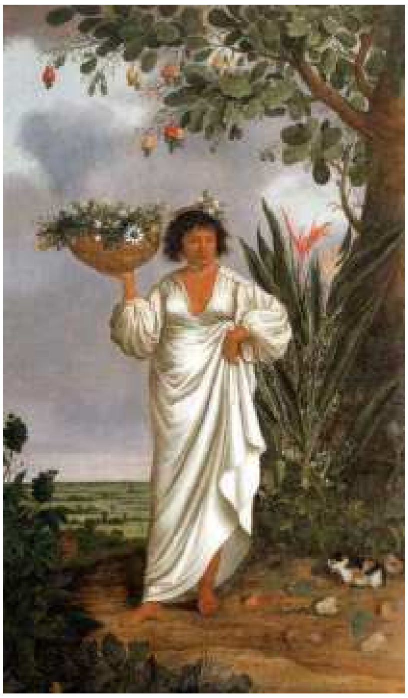

Ekchout's portrait "Mameluka with a basket of flowers", ca. 1640. Copenhagen, National Museum of Denmark

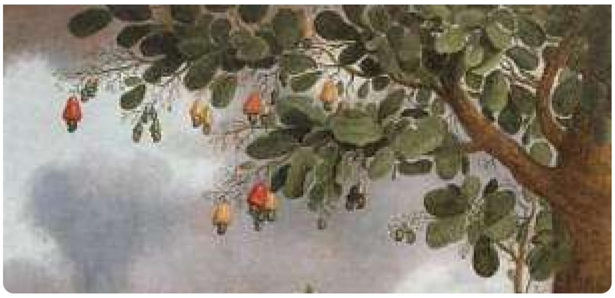

Close- up of the *A. occidentale* branch in Ekchout's portrait "Mameluka with a basket of flowers", ca. 1640. Copenhagen, National Museum of Denmark

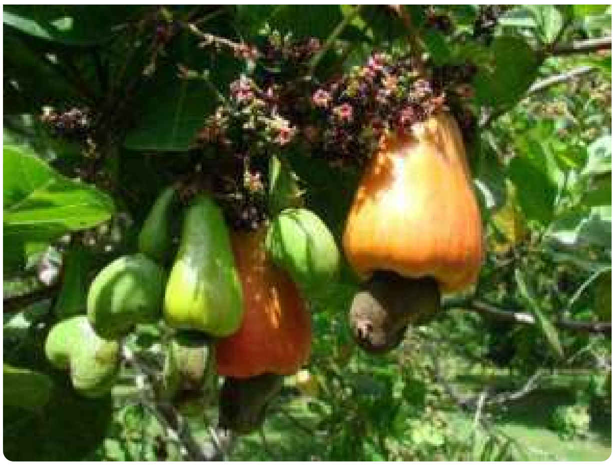

"Merey [Cashew (flower + unripe fruit + ripe fruit] (*Anacardium occidentale*)" by barloventomagico (CC BY-NC-ND 2.0)

Historiae Rerum Naturalium Brasiliae — Marcgrave, 1648 — Page number 95b

Vernacular name(s) Acaiû. Cajû.

Species Anacardium occidentale L.

Family Anacardiaceae

Notes

The woodcut resembles the image of the cashew fruit in Clusius (1605) (the seed that is cut in half), which De Laet (1633, 1640) also used in his books. Although this image is presented very close to the entire fruit that also corresponds to *A. occidentale,* by looking at their different orientation in the multiple treatises (including Piso 1658: 121), we realised that these images came from two woodblocks. This is also seen in Ole Worm´s *Museum Wormianum* (1655: 183, 192).

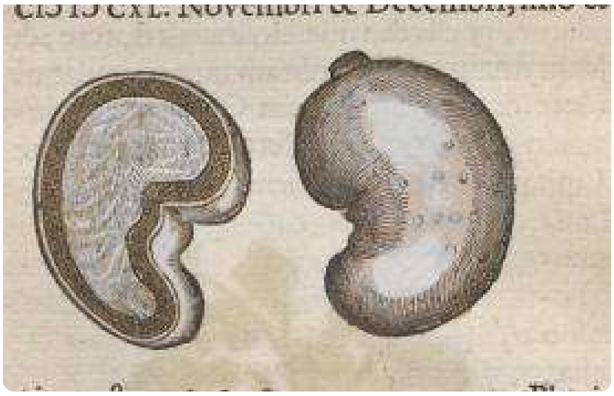

Historiae Plantarum – Arboribus: 95b

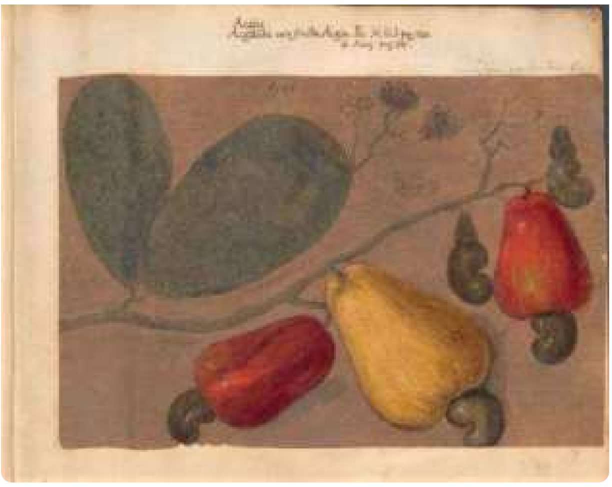

Theatrum Rerum Naturalium: 19

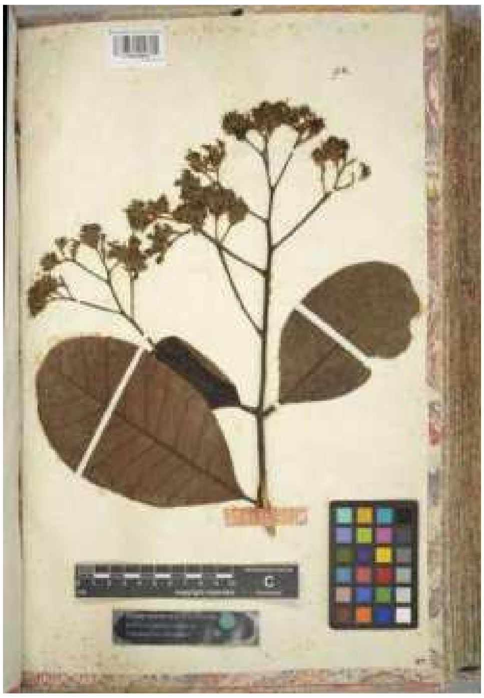

Marcgrave's herbarium: 87

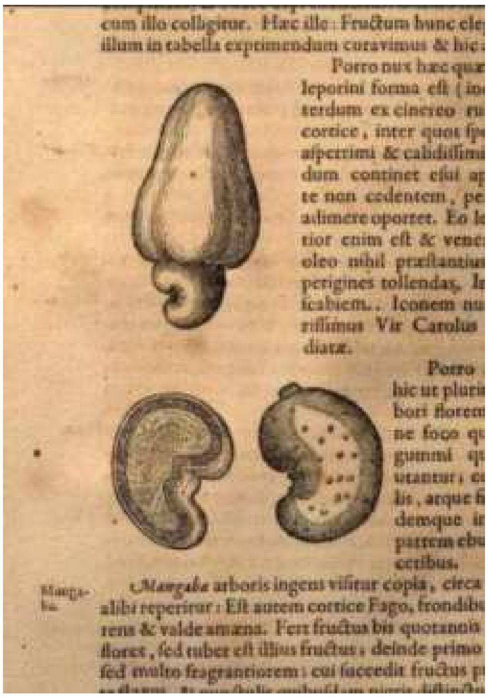

Woodcut of *A. occidentale* fruit and seed in *Novus Orbis seu descriptionis Indiae Occidentalis* by Johannes De Laet (1633: 558)

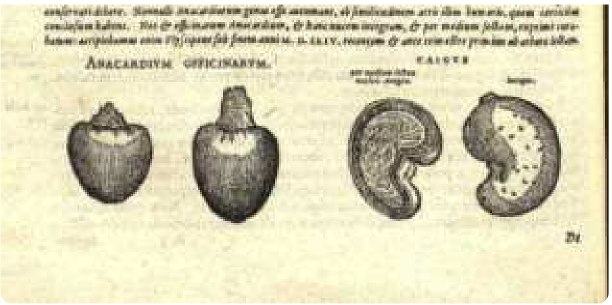

Woodcut of the cashew fruit and seed in *Exoticorum libri decem* by Clusius (1605: 198. livr. I, cap. XXX)

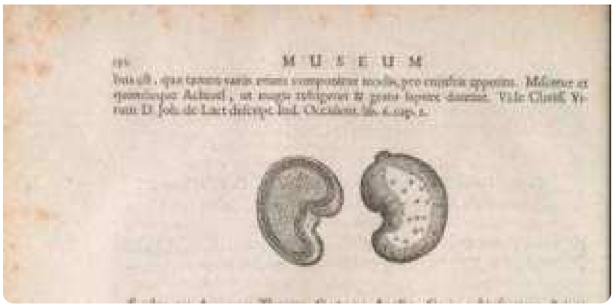

A. occidentale in *Museum Wormianum* by Ole Worm (1655: 183, 192)

Historiae Rerum Naturalium Brasiliae Marcgrave, 1648 Page number 95c

Vernacular name(s) Acaiû. Cajû.

Species Anacardium occidentale L.

Family Anacardiaceae

Notes

This woodcut is very similar to the image of the cashew fruit published by De Laet 1633, 1644).

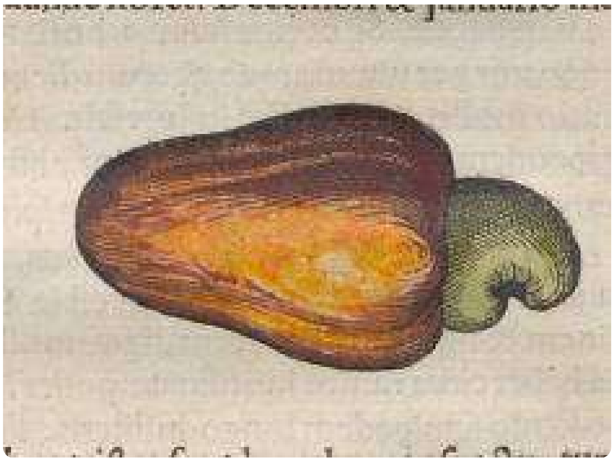

Historiae Plantarum – Arboribus: 95c

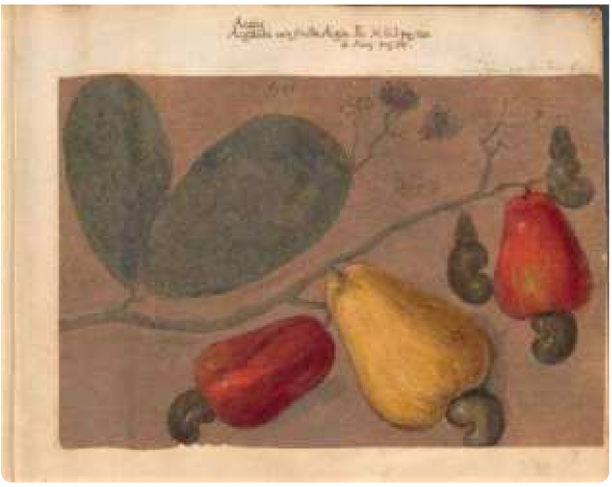

Theatrum Rerum Naturalium: 19

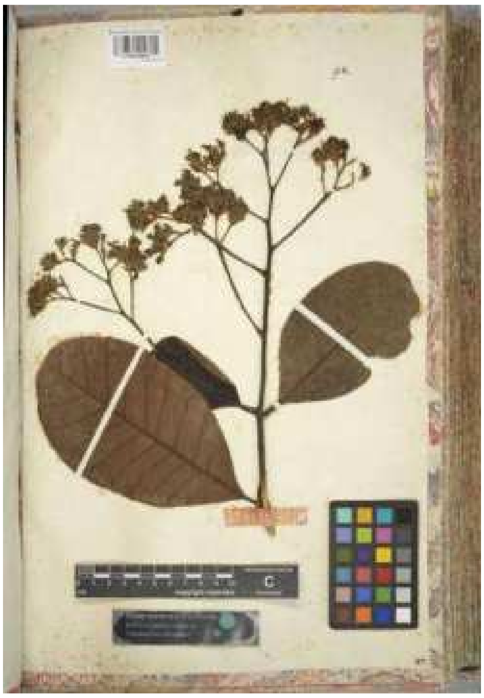

Marcgrave's herbarium: 87

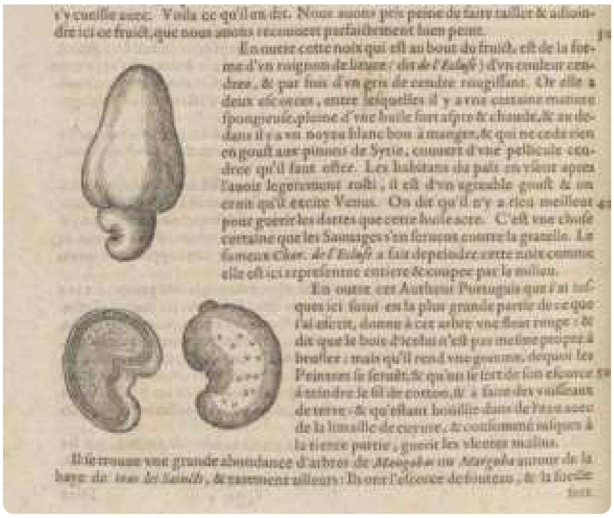

s'y cueille aussi. Voila ce qu'il en dit. Nous auons pris peine de faire tailler & adioindre ici ce fruict, que nous auons recouuert parfaitement bien peint.

En outre cette noix qui est au bout du fruict, est de la forme d'vn roignon de lieure (dit de l'Escluse) d'vn couleur cendree, & par fois d'vn gris de cendre rougissant. Or elle a deux escorces, entre lesquelles il y a vne certaine matiere spongieuse, pleine d'vne huile fort aspre & chaude, & au dedans il y a vn noyau blanc bon à manger, & qui ne cede rien en goust aux pinons de Syrie, couuert d'vne pellicule cendree qu'il faut oster. Les habitans du païs en vsent apres l'auoir legerement rosti, il est d'vn agreable goust & on croit qu'il excite Venus. On dit qu'il n'y a rien meilleur pour guerir les dartes que cette huile acre. C'est vne chose certaine que les Sauuages s'en seruent contre la gratelle. Le fameux Char. de l'Escluse a fait depeindre cette noix comme elle est ici representee entiere & coupee par le milieu.

En outre cet Autheur Portugais que i'ai suiui iusques ici [illegible] en la plus grande partie de ce que i'ai escrit, donne à cet arbre vne fleur rouge: & dit que le bois d'iceluy n'est pas mesme propre à brusler: mais qu'il rend vne gomme, dequoi les Peintres se seruent: & qu'on se sert de son escorce à teindre le fil de cotton, & à faire des vaisseaux de terre: & qu'estant bouillie dans de l'eau auec de la limaille de cuiure, & consommé iusques à la tierce partie, guerit les vlceres malins.

Il se trouue vne grande abondance d'arbres de *Mangaba* ou *Margaba* autour de la baye de *tous les Saincts*, & rarement ailleurs: Ils ont l'escorce de fouteau, & la fueille [illegible]

Woodcut of *A. occidentale* in *L'histoire du Nouveau Monde, ou, Description des Indes Occidentales*.by Johannes De Laet (1640: 492)

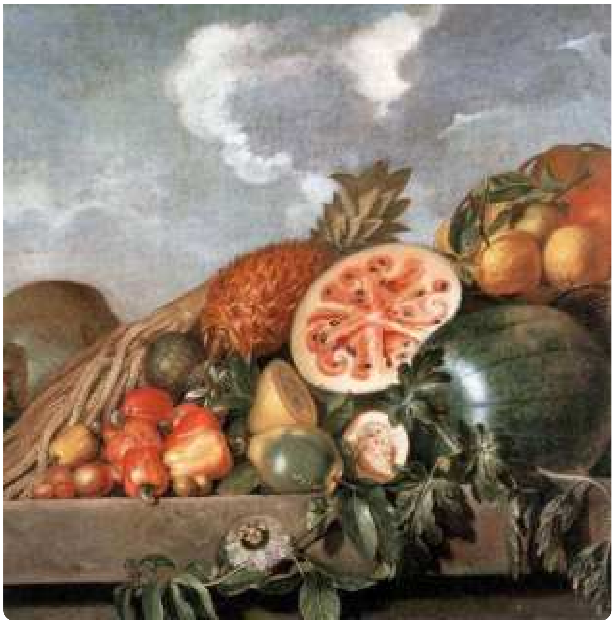

Ekchout's still-life with watermelon, pineapple and other fruits, ca. 1640. Copenhagen, National Museum of Denmark

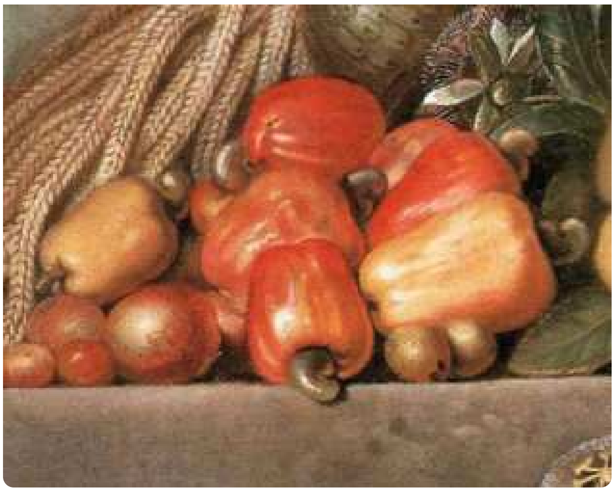

Close - up of the *A occidentale* fruit in Ekchout's still-life with watermelon, pineapple and other fruits, ca. 1640. Copenhagen, National Museum of Denmark

Historiae Rerum Naturalium Brasiliae	Marcgrave, 1648	Page number	96a

Vernacular name(s) Caa-opia

Species Vismia guianensis (Aubl.) Pers.

Family Hypericaceae

Notes

No resemblance between the woodcut and the specimen. The woodcut shows mostly a fruiting branch, while the specimen consists of a flowering one.

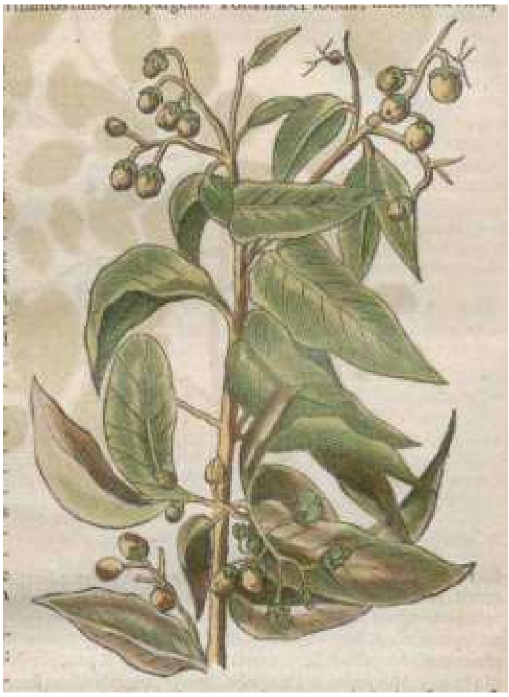

Historiae Plantarum – Arboribus: 96a

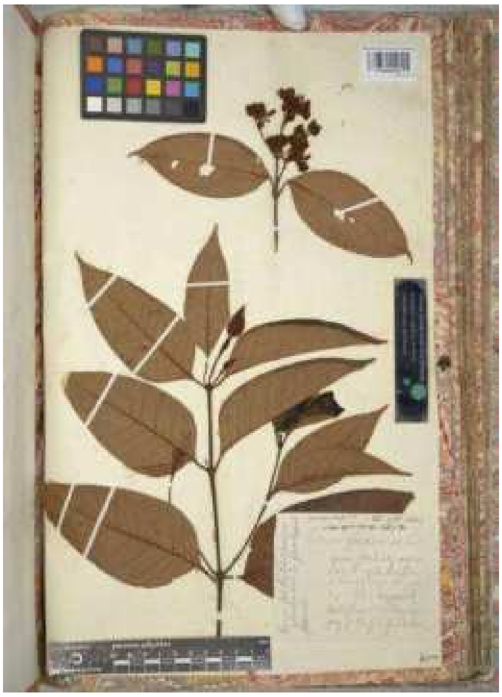

Marcgrave's herbarium: 157

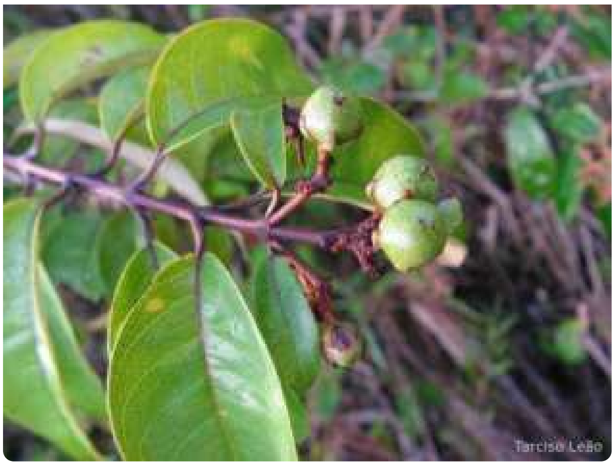

Fruiting branch. "*V. guianensis*, lacre" by Tarciso Leão (CC BY 2.0)

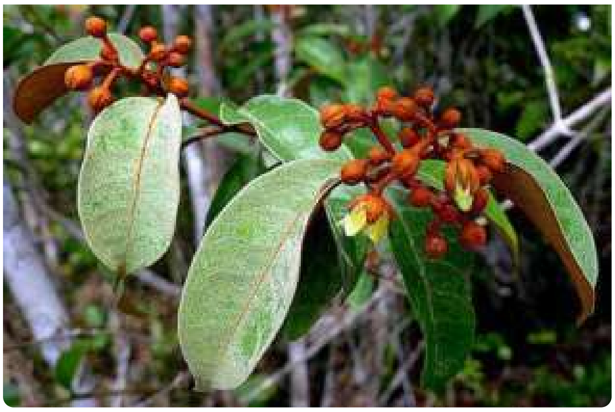

Flowering branch. "*V. guianensis*" by Alex Popovkin, Bahia, Brazil (CC BY-NC-SA 2.0)

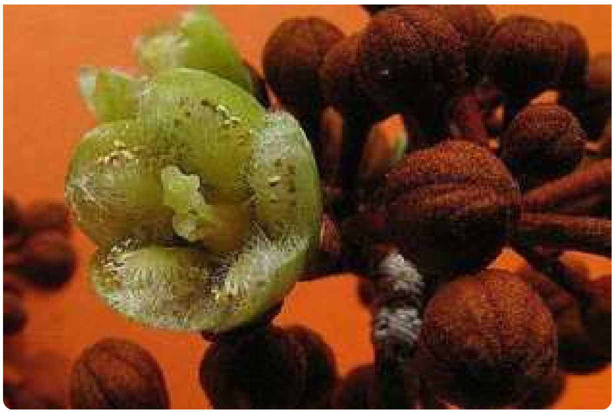

Open flower and flower buttons. "*V. guianensis*" by Alex Popovkin, Bahia, Brazil (CC BY-NC-SA 2.0)

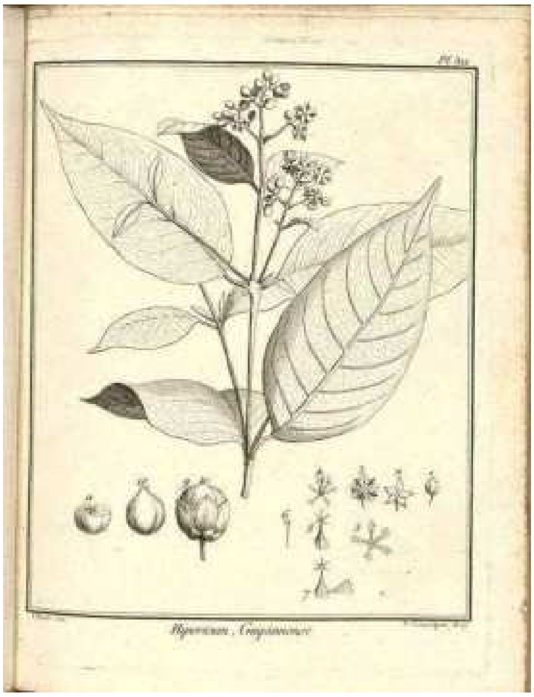

V. guianensis in *Histoire des plantes de la Guiane Françoise* by Aublet, J.B.C.F. (1775: Vol. IV, t. 311)

Historiae Rerum Naturalium Brasiliae	Marcgrave, 1648	Page number	96b

Vernacular name(s) Mundubiguacu

Species Jatropha curcas L.

Family Euphorbiaceae

Notes

The woodcut is different from the *Theatrum* image and the herbarium specimen, which only shows one leaf. The woodcut shows a weak image compared to the oil painting, in which flowers, fruits, and seeds are represented.

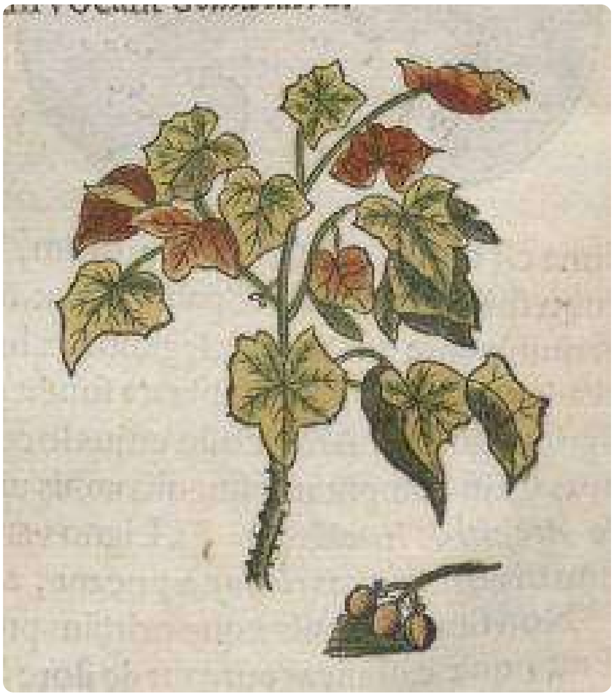

Historiae Plantarum – Arboribus: 96b

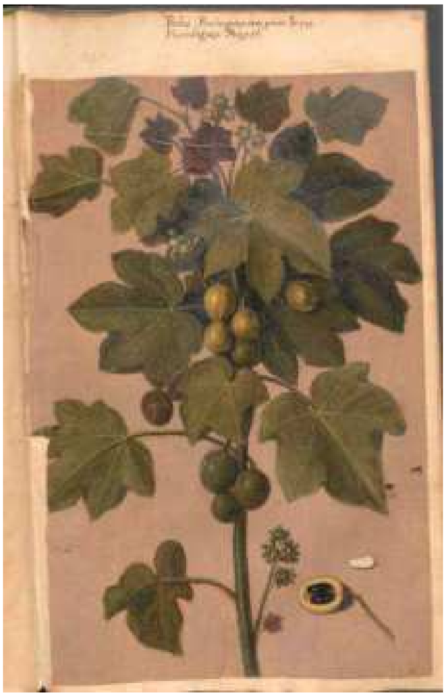

Theatrum Rerum Naturalium: 199

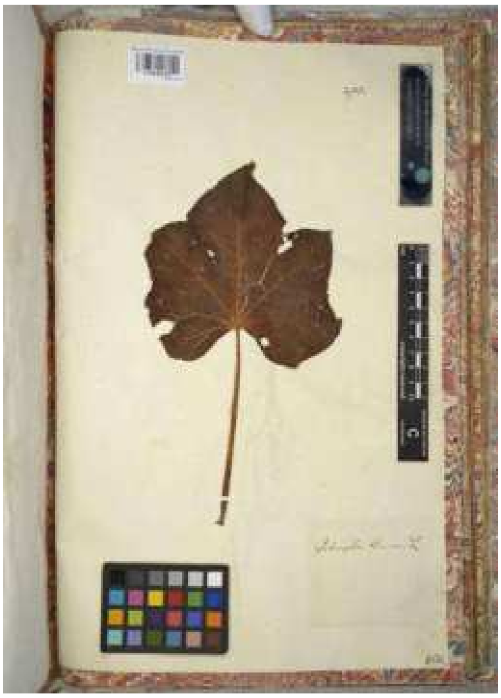

Marcgrave's herbarium: 165

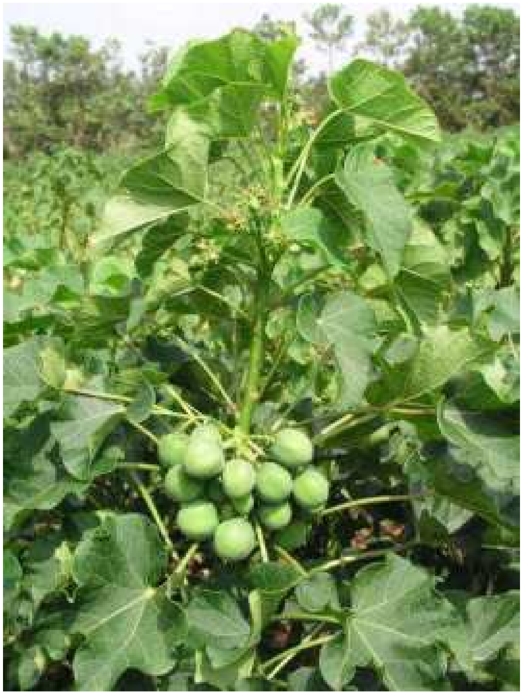

Fruits. "*J. curcas*" by wan_hong (CC BY-NC-SA 2.0)

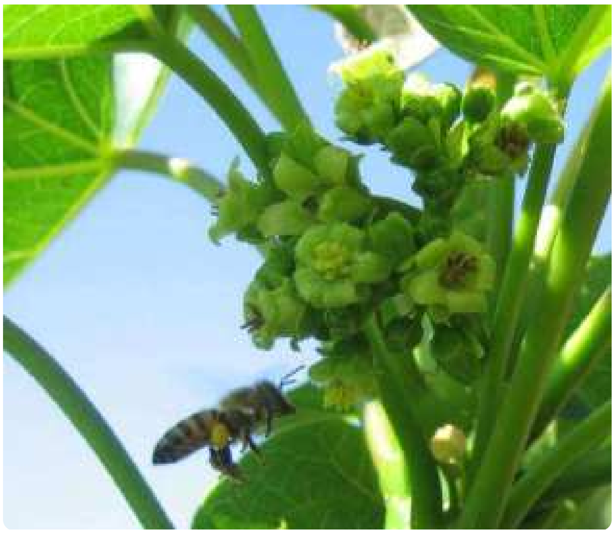

Flowers. "Bee pollinating *J- curcas*" by tonrulkens (CC BY-SA 2.0)

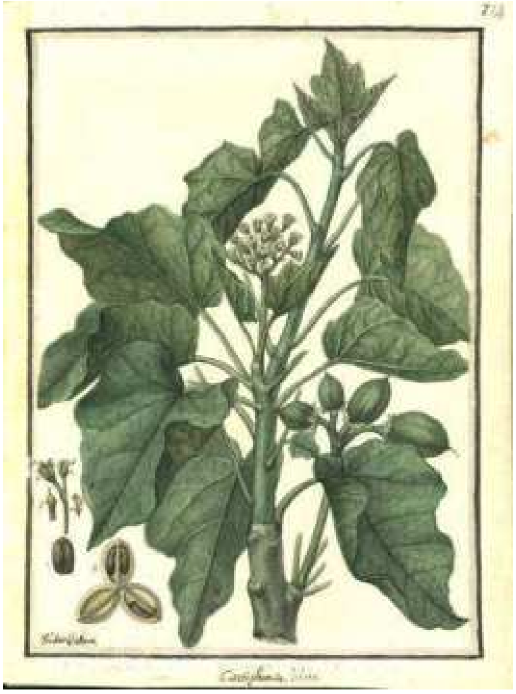

Drawings of the Royal Botanical Expedition to the Viceroyalty of Peru by Ruiz, H., Pavón, J. (1777). Real Jardín Botánico, Madrid, Spain

Historiae Rerum Naturalium Brasiliae Marcgrave, 1648 Page number 97

Vernacular name(s) Pinhones. Nux cathartica

Species Jatropha curcas L.

Family Euphorbiaceae

Notes

De Laet reused the woodcut he previously published in his books on the Americas (De Laet 1633, 1640).

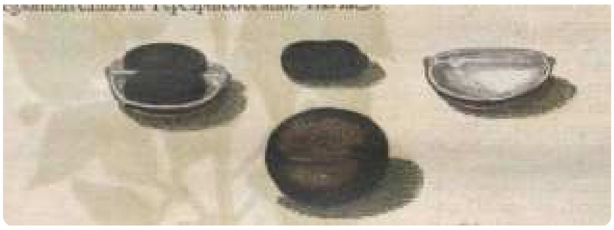

Historiae Plantarum – Arboribus: 97

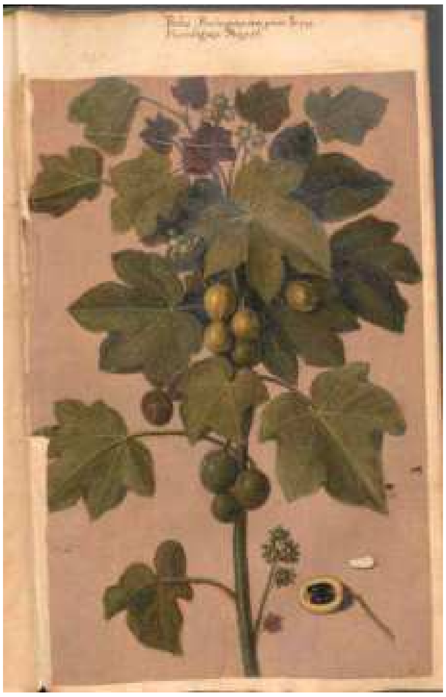

Theatrum Rerum Naturalium: 199

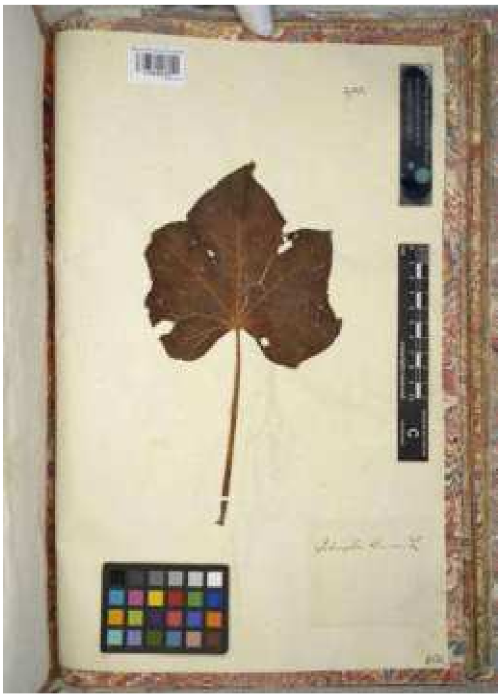

Marcgrave's herbarium: 165

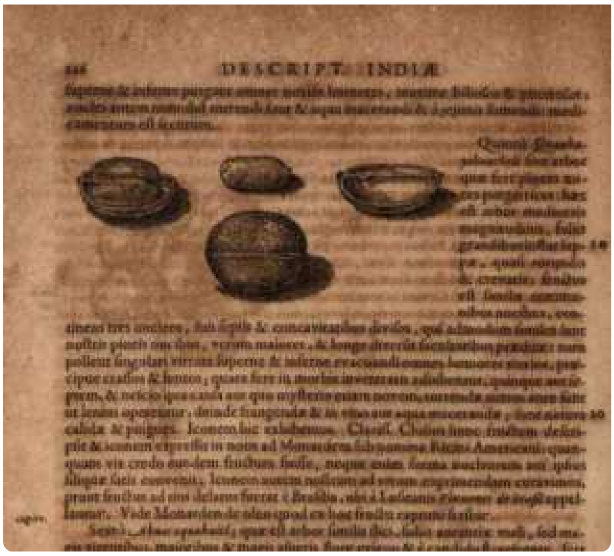

Woodcut of *J. curcas* seeds in *Novus Orbis seu descriptionis Indiae Occidentalis* by Johannes De Laet (1633: 226)

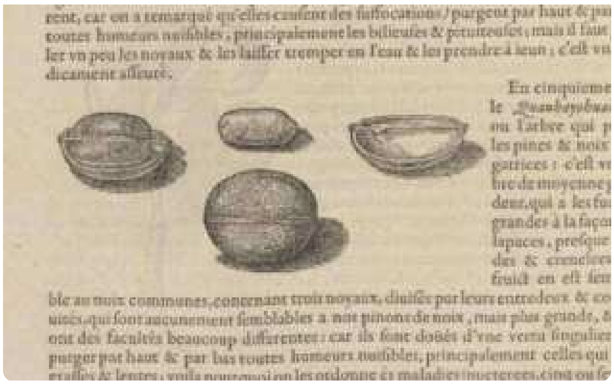

The same woodcut in *L'histoire du Nouveau Monde, ou, Description des Indes Occidentales*.by Johannes De Laet (1640: 136)

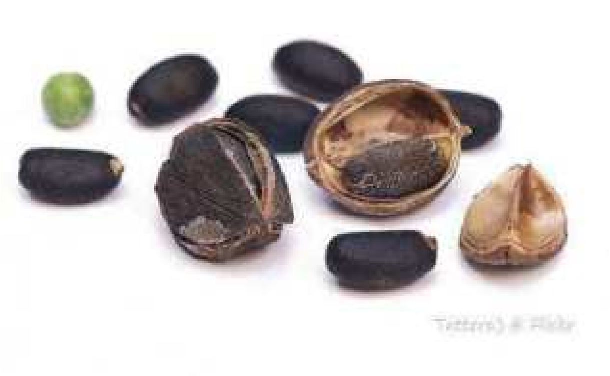

"Seeds of 'Physic nut' - *Jatropha curcas*)" by Tatters (CC BY-NC 2.0)

Historiae Rerum Naturalium Brasiliae Marcgrave, 1648 Page number 98a

Vernacular name(s) Tapia

Species Crateva tapia L.

Family Capparaceae

Notes

The woodcut looks moderately similar to the illustrations in the *Theatrum*. The image is a composite, in which flowers and fruits are mixed (based on the oil paintings or in a model used to elaborate them).

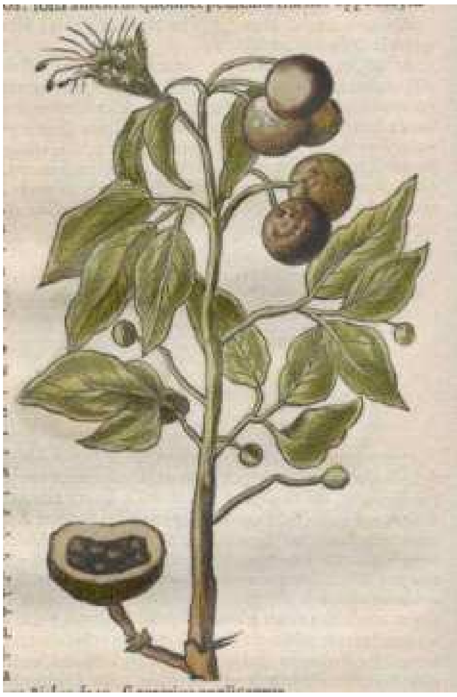

Historiae Plantarum – Arboribus: 98a

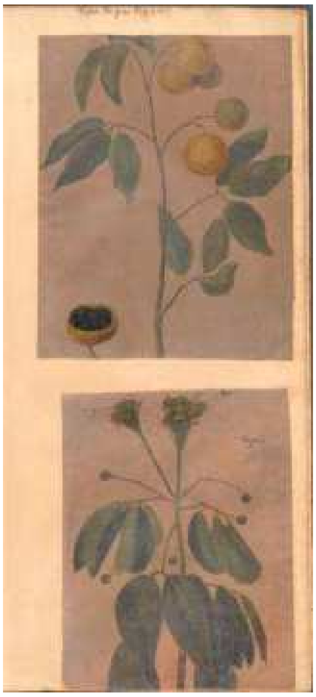

Theatrum Rerum Naturalium: 113

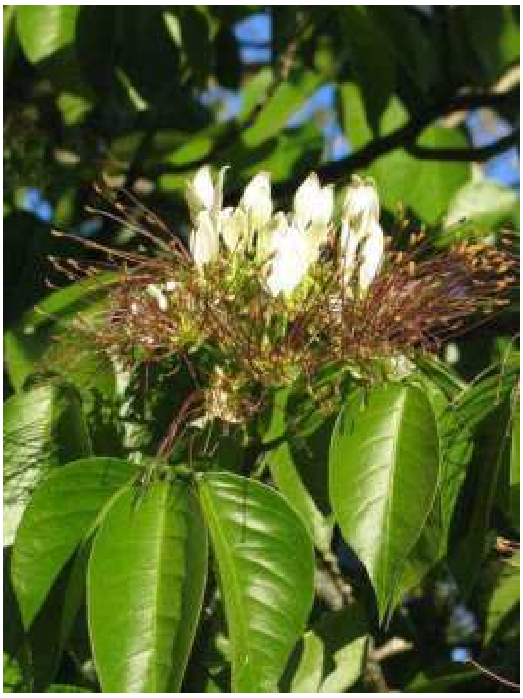

Flowering branch. "TRAPIÁ *C. tapia*" by antoniosergio25 (CC BY-NC-SA 2.0)

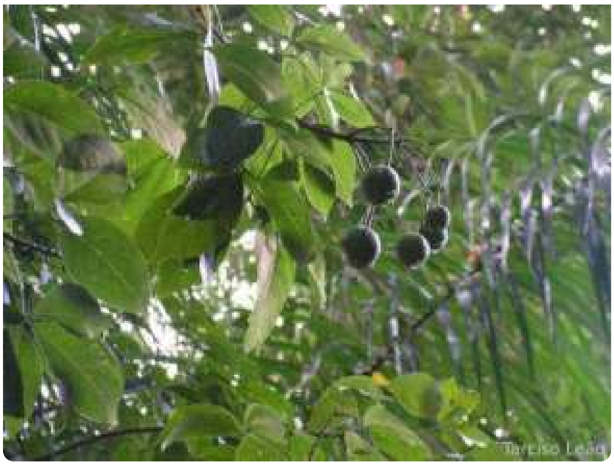

Fruiting branch. "*Crateva tapia*, trapiá" by Tarciso Leão (CC BY 2.0)

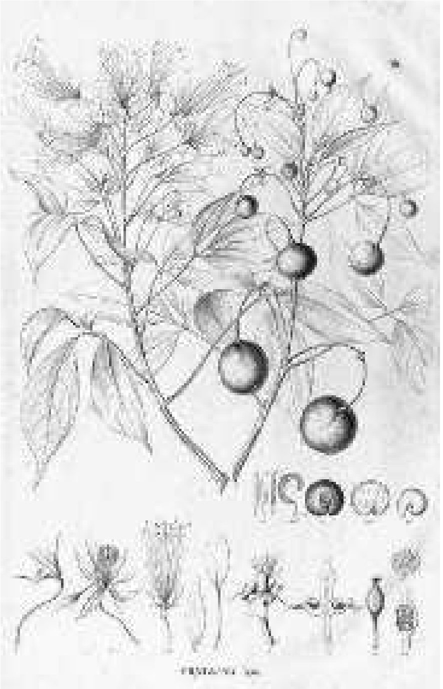

Engraving of *C. tapia* in Martius, C.F.P. von, Eichler, A.G., Urban, I., *Flora Brasiliensis* (1841-1872) Vol. 13 (1): 59

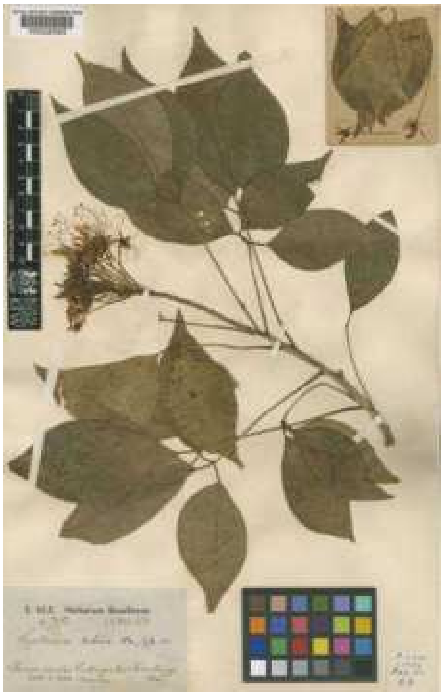

Specimen of *C. tapia* from Kew's Herbarium - K000220563. Retrieved from Plants of the World Online

Historiae Rerum Naturalium Brasiliae	Marcgrave, 1648	Page number	98b

Vernacular name(s) Icicariba

Species Protium heptaphyllum (Aubl.) Marchand

Family Burseraceae

Notes

We did not find any correspondence between this woodcut and the contemporary or older sources.

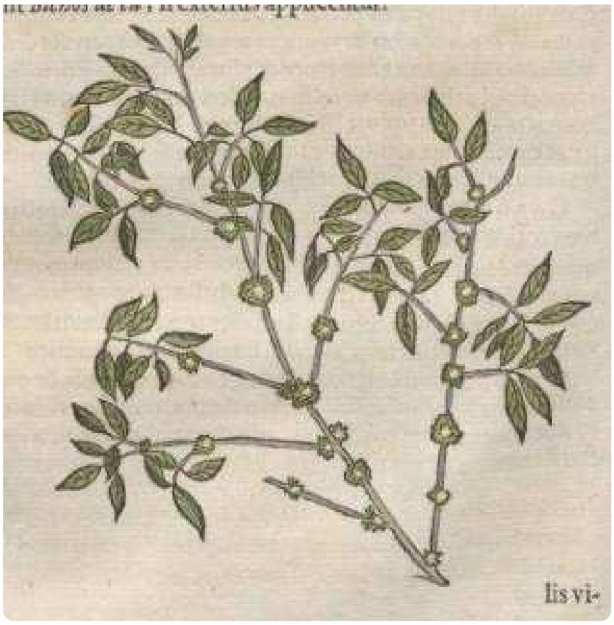

Historiae Plantarum – Arboribus: 98b

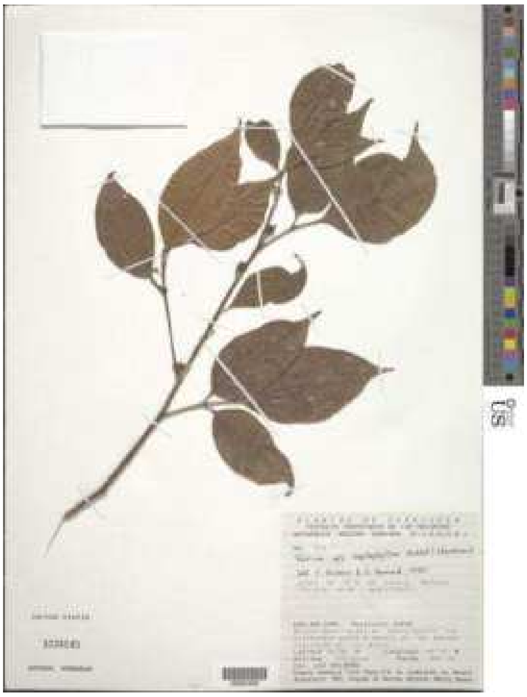

Specimen. "*Protium heptaphyllum* by L. Delgado V. -00892462- Smithsonian National Museum of Natural History (CC0 1.0)

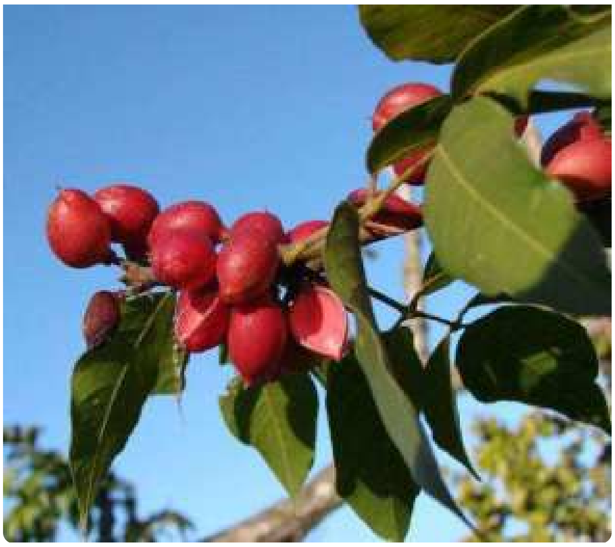

Fruiting branch. "*P. heptaphyllum*" by João de Deus Medeiros (CC BY 2.0)

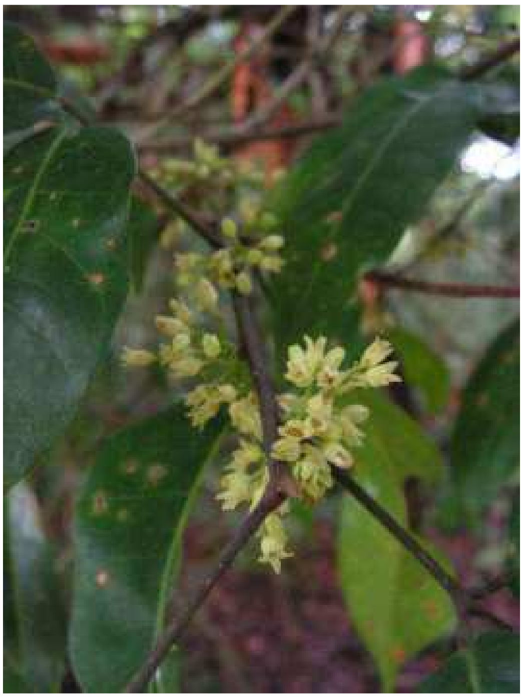

Flowering branch. "*P. heptaphyllum*, amescla" by Tarciso Leão (CC BY 2.0)

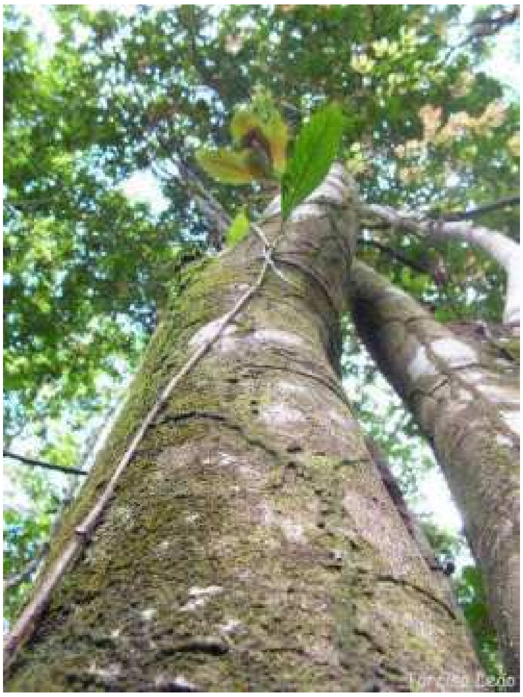

Habit. "*P. heptaphyllum*, amescla-de-cheiro" by Tarciso Leão (CC BY 2.0)

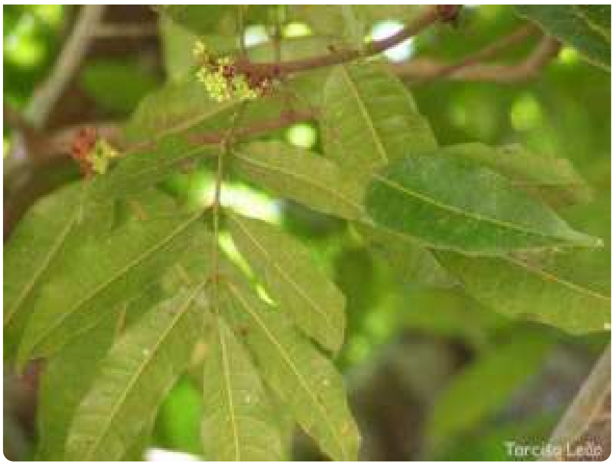

"*P. heptaphyllum*, amescla-de-cheiro" by Tarciso Leão (CC BY 2.0)

Historiae Rerum Naturalium Brasiliae Marcgrave, 1648 Page number 99

Vernacular name(s) Ibira

Species Xylopia frutescens Aubl.

Family Annonaceae

Notes

The woodcut is very similar to the *Theatrum* image (non-reversed). The same species is documented in Piso (1648: 71), but the image is different and it does not resemble our contemporary or older sources.

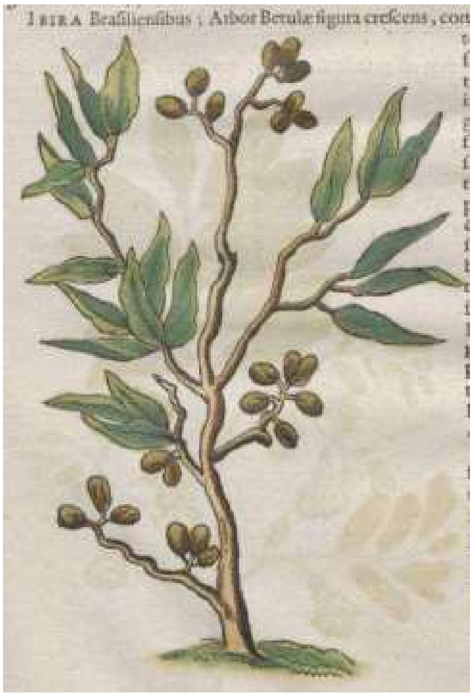

Historiae Plantarum – Arboribus: 99

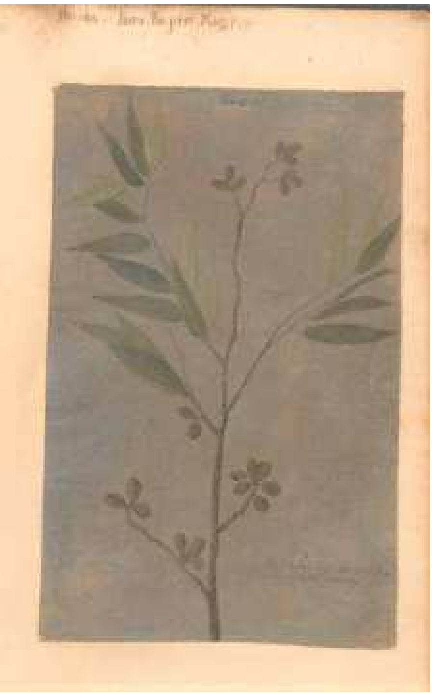

Theatrum Rerum Naturalium: 131

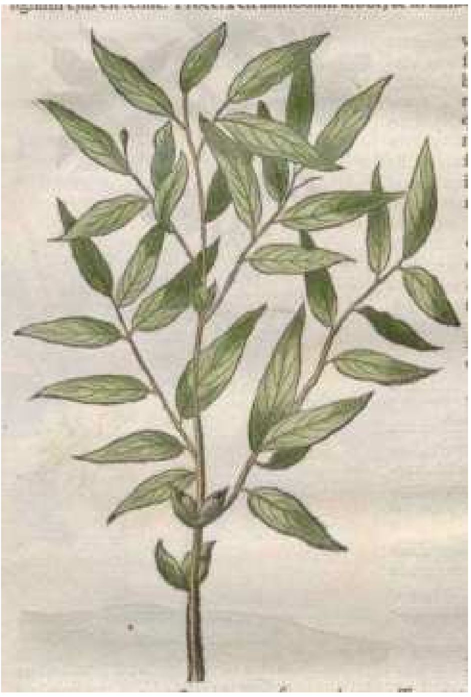

Another woodcut image of *X. frutescens* in the HNB (Piso (1648: 71)

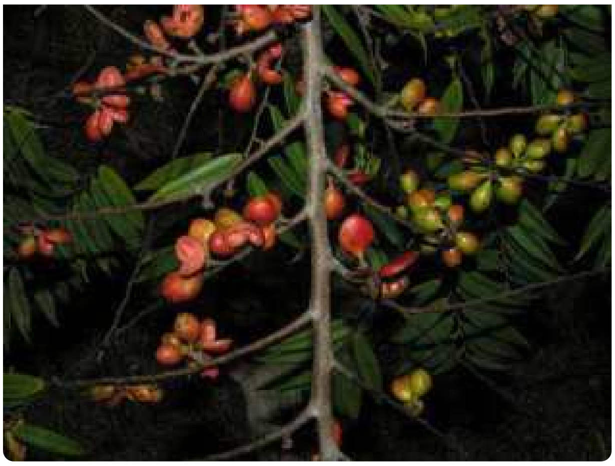

Fruiting branch. "*X. frutescens*" by Reinaldo Aguilar (CC BY-NC-SA 2.0)

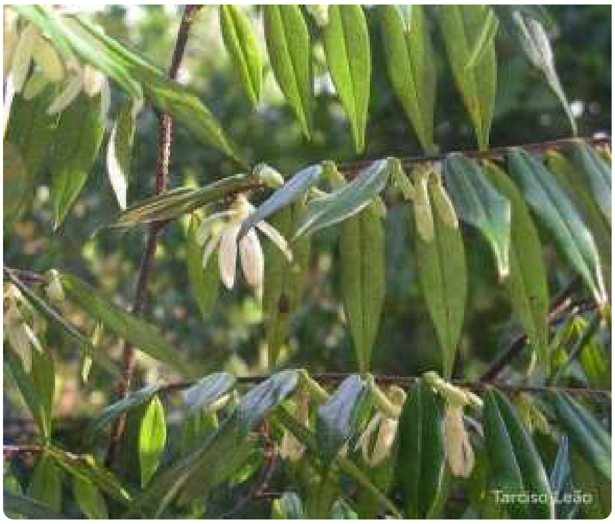

Flowering branch. "*X. frutescens*, embira-vermelha" by Tarciso Leão (CC BY 2.0)

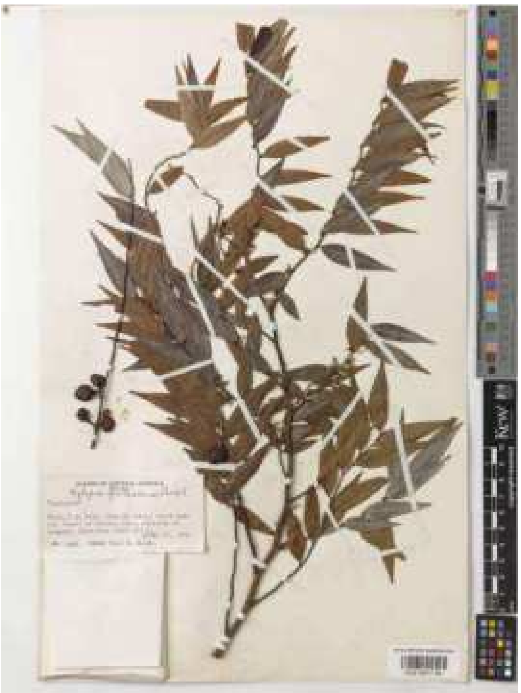

Specimen of *X. frutescens* from Kew's Herbarium - K001501749. Retrieved from Plants of the World Online

Historiae Rerum Naturalium Brasiliae Marcgrave, 1648 Page number 100

Vernacular name(s) Cebipira

Species Bowdichia virgilioides Kunth

Family Fabaceae

Notes

The woodcut differs from the *Theatrum* image. A flowering branch is depicted in the HNB, while the illustration is based on a fruiting branch. According to Marcgrave (1648: 100), this tree flowered from October to November. Hence, this branch was collected during that season.

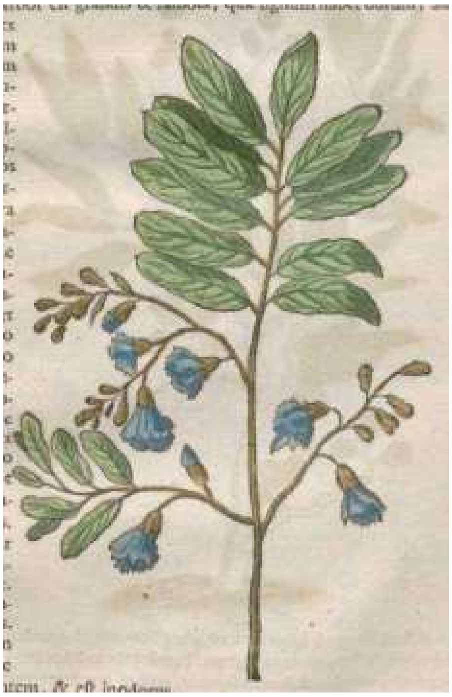

Historiae Plantarum – Arboribus: 100

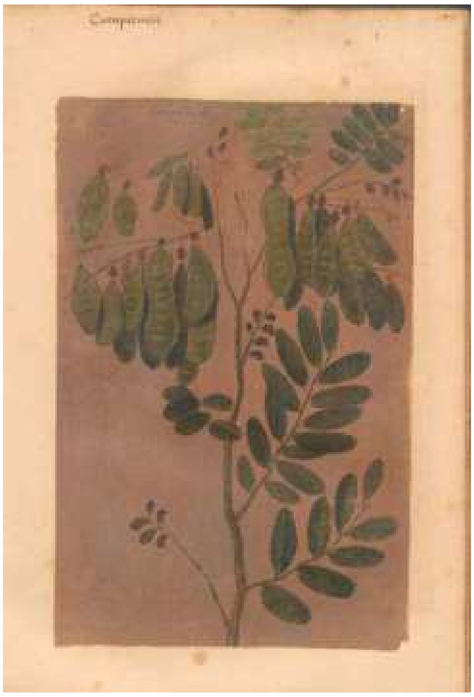

Theatrum Rerum Naturalium: 415

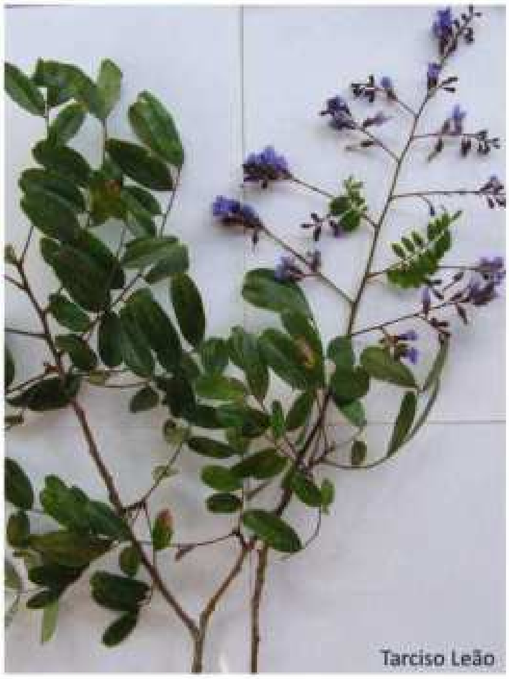

Flowering branch. "*B. virgilioides*, sucupira-mirim" by Tarciso Leão (CC BY 2.0)

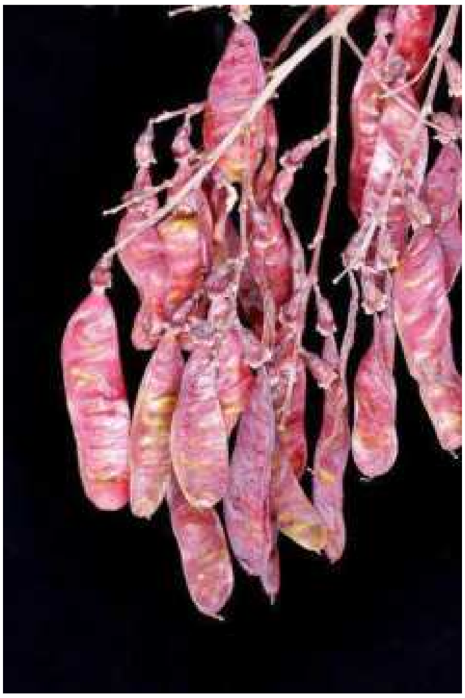

Fruiting branch. "*B. virgilioides*" by Mauricio Mercadante (CC BY-NC-SA 2.0)

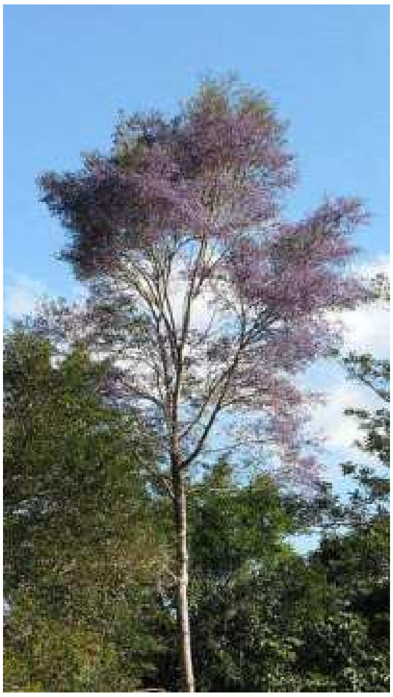

Habit. "*B. virgilioides*" by Alex Popovkin, Bahia, Brazil (CC BY-NC-SA 2.0)

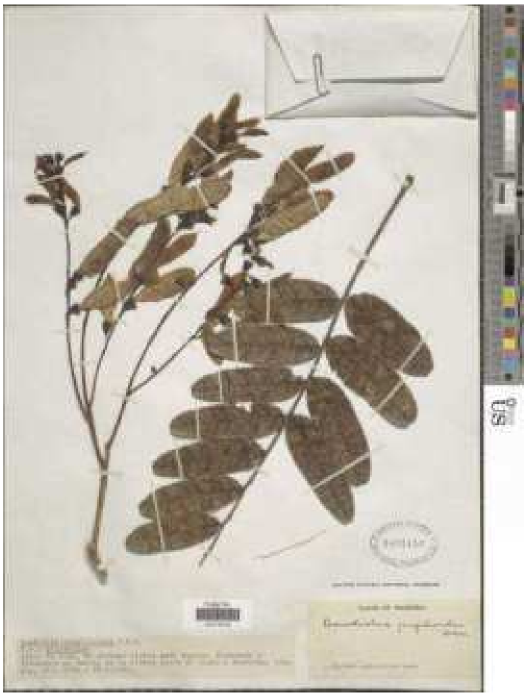

Specimen. "*B. virgilioides*" by Lewelyn Williams -02273578-Smithsonian National Museum of Natural History (CC0 1.0)

Historiae Rerum Naturalium Brasiliae	Marcgrave, 1648	Page number	101a

Vernacular name(s) Jabotapita

Species Ouratea caudata Engl.

Family Ochnaceae

Notes

We did not find any correspondence between this woodcut and the contemporary or older sources.

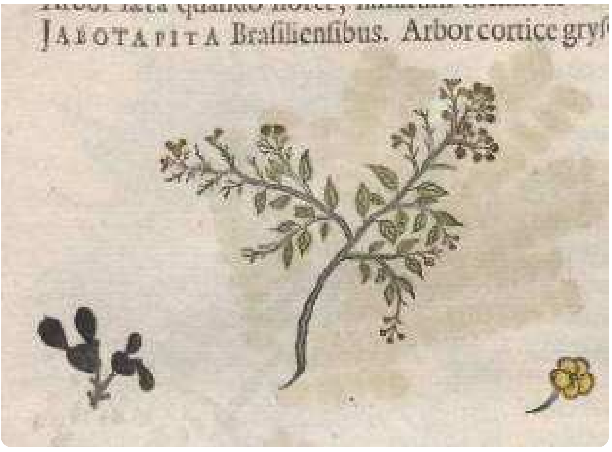

Historiae Plantarum – Arboribus: 101

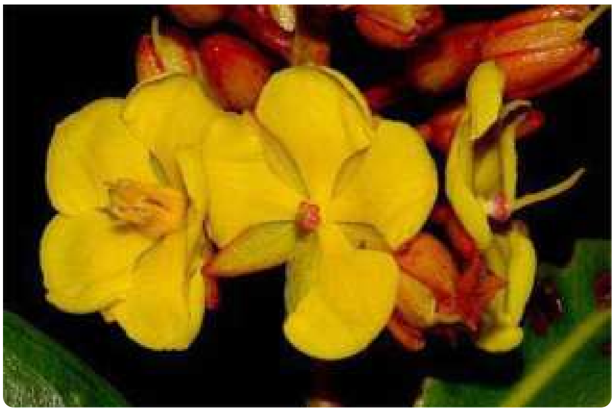

"Flowers showing detail of ovaries of *Ouratea caudata" by* Cláudio Nicoletti de Fraga, Instituto de Pesquisas Jardim Botânico do Rio de Janeiro

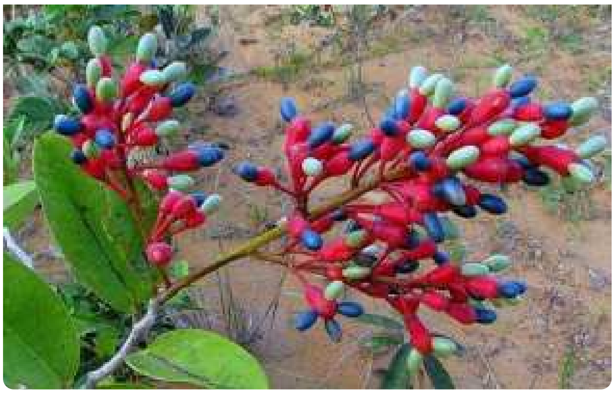

"*Ouratea* sp., Ochnaceae, Atlantic forest, northern littoral of Bahia, Brazil" by Alex Popovkin, Bahia, Brazil (CC BY-NC-SA 2.0)

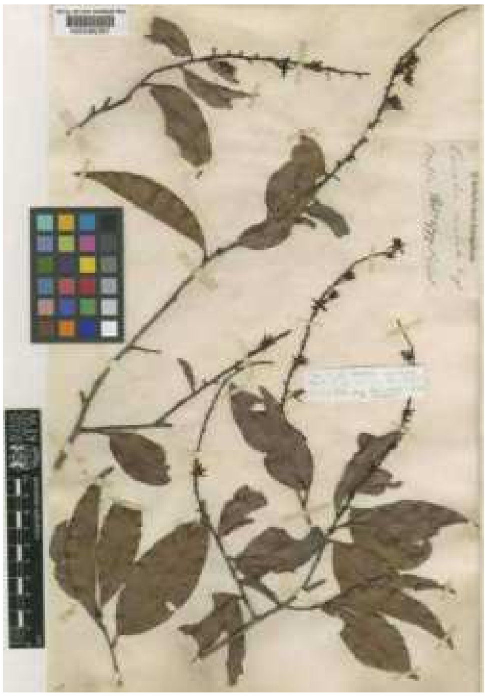

Specimen of *O. caudata* from Kew's Herbarium - K000382067. Retrieved from Plants of the World Online

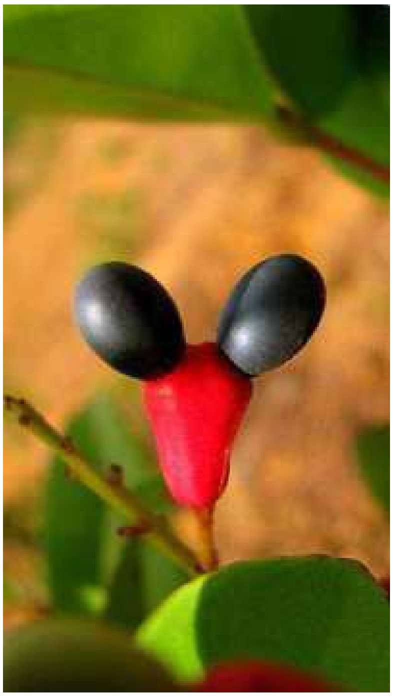

"*Ouratea* sp., Ochnaceae, Atlantic forest, northern littoral of Bahia, Brazil" by Alex Popovkin, Bahia, Brazil (CC BY-NC-SA 2.0)

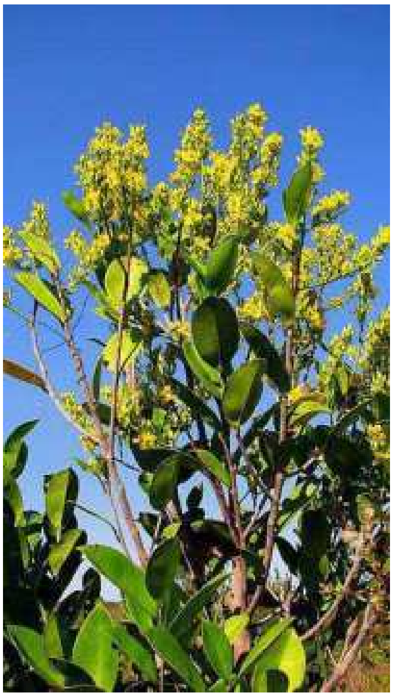

"*Ouratea* sp., Ochnaceae, Atlantic forest, northern littoral of Bahia, Brazil" by Alex Popovkin, Bahia, Brazil (CC BY-NC-SA 2.0)

Historiae Rerum Naturalium Brasiliae Marcgrave, 1648 Page number 101b

Vernacular name(s) Jetaiba

Species Hymenaea cf. courbaril L.

Family Fabaceae

Notes

We consider the correspondence slightly similar between the woodcut and the illustration. Although the tree was not drawn, the fruit in the oil painting bears strong resemblance to the woodcut. De Laet, or the same Marcgrave, indicated that they owed this figure [woodcut] to Piso (Marcgrave 1648: 101).

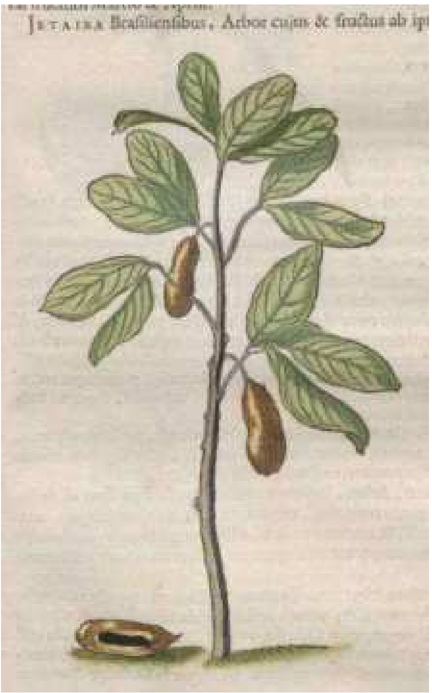

Historiae Plantarum – Arboribus: 101b

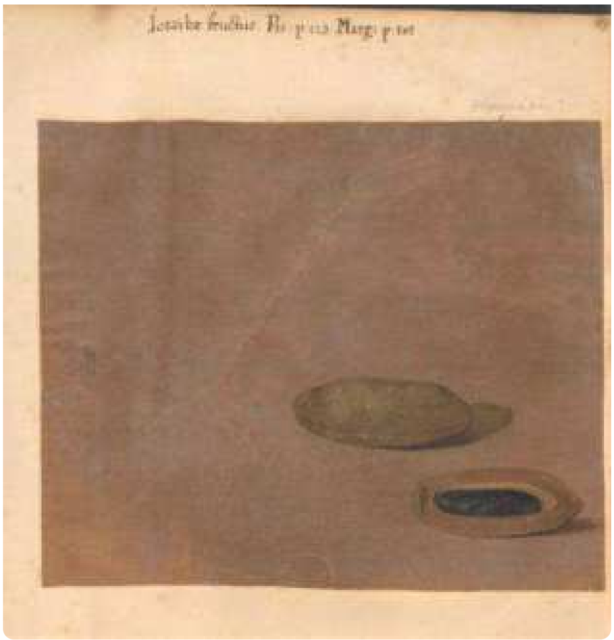

Theatrum Rerum Naturalium: 69

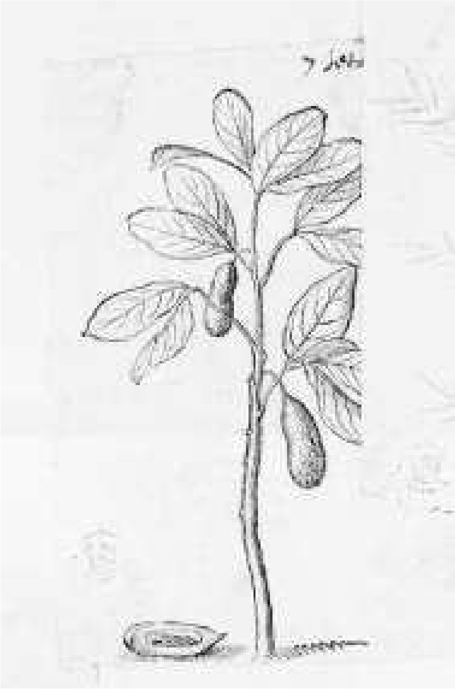

Proof woodcut of *H. courbaril* n De Laet's manuscript: Sloane MS 1554, f. 77v

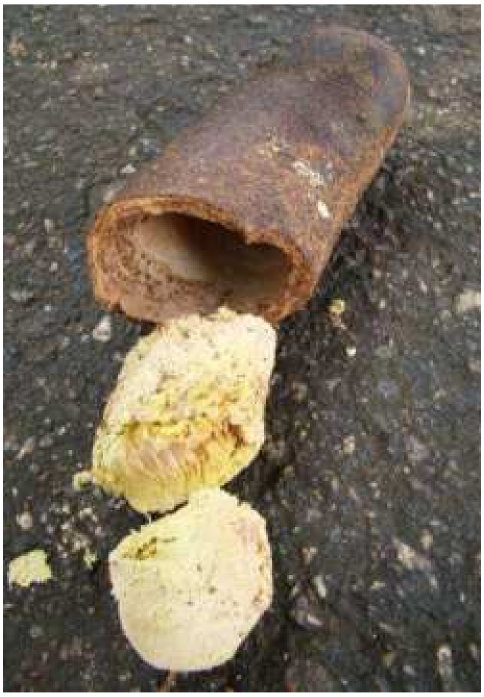

"Jatobá (*H. courbaril*) Brazilian; legume about 18 cm and 200 grs." by mauro halpern (CC BY 2.0)

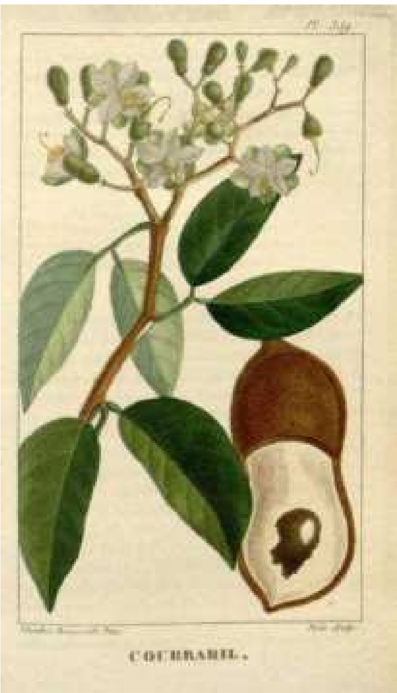

Flore [pittoresque et] médicale des Antilles by Descourtilz, M.E. (1827: Vol. V, t. 359). Missouri Botanical Garden, St. Louis, U.S.A.

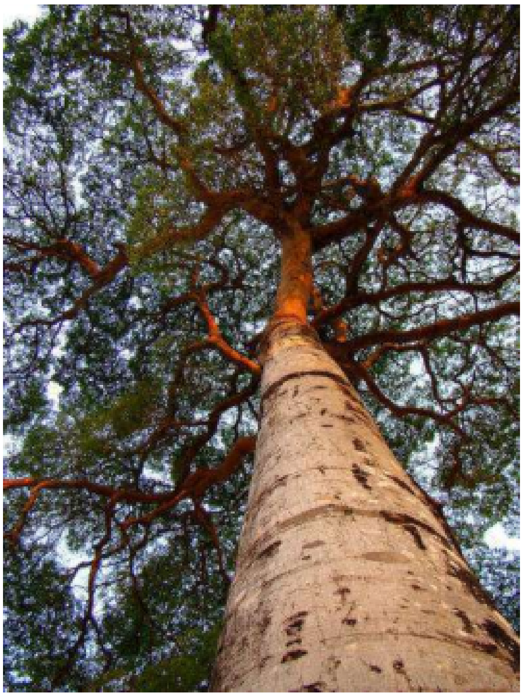

"Jatobá '*H. courbaril* Brazil-cherry" by mauro halpern (CC BY 2.0)

Historiae Rerum Naturalium Brasiliae Marcgrave, 1648 Page number 102

Vernacular name(s) Ibirapitanga. Pao Brasil

Species Paubrasilia echinata (Lam.) Gagnon, H.C.Lima & G.P.Lewis

Family Fabaceae

Notes

We did not find any correspondence between this woodcut and the contemporary or older sources.

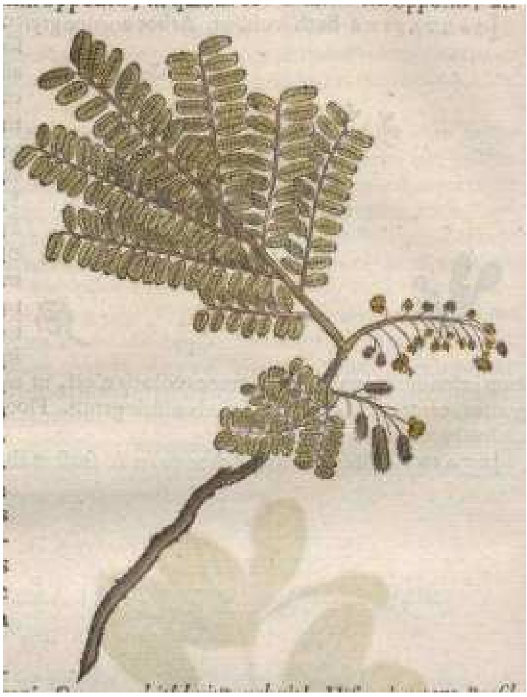

Historiae Plantarum – Arboribus: 102

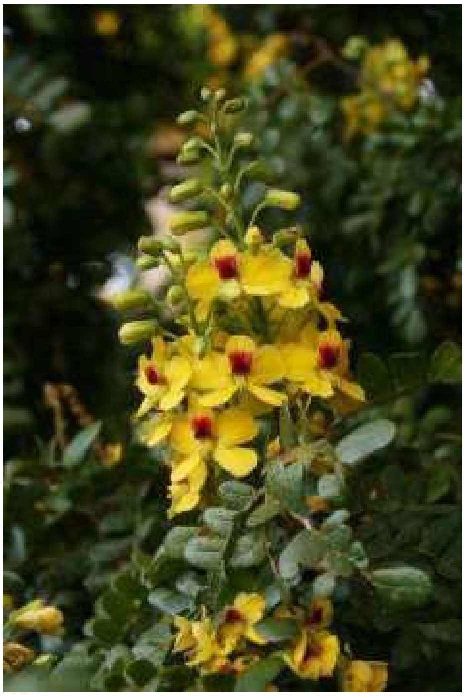

Flowering branch. "*P. echinata*" by Mauricio Mercadante (CC BY-NC-SA 2.0)

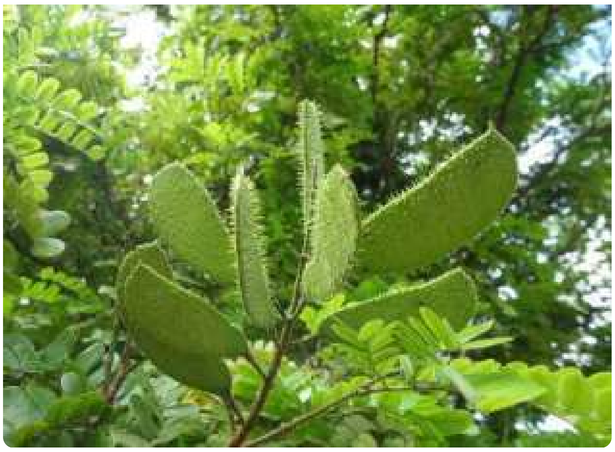

Fruiting branch. "Brazilwood / Pernambuco / pau-brasil '*P. echinata*'" by mauro halpern (CC BY 2.0)

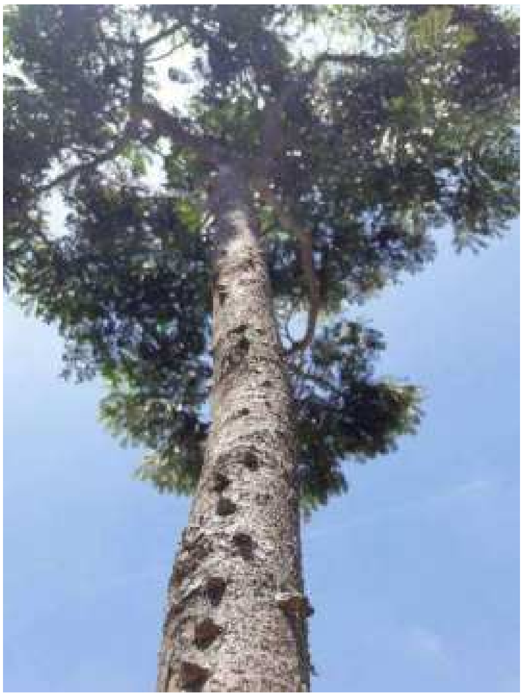

Habit. "Brazilwood / Pernambuco / pau-brasil '*P. echinata*'" by mauro halpern (CC BY 2.0)

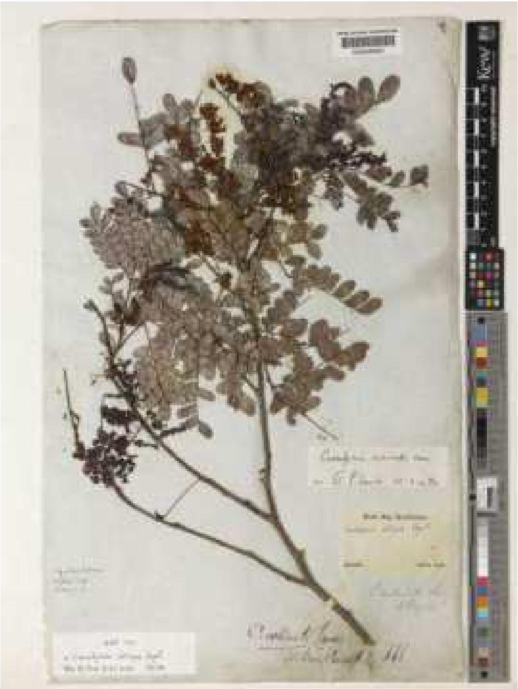

Specimen of *P. echinata* from Kew's Herbarium - K000680625. Retrieved from Plants of the World Online

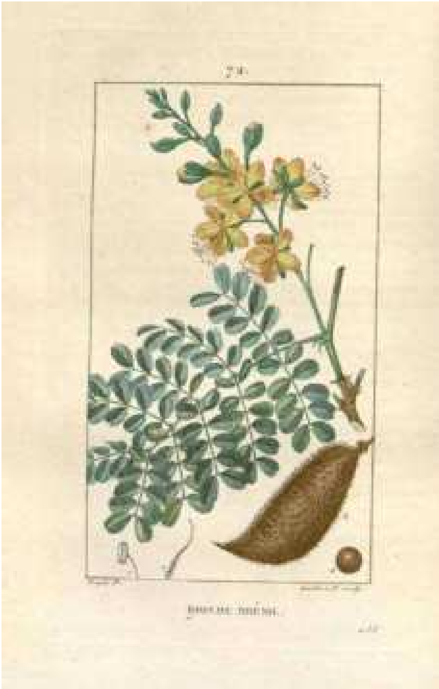

P. echinata in *Flore médicale* by Chaumeton, F.P. (1829: Vol. II, t. 72). Missouri Botanical Garden, St. Louis, U.S.A.

Historiae Rerum Naturalium Brasiliae	Marcgrave, 1648	Page number	103a

Vernacular name(s) Mamaoeira. Papay. Mamao

Species Carica papaya L.

Family Caricaceae

Notes

There is no correlation between the woodcut and the other sources. The specimen in the herbarium (p. 44) shows a single leaf and one female flower and male inflorescence - a part of the latter are mixed with the specimen of *Desmodium incanum* (p. 93). Despite the multiple sources where the Papaya fruit is depicted, De Laet took the woodcut that was used in Clusius (1605: 42, 1611: 79) called "Mamoeira foemina", which in turn, he previously used in his book on the Americas.

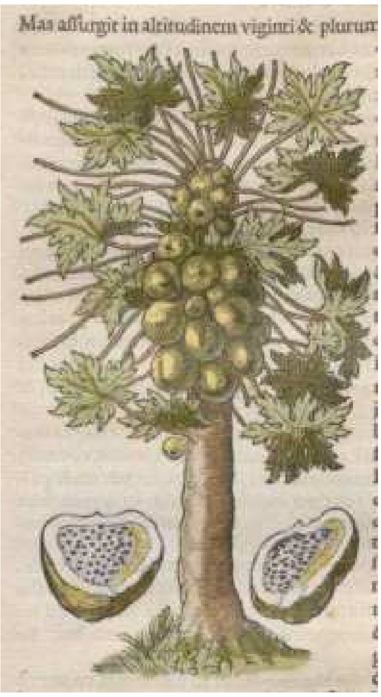

Historiae Plantarum – Arboribus: 103a

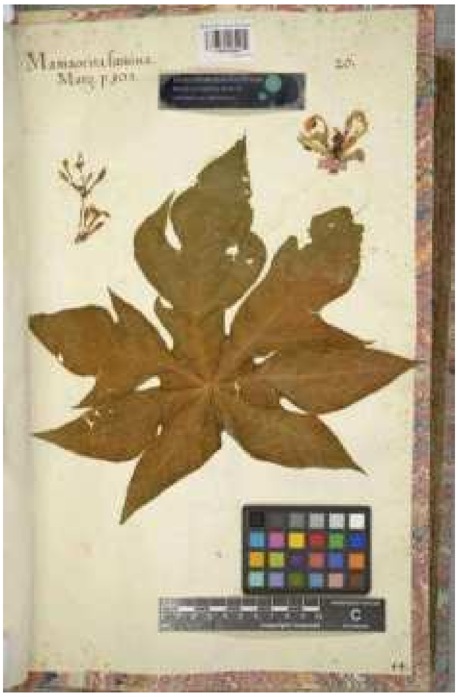

Marcgrave's herbarium: 44

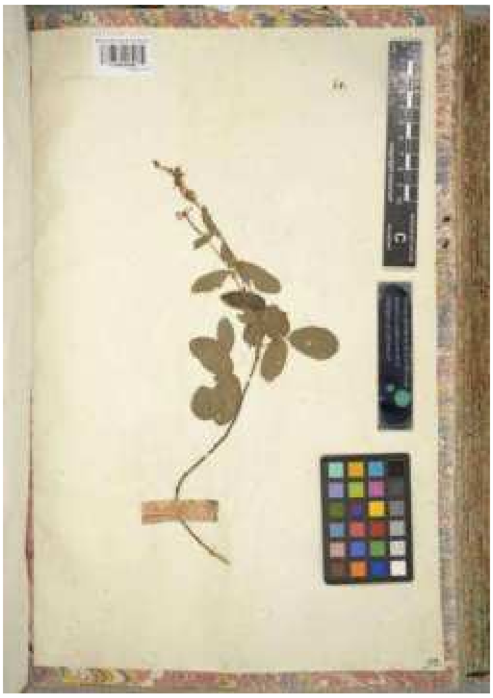

Marcgrave's herbarium: 93 Male flower of *C. papaya* attached to the stem of *Desmodium incanum*

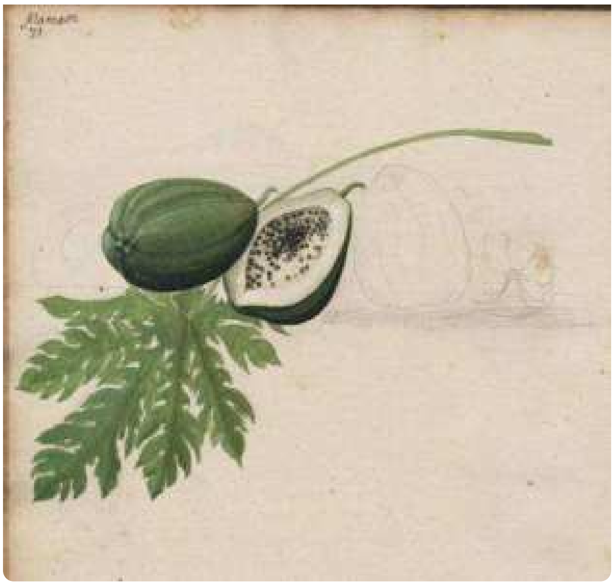

Libri Principis: f. 71

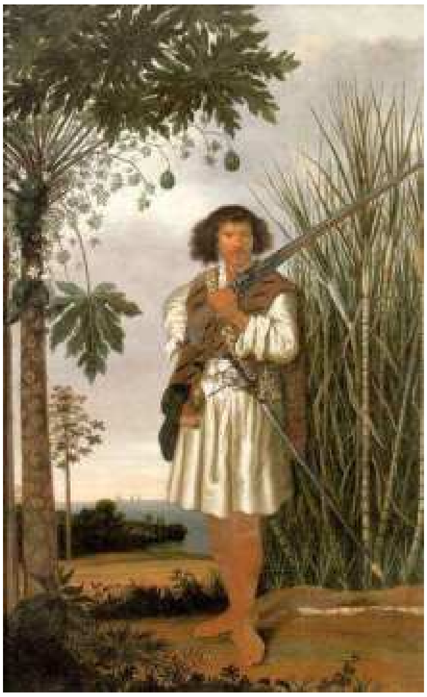

Ekchout's portrait "Biracial man with riffle and sword", ca. 1641. Copenhagen, National Museum of Denmark

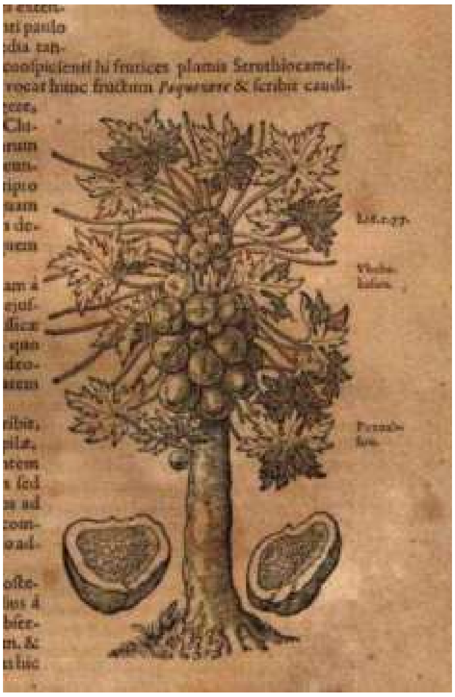

Woodcut of *C. papaya* in *Novus Orbis seu descriptionis Indiae Occidentalis* by Johannes De Laet (1633: 563) taken from Clusius (1605, 1611)

Historiae Rerum Naturalium Brasiliae | Marcgrave, 1648 | Page number 103b

Vernacular name(s) Mamaoeira foemina

Species Carica papaya L.

Family Caricaceae

Notes

Although there are many images of the Papaya in several sources, the image of this woodcut -which represents a female flower- does not correspond with any of those sources. The woodcut, however, could be based on the flower specimen, before this was dissected and prepared as an exsicata.

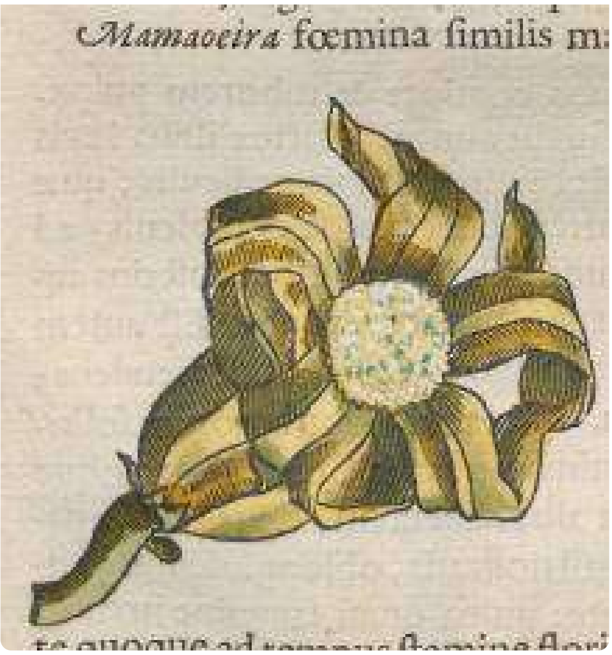

Historiae Plantarum – Arboribus: 103b

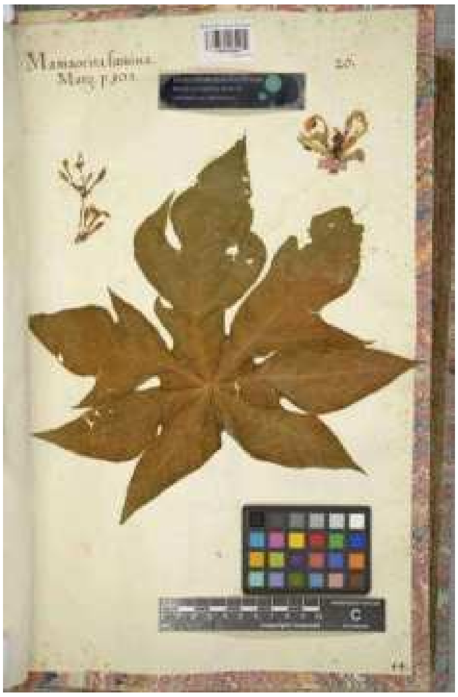

Marcgrave's herbarium: 44

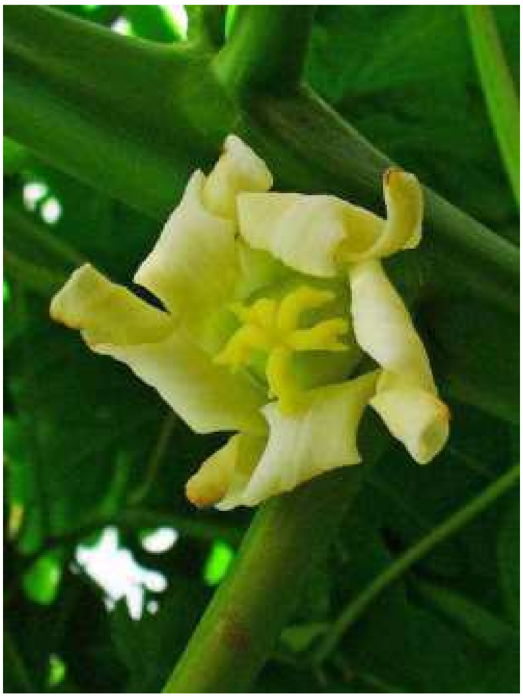

Female flower. "*C. papaya* 003.JPG" by H. Zell (CC BY-SA 3.0)

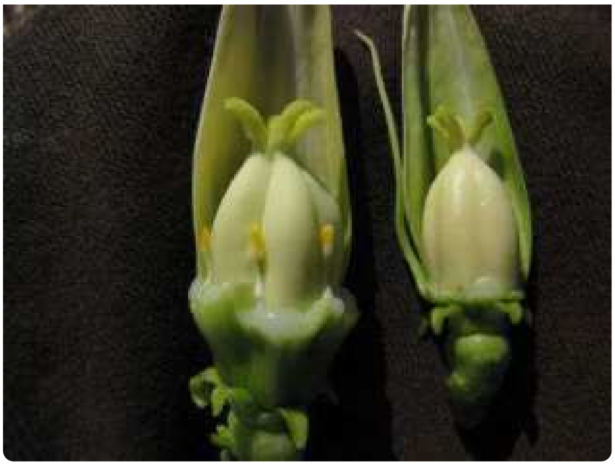

"*C. papaya*:female flower on the right, hermaphrodite flower on the left -Hawea Pl Olinda Maui" by Starr Environmental (CC BY 2.0)

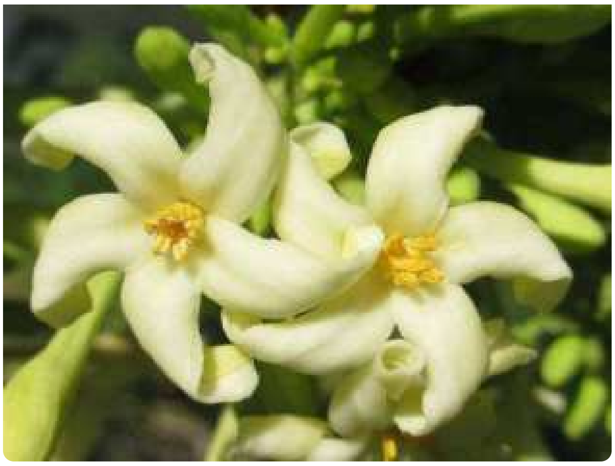

"*C. papaya* - male flowers" by tonrulkens (CC BY-SA 2.0)

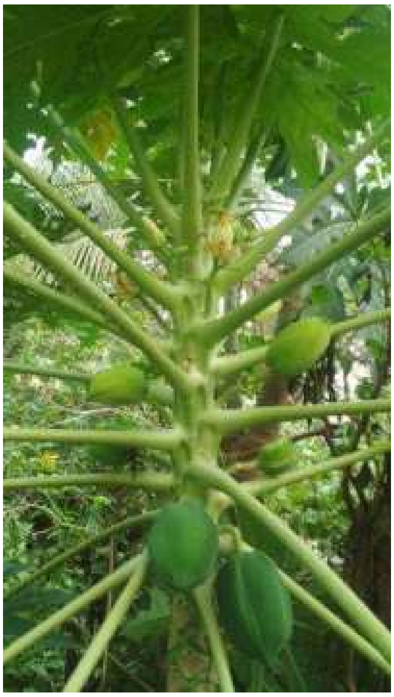

"Papaya, *C. papaya* Female tree" by Dr. Raju Kasambe (CC BY-SA 4.0)

Historiae Rerum Naturalium Brasiliae	Marcgrave, 1648	Page number	105

Vernacular name(s) Araca-iba

Species Psidium cattleyanum Sabine

Family Myrtaceae

Notes

We did not find any correspondence between this woodcut and the contemporary or older sources.

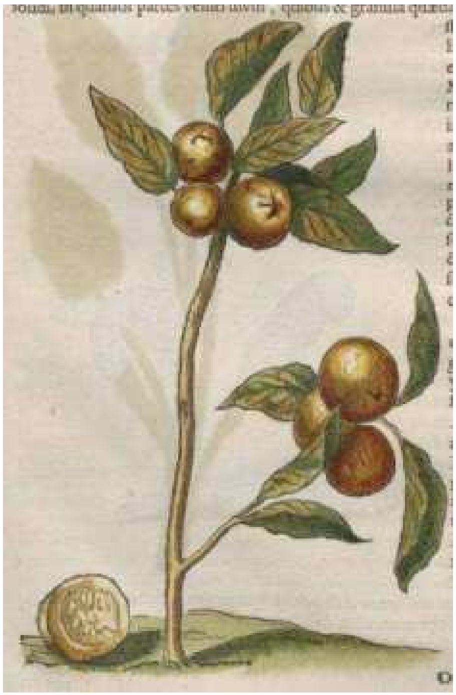

Historiae Plantarum – Arboribus: 105

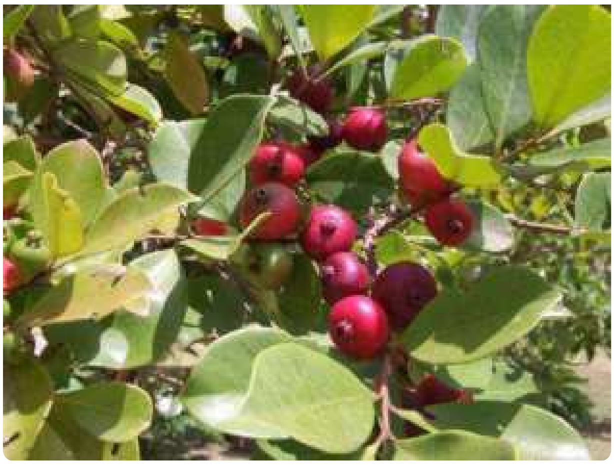

"*Psidium cattleyanum* fruits" by B.navez (CC BY-SA 3.0)

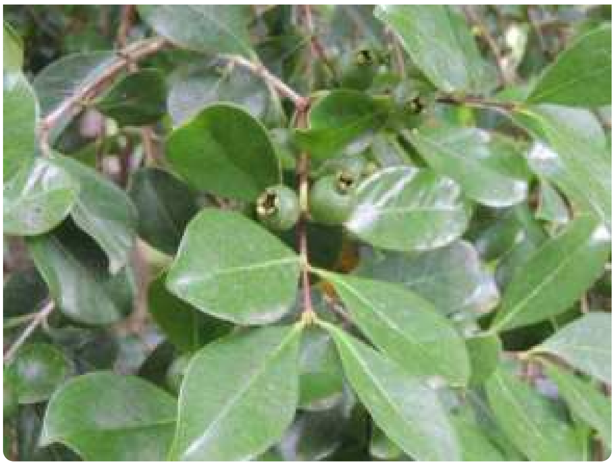

"*P. cattleyanum*, Myrtaceae. 'Guayabo fresa'." by chnelsons (Public Domain)

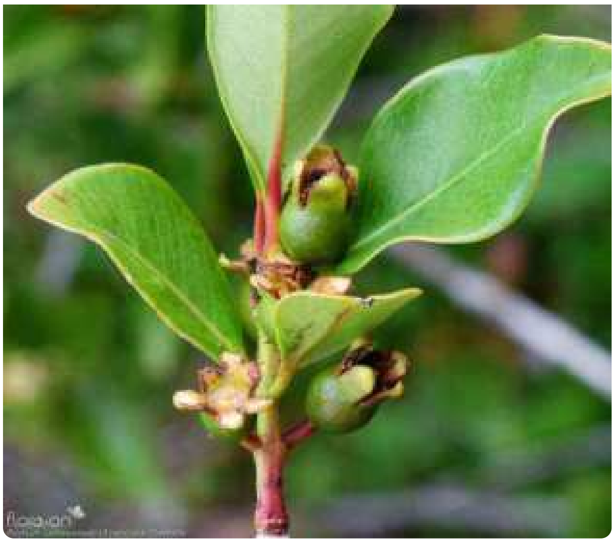

"*P. cattleyanum*" by Francisco Clamote (CC BY-NC 4.0)

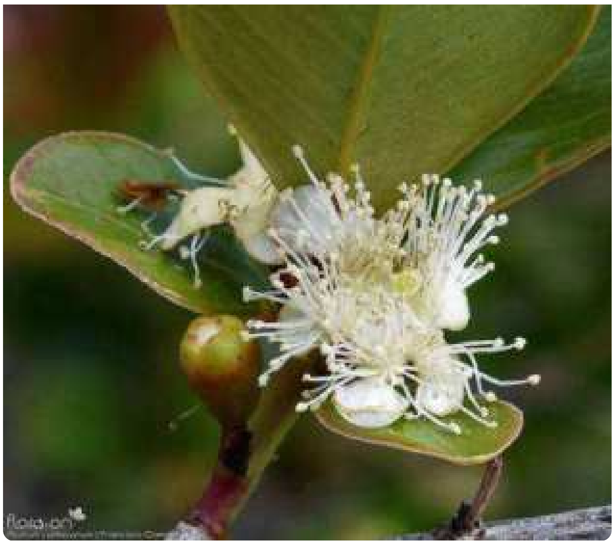

Flowers. "*P. cattleyanum*" by Francisco Clamote (CC BY-NC 4.0)

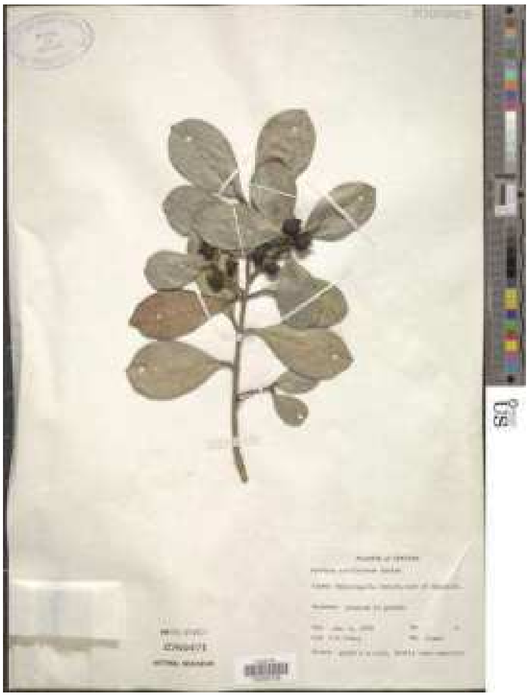

Specimen. "*P. cattleyanum*" by F. R. Fosberg -3050728- Smithsonian National Museum of Natural History (CC0 1.0)

Historiae Rerum Naturalium Brasiliae — Marcgrave, 1648 — Page number 106

Vernacular name(s): Aninga iba

Species: Dieffenbachia seguine (Jacq.) Schott

Family: Araceae

Notes

The woodcut is very similar to the *Theatrum* illustration (non-reversed), but in the HNB the fruit is placed on the left instead of the right side of the plant. The description (Marcgrave 1648: 106) does not correspond to this image, but to *Montrichardia linifera*, which De Laet misplaced in Piso (1648: 104) with the same vernacular name.

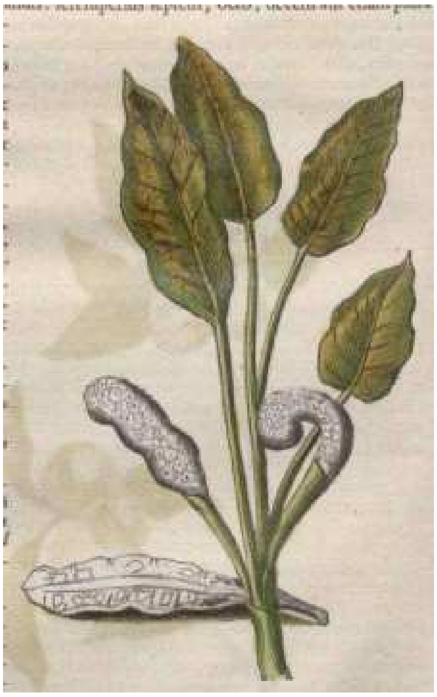

Historiae Plantarum – Arboribus: 106

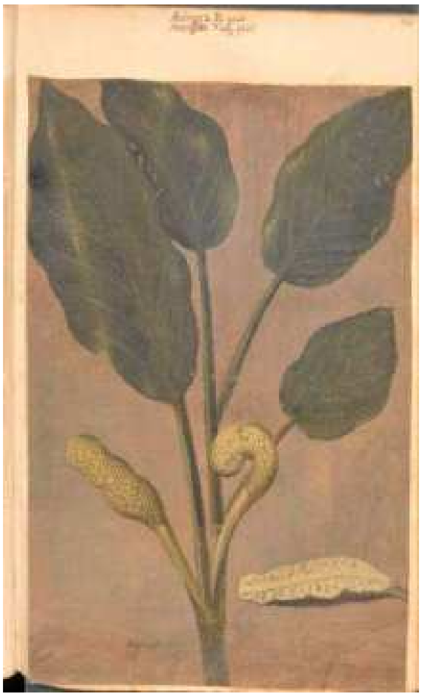

Theatrum Rerum Naturalium: 241

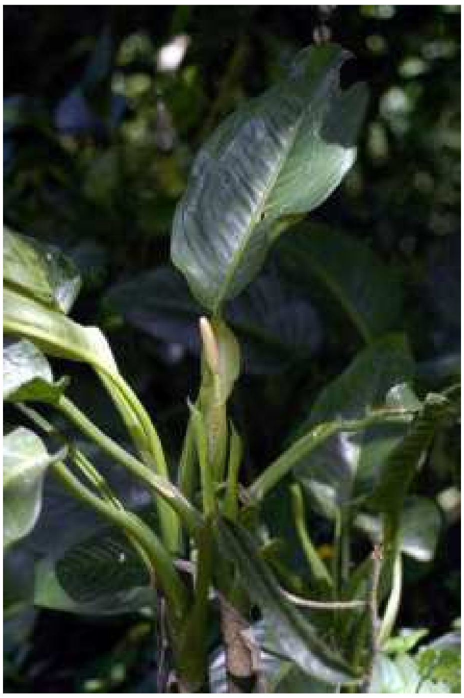

"*D. seguine*" by Pedro Acevedo-Rodríguez (CC0 1.0)

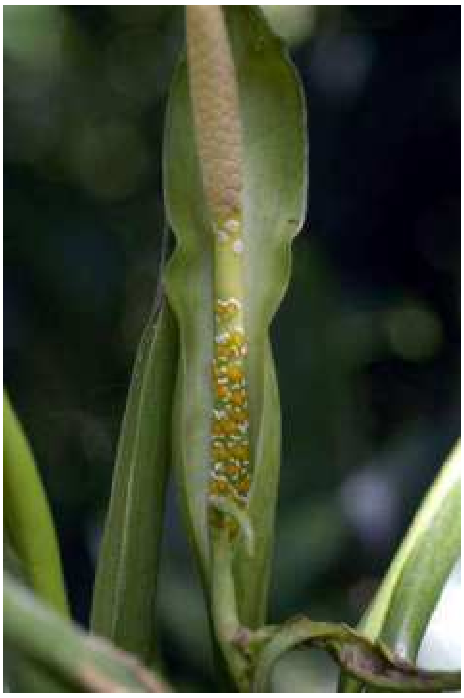

Inflorescence. "*D. seguine*" by Pedro Acevedo-Rodríguez (CC0 1.0)

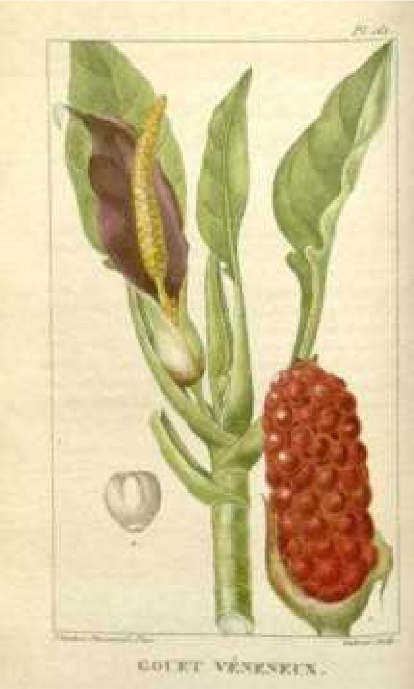

Engravinf of *D. seguine* in *Flore [pittoresque et] médicale des Antilles* by Descourtilz, M.E. (1827: Vol. III, t. 162)

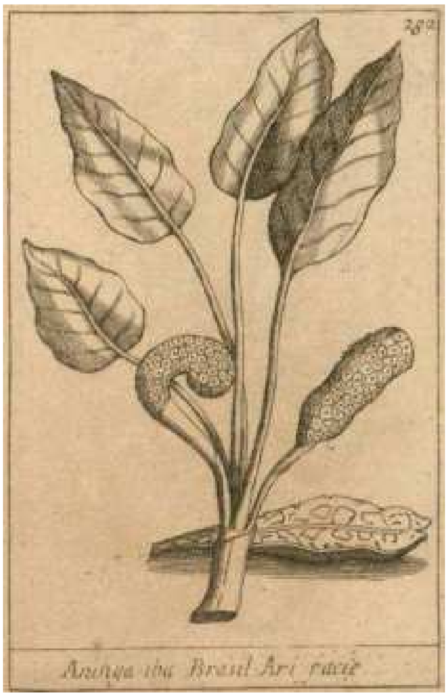

D. seguine engraving copied from the HNB in *Plantae per Galliam, Hispaniam et Italiam observatae* by Barrellier, J. (1714: t. 282)

Historiae Rerum Naturalium Brasiliae Marcgrave, 1648 Page number 107b

Vernacular name(s) Tamarindi. Tamara azecla

Species Tamarindus indica L.

Family Fabaceae

Notes

The woodcut is very similar to the *Theatrum* illustration (non-reversed), although the fruits do not look the same. The *Theatrum* could have been the model to color this woodcut as the different coloration on the top and bottom of the leaves is represented in both images. This distinction is made in the description by Marcgrave. A drawing that served as a model for both the *Theatrum* and the woodcut presents another possibility.

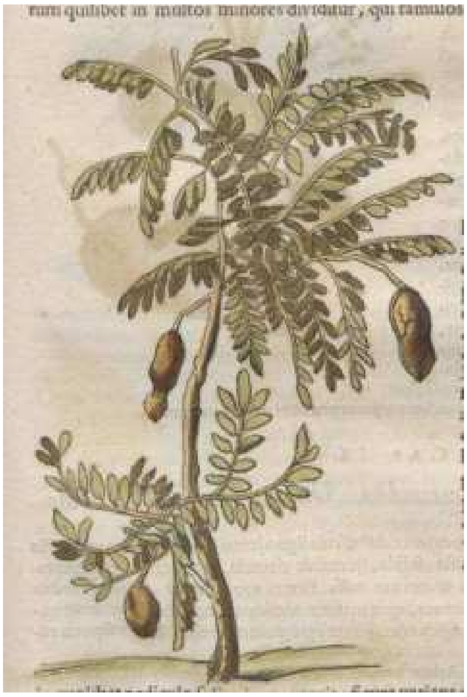

Historiae Plantarum – Arboribus: 107b

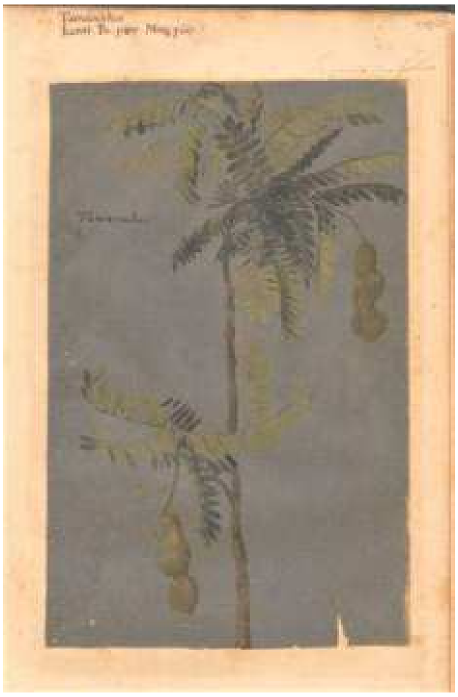

Theatrum Rerum Naturalium: 421

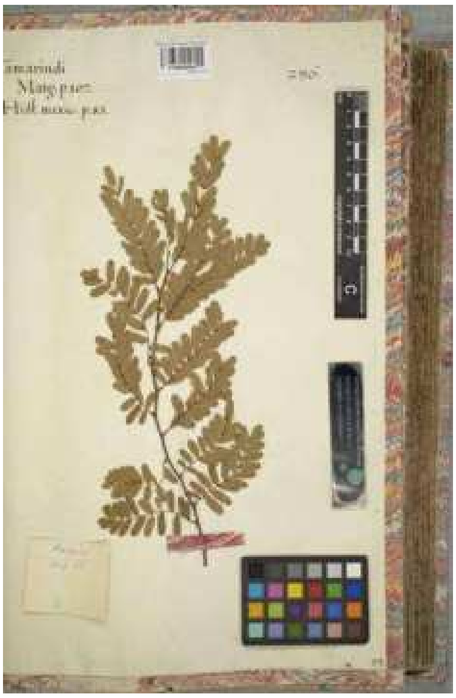

Marcgrave's herbarium: 58

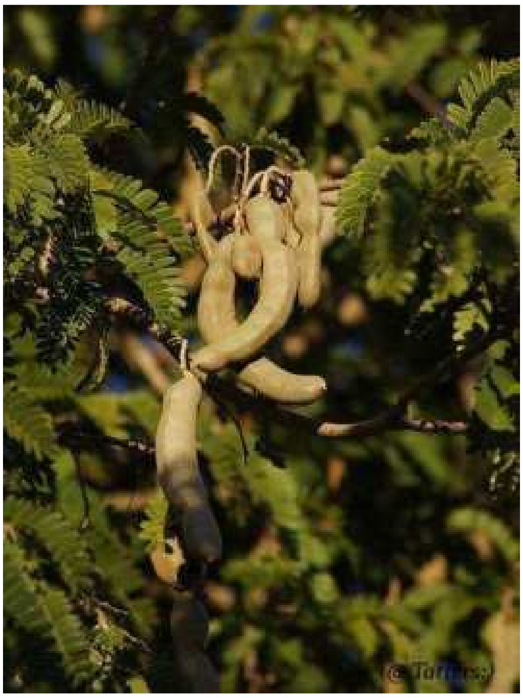

"*Tamarindus indica* fruiting" by Tatters □ (CC BY-SA 2.0)

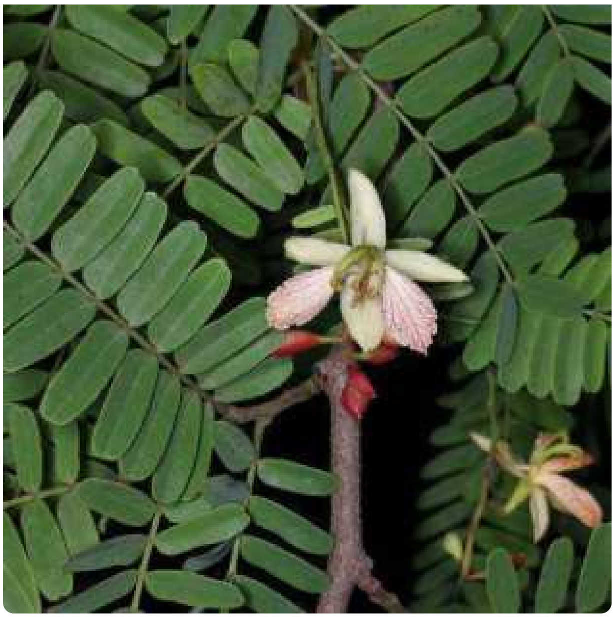

Flower. "*T. indica*" by Mauricio Mercadante (CC BY-NC-SA 2.0)

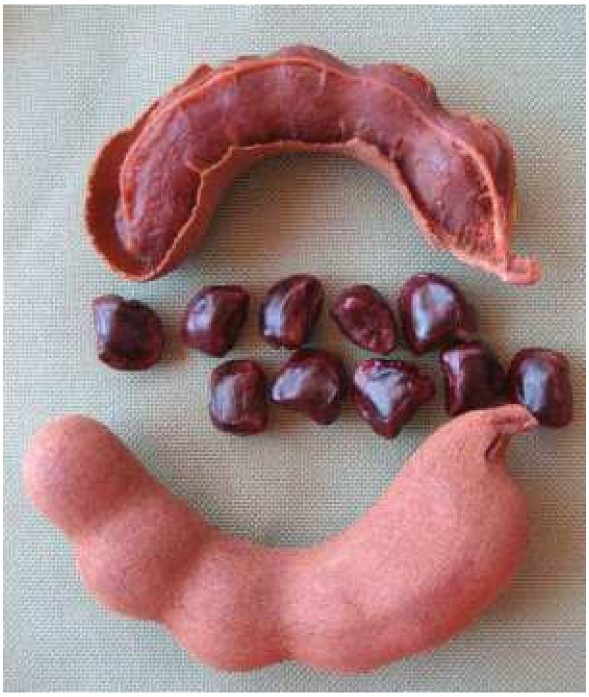

Pod and seeds surrounded by edible sweet pulp. *"T. indica"* by tonrulkens (CC BY-SA 2.0)

Historiae Rerum Naturalium Brasiliae	Marcgrave, 1648	Page number	107a

Vernacular name(s) "nos ramos nasce uma planta"

Species Maxillaria subrepens (Rolfe) Schuit. & M.W.Chase

Family Orchidaceae

Notes

De Laet explained that he tried to delineate the herb that grows on the tree branches [of *Mucuitaiba*] after the dry leaves collected by the author [Marcgrave]. Marcgrave (or a local field assistant) must have collected this orchid after collecting a branch of *Zollernia ilicifolia* (*Mucuitaiba*), which is in the herbarium at Copenhagen on the same page. The specimen looks very similar to the woodcut (in reversed format).

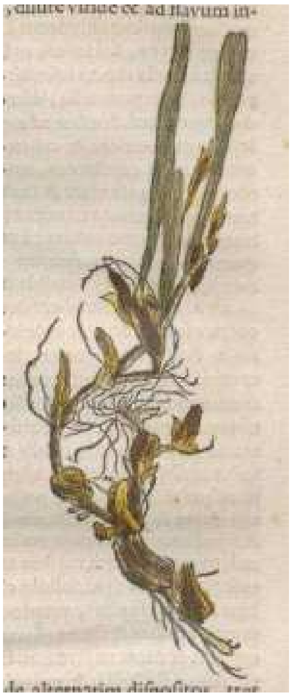

Historiae Plantarum – Arboribus: 107a

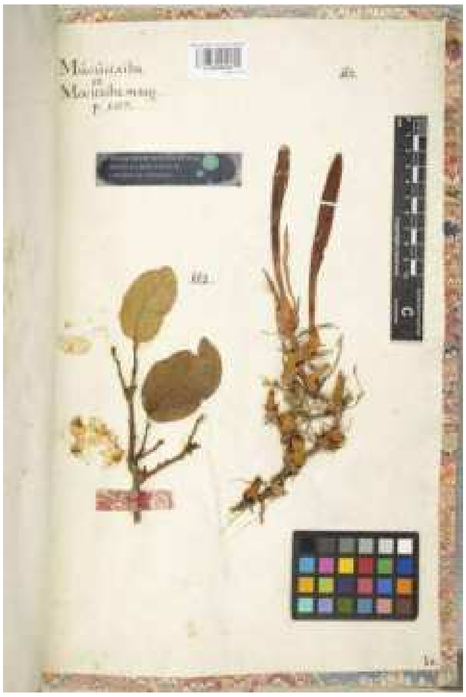

Marcgrave's herbarium: 18

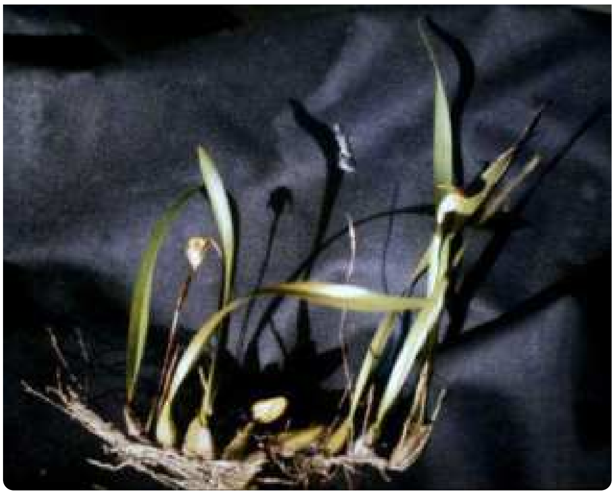

"*Trigonidium acuminatum* [syn. of *M. subrepens*], Suriname" by Maarten Sepp (CC0 1.0)

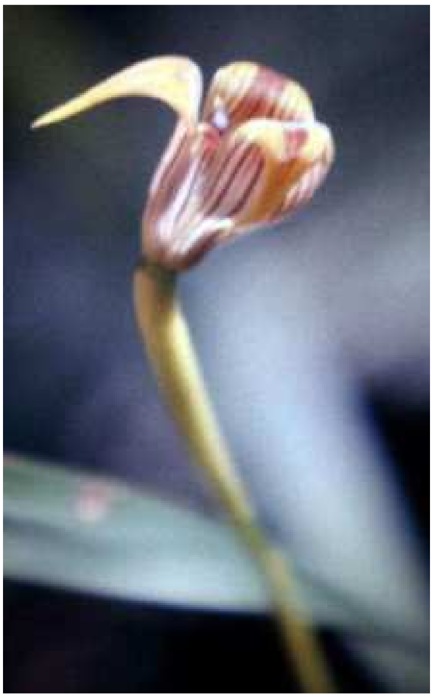

Flower. "*Trigonidium acuminatum* [syn. of *M. subrepens*], Suriname" by Maarten Sepp (CC0 1.0)

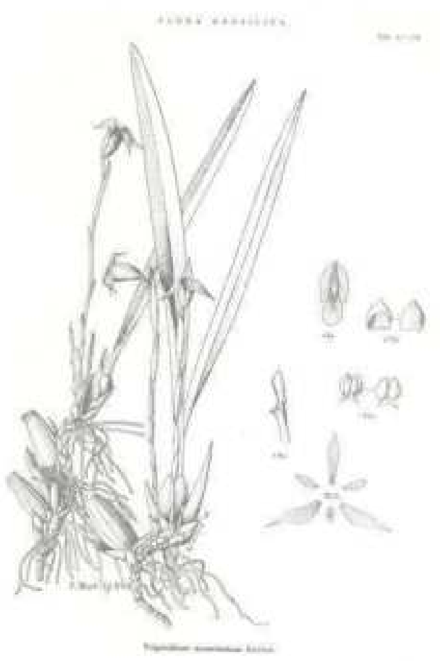

Illustration of *M. subrepens* in *Flora brasilica planejada e iniciada* by Hoehne, F.C. (1953: Vol. X, t. 172)

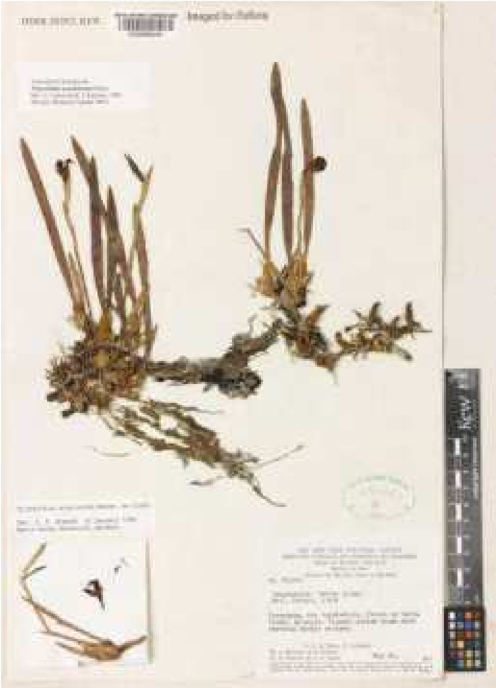

Specimen of *M. subrepens* from Kew's Herbarium - K000886549. Retrieved from Plants of the World Online

Historiae Rerum Naturalium Brasiliae | Marcgrave, 1648 | Page number 108

Vernacular name(s): Umbu (datur et alia species)

Species: Spondias tuberosa Arruda

Family: Anacardiaceae

Notes

The woodcut looks moderately similar to the *Theatrum* image. The tuber (part of the xylopodia system) lies in the front of the illustration and differs from the tuber represented in the woodcut.

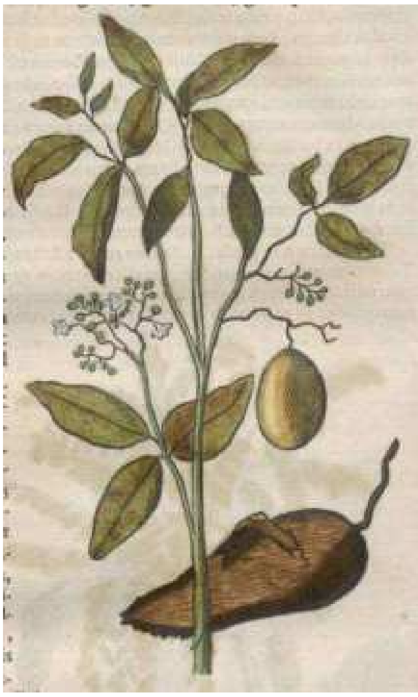

Historiae Plantarum – Arboribus: 108

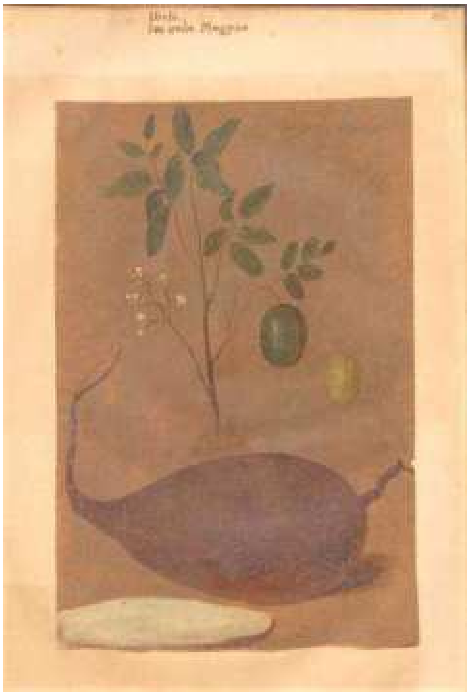

Theatrum Rerum Naturalium: 261

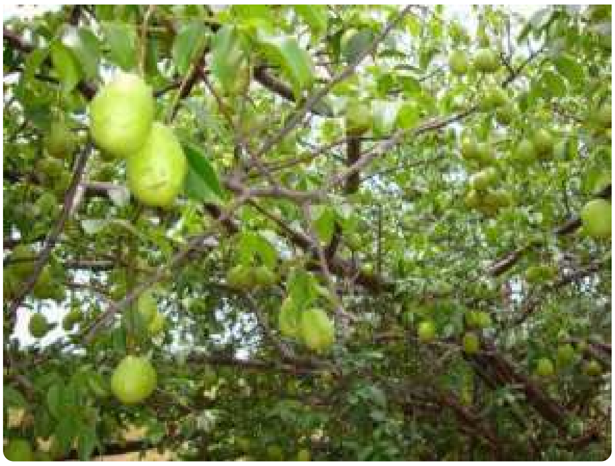

S. tuberosa with fruits. "Umbuzeiro Do Senhor Joaozinho" by ronaldinhoms9.panoramio (CC-BY-3.0)

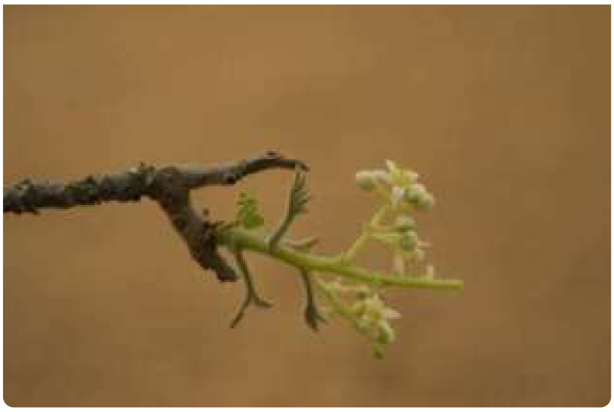

Flowers. "*S. tuberosa*" by Guilherme Jófili (CC BY 2.0)

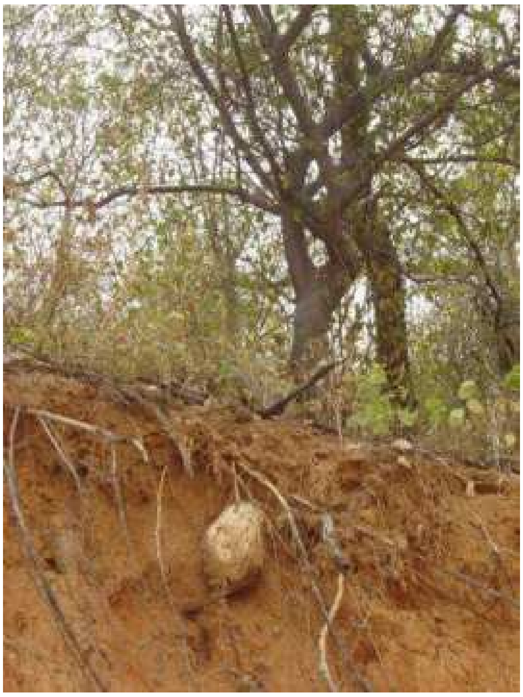

S. tuberosa tuber "batata do umbuzeiro" in http://lampiaoaceso.blogspot.com/2009/09/o-pe-do-umbuzeiro-alimentacao.html, by Ivanildo Silveira

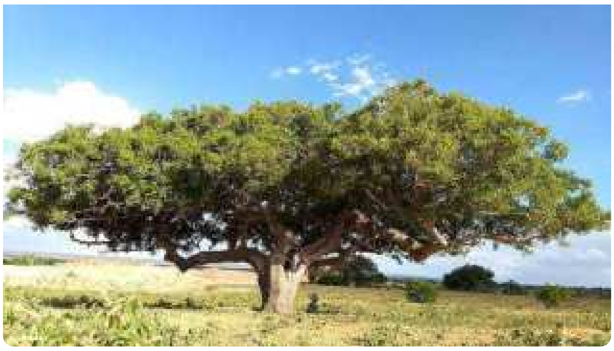

In the sertão. "Spondias tuberosa, popularmente conhecido como umbuzeiro e imbuzeiro" by Gildasio Oliveira (CC-BY-SA-4.0)

Historiae Rerum Naturalium Brasiliae	Marcgrave, 1648	Page number	109a

Vernacular name(s) Japarandiba

Species Gustavia augusta L.

Family Lecythidaceae

Notes

The woodcut is moderately similar to the *Theatrum* illustration (reversed). Notice there was an attempt to erase four of the leaves in the illustration, and these are not reversed compared to the woodcut. Chalk drawings of this species were depicted in the *Misc. Cleyeri* (open and closed flowers, and leaves), which were used as models to create the still-life painting by Ekchout. Notably, the flower does not appear in all the colored copies of the HNB and it differs among these books. The flower was added during the color process, instead of being carved into the woodblock.

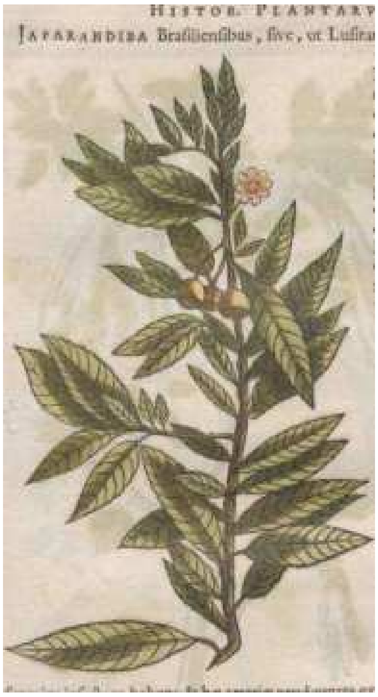

Historiae Plantarum – Arboribus: 109a

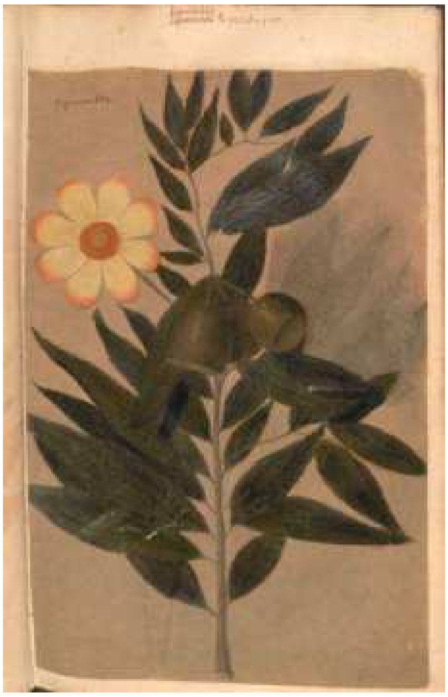

Theatrum Rerum Naturalium: 163

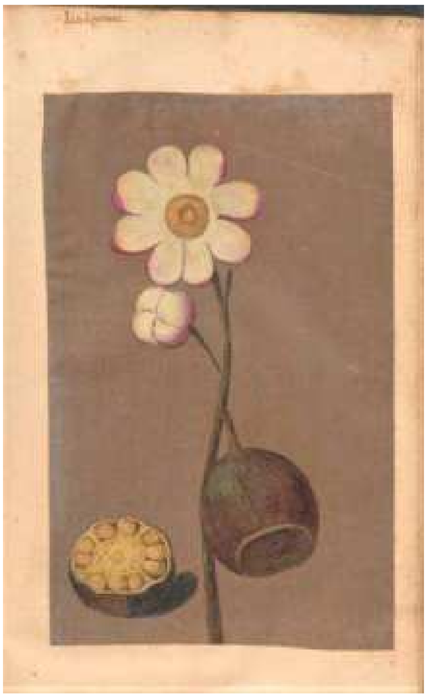

Theatrum Rerum Naturalium: 165

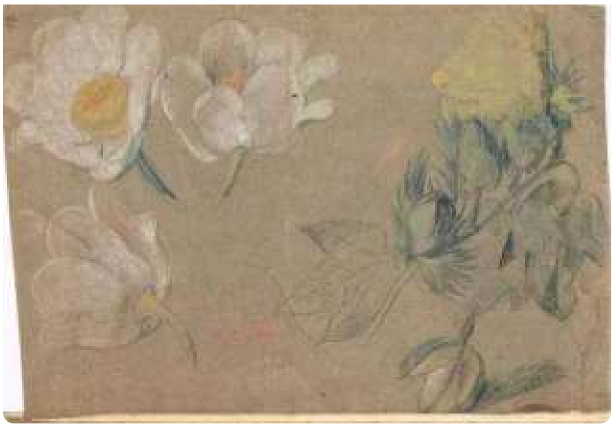

Miscellanea Cleyeri: 13r

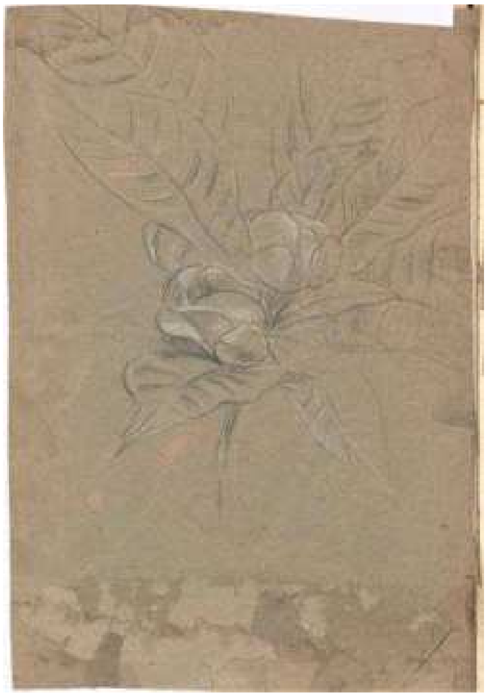

Miscellanea Cleyeri: 13v

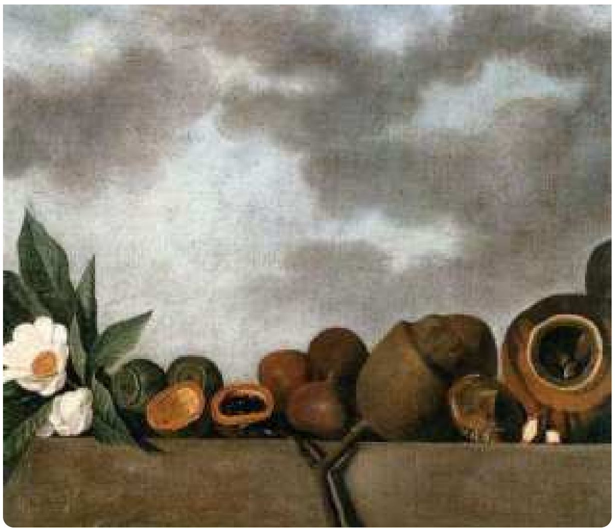

G. augusta flowering branch and fruits on the left in Ekchout's still-life with monkeypot fruit, ca. 1640. Copenhagen, National Museum of Denmark

Historiae Rerum Naturalium Brasiliae Marcgrave, 1648 Page number 109b

Vernacular name(s) Arbor

Species Unknown

Family Unknown

Notes

We did not find any correspondence between this woodcut and the contemporary or older sources. We could not identify the plant represented by the woodcut, hence we could not cross-reference it with the visual sources. Accurate identification of such plant will facilitate this analysis and provide us with more insights about its origin.

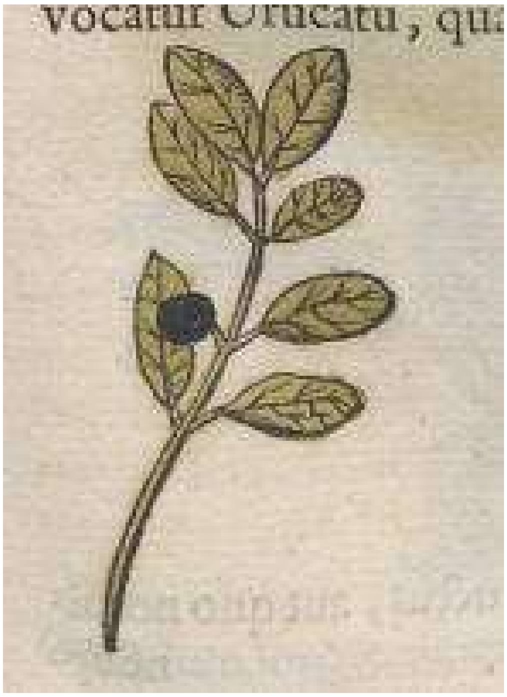

Historiae Plantarum – Arboribus: 109b

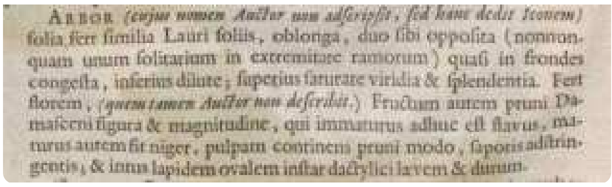

ARBOR *(cujus nomen Auctor non adscripsit, sed hanc dedit Iconem)* folia fert similia Lauri foliis, oblonga, duo sibi opposita (nonnonquam unum foliarium in extremitate ramorum) quasi in frondes congesta, inferius dilute; superius saturate viridia & splendentia. Fert florem, *(quem tamen Auctor non descripsit.)* Fructum autem pruni Damasceni figura & magnitudine, qui immaturus adhuc est flavus, maturus autem fit niger, pulpam continens pruni modo, saporis adstringentis; & intus lapidem ovalem instar dactylici laevem & durum.

HNB (Marcgrave 1648: 109) Latin edition

[illegible]

ÁRVORE *(o autor não lhe dá nome, mas apresenta sua imagem)*. De folhas semelhantes às do loureiro, oblongadas, opostas por pares (algumas vezes aparece uma isolada na extremidade dos ramos) congestas em forma de fronde; são verde-claras na parte inferior, verdes e carregadas e lustrosas na superior. Produz uma flor *(o autor não a descreve)*. Seu fruto é semelhante, quanto à figura e tamanho à ameixa de Damasco; quando não está maduro é amarelo; quando maduro, é preto, contendo uma polpa como a ameixa (*Prunus*), de sabor adstringente; dentro se acha um caroço oval, como o da tâmara; liso e duro.

HNB (1942 [1648]) Portuguese edition

"The leaves are similar to the laurel, oblong, opposed by pairs (sometimes an isolated one appears at the end of the branches) congested in the shape of a frond; they are light green at the bottom, green and heavy and glossy at the top. It produces a flower (which the author does not describe). Its fruit is similar, in terms of the figure and size of the apricot plum; when it is not ripe, is yellow; when ripe, it is black, containing a pulp such as the plum (*Prunus*), with an astringent flavor, inside there is an oval seed, like *tamara*, smooth and hard" (HNB 1648: 109)

English translation, by M. Alcantara-Rodriguez

Historiae Rerum Naturalium Brasiliae Marcgrave, 1648 Page number 110a

Vernacular name(s) Anda

Species Joannesia princeps Vell.

Family Euphorbiaceae

Notes

We did not find any correspondence between this woodcut and the contemporary or older sources. As pointed out by Pickel (2008: 129) this image is not accurate, especially the flowers, which look much larger and greatly different from the flowers of *J. princeps*. The flowers in the woodcut bear resemblance to flowers of the Bignoniaceae species, or as Pickel (2008: 130) suggested, the flowers could belong to *Solandra grandiflora* Sw.,, a liana that entangles in these types of trees.

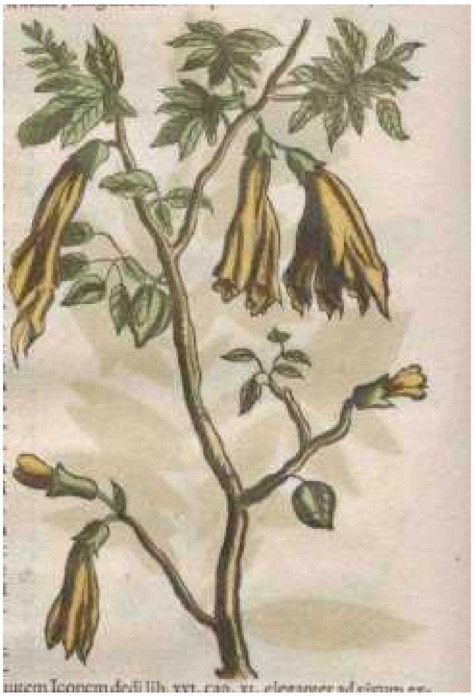

Historiae Plantarum – Arboribus: 110a

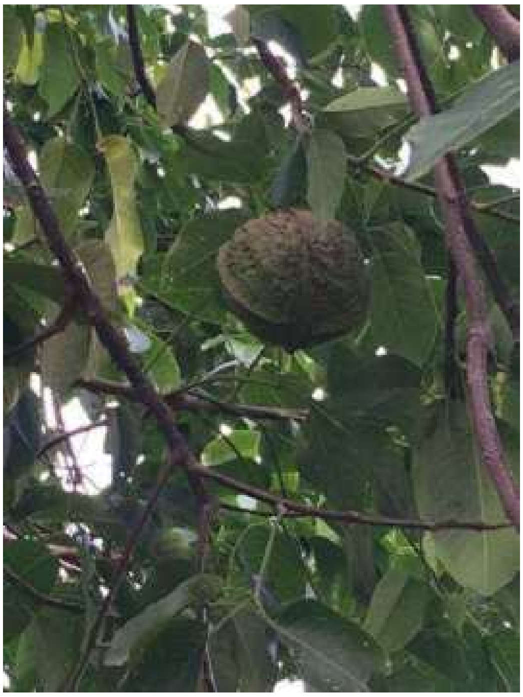

Hanging fruit of *J. princeps* observed in Brazil by Vitor Menescal for iNaturalist (CC BY-NC 4.0)

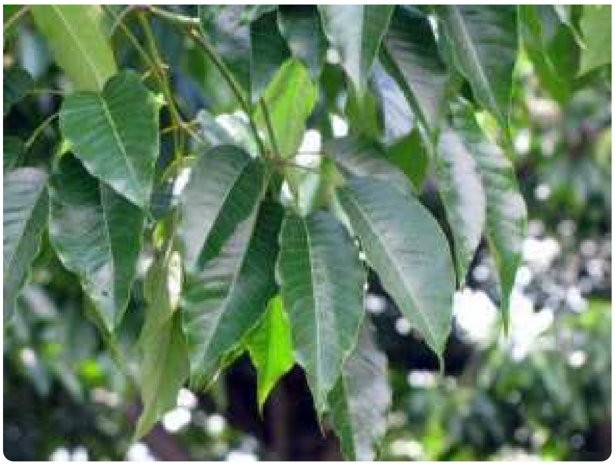

Leaves. "Cotieira (*J. princeps*)" by Mauricio Mercadante (CC BY-NC-SA 2.0)

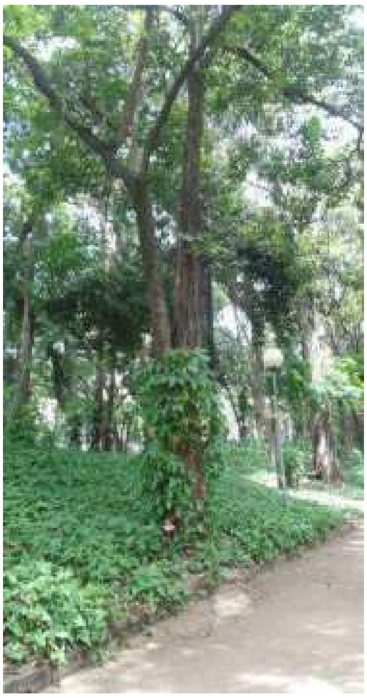

Habit. "*J. princeps* (Cutieira)" by flora.wheberson.com.br (CC BY 2.0)

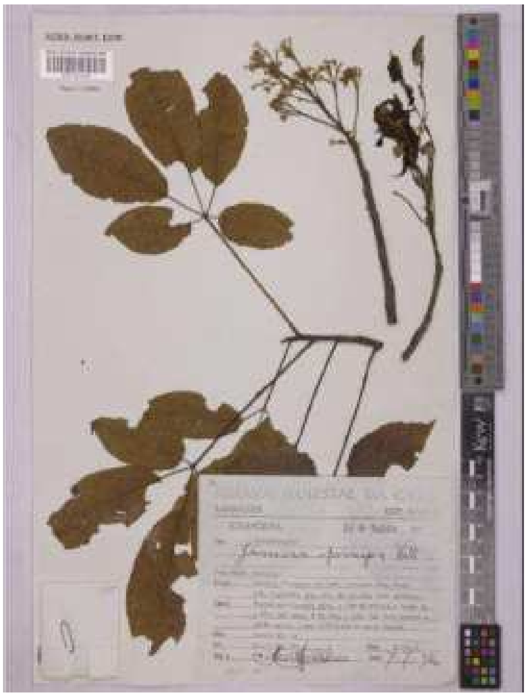

Flowering specimen of *J. princeps* collected in Brazil by Royal Botanic Gardens, Kew -K001205887- Retrieved from GBIF

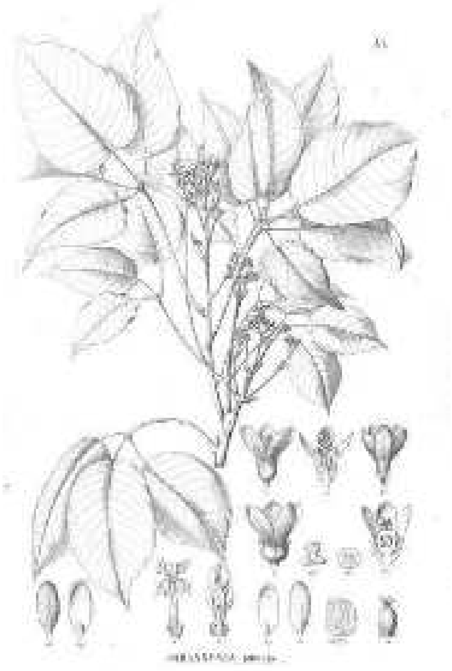

Engraving of *J. princeps* in Martius, C.F.P. von, Eichler, A.G., Urban, I., *Flora Brasiliensis* (1873-1874) Vol. 11(2): 43

Historiae Rerum Naturalium Brasiliae	Marcgrave, 1648	Page number	110b

Vernacular name(s) Anda

Species <u>Joannesia princeps Vell.</u>

Family Euphorbiaceae

Notes

The woodcut was made after a fruit of this species that De Laet received from Brazil in the 1630s.

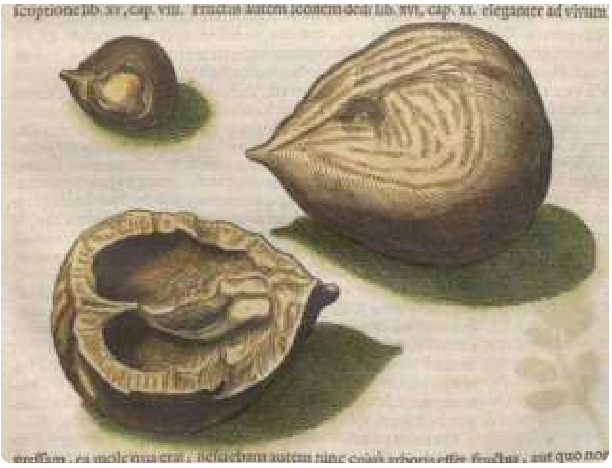

Historiae Plantarum – Arboribus: 110b

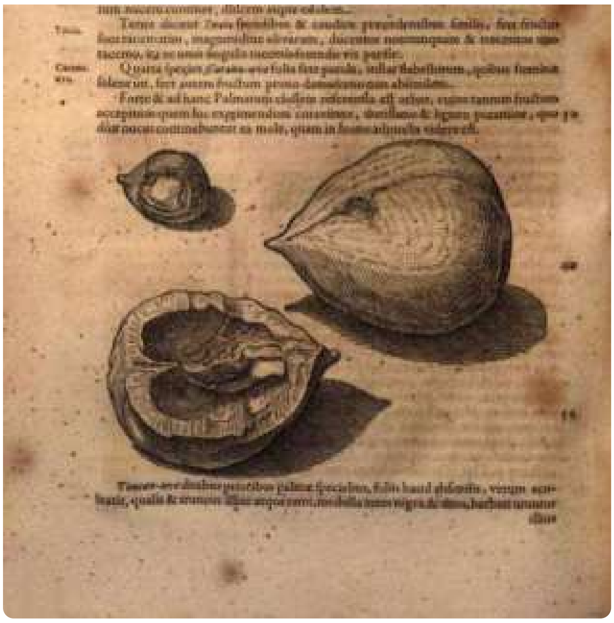

Woodcut of *Anda* nuts in *Novus Orbis seu descriptionis Indiae Occidentalis* by Johannes De Laet (1633: 612)

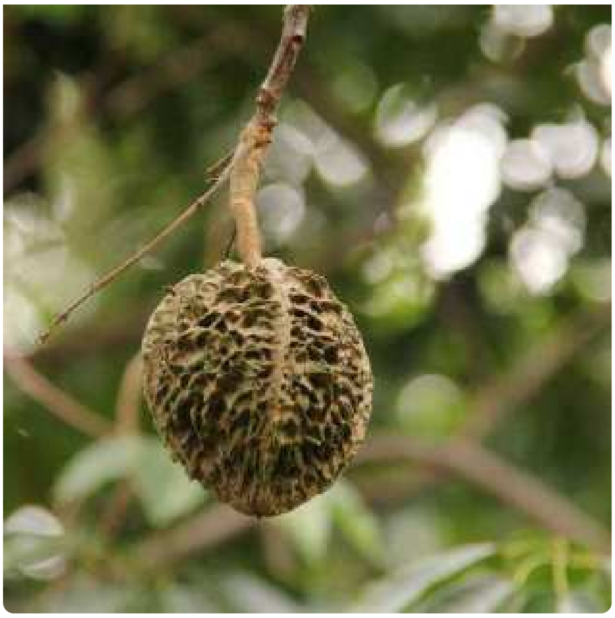

Fruit. "Cotieira (*J. princeps*)" by Mauricio Mercadante (CC BY-NC-SA 2.0)

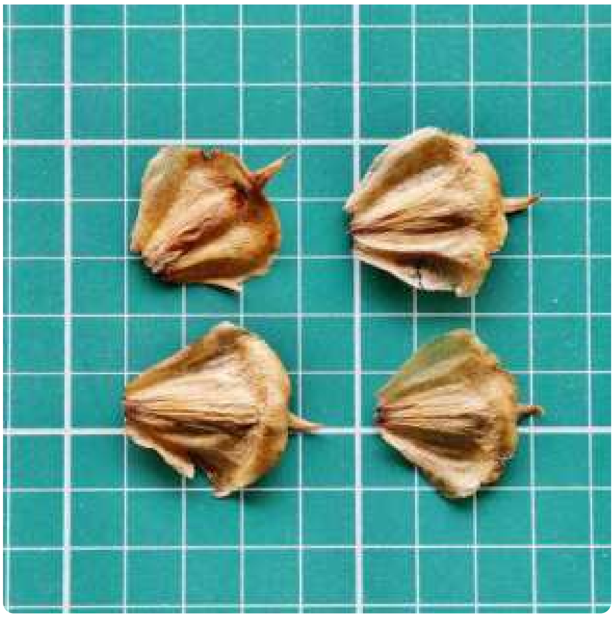

Seeds. "*J. princeps*" by Mauricio Mercadante (CC BY-NC-SA 2.0)

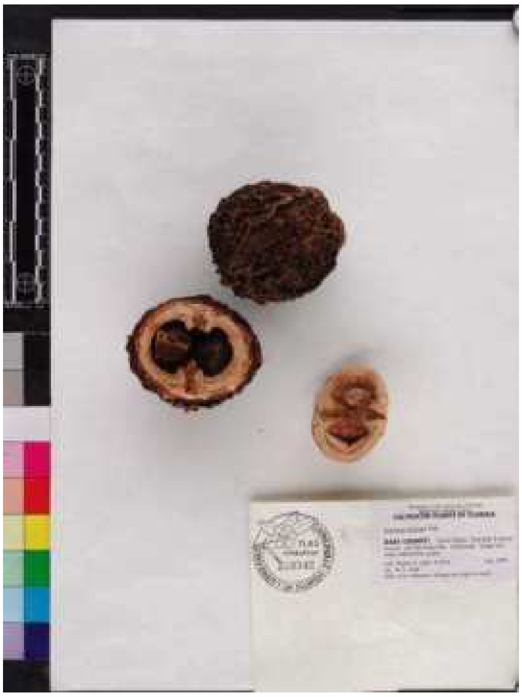

Fruit of *J. princeps* Collected in USA by University of Florida Herbarium, Florida Museum -FLAS 208542- Kathy M. Davis (CC BY-NC 4.0)

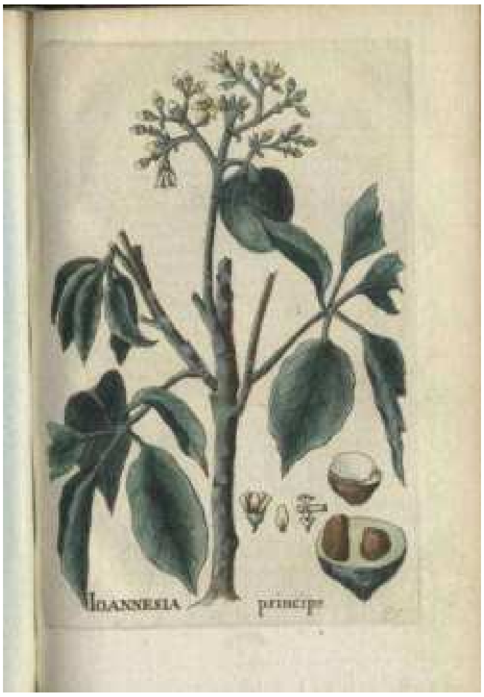

Illustration of *J. princeps* in *Alographia dos alkalis fixos* by Vellozo, J.M. da Conceição (1798). Biblioteca Nacional de Portugal

Historiae Rerum Naturalium Brasiliae	Marcgrave, 1648	Page number	111a

Vernacular name(s)	Guaibi pocaca iba
Species	Samanea saman (Jacq.) Merr.
Family	Fabaceae
Notes	

The woodcut is slightly similar to the *Theatrum* image, but the leaves and flowers look more droopy. Breyne (1678: 28) criticized the woodcut in the HNB and provided another one that came from Brazil (Andrade-Lima et al. 1977: 146). As Andrade-Lima et al. (1977) noticed, this might be connected to Marcgrave.

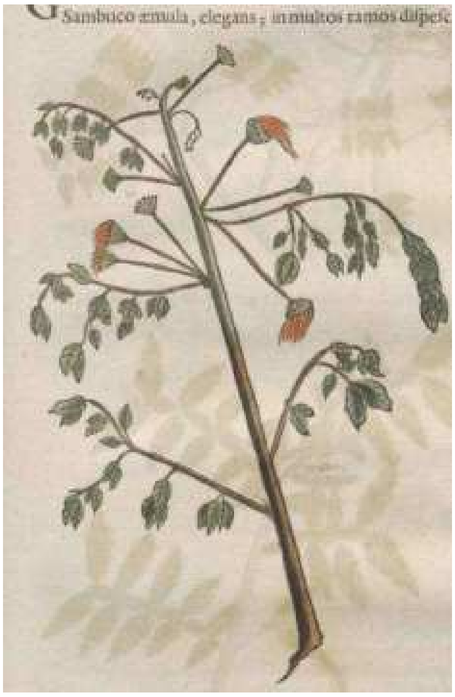

Historiae Plantarum – Arboribus: 111a

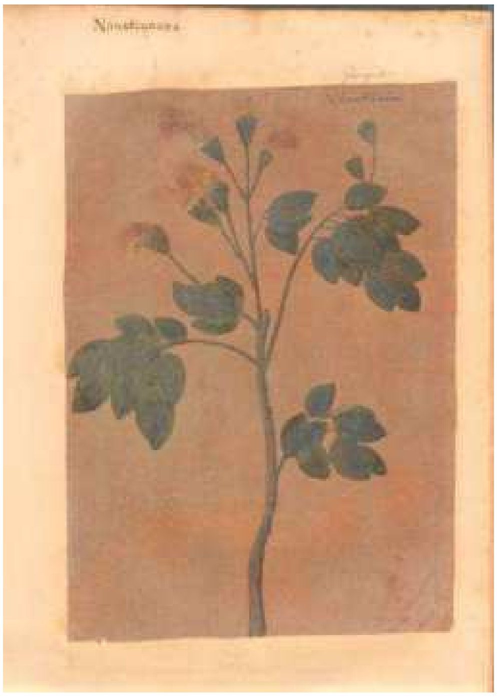

Theatrum Rerum Naturalium: 399

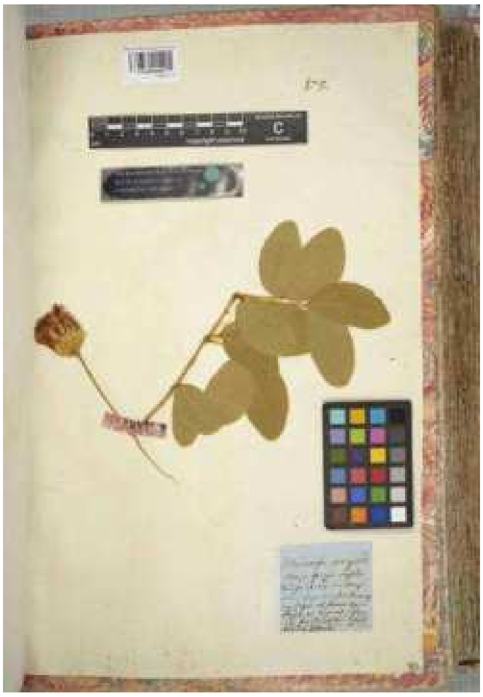

Marcgrave's herbarium: 89

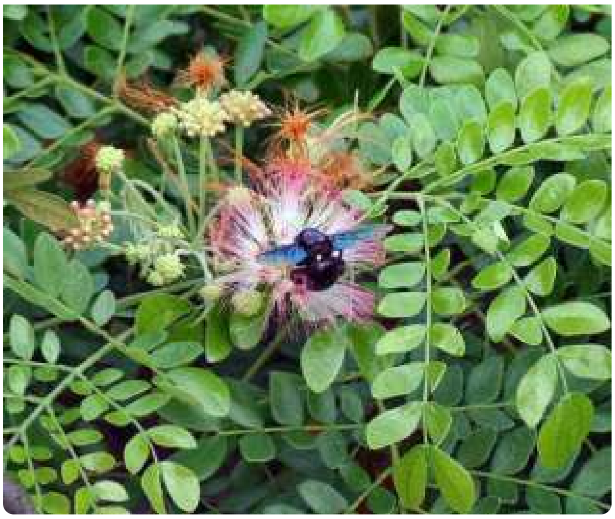

"Flower & flower buds- *S. saman*" by J.M.Garg (CC BY-SA 3.0)

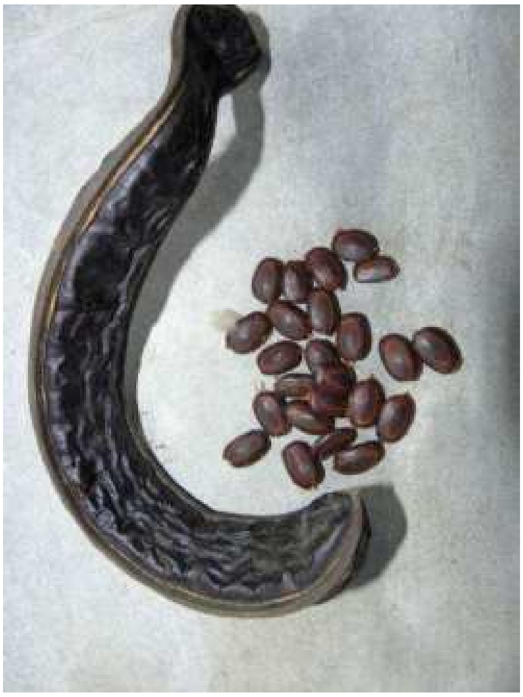

Pod and seeds. "*S. saman*" by Reinaldo Aguilar (CC BY-NC-SA 2.0)

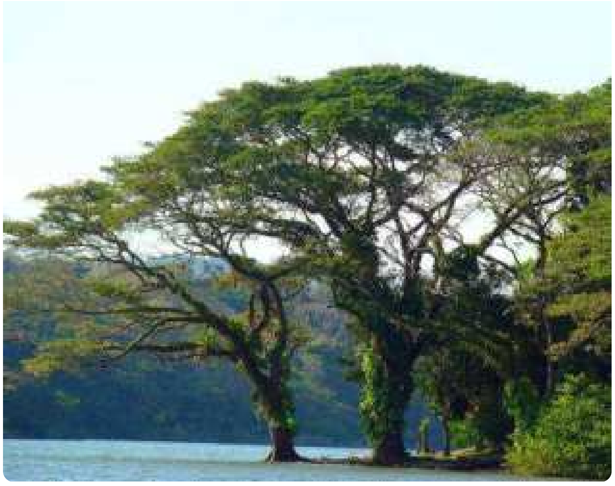

Habit. "*S. saman*, the Rain Tree of Arbol de Lluvia." by Dick Culbert (CC BY 2.0)

Historiae Rerum Naturalium Brasiliae | Marcgrave, 1648 | Page number 111b

Vernacular name(s) Inga species. Lotus

Species Inga vera Willd.

Family Fabaceae

Notes

We did not find any correspondence between this woodcut and the contemporary or older sources.

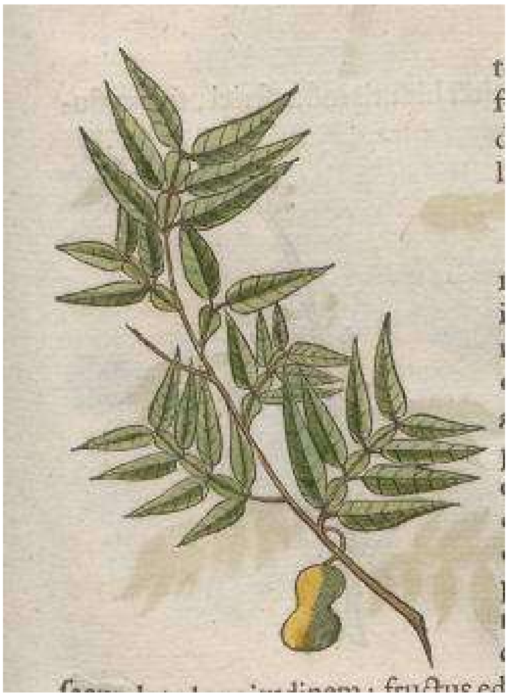

Historiae Plantarum – Arboribus: 111b

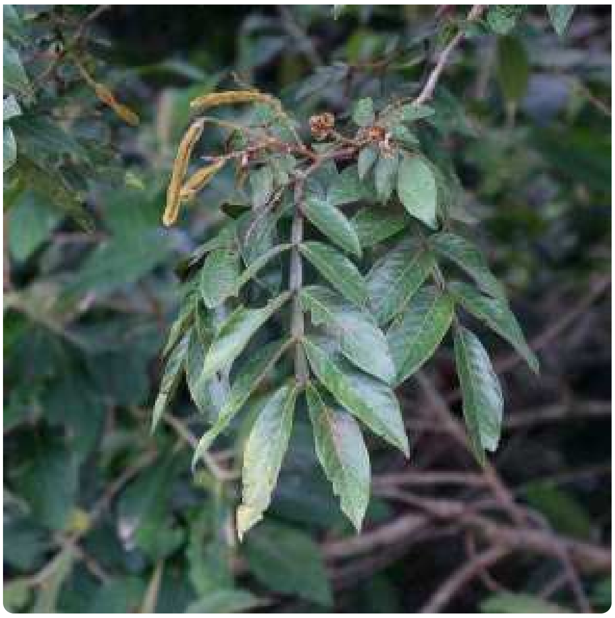

Fruiting branch. "*I. vera*" by Mauricio Mercadante (CC BY-NC-SA 2.0)

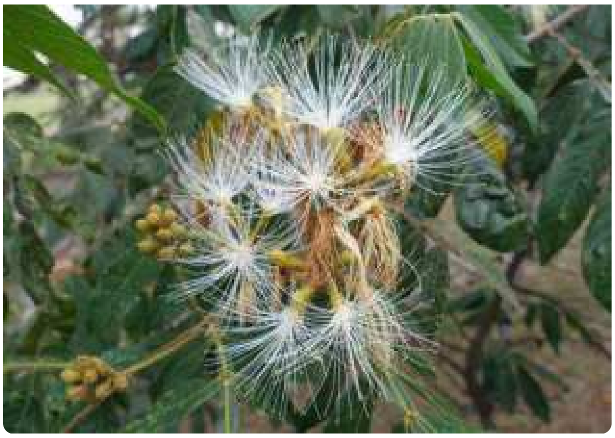

Flowering branch. "Ingá quatro quinas *I. vera*" by mauro halpern (CC BY 2.0)

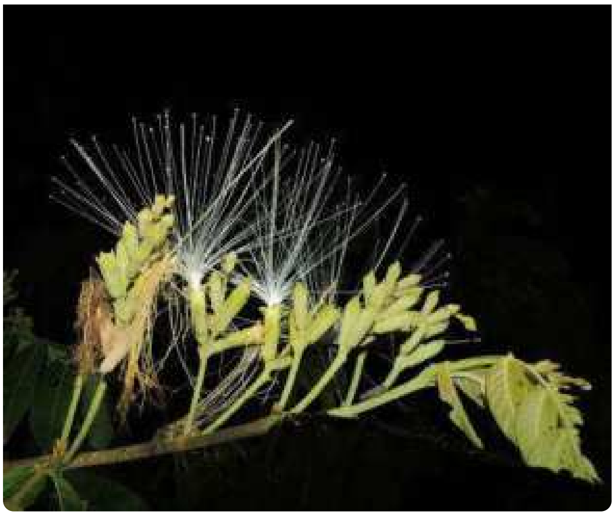

"*I. vera*" by Reinaldo Aguilar (CC BY-NC-SA 2.0)

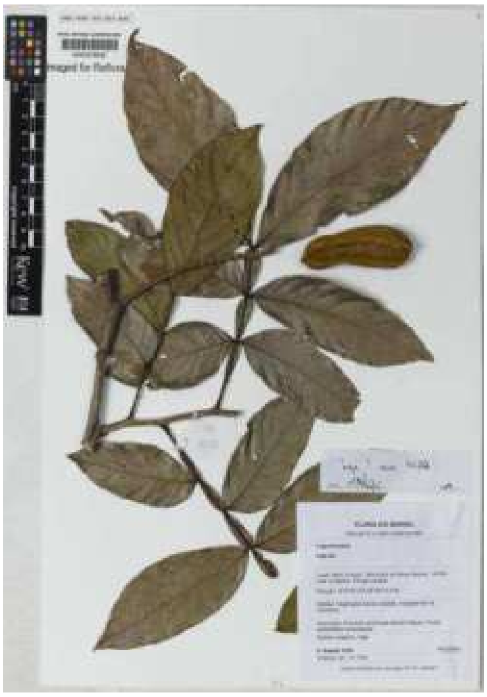

Specimen of *I. vera* from Kew's Herbarium - K000579826. Retrieved from Plants of the World Online

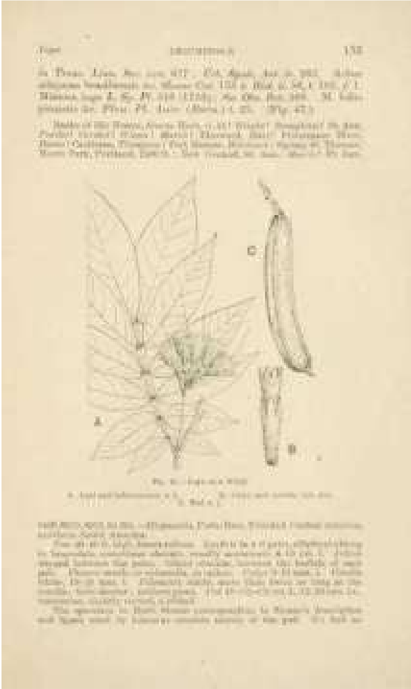

I. vera drawings in *Flora of Jamaica* (1910-1936) by Fawcett, W., Rendle, A.B. (1920: Vol. IV, p. 153 f. 47). New York Botanical Garden, U.S.A.

Historiae Rerum Naturalium Brasiliae Marcgrave, 1648 Page number 112a

Vernacular name(s) Inga opeapiiba

Species Inga ciliata C.Presl

Family Fabaceae

Notes

We did not find any correspondence between this woodcut and the contemporary or older sources.

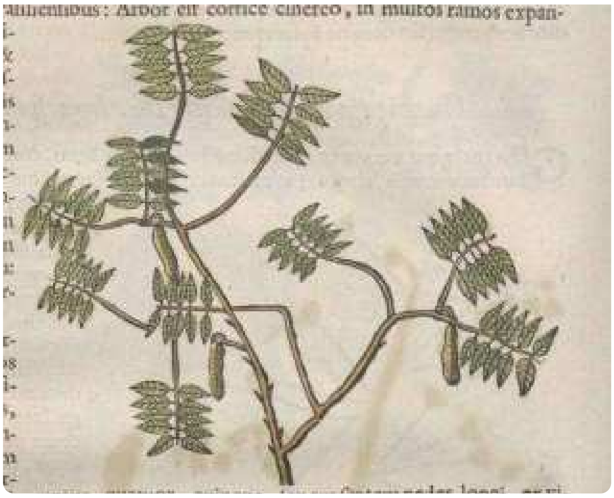

Historiae Plantarum – Arboribus: 112a

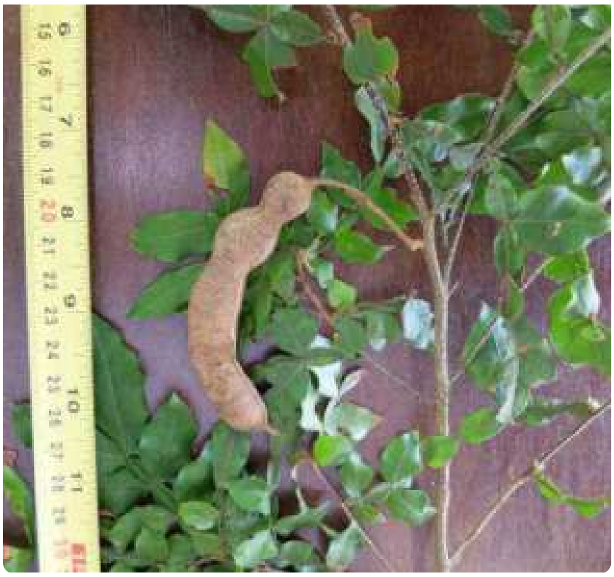

Branch and pod of *I. ciliata* collected in Brazil by M. Pignal for iNaturalist (CC BY-NC 4.0)

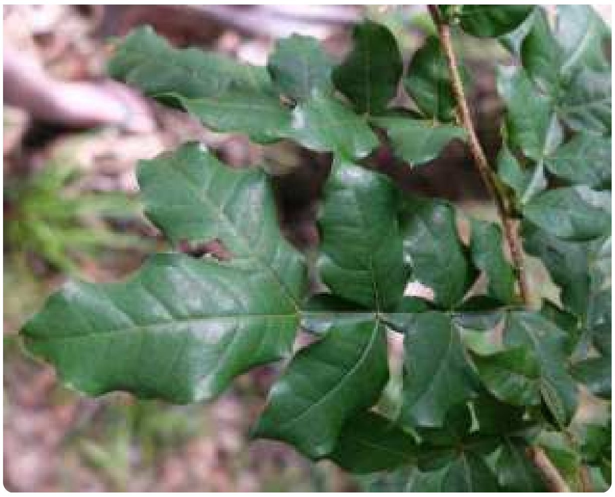

Leaves of of *I. ciliata* collected in Brazil by M. Pignal for iNaturalist (CC BY-NC 4.0)

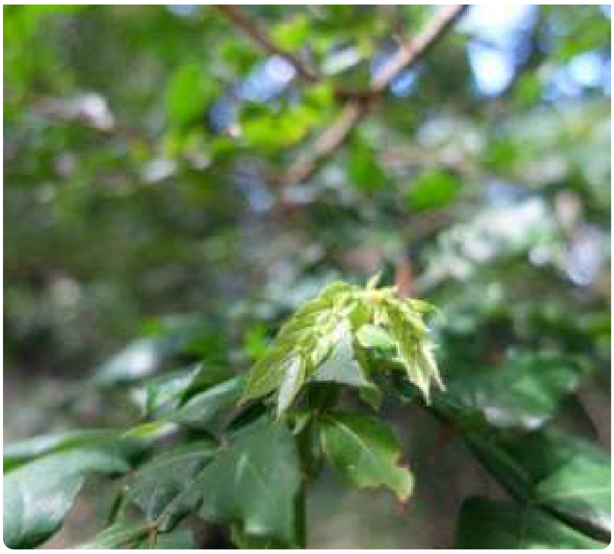

Young apical leaves of of *I. ciliata* collected in Brazil by M. Pignal for iNaturalist (CC BY-NC 4.0)

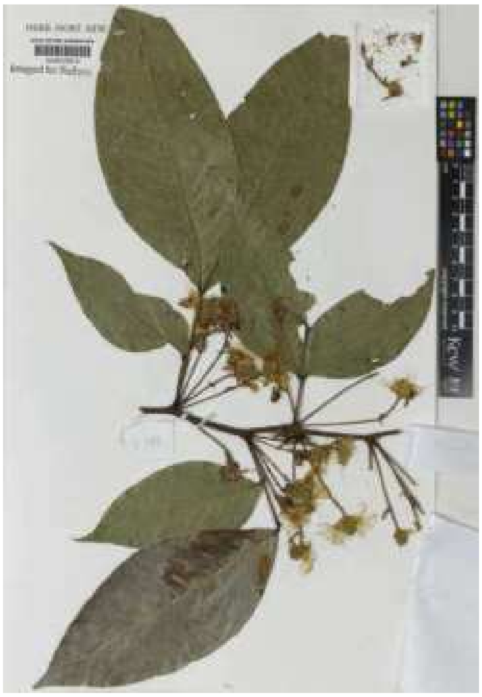

Flowering specimen of *I. ciliata* from Kew's Herbarium - K000876819. Retrieved from Plants of the World Online

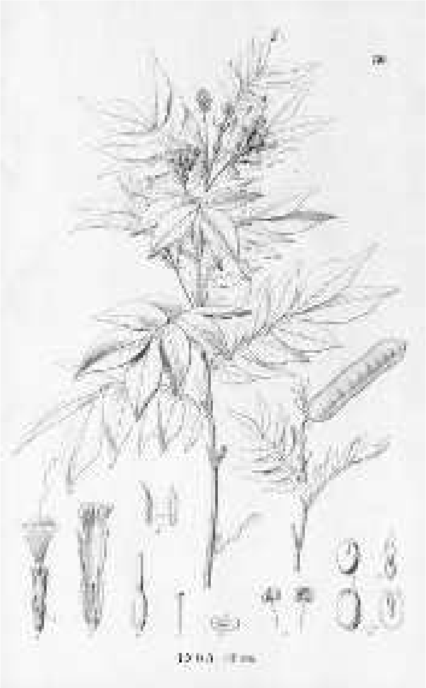

Engraving of *I. ciliata* in Martius, C.F.P. von, Eichler, A.G., Urban, I., *Flora Brasiliensis* (1870-1876) Vol. 15 (2): t. 130, p. 484

Historiae Rerum Naturalium Brasiliae Marcgrave, 1648 Page number 112b

Vernacular name(s) Inga alia species

Species Inga edulis Mart.

Family Fabaceae

Notes

The woodcut differs from the illustrations in the *Theatrum*, which provided a better image of the long pod and it also included a flowering branch.

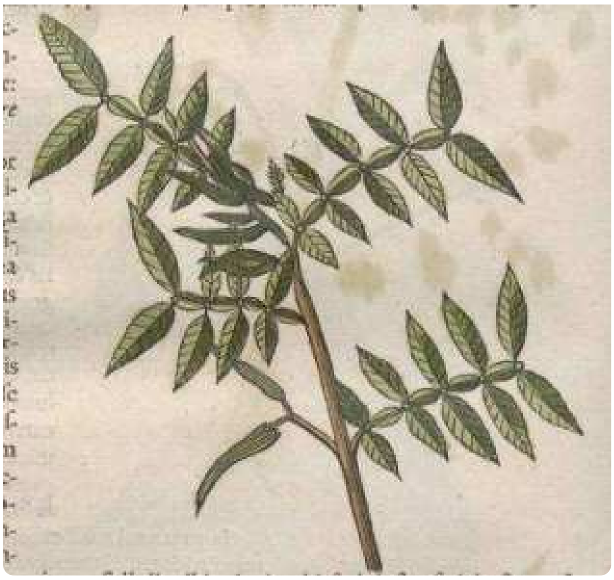

Historiae Plantarum – Arboribus: 112b

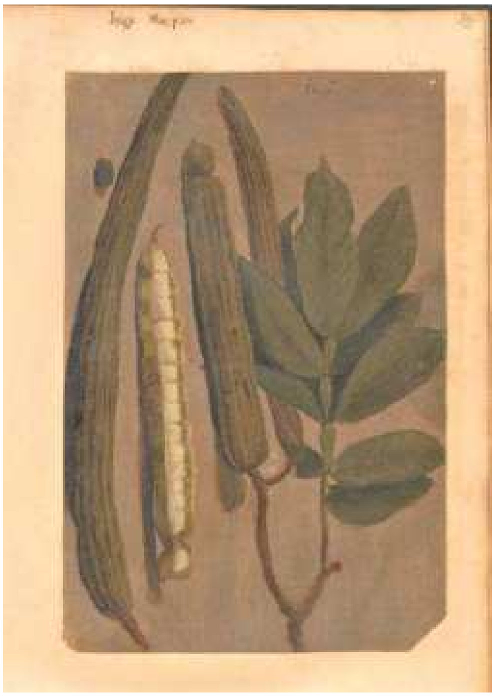

Theatrum Rerum Naturalium: 395

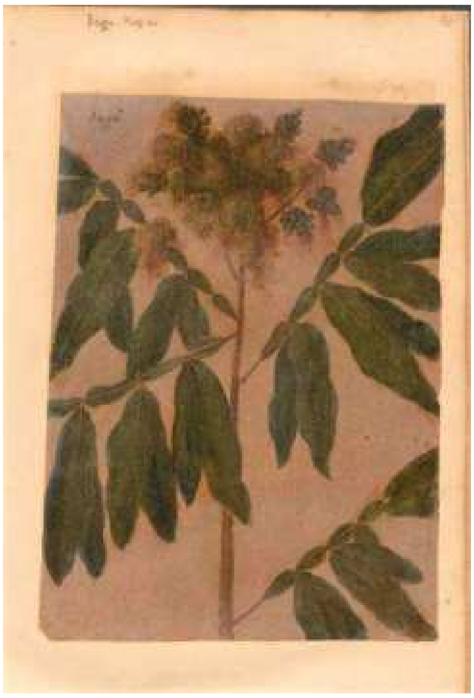

Theatrum Rerum Naturalium: 397

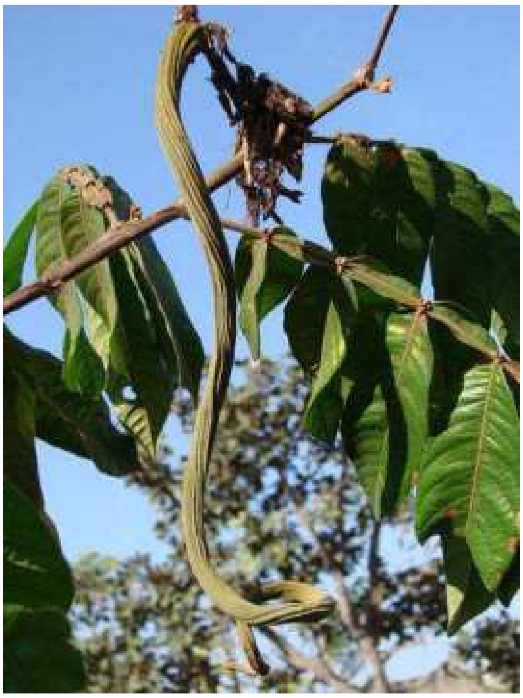

"*I. edulis*" by João de Deus Medeiros (CC BY 2.0)

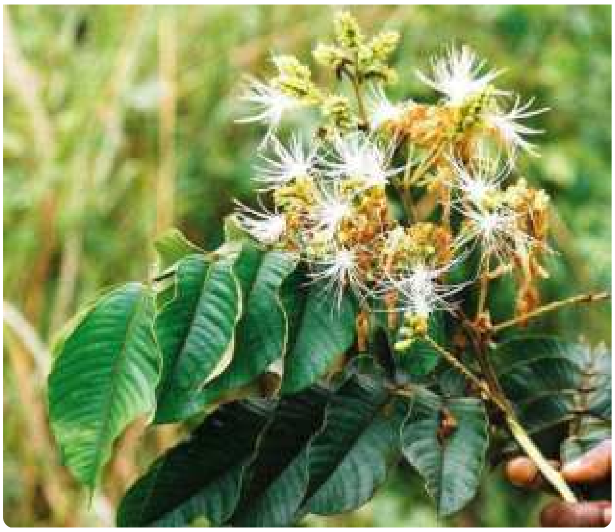

"*I. edulis* in flower" by Scamperdale (CC BY-NC 2.0)

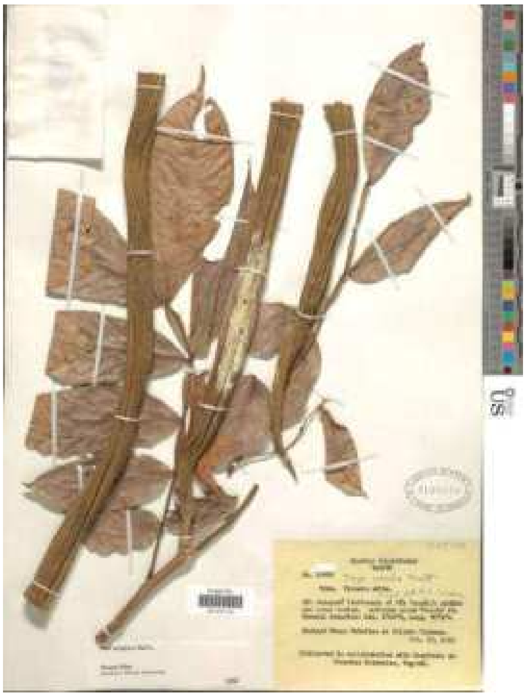

Specimen. "*I. edulis*" by Richard E. Schultes and Isidoro Cabrera-Rodriguez -02187719- Smithsonian National Museum of Natural History (CC0 1.0)

Historiae Rerum Naturalium Brasiliae Marcgrave, 1648 Page number 112c

Vernacular name(s) Inga Brasiliensibus

Species Inga ingoides (Rich.) Willd.

Family Fabaceae

Notes

In Marcgrave's herbarium, one of the specimens (p. 43) consists of leaves, wrongly arranged, while the other (p. 19) includes a compound leaf with wider leaflets and with part of an inflorescence below. This flower could have been the model used for designing the flower in the woodcut.

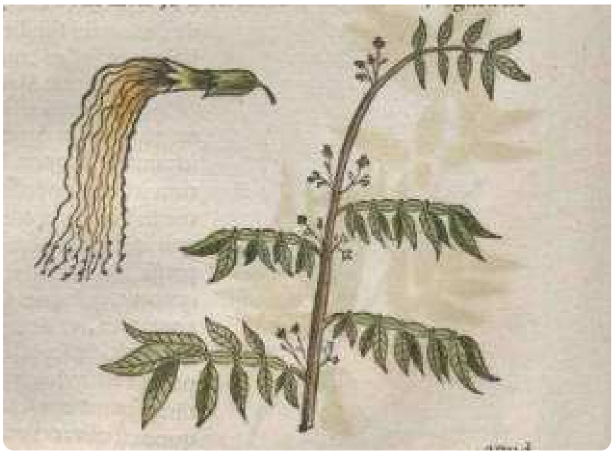

Historiae Plantarum – Arboribus: 112c

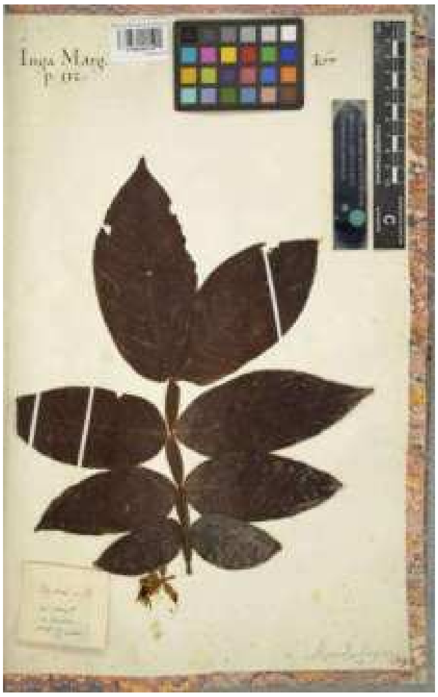

Marcgrave's herbarium: 19

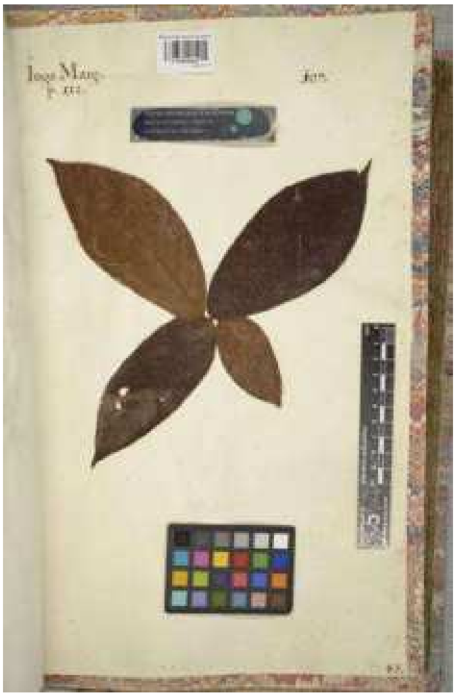

Marcgrave's herbarium: 43

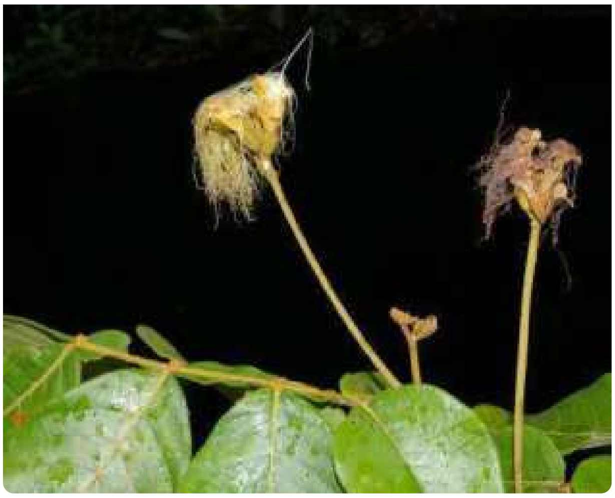

Flowering branch of *I. ingoides* collected in Peru by Vásquez Martínez - 34990 - for MBG (CC BY-NC-SA 3.0)

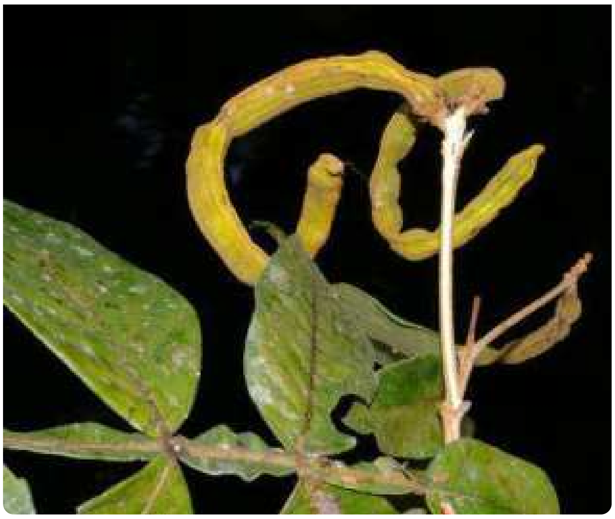

Fruiting branch of *I. ingoides* collected in Peru by Vásquez Martínez - 34990 - for MBG (CC BY-NC-SA 3.0)

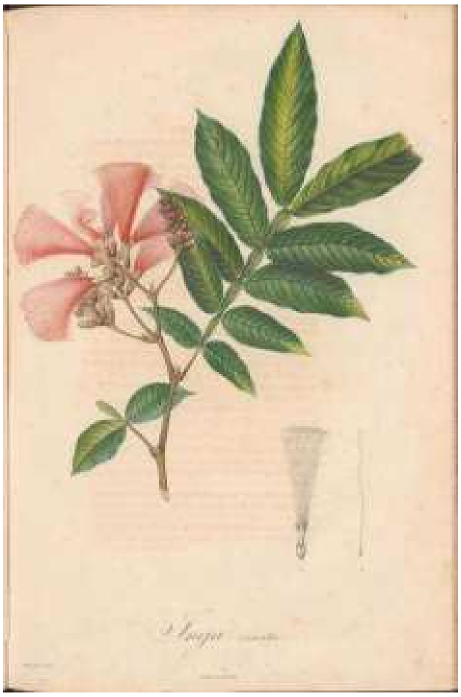

I. ingoides in *Mimoses et autre plantes légumineuses du nouveau continent* by Kunth, K.S. (1819-1824: t.14). ETH Bibliothek, Zürich, Switzerland

Historiae Rerum Naturalium Brasiliae | Marcgrave, 1648 | Page number 113a

Vernacular name(s) Quity. Pao de Sabaon (Sabaon fruit)

Species Sapindus saponaria L.

Family Sapindaceae

Notes

There is no resemblance between the woodcut and the specimen, which consists of a sterile branch.

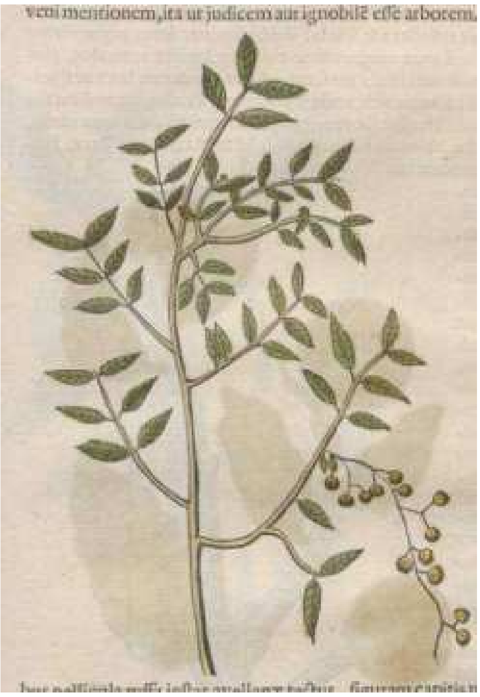

Historiae Plantarum – Arboribus: 113a

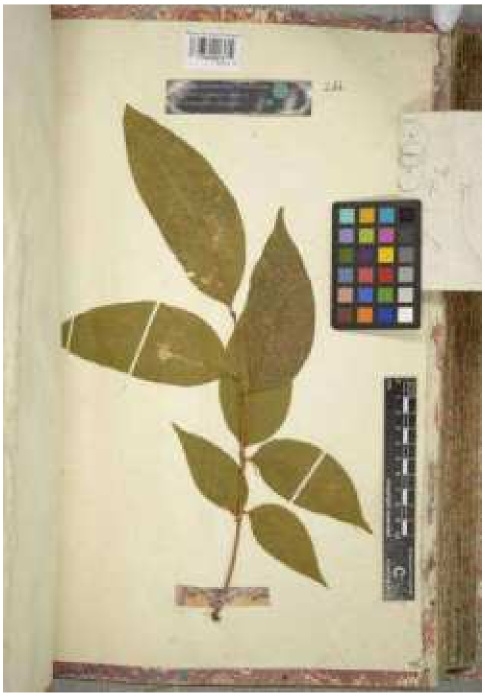

Marcgrave's herbarium: 76

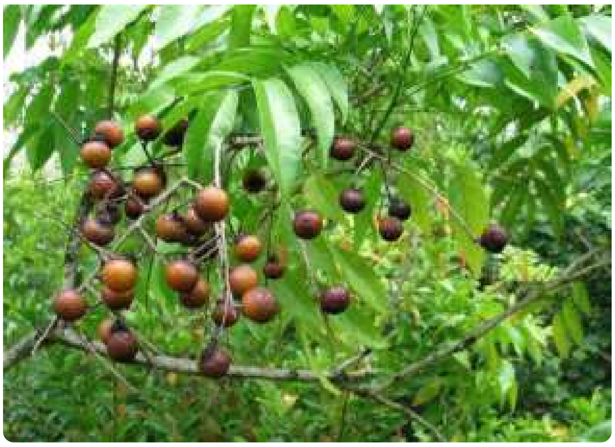

Fruiting branch. "*S. saponaria*" by D.Eickhoff (CC BY 2.0)

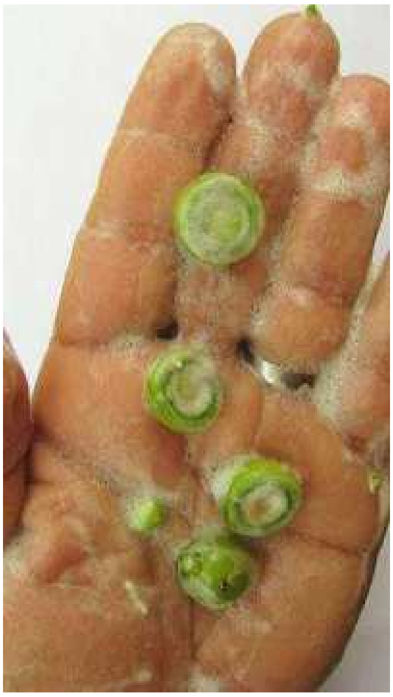

Soapy foam. "*S. saponaria*" by Alex Popovkin, Bahia, Brazil (CC BY-NC-SA 2.0)

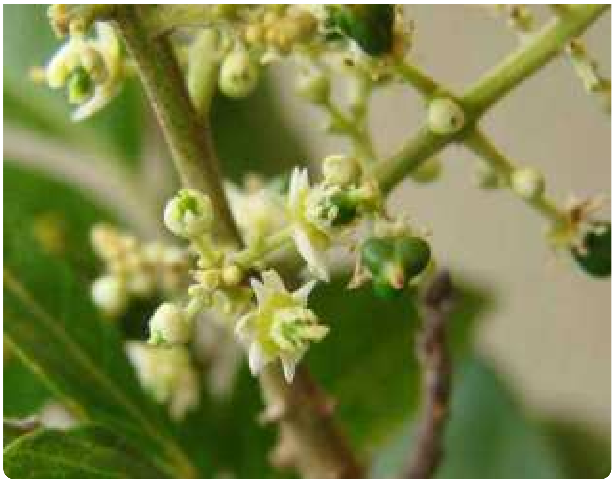

Flowering branch. "*S. saponaria*" by João de Deus Medeiros (CC BY 2.0)

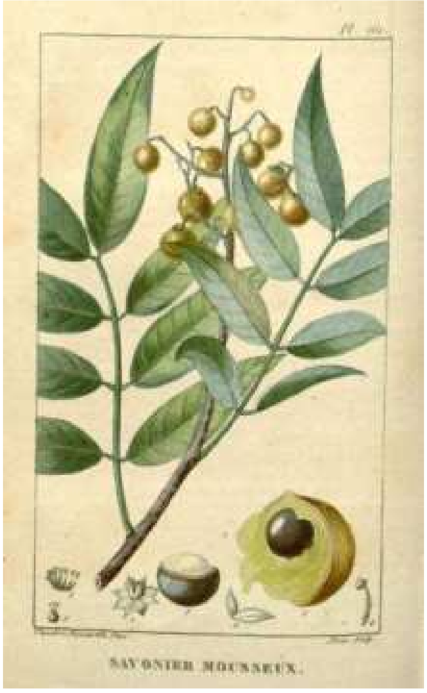

S. saponaria in *Flore [pittoresque et] médicale des Antilles* by Descourtilz, M.E. (1827: Vol. 4, t. 261)

Historiae Rerum Naturalium Brasiliae | Marcgrave, 1648 | Page number 113b

Vernacular name(s) Guity-toroba. Steen-appel

Species Pouteria grandiflora (A.DC.) Baehni

Family Sapotaceae

Notes

We did not find any correspondence between this woodcut and the contemporary or older sources. However, the botanical relative of this species, *P. caimito (*Ruiz & Pav.) Radlk, is depicted in an illustration of the *Theatrum* with the name *Ibapeba*. In the oil painting, one of the fruits on the left-down corner resembles those of *P. grandiflora*, but it does not resemble the fruit depicted in the woodcut.

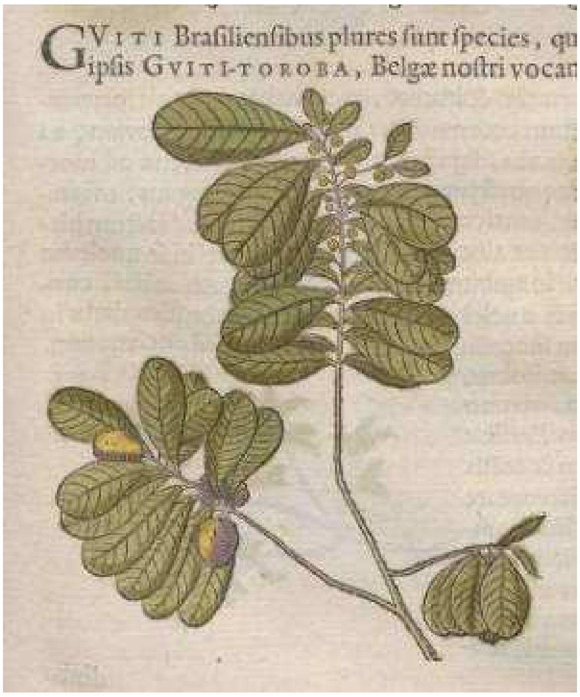

Historiae Plantarum – Arboribus: 113b

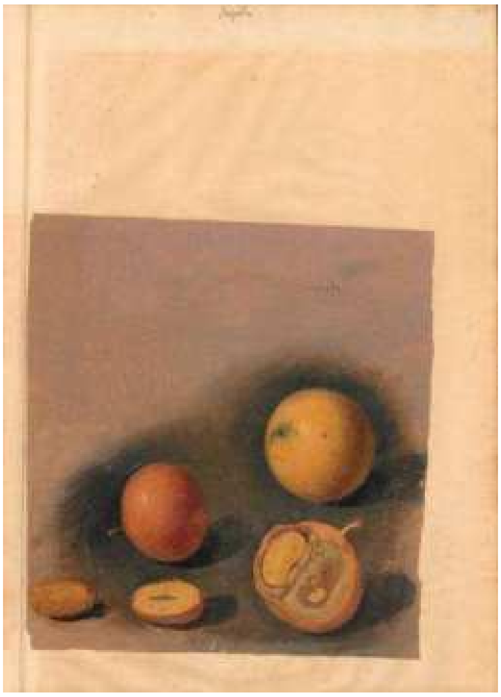

Theatrum Rerum Naturalium: 435

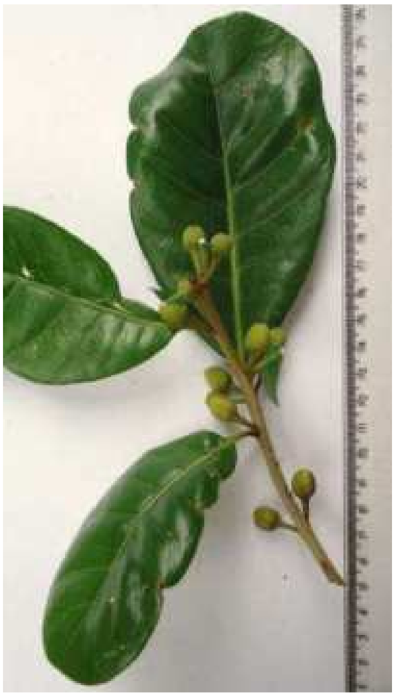

"*P. grandiflora*" by Alex Popovkin, Bahia, Brazil (CC BY-NC-SA 2.0)

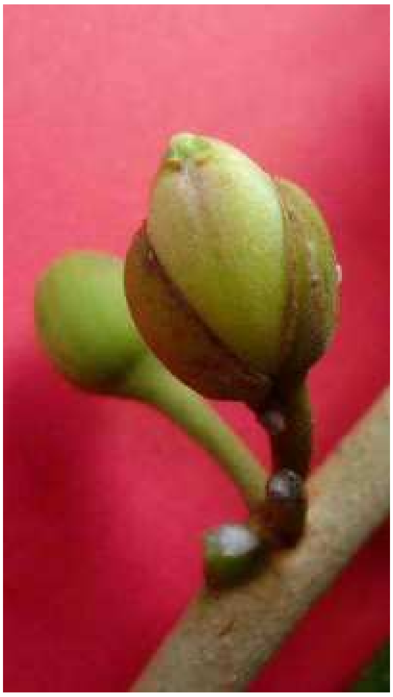

"*P. grandiflora*" by Alex Popovkin, Bahia, Brazil (CC BY-NC-SA 2.0)

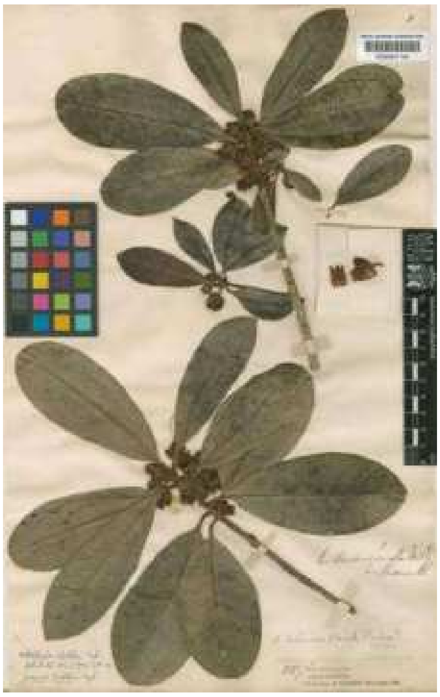

Specimen of *P. grandiflora* from Kew's Herbarium - K000641146. Retrieved from Plants of the World Online

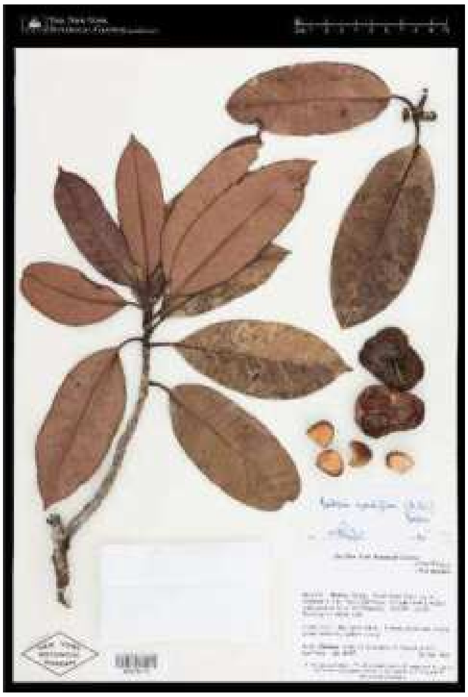

Specimen of *P. grandiflora* with fruit collected in Brazil and kept in The New York Botanical Garden - 375414- (CC BY 4.0)

Historiae Rerum Naturalium Brasiliae	Marcgrave, 1648	Page number	114a

Vernacular name(s) Guitiiba. Guity-coroya

Species Couepia rufa Ducke

Family Chrysobalanaceae

Notes

The woodcut is very similar to one of the illustrations (f. 99), although this one has been placed upside-down in the *Theatrum (*notice the shadow). In the woodcut, the fruit was placed below the branch (like in the oil painting, if we look at it in its proper position), but the branch has the same orientation as in the oil painting.

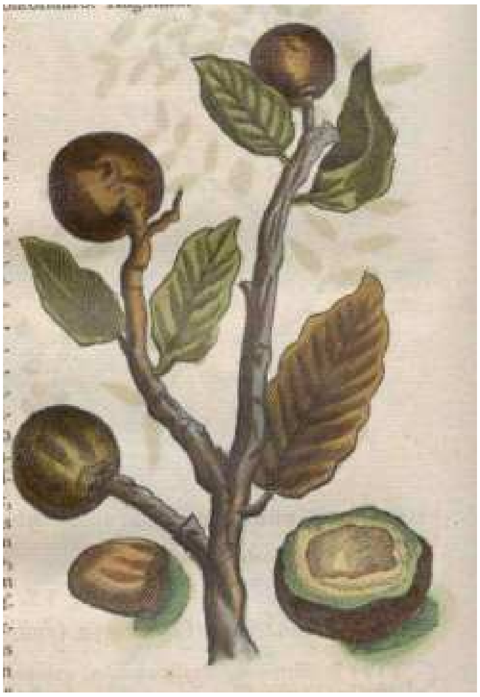

Historiae Plantarum – Arboribus: 114a

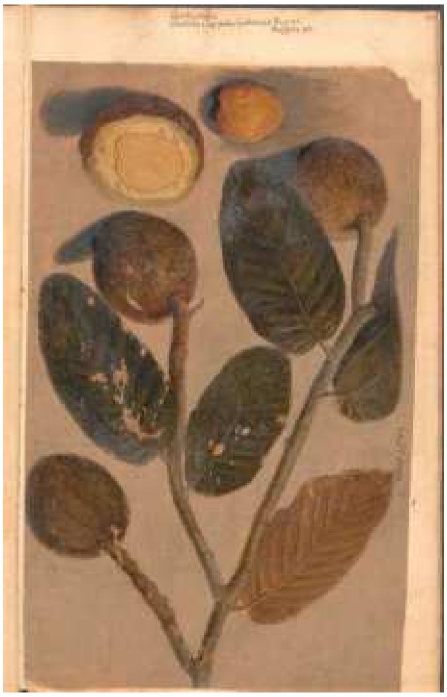

Theatrum Rerum Naturalium: 99

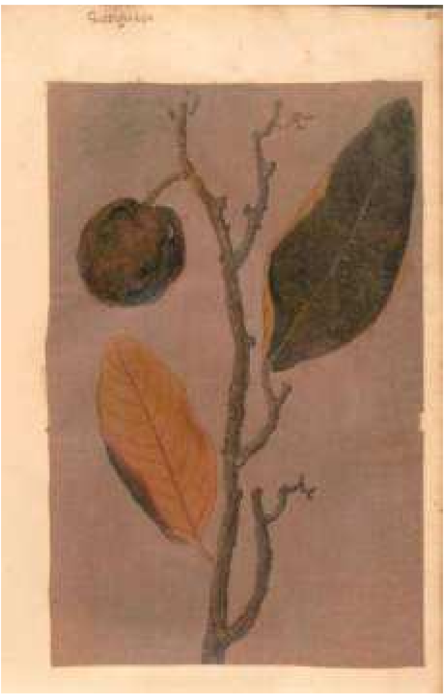

Theatrum Rerum Naturalium: 101

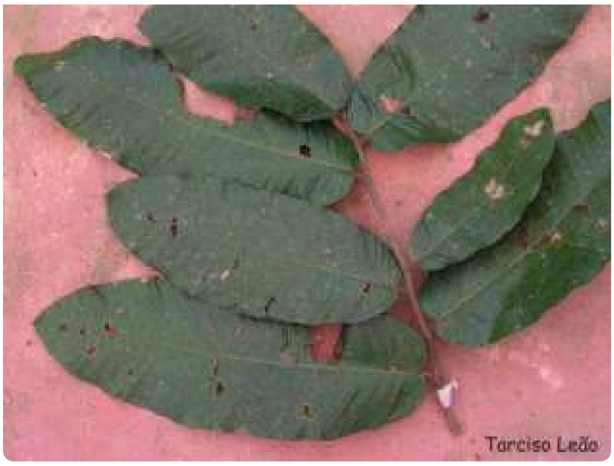

Adaxial leaves. "*C. rufa*, oiti-coró" by Tarciso Leão (CC BY 2.0)

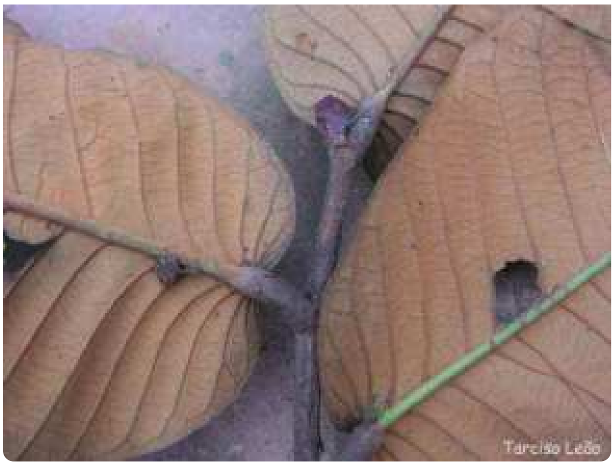

Abaxial leaves. "*C. rufa*, oiti-coró" by Tarciso Leão (CC BY 2.0)

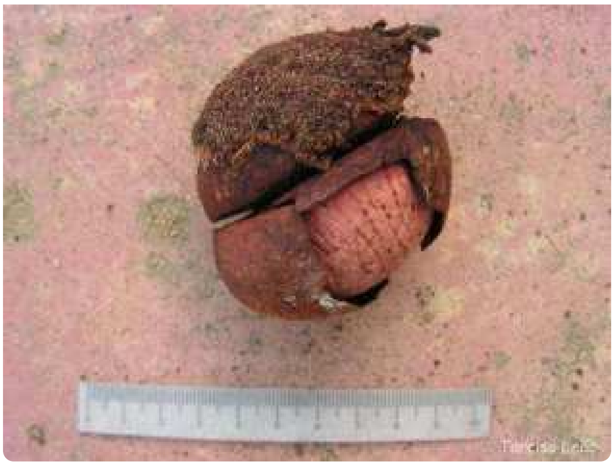

Fruit and seed. "*C. rufa*, oiti-coró" by Tarciso Leão (CC BY 2.0)

Historiae Rerum Naturalium Brasiliae Marcgrave, 1648 Page number 114b

Vernacular name(s) Ibacurapari

Species Moronobea coccinea Aubl.

Family Clusiaceae

Notes

We did not find any correspondence between this woodcut and the contemporary or older sources.

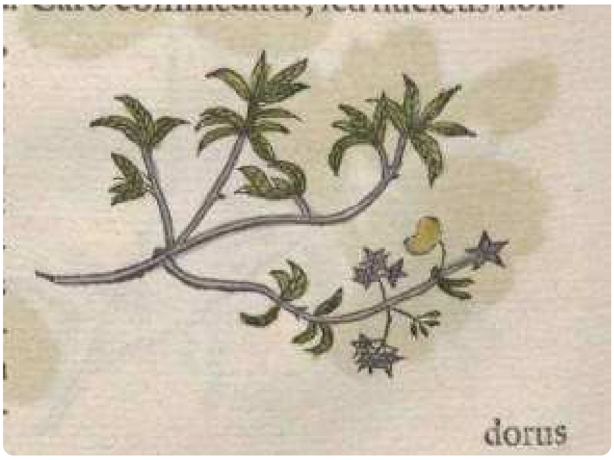

Historiae Plantarum – Arboribus: 114b

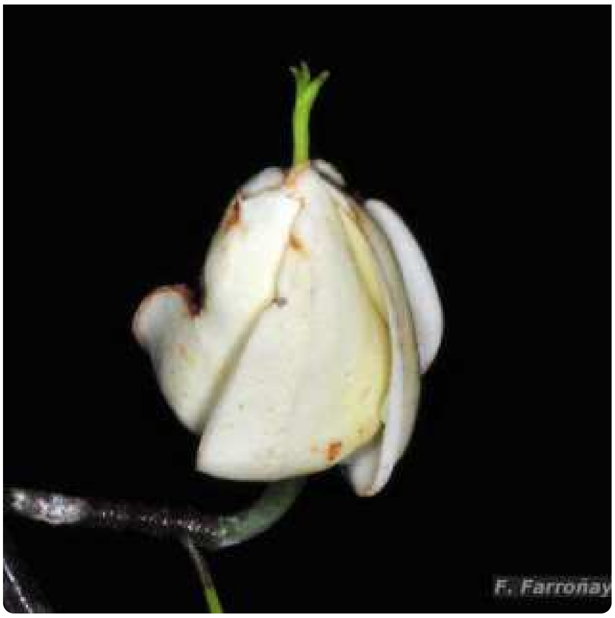

Flower of *M. coccinea* by F. Farroñay. Published by Lucas Cardoso Marinho in Flora e Funga do Brasil.

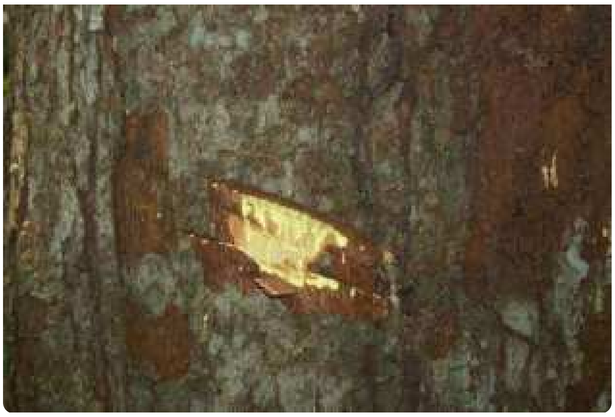

Wood. *M. coccinea* - Specimen D. Sasaki et al. 2552. ID:1104412 © RBG Kew (CC BY 3.0). Retrieved from Plants of the World Online

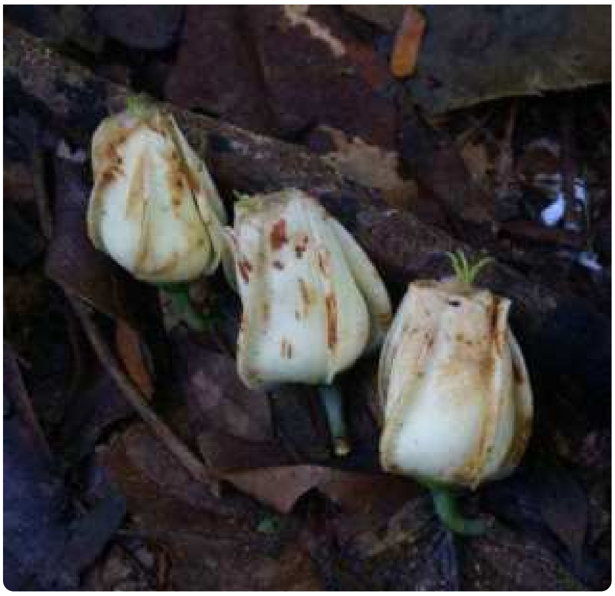

Flowers. *M. coccinea* - Specimen D. Sasaki et al. 2552. ID:1104414 © RBG Kew (CC BY 3.0). Retrieved from Plants of the World Online

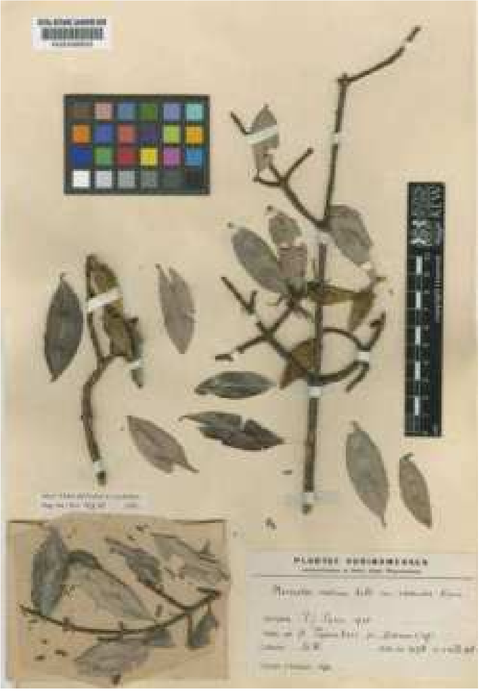

Specimen of *M. coccinea* from Kew's Herbarium - K000488600. Retrieved from Plants of the World Online

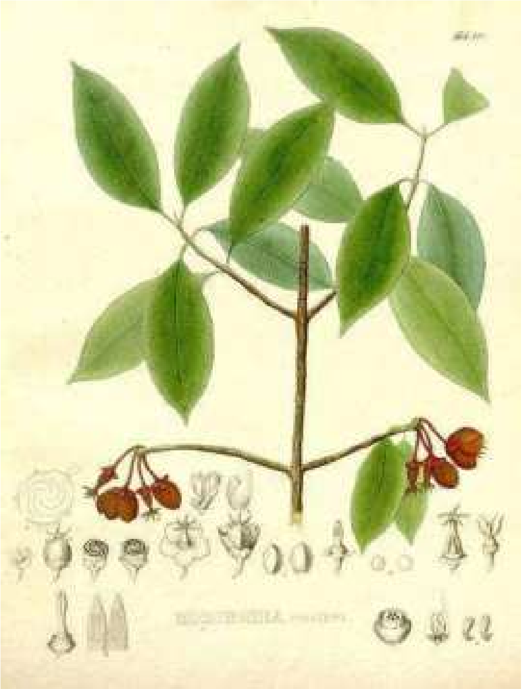

M. coccinea in *Nova genera et species plantarum Brasiliensium* by Martius, C.P.F. von (1829: Vol. III, t. 287)

Historiae Rerum Naturalium Brasiliae	Marcgrave, 1648	Page number	115a

Vernacular name(s) Guiti iba (Guiti miri fruit)

Species Moquilea tomentosa Benth.

Family Chrysobalanaceae

Notes

We did not find any correspondence between this woodcut and the contemporary or older sources.

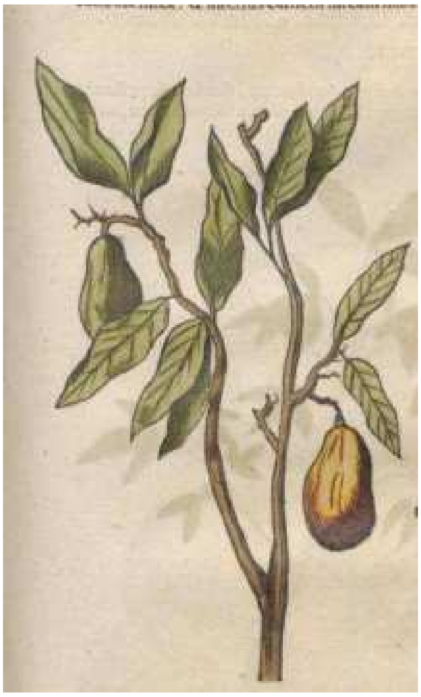

Historiae Plantarum – Arboribus: 115a

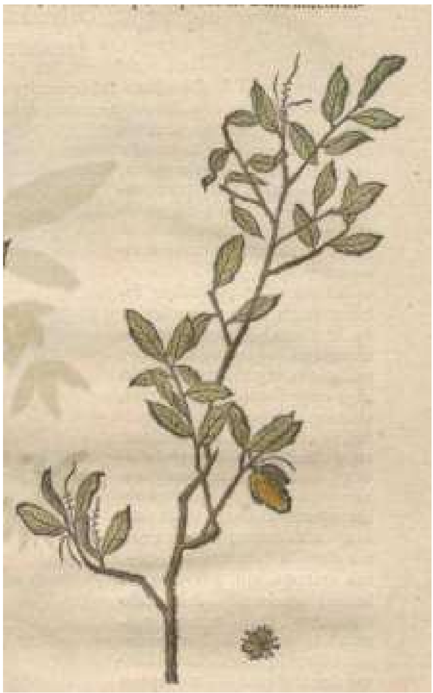

M. tomentosa depicted together with the other woodcut for the same species in Marcgrave (HNB, 1648: 115b)

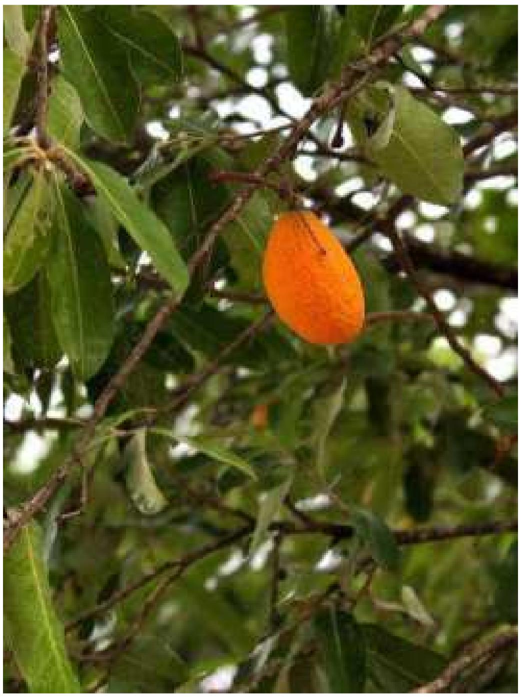

Fruiting branch. "*Licania tomentosa* (syn. of *M. tomentosa*)" by Mauricio Mercadante (CC BY-NC-SA 2.0)

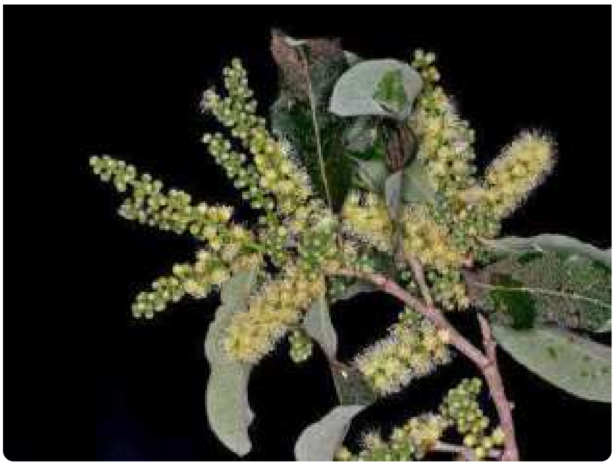

Flowering branch. "*Licania tomentosa* (syn. of *M. tomentosa*)" by Mauricio Mercadante (CC BY-NC-SA 2.0)

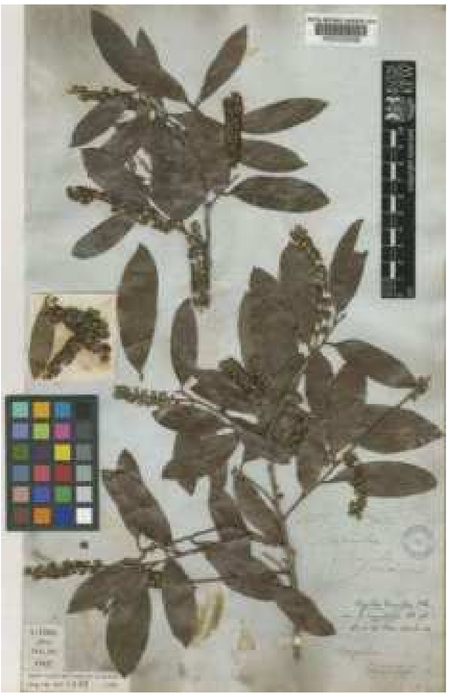

Flowering specimen of *M. tomentosa* from Kew's Herbarium - K000220598. Retrieved from Plants of the World Online

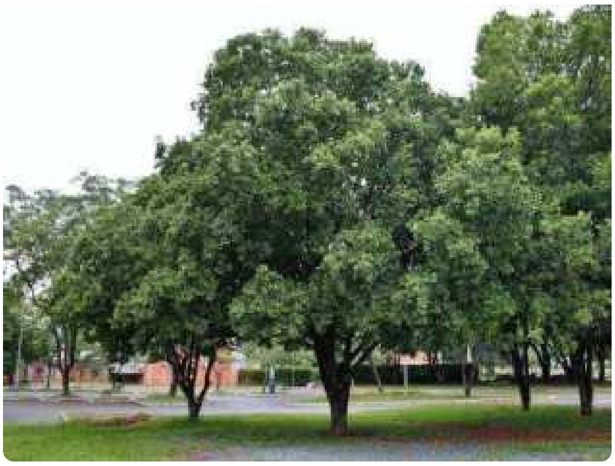

Habit. "*Licania tomentosa* (syn. of *M. tomentosa*)" by Mauricio Mercadante (CC BY-NC-SA 2.0)

Historiae Rerum Naturalium Brasiliae | Marcgrave, 1648 | Page number 115b

Vernacular name(s) Guiti iba (Guiti miri fruit)

Species Moquilea tomentosa Benth.

Family Chrysobalanaceae

Notes

We did not find any correspondence between this woodcut and the contemporary or older sources.

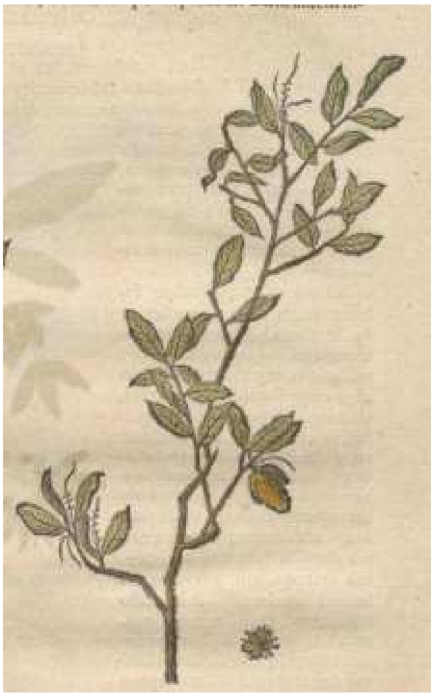

Historiae Plantarum – Arboribus: 115b

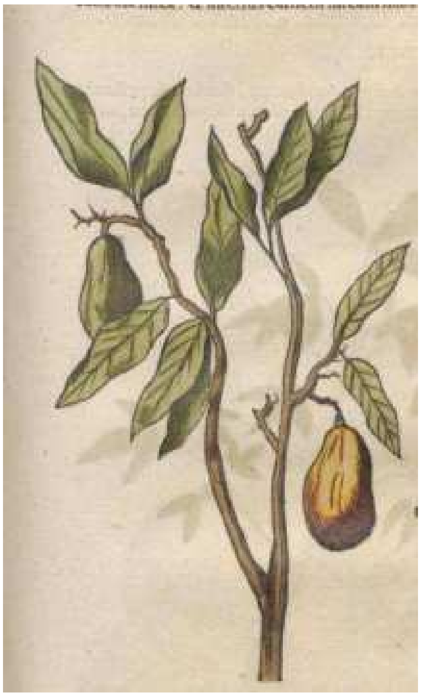

M. tomentosa depicted together with the other woodcut for the same species in Marcgrave (HNB, 1648: 115a)

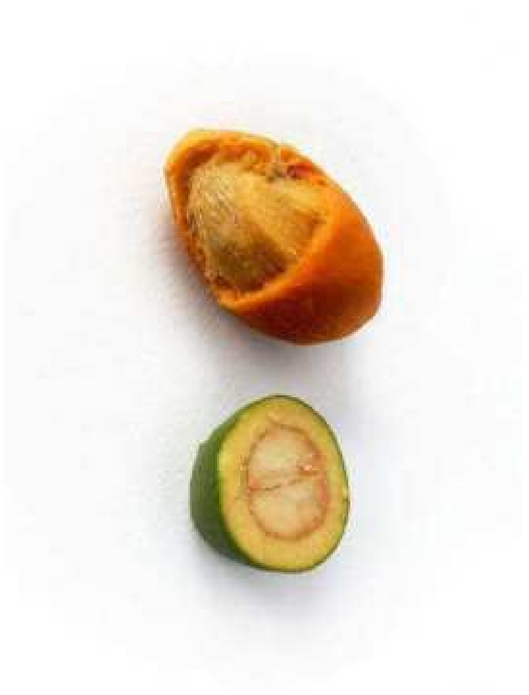

Fruit and seed. "*Licania tomentosa* (syn. of *M. tomentosa*)" by Mauricio Mercadante (CC BY-NC-SA 2.0)

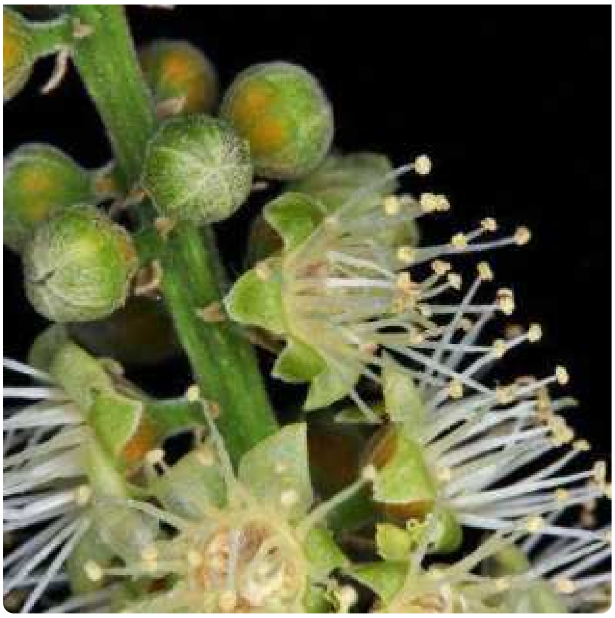

Flower's buds and stamens. "*Licania tomentosa* (syn. of *M. tomentosa*)" by Mauricio Mercadante (CC BY-NC-SA 2.0)

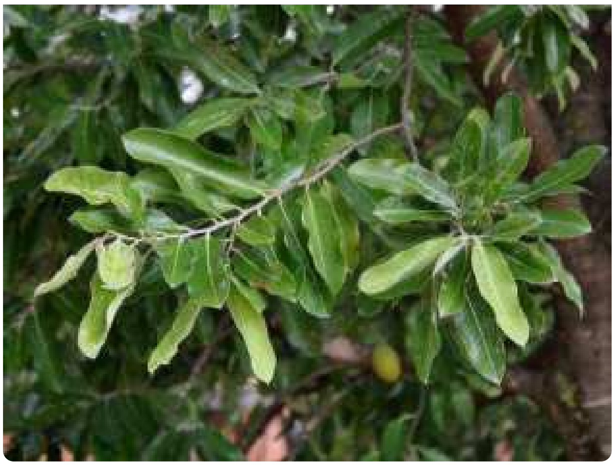

Branch. "*Licania tomentosa* (syn. of *M. tomentosa*)" by Mauricio Mercadante (CC BY-NC-SA 2.0)

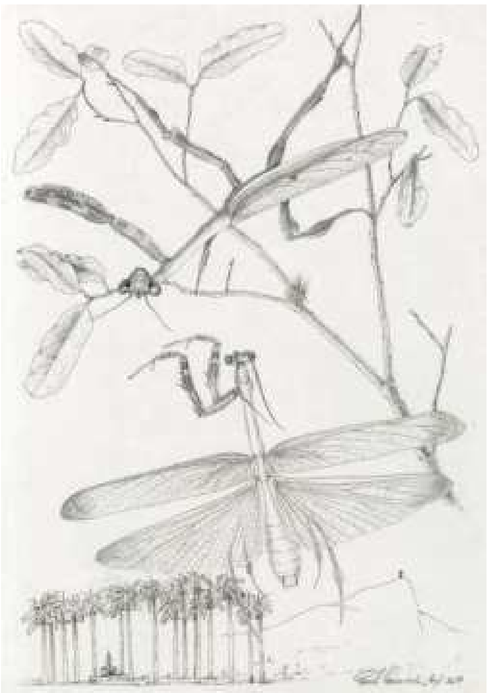

"*Vates phoenix* female perching on *M. tomentosa* (*oiti*). A male specimen is depicted flying". Scientific illustration by Paulo Ormindo (Rivera et al. 2020)

Historiae Rerum Naturalium Brasiliae Marcgrave, 1648 Page number 116a

Vernacular name(s) Ibapurunga

Species Vitex rufescens A.Juss.

Family Lamiaceae

Notes

The woodcut differs from the *Theatrum* illustration.

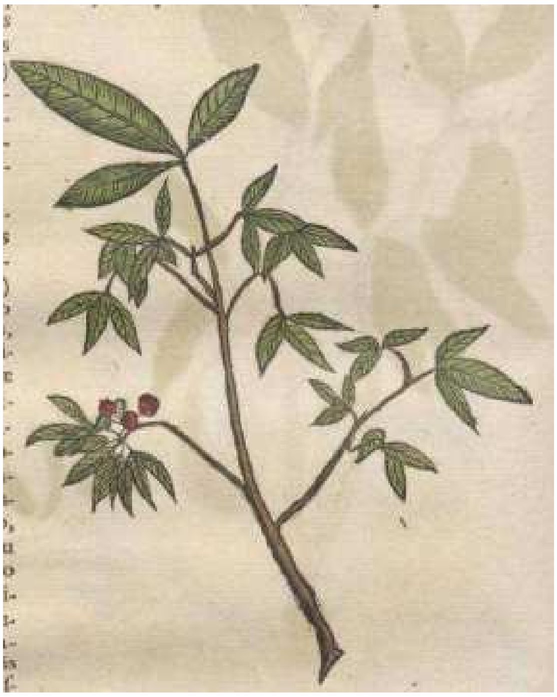

Historiae Plantarum – Arboribus: 116a

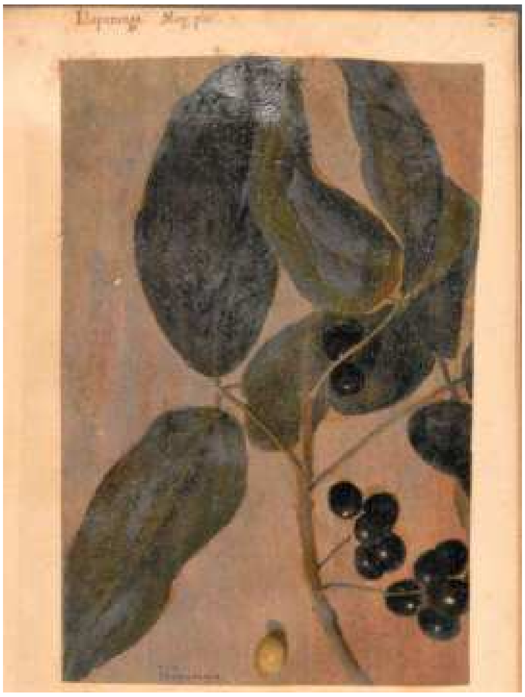

Theatrum Rerum Naturalium: 279

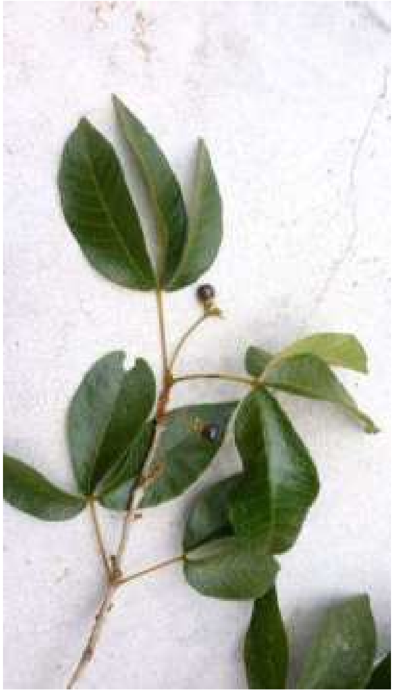

Fruiting branch. "*V. rufescens*" by Alex Popovkin, Bahia, Brazil (CC BY-NC-SA 2.0)

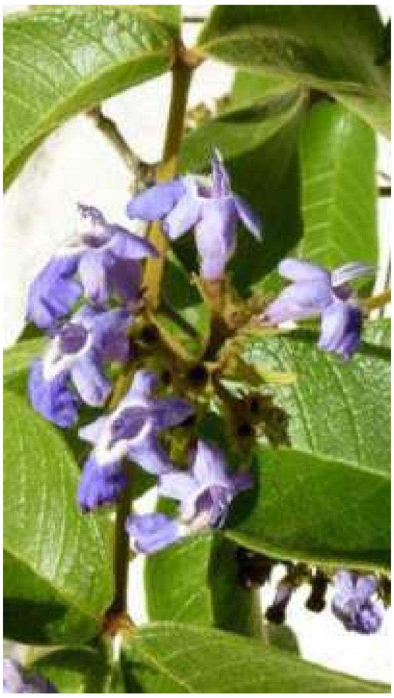

Flowering branch. "*V. rufescens*" by Alex Popovkin, Bahia, Brazil (CC BY-NC-SA 2.0)

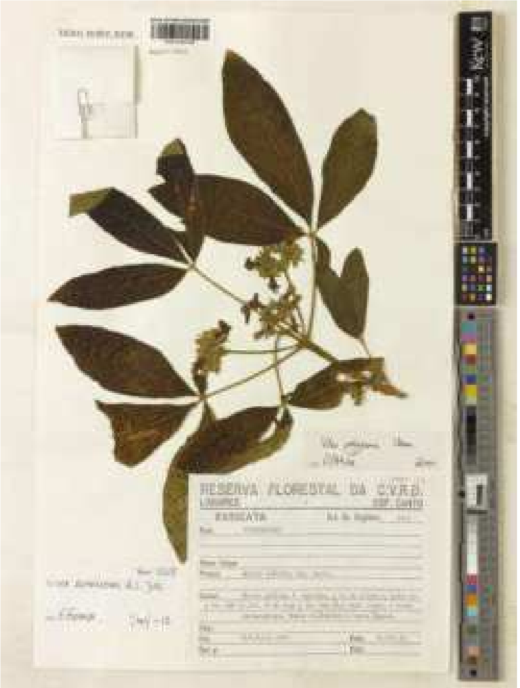

Flowering specimen of *V. rufescens* from Kew's Herbarium - K001220184. Retrieved from Plants of the World Online

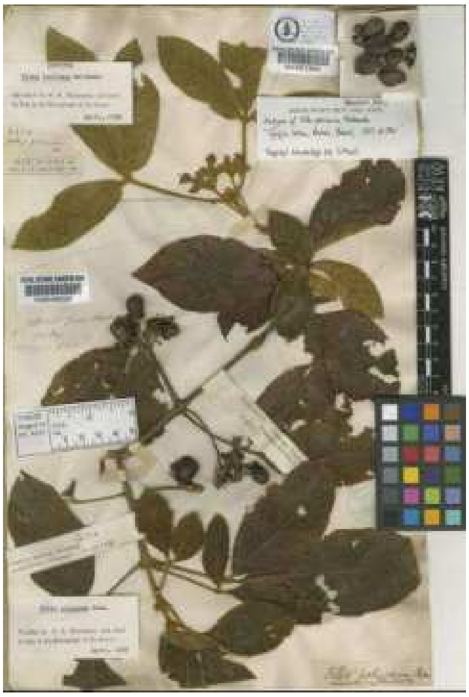

Fruiting specimen of *V. rufescens* from Kew's Herbarium - K000488020. Retrieved from Plants of the World Online

Historiae Rerum Naturalium Brasiliae	Marcgrave, 1648	Page number	116b

Vernacular name(s) Ibipitanga

Species Eugenia uniflora L.

Family Myrtaceae

Notes

The woodcut differs from the other sources. This species was represented in p. 293 of the HNB with a different woodcut (see the other entry for *E. uniflora*).

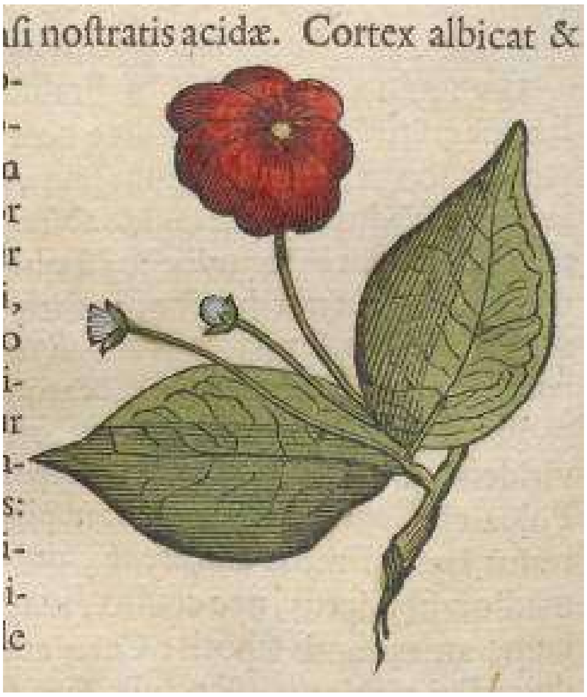

Historiae Plantarum – Arboribus: 116b

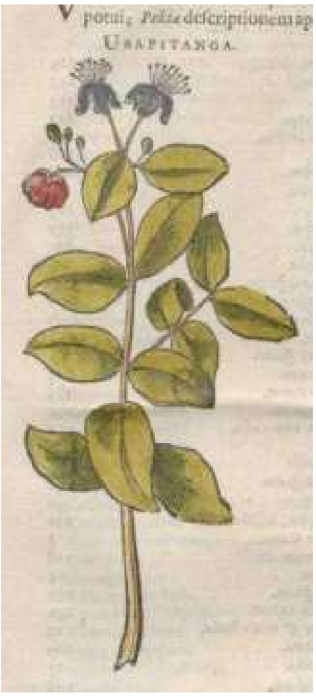

E. uniflora depicted differently in Marcgrave (HNB, 1648: 293)

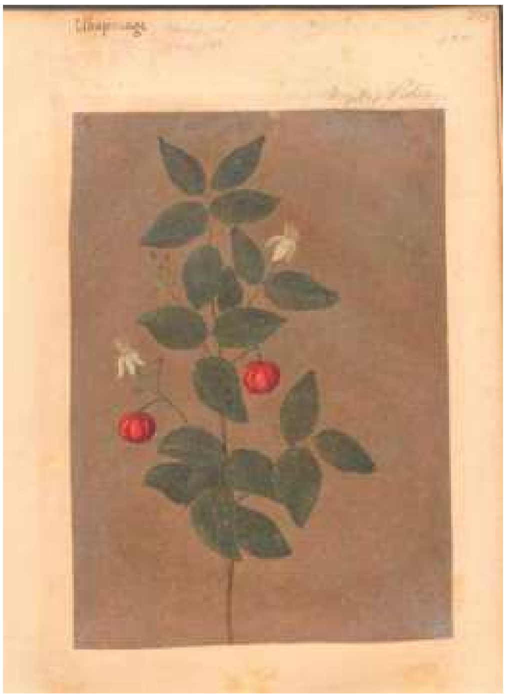

Theatrum Rerum Naturalium: 339

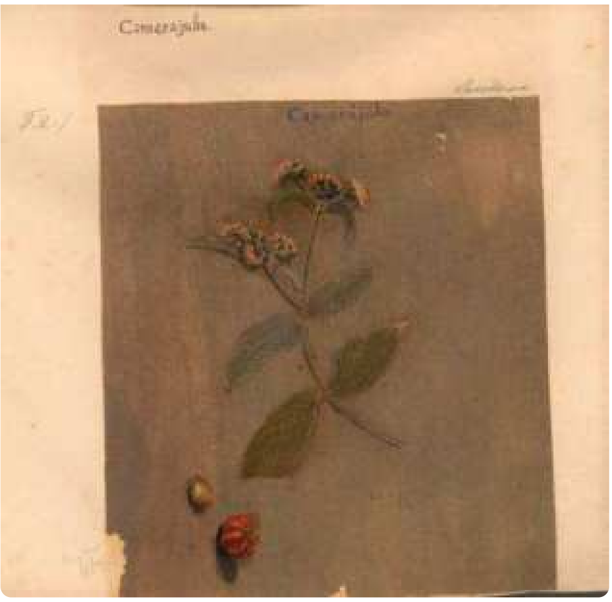

Theatrum Rerum Naturalium: 341 (only the fruit in the left bottom corner)

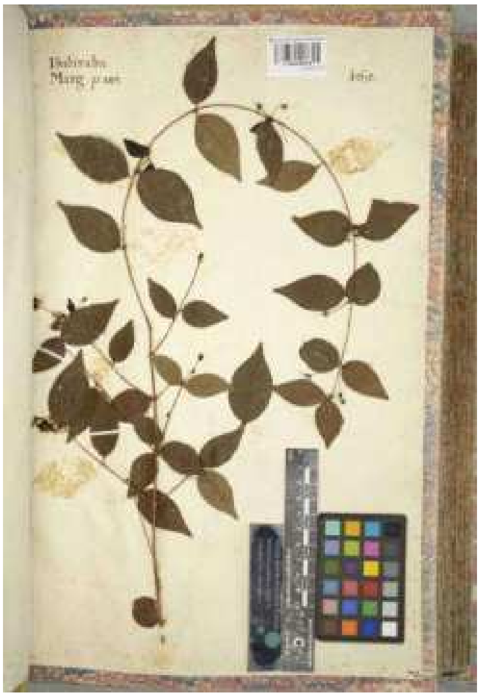

Marcgrave's herbarium: 70

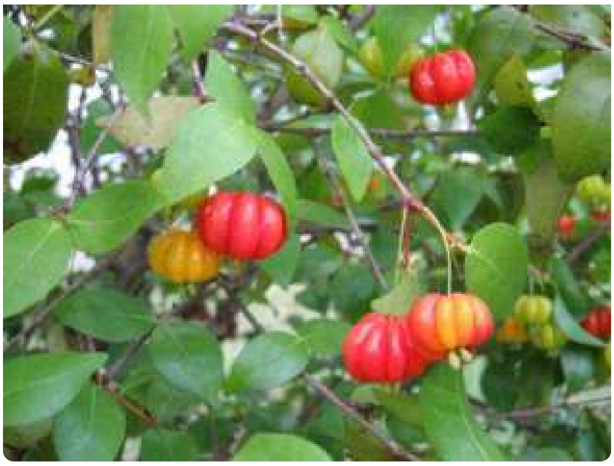

"Pitanga (*E. uniflora*).JPG" by Michael Hermann (CC BY-SA 3.0)

Historiae Rerum Naturalium Brasiliae | Marcgrave, 1648 | Page number 117a

Vernacular name(s) Ibabiraba

Species Campomanesia dichotoma (O.Berg) Mattos

Family Myrtaceae

Notes

The woodcut is different than the other sources. The proof-woodcut in De Laet's manuscript corresponds to the woodcut used in Piso (1648: 73) for the same species (although named differently: *Guabiraba*).

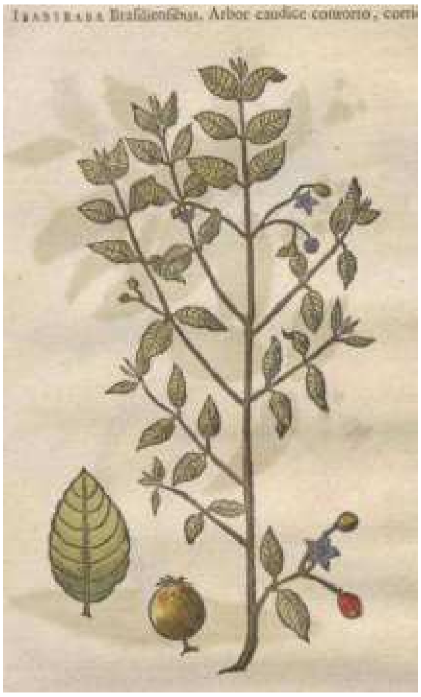

Historiae Plantarum – Arboribus: 117a

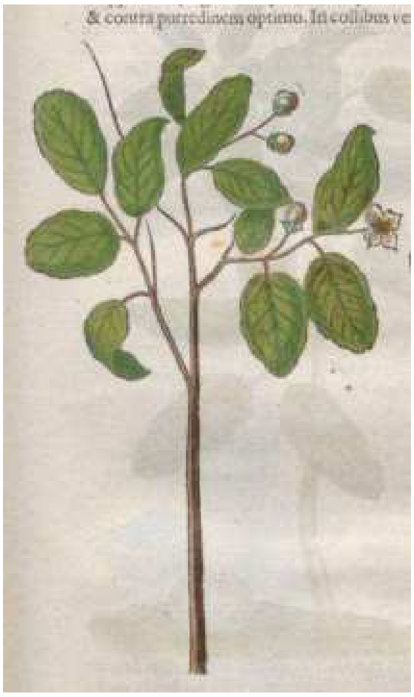

C. dichotoma depicted differently in Piso (HNB, 1648: 73a)

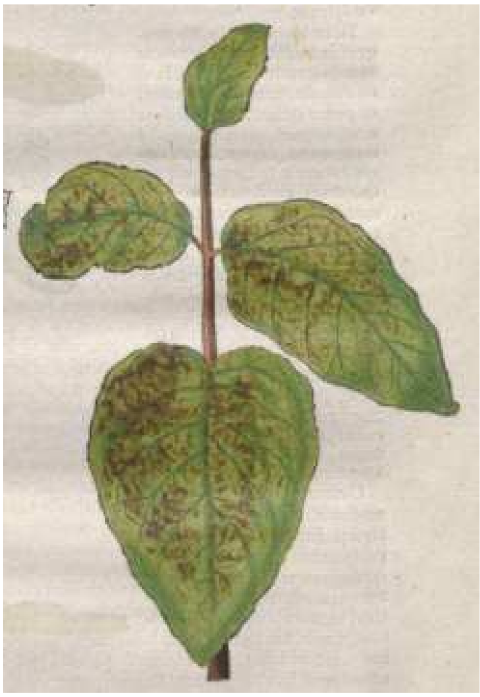

C. dichotoma depicted differently in Piso (HNB, 1648: 73b)

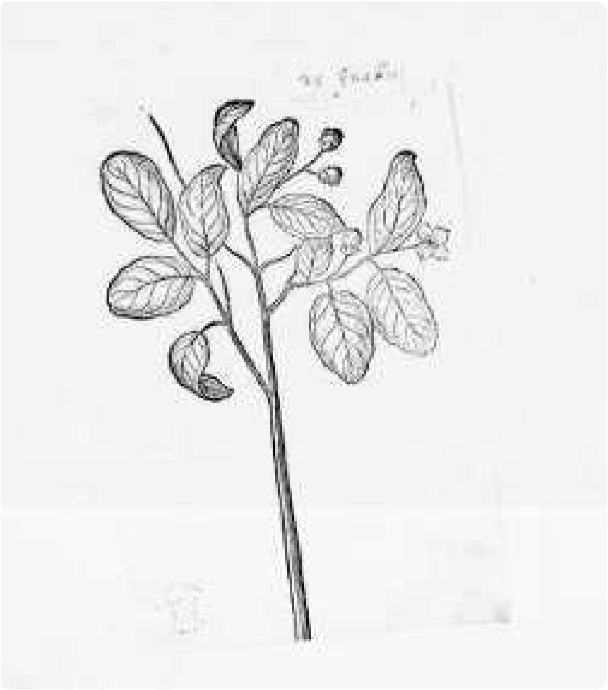

Proof woodcut of *C. dichotoma* in De Laet's manuscript: Sloane MS 1554, f. 65v

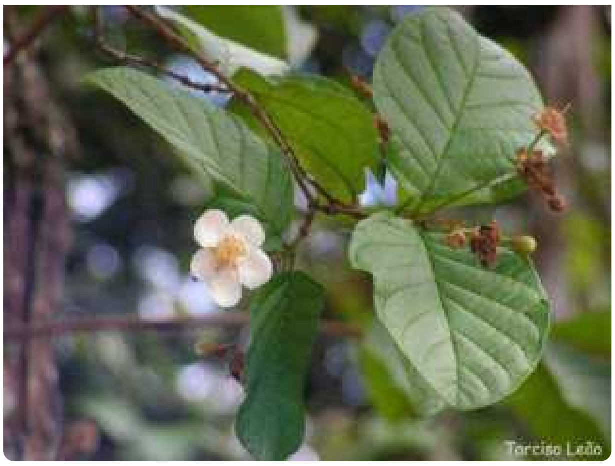

Flowering branch. "*C. dichotoma*, *Guabiraba*" by Tarciso Leão (CC BY 2.0)

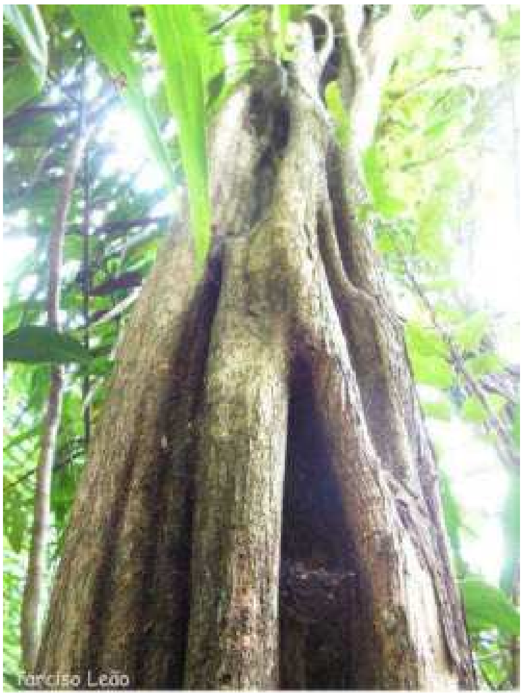

Habit. "*C. dichotoma*, *Guabiraba*" by Tarciso Leão (CC BY 2.0)

Historiae Rerum Naturalium Brasiliae | Marcgrave, 1648 | Page number 117b

Vernacular name(s) Muiva

Species <u>Miconia holosericea (L.) DC</u>

Family Melastomataceae

Notes

There is no strong resemblance between the specimen and the woodcut, although both show the abaxial and adaxial sides of the leaves.

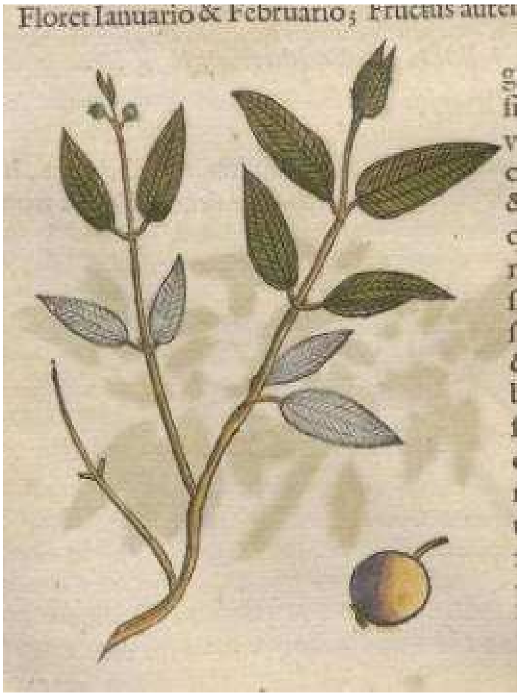

Historiae Plantarum – Arboribus: 117b

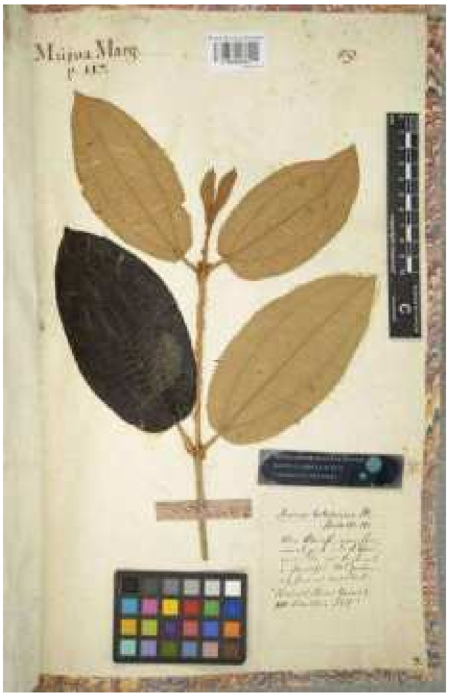

Marcgrave's herbarium: 3

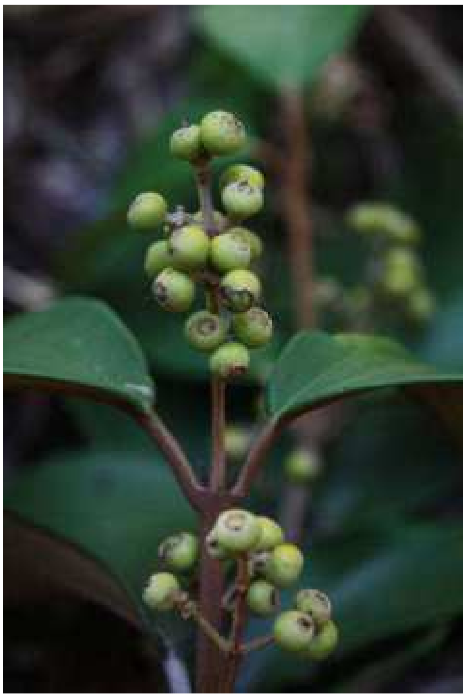

M. holosericea - Specimen D.C. Zappi 3133. ID:1108193 © RBG Kew (CC BY 3.0)

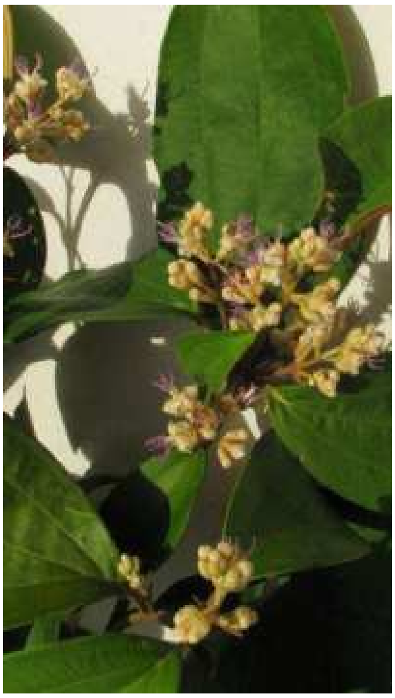

"*M. holosericea*" by Alex Popovkin, Bahia, Brazil (CC BY-NC-SA 2.0)

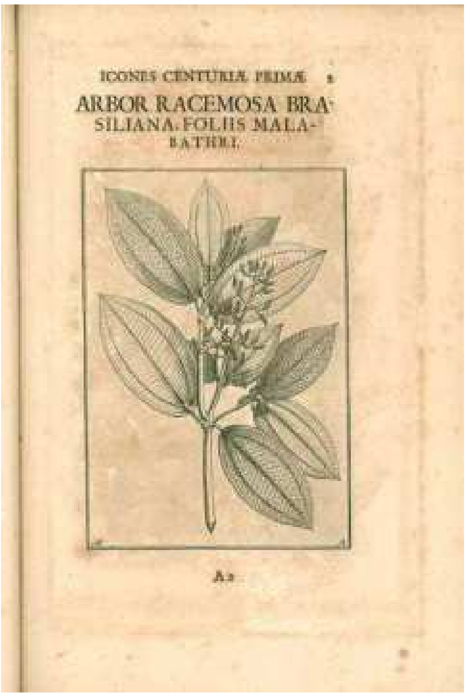

Exoticarum aliarumque minus cognitarum plantarum Centuria prima by Breyne, J. (1678: t.2). Universitäts und Landesbibliothek Sachsen-Anhalt, Halle

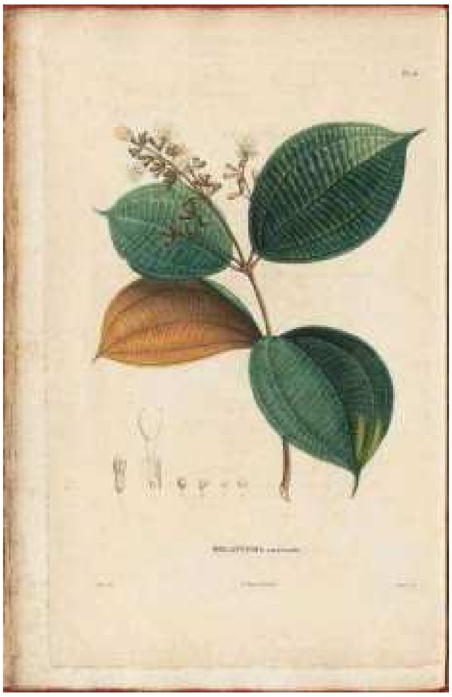

M. holosericea in *Monographia Melastomacearum* by Humboldt, F.H.A. von, Bonpland, A. (1816: Vol. I, t. 18). Missouri Botanical Garden, St. Louis, U.S.A.

Historiae Rerum Naturalium Brasiliae Marcgrave, 1648 Page number 118a

Vernacular name(s) Mureci

Species Byrsonima cydoniifolia A.Juss.

Family Malpighiaceae

Notes

The woodcut is very similar to the *Theatrum* image (non-reversed). There is no resemblance with the specimen. The oil-based illustration is densely crowded with more leaves. In this image, there is a pencil annotation below the vernacular name *Mureçî* with some numbers, including the number 93, which matches the annotation in De Laet's manuscript "in lib. 93".

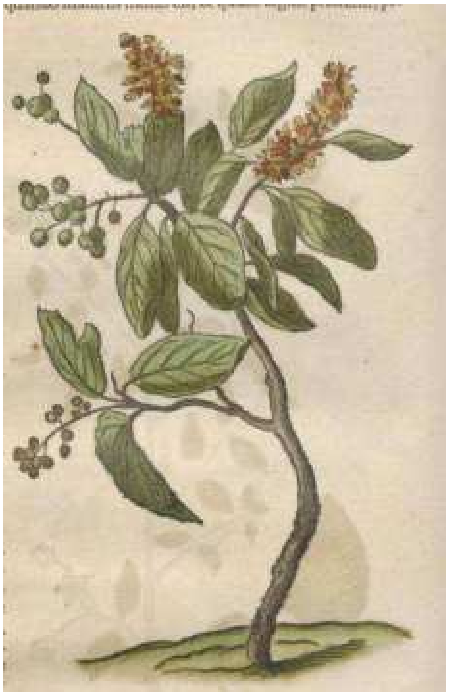

Historiae Plantarum – Arboribus: 118a

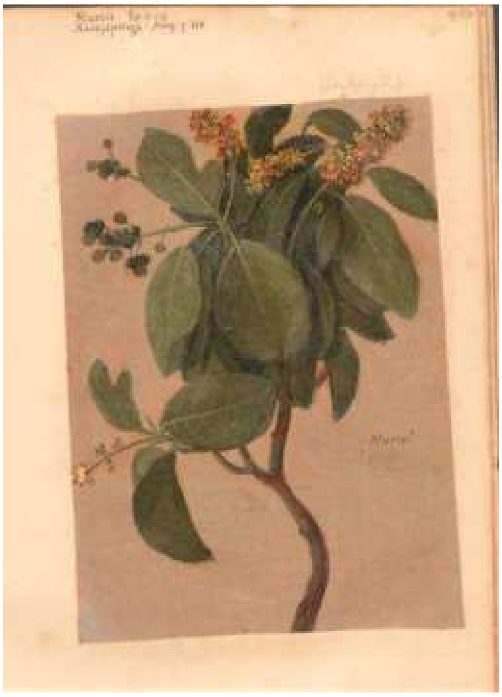

Theatrum Rerum Naturalium: 477

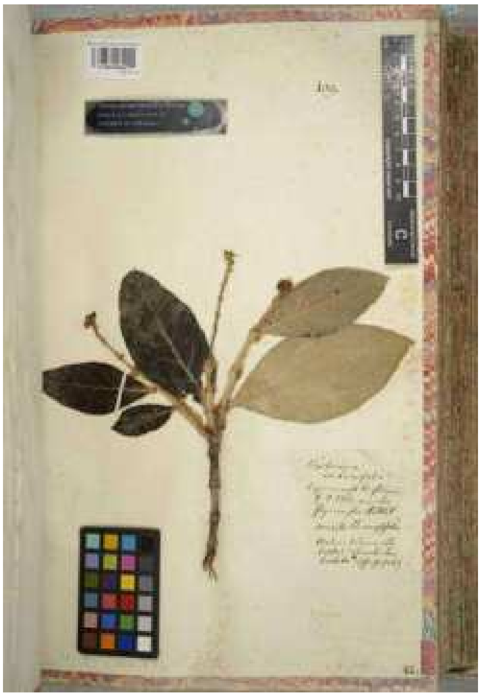

Marcgrave's herbarium: 82

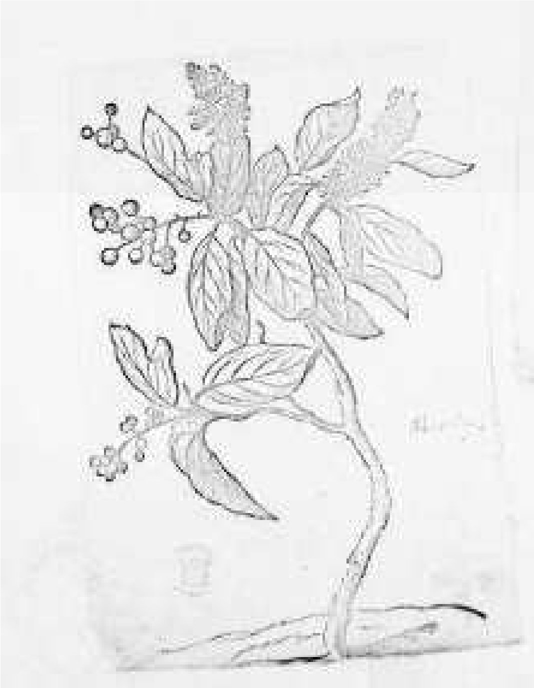

Proof woodcut of *B. cydoniifolia* in De Laet's manuscript: Sloane MS 1554, f. 60v

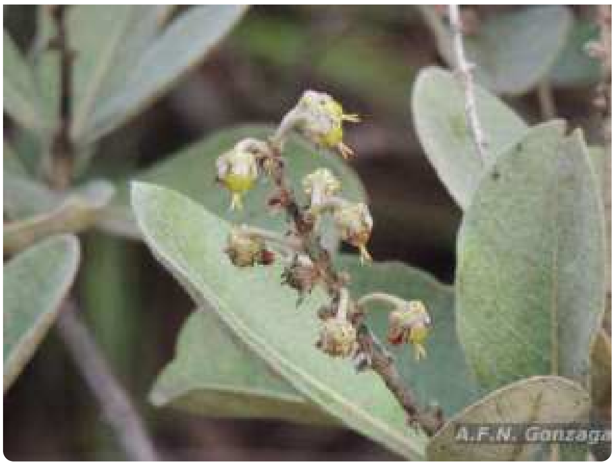

B. cydoniifolia by A.F.N. Gonzaga. Published by Augusto Francener in Flora e Funga do Brasil

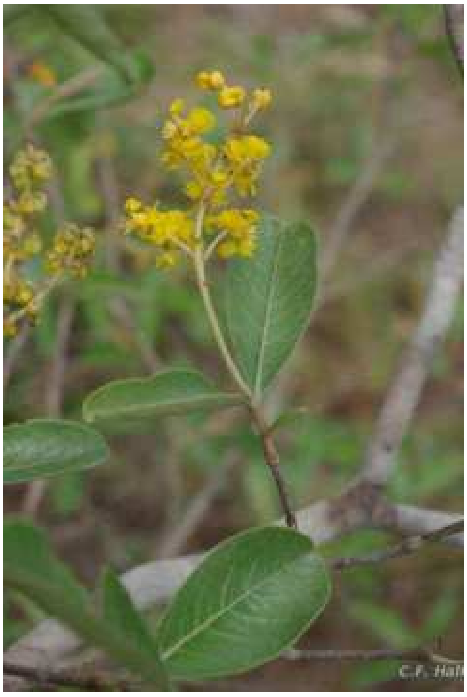

B. cydoniifolia by C.F. Hall. Published by Augusto Francener in Flora e Funga do Brasil

Historiae Rerum Naturalium Brasiliae | Marcgrave, 1648 | Page number 118b

Vernacular name(s): Guirapariba. Urupariba. Pao d' Arco

Species: Handroanthus serratifolius (Vahl) S.O.Grose

Family: Bignoniaceae

Notes

We did not find any correspondence between this woodcut and the contemporary or older sources.

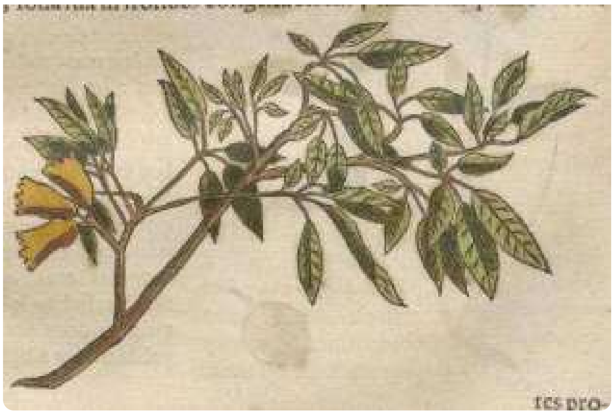

Historiae Plantarum – Arboribus: 118b

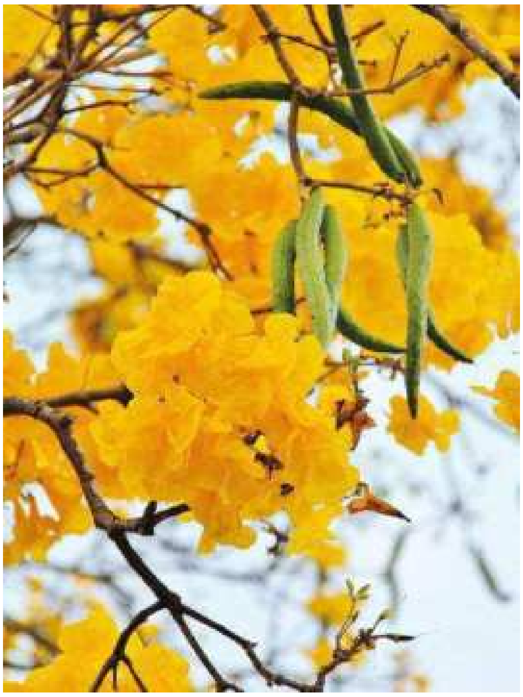

Flowers and pods. "*H. serratifolius*" by Mauricio Mercadante (CC BY-NC-SA 2.0)

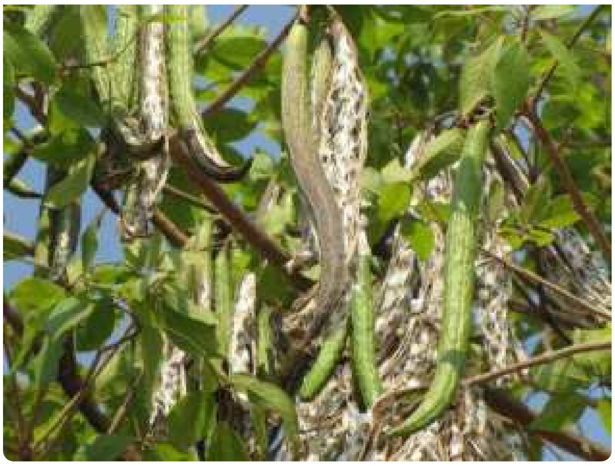

Pods and seeds. "*H. serratifolius*" by Mauricio Mercadante (CC BY-NC-SA 2.0)

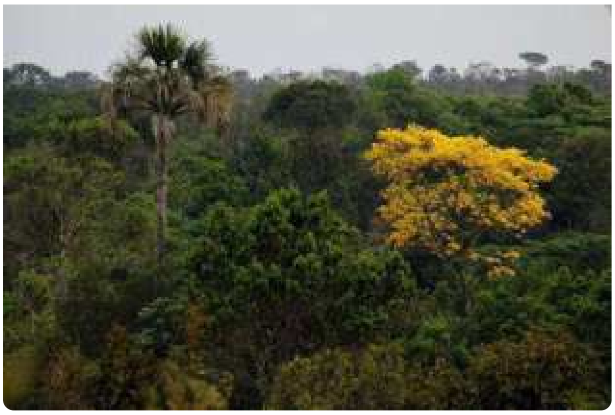

Tree with yellow flowers on the right. "*H. serratifolius*" by Mauricio Mercadante, Jardim Botânico, Brasília, Brasil (CC BY-NC-SA 2.0)

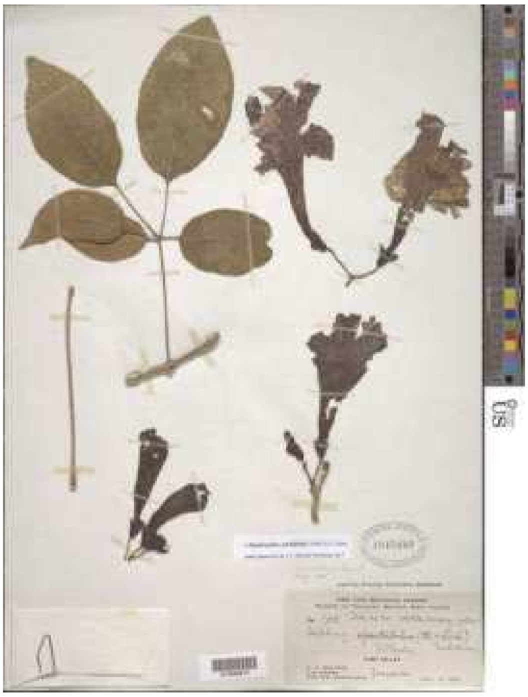

Flowering specimen of *H. serratifolius* -01068815- Smithsonian National Museum of Natural History (CC0 1.0)

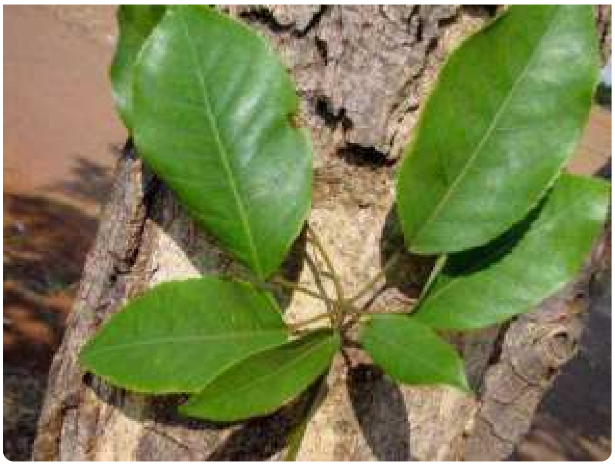

Leaf. "*H. serratifolius*" by Mauricio Mercadante (CC BY-NC-SA 2.0)

Historiae Rerum Naturalium Brasiliae | Marcgrave, 1648 | Page number 119a

Vernacular name(s) Tataiiba

Species Maclura tinctoria (L.) D.Don ex Steud.

Family Moraceae

Notes

The woodcut is very similar to the *Theatrum* image (in reversed format). Both images show a broken/bitten leaf, which gives realism and dimensionality to the plant depiction. The woodcut does not show the spines in the stem; while the oil painting depicts them clearly and it shows an open fruit as well, which is lacking in the HNB. There is no resemblance with the specimens, although one of them (p. 136) does not bear spines, as in the woodcut.

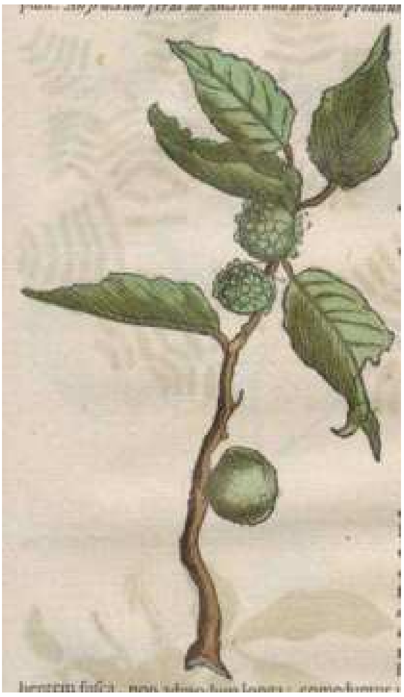

Historiae Plantarum – Arboribus: 119a

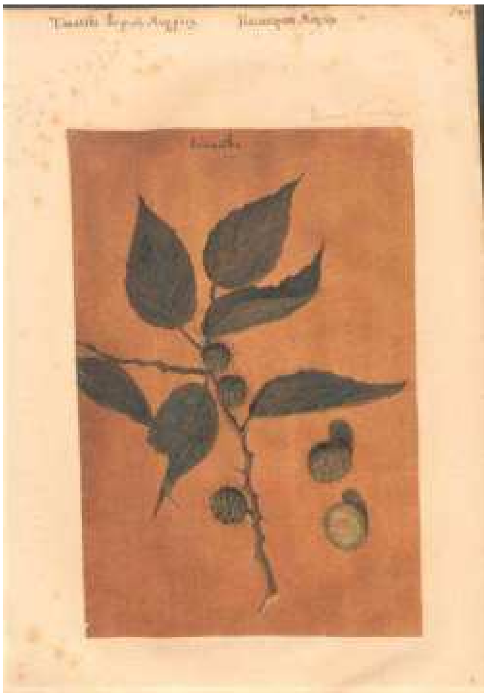

Theatrum Rerum Naturalium: 349

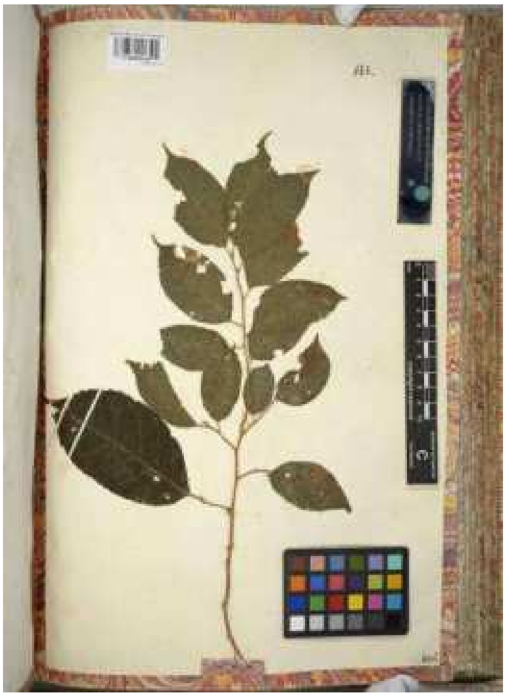

Marcgrave's herbarium: 136

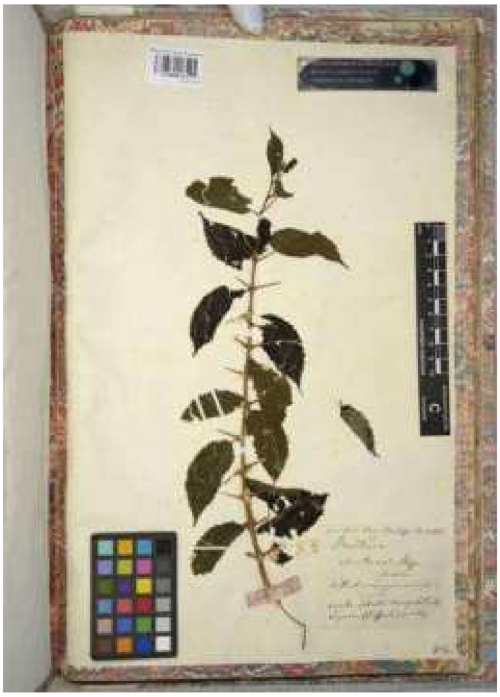

Marcgrave's herbarium: 172

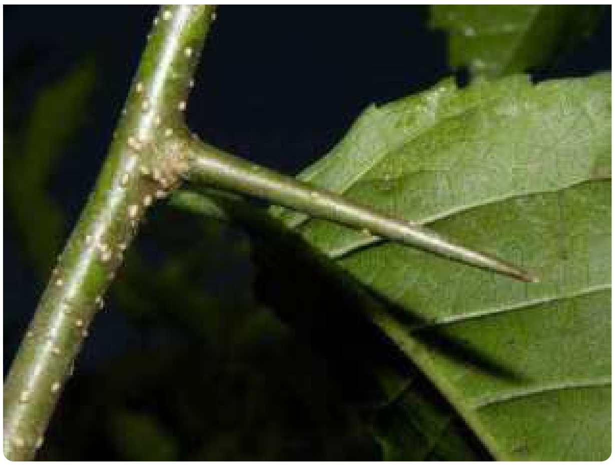

"*M. tinctoria*" by Reinaldo Aguilar (CC BY-NC-SA 2.0)

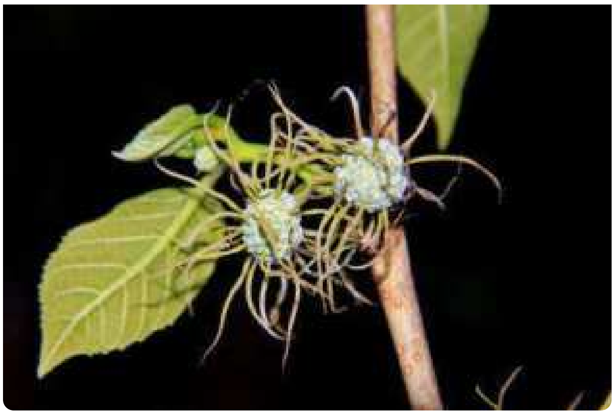

"Amora-branca - *M. tinctoria*" by Marcelo_Kuhlman (CC BY-NC-SA 2.0)

Historiae Rerum Naturalium Brasiliae | Marcgrave, 1648 | Page number 119b

Vernacular name(s) Tataiiba

Species Maclura tinctoria (L.) D.Don ex Steud.

Family Moraceae

Notes

This woodcut is greatly different than the woodcut next to it for the same species (see previous entry), although both depict a fruiting branch, in contrast to the sterile specimens in the herbarium. Marcgrave likely did not see the flower, as De Laet did not find any description of it (Marcgrave 1648: 119), nor it is shown in any of the above-mentioned sources.

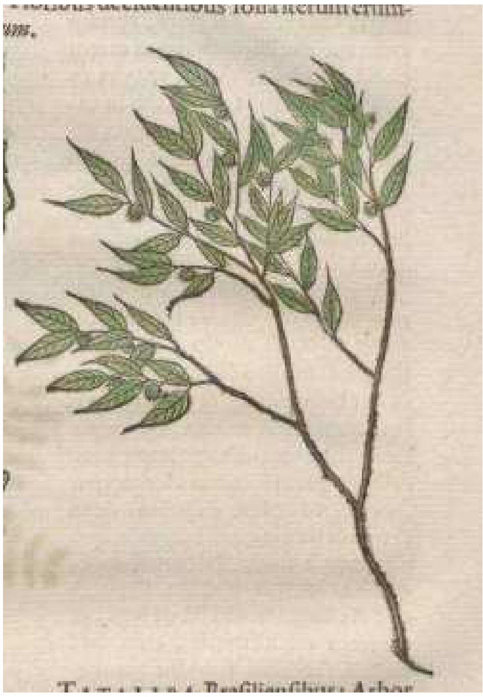

Historiae Plantarum – Arboribus: 119b

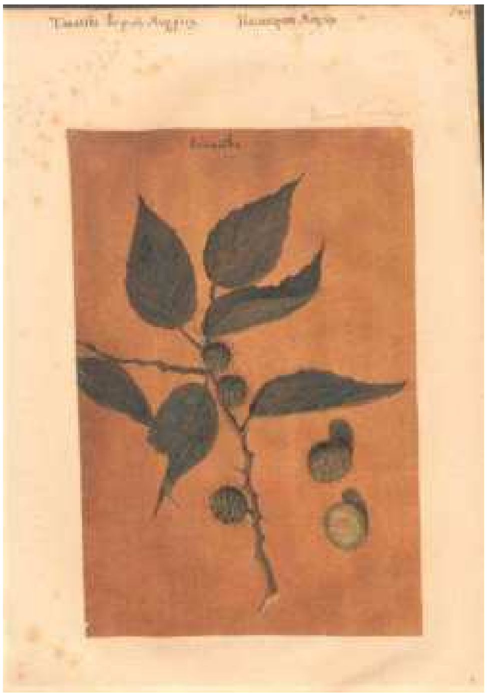

Theatrum Rerum Naturalium: 349

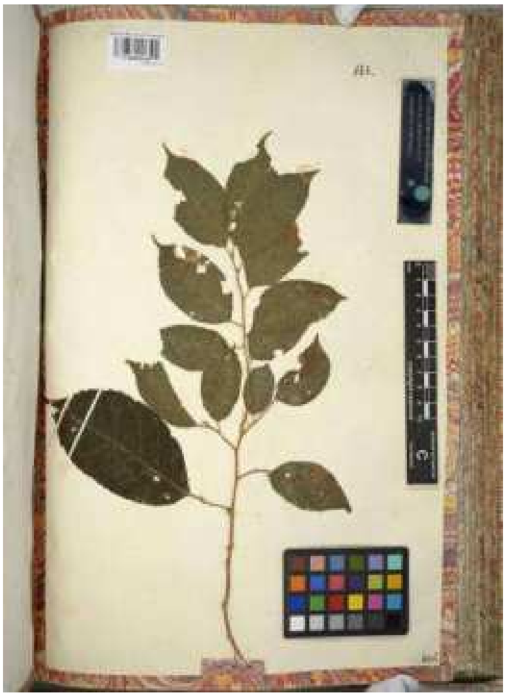

Marcgrave's herbarium: 136

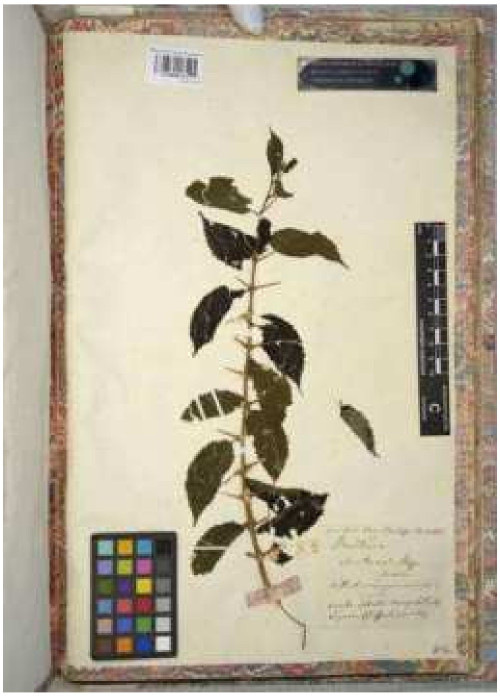

Marcgrave's herbarium: 172

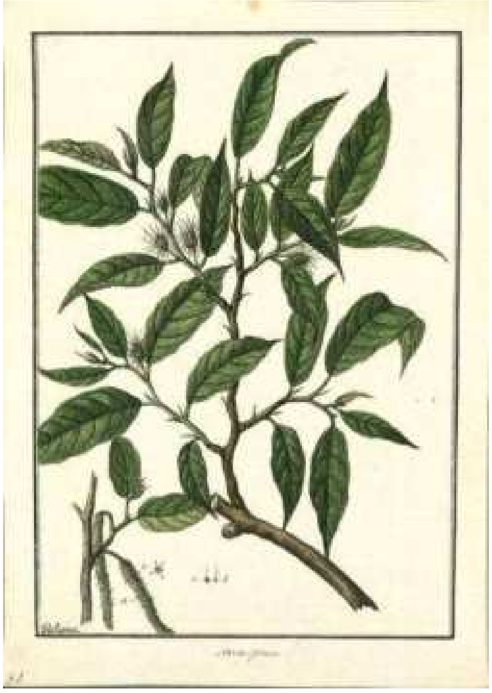

Female and male (bottom left) flowers in *Drawings of the Royal Botanical Expedition to the Viceroyalty of Peru* by Ruiz, H., Pavón, J. (1777)

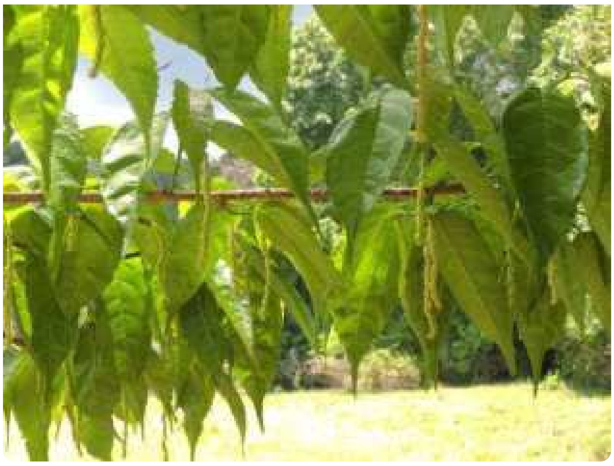

Male flowers. "*M. tinctoria*" by Barry Hammel (CC BY-NC-SA 2.0)

Historiae Rerum Naturalium Brasiliae | Marcgrave, 1648 | Page number 120a

Vernacular name(s) Uty

Species Chloroleucon foliolosum (Benth.) G.P.Lewis

Family Fabaceae

Notes

We did not find any correspondence between this woodcut and the contemporary or older sources. Interestingly, according to his brother Christian, this tree is described in Marcgrave's diary under the name of *Utium* (Whitehead 1979: 312). He observed 400 bird nests on this tree when he encountered it on one of his expeditions in November of 1640 (Whitehead 1979: 309). Marcgrave must have done a voucher for his herbarium collection, as he was on the move, and the woodcut depicts a flat flowering branch (he likely did not see the tree again and hence, the long pods).

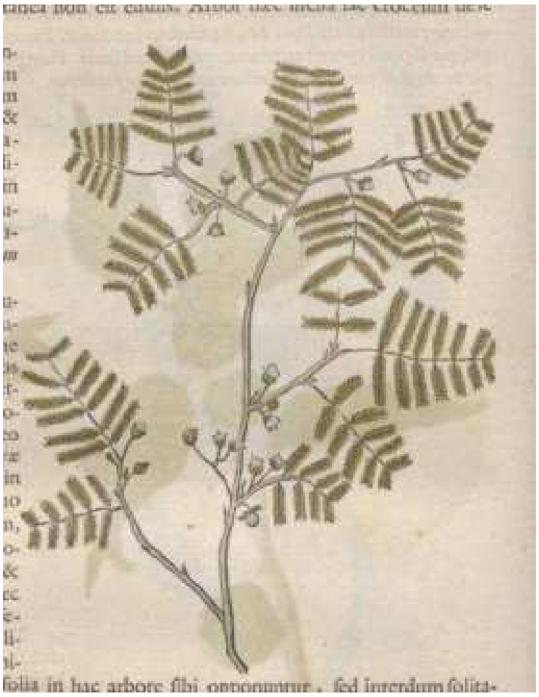

Historiae Plantarum – Arboribus: 120a

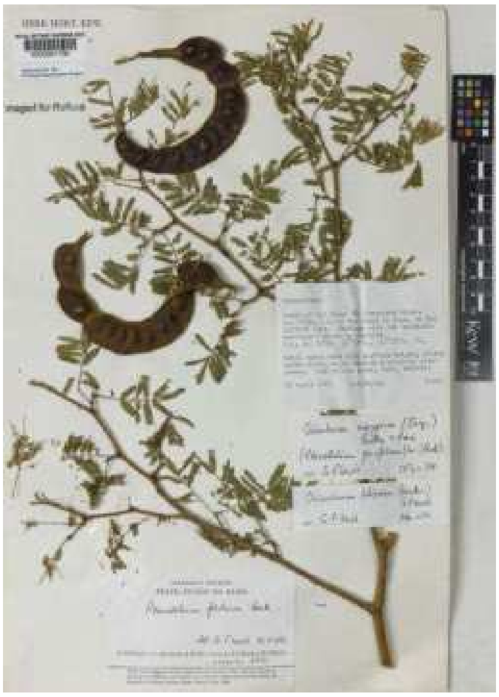

Specimen of *C. foliolosum* from Kew's Herbarium - K000091739. Retrieved from Plants of the World Online

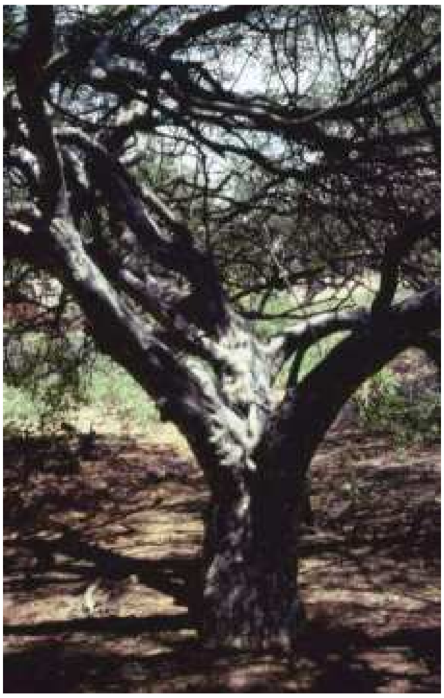

Habit. "*C. foliolosum*" - G.P. Lewis ID:6145 © RBG Kew (CC BY 3.0)

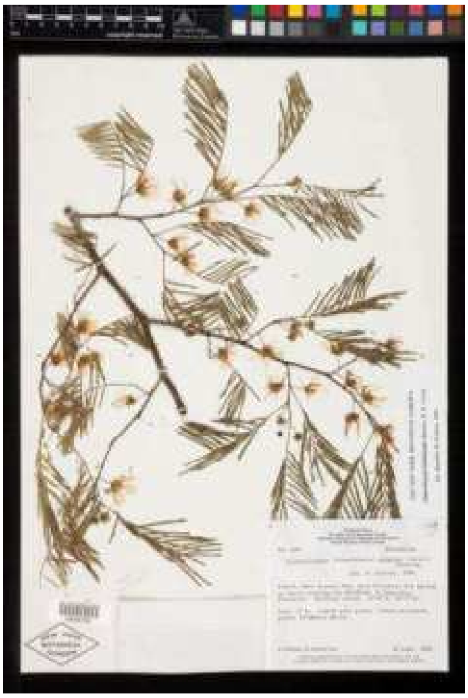

Flowering specimen of *C. foliolosum* collected in Brazil for the New York Botanical Garden -408706- (CC BY 4.0)

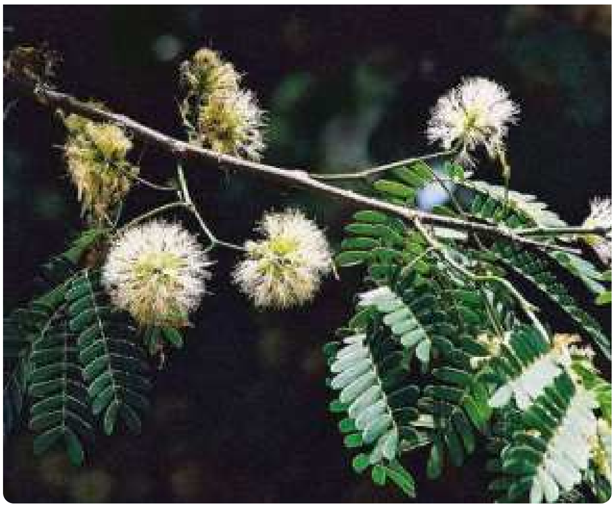

Flowers. *"Chloroleucon tenuiflorum"* a related species to *C. foliolosum* by Dick Culbert (CC BY 2.0)

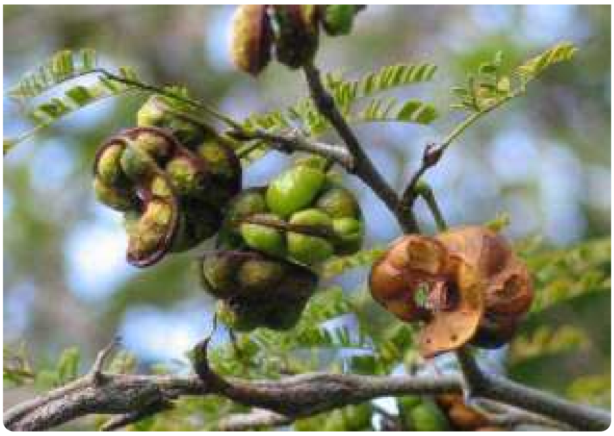

Pods. *"ARAPIRACA "Chloroleucon acacioides"* a related species to *C. foliolosum* by antoniosergio25 (CC BY-NC-SA 2.0)

Historiae Rerum Naturalium Brasiliae	Marcgrave, 1648	Page number	120b

Vernacular name(s) Iito

Species Guarea guidonia (L.) Sleumer

Family Meliaceae

Notes

The woodcut is very similar to the *Theatrum* image (non-reversed). The woodcut presents one seed less than the illustration. There is no resemblance with the specimen, which consists of a flowering branch, in contrast to the fruiting branch of both the HNB and the *Theatrum*.

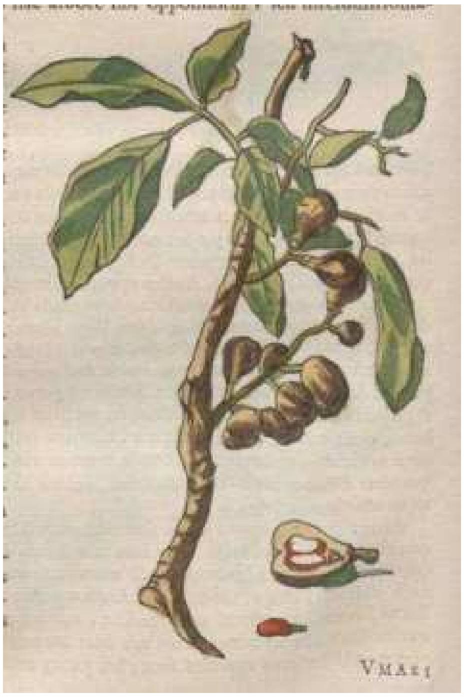

Historiae Plantarum – Arboribus: 120b

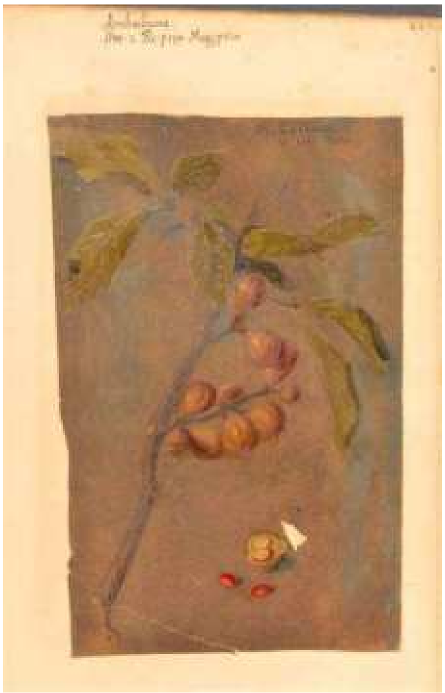

Theatrum Rerum Naturalium: 227

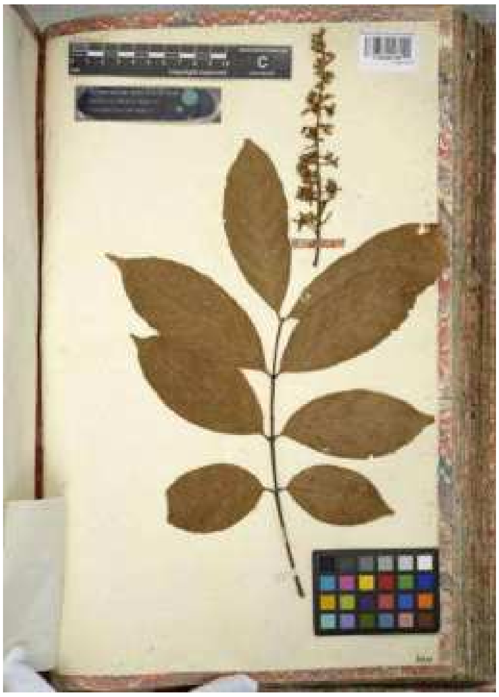

Marcgrave's herbarium: 140

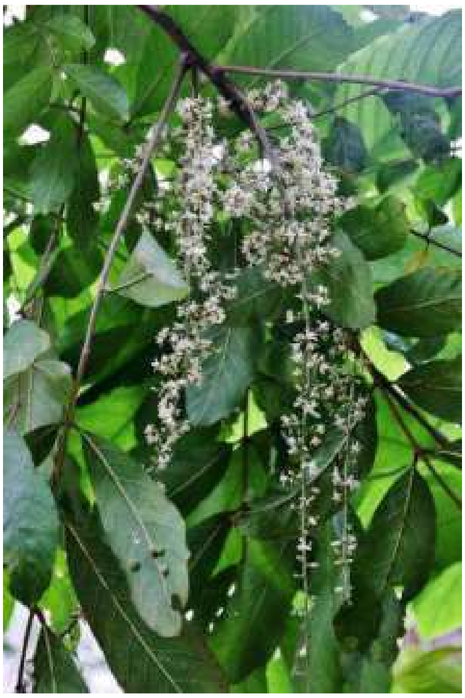

Flowers. "*G. guidonia*" by Mauricio Mercadante (CC BY-NC-SA 2.0)

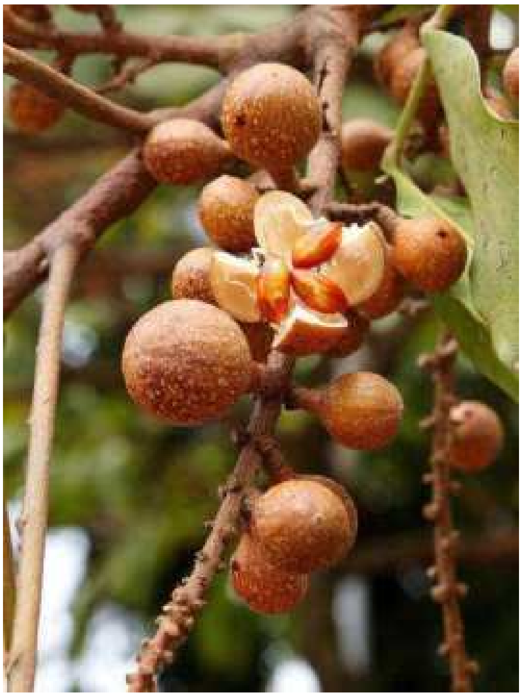

Fruits. "*G. guidonia*" by Mauricio Mercadante (CC BY-NC-SA 2.0)

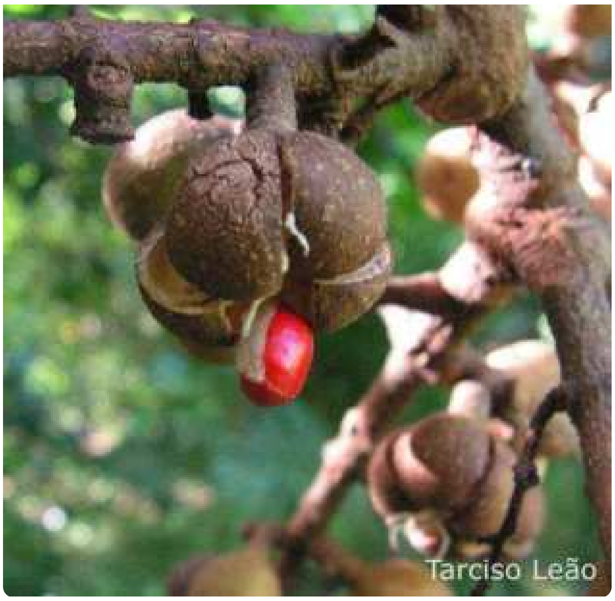

"*G. guidonia*, gitó" by Tarciso Leão (CC BY 2.0)

Historiae Rerum Naturalium Brasiliae Marcgrave, 1648 Page number 121a

Vernacular name(s) Umari

Species Geoffroea spinosa Jacq.

Family Fabaceae

Notes

We did not find any correspondence between this woodcut and the contemporary or older sources.

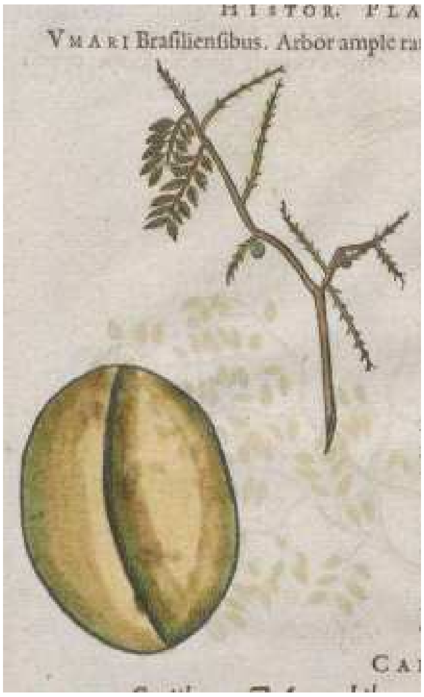

Historiae Plantarum – Arboribus: 121a

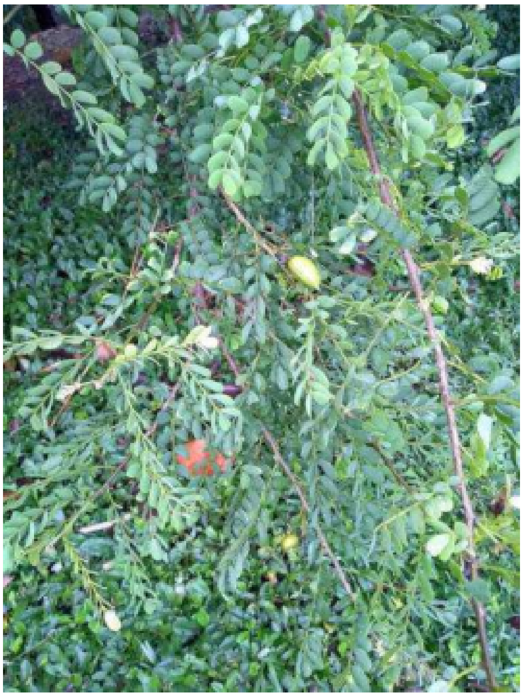

G. spinosa observed in Colombia by Ana Maria Benavides for iNaturalist (CC BY-NC 4.0)

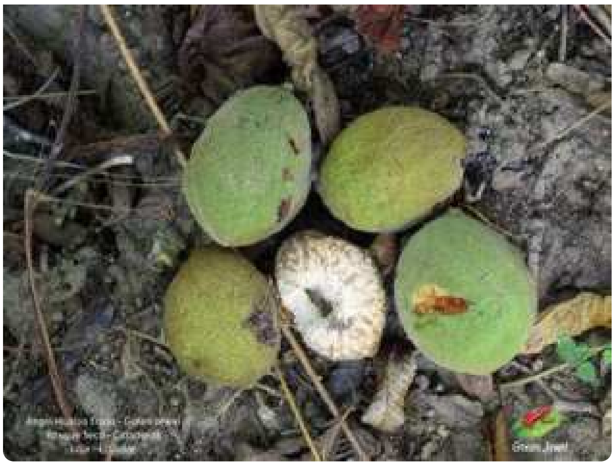

Fruit of *G. spinosa* observed in Ecuador by Green Jewel for iNaturalist (CC BY-NC 4.0)

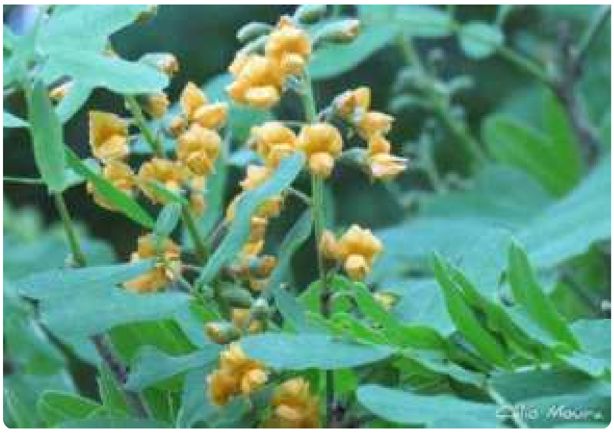

Flowers of *G. spinosa* observed in Brazil by Célio Moura Neto for iNaturalist (CC BY-NC 4.0)

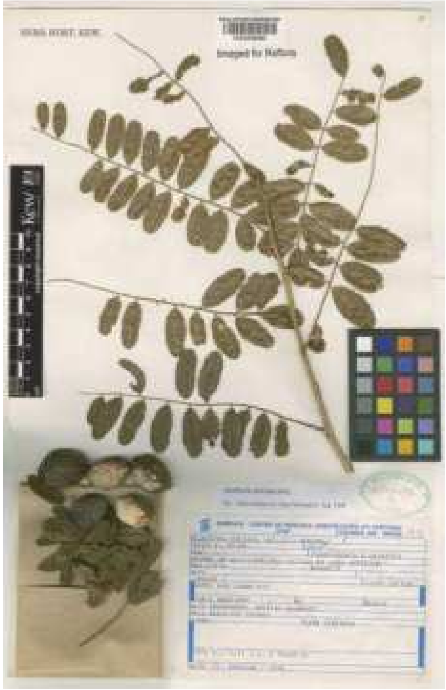

Specimen of *G. spinosa* from Kew's Herbarium - K000908686. Retrieved from Plants of the World Online

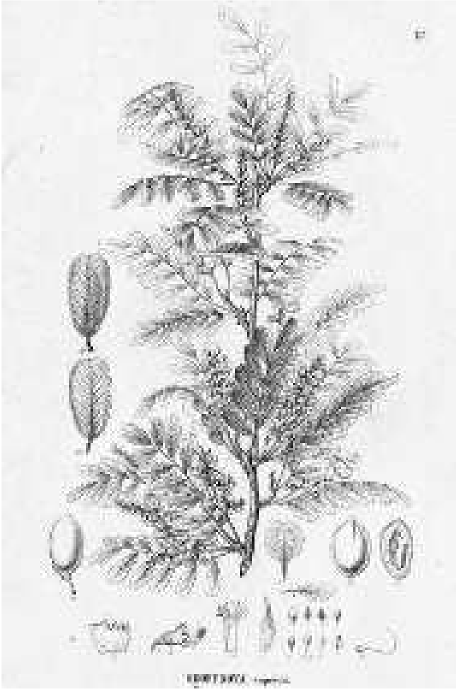

Engraving of *G. spinosa* in Martius, C.F.P. von, Eichler, A.G., Urban, I., *Flora Brasiliensis* (1859-1862) Vol. 15 (1): t. 117, p. 300

Historiae Rerum Naturalium Brasiliae Marcgrave, 1648 Page number 121b

Vernacular name(s) Copiiba

Species Tapirira guianensis Aubl

Family Fabaceae

Notes

The image in De Laet's manuscript is an ink drawing that bears resemblance to the woodcut (in reversed format), but it is not the same. According to Whitehead and Boeseman (1989), this ink drawing was the basis for the woodcut in the HNB.

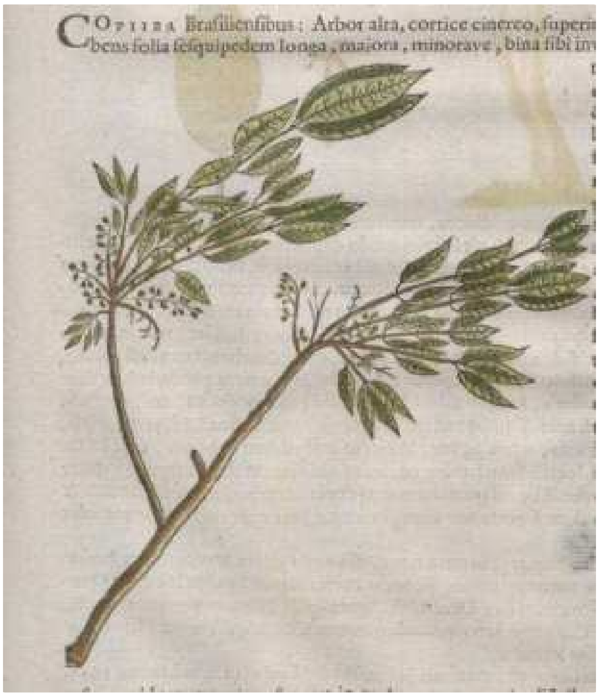

Historiae Plantarum – Arboribus: 121b

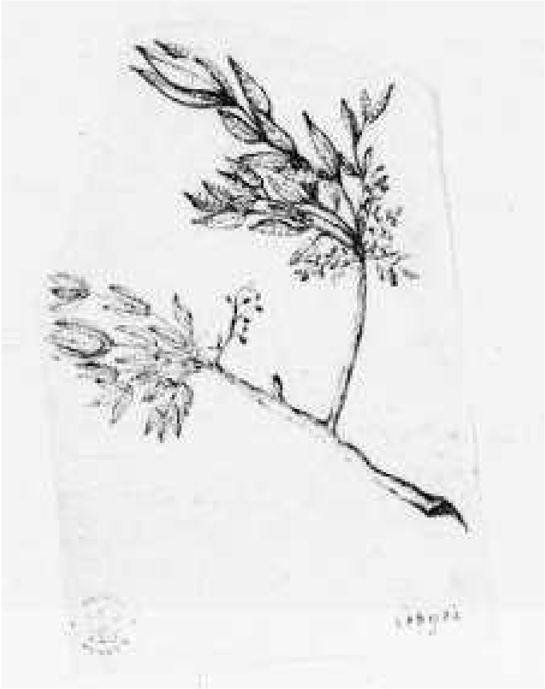

Ink drawing of *T. guianensis* in De Laet's manuscript: Sloane MS 1554, f. 74v

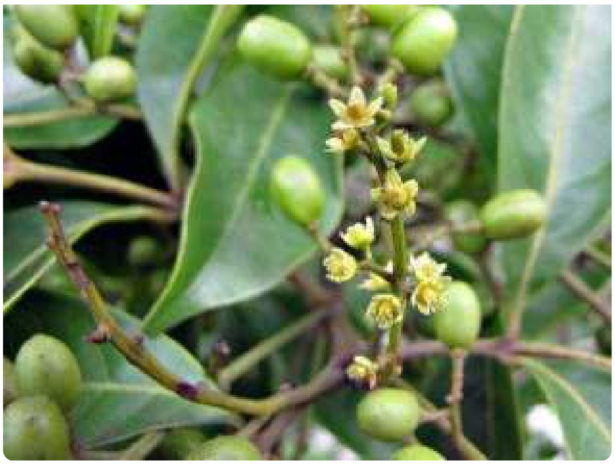

Flowers. "Pombeiro (*T. guianensis*)" by Mauricio Mercadante (CC BY-NC-SA 2.0)

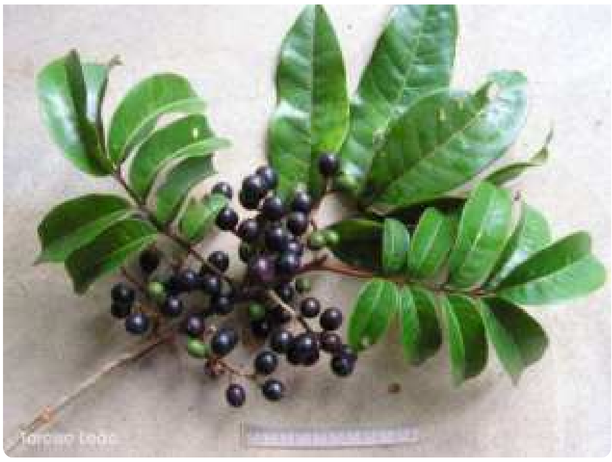

Fruits. "*T. guianensis*, cupiúba, pau-pombo" by Tarciso Leão (CC BY 2.0)

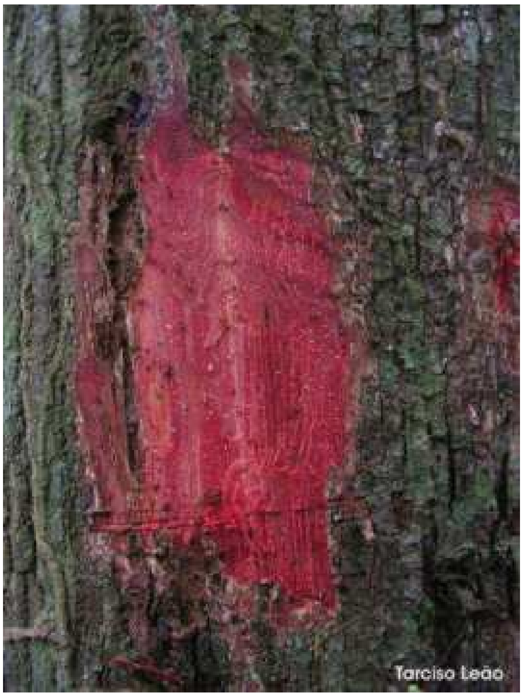

Tree bark. "*T. guianensis*, cupiúba, pau-pombo" by Tarciso Leão (CC BY 2.0)

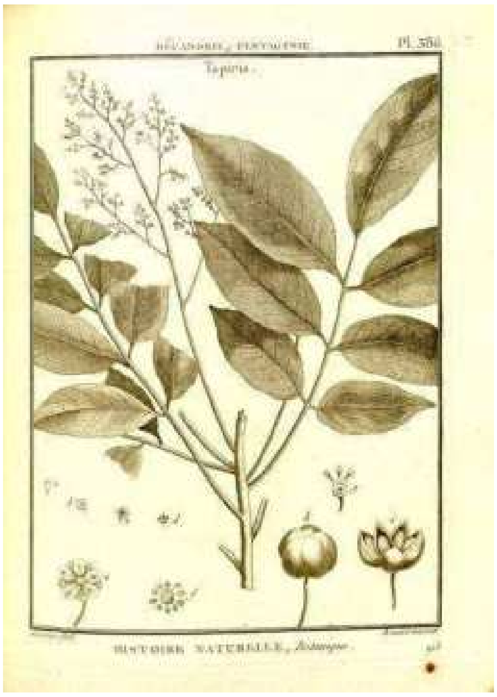

Recueil de planches de botanique de l'encyclopédie by Lamarck, J.B.P.A. de Monet de, Poiret, J.L.M. (1791-1823: Vol. II, t. 386). Missouri Botanical Garden

Historiae Rerum Naturalium Brasiliae	Marcgrave, 1648	Page number	122

Vernacular name(s) Mangabiba. Mangaiba. Mangaba (fruit)

Species Hancornia speciosa Gomes

Family Apocynaceae

Notes

The woodcut differs from the *Theatrum* image. It does not bear resemblance to the other sources either, which only depict the fruits. Marcgrave (1648: 122) documented valuable data about the phenology of the flowering and fruiting stages in several locations.

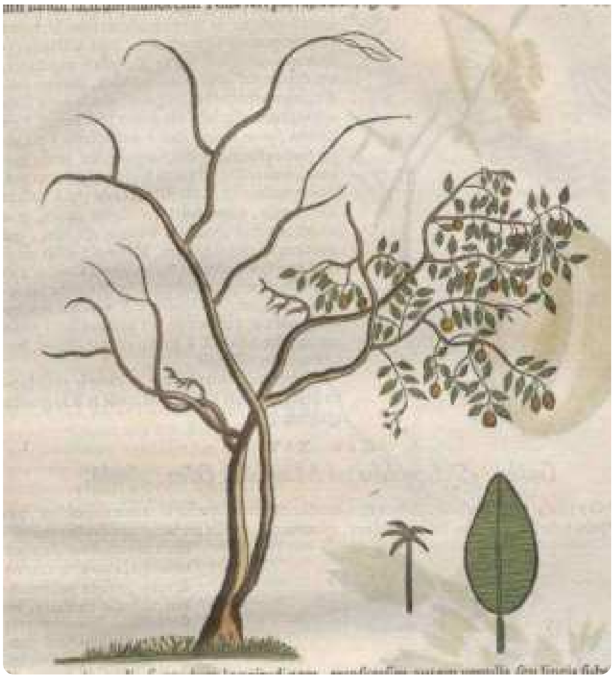

Historiae Plantarum – Arboribus: 122

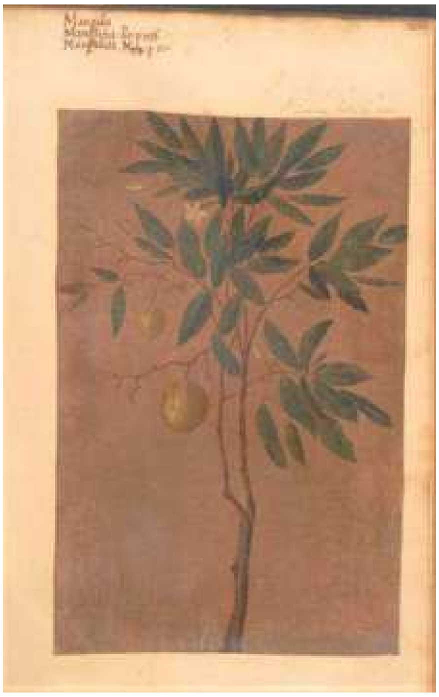

Theatrum Rerum Naturalium: 203

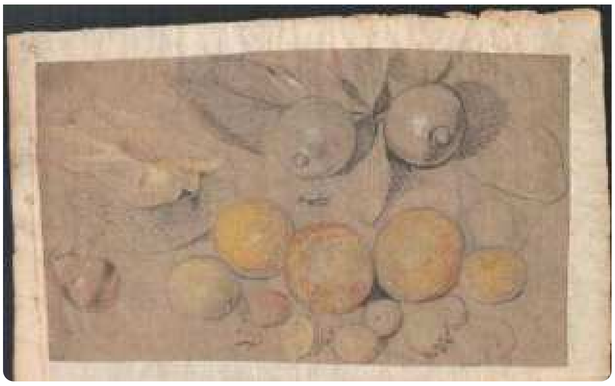

Miscellanea Cleyeri: 51r

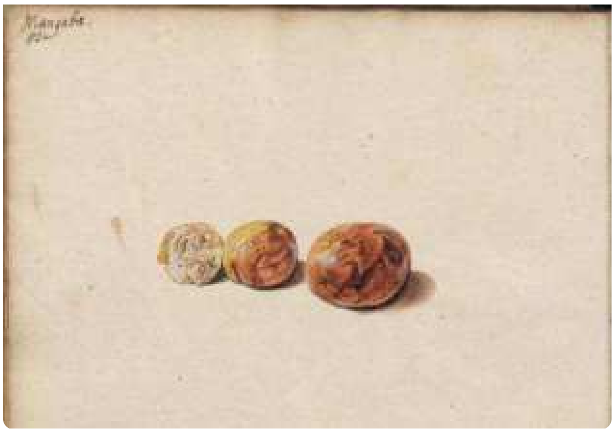

Libri Principis: 71 [51]

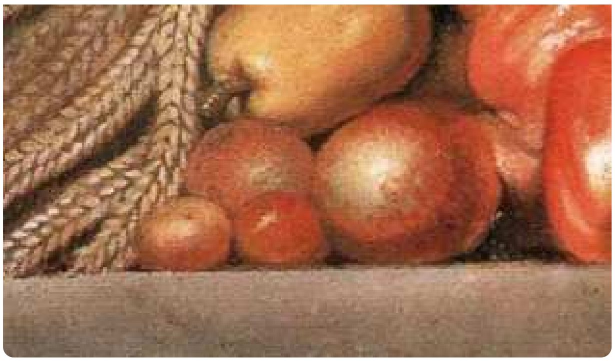

Close - up of Ekchout's still-life "with watermelon, pineapple and other fruit, ca. 1640. Copenhagen, National Museum of Denmark

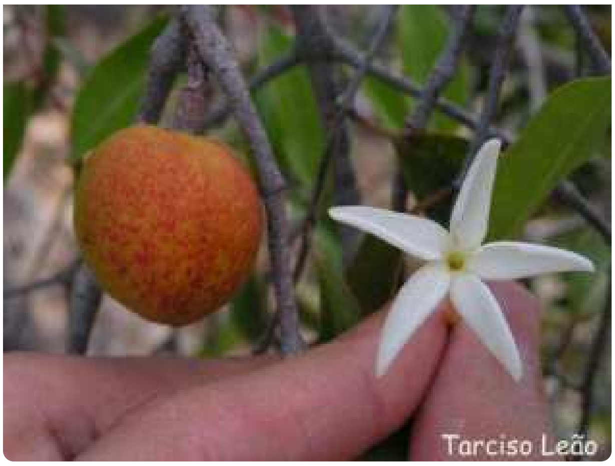

Fruit and flower. "*H. speciosa*" by Tarciso Leão (CC BY 2.0)

Historiae Rerum Naturalium Brasiliae	Marcgrave, 1648	Page number	123

Vernacular name(s) Cuiete. Cochine

Species Crescentia cujete L.

Family Bignoniaceae

Notes

The woodcut does not bear a strong resemblance to the specimen, although the leaves of the latter could have been used as a model to draw the leaves in the upper part of the woodcut. The woodcut image shows the dimorphism of the fruit, as documented by Marcgrave (1648: 123).

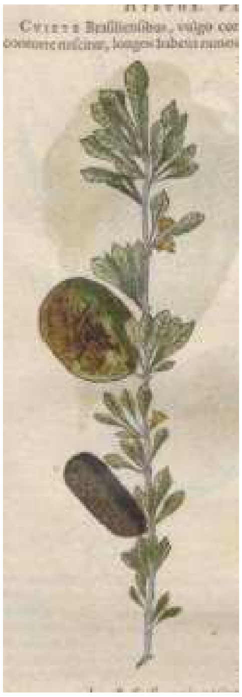

Historiae Plantarum – Arboribus: 123

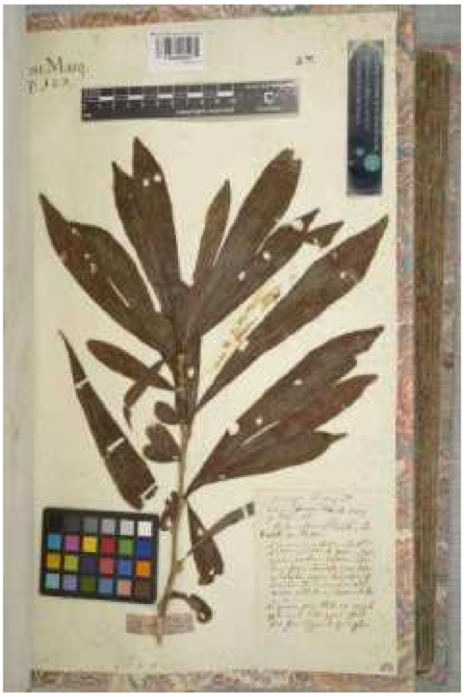

Marcgrave's herbarium: 50

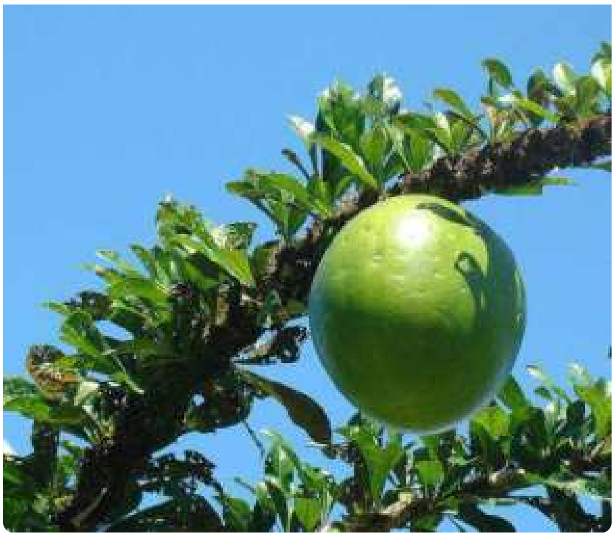

"*C. cujete*, known as Calabash" by Dick Culbert (CC BY 2.0)

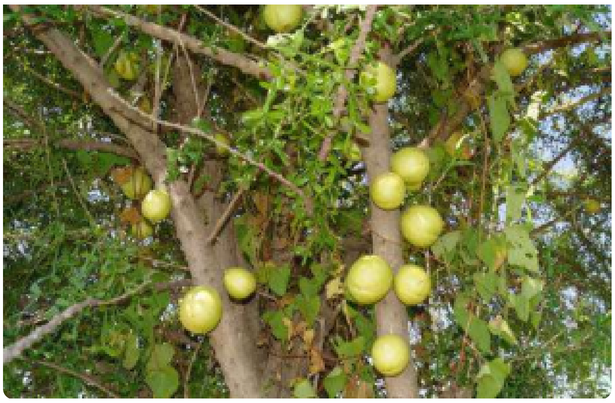

Habit and fruits emerging from the trunk. "*C. cujete* by sergioniebla (CC BY-SA 2.0)

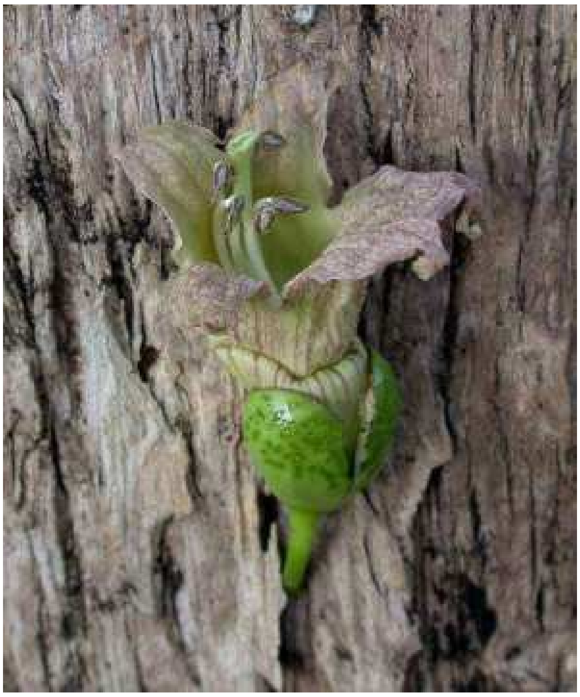

Flower. "*C. cujete*" by LennyWorthington (CC BY-SA 2.0)

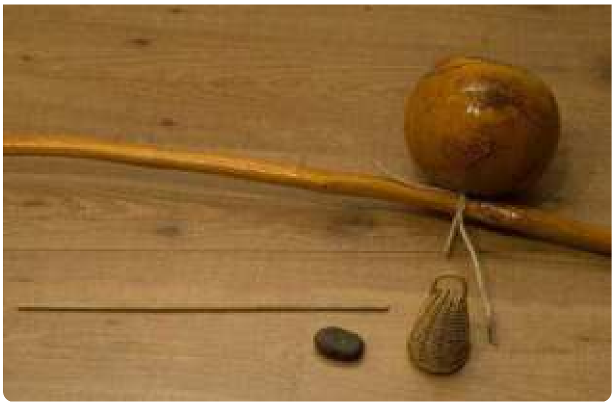

Berimbau: Musical instrument made with the fruit of *C. cujete* played in Brazil and originated in Africa. "O que é berimbau?" by illustir (CC BY 2.0)

Historiae Rerum Naturalium Brasiliae | Marcgrave, 1648 | Page number 124

Vernacular name(s) Apeiba

Species Apeiba tibourbou Aubl.

Family Malvaceae

Notes

The image in De Laet's manuscript is an ink drawing that bears resemblance to the woodcut (in reversed format), but it is not exactly the same. According to Whitehead and Boeseman (1989), this ink drawing was the basis for the woodcut in the HNB.

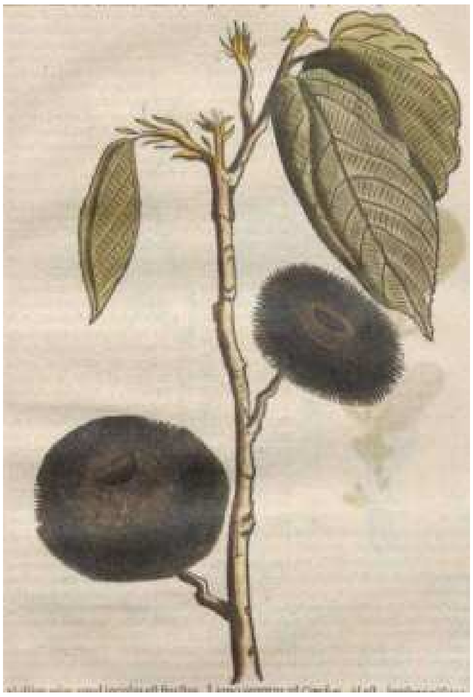

Historiae Plantarum – Arboribus: 124

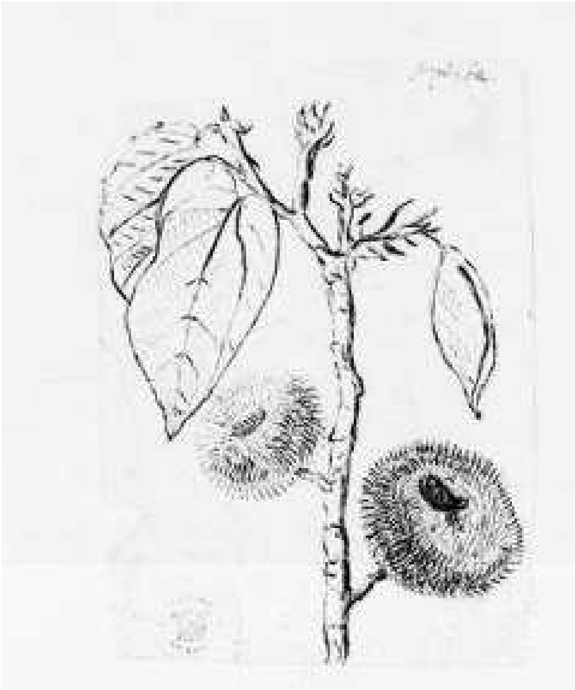

Ink drawing of *A. tibourbou* in De Laet's manuscript: Sloane MS 1554, f. 79v

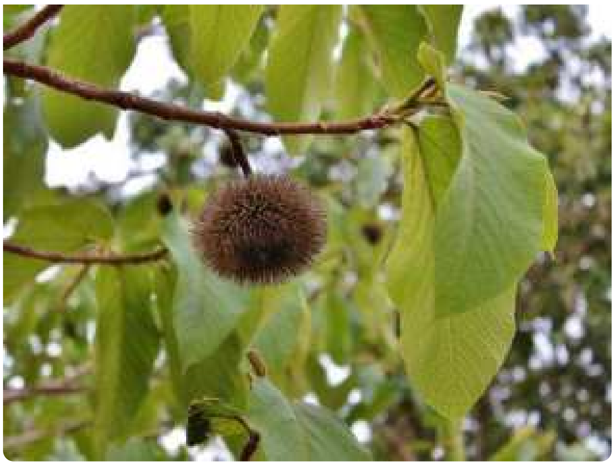

Hanging fruit. "*A. tibourbou*" by Mauricio Mercadante (CC BY-NC-SA 2.0)

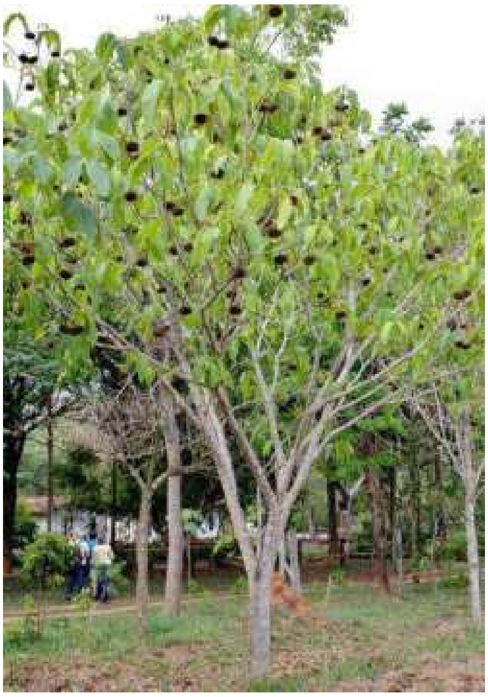

Habit "*A. tibourbou*" by Mauricio Mercadante (CC BY-NC-SA 2.0)

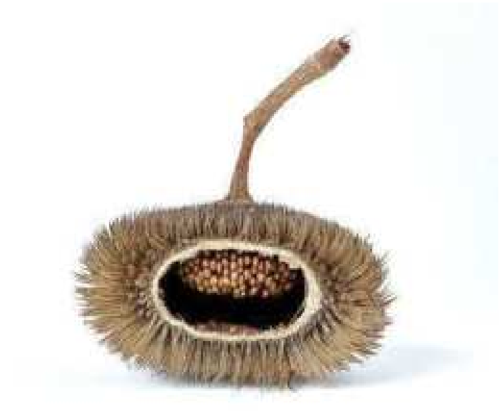

Open fruit and seeds. "*A. tibourbou*" by Mauricio Mercadante (CC BY-NC-SA 2.0)

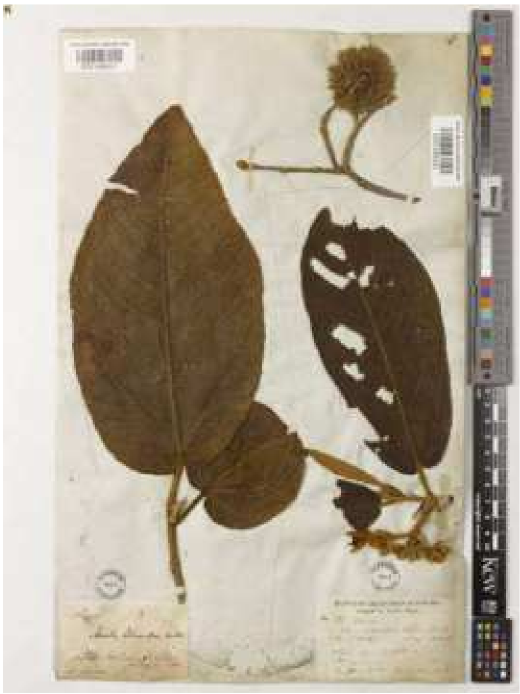

Specimen of *A. tibourbou* from Kew's Herbarium - K001486432. Retrieved from Plants of the World Online

Historiae Rerum Naturalium Brasiliae	Marcgrave, 1648	Page number	125a

Vernacular name(s) Betis

Species Piper divaricatum G.Mey.

Family Piperaceae

Notes

We did not find any correspondence between this woodcut and the contemporary or older sources.

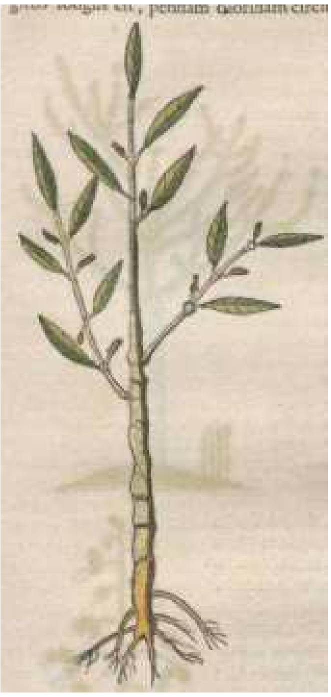

Historiae Plantarum – Arboribus: 125a

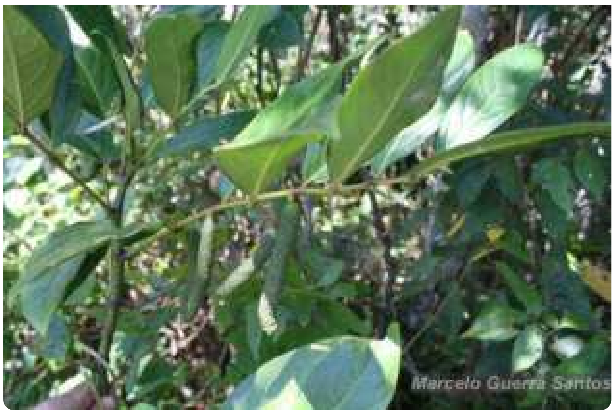

P. divaricatum by Marcelo Guerra Santos. Published online by Elsie Franklin Guimarães for Flora e Funga do Brasil

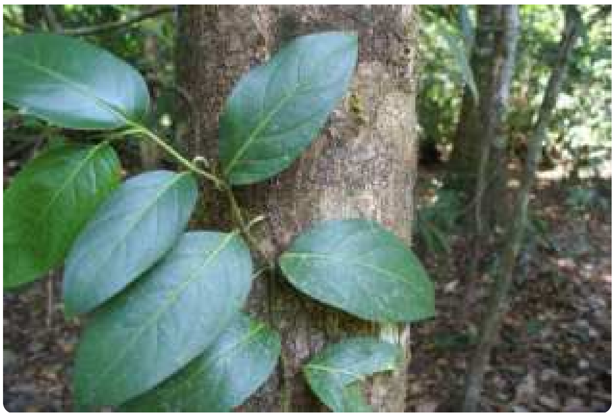

P. divaricatum - Specimen D. Zappi et al. 1409
ID:1104209 © RBG Kew (CC BY 3.0)

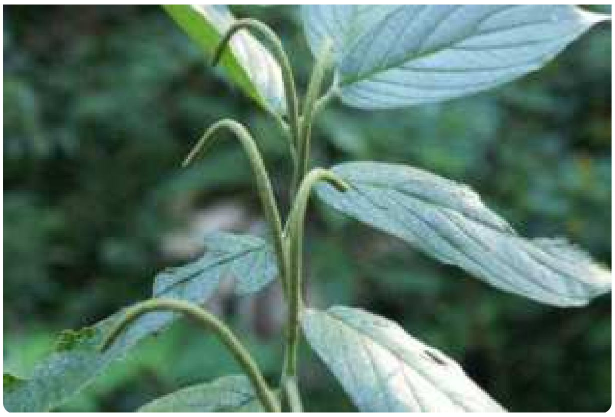

P. divaricatum - Specimen G.S. Henicka 266
ID:1104056 © RBG Kew (CC BY 3.0)

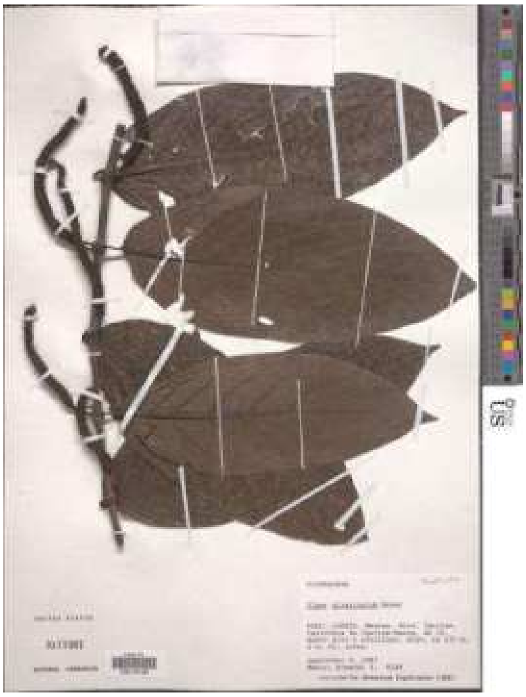

"*P. divaricatum*" by Manuel Rimachi Y. Specimen -03518199- Smithsonian National Museum of Natural History (CC0 1.0)

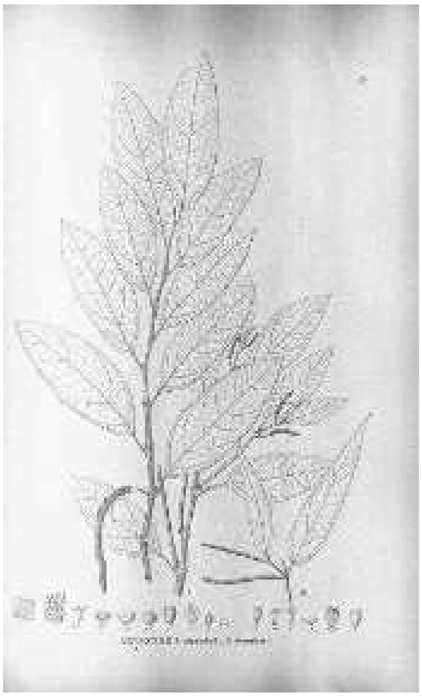

Engraving of *P. divaricatum* in Martius, C.F.P. von, Eichler, A.G., Urban, I., *Flora Brasiliensis* (1852-1863) Vol. 4 (1): t. 21

Historiae Rerum Naturalium Brasiliae	Marcgrave, 1648	Page number	125b

Vernacular name(s) Iamacaru

Species Selenicereus undatus (Haw.) D.R. Hunt

Family Cactaceae

Notes

We did not find any correspondence between this woodcut and the contemporary or older sources.

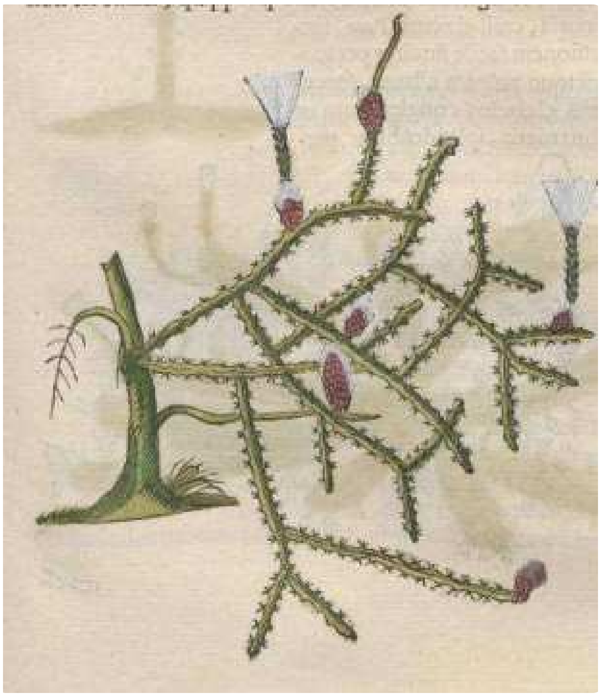

Historiae Plantarum – Arboribus: 125b

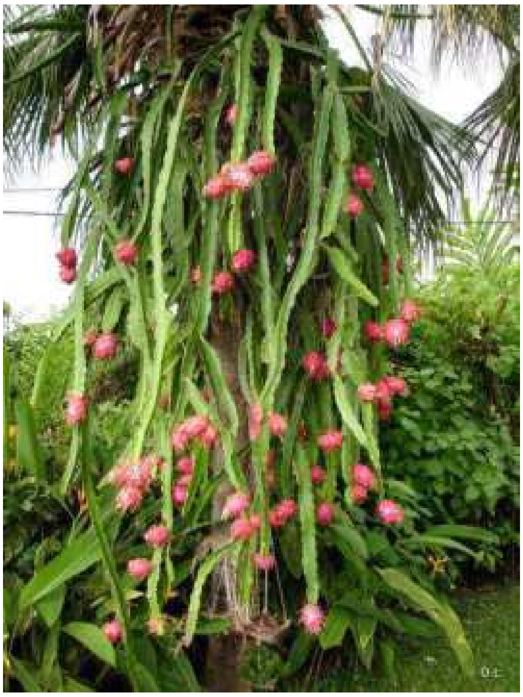

S. undatus. "38 jours après la floraison" by Tominiko974 (CC BY-SA 3.0)

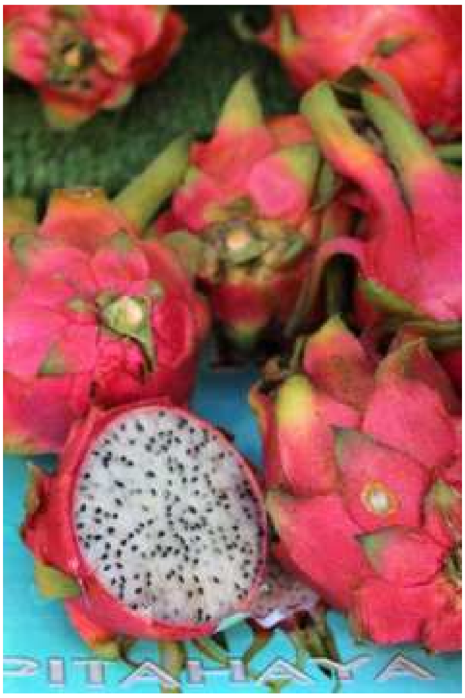

"White-fleshed pitahaya (*S. undatus*) fruits in a market in Cambridge, England by Emőke Dénes (CC BY-SA 4.0)

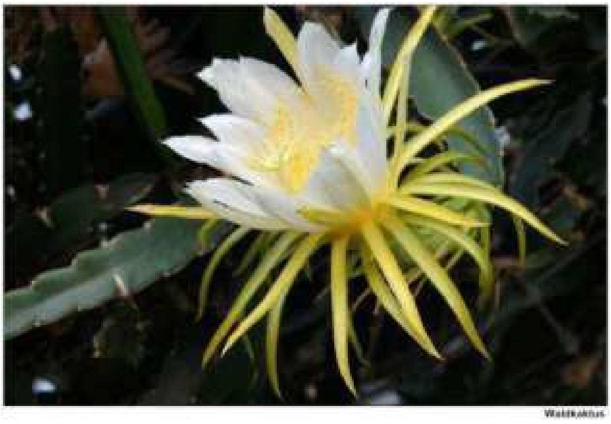

Gigantic blossoms of the "Jungle Cactus" (*Hylocereus undatus*) [syn. of *S. undatus*] by Waldkaktus for Flickr

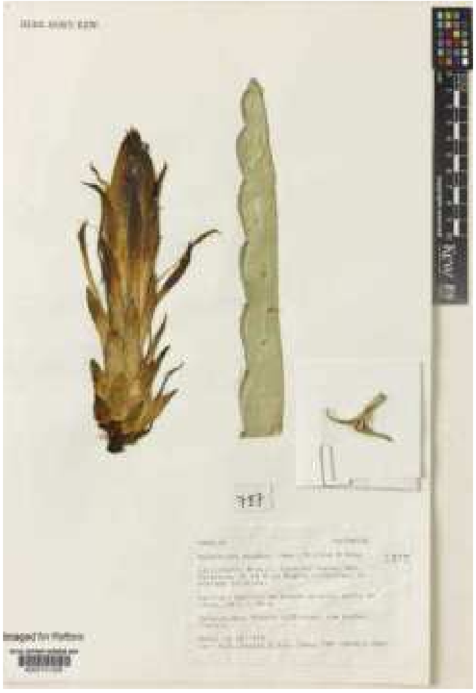

Specimen of *S. undatus* from Kew's Herbarium - K000101208. Retrieved from Plants of the World Online

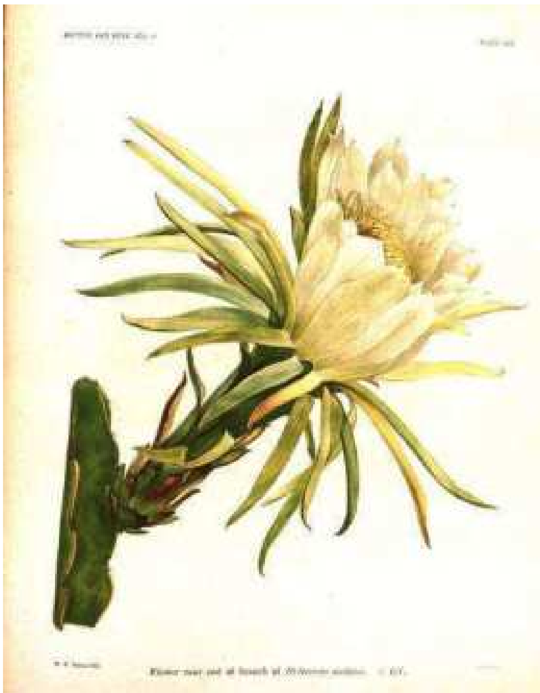

Illustration of *S. undatus* in *Cactaceae* by Britton, N.L., Rose, J.N. (1920: Vol. II, t.30) Missouri Botanical Garden, St. Louis, U.S.A.

Historiae Rerum Naturalium Brasiliae	Marcgrave, 1648	Page number	126a

Vernacular name(s)	Iamacaru. Caxabu. Cardon
Species	Cereus jamacaru DC.
Family	Cactaceae
Notes	

The cactus depicted in the woodcut bears a strong resemblance to the cactus portrayed by Post in his landscape painting "View of the Rio São Francisco Brazil with Fort Maurits and Capibara", as well as to the cactus depicted in Barlaeu's account of Johan Maurits in Brazil, which was designed by Post.

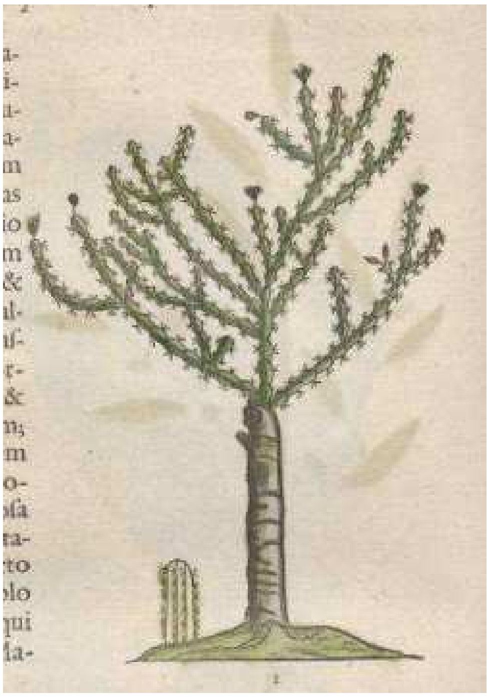

Historiae Plantarum – Arboribus: 126a

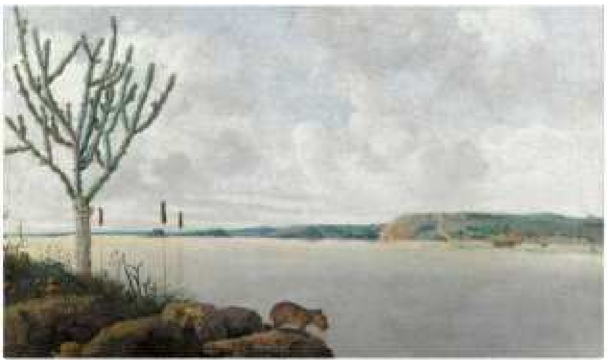

C. jamacaru in the left side of the painting made by Frans Post "The Rio San Francisco Brazil with Fort Maurits and Capibara", ca. 1639

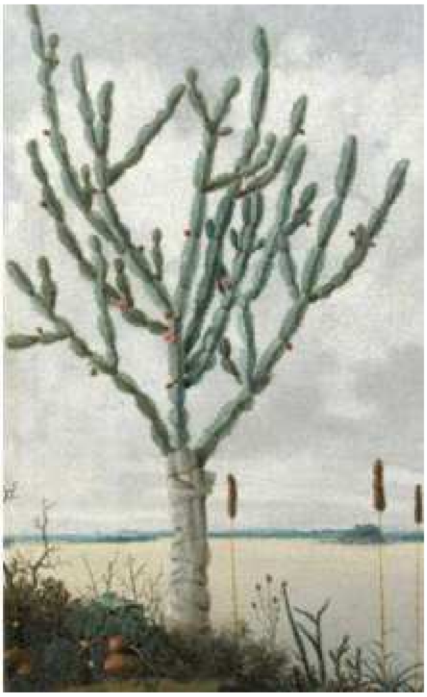

Close- up of *C. jamacaru* in Post painting "The Rio San Francisco Brazil with Fort Maurits and Capibara", ca. 1639

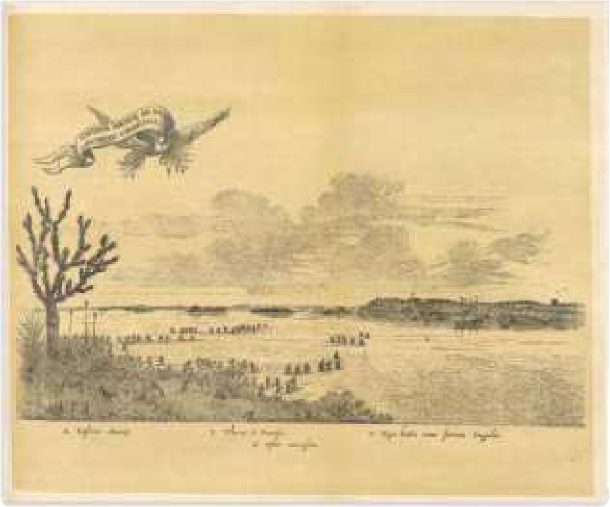

C. jamacaru in *Rerum per octennium in Brasilia et alibi* by Caspar Barlaeus (1647: 102-103), designed made by Post

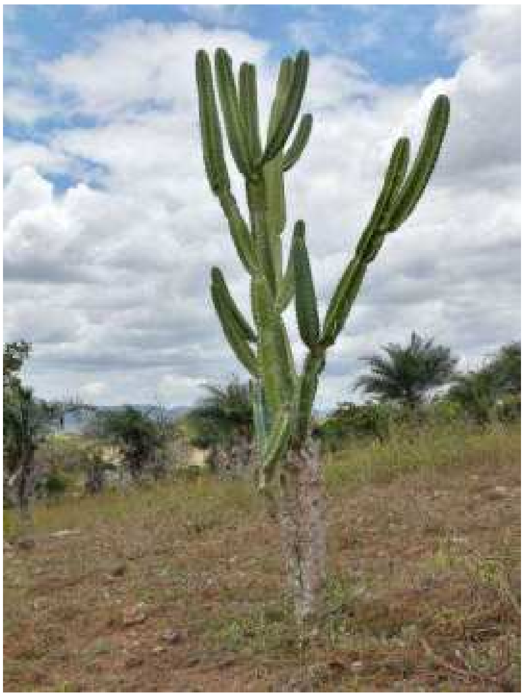

"*C. jamacaru*" by Mauricio Mercadante (CC BY-NC-SA 2.0)

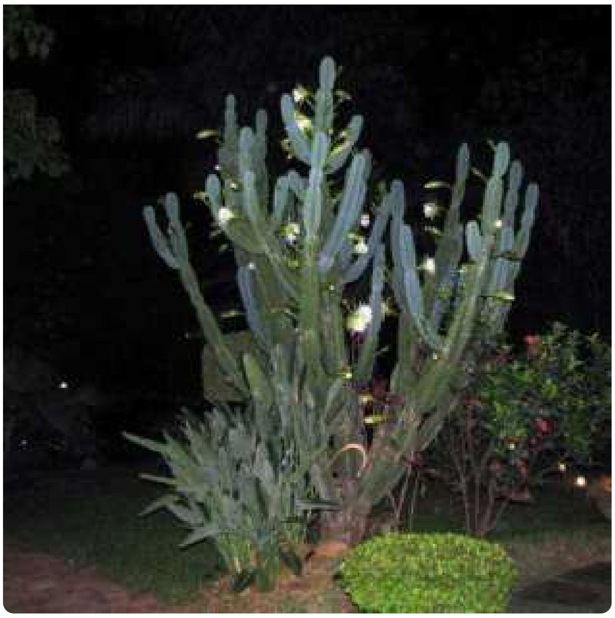

"Mandacaru (*C. jamacaru*)" by Mauricio Mercadante (CC BY-NC-SA 2.0)

Historiae Rerum Naturalium Brasiliae Marcgrave, 1648 Page number 126b

Vernacular name(s) Iamacaru

Species Brasiliopuntia brasiliensis (Willd.) A.Berger

Family Cactaceae

Notes

The woodcut is very similar to the *Theatrum* image (non-reversed). The person that colored the woodcut confused the apical leaves with the red fruits documented by Marcgrave (1648: 126). This error does not occur in the colored copy kept at the State Library of Berlin. De Laet's manuscript includes a pencil lead drawing, which was likely the basis for the woodcut in the HNB. This one is the same as the woodcut, but not reversed.

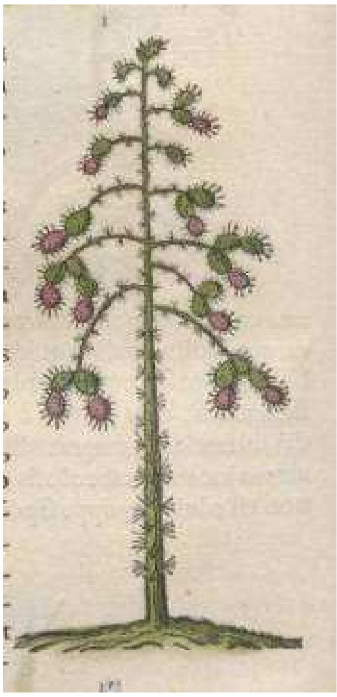

Historiae Plantarum – Arboribus: 126b

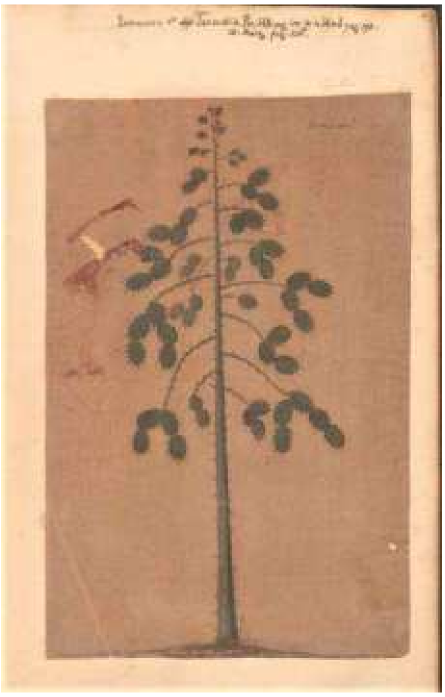

Theatrum Rerum Naturalium: 29

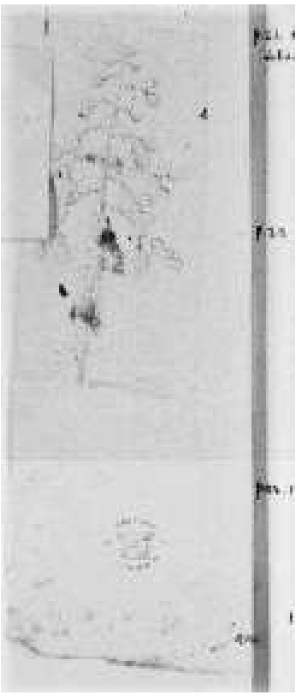

Pencil drawing of *B. brasiliensis* in De Laet's manuscript: Sloane MS 1554, f. 60v

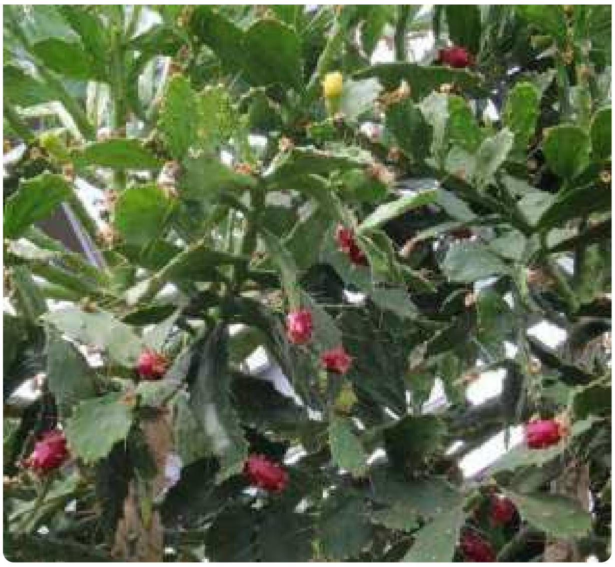

Habit. "*B. brasiliensis*" by Peter A. Mansfeld (CC BY 3.0)

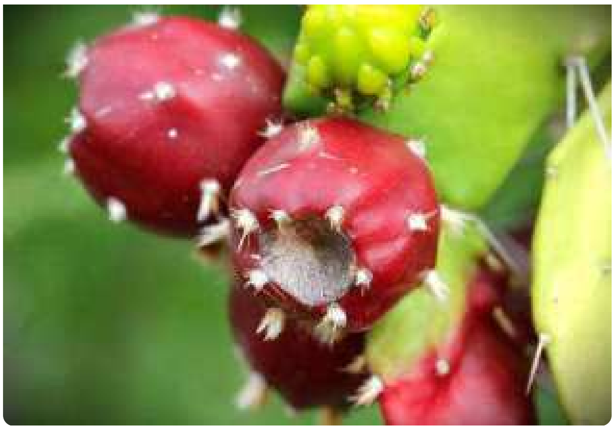

Fruits. "*B. brasiliensis*" by *snowwhite* (CC BY-NC-SA 2.0)

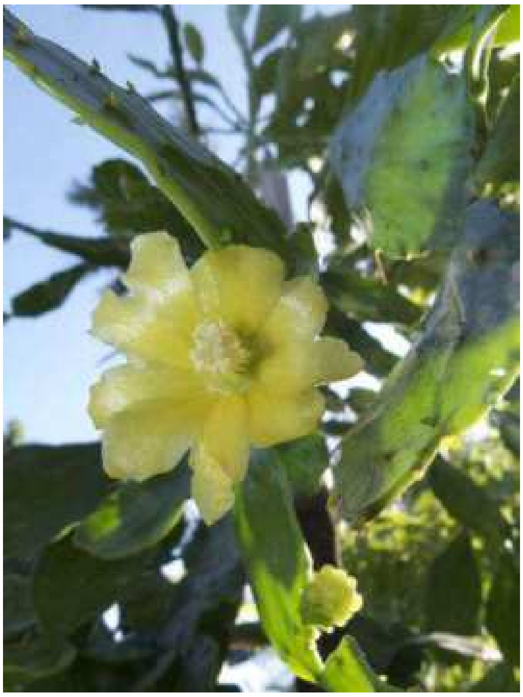

Flower. "*B. brasiliensis* open1" by squarerootofftwo (CC BY-NC-SA 2.0)

Historiae Rerum Naturalium Brasiliae Marcgrave, 1648 Page number 126c

Vernacular name(s) Quarta species Iamacaru

Species Cereus fernambucensis Lem.

Family Cactaceae

Notes

The woodcut differs from the *Theatrum* image. The same species was depicted by a different woodcut in Marcgrave (1648: 24).

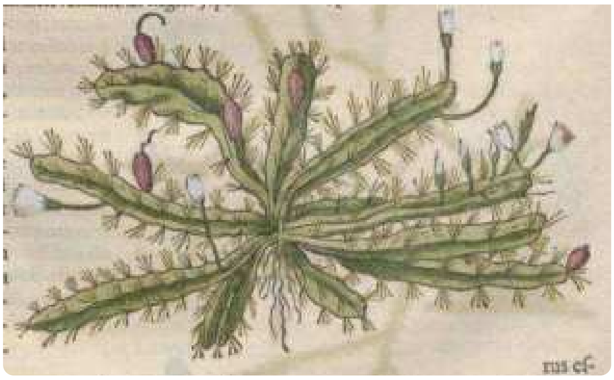

Historiae Plantarum – Arboribus: 126c

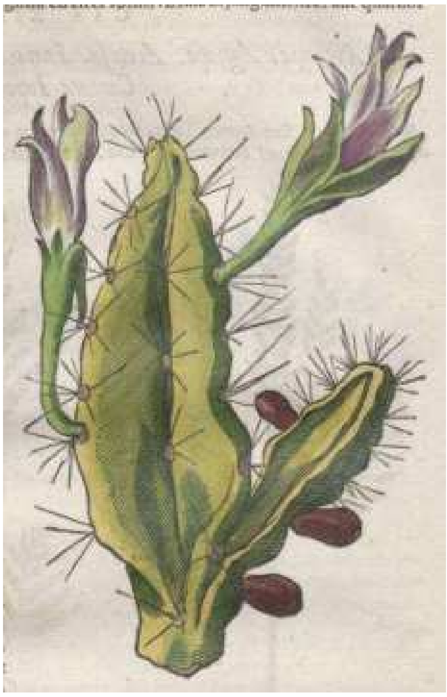

C. fernambucensis depicted differently in Marcgrave (HNB, 1648: 24)

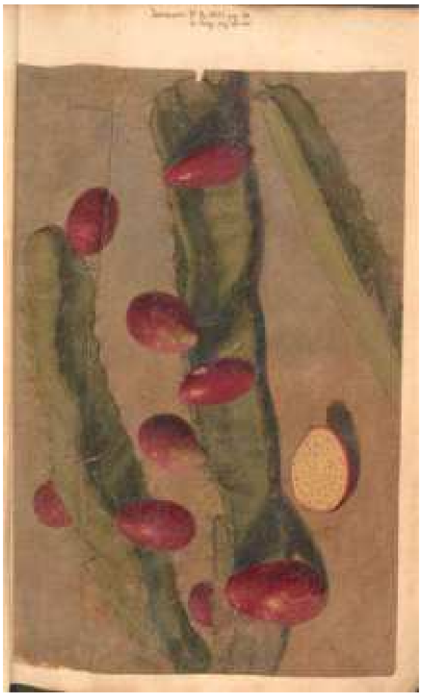

Theatrum Rerum Naturalium: 23

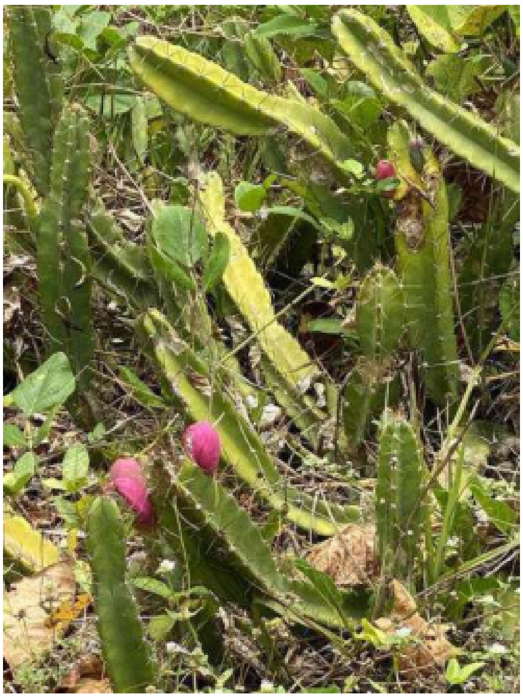

C. fernambucensis observed in Brazil by Pedro Alvaro Neves for iNaturalist. Retrieved from GBIF (CC BY-NC 4.0)

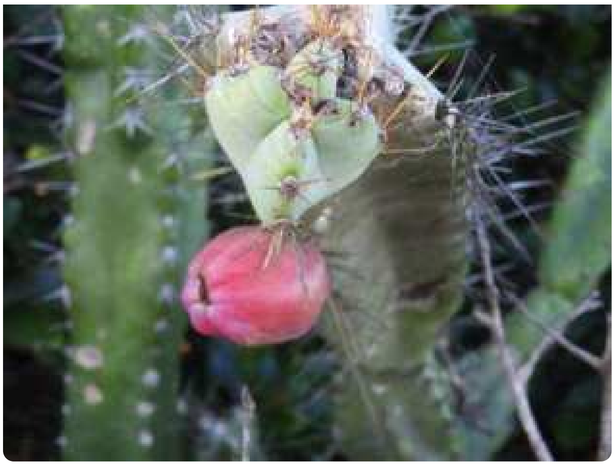

C. fernambucensis observed in Brazil by nena_bergallo for iNaturalist. Retrieved from GBIF (CC BY-NC 4.0)

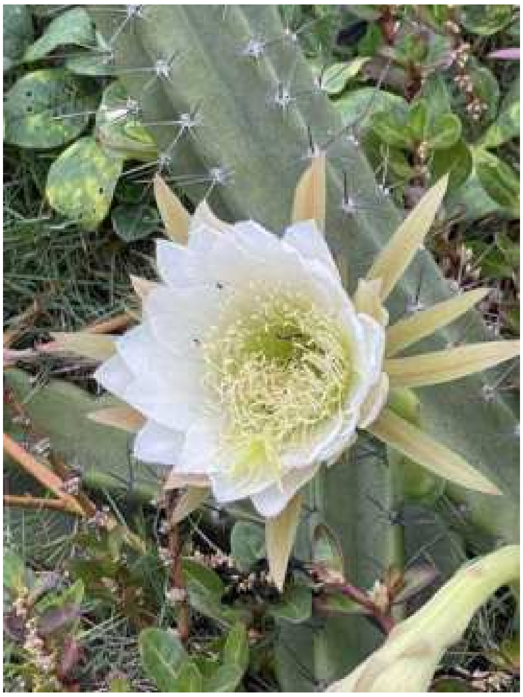

C. fernambucensis observed in Brazil by ssmeds for iNaturalist. Retrieved from GBIF (CC BY-NC 4.0)

Historiae Rerum Naturalium Brasiliae | Marcgrave, 1648 | Page number 127

Vernacular name(s): Iecuiba

Species: Cariniana legalis (Mart.) Kuntze

Family: Lecythidaceae

Notes

We did not find any correspondence between this woodcut and the contemporary or older sources.

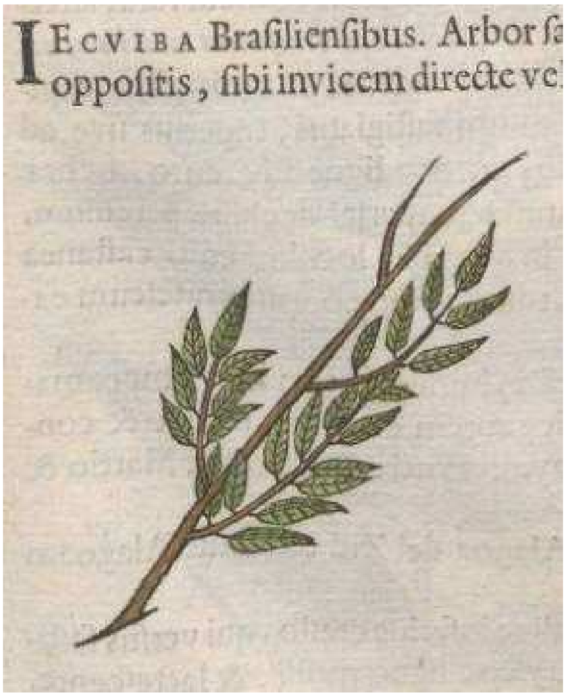

Historiae Plantarum – Arboribus: 127

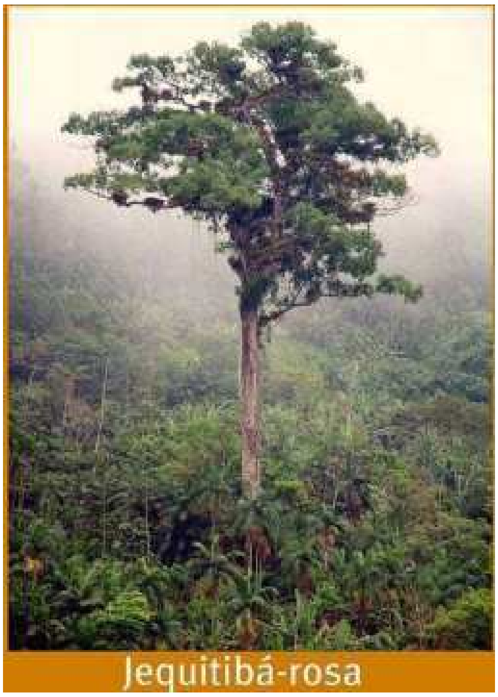

Habit. "Jequitibá-rosa (*C. legalis*) Brazilian booktreemauro" by mauro halpern (CC BY 2.0)

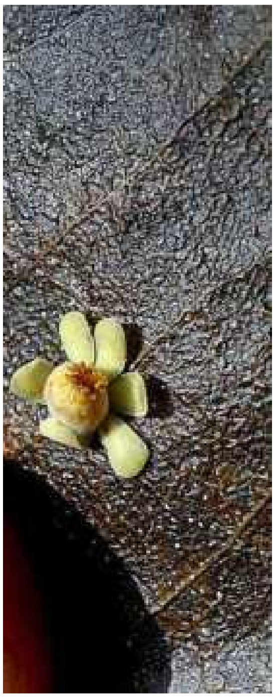

Flower. "*C. legalis*" by Alex Popovkin, Bahia, Brazil (CC BY 2.0)

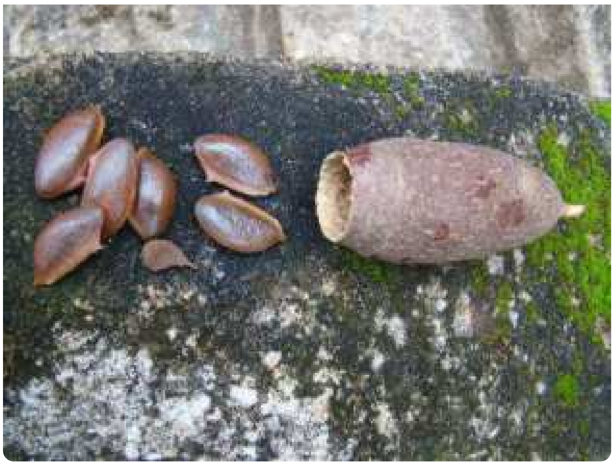

Pod and seeds. "Jequitiba-rosa (*C. legalis*)" by mauro halpern (CC BY 2.0)

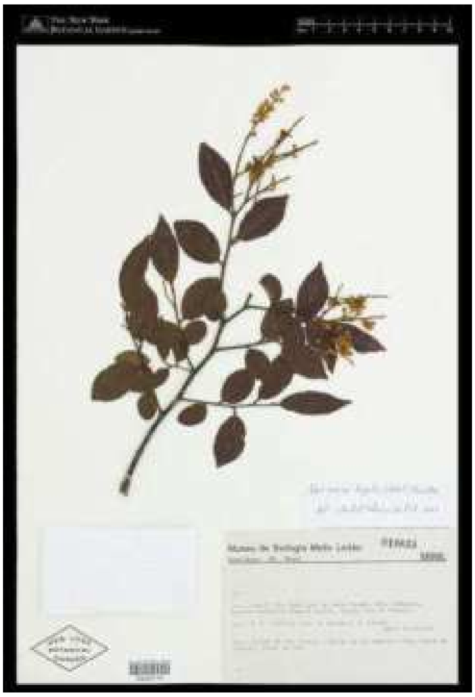

C. legalis collected in Brazil by The New York Botanical Garden (CC BY-NC 4.0)

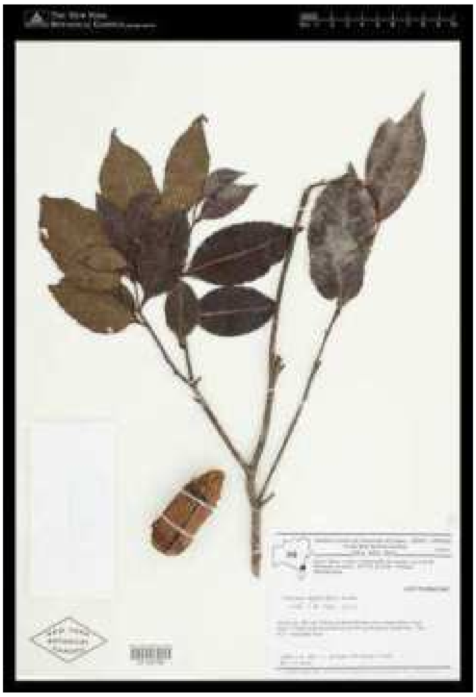

C. legalis collected in Brazil by The New York Botanical Garden (CC BY-NC 4.0)

Historiae Rerum Naturalium Brasiliae | Marcgrave, 1648 | Page number 128

Vernacular name(s) Iacapucaya

Species Lecythis pisonis Cambess

Family Lecythidaceae

Notes

The woodcut differs from the Theatrum illustrations. The pencil lead drawing included in De Laet's manuscript was likely used as the basis to create the woodcut for the HNB (Whitehead and Boeseman 1989). This drawing is not reversed and is very similar to the woodcut.

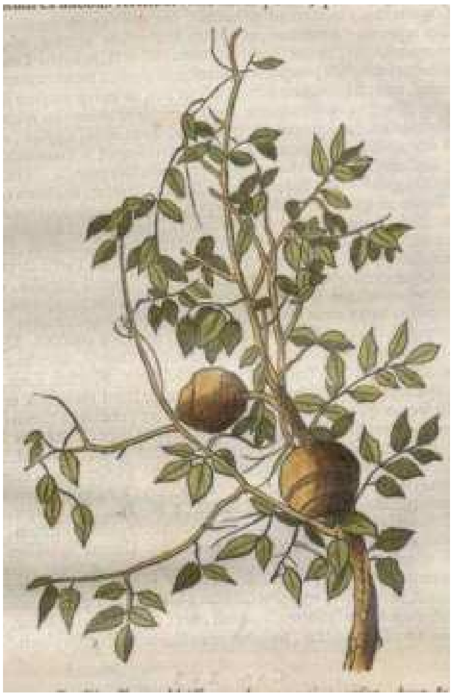

Historiae Plantarum – Arboribus: 128

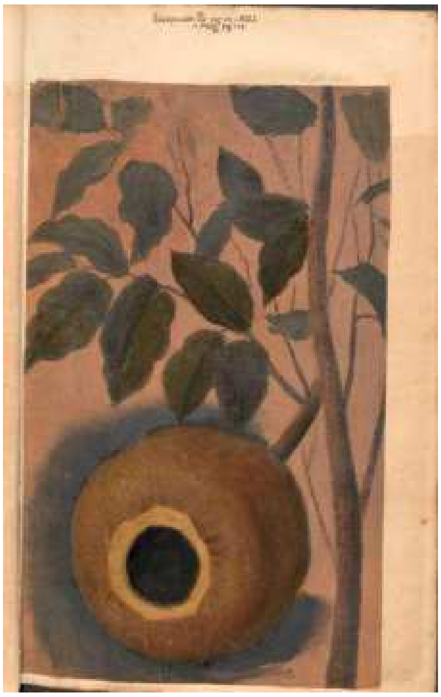

Theatrum Rerum Naturalium: 35

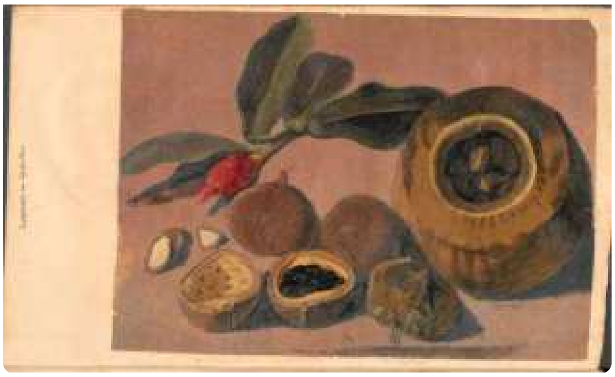

Theatrum Rerum Naturalium: 37

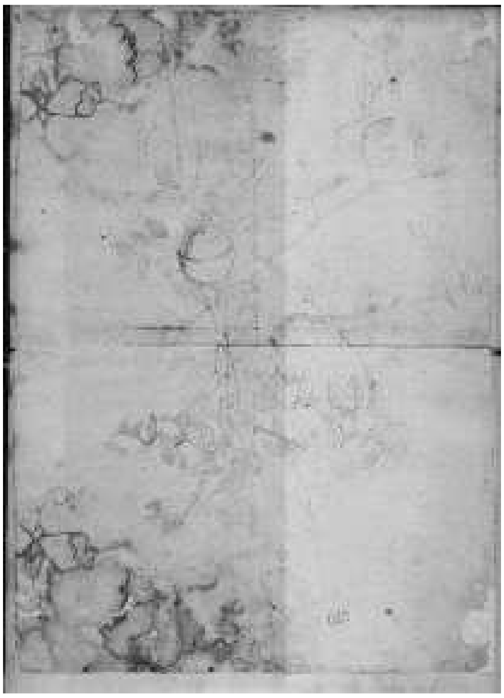

Ink drawing of *L. pisonis* in De Laet's manuscript: Sloane MS 1554, f. 46v and 46r

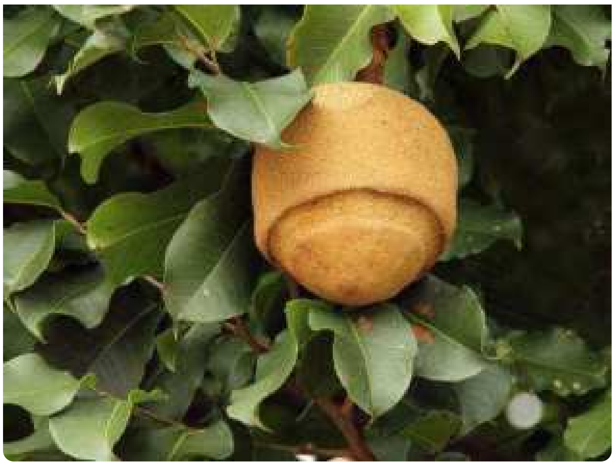

"*L. pisonis*" by Mauricio Mercadante (CC BY-NC-SA 2.0)

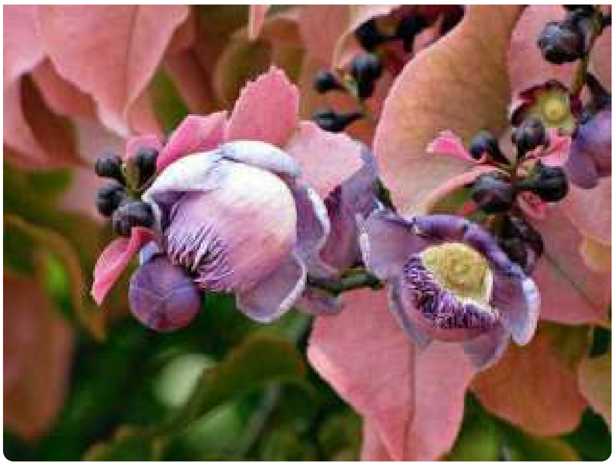

"Sapucaia (*L. pisonis*) " by Mauricio Mercadante (CC BY-NC-SA 2.0)

Historiae Rerum Naturalium Brasiliae | Marcgrave, 1648 | Page number 129a

Vernacular name(s) Iaracatia

Species Jacaratia spinosa (Aubl.) A.DC.

Family Caricaceae

Notes

The woodcut is very similar to the *Theatrum* image (non-reversed). There is a pencil lead drawing in De Laet's manuscript which was likely used as the basis to create the woodcut for the HNB (Whitehead and Boeseman 1989). This drawing is not reversed and is very similar to the woodcut.

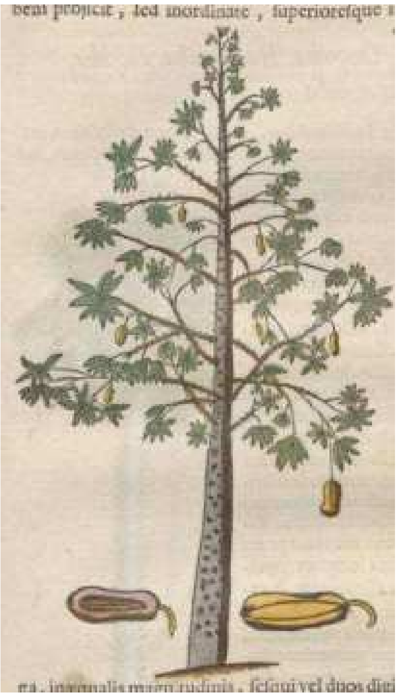

Historiae Plantarum – Arboribus: 129a

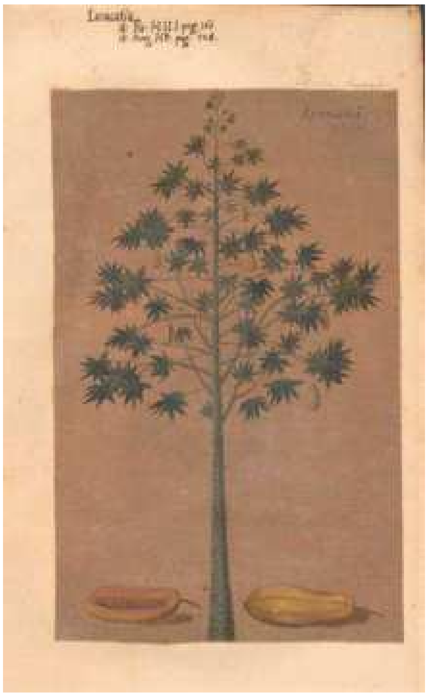

Theatrum Rerum Naturalium: 43

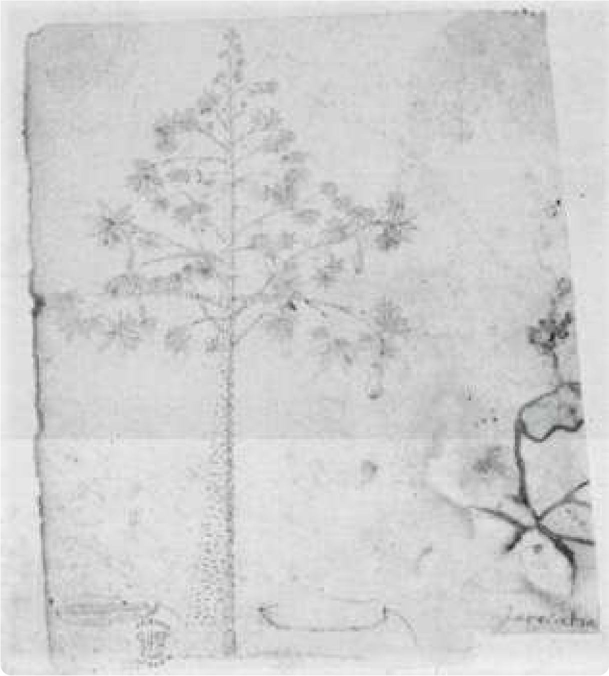

Pencil drawing of *J. spinosa* in De Laet's manuscript: Sloane MS 1554, f. 78v

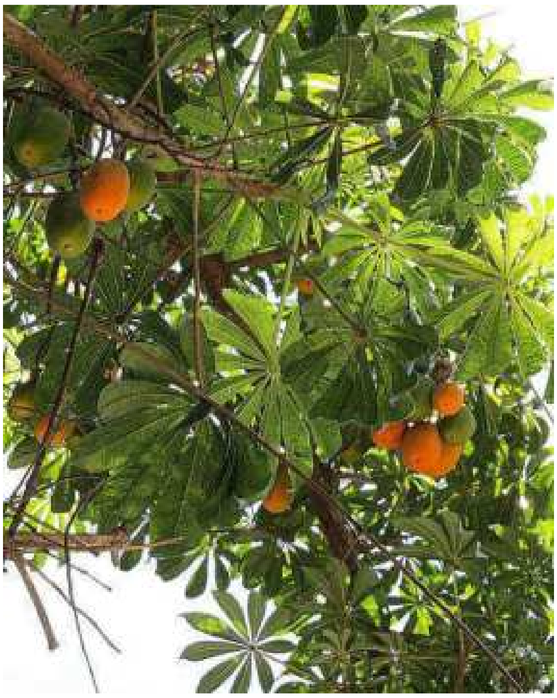

Tree with fruits. "*J. spinosa*" by Dick Culbert (CC BY 2.0)

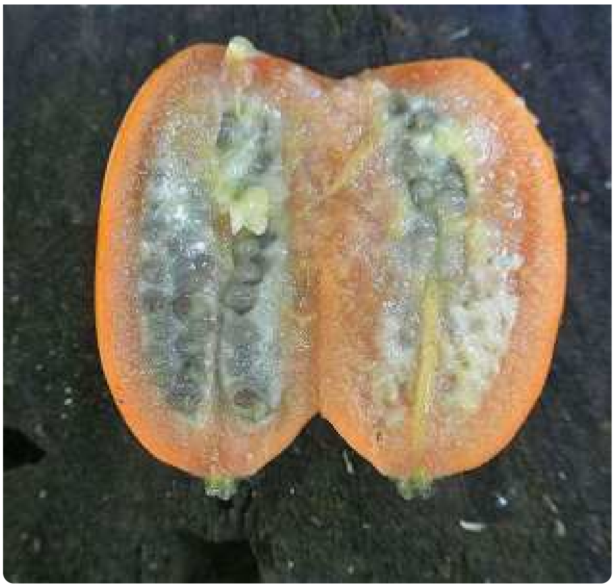

Open fruit and seeds. "*J. spinosa*" by Dick Culbert (CC BY 2.0)

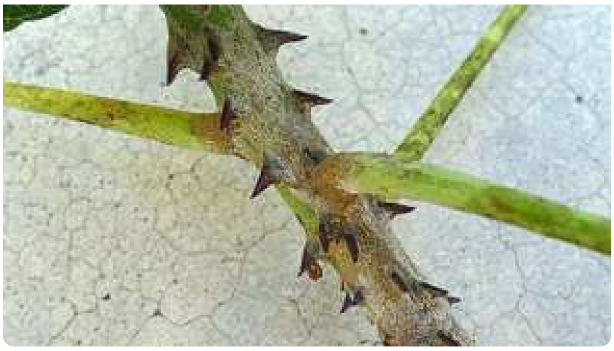

Spines. "*J. spinosa*" by Alex Popovkin, Bahia, Brazil (CC BY-NC-SA 2.0)

Historiae Rerum Naturalium Brasiliae	Marcgrave, 1648	Page number	129b

Vernacular name(s) Acaia. Ibametara

Species Spondias mombin L.

Family Anacardiaceae

Notes

The woodcut bears a strong resemblance with the specimen in Marcgrave's herbarium, as also noticed by Andrade-Lima et al. (1977: 132). De Laet did not indicate this fact in the HNB, as he did with other species. Although Marcgrave (1648: 129) documented flowers and fruits, the specimen consists of a compound leaf.

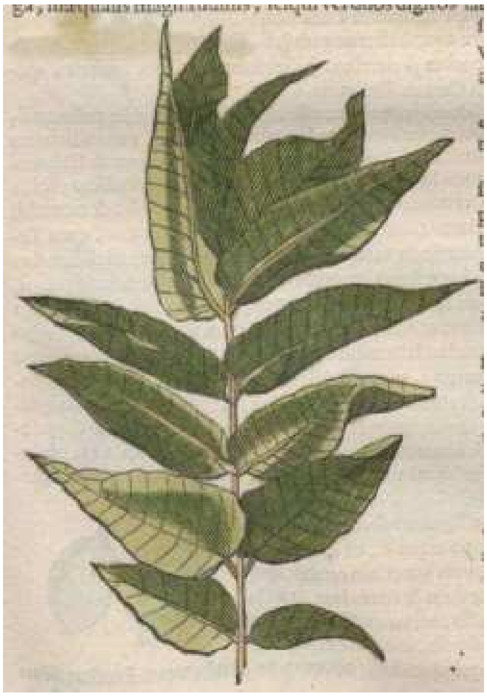

Historiae Plantarum – Arboribus: 129b

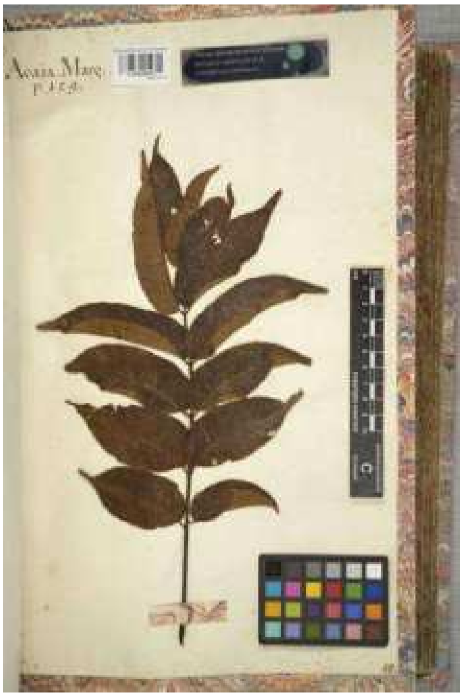

Marcgrave's herbarium: 53

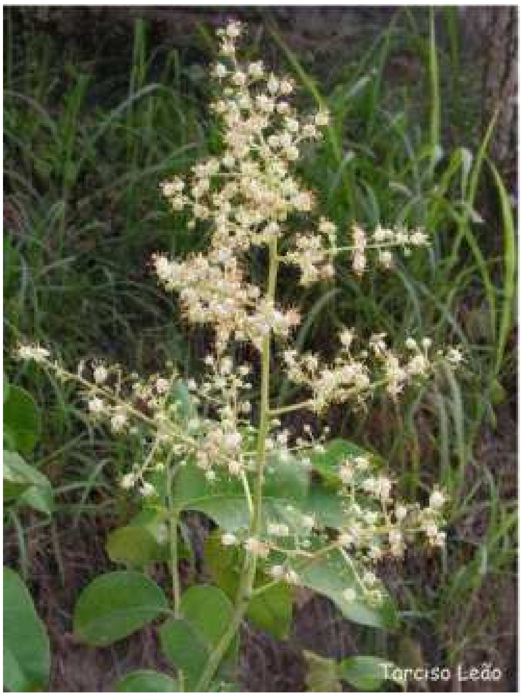

Inflorescence. "*S. mombin*, cajá" by Tarciso Leão (CC BY 2.0)

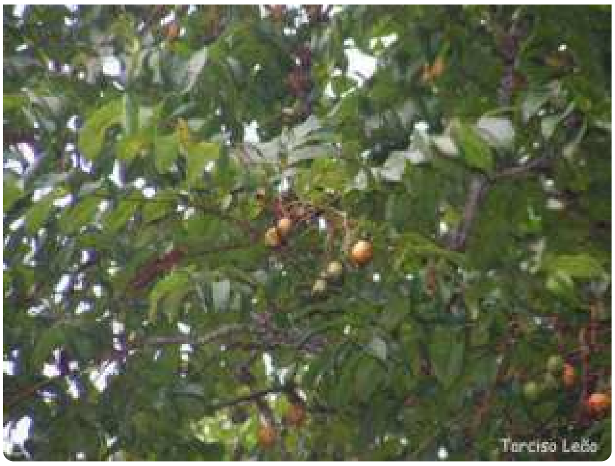

"*S. mombin*, cajá" by Tarciso Leão (CC BY 2.0)

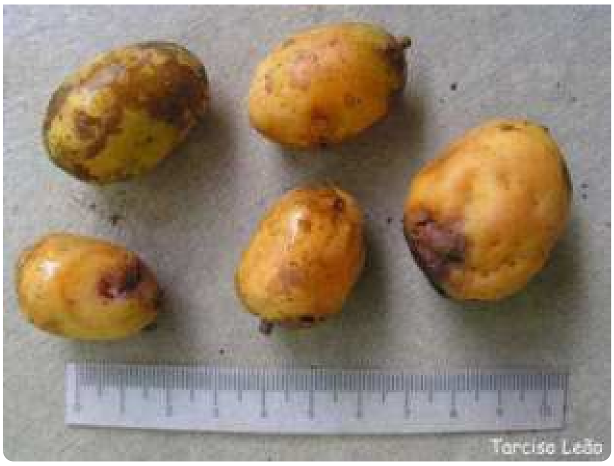

Fruits. "*S. mombin*, cajá" by Tarciso Leão (CC BY 2.0)

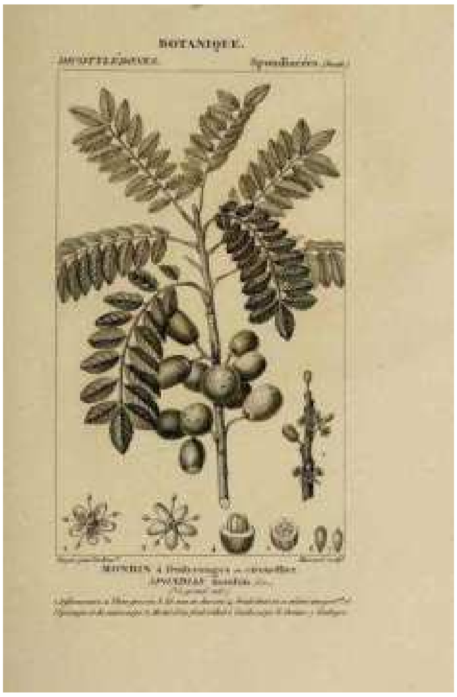

S. mombin in *Dictionnaire des sciences naturelles, Planches Botanique* by Turpin, P.J.F. (1816-1829). Vol. V

Historiae Rerum Naturalium Brasiliae Marcgrave, 1648 Page number 130a

Vernacular name(s) Carana iba. Ananachicariri

Species Copernicia prunifera (Mill.) H.E.Moore

Family Arecaceae

Notes

There is no resemblance between the woodcut and the painting "African woman" by Ekchout. Eckhout's portrait depicts a more elaborated image of this palm tree.

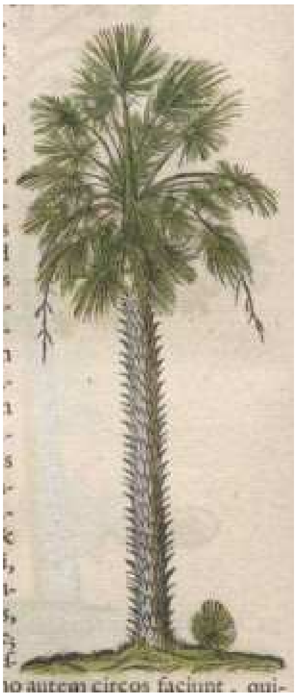

Historiae Plantarum – Arboribus: 130a

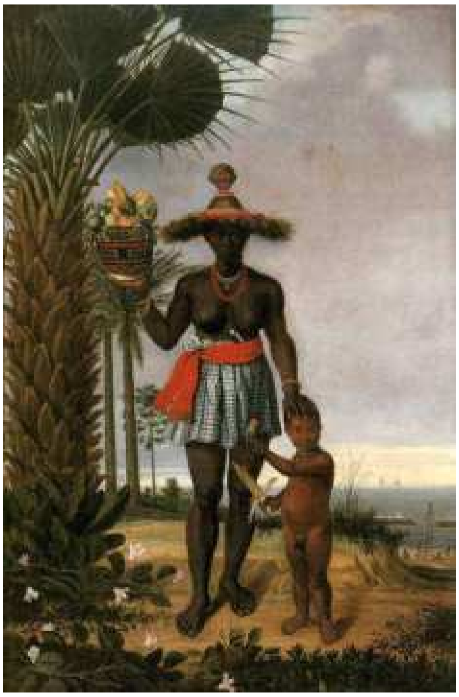

C. prunifera on the left in the portrait "African woman" by Eckhout, ca. 1641. National Museum of Copenhagen, Denmark

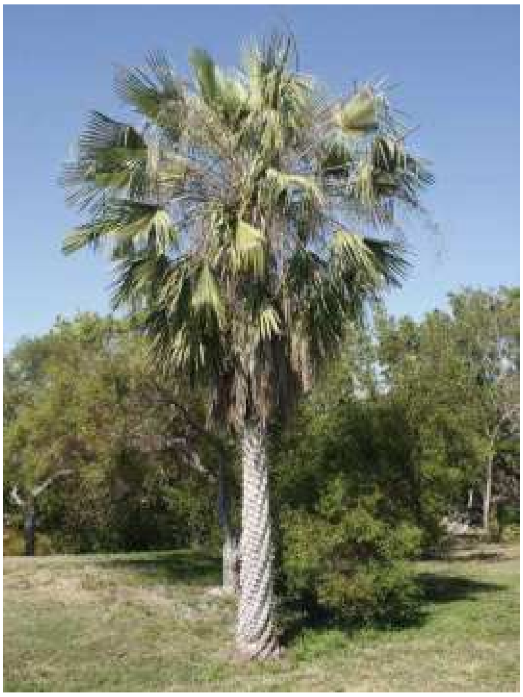

Habit. "*C. prunifera* 1a" by Scott Zona (CC BY-NC 2.0)

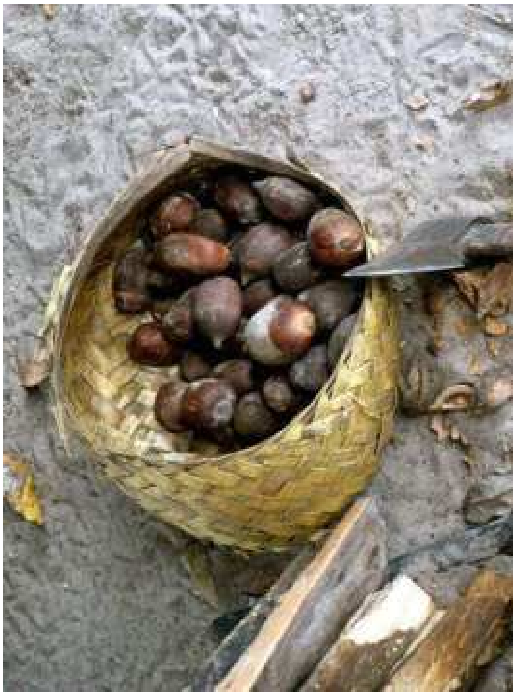

Fruits of *C. prunifera* "Nueces de carnaúba.jpg" by LBM1948 (CC BY-SA 4.0)

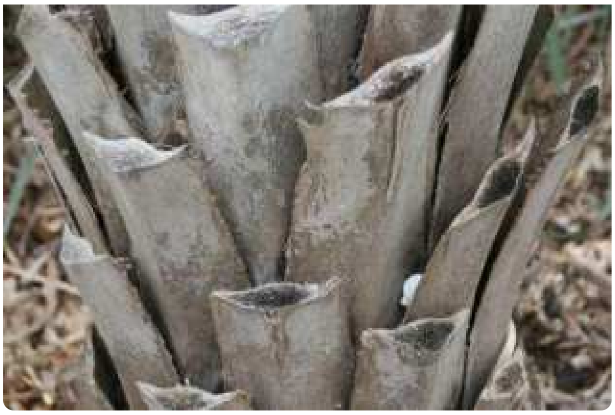

Bark. "*C. prunifera* 0zz.jpg" by Photo by David J. Stang (CC BY-SA 4.0)

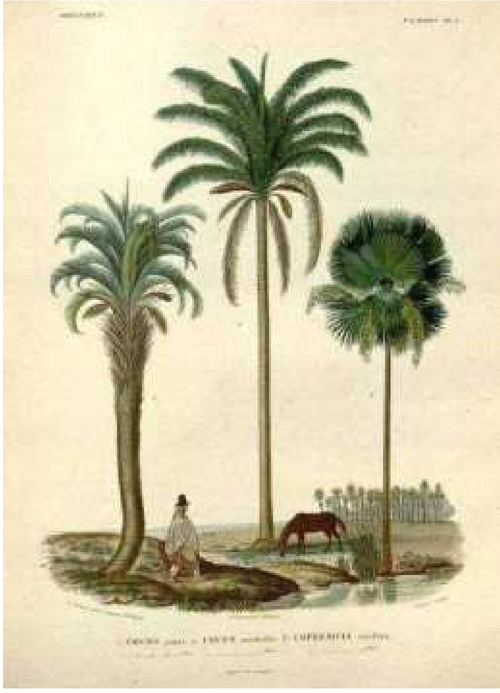

Coconut palms with *C. prunifera* (on the right) in *Voyage dans l'Amérique Méridionale* by Orbigny, A.D. (1835-1847: Vol. 7(3): t.1). Missouri Botanical Garden

Historiae Rerum Naturalium Brasiliae | Marcgrave, 1648 | Page number 130b

Vernacular name(s) Copaiba

Species Copaifera officinalis L.

Family Fabaceae

Notes

We did not find any correspondence between this woodcut and the contemporary or older sources. In De Laet's manuscript, this species is accompanied by four dots, like several of those which were drawn in the Dutch Republic after Marcgrave's herbarium (Andrade-Lima et al. 1977).

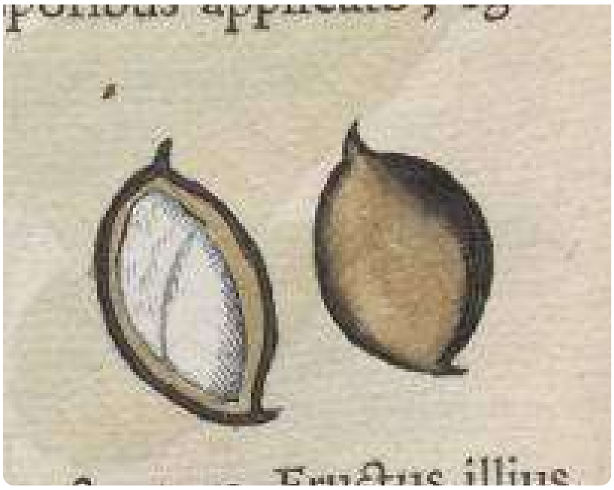

Historiae Plantarum – Arboribus: 130b

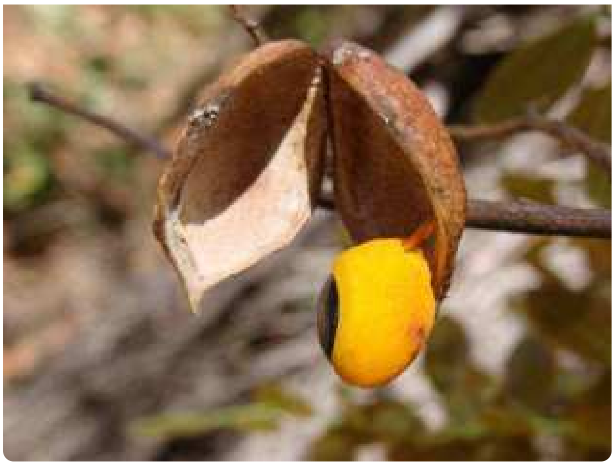

Dehiscent fruit and seed of a related species of *C. officinalis*: "*C. langsdorffii*" by João de Deus Medeiros (CC BY 2.0)

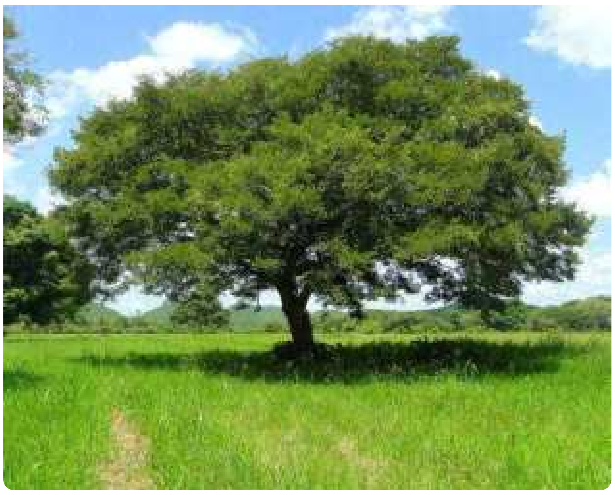

Habit. "Aceite, Palo de Aceite, Copaiba [Copaiba Balsam] (*C. officinalis*)" by barloventomagico (CC BY-NC-ND 2.0)

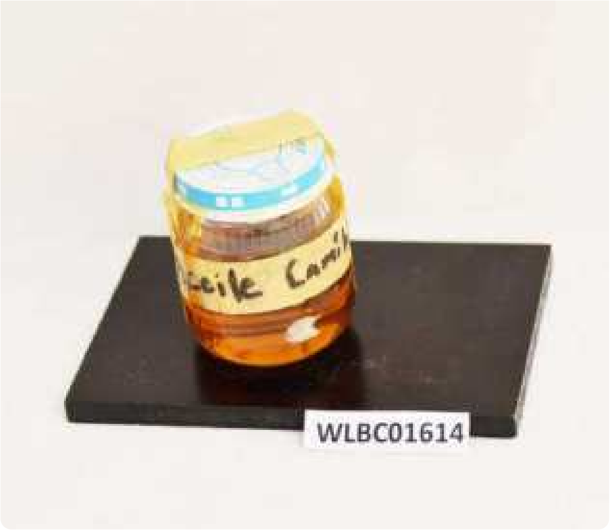

C. officinalis oil in the collection of the Missouri Botanical Garden -Salick 10037, Nicaragua

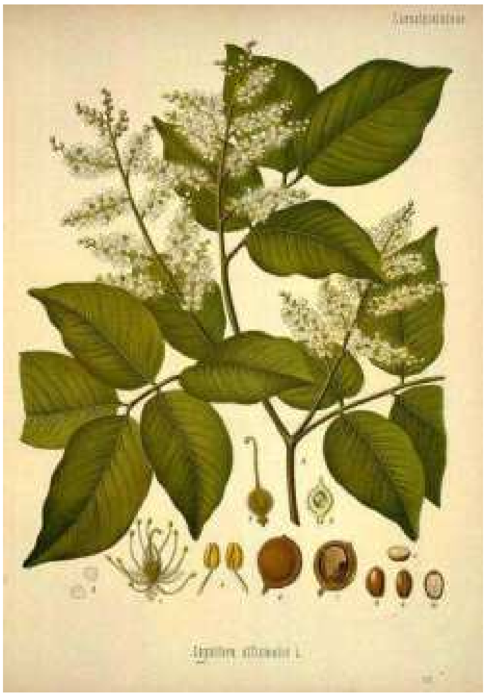

C. officinalis in *Kohler's Medizinal Pflanzen* by Kohler, F.E. (1890: Vol. II, t. 171). Missouri Botanical Garden, St. Louis, U.S.A.

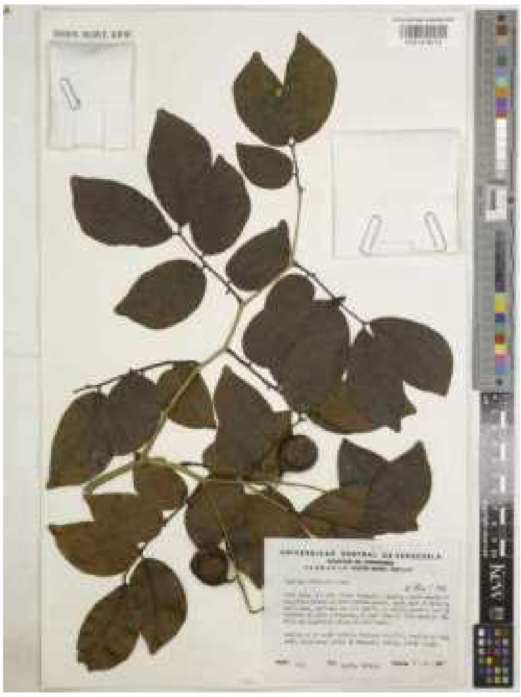

Specimen of C. officinalis from Kew's Herbarium - K001478012. Retrieved from Plants of the World Online

Historiae Rerum Naturalium Brasiliae Marcgrave, 1648 Page number 131a

Vernacular name(s) Ibixuma. Motamba

Species Guazuma ulmifolia Lam.

Family Malvaceae

Notes

The woodcut differs from the *Theatrum* image. In De Laet's manuscript, there is an ink drawing that corresponds to this image. This drawing does not match with any of the woodcuts in the HNB, but -in contrast to what Andrade-Lima et al. (1977) indicated, this bears a strong resemblance to the specimen (p. 37) in Copenhagen. The ink drawing was most likely made after it, possibly in Brazil. Otherwise, why De Laet would spend resources to make a new drawing that he did not use?

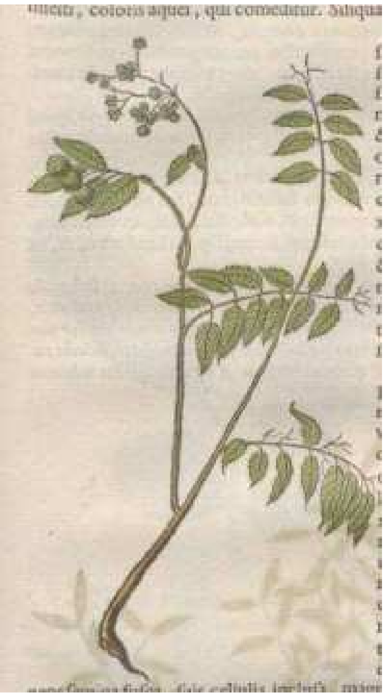

Historiae Plantarum – Arboribus: 131a

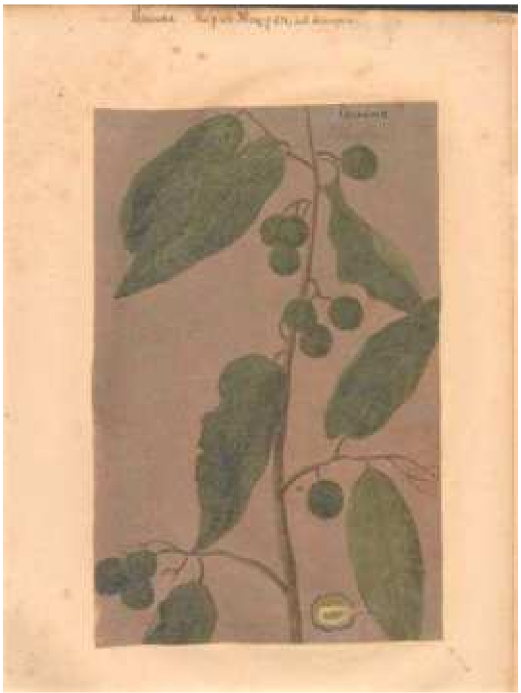

Theatrum Rerum Naturalium: 347

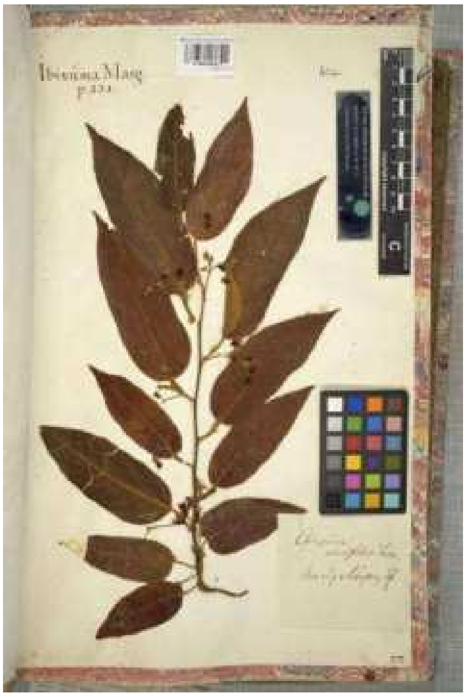

Marcgrave's herbarium: 37

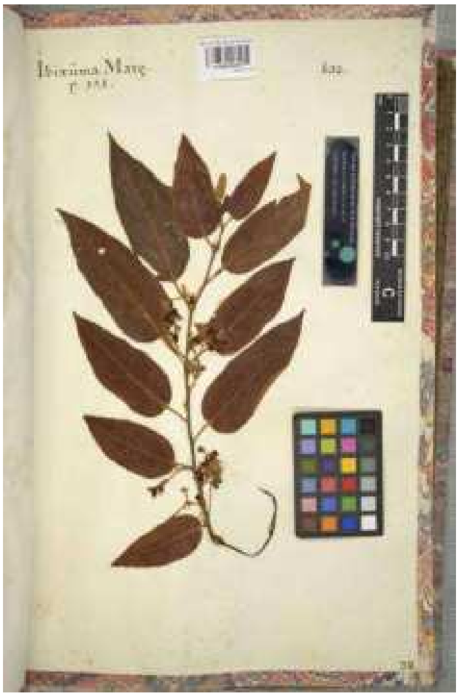

Marcgrave's herbarium: 38

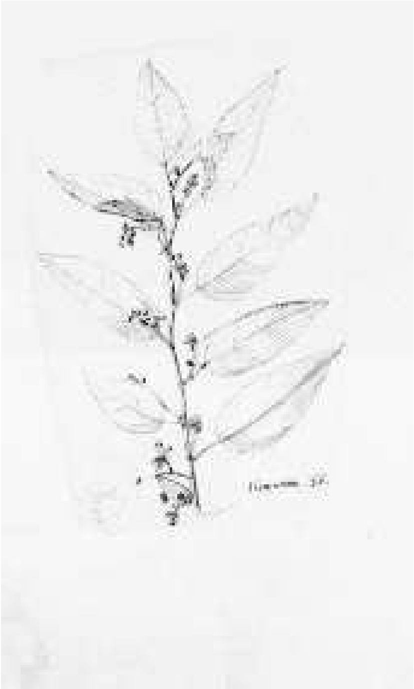

Ink drawing of *G. ulmifolia* in De Laet's manuscript: Sloane MS 1554, f. 58v

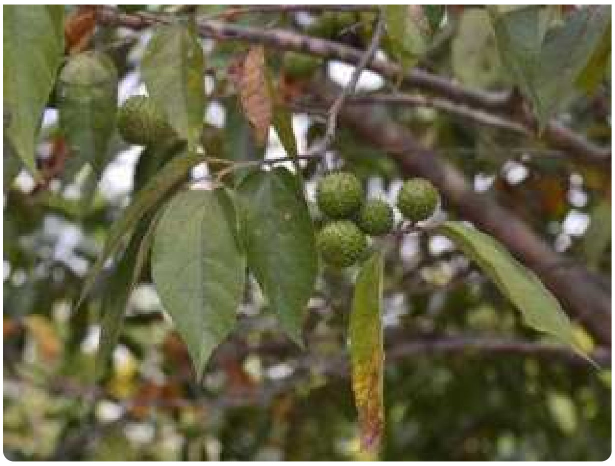

Fruiting branch. "*G. ulmifolia*" by Mauricio Mercadante (CC BY-NC-SA 2.0)

Historiae Rerum Naturalium Brasiliae | Marcgrave, 1648 | Page number 131b

Vernacular name(s): Coapoiba

Species: Clusia nemorosa G.Mey.

Family: Clusiaceae

Notes

The woodcut bears a strong resemblance to the specimen in Marcgrave's herbarium (p. 32) although De Laet did not indicate it in the HNB - as he often did with other woodcuts made after the exsiccates.

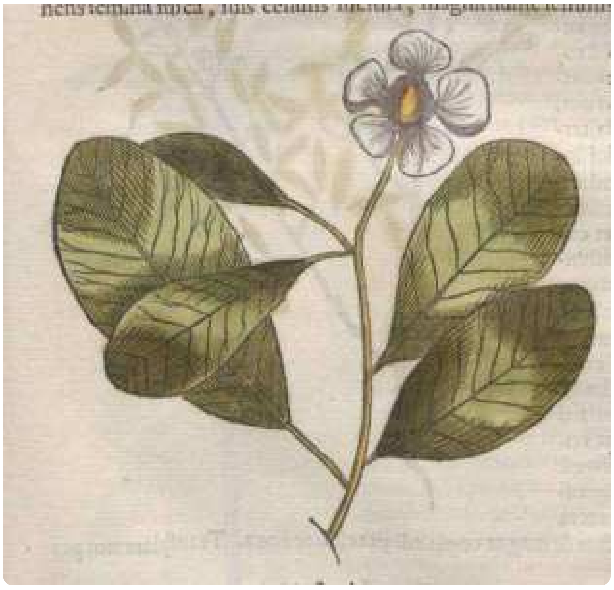

Historiae Plantarum – Arboribus: 131b

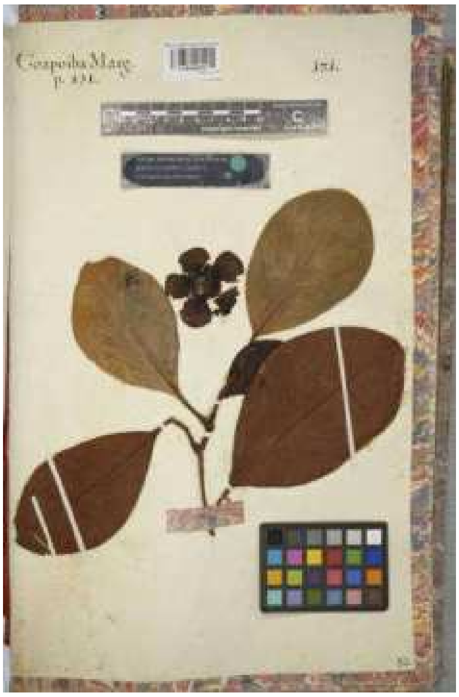

Marcgrave's herbarium: 32

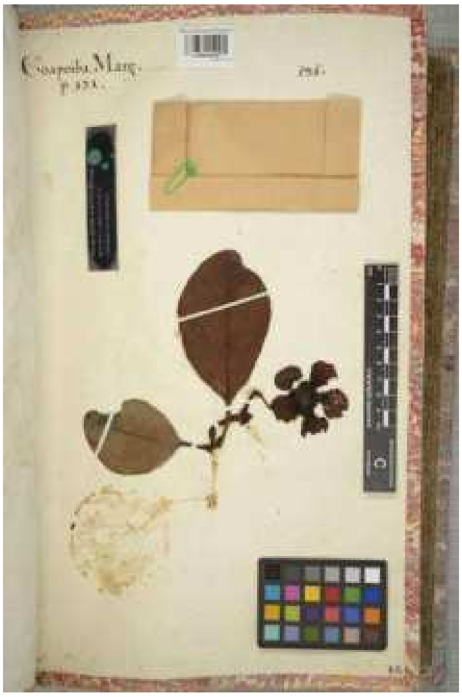

Marcgrave's herbarium: 48

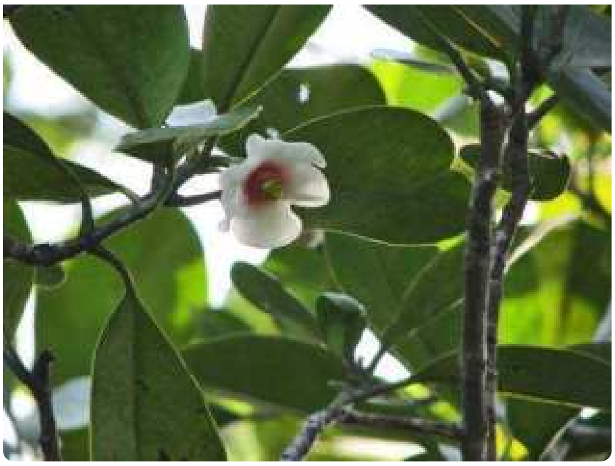

Flowering branch. "*C. nemorosa*" by Alex Popovkin, Bahia, Brazil (CC BY-NC-SA 2.0)

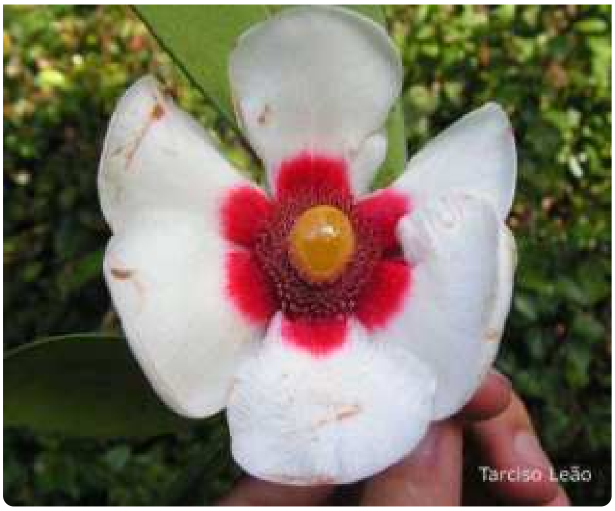

Flower. "*C. nemorosa*, pororoca, orelha-de-burro" by Tarciso Leão (CC BY 2.0)

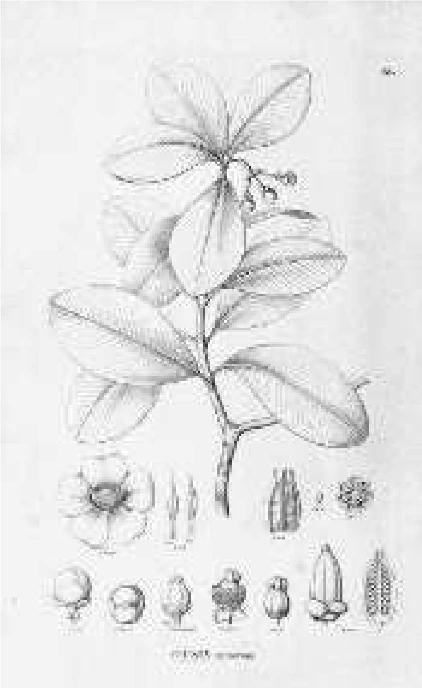

Engraving of *C. nemorosa* in Martius, C.F.P. von, Eichler, A.G., Urban, I., *Flora Brasiliensis* (1858-1879) Vol. 12 (1): t. 88

Historiae Rerum Naturalium Brasiliae	Marcgrave, 1648	Page number	132

Vernacular name(s) Ibiruba

Species Eugenia selloi B.D.Jacks.

Family Myrtaceae

Notes

We did not find any correspondence between this woodcut and the contemporary or older sources.

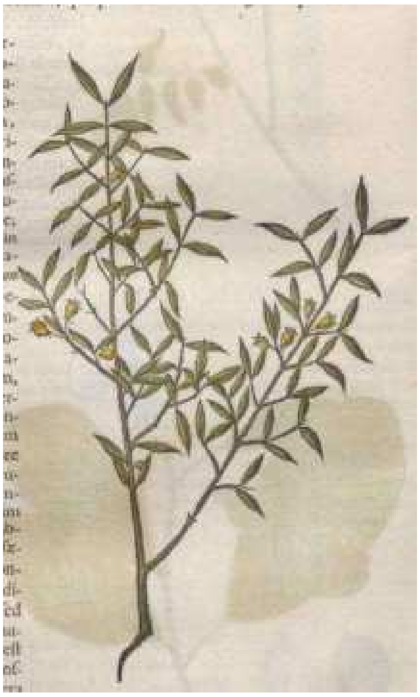

Historiae Plantarum – Arboribus: 132

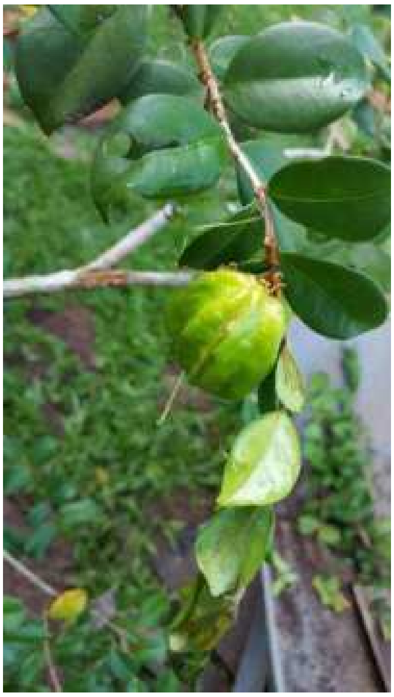

Fruiting branch of *E. selloi*. "Espécime jovem de pitangatuba" by Fredericoscb (CC BY-SA 4.0)

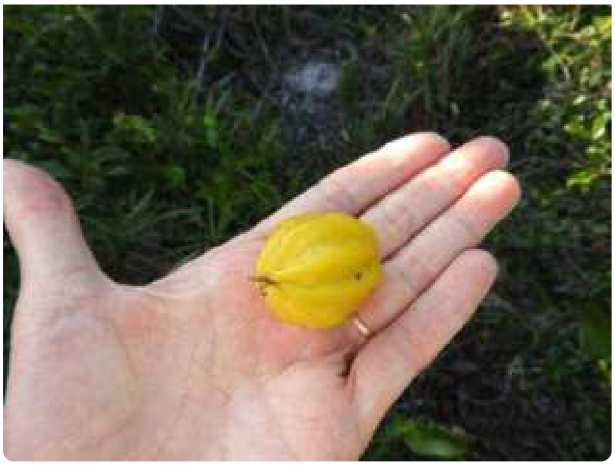

The fruit of *E. selloi* observed in Brazil
by nena_bergallo for iNaturalist. Retrieved from GBIF

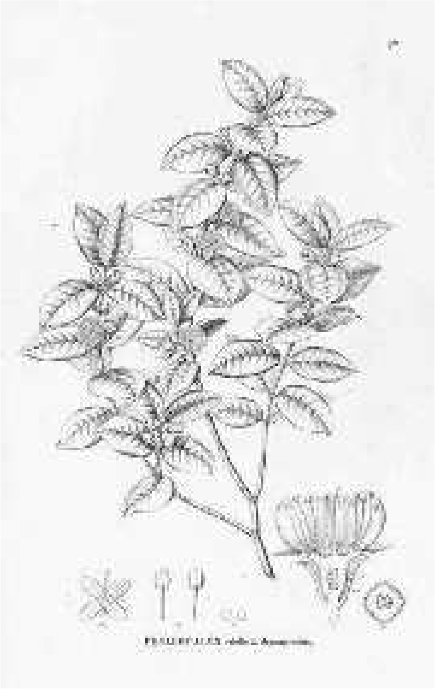

Engraving of *E. selloi* in Martius, C.F.P. von, Eichler, A.G., Urban, I., *Flora Brasiliensis* (1857-1859) Vol. 14 (1): t. 29

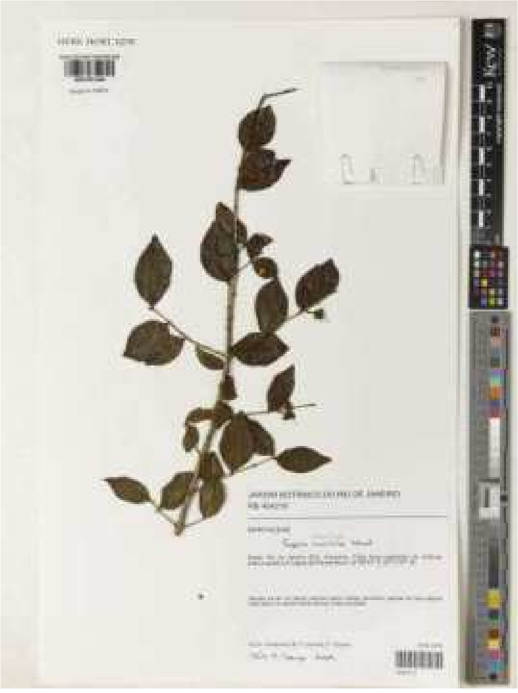

Specimen of *E. selloi* from Kew's Herbarium - K001051669. Retrieved from Plants of the World Online

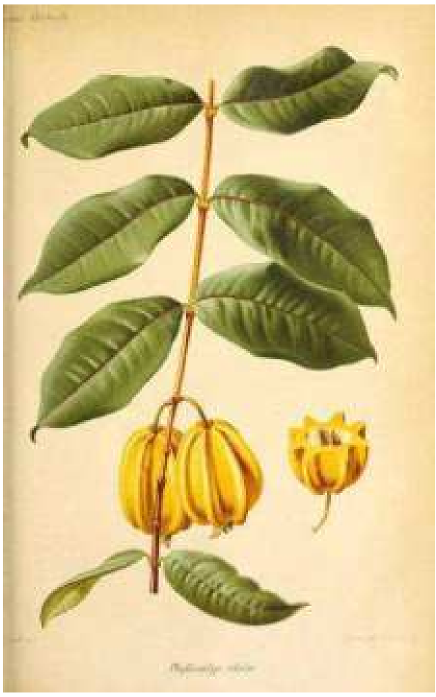

Illustration of *E. selloi* in *Revue horticole*, sér. 4 (1884: Vol. 56). New York Botanical Garden, U.S.A.

Historiae Rerum Naturalium Brasiliae	Marcgrave, 1648	Page number	133a

Vernacular name(s) Arbor

Species Guettarda platypoda DC.

Family Rubiaceae

Notes

Strong correspondence between woodcut and specimen. De Laet (Marcgrave 1648: 133) indicated that someone made this figure after dry plants collected by Marcgrave, although the artist took some freedom with the drawing of the venation, which is not that accurate.

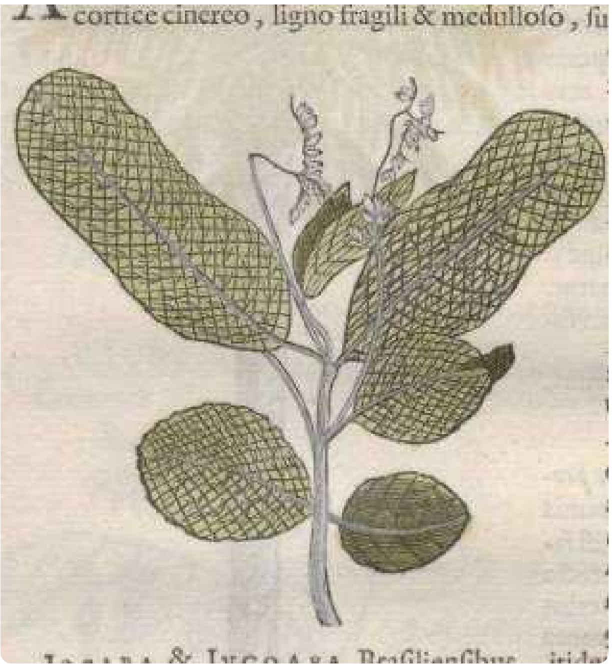

Historiae Plantarum – Arboribus: 133a

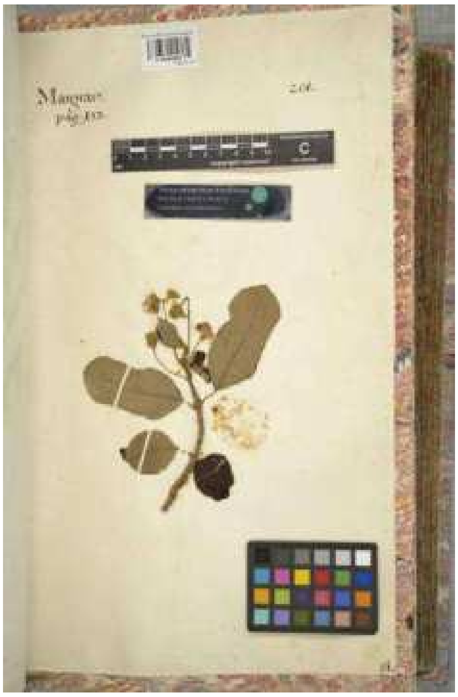

Marcgrave's herbarium: 51

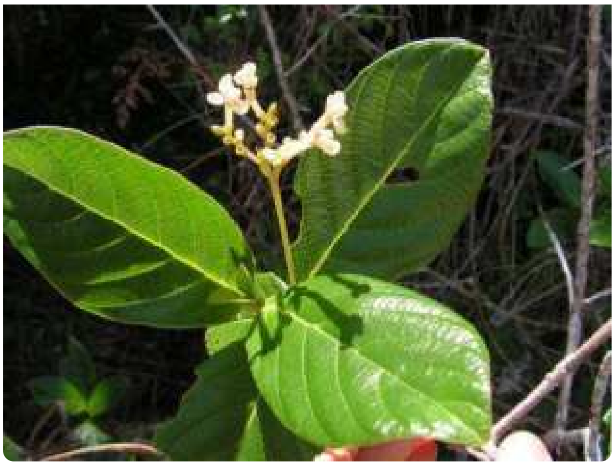

Flowering branch. "*G. platypoda*" by Tarciso Leão (CC BY 2.0)

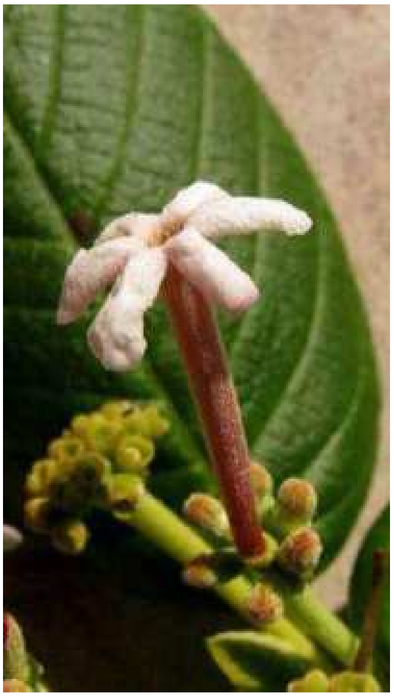

Flower. "*G. platypoda*" by Alex Popovkin, Bahia, Brazil (CC BY-NC-SA 2.0)

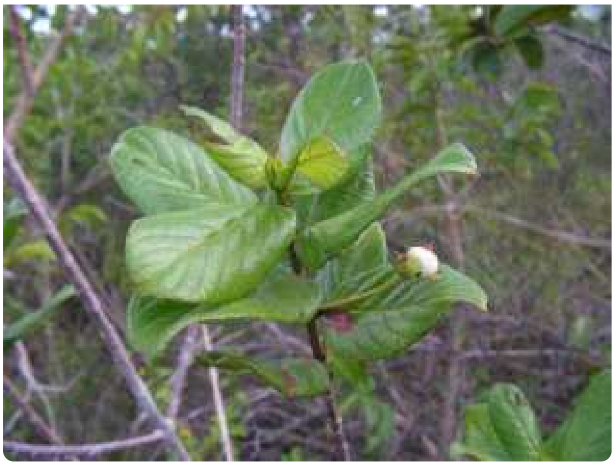

Fruiting branch. "*G. platypoda*" by Tarciso Leão (CC BY 2.0)

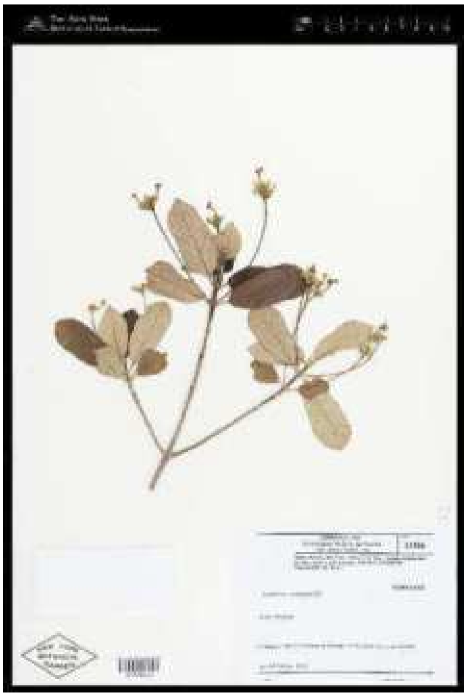

G. platypoda collected in Brazil by The New York Botanical Garden (CC BY-SA 4.0)

Historiae Rerum Naturalium Brasiliae	Marcgrave, 1648	Page number	133b

Vernacular name(s) Iocara. Iucoara

Species Euterpe edulis Mart.

Family Arecaceae

Notes

We did not find any correspondence between this woodcut and the contemporary or older sources.

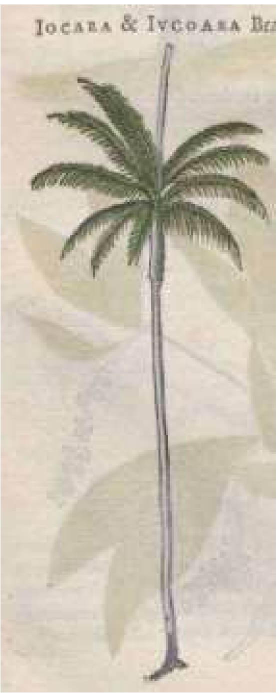

Historiae Plantarum – Arboribus: 133b

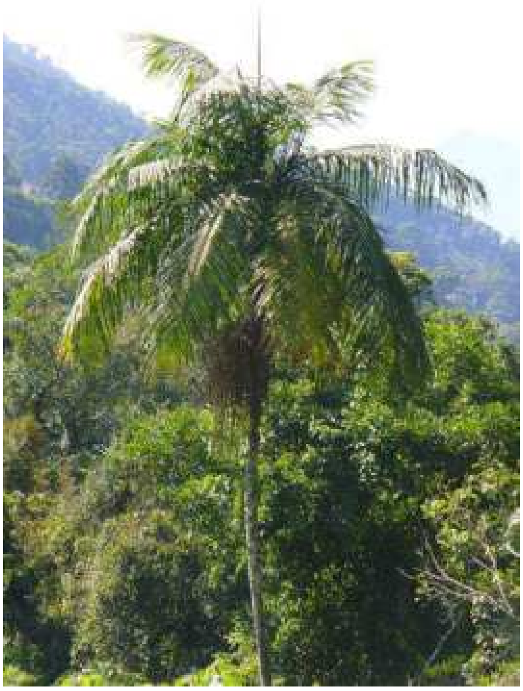

"*E. edulis*" by Scott Zona (CC BY-NC 2.0)

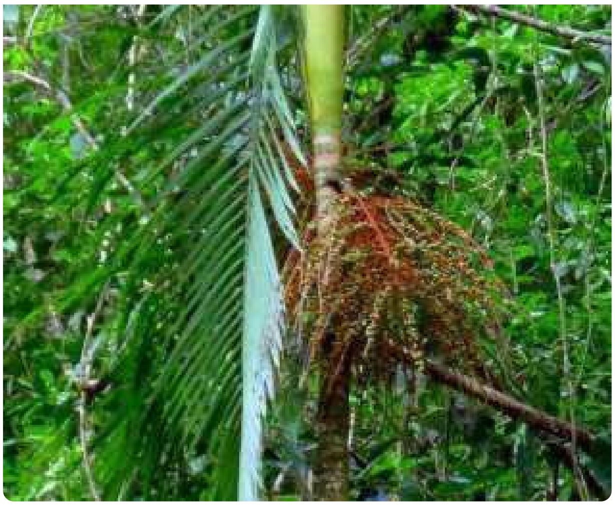

"*E. edulis*" by Alex Popovkin, Bahia, Brazil (CC BY-NC-SA 2.0)

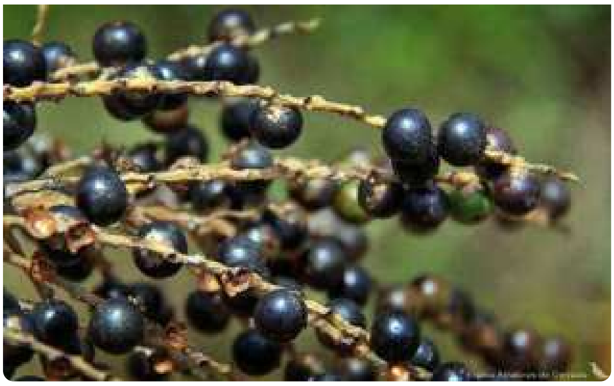

"*E. edulis*- Açaí do cerrado" by Marcelo_Kuhlmann (CC BY-NC-SA 2.0)

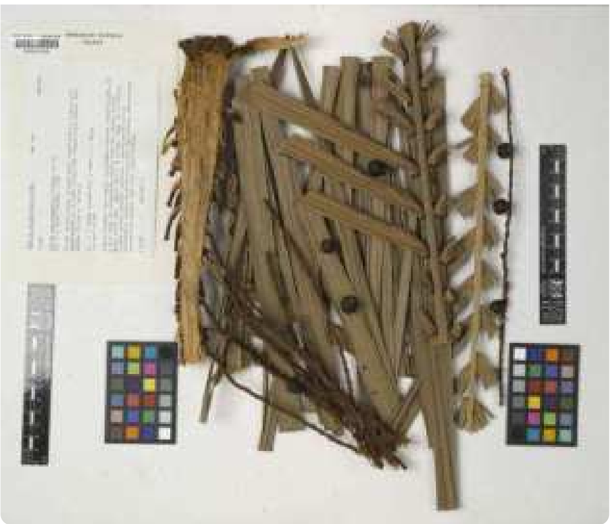

Specimen of *E. edulis* from Kew's Herbarium - K000574535. Retrieved from Plants of the World Online

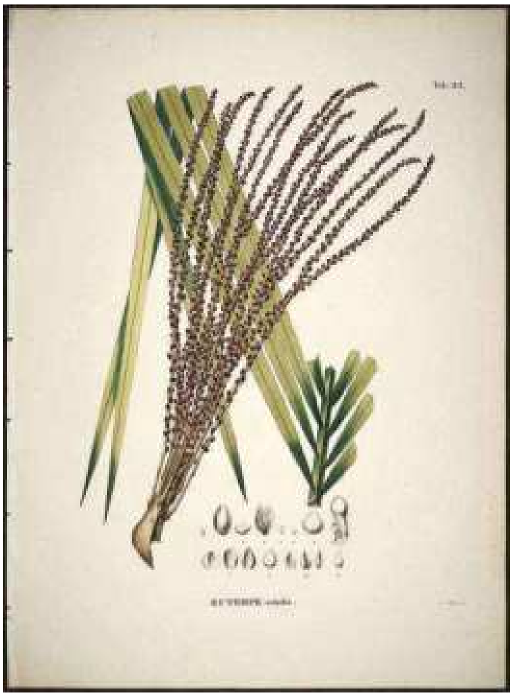

E. edulis in *Historia Naturalis Palmarum* by Martius, C. F.P. von(1839: Vol. II, t. 32). Missouri Botanical Garden, St. Louis, U.S.A.

Historiae Rerum Naturalium Brasiliae | Marcgrave, 1648 | Page number 134a

Vernacular name(s) Pindoba

Species Attalea compta Mart.

Family Arecaceae

Notes

There is no correspondence between the woodcut image and the chalk drawing in the *Misc. Cleyeri*. The palm depicted in Barlaeus' book, presumably drawn by Post, bears some resemblance to the woodcut.

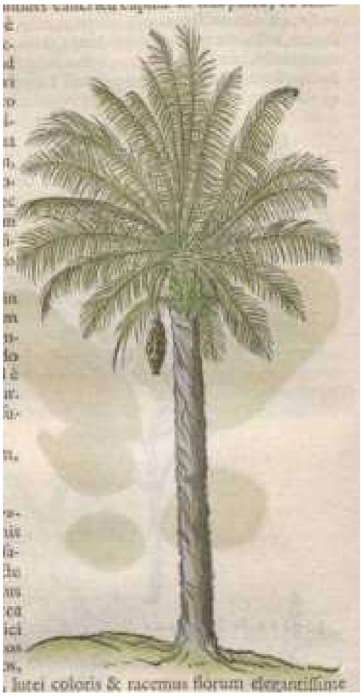

Historiae Plantarum – Arboribus: 134a

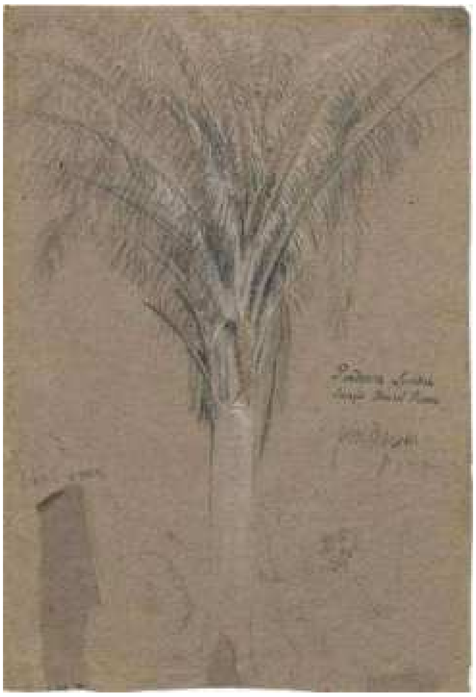

Miscellanea Cleyeri: 67r

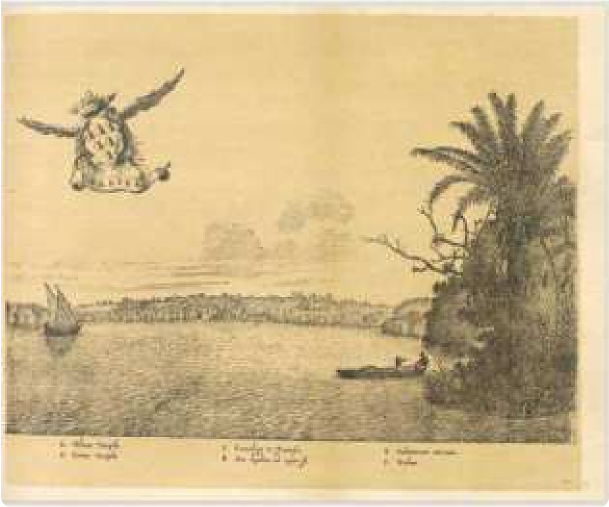

A. compta (on the right) in *Rerum per octennium in Brasilia et alibi* by Caspar Barlaeus (1647: 158-159), designed made by Post

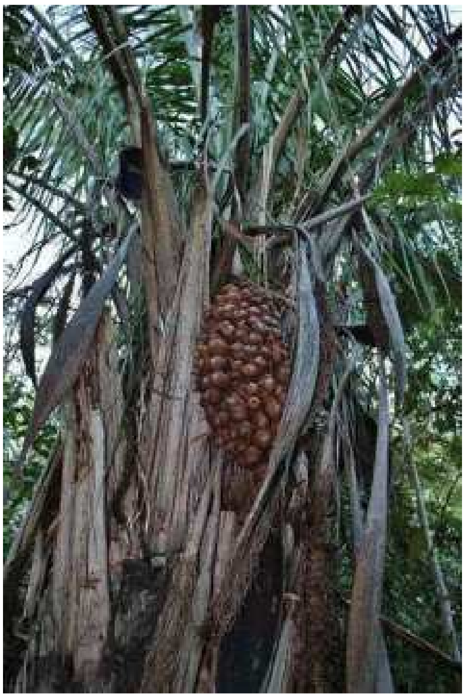

"*A. compta*" by Mauricio Mercadante (CC BY-NC-SA 2.0)

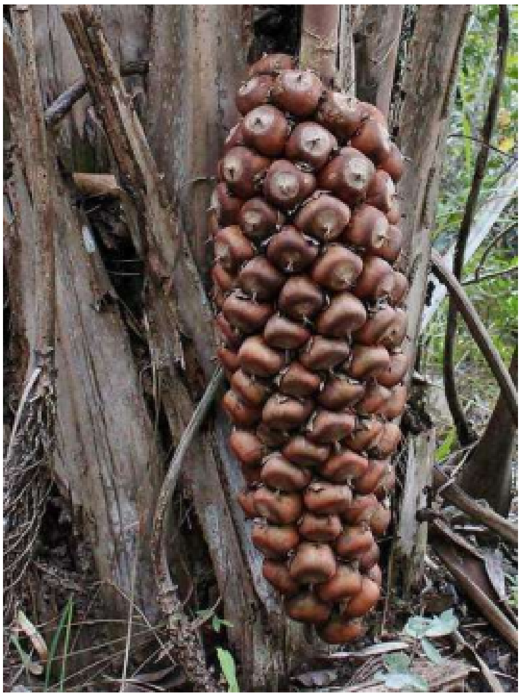

"*A. compta*" by Mauricio Mercadante (CC BY-NC-SA 2.0)

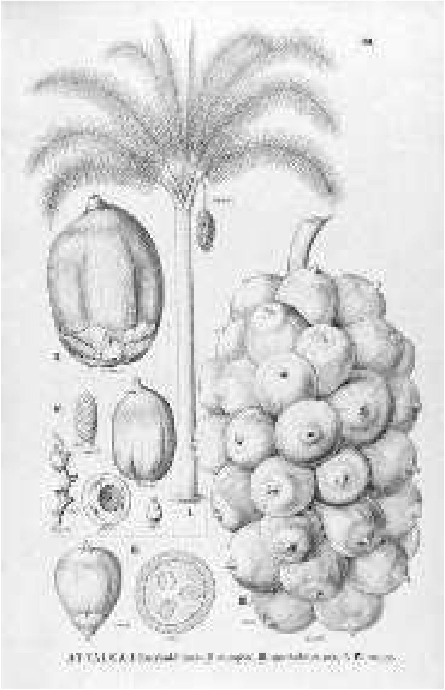

Engraving of *A. compta* in Martius, C.F.P. von, Eichler, A.G., Urban, I., *Flora Brasiliensis* (1878-1882) Vol. 3 (2): t. 99

Historiae Rerum Naturalium Brasiliae | Marcgrave, 1648 | Page number 134b

Vernacular name(s) Arbor

Species Byrsonima sericea DC.

Family Malpighiaceae

Notes

Strong correspondence between woodcut and specimen. De Laet (Marcgrave 1648: 134) indicated that they made this figure after the dry plants that were collected by Marcgrave. De Laet made commentaries on the leaves' coloration and the impossibility of describing the fruits more accurately because they were dry. Jakob Breyne (1678: 34) cited Marcgrave and Piso in his book and included an engraving of this species, which he likely made after a duplicate collected in Brazil by Marcgrave.

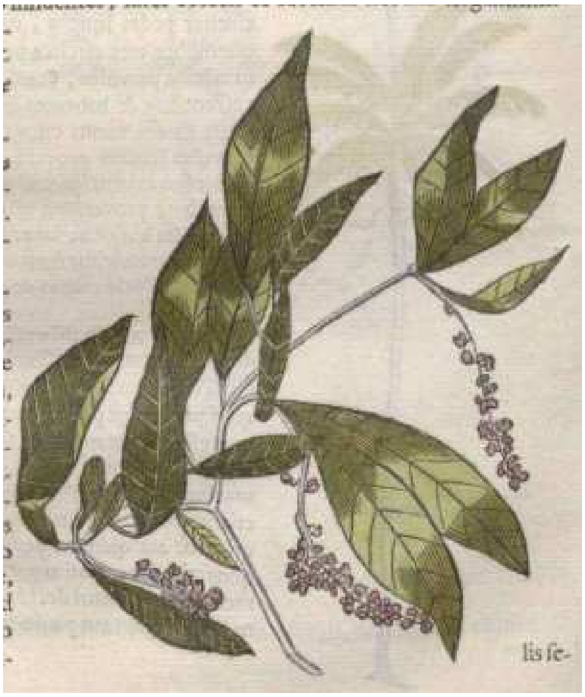

Historiae Plantarum – Arboribus: 134b

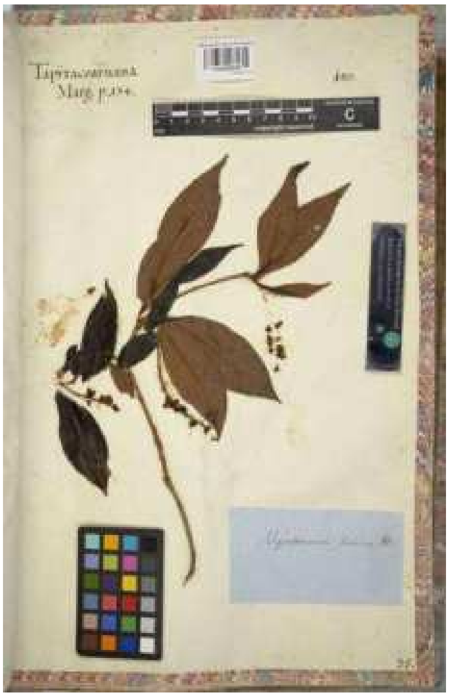

Marcgrave's herbarium: 35

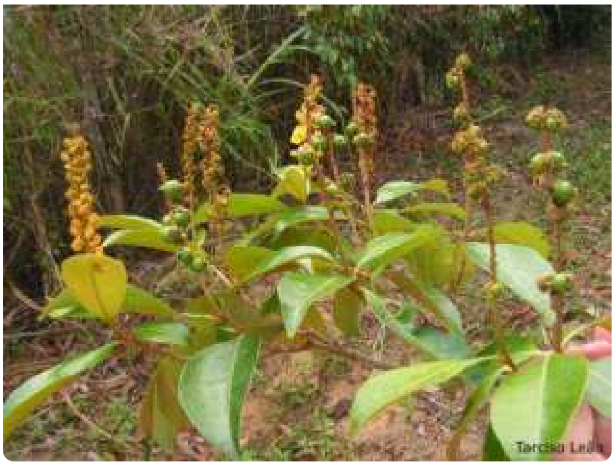

"*B. sericea, murici*" by Tarciso Leão (CC BY 2.0)

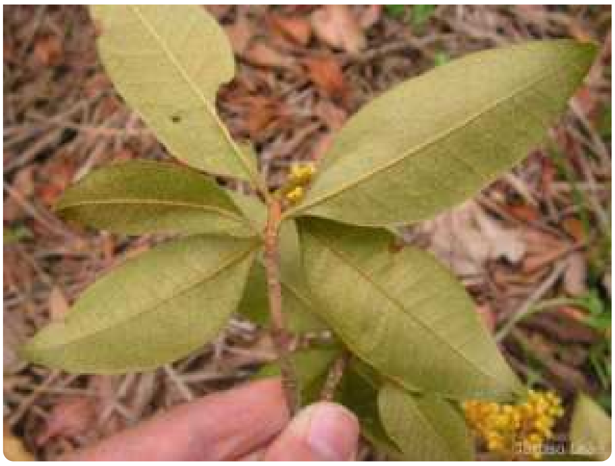

"*B. sericea, murici*" by Tarciso Leão (CC BY 2.0)

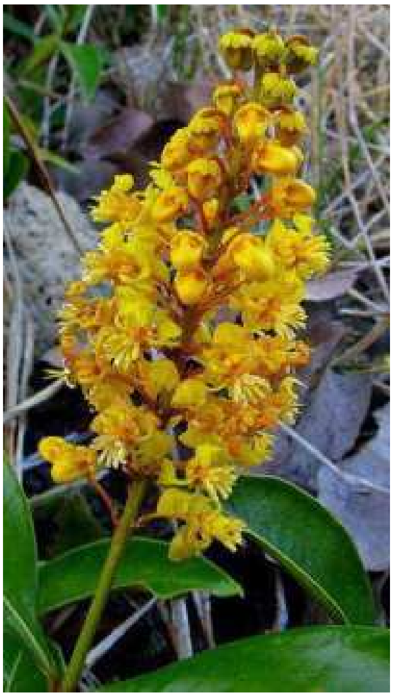

Inflorescence. "*B. sericea"* by Alex Popovkin, Bahia, Brazil (CC BY-NC-SA 2.0)

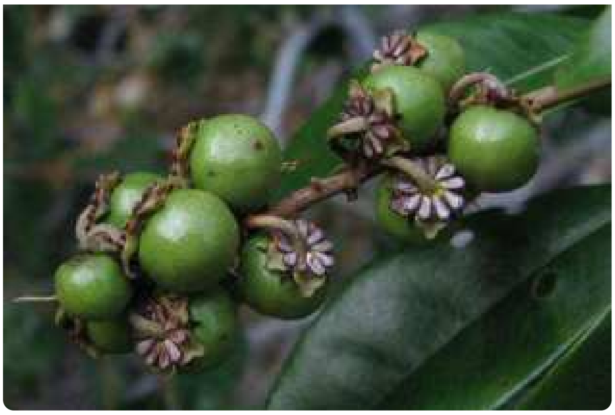

Fruits. "*B. sericea"* by Alex Popovkin, Bahia, Brazil (CC BY-NC-SA 2.0)

Historiae Rerum Naturalium Brasiliae	Marcgrave, 1648	Page number	135b

Vernacular name(s) Quera iba

Species Tabebuia aurea (Silva Manso) Benth. & Hook.f. ex S.Moore

Family Bignoniaceae

Notes

We did not find any correspondence between this woodcut and the contemporary or older sources.

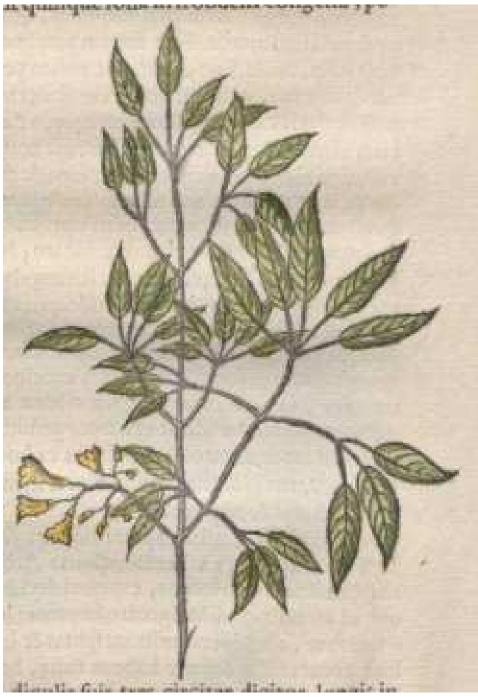

Historiae Plantarum – Arboribus: 135b

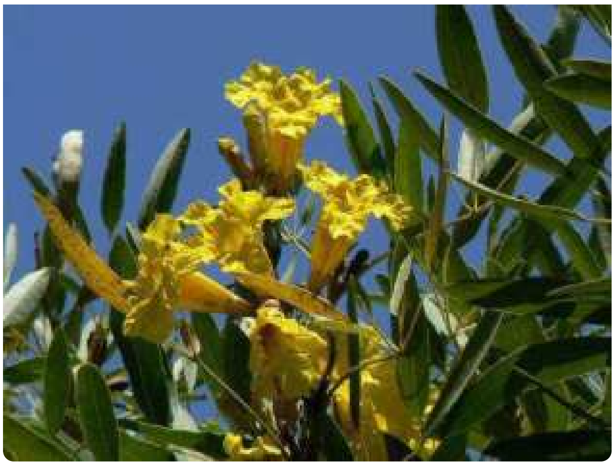

"*T. aurea*" by Dinesh Valke (CC BY-SA 2.0)

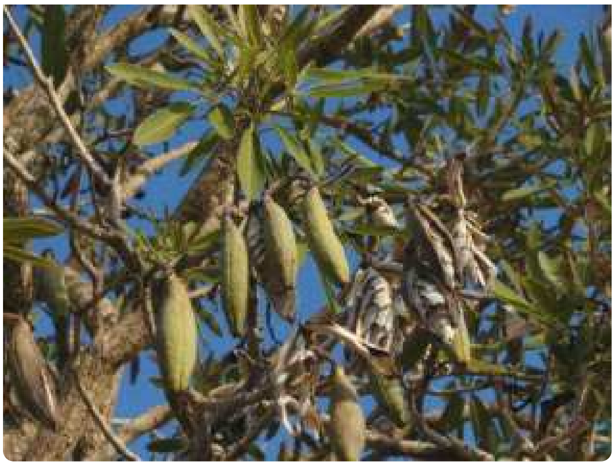

"*T. aurea* seed pods on the tree" by Tatters ✾ (CC BY 2.0)

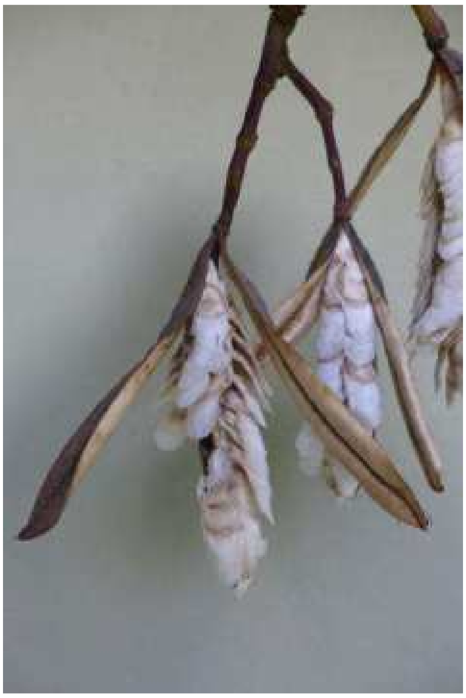

"*T. aurea* fruits 2" by Scott Zona (CC BY-NC 2.0)

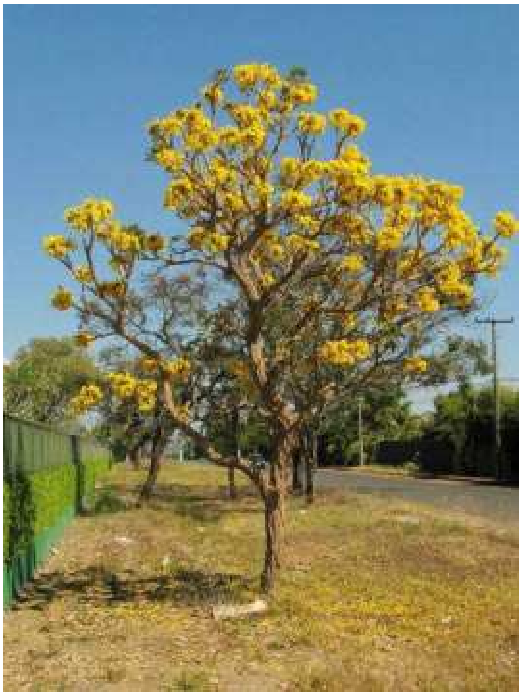

Habit. "*T. aurea*" by Mauricio Mercadante (CC BY-NC-SA 2.0)

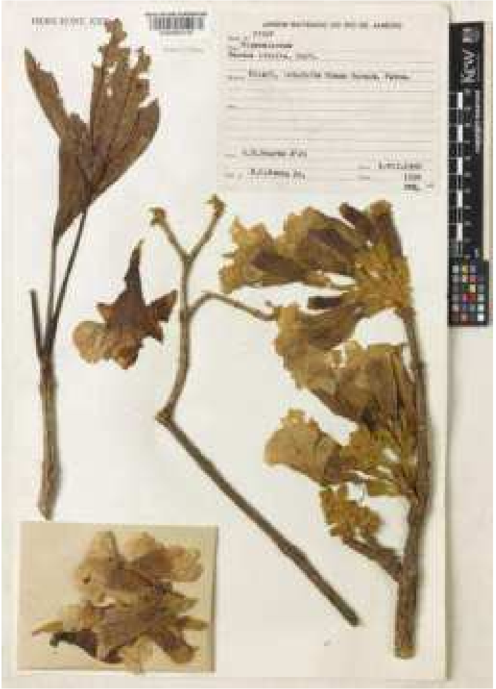

Specimen of *T. aurea* from Kew's Herbarium - K000903176. Retrieved from Plants of the World Online

Historiae Rerum Naturalium Brasiliae Marcgrave, 1648 Page number 135a

Vernacular name(s) Iacaranda ligno albo

Species Swartzia pickelii Ducke (mixed with Sparattanthelium cf. tupiniquinorum)

Family Fabaceae

Notes

The woodcut differs from the *Theatrum* image. We agree with Pickel (2008: 108) that the image was likely misplaced. It looks like *Sparattanthelium tupiniquinorum* or *S. botocudorum*, which was depicted in the *Theatrum* (f. 355) and collected as a specimen (p. 171). These sources do not bear resemblance to the woodcut neither.

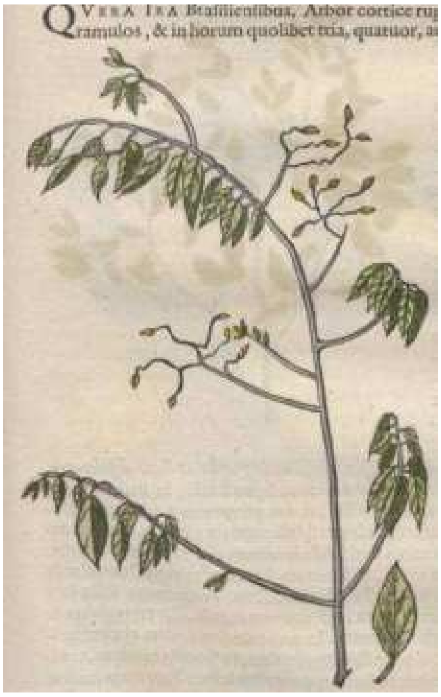

Historiae Plantarum – Arboribus: 135a

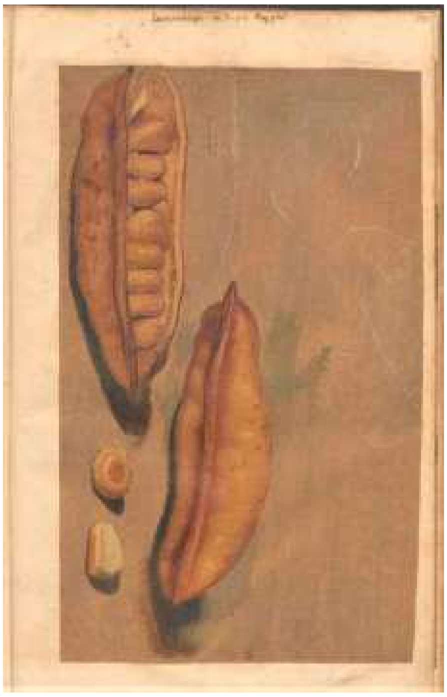

Theatrum Rerum Naturalium: 391

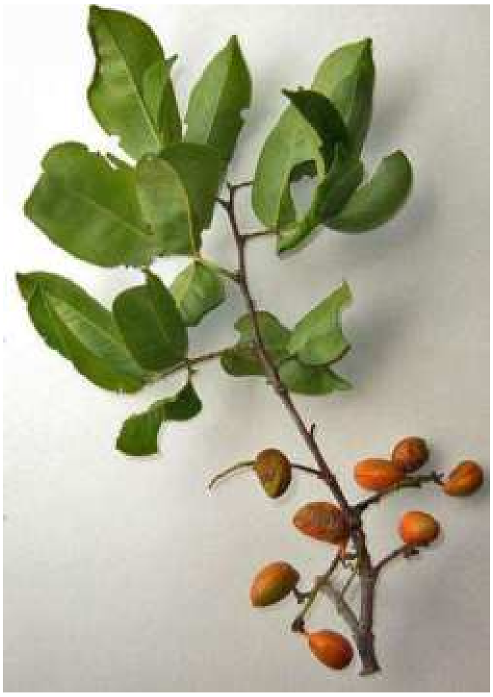

A related species to *S. pickelii* from Brazil "*S. apetala* Raddi var. *subcordata*" by Alex Popovkin, Bahia (C BY-NC-SA 2.0)

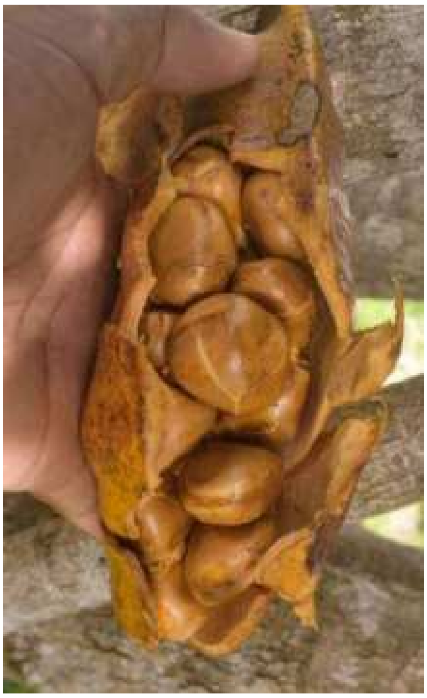

S. pickelii pod and seeds. "Jacarandá branco" in Bioma urbano

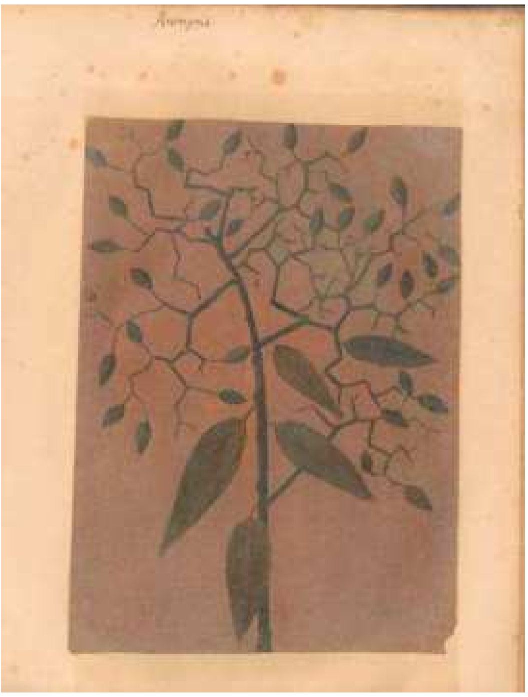

Oil painting of *Sparattanthelium* cf. *tupiniquinorum* in *the Theatrum Rerum Naturalium*: 355

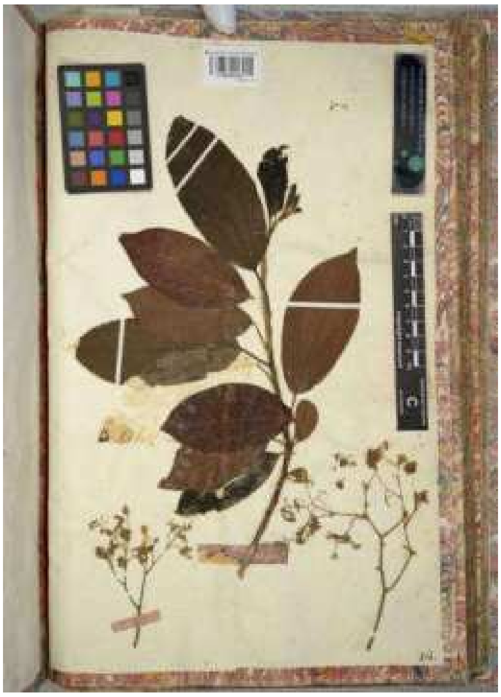

Specimen of *Sparattanthelium* cf. *tupiniquinorum* or *S. botocudorum* in Marcgrave's herbarium: 161

Historiae Rerum Naturalium Brasiliae Marcgrave, 1648 Page number 136

Vernacular name(s) Ibiraba

Species Eschweilera ovata (Cambess.) Mart. ex Miers

Family Lecythidaceae

Notes

The woodcut differs from the *Theatrum* image and the herbarium specimen, which is an sterile shoot.

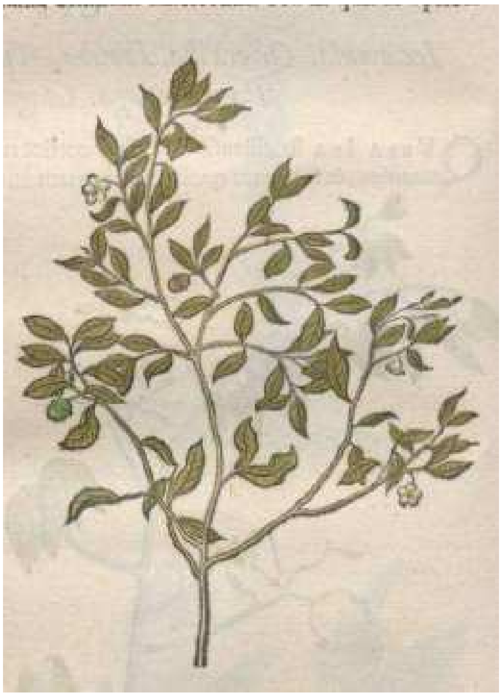

Historiae Plantarum – Arboribus: 136

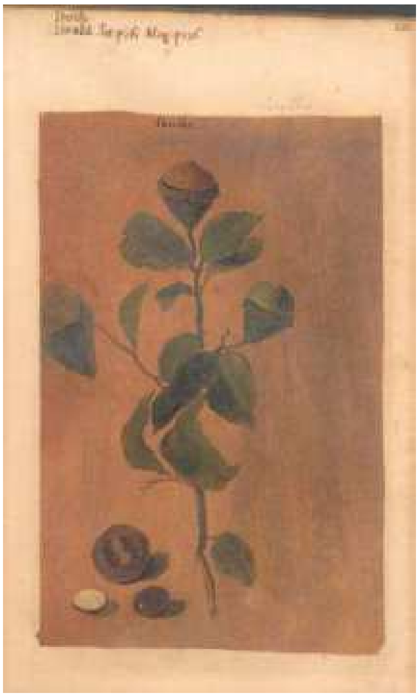

Theatrum Rerum Naturalium: 201

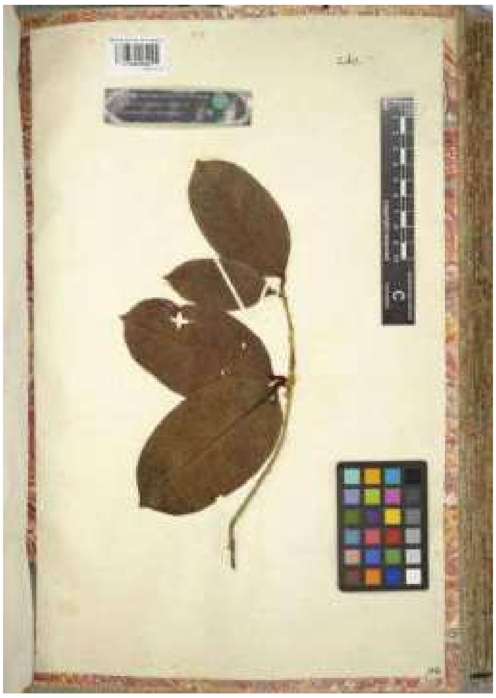

Marcgrave's herbarium: 94

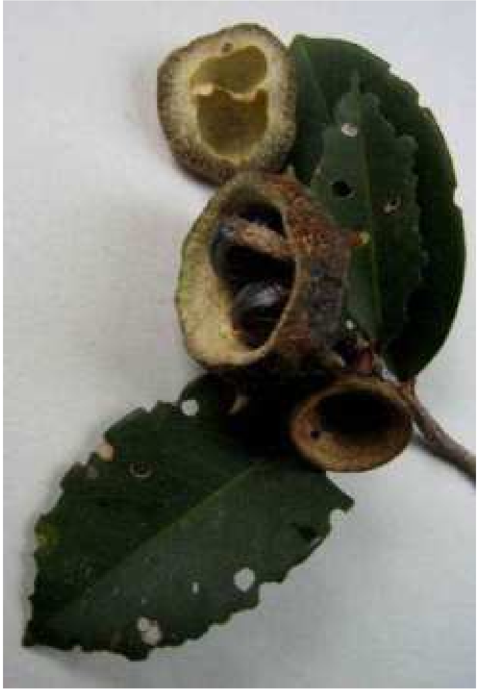

Dehiscent fruit and seeds. "*E. ovata*" by Alex Popovkin, Bahia, Brazil (CC BY-NC-SA 2.0)

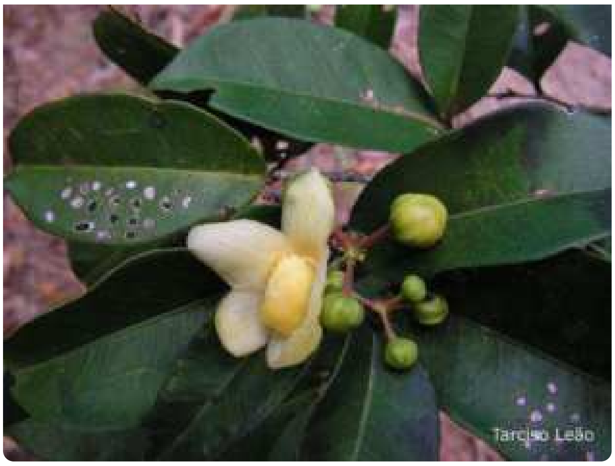

Flower and flower buds. "*E. ovata*, embiriba" by Tarciso Leão (CC BY 2.0)

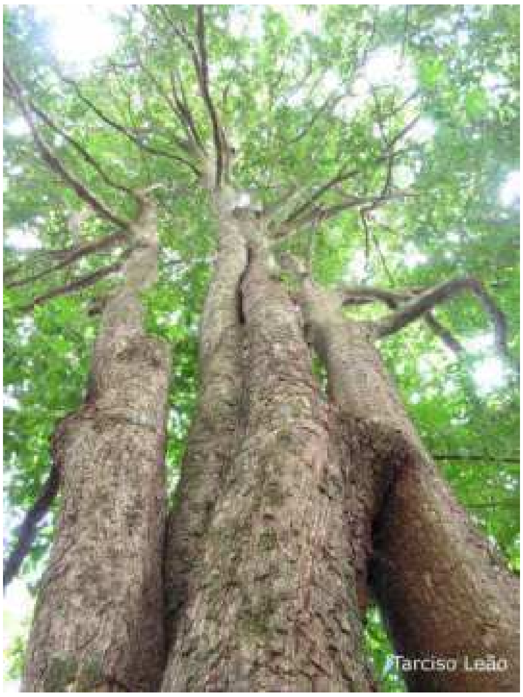

Habit. "*E. ovata*, embiriba" by Tarciso Leão (CC BY 2.0)

Historiae Rerum Naturalium Brasiliae Marcgrave, 1648 Page number 137a

Vernacular name(s)

Species Myroxylon balsamum (L.) Harms

Family Fabaceae

Notes

We did not find any correspondence between this woodcut and the contemporary or older sources.

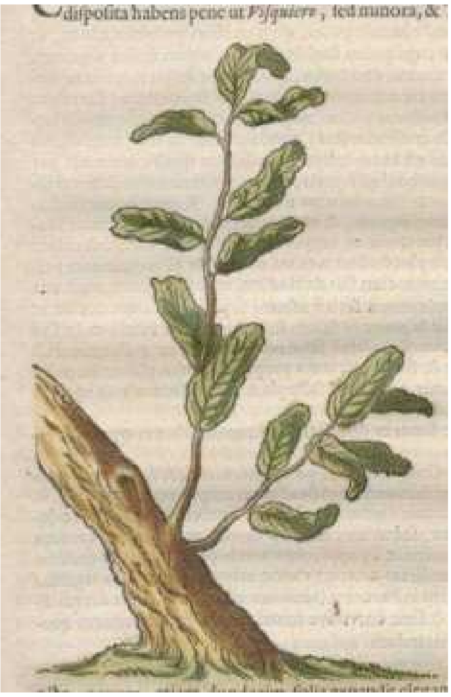

Historiae Plantarum – Arboribus: 137a

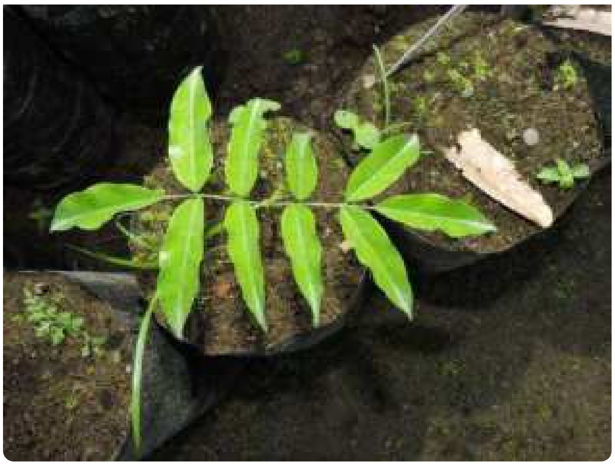

"*M. balsamum*" by Reinaldo Aguilar (CC BY-NC-SA 2.0)

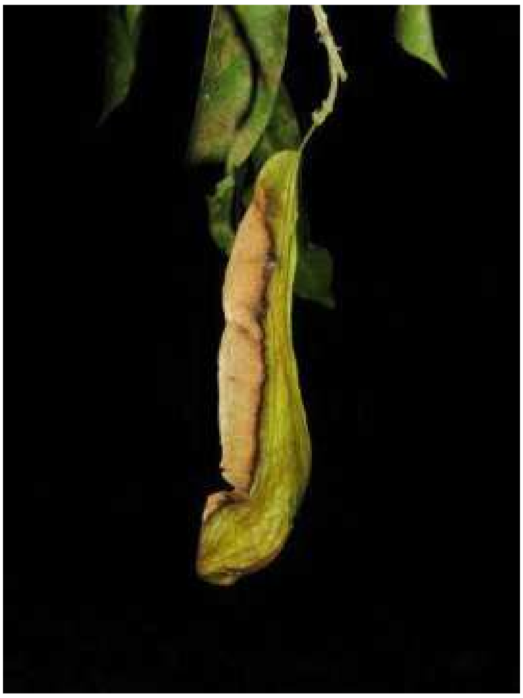

"*M. balsamum*" by Reinaldo Aguilar (CC BY-NC-SA 2.0)

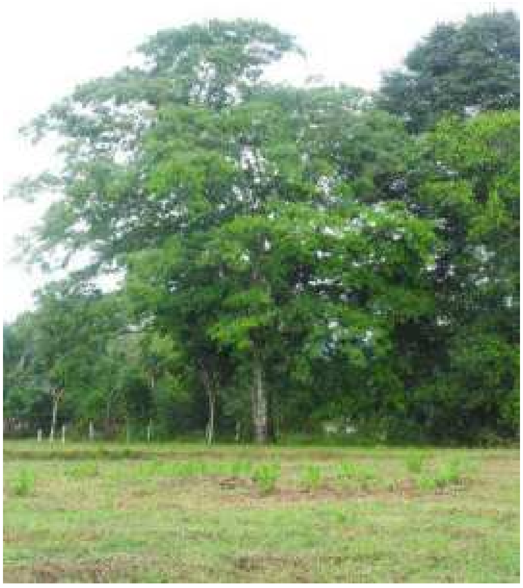

"*M. balsamum* *Sandalo*" by Reinaldo Aguilar (CC BY-NC-SA 2.0)

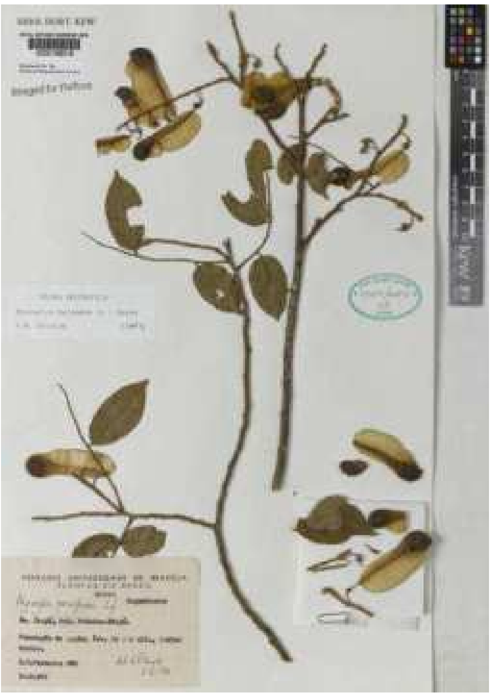

Specimen of *M. balsamum* from Kew's Herbarium - K000188316. Retrieved from Plants of the World Online

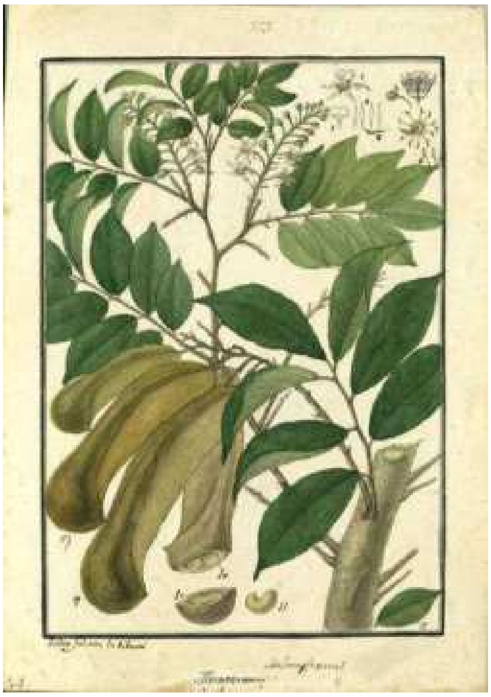

Drawings of the Royal Botanical Expedition to the Viceroyalty of Peru by Ruiz, H., Pavón, J. (1777: 373). Real Jardín Botánico, Madrid, Spain

Historiae Rerum Naturalium Brasiliae Marcgrave, 1648 Page number 137b

Vernacular name(s) Pacoeira. Quibuaaquitiba. Quitiba (fruit). Pacobete. Pacoba

Species Musa × paradisiaca L.

Family Musaceae

Notes

The woodcut does not look like the watercolors or any of the paintings by Eckhout or Post's drawings in Barlaeus' (1647) accounts. The banana bunch is wrongly hanging from the middle of the stem, instead of emerging out of the middle part (Pickel 2008: 62). This woodcut bears resemblance to the woodcut published by de l' Obel (1591: 236) when he described the *Musa* by Serapion the Younger (medieval Arabic botanist-physician). Johan Gerard also used the HNB woodcut of the banana in his *Herball* or General History of Plants (1633: 1515).

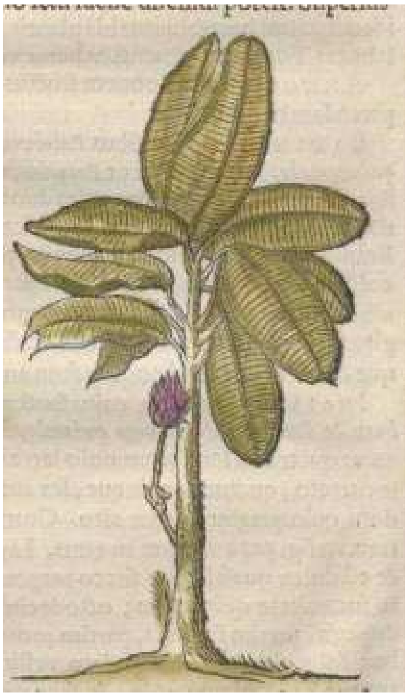

Historiae Plantarum – Arboribus: 137b

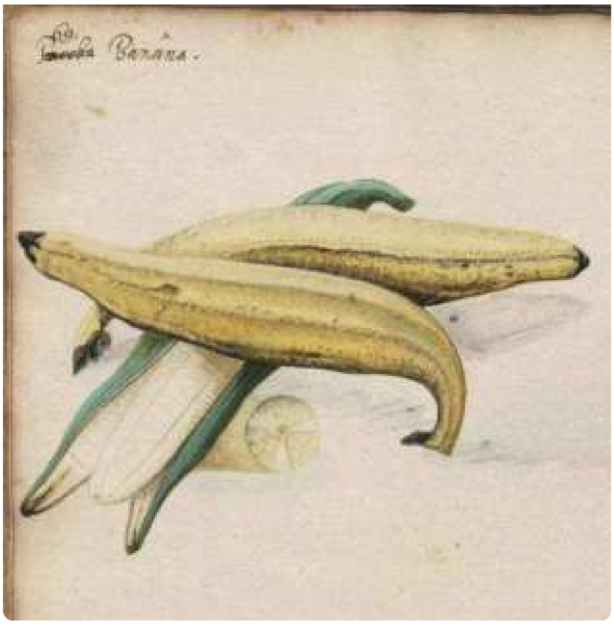

Libri Principis: 89 [69]

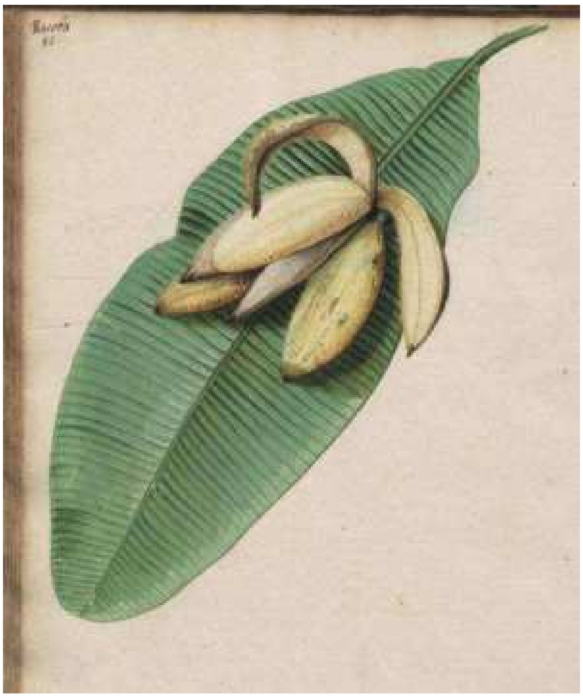

Libri Principis: 101 [81]

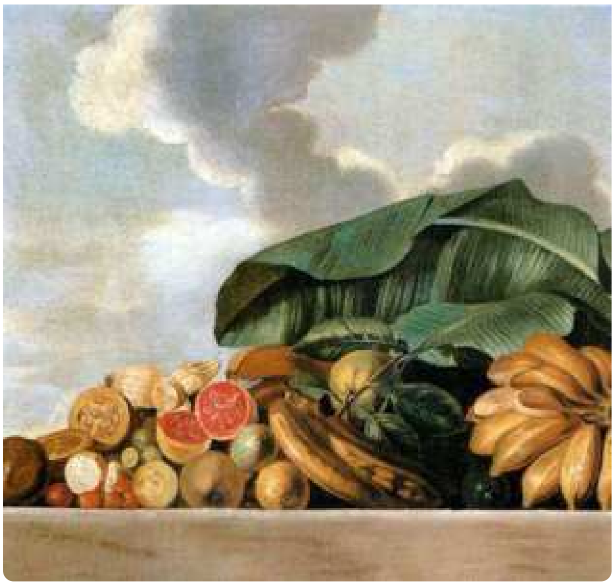

Bananas bunches in Ekchout's still-life with bananas and other fruits, ca. 1640. Copenhagen, National Museum of Denmark

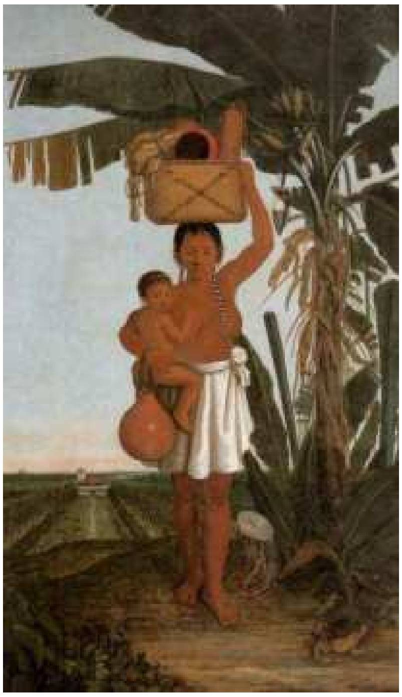

M. paradisiaca plant in Eckhout's portrait "Tupi woman holding a child", ca. 1641. Copenhagen, National Museum of Denmark

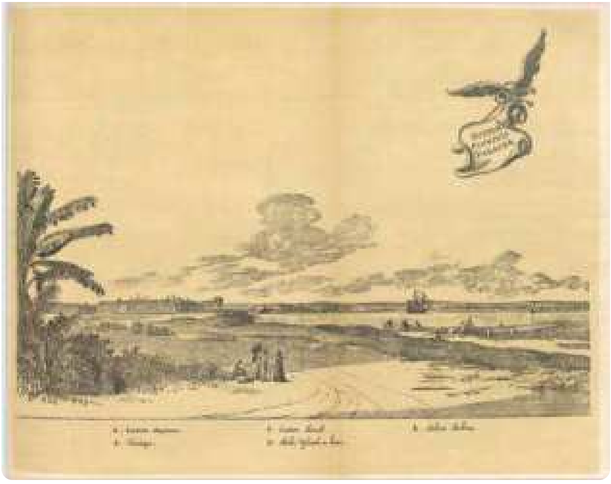

Banana plant (left side) in *Rerum per octennium in Brasilia et alibi* by Caspar Barlaeus (1647: 158-159), designed made by Post

Historiae Rerum Naturalium Brasiliae | Marcgrave, 1648 | Page number 293a

Vernacular name(s) Ubapitanga

Species Eugenia uniflora L.

Family Myrtaceae

Notes

The woodcut looks moderately similar to the illustration in the *Theatrum*, but it does not bear resemblance to the herbarium specimen. There is a proof-woodcut in De Laet's manuscript that corresponds to this woodcut. Marcgrave described *E. uniflora* in p. 116 and documented it as *Ibipitanga*. The woodcut here, under the name *Ubipitanga*, represents *E. uniflora* as well, but De Laet placed it at the end of Marcgrave's section of the HNB, without a description.

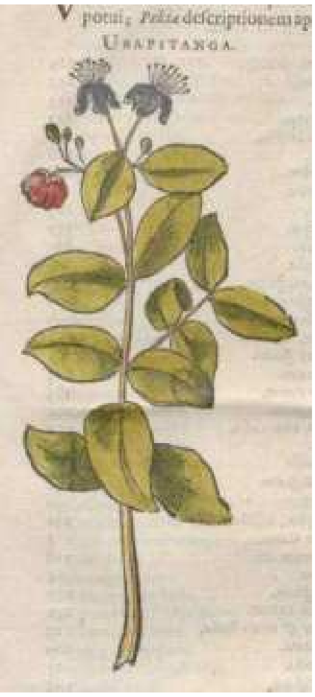

Appendix ad Libros de Plantis: 293a

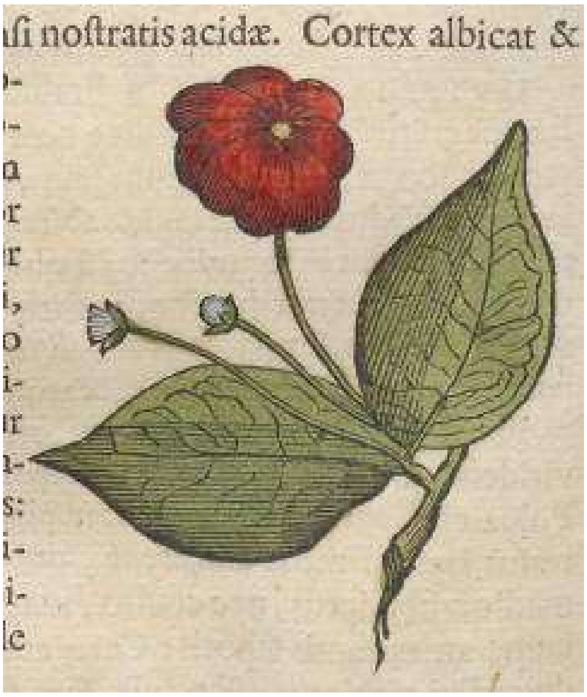

The same species depicted differently in the HNB (Marcgrave 1648: 116)

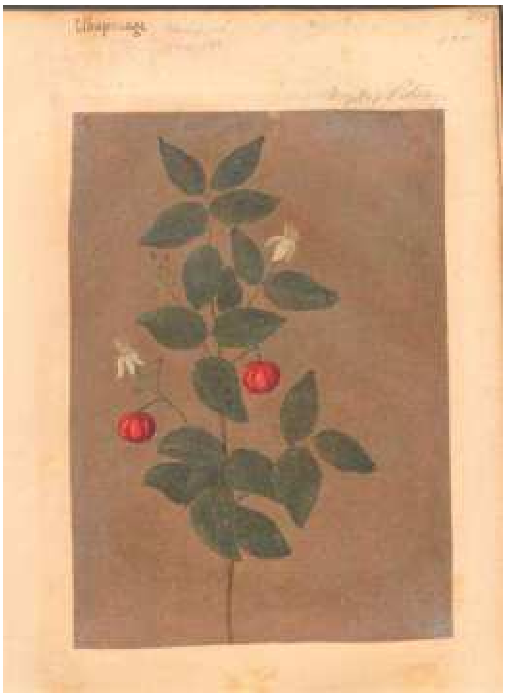

Theatrum Rerum Naturalium: 339

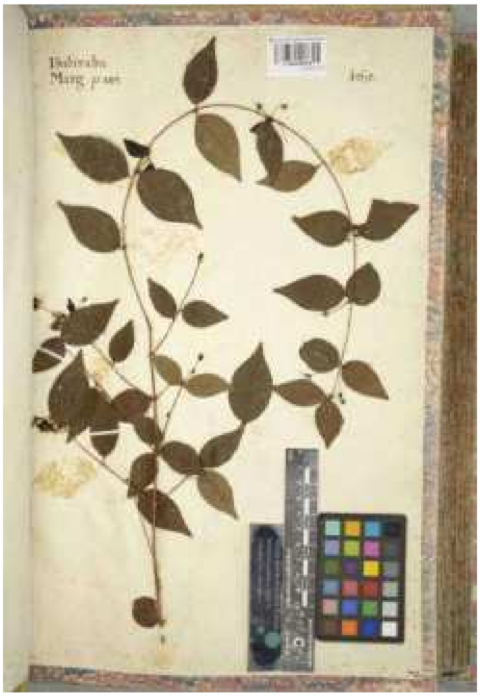

Marcgrave's herbarium: 70

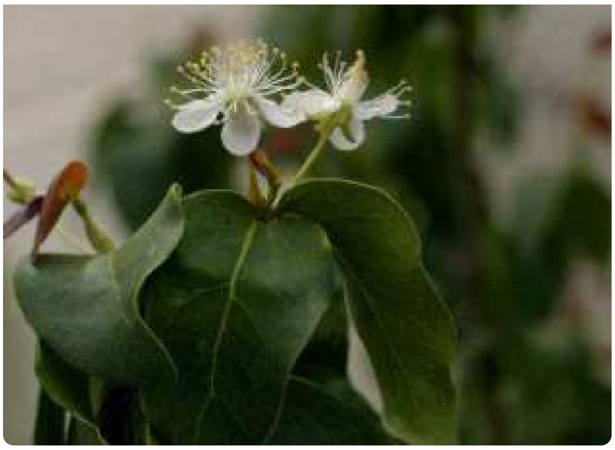

"*E. uniflora* 070307-3586" by Tony Rodd (CC BY-NC-SA 2.0)

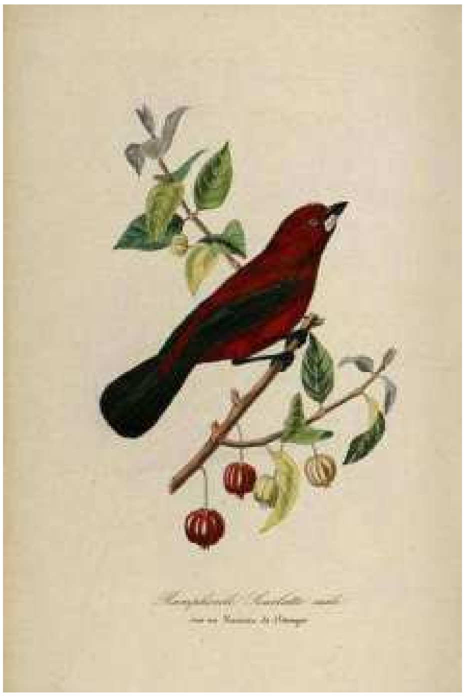

"Brazilian tanager male (*Ramphocelus bresilius*) perched on the branch of a Surinam cherry (*E. uniflora*)." by Swallowtail Garden Seeds

Historiae Rerum Naturalium Brasiliae	Marcgrave, 1648	Page number	293b

Vernacular name(s)	
Species	Jacaranda brasiliana (Lam.) Pers.
Family	Bignoniaceae
Notes	

We did not find any correspondence between this woodcut and the contemporary or older sources.

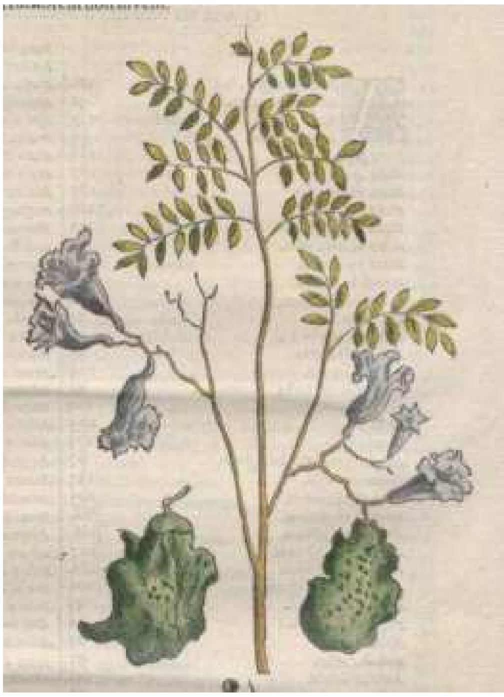

Appendix ad Libros de Plantis: 293b

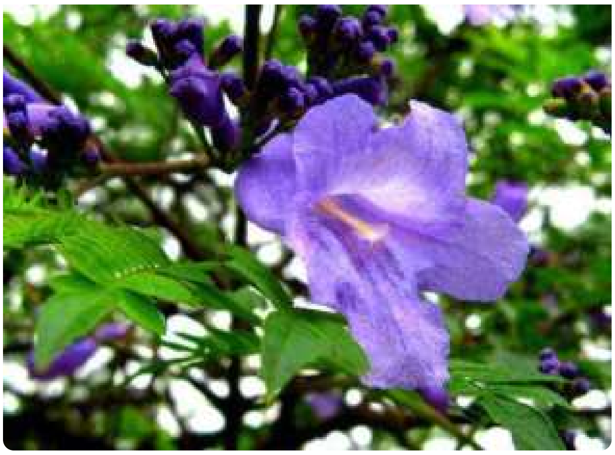

"Jacarandá-boca-de-sapo (*J. brasiliana*) Ceret Sao Paulo. A Brazilian native tree." by mauro halpern (CC BY 2.0)

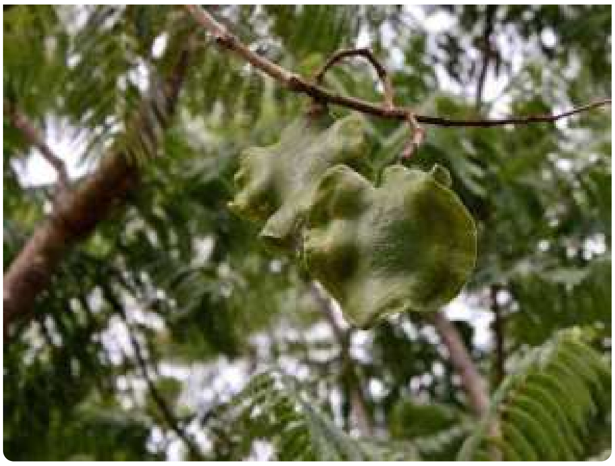

Fruits. "*J. brasiliana*" by Mauricio Mercadante (CC BY-NC-SA 2.0)

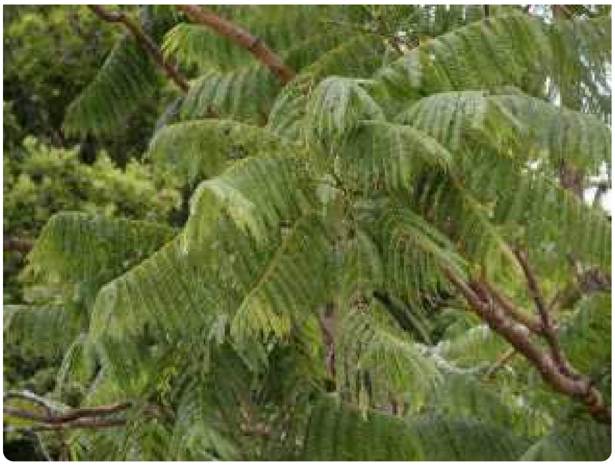

Leaves. "*J. brasiliana*" by Mauricio Mercadante (CC BY-NC-SA 2.0)

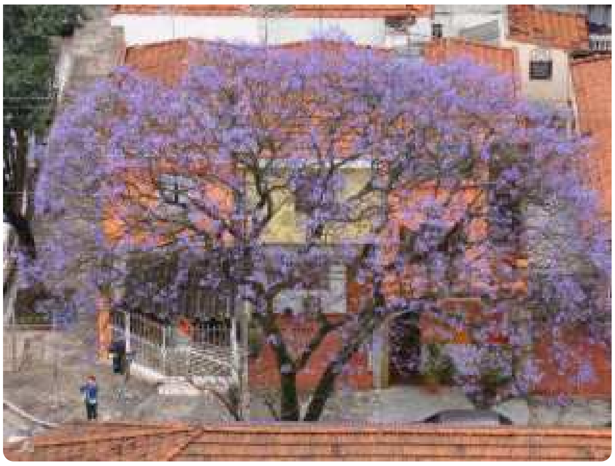

Habit. "Jacarandá-boca-de-sapo (*J. brasiliana* / mimosifolia). from my window office" by mauro halpern (CC BY 2.0)

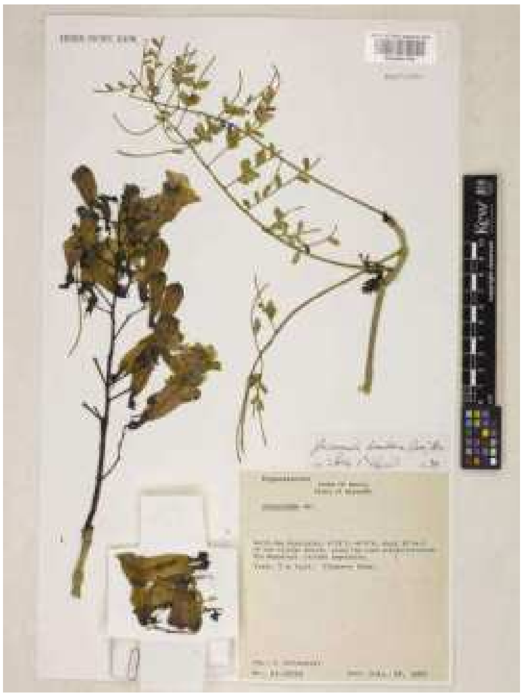

Specimen of *J. brasiliana* from Kew's Herbarium - K000991765. Retrieved from Plants of the World Online

Historiae Rerum Naturalium Brasiliae	Marcgrave, 1648	Page number	293c

Vernacular name(s)	Pekia
Species	Macoubea guianensis Aubl.
Family	Apocynaceae
Notes	

The woodcut differs from the *Theatrum* image. The oil painting illustrates the seeds in a fruit cut in half, as described by Piso (1658: 141).

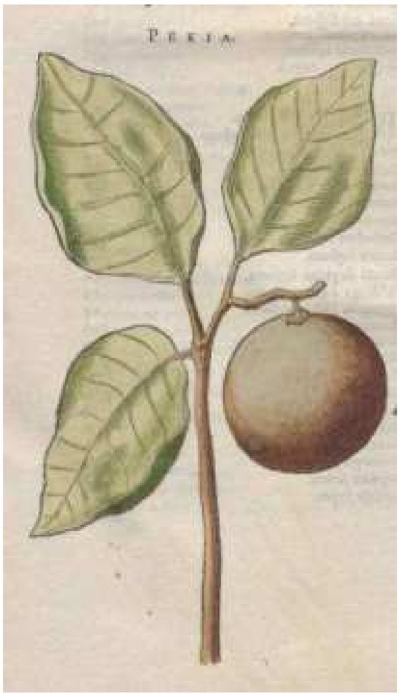

Appendix ad Libros de Plantis: 293c

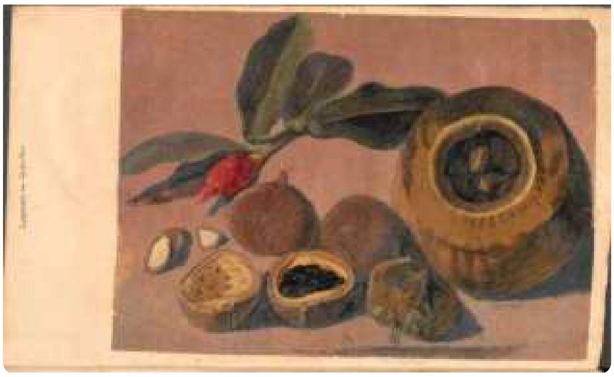

M. guianensis on the left bottom corner in the *Theatrum Rerum Naturalium*: 37

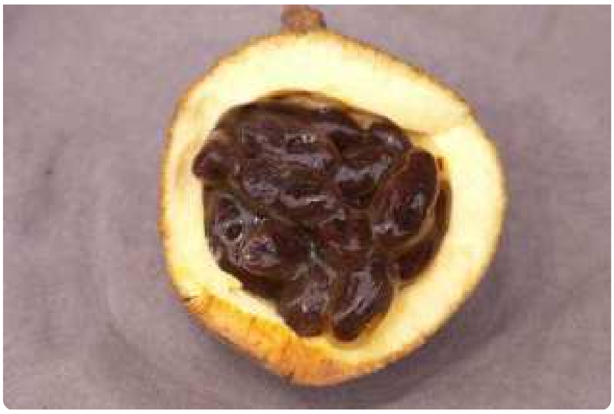

Fruit and seeds. "*M. guianensis*-2" by cetp (CC BY-NC 2.0)

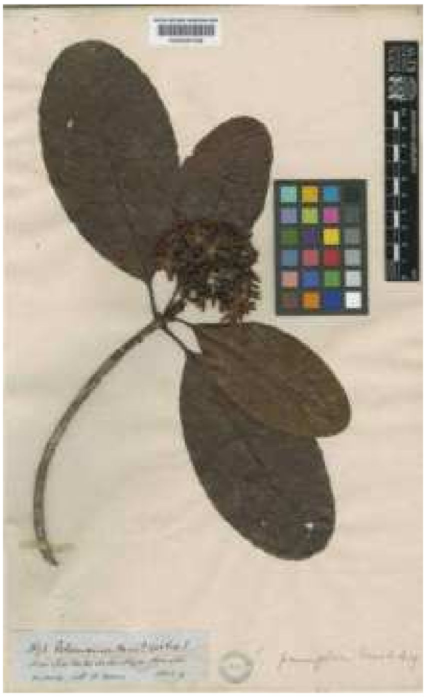

Specimen of *M. guianensis* from Kew's Herbarium - K000587539. Retrieved from Plants of the World Online

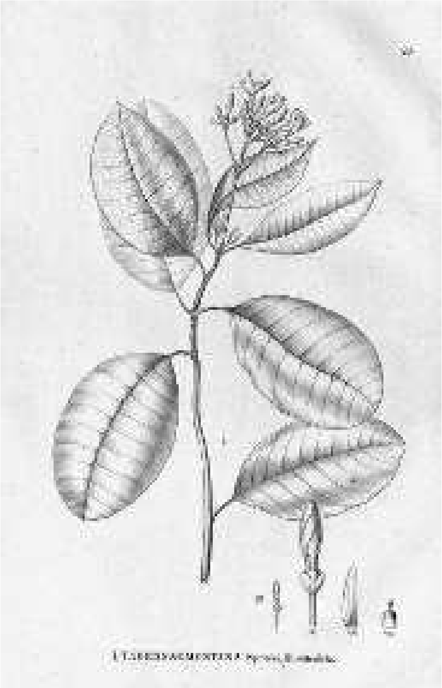

Engraving of *M. guianensis* in Martius, C.F.P. von, Eichler, A.G., Urban, I., *Flora Brasiliensis* (1860-1868) Vol. 6(1): 27

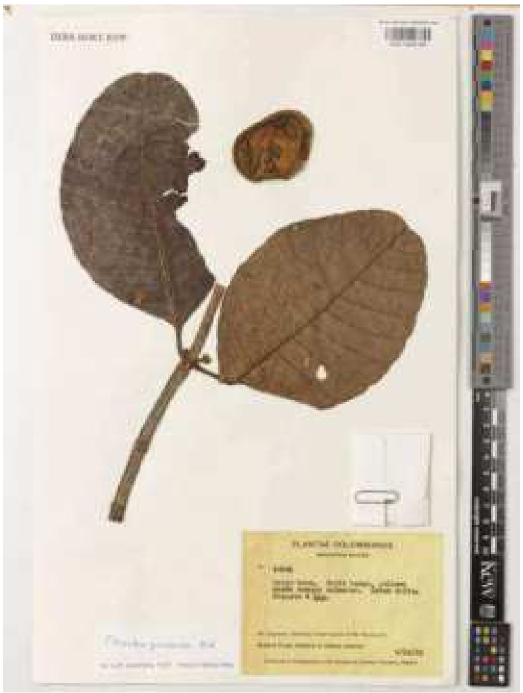

Specimen of *M. guianensis* from Kew's Herbarium - K001489168. Retrieved from Plants of the World Online

Historiae Rerum Naturalium Brasiliae Marcgrave, 1648 Page number 293d

Vernacular name(s)

Species Manihot carthaginensis subsp. glaziovii (Müll.Arg.) Allem

Family Euphorbiaceae

Notes

The woodcut is very similar to the *Theatrum* image (non-reversed). This woodcut is placed at the end of the HNB with a very brief mention by Marcgrave. In Piso (1648: 55), this is known as a wild cassava plant with a more elaborate description, and the name *Cuguaçucuremia*.

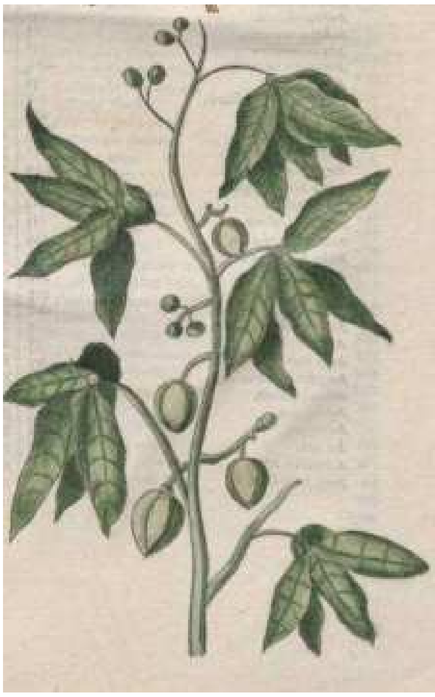

Appendix ad Libros de Plantis: 293d

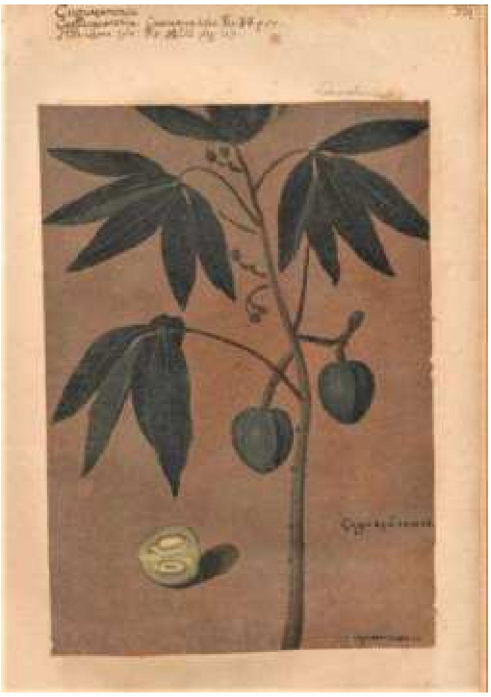

Theatrum Rerum Naturalium: 331

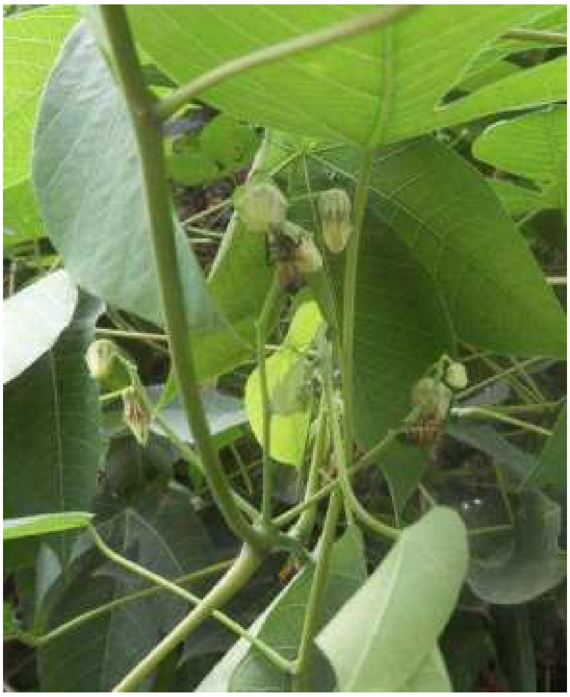

"*M. carthaginensis* subsp. *glaziovii* (EUPHORBIACEAE)" by Scamperdale (CC BY-NC 2.0)

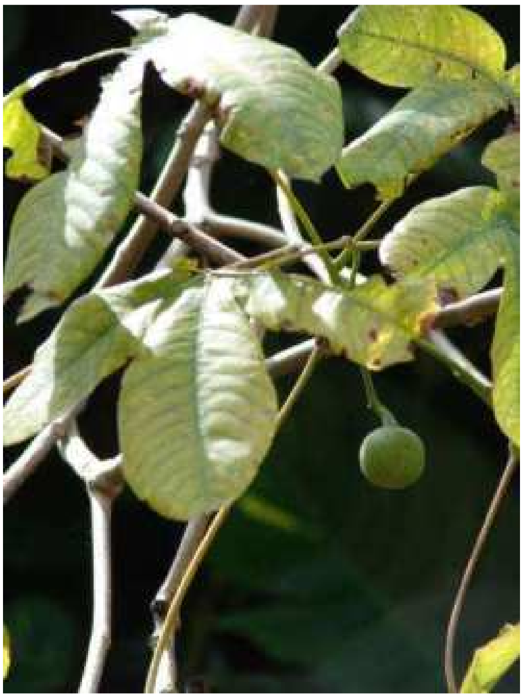

"*M. carthaginensis* subsp. *glaziovii* leaves and fruit-Hana-Maui" by Forest and Kim Starr (CC BY 3.0)

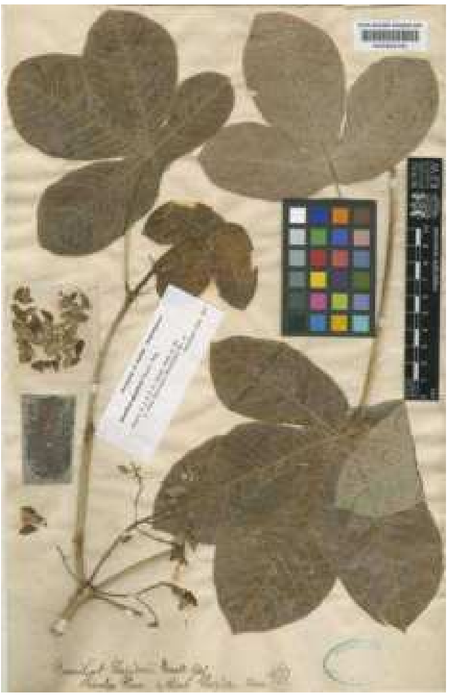

Specimen of *M. carthaginensis* subsp. *glaziovii* from Kew's Herbarium - K000600765. etrieved from Plants of the World Online

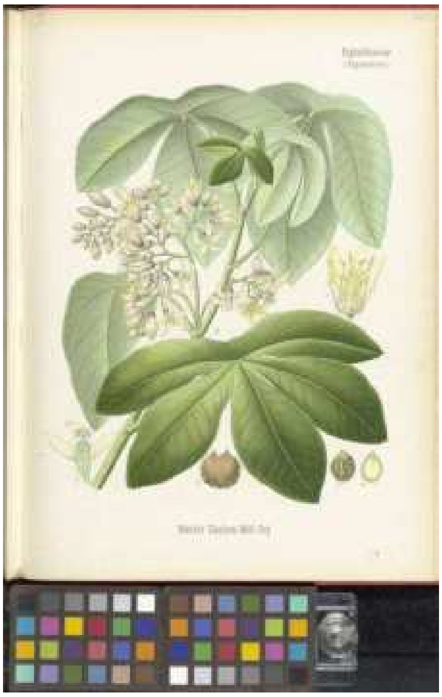

Manihot glaziovii [syn. of *M. carthaginensis* subsp. *glaziovii*] from Köhler's Medizinal-Pflanzen, (1887: Vol. 3)

References

Andrade-Lima, D., Maule, A. F., Pedersen, T. M., & Rahn, K. (1977). Marcgrave's Brazilian Herbarium, collected 1638–44. *Botanisk Tidsskrift*, *71*, 121–160.

Barlaeus, C. (1647). *Rerum per octennium in Brasilia et alibi nuper gestarum sub præfectura illustrissimi Comitis I. Mauritii, Nassoviæ, &c. comitis, nunc Vesaliæ gubernatoris & equitatus fderatorum Belgii ordd. sub Auriaco ductoris, historia.* Ex typographeio Ioannis Blaeu. https://archive.org/details/casparisbarlirer01baer/page/9/mode/thumb

Beschorner, H. (1904). Die Hoflossnitz bei Dresden, Dresden. *Geschichtsbliitter*, *13*(1), 209–226.

Breyne, J. (1678). *Exoticarum aliarumque minus cognitarum plantarum centuria prima, cum figuris aeneis summo studio elaboratis*. Gedani : Typis, sumptibus & in aedibus autoris (imprimebat David-Fridericus Rhetius). https://bibdigital.rjb.csic.es/idurl/1/10814

Breyne, J. P. (1739). *Prodromi fasciculi rariorum plantarum primus et secundus, quondam separatim, nunc nova hac editiones multum desiderata coniunctim editi, notulisque illustrati*. Gedani : sumptibus editoris, typis Thom. Joh. Schreiberi. https://bibdigital.rjb.csic.es/idurl/1/10795

Buvelot, Q. . D. T., Martins, E. D. V., Egmond, F., & Mason, P. (2004). *Albert Eckhout: A Dutch artist in Brazil*. Mauritshuis.

Clusius, C. (1605). *Exoticorvm libri decem :quibus animalium, plantarum, aromatum, aliorum que peregrinorum fructuum historiae describuntur /item Petri Bellonii observationes ; eodem Carolo Clusio interprete ; series totius operis post praefationem indicabitur.* Plantin. https://doi.org/10.5962/bhl.title.7108

Clusius, C. (1611). *Cvrae posteriores, sev Plurimarum non antè cognitarum, aut*

descriptarum stirpium, peregrinorum'que aliquot animalium novae descriptiones: Quibus & omnia ipsius Opera, aliáque ab eo versa augentur, aut illustrantur. Plantin. https://bibdigital.rjb.csic.es/records/item/13494-redirection

De Laet, J. (1633). *Americae utriusque Descriptio Novus orbis seu Descriptionis Indiae Occidentalis*. Elzevier. https://archive.org/details/bub_gb_xLF8OiAonjIC/page/n9/mode/1up

De Laet, J. (1640). *L'histoire du nouveau monde ou Description des Indes Occidentales: contenant dix-huict liures*. Elzevier. https://archive.org/details/lhistoiredunouve00laet/page/n3/mode/2up

Donkin, R. A. (1977). Spanish Red: An Ethnogeographical Study of Cochineal and the Opuntia Cactus. *Transactions of the American Philosophical Society*, *67*(5), 1–84. https://doi.org/10.2307/1006195

Frazão, A., & Lohmann, L. G. (2019). An updated synopsis of Tanaecium (Bignonieae, Bignoniaceae). *PhytoKeys*, *132*, 31–52. https://doi.org/10.3897/phytokeys.132.37538

Gerard, J., & Johnson, T. (1633). *The Herball, or, Generall Historie of Plantes* . Adam Islip, Joice Norton, and Richard Whitakers. https://archive.org/details/gri_33125012606592

Joost, W. (1983). *Die wundersamen Reisen des Caspar Schmalkalden nach West- und Ostindien 1642-1652* (W. Joost (ed.)). Brockhaus Verlag.

L'Obel, M. de. (1591). *Icones stirpium, seu, Plantarum tam exoticarum, quam indigenarum :in gratiam rei herbariae studiosorum in duas partes digestae : cum septem linguarum indicibus, ad diuersarum nationum vsum*. Plantin. https://doi.org/10.5962/bhl.title.9308

Marcgrave, G. (1648). Historia Rerum Naturalium Brasiliae. In *Historia Naturalis*

Brasiliae (pp. 50–293). Apud Franciscum Hackium [Leiden], Apud Lud. Elzevirium [Amsterdam]. http://hdl.handle.net/1887.1/item:1535938

Marcgrave, G. (1942). *História natural do Brasil [1648]* (J. P. de Magalhães (ed.)). Imprensa Oficial do Estado.

Monardes, N. (1580). *Primera y segvnda y tercera partes dela Historia Medicinal: delas cosas que se traen de nuestras Indias Occidentales, que siruen en Medicina*. En Sevilla : En casa de Fernando Diaz. https://bibdigital.rjb.csic.es/idurl/1/13636

Monteiro, C. (2019). *Colonial Representations of Brazil and their Current Display in Western Museums: The Mauritshuis Case and the Dutch Gaze*. Leiden University.

Norton, M. (2006). Tasting Empire: Chocolate and the European Internalization of Mesoamerican Aesthetics. *The American Historical Review*, *111*(3), 660–691. https://doi.org/10.1086/ahr.111.3.660

Pickel, B. J. (2008). *Flora do Nordeste do Brasil segundo Piso e Marcgrave no século XVII* (A. V. De Almeida (ed.)). EDUFRPE.

Piso, W. (1648). De Medicina Brasiliensi. In *Historia Naturalis Brasiliae* (pp. 50–120). Apud Franciscum Hackium [Leiden], Apud Lud. Elzevirium [Amsterdam]. http://hdl.handle.net/1887.1/item:1535938

Piso, W. (1658). *De India Utriusque Re Naturali et Medica*. Apud Ludovicum et Danielem Elzevirios [Amsterdam].

Piso, W. (1948). *História natural do Brasil [1648]* (A. Correia (ed.)). Imprensa Oficial do Estado.

Schoneveld, C. W. (1996). *Sea-changes: Studies in Three Centuries of Anglo-Dutch Cultural Transmission* (Vol. 8). Brill Rodopi.

Thomsen, T. (1938). *Albert Eckhout: ein niederländischer maler, und sein gönner, Moritz der Brasilianer; ein kulturbild aus dem 17. Jahr-hundert* . Levin og Munksgaard.

Whitehead, P. J. P. (1979). The biography of Georg Marcgraf (1610-1643/4) by his brother Christian, translated by James Petiver. *Society for the Bibliography of Natural History* , *9*(3), 301–314.

Whitehead, P. J. P., & Boeseman, M. (1989). *A portrait of Dutch 17th century Brazil. Animals, plants and people by the artists of Johan Maurits of Nassau* . North-Holland Publishing Company.

Worm, O. (1655). *Museum Wormianum, seu, Historia rerum rariorum : tam naturalium, quam artificialium, tam domesticarum, quam exoticarum, quae Hafniae Danorum in aedibus authoris servantur*. Iohannem Elsevirium. https://archive.org/details/gri_museumwormia00worm/page/n185/mode/2up
