# Supplementary material for: Nature portrayed in images in Dutch Brazil: Tracing the sources of the plant woodcuts in the Historia Naturalis Brasiliae (1648)
Source: PLoS One. 2024 Jul 16;19(7):e0276242. doi: 10.1371/journal.pone.0276242 (PMC11251624; doi:10.1371/journal.pone.0276242)
Supplement: S2 Appendix — This PDF file includes the repertoire of distinctive plant woodcuts included in the IURNM (i.e., those that did not appear in its precedent work, the HNB) and their corresponding images retrieved from older or contemporary sources by cross-referencing their scientific names. This appendix allows us to visualize–among botanical illustrations, herbarium vouchers, plant sketches, and other plant materials–the potential sources that were used to elaborate the woodcuts in the IURNM. (PDF) [file pone.0276242.s002.pdf]

Nature portrayed in images in Dutch Brazil

Appendix (S4)

Sources of the plant woodcuts in the *India Utriusque re Naturali et Medica* (1658) Database

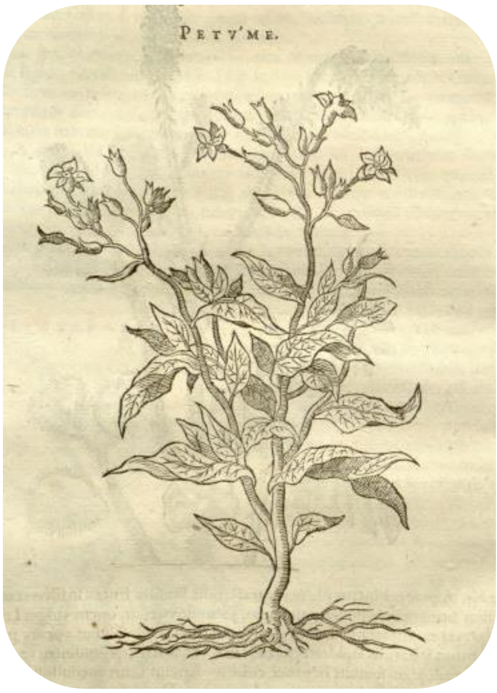
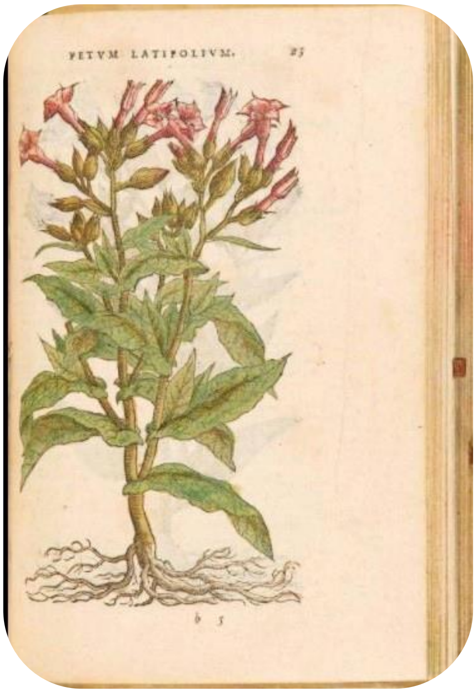

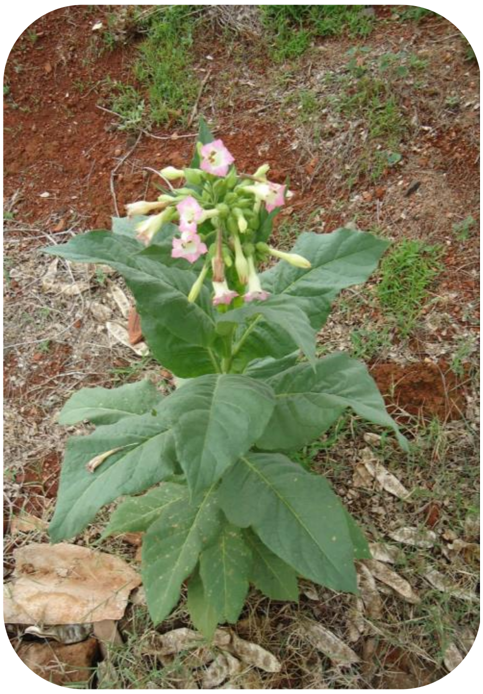

Mireia Alcantara Rodriguez

Nature portrayed in images in Dutch Brazil

Appendix (S4)

Sources of the plant woodcuts in the *India Utriusque re Naturali et Medica* (1658)

Database

Mireia Alcantara Rodriguez

The main research paper to which this appendix belongs is:

Nature portrayed in images in Dutch Brazil: Tracing the sources of the plant woodcuts in the Historia Naturalis Brasiliae (1648) by Mireia Alcantara Rodriguez, Tinde van Andel, and Mariana Françozo.

This appendix is available via the repository of Leiden University, 2022 (https://doi.org/10.17026/dans-xm2-bnhw).

Cover image (from left to right): woodcut of *Nicotiana tabacum* L. in the *India Utriusque re Naturali et Medica* (Piso 1658: 206), a woodcut of the same species in *Simplicium medicamentorum* by Monardes, N. (1579: 25) in his chapter on tobacco, photo of *N. tabacum* in Minas Gerais (Brazil) by Johannes J. Smit (CC BY-NC 2.0).

The research leading to this database received funding from the European Research Council (ERC) Horizon 2020 Research and Innovation Program (Agreement No. 715423), ERC Project BRASILIAE: Indigenous Knowledge in the Making of Science, directed by Dr. M. Françozo at Leiden University.

Preface

I designed this publication to accompany the research article "Nature portrayed in images in Dutch Brazil: Tracing the sources of the plant woodcuts in the Historia Naturalis Brasiliae (1648)" by Mireia Alcantara Rodriguez, Tinde van Andel, and Mariana Françozo. This visual database is directed to everyone attracted to the fascinating world of tropical plants and Renaissance and early modern botanical art, with the main focus on the flora of Dutch Brazil (the northeastern territories of Brazil colonized by the Dutch from 1630 to 1654). This PDF file was built with Filemaker Pro software in a database format, and both files are available (open access) in the repository of Leiden University via: https://doi.org/10.17026/dans-xm2-bnhw.

The main purpose of this file is to present in a visual form the potential sources used to create the plant woodcuts in the India Utriusque re Naturali et Medica (IURNM, 1658). A brief text accompanies every plant entry in which I discuss the various levels of correlation among sources. The IURNM was majorly based on the Historia Naturalis Brasiliae (HNB, 1648), which aimed to portray the natural history of Brazil in the 1640s as perceived by German naturalist George Marcgrave (1610-1643) and Dutch physician Willem Piso (1611-1678) – among others. Piso, discontent with the work made by the editor of the HNB, Johannes de Laet (1581-1649), edited his work to create the IURNM. This file can be read independently but its main function is to complement and depict the data analyzed in the main paper, which is also displayed in an Excel spreadsheet (S3). The main paper aimed to answer what were the sources used to elaborate the plant woodcuts in the IURNM, first, by arranging the woodcut images and their corresponding species found in other visual sources from Dutch Brazil and analyzing their similarities; and second, by searching for the remaining sources in contemporary or older treatises that included engravings

similar to the IURNM woodcuts. By doing so, the various methods of visual-knowledge-making are discussed, including the methods of plant collection and flora representation. This publication presents a catalog of the data on plant woodcuts in the IURNM, including all woodcuts that are not exact copies of those previously used in the HNB. There are 62 database entries and 248 images, which correspond to 59 plant taxa. Related to this research, I created a dataset (S1) and its corresponding PDF file (Appendix S2) to analyze the sources of the plant woodcuts in the HNB. It is recommended to check both visual files (S2 vs. S4) to observe the diversity of visual knowledge-production in both works.

The woodcuts are organized in the same order of appearance as in the IURNM and all the visual sources are arranged by their corresponding species. Each entry can be read independently from the others. The 62 plant woodcuts were retrieved from the section called *Historia Naturalis & Medica*, which includes two chapters on flora in (IV) *De Arboribus, fructibus, & herbis medicis*… (pages 1 to 110 in this file) and (V) *De Noxiis & venenatis, corumque Antidotis* (pages 111 to 124). The database and its entry forms provide information on the page number where the woodcuts are located, the vernacular plant names, species, and botanical family, their presence in the HNB, and notes on the correlation between the woodcuts and contemporary or older plant images that represent the same species. Following, the plant woodcuts and the associated images are displayed. These images were mainly retrieved from the IURNM (Piso 1658), the HNB (Marcgrave 1648; Piso 1648), Marcgrave's herbarium (https://samlinger.snm.ku.dk/en/dry-and-wet-collections/botany/general-herbarium/the-marcgrave-herbarium/), De Laet manuscript (BL Sloane MS 1554), the *Theatrum Rerum Naturalium* (provided as digital images by the Jagiellonian library), the *Miscellanea Cleyeri* (https://jbc.bj.uj.edu.pl/dlibra/doccontent?id=197455)

and the *Libri Principis* (https://jbc.bj.uj.edu.pl/dlibra/publication/193892/edition/183824/content); as well as from treatises digitized and available open access via digital libraries (archive.org, biodiversitylibrary.org). Often, this file includes modern photos of the species portrayed in the woodcuts, mostly retrieved from Creative Commons and Plantillustrations.org.

Historia Naturalis & Medica Piso, 1658 Page number 114

Vernacular name(s) Radix Mandihoca

Species Manihot esculenta Crantz

Family Euphorbiaceae

Presence in the HNB Yes

Marcgrave (1648) 65 (only description)

Piso (1648) 52 (only description)

Notes

This woodcut resembles the cassava plant depicted in Eckhout´s painting of the "Indigenous Tupi". It slightly looks like the manioc plant in the still-life painting by the same author, made after the *Misc. Cleyeri*. Since 1652, Frederick Wilhelm, Elector of Brandenburg, had the drawings that were later bound in the *Misc. Cleyeri*'s collection and the paintings arrived in Copenhagen in 1656 (Buvelot et al. 2004). It is possible that the sketches of this plant used by Ekchout for the above-mentioned works remained in the Dutch Republic and were used by Piso to create this woodcut.

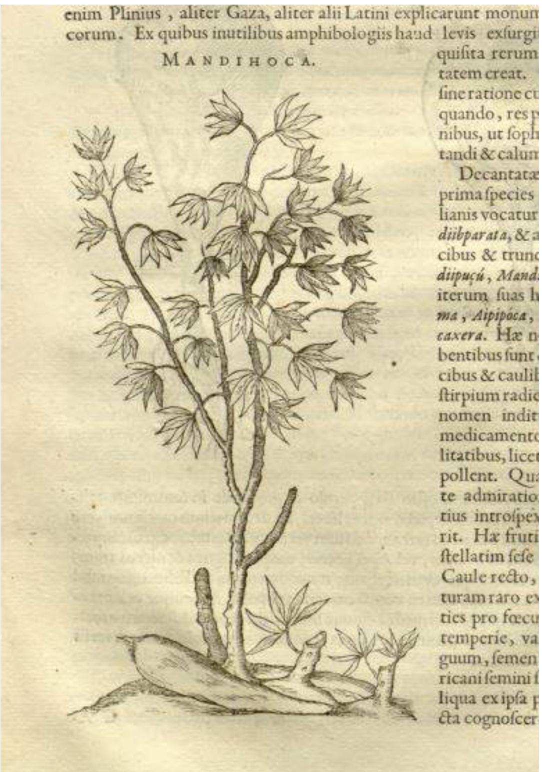

enim Plinius , aliter Gaza, aliter alii Latini explicarunt monum
corum. Ex quibus inutilibus amphibologiis haud levis exſurgi

MANDIHOCA.

quiſita rerum
tatem creat.
ſine ratione cu
quando, res p
nibus, ut ſoph
tandi & calum
Decantatæ
prima ſpecies
lianis vocatur
diibparata, & a
cibus & trunc
diipuçú, Mand
iterum ſuas h
ma, Aipipoca,
caxera. Hæ n
bentibus ſunt
cibus & caulib
ſtirpium radic
nomen indit
medicamento
litatibus, licet
pollent. Qu
te admiratio
tius introſpe
rit. Hæ fruti
ſtellatim ſeſe
Caule recto,
turam raro e
ties pro fœcu
temperie, va
guum, ſemen
ricani ſemini f
liqua ex ipſa p
cta cognoſcer

IV. *De Arboribus, fructibus, & herbis medicis*: 114

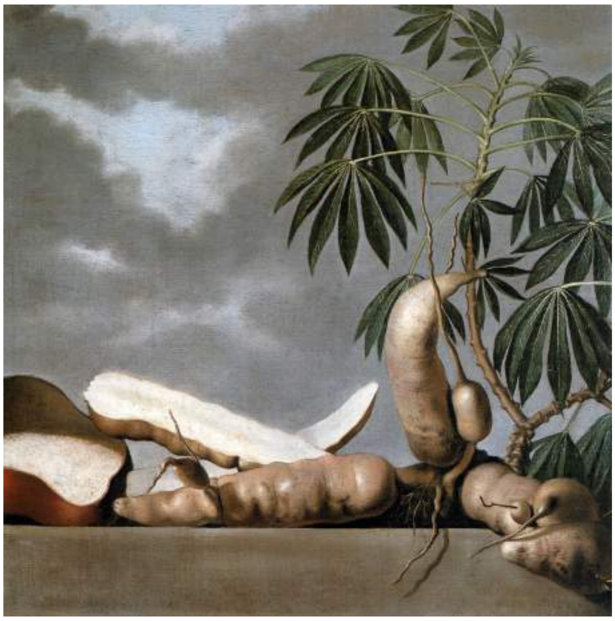

Eckhout still-life with Manioc, c. 1640

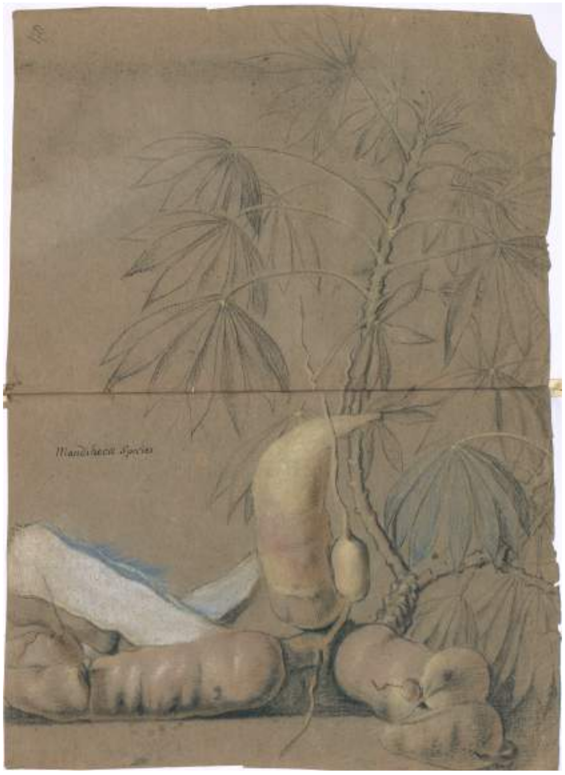

Miscellanea Cleyeri c.1637-44: 69v and 70r

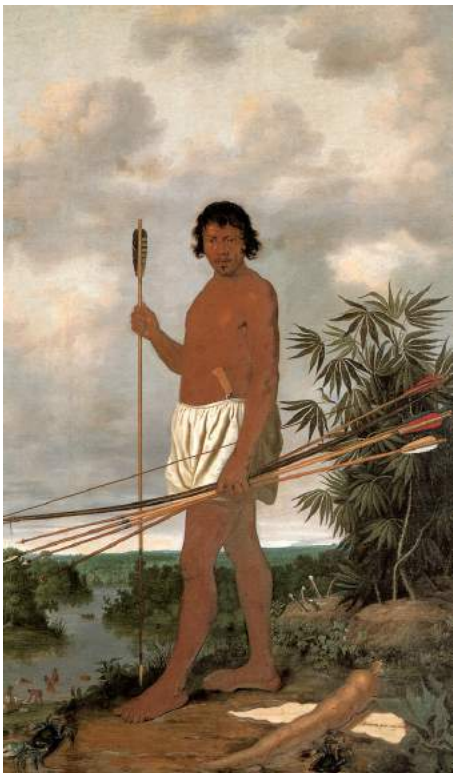

Ekchout portrait of "Indigenous Tupi", 1643

Historia Naturalis & Medica Piso, 1658 Page number 118

Vernacular name(s)	Copaíba
Species	Copaifera officinalis L.
Family	Fabaceae

Presence in the HNB	Yes
Marcgrave (1648)	130b (different woodcut)
Piso (1648)	56 (only description)

Notes

This woodcut was made by mixing three different species: *Clusia nemorosa*, *Tapirira guianensis*, and *Copaifera officinalis* (Pickel 2008: 23). They all share the same vernacular name, hence, Piso combined them and made a composite image. The leaves do not look very much like *T. guianensis*, but they do share similar vernacular names. Hence, Piso likely followed that criteria, and he thought they were all the same plant because of their similar names.

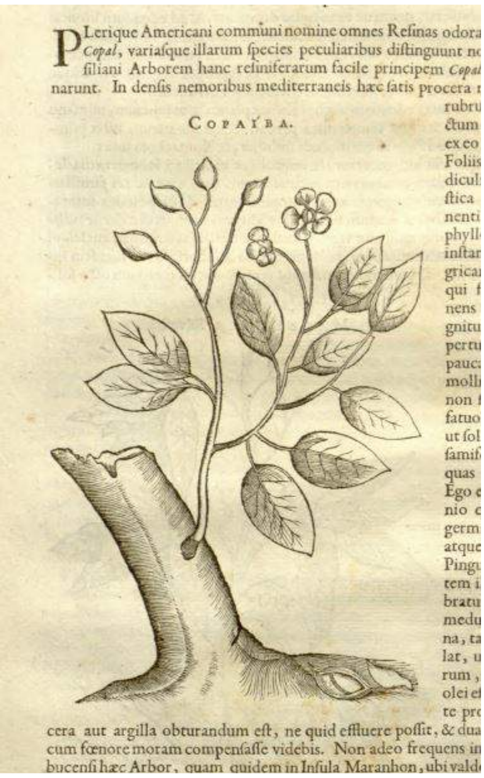

IV. De Arboribus, fructibus, & herbis medicis: 118

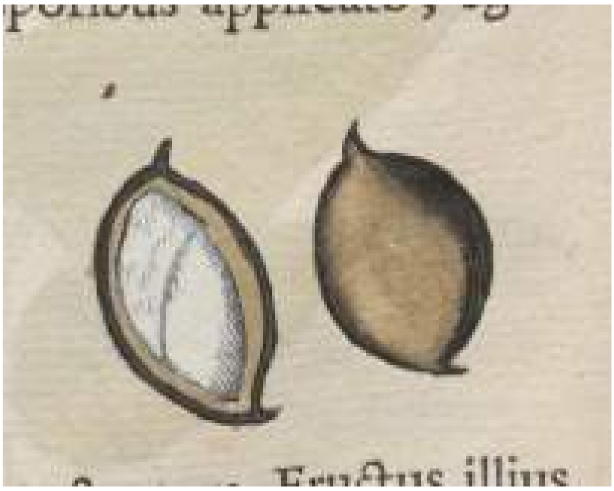

Seeds of Copaiba (*Copaifera officinalis*) in the HNB (Marcgrave 1648: 130b)

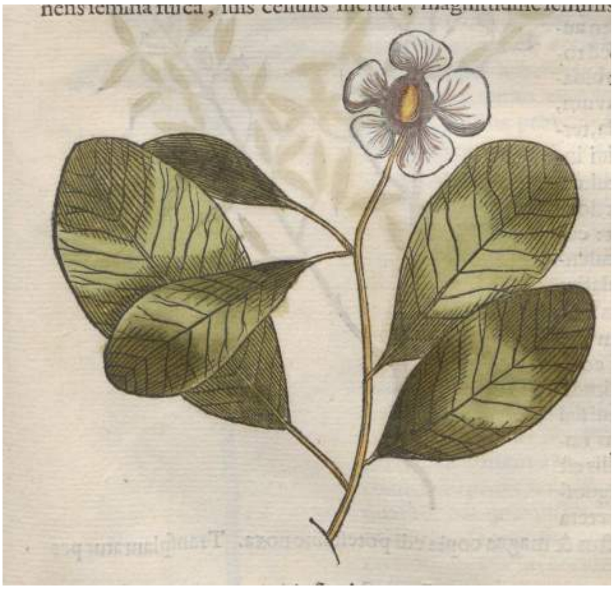

Coapoiba plant (*Clusia nemorosa*) in the HNB (Marcgrave 1648: 131b)

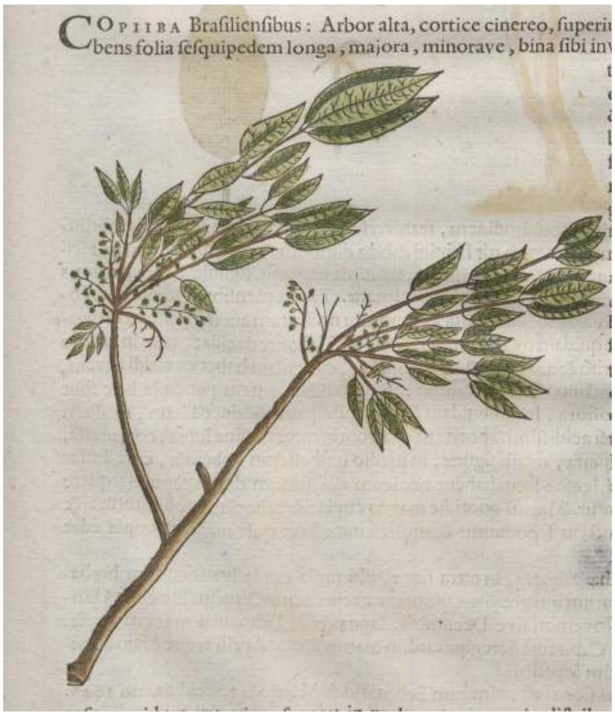

Copiiba plant (*Tapirira guianensis*) in the HNB (Marcgrave 1648: 121b)

Historia Naturalis & Medica Piso, 1658 Page number 120

Vernacular name(s) Acaiaíba

Species Anacardium occidentale L.

Family Anacardiaceae

Presence in the HNB Yes

Marcgrave (1648) 95a (slightly different woodcut)

Piso (1648) 58 (different woodcut)

Notes

This is a modified image made after the woodcut in the HNB (1648). It slightly resembles the cashew tree branches in the portrait by Eckhout of the ¨Mameluka woman¨, which is also depicted in the painting "Dance of the Tapuyas" by the same author.

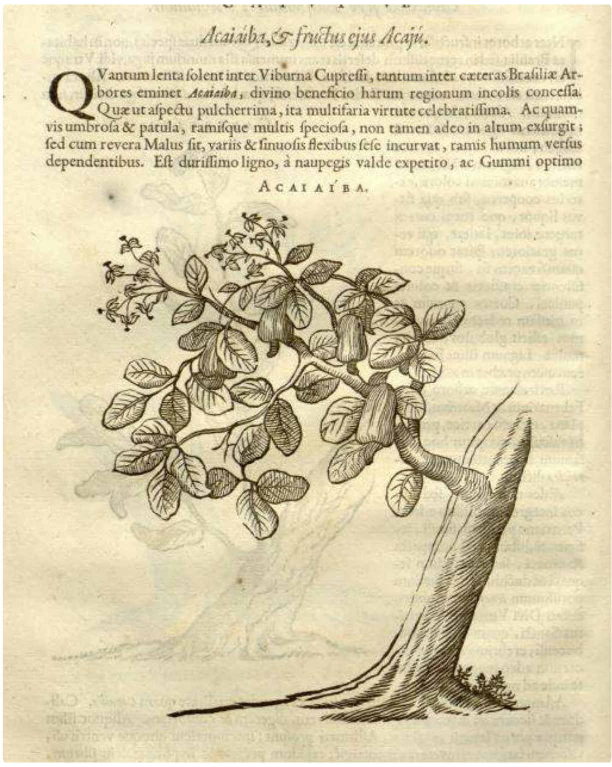

Acaiaiba, & fructus ejus Acajú.

QVantum lenta solent inter Viburna Cupressi, tantum inter cæteras Brasiliæ Arbores eminet *Acaiaiba*, divino beneficio harum regionum incolis concessa. Quæ ut aspectu pulcherrima, ita multifaria virtute celebratissima. Ac quamvis umbrosa & patula, ramisque multis speciosa, non tamen adeo in altum exsurgit; sed cum revera Malus sit, variis & sinuosis flexibus sese incurvat, ramis humum versus dependentibus. Est durissimo ligno, à naupegis valde expetito, ac Gummi optimo

ACAIAÍBA.

IV. De Arboribus, fructibus, & herbis medicis: 120

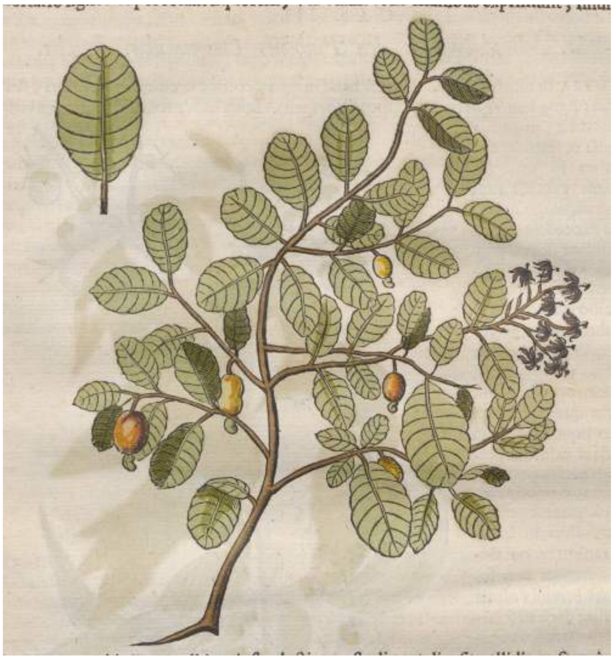

Cashew branch in the HNB (Marcgrave 1648: 95a)

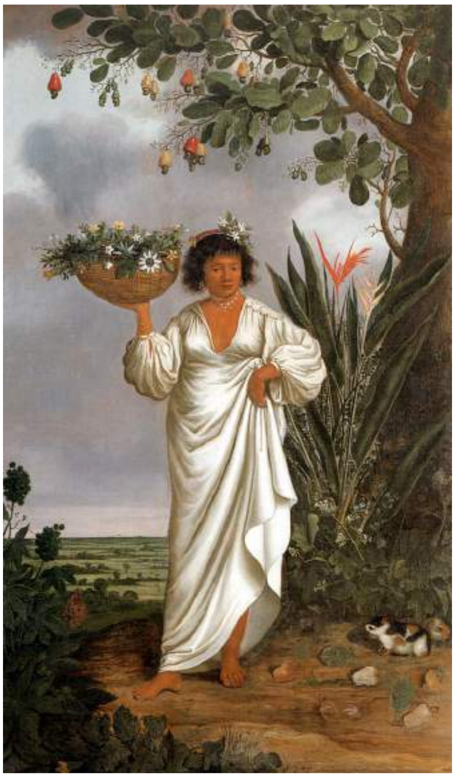

Portrait of "Mameluka woman" and Cashew tree in the back by Eckhout, 1641

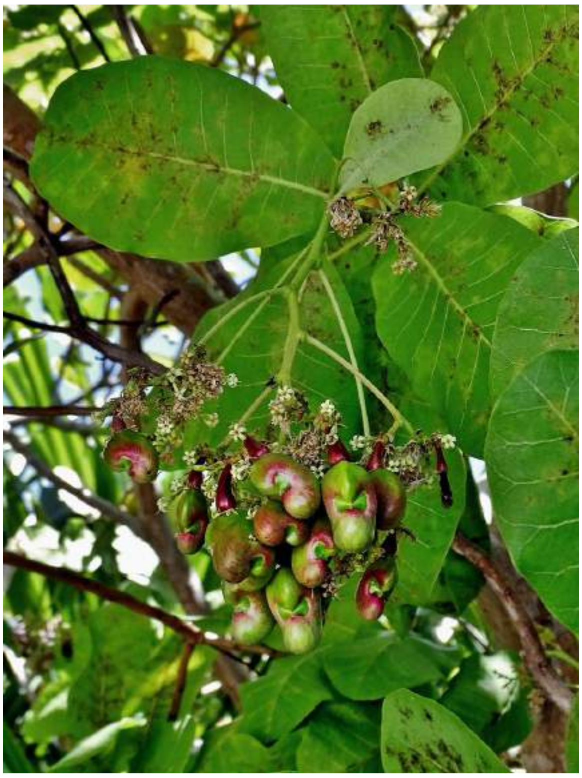

Branch with fruit and flowers. "*Anacardium occidentale*" by Mauricio Mercadante (CC BY-NC-SA 2.0)

Historia Naturalis & Medica Piso, 1658 Page number 123b

Vernacular name(s) Ietaiba. Ietica-cica and Anima (resin)

Species Hymenaea cf. courbaril L.

Family Fabaceae

Presence in the HNB Yes

Marcgrave (1648) 101b (different woodcut)

Piso (1648) 60 (different woodcut)

Notes

This woodcut image is similar to the woodcut for the same, or related, species in the *Exoticorum Libri Decem* by Clusius (1605: 61). An image of this plant seems to appear as well in the chapter on Virginia in the book about the Americas by De Laet (1633: 87, 1640: 96).

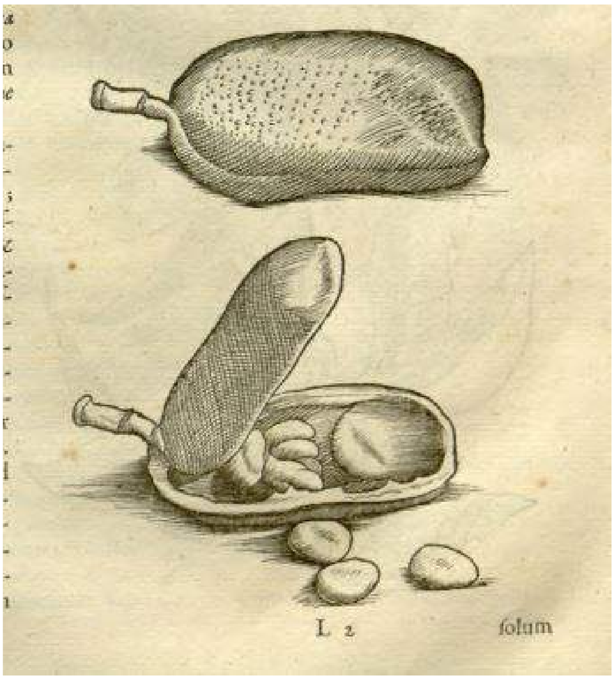

IV. *De Arboribus, fructibus, & herbis medicis*: 123b

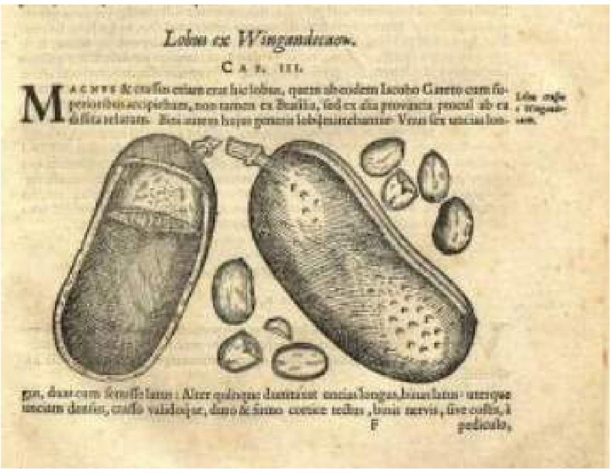

Exoticorum Libri Decem, by Clusius (1605: 61)

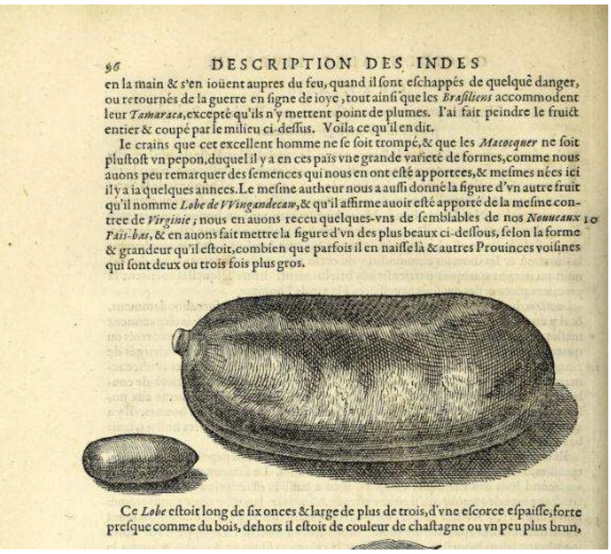

96 DESCRIPTION DES INDES

en la main & s'en ioüent aupres du feu, quand il sont eschappés de quelquê danger, ou retournés de la guerre en signe de ioye, tout ainsi que les *Brasiliens* accommodent leur *Tamaraca*, excepté qu'ils n'y mettent point de plumes. I'ai fait peindre le fruict entier & coupé par le milieu ci-dessus. Voila ce qu'il en dit.

Ie crains que cet excellent homme ne se soit trompé, & que les *Macocquer* ne soit plustost vn pepon, duquel il y a en ces païs vne grande varieté de formes, comme nous auons peu remarquer des semences qui nous en ont esté apportees, & mesmes nées ici il y a ia quelques annees. Le mesme autheur nous a aussi donné la figure d'vn autre fruit qu'il nomme *Lobe de VVingandecaw*, & qu'il affirme auoir esté apporté de la mesme contree de *Virginie*; nous en auons receu quelques-vns de semblables de nos *Nouueaux Païs-bas*, & en auons fait mettre la figure d'vn des plus beaux ci-dessous, selon la forme & grandeur qu'il estoit, combien que parfois il en naisse là & autres Prouinces voisines qui sont deux ou trois fois plus gros.

Ce *Lobe* estoit long de six onces & large de plus de trois, d'vne escorce espaisse, forte presque comme du bois, dehors il estoit de couleur de chastagne ou vn peu plus brun,

L'histoire du nouveau monde ou Description des Indes Occidentales, by De Laet (1640: 96)

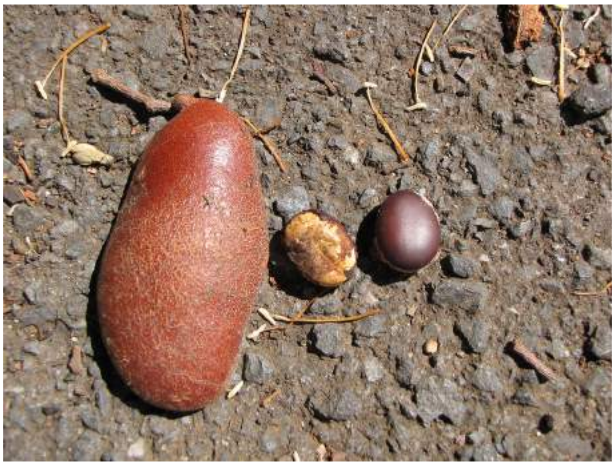

"*Hymenaea_courbaril* pod and seeds-West" by Starr Environmental (CC BY 2.0)

Historia Naturalis & Medica Piso, 1658 Page number 126b

Vernacular name(s) Carnaiba II (Miriti in p. 129)

Species Mauritia flexuosa L.f.

Family Arecaceae

Presence in the HNB No

Marcgrave (1648)

Piso (1648)

Notes

Although identified by Pickel (2008: 44) as *Mauritia flexuosa*, the woodcut resembles more *Copernicia prunifera* (Mill.) H.E.Moore, which is also depicted in p. 126 of the IURNM with a different woodcut, and in the HNB (Marcgrave 1648: 130, Piso 1648: 62). The painting by Eckhout of the "African woman" shows *C. prunifera* next to the woman with her child. Either way, we could not trace the exact source of this woodcut.

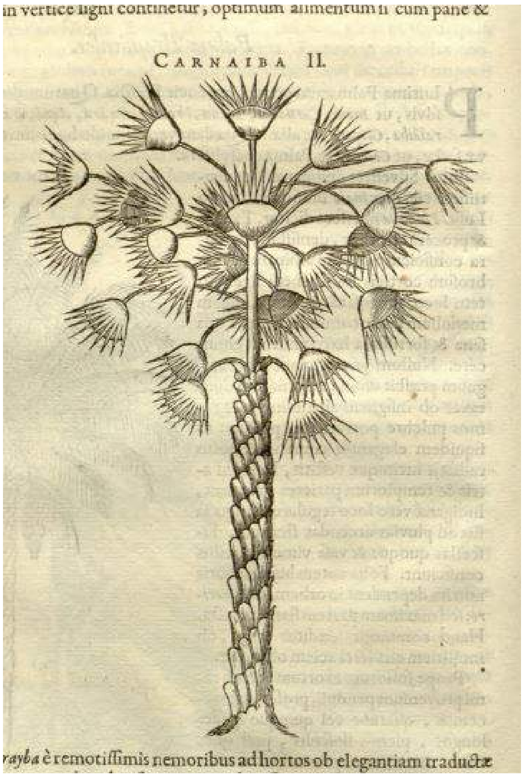

IV. De Arboribus, fructibus, & herbis medicis: 126b

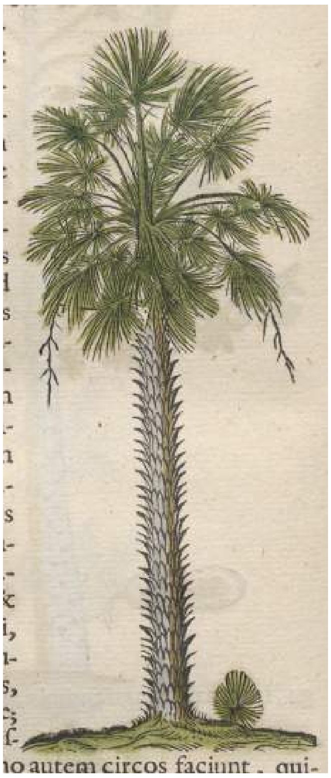

Copernicia prunifera woodcut in the HNB (Marcgrave 1648: 130)

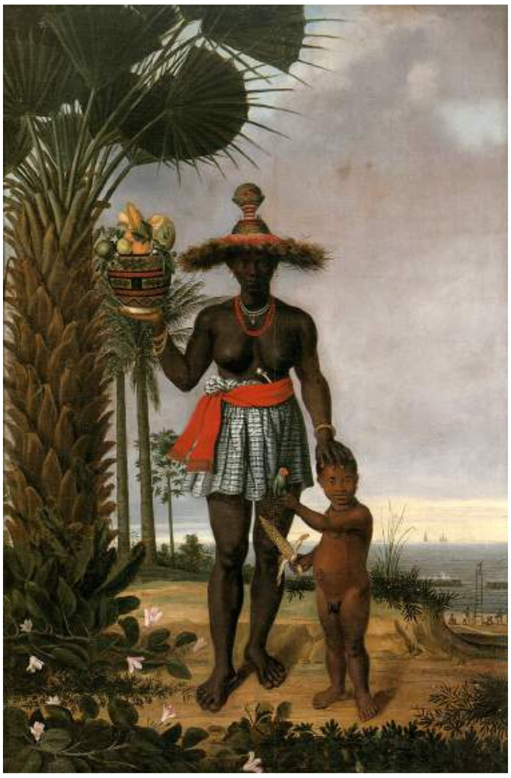

Portrait of "African woman" by Eckhout, 1641

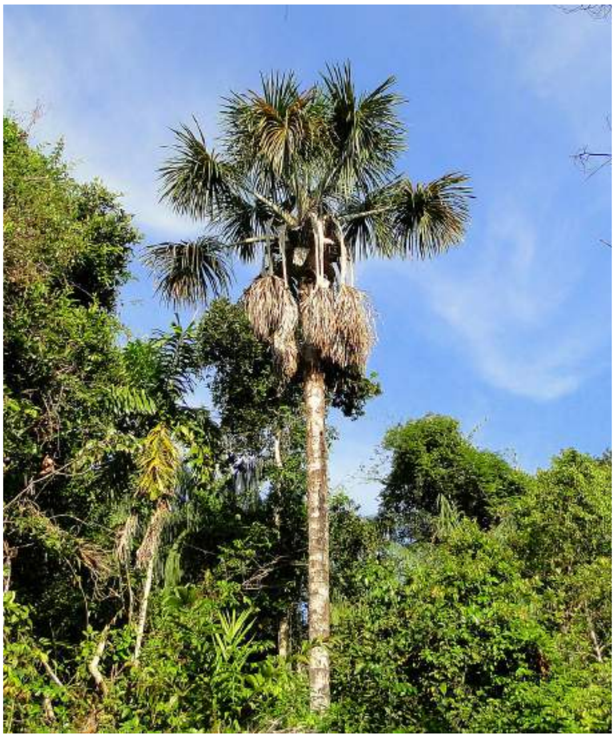

Mauritia flexuosa "Palma Moriche [Moriche Palm]" by barloventomagico (CC BY-NC-ND 2.0)

Historia Naturalis & Medica Piso, 1658 Page number 128

Vernacular name(s) Tucum

Species Astrocaryum vulgare Mart.

Family Arecaceae

Presence in the HNB Yes

Marcgrave (1648)

Piso (1648) 62 (only description)

Notes

The image represented in this woodcut corresponds to the African date palm (*Phoenix dactylifera* L.). Similar Images of this palm were circulating in Renaissance and Modern herbals. Matthioli (1563: 76) depicts a similar one in his *New Kreüterbuch*, in which the inflorescence raceme is illustrated separately in great detail. De l' Obel (1581: Vol. II, 234) and Dodoens (1583: 807) presented a copy (reversed image) of these two woodcuts. Alpini (1592: 11v, 1640: 28) illustrated the date palm in his books on plants from Egypt. He depicted the inflorescence attached to the tree, which is also what Piso did in his woodcut. The leaves in the IURNM are less densely arranged than in the mentioned sources, and more similar to those in Post's drawings.

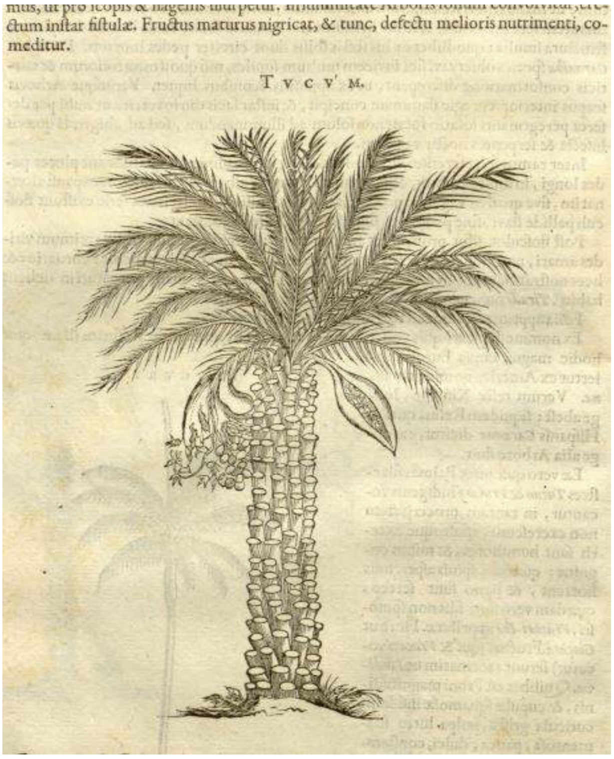

IV. *De Arboribus, fructibus, & herbis medicis*: 128

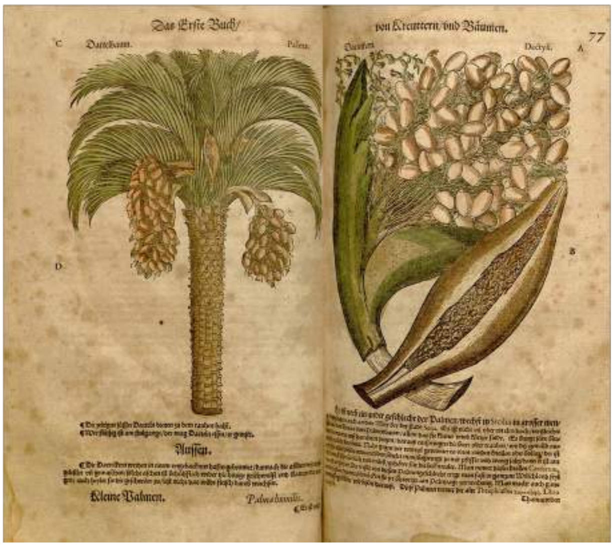

Phoenix dactylifera in *New Kreüterbuch* by Matthioli (1563: 76, 77)

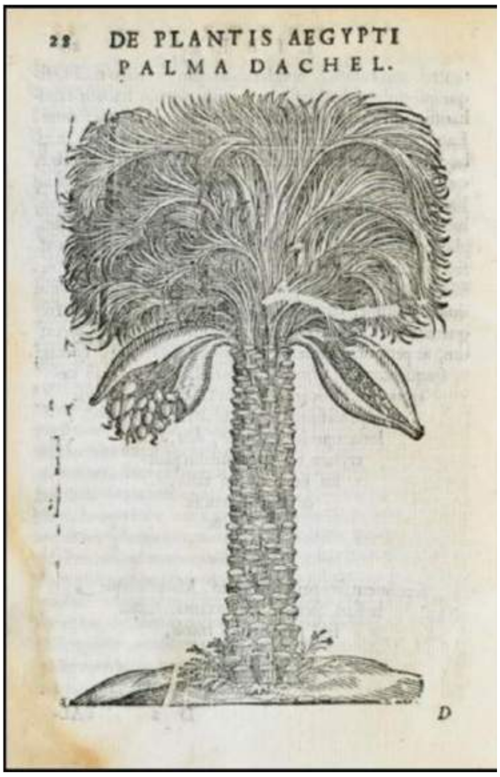

P. dactylifera in *De Plantis Ægypti liber* by Alpini (1640: 28)

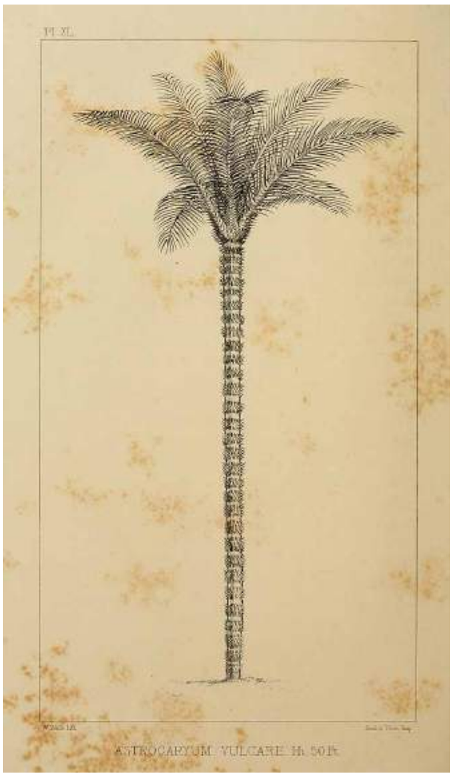

Astrocaryum vulgare engraving in *Palm trees of the Amazon and their uses* by Wallace, A.R. (1853: t. 40, p. 105). Retrieved from Plantillustrations.org

Historia Naturalis & Medica Piso, 1658 Page number 129

Vernacular name(s) Airi

Species Astrocaryum aculeatissimum (Schott) Burret

Family Arecaceae

Presence in the HNB No

Marcgrave (1648)

Piso (1648)

Notes

This woodcut is similar to the woodcut of a tree called *Hebene* in *Les singularitez de la France antarctique* by André Thevet (1558: 73). De Laet (1633: 561, 1640: 496) made a copy of Thevet's woodcut for his treatises on the Americas, which he used in his section on Brazil. Other than these copies or "borrowed" images from older manuscripts, there is no image of this palm tree in the sources under study. However, the fruits of *A. aculeatissimum* are depicted in one of Eckhout's still-life paintings.

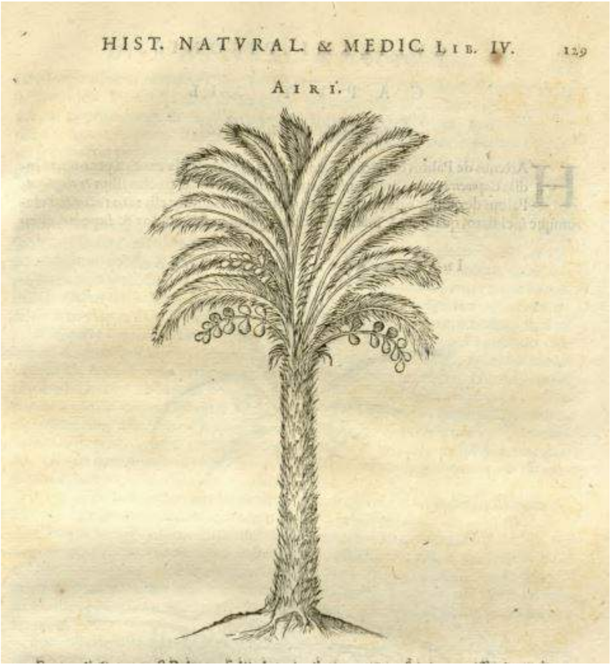

IV. *De Arboribus, fructibus, & herbis medicis*: 129

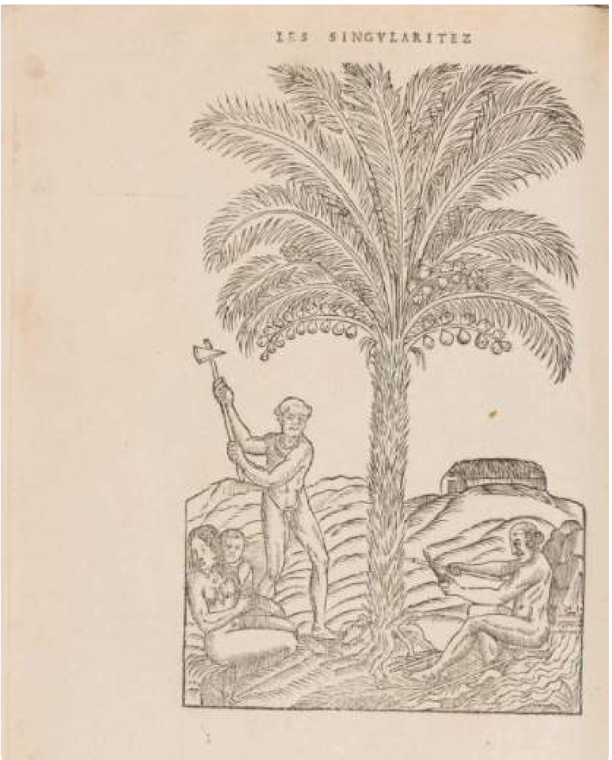

Les singularitez de la France antarctique, by Thevet (1558: 73)

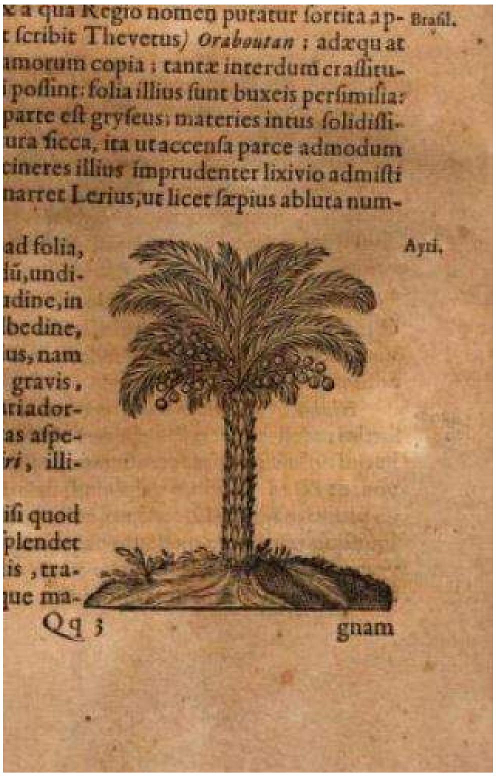

Americae utriusque Descriptio Nouus orbis seu Descriptionis Indiae Occidentalis, by De Laet (1633: 561

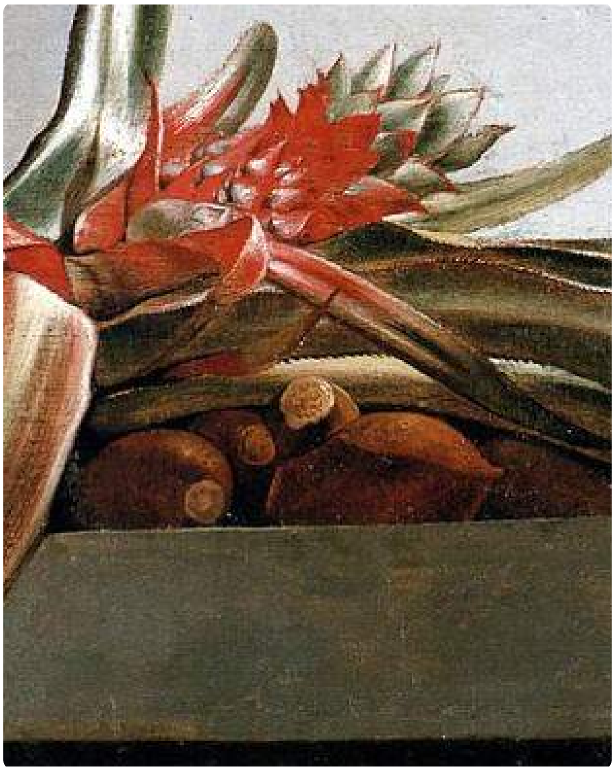

Fruits of *A.aculeatissimum* in still-life by Eckhout, c. 1640 (Identified by Buvelot et al. 2004)

Historia Naturalis & Medica Piso, 1658 Page number 130

Vernacular name(s) Inaiguaçuiba. Inaiaguaçu, Coqueiro, Cocos (fruit)

Species Cocos nucifera L.

Family Arecaceae

Presence in the HNB Yes

Marcgrave (1648) 138 (only description)

Piso (1648) 63 (only description)

Notes

The coconuts strongly resemble those painted by Eckhout in his still-life. The palm leaves show some resemblance to the palms depicted in Barlaeus (1647) and in Post's landscape paintings, but the similarities as not as accurate as with the fruits. Coconut fruits are also depicted in the *Libri Principis* (f. 77), and its inflorescence in the *Misc. Cleyeri* (f. 12r) and the still-life by Eckhout made after the latter. A woodcut of this palm and its fruits was also published in the *Exoticorum Libri Decem* by Clusius (1605: 191), but the image in the IURNM is not similar to the woodcut in Clusius.

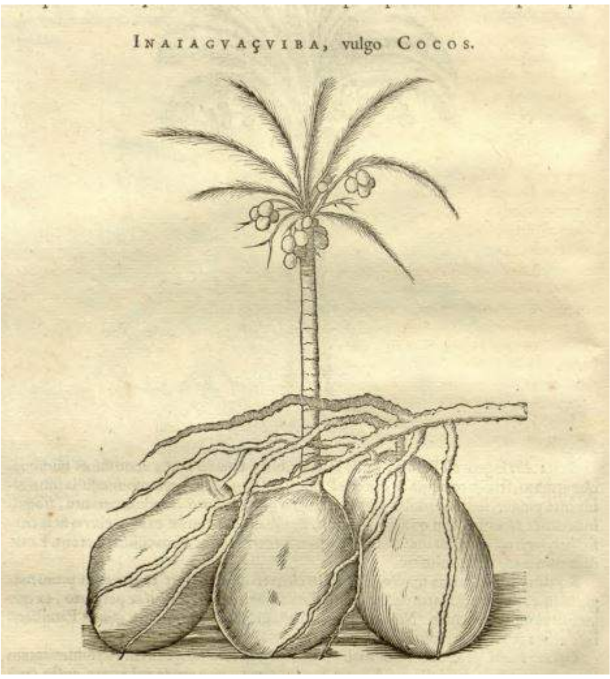

IV. *De Arboribus, fructibus, & herbis medicis*: 130

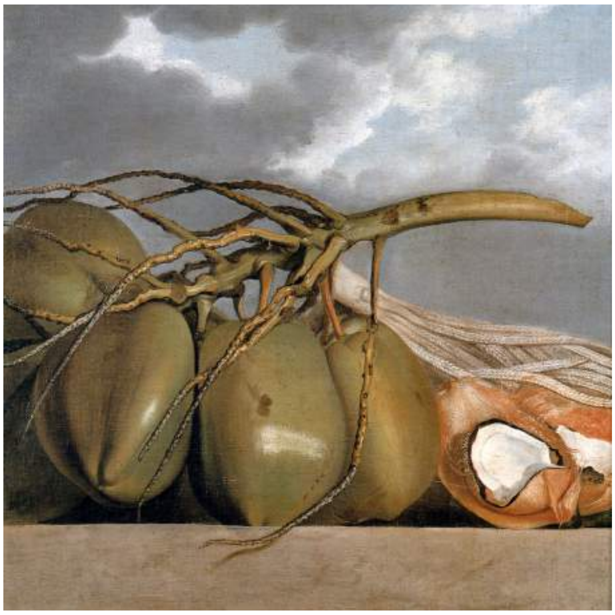

Still-life with coconuts, by Eckhout, c. 1640

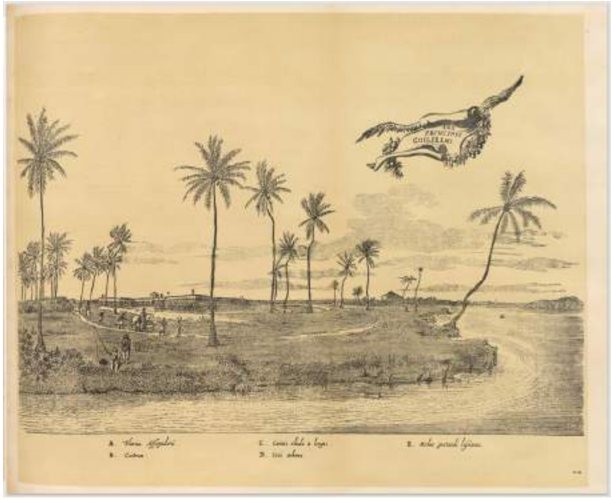

Cocos nucifera palms in woodcut depicted in *Rerum per octennium in Brasilia*, by Barlaeus (1647: 198-199)

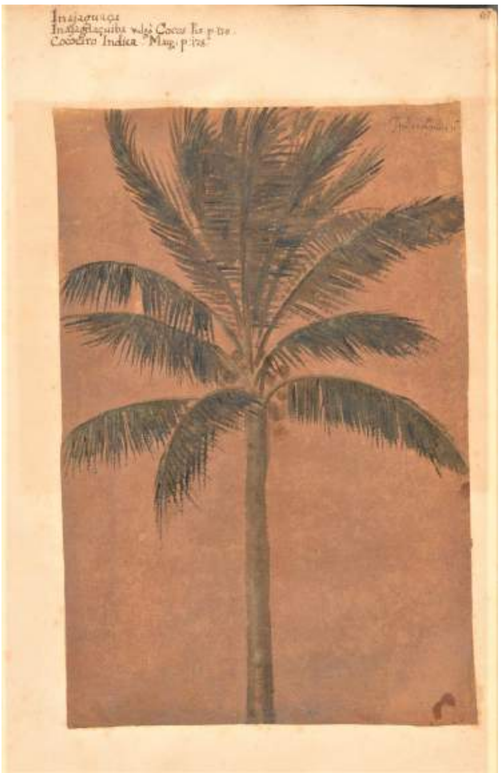

C. nucifera palm in the *Theatrum Rerum Naturalium* f. 67

Historia Naturalis & Medica Piso, 1658 Page number 132

Vernacular name(s) Aroeira

Species Schinus terebinthifolia Raddi

Family Anacardiaceae

Presence in the HNB Yes

Marcgrave (1648) 90

Piso (1648) 64

Notes

This is a slightly modified woodcut from the one printed in the HNB. An image of this branch, which represents *Schinus molle* (not *S. terebinthifolia*) was depicted in Clusius (1611: 94) in his commentaries on Monardes' medicinal plants. Later, De Laet (1633: 404, 1640: 327) also used this image. Unfortunately, none of the scholars realized that the plant described by Marcgrave and Piso -*S. terebinthifolia*- was indeed in the collection of oil paintings of the *Theatrum* (f. 295). For more images, see the supplementary file with the woodcuts of the HNB (S2). For more information on this plant woodcut, see the main paper associated with this database.

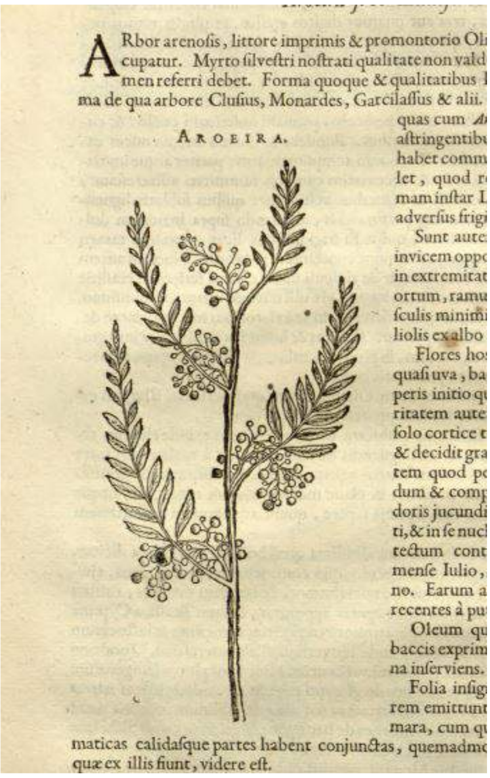

IV. *De Arboribus, fructibus, & herbis medicis*: 132

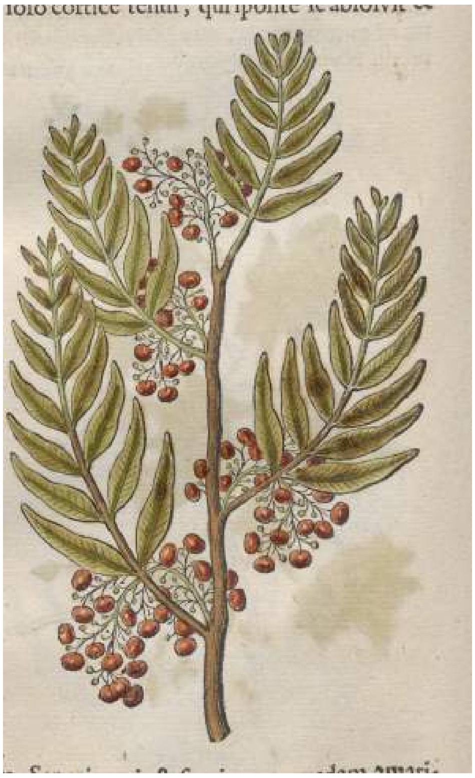

Schinus molle branch in the HNB (Marcgrave 1648: 90)

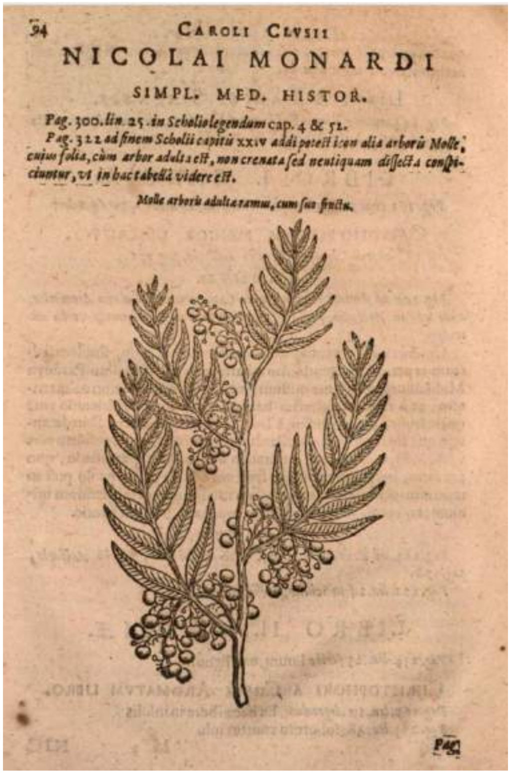

S. molle woodcut in Curae Posteriores, by Clusius (1611: 94)

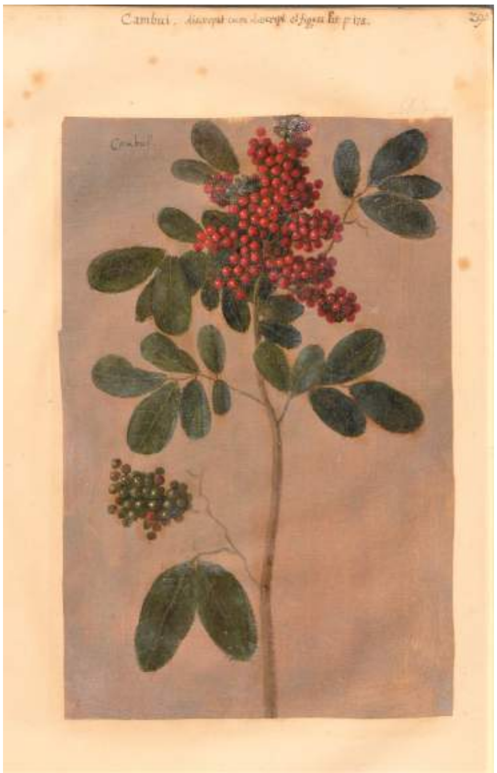

S. terebinthifolia fruiting branch in the *Theatrum Rerum Naturalium*, f. 295

Historia Naturalis & Medica Piso, 1658 Page number 133b

Vernacular name(s) Urucu

Species Bixa orellana L.

Family Bixaceae

Presence in the HNB Yes

Marcgrave (1648) 61 (different woodcut)

Piso (1648) 65 (only description)

Notes

This branch was copied from the woodcut in the *Exoticorum Libri Decem* by Clusius (1605: 74). Clusius obtained the original branch from the aristocrat and naturalia collector Pieter Garet (c.1552/5-1631), who described it to the botanist how Brazilian indigenous peoples used the seeds to color their bodies red (Egmond 2009: 82).
It seems as if the draughtsperson took the fruit from the left of the branch, opened it, and left it in the bottom left corner to depict it next to the branch. The woodcut in the IURNM does not bear resemblance to the oil painting in the *Theatrum* (f. 95).

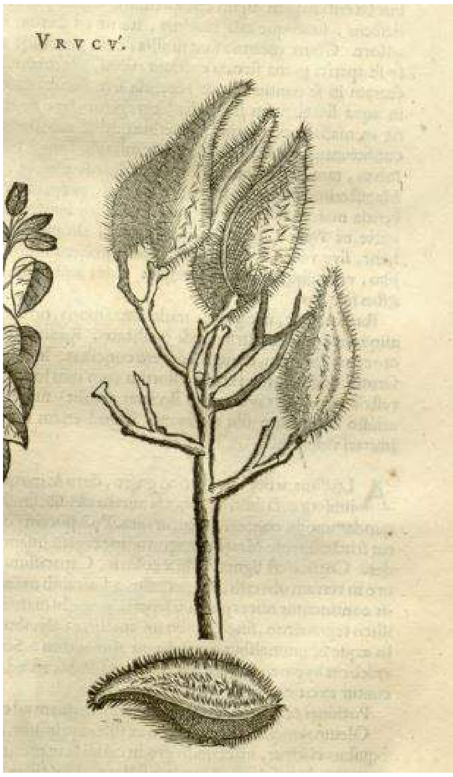

IV. *De Arboribus, fructibus, & herbis medicis*: 133b

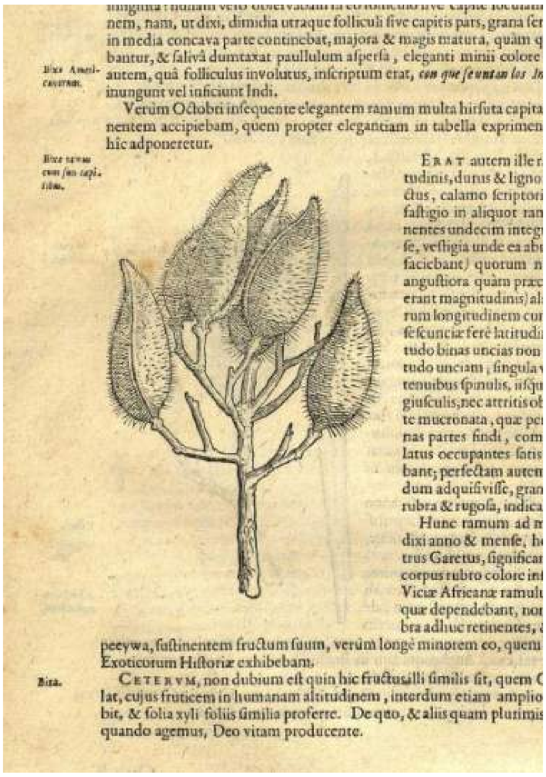

[illegible]
nem, nam, ut dixi, dimidia utraque folliculi five capitis pars, grana fer
in media concava parte continebat, majora & magis matura, quàm q
bantur, & falivâ dumtaxat paullulum afperfa, eleganti minii colore
autem, quâ folliculus involutus, infcriptum erat, *con que feuntan los In*
inungunt vel inficiunt Indi.

Verùm Octobri infequente elegantem ramum multa hirfuta capita
nentem accipiebam, quem propter elegantiam in tabella exprimen
hic adponeretur.

ERAT autem ille r
tudinis, durus & ligno
ctus, calamo fcriptori
faftigio in aliquot ram
nentes undecim integ
fe, veftigia unde ea abi
faciebant) quorum n
anguftiora quàm præc
erant magnitudinis) al
rum longitudinem cur
fefcunciæ ferè latitudi
tudo binas uncias non
tudo unciam, fingula v
tenuibus fpinulis, iifqu
giufculis, nec attritis ol
te mucronata, quæ pe
nas partes findi, com
latus occupantes fatis
bant; perfectam auten
dum adquifiviffe, gran
rubra & rugofa, indica

Hunc ramum ad m
dixi anno & menfe, h
trus Garetus, fignifica
corpus rubro colore in
Viciæ Africanæ ramul
quæ dependebant, no
bra adhuc retinentes,
peeywa, fuftinentem fructum fuum, verùm longè minorem eo, quem
Exoticorum Hiftoriæ exhibebam.

Bixa. CETERVM, non dubium eft quin hic fructus illi fimilis fit, quem C
lat, cujus fruticem in humanam altitudinem, interdum etiam amplio
bit, & folia xyli foliis fimilia proferre. De quo, & aliis quam plurimis
quando agemus, Deo vitam producente.

Bixa orellana branch in *Exoticorum Libri Decem,* by Clusius (1605: 74)

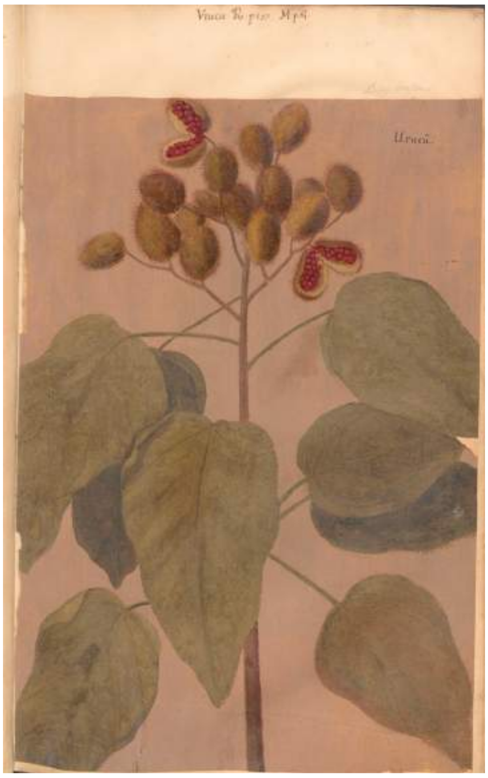

B. orellana fruiting branch in the *Theatrum Rerum Naturalium,* f. 95

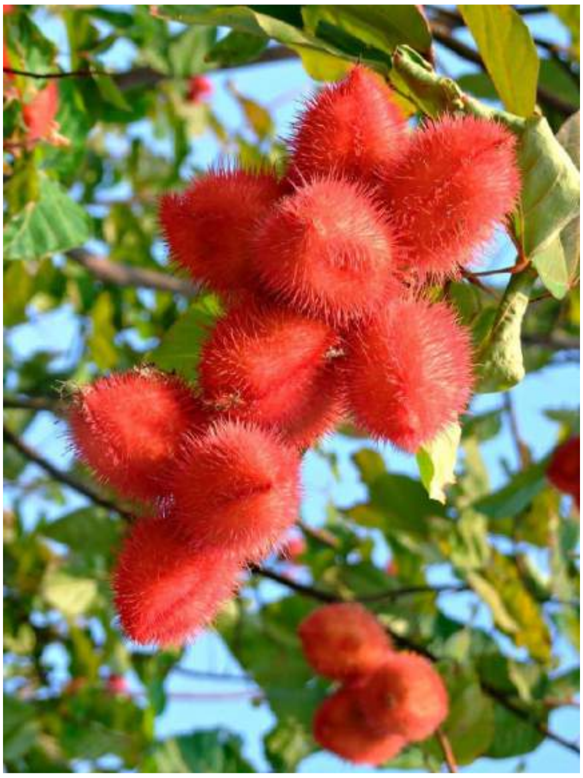

"Annatto Dye Tree or Lipstick Tree (*Bixa orellana*), by kaiyanwong223 (CC BY-NC-SA 2.0)

Historia Naturalis & Medica Piso, 1658 Page number 135a

Vernacular name(s) Iacapucaío

Species Lecythis pisonis Cambess

Family Lecythidaceae

Presence in the HNB Yes

Marcgrave (1648) 128 (slightly different woodcut)

Piso (1648) 66 (slightly different woodcut)

Notes

This is a slightly modified woodcut from the one printed in the HNB, which in turn appears in De Laet's manuscript as a lead pencil drawing (Sloane MS 1554, ff. 46v and 46r). This drawing was likely transferred to the woodblock. Piso remade it for the IURNM. The fruits and leaves are slightly different and the main branch is attached to a trunk. It is striking that the impressive flowers of this plant were not depicted -to our knowledge- in any of the visual sources of Dutch Brazil.

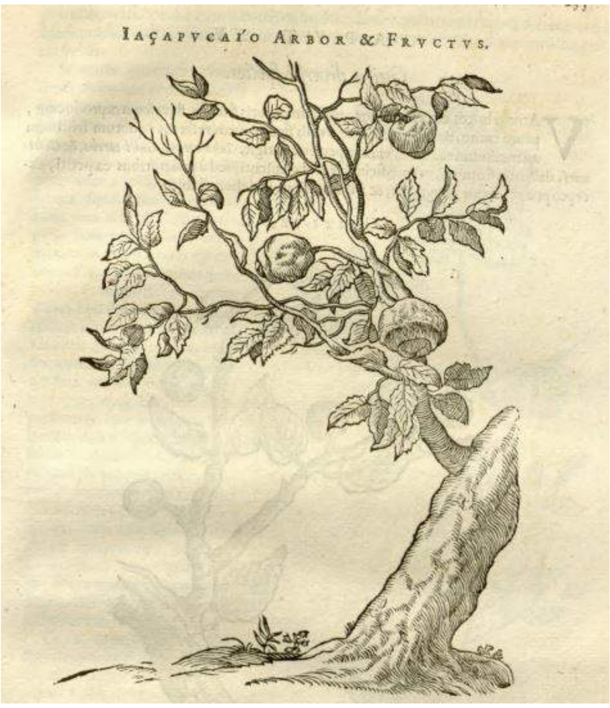

IV. *De Arboribus, fructibus, & herbis medicis*: 135a

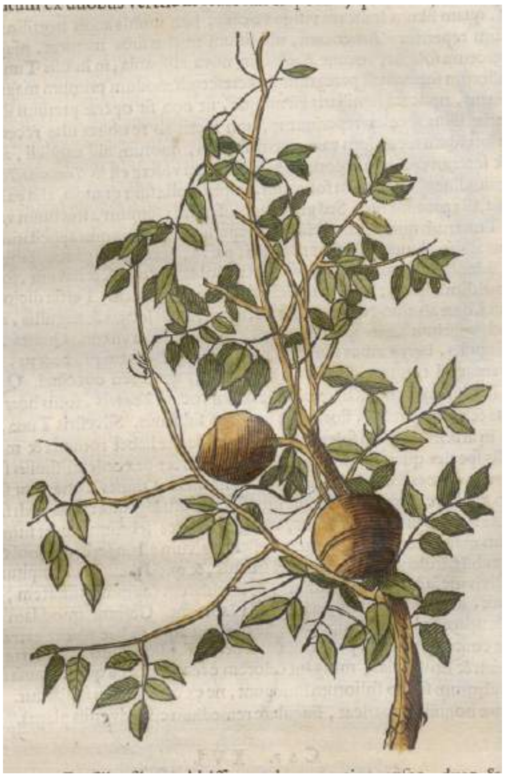

L. pisonis branch with fruits in the HNB (Marcgrave 1648: 128)

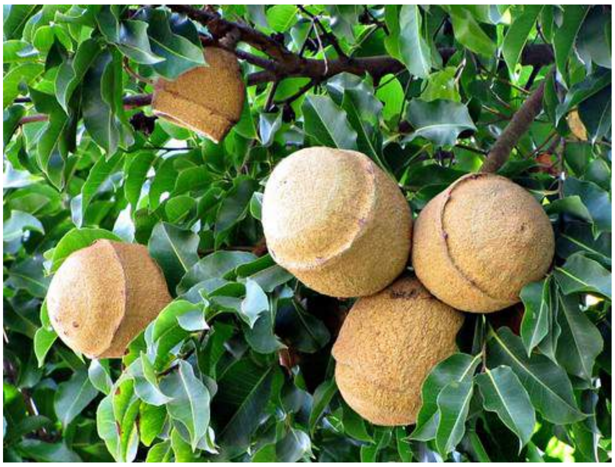

"Sapucaia (*Lecythis pisonis*)" by Mauricio Mercadante (CC BY-NC-SA 2.0)

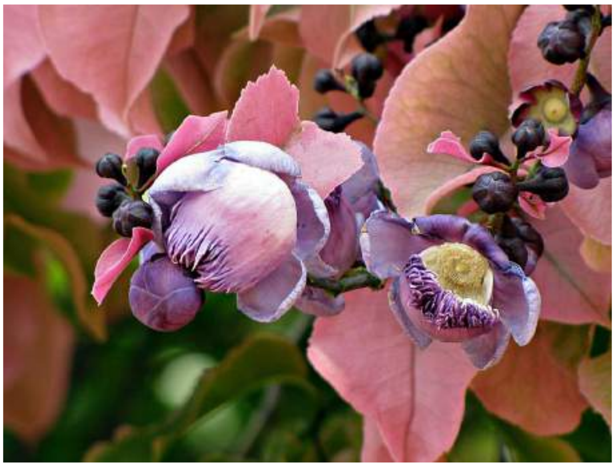

L. pisonis impressive flowers were not depicted in the HNB nor the IURNM. "Sapucaia (*Lecythis pisonis*)" by Mauricio Mercadante (CC BY-NC-SA 2.0)

Historia Naturalis & Medica Piso, 1658 Page number 135b

Vernacular name(s) Iacapucaío fructus

Species Lecythis pisonis Cambess

Family Lecythidaceae

Presence in the HNB Yes

Marcgrave (1648) 128 (different woodcut)

Piso (1648) 66 (different woodcut)

Notes

This big fruit, known as the "monkey pot" bears some resemblance to the oil painting in the *Theatrum* (f. 37). In this illustration, the fruit of *Macoubea guianensis* is also depicted, and the inflorescence of *Costus spiralis* (Alcantara-Rodriguez et al. 2021). A cut seed of *L. pisonis* lays on the left of the folio. This is a characteristic species of eastern Brazil, which impressive fruits were also depicted two centuries later in the *Flora Brasiliensis* by German botanist Carl Friedrich Philipp von Martius, who -as other botanists working in the tropics- followed the work by Marcgrave and Piso.

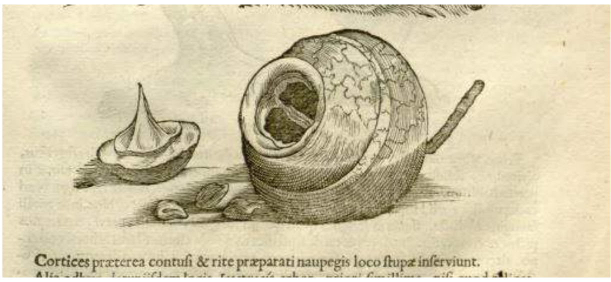

IV. *De Arboribus, fructibus, & herbis medicis*: 135b

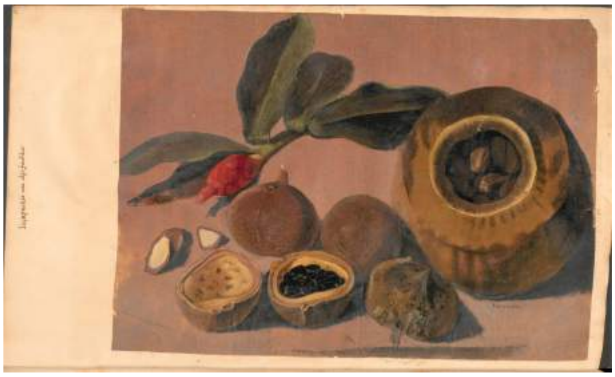

L. pisonis fruit open with seeds, among other fruits, in the *Theatrum Rerum Naturalium,* f. 37

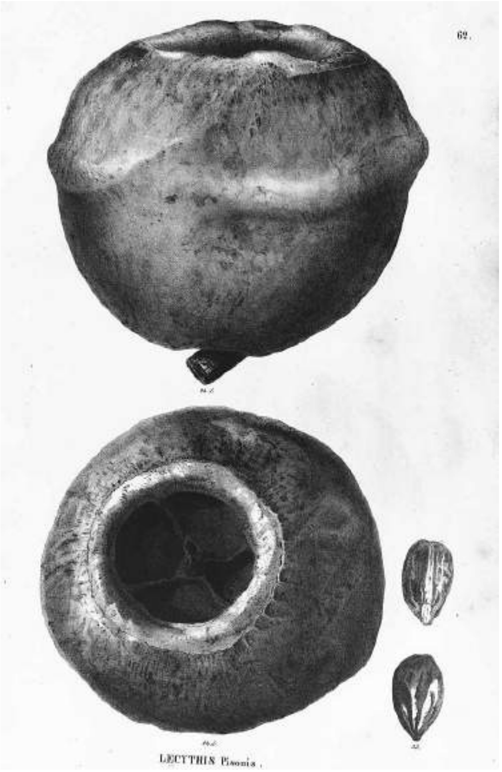

Engraving of *L. pisonis*. Martius, C.F.P. von, Eichler, A.G., Urban, I., *Flora Brasiliensis* (1840-1906). Vol 14 (1): 62

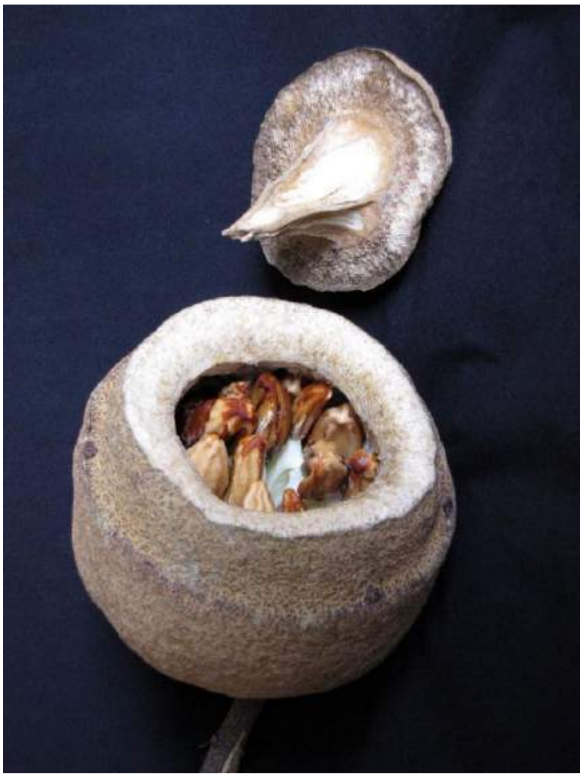

Fruit open with seeds of *L. pisonis*. "Sapucaia (*Lecythis pisonis*)" by Mauricio Mercadante (CC BY-NC-SA 2.0)

Historia Naturalis & Medica Piso, 1658 Page number 136

Vernacular name(s) Guiti-Iba I. Guiti-coroya (fruit)

Species Couepia rufa Ducke

Family Chrysobalanaceae

Presence in the HNB Yes

Marcgrave (1648) 114

Piso (1648) 67

Notes

The woodcut in the HNB (which is the same for both Marcgrave and Piso) is very similar to the illustration in the *Theatrum* (f. 99) (see the Supplementary file with the woodcut images of the HNB - S2). The woodcut in the IURNM, however, is a new image, made after the one in the HNB, but attached to a trunk and with the open fruit and seed laying on the left, bottom corner.

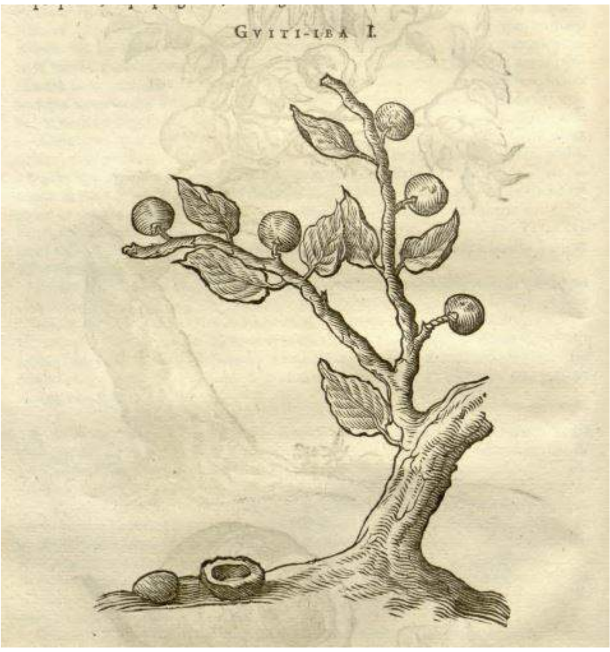

IV. *De Arboribus, fructibus, & herbis medicis: 136*

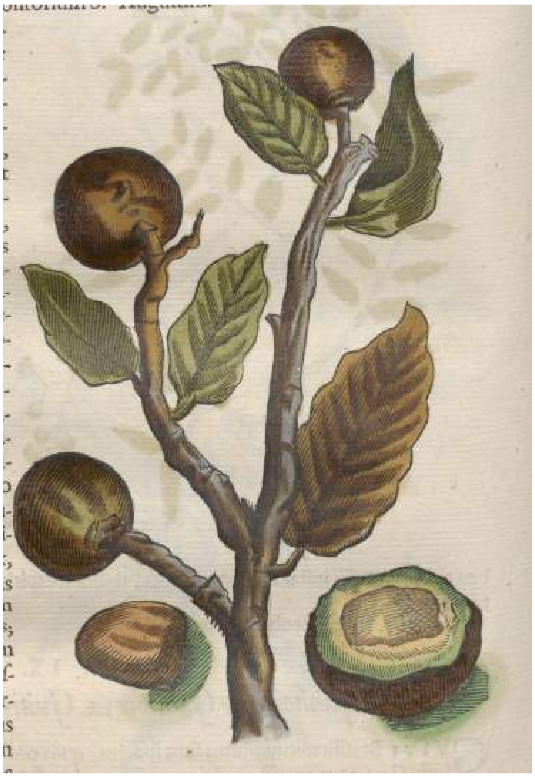

C. rufa in the HNB (Marcgrave 1648: 114)

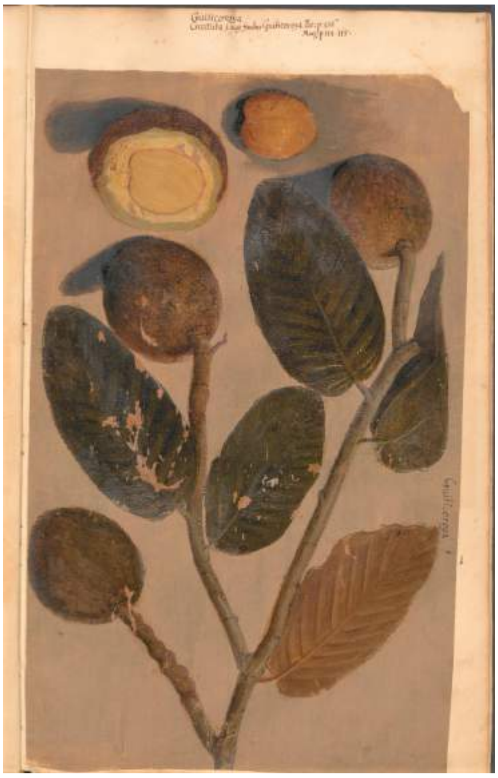

C. rufa in the *Theatrum Rerum Naturaliu*, f. 99

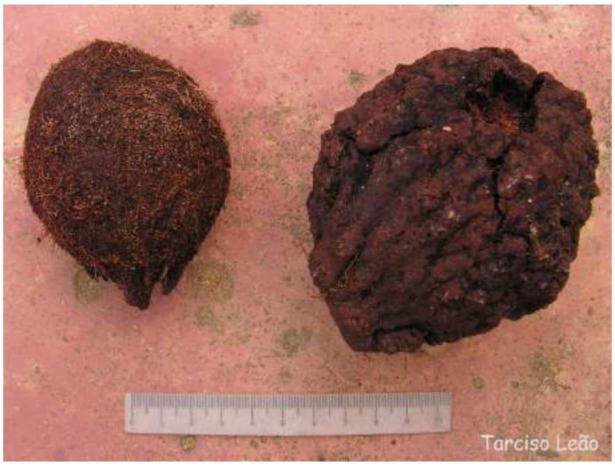

Fruit and seed of "*Couepia rufa*, oiti-coró" by Tarciso Leão, collected in Pernambuco, Brazil (CC BY 2.0)

Historia Naturalis & Medica Piso, 1658 Page number 139

Vernacular name(s) Açaiá

Species Spondias mombin L.

Family Anacardiaceae

Presence in the HNB Yes

Marcgrave (1648) 129b (different woodcut)

Piso (1648) 77 (only description)

Notes

Piso added a new image as he likely preferred to show a fertile branch (with flowers and fruits) rather than the infertile compound leaf made by De Laet after the herbarium specimen (f. 53). Both authors described the leaves, flowers, and fruits of this tree, as well as its medicinal properties. Marcgrave added the use of its wood to make cork to seal bottles and he described its propagation for cultivation. Piso mentioned how certain type of birds build their nests in the branches of these high trees to avoid being eaten by snakes and harmful insects. Surprisingly, this interesting plant-animal relationship observation came from the physician, instead of the naturalist Marcgrave, as one would expect.

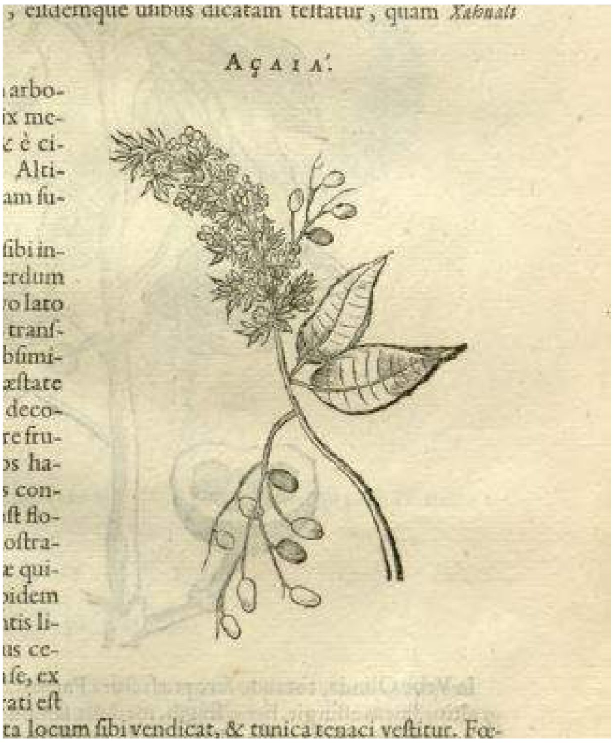

IV. *De Arboribus, fructibus, & herbis medicis: 139*

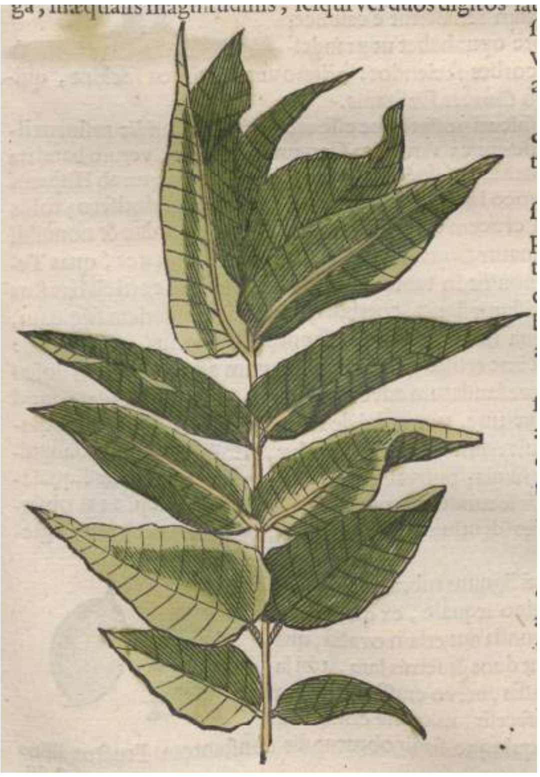

S. mombin infertile branch in the HNB (Marcgrave 1648: 129b)

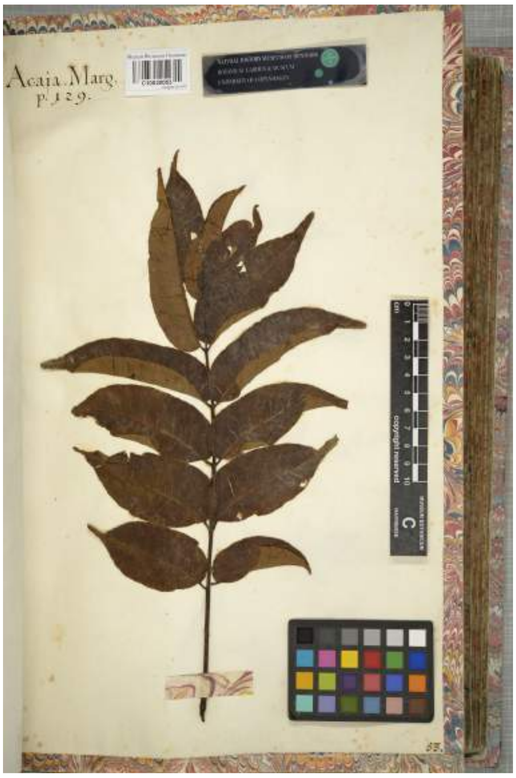

S. mombin in Marcgrave's herbarium, f. 53

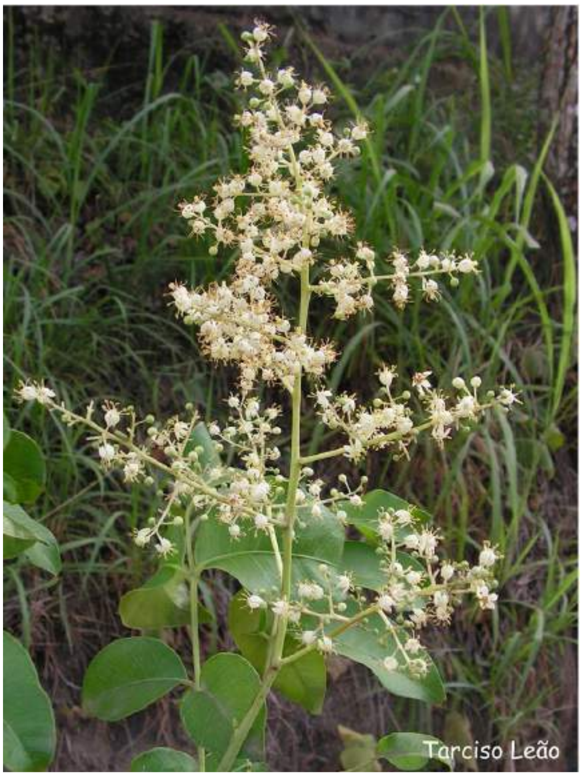

Flowering branch of "*Spondias mombin*, cajá" by Tarciso Leão (CC BY 2.0).

Historia Naturalis & Medica Piso, 1658 Page number 143

Vernacular name(s) Caaroba

Species Jacaranda brasiliana (Lam.) Pers.

Family Bignoniaceae

Presence in the HNB Yes

Marcgrave (1648) 293

Piso (1648) 70 (only description)

Notes

This woodcut is what Pickel (2008) would categorize as a "fantasy woodcut" because Piso mixed several species and wrongly assigned it to the description of *J. brasiliana*. The combined plants are *Senna latifolia* (Piso 1648: 85), and perhaps some kind of *Phaseolus* spp. This image resembles more a member of the Fabaceae family rather than a Bignoniaceae. The *J. brasiliana* woodcut is placed at the end of the HNB (Marcgrave 1648: 293b) with other loose woodcuts, as De Laet could not match it with the description. Later, Piso (1658: 165a) misplaced the same image in his book.

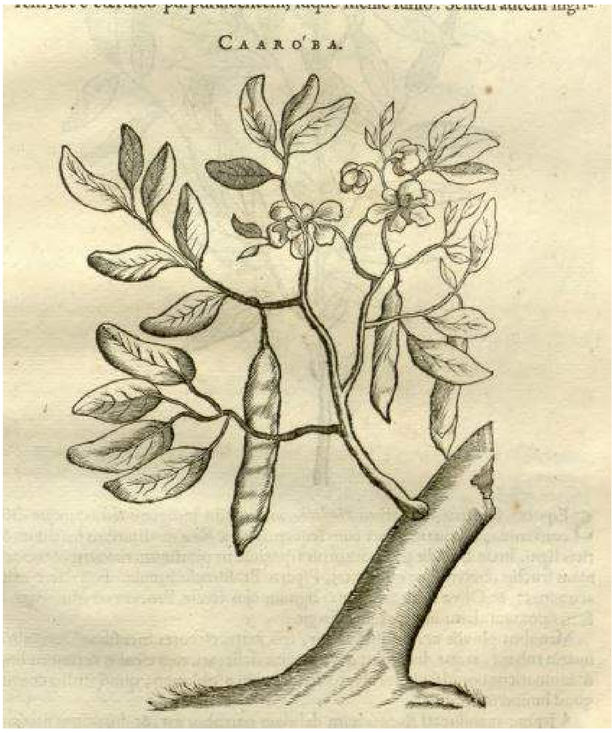

IV. *De Arboribus, fructibus, & herbis medicis*:143

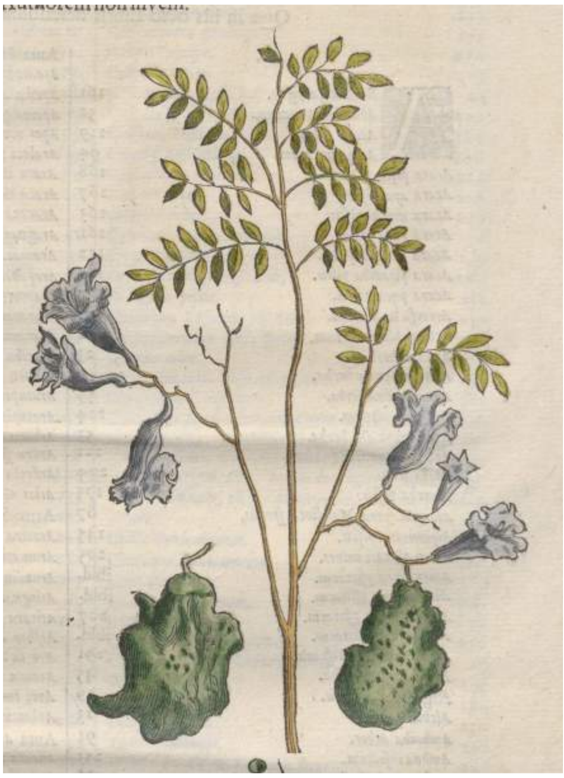

Woodcut of *Jacaranda brasiliana* the HNB (Marcgrave 1648: 293b)

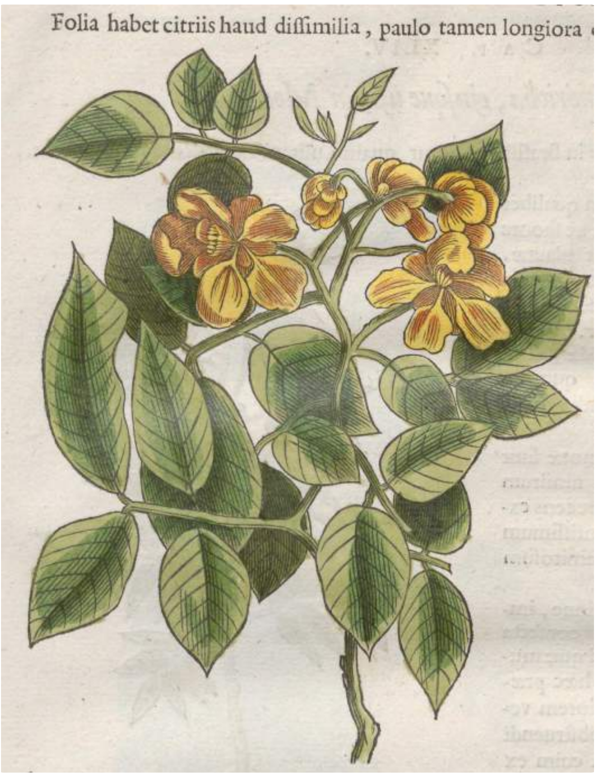

Senna latifolia in the HNB (Piso 1648: 85)

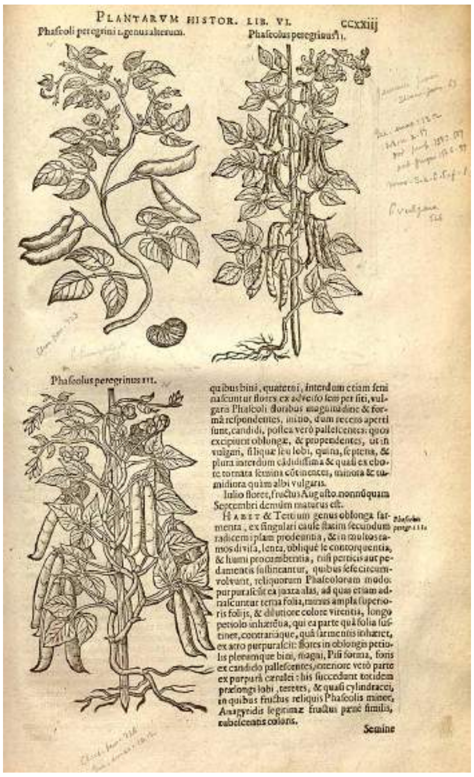

Phaseolus spp. in the *Rariorum Plantarum* by Clusius (1601: vol. 2, p. 223)

Historia Naturalis & Medica Piso, 1658 Page number 146a

Vernacular name(s) Anhuíba. Sassafras

Species Ocotea sassafras (Meisn.) Mez

Family Lauraceae

Presence in the HNB Yes

Marcgrave (1648)

Piso (1648) 98 (only description)

Notes

Piso used an image of the popular Sassafras tree (*Sassafras albidum*), which according to Monardes (1574: 51) was a cure for syphilis, and would also cure many diseases. This woodcut first appeared in Monardes and later in De Laet (1633: 217, 1640: 294), who likely ordered to make a copy based on Monardes' woodcut. Other scholars, such as Dalechamps (1586: 1786) and Bauhin (1650: 483) copied this image for their works due to the popularity of this plant and its virtues as a panacea (Lloyd 1911: 74-75). However, this is not the image of *O. sassafras*, which was never depicted neither in Marcgrave nor Piso.

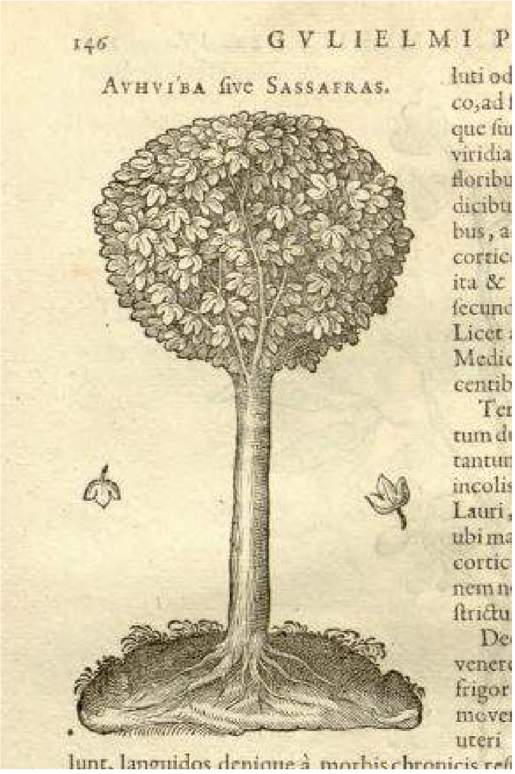

IV. *De Arboribus, fructibus, & herbis medicis*:146a

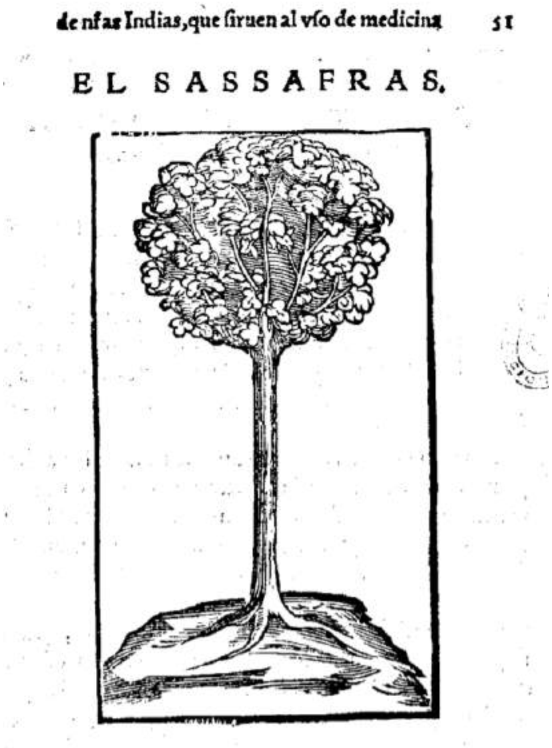

Primera y segunda y tercera partes de la historia medicinal, de las cosas que se traen de nuestras Indias Occidentales, by Monardes (1574: 51)

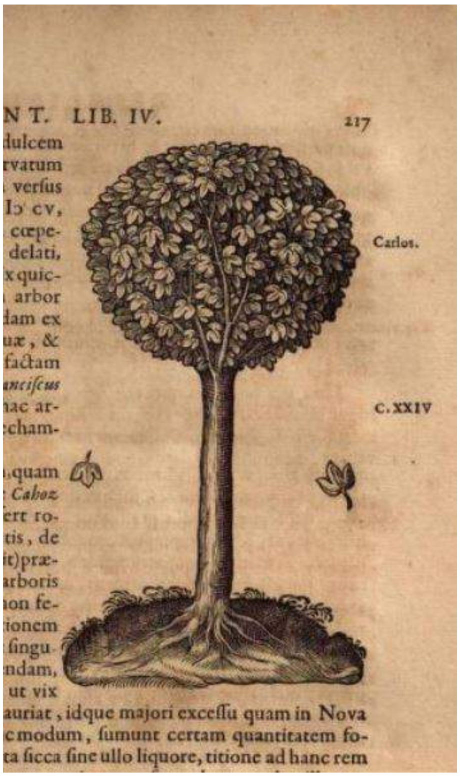

Sassafras albidus, in *Americae utriusque Descriptio Novus orbis seu Descriptionis Indiae Occidentalis*, by De Laet (1633: 217)

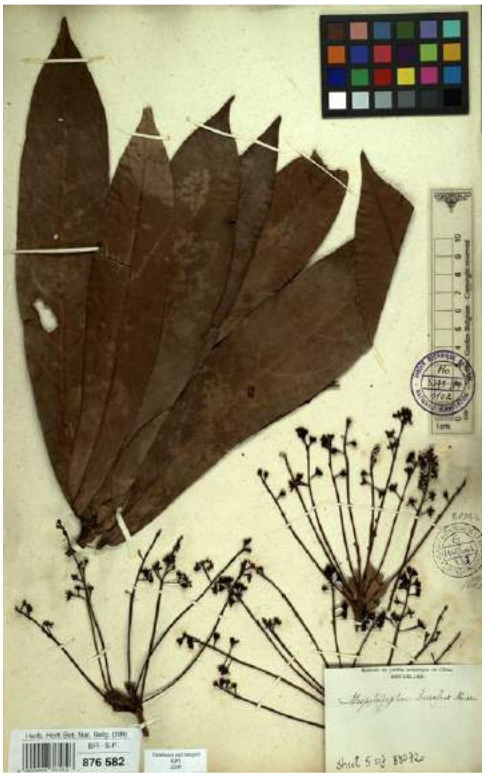

Ocotea sassafras specimen collected in Brazil by Meise Botanic Garden (CC BY-SA 4.0) Retrieved from www.gbif.org

Historia Naturalis & Medica Piso, 1658 Page number 146b

Vernacular name(s) Ibiraee. Guaiacum

Species Pradosia kuhlmannii Toledo

Family Sapotaceae

Presence in the HNB Yes

Marcgrave (1648) 101 (only description)

Piso (1648) 71 (only description)

Notes

As noticed by Pickel (2008), this woodcut does not correspond to *P. kuhlmannii*. It slightly resembles the woodcut that corresponds to *Manilkara salzmannii*, which is also a Sapotaceae. In Marcgrave´s chapter on trees, there is no woodcut, and the information is scarce. De Laet proceeded to describe it by combining information from his accounts, where he called it *Hivorae* (De Laet 1633: 497), and from two pharmacists who crossed the ocean and reported that Indigenous peoples used it against venereal diseases. Nevertheless, De Laet does not show a figure either. This species is endemic to the Atlantic Rainforest in Brazil and it is currently endangered due to habitat loss (www.cncflora.jbrj.gov.br/,www.iucnredlist.org/).

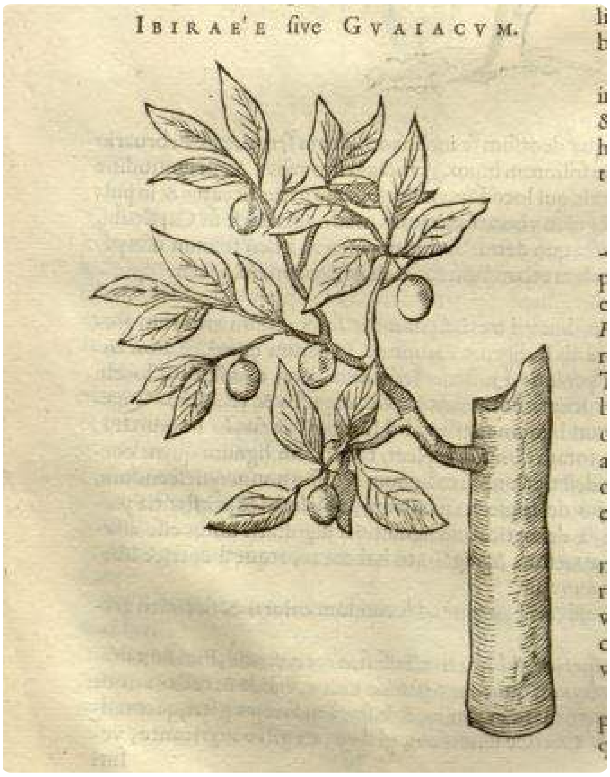

IV. *De Arboribus, fructibus, & herbis medicis*:146b

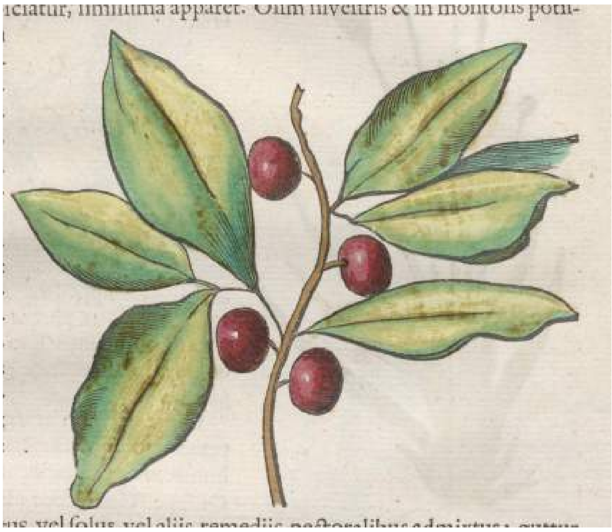

Manilkara salzmannii in the HNB (Piso 1648: 120a)

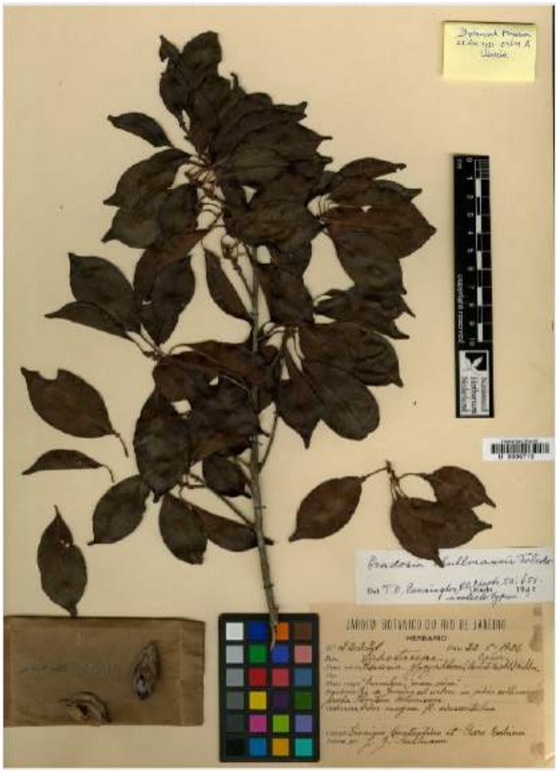

Pradosia kuhlmannii
collected in Brazil
by Naturalis Biodiversity Center (CC0 1.0)

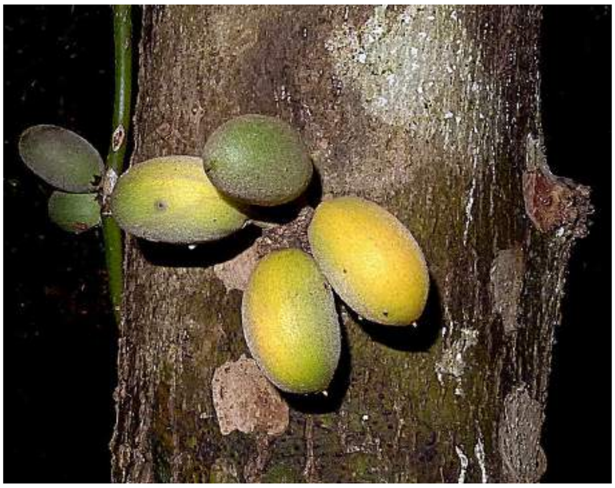

A relative of *P. kuhlmann,* which also bears sessile fruits. "*Pradosia lactescens*, Sapotaceae" by Alex Popovkin, Bahia, Brazil (CC BY-NC-SA 2.0)

Historia Naturalis & Medica Piso, 1658 Page number 153

Vernacular name(s)	Guaiaba
Species	Psidium guajava L.
Family	Myrtaceae

Presence in the HNB	Yes
Marcgrave (1648)	147 (only description)
Piso (1648)	75 (only description)

Notes

There are drawings of the *Guaiaba* fruits in the *Misc. Cleyeri* (f. 51r) and the *Libri Principis* (f. 95 [75]), but they do not resemble the woodcut by Piso (1658). The fruits from the *Libri Principis* resemble those in the still-life by Eckhout for the same species. According to Pickel (2008: 177), the woodcut was made after the description.

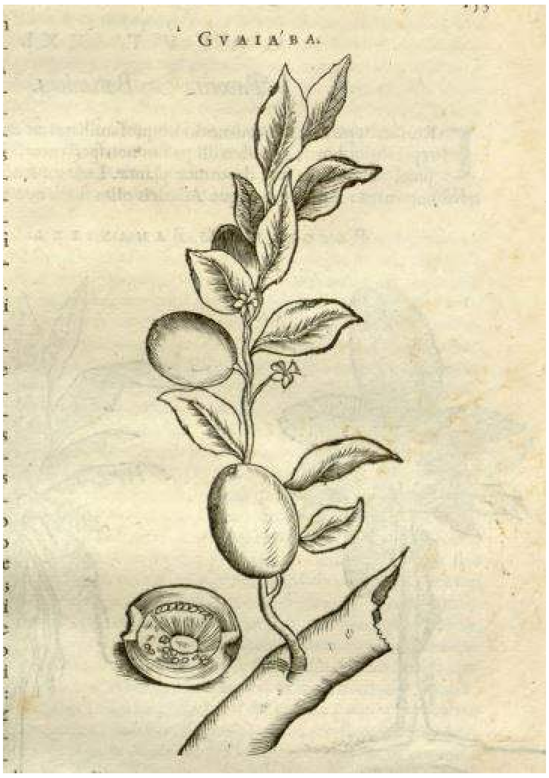

IV. *De Arboribus, fructibus, & herbis medicis*: 153

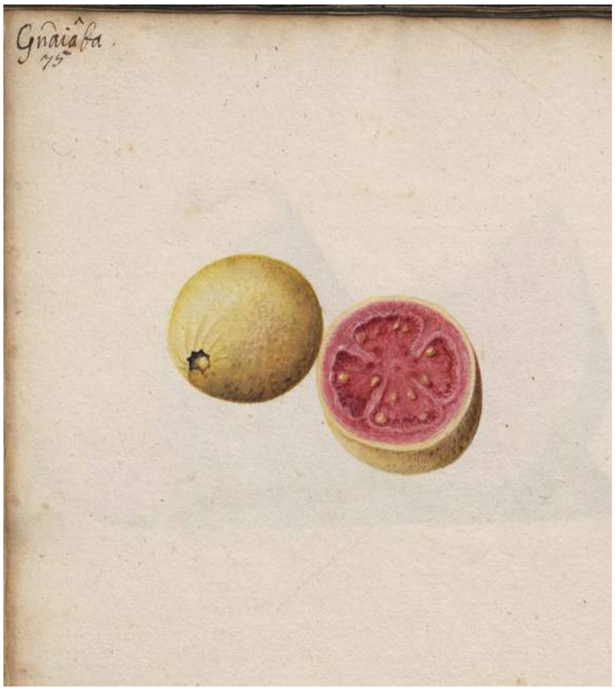

Open fruit of *Psidium guajava* in the *Libri Principis* (f. 95)

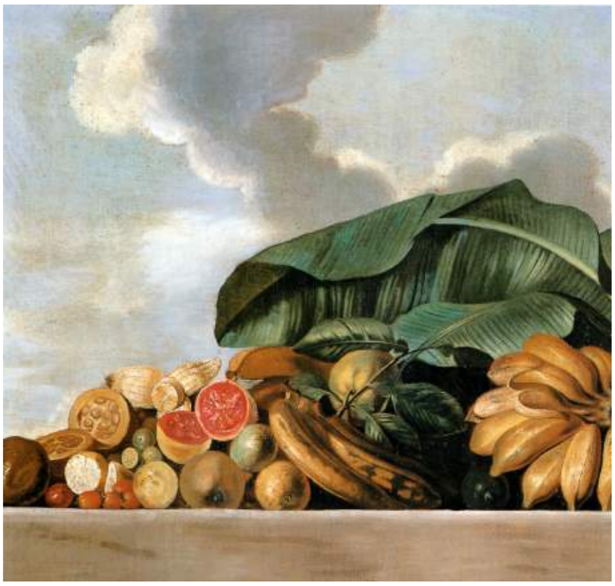

Still-life by Ekchout with *P. guajava* open fruit and closed ones below, c. 1640

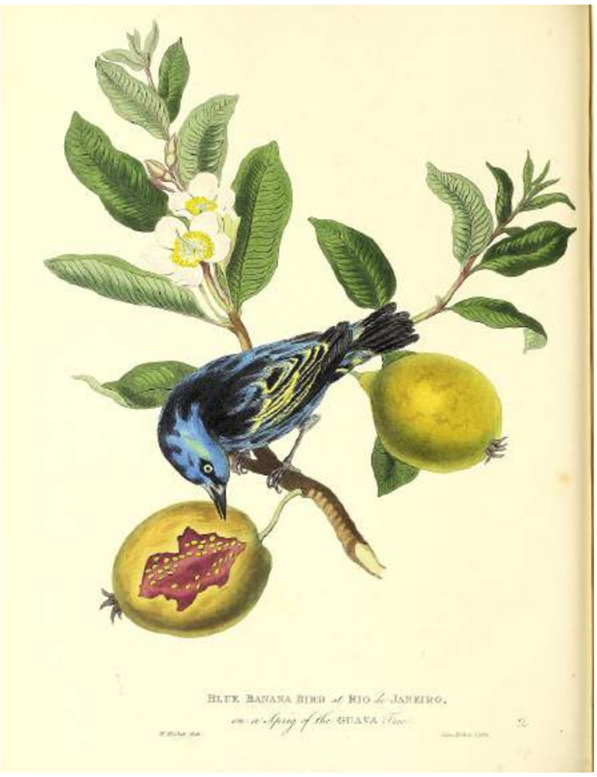

P. guajava flowering and fruiting branch illustrated by Forbes, J., *Oriental memoires* (1765)
Smithsonian Institute, Washington, D.C., U.S.A.

Historia Naturalis & Medica Piso, 1658 Page number 154b

Vernacular name(s)	Pacoeira (fruit)
Species	Musa × paradisiaca L.
Family	Musaceae

Presence in the HNB	Yes
Marcgrave (1648)	137b (different woodcut)
Piso (1648)	75 (different woodcut)

Notes

Piso used the woodcut from De Laet (1633: 563, 1640: 497). The visual repertoire of Dutch Brazil includes several representations of the banana, such as in the *Libri Principis* ff. 89 [69] and 101 [81], the still-life paintings by Eckhout, the landscape portraits by Post, the engravings depicted in Barlaeus (1647: 30-31 and 157-158), woodcuts in de l' Obel (1581: 236), etc. (see S2).

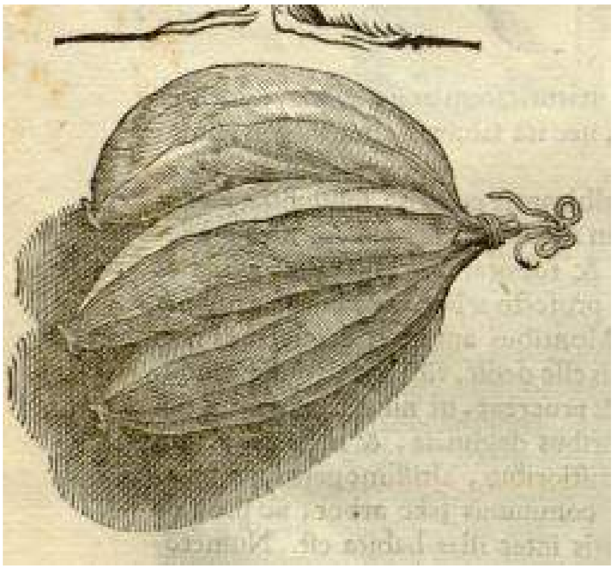

IV. *De Arboribus, fructibus, & herbis medicis*: 154b

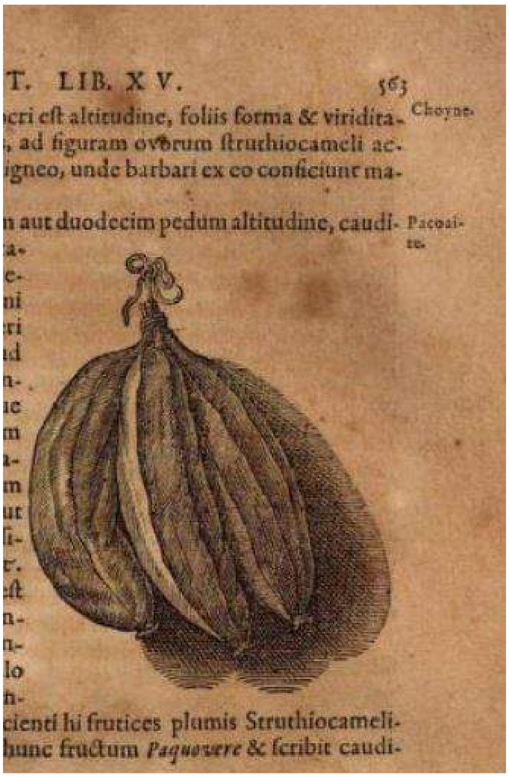

Bananas in *Americae utriusque Descriptio Novus orbis seu Descriptionis Indiae Occidentalis*, by De Laet (1633: 563)

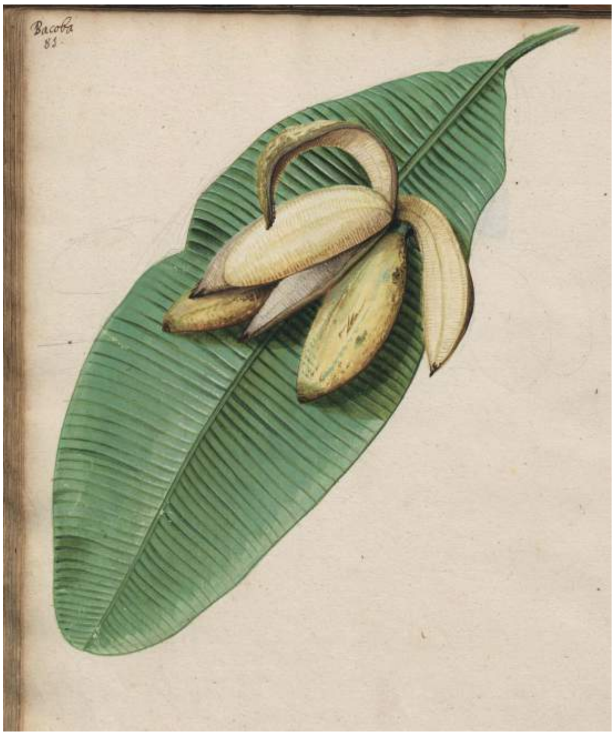

Musa x. *paradisiaca* in the *Libri Principis* (f. 69)

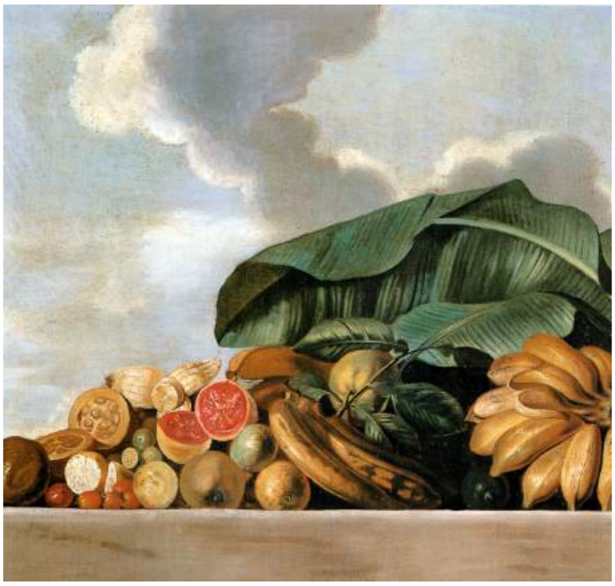

Eckhout still-life with bananas, c. 1640

Historia Naturalis & Medica Piso, 1658 Page number 154c

Vernacular name(s) Bananeira

Species Musa × paradisiaca L.

Family Musaceae

Presence in the HNB Yes

Marcgrave (1648) 137b (different woodcut)

Piso (1648) 75 (different woodcut)

Notes

Of the several sources where this plant is depicted (see previous entry), the greatest similarity is with the banana plant in Barlaeus (1647: 30-31) designed by Post. In the Renaissance scholar's herbals, we found this plant in Matthioli (1563: 79), and later in de l' Obel (1581: 236) which woodcut seems to be made after the former.

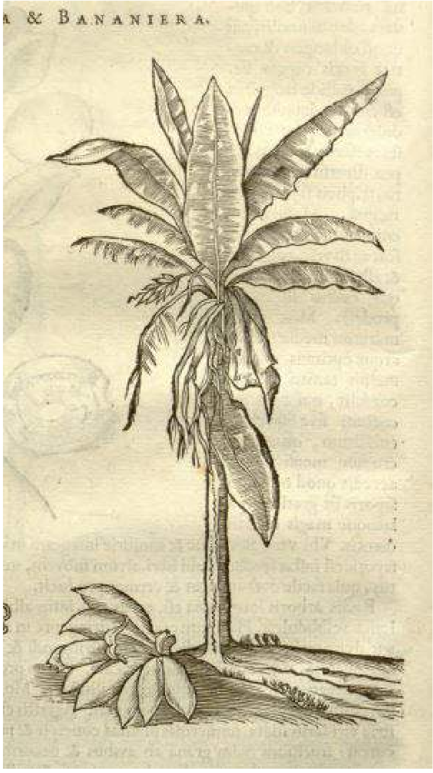

IV. *De Arboribus, fructibus, & herbis medicis*: 154c

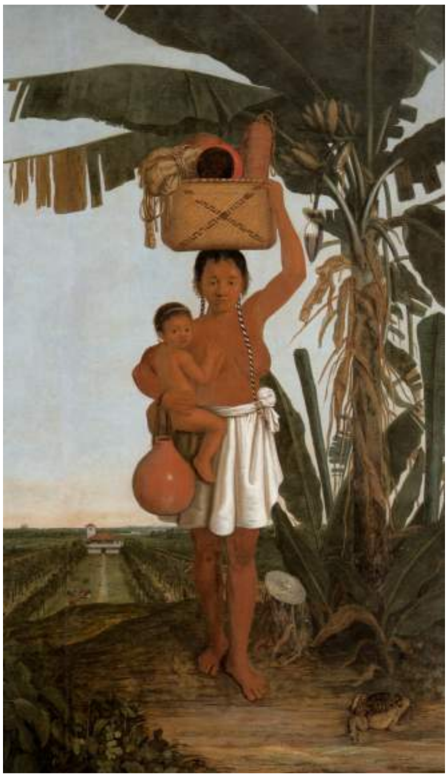

Banana plant in the painting "Tupi woman holding a child" by Eckhout, 1641 (National Museum of Denmark)

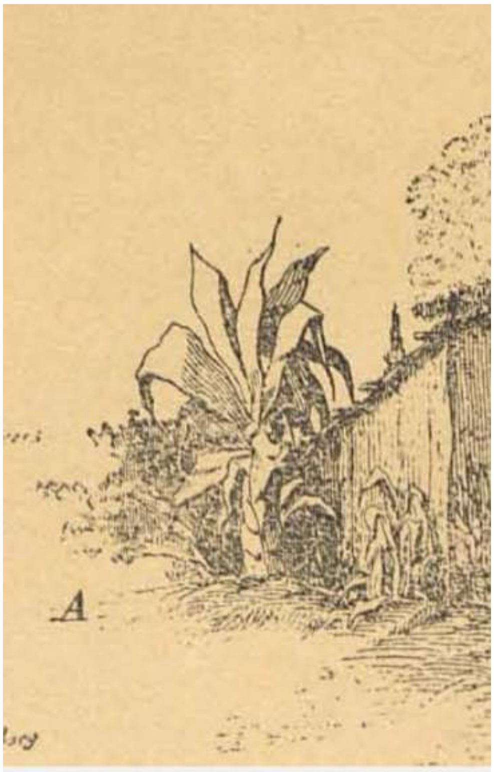

Close- up of the *Musa* sp. in Barlaeus (1647: 30-31)

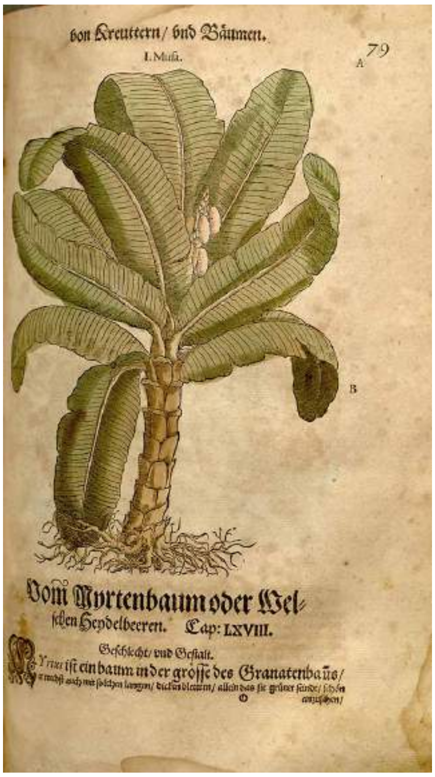

Musa sp. in the *New Kreuterbuch* by Matthioli (1563: 79)

Historia Naturalis & Medica Piso, 1658 Page number 156

Vernacular name(s) Mangaíba

Species Hancornia speciosa Gomes

Family Apocynaceae

Presence in the HNB Yes

Marcgrave (1648) 122 (different woodcut)

Piso (1648) 76 (different woodcut)

Notes

This species is represented in several sources from Dutch Brazil: *Misc. Cleyeri* (f. 51r), *Libri Principis* (f. 71 [51]), and Eckhout's still-life paintings. There is an extensive description of *H. speciosa* in both the HNB and the IURNM, as this tree was abundant and popular at that time (used as medicine and food). The woodcut in the IURNM does not resemble any of those sources, and we did not find it in older ones. Thus, Piso likely created it for his treatise.

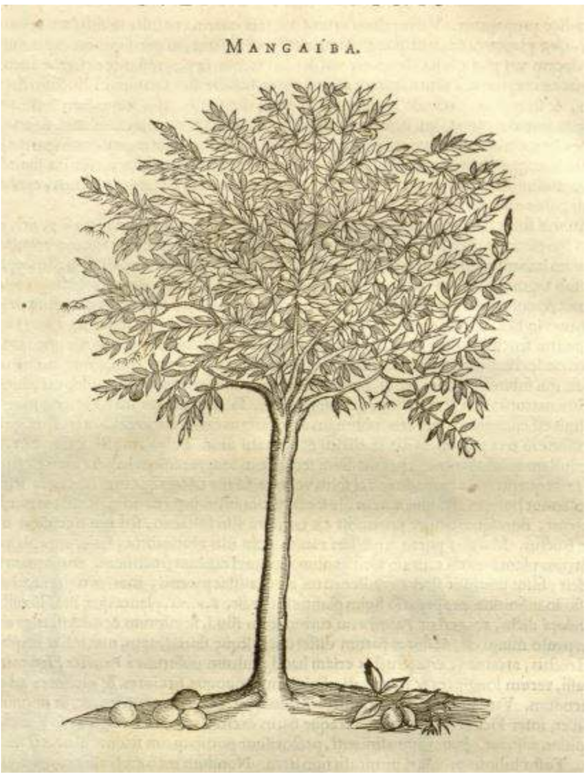

IV. *De Arboribus, fructibus, & herbis medicis*: 156

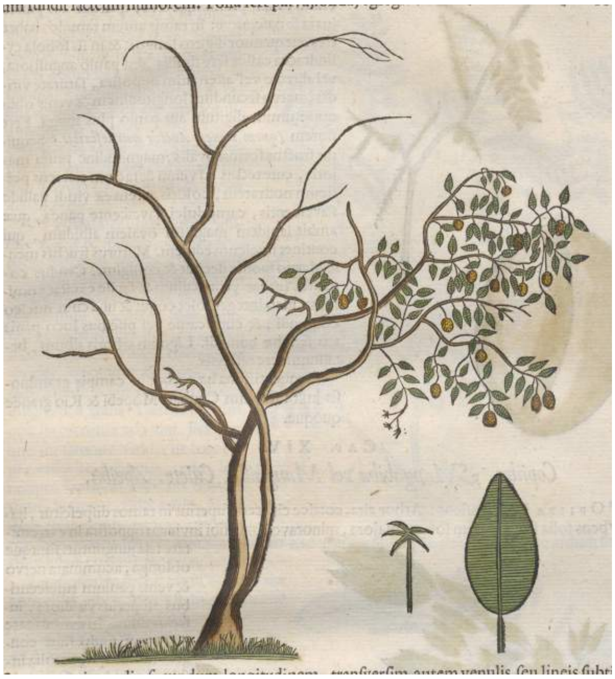

H. speciosa in the HNB (Marcgrave 1648: 122)

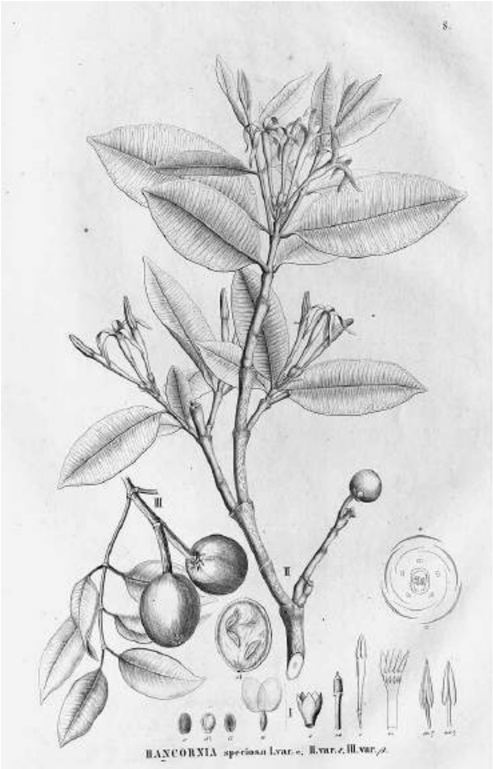

Engraving of *H. speciosa* in Martius, C.F.P. von, Eichler, A.G., Urban, I., *Flora Brasiliensis* (1840-1906). Vol 6 (1): 8

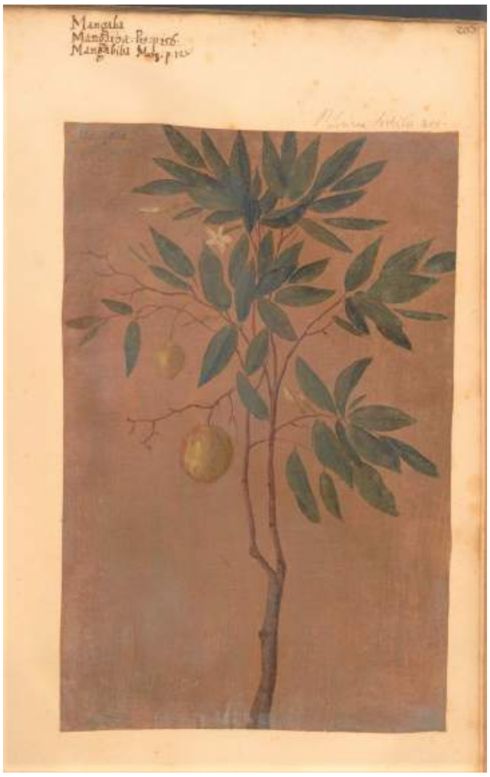

H. speciosa in the *Theatrum Rerum Naturalium* (f. 203)

Historia Naturalis & Medica Piso, 1658 Page number 158b

Vernacular name(s) Cassia fistula (fruit of Tapyracoaynana)

Species Cassia grandis L.f.

Family Fabaceae

Presence in the HNB Yes

Marcgrave (1648) 134 (only description)

Piso (1648) 80 (only description)

Notes

There is no information about the provenance of this pod in the IURNM. Piso could have had the pod, but considering its large length (up to 60 cm long when mature) (https://floradobrasil.jbrj.gov.br/FB82791), this was not drawn at scale -unlike other plant parts he kept. Piso, who partly copied the text from Marcgrave, does not mention the size of these pods; as opposed to Marcgrave who does it: "when mature [the pods], each one measure around two feet long [c. 60 cm] and five fingers wide". These pods are hanging from the trees of *C. grandis* in Ekchout's portrait of the ¨Tarairiú woman¨.

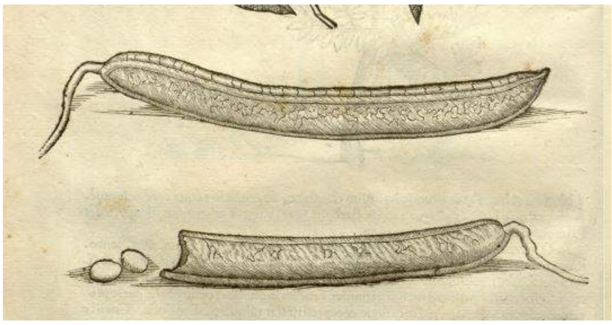

IV. *De Arboribus, fructibus, & herbis medicis*: 158b

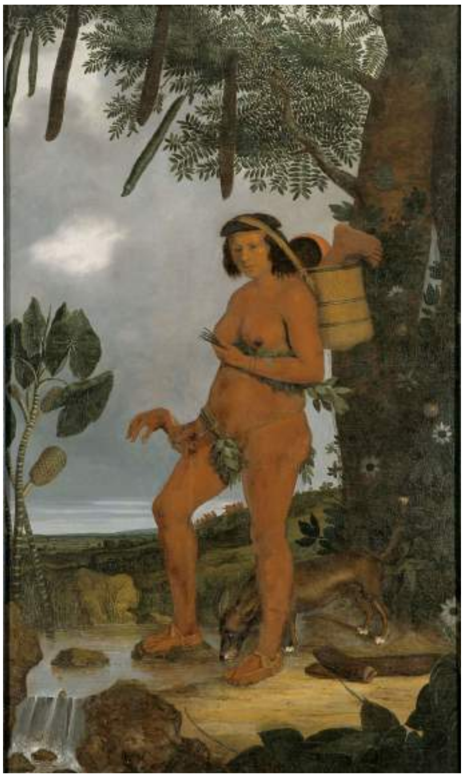

C. grandis tree with hanging pods in the background, In "Tarairiú woman" by Eckhout, 1641 (National Museum of Denmark)

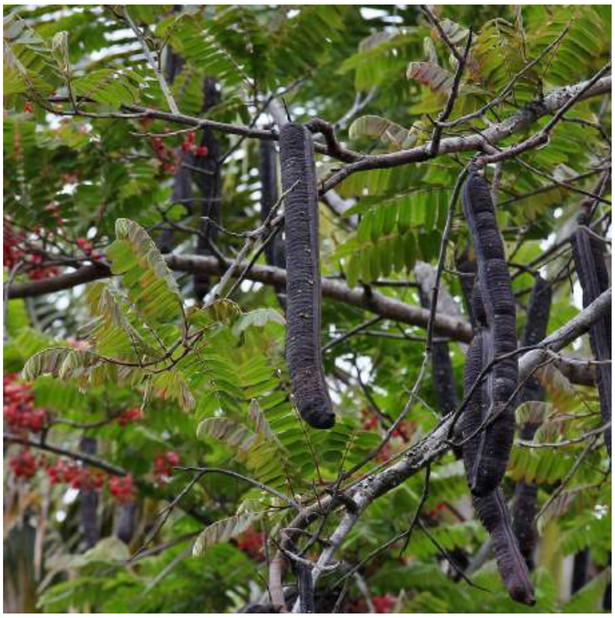

"*Cassia grandis*" by Mauricio Mercadante, in Bahia, Brazil (CC BY-NC-SA 2.0)

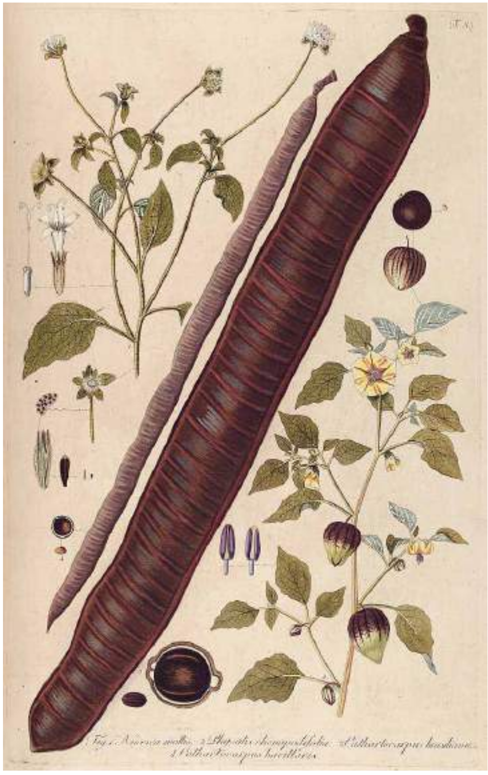

Jacquin, N.J. von, *Fragmenta botanica, figuris coloratis illustrata* (1809: 85)
Missouri Botanical Garden, St. Louis, U.S.A.

Historia Naturalis & Medica Piso, 1658 Page number 159a

Vernacular name(s) Pinoguaçu Mas

Species Carica papaya L.

Family Caricaceae

Presence in the HNB Yes

Marcgrave (1648) 103a (different woodcut)

Piso (1648)

Notes

A very similar woodcut was used in the *Exoticorum Libri Decem* (1605: 42) and the *Curae Posteriores* by Clusius (1611: 80). The plant was called "Mamoeira mas" (*C. papaya* male), represented together with "Mamoeira fem" (*C. papaya* female). De Laet (1633: 563, 1640: 498) mentioned this species in his books, but he only used the woodcut of the female plant, which he later reused for Marcgrave (1648: 103). The botanists distinguished two separate sexes in this species (dioecious), although they missed a third one: the hermaphrodite (male and female sexual organs in the same flower -trioecious). The so-considered "male" plants by the scholars, were hermaphrodites. Otherwise, they would not bear fruit, as Marcgrave indicated in the text, and as shown in the figure first published by Clusius.

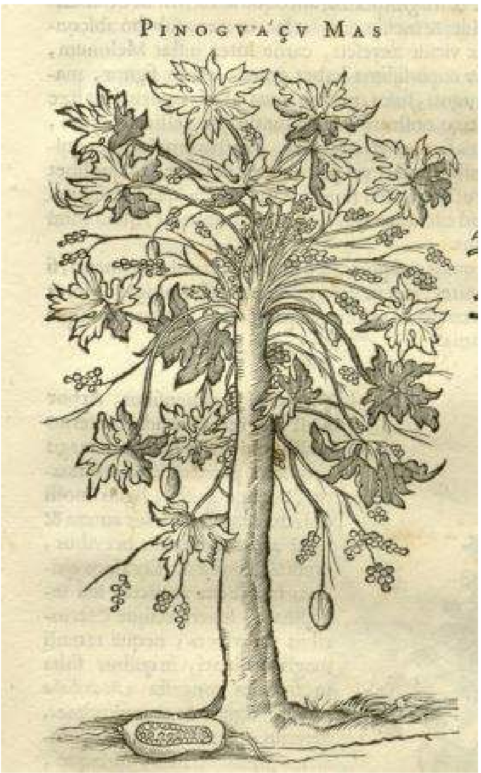

IV. *De Arboribus, fructibus, & herbis medicis*: 159a

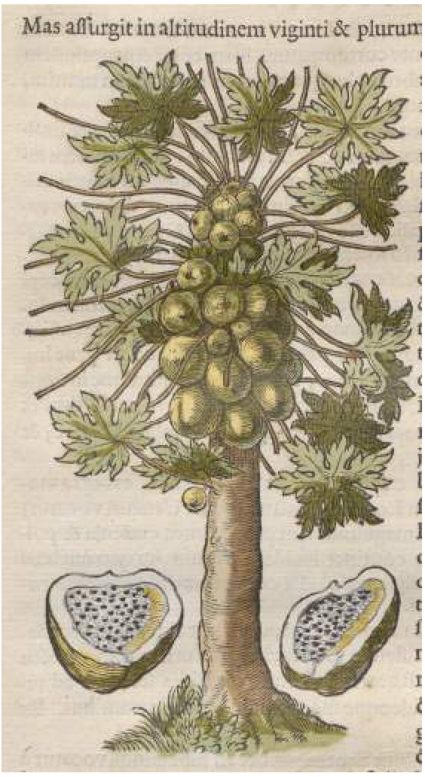

Carica papaya female plant in the HNB (Marcgrave 1648: 103a). Woodcut also used by De Laet (1633: 563, 1640: 498)

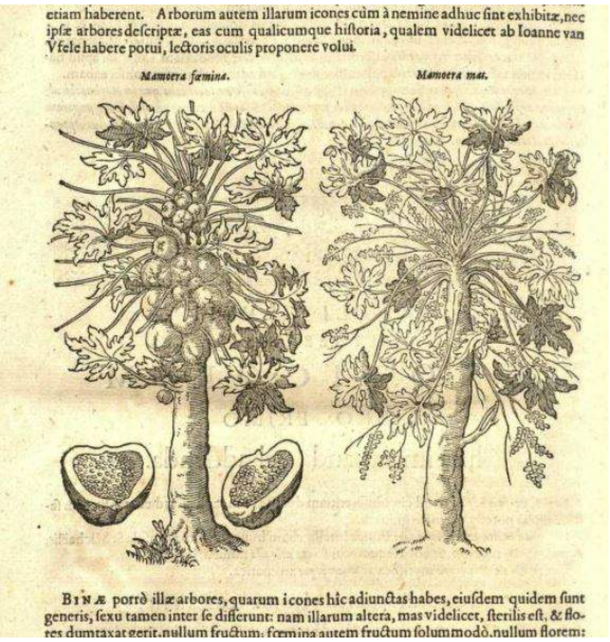

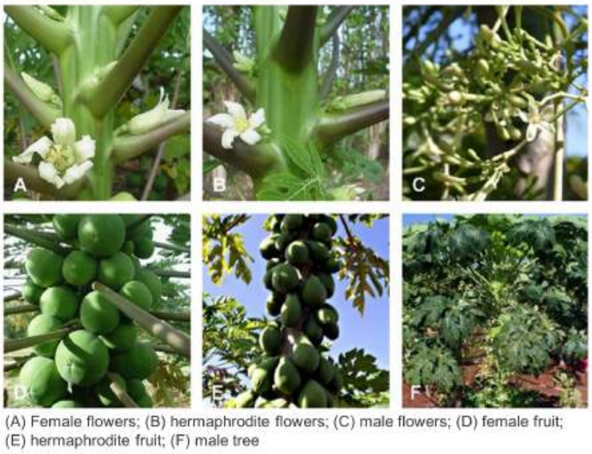

C. papaya, female and hermaphrodite plant, in *Exoticorum Libri Decem,* by Clusius (1605: 42)

Sex in *C. papaya*. Figure retrieved from: Ming R, et al., (2007) *Sex determination in papaya*, Semin Cell Dev Biol, doi:10.1016/j.semcdb.2006.11.013

Historia Naturalis & Medica Piso, 1658 Page number 163

Vernacular name(s) Tatai-iba

Species Maclura tinctoria (L.) D.Don ex Steud.

Family Moraceae

Presence in the HNB Yes

Marcgrave (1648) 119a, 119b

Piso (1648)

Notes

The woodcut in the IURNM is a slightly modified version of the woodcut in the HNB. Piso often combined the images of branches with a trunk, likely to portray the habit of trees in his compositions. The inflorescence of this species was not described by any of the scholars. Marcgrave (1648: 119) stated that this tree grew in the forests, thus perhaps this plant was not that abundant in its immediate surroundings to collect and draw the flowers. The edible fruit is widely described, as well as its wood due to its high quality.

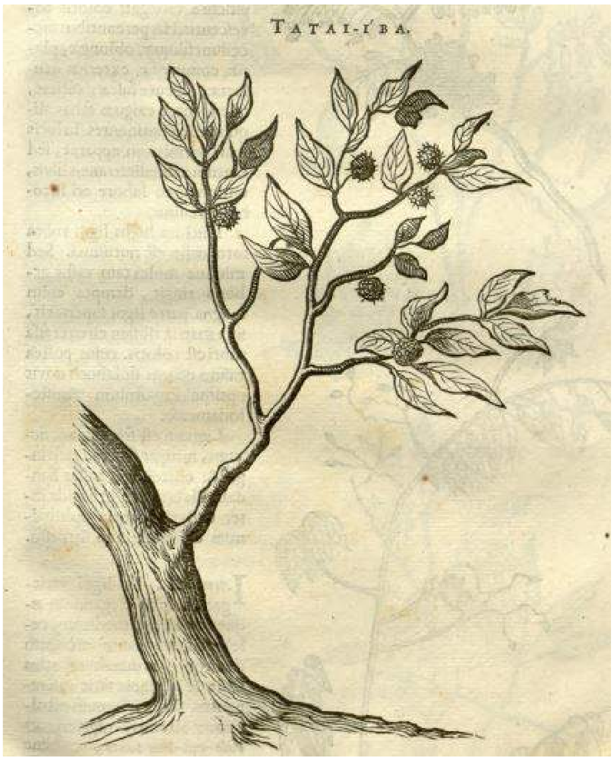

IV. *De Arboribus, fructibus, & herbis medicis*: 163

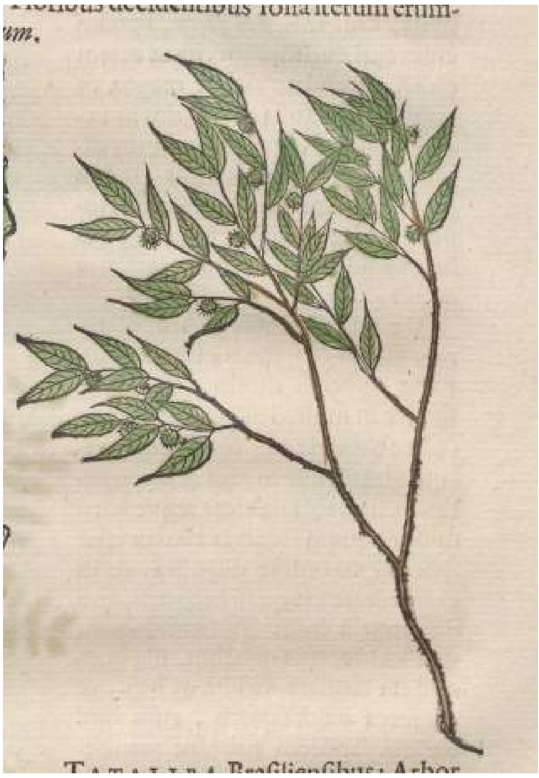

M. tinctoria branch in the HNB (Marcgrave 1648: 119b)

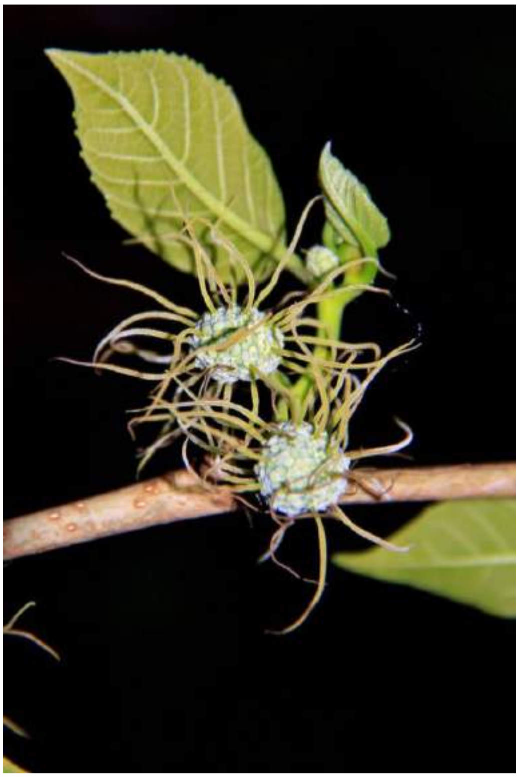

Fruits. "Amora-branca - *Maclura tinctoria* by Marcelo_Kuhlmann (CC BY-NC-SA 2.0)

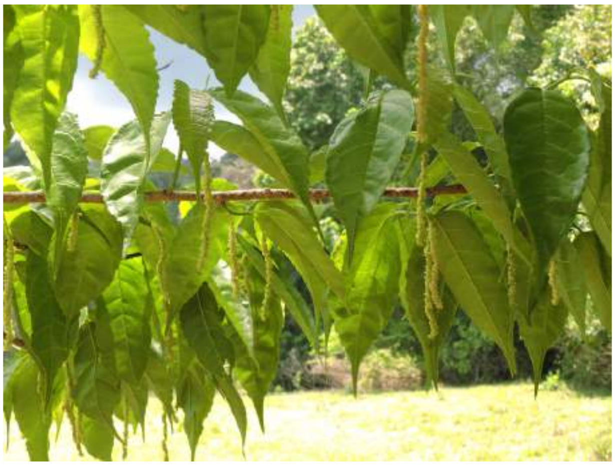

Flowering branch. "*Maclura tinctoria*" by Barry Hammel (CC BY-NC-SA 2.0)

Historia Naturalis & Medica Piso, 1658 Page number 166

Vernacular name(s) Iabotapita

Species Ouratea caudata Engl.

Family Ochnaceae

Presence in the HNB Yes

Marcgrave (1648) 101a (slightly different woodcut)

Piso (1648)

Notes

Piso ordered to make a woodcut based on the image in the HNB. In this new woodcut, the fruit that is drawn separately in the HNB (likely drawn by Marcgrave), is attached to the marginal end of the leaflet (on the left). In addition, the flower petals are more defined than in the HNB, and they seem to be made after the solitary flower depicted in the HNB. Hence, Piso combined in one figure the different elements portrayed by Marcgrave, plus he attached it to a trunk (see previous entry).

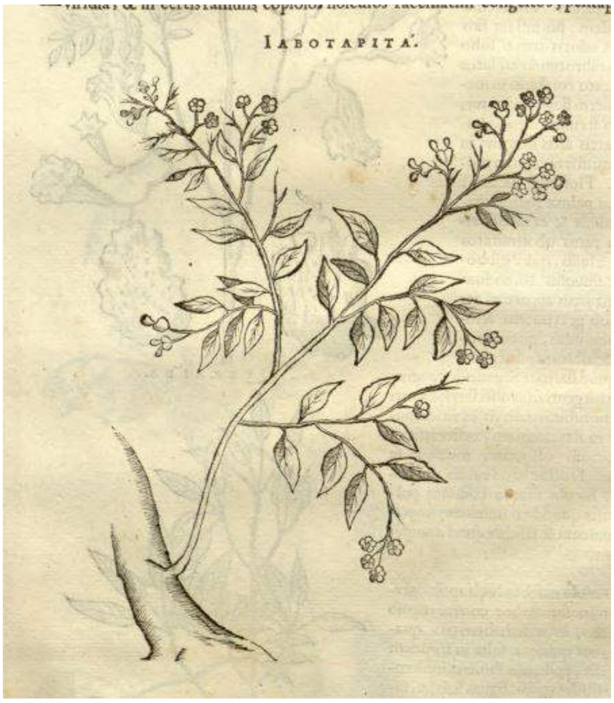

IV. *De Arboribus, fructibus, & herbis medicis*: 166

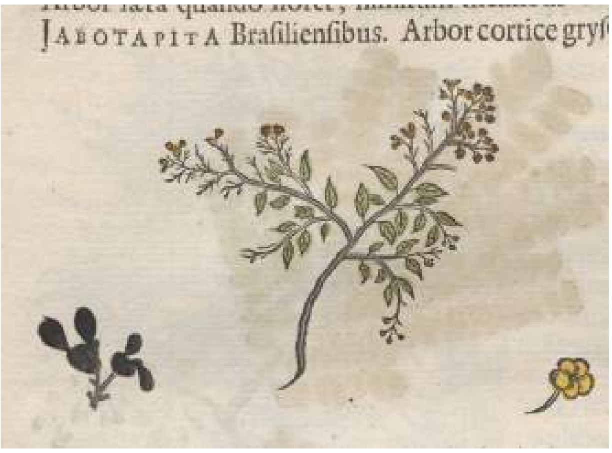

O. caudata in the HNB (Marcgrave 1648: 101a)

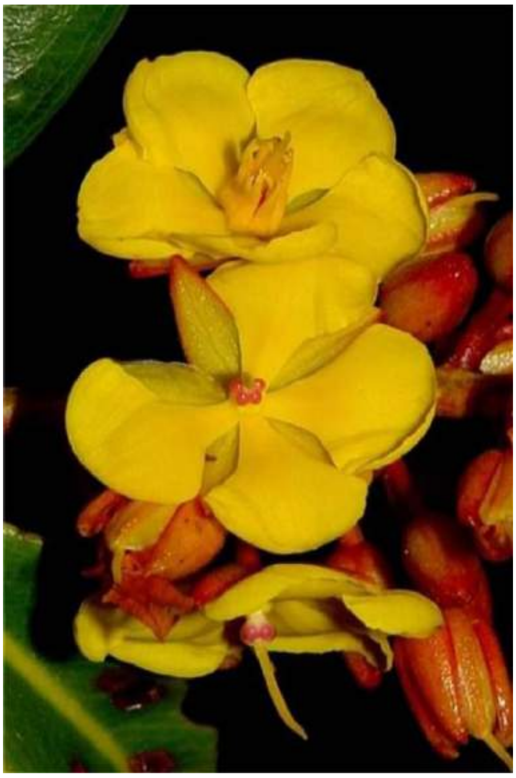

"Flowers showing detail of ovaries of *O. caudata"*
Cláudio Nicoletti de Fraga, Instituto de Pesquisas
Jardim Botânico do Rio de Janeiro

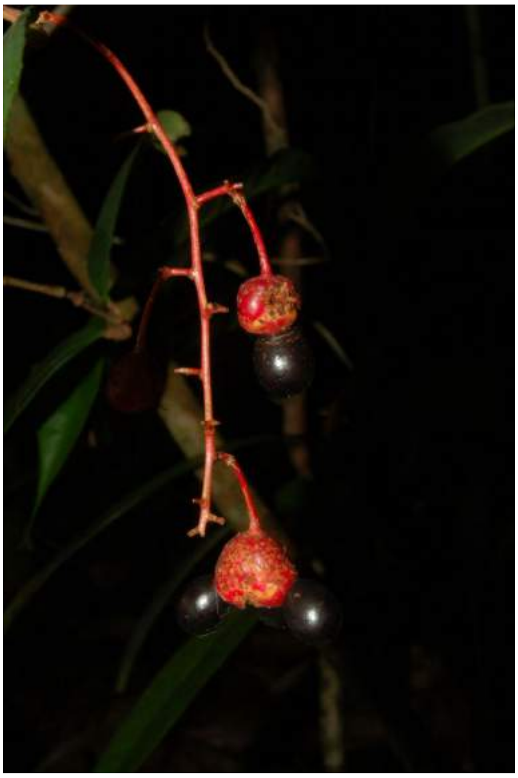

Ouratea - Specimen: Aona L.Y.S. 2474
Lidyanne Aona © RBG Kew

Historia Naturalis & Medica Piso, 1658 Page number 167

Vernacular name(s) Umbu

Species Spondias tuberosa L.

Family Anacardiaceae

Presence in the HNB Yes

Marcgrave (1648) 108 (slightly different woodcut)

Piso (1648) 78 (slightly different woodcut)

Notes

As often occurs with trees in the HNB, Piso added a trunk. In this case, he created a new woodcut that shows the flowers as in the HNB, he added two fruits more, and he depicted the xylopodia. The xylopodia is the root-tuber system of *S. tuberosa*. The woodcut in the HNB looks moderately similar to the *Theatrum* illustration (f. 261), although the tuber is placed in the foreground and it shows a longitudinal cut. The underground tubers are of great importance because they store water and nutrients as an adaptive strategy for the dry seasons in the Caatinga ecosystem in Brazil (Cavalcanti et al., 2010). Nowadays, scientists are alarmed that this endemic species it is threatened by extinction, and urge to assess its population and implement conservation measures (Mertens et al. 2016).

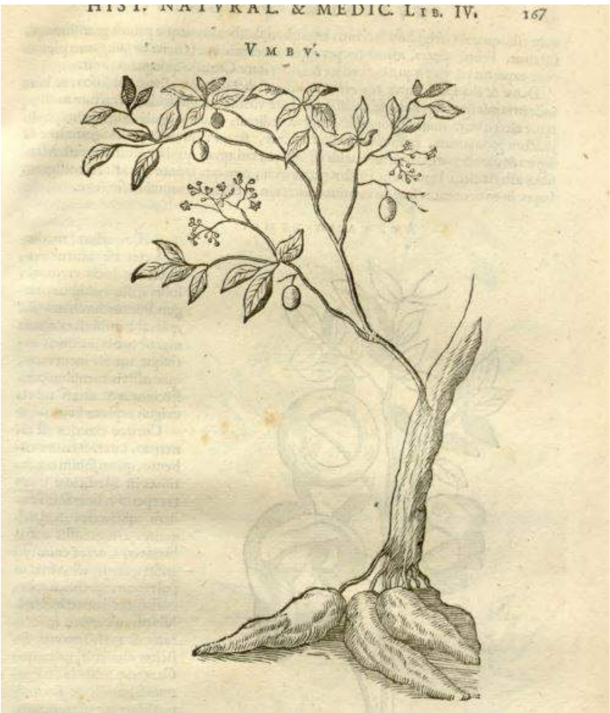

IV. *De Arboribus, fructibus, & herbis medicis*: 167

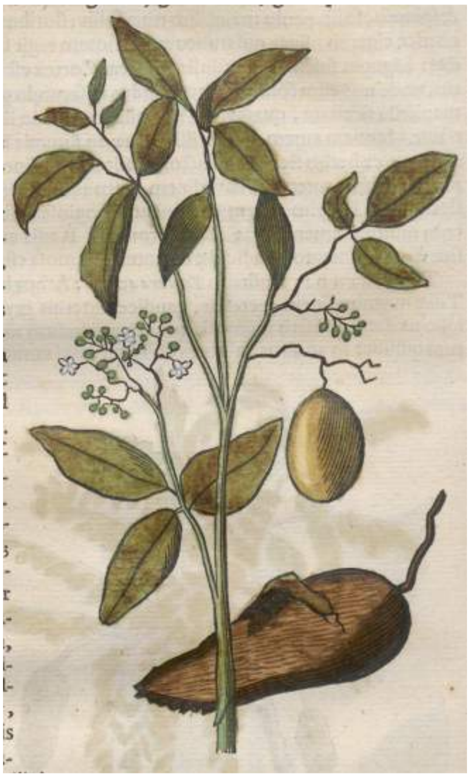

S. tuberosa in the HNB (Marcgrave 1648: 108)

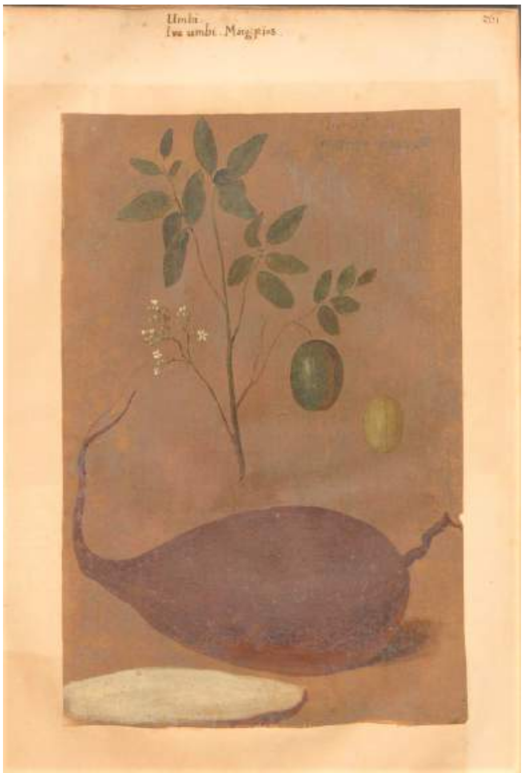

S. tuberosa in the *Theatrum Rerum Naturalium* (f. 261)

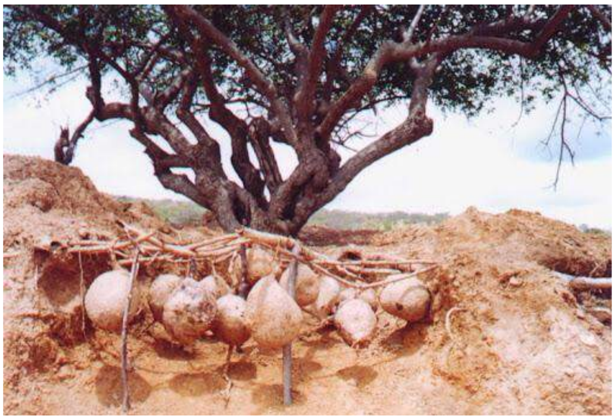

S. tuberosa xylopodia. Photo by Nilton de Brito Cavalcanti, 2009, retrieved from Empresa Brasileira de Pesquisa Agropecuária - EMBRAPA

Historia Naturalis & Medica Piso, 1658 Page number 174

Vernacular name(s) Umari

Species Geoffroea spinosa Jacq.

Family Fabaceae

Presence in the HNB Yes

Marcgrave (1648) 121a (slightly different woodcut)

Piso (1648)

Notes

The woodcut is very similar to the one depicted in the HNB, but here, again, Piso created a composition. The leafy branches of *G. spinosa* are directly attached to a trunk and, instead of one large fruit in the foreground, he depicted several and smaller fruits on the ground next to the plant. As with *Maclura tinctoria*, the flowers are not described by any of the authors. Marcgrave (1648: 121) commented on the abundance of these trees in the grasslands, next to the river Cunhao Mopebi and the Rio Grande [current Rio Grande do Norte state] (Almeida 2016). Hence, it is likely that Marcgrave encountered this plant during one of his expeditions and did not have the chance to observe it flowering.

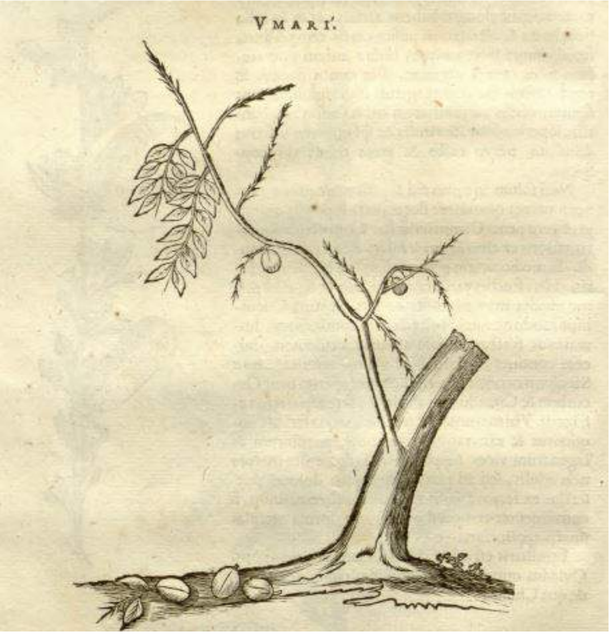

IV. *De Arboribus, fructibus, & herbis medicis*: 174

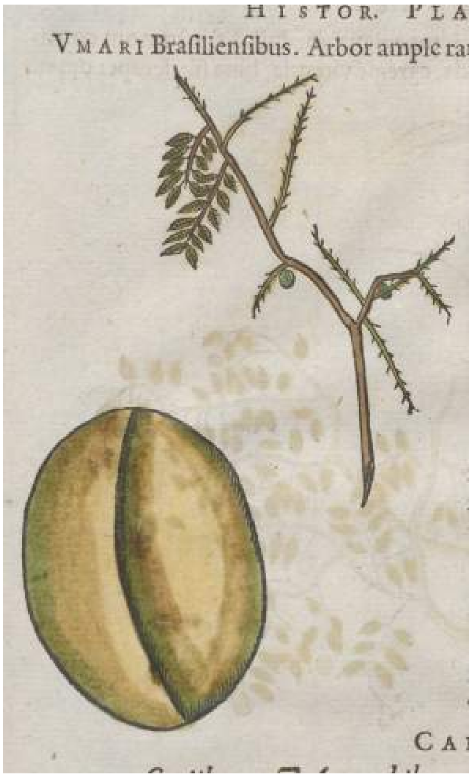

G. spinosa in the HNB (Marcgrave 1648: 121a)

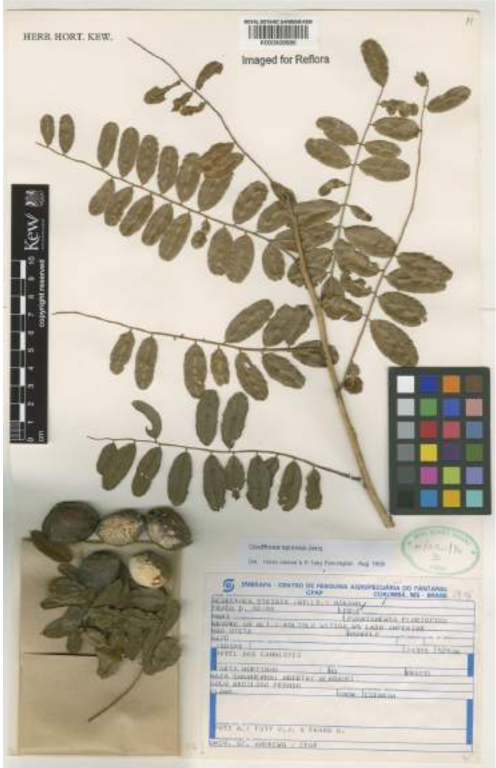

G. spinosa specimen from Kew's Herbarium - K000908686
Retrieved from Plants of the World Online

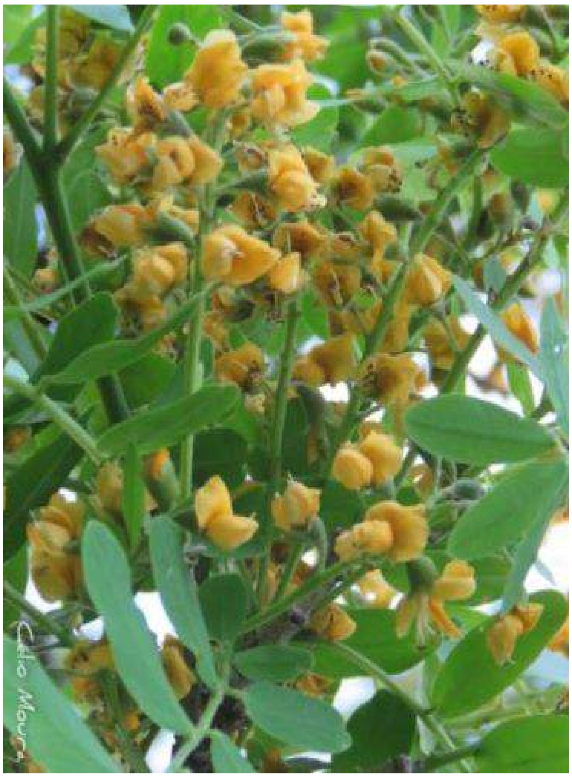

Flowers of *Geoffroea spinosa*, observed in Brazil by Célio Moura Neto (CC BY 4.0)

Historia Naturalis & Medica Piso, 1658 Page number 175a

Vernacular name(s) Saamouna

Species Ceiba erianthos (Cav.) K.Schum.

Family Malvaceae

Presence in the HNB Yes

Marcgrave (1648)

Piso (1648) 81a (slightly different woodcut)

Notes

This woodcut is a modified version of the woodcut published in Piso (1648). The image here includes a spiny trunk, widened in the middle part, as described by Piso in the HNB. In addition, one more leaf has been added. Neither flowers nor fruits are depicted, although the oblong pods with red seeds are mentioned. Piso indicated that this tree is found in the inland forests and that the exude from the crashed thorns is used to heal the inflammation of the eyes. This proves the physician's travels to the interior accompanied by Indigenous peoples, from whom he learned this medicinal use.

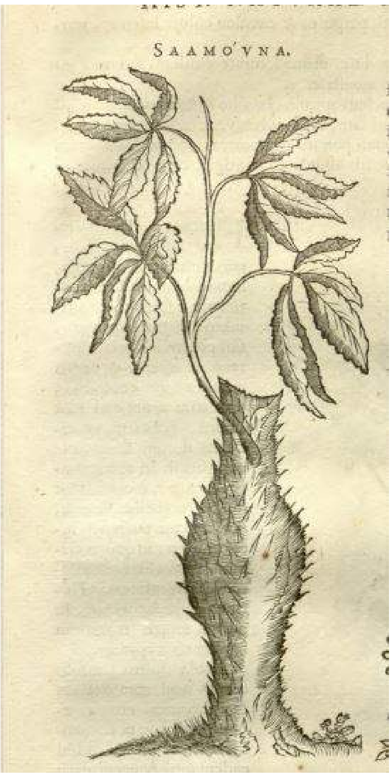

IV. *De Arboribus, fructibus, & herbis medicis*: 175a

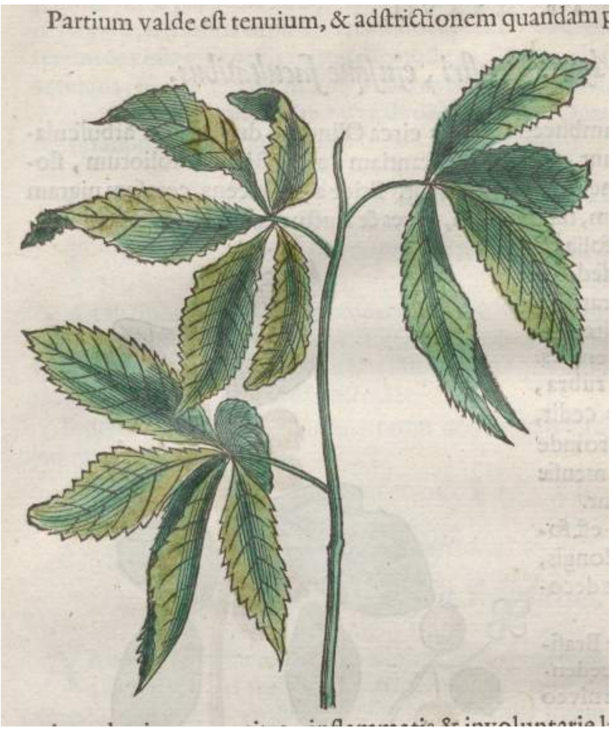

Ceiba erianthos in the HNB (Piso 1648: 81a)

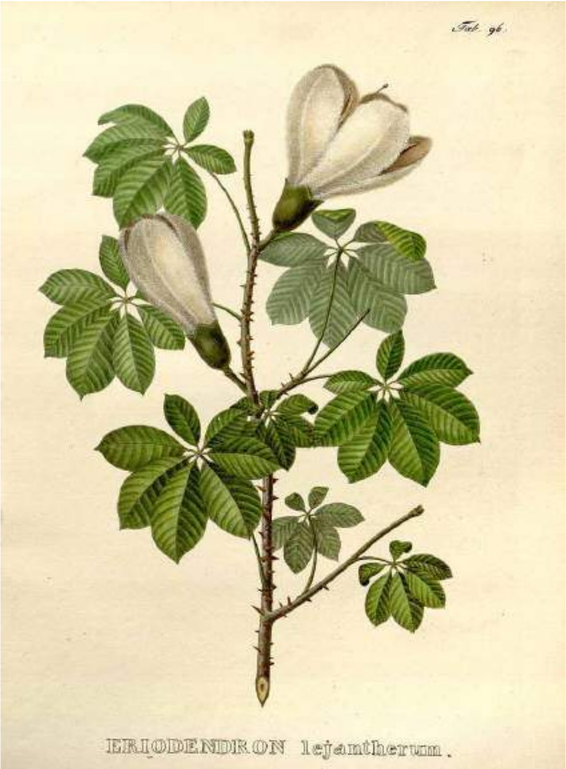

C. erianthos, In: Martius, C.P.F. von, *Nova genera et species plantarum Brasiliensium* (1823: vol. I, p. 96)
Retrieved from www.plantillustrations.org

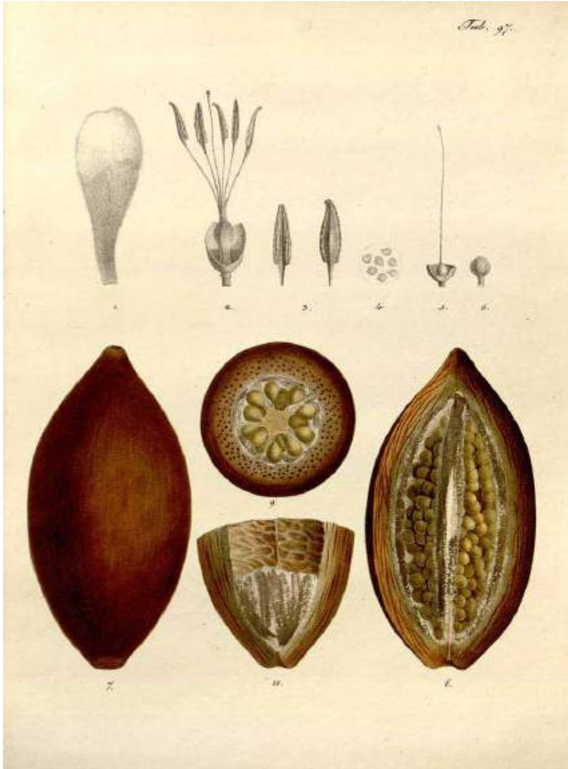

C. erianthos, In: Martius, C.P.F. von, *Nova genera et species plantarum Brasiliensium* (1823: vol. I, p. 97)
Retrieved from www.plantillustrations.org

Historia Naturalis & Medica Piso, 1658 Page number 179a

Vernacular name(s) Munduy-guaçu. Noz catartica americana

Species <u>Jatropha curcas L.</u>

Family Euphorbiaceae

Presence in the HNB Yes

Marcgrave (1648) 96b (different woodcut)

Piso (1648) 83 (different woodcut)

Notes

This woodcut differs from the image in the HNB, and it is slightly similar to the illustration in the *Theatrum* (f. 199). It depicts ring-like scars in the stem, in contrast to the woodcut in the HNB, which includes spines. The *Theatrum* illustration lacks both scars and spines. Because of the similarities with the oil painting, unless the physician visited the Elector of Brandenburg, it is possible that Piso had access to the drawing that was used as a model for the oil painting (already in the former Prussian state since 1652, although not bound yet within the collection named *Theatrum Rerum Naturalium*).

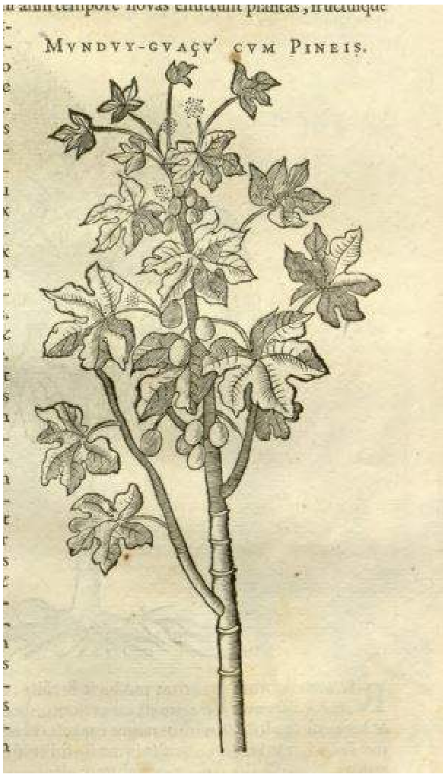

IV. *De Arboribus, fructibus, & herbis medicis*: 179a

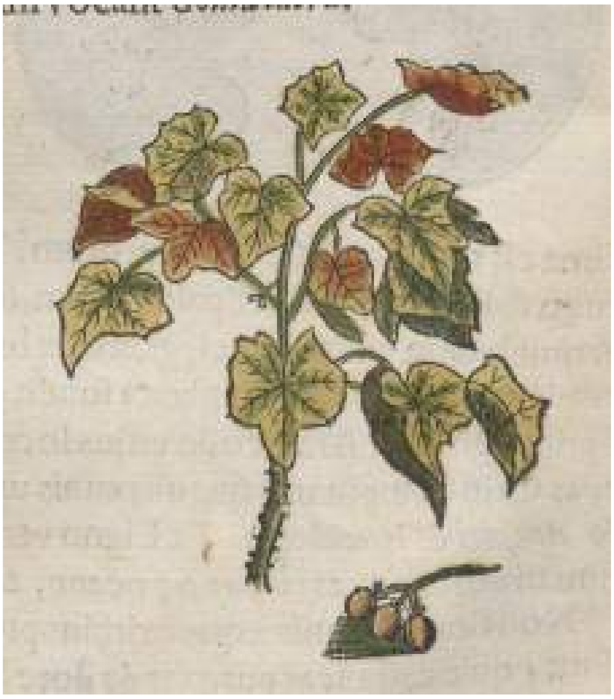

Jatropha curcas in the HNB (Marcgrave 1648: 96b)

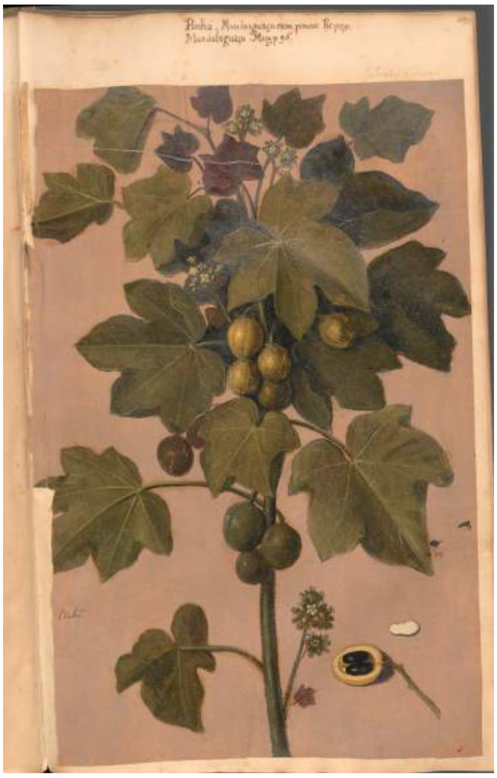

J. curcas in the *Theatrum Rerum Naturalium* (f. 199)

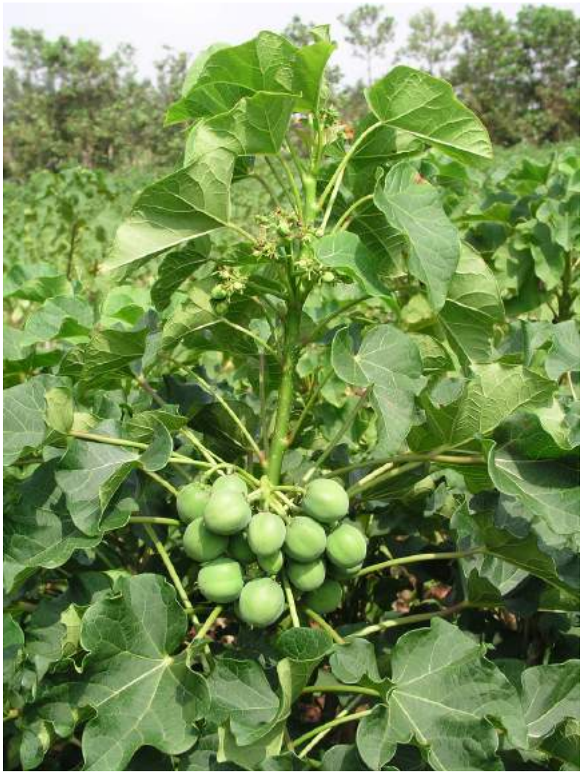

Shrub with fruits and flowers. "*Jatropha curcas*" by wan_hong (CC BY-NC-SA 2.0)

Historia Naturalis & Medica Piso, 1658 Page number 180

Vernacular name(s) Nhambu guaçu. Ricino americano

Species Ricinus communis L.

Family Euphorbiaceae

Presence in the HNB Yes

Marcgrave (1648) 77 (only description)

Piso (1648) 91 (only description)

Notes

The sketch in the *Misc. Cleyeri* depicts this species, but it does not bear a strong resemblance with the woodcut in the IURNM. This image resembles the woodcut entitled *Ricinus*, depicted in the *Opera* of the Syrian physician Yuhanna ibn Masawaih (also known as Ioannis Mesuae) from the ninth century. It is slightly similar to Matthioli's image for the same plant on his *Discorsi on Dioscorides*. Europeans knew this African plant before they traveled to the Americas because it was described in Arabic herbals. Piso (1658), stated that he ordered to design the nuts with the shell at its natural size (bottom right corner). Hence, the woodcut is a composite image that was created by copying the image from an existent woodcut -published in an older treatise- together with an original drawing made at scale from a Brazilian fruit.

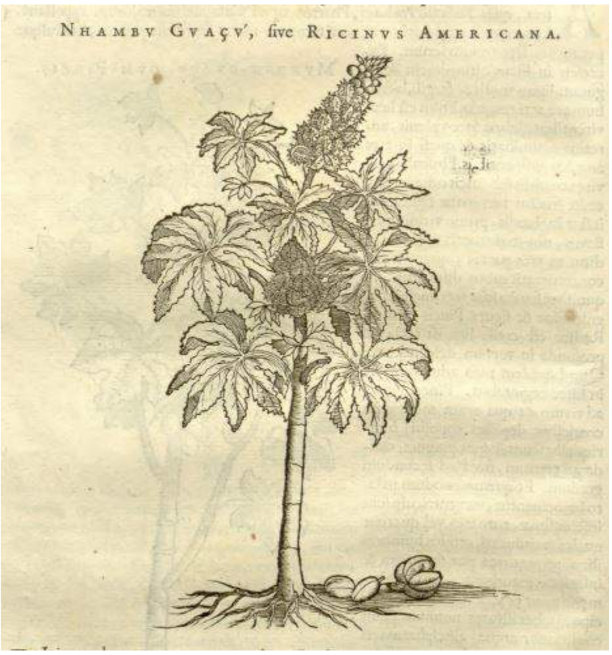

IV. *De Arboribus, fructibus, & herbis medicis*: 180

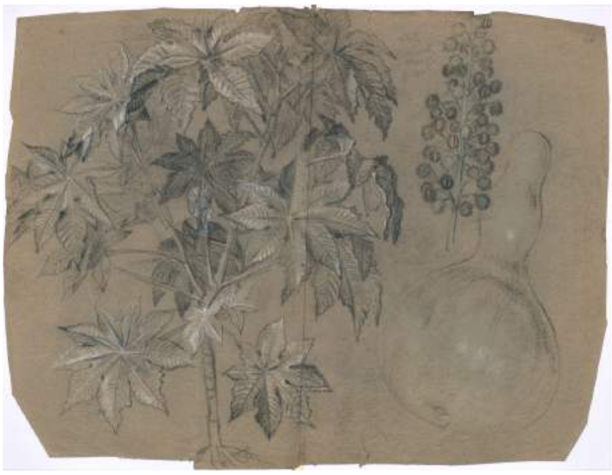

Branch with leaves, infructescence of *Ricinus communis, and fruit of Lagenaria siceraria* in the *Miscellanea Cleyeri* c.1637-44: 65v and 66r

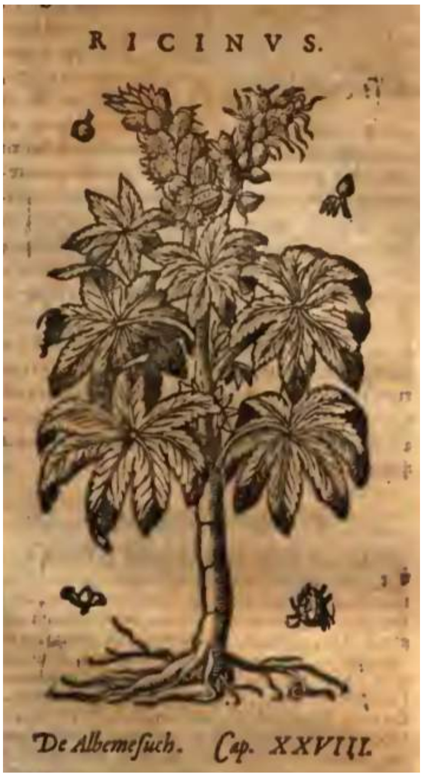

*Damasceni medici clarissimi Opera de medicamentorum.*by Ioannis Mesua (1602: 79)

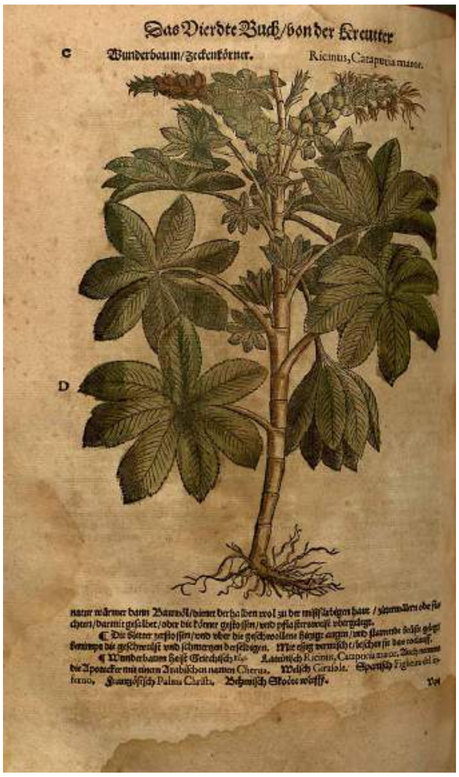

New Kreuterbuch by Pietro Andrea Matthioli (1563: 526)

Historia Naturalis & Medica Piso, 1658 Page number 186

Vernacular name(s) Aminiiu

Species Gossypium barbadense L.

Family Malvaceae

Presence in the HNB Yes

Marcgrave (1648) 59 (only description)

Piso (1648)

Notes

This woodcut bears a strong resemblance to the image of *Gossypium arboreum* depicted in the work on Egyptian plants by Venetian botanist Prospero Alpini (1592: 29, 1640: 71). This matches with the identification of the woodcut by botanist W.E. Safford, who attributed it to *G. arboreum* (Pickel 2008: 142). Images of the cotton plant also circulated in the herbals of Matthioli or Fuchs.

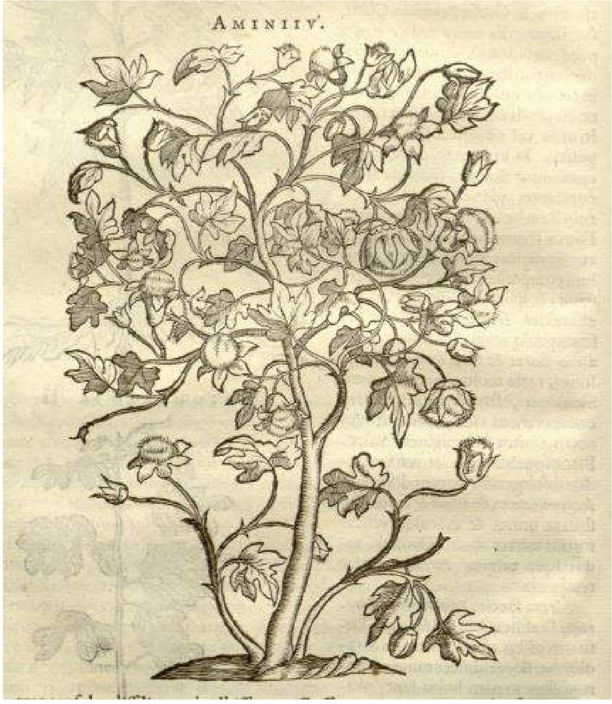

IV. *De Arboribus, fructibus, & herbis medicis*: 186

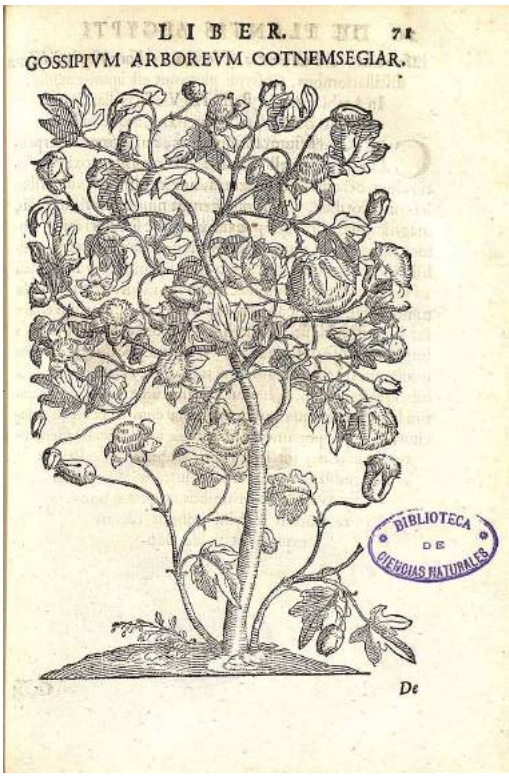

Woodcut of *Gossypium arboreum* in *De Plantis Aegypti liber* by Alpini (1640: 71). Real Jardín Botánico, Madrid, Spain

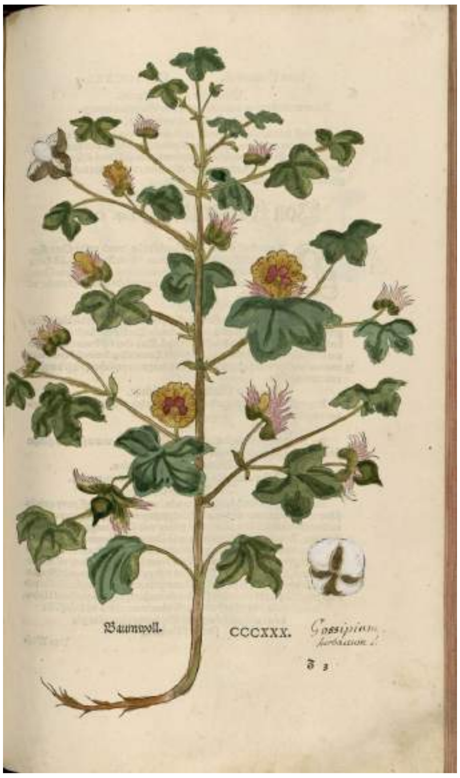

Cotton plant in *New Kreüterbuch* by Fuchs (1543: 330). Retrieved from Plantillustrations.org

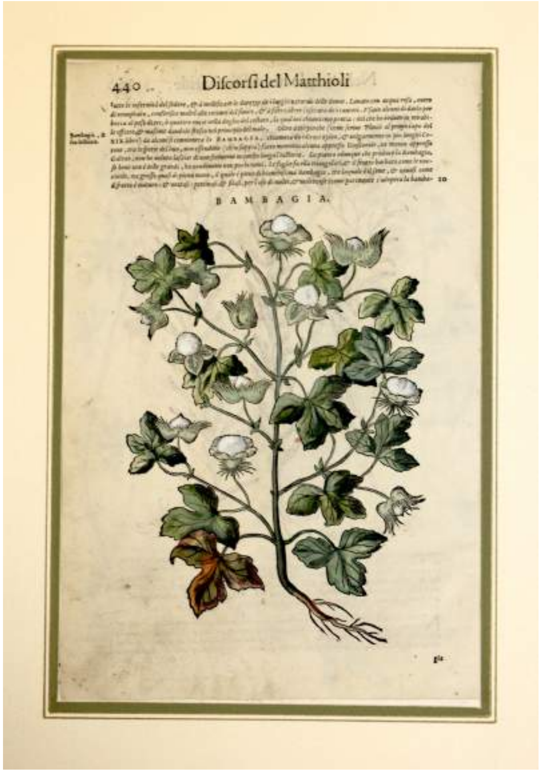

Cotton plant in *Discorsi on Dioscorides' De Materia Medica* by Pietro Andrea Matthioli (1565: 440)

Historia Naturalis & Medica Piso, 1658 Page number 187a

Vernacular name(s) Ibipitanga. Cerasus brasiliana

Species Eugenia uniflora L.

Family Myrtaceae

Presence in the HNB Yes

Marcgrave (1648) 116b, 293

Piso (1648) 121a (different woodcut)

Notes

This woodcut is a modified copy of the image depicted in the HNB for the same species (Marcgrave 1648: 293). In turn, this image bears resemblance to the oil painting in the *Theatrum* (f. 339). Piso, however, ordered to make this figure attached to a trunk, which portrays the habit of the plant. The works of de l' Obel, Dodoens, and Clusius (who shared the woodcuts published by Plantin) often depict the branches or compound leaves of trees similarly attached to trunks.

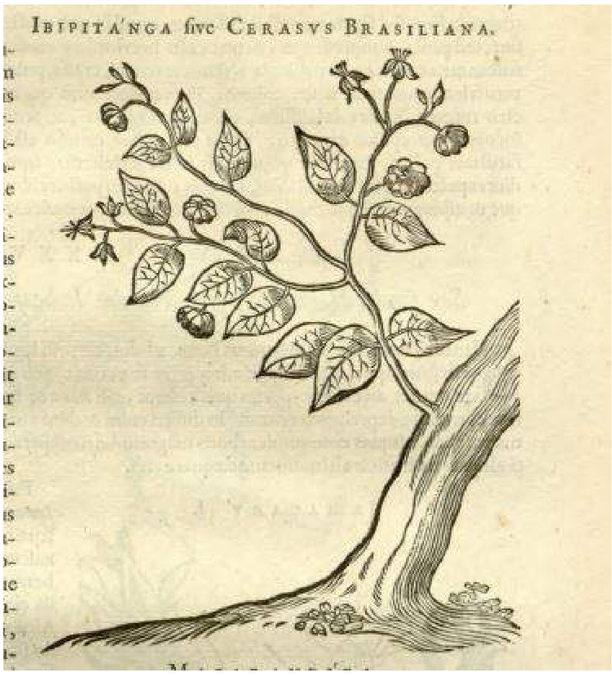

IV. *De Arboribus, fructibus, & herbis medicis*: 187a

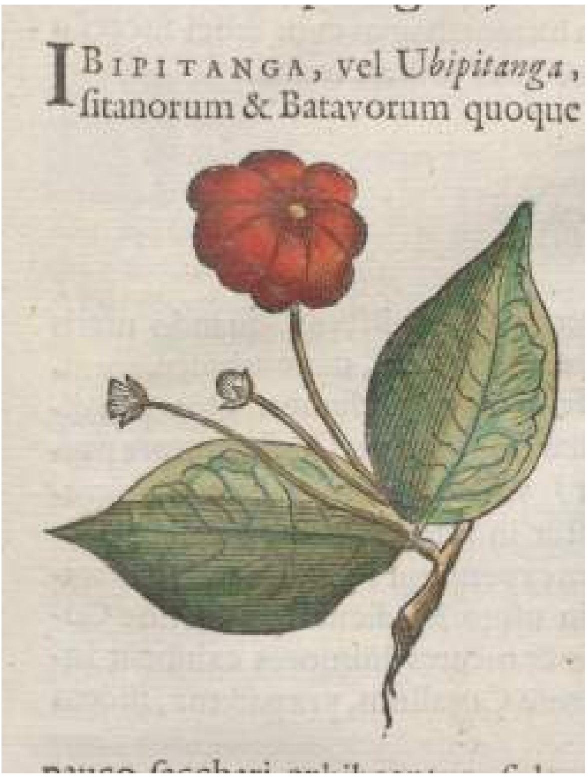

Eugenia uniflora in the HNB (Piso 1648: 121a), which is the same woodcut than in Marcgrave (1648: 116b)

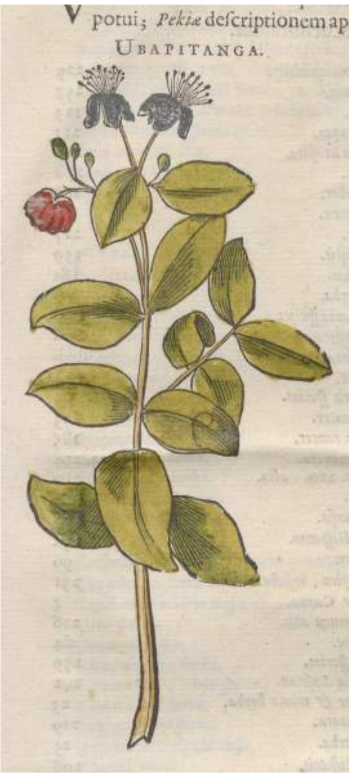

E. uniflora in the HNB (Marcgrave 1648: 293)

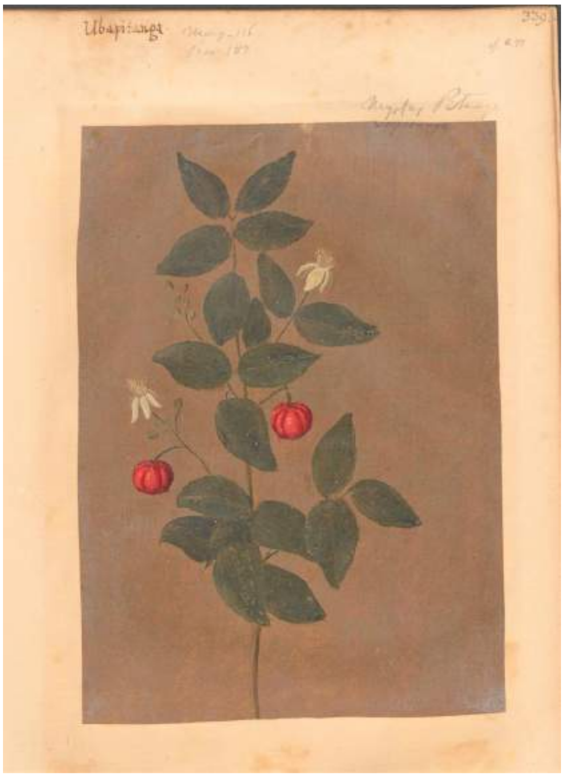

E. uniflora in the *Theatrum Rerum Naturalium* (f. 339)

Historia Naturalis & Medica Piso, 1658 Page number 187b

Vernacular name(s) Maçarandiba

Species Manilkara salzmannii (A.DC.) H.J.Lam

Family Sapotaceae

Presence in the HNB Yes

Marcgrave (1648)

Piso (1648) 120a

Notes

This woodcut is a slightly modified copy of the image depicted in the HNB; here, again, it is attached to a tree branch. According to Pickel (2008: 185), the model for the woodcut was redrawn after the description of this species. The fruits are described as cherry-like fruits, but the flowers are not described either in the HNB nor the IURNM.

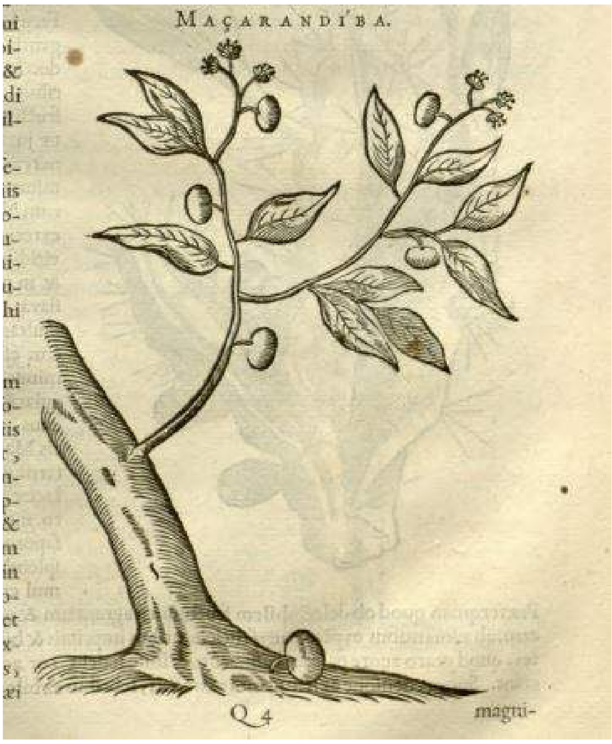

IV. *De Arboribus, fructibus, & herbis medicis*: 187b

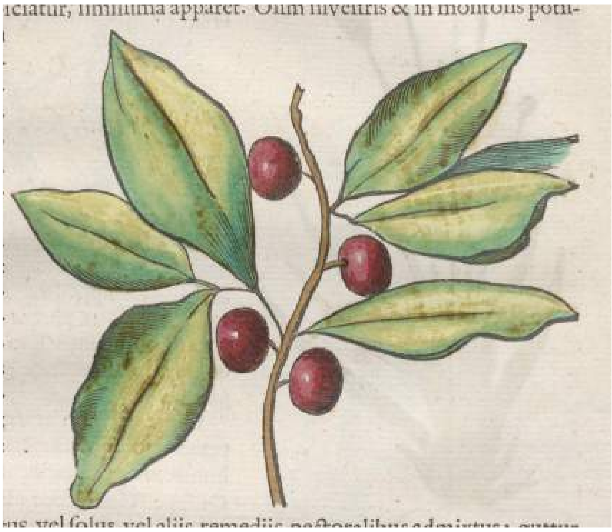

Manilkara salzmannii in the HNB (Piso 1648: 120a)

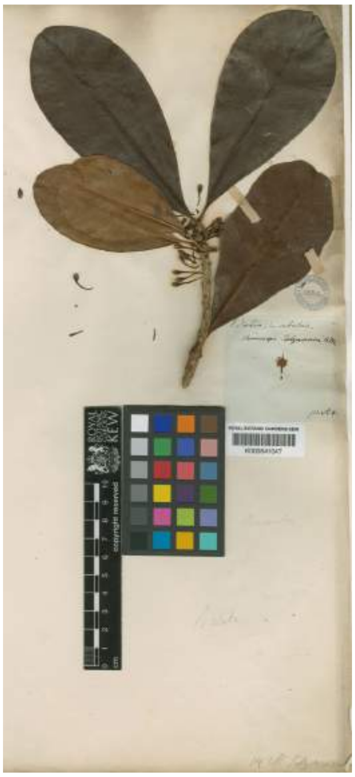

M. salzmannii specimen from Kew's Herbarium - K000641047. Retrieved from Plants of the World Online

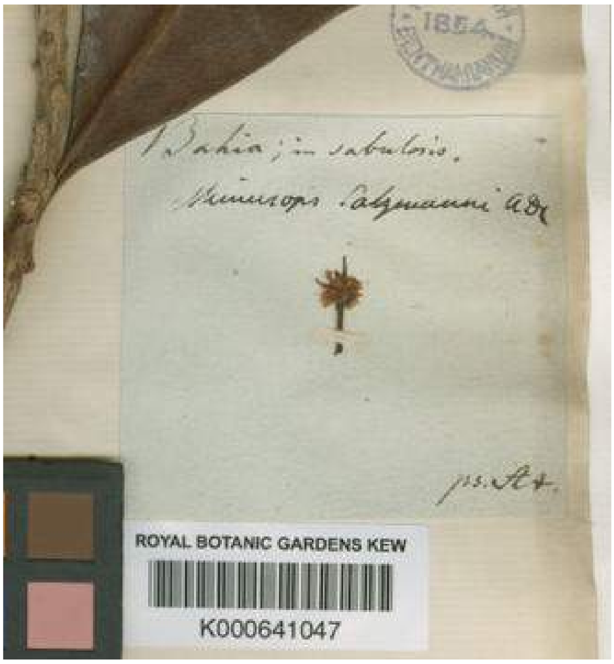

Close-up of the flower of *M. salzmannii*. Specimen from Kew's Herbarium - K000641047. Retrieved from Plants of the World Online

Historia Naturalis & Medica Piso, 1658 Page number 206

Vernacular name(s) Petume. Petum. Tabaco

Species Nicotiana tabacum L.

Family Solanaceae

Presence in the HNB Yes

Marcgrave (1648) 274 (annotations)

Piso (1648)

Notes

This image is a reproduction of the woodcut published in Monardes' chapter on tobacco (1579: 26, *Petum Latifolium*); which woodblock was later used by Clusius (1605: 309). Piso mentioned how the Europeans applied the medicinal uses of the fresh leaves of the tobacco plant to heal wounds, after observing this use by the Indigenous population. He also cited that Europeans "used the dry leaves in abuses and pleasures" (Piso 1658: 206). Interestingly, he mentioned that "chewing the dry leaves appease the tiredness and hunger of those who travel in the hinterland (*sertões*), as it happens to my journey companions and me" (Piso 1658: 207). Marcgrave does not describe the morphology of the plant, as he usually did, but he described the tool *Petumbuaba* used by the native Brazilians to inhale

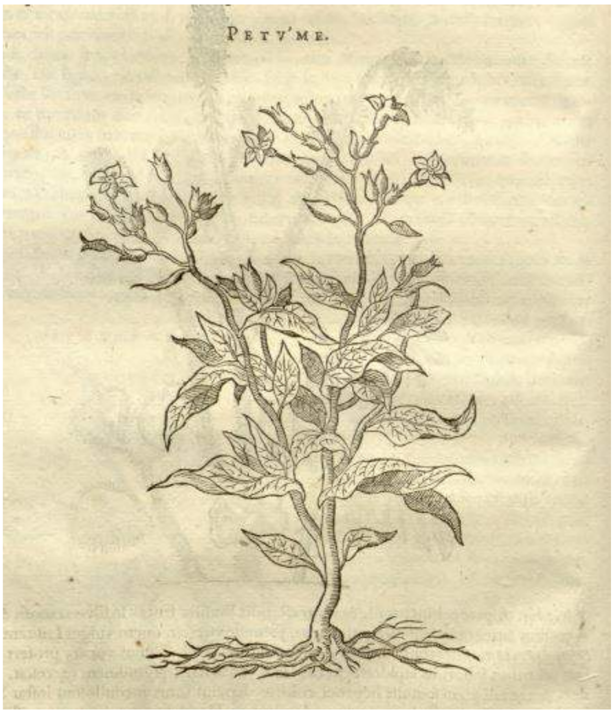

IV. *De Arboribus, fructibus, & herbis medicis*: 206

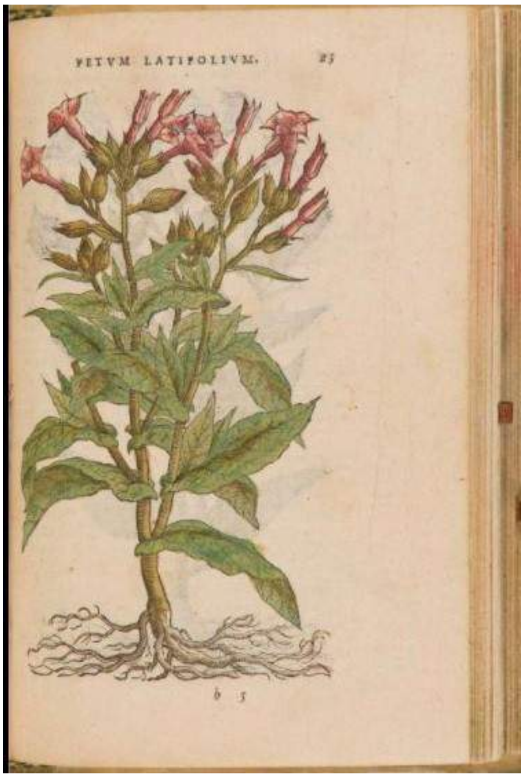

Chapter on tobacco in *Simplicium medicamentorum* by Monardes (1579: 25)

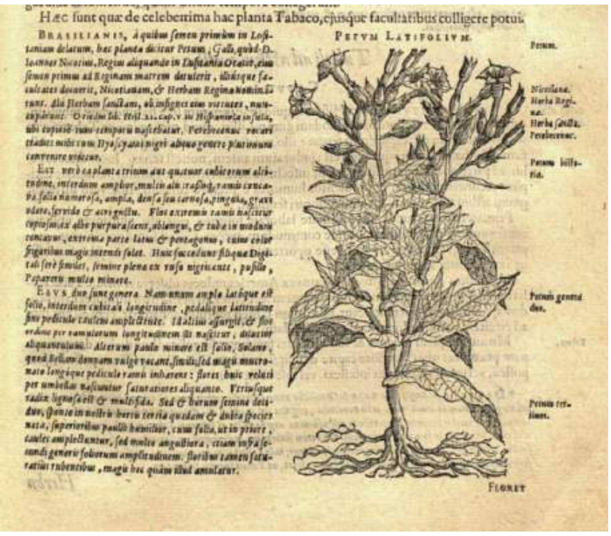

Tobacco plant, in *Exoticorum Libri Decem*, by Clusius (1605: 309)

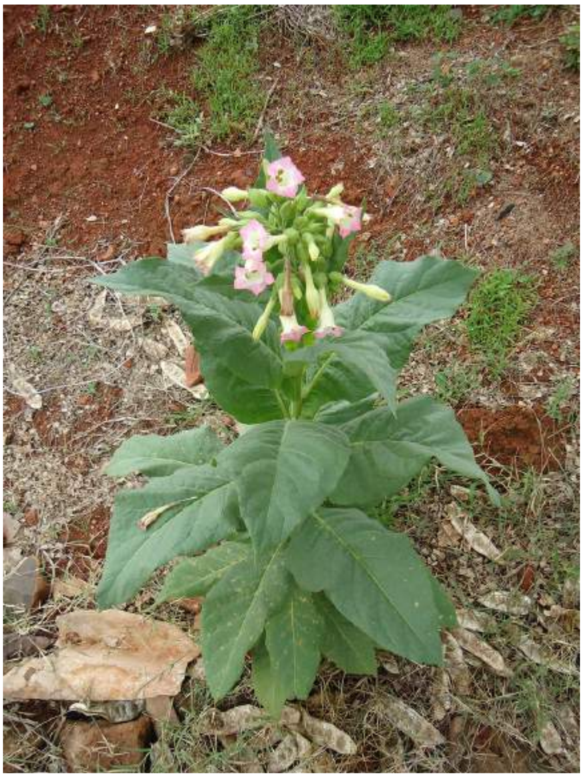

"*N. tabacum*, *fumo*", MG, Brazil, by Johannes J. Smit (CC BY-NC 2.0)

Historia Naturalis & Medica Piso, 1658 Page number 208

Vernacular name(s) Mirabilis peruana

Species Mirabilis jalapa L.

Family Nyctaginaceae

Presence in the HNB No

Marcgrave (1648)

Piso (1648)

Notes

This woodcut was made after the image entitled *Admirabilis Peruana* in *Kruydtboeck* by de L' Obel (1581: 262), which was later used in *Rariorum Plantarum* by Clusius (1601: livr. V, XC). The publishing house Plantin owned the woodblocks and printed both works. Piso copied the design depicted in these herbals for several plants, including for *M. jalapa*, which did not appear in the HNB. Notice how this woodcut is reversed from the original in de l' Obel and Clusius. Additionally, the root is attached to the stem, instead of depicted separately. It is unclear whether Piso observed this plant in Brazil. He mentioned that "it is native of the American continent, but more popular in Peru, from where it was shipped to Europe to cultivate it in the gardens" (Piso 1658: 207). He added it due to the medicinal roots.

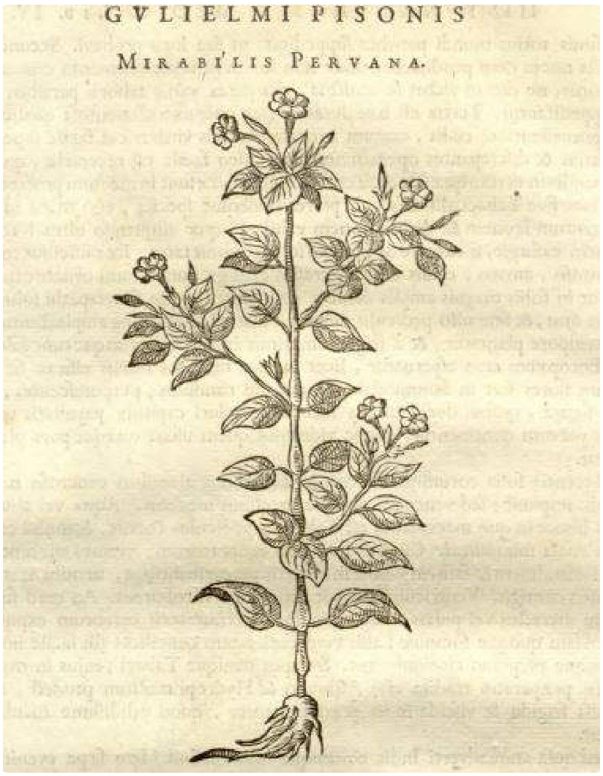

IV. *De Arboribus, fructibus, & herbis medicis*: 208

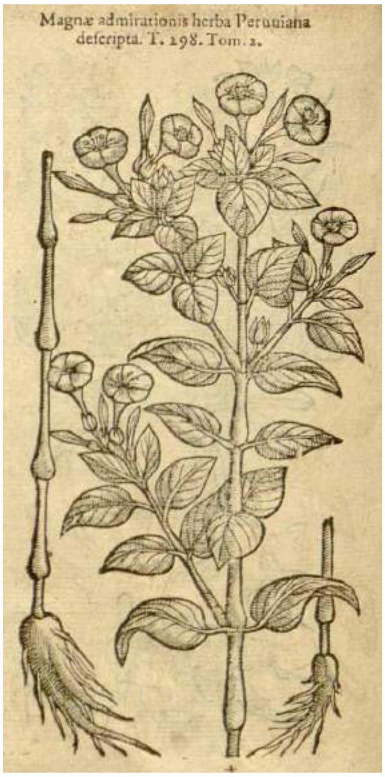

Admirabilis Peruana (*M. jalapa*) in *Kruydtboeck* by Obel (1581: vol. 2, 262)

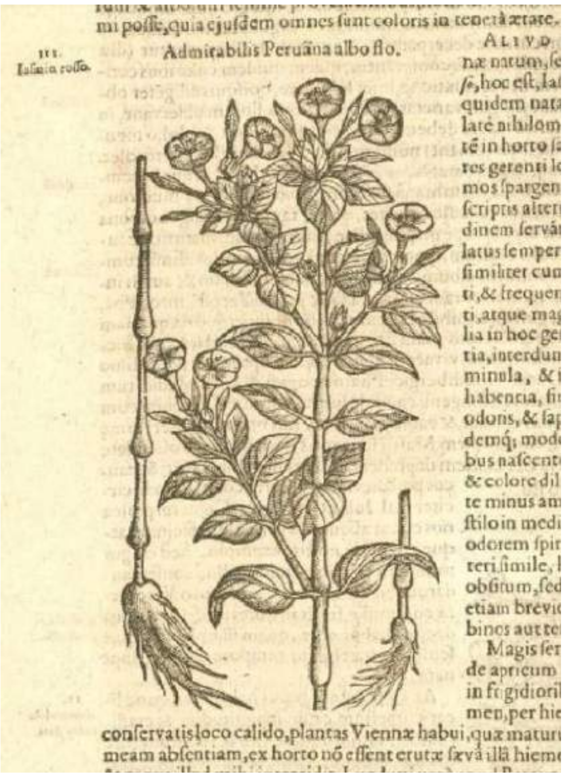

M. jalapa in *Rariorum Plantarum*, by Clusius (1601: livr. V, XC)

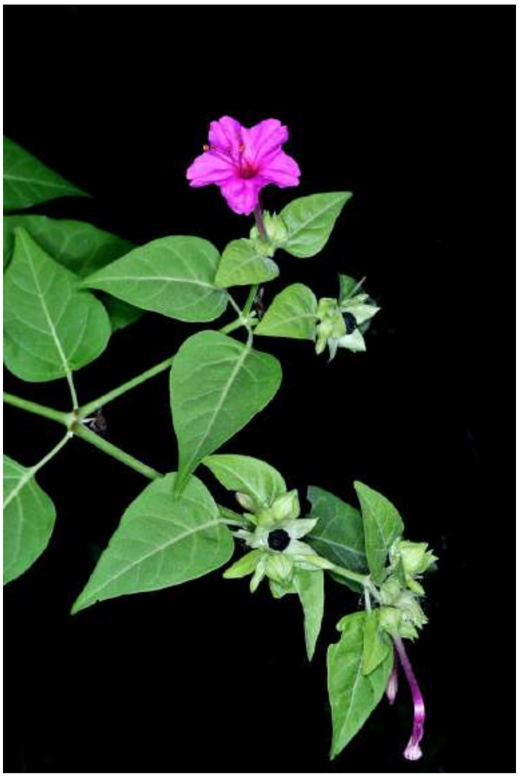

"*Mirabilis jalapa*" by Mauricio Mercadante, Brasília, DF, Brasil (CC BY-NC-SA 2.0)

Historia Naturalis & Medica Piso, 1658 Page number 219

Vernacular name(s) Gólfão. Aguapé

Species Nymphaea amazonum Mart. & Zucc.

Family Nymphaeaceae

Presence in the HNB Yes

Marcgrave (1648) 23 (only description)

Piso (1648) 91 (only description)

Notes

Piso used a similar woodcut to de l' Obel (1591: 594), but in reverse format, as it occurs often with copies made after the originals. The same woodcut was used by Clusius in the *Rariorum Plantarum* (1601: livr. IV, LXXVIJ). There is a similar woodcut entitled *Nenuphare, Nymphaea* in the *Herbarium viva Icones* by Otto Brunfels (1530: 37). This image resembles the woodcut in Dodoens (1553: Vol. I, 221). The botanists publishing with Plantin (such as Dodoens) often based some of their woodcuts on images found in previous herbals (Chen 2020). The *Nymphaea* in Brunfels and Dodoens is *Nymphaea alba* and the plant in de l' Obel and Clusius is *Nuphar lutea*; both Euroasian species. After all, Piso likely copied the image of *N. lutea major* by Clusius, a scholar he often cited throughout the text (Almeida 2016).

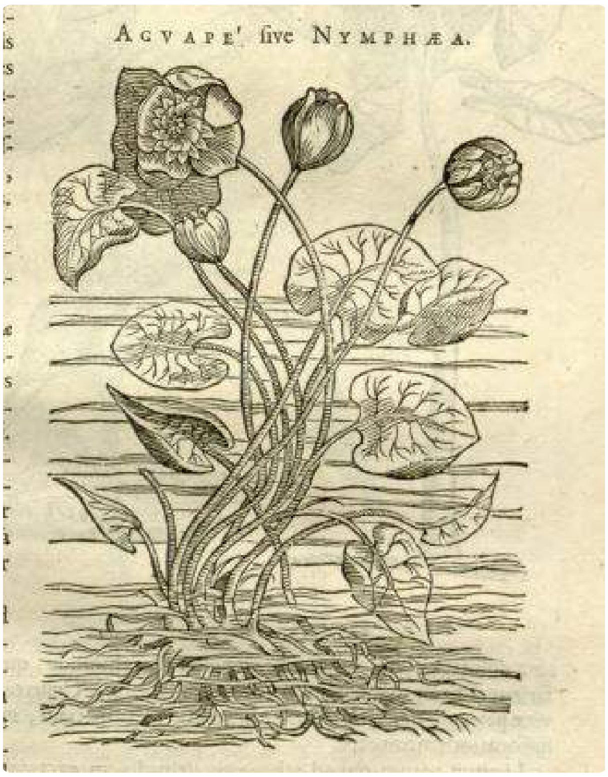

IV. *De Arboribus, fructibus, & herbis medicis*: 219

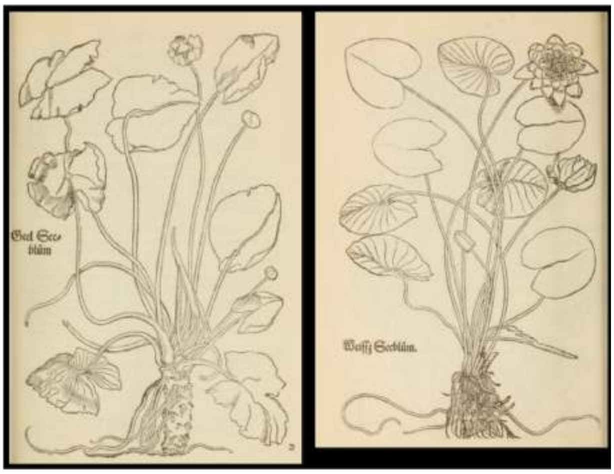

Nymphaea plants in *Herbarium viva Icones* by Otto Brunfels (1530: 36 and 37)

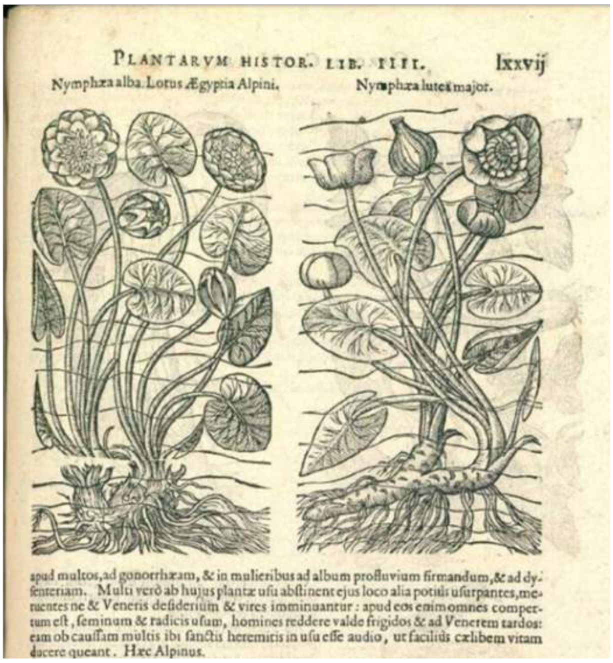

PLANTARVM HISTOR. LIB. IIII. lxxvij

Nymphæa alba. Lotus Ægyptia Alpini. Nymphæa lutea major.

apud multos, ad gonorrhæam, & in mulieribus ad album profluvium firmandum, & ad dyſenteriam. Multi verò ab hujus plantæ uſu abſtinent ejus loco alia potiùs uſurpantes, metuentes ne & Veneris deſiderium & vires imminuantur: apud eos enim omnes compertum eſt, ſeminum & radicis uſum, homines reddere valde frigidos & ad Venerem tardos: eam ob cauſſam multis ibi ſanctis heremitis in uſu eſſe audio, ut faciliùs cælibem vitam ducere queant. Hæc Alpinus.

Nymphaea plants in *Rariorum Plantarum* by Clusius (1601: livr. IV, LXXVIJ)

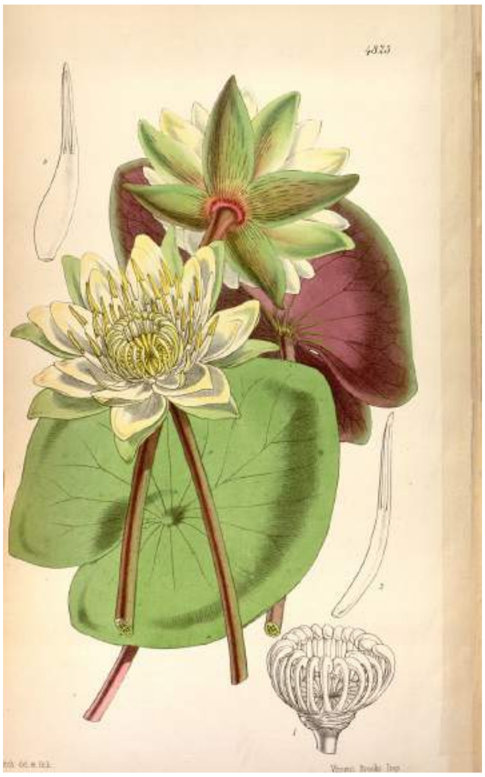

N. amazonum in *Bot. Mag.* 80 (Hooker 1854: 4823) Missouri Botanical Garden. Retrieved from www. biodiversitylibrary

Historia Naturalis & Medica Piso, 1658 Page number 225

Vernacular name(s) Quiya. Piper Brasiliensi

Species Capsicum baccatum L.

Family Solanaceae

Presence in the HNB Yes

Marcgrave (1648) 39 (only description)

Piso (1648) 108 (only description)

Notes

De Laet did not add images of peppers, as he claimed these were "well known to botanists and long cultivated in the gardens" (Marcgrave 1648: 39). Piso, though, disagreed with this and chose to include them in his treatise (1658). To create this woodcut, Piso combined two woodcuts with peppers from Clusius' *Curae Posteriores* (1611: 97 and 99), and he added a few peppers (on the left bottom corner of the woodcut) based on the peppers depicted in Monardes (1579: 69 and 70) entitled *Capsicum* and *Capsicum brasilianum* respectively. The latter was also used in de l' Obel (1591: Vol. I, 316, 317), as Plantin, who was also the publisher of Monardes' (1579) work, used the same woodblocks for Clusius and de l' Obel.

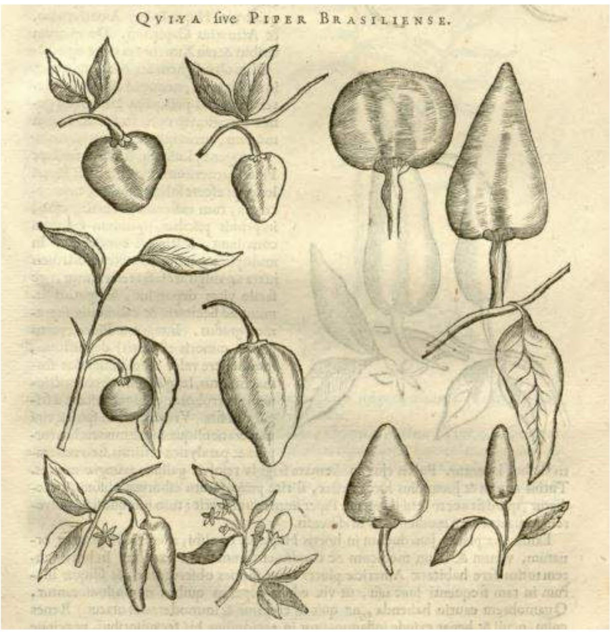

IV. *De Arboribus, fructibus, & herbis medicis*: 225

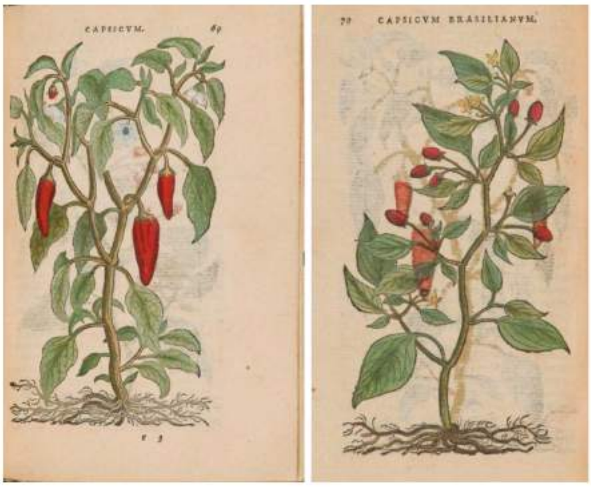

Capsicum spp. in *Simplicium Medicamentorum* by Monardes (1579: 69, 70)

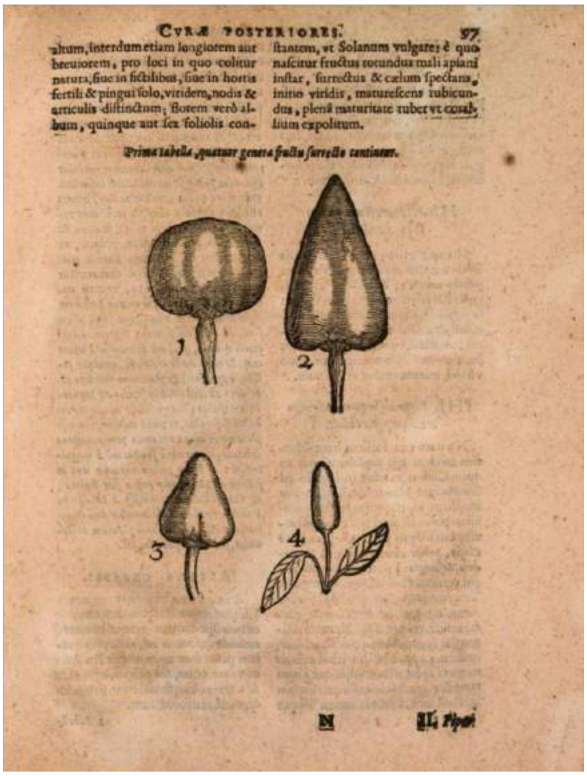

Capsicum spp. in *Curae Posteriores* by Clusius (1611: 97)

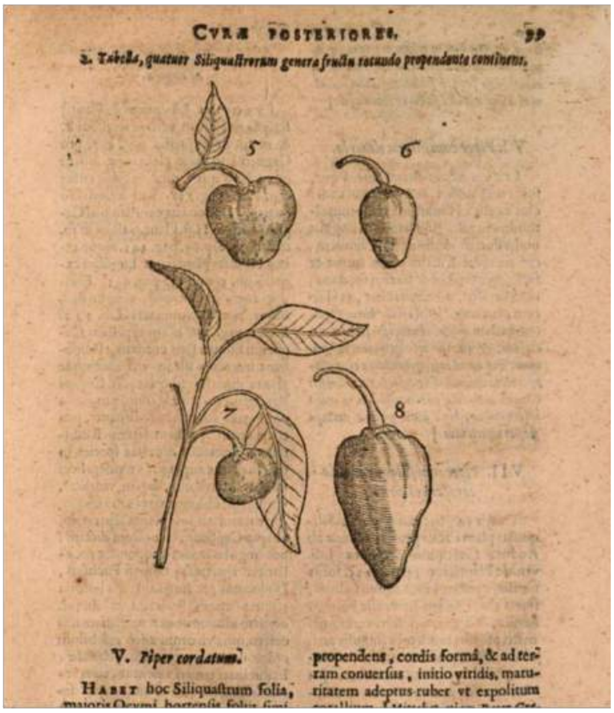

Capsicum spp. in *Curae Posteriores* by Clusius (1611: 99)

Historia Naturalis & Medica Piso, 1658 Page number 226

Vernacular name(s) Quiya

Species Capsicum frutescens L.

Family Solanaceae

Presence in the HNB Yes

Marcgrave (1648) 39 (only description)

Piso (1648) 107 (only description)

Notes

As in the previous entry, Piso wanted to add images of the peppers in his book. In this case, the woodcut resembles the image in Clusius' *Curae Posteriores* (1611: 101), which might also correspond to *Capsicum annuum*. He placed it upside-down and added a little flower and a few leaves. The woodblocks used for Clusius are part of the Plantin-Moretus Museum, kept in Antwerp, Belgium. Piso ordered to make a woodblock based on Clusius' work, but we do not know where this copy is today, or if it is still preserved at all.

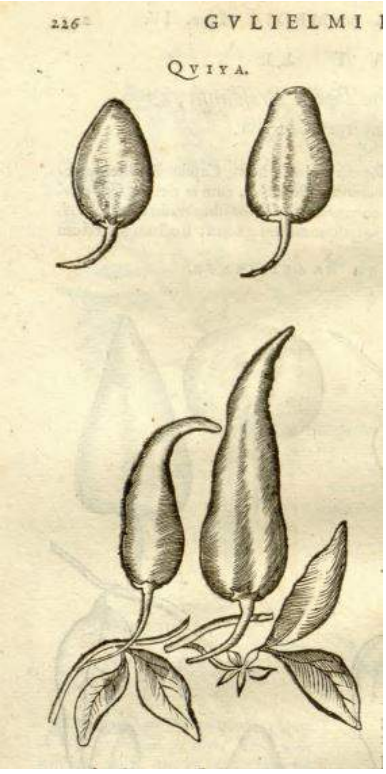

IV. *De Arboribus, fructibus, & herbis medicis*: 226

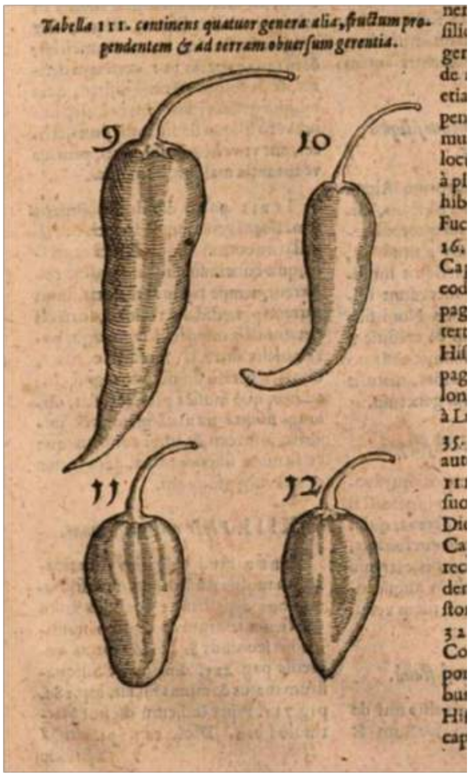

Capsicum spp. in *Curae Posteriores* by Clusius (1611: 101)

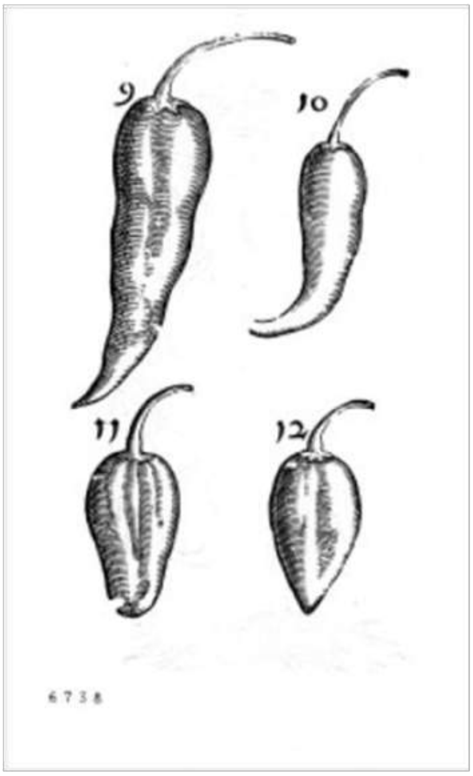

Capsicum spp. Woodblocks Museum Plantin-Moretus, Antwerp, Belgium. Retrieved from Plantillustrations. org

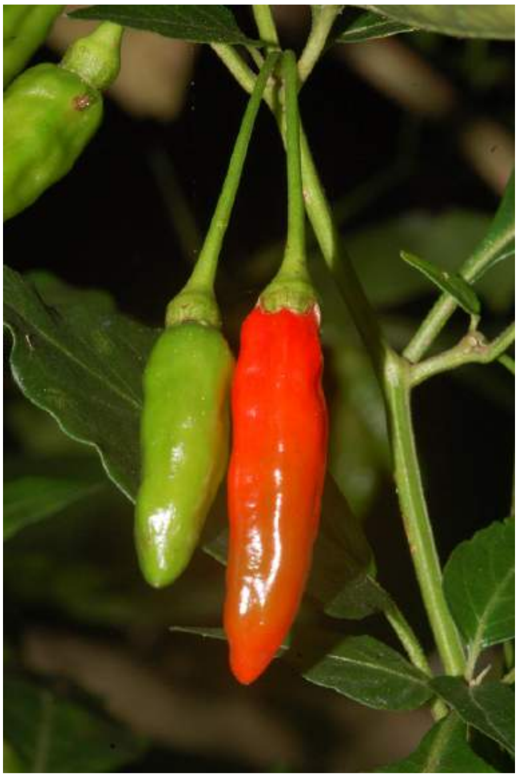

"*Capsicum frutescens*" by Barry Hammel (CC BY-NC-SA 2.0)

Historia Naturalis & Medica Piso, 1658 Page number 233

Vernacular name(s) Conambaia. Filix

Species <u>Adiantum pulverulentum L.</u>

Family Pteridaceae

Presence in the HNB Yes

Marcgrave (1648) 2 (only description)

Piso (1648)

Notes

This woodcut was based on the images of *Filix pumila* depicted in the *Rariorum Plantarum* by Clusius (1601: CCXIJ, livr. VI). The species we can see in Clusius are ferns from the Aspleniaceae family. *A. pulverulentum* is also described in Marcgrave (1648: 2), but it does not include a woodcut. Piso (1658), mentioned that this plant is similar to the European fern. According to Edgington (2013), the fern depicted in Clusius is *Phegopteris connectilis* (Michx.) Watt, a native of forests from the northern hemisphere, hence not from Brazil.

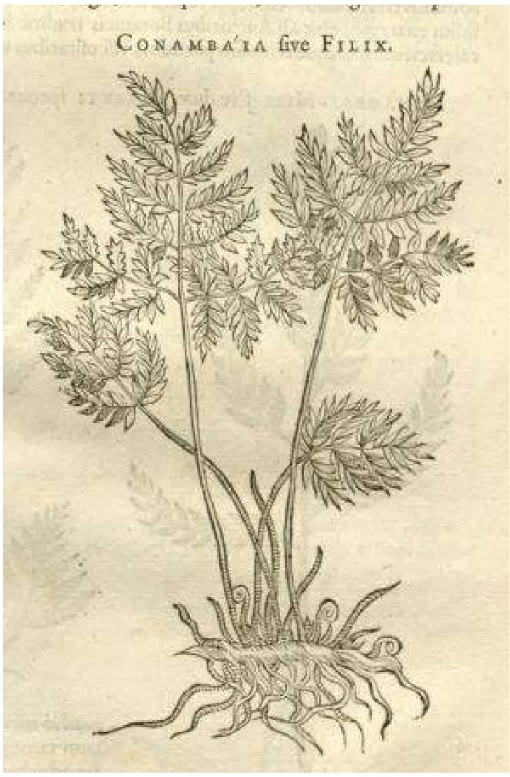

IV. *De Arboribus, fructibus, & herbis medicis*: 233

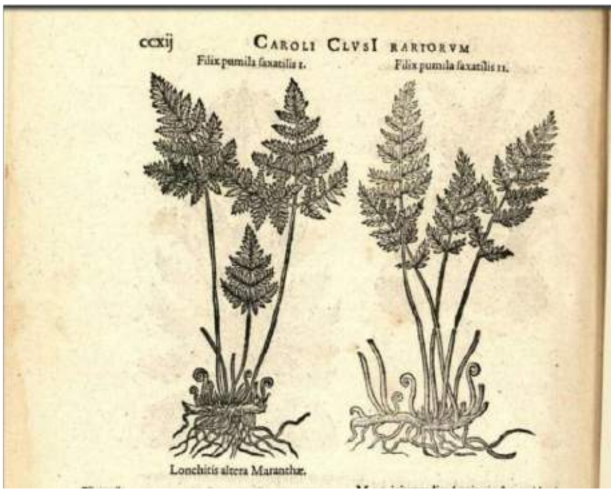

Ferns from the Aspleniaceae family in *Rariorum Plantarum* by Clusius (1601: livr. VI, CCXIJ)

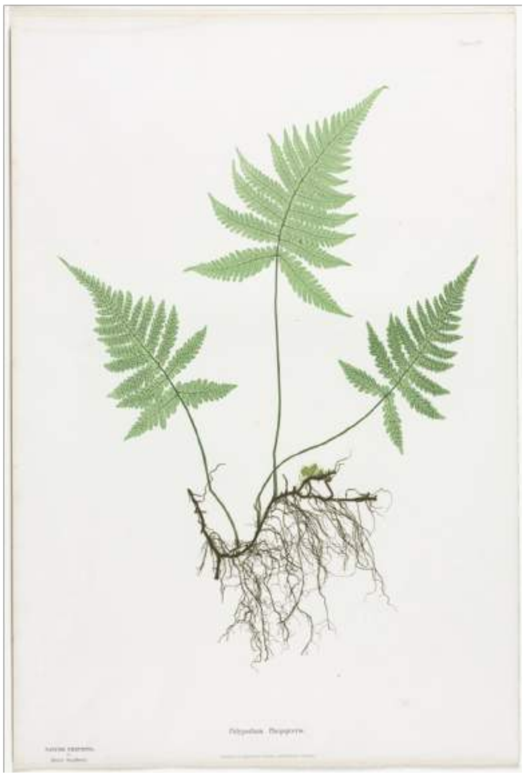

Ferns of Great Britain and Ireland: *Phegopteris connectilis* by Henry Bradbury (British, 1829-1860)

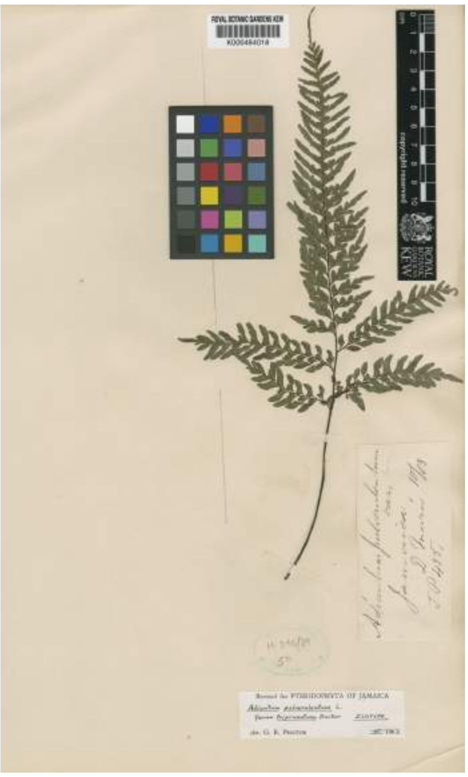

A. pulverulentum specimen from Kew's Herbarium - K000484018. Retrieved from Plants of the World Online

Historia Naturalis & Medica Piso, 1658 Page number 237b

Vernacular name(s)	Iaçapé
Species	Cyperus sp. (Cyperus aff. surinamensis Rottb.)
Family	Cyperaceae

Presence in the HNB	Yes
Marcgrave (1648)	2 (only description)
Piso (1648)	96 (only description)

Notes

So far, the origin of this woodcut is uncertain. There is the possibility that Piso ordered it to be made after the image of *Iuncus Maritimus* (*Cyperus capitatus* Vand., of Mediterranean distribution), which woodcut belonged to the Plantin publishers. This image can be seen in de l' Obel (1591: vol. I, p. 87). Pickel (2008: 37) identified this plant as *C. surinamensis*. It could be *Cyperus sesquiflorus* (Torr.) Mattf. & Kük., which specimen was collected by Marcgrave (Marcgrave's herbarium, p. 120).

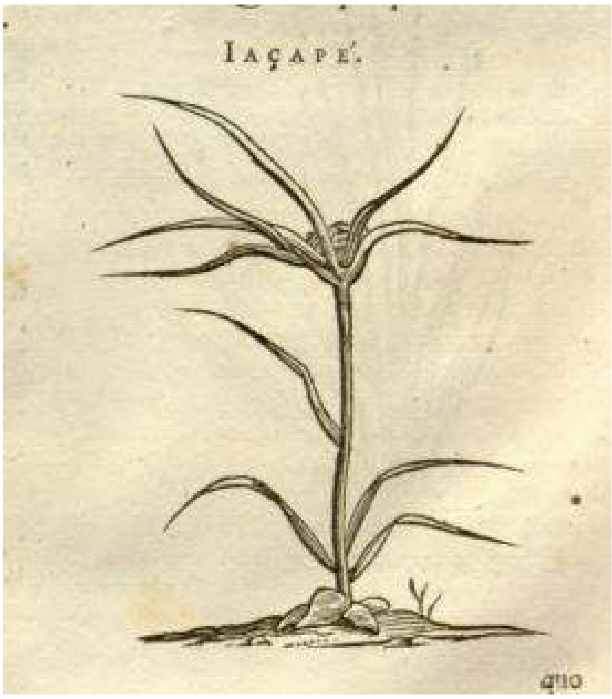

IV. *De Arboribus, fructibus, & herbis medicis*: 237b

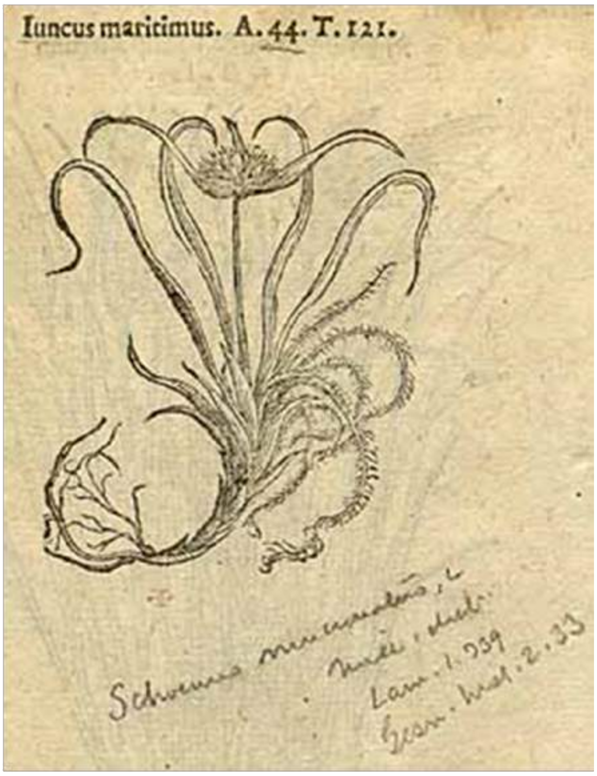

Cyperus capitatus in *Plantarum seu stirpium icones* by Obel (1581: Vol. I, 87)

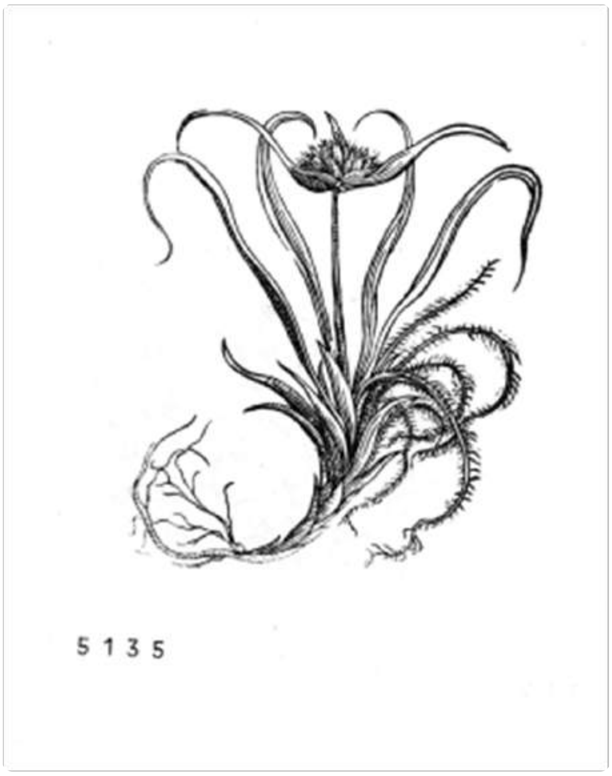

C. capitatus. Woodblocks Museum Plantin-Moretus, Antwerp, Belgium. Retrieved from Plantillustrations

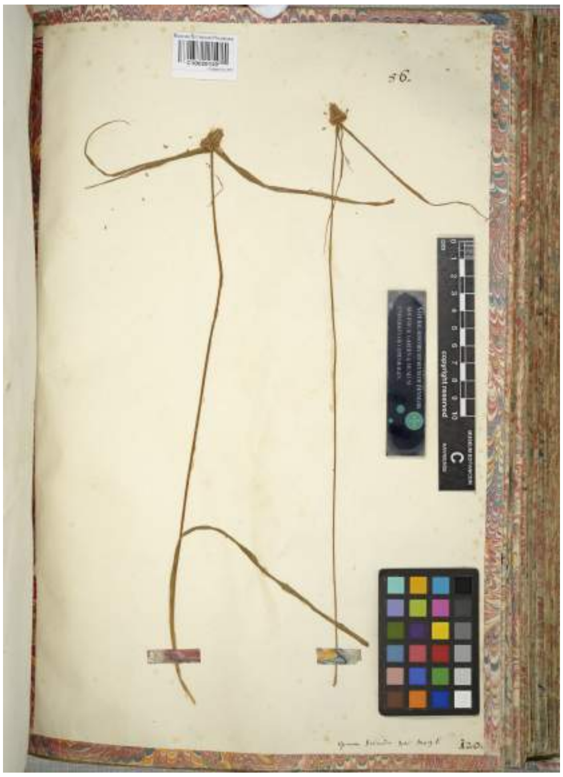

Specimen of *Cyperus sesquiflorus* (Torr.) Mattf. & Kük. in Marcgrave's herbarium, p. 120

Historia Naturalis & Medica Piso, 1658 Page number 240

Vernacular name(s) Erva de Sta Maria. Dracunculus major

Species Asterostigma riedelianum (Schott) Kuntze

Family Araceae

Presence in the HNB Yes

Marcgrave (1648) 27 (only description)

Piso (1648)

Notes

The original image of this plant, which represents the non-Brazilian *Dracunculus vulgaris* Schott, appeared in the *New Kreüterbuch* by Fuchs (1543: plate 130). Later, this image was also used in the *Stirpium Historiae* by Dodoens (1553: Vol. I, p. 329). The latter could have been a copy, as this is a reversed image. This image is also similar to the Aracea (*Dracontis)* depicted in the *Plantarum seu stirpium historia* by de l' Obel (1576: 327). Piso misused a copy of this image, in which he attached to the stem the leaf that belonged to another individual (see Fuchs and Dodoens) to accompany the description of the Brazilian *A. riedelianum*.

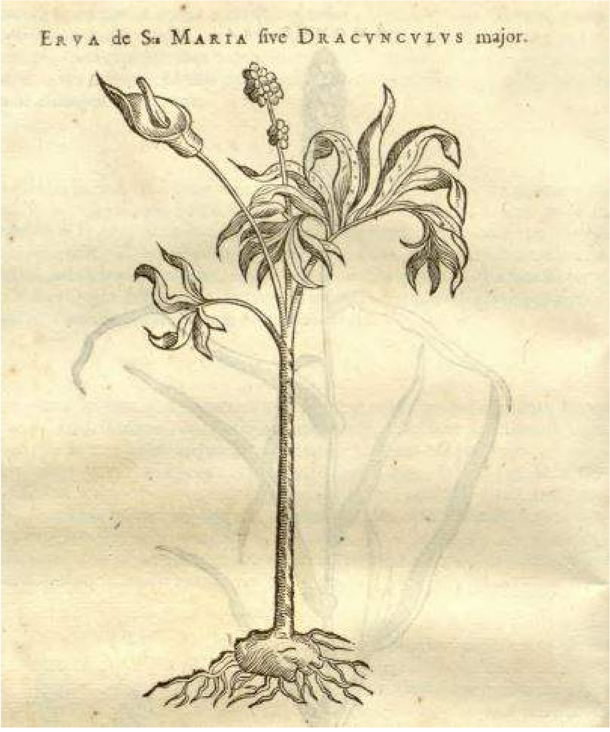

IV. *De Arboribus, fructibus, & herbis medicis*: 240

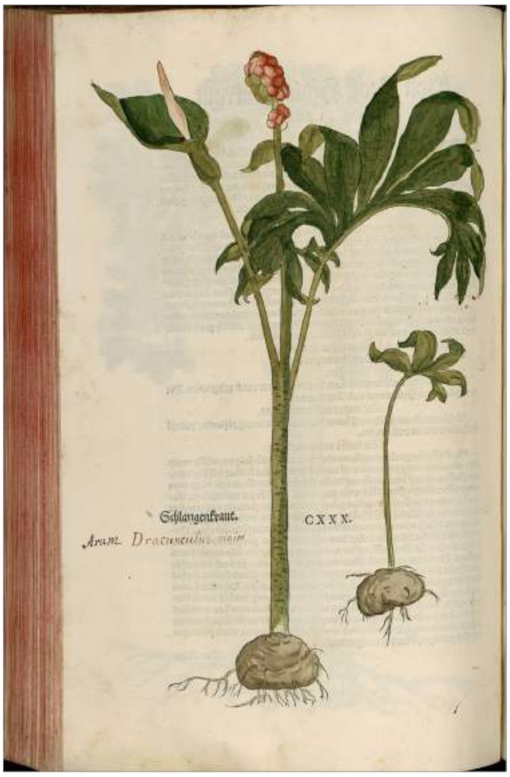

Dracunculus vulgaris in the New *Kreüterbuch* by Fuchs (1543: 130). Retrieved from Plantillustrations.org

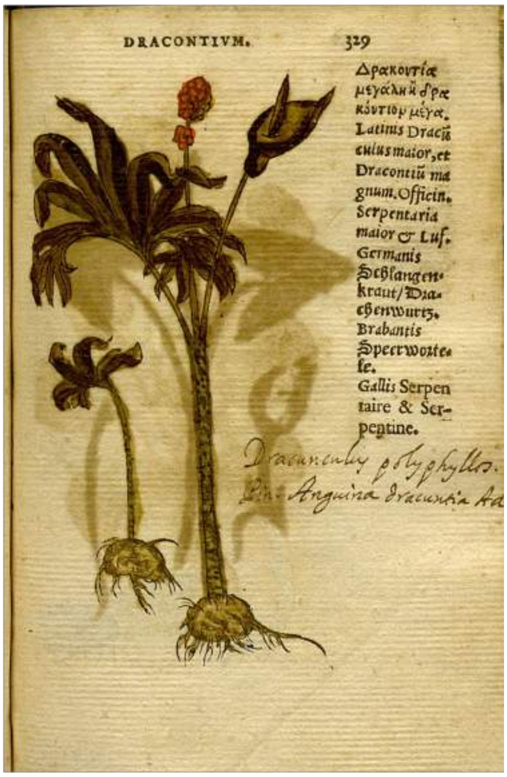
DRACONTIVM. 329

Δρακοντία μεγάλη ἢ δρακόντιον μέγα. Latinis Dracũculus maior, et Dracontiũ magnum. Officin. Serpentaria maior & Luf. Germanis Schlangenkraut/Drachenwurtz. Brabantis Speerwortele. Gallis Serpentaire & Serpentine.

D. vulgaris in *Stirpium Historiae* by Dodoens (1553: Vol. I, 329)

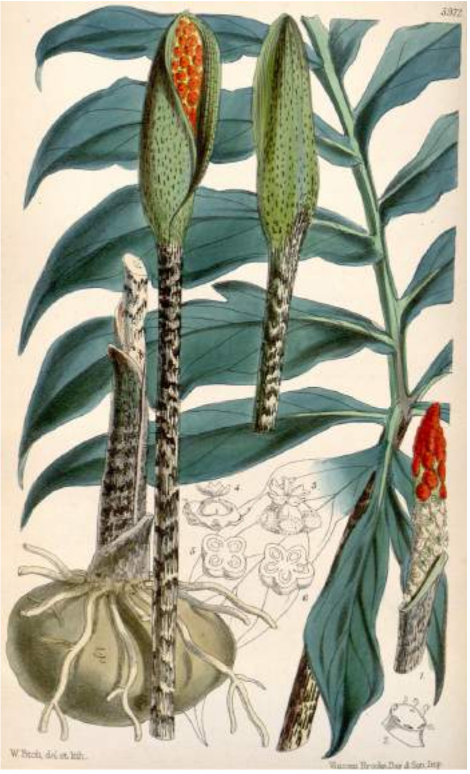

A. luschnathianum, a related species to A. riedelianum, from the SE of Brazil. W. Fitch (1892) - *Curtis's Bot. Mag.* v.98 [ser.3:v.28] (1872)

Historia Naturalis & Medica Piso, 1658 Page number 241a

Vernacular name(s) Capicatinga. Iacarecatinga. Acori species

Species Scleria gaertneri Raddi

Family Cyperaceae

Presence in the HNB No

Marcgrave (1648)

Piso (1648)

Notes

This image does not represent the tropical sedge *S. gaertneri*. This woodcut was probably made after the Iris portrayed in Fuchs (1542: 12) -but without the flower- and later published in Dodoens (1553: 233) as *Gladiolus Luteus*. This Iris is documented in Fuchs as *Acorum officinarum* and corresponds to *Iris pseudacorus* L., of a Mediterranean distribution. It also resembles the *Acorum* depicted in the *New Kreuterbuch* by Matthioli (1563: 4); or the *Acorum legitimum* depicted in the *Atrebat Rariorum alioquot stirpium per Hispanias observatarum*... by Clusius (1576: 521), especially the morphology of the root. However, the strong resemblance lay with the visual sources of Fuchs and Dodoens.

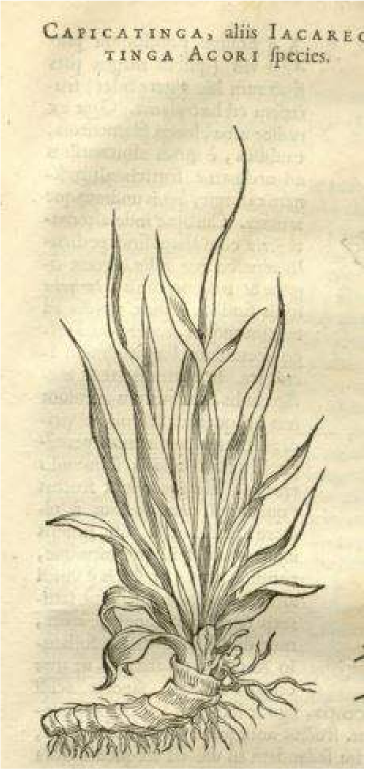

IV. *De Arboribus, fructibus, & herbis medicis*: 241a

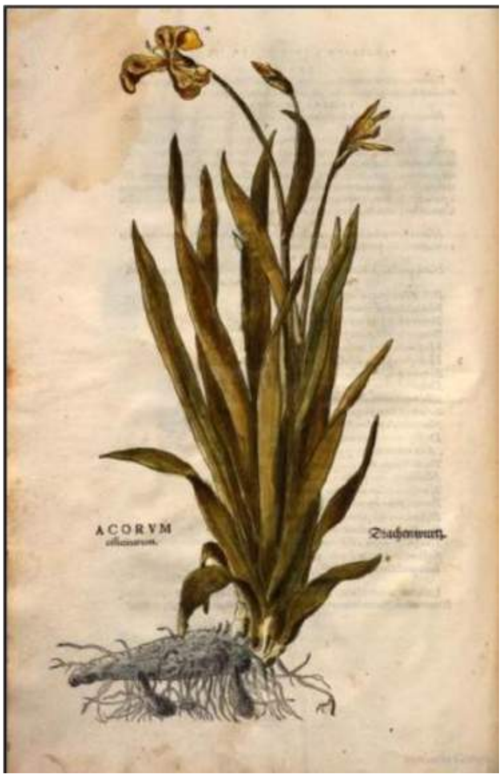

Iris pseudacorus in the *De Historia Stirpium* by Fuchs (1542: 12)

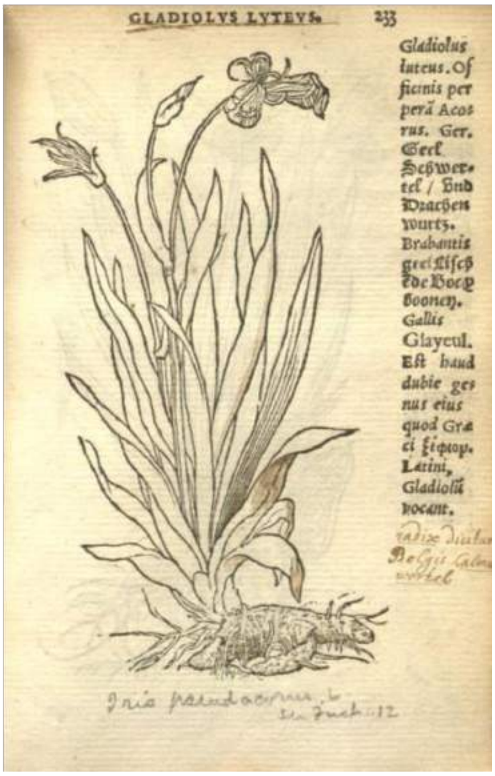

I. pseudacorus in the *Stirpium Historiae* by Dodoens (1553: 233)

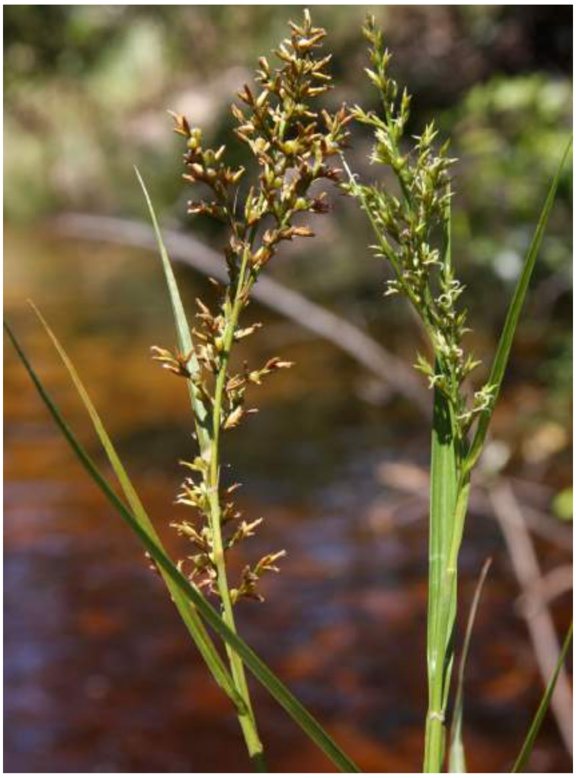

Scleria gaertneri- Specimen Araújo, A.C. 1869
D. Zappi/RBG, Kew. Retrieved from Plants of the World Online

Historia Naturalis & Medica Piso, 1658 Page number 245a

Vernacular name(s) Ibiraeém. Liquiritia silvestris

Species Periandra mediterranea (Vell.) Taub.

Family Fabaceae

Presence in the HNB No

Marcgrave (1648)

Piso (1648)

Notes

According to (2008: 119), this is a "fantasy woodcut" because it does not correspond to the described plant. Piso must have ordered to make a woodcut based on the image depicted in the *Stirpium Historia* by Dodoens (1553: vol. 2, 232). This image corresponds to the Eurasian species *Glycyrrhiza glabra* L. (Fabaceae), which is known for its use to extract licorice, due to its sweet root. It seems that he related this species to the European one, due to its sweet roots, which were already known by the Jesuits in the Portuguese colonies in sixteenth century Brazil as Brazilian licorice (Pereira et al. 1996). Piso never depicted the Brazilian *P. mediterranea*, so we show it here.

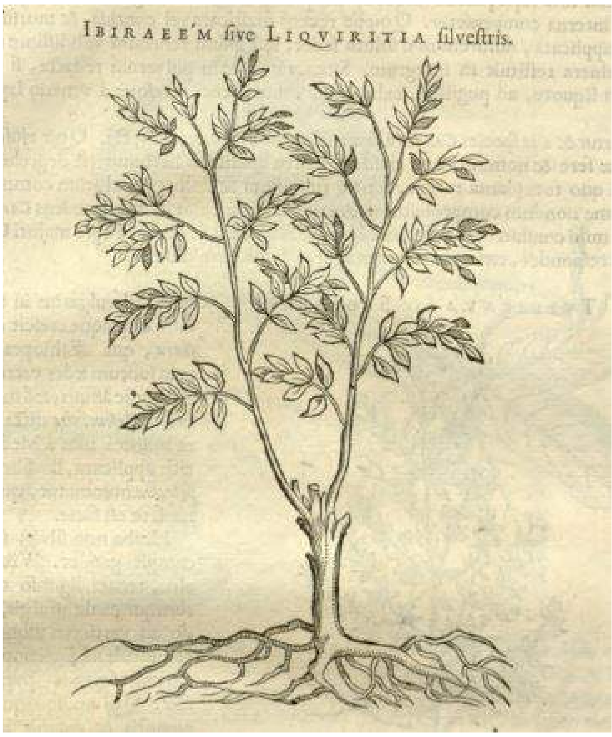

IV. *De Arboribus, fructibus, & herbis medicis*: 245a

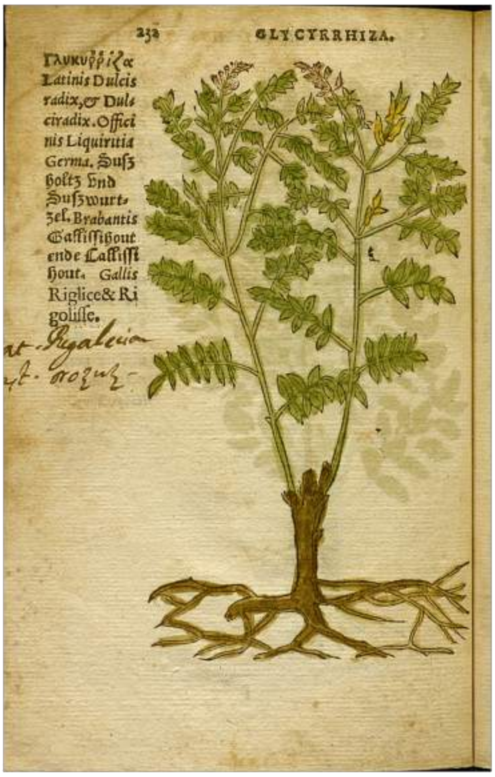

I. pseudacorus in the *Stirpium Historiae* by Dodoens (1553: Vol II, 232)

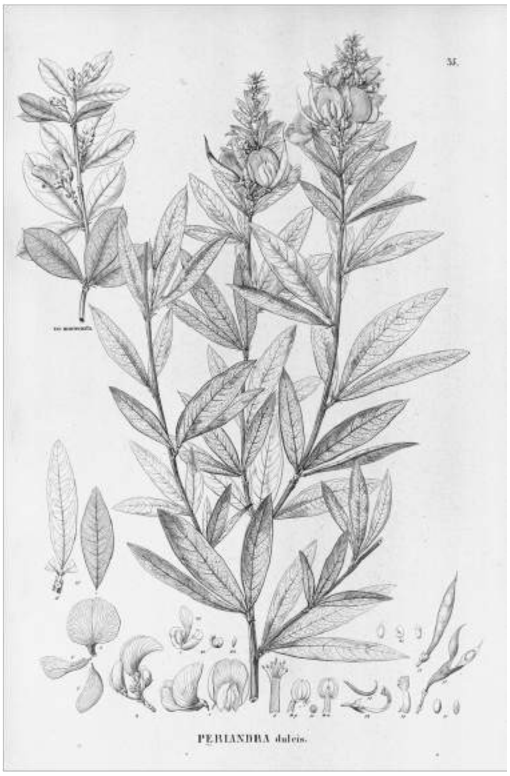

Engraving of *Periandra* in Martius, C.F.P. von, Eichler, A.G., Urban, I., *Flora Brasiliensis* (1840-1906). Plate 35

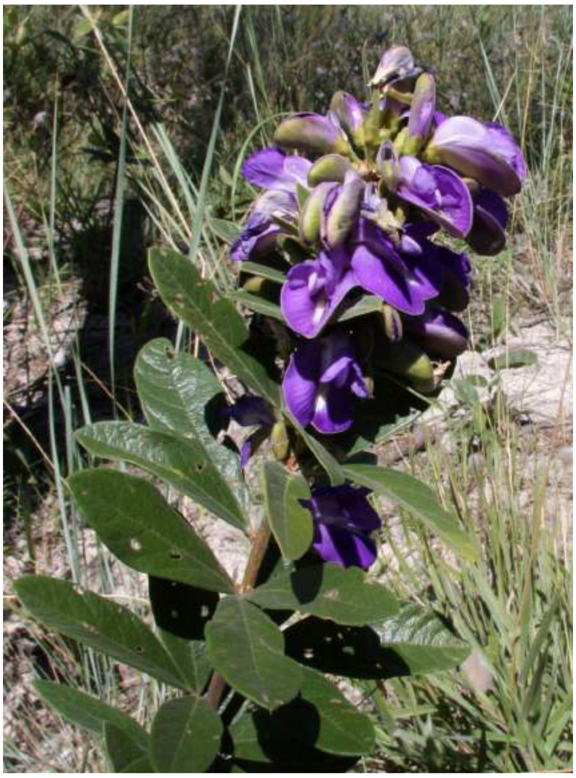

Periandra mediterranea - Specimen Zappi, D.C. 1998 D. Zappi/RBG, Kew. Retrieved from Plants of the World Online

Historia Naturalis & Medica Piso, 1658 Page number 246

Vernacular name(s) Tupeiçava. Scoparia

Species Scoparia dulcis L.

Family Plantaginaceae

Presence in the HNB Yes

Marcgrave (1648) 52 (only description)

Piso (1648) 110a

Notes

This woodcut shows an image of *S. dulcis* that is slightly modified, and presented in the reversed format compared to the image published in the HNB (Piso 1648). Pickel (2008) placed it under the category of "fantasy woodcut". The verticillate leaves (arranged in whorls) are accurately represented in the woodcut, but the flowers do not match well with the flowers in nature.

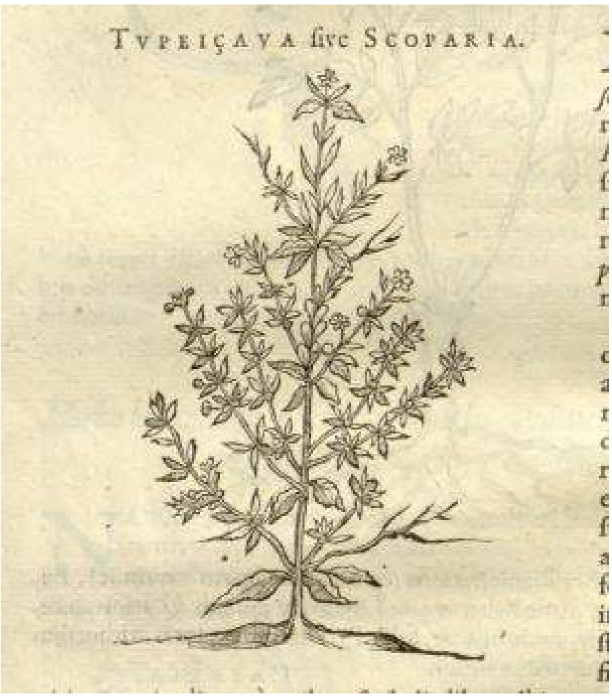

IV. *De Arboribus, fructibus, & herbis medicis*: 246

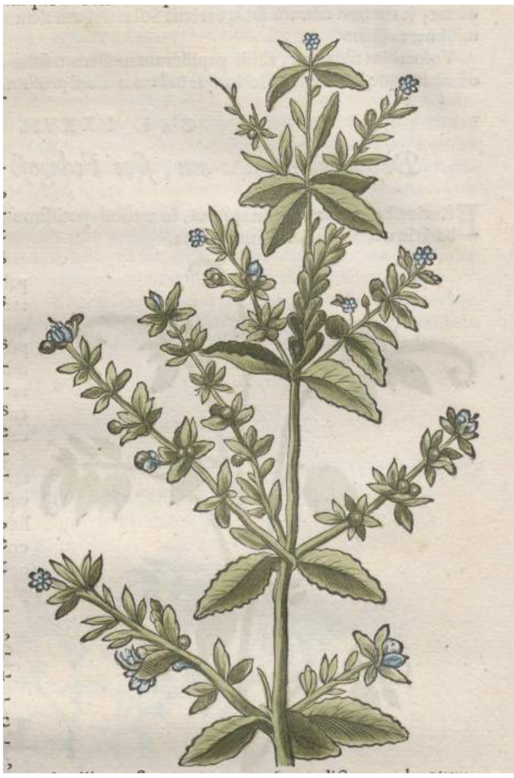

Scoparia dulcis in the HNB (Piso 1648: 110a)

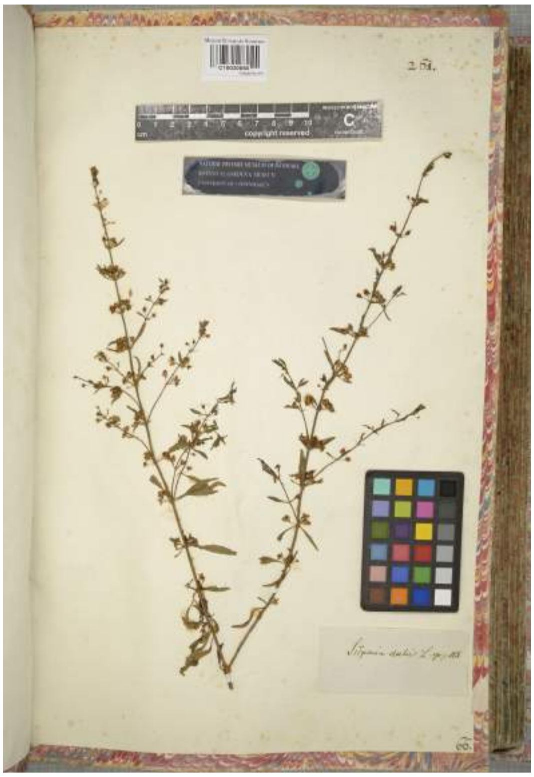

S. dulcis specimen in Marcgrave's herbarium (f. 66)

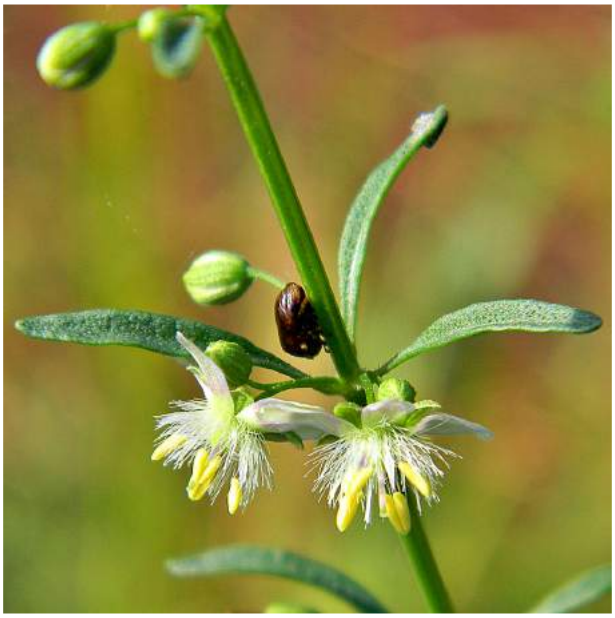

"Tiny Flowers, Tiny Creature (*Scoparia dulcis*)" by Bob Peterson (CC BY 2.0)

Historia Naturalis & Medica Piso, 1658 Page number 251

Vernacular name(s) Cumandatiá

Species Lablab purpureus (L.) Sweet

Family Fabaceae

Presence in the HNB Yes

Marcgrave (1648) 33, 52 (only description)

Piso (1648)

Notes

This woodcut strongly resembles the right corner of the plant woodcut depicted in *De Plantis Aegypti liber* by Alpini (1640: 75), called *Phaseolus Niger Lablab*. This is an African plant that was portrayed based on the plants encountered by botanist Alpini in his travels to Egypt. *De Plantis Aegypti liber* was first published in Venice, in 1592. Hence, this - like other tropical African plants- was introduced to European naturalists in the sixteenth century. Piso (1658) cited Alpini in the text, although he does not acknowledge that the woodcut was copied from him. He also mentioned how this plant was imported from Africa to Brazil.

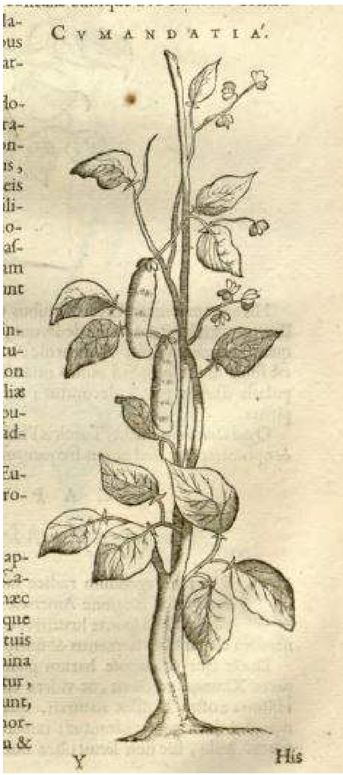

IV. *De Arboribus, fructibus, & herbis medicis*: 251

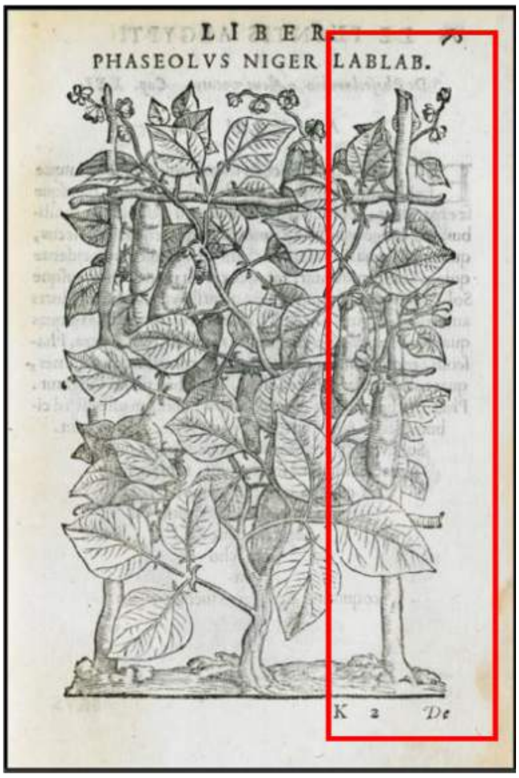

Lablab purpureus in *De Plantis Ægypti liber* by Alpini (1640: 75; extended edition from 1592)

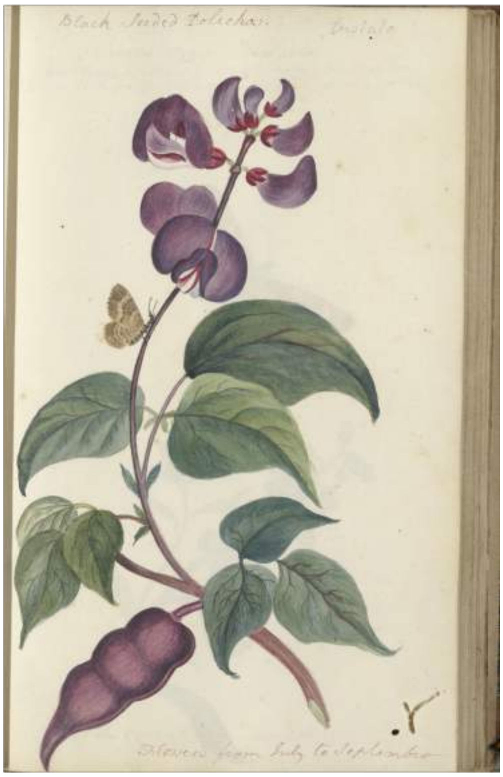

Anonymous, Album of drawings of English moths, butterflies, flowers, and molluscs (1805-1822), based on S.T. Edwards, Bot. Mag., vol. 23: t. 896 (1801)

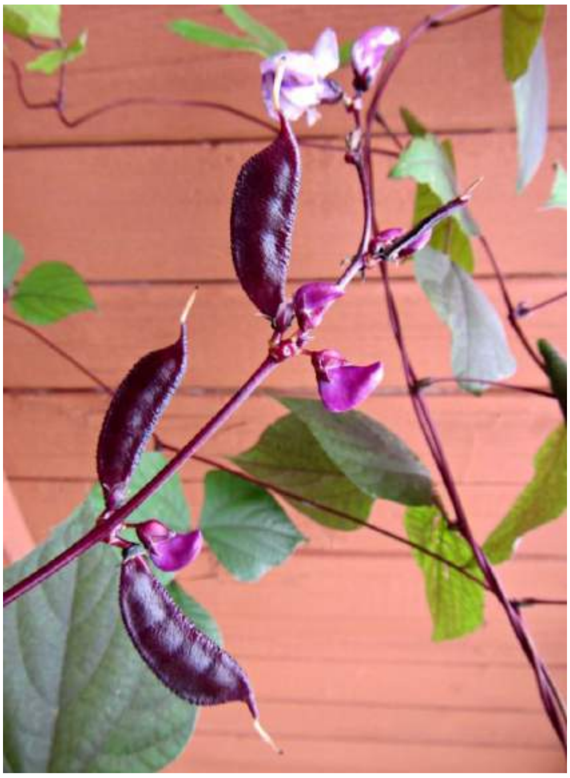

L. purpureus in "I still have blooms in the garden" by CameliaTWU (CC BY-NC-ND 2.0)

Historia Naturalis & Medica Piso, 1658 Page number 252

Vernacular name(s) Guandu

Species Phaseolus lunatus L.

Family Fabaceae

Presence in the HNB No

Marcgrave (1648)

Piso (1648)

Notes

The woodcut resembles the plant in Monardes (1579: 63) called *Phaseolus Alter Brasilianus*. There are similar drawings in the *Rariorum Plantarum* by Clusius (1601: livr. VI, CCXXIJ) -called *Phaseolus peregrinus*-, although the similarities are not as great as with Monardes' image. The woodcut is used again in the *Exoticorum Libri Decem* by Clusius (1605: 336), in the part where he discusses the plants that Monardes documented from the Spanish colonies in the Americas. Piso mentioned the Portuguese (*grãos de bico*) and the Dutch/Frisian (*witte krombekjes*) names of this cultivated plant in Dutch-Brazil. *Guandu* is the Afro-Brazilian name used nowadays for the pigeon pea (*Cajanus cajan*), also documented by Marcgrave (1648: 62) and Piso (1658: 251) and likely introduced to Brazil via the

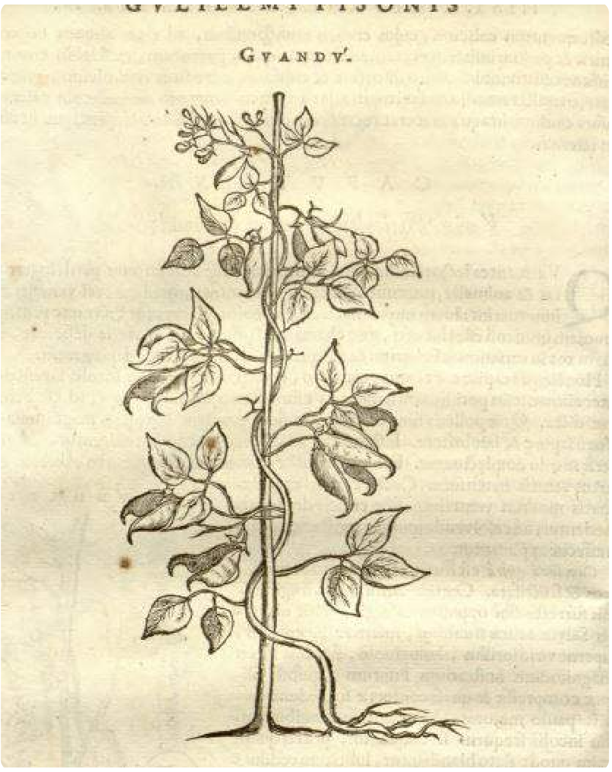

IV. *De Arboribus, fructibus, & herbis medicis*: 252

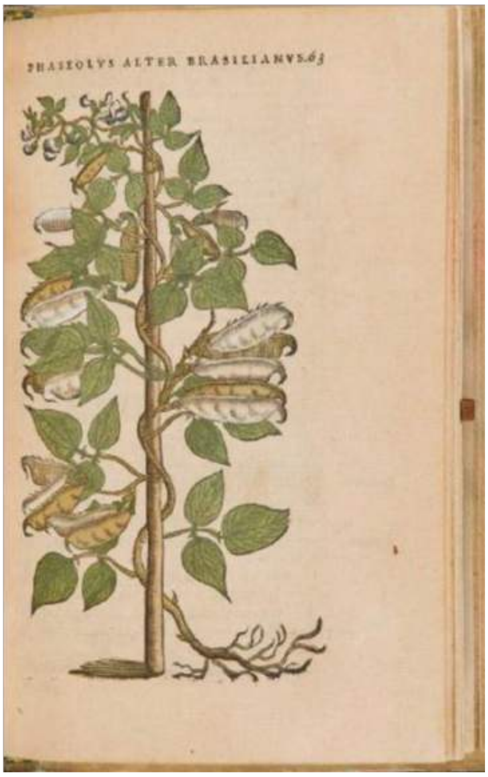

Phaseolus lunatus, named as *Phaseolus Alter Brasilianus* in the *Simplicium medicamentorum* by Monardes (1579: 63)

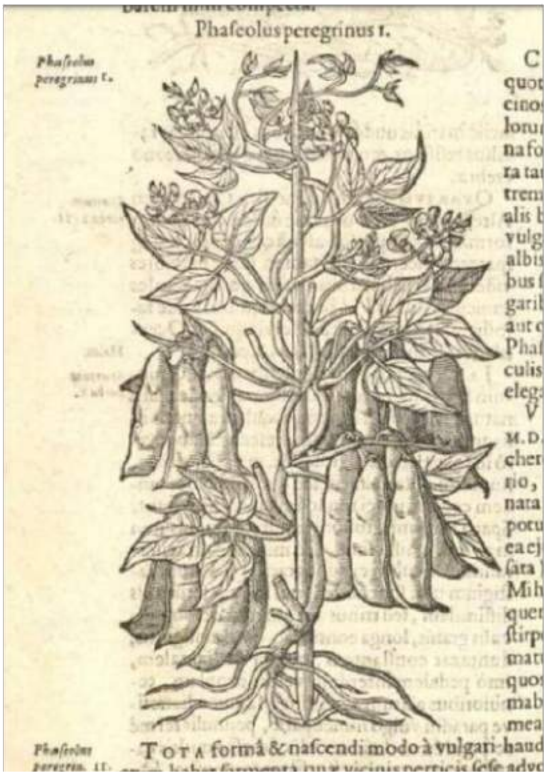

P. lunatus in the *Rariorum Plantarum* by Clusius (1601: Livr. VII, CCXXIJ)

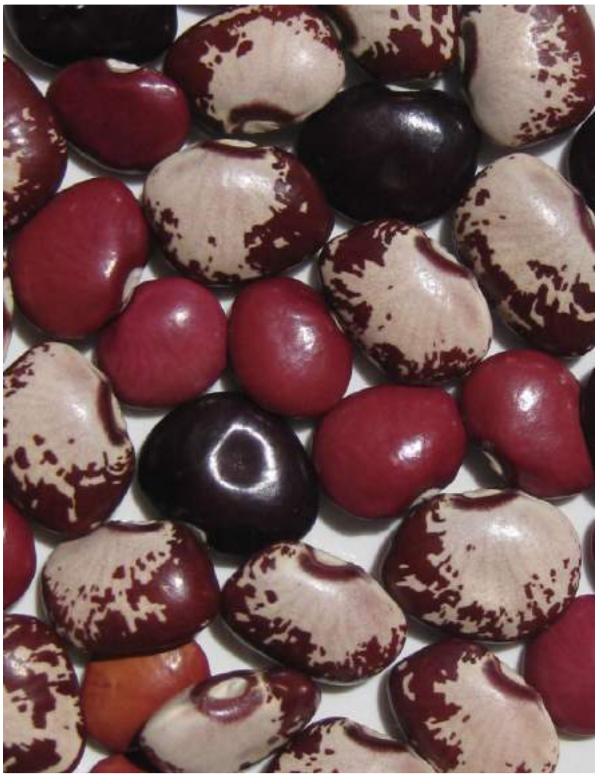

"*Phaseolus lunatus* - seeds" by tonrulkens (CC BY-SA 2.0)

Historia Naturalis & Medica Piso, 1658 Page number 253b

Vernacular name(s) Ietucu. Mechoacana cum Flore

Species Operculina hamiltonii (G. Don) D.F. Austin & Staples

Family Convolvulaceae

Presence in the HNB Yes

Marcgrave (1648) 41 (different woodcut)

Piso (1648) 94 (different woodcut)

Notes

Piso stated in the text that he added the figure himself, representing its size at a natural scale. The sepals of this species are pressed to the corolla tube, resulting in a persistent calyx. The fruit is distinctive and recognizable when is dry (as in a herbarium voucher) and it consists of an operculate capsule, often a four-lobed fruit (Staples et al. 2020). The woodcut, hence, represents the fruit of this species, particularly in the first stage of its dehiscence (before opening completely and releasing the seeds) (Staples et al. 2020). Possibly Piso kept a dry specimen of this fruit and he ordered to make a woodcut for his treatise, but he confused it with the flower.

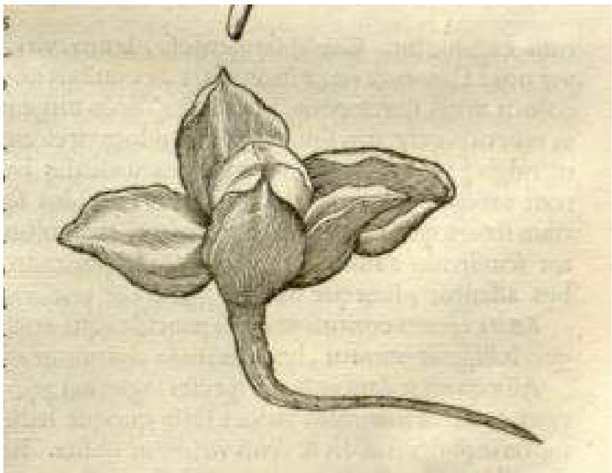

IV. *De Arboribus, fructibus, & herbis medicis*: 253b

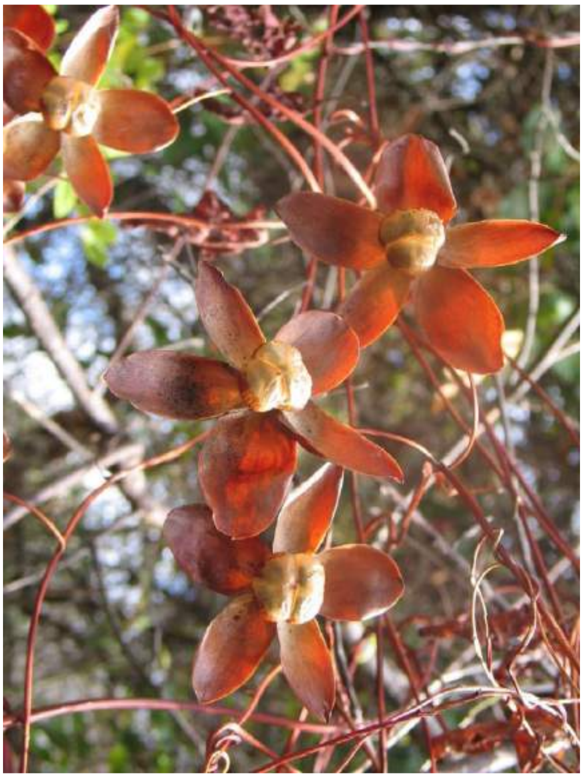

Fruits. "Batata de purga- Operculina alata [syn. of *O. hamiltonii*]" by antoniosergio25 (CC BY-NC-SA 2.0)

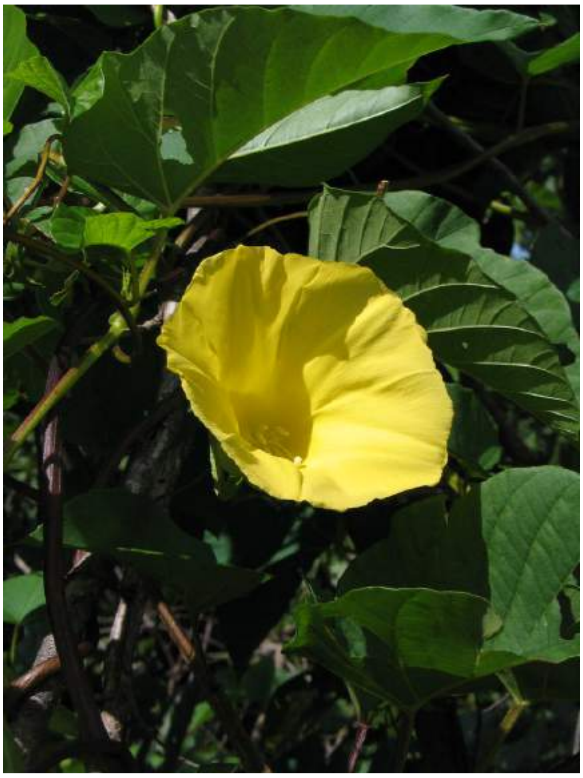

Flower. "Batata de purga- *Operculina alata* [syn. of *O. hamiltonii*]" by antoniosergio25 (CC BY-NC-SA 2.0)

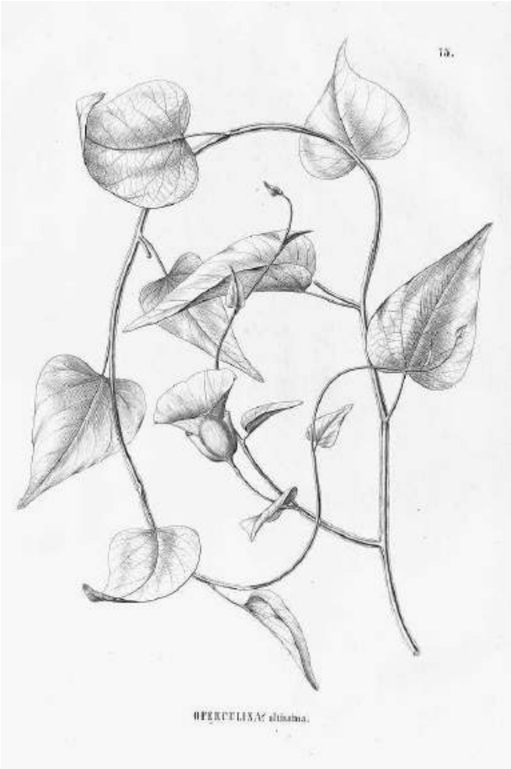

Engraving of *O. hamiltonii* in Martius, C.F.P. von, Eichler, A.G., Urban, I., *Flora Brasiliensis* (1856-1871). Vol. VII, Plate 75

Historia Naturalis & Medica Piso, 1658 Page number 256b

Vernacular name(s) Mundubi fructus

Species Arachis hypogaea L.

Family Fabaceae

Presence in the HNB Yes

Marcgrave (1648) 37 (different woodcut)

Piso (1648)

Notes

Piso based one of his woodcuts on the images of peanuts depicted in De Laet's books (1633: 568, 1640: 503). In his treatises, De Laet was discussing the different Brazilian plants -such as the peanuts- after the knowledge, he obtained from the books of Thevet (1558), Lery (1578), and Clusius (1605: 345), who called it *Manobi* the peanut cultivated in Brazil. There is a watercolor of *A. hypogaea* in the *Libri Principis* (f. 81 [61]). This watercolor, though, bears more resemblance to the peanuts portrayed in the still-life painting by Eckhout than to the woodcut (see S2).

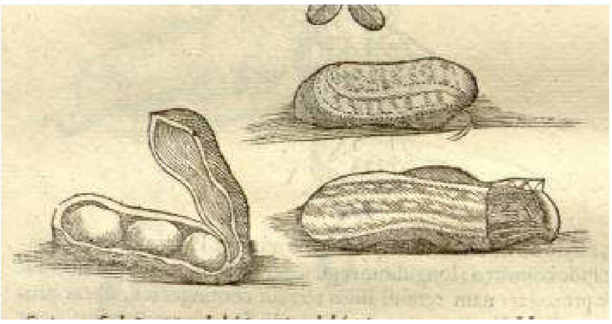

IV. *De Arboribus, fructibus, & herbis medicis*: 256b

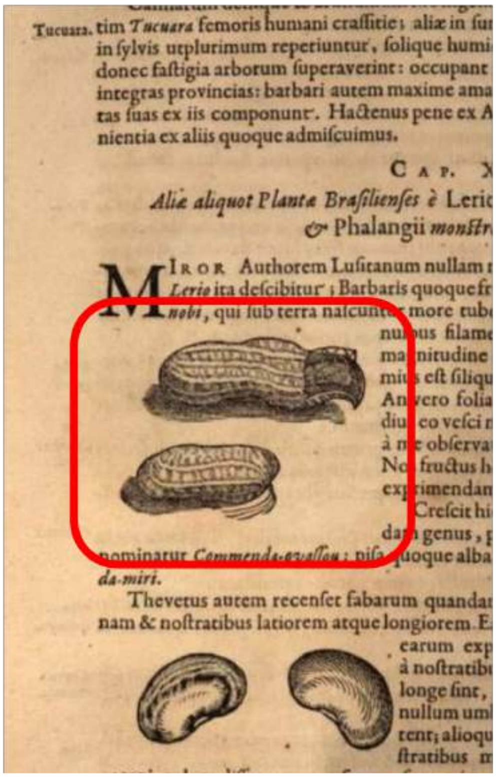

Tucuara. tim *Tucuara* femoris humani craſſitie; aliæ in fu
in ſylvis utplurimum reperiuntur, ſolique humi
donec faſtigia arborum ſuperaverint: occupant
integras provincias: barbari autem maxime ama
tas ſuas ex iis componunt. Hactenus pene ex A
nientia ex aliis quoque admiſcuimus.

C A P. X

Aliæ aliquot Plantæ Braſilienſes è Leri
& Phalangii *monſtr*

MIROR Authorem Luſitanum nullam
Lerio ita deſcibitur; Barbaris quoque fr
nobi, qui ſub terra naſcunt more tub
nu us filam
ma nitudine
mi eſt ſiliqu
An vero folia
diu eo veſci
à m obſerva
No fructus h
ex rimendan
Creſcit hi
da genus,
nominatur *Commenda-ovaſſou*; piſa quoque alba
da-miri.
Thevetus autem recenſet fabarum quanda
nam & noſtratibus latiorem atque longiorem. E
earum exp
à noſtratib
longe ſint,
nullum um
tent; alioqu
ſtratibus

Americae utriusque Descriptio Nouus orbis seu Descriptionis Indiae Occidentalis, by De Laet (1633: 568

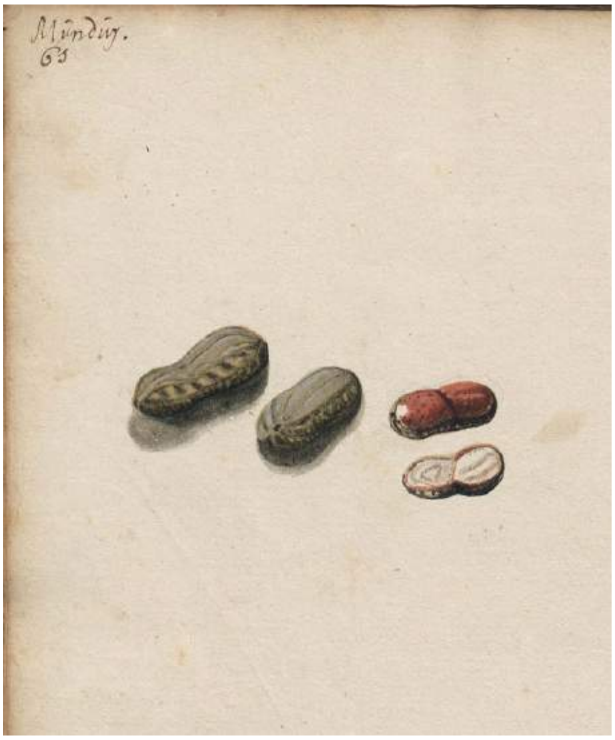

A. hypogaea underground seeds in the *Libri Principis* f. 81 [61]

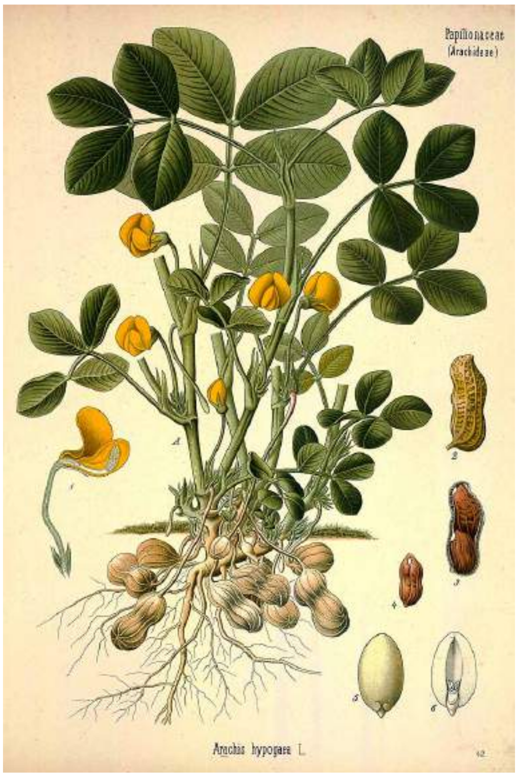

A. hypogaea in the *Medizinal Pflanzen* by Kohler, F.E. (1898: Vol. III, 42). Retrieved from Plantillustrations. org

Historia Naturalis & Medica Piso, 1658 Page number 257

Vernacular name(s) Iupicanga. Vulgo Radix China

Species Smilax rufescens Griseb.

Family Smilacaceae

Presence in the HNB Yes

Marcgrave (1648)

Piso (1648) 99 (different woodcut)

Notes

For this image, Piso combines the woodcut from the HNB with the Pseudo-china radix (*Smilax* cf. *pseudochina* L.) depicted in Clusius (1605: 83) together with the woodcut that was published in the HNB (Piso 1648: 99). He is comparing the Brazilian plant with this nonnative species in his description. Notice that four decades later, after the publication of the HNB, John Parkinson (1640: 1579) reproduced the same image of the root copied from Clusius in his work, as he often did with woodcuts that he borrowed from other authors. Piso did the same for the IURNM but combined it with the existing woodcut for this species. Pickel (2008: 57) identified it as *Smilax campestris* Griseb., but we think it could be *S. rufescens*, which is endemic to Brazil.

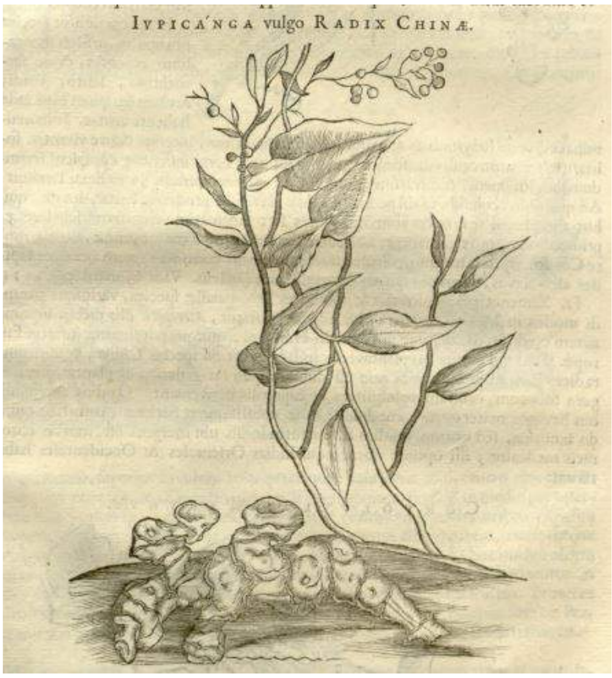

IV. *De Arboribus, fructibus, & herbis medicis*: 257

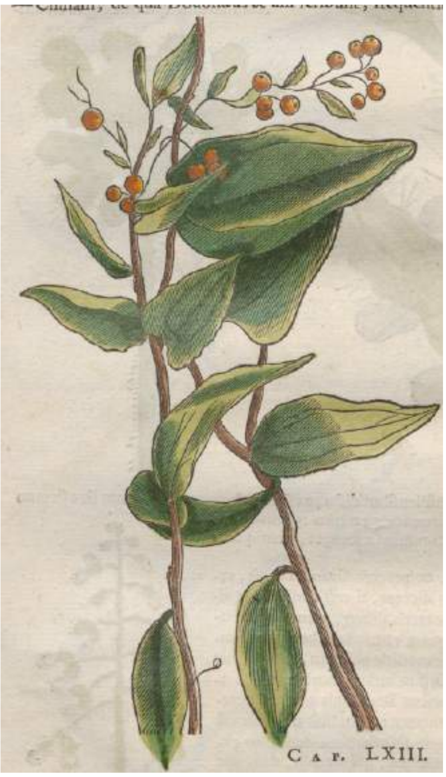

Smilax rufescens in the HNB (Piso 1648: 99)

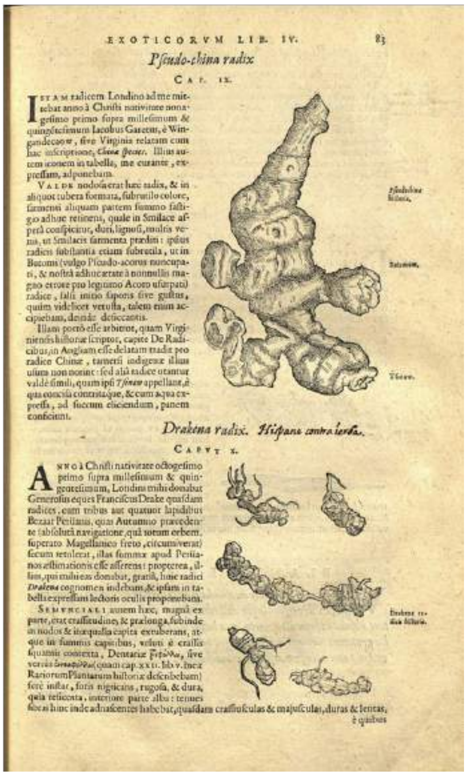

Pseudo-china radix, in *Exoticorum Libri Decem*, by Clusius (1605: 83)

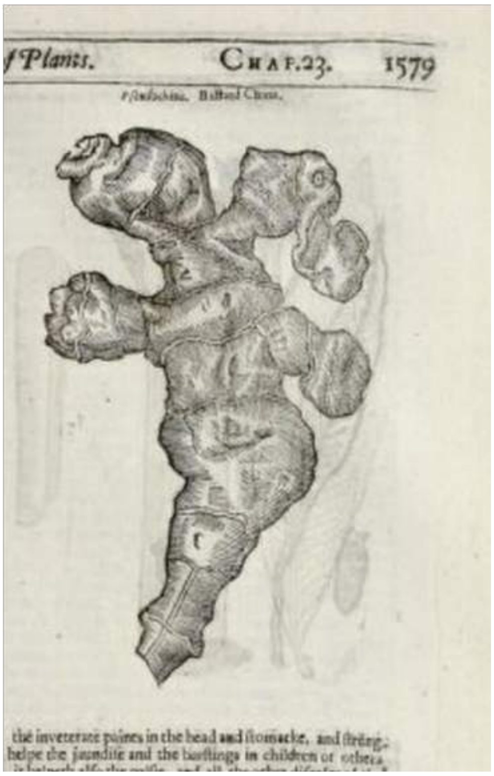

Smilax cf. pseudo*china* root in the *Theatrum botanicum* by Parkinson (1640: 1579)

Historia Naturalis & Medica Piso, 1658 Page number 261b

Vernacular name(s) Caapeba. Cipó de Cobras. Erva de Nossa Senhora

Species Cissampelos glaberrima St. Hil

Family Menispermaceae

Presence in the HNB Yes

Marcgrave (1648) 26a (different woodcut)

Piso (1648) 94b (different woodcut)

Notes

This plant is represented by two woodcuts. One of the images matches with the HNB image. The other figure is new, albeit slightly similar to the first woodcut but with larger roots that split in two. Piso (1658: 261) directed the reader to these different roots by citing this figure, and specifying that one of them becomes larger when it grows older. Intentionally or not, with his modifications, Piso highlighted medicinally valuable plant parts. Interestingly, Ole Worm published an image of the root of *C. glaberrima* in his *Museum Wormiamum* (1655: 157), called Raiz de Nossa Seinora, which came from Brazil. Worm (1655: 158) also added the woodcut from the HNB (*Caapeba*)- which he borrowed from Elzevier, who owned the HNB woodblocks at that time.

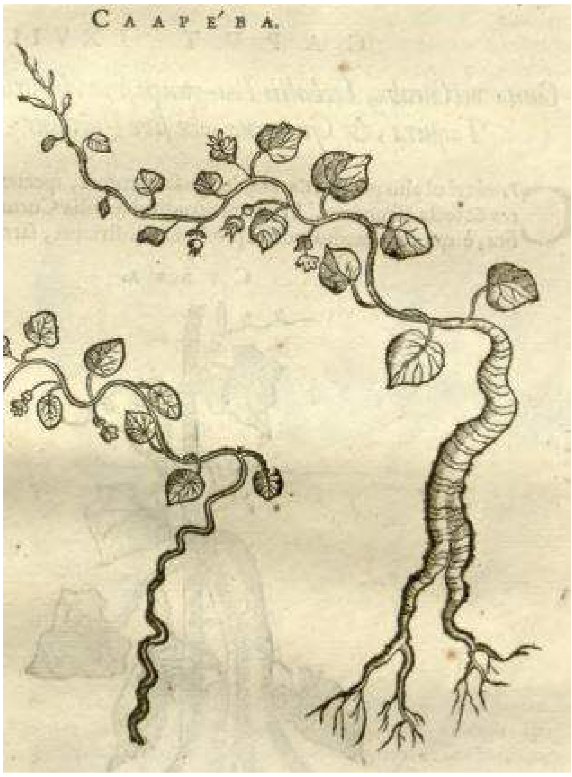

IV. *De Arboribus, fructibus, & herbis medicis*: 261b

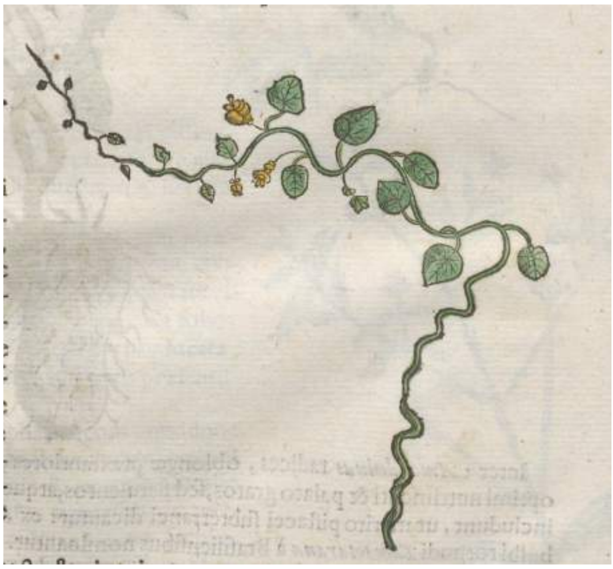

Cissampelos glaberrima in the HNB (Piso 1648: 94b)

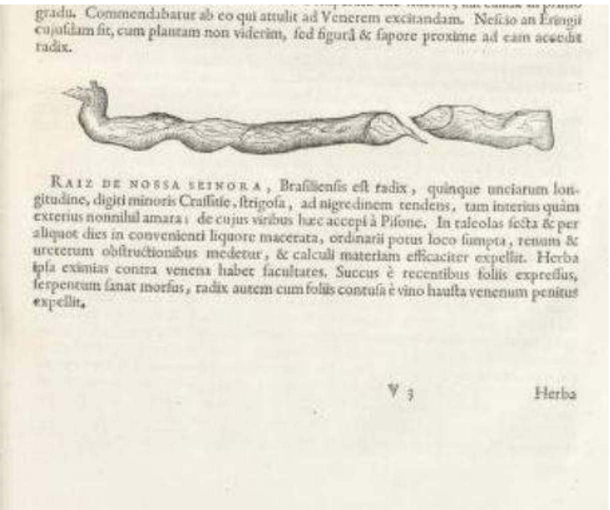

gradu. Commendabatur ab eo qui attulit ad Venerem excitandam. Neſcio an Eringii cujuſdam ſit, cum plantam non viderim, ſed figurâ & ſapore proxime ad eam accedit radix.

RAIZ DE NOSSA SEINORA, Braſilienſis eſt radix, quinque unciarum longitudine, digiti minoris Craſſitie, ſtrigoſa, ad nigredinem tendens, tam interius quàm exterius nonnihil amara; de cujus viribus hæc accepi à Piſone. In taleolas ſecta & per aliquot dies in convenienti liquore macerata, ordinarii potus loco ſumpta, renum & ureterum obſtructionibus medetur, & calculi materiam efficaciter expellit. Herba ipſa eximias contra venena habet facultates. Succus è recentibus foliis expreſſus, ſerpentum ſanat morſus, radix autem cum foliis contuſa è vino hauſta venenum penitus expellit.

V 3 Herba

Root of *C. glaberrima* in *Museum Wormianum* by . Worm (1655: 157)

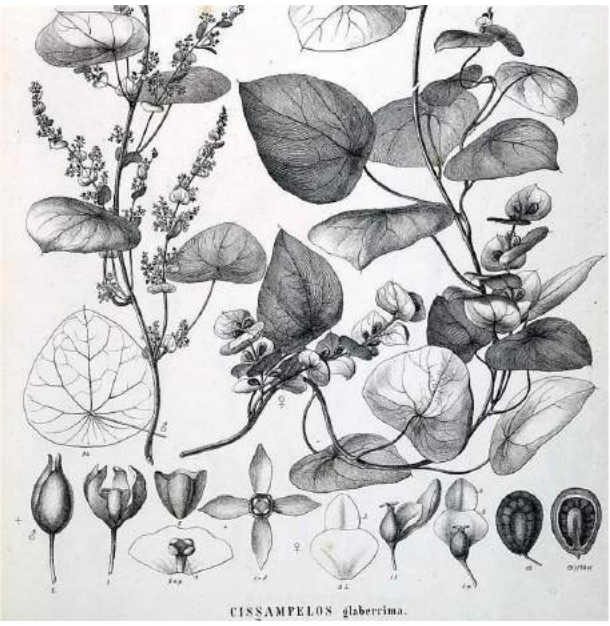

Engraving of *C. glaberrima* in Martius, C.F.P. von, Eichler, A.G., Urban, I., *Flora Brasiliensis* (1841-1872) Vol. 13 (1): 45

Historia Naturalis & Medica Piso, 1658 Page number 262

Vernacular name(s) Curua. Curubá

Species Sicana odorifera (Vell.) Naudin

Family Cucurbitaceae

Presence in the HNB Yes

Marcgrave (1648) 21 (only description)

Piso (1648)

Notes

We could not trace the woodcut to an existent source, but in De Laet's manuscript, there is an entry with the description of this plant in Marcgrave (1648: 21, *Curuba*) that was tagged with the word "Icon". Presumably, there must have been a drawing of this species that came from Brazil or somewhere else, but at least De Laet had it with him when arranging the manuscript. De Laet, however, did not use any image of this plant when he added the description of *Curuba* in the HNB. Eckhout depicted this species in one of his still-life paintings. The style resembles the images of vines and lianas depicted in the woodcuts of Dodoens, Clusius, and de l' Obel (Plantin woodblocks).

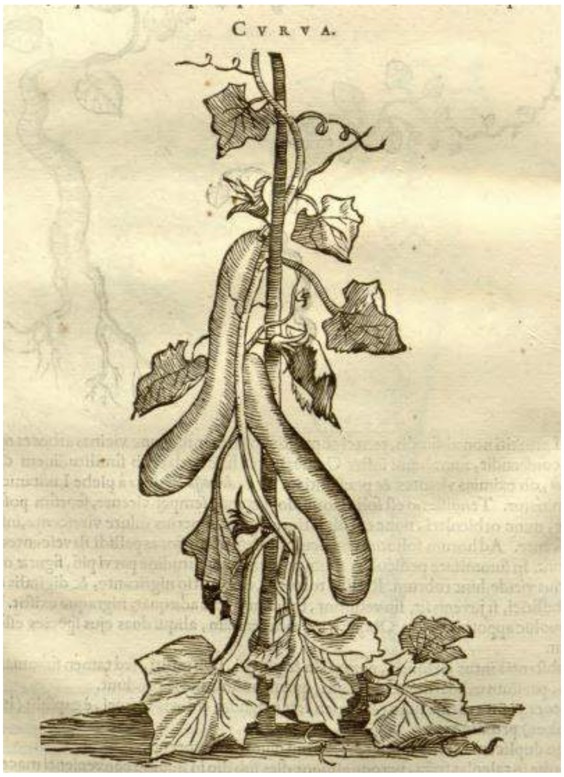

IV. *De Arboribus, fructibus, & herbis medicis*: 262

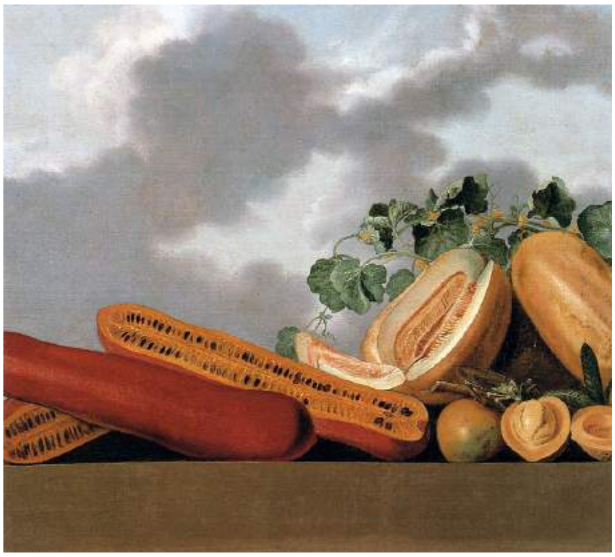

Eckhout still-life with Melons and other fruits, c. 1640

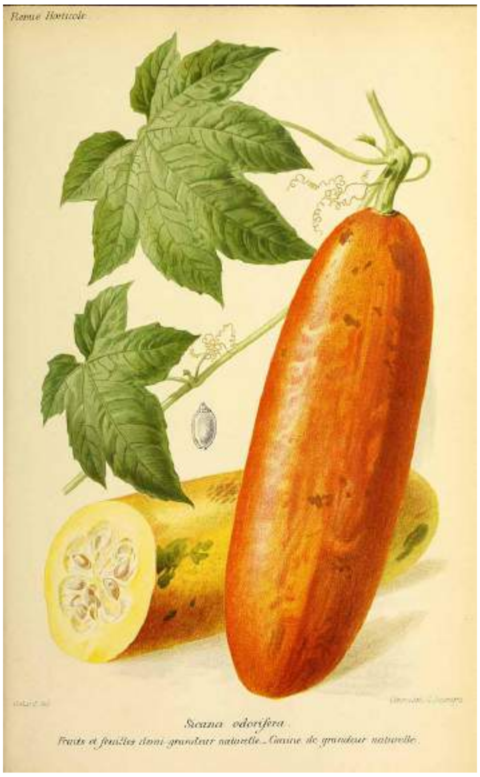

S. odorifera in *Revue horticole* (1890: Vol. 62). Retrieved from Plantillustrations.org

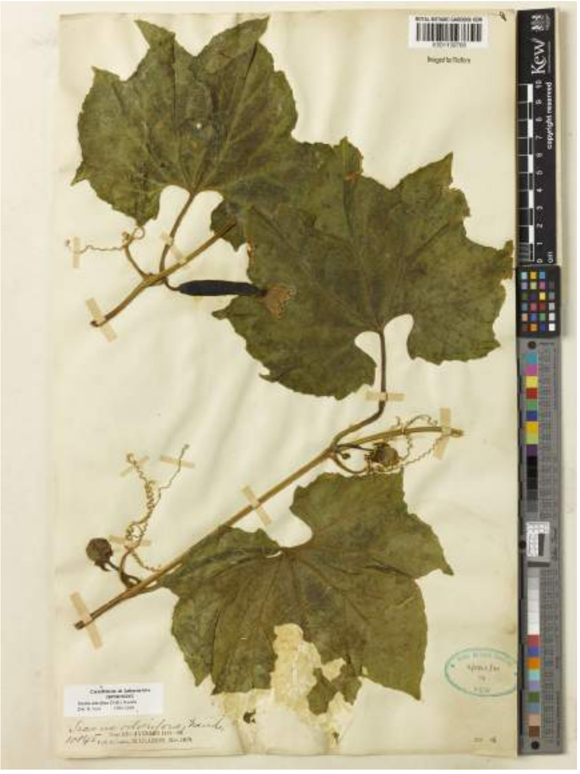

S. odorifera specimen from Kew's Herbarium - K001139708. Retrieved from Plants of the World Online

Historia Naturalis & Medica Piso, 1658 Page number 264b

Vernacular name(s) Guarerua-oba. Cucumer afininus. Pepino asinino

Species Cucumis anguria L.

Family Cucurbitaceae

Presence in the HNB Yes

Marcgrave (1648) 44 (only description)

Piso (1648)

Notes

Piso used the wrong image, as this would correspond to *Ecballium elaterium* (L.) A.Rich, of European distribution (Pickel 2008: 221). *E. elaterium* was depicted in the *New Kreüterbuch* by Fuchs (1543: 403), which woodcut was copied for the work of Dodoens (1553: Vol. I, 372). The lightly lobed and cordate leaves of Piso's image resemble those of the Mediterranean *E. elaterium*, but the tendrils are lacking in *E. elaterium*, although present in the woodcut. The fruits in Piso's woodcut resemble *E. elaterium* fruits and slightly those of *Momordica* species. In contrast, *C. anguria* bears tendrils and its leaves are deeply palmately lobed. Likely, Piso made up the woodcut, or as Pickel (2008) would put it, this would be one of his "fantasy" woodcuts.

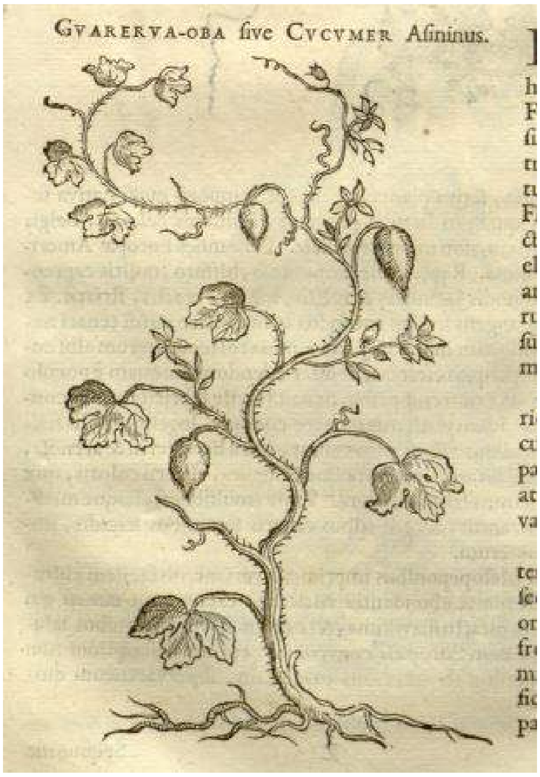

IV. *De Arboribus, fructibus, & herbis medicis*: 264b

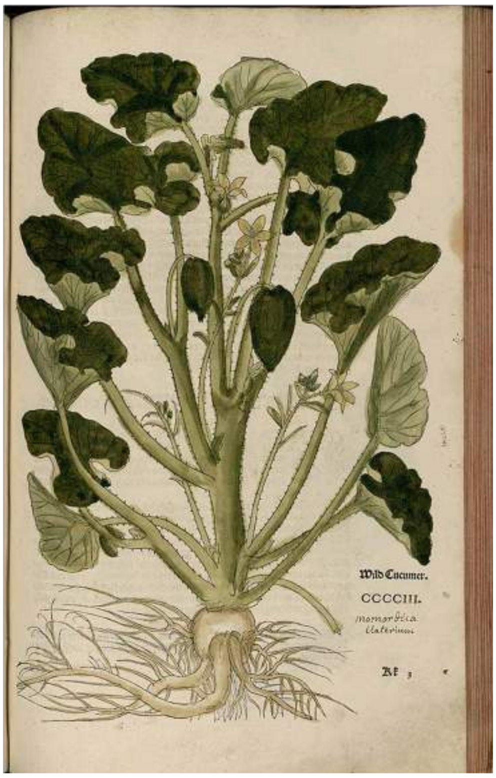

Ecballium elaterium in *New Kreüterbuch* by Fuchs (1543: 403)

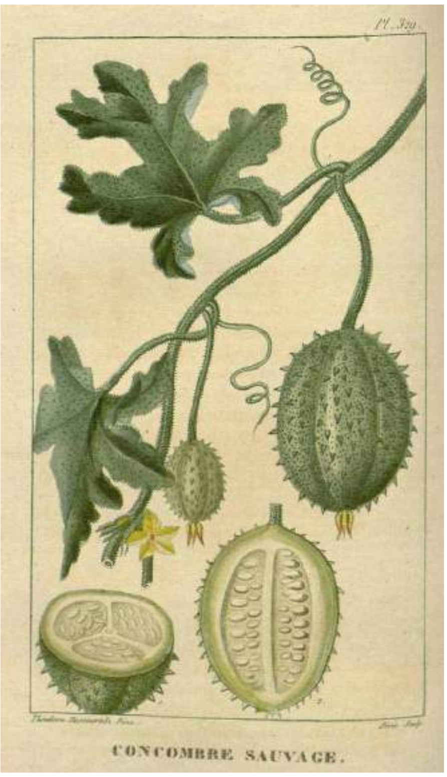

Cucumis anguria in *Flore [pittoresque et] médicale des Antilles* by Descourtilz, M.E., (1827: Vol V, 329)

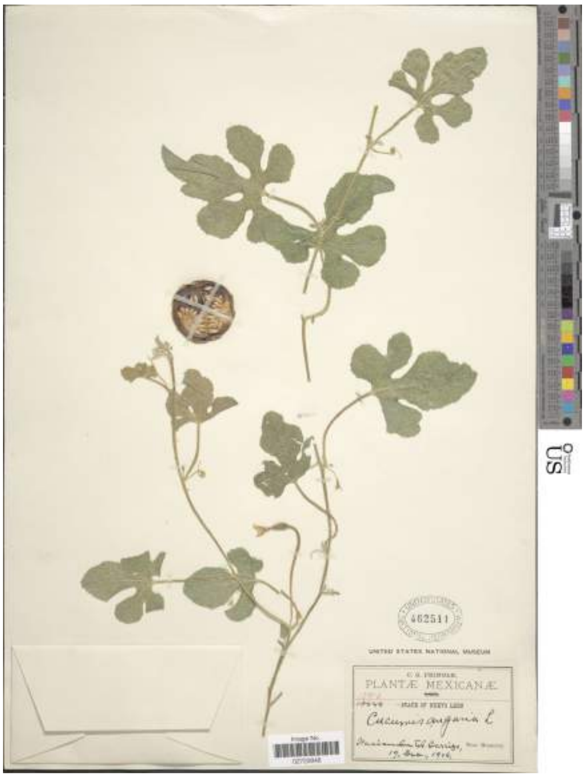

Specimen of "*C. anguria*" by Cyrus G. Pringle (CC0 1.0)

Historia Naturalis & Medica Piso, 1658 Page number 265a

Vernacular name(s) Arbuscula coralloides

Species Plexaura sp. / Plexaurella sp.

Family Plexauridae

Presence in the HNB No

Marcgrave (1648)

Piso (1648)

Notes

This woodcut represents a coral, formed by a colony of marine invertebrates, and identified at the genus level by marine biologists Nicole de Voogd and Eduardo Hajdult. Piso categorized it as a plant because he thought it was an alga as it came from the sea. He (1658: 265) mentioned how Spanish and Flemish magnates gathered it, becoming hard when dry, to build their houses and caves. In contrast, local inhabitants used it as medicine, but Piso disregarded this use as "barbarian". This report, as many others throughout the HNB/IURNM, is proof of early bioprospecting practices by Europeans in a colonial context. The woodcut is very similar to the image in the *Exoticorum Libri Decem* by Clusius (1605: 123) and it was likely made after it.

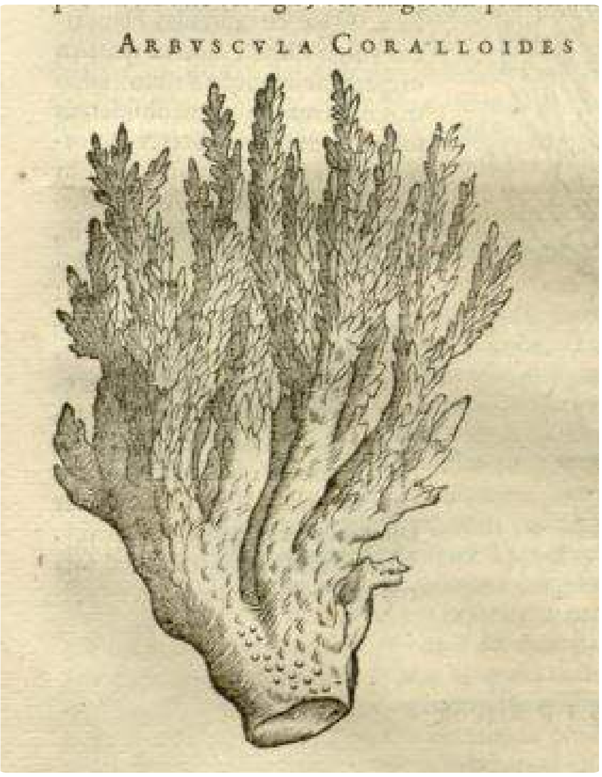

IV. *De Arboribus, fructibus, & herbis medicis*: 265a

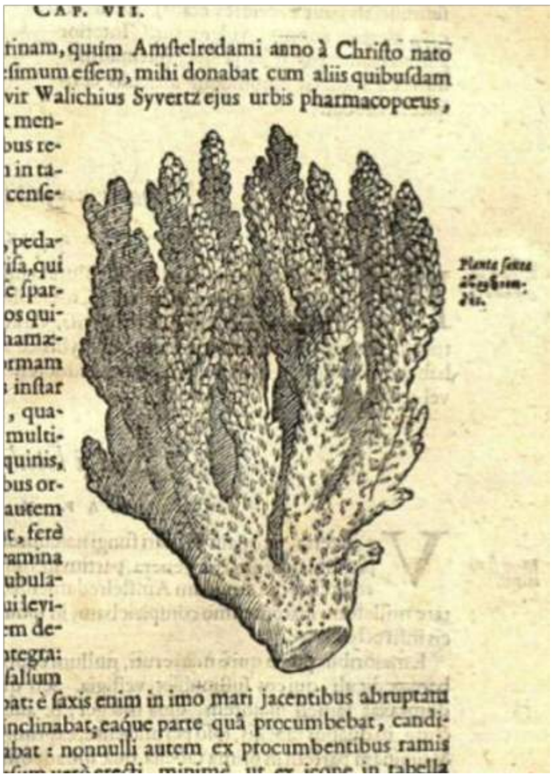
CAP. VII.
tinam, quùm Amstelredami anno à Christo nato
ssimum essem, mihi donabat cum aliis quibusdam
vir Walichius Syvertz ejus urbis pharmacopœus,
t men-
bus re-
in ta-
cense-
, peda-
isa, qui
e spar-
os qui-
hamæ-
rmam
instar
, qua-
multi-
quinis,
bus or-
autem
t, ferè
amina
ubula-
ui levi-
m de-
tegra:
falsum
bat: è saxis enim in imo mari jacentibus abruptam
inclinabat, eaque parte quâ procumbebat, candi-
abat : nonnulli autem ex procumbentibus ramis

Exoticorum Libri Decem by Clusius (1605: 123)

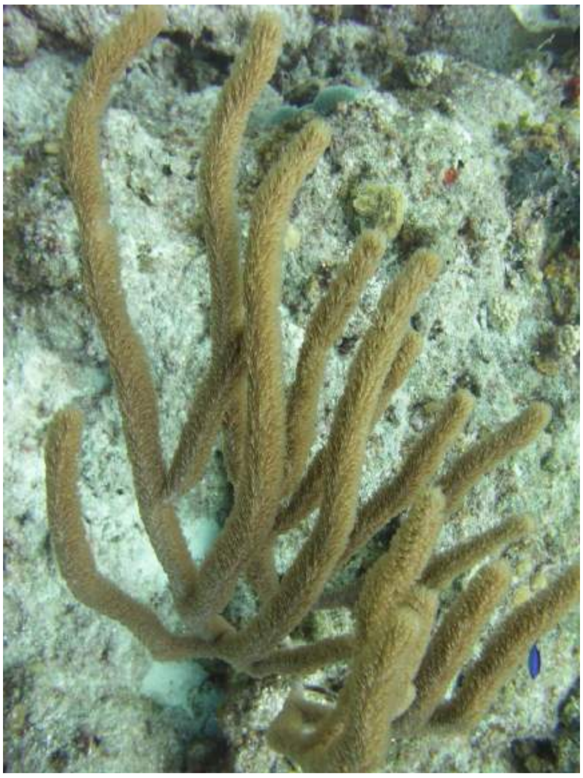

"Bent sea rod (*Plexaura flexuosa*) at Paradise Reef, Cozumel" by Herrera Family (CC BY-ND 2.0)

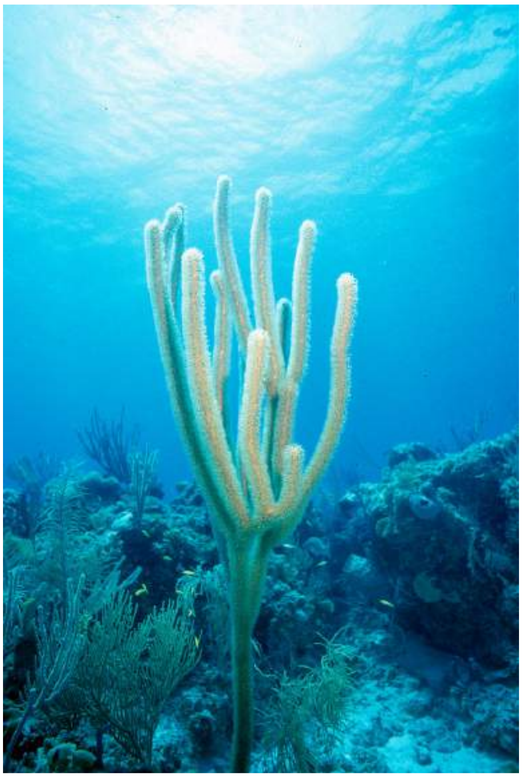

Plexaurella nutans "Giant slit-pore sea rod" by August Rode (CC BY-NC-SA 2.0)

Historia Naturalis & Medica Piso, 1658 Page number 265b

Vernacular name(s) Spongiosa

Species Clathria cf. nicoleae

Family Microcionidae

Presence in the HNB No

Marcgrave (1648)

Piso (1648)

Notes

As with the previous woodcut, Piso categorized this organism as a plant, even though it is a sponge, a member of the basal animal clade. Unlike the previous woodcut of *Arbuscula coralloides*, which was taken from Clusius' work, this could have been made after a specimen from the Brazilian coast. Piso mostly described medicinal and other useful plants with a certain pragmatism, with a utilitarian mindset. Unexpectedly, he mentioned and depicted this sponge because of its beauty and rarity status, as he did not find any utility for it (Piso 1658: 266).

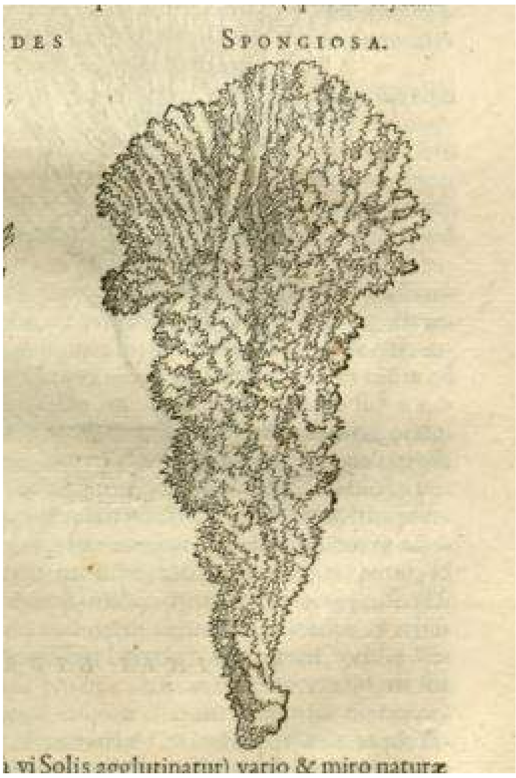

IV. *De Arboribus, fructibus, & herbis medicis*: 265b

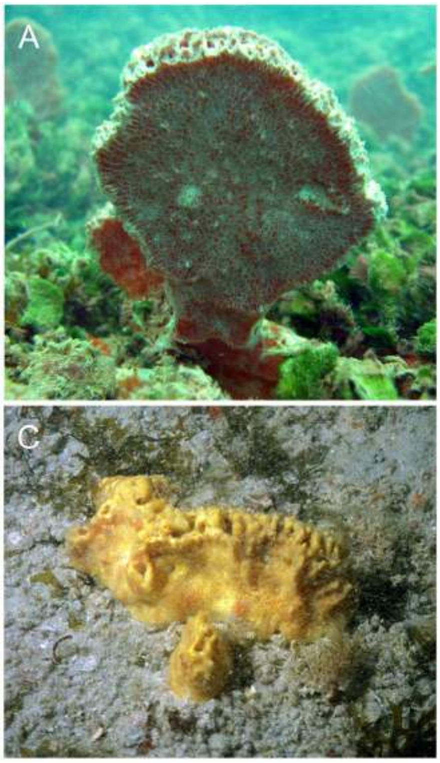

Clathria (Clathria) nicoleae sp. nov. in situ in northeast Brazil. Figure 2 retrieved from Barros at al. 2013 DOI: 10.11646/zootaxa.3640.2.10

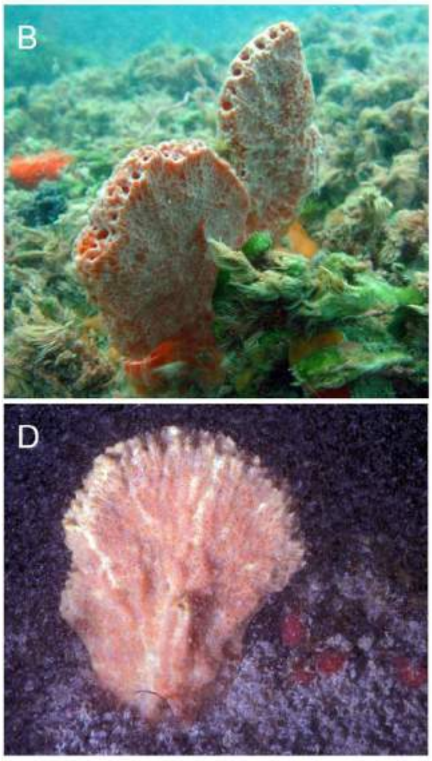

Clathria (Clathria) nicoleae sp. nov. in situ in northeast Brazil. Figure 2 retrieved from Barros at al. 2013 DOI: 10.11646/zootaxa.3640.2.10

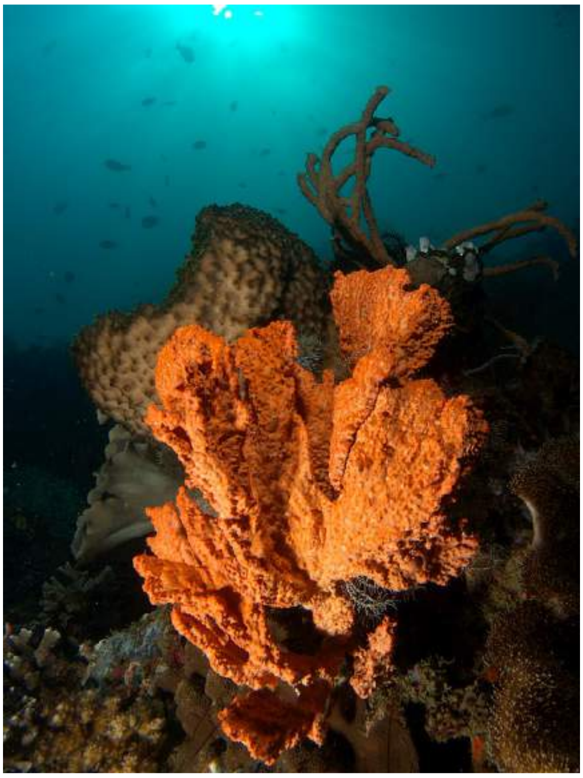

Clathria tuberosa (Sponge) by Nick Hobgood (CC-BY-SA-3.0)

Historia Naturalis & Medica Piso, 1658 Page number 266

Vernacular name(s) Sargaço. Steen-croost. Lentilha marinha

Species Sargassum vulgare C.Agardh

Family Sargassaceae (Algae)

Presence in the HNB No

Marcgrave (1648)

Piso (1648)

Notes

Piso stated that this organism is not an alga but an "arbuscula baccifera" (berry-bearing shrub). He must have categorized it as such because of its multiple round berry-like structures. This is indeed seaweed, and those "berries" are pneumatocysts: gas-filled bladders that aid the fronds to float to facilitate photosynthesis. According to Pickel (2008: 27), Piso provided a figure that is made after a sponge located on the Dutch shores. Piso (1658: 266) described how the large extensions of these organisms damaged the ships of the Flemish people when they returned from the American colonies. According to Pickel (2008: 27), Piso did not see the "root" of this organism as this figure was made after one of the fragments that ended up in the North Sea after being carried out by the currents.

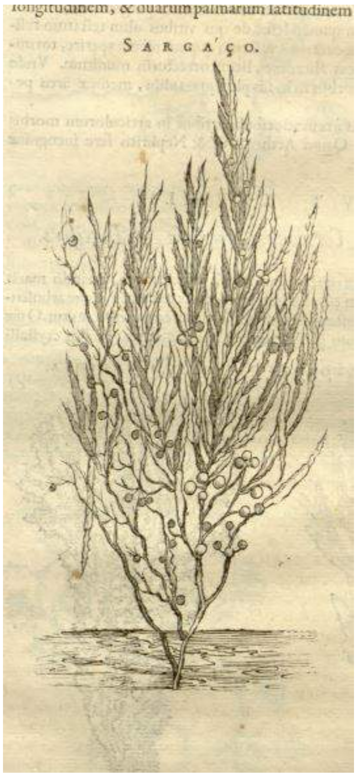

IV. *De Arboribus, fructibus, & herbis medicis*: 266

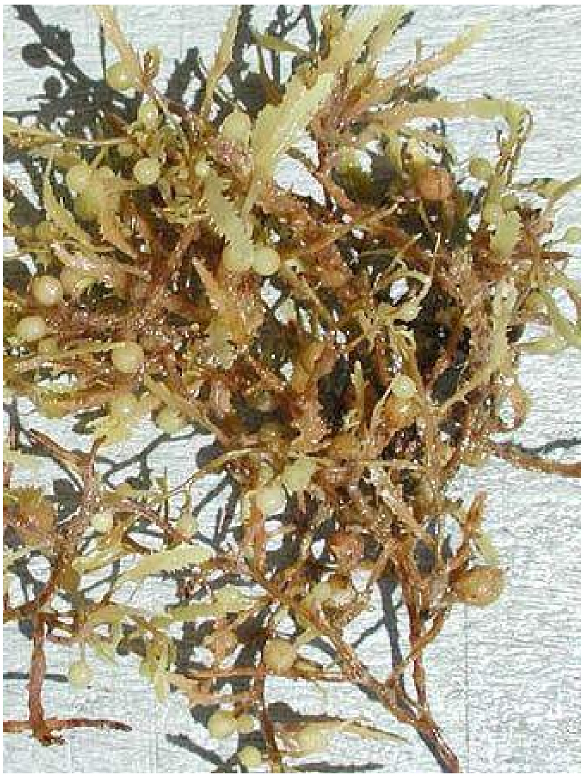

Sargassum and its pneumatocysts.
"Sargassum weeds closeup.jpg". Retrieved from Wikimedia Commons (Public Domain)

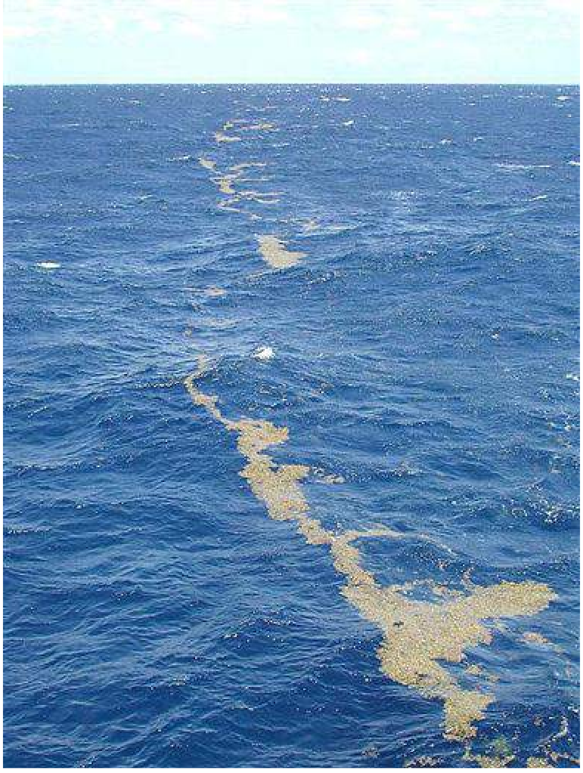

Sargassum seaweed floating in the Sargasso Sea.
Sargassotang i Sargassohavet
Av Ocean Explorer/NOAA (Public Domain)

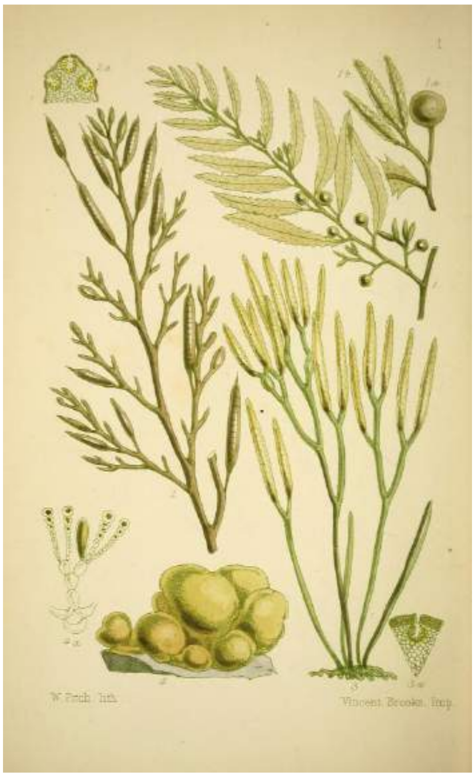

S. vulgare in *British sea-weeds: an introduction to the study of the marine Algae of Great Britain, Ireland ad the Channel Islands* by Gray (1867)

Historia Naturalis & Medica Piso, 1658 Page number 302c

Vernacular name(s) Tangaraca II

Species Palicourea bracteocardia (DC.) Delprete & J.H.Kirkbr.

Family Rubiaceae

Presence in the HNB Yes

Marcgrave (1648) 60 (only description)

Piso (1648) 92b

Notes

Most of the leaves in the main branch are lacking, compared to the woodcut in the HNB. Likely, Piso ordered to modify the woodblock to show the opposite leaves as described by Marcgrave. However, if we consider that the leaves are spirally arranged, the depiction of this species was not incorrect. The painter left the opposite leaves out perhaps to avoid a dense image (see the *Theatrum*). It is unfortunate that the figure of the red and isolated flower in the HNB (bottom left), was also left out. This seems to be one of the multiple flowers that are part of the inflorescence, although it is wrongly attached to a stem with leaves. This flower was likely drawn by using the "megascope", like the one that Marcgrave often used to draw little details of plants and insects.

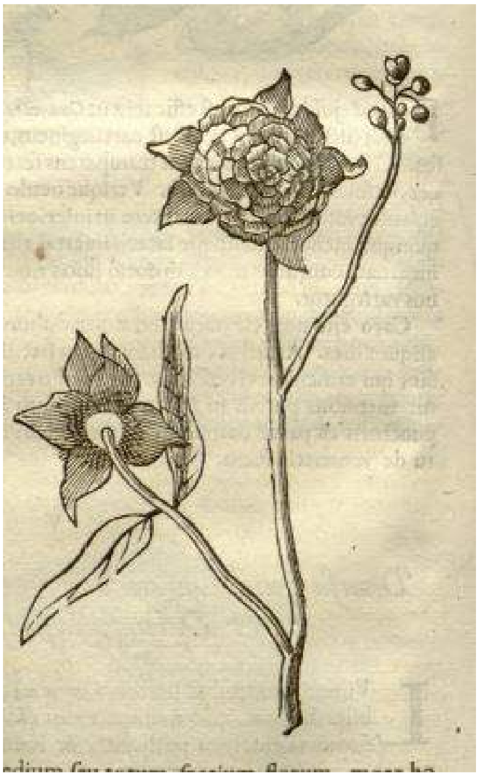

V. *De Noxiis & venenatis, corumque Antidotis*: 302c

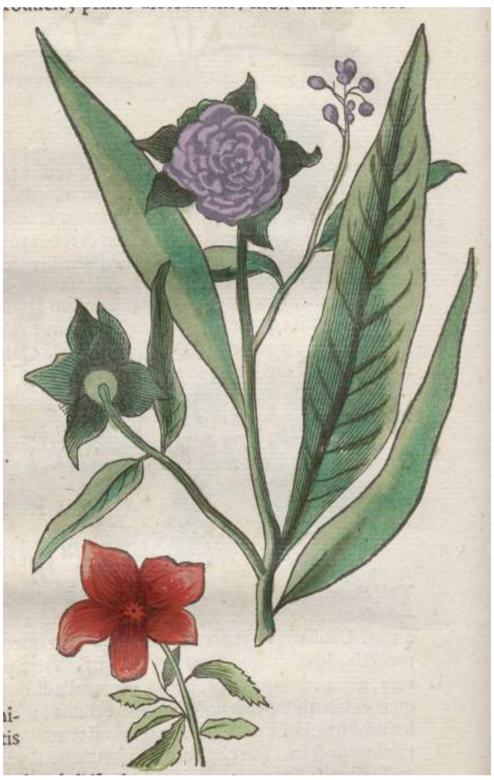

P. bracteocardia in the HNB (Piso 1648: 92b)

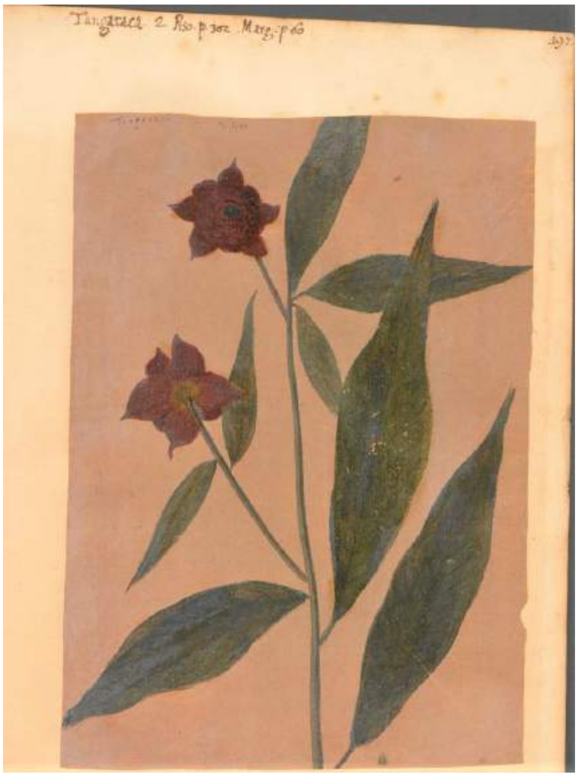

P. bracteocardia in the *Theatrum Rerum Naturalium* (f. 197)

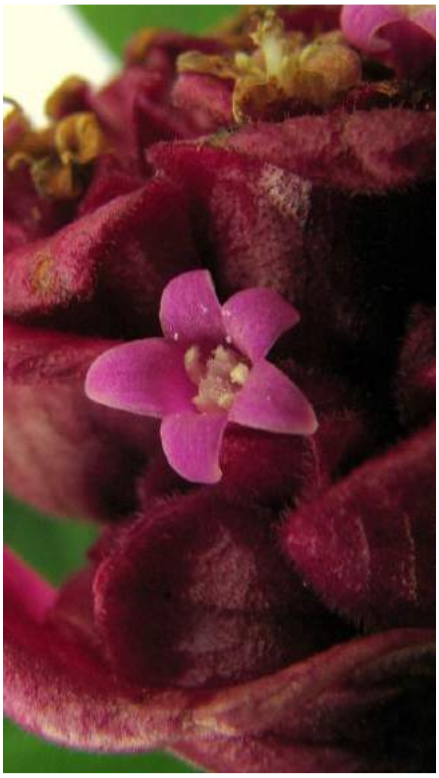

Flower of "*Psychotria bracteocardia*" by Alex Popovkin, Bahia, Brazil (CC BY-NC-SA 2.0)

Historia Naturalis & Medica Piso, 1658 Page number 307a

Vernacular name(s) Mucuná-guaçu

Species Macropsychanthus marginatus (Benth.) L.P.Queiroz & Snak

Family Fabaceae

Presence in the HNB Yes

Marcgrave (1648)

Piso (1648) 48 (only description)

Notes

The pod of this species (formerly *Dioclea marginata*) is depicted in de l' Obel (1581: vol. 2, p. 62) and De Laet (1633: 569, 1640: 504). There is an oil painting of this species in the *Theatrum* (which includes the leaves and a couple of seeds). The images from De Laet and the *Theatrum* do not bear a very strong resemblance to the woodcut in the IURNM. However, this woodcut resembles a bit the pod depicted in de l' Obel (the remnants of the style are imperceptible and the silique is attached to the pedicel), although the remnants of the calyx are only present in Piso (1658) and de l' Obel included two siliques, and one of them is slightly open. Piso describes in detail the fruit of *M. marginatus*, so it seems he knew the plant well and it is possible that the woodcut was made after a bean gathered in Brazil.

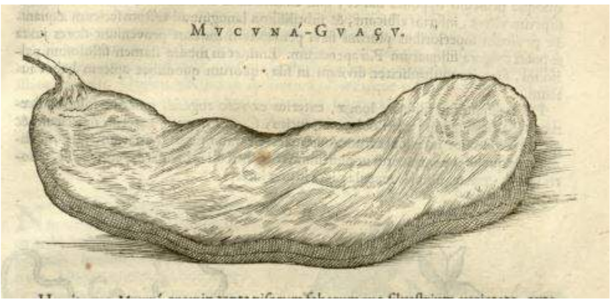

V. *De Noxiis & venenatis, corumque Antidotis*: 307a

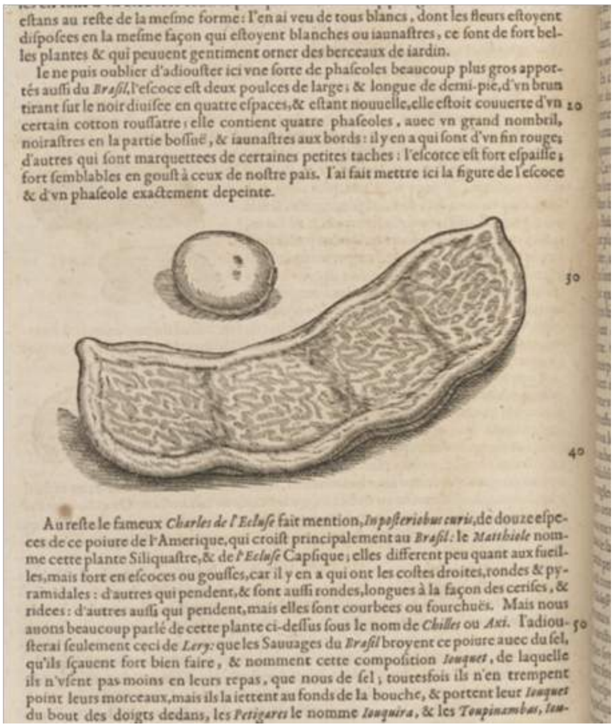

eſtans au reſte de la meſme forme: I'en ai veu de tous blancs, dont les fleurs eſtoyent diſpoſees en la meſme façon qui eſtoyent blanches ou iaunaſtres, ce ſont de fort belles plantes & qui peuuent gentiment orner des berceaux de iardin.

Ie ne puis oublier d'adiouſter ici vne ſorte de phaſeoles beaucoup plus gros apportés auſſi du *Braſil*, l'eſcoce eſt deux poulces de large; & longue de demi-pie, d'vn brun tirant ſur le noir diuiſee en quatre eſpaces, & eſtant nouuelle, elle eſtoit couuerte d'vn certain cotton rouſſatre; elle contient quatre phaſeoles, auec vn grand nombril, noiraſtres en la partie boſſuë, & iaunaſtres aux bords: il y en a qui ſont d'vn fin rouge; d'autres qui ſont marquettees de certaines petites taches: l'eſcorce eſt fort eſpaiſſe; fort ſemblables en gouſt à ceux de noſtre pais. I'ai fait mettre ici la figure de l'eſcoce & d'vn phaſeole exactement depeinte.

Au reſte le fameux *Charles de l'Ecluſe* fait mention, *In poſterioribus curis*, de douze eſpeces de ce poiure de l'Amerique, qui croiſt principalement au *Braſil*: le *Matthiole* nomme cette plante Siliquaſtre, & de *l'Ecluſe* Capſique; elles different peu quant aux fueilles, mais fort en eſcoces ou gouſſes, car il y en a qui ont les coſtes droites, rondes & pyramidales: d'autres qui pendent, & ſont auſſi rondes, longues à la façon des ceriſes, & ridees: d'autres auſſi qui pendent, mais elles ſont courbees ou fourchuës. Mais nous auons beaucoup parlé de cette plante ci-deſſus ſous le nom de *Chilles* ou *Axi*. I'adiouſterai ſeulement ceci de *Lery*: que les Sauuages du *Braſil* broyent ce poiure auec du ſel, qu'ils ſçauent fort bien faire, & nomment cette compoſition *Ieuquet*, de laquelle ils n'vſent pas moins en leurs repas, que nous de ſel; toutesfois ils n'en trempent point leurs morceaux, mais ils la iettent au fonds de la bouche, & portent leur *Ieuquet* du bout des doigts dedans, les *Petigares* le nomme *Iouquira*, & les *Toupinambas*, *Ieu-*

Pod and seed of *M. marginatus* in *L'histoire du nouveau monde ou Description des Indes Occidentales*, by De Laet (1640: 504)

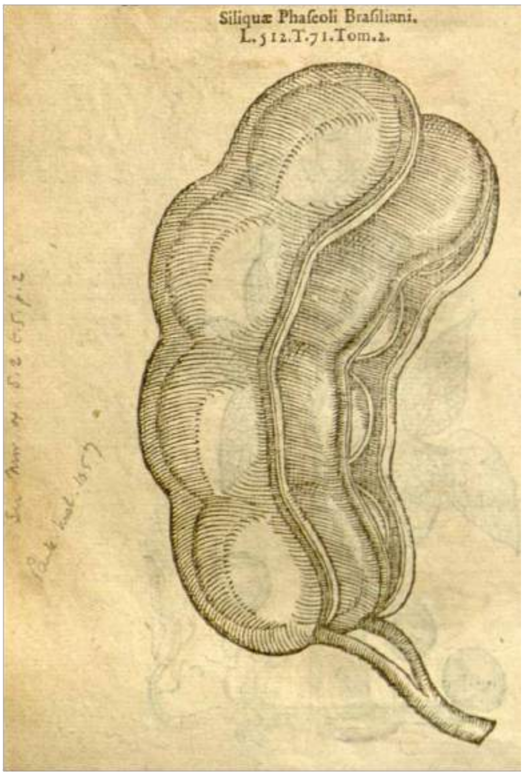

Pod of *M. marginatus* in *Plantarum seu stirpium icones* by Obel (1581: Vol. II, 62)

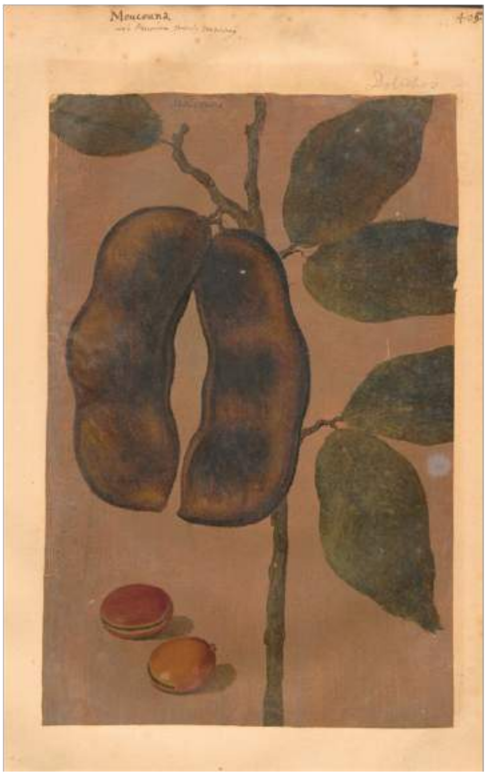

M. marginatus in the *Theatrum Rerum Naturalium* (f. 405)

Historia Naturalis & Medica Piso, 1658 Page number 308a

Vernacular name(s) Ahoay

Species Cascabela thevetia (L.) Lippold

Family Apocynaceae

Presence in the HNB Yes

Marcgrave (1648) 271 (annotations)

Piso (1648) 49 (only description)

Notes

This woodcut depicts the hanging fruits of *C. thevetia* much larger than in nature. This resembles the fruits depicted in the woodcut in *Les singularitez de la France antarctique* by André Thevet (1558: 66v), where he showed a tree with different leaves than *C. thevetia* and hanging fruits. The leaves in Piso, although less oblong than in Thevet, do not match either with the linear-lanceolate leaves of this species. Jean De Léry was, as Thevet, one of the first Europeans to mention and portray these seeds in their treatises (tied with cotton strings as ornaments). They both related them to the dances of Indigenous peoples and the sound that the seeds made when they move (as a rattle). Piso also mentioned this fact, but he focused on the poisonous properties of the fruits.

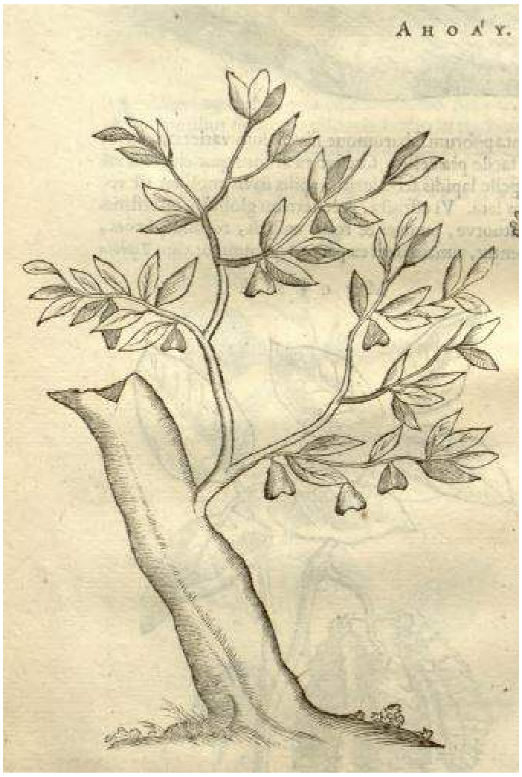

V. *De Noxiis & venenatis, corumque Antidotis*: 308a

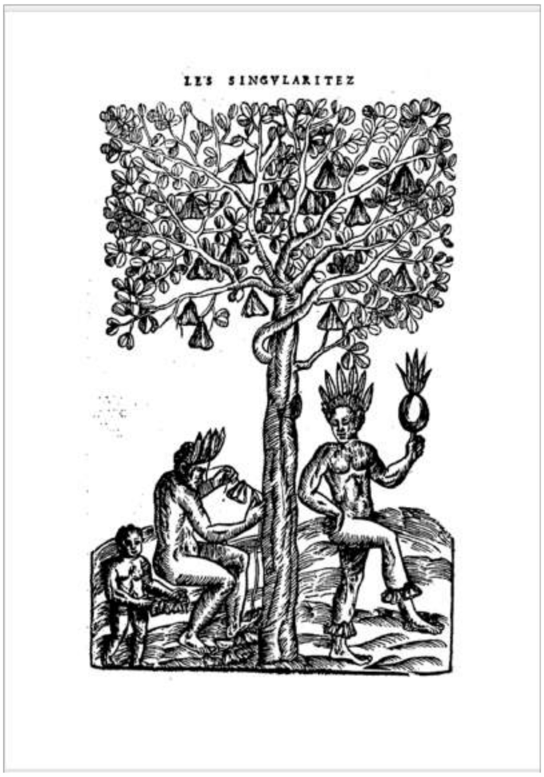

Les singularitez de la France antarctique by Thevet (1558: 66v)

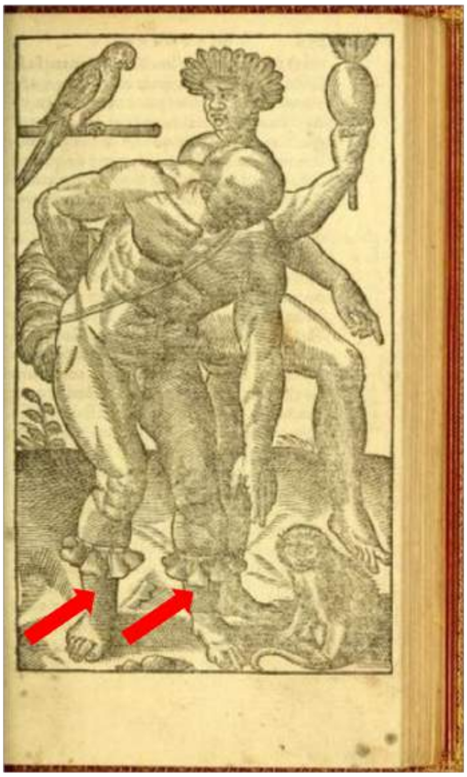

Ankle bracelets made of *C. thevetia* seeds in *Histoire d'un voyage fait en la terre de Brésil* by De Léry (1578: 275)

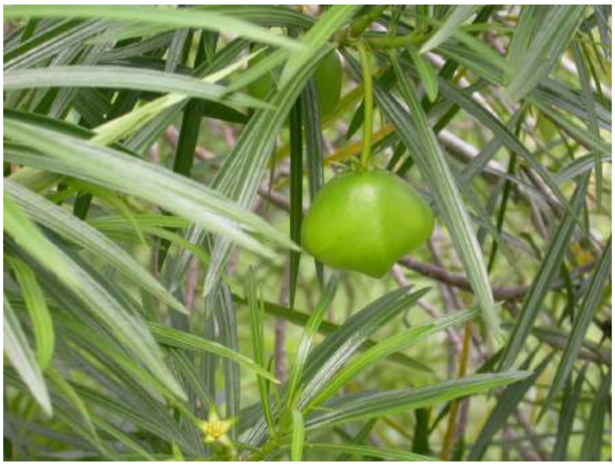

Fruit and leaves. "*Cascabela thevetia* (APOCYNACEAE)" by Scamperdale (CC BY-NC 2.0)

Historia Naturalis & Medica Piso, 1658 Page number 308b

Vernacular name(s) Ahoay

Species Cascabela thevetia (L.) Lippold

Family Apocynaceae

Presence in the HNB Yes

Marcgrave (1648) 271 (annotations)

Piso (1648) 49 (only description)

Notes

This woodcut was copied from de l' Obel (1581, 1591: Vol. II, 185), or from Clusius (1605: 232), who likely used the same woodblock, owned by Plantin printers (notice the reverse format in Piso in comparison to those authors). This image was also copied by John Gerard (1597: 1361, 1633: 1545), probably after de l' Obel woodcut. De Laet (1633: 562, 1640: 497) mentioned this plant, but he only used the image with the more triangular seeds, which was dismissed by Piso. According to Pickel (2008: 189), Piso took the wrong woodcut (he should have copied the one from De Laet), as the bracelet' seeds look like those of *Ormosia* sp. or other Fabaceae species. However, the hanging seeds in the bottom image seem to be those of *C. thevetia*. The bracelet above is bearing those and the image below is a close-up of the seeds.

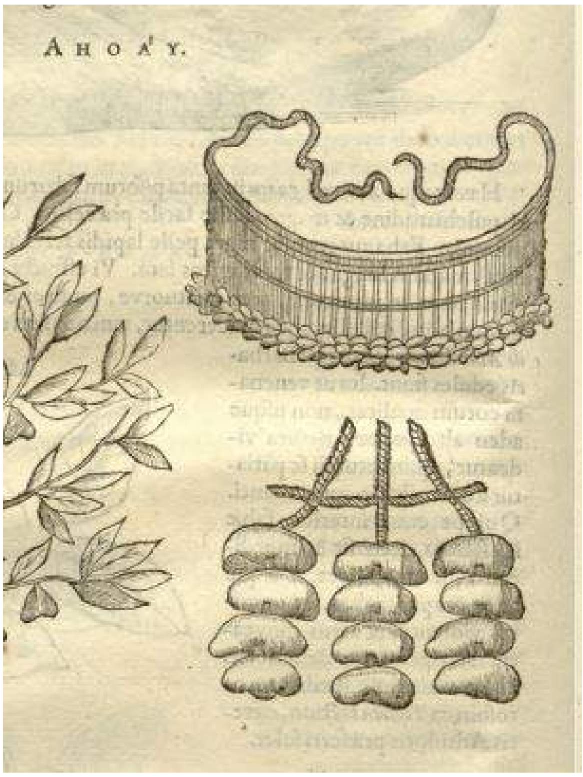

V. *De Noxiis & venenatis, corumque Antidotis*: 308b

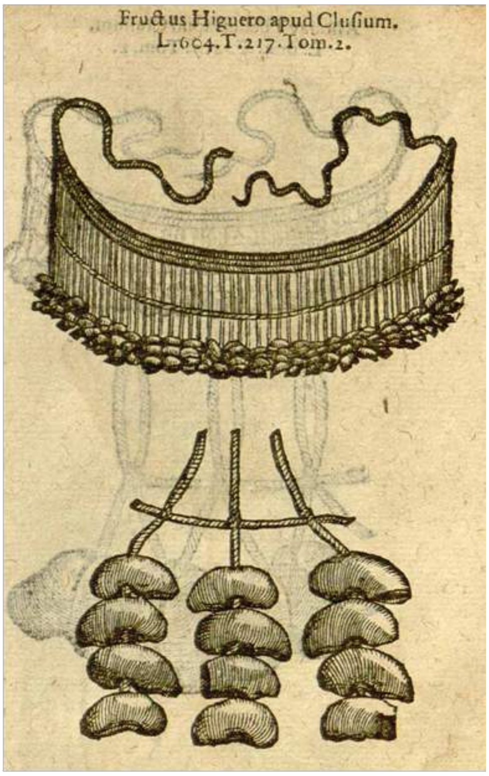

Seeds of *C. thevetia* in *Plantarum seu stirpium icones* by Obel (1581: Vol. II, 185)

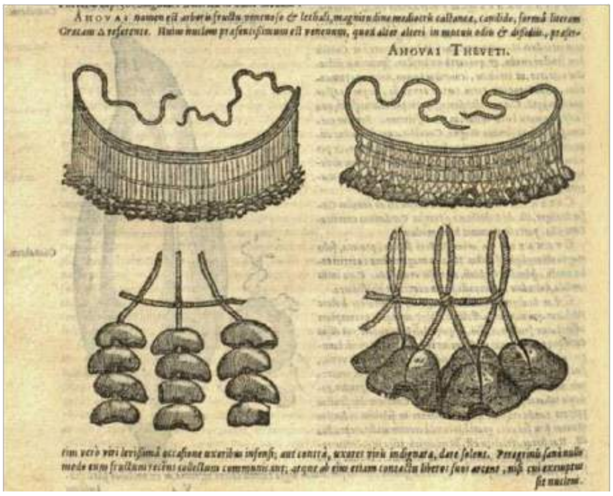

Two different ornaments made of seeds of *C. thevetia* in *Exoticorum Libri Decem* by Clusius (1605: 232)

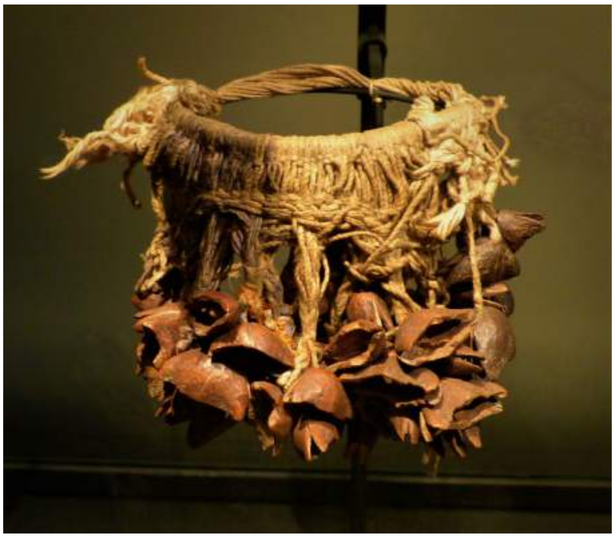

Ankle rattle from the Wayana peoples made with *C. thevetia* seeds. Volkenkunde Museum, Leiden. Photo by Mike Nienart, edited by Lotte Mens

Historia Naturalis & Medica Piso, 1658 Page number 309

Vernacular name(s)	Carapucú. Fungi
Species	Copelandia cyanescens (Berk. & Broome) Singer
Family	Bolbitiaceae

Presence in the HNB	Yes
Marcgrave (1648)	
Piso (1648)	47 (only description)

Notes

This woodcut image, as noticed by Pickel (2008: 27), does not correspond to a Brazilian mushroom. Piso made it after *Amanita muscaria* (L.) Lam. depicted in the section on mushrooms (*Fungorum*) in the *Rariorum Plantarum* by Clusius (1601). The fungi described by Piso is depicted in the *Theatrum* (*Ibibura*) and identified as *Copelandia cyanescens* (Bolbitiaceae) thanks to mycologists Jorinde Nuytinck and Thomas W. Kuyper. This fungus was described as poisonous by Piso, who did not mention that is hallucinogenic (Wartchow et al. 2010). However, he indicated: "from these mushrooms must have originated the witty sayings of Martial and Juvenal on the mushrooms that are eaten recklessly". The allusion to the satirical Roman authors may imply a certain connection to its psychotropic properties.

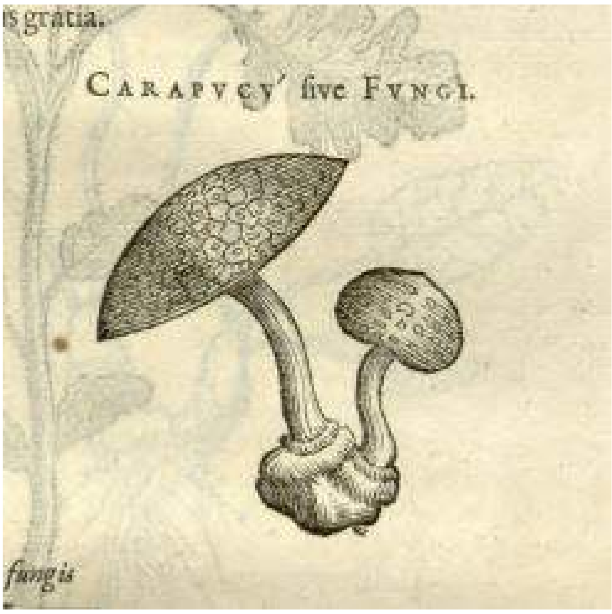

V. *De Noxiis & venenatis, corumque Antidotis*: 309

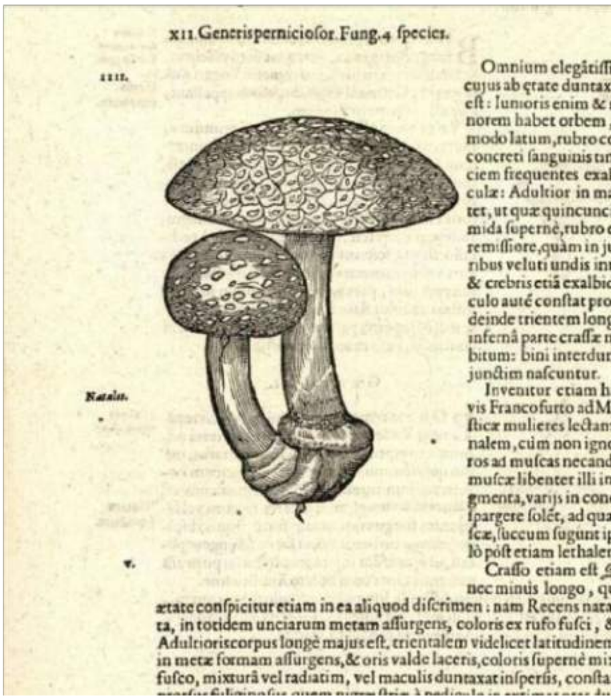

Amanita muscaria in *Rariorum Plantarum* by Clusius (1601: CCLXXX - Fungorum)

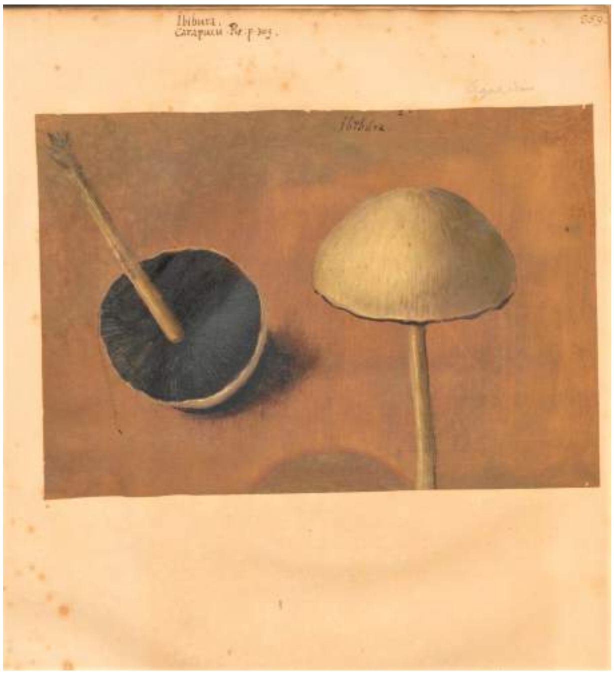

Copelandia cyanescens in the *Theatrum Rerum Naturalium* (f. 359)

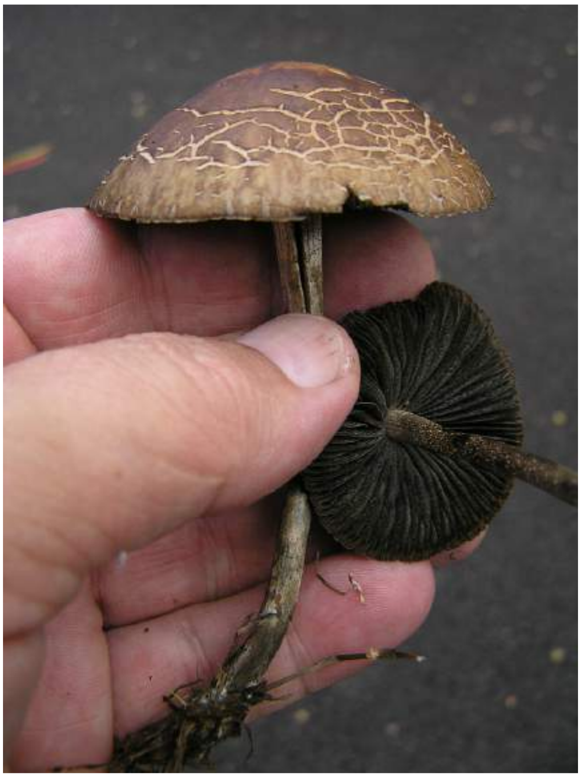

"*Copelandia cyanescens*" by Scot Nelson (CC0 1.0)

Historia Naturalis & Medica Piso, 1658 Page number 314

Vernacular name(s) Limonia malus americana. Antidotalis

Species Citrus × aurantiifolia (Christm.) Swingle

Family Rutaceae

Presence in the HNB No

Marcgrave (1648)

Piso (1648)

Notes

Images of citrus and lemon plants were circulating in older herbals, such as in *Plantarum seu Stirpium* by de l' Obel (1581: Vol. II, p. 144) and in *Rariorum Plantarum* by Clusius (1601: 7), who used the same woodcut than de l' Obel. The origin of this particular woodcut is uncertain, but possibly Piso ordered it to be made. He indicated in the text: "This is what I mainly mean about the roots of the lemon tree, whose engraving is the only one I present here...". This species of lemon is also depicted in the *Libri Principis* (f. 103 [83]), the *Misc. Cleyeri* (f. 51r), and portrayed in one of the still-life paintings by Eckhout based on the sketch from the *Misc. Cleyeri*.

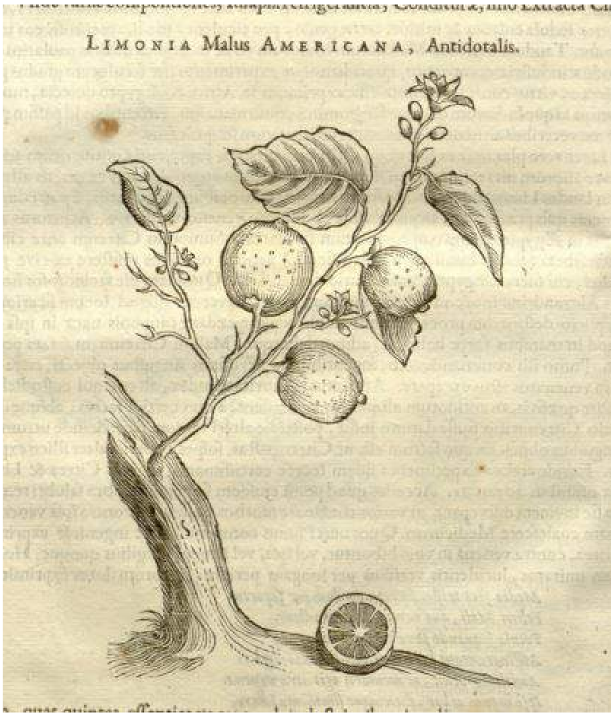

V. *De Noxiis & venenatis, corumque Antidotis*: 314

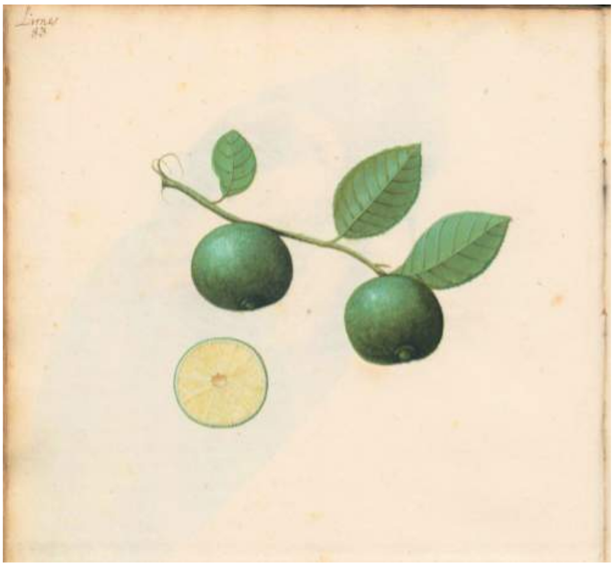

Citrus x *aurantiifolia* watercolor in the *Libri Principis* f. 103 [83]

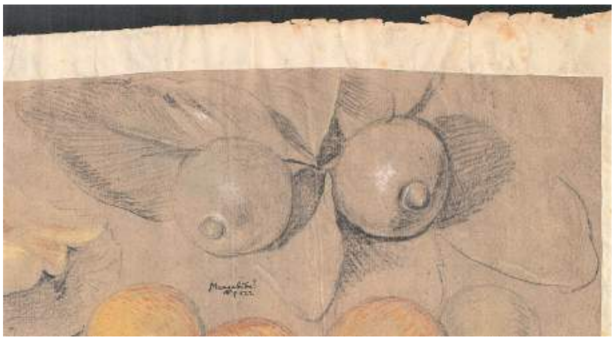

Sketch of *C.* x *aurantiifolia* in the *Miscellanea Cleyeri* c.1637-44: 51r

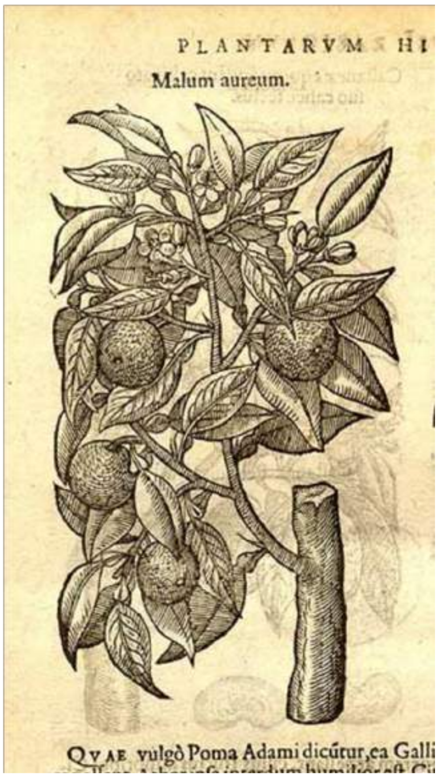

Citrus plant, perhaps *C.* x *aurantiifolia in Rariorum Plantarum* by Clusius (1601: 7)

Historia Naturalis & Medica Piso, 1658 Page number 319

Vernacular name(s)	Guainumbi II
Species	Psittacanthus dichrous (Mart. ex Schult. & Schult.f.)
Family	Loranthaceae

Presence in the HNB	Yes
Marcgrave (1648)	63a (description and woodcut of the plant, but not the bird)
Piso (1648)	

Notes

The plant that is eaten by the bird is *Psittacanthus dichrous*, which is described in Marcgrave (1648: 63), who mentioned that "In the flowers of this shrub, the birds Wanombugh are frequently seen, where you can easily grab them)". The bird *Guainumbi* [Wanombugh] could be the hummingbird *Polytmus guainmubi*. Piso ordered to make a woodcut by merging the image of *P. dichrous* with the image of this hummingbird, which is depicted differently in Marcgrave (1648: 196). This bird image is present in the IURNM (Piso 1658: 318). Piso provided a new image of this bird in his treatise, perhaps made after a stuffed animal. Noteworthy is the depiction of the relationship between pollinator and plant, in contrast to showing fauna and flora separately, as was often the case throughout the HNB and the IURNM.

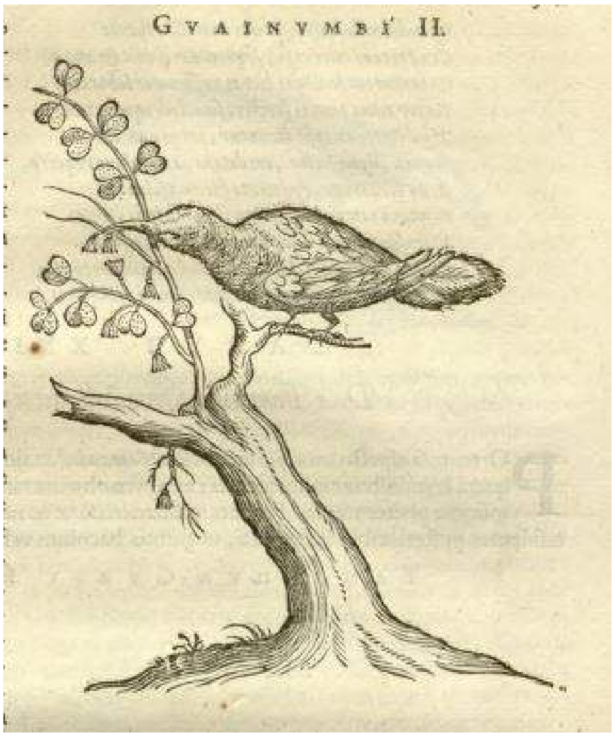

V. *De Noxiis & venenatis, corumque Antidotis*: 319

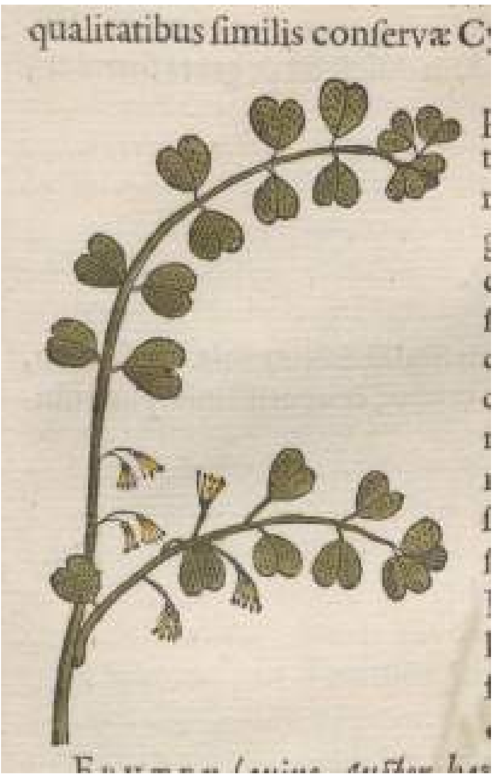

Psittacanthus dichrous in the HNB (Marcgrave 1648: 63)

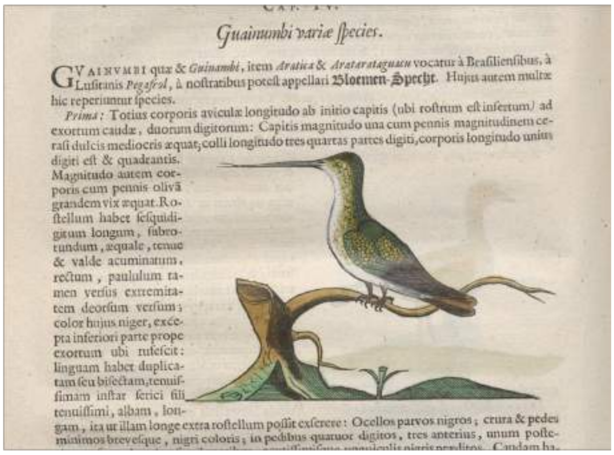

Guainumbi variæ species.

GVAINVMBI quæ & *Guinambi*, item *Aratica* & *Aratarataguacu* vocatur à Brasiliensibus, à Lusitanis *Pegafrol*, à nostratibus potest appellari **Bloemen-Specht**. Hujus autem multæ hic reperiuntur species.

Prima: Totius corporis aviculæ longitudo ab initio capitis (ubi rostrum est insertum) ad exortum caudæ, duorum digitorum: Capitis magnitudo una cum pennis magnitudinem cerasi dulcis mediocris æquat; colli longitudo tres quartas partes digiti, corporis longitudo unius digiti est & quadrantis. Magnitudo autem corporis cum pennis olivã grandem vix æquat. Rostellum habet sesquidigitum longum, subrotundum, æquale, tenue & valde acuminatum, rectum, paululum tamen versus extremitatem deorsum versum; color hujus niger, excepta inferiori parte prope exortum ubi rufescit: linguam habet duplicatam seu bisectam, tenuissimam instar serici fili tenuissimi, albam, longam, ita ut illam longe extra rostellum possit exserere: Ocellos parvos nigros; crura & pedes minimos brevesque, nigri coloris; in pedibus quatuor digitos, tres anterius, unum poste-

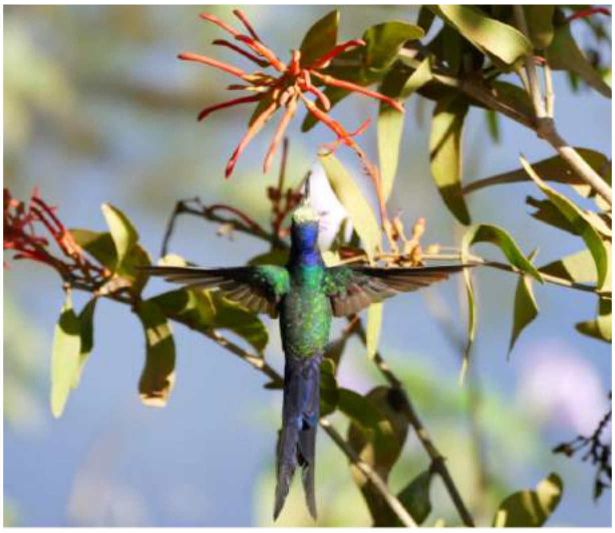

Species of hummingbird in Brazil. *Guainumbi* bird in the HNB (Marcgrave 1648: 196)

A related species to *P. dychrous* (*P. calyculatus*) in MG, Brazil. Swallow-tailed Hummingbird on *Erva de Passerinho* by B. Dupont (CC-BY-SA-2.0)

References

Alcantara-Rodriguez, M., Françozo, M., & Van Andel, T. (2021). Looking into the flora of Dutch Brazil: botanical identifications of seventeenth century plant illustrations in the Libri Picturati. *Scientific Reports*, *11*(1). https://doi.org/10.1038/s41598-021-99226-8

Almeida, A. V. De. (2016). *Historiae Rervm Naturalivm: ensaios histórico-culturais sobre as ciências biológicas*.

Alpini, P. (1592). *Prosperi Alpini De plantis Aegypti liber : In quo non pauci, qui circa herbarum materiam irrepserunt, errores, deprehenduntur, quorum causa hactenus multa medicamenta ad vsum medicin[ae] admodum expetenda, plerisque medicorum, non sine artis iactura, occu*. Apud Franciscum de Franciscis Senensem. https://doi.org/10.5962/bhl.title.162893

Alpini, P. (1640). *De plantis Aegypti liber [...] Editio altera*. Pauli Frambotti Bibliopolae.

Barlaeus, C. (1647). *Rerum per octennium in Brasilia et alibi nuper gestarum sub præfectura illustrissimi Comitis I. Mauritii, Nassoviæ, &c. comitis, nunc Vesaliæ gubernatoris & equitatus fderatorum Belgii ordd. sub Auriaco ductoris, historia.* Ex typographeio Ioannis Blaeu. https://archive.org/details/casparisbarlirer01baer/page/9/mode/thumb

Bauhin, J., Cherler, J. H., Chabrey, D., & Graffenried, F. L. von. (1650). *Historia plantarum universalis, nova, et absolutissima: cum consensu et dissensu circa eas*. [s.n.]. https://doi.org/10.5962/bhl.title.146639

Sloane 1554 Chartaceus, in folio, ff.81. sec XVII.Excerpta, ut testatur in Catalogo MS. veteri Johannes Ward, L.L.D., ex Georgii Marggravii seu Maargravii, Brasiliae Historia Naturali, Johannis de Laet, qui opus istud primum edidit manu

descripta; cum 17th century.

Brunfels, O., & Weiditz, H. (1530). *Herbarum vivae eiconeb [sic] ad naturę imitationem, summa cum diligentia et artificio effigiatę, unà cum effectibus earundem, in gratiam veteris illius, & jamjam renascentis herbariae medicinae ... Quibus adjecta ad calem, appendix isagogica de usu & administratione simplicium ...* Apud Joannem Schottum. https://doi.org/10.5962/bhl.title.8049

Buvelot, Q. . D. T., Martins, E. D. V., Egmond, F., & Mason, P. (2004). *Albert Eckhout: A Dutch artist in Brazil*. Mauritshuis.

Cavalcanti, N. de B., Resende, G. M. de, & Lima Brito, L. T. de. (2010). O crescimiento de plantas de Imbuzeiro (Spondias tuberosa Arruda) no semi-árido de Pernambuco. *Embrapa Semiárido-Artigo Em Periódico Indexado (ALICE)* , *7*(3), 21–31. https://www.alice.cnptia.embrapa.br/bitstream/doc/162568/1/OPB1742.pdf

Chen, J. W.-H. (2020). A Woodblock's Career. Transferring Visual Botanical Knowledge in the Early Modern Low Countries. *Nuncius*, *35*(1), 20–63. https://doi.org/10.1163/18253911-03501002

Clusius, C. (1576). *Atrebat Rariorum alioquot stirpium per Hispanias observatarum historia :libris duobus expressas...* Plantin. https://www.biodiversitylibrary.org/item/30856#page/12/mode/thumb

Clusius, C. (1605). *Exoticorvm libri decem :quibus animalium, plantarum, aromatum, aliorum que peregrinorum fructuum historiae describuntur /item Petri Bellonii observationes ; eodem Carolo Clusio interprete ; series totius operis post praefationem indicabitur.* Plantin. https://doi.org/10.5962/bhl.title.7108

Clusius, C. (1611). *Cvrae posteriores, sev Plurimarum non antè cognitarum, aut*

descriptarum stirpium, peregrinorum'que aliquot animalium novae descriptiones: Quibus & omnia ipsius Opera, aliáque ab eo versa augentur, aut illustrantur. Plantin. https://bibdigital.rjb.csic.es/records/item/13494-redirection

Dalechamps, J. (1586). *Historia generalis plantarum, pars altera, Continens reliquos nouem libros. Eodem in hac parte studio, quo in superiore amplae Plantarum descriptiones digesta*. Lugduni : Apud Gvlielmvm Rovillivm, svb scvto Veneto. https://bibdigital.rjb.csic.es/idurl/1/13561

De Laet, J. (1633). *Americae utriusque Descriptio Novus orbis seu Descriptionis Indiae Occidentalis*. Elzevier. https://archive.org/details/bub_gb_xLF8OiAonjIC/page/n9/mode/1up

De Laet, J. (1640). *L'histoire du nouveau monde ou Description des Indes Occidentales: contenant dix-huict liures*. Elzevier. https://archive.org/details/lhistoiredunouve00laet/page/n3/mode/2up

Dodoens, R. (1583). *Stirpium historiae pemptades sex* . Ex officina Christophori Plantini. https://archive.org/details/mobot31753000817947/mode/thumb

Edgington, J. (2013). *Who found our ferns? A history of the discovery of Britain's ferns, club mosses, quillworts and horsetails*. British Pteridological Society.

Egmond, F. (2009). The exotic world of Carolus Clusius: natural history in the making, 1526-1609. In K. V. Ommen (Ed.), *The Exotic World of Carolus Clusius (1526-1609). Catalogue of an exhibition on the quatercentenary of Clusius' death*. Leidse Universiteitsbibliotheek.

Fuchs, L. (1543). *New Kreüterbuch* . Isingrin. http://www.plantillustrations.org/illustration.php?id_illustration=184723

Gerard, J. (1597). *The Herball, or, Generall historie of plantes /gathered by John Gerarde of London, master in chirurgerie* . Iohn Norton.

https://archive.org/details/mobot31753000817749/page/n18/mode/thumb

Gerard, J., & Johnson, T. (1633). *The Herball, or, Generall Historie of Plantes* . Adam Islip, Joice Norton, and Richard Whitakers. https://archive.org/details/gri_33125012606592

L'Obel, M. de. (1581). *Plantarum seu stirpium icones* . Plantin. http://www.plantillustrations.org/volume.php?id_publication=2710&mobile=0&size=0&id_volume=7624&uhd=0&id_publication=2710

Léry, J. de. (1578). *Histoire d'un voyage fait en la terre de Brésil, autrement dite Amerique*. Antoine Chuppin. https://archive.org/details/histoiredunvoyag01lryj

Lloyd, U. J. (1911). History of the Vegetable Drugs of the United States. *Bulletin of the Lloyd Library of Botany, Pharmacy and Materia Medica*, *18*(4). https://archive.org/details/b24855212/page/74/mode/2up?q=sassafras

Marcgrave, G. (1648). Historia Rerum Naturalium Brasiliae. In *Historia Naturalis Brasiliae* (pp. 50–293). Apud Franciscum Hackium [Leiden], Apud Lud. Elzevirium [Amsterdam]. http://hdl.handle.net/1887.1/item:1535938

Marcgrave, G. (1942). *História natural do Brasil [1648]* (J. P. de Magalhães (ed.)). Imprensa Oficial do Estado.

Matthioli, P. A. (1563). *New Kreüterbuch : mit den allerschönsten und artlichsten Figuren aller Gewechss, dergleichen vormals in keiner Sprach nie an Tag kommen* . Georgen Melantrich von Auentin, Vincenti Valgriss. https://www.biodiversitylibrary.org/item/37059#page/14/mode/1up

Mertens, J., Germer, J., Siqueira Filho, J. A., & Sauerborn, J. (2016). Spondias tuberosa Arruda (Anacardiaceae), a threatened tree of the Brazilian Caatinga? *Brazilian Journal of Biology*, *77*(3), 542–552. https://doi.org/10.1590/1519-6984.18715

Mesuae, I. [Yuhanna ibn M. (1602). *Damasceni medici clarissimi Opera de medicamentorum purgantium delectu, castigatione, & vsu, libri duo* . Iunta. https://archive.org/details/bub_gb_7d7kepnAb-EC/page/n116/mode/1up

Monardes, N. B. (1574). *De simplicibus medicamentis ex occidentali India delatis quorum in medicina usus est*. Plantin.

Parkinson, J. (1640). *Theatrum botanicum = the theater of plants: or, An herball of a large extent...* . Tho. Cotes. https://archive.org/details/gri_33125008297760/page/n1747/mode/2up?q=america

Pereira, N. A., Jaccoud, R. J., & Mors, W. B. (1996). Triaga Brasilica: renewed interest in a seventeenth-century panacea. *Toxicon*, *34*(5), 511–516.

Pickel, B. J. (2008). *Flora do Nordeste do Brasil segundo Piso e Marcgrave no século XVII* (A. V. De Almeida (ed.)). EDUFRPE.

Piso, W. (1658). *De India Utriusque Re Naturali et Medica*. Apud Ludovicum et Danielem Elzevirios [Amsterdam].

Piso, W. (1948). *História natural do Brasil [1648]* (A. Correia (ed.)). Imprensa Oficial do Estado.

Piso, W. (1957). *História Natural, e Médica da Índia Ocidental* (M. L. Leal, F. Carneiro, E. Rodrigues, & J. H. Rodrigues (eds.)). Ministério da Educacão e Cultura, Instituto Nacional do Livro.

Staples, G. W., Simões, A. R., & Austin, D. F. (2020). A Monograph of Operculina (Convolvulaceae) 11. *Annals of the Missouri Botanical Garden*, *105*(1), 64–138.

Thevet, A. (1558). *Les singularitez de la France antarctique, autrement nommée Amerique: & de plusieurs terres & isles decouuertes de nostre temps*. Maurice de la Porte, Clos Bruneau - S. Claude. https://archive.org/details/Les-

singularitez-de-la-France-antarctique-autrement-nommee-Amerique-de-plusieur-PHAIDRA_o_400921/page/n11/mode/thumb

Wartchow, F., Carvalho, A. S., & Sousa, M. C. A. (2010). First record of the psychotropic mushroom Copelandia cyanescens (Agaricales) from Pernambuco State, Northeast Brazil. *Revista Brasileira de Biociências*, *8*(1), 59–60. http://www.ufrgs.br/seerbio/ojs/index.php/rbb/article/view/1297
