# Supplementary material for: Nature portrayed in images in Dutch Brazil: Tracing the sources of the plant woodcuts in the Historia Naturalis Brasiliae (1648)
Source: PLoS One. 2024 Jul 16;19(7):e0276242. doi: 10.1371/journal.pone.0276242 (PMC11251624; doi:10.1371/journal.pone.0276242)
Supplement: Attachment — Submitted filename: Response to Reviewers.docx [file pone.0276242.s006.docx]

**Response to Reviewers [PONE-D-22-26869]**

Dear Dr. Narel Y. Paniagua-Zambrana,

Many thanks for allowing us to revise our manuscript entitled: "Nature portrayed in images in Dutch Brazil: Tracing the sources of the plant woodcuts in the *Historia Naturalis Brasiliae* (1648)", PONE-D-22-26869.

Below, we respond to each point raised by the reviewers. The numbered lines correspond to the manuscript with track changes.

========================================

Reviewers' comments:

1. Is the manuscript technically sound, and do the data support the conclusions?

The manuscript must describe a technically sound piece of scientific research with data that supports the conclusions. Experiments must have been conducted rigorously, with appropriate controls, replication, and sample sizes. The conclusions must be drawn appropriately based on the data presented.

Reviewer #1: Yes

Reviewer #2: Yes

2. Has the statistical analysis been performed appropriately and rigorously?

Reviewer #1: N/A

Reviewer #2: N/A

3. Have the authors made all data underlying the findings in their manuscript fully available?

The PLOS Data policy requires authors to make all data underlying the findings described in their manuscript fully available without restriction, with rare exceptions (please refer to the Data Availability Statement in the manuscript PDF file). The data should be provided as part of the manuscript or its supporting information, or deposited to a public repository. For example, in addition to summary statistics, the data points behind means, medians and variance measures should be available. If there are restrictions on publicly sharing data—e.g. participant privacy or use of data from a third party—those must be specified.

Reviewer #1: Yes

Reviewer #2: Yes

4. Is the manuscript presented in an intelligible fashion and written in standard English?

PLOS ONE does not copyedit accepted manuscripts, so the language in submitted articles must be clear, correct, and unambiguous. Any typographical or grammatical errors should be corrected at revision, so please note any specific errors here.

Reviewer #1: Yes

Reviewer #2: Yes

5. Review Comments to the Author

**Reviewer #1: This is a well-written paper and a carefully conducted study, for which I congratulate the authors. I have only a few minor comments. When the authors state that "...played a significant role in the transmission of (botanical) knowledge" it might be good to clarify that this means "scientific (botanical) knowledge" and not "local knowledge" or LEK.**

Response: We thank the reviewer for these positive comments. We have changed the sentence "transmission of (botanical) knowledge" into "transmitting scientific botanical knowledge" in the abstract and line 34.

**Reviewer #1: In the abstract, please make it clear who did the IDs in "we used the scientific identifications of the portrayed plants..."**

Response: We have changed this sentence in the abstract (line 33) to: "To do so, we used our previous botanical identifications of the portrayed plants, published sources, and digital archival material."

**Reviewer #1: Also, further in the abstract, I do not understand the sentence "availability, economy, and the Indigenous Tupi-based plant names that accompanied the images were crucial when arranging the sources". What are "availability" and "economy"? Please be more specific.**

Response: We change this sentence to specify better what we mean by that. “Substantial differences in the visual-making methodology exist between the two treatises (1648, 1658). In the first book, most of the images were available from Dutch Brazil and carved into the woodcuts, while most of these woodcuts were reused in the second one” (lines 38-40).

**Reviewer #1: Clarify briefly what repository DANS Easy is.**

Response: DANS Easy is a virtual repository. This is used to archive research data to make it accessible worldwide to researchers and interested parties (see more at https://dans.knaw.nl/en/data-services/easy/).

**Reviewer #1: Line 158: specify what "this material" is.**

Response: We have now changed this sentence to "To trace the origins of the woodcut images in the HNB and IURNM, we also…" (line 169).

**Reviewer #1: M&M: why were categories 2 and 3 not assimilated into one "moderate" category? (from "2) moderately similar (they bear a great resemblance but do not share the same features), 3) slightly similar...). How did these 2 categories influence the data? Does there exist any info that could enrich this manuscript on why these specific species were selected to make woodcuts?**

Response: We have elaborated better these distinctions in the text ( Lines 191-198) “We distinguished four categories based on the level of details the artists copied from the *Libri Picturati*'s images into the woodblock's models: 1) very similar (woodcut and illustration share [almost] the same features), 2) moderately similar (they bear a remarkable resemblance but do not share exactly the same features), 3) slightly similar (they are different in most features yet some details, such as the inflorescence, the fruits, a few leaves, etc., look-alike, and 4) different (not enough similar features between images to assume any correlation). The more similar a woodcut and its corresponding species in the *Libri Picturati* were, the more probably the woodcut was made after the image, or they both originated from the same source”.

Referring to *Albizia saman* (an in-text example of a slightly similar woodcut compared to the oil painting in the *Theatrum*), we added in the results (Lines 609-611), "Since their images are only slightly similar, with similar inflorescences but different leaves and spatial composition, this weakens the possibility that they originated from the same source."

Those species were selected to make the woodcuts because their names matched the vernacular names that accompanied the descriptions (as seen in De Laet's manuscript). Often, the names in the manuscript and the illustration in the *Libri Picturati* matched (such as *Bixa orelllana* – line 645). However, De Laet must have had an already made drawing (likely those made by Marcgrave in the field), and he used those to accompany the description in the HNB. We think that in that way, he saved the costs of commissioning a new drawing out of the oil paintings (or the models before them) (see in the manuscript lines 719-732).

**Reviewer #2: I congratulate the authors for the manuscript. This manuscript is one of the products of a very interesting research project that transcending the boundaries of the natural and human sciences. This is yet another set of data, found about Brazil over 400 years ago, from a growing research area: the analysis of historical documentation integrated into botanical collections in Europe. The data on the analysis of woodcuts under study are original! The article is coherently presented and well written. There is much to commend the manuscript! Another relevant and original fact is the use of iconographic sources as the basis of the study. These sources are little investigated from an ethnobiological perspective, as in historical ethnobotanical studies. Iconographic sources can bring relevant information about the geographical areas of study, diversity of knowledge that can help a better understanding of the past, that is, the past relationships between people and plants, making it possible to contribute to the understanding of the current state and possibly the future of these relationships (kept? lost? changed?). In lines 34 and 35, the authors already demonstrate the relevance of the data set studied, with the study of more than 500 images of plant woodcuts related to more than 420 taxa.**

**However, I come to bring some suggestions and reflections below:**

Response: We are grateful to the reviewer for these positive remarks.

**Reviewer #2: Regarding the title 'Dutch Brazil', although a common term in literature, I suggest a reflection! Could you use it as a 'period of Dutch domination in Brazil'?! 'Dutch Brazil' is written both in the title and in lines 54, 56, 59, 69, 97, 134, 205, 579, 720, 750.**

Response: We choose to use the term “Dutch Brazil” because, indeed, as pointed out by reviewer 2, this is the common term used in the literature and therefore it makes our paper more easily accessible/findable for researchers. In the first paragraph of the introduction (line 59), we do explain that this term refers to the Dutch occupation and colonization of part of the Brazilian territory in the seventeenth century.

**Reviewer #2: Still regarding the usual terms, instead of using 'Indigenous Brazilians' both in the keyword and in the textual part, what do you think about using 'Brazil's indigenous people' or 'indigenous peoples of Brazil'?**

Response: Regarding the terms *Brazil's indigenous people* or *indigenous peoples of Brazil*, we prefer not to use either one of these expressions because both imply possession or belonging (*'s* and *of*); that is, they suggest that the indigenous peoples belong to or in Brazil. However, from an archaeological, historical, and anthropological perspective, this is incorrect. Indigenous peoples existed long before the colonization and long before the creation of the nation-state Brazil. For many of them still today, the national borders are less significant than for non-Indigenous peoples, as Indigenous communities transcend borders and have relationships and ways of life that go beyond them. The term *Indigenous Brazilians*, on the other hand, acknowledges that they were born in or live in Brazil but that they are nonetheless Indigenous and not 'property' of the country.

**Reviewer #2: It would be possible to revise the questions or even better to turn them into clearly hypotheses, centered beyond the issue of efforts and the methodological perspective of how to handle and identify the materials (woodcuts and vouchers). Sometimes throughout the manuscript, there is a protocol development bias on how to carry out studies with iconographic sources (woodcuts). I believe there is a wealth of data that could be further explored!**

Response: We have explained in detail how we identified the woodcuts, herbarium vouchers, and botanical illustrations in the *Libri Picturati* in our previous paper (Alcantara-Rodriguez et al. 2021. Looking into the flora of Dutch Brazil: Botanical identifications of seventeenth century plant illustrations in the *Libri Picturati*. Scientific Reports, 11(1), 19736.). Since the current paper is already quite long, we decided to cite our previous work on how we did the identifications instead of adding these methodological details again in the current manuscript.

**Reviewer #2: I think I could review and elaborate on these issues further, especially on lines 129 to 138.**

**The authors could, for example, deepen the central hypothesis about the production of works of illustration for the natural sciences during the period of Dutch domination in Brazil (objects of study, techniques, and materials) considering the importance of Dutch artists and their production techniques of images and objects (over 400 years ago), relating to the European (Dutch) perspectives of the time.**

Response: Our main goal in this manuscript was to trace the (diversity of) visual sources of the woodcuts in the published treatises HNB and IURNM. The importance of Dutch artists on the European worldview in the seventeenth century is substantial but lies beyond the scope of our research. We cite other authors (Whitehead & Boeseman, 1989; Joppien, 1979; Brienen, 2006) who cover this subject in more detail.

**Reviewer #2: Could the authors deepen even more about the history of art, the history of the Brazilian northeast, science, ways of life and the perceptions of Europeans. Seeking to advance and reflect even more on the close relationship between Dutch naturalists, sixteenth-century research and possible "native" Brazilian authors/collaborators, the peculiarities in terms of climate, vegetation, availability of plant resources (shortage of food for long periods, as this region is quite arid and dry).**

Response: Although all very interesting subjects, they fall beyond the scope of the current paper. We have published on African, native Brazilian and European food crops in seventeenth century in our previous paper Alcantara-Rodriguez et al. 2019. Plant knowledge in the *Historia Naturalis Brasiliae* (1648): Retentions of seventeenth-century plant use in Brazil. Economic Botany, 73, 390-404.

**Reviewer #2: Throughout the textual part, it would be interesting to clarify (if possible) a reflection on the possible perspectives of using these findings (of the botanical illustrations) so that they can bring information, as a means of understanding and presenting the indigenous and African heritage in new ways. There is little mention of 'Enslaved Africans', 'Indigenous Brazilians', as seen in lines 42, 43, 97, 287, 297, 587, 607... They could value this information from the beginning of the manuscript!**

Response: To acknowledge the presence of the diverse local groups during the activities of Maurits’ in the colony and their asymmetrical power relationships in the seized land, we added at the beginning of the introduction (lines 64-69), “With the support of the WIC, the count commanded a military guard to accompany Marcgrave when collecting fauna and flora (1). At the same time, local people (often Indigenous Brazilians, but also enslaved Africans and Portuguese) brought the specimens to him while he resided in Maurits’ court. The same applied to Piso, who, like the other members of Johan Maurits’ entourage, was entitled to an assistant for himself.”

The reviewer raised a valid point about decolonizing historical texts and narratives. While we acknowledged the unequal power dynamics between European colonizers and Indigenous and enslaved Africans coexisting in the colony, this information is not discussed in-depth in our manuscript. On the one hand, our aim was to trace the origins of the woodcuts. Information on the use of plants by Indigenous Brazilians and enslaved Africans in seventeenth century Brazil was published in our previous paper: Alcantara-Rodriguez et al. 2019. Retentions of seventeenth-century plant use in Brazil. *Econ. Bot.*

On the other hand, the making of these texts and paintings is attributed to Western scholars and artists (white, European, middle-upper class men), who created them from their Eurocentric perspective; hence, their narratives prevail in the archival visual and textual material. Now, the knowledge embedded in these sources did come from a more heterogenous context, as indicated in our previous paper. The various Indigenous peoples and enslaved Africans played a role in the knowledge-making processes, and using these illustrations could bring up many plant stories that acknowledge their heritage. Future collaborations with Indigenous and Afro-Brazilians will shed more light on the representation of Indigenous and African heritage at present by looking at historical sources together.

We want to encourage further critical studies that go more in-depth with the issues brought up by the reviewer, as we indicated in lines 682-686: “Our database with all species and vernacular names listed in the HNB and the IURNM, Marcgrave’s herbarium, and the *Libri Picturati* (S1, S2, S3, S4), provides a good ground to analyze Western and non-Western plant nomenclature systems and their preservation over time, as well as to review the role of Indigenous and African knowledge embedded in these natural history collections”.

**Reviewer #2: The manuscript envisages a multidisciplinary process, combining biology, art and anthropology (?) It emphasizes the methodological issue, that is, how the authors treated and analyzed the datasets of woodcuts, documents and plant vouchers. However, it would be important to contextualize and deepen the linguistic issue of this region under study, of human groups from the past (16th century) and the current ones. The authors could value even more the vernacular names, the indigenous names of the plants and possible associated data.**

Response: We agree with the reviewer that there is much valuable linguistic data (in the form of local plant names) included in these published and unpublished botanical illustrations from seventeenth century Dutch Brazil. It requires extensive study by linguists to trace these Tupi-related plant names to currently used indigenous plant names in Brazil. In a previous paper, we covered a small part of this interesting field: Geertsma, I. P., Françozo, M., van Andel, T., & Rodríguez, M. A. (2021). What's in a name? Revisiting medicinal and religious plants at an Amazonian market. Journal of Ethnobiology and Ethnomedicine, 17(1), 1-15.

Recently, linguistic elements of the HNB were discussed with indigenous people who speak Tupi-related languages in Brazil, and the results were published some weeks ago by Aline da Cruz and Walkíria Neiva Praça (2023) Reconnecting Knowledges: Historia Naturalis Brasiliae back to Indigenous Societies. [https://www.taylorfrancis.com/chapters/oa-edit/10.4324/9781003362920-8/reconnecting-knowledges-aline-da-cruz-walk%C3%ADria-neiva-praça?context=ubx&refId=57d71bc0-c940-4406-8684-d102d6df66f2](https://www.taylorfrancis.com/chapters/oa-edit/10.4324/9781003362920-8/reconnecting-knowledges-aline-da-cruz-walk%C3%ADria-neiva-pra%C3%A7a?context=ubx&refId=57d71bc0-c940-4406-8684-d102d6df66f2)

**Reviewer #2: Another interesting aspect would be to highlight the importance of these findings for Brazil, mainly for contributing to the history of biodiversity in the Brazilian northeast. I also suggest a better characterization of the Brazilian northeast region, this part is quite superficial, mentioned only in line 56. It would be essential to contextualize the region under study, highlighting its climatic peculiarities, vegetation, human groups, ways of life (a summary of the human population, indigenous languages and origin) both from the period of Dutch rule in Brazil and today.**

**It would be worth characterizing and highlighting the places where the Dutch concentrated their efforts and studies, where they collected and described plant resources (more in Bahia? Pernambuco? coastal vegetation? caatinga? Atlantic forest?).**

**The manuscript lightly describes the "Northeast" of Brazil, it is interesting to highlight the diversity, abundance and richness of the local flora and culture. The northeast is rich and diverse, as it encompasses different biomes and plant formations (caatinga, Atlantic forest and coastal vegetation), and of course, this has implications for the availability of species by environment. I suggest better characterizing the region of origin of the woodcuts.**

Response: In the present paper, we shortly mention "the *sertão*, the dry hinterland of northeastern Brazil" in line 350. The natural vegetation, climate, and native inhabitants of the area colonized by the Dutch do not fall within the scope of our current paper. However, we have incorporated these aspects extensively in our previous paper (Alcantara-Rodriguez et al. 2021. Looking into the flora of Dutch Brazil), in which we describe the vegetation types where the plants grew depicted in both the *Libri Picturati* and the woodcuts and whether they were endemic to Brazil or specific regions of the country.

**Reviewer #2: Will this information be relevant to advancing hypotheses related to the versatility of uses, for example. Introduced and/or native species in Brazil would be important initially for their food potential and secondly for their medicinal potential)? The Northeastern region of Brazil is characterized by long periods of food scarcity (an extremely dry and arid region), could it be that this characteristic provided some bias of interest in certain plants from the 16th century?**

Response: In our previous paper (Alcantara-Rodriguez et al. 2021. Looking into the flora of Dutch Brazil), we specifically mention plants typical to northeast Brazil that can withstand drought and are used for emergency food by the people living there (like *Spondias tuberosa*). Comparisons with seventeenth century and current plant use, based on these plant illustrations have been published in our paper in Economic Botany (Alcantara-Rodriguez et al. 2019).

**Reviewer #2: This data crossing information (past and current) is particularly important to assist in future studies of mapping and availability of plant resources, ways of life. I believe that few Brazilians know and/or have access to the history of plant and cultural diversity in Brazil (in the Northeast region) in the 16th century. This information needs wide visibility and appreciation!**

Response: We fully agree with the reviewer and have published this already (see our previous remark).

**Reviewer #2: The authors could expand the scope and bring some future of returning the scientific knowledge analyzed here to the indigenous and/or traditional communities of the Brazilian northeast, especially to young people? Or bring a perspective on how these data can be used to expand knowledge of plant and cultural diversity in Brazil. Or how to promote greater visibility and exchange of information from this data set, how to return this knowledge about these collections (forgotten in European libraries) to Brazilian institutions, indigenous peoples and/or places in the Brazilian northeast from where they were originally taken? Is there a future perspective that museums/institutions can develop new ways of presenting historical data about plants and cultural diversity?**

*** I know it's out of this scope, but it's worth thinking about!**

Response: We have also thought about this a lot. Due to covid-19, we could not travel to Brazil during the period in which we did our research (2020-2021). However, our supporting information contains all the visual information on which we based our research, so that will become available to the world with the publication of this paper. Many of these images have never been published before with their correct botanical names.

**Reviewer #2: In the methods, it is important to explain more about the historical sources, especially in relation to the transcripts and translations used! Does it highlight whether original (primary), written or iconographic sources were used to analyze the datasets? It would be worth reviewing the methodology described for crossing information, especially between lines 158 to 168.]**

Response: In our methods, we mention that we worked with digital images of the visual sources. We visited the original Marcgrave’s herbarium on an earlier occasion (2018) and consulted original volumes of the HNB and IURNM in the Naturalis Library. For the current paper, however, we worked with digital images.

**Reviewer #2: Would it be possible to list the ethnicities in the database (described in lines 169 to 171) or better highlight in which area of the northeast and possible ethnicities would be related to certain information (vernacular names)?**

Response: We do not know precisely where Marcgrave (and Piso) collected their specimens, as they do not mention this in the written sources for the majority of the species. Hence, we cannot plot these on a map. The few locations indicated in the HNB for specific plants were already highlighted by Almeida (2016). De. Historiae Rervm Naturalivm: ensaios histórico-culturais sobre as ciências biológicas. Recife.

With regard to the Indigenous Brazilians, much has changed since the Dutch left Brazil. People speaking Tupi-related languages around 1640 in Pernambuco have migrated elsewhere, became victims of colonial violence, infectious diseases, or merged with other ethnicities. Therefore, it is not possible to locate peoples in Brazil today who are "the descendants" of those from whom Marcgrave documented the names and plan uses in the 1640s. We have elaborated on this issue in our paper Alcantara Rodriguez, M., Pombo Geerstma, I., De Campos Françozo, M., & van Andel, T. R. (2020). Marcgrave and Piso's plants for sale: The presence of plant species and names from the Historia Naturalis Brasiliae (1648) in contemporary Brazilian markets. Journal of Ethnopharmacology, 259, 112911.

**Reviewer #2: Still in methods, in lines 173 to 175, I could better distinguish and explain the difference between 2) moderately similar and 3) slightly similar. It got a little confusing!**

**As for results, it would be interesting to explore the data further, as initially described here in the review!**

Response: We understand this distinction seems a bit confusing. By looking at the illustrations compared to the woodcuts in the digital files we created (S2 and S4), the reader can better visualize the differences between categories. To make it more evident in the text, we added (lines 191-198), " We distinguished four categories: 1) very similar (woodcut and illustration share [almost] the same features), 2) moderately similar (they bear a remarkable resemblance but do not share exactly the same features), 3) slightly similar (they are different in most features yet some details, such as the inflorescence, the fruits, a few leaves, etc., look-alike), and 4) different (not enough similar features between images to assume any correlation). The more similar a woodcut and its corresponding species in the *Libri Picturati* were, the more probably the woodcut was made after the image, or they both originated from the same source".

In addition, referring to *Albizia saman* (an in-text example of a slightly similar woodcut compared to the oil painting in the *Theatrum*), we added in the results (lines 609-611), "Since their images are only slightly similar, with similar inflorescences but different leaves and spatial composition, this weakens the possibility that they originated from the same source."

The slightly similar images between the HNB and the *Libri Picturati* only accounted for 9%. After all, there are more differences between corresponding images between the woodcuts and the oil paintings (for the same species) than similarities. Hence, these illustrations later included in the *Libri Picturati* (or the sketches or drawings made before them) were not the primary sources used to elaborate the woodcuts in the HNB and IURNM (as suggested before). Instead, several drawings from the field in Brazil (likely made by the naturalist Marcgrave) were used as models by De Laet and ten years later reused by Piso. The nuances in the similarity between images do not change our conclusions. However, we thought it was important to make those distinctions based on how much in detail the artists went into when copying the figures that would end up being carved onto the woodblocks.

**Reviewer #2: For example, in line 206, the finding on the woodcut of *Spighelia anthelmia* L. was described, and then the data were little discussed in line 545. This species is widely known and used in traditional medicine (the 'worm herb'), treats it if it is a spontaneous species, annual and occurring in the tropical region, it is a potent vermifuge, but there is toxicity ... finally, explore even more the information on the species.**

Response: Although plant uses of these plants were extensively described in our other papers, we have added a short sentence (line 227), "The common medicinal plant against intestinal worms, *Spigelia anthelmia* L." Just like elsewhere in the world where this weed grows, it is used against worms, and pretty poisonous as well.

**Reviewer #2: Another very important species on the Brazilian coast and known to be medicinal is *Schinus terebinthifolia* Raddi (but there are doubts about the species of Schinus in the northeast, there are possibly two species of masYc – Schinus, *S. terebinthifolia* and *S. molle*). It would be worth a deepening on the highlighted species.**

Response: Indeed, *Schinus terebinthifolia*, known as Brazilian pepper tree or *Aroeira vermelha*, grows along the Brazilian coast and is the tree with the round leaflets depicted in the oil painting (Figure 13d). *S. molle* also occurs in Brazil, but more in the south (Rio Grande do Sul) and has very thin leaves (as the woodcut in the HNB has, Figure 13 a). We used the example of *S. terebinthifolia* to discuss the role of the vernacular names in the making of the HNB. The editor of the HNB, De Laet, mistook it for another species (*S. molle*), a known medicine elsewhere in South America and depicted in other books on herbal medicine. Surprisingly, the right image of the tree (*S. terebinthifolia*) was included in the *Theatrum* (available to him). However, he did not associate it with the description because their names did not match. He had to reuse a previously published image, ultimately using the wrong one, which Piso also mistook ten years later.

As pointed out by the reviewer, *S. terebinthifolia* is indeed an important medicinal plant. This fact was highlighted in historical sources, such as the HNB, and its medicinal value has persisted today. In the supplementary electronic material - ESM2 accompanying our previous paper Alcantara-Rodriguez et al. 2019, we indicated the uses of *S. terebinthifolia* as described in the HNB and the potential correlations with its uses in modern Brazil. The HNB points out that the resin of this tree and the oil squeezed from the fruits act as a poultice to treat cold affections, and the aromatic leaves were used for baths. In modern literature, we found that the *Aroeira* oil extracted from the bark is used for tumors arising from arthritis or syphilis; the leaves are anti-rheumatic and also used to heal ulcers and wounds.
