# Supplementary material for: Nature portrayed in images in Dutch Brazil: Tracing the sources of the plant woodcuts in the Historia Naturalis Brasiliae (1648)
Source: PLoS One. 2024 Jul 16;19(7):e0276242. doi: 10.1371/journal.pone.0276242 (PMC11251624; doi:10.1371/journal.pone.0276242)
Supplement: Attachment — Submitted filename: PLOSONE-round 2-Response to Reviewers.docx [file pone.0276242.s008.docx]

**Response to Reviewers [PONE-D-22-26869]**

Dear Dr. Godwin Upoki Anywar,

Many thanks for allowing us to revise our manuscript entitled: "Nature portrayed in images in Dutch Brazil: Tracing the sources of the plant woodcuts in the *Historia Naturalis Brasiliae* (1648)".

We have carefully reviewed the manuscript, corrected some minor grammar and spelling mistakes, and added more related literature as suggested by the reviewers. In addition, we provided the link to the scanned images of the *Theatrum Rerum Naturalium*, which are now digitized, to the methods section of the paper.

We would like to take this opportunity to clarify a question regarding the version of our paper being reviewed. Our original submission, submitted in November 2022, was reviewed by 2 referees who made important suggestions to the paper. We revised it accordingly and resubmitted a revised version to Plos One in April 2023, alongside our answers to the reviewers. The paper was then reviewed by another 2 referees, whose suggestions we received as of November 2023. However, when carrying out the required new revisions, we could not help but notice that the paper sent back to us with comments by reviewer #4 was still the first version of the paper, that of November 2022, and not the revised version of April 2023. The feedback given by reviewer #3 likewise suggests he might have read only the November 2022 version of the paper, as some of his comments refer to issues we had already solved in the April 2023 revised version of the paper.

In any case, we have proceeded to revise the manuscript according to these new referees’ suggestions. In order to keep the process consistent, we have applied the new revisions to the April 2023 version of the paper, so that the suggestions of all four referees are taken into account.

For the sake of clarity, we hereby also send you the responses to reviewers #1 and #2.

Below, we respond to each point raised by the reviewers. The numbered lines correspond to the manuscript with track changes.

Review Comments to the Author

**Reviewer #3**: The A. analysed a relevant number of woodcut images from the books of Historia Naturalis Brasiliae (1648). Though a comparison with visual and written sources they could identify a high number of taxa, and they used different tools for comparing data and check the taxonomical identification. In general, the topic of the study is original and very interesting, being an example of cultural and natural heritage that needs to be valorized.
Despite such great element of interest, I believe that the paper should be better organized for a contribution in an international scientific journal, since it deeply illustrates some findings of their work, but the topic is not introduced, clarified in the aims, and later discussed for a wider audience.

**Response**: we appreciate the nice comments about our study. Indeed, it is crucial to value these natural history collections as rich biocultural heritage. Equally relevant is to generate new data out of them, that help us to understand the knowledge-making processes they entailed – in this case, the visual methods employed for the woodcuts. Our main aim was to trace back the sources used to elaborate the woodcuts in the HNB and IURNM, two different yet highly interconnected treatises. Other associated material also connected were the *Libri Picturati*, Marcgrave’s herbarium, and other scholars’ images. While many studies, especially those with an art-history approach, focused on the similarities you can see among images at first sight, due to common image elements, we used instead the botanical identifications of the plants portrayed. We have introduced our topic more explicitly in the introduction and we clarified the aims before discussing it later.

Example in Lines 73 to 76: “The botanical imagery of these sources is the object of this study. By linking the materials and collections from Dutch Brazil, we aim to trace how these natural history books, like the HNB, were produced in the early modern period.”

**Reviewer #3**: The introduction lacks referencing the recent international literature, where usually the aims of one paper originate (The selected references deal mostly with old historical records 4,20,21,11,13–19).

**Response**: Indeed, our aim was highly inspired by modern discussions on the provenance of the woodcuts and by the lack of a systematic analysis of the woodcuts versus the *Libri Picturati* collection and other associated materials. The interconnections of these materials were clear to us when we identified the flora portrayed in the *Theatrum*, the *Misc. Cleyeri* and the *Libri Principis*. As pointed out by Whitehead and Boeseman (1989), Brienen (2006), and Scharf (2019), some of the illustrations in the *Libri Picturati* were similar in structure and composition to the woodcuts in the HNB. But, to what extent? Here is where our original botanical analysis played an important role.

The historical records we used refer the scholars who analyzed the woodcuts or the visual material associated with the HNB when the collection was still in Berlin (e.g., Martius 1843, Lichtenstein, 1817, Schneider 1938). After Whitehead found it in the late 1970s in Krakow, these collections were analyzed by several scholars, mostly from an (art) historical point of view (e.g., Albertin, 1985; Whitehead and Boeseman, 1989; Boeseman, 1990; Teixeira, 1995; Brienen, 2006 Scharf, 2019, etc.). However, the reference numbers cited by the reviewer were disorganized because they were part of the first version of this manuscript (submitted in November 2022), which we revised and sent back in April 2023. The references in our revised manuscript (April 2023) correspond to modern and historical works on the matter under study. To further clarify the aim, we have added a more explicit explanation in the introduction and linked it in more detail to the literature. We now highlighted in more detail the works of these scholars from whom our work builds up and sometimes differs (as elaborated in our discussion). See the section “1.3 Behind the images: previous and present research” from lines 119 to 173.

**Reviewer #3**: Furthermore, the results of the previous works of the A. are not well introduced (Alcantara-Rodriguez M, Françozo M, Van Andel T. Looking into the flora of Dutch Brazil: botanical identifications of seventeenth century plant illustrations in the Libri Picturati. Sci Rep. 2021 Dec 1;11(1) and it is cited only in the methods!). Then, I understand that the previous one is a floristic paper, but it is a bit difficult deeply understand the novelty and originality of the paper.
In fact, the aims (analyze the correlations between the woodcuts and the other visual sources from Dutch Brazil, and trace back the remaining sources that were used to create the woodcuts) **are not supported** by critical analysis of the literature. Indeed, other scientific papers on such field of scientific Museology should be analyzed, considering other relevant cases studies. For example, when citing Hernandez, the A. should also cite the comments of G.B. Bettolo (Accademia Nazionale dei Lincei (Rome, Italy), to comment the Novae Hispaniae Thesaurus), and s relevant literature on such can be also found by the rich Portuguese scholars... (I add a few Medeiros, Maria Franco Trindade, and Ulysses Paulino Albuquerque. "Food flora in 17th century northeast region of Brazil in Historia Naturalis Brasiliae." Journal of ethnobiology and ethnomedicine 10 (2014): 1-20.
Whitehead, P.J.P., 1976. The original drawings for the Historia naturalis Brasiliae of Piso and Marcgrave (1648). Journal of the Society for the Bibliography of Natural History, 7(4), pp.409-422.
da Cruz, A. and Praça, W.N., 2023. Reconnecting Knowledges: Historia Naturalis Brasiliae back to Indigenous Societies. In Toward an Intercultural Natural History of Brazil (pp. 142-165). Routledge.
Alsemgeest, A. and Bos, J., 2023. Census of the Copies of Willem Piso and Georg Marcgraf's Historia Naturalis Brasiliae (Leiden and Amsterdam: Elzevier, 1648). In Toward an Intercultural Natural History of Brazil: The Historia Naturalis Brasiliae Reconsidered (pp. 166-211). Routledge

Ossenbach, C., 2017. Precursors of the Botanical Exploration of South America. Wilhelm Piso (1611-1678) and Georg Marcgrave (1610-1644). Lankesteriana, 17(1), pp.61-71.
Otherwise, if the topic of the paper is the image analysis the title should be changed, and different references should also be searched.
**Response**: We have clarified the paper's topic and added the recommended literature when appropriate. The works by Medeiros and Albuquerque (2014) and Ossenbach (2017) are not included in our revised version of this manuscript. The former compares 17th-century food plants as documented in the HNB with present food uses of these plants in Brazil, which has limited links to our paper. The latter focuses on two species of orchids described in the HNB and collected in Marcgrave’s herbarium. We have carefully examined and cited both works in our diachronic study on medicinal and other useful plants in the HNB and modern Brazil (Alcantara-Rodriguez et al., 2019). However, we decided to leave them out of this paper as they are unrelated to the provenance of the woodcuts in the HNB.

The work by Hernández is cited as follows: “To navigate this vast corpus of literature, we first checked the HNB and IURNM for references to scholars who worked with tropical flora, such as Hernández (33), Monardes (34), or Clusius (35–37).” (lines 225-226). When the woodcuts did not match with the contemporary sources, we had to check in previous works because it was common at the time to “borrow” images from other treatises to complement the visual repertoire of texts on tropical flora. Furthermore, De Laet often cited those scholars, giving us clues as to whose works to study. Hence, instead of Bettolo (1990), we used the work of Almeida (2016) who systematized the entries of the scholars with the related plants, facilitating our search. As for Hernández, it is true that his oeuvre, and the corpus of work that originated from his expedition in New Spain, has profoundly impacted the cultural history of European-Native American knowledge exchanges and the history and historiography of early modern science – so much so that a proper comparison of the HNB and its trajectory to that of the Hernández corpus merits an entire new research project, or at least a dedicated paper to delve in detail into how these historical events and their cultural legacies converge and diverge. For this reason, we chose to remain close to the aim of our paper, that is, checking the possible sources for the woodcuts in the HNB. When analysing Hernández (1628), we did not find any correspondence with the woodcuts, otherwise, this fact would be reflected in our results and we would have discussed it and most likely facilitated our analysis with the work of Bettolo (1990, 1992).

We cite the work of Whitehead (1976) on the original drawings of the HNB (in which he discusses the *Libri Picturati’*s images as the possible source for the woodcuts and suspected it was somewhere in Poland) throughout the paper: see lines 114, 123, and 130.

In Methods (lines 181-184), we added Alsemgeest and Bos (2023) when we cited the digital copy of the HNB we used for this study (as there are over 300 copies around the world): “The surviving copies of the HNB amount to 302, distributed in collections and institutions worldwide (30). We used the digital-colored copy of the HNB located in Leiden University Library in the Netherlands [Shelfmark 1407 B3 (30)] ([HNB Leiden Universiteitsbibliotheek](https://digitalcollections.universiteitleiden.nl/view/item/1535938#page/25/mode/1up)) (Fig 1).

In addition, we incorporated the fantastic paper of Da Cruz and Neiva Praça (2023) in our discussion on the Indigenous Tupi-based plant names of the HNB and the *Libri Picturati*: “It is essential to notice that the Western scholars behind the HNB production oversimplified Indigenous and African languages under a few categories (such as “Tupi” or “Tupinambá,” a language belonging to the administrative and ecclesiastic *lingua franca*). Nevertheless, Indigenous groups spoke more than a thousand languages before the arrival of the Portuguese and their genocide, exposure to foreign diseases, and displacement (<https://pib.socioambiental.org/en/Languages>). Collaborative linguistic projects with modern Indigenous communities in Brazil, such as those by Da Cruz and Neiva Praça (70), highlighted the connections (i.e., cognates) in plant and animal names between the HNB and three Indigenous communities in Brazil. These studies have proven crucial to increasing access to these historical collections (often only available to the scholarly public) and recognizing the agency of those whose heritage may be connected to them”.

(lines 672 to 683).

Moreover, we highlight a few more of these collaborative-based projects in the last part of the discussion (see Further research and recommendations).

Our paper on the botanical identifications of the *Libri Picturari* is cited in methods because we used the species identified in our previous work to cross-reference them with the plant species depicted in the woodcuts. In that way, we can see whether they are similar to each other (or not) and draw conclusions about their origins: “To compare woodcuts, vouchers, and plant illustrations, we cross-referenced all textual and visual sources for each plant species using our recent botanical identifications for the HNB, the IURNM, and the *Libri Picturati* (lines 201-203)”.

This manuscript does not compare to our previous one because the focus is not on identifying the plants depicted but on analyzing which sources were used to create the woodcuts in the natural history treatises under study (as the title states). However, we appreciate this remark, and we have now cited it in the introduction, in section “1.3. Behind the images: previous and present research previous research”.

In lines 140 to 144 we added: “In our previous research, we identified the plant species depicted in the whole Brazilian collection within the *Libri Picturati* and found that a substantial number of species were documented and often illustrated in the HNB and IURNM (21). However, to what extent the images representing these species were comparable between the *Libri Picturati* and the HNB was still a mystery, as well as the potential authors behind their making”.

**Reviewer #3**: In the Methodological section the 4 classes are really ambiguous (the concept of similarity should be better explained...It means similar in the diagnostic elements??? .. and: which were the diagnostic elements? ... which were the limits of the classes of similarity??? In fact, the similarity can also arise from the habitus of a species, and different species can be sometime similar in the general characters, whereas the same species can appear different, if it is analysed in a different phenological status .... Then it must be clarified and specify the limit of each class! (very similar (woodcut and illustration share [almost] the same features), 2) moderately similar (they bear a great resemblance but do not share the same features), 3) slightly similar (woodcuts share some characteristics but not as many as in the previous category), and 4) different (not enough similar features between images to assume any correlation).

**Response**: We have now better elaborated these distinctions and the limits of each class in the text (Lines 211-221) “For the latter, we identified different degrees of similarities based on their botanical elements (or the lack thereof), spatial composition (indicating when the images were similar but reversed), shape, and form. We distinguished four categories: 1) very similar (woodcut and illustration share [almost] the same elements, shape, and form), 2) moderately similar (they bear a remarkable resemblance, especially in their morphology, but do not share precisely the same elements, or these are placed differently), 3) slightly similar (they are different in most elements yet some, such as the inflorescence, the fruits, a few leaves, etc., look-alike), and 4) different (the images do not present any similarity in their composition and botanical elements) ). The more similar a woodcut and its corresponding species in the *Libri Picturati* were, the more probably the woodcut was made after the image, or they both originated from the same source.”.

For example, referring to *Samanea saman* (an in-text example of a slightly similar woodcut compared to the oil painting in the *Theatrum*), we added in the results (Lines 598-600), "Since their images are only slightly similar, with similar inflorescences but different leaves and spatial composition, this weakens the possibility that they originated from the same source."

The slightly similar images between the HNB and the *Libri Picturati* only accounted for 9%. After all, there are more differences between corresponding images between the woodcuts and the oil paintings (for the same species) than similarities. Hence, these illustrations later included in the *Libri Picturati* (or the sketches or drawings made before them) were not the primary sources used to elaborate the woodcuts in the HNB and IURNM (as suggested before).

Finally, the reader can better visualize the differences between categories by looking at the illustrations compared to the woodcuts in the digital files we created (Appendices S2 and S4).

**Reviewer #3**: Furthermore, when using the word “correlation” (see the aims) I would expect some statistical analysis which can prove the level of correlation. Such part of analysis is completely missing... perhaps is it better avoiding such world.... The world “comparison” is much more appropriate.

**Response**: Although we have tabulated the data to calculate the percentages of each woodcut source (HNB/IURNM, *Libri Picturati*, Marcgrave’s herbarium, Eckhout/Post, and other sources – see Figs. 3 and 8 in the main manuscript), we have not used statistical tests because these require unambiguous quantitative differences, which do not apply to the comparison of drawings and therefore to our research. We used the term correlation by following the definition: “a mutual relationship or connection between two or more things” (Exford languages by Google) instead of its definition in statistics (“[interdependence](https://www.google.com/search?sca_esv=580846077&rlz=1C1EKKP_enNL778NL778&sxsrf=AM9HkKmnzevmR0UkWtiGbwOCLZxCEyqbDQ:1699533243078&q=interdependence&si=ALGXSlYwDW0HUer9cZIextq8wqSf5FbBJRthUwkcB7R9-uaJFJjHfBe5SzWZZxzQGKJp3y5ZH_ibQylkljwQmO9_bz2g5x5U0KfoKxN_eXTnFsOBAJfQdvg%3D&expnd=1) of variable quantities”, Oxford Languages). We do not assume a linear dependence of the variables but a connection between images that most likely share a common origin. Hence, to avoid ambiguity, we have followed the reviewer’s advice and changed the word *correlation* in the text for *correspondence*, *matching*, *(inter)connections*, *(dis-) similarities*, etc.

**Reviewer #3**: I finally suggest explaining better the focus of the paper and rephrasing the title and some organization, adding an insertional literature on sources and methods more suitable. (I don’t enter too much in results and discussions, because the previous points are mandatory.

**Response**: The title of the paper relates to the aim of our research, the botanical-visual analysis we have conducted, and the context (“Dutch Brazil”). Therefore, we prefer to keep this title, also because it exactly reflects what we did in research: Tracing the sources of the woodcuts of the HNB (and the IURNM). We have done that by using our scientific identifications, comparing the images of the same plant species in the material remaining from Dutch Brazil, looking at other scholars/artists’ work, and quantifying our results to obtain a clear picture of the visual-making methods.

With regard to the organization of the paper, we followed the reviewer's recommendations carefully and more explicitly mentioned the focus of the paper, edited the methods accordingly, and added the recommended literature when this was relevant to the scope of our manuscript. We hope we improved and enriched our paper by following these suggestions.

**Reviewer #4:** The manuscript Nature portrayed in images in Dutch Brazil: Tracing the sources of the plant woodcuts in the Historia Naturalis Brasiliae is an original contribution to the field. I only left minor corrections to the attached file.

**Response**: We thank the reviewer for their feedback and for pointing out to us the minor mistakes in the text, which we have corrected.

.
