# Supplementary material for: Nature portrayed in images in Dutch Brazil: Tracing the sources of the plant woodcuts in the Historia Naturalis Brasiliae (1648)
Source: PLoS One. 2024 Jul 16;19(7):e0276242. doi: 10.1371/journal.pone.0276242 (PMC11251624; doi:10.1371/journal.pone.0276242)
Supplement: Attachment — Submitted filename: Response to Reviewers.docx [file pone.0276242.s009.docx]

**Response to Reviewers [PONE-D-22-26869R2]**

Dear Dr. Godwin Upoki Anywar,

Many thanks for allowing us to revise our manuscript entitled: "Nature portrayed in images in Dutch Brazil: Tracing the sources of the plant woodcuts in the *Historia Naturalis Brasiliae* (1648)". We are also very grateful to the reviewers for providing their time and expertise in evaluating our paper and enriching the final version.

Below, we respond to each point raised by the reviewers. The numbered lines correspond to the manuscript with track changes.

**Journal Requirements**

Please review your reference list to ensure that it is complete and correct. If you have cited papers that have been retracted, please include the rationale for doing so in the manuscript text, or remove these references and replace them with relevant current references. Any changes to the reference list should be mentioned in the rebuttal letter that accompanies your revised manuscript. If you need to cite a retracted article, indicate the article’s retracted status in the References list and also include a citation and full reference for the retraction notice.
**Response**: We carefully reviewed the reference list and edited all the references according to the journal guidelines (see changes in “Manuscript with track changes”). We eliminated number 72 (Kusukawa, 2010) because we are not using Gessner’s visual methods anymore in comparison to those used in the HNB). We adjusted the referencing numbers that followed. Lastly, we are not including any retracted papers.

**Review Comments to the Author**

**Reviewer #5**: This is a comprehensive study on the provenance of woodcuts in Historia Naturalis Brasiliae, a historic collection of significant botanical and cultural value. Such interdisciplinary collaborations of authors from science and humanities are valuable and very much needed in the study of historic botanical collections. The manuscript is well written, methods are thorough and the arguments and conclusions are clear. The authors have carefully considered the comments addressed by the reviewers and provided substantial answers. I agree with the authors’ decision to keep the original title as it reflects the research they have carried out.
I provide below a few minor comments to help amend the text (line numbers refer to the final text without track changes):
Line 92: Alternatively: “included 26 plant images (15 drawings and 11 proof woodcuts)”, for the sake of clarity as later a reference to 26 images is made (in line 563).
Line 127: "which woodcut" replace with "the woodcut of which"
Line 212: “degrees of similarity” instead of “degrees of similarities”.

**Response**: We agreed with the reviewer’s comments and edited the text according to those.

**Reviewer #5**: Line 449: I would suggest that the authors don’t use the term “Photoshop”. It directly refers to a software developed by a private company and there might be legal restrictions in the use of it. “Image manipulation” could be an alternative.

**Response**: To avoid any potential problem with this trademarked term, we used instead *Image editing*, as similarly advised by the reviewer.

**Reviewer #5**: Lines 531-532: I suggest another phrasing here: When connecting the images and descriptions in De Laet’s manuscript to the corresponding species and woodcuts in the HNB (Supporting Information S5), we find 388 plant descriptions and 165 plant entries with the word Icon next to the entry. Out of these… etc.

**Response**: We agreed with the reviewer’s phrasing and we have applied it to the text.

**Reviewer #5**: Line 641: It is not clear what “their” refers to in heading 4.1, and perhaps it is redundant, please consider omitting it or rephrasing the heading.

**Response**: We changed the pronoun to “its”, as it refers to Brazilian flora. We left the term “Tupi names” as we aim to highlight that the vernacular Indigenous names (in this case from the Tupi linguistic branch) were crucial when organizing the images.

**Reviewer #5**: 669-673: “Our database with all… natural history collections”: This sentence better fits as a conclusion, please consider transferring it to the conclusions section.

**Response**: We completely agreed with the reviewer, so we have moved this sentence to the conclusion (lines 805-809).

**Reviewer #5**: Line 697: Strictly speaking, Gessner was not an author of a popular herbal. His only botanical book, Historia Plantarum, was not finished before his death and was published only in 2001. The authors could rather refer to Fuchs and Matthioli who both wrote popular herbals. And either add birth and death dates for both scholars or for none.´

**Response**: We meant to write “natural history books” instead of herbals as we were referring to Gessner’s *Historia Plantarum*, instead of his postmortem herbal. However, in this context, it is better to refer to popular herbals as those written by Fuchs and Matthioli. In contrast to Marcgrave, the authors could review and edit their work, including the images. As suggested by the reviewer, we added the dates of birth and death for both authors to be consistent.

**Reviewer #5**: Line 732: Not only images never seen before but perhaps also “images of plant species never seen before”? At least not seen in the European scholar circles. This also adds to the argument of generating a new visual language.
Lines 746-7: Consider an alternative phrasing, such as: “In contrast to what is argued in previous research”, the Theatrum … etc.

**Response**: We agreed with the reviewer and adjusted the phrasing accordingly in the text.

**Reviewer #6**: The manuscript presents a systematic analysis of the woodcut images from mid-seventeenth century, namely images of natural elements that originated in Dutch Brazil and circulated in Europe. These images played a significant role in disseminating scientific botanical knowledge. To analyze the (dis-) similarities among the historical visual sources, the authors built a database in FileMaker Pro with all woodcuts and illustrations organized by species and created a spreadsheet with the background information. The analysis are very interesting and the approach is rater creative. I find this work quite interesting overall and well written. With the corrections and edits in response to the previous reviewers in my opinion the work is suitable for publication the way it is.

**Response**: We kindly thank the reviewer for appreciating our work.
